# Identification of DUSP7 as an RNA Marker for Prognostic Stratification in Acute Myeloid Leukemia: Evidence from Large Population Cohorts

**DOI:** 10.1155/2023/4348290

**Published:** 2023-07-26

**Authors:** Xin Gao

**Affiliations:** Anhui Medical College, Hefei, China

## Abstract

**Background:**

The problem of prognostic stratification in acute myeloid leukemia (AML) patients still has limitations.

**Methods:**

The expression profile data and clinical features of AML patients were obtained from multiple publicly available sources, including GSE71014, TCGA-LAML, and TARGET-AML. Single-cell analysis was performed using the TISCH project. All the analysis was conducted in the *R* software.

**Results:**

In our study, three public AML cohorts, GSE71014, TARGET-AML, and TCGA-AML, were selected. Then, we identified the prognosis-related molecules through bioinformatic analysis. Finally, the DUSP7 was noticed as a risk factor for AML patients, which has not been reported previously. Biological enrichment analysis and immune-related analysis were performed to illustrate the role of DUSP7 in AML. Single-cell analysis indicated that the DUSP7 was widely distributed in various cells, especially in monocyte/macrophages and malignant. Following this, a prognosis model based on DUSP7-derived genes was constructed, which showed a good prognosis prediction ability in all cohorts.

**Conclusions:**

Our results preliminarily reveal the role and potential mechanism of DUSP7 in AML, providing direction for future research.

## 1. Introduction

Acute myeloid leukemia (AML) is a highly heterogeneous malignant clonal disease that seriously affects human health [1]. The pathogenesis of AML is extremely complex, and researchers have not yet fully elucidated it [2]. It is now recognized that chromosomal karyotype abnormalities and gene mutation reproducibility play an important initiating role in the occurrence and development of diseases [3]. By classifying these two factors, risk stratification can be performed on most patients. Based on the different risk stratification of patients, corresponding treatment plans can be adopted, which significantly improves the five-year survival rate of AML patients [4]. At the same time, with the development of chemotherapy, hematopoietic stem cell transplantation, gene targeting therapy, biological immunotherapy, and other treatment methods, the complete remission rate and 5-year disease-free survival rate of AML patients have improved, but most patients still have drug resistance and relapse, leading to poor prognosis [5]. These issues suggest that clinical doctors need to further explore the new pathogenesis of AML and improve the existing prognostic evaluation system.

At present, studies have found that the factors affecting the prognosis of AML patients not only include general clinical characteristics such as age and FAB typing, but also include molecular genetics, cytogenetics, and mutations of specific molecules [6]. For instance, Falini et al. noticed that the NPM1 mutation could assist in the genetic typing of AML, thereby indicating clinical prognosis and treatment choices [7]. Nowadays, many gene mutations have been linked to AML patients' prognosis, such as MLL, HOX, RAR-*α*, CBF, NPM1, WNT, RUNX1, WT1, RB, PU.1, p53, MYC, MPL, JUNB, GATA-1, FOS, FES, N-RAS, CEBPA, KIT, and FLT3 [8–10]. Some mutations have been involved in the risk stratification of AML, such as NPM1, RUNX1, c-KIT, RUNX1-RUNX1T1, CEBPA, PML-RAR*α*, BCR-ABL1, ASXL1, TP53, FLT3-ITD, and MLL [11, 12]. However, many cases do not have the above gene mutations, especially 50% of AML patients with normal cytogenetics (CN-AML). These patients lack characteristic chromosome changes and clear molecular markers, and it is difficult to evaluate their prognosis [13]. Numerous pieces of evidence suggest that gene expression levels are of great significance for the prognostic evaluation of AML [14]. As early as 1996, Bergmann et al. observed that the expression of WT-1 can serve as a prognostic and recurrence marker for AML [15]. Paschka et al.'s study of 196 patients with CN-AML also supported the above viewpoint, finding that WT-1 mutation is an independent risk factor for CN-AML [16]. Recent studies have shown that gene expression levels of CPT1A, TGF*β*1, VEGF, CSRP2, and others are all associated with the prognosis of AML [17–19]. As is well known, the prognosis of diseases is influenced by multiple factors, and the relationships between these factors are complex. Therefore, traditional prognostic evaluation systems are no longer able to meet the requirements. With the rise of big data, bioinformatics, and artificial intelligence, their clinical applications in medicine are becoming increasingly widespread and showing great potential [20–22].

In our study, three public AML cohorts, GSE71014, TARGET-AML, and TCGA-AML, were selected. Then, we identified the prognosis-related molecules through bioinformatic analysis. Finally, the DUSP7 was noticed as a risk factor for AML patients, which has not been reported previously. Biological enrichment analysis, single-cell analysis, and immune-related analysis were performed to illustrate the role of DUSP7 in AML. Following this, a prognosis model based on DUSP7-derived genes was constructed, which showed a good prognosis prediction ability in all cohorts.

## 2. Methods

### 2.1. Data Collection Process

The expression profile data and clinical features of AML patients were obtained from multiple publicly available sources. For TCGA-LAML (access time 22/3/2023) and TARGET-AML (access time 22/3/2023), the expression profile of each AML patient was downloaded in a “STAR-Count” form and then extracted into a merge matrix through *R* code. The human genomic reference file obtained from the Ensembl project (https://www.ensembl.org/) was utilized for probe annotation. Clinical information was downloaded in the “bcr-xml” form and organized using Perl code. For GSE71014, the expression profile of patients was downloaded from the direct link “Series Matrix File(s)” of the website https://www.ncbi.nlm.nih.gov/geo/query/acc.cgi?acc=GSE71014. Probe annotation was conducted by the GPL10558 platform. For all downloaded data, data quality control and standardization processing are carried out before data analysis. The interaction data of multiple proteins were downloaded from the STRING database and then visualized through Cytoscape software [23].

### 2.2. Differentially Expressed Genes (DEGs) Analysis

DEGs analysis was performed using the limma package with the specific threshold (|logFC| > 1 and *P* value <0.05) [24].

### 2.3. Prognosis Analysis

For a specific gene list, the expression profile of these genes was firstly combined with the corresponding survival information. Next, the molecules remarkably correlated with AML patients' survival were identified using the univariate Cox regression analysis (*P* < 0.05). Furthermore, LASSO regression analysis was performed for variable optimization. Ultimately, multivariate Cox regression analysis was used to screen independent prognosis markers and model construction. The TCGA-AML cohort was set as the training cohort. The GSE71014 and TARGET-LAML cohorts were set as the validation cohort. Kaplan–Meier (KM) survival curves were used to compare the prognosis difference in different groups. The prediction ability of specific variables on patients' survival was performed using the receiver operating characteristic (ROC) curves.

### 2.4. Biological Enrichment Analysis

Evaluation of biological function was performed using specific *R* packages. Clusterprofiler package was used for the Gene Ontology (GO) and Kyoto Encyclopedia of Genes and Genomes (KEGG) analysis [25]. Gene set enrichment analysis (GSEA) was conducted based on the Hallmark gene set [26].

### 2.5. Sing-Cell Analysis

The single-cell data of GSE116256, GSE135851, GSE154109, PBMC_8K, PBMC_30K, and PBMC_60K were analyzed in the TISCH project [27].

### 2.6. Tissue Microenvironment Analysis

Estimate package was used to quantify the stromal score, immune score, and estimate score of the tissue microenvironment, respectively, representing the corresponding cell components [28]. The microenvironment of bone marrow was quantified using the single sample GSEA (ssGSEA) algorithm [29].

### 2.7. Statistical Analysis

All the statistical analyses were performed in the *R* software. Analysis with a statistic *P* value <0.05 was regarded as statistically significant. For data with different distributions, different analysis methods are used according to statistical requirements.

## 3. Results

**Figure 1:**
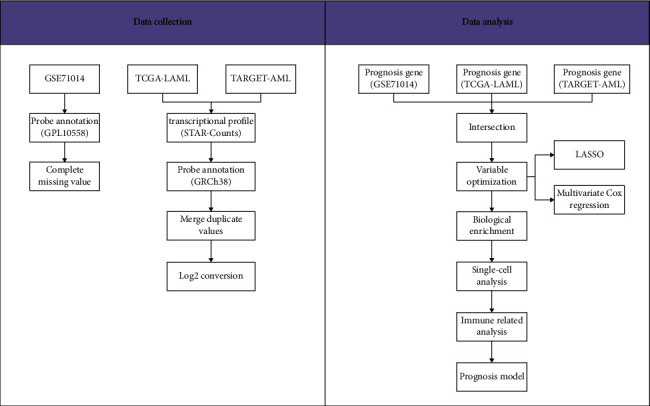
The flowchart of the whole study.

The flowchart of the whole study is shown in Figure 1.

### 3.1. Identification of the Molecules Significantly Correlated with AML Patients' Survival

**Figure 2:**
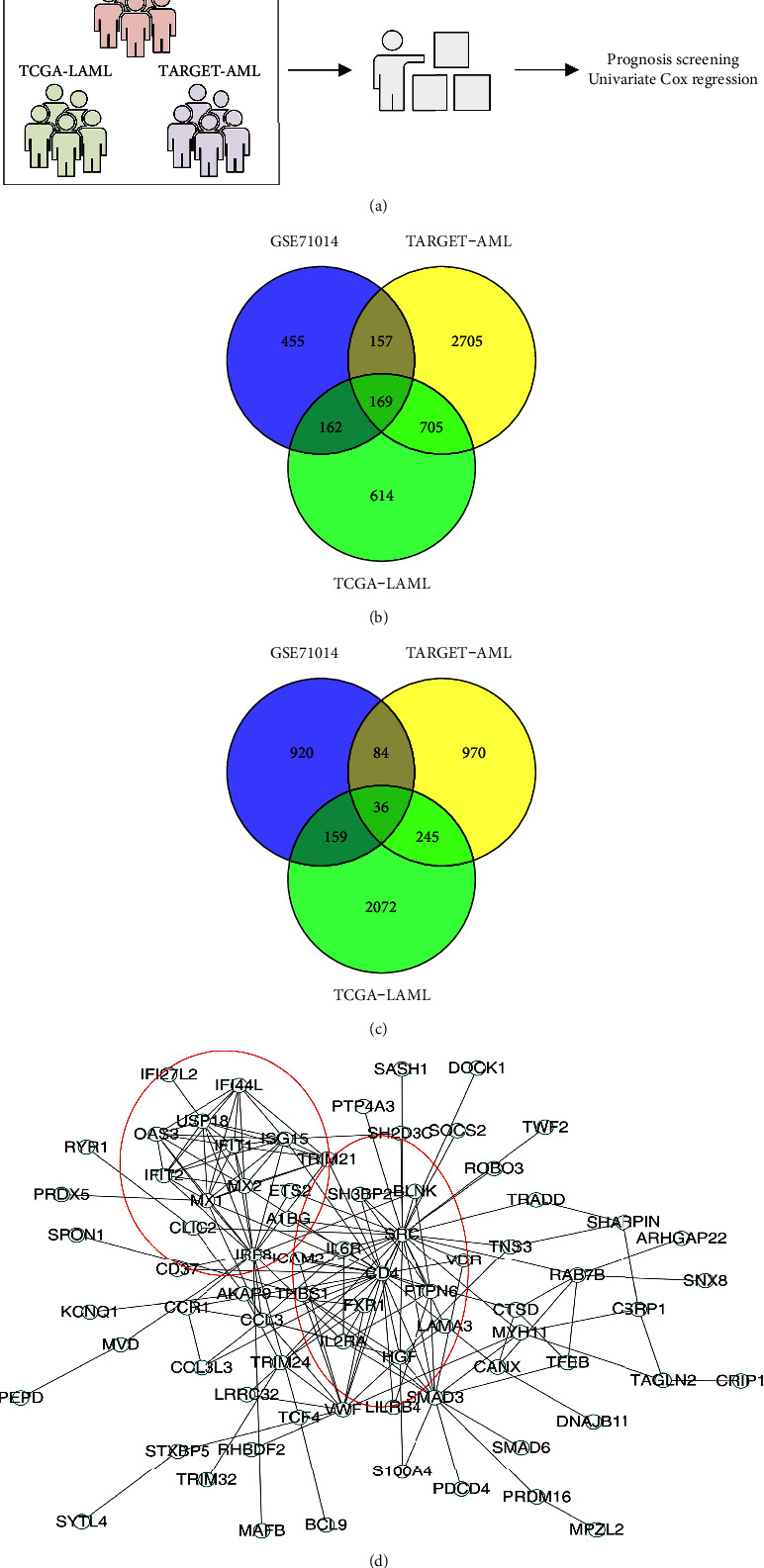
Identification of prognosis-related molecules in three AML cohorts. (a) Three AML cohorts were identified, including GSE71014, TARGET-AML, and TCGA-LAML; (b) 169 molecules were common risk factors in GSE71014, TCGA-LAML, and TARGET-AML cohorts; (c) 36 molecules were common protective factors in GSE71014, TCGA-LAML, and TARGET-AML cohorts; (d) PPI network of prognosis-related molecules.

Firstly, after a comprehensive search, we have finally identified three AML datasets with complete transcriptional profiles and clinical information, including GSE71014, TCGA-LAML, and TARGET-AML (Figure 2(a)). Then, we performed univariate Cox regression analysis to identify the prognosis-related molecules in these three cohorts (Supplementary file 1–3). By taking intersections, we noticed that 169 molecules were common risk factors in GSE71014, TCGA-LAML, and TARGET-AML cohorts, while 36 molecules were common protective factors in GSE71014, TCGA-LAML, and TARGET-AML cohorts (Figures 2(b) and 2(c)). The PPI network of the above prognosis-related molecules is shown in Figure 2(d).

### 3.2. DUSP7 Is Associated with Poor Prognosis of AML Patients

**Figure 3:**
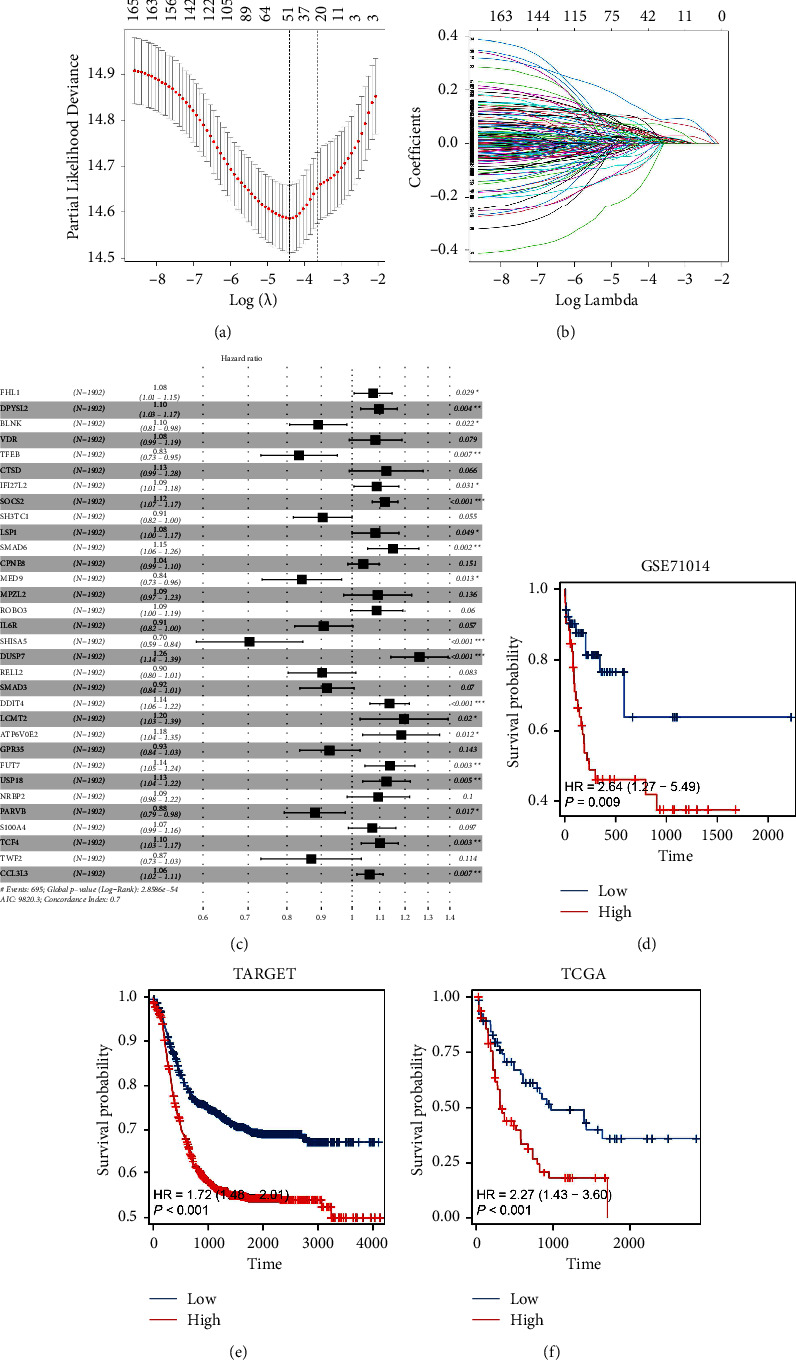
DUSP7 is associated with poor prognosis of AML patients. (a, b) LASSO regression analysis; (c) multivariate Cox regression analysis of prognosis-related molecules; (d) KM survival curves of DUSP7 in GSE71014 cohort; (e) KM survival curves of DUSP7 in TARGET-AML cohort; (f) KM survival curves of DUSP7 in TCGA-LAML cohort.

Then, based on the identified risk genes, we performed LASSO regression analysis to optimize variables (Figures 3(a) and 3(b)). Following this, multivariate Cox regression analysis was performed, and we noticed that the SOCS2, DUSP7, and DDIT4 were significantly correlated with patients' prognosis (*P* < 0.001) (Figure 3(c)). Considering the DUSP7 has not been reported in AML, we selected it for further analysis. KM survival curves in GSE71014, TCGA-LAML, and TARGET-AML cohorts all showed that the AML patients with high DUSP7 expression might have a poor prognosis (Figure 3(d), GSE71014, HR = 2.64, *P*=0.009; Figure 3(e), TARGET-AML, HR = 1.72, *P* < 0.001; Figure 3(f), TCGA-LAML, HR = 2.27, *P* < 0.001).

### 3.3. Biological Enrichment of DUSP7 in AML and Single-Cell Analysis

**Figure 4:**
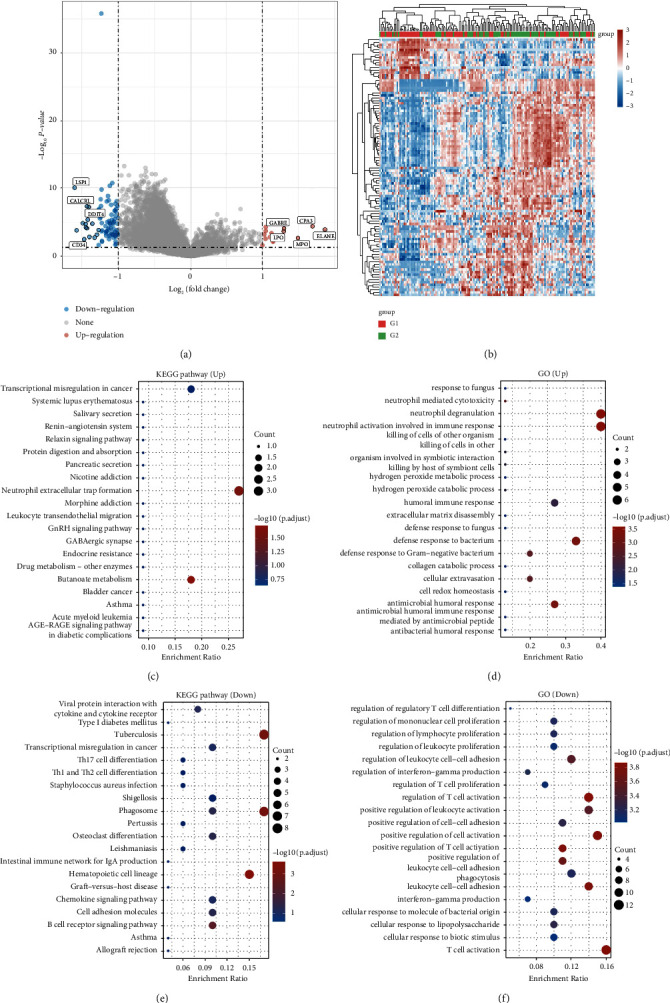
Biological enrichment analysis of DUSP7. (a) DEGs analysis in patients with high and low DUSP7 expression; (b) the heatmap of the DEGs; (c) KEGG analysis of the molecules upregulated in patients with high DUSP7 expression; (d) GO analysis of the molecules upregulated in patients with high DUSP7 expression; (e) KEGG analysis of the molecules downregulated in patients with high DUSP7 expression; (f) GO analysis of the molecules downregulated in patients with high DUSP7 expression.

**Figure 5:**
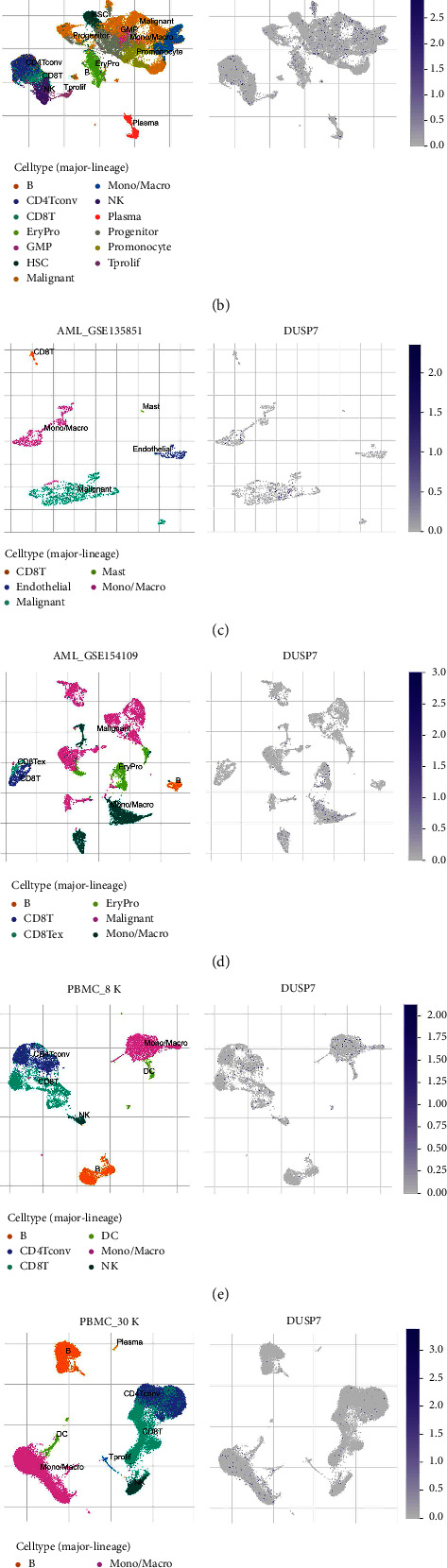
Single-cell analysis of DUSP7. (a) GSEA of DUSP7 based on hallmark gene set; (b–g) single-cell analysis of DUSP7 in multiple cohorts.

Then, we tried to explore the potential biological function of DUSP7 in AML. Limma package was used to screen the DEGs in patients with high and low DUSP7 expression (Figure 4(a)). The heatmap of these DEGs is shown in Figure 4(b). KEGG analysis showed that the molecules upregulated in patients with high DUSP7 levels were mainly enriched in transcriptional misregulation in cancer, systemic lupus erythematosus, salivary secretion, renin-angiotensin system, and relaxin signaling pathway (Figure 4(c)). For GO analysis, the terms of response to fungus, neutrophil-mediated cytotoxicity, neutrophil degranulation, and neutrophil activation-related pathways were enriched (Figure 4(d)). As for the downregulated genes, the terms of viral protein interaction, cytokine and cytokine receptor, tuberculosis, and transcriptional misregulation in cancer were enriched by the KEGG analysis (Figure 4(e)). For GO analysis, these downregulated genes were enriched in the regulation of regulatory T-cell differentiation, mononuclear cell proliferation, lymphocyte proliferation, leukocyte proliferation, and leukocyte cell-cell adhesion (Figure 4(f)). Based on the GSEA, we found that the IL6_JAK_STAT3 signaling, interferon alpha response, bile acid metabolism, coagulation, and UV response DN were the top five pathways DUSP7 involved in (Figure 5(a)). Single-cell analysis indicated that the DUSP7 was widely distributed in various cells, especially in monocyte/macrophages and malignant (Figure 5(b)).

### 3.4. Tissue Microenvironment Analysis

**Figure 6:**
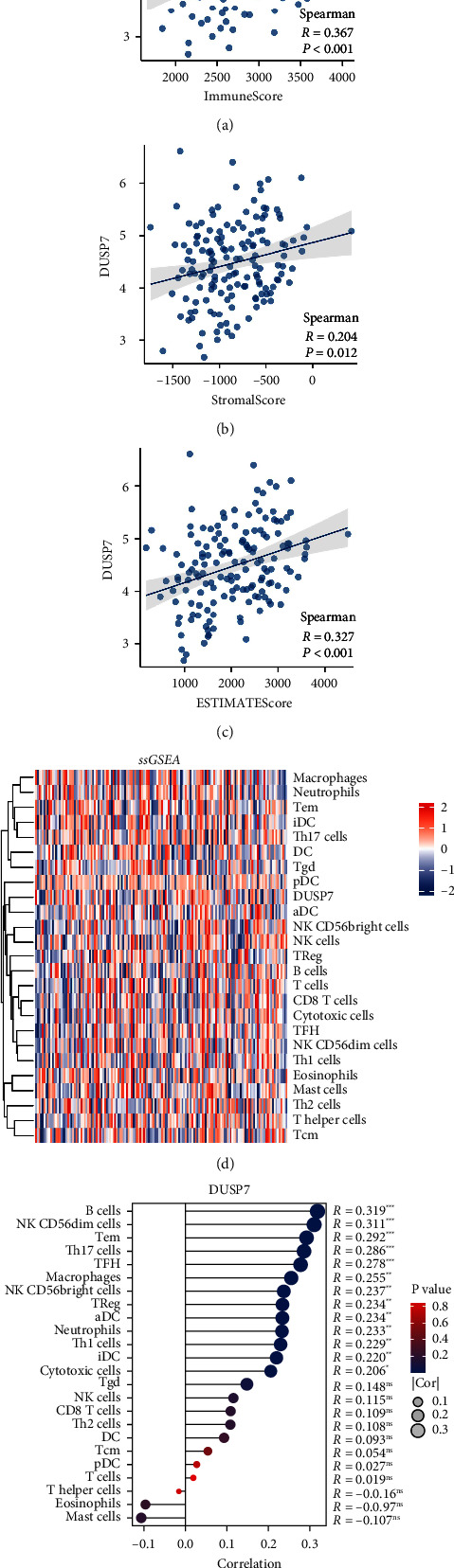
Immune-related analysis of DUSP7. (a) Correlation between DUSP7 and immune score quantified by Estimate package; (b) correlation between DUSP7 and stromal score quantified by Estimate package; (c) correlation between DUSP7 and estimate score quantified by Estimate package; (d) tissue microenvironment of AML was quantified by ssGSEA algorithm; (e) correlation between DUSP7 and quantified cells.

Then, we tried to explore the effect of DUSP7 on the AML microenvironment. Interestingly, we found that the DUSP7 was positively correlated with the immune score, stromal score, and estimate score according to the Estimate package (Figure 6(a), immune score, cor = 0.367, *P* < 0.001; Figure 6(b), stromal score, cor = 0.204, *P*=0.012; Figure 6(c), estimate score, cor = 0.327, *P* < 0.001). Next, the ssGSEA algorithm was used to quantify the tissue microenvironment of AML (Figure 6(d)). Correlation analysis showed that DUSP7 was positively correlated with B cells, NK CD56 dim cells, Tem, Th17 cells, macrophages, and Treg (Figure 6(e)).

### 3.5. Establishment of a Prognosis Model Based on the DUSP7-Derived Molecules

**Figure 7:**
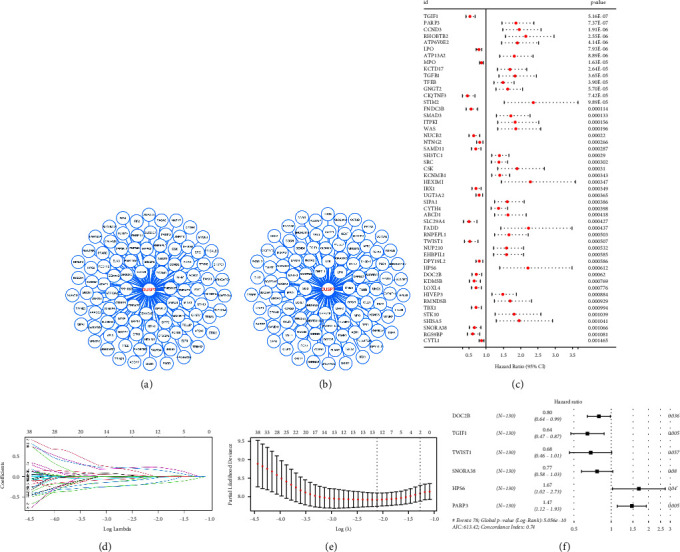
Construction of a prognosis model based on DUSP7-derived genes in AML. (a) The top 100 genes positively correlated with DUSP7 in AML; (b) the top 100 genes negatively correlated with DUSP7 in AML; (c) univariate Cox regression analysis of the genes significantly correlated with DUSP7 in AML; (d)-(e): LASSO regression analysis; (f) multivariate Cox regression analysis.

**Figure 8:**
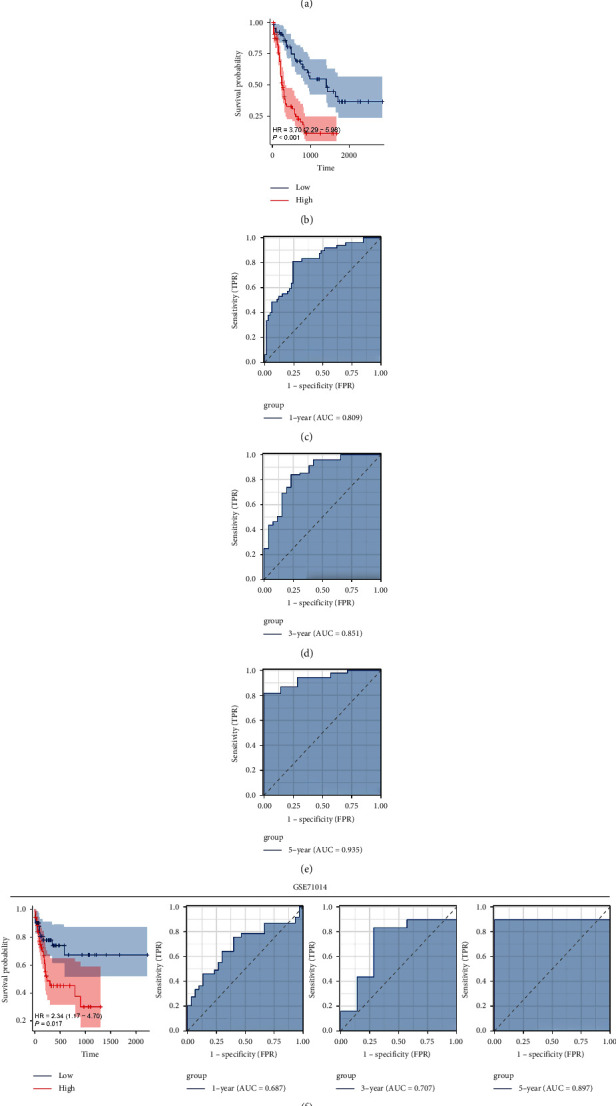
Evaluation of the prognosis model. (a) The overview of the prognosis model; (b) KM survival curve of patients in high- and low-risk groups; (c–e) ROC curves of 1, 3, and 5 years in TCGA-LAML cohort; (f) evaluation of our model in GSE71014; (g) evaluation of our model in TARGET-AML cohort.

We then identified the top 100 molecules positively and negatively correlated with DUSP7 in AML patients (Figures 7(a) and 7(b)). Univariate Cox regression analysis was performed to identify the prognosis-related genes (Figure 7(c)). LASSO regression analysis was then conducted to reduce data dimensions and optimize variables (Figures 7(d) and 7(e)). Multivariate Cox regression analysis was applied to construct a prognosis model based on the molecules identified by LASSO regression analysis, including DOC2B, TGIF1, TWIST1, SNORA38, HPS6, and PARP3 (Figure 7(f)). The risk score of each patient was calculated with the formula of “Risk score=DOC2B*∗* − 0.229+TGIF1*∗* − 0.443+TWIST1*∗* − 0.382+SNORA38*∗* − 0.260+HPS6*∗*0.514+PARP3*∗*0.385”. We noticed a higher proportion of death cases in high-risk patients (Figure 8(a)). KM survival curves indicated that high-risk patients might have a worse prognosis (Figure 8(b), HR = 3.70, *P* < 0.001). ROC curves indicated that the 1-year AUC = 0.809, 3-year AUC = 0.851, and 5-year AUC = 0.935 (Figures 8(c)–8(e)). In the validation cohorts (GSE71014 and TARGET-AML), our model also showed a good prognosis prediction ability (Figure 8(f), HR = 2.34, *P*=0.017, 1-year AUC = 0.687, 3-year AUC = 0.707, 5-year AUC = 0.897; Figure 8(g), HR = 2.57, *P* < 0.001, 1-year AUC = 0.677, 3-year AUC = 0.696, 5-year AUC = 0.686).

## 4. Discussion

AML is a highly heterogeneous hematological malignancy caused by dysgenesis of myeloid hematopoietic stem/progenitor cells, characterized by abnormal clonal proliferation and differentiation arrest of primitive and immature myeloid cells in the bone marrow and peripheral blood [30–32]. AML has complex cytogenetic and epigenetic mutations, including abnormal DNA methylation, resulting in high heterogeneity in disease diagnosis, development, and prognosis [33]. With the continuous research on AML, it is found that abnormal DNA methylation of special gene promoters affects the change of its expression level, especially tumor suppressor, which leads to the occurrence and development of disease [34]. Although various molecular targeted therapies for AML have emerged one after another, increasing the possibility of selecting treatment options for AML, its 5-year survival rate is still very poor, especially for elderly patients with high-risk factors [35]. Therefore, an in-depth exploration of the pathogenesis of AML has important scientific significance and clinical application value for disease prevention, diagnosis, specific treatment, and prognosis stratification.

In our study, three public AML cohorts, GSE71014, TARGET-AML, and TCGA-AML, were selected. Then, we identified the prognosis-related molecules through bioinformatic analysis. Finally, the DUSP7 was noticed as a risk factor for AML patients, which has not been reported previously. Biological enrichment analysis, single-cell analysis, and immune-related analysis were performed to illustrate the role of DUSP7 in AML. Following this, a prognosis model based on DUSP7-derived genes was constructed, which showed a good prognosis prediction ability in all cohorts.

DUSP7, whose full name is dual specificity phosphatase 7, has been reported involved in various pathophysiological processes, especially in cancers [36–38]. For instance, Li et al. found that DUSP7 could affect the dephosphorylation of PEA15 and resistance to breast cancer [39]. Guo et al. have proved that DUSP7 is extremely important for the correct chromosome arrangement during cell division through a series of experiments at the cellular level [40]. Moreover, Tischer and Schuh also supported this viewpoint at the animal level [41]. It is worth noting that over half of AML patients are accompanied by chromosomal abnormalities, and the promoting effect of DUSP7 on AML seems understandable, but the relevant mechanisms still need further exploration.

Biological enrichment analysis showed that the IL6_JAK_STAT3 signaling, interferon alpha response, bile acid metabolism, coagulation, and UV response DN were the top five pathways DUSP7 was involved in. This suggests the importance of metabolism and immunity in AML. AML cells have an atypical metabolic phenotype, characterized by increased mitochondrial mass, and more dependence on oxidative phosphorylation and fatty acid oxidation for survival. Tcheng et al. indicated that AML has a special fatty acid metabolism manner and level [42]. Moreover, in a retrospective study involving 84 patients, researchers found that IFN-*α* can effectively reduce the recurrence rate of AML patients [43]. Also, based on the long-term results of two registered studies, IFN-*α* can prevent recurrence and improve the survival rate [44]. Therefore, the correlation between DUSP7 and IFN-*α* response can partially explain the impact of DUSP7 on the prognosis of AML patients. However, further research is needed to confirm the relationship between DUSP7 and IFN-*α* response.

Immune-related analysis showed that the DUSP7 was positively correlated with B cells, NK CD56 dim cells, Tem, Th17 cells, macrophages, and Treg. Some studies have begun to focus on the role of immune cells in AML. Goswami et al. found that after chemotherapy, AML patients experienced irreversible immune damage to their B cells [45]. Meanwhile, researchers also found that regulatory B cells have clinical indicative significance in AML [46, 47]. Moore et al. found that the AML progression could be suppressed by LC3-associated phagocytosis medicated by bone marrow macrophages [48]. Liu et al. demonstrated that chenodeoxycholic acid can inhibit M2 macrophage polarization through lipid peroxidation [49]. Moreover, Treg is believed to play a role in helping leukemia cells evade immune surveillance, thereby promoting AML progression [50, 51]. Our results suggest that DUSP7 may affect AML progression by affecting the recruitment of local immune cells.

The continuous development of bioinformatics and the massive data in the era of big data have improved the convenience of data secondary mining. Although our results are based on reliable data and algorithms, some limitations cannot be ignored. Firstly, most patients are of Western ethnicity. There are specific differences in genomics among populations of different ethnicities; therefore, racial bias will reduce the universality of our conclusions. Secondly, it cannot be denied that bioinformatics analysis cannot fully reflect the true situation in the body. Therefore, most conclusions only have indicative significance and still require subsequent experimental verification.

## Data Availability

All raw data can be obtained from the corresponding author based on reasonable requirements.

## Supplementary Materials

Supplementary Materials Supplementary file 1: results of univariate Cox regression analysis in GSE71014. Supplementary file 2: results of univariate Cox regression analysis in TARGET-LAM. Supplementary file 3: results of univariate Cox regression analysis in TCGA-LAML.
