# Supplementary material for: Identification of DUSP7 as an RNA Marker for Prognostic Stratification in Acute Myeloid Leukemia: Evidence from Large Population Cohorts
Source: Genet Res (Camb). 2023 Jul 26;2023:4348290. doi: 10.1155/2023/4348290 (PMC10396553; doi:10.1155/2023/4348290)
Supplement: Supplementary Materials — Supplementary file 1: results of univariate Cox regression analysis in GSE71014. Supplementary file 2: results of univariate Cox regression analysis in TARGET-LAM. Supplementary file 3: results of univariate Cox regression analysis in TCGA-LAML. [file 4348290.f1.pdf]

Supplementary file 1. Results of univariate Cox regression analysis in GSE71014

id	HR	HR.95L	HR.95H	pvalue
TSPAN6	7.097344	0.167525	300.6845	0.305239
DPM1	0.57391	0.208284	1.581361	0.282929
SCYL3	0.49131	0.104466	2.310668	0.368288
FGR	1.262221	0.919053	1.733526	0.150284
CFH	0.986194	0.493514	1.970721	0.968605
FUCA2	0.325279	0.09202	1.149826	0.081289
GCLC	0.758665	0.312005	1.844754	0.542367
NFYA	0.221881	0.054931	0.896234	0.034535
LAS1L	0.219516	0.082756	0.58228	0.002315
ENPP4	0.831154	0.521955	1.32352	0.435907
SEMA3F	1.551246	0.248133	9.69789	0.638706
ANKIB1	0.541933	0.2542	1.155352	0.112719
CYP51A1	1.237117	0.757943	2.019227	0.394637
KRIT1	0.214453	0.053568	0.858539	0.029595
RAD52	0.319515	0.033283	3.067331	0.322805
BAD	2.444464	0.903819	6.611289	0.07828
LAP3	0.73364	0.372257	1.445851	0.370887
CD99	1.123164	0.792395	1.592006	0.514026
HS3ST1	2.926541	1.063252	8.055144	0.037646
MAD1L1	2.434279	1.091644	5.42825	0.029686
LASP1	0.844994	0.296303	2.409746	0.752757
SNX11	1.764561	0.400161	7.781057	0.453163
TMEM176/	1.27215	1.024163	1.580185	0.029572
M6PR	1.034755	0.59392	1.802798	0.903998
KLHL13	0.576031	0.265477	1.249867	0.162823
ICA1	0.969018	0.439057	2.138668	0.937893
DBNDD1	0.660576	0.21371	2.041839	0.471432
ALS2	0.832316	0.55786	1.241798	0.368592
CASP10	0.370726	0.041636	3.300935	0.373742
CFLAR	0.859712	0.486292	1.51988	0.603096
TFPI	0.890056	0.598323	1.324034	0.565436
RBM5	0.641775	0.357513	1.152059	0.137339
MTMR7	0.169782	0.010477	2.751219	0.212103
SLC7A2	0.80374	0.196246	3.291787	0.761345
ARF5	3.145547	1.548859	6.388227	0.001523
SARM1	2.102371	0.873436	5.060434	0.097313
POLDIP2	0.492171	0.135953	1.781743	0.280131
PLXND1	1.043601	0.538259	2.023383	0.899466
AK2	0.793881	0.468306	1.3458	0.391372
CD38	0.516299	0.339798	0.784481	0.001954
FKBP4	0.678983	0.416392	1.107173	0.120693
RBM6	0.26708	0.127341	0.560162	0.000477
CAMKK1	0.038841	0.001262	1.195754	0.063209
RECQL	1.26524	0.531032	3.014567	0.595344
HSPB6	0.196621	0.034486	1.12104	0.067053
NDUFAB1	1.200758	0.591618	2.437076	0.612449
PDK4	1.468117	1.003366	2.148135	0.048011
SLC22A16	0.950554	0.629036	1.436411	0.80976
ZMYND10	0.336067	0.022324	5.059196	0.4306
SLC25A13	0.511975	0.256515	1.021844	0.057607
ST7	0.514442	0.323492	0.818106	0.004982
CDC27	0.234825	0.015786	3.49306	0.292843
SLC4A1	1.012979	0.85187	1.204557	0.883993
HCCS	2.641868	1.096242	6.36672	0.03041
DVL2	1.695981	0.697489	4.123868	0.243912
UPF1	1.952006	0.835059	4.562944	0.122614

SKAP2	0.88396	0.606955	1.287386	0.52021
SLC25A5	0.233073	0.04291	1.265961	0.091638
HOXA11	0.999135	0.594892	1.678071	0.997391
POLR2J	3.1398	1.125527	8.75887	0.028824
DHX33	0.383893	0.173033	0.85171	0.018536
THSD7A	3.539326	0.444191	28.20144	0.232629
LIG3	0.207809	0.024426	1.768004	0.15035
RPAP3	0.356507	0.091161	1.3942	0.138248
ACSM3	0.502934	0.2826	0.895054	0.019442
CIAPIN1	0.817829	0.331039	2.020441	0.662976
SPPL2B	1.913689	0.562557	6.509924	0.29879
COPZ2	2.233774	0.607724	8.210553	0.226244
PRKAR2B	1.404668	0.13394	14.73121	0.776883
MSL3	0.997537	0.148913	6.682319	0.997973
CREBBP	1.656266	0.747335	3.670668	0.213988
MPO	0.936254	0.782503	1.120215	0.471733
WDR54	1.882431	1.080328	3.280064	0.02557
CROT	0.309545	0.1198	0.799815	0.015471
ABCB4	0.294976	0.029554	2.944116	0.29831
RHBDD2	1.34633	0.878164	2.064087	0.172556
IBTK	0.59891	0.312687	1.147131	0.122102
ZNF195	0.892655	0.384291	2.073515	0.791721
MYCBP2	0.604	0.299447	1.2183	0.159016
FBXL3	1.984472	0.57889	6.802898	0.275573
ITGAL	1.271821	0.905292	1.786747	0.165652
PDK2	6.898315	1.897836	25.07421	0.003357
ITGA3	4.030758	0.694098	23.40737	0.120393
ZFX	0.647243	0.286244	1.463521	0.295991
LAMP2	0.726298	0.419339	1.257955	0.253828
ITGA2B	1.002271	0.751115	1.337408	0.987704
GDE1	0.59259	0.237951	1.475781	0.261026
CRLF1	1.287381	0.212956	7.782588	0.783186
OSBPL7	0.543575	0.217448	1.358823	0.19222
TMEM98	1.941477	0.883762	4.265095	0.098486
YBX2	0.566388	0.075765	4.23409	0.579667
MAP3K14	4.868308	2.298632	10.31067	3.57E-05
TMEM132/	0.037625	0.001368	1.035189	0.052439
AP2B1	1.005665	0.468468	2.15887	0.988437
ZNF263	0.400189	0.188824	0.848152	0.016861
SPATA20	1.333402	0.648634	2.741081	0.43387
TNFRSF12/	1.783539	0.825668	3.852656	0.140896
MAP3K9	0.311552	0.028915	3.356895	0.3363
RALA	0.950586	0.585495	1.543333	0.837609
BAIAP2L1	1.320834	0.369541	4.720992	0.668526
AGK	0.348166	0.108442	1.117825	0.07626
ALDH3B1	1.237204	0.703262	2.176532	0.460187
TTC22	0.778697	0.083553	7.257293	0.826157
PHTF2	1.698394	0.34949	8.253582	0.511398
FARP2	3.010707	0.827009	10.9604	0.094554
GGCT	0.474371	0.19132	1.176189	0.107465
DBF4	0.482315	0.05694	4.085464	0.503573
TBXA2R	1.712643	0.471414	6.222019	0.413681
IFRD1	0.411861	0.132582	1.279429	0.12506
COX10	0.663222	0.264295	1.664286	0.381684
GTF2IRD1	2.010213	0.279139	14.47649	0.488199
PAF1	0.329365	0.12858	0.843686	0.02066
VPS41	0.198291	0.049437	0.795343	0.022427
ELAC2	0.684693	0.265096	1.768436	0.433979

ARSD	1.194477	0.522163	2.732434	0.673815
PNPLA4	0.233431	0.041989	1.297726	0.096471
ADIPOR2	0.588755	0.305352	1.135191	0.11378
CDKL3	1.984862	0.326596	12.06284	0.456527
PRSS21	1.063362	0.805992	1.402916	0.663915
MARK4	0.61521	0.104806	3.611281	0.590595
PROM1	0.955019	0.82956	1.099452	0.52185
CCDC124	1.239369	0.530766	2.893997	0.619905
CEACAM2	0.663626	0.160462	2.74457	0.571332
PAFAH1B1	0.909468	0.464847	1.779366	0.781687
KIAA0100	0.871691	0.366153	2.07521	0.756337
GAS7	1.168725	0.310796	4.394902	0.817539
TRAPPC6A	0.835836	0.479352	1.457431	0.527298
MATK	0.707489	0.374508	1.336529	0.286339
CD79B	1.857035	1.303406	2.645823	0.00061
SCN4A	1.008511	0.065509	15.52595	0.995152
ST7L	0.425286	0.091558	1.975452	0.275213
TKTL1	1.145879	0.576793	2.276447	0.697421
RPUSD1	1.369395	0.785391	2.387656	0.267732
RHBDF1	1.146615	0.878055	1.497315	0.314976
LUC7L	0.32082	0.056134	1.833586	0.20115
CACNA2D	0.146989	0.012901	1.674746	0.12245
BAIAP3	15.11735	1.456463	156.9104	0.02291
PIGQ	0.761885	0.466073	1.245445	0.278097
TEAD3	0.714201	0.145544	3.504655	0.678338
DNAJC11	0.331854	0.112059	0.982756	0.046441
MYLIP	0.874263	0.588805	1.298114	0.505238
E2F2	0.986782	0.760901	1.279718	0.920084
PSMB1	0.507404	0.196329	1.311361	0.161382
SYN1	29.28004	0.568163	1508.935	0.093173
JARID2	0.636039	0.341465	1.184736	0.153923
CDKL5	0.00782	0.000108	0.566133	0.026394
CAMK1G	1.186546	0.80156	1.756438	0.392723
NADK	1.497565	0.906179	2.474899	0.11512
DLEC1	2.739815	0.075151	99.88718	0.582788
CYTH3	1.858085	0.61664	5.598858	0.270949
ADAM22	0.151326	0.001588	14.42312	0.416712
SYPL1	0.398664	0.115131	1.380454	0.146726
CYB561	2.160519	0.730948	6.386015	0.163571
SPAG9	0.625827	0.181466	2.158309	0.458088
CELSR3	0.584488	0.328029	1.041453	0.068433
AASS	1.829339	0.32116	10.41998	0.496255
PLEKHG6	2.039097	0.209532	19.84387	0.53939
SS18L2	0.664486	0.190678	2.315641	0.521066
MPND	0.830679	0.419743	1.643929	0.594266
MGST1	0.710987	0.445431	1.134864	0.152804
CRY1	0.987713	0.597492	1.632787	0.961553
PGLYRP1	1.017504	0.86683	1.19437	0.831938
NFIX	1.501331	1.116389	2.019006	0.00718
ST3GAL1	1.643576	0.714571	3.780369	0.242336
MMP25	1.009993	0.619559	1.646474	0.968188
IL32	1.094136	0.782316	1.530241	0.599147
PKD1	1.321748	0.634267	2.754387	0.456492
MED24	0.96065	0.489732	1.884395	0.907032
RHOBTB2	1.405441	0.463578	4.260912	0.547547
HEATR5B	1.036973	0.574907	1.870411	0.903978
SEC62	1.366162	0.580584	3.214689	0.474844
RPS20	0.634312	0.082309	4.888312	0.662174

CSDE1	0.254682	0.103636	0.625874	0.002869
UBE3C	0.717114	0.322444	1.594859	0.414862
REV3L	0.337845	0.102293	1.115812	0.075044
MASP2	1.952883	0.052364	72.83224	0.716982
FAM76A	1.184643	0.426258	3.292325	0.745255
TRAF3IP3	1.535161	0.692131	3.405022	0.291609
POMT2	0.558445	0.227929	1.368235	0.202578
VTA1	0.30444	0.137358	0.67476	0.003403
MLXIPL	2.307646	0.485618	10.96588	0.292985
BAZ1B	1.013436	0.227381	4.516885	0.986035
RANBP9	0.545788	0.249515	1.193854	0.12945
ETV7	0.667257	0.097034	4.588397	0.680879
DYRK4	2.161311	0.846894	5.515763	0.106893
ZNF207	0.46678	0.215083	1.013022	0.05395
UQCRC1	1.942278	0.981908	3.841953	0.056455
STARD3NI	0.528276	0.230265	1.211975	0.132017
CD9	1.54653	1.160466	2.061031	0.002924
NCAPD2	0.745959	0.406852	1.367709	0.343351
IFFO1	0.693302	0.210662	2.2817	0.546724
GIPR	0.013411	0.000739	0.243528	0.003558
PHF7	0.010105	0.000484	0.211033	0.003043
SEMA3G	0.033349	0.001673	0.664706	0.025916
NISCH	1.344605	0.673616	2.683968	0.401118
STAB1	1.418066	1.098787	1.83012	0.007279
FUZ	2.091805	0.929091	4.709603	0.074693
SLC6A13	0.768531	0.141154	4.184361	0.76075
IDS	0.82724	0.464755	1.472444	0.519116
PRSS3	1.114588	0.663866	1.87132	0.68155
ZNF200	1.685714	0.346041	8.211843	0.518032
CD4	1.51679	1.123593	2.047585	0.006506
LRRC23	1.085696	0.384129	3.068592	0.876741
BTK	0.800968	0.408149	1.571854	0.518802
HFE	0.922437	0.005135	165.701	0.975681
SCMH1	0.207203	0.075134	0.571423	0.002356
FYN	1.198033	0.710315	2.020632	0.498113
HIVEP2	0.719501	0.242619	2.133719	0.55282
LYPLA2	2.852575	1.22553	6.639728	0.015025
CLCN6	1.058114	0.236201	4.740057	0.941145
MRC2	0.580558	0.137196	2.456698	0.460037
TSPAN9	1.219777	0.93147	1.597319	0.148745
BTBD7	0.485626	0.100489	2.34685	0.368842
APBA3	1.217603	0.558889	2.652683	0.620206
MKS1	0.377566	0.114691	1.242955	0.109111
ABHD5	0.905438	0.632966	1.295201	0.586552
AKAP8L	1.18016	0.587161	2.372056	0.641882
MBTD1	0.943495	0.477432	1.86452	0.867086
UTP18	0.446834	0.188145	1.061204	0.067947
RNF216	0.867021	0.198848	3.780404	0.849366
TTC19	0.68761	0.355904	1.328468	0.264995
PTBP1	1.050708	0.401054	2.752711	0.91982
DPF1	3.255475	0.72579	14.60218	0.123213
LARS2	0.479548	0.184715	1.244981	0.131094
PIK3C2A	0.878782	0.231306	3.338689	0.849513
PLAUR	1.214456	0.925233	1.594088	0.161507
ANLN	0.638876	0.359105	1.13661	0.127429
WIZ	1.429746	0.216009	9.463391	0.710829
RABGAP1	1.731309	0.916753	3.269617	0.090641
QPCTL	0.682988	0.185591	2.51345	0.566276

PPP5C	0.391789	0.029609	5.184115	0.477013
CEP68	0.896547	0.459733	1.748395	0.748618
MAP4K3	0.848304	0.378176	1.902869	0.6898
ZBTB32	3.200424	0.607967	16.84749	0.169836
TYROBP	1.560008	0.95232	2.555469	0.077404
TMEM159	0.660575	0.197489	2.209536	0.5009
BRCA1	0.192213	0.044622	0.827974	0.026876
ERCC1	2.215896	0.784387	6.25991	0.133192
CD22	1.829425	0.51503	6.498257	0.350325
SEMA3B	0.205393	0.009522	4.430203	0.312448
MBTPS2	0.381518	0.053384	2.726593	0.336893
PRICKLE3	1.253875	0.272214	5.775606	0.77158
LTF	0.943535	0.779866	1.141553	0.549872
EXTL3	2.504054	1.083032	5.789571	0.031834
ELOVL5	0.680522	0.389448	1.189142	0.176494
ALOX5	1.64896	0.873605	3.112469	0.122815
CALCOCO	1.363469	0.477968	3.889477	0.562126
UBR7	0.122569	0.02157	0.696488	0.017884
MAP4K5	0.334987	0.156549	0.716815	0.004836
EHD3	0.69943	0.275003	1.778899	0.452898
PSMC4	0.822739	0.306571	2.207968	0.698472
MAN2B2	1.505557	0.602567	3.761739	0.381164
SLC25A39	1.051893	0.75126	1.472829	0.768309
MVP	2.171849	1.358058	3.473289	0.001206
NUB1	0.517102	0.119904	2.230081	0.37647
PGM3	1.732059	0.333	9.009082	0.513802
RWDD2A	3.128947	1.303926	7.508328	0.010644
CLK1	0.85338	0.499201	1.458846	0.562218
POLR3B	0.88675	0.364513	2.157195	0.791021
ANGEL1	0.810678	0.327235	2.00834	0.650226
RNF14	1.784128	0.831022	3.830358	0.137509
DNASE1L1	4.032052	0.58997	27.5564	0.155071
DDX11	0.213078	0.018629	2.437229	0.213693
HEBP1	0.783461	0.360577	1.702299	0.537662
GPRC5A	1.95201	0.134898	28.24618	0.623707
MAMLD1	0.622036	0.214582	1.80317	0.381961
CD6	1.052619	0.795893	1.392156	0.719212
TACC3	1.351799	0.577644	3.163468	0.487135
POLA2	0.418776	0.203886	0.860155	0.01778
ZC3H3	1.045578	0.462516	2.363668	0.91471
CAPN1	2.158934	0.816132	5.71108	0.120997
MDH1	0.392424	0.171206	0.89948	0.027085
SLC30A9	0.524086	0.253916	1.081723	0.080551
MTMR11	1.581615	1.027633	2.434242	0.037173
COX15	0.296063	0.077289	1.134104	0.075681
CCDC88C	1.864679	0.898536	3.869659	0.094378
YAF2	0.492895	0.076142	3.190672	0.457838
ZMYND11	0.222592	0.062184	0.79678	0.020937
WAS	1.77737	0.934283	3.38125	0.079637
DPEP1	0.714696	0.067138	7.608082	0.780737
BID	1.506899	0.491487	4.620148	0.473163
MATR3	0.407402	0.148139	1.120411	0.081913
NPC1L1	0.921575	0.060401	14.06098	0.953159
XYLT2	0.72123	0.263846	1.971498	0.524157
NUDCD3	0.83114	0.382226	1.807292	0.640731
GLT8D1	0.350874	0.093205	1.320875	0.121502
ATP2C1	0.386978	0.194433	0.770197	0.006861
SLC38A5	1.08847	0.614158	1.92909	0.77156

RALBP1	0.810526	0.347544	1.890269	0.626806
RUFY3	0.664875	0.153669	2.8767	0.584977
SLC11A1	1.164147	0.789792	1.715941	0.442597
WWTR1	0.361396	0.024671	5.294017	0.457406
AGPS	0.543149	0.280232	1.052738	0.070646
TTC27	0.833723	0.587688	1.182762	0.308099
ZNF582	0.384019	0.036958	3.990184	0.422946
VSIG2	0.581485	0.072736	4.648678	0.609219
PHLDB1	2.048608	0.781601	5.369484	0.144634
MARCO	1.518639	1.075184	2.144996	0.01772
PRDM11	0.042033	0.001443	1.223997	0.065408
CD74	1.188955	0.924246	1.529479	0.17801
HGF	0.501808	0.26964	0.933879	0.029568
ZRANB1	0.71654	0.314385	1.633123	0.427769
NCDN	0.943159	0.205999	4.318229	0.939903
ZFP64	0.586858	0.03727	9.240772	0.704727
MNAT1	0.340666	0.145832	0.795802	0.012861
SAMD4A	1.345395	0.820943	2.204889	0.239138
RUNX3	0.846484	0.457447	1.56638	0.595576
PLEKHB1	0.581576	0.184642	1.83182	0.354489
SERPINB1	0.644388	0.37733	1.100458	0.107531
SPAST	0.819146	0.345444	1.942426	0.650661
OSBPL5	0.701165	0.426961	1.15147	0.160707
AQR	0.480923	0.128778	1.796012	0.276187
FHL1	1.664226	1.159872	2.387893	0.005691
NLRP2	1.56196	0.982002	2.484433	0.059665
SLC45A4	2.524799	0.752238	8.474194	0.13384
RNF10	0.752886	0.443209	1.278938	0.293758
ZNF839	1.374597	0.48705	3.879515	0.54783
ZDHHC6	0.401396	0.185885	0.866764	0.020125
GRAMD1B	2.255639	0.897483	5.669082	0.083642
RNH1	1.988806	1.230041	3.215625	0.005039
NDUFS1	0.865991	0.257731	2.909776	0.816007
RB1CC1	0.906666	0.451203	1.821895	0.783175
ERP44	3.508243	0.56172	21.91085	0.17931
ALAS1	0.843816	0.184121	3.86717	0.826932
BIRC3	0.777809	0.362928	1.666963	0.518231
AKAP11	0.734345	0.207176	2.602919	0.632468
GLRX2	2.468742	0.783347	7.780318	0.122821
SNAPC1	0.764625	0.31807	1.838118	0.548712
DERA	0.408162	0.213505	0.780292	0.006722
STRAP	0.330333	0.157001	0.695026	0.003517
ABCC2	0.065268	0.008544	0.498597	0.008518
DEF6	0.733735	0.321589	1.674085	0.461943
PLEKHO1	1.397037	0.729966	2.673701	0.312703
GCLM	0.972086	0.456542	2.069801	0.941471
UBR2	0.684293	0.335523	1.395604	0.296813
EHD2	2.726793	1.45761	5.10109	0.001695
DEPDC1	0.020999	0.000507	0.870555	0.042062
CCDC28A	0.509622	0.184572	1.407121	0.19331
RRAGD	1.035378	0.678242	1.580566	0.872029
HSF2	0.504186	0.231596	1.097614	0.084472
PHF20	1.168809	0.436522	3.129543	0.756248
NR1H3	1.782245	1.157171	2.74497	0.008731
TYMP	1.350398	1.08242	1.68472	0.007774
NCAPH2	1.67614	0.338668	8.295583	0.526735
TOMM34	0.398029	0.179441	0.882892	0.023428
SEC63	0.373188	0.113896	1.22278	0.103566

KPNA6	1.195238	0.581303	2.457573	0.627727
VIM	1.898055	1.008078	3.573743	0.047157
FAS	0.923563	0.406395	2.098868	0.849428
RNASET2	0.643722	0.314244	1.31865	0.228613
CD44	0.973442	0.589932	1.606269	0.916107
AGPAT4	3.021178	1.235157	7.389762	0.015403
SLAMF7	2.615552	0.848372	8.063808	0.094186
BTN3A1	0.963799	0.30785	3.017405	0.949509
MIPEP	0.510796	0.266365	0.979532	0.043154
PRKCH	1.207464	0.901891	1.616571	0.205393
IFNGR1	1.222898	0.863278	1.732327	0.257415
B4GALT7	0.710418	0.286676	1.760505	0.460262
SH2D2A	1.131522	0.302187	4.236919	0.854459
VRK2	0.800882	0.33758	1.900028	0.614438
TNFRSF1B	1.210557	0.965231	1.518236	0.098188
VEZT	0.392681	0.118609	1.30006	0.125929
POU2F2	1.572074	0.978211	2.526465	0.061629
BRD9	0.346615	0.051472	2.33412	0.276212
SNX1	0.644758	0.237203	1.752561	0.389665
TBPL1	0.765726	0.367413	1.595853	0.476191
ARNTL2	0.567444	0.060564	5.316588	0.619652
BCLAF1	0.631264	0.302974	1.315275	0.219346
SLC39A9	0.733594	0.141383	3.806407	0.71229
ANK1	0.853452	0.544928	1.336655	0.488754
TFB1M	0.484048	0.193382	1.211608	0.121158
RABEP1	0.804148	0.488137	1.324739	0.392095
HMGB3	0.635879	0.341929	1.182533	0.15263
NUP160	0.833703	0.443953	1.565617	0.571603
BAK1	2.267952	0.990061	5.195241	0.052826
IKZF2	0.100678	0.007398	1.370143	0.084789
GRN	1.256457	0.959833	1.644749	0.096596
FAM13B	0.533361	0.277481	1.0252	0.059387
CENPQ	1.110682	0.647474	1.905275	0.703012
RANBP3	1.527264	0.294007	7.933595	0.614433
ARID4A	0.480972	0.161272	1.434438	0.18923
PNPLA6	1.732621	1.027305	2.922184	0.039305
IFT88	0.951982	0.125162	7.240755	0.962086
ALG1	1.289408	0.625244	2.65908	0.491263
ZCCHC8	0.945408	0.486289	1.837993	0.868546
ABCF2	0.301787	0.056428	1.614008	0.161398
CHPF2	0.928858	0.450231	1.916301	0.841692
LRRC7	0.751063	0.070884	7.958009	0.812116
FUT8	0.244484	0.05285	1.130972	0.071471
UBA6	0.657319	0.367067	1.177082	0.158098
GAB2	1.057023	0.354779	3.149276	0.920693
ATP6V0A1	1.083064	0.751344	1.56124	0.668889
PIAS1	1.00616	0.507102	1.996359	0.985984
SLC4A7	0.519092	0.251612	1.070923	0.075977
APBA2	0.663887	0.190784	2.310182	0.51966
MAP2K3	1.992996	0.763884	5.199784	0.158691
TMSB10	2.079613	0.631614	6.847208	0.228495
ASTE1	1.006715	0.333924	3.035047	0.990516
RNF19A	0.409714	0.120275	1.395678	0.15362
PEX3	0.148824	0.023467	0.943819	0.043247
GABARAPI	1.446281	0.788974	2.651202	0.232715
SH3YL1	0.649831	0.283536	1.489335	0.308376
FAM136A	0.404075	0.227861	0.716563	0.001934
VCL	1.349291	0.576677	3.157027	0.489729

DEPDC1B	0.890459	0.375458	2.111865	0.792312
DAPK2	0.709681	0.204543	2.462309	0.588993
NSMAF	0.599314	0.337282	1.064917	0.080894
STAP1	0.72545	0.489036	1.076154	0.110668
TIMP2	3.157841	1.489247	6.695972	0.002713
RFC1	0.573861	0.202207	1.62861	0.296703
TBC1D23	1.064387	0.483867	2.341388	0.876714
CUL3	0.112579	0.009088	1.394586	0.088954
MYOM2	1.049907	0.682595	1.614875	0.824549
CYP46A1	2.410776	0.590756	9.837981	0.220054
ZZZ3	0.555608	0.257865	1.197141	0.133475
USP2	0.864063	0.012698	58.79892	0.9459
TUBG2	1.171182	0.638237	2.149148	0.609936
RPL26L1	0.995596	0.363119	2.729713	0.993156
FLT4	7.377115	0.06407	849.4133	0.409231
NSUN2	0.464703	0.234082	0.922537	0.028494
FBXO42	3.194544	0.738062	13.82691	0.120264
MFAP3	1.801436	0.577874	5.615703	0.310288
MRI1	0.524571	0.202409	1.3595	0.184221
METTL1	0.348108	0.087932	1.378098	0.132804
AGA	0.387936	0.18765	0.801991	0.010605
PI4K2B	0.607162	0.202374	1.821607	0.373408
MAT2B	0.576952	0.22633	1.470746	0.249334
EDC4	0.516482	0.216482	1.232219	0.136413
TRIO	0.788706	0.22906	2.715688	0.706717
VCAN	1.091844	0.945258	1.26116	0.232248
CLEC16A	0.896809	0.338367	2.376909	0.826649
MSR1	1.550418	0.508157	4.730424	0.441
CDH1	1.149057	0.838111	1.575366	0.388131
ZFYVE16	1.224743	0.331223	4.528664	0.761241
RAI14	2.052533	0.734958	5.732153	0.169974
PNKP	1.065735	0.561085	2.02428	0.845784
BEST2	0.72152	0.069894	7.448285	0.784051
PHLPP2	1.409608	0.624311	3.182699	0.408686
STAU2	0.459139	0.227668	0.92595	0.029635
CTNS	2.905369	0.888372	9.501836	0.077702
RTN4R	1.640798	1.11731	2.409553	0.011545
PHF23	1.344387	0.710086	2.545291	0.36351
INPP4A	0.107352	0.004482	2.5712	0.168457
RAB27B	5.163294	0.84628	31.5021	0.075227
PSMA4	0.763917	0.390008	1.4963	0.4324
MYO16	0.31706	0.031906	3.150729	0.326878
LSG1	0.712786	0.211443	2.402837	0.58502
PARP3	1.932673	0.856556	4.360746	0.112506
TNC	0.490428	0.108799	2.210684	0.353729
THAP3	1.802508	0.313536	10.36256	0.509101
TDP1	0.703288	0.241135	2.051194	0.519248
AIFM2	1.735723	0.316416	9.521438	0.525458
SPATA7	0.498556	0.166406	1.493682	0.213771
MED17	0.474104	0.094797	2.371126	0.363492
RETSAT	1.555086	0.761359	3.176283	0.225621
CAPG	0.859218	0.585293	1.261343	0.438555
AP2S1	1.523753	0.730412	3.178786	0.261597
TG	0.459605	0.028523	7.405974	0.583596
ADAM28	0.326892	0.107809	0.991184	0.048199
DCUN1D1	1.288088	0.428907	3.868372	0.651839
LCP2	1.019784	0.456449	2.278371	0.961903
TRIT1	0.485014	0.194818	1.207482	0.119986

ADRB1	2.512268	1.267799	4.978306	0.008291
CUL7	1.474194	0.365817	5.940801	0.585211
CTNNA1	1.404399	0.978198	2.016296	0.065695
PHKA2	0.564766	0.271706	1.173916	0.125905
CNTLN	0.490424	0.138516	1.736375	0.269362
HSPA5	0.776331	0.515135	1.169965	0.22634
DSG2	0.907392	0.519822	1.583928	0.732424
GEMIN8	0.654751	0.208641	2.054719	0.467966
OFD1	0.638105	0.297622	1.368105	0.248292
GPM6B	1.061235	0.473867	2.37666	0.885123
PREX2	0.011561	9.08E-05	1.47208	0.071294
WDR37	1.039647	0.447718	2.414166	0.927925
YTHDC2	0.594703	0.227144	1.55704	0.289925
CTPS2	0.676873	0.244856	1.87113	0.451889
ATP6V1H	0.364424	0.06198	2.142715	0.264071
POLR2B	0.883243	0.477495	1.633774	0.692369
ARAP2	1.245499	0.398136	3.896324	0.705968
TPR	0.358478	0.105924	1.213192	0.099091
CP	1.147638	0.10792	12.20421	0.909106
DTNBP1	0.694273	0.136815	3.523108	0.65971
XK	0.813818	0.602562	1.099141	0.179106
SCML1	0.604639	0.073428	4.978867	0.639986
WWC3	2.070695	1.071295	4.002427	0.030404
ARHGAP6	9.864663	0.449228	216.6196	0.146431
FAM184B	0.104415	0.008684	1.255432	0.074965
MAP4	0.052856	0.001223	2.283666	0.125969
GOPC	0.540818	0.241632	1.210452	0.134829
USP28	0.107723	0.010045	1.155231	0.065658
HDAC9	0.224055	0.013405	3.744821	0.297853
TSPAN17	1.003043	0.624581	1.610834	0.989969
NOP16	0.662141	0.315385	1.390144	0.275943
CC2D2A	10.48861	0.957761	114.8627	0.054277
RRM2B	0.913413	0.343028	2.432231	0.856174
ZNF800	0.550819	0.312604	0.970562	0.03908
TNFRSF17	1.472149	0.877265	2.470432	0.143144
SNX29	1.171253	0.508691	2.696792	0.710272
MRPS10	0.429289	0.199485	0.923827	0.030573
RSF1	1.086404	0.332069	3.554298	0.891002
VPS13D	0.473495	0.025111	8.928101	0.617821
FAM120A	0.806325	0.3676	1.768662	0.591171
R3HDM1	0.787965	0.294516	2.108166	0.635073
COL9A2	0.890207	0.616927	1.28454	0.534196
KITLG	1.056686	0.128887	8.663276	0.959035
ERCC8	0.021024	0.001137	0.388884	0.009475
ADAMTS6	1.472182	0.307166	7.055855	0.628601
H6PD	0.103545	0.014738	0.727461	0.022616
VAMP3	1.109884	0.455226	2.706005	0.818652
PER3	1.218145	0.606507	2.446596	0.579171
UTS2	1.00549	0.614012	1.646564	0.982643
TNFRSF9	0.92654	0.226393	3.791982	0.915488
LTBP1	1.075702	0.313777	3.687764	0.907583
RCN1	0.641212	0.243255	1.690213	0.368849
ELN	0.425386	0.012009	15.06801	0.638626
RFC2	0.384512	0.114904	1.286719	0.120925
ARID1B	0.196933	0.004351	8.913617	0.403524
CLPTM1L	0.951109	0.405538	2.230636	0.908242
NEDD4L	0.599513	0.121846	2.949763	0.529117
FOXP3	0.45562	0.095433	2.175243	0.324331

PPP1R3F	0.70136	0.211645	2.324207	0.561712
HEXB	1.119621	0.777662	1.611948	0.543427
PTCD2	0.300129	0.101578	0.88678	0.029455
DKK3	1.429137	0.029216	69.90914	0.857229
ARHGEF5	1.010795	0.382538	2.670865	0.982721
NFE2L3	1.795969	0.730754	4.413938	0.201862
LIMA1	0.863873	0.447103	1.669136	0.663238
LETMD1	0.685568	0.254071	1.849893	0.456036
SLC4A8	0.173919	0.010426	2.901117	0.223152
LAMC3	2.221313	0.698523	7.063807	0.176338
PTGER3	0.099699	0.002307	4.307986	0.23018
MAPK9	0.660667	0.214552	2.034381	0.470085
COL23A1	0.710383	0.458667	1.100241	0.125527
BCAR1	0.159917	0.011542	2.215695	0.171694
FAM160A2	1.336636	0.73549	2.429125	0.341101
HERPUD1	1.131197	0.737998	1.73389	0.571578
HOMER3	1.244712	0.947869	1.634518	0.115302
RAD51	0.318226	0.063744	1.588653	0.1628
POLQ	0.523254	0.283492	0.965794	0.038334
PIK3CB	0.757838	0.434527	1.321707	0.328522
CYBA	1.578897	0.854956	2.915841	0.144489
THOC3	0.477531	0.171833	1.32708	0.156387
HEBP2	1.431564	0.93042	2.202635	0.102697
MPHOSPH	0.174252	0.06075	0.49982	0.001155
PLEKHA5	0.783359	0.416129	1.474664	0.449353
PRSS8	0.238304	0.042318	1.341952	0.10386
RRP12	1.015668	0.606415	1.701115	0.952886
FNIP2	1.050999	0.267684	4.126505	0.943174
TTC17	0.572081	0.314713	1.039922	0.067014
ALX4	1.822897	0.261979	12.68403	0.544095
FOXN3	0.063564	0.002658	1.520008	0.088861
AKR7A2	1.356953	0.659792	2.790759	0.406716
MRTO4	0.571486	0.262408	1.244616	0.158856
NNAT	1.433632	0.153027	13.43098	0.752343
USE1	1.395069	0.645496	3.015071	0.397145
MCF2L2	0.417691	0.041487	4.205323	0.458737
NRIP2	0.269923	0.016758	4.347764	0.35572
LAMA3	37.49755	2.087419	673.5908	0.013919
ANAPC4	0.442842	0.241598	0.811717	0.008421
KCNQ1	9.442302	1.303	68.42444	0.026291
TRAPPC3	0.233607	0.093609	0.582977	0.00183
THRAP3	0.572541	0.184836	1.773479	0.333672
PHPT1	1.231296	0.642466	2.359798	0.530725
ENTPD2	0.580224	0.017209	19.56345	0.761688
LY75	1.051968	0.53757	2.058591	0.882417
ARID4B	1.154369	0.5159	2.582996	0.726831
OPN3	1.522778	0.634474	3.654765	0.346474
SDCCAG8	0.409383	0.190112	0.881555	0.022484
HHAT	0.279019	0.111049	0.701056	0.006617
KIF1B	1.644266	0.569043	4.75115	0.358325
FOXC1	1.008357	0.89281	1.138859	0.893379
TBC1D22A	3.302917	1.126076	9.68786	0.029538
SYNE2	0.638382	0.073066	5.577545	0.684866
PLEKHH1	0.096756	0.009768	0.958402	0.045904
ATP9A	1.056699	0.497653	2.243758	0.885858
FAM168A	0.038247	0.002209	0.662317	0.024888
RELT	0.913326	0.089831	9.285917	0.938926
GALC	0.636737	0.406818	0.996597	0.048282

NOP58	0.420669	0.22446	0.788392	0.006896
KCNH2	0.509757	0.240844	1.078924	0.078172
CUL1	0.602329	0.214264	1.693241	0.336396
FAM114A2	0.545828	0.126988	2.346114	0.415771
CYFIP2	0.786902	0.460351	1.345091	0.380955
EIF2AK2	0.92787	0.419177	2.053891	0.853497
USP36	0.855876	0.498741	1.468746	0.57219
PUM2	0.797326	0.457056	1.390922	0.425015
MRPL43	1.077435	0.352303	3.295077	0.895958
ITIH4	0.113977	0.033468	0.38815	0.000513
ZFR	1.117158	0.598354	2.085794	0.728004
ZNF280C	0.166189	0.0264	1.046155	0.05589
TRAF1	1.066832	0.141472	8.044905	0.949957
RC3H2	0.39678	0.170521	0.923254	0.03193
IL17RB	0.147939	0.007543	2.901339	0.208218
TRAF3IP2	2.330608	0.479108	11.33717	0.294496
GYG2	0.485121	0.152767	1.540536	0.219832
DCBLD2	0.926705	0.46007	1.866634	0.831286
SOAT1	0.949412	0.388263	2.321582	0.909405
PKP2	1.086435	0.455927	2.588882	0.851563
MSH4	6.045739	0.624854	58.4952	0.120213
GDI2	1.214138	0.596927	2.469533	0.592209
PRDM1	1.993453	0.946198	4.199815	0.0696
ATG5	1.746824	0.684518	4.457727	0.243222
TMCC3	0.320689	0.045384	2.266015	0.254289
MTA3	2.028209	0.913784	4.501754	0.082153
USP13	0.551497	0.241434	1.259758	0.157934
ATP11B	0.645121	0.339266	1.226713	0.181296
SEC61A1	0.949954	0.340283	2.651947	0.921919
PPP1R12A	0.827444	0.40849	1.676085	0.598933
CROCC	3.239816	1.082093	9.700099	0.035643
POLR3E	0.330154	0.130255	0.836835	0.019526
ATP2B4	0.598133	0.323099	1.107287	0.101918
ZC3H11A	1.253362	0.325725	4.822839	0.742559
RIOK2	1.021504	0.4863	2.145734	0.955194
YIPF1	1.909019	0.683758	5.32989	0.217096
DGKG	1.221824	0.480968	3.103852	0.673621
FLYWCH1	0.180929	0.040487	0.808538	0.025208
UNKL	1.903739	0.126013	28.76068	0.642116
TBXAS1	1.796922	0.956571	3.375524	0.068465
PARP12	1.557211	0.890285	2.723743	0.120525
ALDH18A1	0.348338	0.19005	0.638462	0.000646
TARBP1	0.482203	0.289301	0.803729	0.005139
MXD1	0.839231	0.586653	1.200555	0.33735
DNAJC25	0.359007	0.167055	0.771515	0.008677
SLC2A3	1.064601	0.815182	1.390334	0.645787
PSD	1.404029	0.187918	10.49018	0.740857
CTDP1	1.639092	0.66166	4.060431	0.285685
STYK1	0.85113	0.037232	19.45719	0.919586
WNK1	1.052065	0.555367	1.99299	0.876265
CCAR1	0.942306	0.394226	2.252363	0.893672
OGFR	2.189621	1.334908	3.591587	0.001909
GNA15	0.82351	0.565333	1.199591	0.311651
CREB3L3	0.155656	0.016962	1.428431	0.100035
PIGV	0.904391	0.409495	1.997394	0.803681
PTPRU	0.974302	0.024928	38.08034	0.988894
SNRNP40	1.059565	0.50152	2.238552	0.879495
COL11A1	1.174249	0.011688	117.9773	0.945551

QSER1	2.902083	0.179822	46.83565	0.452759
ACAA1	1.338062	0.575633	3.110331	0.498608
BCAT1	1.368824	0.998103	1.877239	0.051393
HDAC7	2.312903	0.731991	7.308178	0.153158
LZTS1	0.308798	0.026454	3.604542	0.348626
SPAG4	0.286968	0.048369	1.702566	0.16938
NCKAP1	3.82322	0.211726	69.0374	0.363669
MRPS35	0.718523	0.333674	1.547245	0.398303
TNK2	1.638919	0.321103	8.365098	0.552489
MON2	0.426536	0.184754	0.984729	0.045932
GPBP1	1.109791	0.555991	2.215208	0.767689
DGAT2	3.787514	1.037962	13.82061	0.04376
CS	0.339733	0.115553	0.998842	0.049754
LTK	0.465522	0.147442	1.469808	0.192431
MRPS24	2.065525	0.869739	4.905375	0.100235
ELMO2	1.86562	0.432837	8.041209	0.402833
APPBP2	0.631274	0.26592	1.498596	0.297005
POLD1	0.670503	0.380864	1.180408	0.165991
SEZ6	2.407609	0.392675	14.76178	0.342293
EIF4B	0.826436	0.516975	1.321143	0.425778
SLC6A16	1.373947	0.192984	9.781809	0.751075
SPHK2	0.990564	0.476243	2.060327	0.979757
RPL18	2.435458	0.173423	34.20229	0.509058
CA11	2.334625	0.901576	6.045496	0.080719
ISOC2	1.001596	0.585719	1.712755	0.995353
U2AF2	1.143586	0.432732	3.022169	0.786702
EPN1	1.627326	0.944219	2.804635	0.079551
MED29	0.547804	0.244392	1.227903	0.1439
ZNF275	0.424525	0.109338	1.648304	0.215749
MTMR1	1.323381	0.081244	21.55639	0.843986
GPC1	3.497885	0.781091	15.66424	0.101636
ADCK1	1.260345	0.446077	3.560978	0.662379
HAGH	0.789566	0.480831	1.296535	0.350459
RNF4	1.608544	0.540392	4.788036	0.393057
CASP8	0.412783	0.130941	1.301269	0.130932
TM7SF3	0.662466	0.419846	1.045288	0.076791
DLX3	1.457111	0.86175	2.463792	0.160095
SPA17	0.807875	0.424707	1.536736	0.515493
TSPAN32	0.410017	0.198695	0.846089	0.015859
ST3GAL6	0.6098	0.342564	1.085508	0.092743
ATP2C2	1.255696	0.499201	3.15859	0.628536
NGFR	2.558529	0.337622	19.38877	0.363274
TAF2	0.713698	0.357894	1.423228	0.33817
HIPK2	1.113778	0.746915	1.660832	0.597096
TNPO3	0.730186	0.310775	1.71562	0.470605
RFXANK	1.426826	0.562598	3.618625	0.454101
TMEM161/	1.081644	0.391867	2.985587	0.87958
LPAR2	1.02217	0.559803	1.866426	0.943096
CTSA	0.814339	0.544225	1.218516	0.317885
SLC12A2	0.612137	0.306943	1.220788	0.163459
SNX24	1.174375	0.41798	3.299577	0.7604
CNN2	0.802851	0.40972	1.573198	0.522313
ABCA7	0.936539	0.231293	3.792179	0.926788
DDX20	0.460181	0.140507	1.50716	0.199759
BTBD1	0.632857	0.26914	1.488102	0.294287
FAR2	1.131248	0.585678	2.185026	0.713498
POU1F1	0.201924	0.014713	2.771181	0.231222
CHI3L2	0.304375	0.026318	3.520161	0.340917

SBNO2	2.681896	1.473547	4.881123	0.001243
PMS1	0.52226	0.25131	1.085336	0.081763
HMG20B	1.186533	0.59882	2.351058	0.623981
CALCRL	3.093098	1.855586	5.155922	1.48E-05
TAF11	0.38825	0.030328	4.970263	0.467036
ANKS1A	1.204103	0.792584	1.82929	0.38403
AP3D1	2.556164	0.619268	10.55113	0.194473
ZNF76	0.954751	0.31749	2.871113	0.934304
SLC9A3R2	2.874893	0.914157	9.04113	0.070851
NTHL1	1.665479	1.062505	2.610643	0.026126
UHRF1BP1	0.723679	0.310929	1.684342	0.453055
GNAI3	1.152393	0.604925	2.195328	0.666212
IPO5	0.483361	0.25098	0.930903	0.029699
OAT	0.930122	0.592745	1.459525	0.752668
WDR3	0.321717	0.028287	3.658948	0.360589
PKN2	1.190004	0.42182	3.357141	0.742351
WDR18	0.944774	0.489164	1.82474	0.865675
TRAM2	1.278678	0.646719	2.528172	0.479685
NTN1	3.006917	0.916249	9.868003	0.069415
MCM10	0.537837	0.267331	1.082064	0.082064
DGKA	2.330581	0.571757	9.499854	0.237923
ERBB3	0.008334	7.38E-05	0.941404	0.047144
ANKRD44	0.916533	0.291462	2.882138	0.881473
ADAT1	0.609107	0.230893	1.606855	0.316496
PDIA5	0.538377	0.260255	1.113715	0.095006
TBC1D22B	0.802559	0.285591	2.255326	0.676514
NDUFB4	0.164058	0.01056	2.548651	0.196531
SPEN	1.176895	0.581246	2.382951	0.650892
MYLK	3.41921	0.65095	17.95989	0.146314
ZC3H15	1.012118	0.467293	2.192161	0.975631
MAP2K4	0.788105	0.263274	2.35917	0.670351
SLK	0.612423	0.307494	1.219736	0.163049
CYB5R4	1.005204	0.556579	1.81544	0.986269
COL17A1	0.875136	0.607236	1.261228	0.474428
GSTO2	1.355226	0.391227	4.694559	0.631572
SEC61A2	0.495357	0.157245	1.560485	0.230186
PRKCQ	0.82906	0.528113	1.301502	0.415237
TLE2	0.481755	0.104838	2.213783	0.347932
ASB1	0.457155	0.255496	0.817981	0.008369
FAM107B	1.01422	0.707364	1.454192	0.938779
ME1	4.596572	2.005209	10.53679	0.000314
TBC1D1	1.678419	0.483733	5.823642	0.414589
MTHFD2	0.479996	0.189856	1.213532	0.120902
SLC9A7	1.653856	0.513287	5.328864	0.399352
FOXJ2	0.405654	0.133883	1.229092	0.110655
YBX1	0.59319	0.265679	1.324433	0.202547
PDE4A	1.934292	0.761893	4.910773	0.165175
PPP2R5A	0.686482	0.377696	1.247719	0.217213
ELAVL1	0.32989	0.119259	0.912527	0.032655
TIE1	0.626739	0.336295	1.168026	0.141297
DIP2B	0.899365	0.420132	1.925246	0.784753
SMARCD1	1.481004	0.43393	5.054673	0.53065
NFYC	1.095244	0.494525	2.425681	0.82256
ZMYND12	0.408161	0.142562	1.168586	0.094984
SLC9A3	2.221769	0.606332	8.141177	0.228264
NGEF	5.390686	0.423213	68.66398	0.194413
ASPM	0.724434	0.465753	1.126787	0.152624
CD84	0.587513	0.271538	1.271174	0.176811

ELOVL1	1.705559	0.583745	4.983226	0.329083
SPI1	1.808254	1.199242	2.726543	0.004697
MPPED2	1.00823	0.243739	4.170561	0.990973
CLDN18	0.120246	0.001467	9.853421	0.34606
ZBTB11	0.930096	0.420561	2.056963	0.857975
ATXN3	0.272045	0.049096	1.507433	0.136181
GOLGA5	1.029483	0.452726	2.341006	0.944734
LRRC40	0.922874	0.271726	3.13439	0.897627
ISOC1	0.801203	0.466109	1.377203	0.422587
TRMT11	0.136052	0.016651	1.111659	0.062717
THUMPD1	0.431008	0.189945	0.97801	0.044099
KIF26A	1.11291	0.382136	3.24117	0.844495
ATG2B	1.184383	0.146959	9.545262	0.87372
ARFGEF1	0.550503	0.279742	1.083331	0.083947
ZFAT	0.014089	0.000237	0.838322	0.040899
MTFR1	0.632901	0.286101	1.400078	0.258804
STAG3	0.386354	0.110259	1.353802	0.137151
FECH	0.72152	0.401219	1.297524	0.275673
MYO9A	0.430161	0.209166	0.884648	0.02184
DDX3Y	1.018799	0.634519	1.635807	0.938552
PFKP	1.189428	0.815366	1.735096	0.367884
IDI1	1.321718	0.725631	2.407477	0.361927
SP100	1.232183	0.458514	3.311292	0.678907
KLF6	0.995666	0.671009	1.477402	0.982788
NEO1	2.116439	0.820738	5.457668	0.120848
TRAM1	1.090917	0.565025	2.106279	0.795452
PHKA1	0.844763	0.157331	4.535814	0.844039
TNFRSF1A	2.063095	1.220284	3.488007	0.006871
CACNB1	0.612996	0.011208	33.52568	0.810564
EVI5	1.173244	0.632925	2.174823	0.611882
STOML1	1.921824	0.768992	4.802919	0.162147
DHX29	1.013006	0.4707	2.180118	0.973639
DNTTIP2	1.130289	0.706785	1.807555	0.609161
TP53BP1	0.697004	0.394376	1.231854	0.214122
TRO	1.469559	0.853835	2.529296	0.164657
RRP15	0.414893	0.149021	1.155113	0.092192
RHOA	0.954907	0.330626	2.757944	0.93205
DHX8	1.125057	0.283418	4.46604	0.866963
PRKCZ	5.449128	1.589442	18.68139	0.006994
ZFY	0.766925	0.314433	1.870581	0.559669
IARS2	0.792525	0.449439	1.397511	0.421698
NAV3	0.764321	0.265179	2.202988	0.61875
IDH3G	2.541021	0.765647	8.433113	0.127591
ROGDI	1.249164	0.801666	1.946461	0.325558
PDZD4	1.073549	0.534201	2.157443	0.842032
ROCK1	0.933078	0.452004	1.926167	0.851419
CBFB	0.44733	0.135773	1.473809	0.186035
PDK3	0.448788	0.152969	1.316676	0.144568
HYAL2	4.925292	0.975555	24.86636	0.053606
HDAC4	1.444336	0.900657	2.316205	0.127073
RASSF1	0.359396	0.132449	0.97521	0.04451
FGFR3	0.775713	0.35545	1.692871	0.523572
IFI35	2.408247	1.257543	4.611893	0.00802
HEATR6	0.717466	0.326316	1.577485	0.40881
COASY	0.447195	0.152355	1.312612	0.142967
PLEKHH3	1.043659	0.693547	1.570512	0.837615
MEF2A	0.480672	0.086261	2.678447	0.403245
OTUD5	2.427143	0.923982	6.375689	0.071938

TFE3	5.395702	1.843699	15.79086	0.002094
TBC1D25	0.65227	0.089864	4.734418	0.67265
ACSL4	0.778068	0.109556	5.525847	0.8019
INPP5A	8.621583	1.801654	41.25747	0.006997
GPKOW	0.417868	0.161624	1.080369	0.071788
GRIPAP1	0.989326	0.210999	4.638723	0.989139
FTSJ1	0.530272	0.045329	6.203315	0.613184
PRR11	1.021706	0.452359	2.307647	0.958801
ATP11A	0.299971	0.007227	12.45175	0.526485
POLR1A	0.674352	0.102466	4.438064	0.681923
LAPTM4A	0.81014	0.367616	1.785359	0.601495
TTC7A	2.531479	1.173535	5.460756	0.017888
IP6K2	0.978006	0.427416	2.237858	0.958005
SRBD1	0.576291	0.345117	0.962315	0.035134
KIF2A	0.534334	0.238408	1.197583	0.12799
RASGRP2	1.141772	0.449121	2.902656	0.780627
PSME4	0.572327	0.318208	1.029382	0.062424
IFT80	0.14672	0.011542	1.865155	0.139019
SIRT2	5.840378	2.143451	15.9136	0.000559
PPP2R5B	1.605391	0.912947	2.823034	0.100236
PYGM	0.329955	0.040798	2.668501	0.2985
PITX1	1.165698	0.948856	1.432095	0.144282
MAST4	0.989343	0.578339	1.692433	0.968799
SDK2	1.54145	1.000776	2.374226	0.04959
NUP133	0.766857	0.402169	1.462245	0.420183
NUCKS1	0.449459	0.175599	1.150421	0.095368
VPS35	0.894157	0.440121	1.816583	0.757064
DNAJA2	0.464665	0.19322	1.117453	0.086912
BCL3	1.579904	1.111125	2.246458	0.010875
KCNAB2	3.033896	0.822418	11.19203	0.095631
GAL	0.856269	0.273073	2.684988	0.790151
CLEC2D	0.250969	0.041799	1.506873	0.130633
FUNDC1	0.477027	0.166886	1.363532	0.167185
RORA	1.349594	0.292899	6.218549	0.700515
DRD4	1.07975	0.577267	2.019619	0.810201
TGFBR3	0.995034	0.690476	1.433927	0.978696
PLA2G10	1.383699	0.294786	6.49495	0.680596
ATP1B3	1.088747	0.241999	4.898245	0.911762
NEDD4	0.234758	0.04676	1.1786	0.078348
PIGB	0.492057	0.266507	0.908492	0.023408
MAPK6	0.602393	0.321302	1.129395	0.113989
GNB5	0.425276	0.132848	1.361397	0.149789
RAB27A	0.387398	0.168802	0.889073	0.025262
LRP6	0.429571	0.060296	3.060417	0.398983
SCT	1.492464	0.405315	5.495599	0.547119
PHRF1	2.14998	0.716945	6.447378	0.171908
NUCB2	0.396116	0.249916	0.627844	8.12E-05
PFN2	0.103357	0.002165	4.934491	0.249869
SPTB	0.664064	0.143875	3.06503	0.599851
DAPP1	1.06576	0.496156	2.289291	0.870308
SLC44A1	0.769497	0.511297	1.158084	0.209017
SMG6	1.117355	0.197515	6.320945	0.900125
EXOC5	0.399629	0.094853	1.683693	0.211311
CLTCL1	0.616362	0.206676	1.838153	0.385385
FGF22	0.015083	0.000521	0.436628	0.014584
FSTL3	1.273351	0.749647	2.162914	0.371339
DGCR2	2.896821	1.33445	6.288409	0.007155
RNF126	1.308687	0.556383	3.078207	0.537587

MNT	2.201028	1.215106	3.986914	0.009249
ZXDC	1.239072	0.335288	4.579046	0.747888
JMJD6	0.92084	0.298189	2.843655	0.886013
POLB	0.598335	0.31517	1.135911	0.116337
ST6GALNA	1.063027	0.327543	3.450002	0.91895
WIPI1	0.86253	0.512036	1.452939	0.578328
GBA2	0.221724	0.028194	1.743708	0.152273
NDST1	1.245298	0.57067	2.717451	0.581624
ASNS	0.551256	0.302518	1.00451	0.051743
AP3M2	0.646758	0.351699	1.189361	0.160904
ST6GALNA	1.621798	0.785081	3.350264	0.191457
PABPC1	1.791267	0.647444	4.955852	0.261566
TESK2	0.686792	0.259097	1.820491	0.449998
CSNK2A2	1.930303	0.825971	4.511141	0.128886
EIF2B3	0.52816	0.219147	1.272904	0.154933
CAMK2A	0.934406	0.348104	2.508197	0.892874
TCOF1	0.106282	0.002742	4.119018	0.229625
CDC42	0.544154	0.137845	2.148088	0.385063
OSBPL3	0.854833	0.281595	2.595006	0.7819
SLC12A3	5.127879	0.297066	88.51624	0.260681
RAD18	2.744277	0.11765	64.01209	0.529859
ATP2B1	3.020092	1.117654	8.160804	0.029311
TRPM5	2.582051	0.199635	33.39592	0.467662
NCK2	1.170012	0.462673	2.95874	0.74011
MAP4K4	0.352784	0.097693	1.273952	0.111749
MGAT4A	0.478277	0.253372	0.902818	0.022885
RPL31	1.116448	0.293976	4.239991	0.871471
WDR1	1.388406	0.55199	3.49222	0.485617
SNX13	0.775669	0.378835	1.588193	0.487201
ARHGAP1	0.573693	0.320056	1.02833	0.062021
RPS6KA2	0.528003	0.197686	1.410252	0.202616
ING3	0.699814	0.230219	2.12728	0.529185
VASH1	1.089628	0.618276	1.920323	0.766547
SEL1L	0.707118	0.177656	2.814518	0.622915
TRIP13	0.384541	0.200088	0.739034	0.004141
ATP6AP1	0.535144	0.214986	1.332081	0.179044
TCF3	0.993302	0.580369	1.700037	0.980444
TRIB2	0.490571	0.24892	0.966814	0.039642
DAZAP1	0.93144	0.221629	3.914563	0.922761
MBD3	1.115656	0.534133	2.330296	0.770878
HLTF	0.474619	0.234937	0.958827	0.037787
FAM50A	1.139319	0.570621	2.2748	0.711599
FAM3A	0.785262	0.3852	1.600822	0.505918
CPSF1	0.580901	0.293617	1.149273	0.118688
CYBRD1	1.079534	0.745329	1.563596	0.685561
PDCD2	0.386161	0.130139	1.145856	0.086416
RDH11	0.828049	0.332759	2.060543	0.685
PRKACA	4.993059	0.208361	119.6511	0.321106
ACTN1	1.152186	0.693707	1.91368	0.584216
ZFYVE26	0.586879	0.249835	1.378618	0.221297
EPN2	9.326892	1.194718	72.81295	0.033201
PTPN18	0.766117	0.150894	3.889714	0.747918
LIMS2	0.375166	0.088949	1.582361	0.181865
LNX1	0.031392	0.002022	0.487481	0.013383
ALDH3A2	1.243294	0.650775	2.375291	0.509695
TFRC	0.582824	0.394813	0.860366	0.006591
SREBF1	3.364681	1.69753	6.66915	0.000509
TRPC5	0.737779	0.115579	4.709476	0.747796

AFF4	0.777533	0.386476	1.564281	0.480498
UBE2D1	1.610702	0.259103	10.01284	0.609137
MPP5	0.61576	0.155947	2.431332	0.488922
RHOBTB1	0.188667	0.018032	1.97395	0.163842
SMC1A	0.102362	0.016069	0.652059	0.015839
HSD17B10	1.613015	0.701732	3.707707	0.260222
MARK2	3.060868	1.244442	7.528601	0.014842
HMMR	0.821023	0.535925	1.257785	0.36487
CHFR	1.149126	0.452131	2.920591	0.770234
P4HA2	0.413083	0.008389	20.34067	0.656548
FCGR2B	2.067672	1.219295	3.506344	0.007023
NFATC3	0.261539	0.0443	1.544085	0.13876
TRNT1	0.269	0.057336	1.262051	0.09594
ACADVL	0.893918	0.354581	2.253621	0.812118
STK10	2.200364	1.037743	4.665513	0.039727
FBXW11	0.940086	0.082328	10.73461	0.960341
ACAP1	1.202933	0.759392	1.905534	0.431144
CRMP1	0.040096	0.00104	1.545569	0.084296
EVC	0.28521	0.005706	14.25511	0.529616
DERL2	0.878891	0.385359	2.004493	0.758933
SIDT1	0.704776	0.397385	1.249947	0.231382
NDE1	1.429191	0.534405	3.822171	0.476768
TMEM38A	2.23477	0.324354	15.39736	0.414158
AP1M1	0.734481	0.303251	1.778933	0.494148
PVR	9.674788	0.40189	232.9033	0.162018
XRCC1	1.260003	0.389835	4.072516	0.699408
SCARB1	1.581676	0.885722	2.824474	0.121197
CYP2W1	0.011066	0.000364	0.33672	0.009749
MCM2	0.621159	0.404293	0.954355	0.029766
PANX2	1.177127	0.860193	1.610834	0.30822
TP63	0.563311	0.212689	1.491943	0.248134
ALPK1	0.657786	0.325813	1.328009	0.24258
LLGL2	0.592586	0.010538	33.32408	0.799099
PDE8A	0.047559	0.002977	0.75979	0.031219
CLCN4	3.038152	0.322169	28.65073	0.331736
NLE1	0.911096	0.353409	2.348826	0.847198
SDHA	1.744226	0.592931	5.130996	0.312242
SMARCE1	0.560865	0.272118	1.156004	0.117091
GSDMB	0.410047	0.154282	1.089816	0.073855
KDM5A	1.816401	0.238589	13.82846	0.564411
ADAM11	2.204238	0.283885	17.11491	0.449753
PPP2R3A	1.224994	0.157199	9.545943	0.846393
FERMT2	0.295243	0.037437	2.328387	0.246934
DHRS9	1.242903	0.851364	1.81451	0.259993
CD5L	1.591067	0.30461	8.310617	0.581906
PTGS2	1.104216	0.862525	1.41363	0.431538
IGF2BP2	1.692187	0.985405	2.905908	0.056563
MAP3K13	0.002401	8.90E-05	0.064789	0.000334
ST6GAL1	1.135869	0.357347	3.61049	0.829053
TBX21	1.811556	0.627103	5.233164	0.27229
FRY	1.010128	0.409753	2.490179	0.982536
PICALM	0.991804	0.547281	1.797386	0.978357
NSF	1.12001	0.304758	4.11613	0.864484
CLASP1	0.415046	0.178302	0.966131	0.041361
MRPS34	1.559958	0.840924	2.893805	0.158417
NOTCH3	1.556598	1.101702	2.19932	0.012101
CLNS1A	0.505513	0.254058	1.005849	0.051974
TEAD2	1.466574	0.617177	3.484963	0.38587

EED	0.85543	0.236297	3.096791	0.811966
TSG101	1.494728	0.611637	3.652838	0.377973
ATP2A3	0.682878	0.397449	1.173286	0.167197
MGLL	0.590789	0.335081	1.041634	0.068914
BCS1L	0.677485	0.309369	1.483621	0.330264
NUAK1	0.423674	0.052857	3.395919	0.418688
DPP8	0.385627	0.104438	1.423891	0.152796
SLC24A1	0.635617	0.324283	1.245852	0.186913
ZNF532	1.930494	0.936821	3.978141	0.074578
SCARF1	0.868678	0.040394	18.68123	0.928344
LMAN1	1.00729	0.216095	4.695305	0.992621
ZZEF1	0.78397	0.376876	1.630797	0.514872
ENO1	0.954475	0.371978	2.449132	0.922797
ANO8	1.657128	0.913547	3.005947	0.096439
TUBE1	0.583694	0.177595	1.918406	0.375177
ARHGEF1(	4.110472	0.830521	20.34384	0.083207
TXK	0.677491	0.289048	1.587949	0.370306
TACR2	0.568865	0.058859	5.498001	0.625983
ACTR6	0.93481	0.481982	1.813079	0.84191
TIPIN	0.353042	0.139683	0.892298	0.027747
SRI	0.045962	0.007261	0.29093	0.00107
EIF4G3	0.364277	0.177487	0.747648	0.00591
NUP37	0.519884	0.248635	1.087051	0.08218
SEMA3A	1.799218	0.300847	10.76025	0.519796
GTSE1	1.050498	0.436218	2.529804	0.912518
SEMA3C	1.218587	0.293666	5.056618	0.7854
TTC38	1.422247	0.677472	2.985789	0.351909
ACAT1	0.810602	0.56044	1.172428	0.264789
GRAMD4	0.561119	0.339904	0.926304	0.023866
CELSR1	0.751202	0.040475	13.94202	0.847775
WNT8B	0.141639	0.00633	3.169097	0.21774
ZNF638	1.376274	0.412359	4.593404	0.603498
SLC25A40	0.257823	0.084487	0.786779	0.017254
ADD2	0.170437	0.024362	1.192399	0.074639
RASAL2	0.060736	0.002117	1.742812	0.101921
ZNF37A	0.072835	0.004941	1.073725	0.056372
MARK3	1.124986	0.369791	3.422462	0.835645
SLC25A3	0.157991	0.030342	0.822669	0.028392
FNDC3B	0.728348	0.437918	1.211392	0.222024
FOSL2	1.101475	0.761385	1.593473	0.607958
CACNG4	3.819077	0.486079	30.00611	0.202637
FRYL	0.201531	0.017661	2.299688	0.197211
TMEM131	0.413609	0.193847	0.882513	0.022419
FSCN1	1.457253	1.036412	2.048979	0.030338
ACTB	2.867824	0.964838	8.524144	0.058018
PLD1	0.61867	0.367535	1.041402	0.070719
WDR62	0.612391	0.208224	1.801056	0.372947
DLG1	0.355505	0.061317	2.061165	0.24876
RAB7A	0.569772	0.248122	1.308389	0.184763
BCAP29	0.398315	0.047879	3.313674	0.394437
SEC31B	0.224586	0.047827	1.054611	0.058413
SART3	2.271748	0.503154	10.25697	0.286019
ARHGAP1!	0.664108	0.301325	1.463668	0.310031
TUBA3D	1.869239	0.783793	4.45788	0.15836
EXOSC7	0.253342	0.092183	0.696252	0.007771
KIFAP3	0.537712	0.313691	0.921717	0.024044
MKRN2	0.655061	0.279713	1.534093	0.329896
MCM6	0.450426	0.278626	0.728155	0.001136

REXO2	0.490669	0.278566	0.864271	0.013702
RBM7	0.764566	0.352617	1.657776	0.496604
RBMS2	1.720235	0.863522	3.426909	0.122911
BAZ2A	1.906568	0.275415	13.19828	0.513303
PTPN23	2.398677	1.007905	5.708529	0.047956
MLH1	0.167996	0.065566	0.430448	0.000203
UNG	0.09986	0.026237	0.380071	0.000729
FMO4	0.781482	0.244683	2.495937	0.677294
KLHL20	0.658087	0.247952	1.746619	0.400816
SLC46A1	0.813456	0.145414	4.550523	0.814182
PLXNA2	0.433606	0.012165	15.45544	0.646734
SPAG5	0.511592	0.172448	1.517714	0.227046
ANKRD13/	0.335357	0.143457	0.783959	0.011677
TPD52	1.206036	0.067535	21.53718	0.898636
ACACB	0.673442	0.403466	1.124071	0.130402
TRAF4	4.445039	1.290178	15.31446	0.018096
PAG1	0.834056	0.418853	1.660843	0.605617
GPATCH1	0.263846	0.048309	1.44104	0.124007
ICAM3	0.855806	0.592343	1.236451	0.406868
NT5C2	0.622555	0.297436	1.303053	0.208552
MCAM	0.329917	0.023056	4.721006	0.414048
GPC4	0.964666	0.745819	1.247731	0.784068
MBNL3	1.064075	0.375758	3.013255	0.906908
RAP1GAP	1.08275	0.866823	1.352465	0.483568
XAB2	2.020431	1.042061	3.917373	0.037349
ARHGEF1	3.411021	1.220595	9.532289	0.019277
STXBP2	1.332081	0.874181	2.029832	0.182118
MAP2K7	3.358149	1.32826	8.49018	0.010473
DGKD	1.692136	0.427939	6.690967	0.453321
RARB	0.036928	0.000512	2.66112	0.13066
TOP2B	0.558598	0.364	0.857229	0.0077
TM9SF3	0.811591	0.409874	1.60703	0.549217
NFKB2	2.773738	1.268913	6.063164	0.010562
UBE2T	0.760031	0.471984	1.223872	0.258957
PPP1R12B	0.517936	0.083685	3.205576	0.47931
DNAJC10	0.979166	0.461748	2.076385	0.95622
GTF3C1	1.557008	0.802219	3.021958	0.190659
IL4R	1.748356	0.916741	3.334364	0.089875
USP33	0.240174	0.065591	0.879449	0.031244
SNRPA	1.023099	0.487123	2.148804	0.951905
SPAG6	0.996196	0.585178	1.695906	0.988798
EXOSC5	0.755649	0.459807	1.241839	0.26898
DYNC1I2	0.807757	0.478212	1.364399	0.424735
APBB1IP	1.979067	0.902129	4.341626	0.088567
LRCH4	0.693366	0.381328	1.260741	0.229972
FAM76B	0.592643	0.309123	1.136198	0.115153
SIRT6	5.745498	2.117466	15.58974	0.000597
POLD3	0.182198	0.02955	1.123396	0.066566
CAPZB	2.900515	1.220401	6.893625	0.015913
GPR137B	1.990583	0.85045	4.659203	0.112598
NAALAD2	0.426674	0.127816	1.424314	0.166089
SLC25A43	1.012671	0.435197	2.356412	0.976687
UBE2A	0.643275	0.252975	1.635745	0.354176
FGFR1	5.965042	0.16584	214.5547	0.328558
SMC1B	2.698608	0.424207	17.16727	0.292987
FBLN1	0.865252	0.080232	9.331195	0.905049
CST7	0.83808	0.614747	1.14255	0.263928
PIAS2	0.098836	0.016908	0.577743	0.0102

AMPH	1.108046	0.191823	6.400506	0.908715
ARAF	0.458545	0.151816	1.384989	0.166824
MCCC1	0.533318	0.266498	1.067279	0.075731
LAMP3	0.444477	0.021785	9.06859	0.598196
ACER3	0.72035	0.319132	1.62599	0.429717
UBE2K	0.120078	0.017238	0.836473	0.032333
PIK3C3	0.929685	0.464449	1.860945	0.836862
N4BP2	0.680819	0.288521	1.60652	0.38011
TULP3	4.039373	0.406135	40.17511	0.23359
SYNJ2	1.188564	0.401468	3.5188	0.755083
PPP2R5C	0.38162	0.074815	1.946601	0.246556
GNB1	0.711114	0.245199	2.062341	0.530297
HOXA9	1.203088	0.941036	1.538113	0.140186
EDN1	1.869862	0.484035	7.223411	0.364056
MLLT10	0.344138	0.153386	0.772113	0.009675
ZCWPW1	0.451779	0.238031	0.857468	0.015086
P2RY10	0.771863	0.383565	1.553251	0.467981
ITM2A	0.811682	0.636669	1.034803	0.092207
VDAC3	0.493679	0.233145	1.045357	0.065172
PCM1	0.491482	0.277646	0.870009	0.014774
TNRC6C	3.453895	0.330913	36.04993	0.300293
CBFA2T2	0.088693	0.005527	1.42317	0.087125
ITCH	0.337301	0.144404	0.78787	0.012046
PKD2L2	0.041458	0.002973	0.57816	0.01791
TP53INP2	0.938071	0.749101	1.17471	0.57752
SDF4	2.447249	0.971983	6.161663	0.05748
MYH7B	0.070229	0.00429	1.149747	0.062585
TP73	0.455643	0.028315	7.332182	0.579226
TOLLIP	1.942974	0.911004	4.143944	0.085656
UBE2D4	0.580829	0.212494	1.587629	0.289609
RUNX1T1	77.04468	1.252185	4740.419	0.038738
THOC1	0.420494	0.215609	0.820072	0.011022
FKBP7	0.051534	0.003165	0.839093	0.037233
OSBPL6	0.841873	0.235179	3.013671	0.791366
SLC1A3	1.311663	0.727934	2.363482	0.366519
XRCC5	0.725476	0.407823	1.290551	0.27482
LXN	0.605531	0.421286	0.870355	0.006726
SP140	0.605594	0.33271	1.102291	0.100743
MKNK1	1.785038	0.472534	6.743134	0.392837
TNS1	1.023278	0.753625	1.389413	0.882778
REXO1	1.101859	0.561841	2.160917	0.777742
SAR1A	0.421787	0.176074	1.010392	0.052773
CDC14A	0.487888	0.045139	5.27343	0.554571
RAPGEF3	1.336264	0.382141	4.67263	0.649936
CEACAM1	0.933276	0.717833	1.21338	0.606088
SENP1	0.046454	0.00328	0.657911	0.023235
DUSP13	0.957101	0.046617	19.65026	0.977313
CIC	2.924822	1.397281	6.122307	0.004406
LIPE	2.410672	0.774141	7.506819	0.128953
FDFT1	1.659412	0.695379	3.959924	0.253749
PAFAH1B3	1.024354	0.620627	1.690712	0.925015
OPHN1	1.381189	0.174561	10.92847	0.759597
KIF22	1.428857	0.693983	2.941906	0.332776
PGM1	0.821273	0.517751	1.302729	0.402887
DDX1	0.508521	0.282909	0.914053	0.0238
DNM2	2.629205	1.277756	5.410044	0.008646
EPB41L2	1.554321	0.841593	2.870644	0.158834
STX7	0.987039	0.358748	2.715683	0.979845

RABL2B	0.682413	0.235322	1.978941	0.481781
KEAP1	1.187214	0.380714	3.702198	0.76743
DDX43	2.602464	0.993946	6.814072	0.051463
PTPRH	4.511672	0.525972	38.70016	0.169435
SLC35C2	2.192137	0.48921	9.822901	0.30505
CRYBG3	1.17118	0.133389	10.28315	0.886643
RFX3	0.312239	0.041136	2.370029	0.260355
RIF1	0.242062	0.085129	0.688293	0.007801
RAB21	1.069054	0.473676	2.412781	0.872267
SLC4A4	0.828324	0.088385	7.762856	0.868965
SMARCA2	1.129351	0.356429	3.578367	0.836219
SESN1	0.921389	0.473652	1.792366	0.809435
MID2	1.472556	0.175716	12.34051	0.721245
SRCAP	2.834794	0.947858	8.478127	0.0623
KCNN2	0.731187	0.011121	48.0746	0.883449
CNOT4	0.29876	0.043745	2.040393	0.217779
PSEN1	2.00759	0.317785	12.68285	0.45867
CPOX	0.832401	0.52721	1.314263	0.431152
CLDND1	0.675256	0.328416	1.388394	0.285658
HSP90AA1	0.597865	0.240057	1.488988	0.269213
RBL1	0.297202	0.052759	1.674193	0.168919
DLGAP4	3.108448	0.819402	11.79208	0.095481
IGSF9B	8.732062	1.200682	63.50468	0.032304
NDC80	0.654084	0.330702	1.293691	0.222476
AP4E1	0.787623	0.293828	2.11127	0.635111
RSBN1	1.049811	0.561912	1.961346	0.878845
MAGI3	0.207024	0.004925	8.702512	0.408992
CXCL2	1.085193	0.89805	1.311334	0.397243
COL4A4	0.157053	0.008591	2.871133	0.211818
TCF7	0.563008	0.023525	13.47403	0.722892
OSTM1	0.958011	0.529247	1.734134	0.887331
IMPG2	0.113091	0.00939	1.361997	0.086046
PCNP	0.577089	0.327464	1.017004	0.057218
EXD2	0.676741	0.354972	1.290181	0.235601
ARG2	0.546132	0.176279	1.691977	0.294434
MEF2C	1.435123	0.902162	2.282935	0.127197
PTPRC	1.766674	0.652383	4.784206	0.262866
UBA5	0.350105	0.159031	0.770755	0.009143
STK17B	0.902798	0.662546	1.23017	0.517146
CDC14B	1.118102	0.324497	3.852588	0.859618
ZNF510	0.647111	0.183643	2.280252	0.498229
ZNF506	1.151866	0.047657	27.84062	0.930671
JMJD4	1.458147	0.721066	2.94868	0.293828
DUSP12	0.461917	0.20083	1.062426	0.069145
AACS	0.689815	0.289656	1.642792	0.401616
PHLPP1	0.985761	0.40501	2.399257	0.97479
ATP8B1	3.649187	0.553907	24.04118	0.178368
IL12RB2	1.58361	0.835696	3.000877	0.158659
SMARCD3	1.345142	0.799317	2.263689	0.264214
WDR70	0.495183	0.256814	0.954802	0.035902
STRADB	0.892422	0.610168	1.305243	0.55739
BZW1	0.737798	0.044481	12.23758	0.831947
C1QTNF3	0.020455	0.000472	0.885651	0.04306
ME2	0.507611	0.256943	1.002829	0.050958
CCNT2	0.172951	0.038891	0.769116	0.02118
FAM135A	0.561501	0.135308	2.330122	0.426678
COL19A1	1.632403	0.102249	26.06122	0.728819
EPB41L3	1.21899	0.967501	1.53585	0.093013

COBLL1	1.282701	0.902404	1.823266	0.165256
DLG3	0.440803	0.008031	24.19416	0.688529
TRAF5	0.450673	0.143345	1.416905	0.172658
MRPL22	0.6899	0.288333	1.650738	0.404315
GEMIN5	0.673968	0.220583	2.059234	0.488685
NFE2L1	2.050457	0.817755	5.141362	0.12577
GSK3B	0.395933	0.192821	0.812996	0.011605
ITGB5	1.258899	0.725241	2.185244	0.413213
ERC1	0.719209	0.069282	7.466019	0.782489
XPO1	0.687367	0.38443	1.229023	0.206078
RNF13	2.178901	0.934311	5.081405	0.071436
PALB2	0.637811	0.17888	2.274169	0.488117
LYRM2	0.732582	0.442144	1.213804	0.227097
BCKDHB	0.132663	0.035446	0.496513	0.002702
ULK2	1.077446	0.346974	3.345756	0.897337
TNPO1	0.337275	0.095317	1.193434	0.091854
PLOD1	1.608233	0.922767	2.80289	0.093665
P2RX5	0.173573	0.055052	0.547253	0.0028
ITGAE	0.600976	0.341596	1.057308	0.077291
DIS3	0.450435	0.103615	1.958136	0.287462
PIBF1	0.442734	0.15496	1.264929	0.128211
TDRD3	0.403254	0.079214	2.052838	0.274055
NUFIP1	0.28765	0.042412	1.950904	0.20205
PDS5B	0.393066	0.14325	1.07854	0.069808
OXCT1	0.995369	0.376757	2.629705	0.992528
RRAGB	0.22125	0.082906	0.590444	0.002595
CYLD	0.613314	0.152235	2.47088	0.491684
SLC27A5	1.059624	0.583524	1.924176	0.8491
ZNF324	1.658183	0.829017	3.316666	0.152771
ZNF671	1.037299	0.519271	2.072116	0.917385
ZNF416	1.202035	0.46251	3.124013	0.705714
ZNF586	0.632687	0.247132	1.619754	0.339858
ZNF446	0.864467	0.274402	2.723393	0.80355
ZNF264	1.810976	0.556609	5.892166	0.323836
RPS5	1.896863	0.576335	6.243045	0.2922
FAT1	1.271851	0.716352	2.258114	0.411628
YTHDC1	0.660449	0.215817	2.021124	0.467269
CHMP2B	1.000106	0.546308	1.830857	0.999725
SMAP2	1.324148	0.737982	2.375894	0.346543
PPIE	0.448771	0.091567	2.199436	0.323141
ZMPSTE24	0.959648	0.479718	1.919719	0.907311
STARD7	1.063606	0.316791	3.570994	0.920512
REST	0.552288	0.130541	2.336593	0.419825
HAL	0.910578	0.618468	1.340656	0.635055
SSH1	1.235138	0.699762	2.180123	0.466331
GSTP1	1.873989	0.785418	4.471293	0.156901
APLP2	1.1569	0.589202	2.271578	0.672033
WBP11	1.013472	0.257325	3.991547	0.984734
EIF3I	0.68542	0.39518	1.188829	0.178835
TXLNA	0.57601	0.232411	1.427594	0.233567
NCOA1	1.189953	0.411393	3.441934	0.748264
AGBL5	0.667135	0.27225	1.634777	0.376083
EFR3B	1.055629	0.253374	4.398063	0.940728
KIF3C	1.007383	0.417719	2.429437	0.986933
RAB10	1.136188	0.643339	2.006595	0.65995
HADHA	0.603119	0.283152	1.284652	0.189968
MAPRE3	1.302625	0.524091	3.237664	0.569266
CAD	0.792469	0.43621	1.43969	0.445107

CD59	0.735841	0.230683	2.347205	0.604254
CD82	0.892308	0.518118	1.536741	0.681202
BCORL1	1.299373	0.571125	2.956218	0.532361
ATRX	0.232717	0.007788	6.953982	0.400281
FCN1	1.006454	0.863173	1.17352	0.93456
MYNN	1.646903	0.492259	5.509882	0.418118
SCAMP1	0.379592	0.111379	1.29369	0.121535
PREP	1.34682	0.490736	3.696331	0.563246
HACE1	0.612901	0.144925	2.592003	0.505791
SEH1L	0.413005	0.176342	0.967285	0.041693
WDR47	1.074589	0.232436	4.967996	0.926627
WDFY1	0.900649	0.566479	1.431949	0.658264
OVGP1	0.920729	0.231813	3.656998	0.906571
SLC25A24	1.146817	0.526146	2.499662	0.7304
MAP3K4	0.202704	0.058531	0.701999	0.011794
PILRA	1.161002	0.876858	1.537223	0.297234
IGSF9	0.775057	0.085815	7.000133	0.820471
ABCB1	0.585154	0.346686	0.987652	0.044804
ZNF213	1.097823	0.533362	2.259656	0.799963
AKR1B1	1.443211	0.735774	2.830839	0.28583
CPNE3	1.094212	0.791292	1.513093	0.586141
RRN3	0.546421	0.259048	1.152588	0.112501
CTTN	0.71952	0.307047	1.686094	0.44869
WNT11	0.915935	0.107774	7.784201	0.935898
MTIF2	0.115046	0.028367	0.466579	0.002469
DDHD2	0.166447	0.044495	0.622638	0.007726
TTC39A	9.381847	0.817001	107.7343	0.072229
EPS15	0.6842	0.343949	1.361044	0.27947
MGST2	1.338176	0.791587	2.262182	0.276825
CHERP	2.028965	0.738624	5.573474	0.169962
ATG16L1	0.877827	0.40363	1.909124	0.742372
USP40	0.731375	0.044936	11.90373	0.826038
POMGNT1	0.854641	0.416894	1.752032	0.668024
RAD54L	0.465201	0.234155	0.924223	0.028893
MAST2	0.863276	0.251363	2.964817	0.815339
DNAJA1	0.501202	0.267057	0.940637	0.031516
B4GALT1	0.966819	0.488754	1.912495	0.922763
CHMP5	1.199301	0.622994	2.308726	0.586541
NFX1	0.342371	0.081823	1.432581	0.142179
IPO11	0.939961	0.586925	1.505347	0.796649
EIF2AK1	0.378912	0.161212	0.890594	0.026034
EPDR1	0.801435	0.551975	1.163636	0.244659
SNX10	1.172797	0.852253	1.6139	0.327823
SEPHS1	0.450151	0.173065	1.170865	0.101728
MRPL28	2.060973	0.91442	4.645137	0.081126
HBQ1	0.954083	0.771036	1.180586	0.665387
ITPKC	6.210398	1.320543	29.20696	0.02078
CEACAM6	1.032904	0.876769	1.216843	0.698629
FAT2	0.35398	0.02728	4.593216	0.427115
RBM22	0.891589	0.359347	2.212159	0.804524
TMED2	0.638867	0.315329	1.294366	0.213596
ZFAND6	2.755274	1.240995	6.117296	0.012755
PPEF1	0.183626	0.010789	3.125269	0.241202
LAT2	0.812987	0.538013	1.228498	0.325634
HUWE1	1.464244	0.631096	3.397281	0.374517
ZW10	0.333483	0.167325	0.664639	0.001803
ALG9	0.334395	0.119713	0.934066	0.036608
MYBPC2	1.942799	0.144434	26.13274	0.616495

ACOX3	1.246402	0.602741	2.577421	0.552379
MTMR2	0.292765	0.067166	1.276116	0.101973
PPP1R15A	0.90288	0.622419	1.309714	0.590356
HSD17B14	0.587175	0.117186	2.942117	0.517283
TRIP6	1.349197	0.943928	1.928467	0.100312
ACHE	1.469839	0.341057	6.334501	0.605337
FTL	4.623586	1.349611	15.83979	0.014802
BAX	0.849341	0.217599	3.31519	0.814194
NLK	0.929487	0.531165	1.626512	0.797855
PIGS	1.31565	0.503935	3.434839	0.575279
ADAMTS2	3.167832	1.069043	9.387048	0.037487
ATXN7L3	3.91146	1.489661	10.27047	0.005621
PGS1	0.808055	0.273406	2.388219	0.699893
PSMC5	0.318743	0.12033	0.844323	0.021424
UIMC1	0.734352	0.507352	1.062918	0.101724
CETP	0.823099	0.568741	1.191212	0.301964
MMP2	1.129068	0.367932	3.464761	0.831954
LPCAT2	0.928388	0.544323	1.583444	0.785027
OGFOD1	0.46602	0.141999	1.529412	0.207946
SH3BP2	3.708677	1.642836	8.372281	0.001606
NOP14	0.345333	0.113322	1.052356	0.061456
ADD1	1.730047	0.566326	5.285055	0.336026
L2HGDH	0.515238	0.158537	1.674508	0.270154
TXNDC16	0.639623	0.113327	3.610071	0.612785
NID2	0.129125	0.012776	1.305066	0.082852
GMCL1	0.361544	0.172016	0.759893	0.007264
SF3B2	1.296565	0.508304	3.307231	0.586706
GNAS	0.637672	0.030146	13.48848	0.772609
DNM1L	0.56597	0.235547	1.359909	0.203145
PHACTR3	0.79094	0.333552	1.875528	0.594457
ERGIC2	1.385426	0.751476	2.55418	0.29624
AURKA	0.876432	0.554529	1.3852	0.572241
CASS4	9.101769	2.759015	30.026	0.000287
PIR	0.54283	0.294667	0.99999	0.049996
RFX2	1.194012	0.659248	2.162563	0.558475
METTL2A	0.588693	0.144367	2.400549	0.459999
SULT2B1	5.082091	0.179773	143.6678	0.340341
ALG6	0.938308	0.411458	2.139762	0.879665
CNOT3	2.34235	1.195259	4.590304	0.013154
GP6	1.924939	0.615376	6.02134	0.260366
PTPN4	0.730811	0.340091	1.57042	0.421676
DDX18	0.411179	0.201846	0.837613	0.014362
KHSRP	0.638538	0.293859	1.387503	0.257274
GNA11	1.566102	0.983937	2.492718	0.058534
EDEM2	0.780365	0.277546	2.194122	0.638228
DNMT3B	1.235082	0.512185	2.978278	0.638254
TPX2	0.759308	0.457706	1.259647	0.286348
FER1L4	2.294772	0.177326	29.69664	0.524879
PDRG1	0.683611	0.31132	1.501105	0.343234
EPB41L1	120.732	2.335691	6240.642	0.017247
DOCK9	1.148804	0.262101	5.03528	0.854022
ANKRD10	0.42693	0.201777	0.903319	0.026023
TGDS	0.766217	0.274143	2.141541	0.611598
DOCK3	1.934132	1.000349	3.739562	0.049879
COQ9	1.375803	0.475405	3.981518	0.55623
TMEM40	0.103792	0.009728	1.107398	0.060723
KIF9	4.162963	0.171032	101.3277	0.381193
CRLS1	0.802308	0.364454	1.766198	0.584314

PPP1R13B	1.313307	0.521465	3.307558	0.563039
ATRN	2.713328	0.378179	19.46736	0.320804
SMOX	0.994868	0.630166	1.570637	0.982381
SIGLEC1	3.684121	0.765232	17.73677	0.103893
FKBP1A	2.292864	0.976019	5.386395	0.056876
NSFL1C	0.316181	0.138261	0.723055	0.006366
SLC4A11	1.072469	0.375664	3.061754	0.895999
ZNF343	0.748259	0.25694	2.179076	0.594893
EBF4	0.270353	0.026538	2.754151	0.269378
CPXM1	0.978769	0.724161	1.322896	0.888974
XRN2	0.787955	0.261167	2.377305	0.672311
DYNLL1	1.662005	0.892679	3.094348	0.109162
TESC	0.870359	0.647969	1.169076	0.356368
SNX5	0.543144	0.247562	1.191641	0.127859
RPL6	0.223774	0.06373	0.785734	0.019478
SIRPG	1.078012	0.32263	3.601986	0.902864
MAPKAPK	0.083173	0.024634	0.280829	6.19E-05
P2RX7	1.230536	0.762309	1.986358	0.395825
ESF1	6.032405	0.510623	71.2657	0.153732
RBBP9	0.37722	0.128135	1.110505	0.076777
ANAPC5	0.216122	0.082018	0.569499	0.001943
SLC23A2	0.235407	0.021214	2.612216	0.238805
KDM2B	12.10742	1.324753	110.6542	0.027169
TASP1	0.22867	0.08834	0.591917	0.002361
OAS1	2.055826	1.198082	3.527655	0.008898
RPLP0	1.127698	0.325969	3.901294	0.849479
PXN	1.728904	0.82424	3.626501	0.147464
SIRT4	2.122723	0.81646	5.518892	0.122586
KIF16B	1.534223	0.802697	2.932413	0.195316
TRMT6	0.676254	0.269782	1.695145	0.404096
PEBP1	0.963127	0.484523	1.91449	0.914645
BRAP	0.121798	0.013126	1.130208	0.063986
ERP29	0.881614	0.382657	2.031174	0.767311
FUS	0.378941	0.155347	0.924357	0.032937
IGBP1	1.4937	0.638497	3.494363	0.354785
FXYD5	0.77798	0.333313	1.815871	0.561566
ZNF302	0.625124	0.144211	2.709778	0.530124
GRAMD1A	0.748824	0.411071	1.364091	0.344516
CMTM1	0.169516	0.032345	0.888415	0.035733
KCNH4	1.153638	0.215876	6.16503	0.867262
GANAB	0.685467	0.343457	1.368045	0.284111
GMIP	1.697079	0.936666	3.074816	0.081124
RBM41	0.070651	0.003564	1.400422	0.08204
BIRC5	0.686944	0.317235	1.487518	0.340805
LAG3	1.288198	0.992409	1.672148	0.057078
MLF2	2.153223	0.970147	4.779038	0.059368
OTUB2	0.135624	0.004855	3.788901	0.239627
DDX24	0.757208	0.39522	1.450746	0.401826
ZBTB25	0.55935	0.182166	1.717511	0.310099
NECAP1	0.508249	0.26684	0.968058	0.039522
ARHGAP4	1.036496	0.51151	2.100296	0.920757
ANKRD24	0.845587	0.09351	7.646448	0.881324
DHX32	0.429095	0.247152	0.744977	0.002648
RCOR1	0.74426	0.123285	4.49301	0.747459
LTBP4	1.9875	1.154344	3.421992	0.013223
BLVRB	0.968546	0.716543	1.309177	0.835341
SLC9A1	1.243594	0.667219	2.317869	0.492561
SPTLC1	0.742178	0.176968	3.112581	0.683541

PAPOLA	0.692822	0.381865	1.256995	0.227268
CCNK	3.138684	1.132397	8.699545	0.027877
PCBP4	0.457097	0.027052	7.723454	0.587312
RGS1	0.986491	0.777644	1.251426	0.910773
YPEL3	1.852243	1.10985	3.091232	0.018334
MRPS33	0.982002	0.598936	1.610068	0.942607
NDUFB2	0.898739	0.33143	2.437113	0.833856
NUDC	0.433811	0.206812	0.909968	0.027134
MAEA	1.988637	0.917327	4.311084	0.081617
ICAM1	2.09127	0.851906	5.133679	0.107363
STRN4	3.554079	1.389324	9.091816	0.008143
IRAK3	1.095841	0.731619	1.641385	0.657048
LYZ	0.925216	0.738317	1.159428	0.499601
MUL1	1.361987	0.51885	3.575235	0.530379
TFAP4	1.873282	0.59168	5.930888	0.285754
PDCD7	0.788087	0.252161	2.463033	0.682098
SPG21	3.656594	0.716763	18.65425	0.118895
DNAJB11	0.360283	0.181838	0.713842	0.003431
FLT3LG	1.336198	0.864099	2.066227	0.192512
RAB11FIP3	0.666374	0.371491	1.195329	0.173357
GNPTG	0.247166	0.092555	0.660052	0.005289
ZNF268	0.03143	0.000281	3.509975	0.150408
GOLGA3	0.462641	0.215982	0.99099	0.047339
PABPC4	0.532116	0.256483	1.10396	0.090199
CD209	1.312166	0.38246	4.501854	0.665796
MCOLN1	1.853438	1.122382	3.060663	0.015904
USP48	0.327814	0.11045	0.972946	0.044497
EFNB1	2.575839	1.414698	4.690008	0.001971
PDPR	0.763307	0.337009	1.728849	0.517299
GLG1	1.191677	0.713403	1.990593	0.502926
KIF4A	0.719557	0.386111	1.340971	0.300099
TNRC6A	0.338838	0.030856	3.720826	0.376039
PLEKHG2	2.173562	0.882442	5.353748	0.091403
DLL3	0.95839	0.283144	3.243975	0.945532
NAT14	1.761046	1.141925	2.71584	0.010454
PITPNM2	0.010765	0.000991	0.116987	0.000197
EXOC1	1.03075	0.368357	2.884281	0.953997
RBM27	0.705687	0.287563	1.731769	0.446629
OSBPL8	1.264163	0.705001	2.266818	0.431428
DTX2	3.840736	1.25684	11.73678	0.018223
NLRC4	1.141465	0.688178	1.893321	0.608313
PUS7	0.339558	0.165322	0.697423	0.003269
NRCAM	0.565056	0.01726	18.49824	0.748428
LAMB1	0.446956	0.016116	12.39583	0.634768
DLD	0.542133	0.276593	1.062602	0.074567
WDR7	1.375486	0.308328	6.136191	0.676058
TXNL1	0.993567	0.586344	1.683611	0.980865
IL5RA	0.755328	0.293175	1.946005	0.561152
ABCC6	1.15437	0.074024	18.00175	0.918417
CMTM6	0.920785	0.568914	1.490288	0.736918
ITGA6	0.571195	0.148594	2.195679	0.41498
RAPGEF4	0.499226	0.098815	2.52215	0.400583
FH	0.88948	0.370158	2.137397	0.793454
SEL1L3	1.307428	1.001066	1.707547	0.049093
CDV3	0.446831	0.117571	1.698188	0.236978
MYO15A	1.230937	0.173969	8.709626	0.83513
ALKBH5	0.902317	0.281362	2.893693	0.862744
NLRP1	1.57819	0.582582	4.275245	0.36952

PITPNM3	2.337337	0.103471	52.79897	0.593497
SPAG7	0.449197	0.157392	1.282006	0.134737
ZC3HC1	0.646198	0.263543	1.584457	0.339981
ESR1	23.07545	2.082593	255.6795	0.010534
ANGPT2	0.924507	0.282708	3.023308	0.896688
TMEM101	1.750815	0.697225	4.396501	0.233163
CD200	0.910555	0.36125	2.295118	0.842536
CCDC80	0.016431	0.000323	0.835148	0.04038
CMA1	0.270339	0.018131	4.030877	0.342709
PSME1	0.480348	0.153355	1.504575	0.208137
PPP2R3C	0.651363	0.309556	1.370589	0.258718
HAUS4	0.685488	0.37703	1.246301	0.215686
JPH4	1.01584	0.124473	8.290392	0.988294
CEBPE	0.995027	0.730192	1.355916	0.974813
SLC7A8	9.668575	0.271844	343.8787	0.213078
OSGEP	0.24592	0.100464	0.601974	0.002132
SLC22A17	2.187514	0.507786	9.423683	0.293495
RNF31	1.229296	0.529813	2.852264	0.630708
SCFD1	0.56837	0.117423	2.75113	0.482563
HECTD1	0.845827	0.456935	1.565699	0.594066
HNRNPC	0.450731	0.198769	1.022083	0.056433
RPGRIP1	0.93236	0.365409	2.378968	0.883492
SUPT16H	0.657274	0.397437	1.086988	0.102051
TOX4	0.275154	0.085347	0.887078	0.030727
TGM1	1.389972	0.375033	5.151598	0.62226
TINF2	0.873978	0.353555	2.160451	0.770504
TBL1Y	0.139618	0.005989	3.254601	0.220403
SEMA6A	3.734103	0.801264	17.4019	0.093385
TRPM7	0.994858	0.169397	5.842726	0.995446
TYRO3	2.275797	0.72513	7.142514	0.158779
WDR76	0.284127	0.073955	1.091579	0.066897
CAPN3	0.59109	0.163701	2.134298	0.422186
SNAP23	0.567323	0.270657	1.189163	0.133319
PHGDH	1.186432	0.954563	1.474623	0.123357
COL9A3	1.28691	0.314474	5.266371	0.725697
EZR	0.726391	0.361067	1.461346	0.370092
MYL6	1.175334	0.469439	2.942684	0.730089
TEKT2	0.63417	0.113043	3.557688	0.604731
CLSPN	0.331868	0.059944	1.837331	0.206492
RFFL	1.049945	0.185589	5.939919	0.956041
UNC13D	1.413434	0.742076	2.692172	0.292543
MFSD11	0.844457	0.44204	1.613219	0.608716
DPYSL2	1.327875	1.053345	1.673954	0.016406
GPATCH2	0.791203	0.320513	1.953122	0.611468
NUP50	0.38789	0.097585	1.541831	0.178614
COMT	0.626834	0.295475	1.329793	0.22353
VNN3	1.353683	0.725327	2.526387	0.341485
ECHDC1	0.712537	0.338962	1.497834	0.371259
LRRFIP2	0.440473	0.172051	1.127671	0.087368
SEC22C	0.315745	0.081045	1.230112	0.096617
XYLB	0.342967	0.051999	2.262083	0.266203
HDAC6	0.462118	0.190056	1.123631	0.0886
CDC6	0.334703	0.034557	3.241745	0.344779
UPRT	0.297171	0.11881	0.743291	0.009481
CDC23	0.43992	0.211426	0.915356	0.028053
AAAS	1.780541	0.645018	4.915098	0.265454
CBX5	0.897623	0.533276	1.510899	0.68435
MSH2	0.472358	0.191041	1.167927	0.104403

MAP3K1	1.515304	0.956405	2.400809	0.076707
DHPS	0.745803	0.29184	1.905916	0.540093
HOOK2	1.015724	0.382001	2.700763	0.975055
ARCN1	1.122307	0.567747	2.218544	0.739993
TMEM38B	0.997986	0.403128	2.47062	0.996521
PSMD5	0.612423	0.215639	1.739306	0.357213
PTGS1	0.925822	0.543169	1.578049	0.776965
NUP188	0.915277	0.382478	2.190274	0.842375
CRAT	1.15322	0.173971	7.644465	0.88256
SH2D3C	2.306204	1.110823	4.78796	0.024964
NANS	2.109823	0.808618	5.504886	0.127053
TBC1D2	1.679277	1.108958	2.542902	0.014346
PDE6C	0.386648	0.010999	13.59121	0.600831
CWF19L1	0.683615	0.235296	1.986138	0.484568
SEMA4G	1.376333	0.419553	4.515029	0.598201
BTAF1	0.623179	0.37617	1.032384	0.066325
IKZF5	0.328804	0.108227	0.998933	0.04978
BLNK	5.329296	1.534314	18.51082	0.008443
SORBS1	0.454632	0.011056	18.69484	0.677626
BAMBI	0.959786	0.745726	1.235291	0.749886
IL11	0.546975	0.087713	3.410911	0.518225
WAC	0.665249	0.273818	1.616243	0.368156
CREM	0.736388	0.296599	1.828286	0.509567
NUBP2	1.38491	0.713709	2.687337	0.335664
TPSD1	0.331909	0.022918	4.806847	0.418681
HIVEP1	1.302264	0.552316	3.070508	0.546185
TREM2	2.864233	0.715193	11.47079	0.137157
KCNK16	0.3932	0.018059	8.561194	0.552603
CRISP3	1.151052	0.836186	1.584479	0.388271
FKBP5	1.149057	0.734928	1.796545	0.542312
SRPK1	0.474461	0.24952	0.902183	0.022971
BRPF3	0.829371	0.354127	1.942399	0.666554
MRPS18A	1.101521	0.457879	2.649931	0.829078
TMEM14A	1.151957	0.774688	1.712952	0.484666
EFHC1	0.261106	0.110398	0.617552	0.002233
NCR2	1.129288	0.395449	3.22492	0.820342
HSP90AB1	0.871994	0.51664	1.471765	0.608032
MLN	15.41118	0.51601	460.2708	0.114521
CDC5L	1.030795	0.392423	2.707637	0.950917
ITPR3	1.183195	0.853844	1.639585	0.31218
ZNF184	1.007673	0.418531	2.426119	0.986396
SIRT1	1.129309	0.580728	2.196105	0.720067
HNRNPH3	0.423136	0.219011	0.817513	0.010479
IFT74	0.73184	0.355754	1.505508	0.396279
JAK2	0.937242	0.535134	1.641497	0.820677
IL12RB1	0.965387	0.459309	2.029072	0.925944
ABL1	0.348286	0.12294	0.986685	0.047123
ACOT7	4.800745	1.379857	16.70256	0.013659
SH3GLB1	0.873061	0.398773	1.91145	0.734206
CDC7	0.472873	0.255213	0.876163	0.017308
SYDE2	0.190168	0.016366	2.209694	0.18471
PCSK5	1.129503	0.620089	2.057408	0.690617
SCD	1.197952	0.901657	1.591612	0.212811
TMED1	0.698594	0.347681	1.403682	0.3137
ABLIM1	1.178972	0.614659	2.261375	0.620292
ERMP1	1.068677	0.617657	1.849035	0.812304
RAB18	1.374427	0.541728	3.487084	0.503164
NRP1	14.04479	1.387595	142.1569	0.025264

HSD17B7P	0.332331	0.050721	2.177478	0.250718
PRTFDC1	1.40963	0.666773	2.980107	0.368732
TSPAN15	3.333858	0.490251	22.67128	0.218271
MAST3	1.286012	0.83956	1.969873	0.247609
MZF1	0.46578	0.189504	1.144836	0.095878
OCEL1	1.670128	0.934894	2.983575	0.083175
MYO9B	1.562212	0.675065	3.615219	0.297381
KCNK6	2.714257	1.107307	6.653249	0.029051
PSMD8	0.737047	0.28689	1.893546	0.526234
FBXL19	0.197571	0.034668	1.125943	0.067795
STX1B	1.057723	0.097494	11.47539	0.963203
HSD3B7	0.863528	0.467044	1.596597	0.639844
SETD1A	2.010178	0.984718	4.103528	0.055153
BCL7C	4.411258	0.83879	23.19912	0.079705
CIRBP	0.906053	0.505269	1.624742	0.740568
PRKY	0.883995	0.611683	1.277536	0.511635
IGFALS	0.353724	0.056517	2.213863	0.266728
HNRNPM	0.71647	0.302348	1.69781	0.448785
NDUFB7	1.482257	0.795718	2.761134	0.214975
TECR	2.03276	0.981994	4.20788	0.056003
TIMM13	4.641162	1.0979	19.61962	0.036893
CDC34	1.599333	1.024381	2.496987	0.038834
MTAP	0.265518	0.122813	0.574045	0.000749
POLR2E	0.725976	0.271782	1.939204	0.522937
POLRMT	1.510721	0.735183	3.10437	0.261529
HCN2	1.058624	0.090013	12.45026	0.963866
RASSF7	1.503453	0.749142	3.017281	0.251255
GADD45B	1.337755	0.990348	1.807029	0.057862
PALM	1.697771	1.075565	2.679919	0.023041
MADCAM1	1.097771	0.039295	30.66835	0.956215
MKNK2	1.585858	0.837501	3.002917	0.156898
ARVCF	2.777222	0.384677	20.05046	0.311179
TRMT2A	0.420649	0.055262	3.201926	0.403042
RANBP1	0.38647	0.175796	0.849617	0.018008
ZDHHC8	1.130686	0.575965	2.219669	0.721177
KLHL22	0.880374	0.383917	2.018816	0.763497
MED15	2.706183	0.60716	12.06178	0.191689
SERPIND1	1.227786	0.100547	14.99262	0.872304
SNAP29	0.882101	0.367051	2.119875	0.779155
CRKL	0.859059	0.326318	2.261546	0.758382
LZTR1	1.224711	0.693092	2.164094	0.48526
MMP11	1.78625	0.944861	3.376888	0.074195
CECR2	0.025827	0.001199	0.556341	0.019579
SMARCB1	1.399774	0.692461	2.829572	0.34899
DERL3	1.313557	0.396552	4.351091	0.655362
BCL2L13	1.086748	0.392705	3.007399	0.872736
DDTL	1.240611	0.316936	4.856241	0.756822
OSM	1.348988	1.046452	1.738991	0.020866
CABIN1	1.297358	0.734189	2.292513	0.370133
TBC1D10A	2.683612	1.177105	6.118211	0.018887
SUSD2	0.445496	0.089726	2.211906	0.322673
SF3A1	0.31689	0.07429	1.35172	0.120482
GGT5	1.072712	0.480791	2.393371	0.863891
RNF215	0.64112	0.161397	2.546736	0.52761
SEC14L2	0.140544	0.012357	1.598553	0.113693
SEC14L3	0.395529	0.046772	3.344792	0.394484
PPIL2	6.703772	0.435371	103.2235	0.172604
UPB1	0.743128	0.313824	1.75971	0.499666

YPEL1	0.707741	0.386169	1.297095	0.263408
SNRPD3	0.771225	0.425342	1.398375	0.392225
PES1	0.490868	0.193966	1.242235	0.133076
MAPK1	0.783454	0.308459	1.989893	0.607849
GGT1	0.656191	0.221315	1.945582	0.447408
PRODH	0.994981	0.757881	1.306256	0.971097
PPM1F	1.094576	0.632846	1.893187	0.746495
SLC35E4	6.999323	1.933042	25.34374	0.003038
TOP3B	0.804934	0.362547	1.78713	0.593879
CYTH4	1.498431	0.974582	2.303855	0.065379
MFNG	0.804875	0.410924	1.576506	0.526837
CARD10	1.219503	0.653317	2.276362	0.533174
LRP5L	0.818075	0.542409	1.233841	0.338199
SLC25A1	1.971846	1.135406	3.424481	0.015914
LGALS2	1.138361	0.916815	1.413443	0.240597
GGA1	0.069747	0.009034	0.53849	0.010664
HIRA	0.514995	0.096792	2.740097	0.436523
SH3BP1	1.620748	0.967051	2.716324	0.066832
SEZ6L	2.745839	0.908123	8.302433	0.073575
LGALS1	1.358953	0.950418	1.943096	0.09272
HPS4	0.087865	0.017206	0.448693	0.003464
PIK3IP1	1.165026	0.819573	1.656089	0.39467
SRRD	0.846259	0.382944	1.870127	0.67989
PATZ1	1.327076	0.394674	4.462243	0.647413
TRIOBP	1.445728	0.571949	3.654399	0.435924
TFIP11	0.461176	0.081855	2.598281	0.38024
GCAT	1.56441	1.113768	2.197386	0.009836
CRYBB1	0.394978	0.058991	2.644614	0.338309
ANKRD54	0.991524	0.466005	2.109673	0.98237
EIF3L	0.612997	0.24124	1.557642	0.303689
MICALL1	2.36427	0.900323	6.208635	0.080671
POLR2F	0.700708	0.316139	1.553089	0.381118
SOX10	0.157012	0.023461	1.050787	0.056277
CCDC134	1.784635	0.368682	8.638672	0.471613
DEPDC5	0.3002	0.013853	6.505401	0.443238
PICK1	1.27127	0.639776	2.526084	0.493284
TTC28	2.482087	0.212184	29.03499	0.468767
SLC16A8	0.368963	0.028358	4.800459	0.446274
CENPM	0.683314	0.411849	1.133713	0.140444
SLC5A4	0.221627	0.016868	2.911956	0.251542
KDELR3	0.357693	0.03237	3.952601	0.401623
CYP2D6	0.092973	0.006118	1.412841	0.087076
DDX17	0.727667	0.351031	1.50841	0.392682
DMC1	0.745092	0.22606	2.455818	0.628716
TCF20	0.541623	0.067238	4.362919	0.564584
HSCB	0.681156	0.327776	1.415517	0.303554
CBY1	0.805087	0.184393	3.51512	0.773111
TOMM22	0.440519	0.204588	0.948522	0.036169
XBP1	0.701432	0.357925	1.374608	0.301561
JOSD1	0.697962	0.364498	1.336497	0.277974
FBXO7	0.58518	0.284062	1.205497	0.146187
GTPBP1	2.783013	0.911343	8.498622	0.072339
POLDIP3	0.543756	0.166418	1.776668	0.31319
RAB36	2.159365	0.281783	16.54768	0.45875
TIMP3	0.996209	0.145304	6.830025	0.996915
SBF1	1.169515	0.626092	2.184609	0.623303
CYB5R3	1.852748	0.836379	4.10421	0.128597
DNAL4	1.115217	0.489562	2.540452	0.795168

LMF2	2.262304	1.039873	4.921775	0.039537
RHBDD3	1.566691	0.690381	3.555311	0.282911
PACSIN2	1.894797	0.714504	5.024823	0.199006
TTLL1	2.635246	0.287153	24.18406	0.391587
RASL10A	1.706607	1.030277	2.826916	0.037912
AP1B1	1.957258	0.760784	5.035412	0.163656
HMGXB4	0.46589	0.099651	2.178146	0.331717
TOM1	1.858324	0.929582	3.714969	0.079541
NEFH	0.687053	0.284793	1.657491	0.403514
CHKB	1.066167	0.489469	2.322338	0.871854
BIK	1.076172	0.842117	1.375278	0.557419
HMOX1	1.302045	1.030213	1.645601	0.027168
MCAT	1.272005	0.487161	3.321282	0.623193
THOC5	0.304925	0.094605	0.982813	0.046702
MCM5	0.864064	0.48962	1.524869	0.614154
APOBEC3I	0.778964	0.407368	1.489523	0.450106
ARSA	2.022666	1.114493	3.670888	0.020535
TSPO	1.772252	1.200031	2.617331	0.00402
RASD2	0.482902	0.087445	2.666742	0.403753
TTLL12	0.91941	0.564204	1.498243	0.735932
CBX7	1.235753	0.822852	1.855843	0.30762
PDGFB	0.417372	0.015793	11.03029	0.600964
ACR	0.469637	0.064	3.446254	0.457339
CABP7	1.600116	0.153694	16.65891	0.694135
RPL3	0.357789	0.058668	2.181968	0.265203
ZMAT5	3.372363	1.13569	10.01403	0.02859
SYNGR1	0.629295	0.345419	1.146468	0.130192
ASCC2	0.570742	0.262522	1.240836	0.156959
MTMR3	0.925331	0.275593	3.106897	0.900068
APOL4	3.558283	0.660657	19.16483	0.139553
APOL1	0.011762	0.000388	0.357011	0.010727
PNPLA3	33.34265	1.905502	583.4328	0.016329
MYH9	1.453034	0.753934	2.800388	0.264334
CACNA1I	1.092731	0.186983	6.385933	0.921573
SAMM50	0.341286	0.168317	0.692007	0.002875
TXN2	0.747031	0.24914	2.239926	0.602675
FOXRED2	0.422388	0.142352	1.253311	0.120405
GRAP2	1.1567	0.787222	1.699591	0.458432
EIF3D	0.582279	0.300402	1.128651	0.109253
TNRC6B	0.165179	0.035838	0.761311	0.020901
SGSM3	1.610964	0.630208	4.118012	0.319358
NCF4	0.895019	0.572293	1.399737	0.626898
CSF2RB	0.340588	0.102234	1.134649	0.079392
SLC25A17	0.231983	0.073253	0.734655	0.012982
UPK3A	2.395192	0.493785	11.6183	0.278311
FAM118A	0.926834	0.590359	1.455085	0.741276
KCTD17	2.959618	0.978144	8.955067	0.054751
ST13	0.563354	0.324502	0.978016	0.041455
IL2RB	1.014001	0.752116	1.367074	0.927322
RBX1	1.48517	0.757038	2.913631	0.249978
EP300	2.026396	1.095781	3.747355	0.02435
L3MBTL2	0.573209	0.158613	2.071514	0.395904
CHADL	1.893855	0.38092	9.415864	0.43513
RANGAP1	0.876312	0.43391	1.769772	0.712747
ZC3H7B	0.340512	0.043843	2.644652	0.302978
PHF5A	1.613006	0.774054	3.361252	0.201858
ACO2	2.189259	0.920893	5.204575	0.076155
POLR3H	0.262528	0.036137	1.907206	0.186223

TRMU	0.626239	0.253068	1.549679	0.311345
PMM1	1.061398	0.726207	1.5513	0.758284
CERK	0.351802	0.11676	1.059989	0.063391
BRD1	2.21722	0.815047	6.031635	0.118893
ZBED4	0.579127	0.260879	1.285609	0.179434
MLC1	1.228396	0.611493	2.467658	0.563272
HDAC10	0.856343	0.355881	2.060588	0.729218
KCNK10	0.624234	0.008352	46.65659	0.830477
ABHD4	1.914117	1.144282	3.201872	0.013383
KHNYN	0.931582	0.436518	1.988108	0.85461
FKBP3	0.742937	0.313331	1.761572	0.499948
SDR39U1	0.465155	0.10094	2.14353	0.326168
CTSG	1.003303	0.826495	1.217933	0.973408
GZMH	0.95168	0.724617	1.249895	0.72176
GZMB	0.969938	0.731978	1.285257	0.831692
RBM23	2.452528	0.910783	6.604091	0.075888
PRMT5	0.68537	0.332526	1.412617	0.305921
COCH	0.845333	0.469438	1.522221	0.575558
AP4S1	0.103854	0.007471	1.443683	0.091696
POLE2	0.320171	0.171335	0.5983	0.000357
SOS2	0.840213	0.241796	2.91964	0.784118
CDKL1	0.264367	0.042277	1.653153	0.154884
NIN	0.542346	0.208577	1.410219	0.209504
PYGL	0.766469	0.458682	1.280789	0.30998
TRIM9	1.28204	0.421351	3.90085	0.661662
PSMC6	1.14405	0.587891	2.22635	0.691983
GNPNAT1	1.567948	0.386195	6.36585	0.529262
DDHD1	0.947301	0.086742	10.34537	0.964598
CDKN3	1.007328	0.673191	1.507313	0.971676
CGRRF1	1.113903	0.528848	2.346195	0.776552
ATP6V1D	1.037837	0.557339	1.932586	0.906798
PLEK2	1.348871	0.744883	2.442605	0.323249
PIGH	0.592341	0.197264	1.778671	0.35058
PSMA3	1.042327	0.503656	2.157118	0.911049
VTI1B	1.025049	0.448883	2.340753	0.953173
TIMM9	0.62749	0.264469	1.488812	0.290436
GSTZ1	0.132148	0.020956	0.833339	0.03124
KIAA0586	0.184618	0.061824	0.551306	0.002472
TMED8	0.061514	0.003559	1.063162	0.055131
AHSA1	0.600436	0.243008	1.483585	0.269046
DAAM1	0.956103	0.547103	1.670863	0.874766
SPTLC2	0.881324	0.495245	1.56838	0.667496
RIN3	1.851779	1.012007	3.388403	0.045643
LGMN	2.052723	1.086206	3.879255	0.026787
ALKBH1	1.818061	0.355587	9.295479	0.472755
SNW1	0.391577	0.149124	1.02822	0.05698
CHGA	2.815356	0.398541	19.8881	0.29941
ITPK1	2.08928	1.281543	3.406122	0.003129
DHRS7	0.470256	0.232812	0.94987	0.035436
PPM1A	0.324073	0.080094	1.311244	0.114108
ASB2	0.474637	0.070876	3.178503	0.442445
ERH	0.594453	0.332181	1.063802	0.079832
HIF1A	0.933636	0.538584	1.61846	0.806735
SLC10A1	2.751732	0.387588	19.53629	0.311448
EIF5	0.367709	0.160747	0.841134	0.0178
SLC8A3	1.28034	0.352182	4.654611	0.707469
DICER1	1.177291	0.322107	4.302965	0.805049
ZFYVE21	1.472142	0.854592	2.535952	0.163413

MTHFD1	0.277908	0.106573	0.724693	0.008833
TCL1A	1.553851	0.740066	3.262481	0.24419
ZC3H14	0.055121	0.004049	0.750383	0.029591
VRK1	0.375302	0.153844	0.915552	0.031251
PSMC1	0.502735	0.25982	0.972759	0.041154
PAPLN	1.26983	0.70557	2.285343	0.42559
RPS6KA5	0.411958	0.122082	1.390128	0.152964
PSMB5	1.011847	0.560212	1.827583	0.968857
YY1	0.737343	0.277754	1.957398	0.540742
ACIN1	1.469638	0.539416	4.004027	0.451511
CCNB1IP1	0.503371	0.246052	1.02979	0.060165
TRIP11	0.443313	0.218015	0.901437	0.02467
APEX1	0.870111	0.517867	1.461944	0.599214
PABPN1	0.765749	0.381803	1.535796	0.452258
ARHGAP5	0.897414	0.6957	1.157614	0.404709
CINP	1.768379	0.665894	4.696193	0.252635
SRP54	0.83993	0.353254	1.997095	0.69304
CHD8	0.517188	0.201237	1.3292	0.170978
PCK2	1.987686	0.695697	5.67905	0.199649
PSMA6	0.910432	0.444587	1.864393	0.797496
NFKBIA	1.011712	0.715729	1.430097	0.947425
PSME2	1.118538	0.607615	2.059079	0.719001
BRMS1L	0.379339	0.060413	2.3819	0.301097
REC8	1.724467	1.022567	2.908157	0.040987
TM9SF1	2.498568	0.610803	10.2207	0.20264
SEC23A	1.341021	0.347526	5.174686	0.67018
GMPR2	0.549675	0.185152	1.631857	0.281085
PNN	0.579821	0.402435	0.835395	0.003442
RABGGTA	0.883834	0.19232	4.061787	0.873909
NFATC4	0.671954	0.106647	4.233808	0.672053
PLTP	1.004429	0.622452	1.620811	0.985559
PCIF1	1.730473	0.832657	3.596363	0.141752
GSS	1.114637	0.395393	3.142234	0.837384
MMP9	0.932502	0.792458	1.097294	0.39996
TRPC4AP	1.335144	0.442647	4.02716	0.607861
PYGB	2.109973	1.177917	3.779544	0.012055
ABHD12	4.18497	1.314772	13.32092	0.015384
PROCR	6.33049	1.669187	24.00876	0.006663
GINS1	1.876319	0.233044	15.10692	0.554297
NINL	1.330599	0.184654	9.58815	0.776817
CD40	4.852527	1.382903	17.02724	0.013658
ZMYND8	0.726765	0.329872	1.601185	0.428413
SGK2	9.674879	0.05176	1808.404	0.395098
IFT52	0.338843	0.113531	1.011307	0.052403
MYBL2	1.820302	0.363252	9.121773	0.466335
NDRG3	0.718516	0.238207	2.167303	0.557312
SLA2	0.727876	0.344322	1.538685	0.405607
NFATC2	0.121113	0.002695	5.442122	0.276887
PABPC1L	0.674643	0.449873	1.011713	0.056957
STK4	0.820849	0.41785	1.612524	0.566616
SALL4	0.974781	0.395647	2.401632	0.955724
ADNP	0.919101	0.308255	2.740419	0.879703
PFDN4	0.745725	0.285854	1.945415	0.548697
DOK5	1.04061	0.125871	8.603003	0.970536
CSTF1	0.668717	0.02636	16.96455	0.807303
RAE1	0.344188	0.058139	2.037611	0.239797
TPD52L2	1.651746	0.588485	4.636079	0.340569
DNAJC5	1.407939	0.667227	2.970938	0.369205

CTSZ	1.257491	0.908979	1.739626	0.166467
PRPF6	1.419684	0.553983	3.638203	0.465476
TUBB1	1.004722	0.811515	1.243929	0.965515
PSMA7	0.418489	0.149686	1.170002	0.096783
SLCO4A1	1.841039	0.809599	4.186547	0.145375
NTSR1	1.4847	0.927825	2.375808	0.099424
TCFL5	0.938234	0.444146	1.981968	0.867298
DIDO1	0.367981	0.128537	1.053472	0.062477
SLC17A9	0.836951	0.505987	1.384399	0.488189
BIRC7	1.384795	0.017826	107.5785	0.883453
ARFGAP1	1.386258	0.67244	2.857818	0.376242
AVP	2.025004	1.244581	3.294796	0.004498
COL20A1	5.863553	1.350355	25.4609	0.018231
EEF1A2	1.220585	0.85916	1.734052	0.265865
PTK6	0.887391	0.214962	3.663268	0.868825
GMEB2	1.735923	0.837791	3.596873	0.137858
CDC25B	1.780972	0.843305	3.761228	0.130241
ISM1	14.58383	0.845174	251.6502	0.065153
RNF24	0.734525	0.408892	1.319484	0.301917
ARFRP1	0.846274	0.229601	3.119234	0.801984
TRIB3	1.416513	0.955931	2.099011	0.082679
RASSF2	1.405739	0.489644	4.035794	0.526793
CSNK2A1	0.482772	0.176684	1.319125	0.155633
CDS2	1.103195	0.378552	3.214981	0.857183
HM13	5.032736	1.038974	24.37831	0.044701
SNPH	0.834859	0.165495	4.211545	0.826965
MYLK2	1.006785	0.190745	5.313968	0.993644
SIRPB1	1.114023	0.703064	1.765196	0.645671
SEC23B	1.417414	0.632225	3.177761	0.397076
FERMT1	0.641494	0.253053	1.626198	0.349567
PLCB4	0.616653	0.018357	20.71533	0.78745
MYL9	1.173783	0.737692	1.867672	0.498941
HCK	1.16558	0.881828	1.540637	0.28173
TM9SF4	1.655699	0.721631	3.798813	0.234043
CRNKL1	0.831823	0.299112	2.313283	0.724199
POFUT1	0.339541	0.034165	3.37442	0.356572
SAMHD1	1.396702	0.828142	2.355606	0.210256
KIF3B	1.721183	0.67417	4.394249	0.256167
NOP56	0.353034	0.163861	0.760602	0.007844
MANBAL	3.844553	1.326582	11.14186	0.013119
IDH3B	1.115033	0.175108	7.100171	0.908224
MAPRE1	0.328734	0.150541	0.717851	0.005241
JAG1	1.080545	0.718738	1.624484	0.709607
CDK5RAP1	0.05003	0.008469	0.295545	0.00095
SNTA1	1.385417	0.535487	3.584365	0.501476
OXT	2.383386	1.023016	5.552728	0.044147
E2F1	1.028411	0.429987	2.459678	0.949792
RPRD1B	1.212025	0.357518	4.108898	0.757546
PXMP4	2.518741	0.277657	22.84853	0.411614
CHMP4B	1.13117	0.473594	2.701779	0.781428
BPI	1.004224	0.856355	1.177627	0.958632
CST3	1.561077	1.082336	2.251577	0.017155
ASIP	3.261639	0.773833	13.74753	0.107256
ACTR5	0.389473	0.144629	1.048818	0.062089
AHCY	1.083408	0.621281	1.88928	0.777668
PPP1R16B	1.312693	0.927525	1.857809	0.124686
FAM83D	0.738748	0.431383	1.265112	0.269942
DHX35	0.465168	0.191789	1.128225	0.09044

DNTTIP1	2.109884	0.852704	5.220585	0.106258
MAP1LC3/	0.907071	0.494467	1.663971	0.752712
PIGU	0.677991	0.381902	1.203639	0.184495
TNNC2	0.738682	0.272328	2.003649	0.551894
ACOT8	1.114799	0.500563	2.482754	0.790228
ZNF516	0.487154	0.096175	2.467563	0.384953
ADNP2	0.517636	0.265036	1.010983	0.053857
USP14	0.306256	0.133602	0.702028	0.005177
VAPA	0.239506	0.061984	0.925454	0.038238
METTL4	0.332125	0.093393	1.181101	0.088601
LPIN2	1.193349	0.518399	2.747075	0.677761
SMCHD1	0.942379	0.225166	3.944115	0.935241
MYOM1	0.661868	0.339128	1.29175	0.226425
MYL12A	0.797028	0.442339	1.436124	0.450153
CEP76	0.85543	0.11223	6.520195	0.880224
CEP192	0.212655	0.058848	0.768458	0.018188
RNMT	0.548252	0.251969	1.192925	0.129716
SMAD7	0.5212	0.252275	1.076799	0.078391
RNF125	0.822321	0.404365	1.672279	0.589082
ANKRD12	0.703283	0.341319	1.449106	0.339935
POLI	0.443426	0.108318	1.81528	0.258119
MIB1	0.768631	0.347531	1.699972	0.515845
RBBP8	0.167376	0.041602	0.673404	0.011847
RIOK3	0.806115	0.416474	1.560293	0.522397
CSTF2	0.567171	0.20632	1.55914	0.271706
VSIG1	1.418931	0.157237	12.80468	0.755238
PSMD10	0.645437	0.318202	1.309194	0.224998
ATG4A	0.516822	0.13585	1.966169	0.332932
STS	0.91724	0.234088	3.594066	0.901332
TBL1X	1.345992	0.885653	2.045605	0.164119
GPR143	4.462994	0.535221	37.21512	0.166874
PGRMC1	0.772641	0.453705	1.315777	0.342299
POLA1	0.293104	0.148695	0.577761	0.000394
MID1	0.037295	0.000352	3.952845	0.16688
NKAP	0.461475	0.134444	1.584005	0.219075
RHOXF1	2.414884	0.491555	11.86371	0.277681
NXT2	0.849407	0.52849	1.365197	0.500211
ALG13	0.81669	0.373476	1.785874	0.61197
PRPS2	0.490127	0.245447	0.978724	0.043289
TLR8	1.430024	1.00057	2.043803	0.049635
MOSPD1	1.057578	0.476483	2.34735	0.890544
AMMECR1	0.694154	0.296156	1.627014	0.400916
CHRDL1	3.563907	0.100942	125.8295	0.484631
WDR13	1.658102	0.749367	3.668832	0.212059
SUV39H1	0.405374	0.190361	0.863241	0.019217
SRPX	0.487015	0.047517	4.991514	0.544562
XIAP	1.076419	0.294576	3.933373	0.911316
STAG2	0.528319	0.147446	1.893047	0.327146
ATP11C	1.495168	0.38415	5.819411	0.561828
MCF2	0.205768	0.023382	1.810785	0.154201
ABCD1	1.611168	0.997867	2.60141	0.05103
CCDC22	1.332363	0.649311	2.733964	0.433953
CACNA1F	1.493798	0.624378	3.573848	0.367213
SYP	5.198345	0.383484	70.46646	0.215222
PLP2	1.195082	0.564068	2.532004	0.641764
BMX	0.878567	0.219605	3.514851	0.854787
PLS3	0.947281	0.375625	2.388931	0.908638
RENBP	1.788854	1.1002	2.908561	0.019027

ELF4	1.195909	0.569802	2.509992	0.636231
SMARCA1	3.408098	0.875542	13.26623	0.077013
MTMR8	5.472746	0.118489	252.7744	0.384722
ASB9	0.751082	0.473222	1.192091	0.22457
ZC3H12B	1.534526	0.682657	3.449423	0.300113
RBBP7	0.340417	0.141305	0.820101	0.016303
KCND1	1.414578	0.256058	7.814759	0.690837
SLC25A14	0.106941	0.022932	0.498703	0.004433
FMR1	0.046715	0.003833	0.569326	0.016327
PIM2	0.892921	0.566327	1.407858	0.62589
SCML2	0.494919	0.223104	1.097897	0.083592
SLC35A2	0.979029	0.149908	6.393881	0.982339
PQBP1	1.300446	0.358551	4.716651	0.689419
PCSK1N	2.452429	0.899048	6.689754	0.079754
EMD	0.612772	0.260251	1.442797	0.262312
TAZ	1.490425	0.47347	4.691669	0.495196
PGK1	0.732228	0.296526	1.808132	0.4992
GATA1	0.723916	0.484746	1.081092	0.114356
MAGT1	0.830701	0.491273	1.404645	0.488869
SMS	0.78761	0.350039	1.772171	0.56392
PHEX	10.10486	1.077168	94.79311	0.042863
UBL4A	0.7817	0.419777	1.455666	0.437529
CD99L2	0.74872	0.382735	1.464675	0.397962
EEA1	0.313772	0.066445	1.481718	0.143331
RP2	1.272156	0.709328	2.28157	0.419292
USP11	0.670097	0.385244	1.165571	0.156342
PCYT1B	3.312204	0.298321	36.77485	0.329507
HTATSF1	4.747819	0.224612	100.3586	0.317002
CD40LG	0.56975	0.121513	2.671436	0.475492
TIMP1	1.431444	0.895736	2.287538	0.133715
GABRE	0.230658	0.003551	14.98062	0.490926
FGD1	0.454034	0.027694	7.443744	0.580058
PIN4	1.773399	0.489417	6.425899	0.383118
PORCN	4.534092	0.694719	29.59183	0.114247
MAGED2	0.680052	0.137302	3.368274	0.636686
RBM3	0.557435	0.191148	1.625618	0.284534
KLF8	0.146074	0.00934	2.284579	0.170345
SYTL4	1.741542	1.242997	2.440045	0.001263
ZDHHC15	2.310064	0.457597	11.66179	0.310783
CENPI	0.350832	0.028371	4.338367	0.414326
GLA	0.392413	0.200448	0.768219	0.006347
ARMCX3	0.453186	0.11308	1.816208	0.263811
BEX4	1.114756	0.805207	1.543308	0.512752
NDFIP2	0.515493	0.287731	0.923549	0.025928
TNFSF13B	1.353113	0.950832	1.925591	0.092979
FNDC3A	0.408921	0.164972	1.013606	0.05351
MLNR	0.022454	0.000649	0.777026	0.035774
CDADC1	0.503291	0.095193	2.660945	0.419039
CAB39L	2.646328	0.120467	58.13264	0.536994
KLF5	1.032606	0.527057	2.023071	0.925501
STK24	0.889178	0.178571	4.427588	0.885968
ACP5	1.3227	0.771691	2.267145	0.309024
DNAJC3	0.82472	0.391742	1.736256	0.611896
ARHGEF7	0.331253	0.047409	2.314503	0.265319
FGF9	0.481053	0.140974	1.641524	0.242593
PARP4	0.610613	0.360679	1.033741	0.066295
MRPS31	0.395237	0.172678	0.904648	0.028009
SLC25A15	0.359446	0.15322	0.843246	0.01868

KPNA3	0.903594	0.461401	1.769571	0.767519
FLT1	0.358933	0.026019	4.951544	0.444132
DGKH	0.252552	0.011396	5.596841	0.384014
KATNAL1	0.115736	0.030439	0.440051	0.001553
INTS6	0.689467	0.226614	2.097689	0.512476
DHRS12	3.864795	0.544814	27.41605	0.17624
TSC22D1	1.046738	0.714143	1.53423	0.814868
CLN5	1.350489	0.603233	3.023411	0.464948
OLFM4	0.839659	0.59239	1.190141	0.326142
MSLN	0.835957	0.419987	1.663915	0.609926
MGRN1	1.866165	1.064802	3.270629	0.02931
ZNF629	1.545744	0.653863	3.654167	0.321144
TRADD	2.386679	1.051455	5.417479	0.037532
HSF4	2.800178	0.34929	22.44837	0.332273
CORO1A	1.625988	1.02299	2.58442	0.039773
MAPK3	2.062288	1.124773	3.781235	0.019278
GDPD3	1.140626	0.214239	6.072788	0.877439
ELMO3	1.219679	0.449367	3.310475	0.696679
PHKB	0.43581	0.166505	1.140687	0.090678
LYRM1	0.79315	0.45378	1.386327	0.415984
NUTF2	0.365035	0.13651	0.976124	0.044631
NUP93	1.364209	0.513832	3.621936	0.533017
CENPT	0.908306	0.384126	2.147788	0.826629
TSNAXIP1	0.573758	0.114027	2.887011	0.500381
NFAT5	0.211454	0.040149	1.113672	0.066809
LONP2	0.288127	0.10024	0.828185	0.020893
N4BP1	2.578203	0.488448	13.60868	0.264506
ARL2BP	0.771632	0.330429	1.801945	0.549097
PLLP	1.021042	0.109873	9.488425	0.985393
CCL22	0.105235	0.007696	1.438903	0.09155
DHODH	0.324242	0.04993	2.10559	0.238038
CCL17	0.25632	0.018973	3.462823	0.305425
CTCF	1.325485	0.513911	3.418708	0.559969
ACD	0.591614	0.262654	1.332582	0.205174
POLR2C	4.735417	1.247264	17.97869	0.022338
PARD6A	1.752267	0.952306	3.224216	0.071406
ZNF821	1.580643	0.4941	5.056537	0.440311
MMP15	0.888211	0.279991	2.817656	0.840494
CYB5B	0.528365	0.243383	1.147041	0.106725
NME3	2.262933	1.24612	4.109449	0.007301
NDRG4	0.015464	9.57E-05	2.498405	0.108048
PSMD7	0.346041	0.123933	0.966205	0.042807
SETD6	0.265375	0.099752	0.705992	0.007876
SLC38A7	1.493468	0.345317	6.459115	0.591379
VAC14	1.776648	0.388181	8.131449	0.458942
HAS3	1.057818	0.032131	34.82535	0.974848
COG4	1.658124	0.654174	4.202821	0.286582
SMPD3	3.166805	0.821014	12.21496	0.094204
SLC7A6OS	1.189377	0.360162	3.927723	0.776
SLC7A6	1.144028	0.478347	2.736088	0.762313
PLA2G15	1.697197	0.859558	3.351113	0.127517
ESRP2	0.111916	0.014278	0.877214	0.0371
WDR59	0.64683	0.327037	1.279334	0.210558
MON1B	0.965809	0.383775	2.43056	0.941105
AXIN1	0.952698	0.319862	2.837578	0.930655
HCFC1R1	1.135933	0.428373	3.012198	0.797832
MLYCD	0.727507	0.315112	1.679616	0.456136
MPG	1.724005	0.328708	9.042061	0.519484

HSDL1	0.326029	0.123576	0.860161	0.023556
TAF1C	0.557926	0.174854	1.780237	0.324276
NAGPA	1.136619	0.593661	2.176163	0.699176
WFDC1	0.950639	0.436933	2.068316	0.898442
COTL1	1.371683	0.990729	1.899122	0.05693
USP10	1.015393	0.430465	2.395135	0.972169
CRISPLD2	1.178269	0.905819	1.532667	0.221441
TSC2	1.890102	0.583735	6.12005	0.288241
ZNF500	2.504469	1.096199	5.721925	0.029418
NME4	0.694911	0.427436	1.129763	0.14213
ABCC1	1.829817	0.715899	4.676958	0.206972
NOMO3	0.573828	0.276961	1.188899	0.135066
LMF1	0.34069	0.100614	1.153608	0.08357
FOXF1	0.157354	0.014459	1.712511	0.128943
MTHFSD	0.627428	0.234885	1.675989	0.352456
CLCN7	1.225838	0.688376	2.182934	0.489175
HAGHL	0.529397	0.221399	1.265863	0.152738
SLC7A5	0.808973	0.579293	1.12972	0.213448
METRN	1.661001	0.959394	2.875693	0.069995
FBXO31	1.353415	0.724751	2.527396	0.342263
STUB1	1.758888	0.7457	4.148704	0.197136
RHBDL1	0.667682	0.122761	3.631435	0.640155
NUBP1	2.469812	0.795185	7.671129	0.117907
UBE2I	1.32303	0.072595	24.1121	0.850089
MEFV	1.460553	0.965235	2.210048	0.073048
CRYM	3.961329	0.110888	141.514	0.450534
EEF2K	0.725828	0.304624	1.729433	0.469453
GSPT1	0.704429	0.344562	1.440148	0.336915
ZNF174	0.778407	0.138585	4.372162	0.776027
CLUAP1	0.204386	0.036782	1.13571	0.069596
UBFD1	0.818822	0.36128	1.855813	0.632067
PRSS33	0.349433	0.046374	2.632999	0.307533
EARS2	0.596694	0.280012	1.27153	0.18101
GGA2	0.818745	0.417694	1.604864	0.560306
AQP8	0.160887	0.01208	2.142734	0.166643
CPPED1	1.162786	0.605819	2.23181	0.650276
USP31	0.36943	0.023265	5.866139	0.48027
HMOX2	1.947343	0.444132	8.538322	0.376839
DNAJA3	0.648997	0.216925	1.941669	0.439396
BFAR	0.663449	0.343938	1.27978	0.22094
RRN3P2	0.62974	0.3451	1.149151	0.131826
RBL2	0.700891	0.430035	1.142344	0.15387
QPRT	1.248479	0.960409	1.622955	0.097289
XYLT1	0.872107	0.455704	1.668999	0.679444
PYCARD	2.04328	1.159073	3.602012	0.0135
RPGRIP1L	1.10018	0.075247	16.08569	0.944385
MAZ	4.107769	1.076274	15.67795	0.038685
STX4	0.397324	0.145207	1.087183	0.072303
CDIPT	5.4938	1.392313	21.67748	0.014993
BCKDK	0.979682	0.438804	2.187254	0.960048
NOMO1	0.608057	0.215203	1.718066	0.347864
IL21R	0.856433	0.143843	5.099148	0.864805
SYT17	1.240729	0.453415	3.395144	0.674506
TMC5	0.069797	0.005674	0.858509	0.037607
RNF40	3.011453	0.905109	10.01962	0.07227
AQP9	1.110908	0.921477	1.339282	0.270183
AAGAB	0.458397	0.182353	1.152311	0.097211
IQCH	0.177354	0.003433	9.162995	0.390145

LACTB	1.331538	0.615157	2.88218	0.467379
CORO2B	9.288012	0.814848	105.869	0.072645
CSK	2.169446	1.24493	3.780531	0.006274
HERC1	0.634124	0.306759	1.31084	0.218911
TRIP4	1.113471	0.43415	2.855739	0.823015
MTFMT	0.07737	0.009469	0.632171	0.016947
ACSBG1	0.759529	0.401707	1.436081	0.397359
IGDCC4	0.082703	0.003137	2.180273	0.135422
RAB11A	0.733071	0.355347	1.512305	0.400669
CTSH	1.28721	1.020992	1.622844	0.032704
TTC23	0.989591	0.30519	3.208787	0.986091
CD276	4.406902	0.767196	25.31399	0.096343
FAH	1.234936	0.745371	2.046051	0.412693
RPAP1	1.141564	0.465191	2.80136	0.77253
HOMER2	1.061461	0.637212	1.768171	0.8188
EHD4	1.616969	1.075509	2.431024	0.020896
TMEM87A	0.662907	0.302204	1.454138	0.304998
CEP152	0.256585	0.089932	0.732067	0.01099
ATP8B4	0.844442	0.626188	1.138768	0.267764
DTWD1	0.529258	0.178299	1.571036	0.251712
TGM5	0.814737	0.244214	2.718087	0.738901
FAM189A1	0.093462	0.009026	0.967789	0.046876
GABPB1	0.667623	0.203595	2.189252	0.5049
BMF	12.11868	4.321185	33.9866	2.12E-06
DMXL2	1.282578	0.862432	1.907404	0.219048
SCG3	2.578197	0.121113	54.88359	0.543855
DNAJC17	0.45668	0.152163	1.370609	0.162188
EIF3J	0.501327	0.253178	0.992698	0.047593
SPG11	0.673665	0.313354	1.448281	0.31176
RHOV	0.429914	0.04974	3.71582	0.442999
VPS18	1.650054	0.962984	2.827335	0.068351
OIP5	0.60066	0.341981	1.055007	0.076123
SLC30A4	0.430273	0.027265	6.790306	0.549084
MYEF2	1.057294	0.07993	13.98564	0.966271
SGK3	0.437752	0.147127	1.302461	0.137556
CSPP1	0.497497	0.189135	1.308605	0.157101
ZDHHC2	0.394333	0.182102	0.853912	0.018246
BRF2	1.352524	0.627187	2.916708	0.441205
TRIM35	0.392466	0.041294	3.730084	0.415581
ZFAND1	0.577182	0.295732	1.126491	0.10721
CA2	0.891625	0.729124	1.090343	0.263815
FZD3	0.687483	0.240127	1.968264	0.485041
INTS9	0.724514	0.317527	1.653149	0.443889
RIPK2	0.935082	0.591911	1.477214	0.773584
NBN	1.036957	0.297755	3.611295	0.954542
DECR1	0.72896	0.351769	1.510602	0.395121
SFRP1	2.470905	0.605429	10.08436	0.207442
LAPTM4B	1.290333	1.041047	1.599311	0.019952
UBE2W	1.205923	0.367977	3.952006	0.757183
POP1	0.535467	0.151968	1.886744	0.331042
NIPAL2	0.146886	0.008342	2.586513	0.189985
IKBKB	0.841085	0.281067	2.516924	0.756973
PLAT	0.800652	0.098887	6.48259	0.834954
DKK4	0.285016	0.013758	5.904313	0.416966
STK3	0.737919	0.39591	1.375373	0.338728
GDAP1	0.122648	0.008769	1.71548	0.118995
RAB2A	0.94108	0.48017	1.844412	0.859599
EIF3E	0.799227	0.28154	2.268823	0.673763

NDRG1	0.747845	0.415333	1.346566	0.332884
IL7	1.813991	0.194214	16.94294	0.60139
ARMC1	0.891976	0.359014	2.216125	0.805531
TRPS1	3.037169	0.817095	11.28926	0.097234
SPAG1	0.172554	0.028298	1.0522	0.056806
CHRAC1	0.536268	0.204471	1.406471	0.205286
NCALD	2.33977	1.330401	4.114942	0.003167
SNX16	0.770416	0.208334	2.848982	0.695875
UBR5	0.539067	0.233839	1.242706	0.147044
GSDMD	1.019747	0.487799	2.131789	0.958549
EEF1D	2.180371	0.911596	5.215045	0.079785
SQLE	1.950827	1.12287	3.389283	0.017732
SH2D4A	2.548601	0.81254	7.993904	0.108705
INTS10	0.351957	0.181069	0.684126	0.002074
ERI1	0.619131	0.364554	1.051486	0.076033
SLC39A14	1.612782	0.697881	3.727094	0.263428
MTMR9	0.868272	0.423372	1.780694	0.69991
LEPROTL1	1.082643	0.615152	1.905409	0.783076
DCTN6	1.146971	0.47744	2.755413	0.759111
R3HCC1	0.709504	0.296945	1.695251	0.439971
GSR	0.835708	0.260665	2.679335	0.762702
TNFRSF10/	1.149327	0.526535	2.508767	0.726756
UBXN8	0.473644	0.132243	1.696406	0.250953
PPP2CB	1.717392	0.625525	4.715136	0.293949
ERICH1	1.057447	0.472306	2.367518	0.891954
TUSC3	0.380889	0.027798	5.218962	0.469829
KLHDC4	0.656905	0.293712	1.469209	0.306217
MCM4	0.461868	0.271668	0.785228	0.004332
KCTD9	1.189312	0.367607	3.847763	0.772262
ASAH1	1.731645	0.535777	5.596721	0.358955
BNIP3L	1.080767	0.753656	1.549853	0.672812
MAN2B1	1.511945	0.918745	2.488153	0.103838
KCNN4	1.998703	1.027726	3.887041	0.041295
TULP2	3.370078	0.250253	45.38369	0.359781
NUCB1	1.354102	0.610242	3.004697	0.456009
DHDH	1.518473	0.227724	10.12526	0.666108
GYS1	1.77483	0.605674	5.200853	0.295618
MAP4K1	0.896835	0.487419	1.650148	0.726344
ECH1	0.682938	0.278745	1.673227	0.404229
HNRNPL	0.617472	0.389675	0.978436	0.040094
NFKBIB	2.011265	0.800604	5.052669	0.137073
LHB	3.030873	0.20506	44.79754	0.419707
SARS2	1.086401	0.558825	2.112053	0.806981
SNRNP70	1.089892	0.566959	2.095152	0.796293
CLPTM1	2.067888	1.144607	3.735918	0.016061
RELB	1.915418	1.042718	3.518519	0.03619
LIN7B	3.929725	1.414195	10.91981	0.008676
FCGRT	1.162538	0.894758	1.510458	0.259541
PIH1D1	2.850358	1.069669	7.595378	0.036202
CKM	0.918617	0.639563	1.319426	0.645881
ARHGEF18	1.116974	0.502705	2.481835	0.78595
PPP1R13L	2.468129	0.944764	6.447811	0.065184
PEX11G	1.633765	0.716324	3.726231	0.24325
ERCC2	0.987006	0.451925	2.155626	0.973821
DOT1L	0.750012	0.238695	2.356642	0.622397
PLEKHJ1	3.100656	1.262058	7.61777	0.013608
KLC3	0.911122	0.086256	9.62421	0.938315
CD37	1.759954	1.184657	2.614631	0.005126

SF3A2	1.331004	0.698906	2.534776	0.384309
AMH	0.565803	0.111175	2.879533	0.492712
LYL1	0.995713	0.528009	1.877702	0.989408
OAZ1	2.979359	0.493772	17.97709	0.233868
TRMT1	0.949867	0.475461	1.897629	0.884185
STX10	2.263693	1.080627	4.741974	0.03035
RETN	0.929895	0.744021	1.162205	0.522944
FCER2	0.836829	0.531839	1.316718	0.441148
DMPK	0.320537	0.020991	4.894623	0.413319
TBC1D17	3.323829	1.328649	8.31509	0.010248
IL4I1	1.580066	0.60354	4.136612	0.351523
CCDC130	1.058298	0.454819	2.462508	0.895378
PTOV1	0.968281	0.504634	1.857917	0.922774
SGTA	1.829737	0.904844	3.700015	0.092637
KIR3DX1	0.344066	0.04483	2.640661	0.304847
LILRB1	1.38646	0.589391	3.261452	0.454057
MED25	1.199255	0.699803	2.055169	0.508524
LILRA1	1.504159	0.74272	3.046226	0.256857
SNAPC2	1.831107	0.946845	3.541186	0.072233
TIMM44	0.969397	0.375259	2.504216	0.94882
CCDC61	2.057379	0.440235	9.614883	0.359116
IL27RA	2.356361	1.508035	3.681904	0.000167
ASF1B	0.927816	0.599291	1.436436	0.736901
TNNT1	1.213011	0.697992	2.10804	0.493443
VRK3	1.111704	0.454263	2.720641	0.816612
FAM32A	0.532763	0.267246	1.062081	0.073638
MED26	1.491841	0.654142	3.402304	0.341627
OLFM2	0.09273	0.006794	1.265723	0.074544
RASAL3	0.659579	0.339985	1.279601	0.218402
AKAP8	0.43293	0.16511	1.135171	0.088721
ILVBL	0.853299	0.469668	1.550284	0.602532
ZNF419	0.964138	0.333053	2.791036	0.94631
SYDE1	0.836365	0.268068	2.609442	0.758233
AURKC	0.022559	0.00016	3.171298	0.132946
POP4	1.412098	0.398405	5.005006	0.592994
CCNE1	0.945683	0.433834	2.061426	0.88829
PDCD5	0.415651	0.157776	1.095006	0.075679
ANKRD27	0.595128	0.339727	1.042533	0.069624
RPS16	0.742872	0.045048	12.25044	0.835346
TIMM50	0.094343	0.004049	2.198351	0.141666
FBL	0.694868	0.384097	1.257081	0.228765
DYRK1B	3.234214	1.497094	6.986964	0.00282
CLC	1.061986	0.918772	1.227523	0.415806
GPI	0.726487	0.4153	1.27085	0.262753
AKT2	0.648673	0.159225	2.642647	0.545871
PLD3	1.224781	0.605551	2.477232	0.572622
PRX	0.319727	0.010583	9.659777	0.511993
PIAS4	1.237179	0.521488	2.935083	0.629193
NUMBL	0.989448	0.293325	3.337624	0.986357
EBI3	1.007232	0.469645	2.160178	0.985232
SHD	0.72558	0.550334	0.956632	0.022948
TBCB	2.729121	1.137284	6.549023	0.024576
FSD1	2.2691	0.130182	39.55099	0.5742
POLR2I	1.272269	0.66433	2.436546	0.467627
CLIP3	1.289076	0.905859	1.834409	0.158338
SLC1A5	1.311669	0.79621	2.160832	0.286786
PRKD2	2.582038	1.252964	5.320922	0.010133
TJP3	2.028859	0.476186	8.644235	0.338732

APLP1	0.921329	0.015224	55.7581	0.968778
CCDC9	1.911291	0.772763	4.727235	0.160907
HNRNPUL	0.57933	0.108502	3.093251	0.52301
FZR1	5.616595	0.754592	41.80556	0.091983
BBC3	3.6789	1.209726	11.18791	0.021706
TGFB1	1.033882	0.131563	8.124745	0.974729
DENND3	0.631152	0.354111	1.124935	0.11859
CEACAM4	1.439515	0.702761	2.94866	0.319351
MRPL4	1.107637	0.381282	3.217726	0.850969
SIGLEC8	0.105337	0.008578	1.293523	0.078606
CD79A	1.545954	0.690935	3.459047	0.289049
RPS19	2.155408	0.151463	30.67275	0.570815
NKG7	0.951818	0.653039	1.387293	0.797249
ICAM5	1.342493	0.508153	3.546743	0.552379
ETFB	0.754154	0.226926	2.506314	0.645173
CD33	0.825854	0.605448	1.126495	0.227057
TYK2	1.003491	0.513613	1.96061	0.991863
CDC37	0.453954	0.16522	1.247271	0.125649
NAPA	2.222971	0.922126	5.358921	0.075178
RABAC1	1.620734	0.974925	2.694339	0.062597
PTPRS	1.877257	0.093849	37.55064	0.680313
CNFN	1.670446	0.83743	3.33209	0.145289
MEGF8	0.760916	0.183751	3.150961	0.706262
KDELR1	1.971619	0.90448	4.297811	0.087738
CYTH2	0.911731	0.537679	1.546004	0.731616
GRWD1	1.291973	0.802248	2.080646	0.292032
GRIN2D	0.24009	0.020453	2.818326	0.256207
CLEC11A	0.955118	0.790668	1.153771	0.633852
CCDC114	2.330556	0.265178	20.4824	0.445466
CARD8	0.682945	0.341444	1.366004	0.280962
LIG1	0.511542	0.267494	0.978248	0.042719
SIGLEC6	2.722682	0.645319	11.48734	0.172681
ZNF175	1.206982	0.535175	2.72211	0.650287
PLA2G4C	1.338806	0.809229	2.21495	0.255994
SIGLEC5	1.440904	0.623374	3.330592	0.392861
HAS1	0.41578	0.050696	3.410002	0.413701
RAB3D	1.44016	0.449421	4.614957	0.539288
DBP	1.937582	0.952206	3.94266	0.068024
TMEM205	1.14204	0.593135	2.19892	0.69112
CAPS	1.21742	0.412123	3.596288	0.721852
FAM83E	0.07882	0.004557	1.363278	0.080657
RASIP1	3.307918	0.970358	11.27659	0.055892
BCAT2	1.049995	0.510173	2.161009	0.894611
MIER2	2.706203	1.228797	5.959924	0.013457
PLEKHA4	3.143256	1.457328	6.779572	0.003497
PPP2R1A	2.178546	0.985486	4.815966	0.054375
TNPO2	0.605288	0.280893	1.304321	0.199949
GCDH	0.986184	0.407975	2.38387	0.975355
LILRB5	2.14145	0.664817	6.897858	0.201983
KLF1	0.864326	0.605746	1.233286	0.42146
DNASE2	1.315129	0.821314	2.105851	0.254102
MAST1	1.725718	0.62847	4.738656	0.289719
LENG1	3.398754	0.978124	11.80988	0.05421
PRPF31	0.703739	0.278728	1.776814	0.457165
TFPT	2.771924	1.273993	6.031087	0.010155
JAK3	0.450978	0.084943	2.39432	0.349829
RPL18A	0.38327	0.025156	5.839444	0.490121
SLC5A5	6.483831	0.167375	251.1732	0.316391

KCNN1	0.137564	0.008276	2.286545	0.166588
ARRDC2	7.048297	2.177852	22.81078	0.001119
RAB3A	1.530565	0.218852	10.70417	0.667987
ISYNA1	1.130073	0.720224	1.773151	0.594701
ELL	3.444098	1.588428	7.467639	0.001737
CRTC1	2.785067	1.321546	5.869337	0.007082
COPE	2.586965	1.290486	5.185942	0.007392
DDX49	1.018116	0.46245	2.241453	0.964434
ETV2	0.802036	0.298311	2.156347	0.661986
ARMC6	1.447929	0.693183	3.02445	0.324689
TMEM147	1.60848	0.789495	3.277043	0.190535
HAMP	1.899097	0.595014	6.061312	0.27873
USF2	1.983442	0.935252	4.206395	0.074189
LSR	15.75287	0.984618	252.0296	0.051295
FKBP8	1.911899	1.072424	3.408502	0.028021
ZNF14	1.10705	0.444962	2.754305	0.826893
SCN1B	3.681255	0.443976	30.52335	0.227207
PBX4	1.412683	0.504318	3.957175	0.510923
ERF	2.683316	1.55737	4.623298	0.000377
GSK3A	8.939338	1.491607	53.57426	0.016501
ATP13A1	1.099116	0.563045	2.145573	0.781846
ZNF574	1.232037	0.531178	2.857639	0.626885
GRIK5	1.684647	0.900638	3.151139	0.102592
SIPA1L3	2.336516	1.018729	5.358942	0.045094
ZNF85	0.621992	0.271417	1.425387	0.261758
ETHE1	0.674979	0.26228	1.737064	0.415063
CADM4	1.982612	0.38879	10.11022	0.41028
AVL9	0.314298	0.089799	1.100051	0.070175
RUNDC3B	0.804241	0.158166	4.089407	0.79289
GTPBP10	2.390369	0.176188	32.43044	0.512469
RASA4	1.151689	0.362289	3.661127	0.810843
CDK6	0.704544	0.525068	0.945368	0.019571
PMPCB	0.461495	0.235032	0.906166	0.024693
DNAJC2	1.198431	0.507183	2.83179	0.679911
TFPI2	6.686703	0.363209	123.1028	0.20107
BET1	0.846712	0.393726	1.820861	0.670167
NAMPT	1.000044	0.75389	1.326571	0.999757
PIK3CG	0.835747	0.439376	1.589692	0.584409
PON2	0.747083	0.547505	1.019413	0.065957
ITGB8	0.023652	0.001527	0.366234	0.007395
HBP1	1.720686	0.837364	3.535811	0.139693
DUS4L	0.20331	0.068464	0.60375	0.004123
SP4	1.01434	0.510274	2.01634	0.967601
WDR91	1.712574	0.677401	4.329651	0.255583
DNAH11	2.773059	0.215506	35.68286	0.433921
CBLL1	0.484702	0.235051	0.99951	0.049845
MTPN	0.93251	0.480605	1.809334	0.836308
MPP6	1.65954	1.010781	2.724699	0.045247
ZC3HAV1	0.900036	0.395198	2.049769	0.801965
TTC26	0.368949	0.102338	1.330141	0.127524
OGDH	8.078271	2.016303	32.3654	0.003175
ADAP1	1.735413	1.195938	2.51824	0.003709
TFEC	0.293127	0.015064	5.703786	0.417773
CAV2	1.082184	0.698524	1.676566	0.723629
CAV1	1.297069	0.787439	2.136531	0.307023
MET	1.365013	0.367382	5.071729	0.642178
RNF32	1.102036	0.179184	6.777865	0.916509
LMBR1	0.384237	0.152266	0.969608	0.042835

HOXA1	1.123674	0.04695	26.89312	0.942622
DNAJB6	0.487852	0.247982	0.959746	0.03762
HOXA2	0.811159	0.423212	1.554724	0.52836
HOXA3	1.308837	0.469546	3.648318	0.606852
LFNG	2.898801	1.63256	5.147163	0.00028
HOXA5	1.121712	0.974302	1.291425	0.110089
HOXA6	1.18564	0.952868	1.475275	0.126756
IQCE	0.400025	0.040112	3.98935	0.434908
TSPAN12	0.365946	0.046672	2.869326	0.33869
SSBP1	0.36854	0.194698	0.697603	0.002169
HOXA13	2.562833	1.11719	5.879135	0.026313
HIBADH	0.445593	0.202663	0.979722	0.044333
TAX1BP1	0.913075	0.410513	2.030887	0.823569
CPVL	1.318436	1.045774	1.662189	0.019357
CHN2	0.821567	0.390263	1.729532	0.604815
GRB10	3.126449	1.309557	7.464114	0.010247
ABHD11	2.238043	0.610576	8.203465	0.224155
FKBP14	1.000848	0.464067	2.158519	0.998276
PLEKHA8	0.355625	0.037019	3.416326	0.370438
STX1A	1.340522	0.509281	3.528502	0.552856
NOD1	0.617401	0.270965	1.406764	0.251091
CRHR2	0.292904	0.009292	9.233139	0.485528
EPHB6	1.716556	0.916094	3.216447	0.091712
CASP2	0.393918	0.189386	0.81934	0.012659
CHCHD2	1.306972	0.431427	3.959359	0.635924
CCL24	1.679429	0.127169	22.17899	0.693765
HSPB1	1.930507	1.314714	2.83473	0.000791
NPTX2	1.198562	1.010677	1.421375	0.037338
PDAP1	0.179709	0.03113	1.03743	0.054999
BUD31	0.637948	0.282759	1.439313	0.278916
PTCD1	0.837017	0.39289	1.78319	0.644763
CYP3A5	10.4254	1.246225	87.21463	0.030536
ZKSCAN1	1.495064	0.658914	3.392274	0.336025
EIF3B	0.741229	0.312157	1.760073	0.497355
SNX8	2.235755	1.318822	3.7902	0.002812
NUDT1	0.899021	0.405283	1.99426	0.793423
TAF6	3.11759	1.363474	7.128384	0.007045
WASL	1.058129	0.412721	2.712815	0.906365
AIMP2	0.339599	0.137469	0.838934	0.019254
TFR2	0.682754	0.389075	1.198104	0.183505
FSCN3	2.902073	0.424913	19.82059	0.277095
MOSPD3	1.508162	0.811514	2.802851	0.193787
PCOLCE	0.698017	0.176053	2.767503	0.60897
FBXO24	0.390465	0.008115	18.78689	0.634192
RBM28	0.296041	0.097944	0.894799	0.031011
USP42	0.58149	0.170658	1.981339	0.386062
IMPDH1	2.359041	0.770576	7.221963	0.132727
AGFG2	2.157147	0.308795	15.06919	0.438249
LSM5	0.855429	0.483193	1.514422	0.592083
SERPINE1	0.923318	0.549526	1.551364	0.763156
AP1S1	2.332141	1.151792	4.722105	0.018644
C1GALT1	2.033719	0.842249	4.910678	0.114505
PLOD3	1.324112	0.68571	2.556873	0.403052
RPA3	0.530148	0.277939	1.011218	0.054091
ZNHIT1	1.912839	0.724324	5.051543	0.190523
CLDN15	1.110701	0.550813	2.239703	0.769214
GLCCI1	0.793954	0.389042	1.620293	0.526111
PHF14	0.384855	0.152431	0.971673	0.043303

NRF1	0.544812	0.02844	10.43658	0.686848
TMEM106I	0.574651	0.322429	1.024175	0.060252
EZH2	0.325204	0.140985	0.750135	0.008435
ZNF862	3.135476	0.439658	22.36106	0.254237
SFRP4	1.573149	0.086439	28.63062	0.759554
MEST	0.614423	0.275633	1.369631	0.233692
ANKMY2	0.363873	0.194348	0.681268	0.001581
TSPAN13	0.96394	0.718409	1.293386	0.806575
RARRES2	1.394307	0.160278	12.12949	0.76329
AGR2	0.569293	0.198845	1.629888	0.293849
AHR	1.472648	0.714522	3.035163	0.294186
CHCHD3	0.217532	0.081378	0.581487	0.00236
GIMAP2	1.823669	1.176967	2.825713	0.007161
TMEM176I	1.351357	1.030531	1.772064	0.029448
PSMA2	0.451962	0.186816	1.09343	0.078101
MRPL32	0.609088	0.286461	1.295074	0.197691
BLVRA	1.236055	0.915404	1.669025	0.166634
STAG3L4	0.291802	0.10091	0.843811	0.023
RHEB	0.851511	0.294779	2.459707	0.766468
PRKAG2	2.297088	0.942139	5.600675	0.067416
AEBP1	1.163169	0.889744	1.52062	0.268937
POLD2	0.917196	0.484319	1.736971	0.790787
BCL7B	1.173159	0.477455	2.882578	0.727707
YKT6	0.471641	0.089367	2.489111	0.375891
TBL2	1.022737	0.490158	2.133986	0.952227
CLIP2	1.062705	0.50361	2.242493	0.87318
EIF4H	0.853366	0.312098	2.333351	0.757344
LIMK1	1.366502	0.253256	7.373284	0.716547
FKTN	0.633125	0.088009	4.554621	0.649818
FSD1L	0.234435	0.054784	1.003214	0.050508
CNTNAP3	0.614819	0.106219	3.558714	0.587143
SPIN1	0.797454	0.403665	1.575397	0.514691
PRUNE2	2.747046	0.067024	112.5897	0.593764
MEGF9	1.048011	0.645544	1.701397	0.84956
TRIM14	0.289943	0.034867	2.411102	0.251958
CORO2A	1.033818	0.399787	2.673374	0.945299
TGFBR1	0.871992	0.129203	5.885087	0.888184
SEC61B	1.620696	0.506178	5.189188	0.416083
C5	0.655626	0.362751	1.184958	0.162118
OGN	0.053777	0.001808	1.599376	0.091286
ASPN	0.141641	0.005795	3.461761	0.230725
ECM2	1.024979	0.032448	32.37731	0.988826
TLE4	1.172561	0.626629	2.194117	0.618524
LHX6	1.390425	0.461629	4.187957	0.557938
PTGR1	1.164936	0.750375	1.808531	0.496327
SUSD1	0.845344	0.432275	1.653131	0.623435
AKNA	1.773817	1.082525	2.906563	0.022925
TNFSF8	2.708664	1.156136	6.346018	0.021793
DNM1	1.047588	0.528966	2.074688	0.893919
ENG	1.441439	0.922946	2.25121	0.107955
AK1	1.166021	0.583191	2.331324	0.663918
CDC37L1	1.045219	0.138442	7.891255	0.965798
TBC1D13	0.622313	0.284785	1.359879	0.234347
DOCK8	1.552396	0.855537	2.816865	0.147976
KANK1	0.284137	0.07326	1.102012	0.068835
TESK1	2.588668	1.293086	5.182333	0.007237
FUBP3	0.429455	0.095142	1.93848	0.271685
CREB3	1.201265	0.365561	3.947457	0.762575

RGP1	1.31868	0.150715	11.53777	0.802609
DDX58	2.186121	0.897703	5.32373	0.085011
EDF1	1.48953	0.443239	5.005643	0.519377
PIP5K1B	0.718816	0.259779	1.988985	0.524919
GLIS3	0.07572	0.000735	7.803581	0.275179
BAG1	0.194776	0.047531	0.79817	0.023013
RAPGEF1	0.630288	0.148161	2.681285	0.532082
NPDC1	1.49624	1.128209	1.984327	0.005151
APBA1	0.585118	0.013202	25.93216	0.781739
SETX	0.679236	0.308682	1.494615	0.336431
PTGDS	1.186979	0.955738	1.47417	0.121028
ABCA2	1.167946	0.478569	2.850371	0.733072
SHB	3.350839	0.716772	15.66485	0.124351
UBE2R2	1.112899	0.359863	3.44171	0.852681
EXOSC3	1.561203	0.716597	3.401292	0.262201
ZFAND5	0.680175	0.416504	1.110765	0.12352
DVL1	4.269768	0.389809	46.7688	0.234613
PDLIM1	1.027686	0.79554	1.327575	0.834407
CCNJ	0.984463	0.324811	2.983786	0.977918
DNTT	1.205692	1.043084	1.39365	0.011386
GATA3	2.763218	0.753421	10.13427	0.12529
HPS1	1.230674	0.57706	2.624611	0.591171
PHYH	1.184445	0.529559	2.649203	0.680232
RASSF4	1.368447	0.866794	2.160428	0.178182
DNMBP	0.671109	0.110223	4.086157	0.665218
RAB11FIP2	0.536173	0.299985	0.958319	0.035409
CXCL12	1.026577	0.603966	1.744899	0.922795
ERLIN1	0.506806	0.270669	0.948954	0.033696
EIF3A	0.635999	0.346413	1.167668	0.144313
CUBN	2.608133	0.209346	32.49341	0.456344
TRDMT1	0.760026	0.201829	2.862028	0.685025
DDX50	0.54965	0.264423	1.142543	0.108928
MAPK8	1.678216	0.129982	21.66762	0.691605
SEC23IP	0.661789	0.303506	1.443021	0.299318
ATE1	0.097221	0.011773	0.802857	0.030479
NSMCE4A	0.25434	0.083534	0.7744	0.015952
PLEKHA1	0.811089	0.423846	1.552133	0.527186
UNC5B	23.69281	3.903524	143.8057	0.000581
CDH23	1.775132	0.726229	4.338983	0.208223
SPOCK2	1.157189	0.894825	1.496477	0.265763
PPP3CB	0.361808	0.14436	0.906796	0.030108
BMPR1A	0.822763	0.180706	3.746077	0.800845
MINPP1	0.853091	0.564926	1.288249	0.449921
ACTA2	0.779456	0.506313	1.199952	0.257681
LIPA	1.119834	0.653664	1.918461	0.680296
LZTS2	2.842456	1.42807	5.657675	0.002934
SFXN3	3.918378	1.746378	8.791733	0.000926
KAZALD1	7.890215	1.324812	46.99195	0.023273
FBXW4	0.697984	0.308145	1.581011	0.388734
NPM3	0.85938	0.486987	1.516536	0.601009
TNKS2	1.538489	0.208835	11.33408	0.672435
GBF1	3.240712	1.085012	9.679352	0.035195
ARHGAP2	1.315637	0.987853	1.752184	0.060605
CPEB3	1.924962	0.612961	6.045211	0.262003
FBXL15	1.998875	1.056073	3.783355	0.033374
CUEDC2	0.970088	0.394771	2.383835	0.947216
SUFU	1.27848	0.284102	5.753253	0.748869
ANKRD26	0.240024	0.054678	1.053645	0.058661

ACBD5	0.110621	0.007789	1.570987	0.103885
LHPP	1.100568	0.624176	1.940559	0.740525
LARP4B	0.954494	0.454628	2.003966	0.90205
GTPBP4	0.398117	0.19155	0.827447	0.01361
BCCIP	0.110964	0.029843	0.412597	0.001034
MTPAP	1.221659	0.124547	11.98302	0.863548
SH3PXD2A	1.136256	0.835577	1.545133	0.41534
PITRM1	1.427062	0.498596	4.084476	0.507451
MAP3K8	0.590559	0.389802	0.894712	0.012961
EBF3	0.719318	0.328124	1.576901	0.410704
GLRX3	1.207175	0.519467	2.805318	0.661652
SORCS1	0.190993	0.041633	0.876195	0.033171
XPNPEP1	0.265177	0.105279	0.667926	0.004859
SMC3	0.608759	0.283497	1.307205	0.203047
SHOC2	1.203817	0.708147	2.046433	0.493218
TFAM	0.414087	0.23629	0.72567	0.002068
CCDC6	1.506118	0.86551	2.620874	0.147353
CUL2	1.070191	0.451818	2.534891	0.877462
CCNY	0.942835	0.207369	4.286748	0.939273
UBE2S	5.546058	0.76264	40.33196	0.090592
RPL28	1.462355	0.683805	3.127328	0.327116
ZMIZ1	1.298412	0.75827	2.223316	0.341297
DNAJC12	0.793521	0.55555	1.133426	0.203574
PPIF	1.675702	0.65354	4.296566	0.282568
PBLD	0.346927	0.004615	26.07932	0.630996
TSPAN14	1.489448	0.95597	2.320634	0.078249
TBC1D12	3.182038	0.52028	19.46138	0.21028
KRT23	0.694542	0.196573	2.453995	0.571397
CRYBA1	0.904327	0.061606	13.27478	0.941512
NUFIP2	0.578365	0.337622	0.990772	0.046183
GIT1	2.501971	1.220669	5.12822	0.012262
RPL19	4.308875	0.524832	35.37588	0.173891
FBXL20	0.351683	0.143124	0.86415	0.02271
RUNDC3A	1.374359	0.956387	1.974998	0.08563
UBTF	2.250914	0.34292	14.77493	0.398039
CSF3	0.064584	0.0003	13.88114	0.31735
PSMD3	1.703384	0.689166	4.210185	0.248651
CASC3	1.109492	0.556052	2.213773	0.768148
RAPGEFL1	1.029988	0.602002	1.762246	0.914127
RGS9	0.119482	0.003448	4.140038	0.240175
RNF43	0.123417	0.007312	2.083045	0.146774
RAD51C	0.09618	0.026496	0.34913	0.000371
MTMR4	0.194616	0.077792	0.486879	0.000468
TRIM37	0.513826	0.060472	4.365909	0.541903
P2RX1	0.798291	0.49306	1.292476	0.359473
DHX40	0.075086	0.014292	0.394484	0.002221
TUBD1	0.622476	0.250932	1.544153	0.306465
KPNB1	0.476567	0.23359	0.972285	0.041628
GOSR2	1.288426	0.186181	8.916271	0.797362
PNPO	0.642194	0.322655	1.278188	0.207287
RPS6KB1	0.59373	0.296311	1.189678	0.141515
TRIM16L	1.308832	0.748991	2.287134	0.344631
CDK5RAP3	0.294492	0.156301	0.55486	0.000155
CBX1	1.283123	0.752467	2.188007	0.359914
RECQL5	0.190624	0.02064	1.760545	0.143937
PIGL	0.193773	0.028039	1.339139	0.096137
GALK1	1.506654	0.869431	2.61091	0.143964
INTS2	0.431225	0.196967	0.944096	0.035391

CAMTA2	1.83427	0.72593	4.634808	0.199594
MED13	0.797871	0.162095	3.927299	0.781248
HOXB6	1.493455	0.744076	2.997555	0.259172
ENO3	0.329493	0.099168	1.094765	0.069959
PFN1	2.531767	0.8296	7.726433	0.102722
RNF167	1.706888	0.74531	3.909063	0.20599
SLC25A11	1.614346	0.694061	3.754877	0.266129
RASD1	1.347508	1.0474	1.733606	0.020328
CHRNE	1.207111	0.149732	9.731527	0.859697
RAI1	1.168394	0.625642	2.18199	0.625299
NUP88	0.399742	0.192825	0.828699	0.013697
C1QBP	0.711599	0.363772	1.392009	0.320298
SLC6A4	0.34009	0.031031	3.727294	0.37728
BLMH	0.553478	0.265842	1.152332	0.113877
CPD	0.986688	0.715024	1.361565	0.934993
GOSR1	0.442086	0.073898	2.644747	0.371139
CCDC47	0.550252	0.228863	1.322963	0.181985
MED31	0.897523	0.197105	4.086893	0.888828
DRG2	1.387288	0.51218	3.757601	0.519644
FTSJ3	0.669866	0.21766	2.061566	0.484809
AKAP10	0.528857	0.182908	1.529123	0.239605
SMARCD2	1.874805	0.971317	3.61869	0.061037
ICAM2	2.026845	1.150585	3.570447	0.014464
SYNGR2	2.385614	0.490278	11.60802	0.281471
B9D1	1.622212	0.713184	3.689891	0.248576
UTP6	0.594316	0.306119	1.153835	0.124236
DDX5	0.602994	0.34591	1.051143	0.074415
CYTH1	1.687293	1.009341	2.820611	0.045996
PSMD11	1.499217	0.40235	5.586317	0.546255
LGALS3BP	1.064644	0.867766	1.306189	0.548218
CCL7	1.447961	0.220807	9.495146	0.699665
CCL2	1.963981	1.177074	3.276957	0.009763
CCL1	1.300528	1.04347	1.620911	0.019352
PEX12	0.588559	0.013466	25.72339	0.78329
DHX58	1.302891	0.620637	2.735133	0.48438
KAT2A	0.592267	0.344569	1.018026	0.05805
RAB5C	3.17036	1.396842	7.195646	0.005795
NAGLU	1.232207	0.5549	2.736229	0.607954
HSD17B1	0.284913	0.054091	1.500727	0.138581
MLX	3.030649	0.82196	11.17431	0.095821
CNTNAP1	1.004093	0.326288	3.089915	0.994317
ABI3	1.472173	1.06629	2.032555	0.018774
EZH1	4.415584	0.643088	30.31836	0.130827
DLX4	7.941981	0.084241	748.749	0.371672
PPP1R9B	1.7059	0.749498	3.882724	0.203091
COL1A1	1.324734	0.45435	3.862491	0.606511
MRPL27	0.858622	0.148468	4.965587	0.864828
VAT1	0.981131	0.64644	1.489106	0.928695
LRRC59	1.189103	0.365823	3.865163	0.773367
RND2	1.14026	0.228581	5.688099	0.872823
ALOX12	1.200867	0.73579	1.959909	0.463936
HDAC5	1.642622	0.124851	21.61144	0.705823
ABCC3	1.589711	0.822471	3.072668	0.167992
MPP2	3.508067	0.39661	31.02929	0.259128
SMURF2	0.554252	0.05268	5.831323	0.623085
DUSP3	1.487913	0.836561	2.646414	0.1762
EFTUD2	1.027871	0.373872	2.825885	0.957513
HLF	1.736079	0.599274	5.029365	0.30941

SLC16A6	2.27407	1.2374	4.179241	0.008145
PRKAR1A	0.890034	0.558512	1.418339	0.624139
FAM20A	1.997495	0.984843	4.051394	0.055157
YWHAE	0.769479	0.207	2.860376	0.695679
MMD	1.101302	0.78651	1.542084	0.574259
RANGRF	1.162857	0.723234	1.869709	0.533485
DPH1	0.937253	0.260358	3.373986	0.921014
MAP2K6	0.021992	0.0009	0.537252	0.019232
DHRS7B	1.801724	0.818521	3.965946	0.143603
WSB1	0.618522	0.31107	1.229849	0.170691
RCVRN	1.477867	0.320744	6.80944	0.616289
SLC9A3R1	1.928941	1.012509	3.674842	0.045742
MYH3	0.343653	0.15794	0.747737	0.007084
NAT9	0.464307	0.205779	1.047634	0.064619
TMEM104	1.689493	0.81782	3.490238	0.156576
VTN	0.717948	0.041227	12.50263	0.820194
TNFAIP1	2.316334	0.974747	5.504408	0.057165
IFT20	1.203364	0.607819	2.382428	0.595258
TMEM97	0.444181	0.170876	1.154617	0.095914
CDR2L	1.149365	0.716374	1.844065	0.563852
PMP22	0.530345	0.130542	2.154597	0.375218
UNC119	1.84513	0.903829	3.766761	0.092517
ALDOC	1.72746	1.014315	2.942003	0.04419
SUPT6H	4.91814	1.285855	18.81091	0.019949
RAB34	1.543467	1.139024	2.09152	0.005117
PHF12	4.757491	0.567064	39.91388	0.150652
TMEM33	0.621245	0.19691	1.960008	0.416776
GNRHR	2.334083	0.297641	18.30371	0.419863
SLAIN2	0.941733	0.230455	3.848301	0.933385
OCIAD1	0.402819	0.151363	1.072011	0.068649
DCUN1D4	0.294366	0.113999	0.760109	0.011515
USP46	0.271415	0.069048	1.06688	0.061864
CHIC2	1.24034	0.431742	3.563337	0.68914
NMU	0.553794	0.239501	1.280528	0.167036
PF4V1	1.02917	0.732191	1.446604	0.868536
NFKB1	0.812491	0.415259	1.589711	0.544279
AREG	0.638201	0.039978	10.18823	0.750688
MANBA	0.585354	0.309718	1.106298	0.099163
UBE2D3	0.207276	0.031138	1.379794	0.103715
ELF2	0.346796	0.130581	0.921019	0.033582
NDUFC1	1.935266	0.991837	3.776076	0.052876
TBC1D9	1.472468	0.970919	2.233102	0.068596
ZNF330	0.728347	0.237989	2.229055	0.57861
INPP4B	1.269731	0.743464	2.168519	0.381865
GAB1	1.125548	0.078282	16.1833	0.930705
KLHL2	1.23168	0.713131	2.127289	0.45484
RPL34	0.947611	0.63061	1.423965	0.795656
WFS1	1.456875	0.860247	2.467296	0.161535
GRPEL1	0.773634	0.310078	1.930187	0.582179
GAR1	0.286445	0.130602	0.628249	0.001809
FRG1	0.638303	0.234623	1.736535	0.379305
CLCN3	1.428968	0.516382	3.954342	0.49187
AADAT	0.782905	0.028443	21.54982	0.884949
GALNT7	0.921651	0.307863	2.759153	0.884051
DHX15	0.489559	0.25225	0.950123	0.034756
SEPSECS	0.17064	0.018782	1.550273	0.116289
SLC2A9	2.238876	0.753258	6.65452	0.147017
FBXW7	0.969484	0.182166	5.159582	0.971018

NEIL3	0.210311	0.049337	0.896499	0.035061
TBC1D19	0.364546	0.126497	1.050563	0.061675
CLNK	0.577792	0.059833	5.579554	0.63542
SH3D19	1.450627	0.278262	7.562376	0.658808
STIM2	1.063419	0.302555	3.737702	0.923618
MFSD10	0.334776	0.168388	0.665574	0.001802
GLRB	0.135899	0.014218	1.298954	0.083119
BST1	1.055396	0.744356	1.496409	0.762151
RAPGEF2	1.245008	0.774446	2.001488	0.365619
HGFAC	0.283226	0.028104	2.854308	0.284531
SNX25	1.746531	0.504931	6.04117	0.378472
LRP2BP	4.130848	0.562502	30.3357	0.163203
UFSP2	0.931318	0.393801	2.202518	0.871289
KLF3	1.364155	0.318225	5.847814	0.675834
KLHL5	0.517008	0.130618	2.046407	0.347312
NCAPG	0.785463	0.52773	1.169068	0.234
UGDH	0.715421	0.350078	1.462037	0.358433
HTATIP2	1.197387	0.818451	1.751767	0.353431
CTSC	1.674699	0.699891	4.007219	0.246719
CCDC34	0.546318	0.256454	1.163804	0.117157
ZBTB16	0.758556	0.481611	1.194754	0.233164
ELP4	1.329355	0.452518	3.905227	0.604599
MTCH2	0.397938	0.082996	1.907984	0.249253
FNBP4	0.563537	0.323809	0.980743	0.042484
TECTA	0.292467	0.039978	2.139585	0.225957
CRTAM	0.63076	0.061774	6.440587	0.697471
HSPA8	0.938092	0.501824	1.753637	0.841311
VWA5A	1.176416	0.613608	2.25544	0.624663
DNAJC4	2.223123	0.839484	5.887278	0.107871
SIAE	2.331755	0.419089	12.97358	0.333634
LPXN	0.953999	0.594116	1.53188	0.845477
DTX4	1.568482	0.18486	13.30808	0.679917
ATG2A	1.216522	0.638392	2.318209	0.551336
EHD1	0.783063	0.408504	1.501058	0.461386
OSBP	1.565601	0.658309	3.723336	0.31052
UNC93B1	1.728872	1.174453	2.545015	0.005519
PUS3	1.213896	0.440575	3.344588	0.707775
DCPS	0.832498	0.344776	2.010154	0.683571
FOXRED1	0.32887	0.135475	0.798344	0.013984
NRXN2	1.160041	0.698008	1.927907	0.566786
MS4A6A	1.18941	0.966462	1.463789	0.101452
MS4A4A	2.341923	1.237713	4.431238	0.008912
ST3GAL4	1.111094	0.706392	1.747656	0.648491
CPT1A	0.759341	0.188894	3.052494	0.698135
CCND1	1.209937	0.834341	1.754617	0.314939
CCDC86	0.888586	0.490586	1.609471	0.696726
PRPF19	0.864678	0.390028	1.916962	0.720384
TMEM109	1.427497	0.756712	2.692899	0.271723
HPX	0.051497	0.003387	0.782984	0.032668
TRIM3	49.02973	5.167675	465.183	0.000697
CHORDC1	0.937733	0.284378	3.092168	0.915894
FOLR1	0.672403	0.003253	138.9758	0.883988
FOLR3	1.033059	0.873679	1.221513	0.703631
PANX1	2.095964	0.66352	6.620853	0.207314
ARHGEF17	1.427254	0.755794	2.695251	0.27274
CEP164	7.281762	2.683639	19.75827	9.69E-05
RNF141	1.61226	0.508133	5.115553	0.417499
EIF4G2	0.577254	0.319064	1.044372	0.069303

IL10RA	1.351608	1.022895	1.785953	0.034075
BIRC2	0.692623	0.26365	1.819555	0.456104
UBE4A	0.528156	0.224401	1.243086	0.14382
DDX6	0.855008	0.232012	3.150868	0.81391
UPK2	0.053259	0.003187	0.890055	0.041248
CBL	1.509597	0.774717	2.941567	0.226275
HIPK3	1.044157	0.356627	3.057153	0.937164
FBXO3	0.583995	0.119197	2.861226	0.507085
PDHX	0.323707	0.119594	0.876183	0.026409
SLC1A2	0.067723	0.005438	0.84345	0.036414
COMMD9	1.159749	0.508055	2.64739	0.724889
SLC15A3	1.181149	0.963026	1.448677	0.109979
CD5	1.159233	0.75838	1.771962	0.494927
ACCS	0.726383	0.549563	0.960093	0.024697
MDK	2.071447	1.197529	3.583121	0.009196
AMBRA1	4.23897	1.075081	16.71397	0.039076
MADD	1.417483	0.315635	6.365763	0.648935
PTPMT1	0.476217	0.202686	1.118888	0.088715
SLC22A18	2.45242	1.164143	5.166345	0.018287
CD81	1.131258	0.569764	2.246095	0.724512
SLC35F2	0.84255	0.544111	1.304681	0.44255
SOX6	1.58032	0.308246	8.102012	0.583173
PITPNM1	1.425689	0.905775	2.244032	0.125432
RPS13	1.53357	0.52514	4.478496	0.434206
AIP	2.084039	1.066227	4.073448	0.031753
NUP98	0.334664	0.06348	1.764345	0.196857
NDUFS8	0.793516	0.35333	1.782095	0.575291
TCIRG1	1.501544	0.80795	2.790564	0.198603
CHKA	0.339309	0.088383	1.302631	0.115312
EXPH5	0.032777	0.002786	0.385573	0.006573
HPS5	0.673673	0.191575	2.368973	0.538101
GTF2H1	0.641636	0.298367	1.379832	0.256026
POU2AF1	1.48723	0.892854	2.477284	0.127351
VWF	1.289458	1.037604	1.602443	0.021853
PSMD9	0.862813	0.302867	2.457997	0.782356
PPFIBP1	1.647111	0.442211	6.135025	0.457008
PRPF40B	0.039841	0.001349	1.176758	0.062078
CD69	0.941915	0.718668	1.234512	0.66461
PRDM4	0.438635	0.207921	0.925356	0.030492
CLEC2B	0.832238	0.529964	1.30692	0.425158
COQ5	0.561766	0.265621	1.188084	0.131301
SELPLG	1.509182	0.917886	2.481386	0.104752
CORO1C	1.648786	0.834249	3.258612	0.150266
CAPRIN2	0.647697	0.337965	1.241288	0.190641
TSPAN11	0.233745	0.003957	13.8072	0.484884
KCTD10	1.727611	0.776822	3.842116	0.180022
SLC11A2	0.557695	0.351918	0.883796	0.012925
MLEC	0.711445	0.414935	1.219839	0.215865
MVK	2.528744	0.934297	6.844234	0.067822
CSRNP2	0.777447	0.333533	1.812187	0.559874
CAMKK2	0.657344	0.380428	1.13583	0.132702
BIN2	0.91681	0.486406	1.728066	0.788264
IL23A	1.867952	0.706688	4.937464	0.207693
PTGES3	1.050472	0.582337	1.894938	0.870056
BCL7A	1.041817	0.272011	3.99022	0.952321
RSRC2	0.720067	0.334621	1.549503	0.400949
CYP27B1	0.293953	0.058949	1.4658	0.135308
LIN7A	0.85973	0.498287	1.483354	0.58707

KRT18	2.309456	1.16331	4.584839	0.016742
GLI1	0.420809	0.036869	4.802895	0.485945
PPM1H	0.536772	0.304014	0.947732	0.031949
METAP2	0.465669	0.234522	0.924637	0.028973
LTA4H	1.006163	0.599155	1.689654	0.981465
ELK3	4.745532	1.01483	22.19098	0.047853
SLC6A12	1.183016	0.712643	1.963853	0.515746
WNT5B	4.286948	0.583283	31.50772	0.152643
MAGOHB	0.556867	0.240359	1.290156	0.172045
TRPV4	1.555315	0.250052	9.673991	0.635768
ITFG2	0.634677	0.314906	1.279162	0.203575
FOXM1	0.946689	0.490832	1.825917	0.87015
PRR4	0.68234	0.265075	1.756439	0.428173
PARP11	0.752537	0.329473	1.71884	0.4999
ARPC3	1.284763	0.553531	2.981977	0.559714
GPN3	1.016536	0.315125	3.279168	0.978103
VPS29	0.541509	0.277441	1.056914	0.072219
RAD51AP1	0.501808	0.290617	0.866473	0.013352
CUX2	1.815007	0.767535	4.291983	0.174634
SH2B3	1.89245	1.237676	2.893622	0.003238
MANSC1	1.171776	0.564204	2.43362	0.670758
DUSP16	1.305452	0.432131	3.943721	0.636542
CREBL2	1.179708	0.381077	3.652045	0.774382
ACAD10	0.718461	0.311889	1.655028	0.43739
ALDH2	1.494628	1.143483	1.953604	0.003269
CDKN1B	1.473488	0.854026	2.542272	0.163638
GPRC5D	2.192594	0.390672	12.30565	0.372373
GSG1	0.183418	0.002623	12.82561	0.433857
SCNN1A	0.678419	0.140366	3.278955	0.629333
LTBR	2.024207	1.236173	3.314597	0.005069
OGFOD2	0.102459	0.011712	0.896313	0.039505
CDK2AP1	0.483564	0.190519	1.227352	0.126292
OAS3	2.74378	1.36707	5.506907	0.004517
OAS2	1.584871	0.917495	2.737689	0.098696
ART4	1.011453	0.402256	2.54325	0.980688
MGP	1.423087	0.505763	4.0042	0.50384
RASAL1	0.154084	0.016655	1.425515	0.099428
ARHGDIB	0.546302	0.238878	1.249368	0.152012
GTF2H3	0.262343	0.101101	0.680742	0.005951
EIF2B1	0.243894	0.062704	0.948648	0.041747
DDX55	0.287599	0.137047	0.603542	0.000984
SLC38A1	1.133297	0.716248	1.79318	0.593008
VDR	3.921642	1.165362	13.197	0.027305
RFC5	0.095066	0.024344	0.371238	0.00071
STX2	1.467056	0.842826	2.553615	0.175326
COPZ1	1.862165	0.714194	4.855341	0.203529
CAND1	0.747887	0.362154	1.544467	0.432367
IFNG	1.009385	0.640964	1.589571	0.967842
RAB5B	0.512182	0.244986	1.070799	0.075377
MDM1	0.030133	0.002999	0.302765	0.002931
NUP107	0.29987	0.149606	0.601058	0.000687
CNOT2	0.722825	0.459018	1.138246	0.161201
TIMELESS	0.497964	0.311485	0.796085	0.003584
CPSF6	0.183955	0.056587	0.59801	0.004882
KRR1	0.346912	0.132382	0.909097	0.031251
MRPL51	0.425443	0.212577	0.851463	0.01577
GAPDH	1.385095	0.468333	4.096419	0.555976
NOP2	0.363044	0.093298	1.41269	0.143855

CHD4	1.254389	0.598577	2.628721	0.548224
ACRBP	1.134625	0.837063	1.537965	0.415716
UHRF1BP1	1.456621	0.507281	4.182581	0.48463
COPS7A	1.599256	0.669888	3.817984	0.290252
ING4	0.823822	0.241532	2.809903	0.756878
GNB3	0.17496	0.036626	0.835765	0.028902
CDCA3	0.851041	0.534167	1.355889	0.497292
CHPT1	1.202557	0.617179	2.343151	0.587843
USP5	1.571538	0.796411	3.101074	0.19239
TPI1	1.40519	0.512802	3.85053	0.50835
GNPTAB	0.881306	0.300085	2.588267	0.818197
SPSB2	1.21334	0.760912	1.934777	0.416643
ENO2	1.389921	0.865386	2.232391	0.173225
ATN1	3.930549	1.259086	12.27018	0.018442
PTPN6	4.341491	1.059868	17.78385	0.041272
LPCAT3	1.553611	0.876698	2.753182	0.131249
NT5DC3	0.943032	0.432674	2.055379	0.882695
SUDS3	0.62952	0.120526	3.288046	0.583205
GOLT1B	1.212584	0.602156	2.441824	0.5894
LDHB	0.547148	0.322655	0.927835	0.025226
PRKAB1	0.490088	0.207908	1.155251	0.103081
CMAS	0.59797	0.291382	1.227144	0.16094
HCFC2	0.868591	0.276672	2.726878	0.809276
CLEC4A	1.219268	0.932917	1.593511	0.146628
RAB35	2.702997	0.977613	7.473501	0.055323
PHC1	0.826726	0.352268	1.940215	0.661984
COX6A1	2.775376	1.051725	7.323886	0.039224
RIC8B	0.41519	0.139525	1.235501	0.114136
FGFR1OP2	1.029739	0.544565	1.947173	0.928161
KLRB1	0.974426	0.747373	1.270458	0.84821
BTN3A3	0.997355	0.54905	1.811704	0.993061
DSE	1.857773	0.58893	5.860327	0.290651
RWDD1	0.524704	0.163618	1.682666	0.278047
MAK	10.97392	0.427092	281.9698	0.148089
TMEM14C	0.57909	0.266888	1.256501	0.166897
PAK1IP1	0.536984	0.170731	1.688926	0.287541
GCNT2	3.458351	0.398817	29.98919	0.260225
NEDD9	1.605434	0.526429	4.896041	0.405344
ASF1A	1.303066	0.688549	2.466028	0.416004
MCM9	0.07584	0.004977	1.155734	0.063479
RNGTT	0.884147	0.407748	1.917158	0.755185
MAN1A1	0.847415	0.525755	1.365869	0.496639
GABRR2	0.989083	0.062789	15.58062	0.993774
SERINC1	1.153609	0.610245	2.180784	0.660071
HDDC2	0.306573	0.183998	0.510805	5.65E-06
HINT3	0.187751	0.031516	1.118485	0.066212
NCOA7	0.905444	0.64765	1.26585	0.56123
SASH1	4.775694	2.616246	8.71755	3.54E-07
UST	1.308427	0.803208	2.131431	0.280248
FBXO5	0.859276	0.363452	2.031507	0.729741
MTRF1L	0.279552	0.07852	0.995276	0.049152
PPARD	3.687819	0.202594	67.12935	0.378033
FANCE	0.755912	0.403135	1.417397	0.382972
SLC26A8	0.749521	0.229188	2.451183	0.633419
MAPK14	0.437624	0.102345	1.871266	0.264969
RHAG	1.021671	0.836101	1.248429	0.833949
KCTD20	0.946293	0.451012	1.98547	0.88392
STK38	0.903865	0.569465	1.434631	0.66806

SOD2	1.116435	0.670249	1.859647	0.672243
MRPL18	0.50914	0.243124	1.066217	0.073462
MCM3	0.425535	0.228852	0.791254	0.006938
RNF8	1.255172	0.185368	8.49909	0.815847
PHACTR1	0.85801	0.435564	1.69018	0.657973
MDGA1	2.000423	0.673702	5.939858	0.211787
FBXO9	0.03361	0.002284	0.494665	0.013399
CD83	1.072427	0.835851	1.375962	0.582395
MDN1	0.228966	0.06426	0.81583	0.02297
BACH2	2.074099	0.549413	7.829962	0.281774
TREML2	1.344073	0.591219	3.055603	0.480376
ZNF451	0.412577	0.114726	1.483706	0.175171
BAG2	1.956489	0.549752	6.96287	0.300095
RAB23	1.063164	0.25535	4.426533	0.932928
KHDRBS2	0.331699	0.021663	5.078926	0.427976
FBXL4	0.247853	0.018679	3.288695	0.290297
CCNC	0.543946	0.19772	1.496446	0.238285
E2F3	0.531937	0.233966	1.209393	0.131993
PTP4A1	0.518074	0.229698	1.168492	0.113027
ASCC3	0.468751	0.10119	2.17143	0.332712
BVES	8.574223	0.119017	617.7057	0.324808
MED23	0.315422	0.096691	1.028957	0.055793
WASF1	0.434991	0.159711	1.184748	0.103453
GPLD1	0.269667	0.01158	6.279935	0.414508
ALDH5A1	0.830332	0.508296	1.356395	0.457752
VNN1	1.391134	0.992404	1.950068	0.055401
VNN2	1.100318	0.822619	1.471761	0.51945
SMAP1	0.585222	0.166518	2.056747	0.40346
RPS12	1.210937	0.116259	12.61296	0.872816
B3GAT2	0.085716	0.002952	2.488992	0.152889
GMNN	0.674757	0.390937	1.164628	0.157746
SOBP	0.887378	0.08849	8.898575	0.919088
SNX3	1.062395	0.237131	4.75974	0.93695
HBS1L	1.008399	0.044572	22.81422	0.995807
TRIM38	1.337957	0.698719	2.562014	0.379744
PEX7	0.299468	0.068823	1.303066	0.108027
ZBTB24	0.933531	0.245938	3.543495	0.9195
FIG4	0.283243	0.11715	0.684819	0.005103
PERP	1.330079	0.191694	9.228834	0.772883
SLC16A10	0.403041	0.109003	1.490253	0.173194
HECA	0.984033	0.619653	1.562682	0.945618
PHACTR2	1.155477	0.65819	2.028484	0.614757
EPM2A	0.189886	0.014434	2.498063	0.206367
SLC39A7	0.545034	0.058122	5.110984	0.595116
PHF1	1.348539	0.549167	3.311485	0.514163
CUTA	0.740525	0.338663	1.619242	0.451717
QKI	1.529622	0.305169	7.667043	0.6053
MDFI	0.412816	0.070302	2.424075	0.327285
TFEB	3.965534	1.746996	9.001428	0.000988
CCND3	1.345934	0.79616	2.275344	0.267423
BYSL	1.169108	0.620371	2.203218	0.628916
FAM120B	0.985153	0.416721	2.328961	0.972818
TBP	0.388734	0.100608	1.50201	0.170661
GUCA1B	1.024213	0.185677	5.649668	0.978093
PRPH2	3.447767	0.444219	26.75952	0.236473
PPP2R5D	1.622466	0.470938	5.589687	0.443198
MRPL2	1.099006	0.553597	2.181759	0.787287
PTK7	1.326749	0.838706	2.098784	0.226944

SRF	0.537343	0.297721	0.969826	0.03924
CUL9	0.830777	0.369094	1.869959	0.654244
DUSP22	2.769802	1.022678	7.501677	0.045062
EXOC2	1.305757	0.322844	5.281204	0.708261
COX7A2	1.033515	0.616356	1.733012	0.900524
TMEM30A	0.554331	0.294958	1.041784	0.066832
GMDS	0.734489	0.500857	1.077101	0.114167
SENP6	0.611078	0.223902	1.667764	0.336309
VEGFA	0.624066	0.163608	2.380435	0.490025
PRPF4B	0.366273	0.123452	1.086707	0.070279
TTK	0.776352	0.419217	1.437734	0.420719
SLC29A1	0.746658	0.31162	1.78903	0.512286
BTN2A1	0.545668	0.158429	1.879419	0.337056
LAMA4	0.116663	0.002975	4.574213	0.251079
CLIC5	0.073363	0.0039	1.380207	0.081029
LY86	1.148216	0.935984	1.408572	0.18501
HARS2	0.614845	0.256588	1.473314	0.275337
NUDT12	4.8974	0.595844	40.25306	0.139354
CEP72	2.153058	0.503019	9.215677	0.301258
MAN2A1	0.799879	0.229856	2.78351	0.725618
HMGCS1	0.937119	0.528887	1.660451	0.823907
DAP	1.121692	0.505902	2.48703	0.777427
BRD8	1.052146	0.389821	2.839793	0.920074
KIF20A	0.586273	0.342692	1.002987	0.051286
NNT	0.651957	0.242035	1.756143	0.39748
MRPS30	0.58529	0.263901	1.298079	0.187495
HSPA9	0.486865	0.256667	0.923523	0.027557
MRPS27	0.945341	0.509442	1.754212	0.858566
PFDN1	1.016007	0.366583	2.815925	0.975643
HBEGF	1.272054	1.00963	1.602687	0.041224
LOX	1.085115	0.301236	3.908816	0.90058
GZMK	1.033493	0.781072	1.36749	0.81764
CDH9	0.36806	0.079124	1.712107	0.202533
APBB3	0.65019	0.376739	1.122123	0.122072
TMCO6	1.156454	0.423136	3.16065	0.7769
SPARC	1.347157	0.826416	2.196027	0.231989
IK	0.693183	0.349789	1.373693	0.293659
HMGCR	0.960298	0.499046	1.84787	0.903447
FAF2	0.842929	0.373194	1.903914	0.681047
PDE8B	1.223672	0.303721	4.930096	0.776478
CLK4	1.181496	0.554997	2.515209	0.665283
HAVCR1	0.244863	0.016298	3.678773	0.308789
ITK	0.94564	0.653038	1.369345	0.767308
RNF130	0.649002	0.288401	1.460478	0.296166
THG1L	0.634521	0.265846	1.514472	0.30544
ARSB	0.671339	0.163526	2.75612	0.580262
CLINT1	0.574178	0.30122	1.094481	0.09186
THBS4	0.666652	0.332805	1.335389	0.252628
CNOT6	0.610074	0.312208	1.192124	0.148231
BTNL8	1.256701	0.151611	10.41678	0.832303
TTC1	0.871316	0.366649	2.070623	0.755112
MSH3	0.094245	0.015632	0.56821	0.009977
RASGRF2	0.546559	0.038994	7.660755	0.65382
CCNG1	1.24098	0.609576	2.526398	0.551676
POLR3G	0.494667	0.053169	4.602263	0.536232
LMNB1	0.691587	0.412974	1.158164	0.160977
ARRDC3	1.638047	0.791219	3.391222	0.183777
GOLPH3	1.160067	0.678788	1.982587	0.587125

SUB1	0.738438	0.413954	1.317274	0.304513
NPR3	0.935734	0.591083	1.481347	0.776868
FAM172A	0.429786	0.177704	1.039456	0.060919
SLC27A6	0.787383	0.209156	2.964152	0.72377
LNPEP	1.395773	0.083516	23.32697	0.816484
PDE4D	0.830347	0.435514	1.583131	0.572309
RAD1	1.690442	0.135608	21.07241	0.683393
BRIX1	0.373284	0.17497	0.796369	0.010805
PRLR	2.511801	0.52842	11.93964	0.246874
SLC12A7	0.236234	0.018436	3.027021	0.267501
RAD50	0.246141	0.046952	1.290359	0.097238
ST8SIA4	1.433784	0.4715	4.359988	0.525433
GNPDA1	1.311744	0.989976	1.738095	0.058785
PCDH12	1.084498	0.171007	6.877708	0.93141
SKP1	0.491137	0.255346	0.944661	0.033126
NUP155	0.138222	0.028933	0.660343	0.013136
PPP2CA	0.610472	0.25612	1.455083	0.265436
NR3C1	1.157465	0.200042	6.697217	0.870307
PPWD1	0.731104	0.400037	1.33616	0.308673
LIFR	0.309465	0.03777	2.535571	0.27441
TRIM23	0.288531	0.032788	2.539053	0.26263
SEC24A	1.145779	0.518803	2.53046	0.736392
TXNDC15	1.959245	0.86553	4.435017	0.106634
TTC33	0.907916	0.448838	1.836546	0.788115
TCERG1	0.180428	0.058334	0.558065	0.002955
DPYSL3	1.244024	1.036097	1.493679	0.019285
SMAD5	0.530026	0.181393	1.548723	0.245889
CSNK1A1	0.657136	0.192609	2.241991	0.502506
ERGIC1	1.687061	0.705874	4.03213	0.23942
PDGFRB	1.752276	1.159477	2.648153	0.007762
ATP6V0E1	1.048316	0.468971	2.343354	0.90847
BNIP1	0.689804	0.254718	1.868059	0.465042
CPEB4	1.279726	0.777384	2.106679	0.332142
HRH2	1.824763	0.292557	11.38157	0.519593
DBN1	1.123846	0.718623	1.757569	0.608831
ZNF346	0.235074	0.091322	0.605112	0.002689
UNC5A	0.245834	0.014409	4.194149	0.332339
EHHADH	0.631448	0.18784	2.122695	0.457361
SMC4	0.820265	0.36867	1.825031	0.627271
ACTR8	0.177501	0.048072	0.655409	0.00949
TBCCD1	0.831477	0.182853	3.780924	0.811236
CRBN	0.965206	0.500094	1.862896	0.915933
BCL6	1.105429	0.726161	1.682784	0.640139
HGD	3.692263	1.079892	12.62422	0.037297
ARL6	0.063231	0.000919	4.348459	0.20088
NPHP3	0.658627	0.367373	1.18079	0.160908
CD86	1.416807	1.004562	1.998228	0.047044
AMOTL2	0.409281	0.112775	1.485357	0.174349
NIT2	0.311703	0.14779	0.657411	0.002202
FAM162A	0.650513	0.357879	1.18243	0.158438
OGG1	0.358645	0.14478	0.888428	0.026721
KPNA1	0.831349	0.33607	2.056541	0.689381
PCCB	0.438014	0.215432	0.890565	0.022603
UBE3A	0.441259	0.155151	1.254969	0.125005
ARMC8	0.139873	0.032344	0.604883	0.008467
CEP70	0.944864	0.542487	1.645694	0.841223
RBP2	0.121932	0.01426	1.042633	0.054627
SLC25A36	0.645686	0.397597	1.048576	0.077023

GRK7	5.968372	0.63189	56.37291	0.118926
RNF7	1.094809	0.0956	12.5377	0.941954
TFDP2	0.694657	0.390825	1.234693	0.214404
XRN1	0.565886	0.209725	1.526887	0.260904
KAT2B	0.899257	0.633809	1.275877	0.551883
SERPINI2	1.334063	0.766863	2.320785	0.307584
PDCD10	0.946282	0.374469	2.391249	0.907068
PFKFB4	1.116828	0.800138	1.558862	0.51606
COL7A1	0.554567	0.195131	1.576098	0.268605
PRKAR2A	0.762121	0.245358	2.367267	0.638524
HES1	1.413855	0.719296	2.779085	0.315187
USP4	0.754095	0.198557	2.863963	0.678482
ACAP2	0.966245	0.54729	1.705913	0.905753
ECT2	0.603477	0.326343	1.115956	0.107358
GNAT1	1.014538	0.017344	59.34522	0.994453
GNAI2	1.817803	0.883307	3.740951	0.104591
TFG	0.5751	0.170181	1.943454	0.373227
USP9Y	0.439647	0.156317	1.236525	0.119335
HYAL1	0.009699	6.21E-05	1.514669	0.072042
TUSC2	1.501017	0.547586	4.114519	0.429871
RPL24	1.180621	0.354288	3.934267	0.78688
CYB561D2	1.356925	0.628416	2.929981	0.437076
FXR1	0.210236	0.08538	0.517676	0.000694
CBLB	1.060286	0.460618	2.440647	0.890544
BBX	0.926652	0.512289	1.676172	0.801111
IFT57	0.768812	0.344786	1.714314	0.520502
GNB4	1.728079	0.638621	4.676101	0.281473
HHLA2	1.598384	0.176912	14.44121	0.676229
IQCG	0.911946	0.343994	2.417617	0.852995
GBE1	0.675123	0.381626	1.194339	0.177083
UMPS	2.11648	0.564178	7.939843	0.266375
NCBP2	0.352132	0.117279	1.057281	0.062791
SNX4	0.670288	0.317996	1.412865	0.293023
FRMD4B	1.54131	0.319138	7.443912	0.59026
SLC41A3	0.858478	0.225897	3.262487	0.822746
PLXNA1	1.430168	0.665466	3.073607	0.359348
ATP6V1A	0.9351	0.513407	1.703155	0.826379
ABTB1	1.016822	0.61732	1.674865	0.947762
PODXL2	1.35022	0.807618	2.257373	0.252161
UPK1B	2.108263	0.314982	14.11118	0.441918
CSPG5	0.230852	0.021986	2.423925	0.221724
KLHL18	0.895107	0.335385	2.388948	0.824903
SCAP	0.596678	0.303744	1.172119	0.133885
NEK11	2.664191	0.718777	9.874981	0.142657
MRPL3	0.639459	0.303887	1.34559	0.238811
PLSCR4	1.180016	0.58437	2.382803	0.644328
HEMK1	0.286514	0.086424	0.949852	0.040944
CISH	2.11719	1.450549	3.090205	0.000101
MAPKAPK	1.200397	0.510489	2.822688	0.675447
ACVR2B	0.458114	0.010948	19.16909	0.681981
WDR48	0.67983	0.369636	1.250337	0.214484
COMMD2	1.013396	0.464534	2.210759	0.973326
GORASP1	1.03587	0.454014	2.363418	0.933266
RRP9	1.066472	0.480057	2.369226	0.874438
ABCC5	0.253108	0.098811	0.64835	0.004198
ABHD14B	2.696457	1.193164	6.093779	0.017102
EIF1B	0.658815	0.329745	1.316279	0.237303
KLHL24	0.942371	0.560252	1.585113	0.822976

PLCH1	0.656459	0.129894	3.317606	0.610627
VIPR1	1.773605	1.207417	2.605294	0.003493
DNAH1	1.002107	0.436891	2.298555	0.996035
SSR3	0.711347	0.272546	1.856618	0.48653
ZBTB47	2.304601	0.747031	7.109725	0.146346
NKTR	0.634003	0.440894	0.911693	0.01394
CLCN2	1.802236	0.285261	11.38627	0.53113
FOXP1	1.102715	0.282219	4.308642	0.888175
EIF4G1	3.68701	0.846838	16.05272	0.082129
SPCS1	1.04068	0.296349	3.654517	0.950389
NEK4	0.108263	0.013057	0.897695	0.039402
SLC4A3	1.214097	0.167776	8.785725	0.847647
INO80D	0.756986	0.416309	1.37645	0.361441
EEF1B2	0.708497	0.280511	1.789478	0.466015
ADAM23	0.094597	0.00521	1.717644	0.110881
DGUOK	0.083531	0.009919	0.703418	0.022397
LMAN2L	0.62732	0.326818	1.204128	0.161031
RTKN	1.568618	0.397146	6.195605	0.520644
TTL	0.39125	0.134101	1.141499	0.085848
IL1A	1.501885	0.7455	3.025696	0.255073
CCL20	1.048576	0.729391	1.507436	0.797857
PIKFYVE	0.113269	0.003762	3.410237	0.209927
FAHD2A	0.67438	0.239689	1.897416	0.455406
NCL	0.323071	0.14852	0.702765	0.004378
ACTR1B	1.717274	0.768777	3.836003	0.187271
SLC35F5	0.509052	0.188697	1.373277	0.182366
ZAP70	0.636973	0.232483	1.745228	0.380455
ACTR3	1.023082	0.605155	1.729632	0.932122
STEAP3	1.002638	0.216778	4.637382	0.99731
EPB41L5	1.389029	0.23123	8.344073	0.719434
TFCP2L1	3.445158	0.605205	19.61173	0.163312
TP53I3	2.081497	0.877173	4.939308	0.096367
DNAJC27	0.388023	0.205262	0.733512	0.00357
POMC	1.085785	0.75854	1.55421	0.652884
STAM2	1.876822	0.62292	5.654759	0.263222
OTOF	0.421821	0.07144	2.490654	0.340725
GPD2	0.436236	0.077489	2.45587	0.346754
CENPA	0.877044	0.473603	1.624157	0.676449
CYTIP	1.175902	0.694498	1.990999	0.546454
ACVR1	1.65022	1.035373	2.630191	0.035194
TANC1	1.219402	0.636325	2.336764	0.550007
SLC30A3	1.846269	0.178144	19.13462	0.607287
MPV17	0.845922	0.402485	1.777914	0.658826
GTF3C2	0.508795	0.207008	1.250541	0.140835
EIF2B4	0.31264	0.074179	1.317671	0.113168
NRBP1	1.268742	0.466536	3.450336	0.640991
ITGA4	0.574318	0.332992	0.990537	0.046135
PSMD14	0.344016	0.142548	0.830229	0.017603
SNX17	0.919464	0.442375	1.911081	0.822032
ASB3	0.389828	0.130036	1.168642	0.092617
PPM1G	0.315501	0.085021	1.170776	0.084654
REEP6	0.890178	0.383968	2.063755	0.786266
PCSK4	0.564454	0.154147	2.06691	0.387814
APC2	0.814317	0.122663	5.405948	0.831575
IFIH1	1.497388	0.824363	2.71988	0.18493
RPS15	1.032036	0.399474	2.666254	0.948081
GCA	0.989376	0.725906	1.348473	0.946099
INO80B	1.79269	0.757045	4.245106	0.184461

MOGS	0.865421	0.352775	2.123036	0.752241
TTC31	0.624303	0.234248	1.663849	0.346203
NDUFS7	1.552616	0.809265	2.978774	0.185712
PCGF1	5.320615	1.55321	18.2261	0.007794
CLIP4	1.734083	0.949137	3.168187	0.073422
SPTBN1	0.982168	0.324014	2.977197	0.974631
AUP1	0.48712	0.080075	2.963279	0.434944
RTN4	1.549146	0.509374	4.711378	0.440538
HTRA2	1.071031	0.528105	2.17212	0.849143
LOXL3	0.964428	0.487892	1.906409	0.91703
GALNT3	0.981137	0.455556	2.113084	0.961197
POLE4	0.844676	0.480136	1.485991	0.558085
TACR1	0.041025	0.000605	2.783614	0.13776
CCDC88A	0.748331	0.333664	1.678333	0.481753
MRPL19	0.665549	0.31173	1.420959	0.292757
LANCL1	0.636277	0.346953	1.166869	0.143959
WDR75	0.434052	0.178168	1.057438	0.066205
EFEMP1	0.186524	0.006751	5.153361	0.321364
FANCL	0.282152	0.08292	0.96008	0.04285
FN1	3.434423	0.790493	14.9214	0.099706
STAT1	1.031511	0.630087	1.688681	0.901821
GLS	0.582359	0.31217	1.086405	0.089231
PAPOLG	3.69881	0.72269	18.93094	0.116389
PECR	1.036745	0.635501	1.691325	0.885098
UNC50	1.061803	0.502087	2.245482	0.875296
IGFBP2	1.232193	1.012826	1.499072	0.036855
ELMOD3	0.184841	0.040814	0.837122	0.028477
IGFBP5	1.650336	0.782721	3.479669	0.188075
USP34	0.851294	0.461016	1.571966	0.60691
KCNJ13	3.741117	0.452819	30.90847	0.220722
CCT4	0.475984	0.183121	1.237216	0.127707
GGCX	0.820145	0.273632	2.458182	0.723322
EHBP1	0.528622	0.274827	1.016787	0.056122
TXNDC9	1.242769	0.745555	2.071577	0.404463
COQ10B	0.640565	0.39042	1.050979	0.077877
GNLY	0.923962	0.648108	1.317229	0.662038
SF3B1	0.407925	0.15712	1.059081	0.065469
ST3GAL5	1.640049	0.904202	2.974734	0.103422
CHST10	1.043616	0.500151	2.177611	0.909429
PDCL3	0.299662	0.114911	0.781449	0.013731
HSPE1	0.882402	0.343564	2.266341	0.7949
PLCD4	0.095428	0.007769	1.172217	0.066387
ZNF142	0.941308	0.328184	2.699891	0.910422
IL1R2	2.239871	0.90856	5.521947	0.079831
IL1R1	2.602288	1.074671	6.301371	0.034043
IL1RL1	0.47069	0.060228	3.67853	0.472552
IL18R1	0.438603	0.213896	0.899376	0.024486
IL18RAP	0.793223	0.581031	1.082908	0.144708
FHL2	0.757353	0.357125	1.606113	0.468688
UXS1	1.861697	0.290557	11.92851	0.511958
ABCB6	0.925997	0.601949	1.424492	0.726435
STK16	1.904483	0.312037	11.62378	0.485158
HDLBP	0.341542	0.038161	3.056793	0.336694
PPP1R7	2.817397	0.934917	8.490305	0.065711
PASK	1.34326	0.493465	3.656482	0.563552
STK25	1.348737	0.52843	3.442442	0.531462
TPO	0.33686	0.011093	10.2297	0.532116
PROC	3.62268	1.435675	9.141211	0.006416

ID2	1.103432	0.81968	1.485411	0.516372
TAF1B	0.826447	0.313098	2.181472	0.700299
HPCAL1	1.636098	0.700812	3.819594	0.255077
ODC1	0.859433	0.505355	1.461598	0.576081
BIRC6	0.703984	0.245296	2.02039	0.514065
NOL10	0.80434	0.212726	3.041293	0.748316
PLEKHB2	0.625881	0.22578	1.734998	0.367709
GORASP2	0.571995	0.214629	1.524386	0.264002
STRN	2.208656	0.172372	28.30016	0.542576
CEBPZ	1.322152	0.555488	3.14694	0.527922
PRKD3	1.323487	0.285774	6.129373	0.720066
QPCT	1.130626	0.706418	1.809575	0.608912
RAB3GAP1	0.600654	0.275399	1.310047	0.20013
SLC25A12	0.580587	0.180552	1.86695	0.361576
DLX2	0.59037	0.081467	4.278239	0.601998
LCT	2.056258	1.335409	3.166217	0.001063
SDC1	1.445933	0.162529	12.86368	0.740888
PLCL1	0.784836	0.140351	4.388748	0.782648
SLC1A4	0.952379	0.427158	2.123399	0.905063
SOS1	0.661009	0.253989	1.72028	0.396257
KYNU	1.206373	0.90643	1.605567	0.198309
WIPF1	0.662441	0.182383	2.406085	0.531453
COX7A2L	0.99791	0.478035	2.083164	0.995555
PNO1	0.455575	0.184584	1.124411	0.088086
PLEK	0.754269	0.408382	1.393113	0.367662
RND3	4.044135	0.621158	26.3299	0.143795
ATF2	0.359368	0.147897	0.873216	0.023868
THADA	0.208969	0.034507	1.265504	0.088434
AAK1	0.65354	0.130334	3.277073	0.605107
TRAK2	1.000793	0.685975	1.460092	0.996718
TIA1	0.404141	0.20534	0.795413	0.008728
PCYOX1	0.918405	0.349416	2.413933	0.862941
KISS1R	1.174541	0.49947	2.762023	0.712312
EPAS1	1.120725	0.548646	2.289319	0.754472
ARID3A	0.984125	0.497078	1.948391	0.963374
SUMO1	1.159965	0.46181	2.913575	0.752163
GRIN3B	1.062633	0.429605	2.628434	0.895401
NFE2L2	1.649677	0.909146	2.993393	0.099631
MSH6	0.442044	0.245822	0.794896	0.006398
PLEKHA3	0.490437	0.19806	1.214423	0.123552
SPR	1.62143	0.71122	3.696514	0.250357
EPHA4	1.157413	0.37405	3.581356	0.799758
PARD3B	1.379599	0.030909	61.57681	0.868125
FARSB	1.121125	0.129516	9.704733	0.917307
ALMS1	1.852127	0.321216	10.67932	0.490506
BCL9	4.194069	1.943835	9.049231	0.000258
DHCR24	1.331925	0.879887	2.016196	0.17541
DNAJC16	1.199973	0.172777	8.334083	0.853731
MORN1	1.244768	0.194871	7.951158	0.81699
GPX7	0.988914	0.654419	1.494382	0.957795
CACYBP	0.131566	0.02813	0.615345	0.00997
SCP2	0.546905	0.164139	1.822266	0.325731
TPSG1	0.379763	0.073253	1.968787	0.248849
RALGPS2	0.138074	0.016749	1.138228	0.065817
ANGPTL1	6.622957	0.157853	277.8767	0.321374
FAM20B	0.519192	0.260295	1.035594	0.062789
TMEM59	1.167481	0.54373	2.506783	0.691242
LRRC42	0.357383	0.13218	0.966274	0.042604

MRPL37	0.885383	0.397113	1.974008	0.766028
ICMT	1.190173	0.270133	5.243768	0.818012
RPL22	0.592177	0.344562	1.017735	0.057915
CHD5	0.851702	0.168166	4.313574	0.84623
QSOX1	1.678608	0.878884	3.206025	0.116668
STXBP3	0.682569	0.363293	1.282436	0.235284
PHF13	1.494814	0.778784	2.869175	0.226891
ERRFI1	0.719553	0.241455	2.144318	0.554684
PARK7	0.496521	0.247928	0.994374	0.048168
AMPD2	0.114393	0.004673	2.800022	0.183886
MECR	0.200288	0.047125	0.85125	0.029398
KCNC4	1.220739	0.014985	99.44534	0.929206
EDEM3	0.968565	0.270377	3.469665	0.960872
WDR77	0.307121	0.08237	1.14511	0.07872
RAP1A	0.498955	0.117499	2.118787	0.346039
HDAC1	0.573652	0.327959	1.003407	0.05141
CAPZA1	1.399356	0.761797	2.570495	0.278799
S100PBP	0.121469	0.027504	0.536451	0.005407
RNF19B	2.195617	1.285764	3.749313	0.003969
SCAMP3	0.974276	0.308597	3.075902	0.964563
TRIM62	14.59456	2.583553	82.44502	0.00241
ASH1L	1.117228	0.186937	6.677119	0.903277
DLGAP3	14.08053	2.199814	90.12636	0.005233
SFPQ	0.905603	0.426434	1.923195	0.796377
RHOU	1.171181	0.876603	1.564749	0.285077
GON4L	1.051444	0.18404	6.007014	0.95501
ARHGEF2	0.977023	0.41507	2.299789	0.957557
MEF2D	1.167982	0.621018	2.196686	0.62995
DOCK7	0.771381	0.203407	2.925306	0.702707
SRM	0.850311	0.500166	1.445578	0.549239
DLEU2L	0.771774	0.296837	2.006609	0.595143
FBXO6	1.251915	0.780215	2.008793	0.351722
MAD2L2	2.114746	0.991281	4.511486	0.052707
DNAJC6	5.631728	0.12277	258.3389	0.37591
LEPR	1.176213	0.391948	3.52975	0.772224
IVNS1ABP	0.44369	0.212998	0.924236	0.029977
KIAA2013	1.479764	0.690917	3.169267	0.313225
MFN2	1.540959	0.695303	3.415137	0.2869
PRG4	0.490018	0.010591	22.67259	0.715406
MIIP	1.129159	0.691353	1.844211	0.627455
SMG7	0.704693	0.207929	2.388279	0.574108
NCF2	1.152499	0.924626	1.43653	0.206677
PDC	4.925127	0.046064	526.5924	0.503598
SLC35D1	0.612632	0.05509	6.812819	0.690122
PLA2G4A	1.465424	0.707821	3.033911	0.303365
GADD45A	1.063784	0.704559	1.606164	0.768653
PRDM2	2.807743	0.193782	40.68198	0.449125
RGS2	1.20013	0.898838	1.602417	0.216138
UCHL5	0.193574	0.071986	0.520533	0.001139
BCAS2	1.353484	0.589159	3.109383	0.475686
CTH	0.489549	0.058536	4.094219	0.509796
AGMAT	1.208011	0.429124	3.400632	0.720444
OLFML3	0.559146	0.063532	4.921063	0.600348
PLEKHM2	1.575291	0.674979	3.676472	0.293286
CRYZ	0.787726	0.460471	1.34756	0.383737
PHTF1	0.315591	0.143685	0.693165	0.004067
ZBTB17	1.624509	0.779639	3.384937	0.195185
CD58	0.684421	0.371322	1.261527	0.224238

TFAP2E	6.151028	0.931574	40.6142	0.059246
CD2	1.117369	0.786794	1.586838	0.53519
TTF2	0.424848	0.231895	0.778352	0.005586
NR5A2	2.221214	0.898136	5.493366	0.084092
KIF21B	0.809377	0.171701	3.815308	0.789208
TMEM9	1.650133	1.041304	2.614931	0.032985
MAP7D1	3.376036	1.530591	7.446547	0.002573
WARS2	0.855699	0.331726	2.207305	0.74721
OSCP1	0.9908	0.264281	3.714544	0.989063
MRPS15	0.388594	0.167355	0.902304	0.027868
EXOC8	1.025578	0.484672	2.170147	0.947344
GNPAT	0.869666	0.449616	1.682141	0.678229
TSNAX	0.712737	0.390237	1.301759	0.270514
RRAGC	0.45847	0.187098	1.123448	0.088118
NID1	1.606261	0.597709	4.316604	0.347426
LGALS8	0.312188	0.070528	1.381877	0.125075
NT5C1A	1.514975	0.164698	13.93552	0.713694
HPCAL4	2.763026	0.640866	11.9125	0.172826
MTR	0.091022	0.021784	0.380329	0.00102
BMP8B	1.222682	0.747293	2.000491	0.423512
SIPA1L2	1.224891	0.832221	1.802836	0.303644
RLF	0.630779	0.317841	1.25183	0.187609
KMO	3.72076	1.269066	10.90885	0.016659
ZNF684	0.617863	0.231781	1.647051	0.335802
KCNQ4	0.03718	0.000458	3.021455	0.142332
RIMS3	1.402211	0.781139	2.517088	0.257428
AKT3	5.726183	0.207394	158.1007	0.302656
ETV3	0.594086	0.247648	1.42516	0.243454
ACADM	0.410101	0.204984	0.820469	0.011761
SLAMF1	1.497428	0.822193	2.727209	0.18686
CD48	0.901025	0.727939	1.115265	0.338259
PADI2	0.870812	0.168667	4.495908	0.868813
SDHB	0.846995	0.254825	2.81526	0.78641
MFAP2	4.522852	0.419877	48.71952	0.213352
RPF1	0.67198	0.29287	1.541838	0.348166
KDM5B	0.719284	0.485777	1.065033	0.099898
UAP1	0.984965	0.596167	1.627323	0.952843
CTBS	1.329419	0.659177	2.681156	0.426292
KLHL12	1.425513	0.561667	3.61796	0.455628
SSX2IP	0.743493	0.183185	3.017617	0.678368
ZNHIT6	0.443371	0.229055	0.858214	0.015791
PLA2G2D	0.552559	0.160167	1.906272	0.347799
RBBP5	0.555263	0.266817	1.155539	0.115639
GBP3	0.891622	0.367617	2.16255	0.799679
GBP1	1.110439	0.772875	1.595439	0.571013
KIF17	0.711072	0.393151	1.286081	0.259405
GPR89A	0.339054	0.141086	0.814809	0.015616
CD160	0.691607	0.372083	1.28552	0.243675
ECE1	2.805427	0.824544	9.545181	0.098706
HMGCL	1.224756	0.596118	2.516327	0.581048
GALE	0.007278	0.000297	0.178621	0.002571
ID3	1.382294	0.871126	2.193412	0.169352
CR2	2.049532	0.189128	22.21027	0.555034
CD46	1.011942	0.528247	1.938538	0.971448
PRPF3	0.727221	0.380172	1.391084	0.335789
APH1A	2.92949	1.394623	6.153571	0.004535
SLC2A1	1.035798	0.777219	1.380405	0.810313
EBNA1BP2	0.306647	0.162909	0.577211	0.000249

CDC20	0.806304	0.598302	1.08662	0.157284
MPL	0.859437	0.682697	1.081932	0.197203
ARTN	1.020678	0.314242	3.315226	0.972836
IPO13	2.720521	1.218835	6.072386	0.014565
ATP6V0B	1.887427	0.870353	4.093029	0.107753
B4GALT2	2.538715	0.134065	48.07437	0.534689
ERI3	0.699537	0.223432	2.190159	0.539446
PTCH2	1.259343	0.100921	15.71469	0.857891
AKR1A1	1.020744	0.351197	2.966766	0.969913
PRDX1	1.352377	0.729168	2.508233	0.338169
PIK3R3	0.565307	0.101143	3.159626	0.515921
BLZF1	0.883852	0.475827	1.641759	0.695955
SLC19A2	1.833344	0.472282	7.11683	0.381079
FAAH	1.257036	0.817054	1.93395	0.298
NSUN4	0.732059	0.276047	1.941373	0.530797
TMED5	0.985086	0.549489	1.765996	0.959762
DR1	0.711258	0.37952	1.332969	0.287712
CNN3	1.126237	0.477869	2.654304	0.785785
F3	0.994379	0.528786	1.869921	0.986041
ABCD3	0.615215	0.205767	1.839407	0.384664
VAMP4	0.82199	0.226399	2.984402	0.765728
DPH5	0.698505	0.418737	1.165192	0.169329
FASLG	0.175283	0.028131	1.092184	0.062112
PTBP2	0.549115	0.312758	0.964091	0.036861
TNFSF4	1.054896	0.626603	1.775936	0.840627
PRDX6	0.279178	0.119446	0.652519	0.003224
DARS2	0.50863	0.235771	1.097271	0.084828
IRF6	0.071845	0.004151	1.243606	0.07028
RCAN3	1.807065	0.701862	4.652598	0.220094
SYF2	0.981552	0.292995	3.288263	0.975919
SLC35A3	0.288607	0.129345	0.643969	0.002407
RCOR3	0.442678	0.230946	0.848527	0.014099
STMN1	0.223868	0.087459	0.573035	0.001802
MAN1C1	1.316088	0.450931	3.841138	0.61525
NEK2	0.637789	0.31434	1.294063	0.212816
RPS6KA1	1.208464	0.225615	6.472909	0.82499
DHDDS	1.207676	0.394325	3.698677	0.741076
NENF	0.561466	0.280155	1.125251	0.103678
NSL1	2.069839	0.121107	35.37565	0.615454
ARID1A	0.475708	0.132496	1.707959	0.254628
CENPF	0.735969	0.442163	1.225002	0.238282
RPA2	0.314774	0.127562	0.776739	0.012135
PPP1R8	0.777881	0.084197	7.186666	0.824765
STX12	1.467792	0.667431	3.227921	0.339874
SLC5A9	2.21761	0.395613	12.43081	0.365164
OSBPL9	0.385869	0.131436	1.132828	0.083097
TXNDC12	0.798244	0.379587	1.678648	0.552402
RCN2	0.200935	0.057485	0.70236	0.011961
MUC5B	2.023348	0.141594	28.91313	0.603501
CTSD	1.574391	1.104424	2.244344	0.012106
STAG1	0.390991	0.1383	1.105373	0.076556
STK11	1.841029	0.997312	3.398524	0.051015
MMP8	0.972917	0.781968	1.210493	0.805444
APOA1	0.965108	0.432877	2.151727	0.930816
KPTN	1.213486	0.396959	3.709569	0.734311
RPS25	1.22474	0.336607	4.456194	0.758355
KIF14	0.405305	0.183934	0.893104	0.025064
DDX59	0.89783	0.199153	4.047636	0.888447

ATF6	0.723506	0.355086	1.474184	0.372805
CRYGD	0.854902	0.629253	1.161469	0.31604
MREG	0.061587	0.005241	0.723654	0.026606
FASTKD2	0.46537	0.150995	1.434281	0.182878
NRP2	0.284418	0.0083	9.746765	0.485641
CREB1	0.584684	0.107913	3.167881	0.533608
KLF7	0.738555	0.084884	6.425954	0.783654
B4GALT6	0.566555	0.301328	1.065233	0.077767
CA14	0.063315	0.004455	0.899784	0.041555
SPCS2	1.193664	0.236169	6.0331	0.830433
USP35	2.931905	0.548085	15.68381	0.208693
CASP8AP2	0.735868	0.077325	7.002949	0.789616
HMGN3	0.825474	0.349646	1.948846	0.661674
ANKRD13(	0.75278	0.220769	2.566837	0.650009
PHF3	0.773699	0.397845	1.504629	0.44961
PLAGL1	0.505444	0.275882	0.926027	0.027192
FBXO30	1.437555	0.736783	2.804848	0.287212
TNFAIP3	1.008047	0.794978	1.278222	0.947258
AKAP7	1.107219	0.443895	2.761765	0.827115
RAB32	0.995368	0.651092	1.521687	0.982897
MYB	0.552167	0.375284	0.812421	0.002576
ALDH8A1	0.293741	0.010453	8.254626	0.47166
SGK1	1.271362	0.994273	1.625671	0.055598
RNF146	0.915874	0.311044	2.69681	0.873287
ARG1	0.912245	0.718858	1.157656	0.449891
PMFBP1	0.080115	0.005816	1.103501	0.059246
FBXL5	1.420949	0.492852	4.096754	0.515498
MED28	0.724524	0.283331	1.852725	0.501149
SLC16A7	0.546696	0.060458	4.943565	0.590922
ZNF430	0.545899	0.160702	1.854399	0.331959
VAMP8	0.373726	0.18577	0.751848	0.005785
DCLRE1B	0.590953	0.161004	2.169041	0.427851
FOXO3	1.426383	0.534096	3.809365	0.478578
ARMC2	1.589065	0.06661	37.90916	0.774747
GHRH	0.354686	0.043868	2.86773	0.331045
RPN2	0.649636	0.340541	1.23928	0.190551
TGIF2	1.234444	0.70727	2.154555	0.458586
OLFM3	0.770939	0.048409	12.27763	0.853849
PKD2	0.539137	0.25742	1.129159	0.101441
ABCG2	0.389036	0.037785	4.00556	0.427458
SPP1	0.84304	0.36984	1.921684	0.68463
STBD1	0.804378	0.280446	2.307121	0.685537
CCNI	0.48812	0.20294	1.174045	0.109236
RARRES1	1.041281	0.071685	15.12553	0.976363
MFSD1	0.454526	0.247116	0.836022	0.011213
RAB3GAP2	0.826401	0.355863	1.919107	0.65736
PPL	3.830587	1.135816	12.91882	0.030366
UBN1	0.525819	0.16339	1.692182	0.281081
KLF12	0.884519	0.298971	2.616886	0.824524
UCHL3	0.325915	0.119072	0.892066	0.029088
HS1BP3	2.250887	1.336761	3.790127	0.002275
WDR35	0.274384	0.058315	1.291046	0.101699
CCND2	0.922678	0.644764	1.320381	0.659869
ELL2	1.214458	0.732442	2.013684	0.451392
CYP20A1	1.060099	0.272202	4.128585	0.932949
NDUFB3	1.059415	0.571854	1.962669	0.854433
GTF3C3	0.536436	0.277632	1.036495	0.063839
SATB2	1.517962	0.623803	3.693813	0.357643

UBE2B	0.422848	0.101254	1.76585	0.237899
TRPM6	0.273418	0.032191	2.322332	0.23482
KLF9	1.172199	0.937968	1.464924	0.162439
TJP2	1.307487	0.424552	4.02665	0.640382
ITGB1BP1	0.801981	0.418326	1.537492	0.506337
CPSF3	0.511123	0.254459	1.026674	0.059296
PIGZ	0.662063	0.269921	1.62391	0.367664
SENP5	0.230477	0.059394	0.894357	0.033893
CCDC92	0.992973	0.550202	1.792061	0.981324
HEATR1	0.454399	0.253086	0.815845	0.008252
RAD23B	0.489321	0.265338	0.90238	0.022084
FKBP15	1.583587	0.896115	2.798468	0.11356
CTNNAL1	0.725362	0.408648	1.28754	0.272766
SET	0.430494	0.241516	0.767341	0.004264
GLE1	0.679364	0.290399	1.589313	0.37264
RAB14	0.615932	0.099809	3.801003	0.601726
TRIM32	3.838311	1.837278	8.018729	0.000346
FBXW2	0.170719	0.037508	0.777026	0.02224
PHF19	0.854417	0.303117	2.408406	0.766031
NEK6	2.426014	1.297428	4.536316	0.005514
BSPRY	1.429006	0.862066	2.368795	0.166244
PPP6C	1.377039	0.449322	4.220221	0.575547
NDUFA8	0.624371	0.259008	1.505125	0.294093
HDHD3	1.227519	0.680385	2.214633	0.495946
RBM18	0.450526	0.124849	1.625759	0.223319
SLC46A2	1.447808	1.012789	2.069678	0.04239
HSDL2	0.636962	0.327818	1.237642	0.183233
MAPKAP1	0.244491	0.049679	1.203234	0.0832
NR4A3	0.679188	0.109125	4.227234	0.678367
INVS	0.566803	0.121493	2.644314	0.469989
GALNT12	2.147004	0.860512	5.356842	0.101437
DENND1A	1.596391	0.694695	3.668465	0.27053
ALG2	0.479291	0.040539	5.666637	0.559508
CSF3R	1.186848	0.665239	2.117448	0.561942
KDSR	0.919242	0.340593	2.480985	0.867979
VPS4B	1.088911	0.546853	2.168275	0.808479
ONECUT2	2.852669	0.004051	2008.884	0.754029
ZBTB45	1.802158	0.932757	3.481907	0.079636
YLPM1	0.83402	0.138709	5.014749	0.842809
PROX2	0.085837	0.006138	1.200385	0.068112
VSX2	0.074125	0.005763	0.953406	0.045871
FCF1	0.245022	0.078323	0.76651	0.015652
PGF	1.070878	0.379184	3.024335	0.897139
IFI27L2	1.582337	1.044403	2.397342	0.030394
NEK9	0.037638	0.002187	0.647611	0.023868
ACYP1	0.245953	0.098212	0.615945	0.002748
NPC2	0.864247	0.484846	1.540538	0.62081
DNAL1	0.111936	0.009439	1.327464	0.082658
ACOT2	3.304335	0.95701	11.40911	0.058696
LTBP2	0.602305	0.114511	3.167996	0.549458
MLH3	0.410862	0.054658	3.088426	0.387436
TTLL5	0.363996	0.158287	0.837041	0.017377
FLVCR2	1.597537	1.119947	2.27879	0.009736
ABCD4	0.695678	0.021373	22.64394	0.838191
DLST	0.257353	0.091363	0.724913	0.010205
TGFB3	1.378134	0.322889	5.882065	0.664885
RBM25	0.56054	0.33809	0.929354	0.024833
ALDH6A1	0.684225	0.34929	1.340329	0.26867

GPR68	1.472566	0.588724	3.683308	0.408039
EIF2B2	0.814509	0.344201	1.927434	0.640608
COQ6	0.623783	0.086145	4.516858	0.640335
ZNF410	0.808563	0.449601	1.454121	0.477928
RHOQ	1.334804	0.457773	3.892107	0.596877
SUPT7L	0.454375	0.088115	2.343046	0.345901
KLHL29	1.463537	0.348995	6.137459	0.60257
DNMT3A	0.531403	0.061584	4.585468	0.565304
TMEM214	1.299758	0.566308	2.98313	0.536235
ATAD2B	1.124443	0.330799	3.822173	0.850969
FKBP1B	0.428241	0.107236	1.71016	0.229972
ATL2	0.611775	0.236534	1.582306	0.310819
YPEL5	0.623267	0.339669	1.143648	0.126868
FAM98A	1.106741	0.597368	2.050454	0.747181
YIPF4	1.977741	0.614147	6.368926	0.253075
AFTPH	1.353398	0.559326	3.274808	0.502078
CNRIP1	0.477925	0.281906	0.810245	0.006121
BCL11A	1.030319	0.639202	1.660754	0.902405
CRIPT	0.403778	0.195814	0.83261	0.014046
EPCAM	0.840063	0.583078	1.210311	0.349564
SLC17A5	2.807449	0.058349	135.0795	0.601453
OGFRL1	1.132175	0.706728	1.813739	0.605637
IDE	0.356528	0.156542	0.812001	0.014055
ELOVL3	1.417202	0.376188	5.338989	0.606375
IFIT3	1.384106	0.629485	3.043362	0.418751
NKX2-3	1.298542	1.030029	1.637052	0.027085
IFIT2	1.870294	1.188451	2.943325	0.006805
GPAM	0.608076	0.221392	1.670148	0.33455
CUTC	0.297958	0.122991	0.721831	0.007319
CNNM1	0.910191	0.347512	2.383937	0.848095
MXI1	1.513677	0.507595	4.51387	0.457107
SMNDC1	0.684962	0.321885	1.457579	0.326061
HELLS	0.132678	0.033372	0.52749	0.004127
TCTN3	0.782486	0.266338	2.298903	0.655544
AVPI1	1.383551	1.0295	1.859361	0.031339
KCNIP2	0.272233	0.006113	12.12314	0.501743
GOT1	0.435697	0.192166	0.987852	0.046676
SFRP5	2.813941	0.599307	13.21235	0.189814
GNA13	1.063679	0.562003	2.013179	0.84958
HOXB8	1.101116	0.963632	1.258216	0.156907
HOXB5	1.196811	1.010598	1.417335	0.03733
HOXB3	1.345791	1.002792	1.80611	0.047868
DUSP1	1.422273	0.950238	2.128793	0.086914
PANK3	0.514979	0.102663	2.58323	0.419929
TEK	1.471785	0.423787	5.111416	0.542911
RCL1	0.482621	0.232591	1.001429	0.05045
INSL6	3.203159	0.097858	104.8477	0.513061
MLANA	2.276882	0.170421	30.41988	0.533874
CD274	0.146245	0.012618	1.694944	0.12408
NUP43	0.374928	0.149689	0.939088	0.036249
MTHFD1L	0.447604	0.222788	0.899285	0.023935
PCMT1	0.365825	0.188141	0.711318	0.003037
PLEKHG1	1.243411	0.050953	30.34325	0.893674
MYCT1	2.174987	0.239765	19.73003	0.489794
WDR55	0.994376	0.309488	3.194895	0.992443
ARAP3	1.285232	0.914917	1.805433	0.147854
SLC25A2	0.460892	0.029398	7.225709	0.581213
TNN	8.123225	1.683166	39.20397	0.009099

MRPS14	0.5076	0.147977	1.7412	0.280963
CENPL	0.372209	0.115125	1.20339	0.098794
ACAT2	0.454524	0.137387	1.503726	0.196465
TCP1	0.289885	0.13113	0.64084	0.002218
SNX19	0.591851	0.237028	1.477832	0.261266
KCNJ5	0.831466	0.021435	32.25282	0.921229
ARR3	9.615883	0.995087	92.92169	0.050499
PDZD11	97.83426	5.577366	1716.14	0.001713
SLC10A7	0.236531	0.033282	1.680996	0.149622
NUDCD1	0.552002	0.17505	1.740688	0.31056
ENY2	0.521987	0.187912	1.449988	0.212334
MASTL	0.466927	0.153815	1.417422	0.178871
KIAA1217	0.49629	0.052906	4.655494	0.539624
PLXDC2	1.346559	0.851615	2.129156	0.203067
EPC1	0.629871	0.301671	1.315136	0.218457
IQSEC3	1.568527	0.330394	7.446503	0.571111
CCDC77	0.432899	0.174416	1.074454	0.071054
TAF12	1.961712	0.738499	5.210996	0.176435
MTRF1	0.354817	0.1508	0.834852	0.017624
DNAJC15	0.995321	0.491116	2.01717	0.989618
UFM1	0.659165	0.345573	1.257331	0.205886
WBP4	0.607899	0.294813	1.253475	0.17763
ELF1	0.793993	0.490634	1.284919	0.34761
SMAD9	1.963228	0.204793	18.82034	0.558587
HSPH1	0.788141	0.495389	1.253897	0.31493
KBTBD7	0.962315	0.428063	2.16335	0.925951
ALG5	0.5531	0.222174	1.376933	0.203154
EXOSC8	0.215191	0.095084	0.487012	0.000227
ETF1	0.350395	0.157351	0.78027	0.010247
TGFBI	1.228082	0.990841	1.522125	0.060662
FAM53C	0.747953	0.455923	1.227036	0.250199
SIL1	0.969309	0.422095	2.225942	0.941416
PAIP2	1.756491	0.357777	8.623423	0.487755
MYOT	0.576099	0.020402	16.26759	0.746278
KDM3B	0.799076	0.350384	1.822351	0.593867
EGR1	1.135298	0.901515	1.429705	0.280742
SERP1	1.090482	0.558739	2.128278	0.799583
PLS1	1.248482	0.557848	2.794139	0.589241
ZFP30	1.725247	0.520372	5.719898	0.372495
NR2C1	0.226887	0.084623	0.608318	0.003201
UTP20	0.351687	0.00732	16.89677	0.596836
TMPO	0.875665	0.234038	3.276342	0.843658
ARL1	0.727728	0.334513	1.583161	0.422868
GLT8D2	1.157894	0.160086	8.374987	0.884539
SOCS2	1.267929	1.029519	1.561549	0.025502
NFYB	1.266159	0.413846	3.873801	0.679154
APAF1	1.341911	0.592292	3.040268	0.480939
DUSP4	2.6674	0.338681	21.00807	0.35147
CLU	7.383272	0.324037	168.2298	0.210046
TNFRSF10	1.081451	0.439361	2.661904	0.864707
SORBS3	4.227137	1.665899	10.72615	0.002412
PTK2B	1.692827	0.467084	6.135224	0.422988
CHRNA2	5.108641	0.565397	46.15905	0.14644
PPP3CC	0.973458	0.507771	1.866235	0.935432
PDLIM2	0.245108	0.01339	4.486739	0.343163
EPHX2	1.139828	0.558965	2.324311	0.718849
RNF170	0.666809	0.35303	1.259478	0.211678
NPPB	2.46436	0.135672	44.76291	0.542069

UBIAD1	0.885458	0.354676	2.210573	0.794396
TARDBP	0.350386	0.162383	0.756057	0.007526
TNFRSF8	2.628785	0.901975	7.661534	0.076572
ZNF706	0.752671	0.25867	2.190099	0.602097
LYPLA1	0.75511	0.326489	1.746436	0.511439
CRISPLD1	0.573197	0.124727	2.634194	0.474479
COPS5	0.496003	0.222944	1.103502	0.085693
RDH10	0.793007	0.39104	1.608171	0.520273
EPX	0.867973	0.437077	1.72367	0.68583
AKAP1	0.737869	0.141852	3.838168	0.717862
COIL	0.716965	0.290047	1.772258	0.471152
TRIM25	1.317315	0.656707	2.642457	0.437772
SCPEP1	1.051048	0.844683	1.30783	0.655283
SPOP	0.505536	0.143677	1.77875	0.287905
SLC35B1	0.243915	0.071865	0.827861	0.023639
TEX14	0.374717	0.013998	10.03107	0.55838
FAM117A	1.432546	0.847601	2.421173	0.17945
NCAPH	0.176826	0.021133	1.479558	0.109925
MND1	0.701789	0.378124	1.302502	0.261717
TRIM6	0.531807	0.247426	1.143044	0.105769
ADCY7	1.270932	0.728171	2.218256	0.39885
TSHZ3	1.406224	0.829564	2.383742	0.2055
ECHDC2	0.954953	0.770797	1.183107	0.673255
TAS2R10	0.21903	0.030509	1.572466	0.13107
PRB2	0.172343	0.002825	10.5143	0.401877
PYROXD1	0.750685	0.399177	1.411725	0.373509
PSPC1	0.202487	0.048609	0.843482	0.028251
ZNF549	1.171597	0.443996	3.091556	0.749049
A1BG	0.002346	8.77E-05	0.062773	0.000305
ZSCAN18	0.94328	0.616778	1.442623	0.78764
ZNF211	0.564249	0.276971	1.149497	0.114977
PDZRN3	4.518959	0.079828	255.8137	0.463911
LHX4	0.037457	0.002654	0.528573	0.015013
RNF2	0.383322	0.075867	1.93674	0.245973
SEC22A	0.44181	0.176678	1.10481	0.080669
CSTA	1.18	0.824322	1.689146	0.365804
DPPA4	1.407768	0.997323	1.987132	0.051809
POPDC2	0.533539	0.054264	5.24593	0.590094
B4GALT4	0.661794	0.17565	2.493438	0.541896
CD80	7.993582	0.980898	65.14171	0.052144
KIF18A	0.172156	0.020219	1.465797	0.107393
MAPK8IP1	0.414274	0.042522	4.036129	0.448036
CRY2	2.532192	1.114156	5.755024	0.026553
PEX16	3.914264	1.519763	10.08149	0.004698
DEPDC7	0.62658	0.170813	2.298427	0.480833
CAT	0.700434	0.433999	1.130437	0.144858
PILRB	0.243469	0.083721	0.708033	0.00949
ZMYM2	0.357428	0.114265	1.118061	0.077032
GJB6	0.931993	0.48232	1.800899	0.834011
TBC1D15	0.922125	0.493838	1.721852	0.799142
HCRTR1	1.188501	0.119989	11.77223	0.882653
ZCCHC17	0.608801	0.23265	1.593122	0.311958
FABP3	0.256162	0.017977	3.650095	0.315009
KHDRBS1	0.519601	0.237498	1.136787	0.101212
TMEM39B	1.562816	0.69323	3.523209	0.281685
CCRL2	2.793568	1.472316	5.300506	0.001668
CCR2	2.023388	1.070719	3.823693	0.029977
POLR3GL	0.731987	0.333015	1.608955	0.437499

TNFSF10	1.079563	0.827699	1.408066	0.572206
ZNF639	0.670611	0.10132	4.438586	0.678597
PIK3CA	0.214417	0.030853	1.490128	0.119536
PDS5A	0.698622	0.405846	1.202604	0.195592
TMEM156	0.639986	0.273886	1.495449	0.302705
LIAS	0.6008	0.21383	1.688072	0.333738
ZSCAN20	0.963568	0.070406	13.18735	0.97782
CLCC1	0.259111	0.062459	1.074919	0.062823
GPSM2	0.521238	0.323399	0.840104	0.007464
GTDC1	0.089005	0.007262	1.090807	0.058493
CXCR4	1.174687	0.787525	1.752185	0.430015
ZRANB3	0.085764	0.004167	1.765337	0.111461
ACVR2A	6.236746	0.592459	65.65345	0.127483
POLK	1.763182	0.240343	12.93491	0.577
FLT3	0.991748	0.723856	1.358783	0.958864
RPL21	0.797551	0.452992	1.40419	0.433167
MTIF3	0.86045	0.39375	1.880315	0.706301
GTF3A	0.619688	0.321981	1.192661	0.15199
RASL11A	0.630368	0.136126	2.919097	0.555138
UBL3	0.905187	0.473686	1.729762	0.763047
FYTTD1	0.445715	0.1084	1.832666	0.262626
XPNPEP2	1.0445	0.191884	5.685631	0.959834
SASH3	1.433062	0.822587	2.496595	0.203939
OCRL	0.90082	0.358794	2.261681	0.824015
MRPS2	1.35669	0.628185	2.930042	0.437452
FMOD	0.572845	0.042568	7.708815	0.674434
LAX1	0.745295	0.464985	1.194587	0.221971
KIAA1191	0.224735	0.062936	0.802494	0.021517
COPA	0.566559	0.228997	1.401716	0.218954
CD244	0.901483	0.54851	1.481601	0.682438
LY9	0.455828	0.198679	1.045805	0.063702
HS3ST2	0.006622	0.00024	0.182354	0.003017
RBBP6	0.257733	0.047815	1.389237	0.114688
ZC3H7A	0.472239	0.196279	1.136189	0.093948
SERAC1	0.375848	0.060969	2.316945	0.291649
ANXA11	1.164614	0.432487	3.136108	0.763022
ZNF205	2.286622	0.605318	8.637847	0.222589
RPL5	0.376479	0.179712	0.788688	0.009622
ODF2L	0.145142	0.02939	0.716773	0.017854
SPATA1	11.02094	0.494314	245.7166	0.129742
LRRC39	0.691914	0.05107	9.374318	0.781808
RWDD3	0.948414	0.376645	2.388157	0.910499
ZNF644	1.280352	0.295104	5.554997	0.741359
CCDC18	0.057841	0.009591	0.348805	0.001878
RPAP2	0.733944	0.346087	1.556467	0.419968
BBS9	0.376297	0.063684	2.22348	0.28088
PMS2	0.145018	0.029213	0.719904	0.018178
ZMIZ2	0.288713	0.076461	1.090169	0.066861
OCM	7.923097	0.612584	102.4765	0.113025
EEPD1	0.764197	0.27868	2.095586	0.601313
KIAA0087	0.52229	0.055394	4.924447	0.570455
KLHL7	0.675636	0.163188	2.797295	0.588568
HERPUD2	1.514868	0.627486	3.657175	0.355694
CBX3	2.830513	0.400014	20.02879	0.297325
HNRNPA2	0.619132	0.415498	0.922568	0.018472
WIPF3	3.533677	0.29132	42.86314	0.321504
NPY	3.274735	0.443001	24.20739	0.245135
FAM126A	1.187824	0.045297	31.1486	0.917747

HOXA7	1.449166	0.690879	3.039724	0.326313
INHBA	4.602386	0.658704	32.157	0.123788
FKBP9	0.429516	0.023669	7.7942	0.567689
ARL4A	1.19164	0.7112	1.996632	0.505538
POLM	2.224465	1.029213	4.807797	0.042034
RAMP3	3.427611	0.616494	19.05698	0.159323
TWIST1	1.00283	0.71825	1.400164	0.98676
SMU1	0.385096	0.130539	1.136054	0.083834
GLIPR2	1.58557	1.070927	2.347529	0.021323
CLTA	6.056466	1.689056	21.71673	0.005701
RECK	1.804204	0.913684	3.562667	0.089145
SPINK4	0.556123	0.144865	2.134899	0.392589
ACO1	1.062697	0.54492	2.072457	0.858377
DCAF10	0.384569	0.122608	1.20623	0.10132
KIAA1549	6.44017	0.989373	41.92129	0.051321
TRIM24	0.324141	0.128686	0.816462	0.01684
CALD1	2.328408	0.269788	20.09536	0.442141
NUDT10	2.321732	0.720448	7.482065	0.158305
NEUROG3	13.70964	2.808628	66.92028	0.001209
PLAU	1.511047	1.015549	2.248306	0.041744
SRGN	0.971588	0.590557	1.598462	0.909657
BICC1	1.958343	0.146137	26.24327	0.611758
CISD1	1.11385	0.574602	2.159165	0.749518
EGR2	1.390866	1.065391	1.815772	0.01528
ECD	0.636879	0.208058	1.949529	0.429285
P4HA1	0.692247	0.273783	1.750313	0.437065
SLC25A16	0.43425	0.05481	3.440486	0.429592
ZWINT	0.296648	0.08961	0.982036	0.04663
VPS26A	1.059852	0.544416	2.063286	0.864205
RBM19	30.54624	0.509278	1832.148	0.101645
CIT	0.899368	0.194954	4.148991	0.891849
IFT81	0.178014	0.010598	2.990121	0.230519
ACADS	1.587518	0.871348	2.892315	0.131038
HVCN1	1.432735	0.983073	2.088074	0.061327
NME2P1	6.209296	0.276367	139.5078	0.25013
DDX54	0.542215	0.241053	1.219639	0.138899
MED13L	1.303361	0.65761	2.583216	0.447799
CDKN2C	1.176355	0.52571	2.632275	0.692664
RNF11	0.92631	0.301229	2.848495	0.893754
RASSF8	0.018416	0.00138	0.245781	0.002516
ITPR2	0.573648	0.311346	1.056935	0.074689
CCDC91	0.709002	0.350374	1.434707	0.338942
NECAB1	2.746362	0.523142	14.41772	0.232421
WWP1	0.704526	0.185367	2.677697	0.607173
ACOT9	0.943653	0.418613	2.127217	0.888779
PRDX4	0.75726	0.416311	1.377439	0.36235
PKN1	2.740659	0.885829	8.479297	0.08019
GIPC1	2.872299	1.478863	5.578679	0.001838
EBPL	0.735943	0.473609	1.143585	0.172768
ATP7B	0.916464	0.427961	1.962579	0.82235
ZC3H13	0.090386	0.013498	0.605239	0.01323
GUCY1B2	0.443459	0.087004	2.260307	0.327794
NLN	0.495482	0.118838	2.065858	0.335058
CENPK	0.460566	0.241624	0.877897	0.018491
OPTN	1.148468	0.768819	1.71559	0.499014
ATF1	0.996755	0.457977	2.169367	0.993465
TSFM	1.16565	0.304853	4.457037	0.822762
ARHGAP9	0.785212	0.428298	1.439552	0.434286

NCKAP1L	0.51847	0.266848	1.007357	0.052582
MMP19	10.03198	0.871037	115.5413	0.064424
PFDN5	3.057697	1.076674	8.683694	0.035845
SPATS2	1.692972	0.764161	3.750719	0.194554
ORMDL2	2.668963	0.853726	8.343856	0.091405
NR4A1	1.525737	0.404424	5.756018	0.532868
PDE1B	1.58551	0.541055	4.646185	0.400783
CDK2	0.30635	0.127763	0.734569	0.008019
LRP1	1.416865	0.96364	2.083253	0.076453
NFE2	0.845692	0.49651	1.440444	0.537351
IKZF4	1.463769	0.40287	5.318393	0.562707
SMUG1	0.936283	0.362458	2.418562	0.891843
TUBA1B	0.281815	0.096201	0.825559	0.020914
KBTBD4	0.521496	0.09211	2.952526	0.461721
SARDH	6.690544	1.178145	37.9948	0.031955
DBH	0.925443	0.071684	11.94749	0.952659
ATPAF1	0.350354	0.122938	0.998458	0.049663
STIL	0.539895	0.26286	1.108904	0.093255
HJURP	0.738498	0.440322	1.238593	0.250577
COL10A1	0.826328	0.085847	7.953939	0.868854
AMD1	0.371019	0.199828	0.688868	0.001687
USP45	0.102986	0.01561	0.679464	0.018205
MORF4L2	0.922195	0.547829	1.55239	0.760496
RAB9B	0.976846	0.325508	2.931508	0.966674
RAB9A	0.968446	0.410045	2.287282	0.941711
METTL8	0.302765	0.046583	1.967816	0.210888
TTC21B	8.014517	0.202565	317.095	0.26739
NMI	0.856188	0.441412	1.660711	0.645994
TNFAIP6	0.914797	0.420201	1.991558	0.822482
BAZ2B	0.765694	0.442415	1.325195	0.340123
SLC36A1	1.096028	0.646313	1.858662	0.733658
LPGAT1	0.387955	0.086793	1.734112	0.215199
BATF3	1.337029	0.86247	2.072707	0.194114
G0S2	1.13221	0.914654	1.401514	0.254058
KCNJ2	1.95155	1.106902	3.440726	0.020832
RAP2C	0.485372	0.242379	0.971972	0.04133
EXOSC9	0.163407	0.075336	0.354436	4.53E-06
PLA2G12A	0.467908	0.096578	2.266955	0.345493
B9D2	0.540227	0.248818	1.172925	0.119535
PFKFB2	1.322621	0.176553	9.908205	0.785509
RAB38	1.497376	1.018254	2.201941	0.040179
MXD4	1.633781	0.928391	2.875124	0.088698
CKS2	0.884524	0.567453	1.378762	0.587959
ACSL3	1.963435	0.784708	4.912753	0.149344
CHPF	2.47847	0.918346	6.689	0.073164
DNPEP	1.554366	0.665959	3.62793	0.307768
OBSL1	1.14179	0.05202	25.061	0.932945
FAM124B	1.429313	0.924619	2.209491	0.107989
SLC12A4	0.898966	0.349573	2.311792	0.82508
GCNT7	11.06242	0.50574	241.9764	0.126789
PI3	1.515843	1.111999	2.066352	0.008499
SNX21	2.131736	0.372913	12.18595	0.394776
SLPI	1.093218	0.856815	1.394847	0.473424
TTPAL	0.738948	0.285311	1.913861	0.53324
PREX1	2.892398	1.473402	5.677993	0.002028
KCNS1	1.350204	0.33099	5.507867	0.675523
SDC4	2.367714	1.139573	4.919446	0.02088
NCOA3	1.452498	0.404572	5.214769	0.567063

PIGT	0.802703	0.349017	1.846135	0.60503
MATN4	2.52559	0.404922	15.75266	0.321206
NCOA5	1.557572	0.697615	3.477606	0.279565
VAPB	3.302644	0.527285	20.68607	0.201861
PARD6B	5.054265	0.497005	51.39906	0.170951
CHD6	1.062786	0.334689	3.374823	0.91773
PLCG1	1.363414	0.710622	2.615876	0.351118
TOX2	0.973253	0.228258	4.149784	0.970771
ARFGEF2	0.365297	0.138751	0.961737	0.041454
ZNFX1	4.598271	2.111405	10.01423	0.000122
ZNF831	0.965756	0.336261	2.773693	0.948388
CSE1L	0.470209	0.213255	1.036771	0.061421
RAB22A	0.648388	0.319556	1.315593	0.230073
STAU1	1.602486	0.428369	5.994747	0.483594
CDH26	0.475891	0.22617	1.001333	0.050412
SNAI1	6.583928	1.725018	25.12907	0.005819
MOCS3	1.412858	0.213505	9.349519	0.719997
STX16	0.382552	0.170016	0.860777	0.020217
PPP4R1L	0.48768	0.023793	9.995678	0.641214
PMEPA1	10.3059	1.454771	73.0091	0.019532
RNF114	0.54337	0.213648	1.381951	0.20029
DDX27	0.513542	0.219843	1.199609	0.123675
BCAS4	1.834166	0.853103	3.943448	0.120383
TP53TG5	3.703666	0.301327	45.5225	0.306376
ZBP1	1.234587	0.686087	2.221591	0.482021
NEURL2	1.151323	0.539567	2.456682	0.715557
MTRR	0.236602	0.056009	0.999497	0.04992
FASTKD3	0.846232	0.385522	1.857504	0.677241
PEPD	3.365281	1.770804	6.395466	0.000212
IQSEC2	2.593819	1.049489	6.410641	0.038961
VAMP7	0.525168	0.256215	1.076443	0.078612
IL9R	0.714589	0.163948	3.114631	0.654587
XG	1.588753	0.167719	15.04978	0.686538
STAMBP	0.262125	0.062702	1.09581	0.066565
NAGK	1.61943	1.021855	2.566464	0.040171
MCEE	0.572718	0.218397	1.501877	0.257167
PAIP2B	0.685004	0.131814	3.559794	0.652754
SNRNP27	0.554781	0.164851	1.867035	0.341309
MPHOSPH	1.299597	0.368006	4.589476	0.683949
IL17C	0.380435	0.032546	4.446913	0.441052
ATP8A1	0.583418	0.154088	2.208977	0.427627
USP22	0.530703	0.030095	9.35866	0.665245
POF1B	0.091033	0.010176	0.814361	0.032061
HIF3A	0.008066	2.03E-05	3.20416	0.114428
ZNF576	1.619182	0.573583	4.570835	0.362734
IRGC	1.041669	0.052338	20.73225	0.978657
ZNF45	0.340759	0.147192	0.788875	0.011948
LYPD3	1.40062	0.487228	4.026318	0.531734
CEACAM8	1.005922	0.856451	1.181478	0.942653
USP9X	0.990938	0.260967	3.762767	0.989331
CRISP2	2.614985	1.089388	6.277053	0.031429
F13A1	1.228904	1.015977	1.486455	0.033734
TRERF1	0.905187	0.217316	3.770373	0.891159
PACSIN1	1.650692	1.135495	2.399644	0.008648
BTN2A2	0.122097	0.021239	0.701883	0.01844
SIRT5	0.327791	0.077679	1.383214	0.128926
MRS2	0.232979	0.092714	0.585448	0.001943
WRNIP1	0.588519	0.29866	1.159695	0.125557

SNRPC	0.465935	0.137165	1.582739	0.220935
SERPINB6	0.986903	0.702017	1.387399	0.939531
XPO5	0.281762	0.12662	0.626994	0.00191
ABCC10	0.836696	0.327955	2.134626	0.709066
PEX6	1.859998	1.03533	3.341535	0.037883
NQO2	0.660219	0.376691	1.157154	0.147018
UNC5CL	1.554662	0.416452	5.803729	0.511463
AARS2	0.341806	0.118514	0.9858	0.046986
ZNF391	0.067751	0.003137	1.463197	0.085949
RPS10	2.094458	0.197547	22.20613	0.539414
MOCS1	10.52388	1.774833	62.40134	0.00955
MED20	0.92786	0.471808	1.824734	0.828217
OR2B6	1.216877	0.161284	9.181251	0.849018
TBCC	1.688722	1.011153	2.820325	0.045247
SPDEF	4.653084	0.311416	69.52492	0.265109
TCP11	3.222848	0.712878	14.57017	0.128438
MAD2L1BI	2.397693	0.547218	10.50574	0.245993
APOBEC2	0.819192	0.218832	3.066621	0.767135
KLHDC3	0.574164	0.26081	1.264002	0.168182
GNMT	0.189335	0.048006	0.746736	0.017449
DNAH8	1.370922	0.120247	15.62975	0.799439
TREM1	1.122867	0.821273	1.535215	0.467743
MEA1	1.184218	0.401391	3.493779	0.75937
CDKN1A	1.378698	1.014126	1.874333	0.040415
SOX4	0.925548	0.701405	1.221319	0.584484
GLO1	0.701392	0.360422	1.364929	0.296422
CPNE5	1.67838	0.483754	5.82313	0.414585
KCNK17	1.01037	0.731244	1.396043	0.950134
RREB1	0.540939	0.104839	2.791098	0.46299
SSR1	0.437297	0.215993	0.885348	0.021543
RIOK1	0.077036	0.013341	0.444843	0.004165
NRN1	0.120656	0.016704	0.871505	0.036056
SLC35B3	0.802272	0.34476	1.86692	0.60918
RPP40	0.860752	0.525101	1.410956	0.552066
ATXN1	0.52566	0.223808	1.234622	0.139897
NUP153	0.980189	0.521678	1.841689	0.950416
DEK	0.720609	0.423596	1.225881	0.226782
EEF1E1	0.22069	0.062584	0.778219	0.018777
RUNX2	0.718249	0.189884	2.716823	0.625874
LRRFIP1	1.224672	0.793877	1.889239	0.35949
RAB17	0.262688	0.044728	1.542773	0.138887
CXCL6	1.330372	0.219027	8.080704	0.756459
EREG	0.799412	0.512004	1.248152	0.324696
AHNAK	1.735312	1.076409	2.797548	0.023688
WNT1	0.370176	0.047476	2.886297	0.342929
SH3TC1	1.565652	1.168216	2.098301	0.002694
CNOT1	0.307333	0.05552	1.701267	0.176586
LRRC29	0.334408	0.04558	2.453479	0.281351
BBS2	0.488277	0.214014	1.114014	0.088491
MT1G	1.237043	0.994936	1.538063	0.055584
MT2A	1.354482	1.016753	1.804393	0.038126
GOT2	0.693205	0.286621	1.676544	0.416103
DOK4	1.84337	0.958118	3.54655	0.066978
GPR18	1.276689	0.73546	2.216212	0.385361
CLYBL	0.332672	0.106693	1.037284	0.057844
TMTC4	0.24725	0.078468	0.77908	0.01702
RAP2A	0.927245	0.524425	1.639478	0.795036
SLC10A2	0.655265	0.145677	2.94742	0.581634

ABCC4	0.588624	0.338589	1.023298	0.060335
EFNB2	0.582673	0.187413	1.811552	0.350675
TM9SF2	1.316139	0.607003	2.853732	0.486626
IRF1	0.910149	0.597718	1.385891	0.660784
UPF3B	0.374338	0.177846	0.787923	0.009663
RNF113A	0.701143	0.264243	1.860415	0.475785
NDUFA1	1.010872	0.530074	1.927774	0.97381
BMP4	0.646429	0.031471	13.27778	0.777232
PTGER2	0.825199	0.597745	1.139204	0.242888
FAM193A	1.576153	0.535715	4.637276	0.4086
GRK4	0.097443	0.011046	0.859603	0.036069
HS3ST3B1	2.613863	0.336047	20.3313	0.358602
SLC25A35	0.589985	0.211178	1.64829	0.314121
MRPS7	1.334276	0.502445	3.543257	0.562764
GGA3	1.224079	0.346395	4.325607	0.753581
ARMC7	1.643655	0.908114	2.974958	0.100681
NUP85	0.690769	0.288894	1.65168	0.405539
SLC25A19	0.766264	0.403191	1.456282	0.416433
MIF4GD	0.743741	0.336364	1.644499	0.464606
NT5C	0.699688	0.346813	1.411607	0.318627
MSTO1	0.448081	0.169352	1.185561	0.105859
TTF1	0.556936	0.094181	3.293412	0.518612
GTF3C4	0.238773	0.023546	2.421338	0.2256
DDX31	0.961468	0.155714	5.936644	0.966255
PPP1R12C	2.778653	1.268312	6.08755	0.010651
MBOAT7	1.523272	0.927771	2.501004	0.09619
SRMS	0.103452	0.004154	2.576269	0.166652
OPRL1	5.893571	1.692599	20.52121	0.005324
SLC2A4RG	0.248265	0.092336	0.667517	0.005764
PPDPF	1.96235	1.237614	3.111483	0.004151
IL1B	1.070528	0.808961	1.416671	0.633512
CHCHD5	1.916878	0.959842	3.828151	0.065209
PAX8	1.23163	0.248215	6.111286	0.798782
INSIG2	0.783356	0.366225	1.675598	0.529084
POLR1B	0.846409	0.201691	3.552014	0.819746
CCDC93	0.452151	0.224296	0.911476	0.02648
PSD4	1.270877	0.626787	2.576836	0.50627
SLC25A23	1.635332	1.155364	2.314692	0.005526
PSPN	4.256713	0.653193	27.74003	0.129865
GTF2F1	1.747272	0.769498	3.967473	0.182287
ALKBH7	1.444084	0.855348	2.438046	0.16906
CLPP	1.512933	0.75971	3.012948	0.238776
TNFSF9	1.403981	0.876528	2.24883	0.158045
THOC2	1.202864	0.340448	4.249932	0.774255
MED1	1.008299	0.228987	4.439845	0.991281
RPL23	1.142009	0.393142	3.317338	0.807183
ATG4C	0.248322	0.068816	0.896068	0.033373
CD70	1.202119	0.878352	1.64523	0.250224
C3	0.932077	0.021576	40.26616	0.970797
SH2D3A	0.640154	0.120225	3.408574	0.60114
TRIP10	2.146632	0.949524	4.852988	0.06643
GPR108	2.670458	1.429896	4.987318	0.002056
TNFSF14	0.798306	0.187547	3.398035	0.76051
FOSB	0.979879	0.771626	1.244337	0.867579
OPA3	1.413909	0.330431	6.050087	0.640517
SNRPD2	0.087864	0.012439	0.620658	0.014763
RTN2	2.269484	0.873965	5.893322	0.092322
EML2	0.779605	0.218699	2.779084	0.701054

VASP	0.790749	0.330643	1.891118	0.597687
SYMPK	1.39186	0.611414	3.168515	0.430826
SDCBP2	4.195503	0.060095	292.905	0.507991
PANK2	1.141337	0.333254	3.90888	0.833296
GNRH2	0.241976	0.003782	15.48099	0.503654
CD93	1.085653	0.892481	1.320637	0.411027
GZF1	0.682456	0.281958	1.651829	0.396919
NAPB	0.472109	0.257465	0.865697	0.01526
CENPB	1.707083	0.78805	3.697902	0.175099
PSMF1	0.135863	0.014768	1.249911	0.077909
DTD1	2.082368	0.997099	4.348875	0.050911
RBCK1	0.860928	0.374129	1.981123	0.724717
TMX4	0.410704	0.19391	0.869879	0.020124
STK35	2.419393	0.974646	6.005732	0.056832
SNRPB	0.480989	0.193352	1.196524	0.115466
NRSN2	1.103718	0.434831	2.801531	0.835506
RRBP1	1.476519	0.761505	2.862897	0.248713
BMP2	1.062926	0.488203	2.314227	0.877824
ZNF133	1.24583	0.401819	3.862662	0.703412
MKKS	0.46761	0.098083	2.229329	0.340138
BFSP1	4.979147	0.300254	82.56968	0.262583
DSTN	0.989977	0.557607	1.757609	0.972562
SNRPB2	0.485485	0.197056	1.196086	0.116241
TBC1D20	2.35392	0.569733	9.725499	0.23692
ITPA	0.988239	0.382802	2.551231	0.980494
MCM8	0.131315	0.02658	0.648736	0.012742
FAM110A	1.533585	0.807374	2.913002	0.191448
SIRPD	1.561031	0.603282	4.039273	0.358561
MRPS26	0.365702	0.176332	0.758444	0.006875
S1PR4	1.735676	1.197597	2.515512	0.003587
NCLN	0.650306	0.32394	1.305484	0.226188
HNRNPR	0.400112	0.13953	1.147355	0.088339
ZNF436	0.259377	0.027825	2.417887	0.236095
MAX	0.413024	0.139117	1.226231	0.111242
ARMCX5	1.024952	0.376985	2.786652	0.96148
MMP24	1.660422	0.186571	14.77726	0.649371
NECAB3	4.11445	1.331754	12.71158	0.01398
ID1	1.418923	0.960551	2.09603	0.078787
RALY	1.646449	0.779677	3.476817	0.191076
DYNLRB1	0.740014	0.262847	2.083419	0.568603
EIF2S2	0.497398	0.212762	1.162824	0.107005
ERGIC3	1.919296	0.871593	4.226393	0.105504
ROMO1	0.78255	0.290234	2.109968	0.628019
CEP250	0.454332	0.049346	4.18303	0.486097
PLAGL2	0.942839	0.494492	1.797693	0.858128
AMOT	2.863967	1.153728	7.109392	0.023316
TMEM115	2.246859	0.990657	5.095989	0.052685
PSMB2	0.581582	0.247293	1.36776	0.214161
UROD	0.778759	0.476571	1.272562	0.318287
ST3GAL3	0.266606	0.084388	0.842282	0.024297
TMEM53	0.918683	0.30635	2.754952	0.879687
HECTD3	1.878751	0.821067	4.298927	0.135398
KLC1	1.099308	0.287998	4.196128	0.889814
XRCC3	0.735701	0.331897	1.630794	0.449801
TUBGCP3	0.80687	0.154119	4.224254	0.799443
MCF2L	1.227707	0.065171	23.12798	0.891067
PCID2	0.251024	0.093276	0.675556	0.00621
PROZ	2.045174	0.149639	27.95214	0.59178

LRFN3	1.133698	0.748587	1.716927	0.553472
CAPNS1	2.259403	1.196952	4.264918	0.011918
PDCD2L	0.85928	0.376243	1.962459	0.718902
RBM42	1.889317	0.955243	3.736763	0.067493
KIRREL2	0.025977	0.000363	1.860242	0.093907
UBA2	0.256861	0.095721	0.689275	0.006958
FFAR2	1.179044	0.892319	1.557901	0.246638
HCST	0.993335	0.605697	1.629056	0.978863
FFAR1	0.17606	0.018109	1.711673	0.134446
COX6B1	1.107734	0.454716	2.69855	0.821807
THRA	0.901284	0.207772	3.909638	0.889588
CCR7	1.056323	0.752179	1.483447	0.751806
NR1D1	2.379293	0.34994	16.17714	0.375443
FRMD8	1.921054	0.780896	4.725916	0.155173
PRDX5	2.551846	1.115435	5.838004	0.026508
BCL2L12	0.704148	0.272437	1.819963	0.469071
IRF3	1.586549	0.676732	3.719547	0.288355
PRMT1	0.86175	0.397282	1.869237	0.706458
RRAS	1.37087	1.082365	1.736276	0.008885
PRRG2	1.237048	0.480767	3.183014	0.659099
SCAF1	4.553537	1.488273	13.93205	0.007888
PRR12	4.549324	0.96261	21.50024	0.055892
TSKS	1.058261	0.527193	2.124301	0.87345
FLRT1	3.266977	0.343141	31.10427	0.303167
ASL	0.576092	0.104559	3.174122	0.526477
SBDS	0.668855	0.392601	1.139496	0.138989
CSN1S1	0.73994	0.374241	1.462993	0.386501
STAT5A	1.158606	0.734469	1.827671	0.526728
WNK4	1.120272	0.094359	13.30032	0.928314
BECN1	0.756613	0.356419	1.606153	0.467719
PRKCG	0.257993	0.023185	2.870792	0.27042
TRAP1	1.275432	0.677704	2.400349	0.450799
GLIS2	2.467715	0.301998	20.16442	0.399337
DNAJC8	0.544605	0.224833	1.319175	0.178211
AHDC1	1.901725	1.092462	3.310468	0.023048
IFI6	1.098486	0.696485	1.732517	0.686171
ZNF384	1.085631	0.268817	4.384374	0.908157
EMG1	1.378597	0.664371	2.860646	0.388661
UXT	1.016363	0.258083	4.002568	0.981484
CFP	1.584872	1.168969	2.148747	0.003024
ELK1	1.136639	0.429947	3.004901	0.796245
TIMM17B	0.668603	0.310078	1.441668	0.304481
KTN1	0.537915	0.250044	1.157205	0.112647
RHOJ	1.973299	0.0876	44.45121	0.668858
DLGAP5	0.886576	0.616819	1.274307	0.515437
HSPA2	5.436932	0.935758	31.58962	0.059294
ZBTB1	2.330359	0.294489	18.44067	0.422775
TRMT5	0.241986	0.069555	0.841884	0.025712
SGPP1	1.197459	0.273645	5.240042	0.810898
PLEKHG3	1.277785	0.897188	1.819837	0.174259
PZP	1.124995	0.116911	10.8254	0.918791
RHOT1	0.404688	0.133386	1.227809	0.110146
EVI2A	1.784545	1.145814	2.779336	0.010404
OMG	4.362153	0.362951	52.42682	0.245611
AIF1L	1.434676	1.140524	1.804693	0.002048
FAM78A	2.034425	0.803842	5.148883	0.133854
NUP214	2.452178	1.365492	4.40367	0.002675
CTAG2	0.748673	0.146239	3.832829	0.728294

AVPR2	0.782014	0.151675	4.031947	0.768887
SLC10A3	0.483148	0.200746	1.162821	0.104519
MAP2K2	2.056328	1.076201	3.929086	0.02909
HNRNPH2	0.67907	0.312624	1.47505	0.328132
ARMCX1	1.728761	1.047303	2.853628	0.032298
TIMM8A	0.390327	0.154139	0.988429	0.047198
ZC4H2	0.969661	0.200123	4.698317	0.969475
CANX	0.523611	0.282415	0.970801	0.039972
IPPK	2.600139	0.584877	11.55922	0.209354
ZNF484	0.859262	0.289939	2.54651	0.784357
OMD	0.719475	0.050888	10.17228	0.807537
FGD3	1.263403	0.669833	2.382962	0.470177
HIVEP3	7.1576	1.739417	29.45312	0.006393
PPCS	1.299497	0.589185	2.866154	0.516247
BCL11B	0.80551	0.427244	1.518681	0.503825
COX7C	0.914296	0.460456	1.815456	0.797935
TRAF2	1.075632	0.321781	3.595563	0.905741
ABHD8	1.611464	0.81887	3.171218	0.16715
ATP13A4	1.233129	0.113108	13.44386	0.863498
HELB	0.321573	0.087826	1.17743	0.086656
RAP1B	1.604727	0.839045	3.069142	0.152851
BEST3	0.707771	0.05163	9.702589	0.795823
RAB3IP	0.256623	0.103282	0.63763	0.0034
PTPRB	0.860921	0.057482	12.89417	0.913643
DYRK2	0.566505	0.170027	1.887506	0.354738
YEATS4	0.884935	0.491047	1.594777	0.684164
TAS2R3	5.968322	0.564602	63.09028	0.137585
TAS2R4	1.008093	0.068511	14.83337	0.995312
TAS2R5	0.119223	0.014748	0.963822	0.046095
LRRC61	1.178337	0.531298	2.613367	0.686363
IDUA	1.200709	0.712124	2.024511	0.492562
FGFRL1	1.471845	0.881798	2.456716	0.139217
TMEM175	1.068196	0.609259	1.872836	0.817871
PIN1	0.653155	0.25211	1.692166	0.380505
FBXL12	0.777342	0.284439	2.124397	0.623404
UBR4	0.895679	0.498196	1.610294	0.712785
HP1BP3	0.29436	0.089947	0.963324	0.043204
SIN3B	5.408398	2.296963	12.73454	0.000112
SLC35E1	2.119348	0.72878	6.163226	0.167873
EPS15L1	0.390075	0.133106	1.143135	0.086143
KLF2	1.557276	1.083989	2.237206	0.016563
F2RL3	2.623211	0.160086	42.98457	0.499088
GFER	0.168371	0.020755	1.365893	0.09531
SYNGR3	0.474897	0.035289	6.390807	0.57449
PKMYT1	0.918256	0.496519	1.698211	0.785743
WFIKKN1	1.042864	0.172628	6.300055	0.96352
WDR24	1.333486	0.569736	3.12107	0.507128
FBXL16	0.969162	0.205636	4.567654	0.968412
CHTF18	0.47903	0.209882	1.093327	0.080456
GNG13	1.710641	0.135911	21.53093	0.677794
MACF1	0.354499	0.126885	0.99042	0.047892
SMARCA4	0.612043	0.294728	1.27099	0.187906
TICAM1	2.526919	1.190775	5.362321	0.015744
IL17B	0.016633	0.000635	0.435682	0.013947
TUBA4A	1.089707	0.861189	1.378862	0.474339
VIL1	1.271491	0.096371	16.77568	0.855202
AAMP	1.855169	0.751085	4.582238	0.180401
PNKD	2.153731	0.836275	5.546691	0.111943

TNFRSF19	0.130549	0.022681	0.751426	0.022608
RNF6	0.012173	0.000436	0.339999	0.00946
ECHS1	0.395757	0.200484	0.781224	0.007551
AKAP9	0.07848	0.006594	0.934108	0.04402
GNG11	1.184401	1.016194	1.380449	0.030348
HIP1	1.641134	1.058089	2.545458	0.02696
PTPN12	1.244688	0.729585	2.123466	0.421896
POR	2.853115	1.592728	5.110895	0.000424
FGL2	1.163734	0.961356	1.408714	0.119792
STYXL1	1.215087	0.651479	2.266282	0.540159
STEAP4	3.611543	0.774268	16.8459	0.102185
GNAI1	1.710495	0.653075	4.480029	0.274538
PEX1	0.275466	0.101379	0.748488	0.011472
CASD1	0.643418	0.250824	1.650506	0.358913
ZNF780B	5.792064	0.289927	115.712	0.250303
LRFN1	2.96334	1.002362	8.760688	0.049503
ZFP36	1.290851	0.955716	1.743505	0.095987
SRD5A3	1.405483	0.408832	4.831774	0.589016
SPINK2	1.127771	0.952579	1.335183	0.162728
PAICS	0.382073	0.1805	0.80875	0.011911
KDR	0.249354	0.037137	1.674272	0.152858
PPAT	0.598566	0.3046	1.176234	0.136481
TUBGCP6	1.090911	0.352725	3.373975	0.87994
ADM2	0.964583	0.284254	3.273204	0.953873
DGCR6L	1.351142	0.74801	2.440589	0.318488
DGCR8	1.265373	0.436726	3.666299	0.664552
ASPHD2	1.808853	1.096144	2.984964	0.020386
VPREB3	1.138818	0.725252	1.788213	0.572323
SDF2L1	0.979006	0.617259	1.552757	0.928161
YWHAH	0.450466	0.185233	1.095483	0.078606
RFPL2	2.018197	0.392253	10.38391	0.400795
GNAZ	0.528198	0.105823	2.636417	0.436489
MGAT3	0.415344	0.200781	0.8592	0.017829
ADORA2A	1.421689	0.954039	2.118571	0.083848
ATF4	0.322331	0.149797	0.693587	0.003782
A4GALT	1.148658	0.680109	1.940005	0.604247
CDC42EP1	3.883397	1.072574	14.06035	0.038763
APOL3	1.262842	0.378734	4.210788	0.704094
TPST2	0.984206	0.475466	2.037289	0.965791
BAIAP2L2	0.428995	0.160075	1.149691	0.092448
MPST	1.057779	0.433462	2.581301	0.901785
GALR3	5.014755	0.135202	186.0018	0.381798
TST	0.769596	0.462877	1.279557	0.312677
IGLL1	0.781736	0.603692	1.01229	0.061856
APOL2	1.296991	0.055068	30.54723	0.871833
RAC2	0.702636	0.307126	1.607474	0.403259
LIF	0.031475	0.002533	0.391193	0.007147
APOBEC3/	1.14331	0.847523	1.542329	0.380577
APOBEC3I	1.749079	0.532585	5.744202	0.356773
RIBC2	0.950028	0.081961	11.012	0.967291
KRT17	1.566875	1.104669	2.222472	0.011797
DOCK4	8.340913	0.628353	110.7193	0.107885
POT1	1.195853	0.191697	7.460035	0.848143
ATP6V1F	1.470918	0.58508	3.697955	0.411986
LSM8	0.453341	0.159127	1.291528	0.138598
PRKRIP1	0.306426	0.121544	0.772531	0.012176
VGF	1.660733	0.533873	5.166082	0.380994
PODXL	0.05433	0.003155	0.935478	0.044867

MKLN1	0.724589	0.456726	1.149549	0.171282
DNAJB9	1.106544	0.669142	1.829866	0.693218
FLNC	0.9461	0.553392	1.617487	0.839526
LRRC4	0.158625	0.01365	1.843376	0.141222
CALU	0.618855	0.287604	1.331626	0.219663
CCDC136	1.077357	0.630327	1.841422	0.785279
SMO	1.573772	0.802093	3.087867	0.187278
IRF5	1.400549	0.982029	1.997435	0.062908
LRRC17	1.366833	0.364263	5.12881	0.643244
KLHDC10	0.863204	0.173417	4.296694	0.857432
NDUFA5	1.407574	0.748643	2.646473	0.288564
OPN1SW	0.172036	0.010658	2.77705	0.214889
MRPS12	1.21052	0.388103	3.775698	0.742022
MYO1B	1.64582	0.85751	3.158824	0.134176
MTX2	0.257299	0.085479	0.774491	0.015757
GAD1	0.336733	0.077484	1.463384	0.146492
OSGEPL1	0.475346	0.161149	1.40214	0.177807
ORMDL1	0.183719	0.064581	0.522638	0.001491
HAT1	1.266335	0.733133	2.187333	0.39713
HERC2	0.42772	0.198734	0.920549	0.029882
SNRPN	0.690267	0.492155	0.968128	0.031743
PSMG2	3.999254	1.574118	10.16063	0.003572
TWSG1	0.713982	0.329437	1.547397	0.393275
ARHGAP2:	1.277687	1.001078	1.630725	0.048997
WDFY4	1.54932	0.60894	3.941918	0.358154
EIF2AK4	0.574296	0.245852	1.34152	0.200111
MYO5C	1.291247	0.87346	1.908867	0.199979
CGNL1	1.163988	0.190473	7.113177	0.869397
TMOD2	1.789164	0.631163	5.071763	0.273819
TTBK2	0.038618	0.001482	1.0062	0.050436
ELL3	1.032389	0.135429	7.870027	0.975463
INO80	1.170692	0.32138	4.264482	0.811154
DLL4	0.445808	0.017633	11.27095	0.623993
ALDH1A2	7.829767	0.513199	119.4571	0.13883
IVD	1.050228	0.519742	2.122166	0.891388
DUT	0.301461	0.13601	0.668176	0.003148
CHAC1	0.477749	0.065435	3.488101	0.466465
CLN6	1.621818	0.724292	3.631537	0.239717
ARPP19	0.671183	0.291437	1.545742	0.34888
VPS13C	0.913671	0.325286	2.566345	0.863956
LOXL1	30.10348	0.472239	1918.985	0.108264
ANAPC13	0.613334	0.296559	1.268477	0.187329
MBD4	0.943264	0.541653	1.642652	0.8365
COPB1	0.964469	0.470238	1.978149	0.92137
PSMA1	0.051884	0.013002	0.207037	2.78E-05
SUMF2	0.593211	0.307664	1.143777	0.119012
PALLD	1.088788	0.732057	1.619353	0.674485
SPCS3	0.959707	0.416327	2.212293	0.923108
MYOD1	14.69524	1.13681	189.9614	0.039575
SERGEF	3.957515	1.482311	10.56588	0.006041
TPH1	0.0193	0.001235	0.301676	0.004888
E2F8	0.299248	0.05919	1.512918	0.144511
DCTD	0.69137	0.289624	1.650389	0.405754
SOX15	1.429702	1.05698	1.933856	0.020365
RPAIN	0.313777	0.144123	0.683139	0.003501
USP6	1.133608	0.561093	2.290295	0.726718
SHBG	0.156434	0.012609	1.940789	0.148776
PLD2	4.141715	1.609229	10.65964	0.003216

CD68	1.496487	1.062598	2.107545	0.021026
TXNDC17	1.618867	0.844022	3.105048	0.147154
ATP1B2	0.922224	0.484786	1.754378	0.805088
FXR2	2.61625	0.828776	8.25888	0.101055
KIF1C	1.288602	0.411369	4.036512	0.66339
MPDU1	1.791387	1.034473	3.102128	0.037439
PHF20L1	0.340737	0.051787	2.241904	0.262682
LRRC6	0.734673	0.296547	1.820098	0.505336
CCNT1	0.591343	0.259894	1.3455	0.2104
PUS7L	1.096337	0.432015	2.782207	0.846512
KRI1	0.6578	0.186143	2.324558	0.515493
ILF3	0.438879	0.19252	1.000489	0.050136
SLC44A2	0.917558	0.672267	1.252348	0.587729
CDKN2D	1.061901	0.623138	1.809603	0.82522
MTUS1	0.008191	5.89E-05	1.139576	0.056381
SIGLEC9	1.414568	0.798337	2.506463	0.234717
NGDN	0.784143	0.376945	1.631221	0.515276
RIPK3	1.641795	0.729269	3.696154	0.231133
ADCY4	0.475962	0.232334	0.975059	0.042459
RAB2B	0.459243	0.220713	0.955557	0.037382
BCL2L2	0.422881	0.229647	0.77871	0.005729
PARP2	0.356911	0.131866	0.966018	0.042558
HEATR5A	0.26376	0.06773	1.027158	0.05469
SNX6	0.736431	0.22818	2.376769	0.608813
EAPP	1.039446	0.498071	2.169266	0.91791
NRL	0.185286	0.010637	3.227553	0.247559
RNASE1	1.911653	1.241806	2.942827	0.003241
NEDD8	0.496666	0.181738	1.357322	0.172456
DAD1	1.223074	0.675381	2.214911	0.506303
TEP1	1.170915	0.120423	11.38524	0.89185
EPB41L4A	0.685579	0.136709	3.438109	0.646336
REEP5	0.931733	0.619577	1.401159	0.734106
ITFG1	0.592238	0.141575	2.477459	0.4731
QRICH2	0.476523	0.054199	4.189596	0.503935
FOXJ1	0.14019	0.021194	0.927297	0.041523
SEC14L1	1.281831	0.554569	2.962824	0.561364
RHBDF2	1.971023	1.004758	3.866535	0.048408
AANAT	0.031251	0.001254	0.779093	0.034679
ARHGEF6	0.632694	0.389675	1.02727	0.064146
MAP7D3	0.749836	0.231656	2.427113	0.630946
ASH2L	0.408192	0.195704	0.851389	0.016899
CHRNA10	0.270041	0.056701	1.286089	0.100174
CDKN1C	1.416137	0.968056	2.071619	0.073022
RPS4Y1	0.997956	0.891671	1.116911	0.971593
KLF16	0.590742	0.116827	2.987115	0.524406
DOHH	1.882869	0.872142	4.064932	0.107057
SHC2	21.07066	0.334765	1326.221	0.149256
LBP	0.346065	0.067574	1.772297	0.202921
CBFA2T3	3.132272	0.991145	9.898784	0.051797
GAMT	0.642093	0.327554	1.258673	0.197033
PHF10	0.573185	0.147713	2.224187	0.421125
PRRG3	0.174508	0.012581	2.4205	0.193212
STARD8	1.757012	1.221466	2.527364	0.002378
GDPD2	6.44024	0.603476	68.72967	0.123104
SAT1	0.88406	0.602247	1.297743	0.52921
GNL3L	0.743841	0.320831	1.72458	0.490359
SH3BP4	1.35176	0.998207	1.830536	0.051371
MOSPD2	1.071665	0.530559	2.164632	0.846993

DOCK6	1.541365	0.673814	3.525911	0.305444
ECSIT	2.830327	1.267055	6.322334	0.011175
LDLR	1.336572	0.981784	1.819571	0.065305
ELOF1	2.15822	1.087498	4.283148	0.027819
TSPAN16	0.490394	0.053207	4.519865	0.529485
PRKCSH	1.270248	0.558174	2.890728	0.568564
CDC16	0.493293	0.239071	1.017849	0.055864
APOE	0.96753	0.734671	1.274195	0.814224
TOMM40	0.515136	0.240768	1.10216	0.087394
APOC1	0.690469	0.477188	0.999077	0.04943
GADD45G	0.607856	0.336767	1.097163	0.098492
XPO7	1.131714	0.716501	1.787544	0.59574
FAM98C	3.079087	1.100858	8.612172	0.032107
SAFB2	0.69885	0.317665	1.537441	0.373069
RPL36	0.912779	0.297804	2.797701	0.87312
ATP8B3	1.105614	0.692056	1.766305	0.674459
GTPBP3	1.833062	0.412046	8.154714	0.426189
PLVAP	4.126045	1.153806	14.75487	0.029256
BST2	1.403593	0.965267	2.040962	0.075915
SLC27A1	1.834317	1.073364	3.134743	0.026493
NSUN5	0.481908	0.223694	1.038179	0.062283
USHBP1	1.286409	0.138558	11.94338	0.824685
DDA1	0.86361	0.345792	2.156848	0.753521
MRPL34	2.63521	1.135304	6.116716	0.024112
PGLS	2.272973	1.289868	4.005377	0.004504
LSM7	1.312126	0.698937	2.463276	0.397931
TULP4	0.680681	0.211462	2.191068	0.518991
SNX9	0.206919	0.020189	2.120768	0.184569
RTN4IP1	0.356296	0.113215	1.121288	0.077689
QRSL1	0.394793	0.055008	2.833446	0.35536
RSPH3	0.586979	0.275907	1.248772	0.166608
MLLT1	0.983053	0.157133	6.150176	0.985423
FUT5	0.13259	0.011463	1.533623	0.105748
ACTN4	1.392493	0.608197	3.188174	0.433389
NDUFA10	0.821461	0.299469	2.253313	0.70246
ARPC1B	1.513018	0.936945	2.443284	0.090342
ZSWIM6	1.647666	0.957394	2.835619	0.071423
FCHO1	1.577951	0.883871	2.817072	0.12295
MAP1S	2.100152	1.209565	3.646466	0.008393
KLHDC7B	2.284696	0.850671	6.13614	0.101189
PXDN	1.194785	0.972209	1.468317	0.090646
SSBP4	2.314925	1.265863	4.233379	0.006421
GDF15	1.054044	0.822314	1.351076	0.67776
PGPEP1	0.797571	0.191411	3.323311	0.756082
LSM4	0.769155	0.36727	1.610806	0.486483
JUND	1.04899	0.468582	2.34832	0.907397
HRC	0.118206	0.011383	1.227515	0.07373
TRPM4	1.082806	0.780658	1.501899	0.633656
SULT4A1	0.342278	0.011525	10.1653	0.535481
ZNF557	2.7939	0.881677	8.853446	0.080817
CRB3	7.722107	0.342538	174.0857	0.198459
CAMSAP1	0.818887	0.35655	1.880732	0.637644
UBAC1	0.858107	0.474751	1.551019	0.612376
ZBTB46	1.24024	0.870604	1.766813	0.233069
SAMD10	2.814242	0.548063	14.4508	0.215145
LSP1	2.18434	1.113089	4.286575	0.02312
TNNT3	0.748389	0.316315	1.770658	0.509493
TNNI2	1.221698	0.694084	2.150383	0.487598

H19	1.53446	1.228806	1.916144	0.000158
COL5A1	0.875553	0.562644	1.362483	0.555835
ATXN10	0.429351	0.104667	1.761218	0.240387
TUBGCP2	2.780437	1.106199	6.988644	0.029661
CYP2E1	0.559613	0.313645	0.998474	0.049399
PNPLA7	0.999249	0.614373	1.625233	0.997584
HBZ	1.123664	0.906475	1.392891	0.287353
PAK4	3.612256	0.968702	13.46998	0.055798
MNX1	1.163794	0.792472	1.709105	0.439142
ZNF337	0.453933	0.21364	0.964499	0.039979
TAF4	1.613491	0.664628	3.917004	0.290427
LAMA5	0.836984	0.684841	1.022927	0.082122
OSBPL2	0.558868	0.161049	1.939367	0.359373
ADRM1	0.733372	0.452878	1.187593	0.207348
ASS1	2.838273	1.128838	7.136358	0.026583
EXOSC2	0.23121	0.090322	0.59186	0.002261
POMT1	0.24128	0.058581	0.993775	0.048998
UCK1	2.607658	0.471116	14.43356	0.272271
FIBCD1	1.683957	0.645213	4.395001	0.286994
CHMP2A	1.324478	0.337413	5.1991	0.687111
UBE2M	0.629167	0.237187	1.66894	0.35189
TRIM28	1.154643	0.686167	1.942969	0.588142
YIPF2	0.275365	0.068348	1.109407	0.069688
ATG4D	1.495752	0.552993	4.045753	0.427735
EIF2S3	0.546596	0.293482	1.018007	0.056947
TMEM160	1.320552	0.765818	2.277116	0.317215
ZC3H4	1.405639	0.615156	3.2119	0.419339
NPAS1	0.894959	0.526445	1.521434	0.681871
GMFG	1.335887	0.573117	3.113838	0.502404
MAP3K10	1.401462	0.322068	6.098384	0.652813
LRRC47	0.552316	0.205417	1.485046	0.239456
SESN2	1.490771	0.629064	3.532861	0.36439
SMPDL3B	0.479809	0.169262	1.360125	0.167158
MED18	1.054191	0.251792	4.413644	0.942416
CLIP1	1.527164	0.422151	5.524642	0.518661
CCDC62	0.041195	0.00387	0.438562	0.008217
HIP1R	0.090831	0.008704	0.947834	0.044991
ZNF317	0.496671	0.218562	1.128658	0.094725
PPAN	1.178976	0.613629	2.265188	0.621183
EIF3G	1.540999	0.686926	3.456963	0.294179
ANGPTL6	0.886038	0.54584	1.438268	0.624466
DNMT1	0.45608	0.237761	0.874865	0.018167
ZNF426	0.769961	0.376375	1.575131	0.474091
SLC6A8	1.02484	0.633151	1.658841	0.920455
DKC1	0.488623	0.278195	0.85822	0.012703
PLXNA3	1.199028	0.651009	2.208371	0.560232
MPP1	0.769236	0.48752	1.213741	0.259532
ZNF331	1.068459	0.417941	2.731496	0.890028
ZNF236	0.418808	0.140445	1.24889	0.11846
LRP3	1.110441	0.803396	1.534832	0.525845
NOL11	0.577843	0.297283	1.123179	0.105793
UBE4B	0.465053	0.233814	0.924985	0.029093
CASZ1	1.12994	0.302169	4.225334	0.855944
PKDREJ	0.218699	0.026862	1.78054	0.155392
HABP4	1.163098	0.430656	3.141249	0.765662
SLC35D2	1.521543	0.658225	3.517178	0.32622
PRRG1	0.542515	0.129185	2.2783	0.403562
UBA1	1.473585	0.737467	2.944478	0.272329

PPIL4	0.096494	0.009229	1.008913	0.050872
ULBP2	2.649181	0.61363	11.43711	0.19171
AKAP12	0.761374	0.038188	15.17986	0.858286
SYNE1	0.057518	0.000387	8.555524	0.263184
ULBP3	0.184342	0.01823	1.864032	0.15202
LATS1	0.434259	0.032555	5.792706	0.528017
EPS8L1	0.131501	0.001034	16.72962	0.41191
LILRB2	1.453347	0.891786	2.368524	0.133523
TTLL9	0.197904	0.002899	13.51214	0.452198
RBM39	0.564128	0.15257	2.085861	0.390867
ZNF341	1.430784	0.638791	3.204715	0.383938
GGT7	0.481638	0.107492	2.158073	0.339717
ACSS2	5.790086	1.926379	17.40316	0.001762
EDA2R	0.435725	0.064477	2.944583	0.394127
ARHGEF9	1.458155	0.272842	7.792838	0.659165
HIGD1B	0.241015	0.017843	3.255457	0.284037
ATP6V1E1	0.352999	0.145857	0.854318	0.020937
ZNF227	0.406961	0.114016	1.452577	0.166091
ZNF428	0.665992	0.346462	1.280212	0.222809
ZNF141	0.410528	0.088744	1.899101	0.254599
CCL25	2.248555	0.652711	7.74615	0.199158
COX4I1	1.145726	0.345843	3.795616	0.823848
GINS2	0.57736	0.384733	0.86643	0.007996
CHMP1A	2.337649	1.061636	5.14734	0.03499
SH3BGRL	0.566405	0.214827	1.493362	0.250468
COX7B	1.166405	0.733236	1.855475	0.515761
F12	1.155232	0.738778	1.806444	0.526973
PRR7	1.495413	0.977681	2.28731	0.063473
NFATC1	4.771179	1.935065	11.76402	0.00069
CAP1	1.296501	0.678432	2.477645	0.431961
PPT1	0.83	0.452682	1.521817	0.546904
RAB11FIP4	1.340557	0.524104	3.428885	0.540766
ABCB7	0.21605	0.087242	0.535037	0.000927
TRAF3	0.568822	0.180024	1.797305	0.336473
HAUS8	1.835914	0.569481	5.918685	0.309036
MRPS25	0.294519	0.140629	0.616806	0.001191
SH3BP5	9.067897	0.42232	194.7024	0.158817
HACL1	0.263565	0.144212	0.481698	1.46E-05
TBC1D5	0.46348	0.099709	2.154403	0.32663
CAPN7	0.428365	0.078687	2.331989	0.326793
RFTN1	1.084577	0.691563	1.70094	0.723617
SLC6A6	1.742432	0.667581	4.547864	0.25662
KCNC3	0.374142	0.019468	7.190288	0.514474
NAPSA	1.276176	1.013768	1.606506	0.037858
NAPSB	1.232617	1.024121	1.483559	0.026962
NR1H2	1.431672	0.705025	2.907247	0.32077
LRRC4B	4.409133	0.143745	135.2426	0.395638
PDLIM4	1.674581	0.111208	25.21597	0.709439
KIF3A	0.104867	0.008382	1.31201	0.080238
MGAT1	1.941669	1.170434	3.221095	0.010189
TUBG1	0.858847	0.415599	1.77483	0.681168
PSME3	0.778856	0.326078	1.860342	0.573706
RPL27	0.682202	0.19475	2.389726	0.549898
PSMC3IP	0.138652	0.022791	0.843497	0.031976
AOC3	0.584492	0.0683	5.001945	0.623945
ACLY	0.635493	0.292137	1.382404	0.252908
VPS25	1.962642	0.721065	5.342046	0.186886
RAMP2	0.984949	0.058909	16.46815	0.99158

AOC2	7.297371	0.094106	565.8702	0.37061
NDUFA2	1.437663	0.469967	4.39792	0.524552
ANKHD1	0.217781	0.018431	2.573277	0.226361
DIAPH1	1.43365	0.673201	3.053103	0.350316
NDFIP1	1.641315	1.091401	2.468309	0.017309
UBE2D2	0.257959	0.065968	1.008714	0.051476
EXOC4	0.35562	0.105079	1.203526	0.096485
ACAP3	1.279746	0.46543	3.518787	0.63267
PPFIA1	0.597857	0.168749	2.118132	0.425425
TMEM204	1.473556	0.957695	2.267287	0.077841
KREMEN2	0.091715	0.006708	1.253899	0.073389
THOC6	0.921117	0.494626	1.715351	0.795631
TRAF7	3.101217	0.890303	10.80255	0.075489
BARX1	0.196729	0.01689	2.291425	0.194282
NINJ1	1.243445	0.975816	1.584476	0.078071
CA6	0.507085	0.065659	3.916219	0.514987
NPHP4	2.803322	1.011127	7.772131	0.047565
MAP1B	0.739364	0.006898	79.25283	0.899251
IL13RA1	1.10069	0.852427	1.421258	0.461949
WDR44	1.105541	0.289631	4.219925	0.88328
CKMT2	0.601911	0.051732	7.003382	0.685154
ZCCHC9	0.555311	0.216068	1.427193	0.221943
TOP2A	0.852016	0.620884	1.16919	0.321262
STARD3	1.540074	0.72125	3.288499	0.264549
RARA	3.539365	1.33282	9.398944	0.011196
PPP1R1B	0.749363	0.024436	22.98065	0.868786
KHDRBS3	0.965858	0.655836	1.42243	0.860383
CHD1L	0.492397	0.267221	0.90732	0.023096
PEX11B	1.4251	0.769473	2.63935	0.25992
FMO5	0.871801	0.291981	2.603034	0.805822
PIAS3	1.890989	0.598373	5.975939	0.277826
PRKAB2	0.572637	0.203333	1.612688	0.291278
PDHA1	0.291812	0.109286	0.779187	0.013976
RAI2	6.130977	0.377822	99.48821	0.202171
MCCC2	0.346716	0.079253	1.516807	0.159516
ZNF304	2.04023	0.814266	5.112011	0.128126
ZSCAN5A	0.976857	0.349619	2.729401	0.964375
ZNF132	0.891116	0.15434	5.145053	0.897462
CHSY1	0.453218	0.250709	0.819302	0.0088
SNRPA1	0.315086	0.15736	0.630903	0.001113
LLGL1	1.737057	0.895951	3.367782	0.102111
THAP1	1.451893	0.464954	4.533769	0.521003
ACTR10	0.564541	0.221851	1.436577	0.230227
ABHD12B	0.330671	0.096364	1.134698	0.078562
GCH1	1.689473	0.971635	2.937645	0.063167
LGALS3	1.126996	0.902483	1.40736	0.291538
PODNL1	2.780628	0.377968	20.45645	0.315184
DNAJB1	1.038506	0.709587	1.519889	0.845832
ZSWIM4	0.882607	0.34046	2.288061	0.797228
FBXW9	0.779281	0.388665	1.562473	0.482289
RFX1	1.56552	0.825834	2.96773	0.169583
ZNF20	0.822193	0.422214	1.601086	0.564777
DCAF15	2.382843	1.047311	5.42145	0.038436
CC2D1A	3.271785	1.175061	9.109807	0.023285
TRIM21	2.397997	1.203654	4.777445	0.012881
SPATA6	0.976383	0.41806	2.280349	0.955958
LRRC41	0.886343	0.379632	2.069383	0.780328
CCT6B	0.653701	0.258336	1.654146	0.369474

DHX30	1.749668	0.606168	5.050314	0.300964
RAF1	1.182612	0.465835	3.002291	0.724198
PPARG	1.293155	0.445804	3.751087	0.636112
NUP210	1.064149	0.654328	1.730651	0.802141
FCRLA	1.171233	0.777125	1.765207	0.450137
HSD17B7	1.039214	0.548711	1.968186	0.906032
ENOSF1	0.64275	0.395931	1.043432	0.073779
EMILIN2	1.35468	0.876403	2.093963	0.171873
ARFIP2	0.790986	0.098353	6.361368	0.825526
TRIM5	0.806169	0.317437	2.047362	0.650476
CNGA4	0.603373	0.011118	32.74588	0.804192
TRIM22	1.237932	0.761192	2.013257	0.389664
RRP8	0.915616	0.330218	2.538781	0.865462
EFR3A	1.036462	0.648956	1.655359	0.880829
PTCD3	0.137026	0.022118	0.848919	0.032681
IMMT	1.092756	0.457913	2.607737	0.841572
MRPL35	0.69641	0.280415	1.729532	0.435645
ILKAP	0.133875	0.021528	0.832531	0.031042
PER2	0.249326	0.048656	1.277608	0.095694
RAMP1	0.507383	0.306785	0.839147	0.008214
SCLY	0.432992	0.136151	1.377021	0.156193
PTPRE	1.322148	0.755357	2.314238	0.328226
RAN	0.407239	0.18584	0.892397	0.024808
PRKAA1	0.761161	0.38808	1.492905	0.427171
CARD6	1.834072	0.478128	7.035402	0.376564
INPP5K	2.772741	1.025343	7.49807	0.044508
MYBBP1A	1.356101	0.613194	2.999067	0.451915
RPA1	0.306316	0.133014	0.705409	0.005437
SERPINF1	0.788574	0.443454	1.402286	0.418655
UBE2G1	0.441371	0.109451	1.779872	0.250314
EEFSEC	0.613332	0.175422	2.14441	0.444002
TBC1D14	1.759086	1.012624	3.055807	0.045018
TMEM128	1.460451	0.589059	3.620891	0.413606
COQ3	0.391101	0.159094	0.961447	0.040792
SEC61G	1.089605	0.694056	1.71058	0.709206
LANCL2	0.498569	0.180085	1.380302	0.180368
FIGNL1	0.461351	0.179624	1.184949	0.107975
GRSF1	0.481895	0.220037	1.055381	0.067971
ANKRD17	0.424043	0.142807	1.259131	0.122345
UTP3	1.19606	0.395098	3.620774	0.751401
ITGB4	0.033393	0.000285	3.906739	0.161779
WBP2	1.097897	0.584455	2.062396	0.771554
UNK	0.460309	0.049385	4.29048	0.495733
TRIM47	1.527665	0.928997	2.51213	0.09497
ZRANB2	0.564903	0.297314	1.073329	0.08118
EIF5A	0.565862	0.300559	1.065347	0.077753
KDM6B	2.747333	1.215816	6.208045	0.015108
CLEC10A	1.745734	1.090941	2.79354	0.020188
GUCY2D	3.242094	0.53534	19.63456	0.20055
GPS2	1.721897	0.708066	4.187363	0.230698
XAF1	1.033086	0.608671	1.753435	0.904013
DLG4	2.518651	1.472861	4.306993	0.00074
VPS13B	0.231812	0.008483	6.334903	0.386409
MATN2	1.66562	0.757097	3.66438	0.204707
REEP2	1.575264	0.300049	8.270174	0.591192
PCBD2	0.285941	0.046957	1.741231	0.174375
SDF2	0.590556	0.21641	1.611551	0.30381
FLOT2	0.740862	0.350789	1.564689	0.431681

ERAL1	0.978815	0.457627	2.09358	0.955979
PRMT7	1.073809	0.49607	2.324404	0.856574
NIP7	0.44331	0.210241	0.934755	0.03258
TERF2	1.885928	0.921609	3.859254	0.082472
VPS4A	2.247903	0.763807	6.615635	0.141363
HSPA12B	1.09303	0.082933	14.40578	0.946095
BTBD3	1.017468	0.306364	3.379124	0.977441
PCNA	0.20864	0.057215	0.760831	0.017594
NXT1	0.571064	0.303451	1.074687	0.082443
POLR3F	0.187236	0.064473	0.543749	0.00207
RIN2	1.551308	1.069108	2.250994	0.02079
PTPRA	1.647147	0.228162	11.89111	0.620736
DAP3	0.58587	0.228071	1.504982	0.266683
ARHGEF11	2.310306	0.833083	6.406939	0.107605
FCRL2	0.192928	0.005136	7.247732	0.3738
SYT11	1.125384	0.644503	1.965063	0.677881
IGHMBP2	2.954729	0.818069	10.672	0.098231
ACY3	0.950511	0.666229	1.356098	0.77953
MMACHC	0.578986	0.2744	1.221667	0.151451
DPH2	0.454314	0.128187	1.610161	0.221663
TOE1	0.485758	0.159987	1.474877	0.202583
NASP	0.143474	0.055845	0.368601	5.50E-05
MUTYH	1.011601	0.273219	3.745481	0.986221
CTNNBL1	0.436627	0.227753	0.837059	0.012574
LPIN3	0.056208	0.003819	0.827264	0.035889
ZSWIM3	9.260983	0.614145	139.6508	0.107878
RBM38	1.189982	0.934103	1.515953	0.159098
VSTM2L	1.671912	0.572159	4.885508	0.347509
SERINC3	0.394583	0.079086	1.96869	0.256808
PPP1R3D	1.892474	0.77226	4.637632	0.163061
AP3B1	0.77109	0.372767	1.595048	0.483331
ZBED3	0.700264	0.378352	1.296065	0.256654
ANGPTL3	4.57728	0.324703	64.52503	0.25985
FBXO44	0.061481	0.002084	1.813836	0.106281
CASP9	0.719552	0.201786	2.565859	0.611898
DCTN4	1.020359	0.479986	2.169088	0.958227
PDE6A	0.034733	0.000644	1.874174	0.098686
MTUS2	0.129792	0.001888	8.921039	0.344137
ZMYM5	0.400274	0.16066	0.997257	0.049315
USPL1	0.526491	0.290099	0.955511	0.034891
XPO4	0.693358	0.292555	1.643263	0.40552
POMP	0.338757	0.050061	2.292344	0.267172
CDK8	0.20129	0.0489	0.828579	0.026389
ALOX5AP	0.988484	0.748441	1.305515	0.934961
WASF3	0.291766	0.045295	1.879401	0.194946
GPR12	1.016399	0.670887	1.539852	0.938829
MYH10	1.029141	0.808853	1.309422	0.815189
PEMT	0.74632	0.218458	2.54966	0.640646
SCO1	0.758232	0.314546	1.827764	0.537551
MPRIP	0.424076	0.108844	1.652286	0.216356
CHI3L1	1.093365	0.613073	1.949924	0.762349
MYBPH	1.368977	0.976045	1.920095	0.068836
PIK3C2B	1.367247	0.673458	2.775772	0.386617
DSTYK	0.831767	0.361988	1.911214	0.664315
CHIT1	1.467238	0.538245	3.999642	0.453677
SLC41A1	0.914003	0.422137	1.978983	0.819534
LGR6	0.078009	0.001075	5.658507	0.243192
TMCC2	1.115775	0.889689	1.399313	0.343006

CCNA1	0.908841	0.734579	1.124443	0.378822
COG6	0.459265	0.178139	1.184041	0.107321
RXFP2	2.229786	0.144441	34.42186	0.565772
EPSTI1	1.146648	0.882399	1.49003	0.305896
RFXAP	0.071718	0.01109	0.463787	0.005663
TPT1	2.545271	0.00401	1615.696	0.776608
RFC3	0.3063	0.093289	1.005692	0.051104
STARD13	28.26051	0.355572	2246.117	0.13445
MORC4	14.1378	1.195159	167.2391	0.035606
BEX2	1.27723	0.989955	1.647869	0.059795
TCEAL4	0.537176	0.260384	1.108202	0.092588
BEX1	1.004409	0.834855	1.208397	0.962808
FAM104A	1.072657	0.564228	2.039236	0.83056
SLC39A11	0.974722	0.51618	1.840603	0.937082
EPHB2	4.63143	0.513961	41.73493	0.171761
SRRM1	0.337972	0.143401	0.796544	0.013138
BTBD2	0.78342	0.434255	1.413333	0.417482
PRAM1	1.224314	0.695457	2.155339	0.483086
ZNF414	3.492546	1.302085	9.367958	0.01298
PDE6B	0.584798	0.192499	1.776576	0.343999
HSPBP1	1.33717	0.458424	3.900366	0.594749
CSNK1G2	0.687653	0.286606	1.64988	0.401675
CNDP2	0.954525	0.611619	1.489682	0.837619
MACROD1	1.046974	0.660297	1.660093	0.845256
WDR74	0.686792	0.269794	1.748309	0.430621
LGALS12	0.826999	0.409045	1.672009	0.59691
RTN3	1.318647	0.423218	4.108592	0.633337
MYH11	0.046507	0.002476	0.873581	0.040337
MED10	0.312502	0.124575	0.783926	0.013184
PDZD2	1.275252	0.262527	6.194656	0.763024
MORC2	0.938378	0.383855	2.293971	0.889089
MYO18B	0.146271	0.023827	0.89793	0.037871
SLC2A11	1.379764	0.134935	14.10868	0.786095
C1QTNF6	0.656755	0.019156	22.51608	0.815657
FAM83F	1.052428	0.450726	2.45738	0.905984
SEC14L4	0.958231	0.626036	1.466697	0.844257
GIMAP6	2.260458	1.099587	4.646898	0.026544
GIMAP4	1.187219	0.934156	1.508837	0.160589
ADCK2	1.326084	0.67851	2.591707	0.409084
MKRN1	0.960955	0.542747	1.701407	0.891314
AGAP3	1.976639	0.507477	7.699074	0.325995
KRBA1	0.898123	0.541511	1.489583	0.677235
ACTR3B	1.553604	0.086479	27.91052	0.764973
BTG1	1.052491	0.761502	1.454673	0.756684
ATP13A3	0.904584	0.030842	26.53148	0.953611
KRAS	1.254725	0.302534	5.203824	0.754541
IPO8	0.818457	0.458164	1.462077	0.498561
IMPA1	0.769706	0.426357	1.389559	0.385156
LRRCC1	0.724788	0.178534	2.942389	0.652519
E2F5	1.591623	0.800915	3.162964	0.184712
CA1	1.075718	0.928388	1.246428	0.331442
CCDC59	0.742314	0.392949	1.402293	0.358534
SWAP70	0.657699	0.373507	1.158126	0.146658
ARNTL	0.58039	0.210018	1.603922	0.294173
LYVE1	2.121704	0.880019	5.115373	0.093874
AMPD3	0.034111	0.001817	0.640229	0.023943
SBF2	19.47218	3.942986	96.16206	0.000269
MICAL2	1.750876	0.756075	4.054583	0.1911

RRAS2	2.058384	0.919692	4.606916	0.079038
HSD17B4	0.573421	0.323905	1.015146	0.056342
ZFC3H1	0.605339	0.325816	1.12467	0.112235
RNF122	0.653544	0.306622	1.392981	0.270646
DPF2	0.67238	0.266413	1.696974	0.400716
MEN1	0.962893	0.159741	5.80415	0.967091
NUMB	1.089313	0.583785	2.032602	0.788084
COX16	0.302581	0.057973	1.579291	0.156213
TTC9	0.641687	0.133086	3.093949	0.580428
MED6	0.685693	0.340517	1.380766	0.290718
EIF2S1	0.443117	0.181238	1.083396	0.074365
ADAM20	3.563762	0.140896	90.14016	0.440707
LOXL2	0.257874	0.023472	2.833173	0.26772
ELP3	0.572899	0.275917	1.189535	0.135082
ADAMDEC	1.206343	0.55864	2.605009	0.632936
MBD2	4.238095	0.300538	59.76425	0.28481
IER3IP1	0.704584	0.361482	1.373345	0.303814
MRPS36	0.413158	0.161063	1.059828	0.065905
CCNB1	0.648486	0.339008	1.240484	0.190613
CDK7	0.773217	0.329159	1.816338	0.555015
CD180	5.837082	1.916093	17.78177	0.001908
IRAK2	1.110622	0.614165	2.008388	0.728499
CAMK1	1.246299	0.929227	1.671564	0.141582
THUMPD3	1.143195	0.377287	3.463921	0.812963
VHL	0.781878	0.306069	1.997368	0.607112
ARL8B	1.082572	0.575527	2.036329	0.805586
EDEM1	0.64006	0.311909	1.313451	0.223774
MEIS2	1.622873	0.092598	28.44257	0.740347
GNAT2	0.400448	0.044715	3.586236	0.413248
GSTM1	1.011743	0.740424	1.382485	0.941574
PRPF38B	0.290878	0.114859	0.736645	0.009196
TSPAN2	1.150822	0.634708	2.086612	0.64359
TSHB	0.129345	0.006439	2.598142	0.181487
GSTM5	1.966663	1.076257	3.593716	0.027886
GSTM3	0.83815	0.220737	3.182498	0.795356
VAV3	1.2868	0.618076	2.679048	0.500332
PSRC1	2.054993	0.923312	4.573746	0.077647
PTPN22	0.38098	0.152444	0.952124	0.038928
SORT1	1.27146	0.973548	1.660535	0.077875
WNT2B	1.088187	0.02857	41.44771	0.963703
PTGFRN	1.289115	0.764237	2.17448	0.34109
NOTCH2	1.775478	0.353801	8.909885	0.48548
TRIM45	0.888096	0.266279	2.961977	0.846877
CEPT1	0.859911	0.356206	2.075896	0.73714
AP4B1	0.469018	0.237111	0.927743	0.029595
NAPG	0.932338	0.215898	4.026228	0.925216
SPIRE1	1.403504	1.029565	1.913257	0.032011
PPHLN1	0.335924	0.124394	0.907158	0.031381
FKBP11	0.846584	0.468208	1.53074	0.581555
ARF3	1.665919	0.880989	3.150195	0.116381
TMEM106	0.644786	0.371654	1.118644	0.118496
SLC38A2	0.581237	0.35413	0.953989	0.03185
YWHAQ	0.349285	0.160475	0.760244	0.008031
KIDINS220	1.158721	0.564628	2.377908	0.687954
GRHL1	0.24392	0.0026	22.88526	0.542579
ROCK2	0.239346	0.061359	0.933619	0.039507
RSAD2	0.970101	0.583224	1.613611	0.906921
MYCN	0.902225	0.705603	1.153638	0.411989

LPIN1	0.972793	0.633687	1.493364	0.899623
CMPK2	0.941482	0.379657	2.33471	0.89646
IAH1	1.301312	0.563216	3.006688	0.53764
LDHA	0.457222	0.163879	1.275647	0.134937
IL6ST	1.301807	0.082751	20.47945	0.851195
FST	0.005048	6.00E-05	0.424608	0.019348
NAV1	1.537173	0.987735	2.39224	0.056744
CDC73	0.418602	0.051134	3.42685	0.416903
TIMM17A	0.164126	0.012118	2.223	0.174102
RPS15A	1.000559	0.385137	2.599377	0.999085
RBM17	0.656702	0.289175	1.491338	0.314947
IL2RA	2.41656	1.232603	4.737747	0.010205
ANKRD16	0.787914	0.237297	2.616167	0.697054
ECHDC3	1.460048	0.93456	2.28101	0.096384
IL15RA	1.11225	0.155468	7.957262	0.915609
CCNH	0.334782	0.13352	0.839417	0.019638
HRH4	0.041613	0.001813	0.955251	0.046745
KCTD1	0.995625	0.151263	6.553278	0.996361
CABLES1	0.974876	0.66	1.439972	0.898264
DOCK2	0.544248	0.272352	1.087587	0.085021
EMP1	1.178439	0.915634	1.516673	0.202186
SOX5	0.070483	0.000674	7.366724	0.263512
KLRD1	0.982646	0.522339	1.848597	0.956699
KLRC1	1.060584	0.226206	4.972621	0.940524
PRH2	0.125204	0.007159	2.189836	0.154703
LRP4	0.978408	0.206096	4.644844	0.978087
MYBPC3	0.665971	0.169413	2.617972	0.560548
DDB2	0.57799	0.241191	1.385097	0.218925
ACP2	1.263547	0.800232	1.99511	0.315509
RAB33A	0.630778	0.414039	0.960975	0.03193
RBMX2	6.121389	1.956757	19.14975	0.001848
PIWIL4	0.551861	0.357688	0.851443	0.007213
PUM1	0.505917	0.238095	1.074999	0.076411
PHC2	3.289228	0.521226	20.75688	0.205245
CDCA8	0.669811	0.355128	1.263341	0.215752
GNL2	0.415589	0.21338	0.809422	0.009834
HOOK1	1.074243	0.425033	2.715074	0.879672
BTF3L4	0.31467	0.169251	0.585034	0.000258
PRPF38A	0.1089	0.029947	0.396009	0.000762
DSC2	1.033471	0.765735	1.394821	0.829605
RNF138	0.460757	0.245813	0.863654	0.015639
ELP2	0.440004	0.233902	0.827714	0.010882
DSC3	0.633259	0.041752	9.604759	0.741915
DTNA	2.480953	0.791764	7.773941	0.11893
DAGLA	3.479198	1.689527	7.164621	0.000717
SLC43A3	0.937886	0.236609	3.717648	0.927285
TIMM10	0.645154	0.372778	1.116547	0.117339
DHX34	0.719508	0.134147	3.859156	0.700884
APLNR	3.492663	0.574291	21.2413	0.174519
FADS2	2.254971	1.051409	4.836264	0.036731
TCN1	1.039121	0.873611	1.235987	0.664636
TMEM165	0.458722	0.186081	1.130833	0.09048
CLOCK	0.546415	0.22362	1.335165	0.18489
COL4A2	1.418416	0.307332	6.546348	0.654186
CLDN10	1.108799	0.746735	1.646416	0.608623
UBAC2	0.915452	0.34232	2.448158	0.860285
ARGLU1	0.587446	0.339275	1.017149	0.057531
BIVM	0.775577	0.513198	1.1721	0.227715

ERCC5	0.797428	0.448412	1.418094	0.440895
TPP2	0.502128	0.240201	1.049673	0.067085
CARS2	0.781035	0.368675	1.654617	0.518779
STT3A	0.848916	0.55455	1.299537	0.450882
ACRV1	0.010405	0.000321	0.336767	0.010069
ETS1	1.248551	0.824055	1.891718	0.295044
SLC37A2	0.160805	0.009832	2.630148	0.199934
KLB	0.957384	0.143617	6.38215	0.964111
TMED7	1.00433	0.562057	1.794619	0.988361
APC	1.306519	0.140032	12.19001	0.814482
WDR36	0.79158	0.376986	1.662126	0.536891
OSTF1	0.744553	0.436098	1.271182	0.279792
RFK	0.843074	0.266624	2.665821	0.77134
UBQLN1	0.416263	0.149603	1.158233	0.093226
ANXA1	0.874073	0.548201	1.393656	0.571769
AGTPBP1	0.609831	0.366247	1.015416	0.057282
GOLM1	1.750848	0.604728	5.069167	0.301772
PSAT1	0.960307	0.290366	3.175955	0.947085
ISCA1	1.163844	0.680793	1.98964	0.579178
ADAM19	2.296815	0.65042	8.110691	0.196442
HAVCR2	1.085025	0.852682	1.380679	0.506867
CCNJL	1.48293	0.405796	5.419176	0.55123
TAOK3	1.744081	0.356351	8.53602	0.492407
USP30	0.52635	0.200041	1.384933	0.193526
SDS	5.053702	0.549327	46.49305	0.152465
HNF1A	0.051598	0.005073	0.524821	0.012255
FBXO21	4.132242	1.572542	10.85848	0.003998
OASL	3.006441	1.546269	5.845483	0.001176
HRK	1.569567	0.635648	3.875642	0.328333
RNFT2	0.521487	0.15797	1.721515	0.285298
P2RX4	1.267574	0.667115	2.408497	0.469082
DTX1	2.35674	0.949675	5.848553	0.064513
TRAFD1	2.067569	0.752829	5.678368	0.158787
DMTF1	0.525742	0.287024	0.963001	0.037338
CCDC146	0.469762	0.122998	1.794152	0.269146
TMEM60	1.81406	0.90544	3.634494	0.092998
CD36	1.026047	0.779855	1.34996	0.854256
UGT2B28	0.25684	0.059444	1.109729	0.06868
PNPLA8	1.44067	0.754896	2.749424	0.268184
RINT1	0.960007	0.245918	3.747651	0.953163
SRPK2	0.420169	0.073582	2.399248	0.329337
TES	0.377972	0.092132	1.55063	0.176729
MDFIC	1.120184	0.632664	1.983377	0.697013
MTO1	0.580616	0.149026	2.262125	0.433322
ANKRD6	1.553617	0.490326	4.922699	0.453997
KHDC1	2.920547	1.055759	8.079108	0.038972
SYNCRIP	0.452731	0.234124	0.875455	0.018508
SNX14	0.99146	0.346441	2.8374	0.987244
NT5E	0.807385	0.380836	1.711683	0.576803
AKIRIN2	1.758046	0.937704	3.296059	0.078512
LCA5	0.132534	0.012149	1.445754	0.097397
MAP3K7	0.35467	0.135614	0.927562	0.034578
CGA	4.083327	0.774649	21.52402	0.097139
LMO2	1.077865	0.649747	1.788069	0.771547
PHF21A	1.000957	0.465545	2.152131	0.998046
NAT10	0.554286	0.31487	0.975747	0.040849
PRRG4	2.034831	0.766418	5.40245	0.153874
CAPRIN1	0.468885	0.180778	1.216152	0.119343

DNAJC14	0.913964	0.336906	2.479413	0.859758
CD63	0.975788	0.53826	1.768963	0.93564
AVIL	1.000598	0.105237	9.51371	0.999585
GDF11	0.393306	0.181027	0.854509	0.018418
GLS2	4.58844	0.445748	47.23245	0.200287
ITGA7	1.658698	0.289438	9.505581	0.56997
FAM186B	0.230705	0.024696	2.155239	0.198299
RDH5	0.416095	0.194817	0.888704	0.02353
AGAP2	2.462897	0.524747	11.5596	0.253224
BLOC1S1	1.553781	0.894223	2.699813	0.11797
CDK4	0.447668	0.242347	0.826939	0.010262
PPP1R1A	8.460495	0.499619	143.2692	0.139068
TROAP	0.73614	0.459292	1.179864	0.203102
TSPAN31	0.826592	0.342515	1.994811	0.671795
TFCP2	0.446467	0.206522	0.965188	0.04036
COQ10A	0.91696	0.617427	1.361806	0.667479
PAN2	0.328384	0.147633	0.730434	0.006332
ESPL1	0.76451	0.363493	1.60794	0.47902
ZC3H10	2.23027	1.272024	3.910384	0.005113
HNRNPA1	0.331153	0.159551	0.687318	0.003013
ACVR1B	0.652548	0.166098	2.563655	0.540901
OS9	3.051691	1.131595	8.229812	0.02751
KCNH3	1.122441	0.611302	2.060965	0.709484
LTV1	0.422192	0.196304	0.908012	0.027318
MAP7	1.024119	0.700858	1.49648	0.901982
CD164	1.222032	0.812556	1.837858	0.335528
AHI1	0.46264	0.256685	0.833846	0.010332
HEY2	0.225734	0.019003	2.681497	0.238487
PKIB	1.038849	0.430553	2.506558	0.932412
SMPD2	1.307686	0.56596	3.021492	0.530134
MICAL1	0.885184	0.639381	1.225483	0.462441
REPS1	0.102832	0.018736	0.564388	0.008834
STX11	1.131522	0.881231	1.452902	0.332681
TEC	0.465464	0.198795	1.089852	0.078113
SEMA4F	1.889945	0.654944	5.45373	0.239089
CCT7	0.65337	0.31086	1.373261	0.261426
EGR4	0.539563	0.080912	3.598057	0.523901
RAB11FIP5	1.349888	0.89463	2.036819	0.152873
SMYD5	1.622533	0.331688	7.937029	0.550157
DYSF	1.063003	0.854953	1.32168	0.582457
CCDC142	1.726384	0.442552	6.734581	0.43175
EMX1	2.436154	0.079491	74.66023	0.610112
KCNMB4	0.957184	0.566741	1.616616	0.870008
USP15	1.273359	0.608554	2.66442	0.521195
GNS	1.24851	0.828121	1.882306	0.289327
CPM	1.271918	0.152331	10.62012	0.824208
MDM2	1.252242	0.028839	54.37422	0.906931
KLHL36	1.028293	0.550569	1.920532	0.930247
MPHOSPH	0.610803	0.226884	1.644363	0.329235
KIAA0513	1.243599	0.783552	1.973752	0.354957
DYNC1LI2	0.957961	0.510249	1.798514	0.893693
FBXL8	1.822293	0.793865	4.183017	0.156931
FHOD1	1.215142	0.611493	2.414694	0.578103
CCDC102A	1.060868	0.707105	1.59162	0.775275
SLC9A5	0.419296	0.031635	5.557353	0.509771
AGT	2.185404	0.735749	6.491329	0.159278
KCNK1	1.111992	0.170515	7.251727	0.91165
URB2	0.702982	0.247955	1.993037	0.507431

EGLN1	0.829918	0.447366	1.539601	0.554322
COG2	0.788576	0.230818	2.694127	0.704746
ABCB10	0.554026	0.322223	0.952586	0.03271
TAF5L	0.279058	0.047233	1.648687	0.159044
GLUL	0.871772	0.111167	6.836449	0.896096
STX6	0.716211	0.332366	1.543355	0.394154
RNASEL	1.120629	0.519362	2.417983	0.77162
DHX9	0.144094	0.026672	0.778455	0.024388
KIAA1614	2.067193	0.160228	26.67008	0.577831
CEP350	0.78165	0.403933	1.512567	0.464539
NPL	1.033009	0.747912	1.426782	0.843751
PIGC	0.668453	0.291406	1.533359	0.341675
LAMC1	1.269225	1.010478	1.594229	0.040406
RC3H1	0.177659	0.01736	1.818156	0.145349
GPR55	2.578734	0.722076	9.209375	0.144678
SP110	0.919894	0.444267	1.904723	0.822099
MRPL44	1.594504	0.276926	9.180943	0.601412
CHRND	0.239287	0.016615	3.4462	0.29334
DOCK10	1.043238	0.74132	1.46812	0.808136
TTLL4	2.830442	1.097887	7.297108	0.0313
USP37	0.266715	0.078748	0.903346	0.033731
HTR2B	0.055115	0.004026	0.754457	0.02993
ITM2C	0.962111	0.707101	1.309089	0.805815
SERPINE2	1.174642	0.894608	1.542334	0.246684
DNAJB2	2.725105	1.13656	6.533923	0.024649
WNT10A	0.406269	0.053442	3.088505	0.384116
TMBIM1	1.933129	0.918124	4.070244	0.082723
CYP27A1	1.114033	0.900274	1.378548	0.320484
EIF4E2	1.428192	0.664604	3.069095	0.361154
ARMC9	0.24624	0.029139	2.080836	0.198086
CAB39	0.756021	0.421153	1.35715	0.348793
COX5B	2.45169	1.098305	5.47278	0.028609
REV1	0.206527	0.074793	0.570286	0.002337
TSGA10	0.050978	0.009776	0.265819	0.000412
MFSD9	0.343928	0.053283	2.219976	0.261957
TMEM127	1.937972	0.87196	4.307237	0.104435
EDAR	0.220156	0.020299	2.387686	0.213365
TGFBRAP1	1.051581	0.670019	1.650436	0.826887
GCC2	3.119908	0.259455	37.51649	0.369883
MRPS9	0.593812	0.259974	1.356341	0.216187
ANKRD36	0.333841	0.082026	1.358706	0.125539
EPC2	1.702335	0.334454	8.664686	0.52167
ARHGEF4	1.959243	0.047658	80.54556	0.722808
ISCU	0.896911	0.407123	1.975939	0.787176
ALDH1L2	1.285756	0.048669	33.96746	0.880398
STAB2	1.887601	0.277351	12.84667	0.516156
USP44	1.093334	0.642551	1.860367	0.742135
SCYL2	0.648337	0.330728	1.270954	0.207017
CKAP4	1.057744	0.843618	1.326218	0.626658
PLXNC1	1.011896	0.481045	2.128563	0.975135
APPL2	0.95104	0.457113	1.978672	0.893168
PWP1	0.776493	0.336791	1.790253	0.552812
DRAM1	1.272599	0.870415	1.860614	0.213555
SLC41A2	0.727214	0.128318	4.121338	0.718925
VILL	0.631947	0.238711	1.672971	0.355506
FLNB	1.171606	0.83251	1.648822	0.363631
NEK3	0.80726	0.068841	9.466269	0.864648
VPS36	0.586662	0.333117	1.033188	0.064762

RNASEH2B	0.926912	0.573059	1.499261	0.757058
CKAP2	0.508249	0.240074	1.075989	0.076965
TBC1D4	1.339264	0.572274	3.134212	0.500706
THSD1	0.228917	0.001155	45.38396	0.584849
LRCH1	8.684563	0.273032	276.2371	0.220749
SUCLA2	0.63712	0.273674	1.483229	0.295749
RCBTB1	0.486583	0.159768	1.48192	0.204894
MED4	0.308945	0.099686	0.957483	0.041826
PHF11	1.453341	0.479589	4.404184	0.50866
COG3	0.534919	0.171877	1.664787	0.280116
LMO7	0.232789	0.019416	2.790984	0.250099
ITM2B	1.881212	0.595533	5.942512	0.281577
SPRY2	0.842815	0.623798	1.13873	0.265361
NUDT15	0.690519	0.240444	1.983064	0.491459
EDNRB	1.154482	0.0088	151.466	0.95396
RCBTB2	1.451013	0.836274	2.517641	0.185494
LCP1	0.899802	0.494284	1.638013	0.729772
SETDB2	0.510657	0.25694	1.01491	0.055145
SCRN1	1.22857	0.981885	1.53723	0.071845
TNS3	1.651618	1.235688	2.207549	0.0007
SPDYE1	1.164277	0.27906	4.85752	0.834683
CHST12	0.776652	0.540631	1.115713	0.171451
IGF2BP3	1.045383	0.587731	1.859395	0.879931
GPNMB	2.079526	1.04756	4.128097	0.036369
RAPGEF5	1.897369	0.140451	25.63179	0.629677
RAC1	8.333495	1.635202	42.47007	0.010716
KDELR2	0.977834	0.444295	2.152084	0.955587
IL6	1.408752	0.827365	2.398679	0.206926
ZDHHC4	0.270037	0.055647	1.3104	0.104265
AOAH	1.102942	0.767613	1.584759	0.596226
BZW2	0.513239	0.306481	0.859481	0.011225
TBRG4	1.314962	0.603341	2.865917	0.490931
DDX56	1.134367	0.502825	2.559118	0.761343
HUS1	0.007525	0.000243	0.233156	0.005252
NACAD	1.959609	0.164317	23.36982	0.594758
DBNL	1.495993	0.733825	3.049768	0.267708
CCM2	1.526793	0.644156	3.618842	0.336512
MYO1G	1.462821	0.92628	2.310152	0.102786
TTYH3	1.973259	1.2492	3.116996	0.00357
TTC5	0.910308	0.427233	1.939597	0.807632
ZFHX2	1.633146	0.393866	6.771757	0.499069
MTHFS	0.668304	0.2949	1.51451	0.334288
ADAMTS7	2.691805	0.148792	48.69756	0.50267
IREB2	0.444484	0.20505	0.963504	0.039961
ALPK3	0.940888	0.094766	9.341648	0.958507
TM6SF1	1.081485	0.729067	1.604256	0.697009
CIB2	2.047751	1.234674	3.396268	0.005493
CALCOCO	0.727373	0.41734	1.267722	0.261423
RSAD1	0.294176	0.109957	0.787026	0.014812
NMT1	0.748597	0.339422	1.651033	0.473061
MYCBPAP	1.183754	0.198796	7.048792	0.852984
VEZF1	0.887754	0.521657	1.510779	0.660738
CHAD	0.13148	0.019262	0.897454	0.038418
TACO1	1.797	0.702763	4.595016	0.221107
TEX2	0.748499	0.415007	1.34998	0.335701
DCAF7	1.063621	0.600687	1.883325	0.832435
LIMD2	1.57714	0.283626	8.769885	0.602732
BRIP1	0.509378	0.135626	1.9131	0.317737

RTP4	1.185325	0.647283	2.170605	0.581772
ACTL6A	0.906992	0.513823	1.601009	0.736339
NDUFB5	0.60456	0.247568	1.476336	0.269258
MRPL47	0.86431	0.424314	1.760561	0.687887
ERMN	0.075641	0.002655	2.154716	0.130851
GALNT5	3.509951	0.367317	33.53983	0.275584
TANK	2.131424	0.791161	5.742157	0.134475
BLK	1.160878	0.635193	2.121621	0.627765
SKIL	3.395398	0.158177	72.88492	0.434612
HLX	1.814984	1.223124	2.69324	0.003075
VPS45	0.888523	0.415972	1.897898	0.760185
IL10	0.866305	0.202486	3.706355	0.846554
KCTD3	0.830592	0.403852	1.708257	0.613899
RPS6KC1	1.012316	0.463717	2.209933	0.975485
CBWD2	3.019732	0.184461	49.43474	0.438427
IL1RN	1.438575	0.945136	2.189629	0.089754
SMPD4	0.319935	0.088659	1.154519	0.081767
WDR33	0.461607	0.118794	1.793706	0.264309
CCDC115	0.94119	0.363505	2.436938	0.900627
SAP130	0.73896	0.297882	1.83315	0.514019
BIN1	1.427744	1.024669	1.989376	0.035383
IMP4	0.953894	0.436372	2.08518	0.905831
HS6ST1	1.31124	0.340674	5.046913	0.693546
GYPC	0.867162	0.608207	1.23637	0.430957
STAM	0.476187	0.275613	0.822726	0.007828
ABI1	0.36706	0.06489	2.076319	0.256962
YME1L1	0.715426	0.311637	1.642408	0.429651
DNAJC1	1.436134	0.79465	2.595458	0.230634
NIPSNAP3	0.972108	0.456232	2.071302	0.941573
LRRC8A	1.475681	0.706786	3.08104	0.300195
CDK9	0.7114	0.29556	1.712308	0.447355
TXN	0.775806	0.433262	1.389171	0.393071
ODF2	0.247798	0.049482	1.240926	0.089631
TOR1B	2.340924	0.732353	7.482623	0.151406
SMC2	0.363043	0.124938	1.054922	0.062642
KLF4	1.296729	1.034678	1.625149	0.024075
TOR1A	0.666406	0.279725	1.587617	0.35949
RALGPS1	0.695841	0.163498	2.961485	0.623611
ST6GALNA	1.718513	1.120677	2.635271	0.013054
TMOD1	0.831548	0.598988	1.154401	0.270411
DAB2IP	0.008315	0.000104	0.661898	0.031974
STXBP1	0.889357	0.072068	10.97516	0.92713
SLC2A8	1.284984	0.871576	1.894481	0.205518
ANGPTL2	0.20191	0.024891	1.637843	0.13413
CDK5RAP2	0.330215	0.130941	0.832753	0.018887
SLC31A2	1.149248	0.760709	1.736238	0.508753
SLC31A1	1.440921	0.782271	2.654134	0.241171
TLR4	1.448326	1.061713	1.975721	0.019391
ZNF189	1.265978	0.316733	5.060106	0.738665
STX17	0.827129	0.132683	5.156225	0.838921
PRPF4	0.633331	0.286314	1.40094	0.259473
FPGS	0.49549	0.069115	3.55219	0.484733
USP20	1.004422	0.135337	7.454449	0.996558
ATP6V1G1	0.22584	0.070007	0.728555	0.012777
TEX10	0.475677	0.243248	0.930198	0.029899
GARNL3	1.983122	0.394023	9.981084	0.406316
MRPL50	2.119234	1.087357	4.130339	0.027388
DPM2	1.067979	0.517762	2.202905	0.85869

WDR38	1.33184	0.013772	128.7966	0.902222
TSTD2	0.554256	0.123844	2.480536	0.440229
HEMGN	1.069684	0.754553	1.516426	0.705195
PSMB7	0.515037	0.276032	0.960987	0.037068
NR5A1	1.499234	0.43525	5.164164	0.521042
RABEPK	0.673618	0.311166	1.458261	0.316041
GOLGA1	0.597361	0.220593	1.617642	0.310731
XPA	1.211	0.373559	3.925802	0.749698
NCBP1	0.503213	0.15753	1.60746	0.246481
ANP32B	0.643921	0.259078	1.600421	0.343335
PDCL	0.619323	0.207703	1.846677	0.390036
RPL35	0.909382	0.28955	2.856074	0.870771
LMX1B	2.110209	0.286937	15.51901	0.463211
ARPC5L	1.442622	0.590508	3.524351	0.421337
ENPP2	1.355208	0.842165	2.180795	0.210477
DSCC1	0.23956	0.087313	0.65728	0.005522
DERL1	1.030109	0.542423	1.956268	0.927769
MYC	1.003849	0.723063	1.393673	0.981691
RANBP6	0.736649	0.255856	2.120926	0.571067
POLR1E	0.460117	0.233081	0.908304	0.025278
PLAA	0.392385	0.052734	2.919688	0.360931
IL11RA	0.508049	0.171609	1.504078	0.221382
UBAP2	0.603973	0.26088	1.398283	0.239097
APTX	0.466482	0.11413	1.906652	0.288439
RNF38	0.478248	0.153325	1.491742	0.20377
TLN1	1.29606	0.725968	2.313839	0.3805
SIT1	1.845912	0.538515	6.327377	0.329443
DNAJB5	8.617695	0.058447	1270.624	0.397894
SPAG8	0.10241	0.003851	2.723716	0.1734
DCTN3	1.56278	0.284276	8.591222	0.607637
CD72	1.370266	0.635983	2.952329	0.421203
TMEM8B	4.60879	0.903392	23.51243	0.066097
GRHPR	1.488822	0.709566	3.123867	0.292544
ALDH1B1	1.009675	0.435425	2.34126	0.982099
DENND4C	0.472741	0.233736	0.956137	0.03709
RPS6	0.825743	0.204782	3.329647	0.787819
CNPY3	1.440642	0.657043	3.158772	0.362067
FOXP4	1.051208	0.153444	7.201569	0.959434
PPIL1	0.642076	0.326132	1.264097	0.199882
KLC4	0.029188	0.001325	0.643004	0.0251
KIF13A	0.257289	0.034996	1.891564	0.182289
PIM1	1.466891	1.025207	2.098864	0.036068
GMPR	0.977066	0.712845	1.339223	0.885324
YIPF3	1.667747	0.485955	5.723541	0.416242
TMEM14B	0.689151	0.320892	1.480028	0.33976
TMEM63B	1.495411	0.767079	2.915286	0.237427
FRS3	1.333191	0.597941	2.972535	0.482099
TJAP1	0.795518	0.280666	2.254814	0.666932
CAPN11	1.450876	0.973958	2.161326	0.06722
IRF4	2.016197	1.151849	3.529153	0.014093
SLC22A23	1.693198	0.619331	4.629056	0.304765
TUBB2A	1.136961	0.823082	1.570536	0.436133
LRRC1	1.285472	0.168827	9.787766	0.808423
GCM1	0.839918	0.109381	6.449574	0.866793
BPHL	0.477567	0.192467	1.184982	0.110956
RIPK1	0.920852	0.390845	2.169573	0.850421
TUBB2B	0.30104	0.045448	1.994037	0.213312
HMGA1	2.560686	0.511733	12.81354	0.252416

TCF19	0.28973	0.045554	1.842743	0.189386
FLOT1	1.221567	0.753113	1.98141	0.417369
IER3	1.213443	0.993522	1.482045	0.057925
MDC1	0.393828	0.180145	0.860979	0.019541
PGBD1	0.378347	0.179196	0.798824	0.010802
TPMT	0.683419	0.336726	1.387067	0.291888
RNF144B	1.001241	0.693951	1.444602	0.994711
NRM	1.265276	0.564701	2.834991	0.567574
MTCH1	2.471616	0.909833	6.714292	0.075957
VARS2	0.351017	0.142721	0.86331	0.022603
TAF8	0.391849	0.145264	1.05701	0.064248
FAM8A1	0.322447	0.133944	0.776239	0.011568
FGFBP2	0.979427	0.750081	1.278899	0.878622
CPEB2	1.094586	0.250363	4.785524	0.904425
FHDC1	0.984303	0.646236	1.499223	0.94125
TLR2	1.891208	0.76567	4.671293	0.167213
MYO7A	1.620607	0.99349	2.643578	0.053137
FCHSD2	0.422223	0.190208	0.937246	0.034069
ARRB1	2.690071	1.33251	5.430714	0.005765
SLCO2B1	2.256938	0.962072	5.294584	0.061333
ANKRD42	1.168934	0.084413	16.18715	0.907329
IL18BP	1.166816	0.222279	6.125005	0.855296
NUMA1	2.331211	0.882356	6.159135	0.087735
CCDC90B	0.802627	0.33368	1.93062	0.623447
SYTL2	1.34644	0.231411	7.834114	0.740593
RAB30	1.274716	0.407171	3.990707	0.676787
CREBZF	0.606768	0.34145	1.078249	0.088542
LRRC32	1.753905	1.064855	2.888827	0.027329
PRCP	1.316763	0.728573	2.379809	0.362148
NARS2	0.357197	0.164739	0.774494	0.00913
RNF121	0.457393	0.144907	1.443744	0.182278
MRPL15	1.08333	0.555968	2.110922	0.814081
PI15	0.744037	0.369567	1.497946	0.407597
GGH	0.628656	0.365571	1.081074	0.093321
SLCO5A1	1.255555	0.470217	3.352538	0.649717
TGS1	0.222	0.019216	2.564726	0.227991
SDCBP	1.195167	0.768184	1.859482	0.429204
NEK1	0.473275	0.219847	1.01884	0.055844
DDX60	1.067325	0.587254	1.939846	0.830747
SORL1	0.868107	0.632956	1.190619	0.380208
BUD13	0.83825	0.237197	2.962367	0.78414
TRPC6	0.866446	0.06567	11.43177	0.91327
MMP7	1.005315	0.647177	1.561643	0.981179
DCUN1D5	0.524125	0.201087	1.366114	0.186267
SLC37A4	0.515019	0.291874	0.908766	0.022013
RDX	1.060498	0.546252	2.058859	0.862231
PPP2R1B	0.278624	0.031936	2.430881	0.247576
FDX1	0.708155	0.263204	1.905304	0.494364
FXYD6	1.311019	0.857818	2.003654	0.21082
TMPRSS13	1.131326	0.174766	7.323509	0.896973
CASP1	1.616027	0.979797	2.665392	0.060106
CASP5	1.379416	0.744542	2.555652	0.306605
ALKBH8	0.679728	0.228951	2.018033	0.486836
MAP2K5	1.924056	0.463565	7.985921	0.367467
CTDSPL2	0.893881	0.422679	1.890375	0.769083
SLTM	0.342302	0.167713	0.698637	0.003228
THBS1	1.461141	1.074024	1.987787	0.015749
MAPKBP1	2.545724	1.385811	4.676474	0.002599

NUSAP1	0.718686	0.464511	1.111943	0.137955
NDUFAF1	0.809167	0.409711	1.598082	0.541975
KIF23	0.5288	0.191841	1.457612	0.218096
ITGA11	0.269699	0.01504	4.836116	0.373578
RTF1	0.658454	0.21095	2.055278	0.47183
PARP6	0.397999	0.066298	2.389262	0.313696
RPLP1	1.530066	0.402978	5.809499	0.532105
PAQR5	0.283323	0.01135	7.07211	0.442314
TUBGCP4	0.375159	0.161358	0.87225	0.022758
ITPKA	0.756542	0.462058	1.23871	0.267419
UACA	0.129163	0.005731	2.911261	0.197863
SMAD6	3.13754	1.560749	6.307329	0.00133
PLCB2	1.393383	0.80806	2.40269	0.232742
TMEM62	1.044345	0.367837	2.965053	0.935046
PAK6	0.655103	0.29283	1.465561	0.303223
ADAM10	1.016771	0.529921	1.950898	0.960105
DUOX1	0.149686	0.004071	5.503794	0.301761
ZNF280D	0.661801	0.316948	1.381869	0.271804
BCL2L10	0.351167	0.088344	1.395878	0.137205
RSL24D1	0.700783	0.35358	1.388928	0.308347
SPTBN5	0.448308	0.0521	3.857611	0.46504
GCHFR	0.921826	0.64142	1.324816	0.660012
BCAR3	1.391462	0.359492	5.385848	0.632361
TTLL7	0.705767	0.192328	2.589885	0.599346
FNBP1L	1.062563	0.57144	1.975779	0.847938
GTF2B	0.746786	0.378483	1.473487	0.399761
RABGGTB	0.462829	0.255542	0.83826	0.011018
IFI44L	1.392354	1.013417	1.912984	0.041133
IFI44	1.419977	0.893058	2.257787	0.138355
SLC44A5	28.91687	3.043896	274.709	0.0034
DBT	0.324805	0.07155	1.474459	0.145142
IFT172	0.295544	0.098655	0.885375	0.029448
CGREF1	2.854187	0.385423	21.13622	0.304579
HADHB	0.478492	0.193613	1.182536	0.110317
KHK	1.860506	0.142998	24.20651	0.635315
ADCY3	0.901551	0.682115	1.191578	0.466446
PPM1B	0.192383	0.046424	0.797236	0.023064
PNPT1	0.386744	0.13408	1.115533	0.078802
DYNC2LI1	0.798639	0.153181	4.163852	0.789564
THUMPD2	0.498314	0.240186	1.033854	0.061407
CYP1B1	1.291414	1.030668	1.618126	0.026251
RAB1A	1.462887	0.513805	4.16508	0.476104
ACTR2	0.884614	0.513591	1.52367	0.658527
PREB	1.023754	0.424318	2.470012	0.958336
SLC5A6	0.890929	0.461081	1.72151	0.731113
PREPL	0.439815	0.168571	1.147514	0.093201
SLC3A1	0.3997	0.043211	3.697184	0.419121
EMILIN1	1.220157	0.759648	1.959833	0.410521
FBXO11	0.443976	0.128498	1.533988	0.199287
SIX3	0.079065	0.006732	0.928634	0.043501
CENPO	0.392078	0.068216	2.253501	0.294012
LRPPRC	0.426993	0.217466	0.838399	0.013437
DTNB	0.546766	0.075421	3.963805	0.550279
ACTR1A	0.854285	0.359975	2.027371	0.720966
CYP2C8	0.513697	0.223594	1.180195	0.116514
MYOF	1.360517	1.058082	1.749396	0.01639
LOXL4	0.786934	0.424573	1.45856	0.446613
STAMBPL1	0.388698	0.211643	0.713872	0.002314

CH25H	2.682469	1.150691	6.25332	0.022313
ATAD1	0.699013	0.328686	1.486581	0.352309
KIF11	0.644606	0.378142	1.098839	0.106612
DUSP5	1.350242	0.981298	1.857899	0.065179
CALHM2	2.571004	1.539292	4.294222	0.000309
ARL3	1.247521	0.510655	3.047671	0.627477
CEP55	0.909115	0.541358	1.526698	0.718658
KIF20B	0.83887	0.426932	1.648279	0.61016
ENTPD1	1.052217	0.756807	1.462939	0.7621
EXOC6	0.235438	0.046897	1.181983	0.07894
PLCE1	0.07261	0.003977	1.325827	0.076784
DBR1	0.869181	0.22962	3.290112	0.836449
DNAJC13	0.709088	0.362819	1.38583	0.314636
GPR87	1.424765	0.110666	18.34314	0.785979
ANXA7	0.273239	0.066305	1.126006	0.072541
FAM149B1	0.535894	0.267189	1.074829	0.078961
ASCC1	0.961536	0.427155	2.164441	0.924516
ADAMTS1	1.616773	0.109379	23.89813	0.726631
RPS24	0.767485	0.348018	1.692536	0.51193
TET1	1.840325	0.29194	11.60098	0.516142
DNA2	0.17269	0.037171	0.80228	0.025021
AOX1	0.605482	0.067177	5.457386	0.654693
ATIC	0.312952	0.150938	0.64887	0.001793
SMARCAL	0.334578	0.139576	0.802015	0.014105
BARD1	0.463882	0.255081	0.843601	0.011824
STAT4	1.244888	0.885834	1.749478	0.207056
ASNSD1	0.910492	0.500307	1.656974	0.758886
METTL5	0.69113	0.317185	1.505938	0.352544
SSB	0.958714	0.344594	2.667286	0.935631
NAB1	1.18803	0.548221	2.574536	0.662364
PPIG	1.070256	0.393787	2.908797	0.894116
FASTKD1	0.539305	0.262072	1.10981	0.093543
IDH1	1.204227	0.742689	1.952583	0.451078
OLA1	0.525707	0.038952	7.094987	0.62819
CIR1	0.959971	0.437222	2.107728	0.918909
FAM117B	1.49432	0.797137	2.801265	0.210278
WDR12	0.275836	0.133434	0.570209	0.000509
ABI2	5.036885	0.912119	27.81459	0.063674
ITGAV	1.520192	0.901452	2.563621	0.116216
SLC40A1	0.813741	0.537312	1.232384	0.330413
SLC35A5	0.857945	0.46645	1.578023	0.622165
SENP7	0.280557	0.09943	0.791631	0.01633
COX17	1.799452	0.746169	4.339539	0.190862
PARP9	1.403329	0.855749	2.301298	0.179373
MNS1	0.378894	0.148394	0.967426	0.042437
USP8	1.235221	0.555296	2.747673	0.604546
SECISBP2L	0.769672	0.33647	1.760614	0.535194
TMOD3	1.27769	0.209263	7.80115	0.790646
SPPL2A	0.850683	0.503452	1.437399	0.54568
GLCE	1.048919	0.592865	1.855787	0.869677
SHF	2.311376	0.684135	7.809075	0.177387
APH1B	1.093104	0.58472	2.043502	0.780339
PARP16	0.644297	0.184573	2.249081	0.490692
PPCDC	0.757373	0.132607	4.325671	0.754595
SEMA7A	4.562436	1.13429	18.35142	0.032565
UBL7	0.924117	0.396989	2.151176	0.854751
ARHGAP2	1.165394	0.461738	2.941376	0.745916
FAM13A	1.653399	0.235277	11.61918	0.613244

HERC3	1.109243	0.485104	2.536402	0.80592
HERC6	1.40493	0.636696	3.100112	0.399815
HERC5	0.667467	0.45699	0.974882	0.036476
PCDH10	0.570613	0.030807	10.56894	0.706383
COPS4	0.930463	0.436916	1.981531	0.851766
HNRNPD	0.313247	0.132246	0.741979	0.008333
PRKG2	2.74467	0.287158	26.23368	0.380686
RASGEF1B	0.496004	0.112478	2.187272	0.354359
SEC31A	0.820889	0.308881	2.181612	0.692279
BBS7	0.801025	0.114156	5.620747	0.823389
KIAA1109	0.255444	0.033177	1.966759	0.190035
BMPR1B	5.617042	0.501355	62.93175	0.161542
RAP1GDS1	0.333071	0.096087	1.154541	0.083026
LARP1B	0.093342	0.01957	0.445202	0.002928
MMRN1	1.04392	0.704679	1.546477	0.830261
PDE5A	1.866306	0.236233	14.74433	0.554065
PRDM5	0.111926	0.007765	1.613407	0.107705
NAAA	2.162859	1.282632	3.647158	0.003808
NUP54	0.574434	0.26618	1.239664	0.157789
CXCL9	0.969783	0.482811	1.947925	0.931287
BMP2K	0.389015	0.131277	1.152775	0.088484
G3BP2	0.706273	0.306602	1.626937	0.414041
FRAS1	0.456298	0.00439	47.42635	0.740529
SCARB2	1.555127	0.937235	2.580377	0.087438
CCNG2	1.849125	0.747388	4.574952	0.183522
CNOT6L	0.470311	0.090965	2.431619	0.368155
USO1	0.678857	0.377796	1.219829	0.195179
ANXA3	0.989554	0.811359	1.206885	0.917438
PPA2	0.534036	0.164087	1.738068	0.297474
CENPE	0.736946	0.368558	1.473549	0.387919
GSTCD	0.000726	7.38E-06	0.07131	0.002014
INTS12	0.606176	0.291936	1.258663	0.179322
CASP6	0.635681	0.418121	0.966443	0.034035
LEF1	0.774208	0.544993	1.099827	0.153079
HADH	0.373641	0.202972	0.687818	0.001567
EGF	0.592254	0.121033	2.898087	0.517911
PAPSS1	0.866655	0.510838	1.470313	0.595658
SEC24B	0.707396	0.080212	6.238572	0.755295
PPP3CA	0.65627	0.276223	1.559213	0.340115
SLC39A8	0.502707	0.316969	0.797283	0.00347
FBN2	1.086331	0.707772	1.667366	0.70483
MAPK8IP3	1.192116	0.489433	2.903651	0.698837
RGS3	0.034664	0.000116	10.32746	0.247399
TTLL8	16.82662	0.966117	293.0648	0.052828
RNF185	4.449136	0.894941	22.11856	0.068105
PARVG	1.15638	0.539063	2.480625	0.709059
B4GALNT3	5.06342	0.618827	41.43033	0.130417
PDE6H	1.135962	0.481099	2.682213	0.771194
ERP27	0.775899	0.359414	1.675001	0.518127
ETV6	0.752131	0.472536	1.19716	0.229697
GABARAPI	1.135043	0.756387	1.70326	0.54074
KIF21A	0.981141	0.382952	2.513725	0.96836
CPNE8	1.413857	1.063435	1.87975	0.017163
YARS2	1.378285	0.576355	3.296007	0.470758
FGD4	0.745079	0.383793	1.446464	0.384629
ALG10	1.872388	0.374321	9.365853	0.445094
AEBP2	1.328416	0.393571	4.483783	0.647273
ETNK1	0.518658	0.197628	1.361176	0.182335

ZCRB1	0.802905	0.263199	2.449311	0.699674
TMEM117	0.398029	0.032144	4.928625	0.473031
PRICKLE1	1.165928	0.895517	1.517993	0.254168
C1RL	1.021227	0.536739	1.943042	0.948968
NDUFA9	0.934831	0.317977	2.748342	0.902519
CLSTN3	2.728495	1.262981	5.894535	0.010648
KLRG1	0.769966	0.283331	2.092421	0.608308
VAMP1	0.643742	0.215709	1.921128	0.429785
TAPBPL	0.877588	0.499946	1.540489	0.649225
CD27	1.190724	0.852054	1.664007	0.306632
RBP5	0.900944	0.068561	11.83917	0.936735
PEX5	0.334092	0.141654	0.787958	0.012268
SLC38A4	2.686346	0.540424	13.35334	0.227126
AMIGO2	0.184472	0.028656	1.187547	0.075232
COL2A1	2.03654	0.27129	15.28807	0.489224
LLPH	0.515876	0.249464	1.0668	0.074176
INHBE	0.697675	0.087553	5.559497	0.733887
GLIPR1	1.032585	0.708303	1.505333	0.867585
PHLDA1	1.217908	0.885782	1.674565	0.224969
TMEM19	1.768764	0.789042	3.964968	0.166153
DUSP6	1.821451	1.268875	2.614666	0.00115
TMTC3	0.091138	0.006533	1.271369	0.074846
LUM	1.024854	0.717143	1.464596	0.892794
SNRPF	0.546635	0.278743	1.071987	0.078804
AMDHD1	1.116508	0.465713	2.676736	0.804886
NEDD1	2.090719	0.493939	8.849492	0.316427
SYCP3	1.507399	0.017278	131.5123	0.857157
GAS2L3	1.646586	0.48965	5.537114	0.420266
SLC15A4	1.99717	0.847869	4.704368	0.11355
TDG	0.444991	0.179448	1.103475	0.080557
SDSL	1.42628	1.006886	2.020362	0.045653
MMAB	0.424465	0.040164	4.485837	0.476267
GLTP	1.086436	0.579623	2.036398	0.795928
GIT2	0.444949	0.192472	1.028612	0.058227
TCHP	0.290124	0.068419	1.230236	0.093182
MTMR6	0.874532	0.474392	1.612181	0.667488
SLC46A3	0.950943	0.467207	1.935531	0.889669
SLC7A1	0.615398	0.362817	1.043819	0.071722
LNX2	1.069913	0.495238	2.31144	0.86348
SUOX	0.757488	0.24798	2.313848	0.625902
CCDC65	1.456042	0.470011	4.51066	0.514874
SLC39A5	0.526635	0.071598	3.873612	0.528793
TARBP2	0.707957	0.322372	1.554734	0.389526
DHH	0.709431	0.08119	6.198928	0.756258
ACVRL1	1.370313	0.566954	3.312013	0.484137
GPR84	1.10365	0.710039	1.715458	0.661199
N4BP2L1	1.156697	0.535989	2.496222	0.710704
SMARCC2	1.049714	0.35586	3.096442	0.929949
BRCA2	0.378998	0.063721	2.254186	0.286193
MAP3K12	1.845841	0.548116	6.216068	0.322468
ITGB7	1.204961	0.86951	1.669827	0.262708
GALNT6	1.14261	0.418619	3.118725	0.794691
CSAD	1.069201	0.319507	3.577983	0.913541
LMBR1L	3.55627	0.761821	16.60108	0.106548
ESYT1	0.491855	0.242326	0.998334	0.049463
TMBIM6	1.043462	0.435713	2.498922	0.923933
ANKRD52	1.542131	0.372161	6.390161	0.550368
ZNF740	0.436395	0.115659	1.646572	0.220991

WDFY2	0.547682	0.268313	1.11793	0.098178
HNRNPA1	0.186769	0.055851	0.624569	0.006446
ESD	0.726865	0.376668	1.402649	0.341534
RB1	1.091077	0.578238	2.058751	0.787877
SBNO1	0.219543	0.060272	0.799693	0.021512
MORN3	0.856632	0.097298	7.541926	0.889108
SETD1B	3.106403	1.162492	8.300908	0.023809
VPS33A	0.216246	0.0595	0.785917	0.020026
VPS37B	1.477213	0.87542	2.492701	0.143866
RHOF	1.711578	0.80303	3.648058	0.163971
DENR	0.5615	0.252622	1.24804	0.156704
DIAPH3	0.422057	0.073253	2.431745	0.334326
SLAIN1	0.576292	0.328462	1.011114	0.054677
RBM26	0.291895	0.09092	0.937119	0.038537
MBNL2	0.879226	0.349028	2.214829	0.78481
ABHD13	4.781569	0.627922	36.4112	0.130864
RAB20	2.007154	1.177849	3.42036	0.010411
GRTP1	0.325292	0.017673	5.987528	0.449836
CUL4A	0.206488	0.039489	1.079723	0.061611
CDH24	0.209603	0.005042	8.714128	0.411302
REM2	1.22437	0.381789	3.926464	0.733507
CBLN3	1.292925	0.342573	4.879699	0.704604
TSSK4	0.307824	0.02123	4.463368	0.387827
TMX1	0.409191	0.135888	1.232173	0.112115
FRMD6	0.152184	0.015204	1.523307	0.109187
PELI2	0.953	0.562349	1.615026	0.858038
RTN1	2.582165	1.073982	6.208278	0.034055
SLC38A6	1.45637	0.587113	3.612616	0.417327
RDH12	0.438618	0.024998	7.695976	0.572874
RAB15	1.974748	0.958811	4.067151	0.064912
WDR89	0.259664	0.023361	2.88625	0.272493
ESR2	1.331274	0.102404	17.30678	0.826926
STON2	3.414538	0.456374	25.54718	0.231699
GPR65	1.275615	0.869656	1.871077	0.21297
PTGR2	1.432387	0.705651	2.907577	0.319832
JDP2	1.081739	0.565642	2.068727	0.81226
AK7	0.064519	0.003979	1.046061	0.05382
SLC24A4	1.284064	0.644561	2.558051	0.477068
FBLN5	0.904837	0.333556	2.454547	0.844296
WDR20	3.845056	0.805529	18.35372	0.091259
NIPA2	0.349926	0.107329	1.140873	0.081615
SLC12A6	0.087926	0.011715	0.659937	0.018074
MFAP1	1.357469	0.624139	2.95242	0.440754
TCF12	0.696272	0.262777	1.844887	0.46652
SORD	0.712784	0.364184	1.395068	0.323053
SERF2	0.789458	0.231164	2.696113	0.705983
ZSCAN29	0.202291	0.036851	1.110466	0.065862
LYSMD2	1.294924	0.688187	2.436589	0.422942
SLC27A2	0.549712	0.369028	0.818861	0.003253
HDC	0.830093	0.680764	1.012178	0.065719
BNIP2	0.701351	0.407938	1.205804	0.199466
GTF2A2	0.451279	0.179104	1.137065	0.091501
SRP14	0.400814	0.135933	1.181846	0.097493
BAHD1	2.208531	0.668519	7.296141	0.19377
DISP2	0.272126	0.019551	3.787669	0.332685
CDAN1	0.043559	0.004986	0.380568	0.004604
TLE3	1.624619	0.882078	2.992237	0.119399
ANP32A	1.33823	0.737767	2.427406	0.337584

COMMD4	0.870985	0.18752	4.045514	0.860069
UBE2Q2	0.617382	0.321266	1.186433	0.147889
PSTPIP1	1.220219	0.877142	1.697484	0.237333
ETFA	0.471551	0.199585	1.114114	0.086596
BCL2A1	1.267961	0.953217	1.68663	0.102925
HMG20A	1.14681	0.308402	4.26448	0.838019
SCAPER	1.441458	0.437089	4.753723	0.548112
TSPAN3	0.639935	0.369542	1.108172	0.111083
WDR61	0.315356	0.128001	0.776943	0.012122
NCOA2	1.143963	0.212894	6.14697	0.875421
NEIL1	0.456679	0.047182	4.420246	0.498573
MAN2C1	1.127703	0.576889	2.204431	0.725272
DNAJA4	0.870122	0.622602	1.216046	0.415291
TPM1	0.960892	0.512195	1.802658	0.901096
IGF1R	0.779971	0.215829	2.818687	0.704618
ARRDC4	1.032684	0.56068	1.902038	0.9178
PIF1	1.392349	0.429664	4.511979	0.58111
USP3	1.012922	0.630142	1.628222	0.957718
BBS4	0.415002	0.182661	0.942874	0.035689
PML	0.326891	0.020263	5.27346	0.430653
CYP1A1	0.100125	0.013169	0.761253	0.02618
ADAMTS1	0.410906	0.03032	5.56874	0.503646
LINS1	0.915792	0.132375	6.335615	0.928971
ULK3	1.938688	0.685698	5.481291	0.211878
PCSK6	1.703131	0.1202	24.13191	0.693832
SCAMP2	1.953505	0.795855	4.795072	0.143858
HAPLN3	1.288966	0.559294	2.970592	0.551251
POLG	0.796636	0.291224	2.179184	0.657897
RLBP1	3.796986	0.322734	44.67181	0.288784
FANCI	0.356367	0.195725	0.648859	0.000739
ABHD2	0.748394	0.135658	4.128726	0.739421
DET1	0.649886	0.256665	1.645536	0.363248
MFGE8	1.245753	0.899237	1.725796	0.186395
ZNF710	0.604147	0.042886	8.510828	0.708864
UNC45A	1.403873	0.275258	7.160048	0.68321
MCTP2	0.413382	0.213356	0.800936	0.008851
FURIN	1.75403	0.909218	3.383811	0.093721
IQGAP1	1.137603	0.751182	1.722804	0.542634
CRTC3	0.95755	0.557121	1.645783	0.875262
SEC11A	0.889018	0.412313	1.916876	0.764111
PMM2	1.292438	0.514176	3.248683	0.585416
ITGAX	1.001566	0.593316	1.690724	0.995326
TGFB1I1	1.405161	0.552267	3.575219	0.475294
ARMC5	0.934966	0.538718	1.62267	0.811059
PARN	0.279198	0.119773	0.65083	0.00313
FTO	0.940072	0.738298	1.196991	0.616149
UQCRC2	0.799425	0.361438	1.768158	0.580443
CDR2	0.595035	0.290366	1.219381	0.156149
IGSF6	1.2902	0.914451	1.820346	0.146847
ARHGAP1	1.688674	0.48938	5.827005	0.407038
NKD1	0.131974	0.015933	1.093129	0.060461
DHX38	1.081285	0.377268	3.09906	0.884341
TXNL4B	0.494463	0.09716	2.516417	0.396242
ZFHX3	1.275787	0.854912	1.903861	0.233072
CPNE2	2.644434	1.151322	6.073911	0.0219
NLRC5	1.441432	0.836058	2.485149	0.188287
KATNB1	0.67262	0.342466	1.321059	0.249526
KIFC3	1.161837	0.771108	1.750551	0.473255

ADAMTS1	0.070253	0.002787	1.770983	0.106776
NUDT7	0.972644	0.63875	1.481075	0.897134
GCSH	0.591811	0.18422	1.901212	0.378339
CMTM3	2.033939	0.445385	9.288383	0.359559
CMTM2	0.981521	0.727367	1.32448	0.902905
NOL3	6.034407	1.145825	31.77978	0.033959
MAP1LC3I	0.843872	0.436037	1.633164	0.61433
MBTPS1	0.389781	0.146467	1.037291	0.059207
ZCCHC14	1.479021	0.777795	2.812443	0.232636
OSGIN1	5.308147	0.200198	140.743	0.318203
IRF8	1.240749	1.002532	1.535569	0.047344
RHOT2	1.25787	0.550563	2.873851	0.586289
RPL3L	0.365311	0.048303	2.762813	0.32931
RPS2	2.256619	0.740124	6.880376	0.152465
NDUFB10	0.954618	0.403583	2.258016	0.915795
PDPK1	0.319628	0.052646	1.940549	0.215159
TIGD7	0.606732	0.281014	1.309982	0.203236
DEF8	1.92062	0.976234	3.778585	0.058718
TCF25	0.564539	0.215902	1.476156	0.243675
GALNS	0.831372	0.362295	1.90778	0.663
GAS8	0.66377	0.295098	1.49303	0.321746
MED9	3.605938	1.583407	8.211906	0.002255
NCOR1	0.613469	0.258529	1.455711	0.26774
COPS3	0.289654	0.141616	0.592442	0.000689
ZNF287	3.065433	0.304333	30.87693	0.341848
KSR1	0.839909	0.224871	3.137122	0.795263
RANBP10	1.135932	0.632965	2.038566	0.669258
DPEP3	1.234119	0.989157	1.539743	0.062407
GFOD2	0.603246	0.273331	1.331372	0.210804
NOB1	1.341251	0.637013	2.824046	0.439602
PRPSAP2	0.718824	0.240905	2.14486	0.553925
PCTP	1.030705	0.518859	2.047482	0.931179
TOM1L1	1.424736	0.413641	4.907337	0.574804
TOB1	1.012525	0.666669	1.537805	0.953448
VPS53	0.581563	0.097442	3.470919	0.552056
SGSM2	0.65104	0.343374	1.234378	0.188556
NPEPPS	1.428834	0.162032	12.5998	0.747977
SKAP1	0.935272	0.615408	1.421387	0.754008
LRRC46	0.51083	0.073144	3.567565	0.498169
SCRN2	1.480986	0.328016	6.686624	0.609624
SSH2	1.3507	0.86337	2.113102	0.187983
RHBDL3	1.091581	0.357874	3.329525	0.877606
ARSG	1.442894	0.556589	3.740535	0.450611
G6PC3	1.667805	0.966873	2.876876	0.065937
CLTC	0.656736	0.321558	1.34129	0.24848
BCAS3	0.965505	0.170483	5.467983	0.96835
PTRH2	0.875172	0.326652	2.34478	0.790879
SS18	0.220037	0.05098	0.949715	0.042447
TAF4B	0.694626	0.301244	1.601708	0.392641
AFG3L2	0.419627	0.18611	0.946145	0.036312
IMPA2	0.669883	0.302308	1.484394	0.323674
GNAL	5.388475	0.170229	170.5685	0.339331
SLC39A6	0.622447	0.277176	1.397814	0.250727
RPRD1A	0.367536	0.165264	0.817374	0.014109
GALNT1	0.446212	0.15211	1.308955	0.141658
ADCYAP1	2.07875	0.100464	43.01259	0.635936
ESCO1	0.866581	0.312441	2.403541	0.783221
OSBPL1A	1.196041	0.467346	3.060937	0.708865

PELP1	0.936621	0.462245	1.897821	0.855798
NPC1	2.048674	0.936708	4.480655	0.072461
SLC14A1	0.54999	0.22983	1.316144	0.1793
ARRB2	2.23536	1.019088	4.903243	0.044734
ZMYND15	1.16268	0.797947	1.694126	0.432586
MINK1	0.979334	0.18048	5.314124	0.980693
SAT2	2.388494	1.076595	5.299026	0.032235
ASGR1	1.202764	0.97081	1.49014	0.091228
PIK3R5	1.715842	0.452356	6.508405	0.427353
TP53	1.531619	0.885919	2.647937	0.126934
ARHGDIA	1.734831	0.953812	3.155379	0.071071
TMC6	1.099155	0.633868	1.905982	0.736399
SLC16A3	1.357072	0.894748	2.058282	0.150811
CARD14	0.5994	0.046517	7.723633	0.694722
TTYH2	4.156376	0.923735	18.70175	0.063371
RAB40B	2.355502	1.07194	5.176029	0.032932
EIF4A3	0.567804	0.19363	1.665042	0.302489
CSNK1D	1.340033	0.608845	2.949335	0.467109
ANAPC11	1.228385	0.14178	10.64274	0.851879
TBCD	1.076826	0.391699	2.960317	0.885931
FN3KRP	0.411622	0.176157	0.961826	0.04038
NARF	0.567235	0.274425	1.172468	0.1259
RPTOR	3.616269	0.841779	15.53543	0.083921
FOXK2	6.021408	0.646919	56.04623	0.114723
TRIM65	0.886351	0.266064	2.95274	0.844224
CBX8	1.536223	0.367942	6.414006	0.556006
SECTM1	2.419805	0.505519	11.58307	0.268683
RNF157	0.744121	0.197388	2.80522	0.662463
ZNF750	0.276284	0.02001	3.814665	0.336867
CBX4	1.458036	0.865099	2.457371	0.156815
RNF165	1.412659	0.884501	2.256193	0.148123
DYM	0.932275	0.401642	2.163957	0.87034
ELAC1	0.753707	0.192705	2.947901	0.684494
MBD1	0.401412	0.14257	1.130195	0.083947
SMAD4	1.067621	0.529018	2.154588	0.855077
TNFRSF11	7.06374	0.748334	66.67664	0.087849
ZCCHC2	2.103334	0.2578	17.16066	0.487531
PMAIP1	0.909068	0.582898	1.417752	0.674153
ERBB2	0.222832	0.009214	5.389286	0.355661
PNMT	0.30219	0.084498	1.080729	0.065686
ARL5C	2.667368	0.555812	12.80083	0.220193
IGFBP4	1.894533	0.892983	4.019397	0.095908
FKBP10	0.165602	0.014331	1.9136	0.149816
TXNL4A	0.391252	0.07399	2.068898	0.269434
SAMD1	2.000068	1.093442	3.658422	0.024455
BRD4	2.970293	0.750296	11.75889	0.120964
SLC39A3	0.740252	0.459503	1.192535	0.216375
NFIC	1.202626	0.287021	5.039033	0.800725
PRDM15	0.089368	0.005164	1.546663	0.09688
PFKL	2.382455	0.693889	8.180113	0.167793
FEM1A	0.721296	0.335784	1.549416	0.402317
VAV1	1.060211	0.644952	1.742837	0.817664
CIB3	0.107639	0.005327	2.17514	0.146142
SH3GL1	1.128791	0.481871	2.644212	0.780287
DUS3L	1.076281	0.587096	1.973067	0.812095
DPP9	1.261455	0.513238	3.100451	0.612704
CCDC97	1.365302	0.669267	2.785211	0.391997
TMEM91	1.21486	0.775618	1.902849	0.395264

ZFP14	0.471263	0.101416	2.189885	0.337115
SIRT3	0.414553	0.010811	15.89626	0.636018
IFITM3	1.435761	1.18707	1.736554	0.000194
COL6A1	1.352092	1.007638	1.814293	0.044359
IFNAR1	0.796184	0.443897	1.428054	0.444494
SOD1	0.806915	0.339379	1.918537	0.627327
COL6A2	1.715025	0.827716	3.553528	0.146709
SIK1	0.948792	0.661441	1.360977	0.775203
TRPM2	0.813401	0.316641	2.089498	0.667884
SCYL1	0.985354	0.410916	2.362824	0.973624
TMEM50B	0.513816	0.162691	1.622757	0.25643
APP	0.910925	0.635421	1.305882	0.611675
URB1	0.460431	0.096842	2.189093	0.329551
AKT1	2.280175	0.645767	8.051193	0.200351
EMP3	1.898949	1.19703	3.012461	0.006453
SAE1	0.169113	0.052592	0.543792	0.002862
NTN5	6.502166	0.528979	79.92405	0.143605
LMTK3	1.642045	1.08175	2.492546	0.01986
GEMIN7	1.183919	0.065668	21.34473	0.908905
ADAMTS1	0.35904	0.033123	3.891798	0.399557
RNPEPL1	1.6522	0.879168	3.10494	0.118786
CAPN10	2.184423	0.041788	114.1885	0.698708
MYO1F	1.27602	0.828014	1.966424	0.269307
NLRP12	1.339663	0.818384	2.192978	0.244868
ZNF787	1.339079	0.59115	3.033296	0.483998
CARM1	1.530869	0.79969	2.930583	0.198692
EVI5L	1.486812	0.682829	3.237428	0.317781
SLC47A1	1.030963	0.289061	3.677027	0.962513
PSMB6	0.497454	0.153014	1.617243	0.245725
GPR32	0.568456	0.036423	8.871935	0.687024
SIGLEC10	0.900798	0.697609	1.16317	0.423107
ZNF473	0.431852	0.034424	5.417608	0.515267
FAM71E1	1.297113	0.462612	3.636963	0.620929
RPS11	3.792077	0.252523	56.94475	0.334893
PTH2	0.088472	0.008895	0.880011	0.038544
RPL13A	1.016021	0.49744	2.075222	0.965208
NOSIP	1.787196	1.055483	3.026171	0.030701
RCN3	1.141278	0.461019	2.825299	0.775083
ZNF614	0.717013	0.316755	1.623046	0.424824
SLC2A5	1.324285	0.988505	1.774124	0.059772
RERE	1.910948	0.705612	5.175253	0.202663
PRDM16	5.526317	1.681531	18.16213	0.004862
PADI3	3.255017	0.251759	42.0844	0.366125
EPHA2	4.340485	0.543549	34.66073	0.166097
ARHGEF19	0.820834	0.334296	2.015486	0.666631
EFHD2	1.431961	0.875171	2.342987	0.152944
PEX14	1.698708	0.85098	3.390926	0.13299
PGD	1.4964	0.806617	2.776058	0.201122
SH3BGRL3	2.68633	1.51023	4.778326	0.000771
CNKSR1	0.502274	0.058634	4.302584	0.529755
RPL11	0.192103	0.018662	1.977482	0.165502
KIAA0319L	0.50378	0.13275	1.911825	0.313657
PLK4	0.476347	0.265754	0.853824	0.012749
MAP3K6	1.519436	1.036282	2.227854	0.032154
GPN2	0.79804	0.340515	1.870308	0.603654
SYTL1	1.629792	0.986474	2.692643	0.056547
WDTC1	3.431711	0.705891	16.68336	0.126443
NBPF3	0.732103	0.085582	6.262722	0.775842

HSPG2	2.182021	0.446269	10.66895	0.335263
ITGB3BP	0.775734	0.435847	1.380675	0.387957
SERBP1	0.432487	0.218417	0.856363	0.01618
BCL10	1.330365	0.294998	5.999601	0.710309
PRKACB	0.821932	0.233124	2.897912	0.760356
PIGK	0.835648	0.418952	1.666796	0.610277
TINAGL1	2.317464	0.090289	59.48274	0.611728
RPS8	1.002441	0.354943	2.831126	0.996328
KIF2C	0.574677	0.295794	1.116501	0.102101
PTPRF	1.180168	0.656429	2.121779	0.579921
BEST4	1.900879	0.450179	8.026447	0.382124
LMO4	1.114742	0.834892	1.488396	0.461443
MTF2	0.367889	0.167956	0.805818	0.012432
IGSF3	1.256357	0.186602	8.458821	0.814554
ZNF697	2.811414	1.098359	7.196233	0.031114
CTTNBP2N	2.849201	0.603287	13.45619	0.186193
PSMA5	0.345003	0.159809	0.744807	0.006722
FNDC7	1.536857	0.110941	21.29003	0.748635
CD53	0.943062	0.547938	1.623113	0.832409
CELSR2	1.673396	0.337119	8.306433	0.528806
ITGA10	0.936001	0.097551	8.980918	0.954285
GPR161	0.236796	0.007791	7.197407	0.408265
ALDH9A1	1.641993	0.6453	4.178122	0.298013
ATP1B1	0.938418	0.634745	1.387374	0.750009
TIPRL	0.374268	0.11852	1.181881	0.093908
NME7	0.548695	0.27625	1.089833	0.086479
POGK	1.033363	0.495833	2.153625	0.9302
CREG1	0.699656	0.330986	1.478969	0.349668
DCAF6	0.455758	0.188161	1.103925	0.081698
GPA33	0.758856	0.54001	1.066392	0.111913
TBX19	0.715377	0.135294	3.78262	0.693435
UCK2	0.483064	0.226927	1.028308	0.059087
TMCO1	0.650022	0.277986	1.519966	0.320273
XCL1	6.16459	0.208888	181.9265	0.292253
XCL2	4.099884	0.55209	30.44618	0.167813
POU2F1	0.376298	0.154238	0.918064	0.031727
ILDR2	0.281554	0.011088	7.149684	0.442483
MGST3	1.737772	0.912493	3.30945	0.092697
ADCY10	0.954321	0.070866	12.85148	0.971886
UFC1	0.681407	0.394196	1.177879	0.169539
PPOX	0.731493	0.427758	1.250898	0.253376
FCGR2A	1.179609	0.76282	1.824123	0.457666
NUF2	0.372109	0.094497	1.465287	0.157468
RGS5	1.227217	0.048974	30.75205	0.900855
SDHC	1.079111	0.331889	3.508647	0.899288
PFDN2	3.723224	0.669486	20.70601	0.133193
NR1I3	63.75241	0.379793	10701.54	0.111928
USP21	2.715531	0.734553	10.03891	0.134258
PRCC	4.368233	1.375571	13.87167	0.01239
FCRL5	0.551389	0.048603	6.255376	0.630937
MRPL24	0.894084	0.293834	2.720538	0.84368
PIGM	1.36051	0.605804	3.055425	0.45579
CASQ1	1.060018	0.129357	8.686313	0.956688
ISG20L2	1.773191	0.923817	3.403495	0.085112
CRABP2	0.692216	0.112429	4.261919	0.691608
HDGF	0.818138	0.40417	1.656112	0.57693
ABL2	0.872462	0.022184	34.31329	0.941946
XPR1	1.986124	0.685029	5.758422	0.206433

RGS16	1.330758	0.357627	4.951853	0.669948
TOR1AIP1	0.723729	0.384544	1.362089	0.31626
RGL1	2.522112	1.184047	5.372293	0.016492
LYPLAL1	1.575753	0.58784	4.223936	0.366057
LHX9	0.950043	0.043682	20.66258	0.973981
TUFT1	1.425767	0.900626	2.257111	0.130178
SF3B4	1.607479	0.597566	4.324194	0.347142
ECM1	3.395354	1.887314	6.10838	4.51E-05
ZNF687	1.250617	0.414182	3.776221	0.691633
TARS2	1.479942	0.672584	3.25644	0.329941
SNX27	0.917203	0.530641	1.585367	0.756911
SETDB1	0.785052	0.360757	1.70837	0.541846
ADAMTSL4	2.508909	1.48593	4.236152	0.000578
MCL1	0.927872	0.648164	1.328284	0.682544
CTSK	0.853863	0.421467	1.729871	0.660974
RFX5	0.347787	0.106664	1.133985	0.079868
PI4KB	0.914637	0.252691	3.310605	0.891858
PIP5K1A	0.731429	0.1417	3.775516	0.708792
ANP32E	3.570738	0.76634	16.63774	0.105014
ANXA9	2.404203	0.154346	37.44953	0.531205
SELENBP1	1.001927	0.805636	1.246044	0.986195
ENSA	1.295668	0.566489	2.963439	0.539451
SEMA6C	2.107509	0.965466	4.600467	0.061244
MRPL9	0.589455	0.197233	1.761655	0.344026
ARNT	1.434627	0.526471	3.909341	0.480423
POGZ	0.124686	0.016634	0.934615	0.042789
HORMAD1	0.880746	0.068815	11.27253	0.922228
GOLPH3L	0.971494	0.425711	2.216999	0.945228
GABPB2	0.799541	0.102864	6.214657	0.830682
DTL	0.560138	0.286288	1.095943	0.090563
DYRK3	0.523684	0.187013	1.466449	0.218228
INTS7	0.453997	0.132048	1.5609	0.210103
VASH2	1.648069	0.381925	7.111698	0.503043
TAF1A	0.046382	0.002812	0.764971	0.031769
SMYD2	0.334819	0.093322	1.20126	0.093222
SUSD4	0.735388	0.028043	19.28467	0.853691
DUSP10	0.668319	0.37338	1.196234	0.174867
TP53BP2	0.849267	0.352741	2.04471	0.715519
ATP8B2	3.418337	1.059477	11.02905	0.039721
ADAM15	2.97815	1.471068	6.029207	0.002425
JTB	0.580491	0.16054	2.098976	0.406907
RAB13	1.468831	0.944794	2.283529	0.087688
S100A8	1.065837	0.821522	1.382811	0.631236
TPM3	0.76749	0.148515	3.966215	0.752163
NUP210L	0.282907	0.010873	7.360906	0.447618
SNAPIN	0.603754	0.205978	1.769693	0.357761
SLC27A3	1.609319	0.984806	2.629867	0.057583
UBAP2L	1.23954	0.643358	2.388185	0.521008
SLC39A1	1.14756	0.496501	2.652351	0.74746
HAX1	0.759372	0.306394	1.882037	0.552231
CREB3L4	0.71662	0.407521	1.260167	0.247268
EFNA3	46.11201	2.760686	770.2133	0.007657
AQP10	1.142199	0.756682	1.724132	0.526831
GATAD2B	1.57012	0.822243	2.998234	0.17164
ILF2	0.527834	0.282527	0.986132	0.045096
RIT1	0.96449	0.303429	3.065763	0.951139
INTS3	0.814578	0.334356	1.984524	0.651698
PKLR	0.129927	0.020228	0.834521	0.031508

HCN3	0.783011	0.20098	3.050585	0.724438
ACTA1	0.06278	0.006224	0.633293	0.018908
GALNT2	2.310092	0.812972	6.56422	0.116097
TTC13	0.258563	0.07063	0.946545	0.041058
SCCPDH	0.448438	0.255787	0.786188	0.005114
LYST	1.805551	0.758029	4.300645	0.182091
CEP170	0.46276	0.139184	1.538587	0.208733
ACP1	0.662325	0.312478	1.403857	0.282411
SNAP47	1.754096	0.710106	4.332947	0.223233
SRP9	0.280289	0.086691	0.906234	0.033636
NVL	0.164856	0.029398	0.924451	0.040436
DEGS1	1.339954	0.722318	2.485715	0.353306
FBXO28	0.914358	0.393643	2.123878	0.835058
ARF1	5.680619	1.132594	28.49161	0.034747
CNIH4	1.373011	0.913248	2.064235	0.127568
ITPKB	1.717925	0.893547	3.302864	0.104701
GUK1	1.266332	0.803484	1.995802	0.309008
CDC42BPA	0.5363	0.057786	4.977326	0.583612
CNIH3	0.136747	0.010249	1.824514	0.132301
MBOAT2	1.235496	0.676717	2.25567	0.491117
PARP1	0.771323	0.408737	1.455555	0.422915
PYCR2	2.241451	0.925521	5.428406	0.073702
LBR	0.594826	0.233848	1.513027	0.275453
WNT9A	0.190064	0.003995	9.042846	0.39947
EPHX1	1.859088	1.222931	2.826169	0.003711
REN	0.916555	0.494336	1.699399	0.782084
SOX13	0.534657	0.224357	1.274122	0.157601
ETNK2	1.920908	0.37659	9.798157	0.432315
PPFIA4	0.840354	0.206978	3.411931	0.80778
PLEKHA6	1.727986	0.769337	3.88118	0.185234
PTPN7	0.398254	0.124179	1.277236	0.121522
SYT2	1.601488	0.179897	14.25683	0.672894
ARL8A	0.124431	0.017484	0.885551	0.037401
GDF7	0.166263	0.021218	1.302825	0.087614
PDIA6	0.637547	0.294004	1.382519	0.254376
RHOB	1.295403	1.01296	1.656599	0.039153
ATP6V1C2	9.915051	0.428855	229.2342	0.152254
GALM	1.119407	0.604526	2.072817	0.719717
EML4	1.196473	0.733494	1.951683	0.472445
CALM2	1.258844	0.524045	3.023953	0.60668
CHAC2	0.791466	0.353126	1.773925	0.570068
RPS27A	0.864805	0.265085	2.821309	0.809741
VPS54	0.273845	0.08147	0.920478	0.036267
ASXL2	0.547083	0.286677	1.044034	0.067359
ETAA1	1.121935	0.250225	5.030418	0.880536
SNRPG	1.127905	0.578345	2.199675	0.723953
ABHD1	2.990411	0.521633	17.14339	0.218882
MEIS1	1.185381	0.890758	1.577451	0.243419
CIAO1	0.528805	0.237849	1.175685	0.118068
ZNF514	0.690097	0.318792	1.493869	0.346527
SNRNP200	0.411954	0.180682	0.939252	0.034944
MRPS5	2.016625	0.620918	6.549614	0.243188
ANKRD53	1.048081	0.317184	3.46321	0.938618
TPRKB	0.991378	0.530334	1.853228	0.978355
EXOC6B	3.294904	0.140979	77.00738	0.458359
SFXN5	0.767216	0.358463	1.642066	0.494904
TEX261	0.830658	0.266323	2.590813	0.749206
DQX1	0.035036	0.001098	1.118194	0.057863

DUSP11	1.832573	0.814616	4.122584	0.143113
ST6GAL2	0.423178	0.032011	5.594373	0.513849
THNSL2	0.704273	0.14539	3.411526	0.663184
RALB	0.885058	0.470494	1.664902	0.704878
TMEM177	1.691236	0.919006	3.112365	0.091307
RABL2A	0.729922	0.196665	2.709102	0.637997
SLC20A1	1.091225	0.608863	1.955732	0.769323
FBLN7	1.097912	0.236695	5.092668	0.905023
ZC3H8	0.279232	0.117403	0.664125	0.003904
LIPT1	0.566761	0.174005	1.846034	0.345961
FAHD2B	1.507923	0.709333	3.205594	0.285774
LYG1	0.320873	0.092552	1.112449	0.073138
AFF3	0.941484	0.45904	1.930972	0.869319
UBXN4	0.67711	0.330558	1.386984	0.286516
SPOPL	0.905405	0.446597	1.835567	0.782863
POLR2D	0.311185	0.12298	0.787418	0.013719
AMMECR1	0.82131	0.291533	2.3138	0.709511
PKP4	0.574446	0.227678	1.449367	0.240397
SCRN3	0.538097	0.085722	3.377752	0.508468
CDCA7	0.631493	0.410243	0.972066	0.036735
DLX1	0.739629	0.137239	3.986107	0.72563
UBR3	0.416145	0.183349	0.944518	0.036042
PHOSPHO	1.359388	0.435031	4.247824	0.597384
FAM171B	0.591634	0.029684	11.79184	0.731003
HSPD1	0.247657	0.071523	0.857541	0.027631
CCDC150	0.072163	0.00691	0.753626	0.028072
PTH2R	0.923069	0.66948	1.272712	0.625215
NBEAL1	1.628144	0.174992	15.14841	0.668412
SPAG16	0.331793	0.062856	1.751422	0.193695
SUMF1	0.447781	0.200785	0.998619	0.049606
RHBDD1	0.778512	0.203499	2.9783	0.714561
HES6	0.75083	0.563282	1.000823	0.050659
ANKMY1	0.095443	0.02072	0.439632	0.002574
COPS7B	0.242804	0.110185	0.535043	0.000446
DIS3L2	1.492874	0.327361	6.808004	0.604756
CPNE9	0.528762	0.069718	4.010304	0.537616
FANCD2	0.094096	0.028794	0.307492	9.16E-05
VGLL4	1.119603	0.573846	2.184404	0.740424
RAB5A	0.684505	0.346118	1.353723	0.275938
CTDSP1	1.710637	0.857814	3.41132	0.127392
STK11IP	1.36055	0.60146	3.077669	0.459745
GMPPA	1.221661	0.324805	4.594937	0.767067
EAF1	1.021984	0.534947	1.952441	0.947503
CNTN4	0.008503	0.000203	0.356563	0.012386
DYNC1LI1	1.159058	0.621973	2.159925	0.642094
OSBPL10	0.746067	0.253981	2.191567	0.594153
SLC25A38	0.430416	0.205023	0.903596	0.025889
ITGA9	1.105405	0.722678	1.690825	0.643979
GOLGA4	0.65969	0.216278	2.012185	0.464724
CTDSPL	1.381001	1.019325	1.871006	0.037205
STAC	1.731918	0.21153	14.18019	0.608674
IQSEC1	1.225754	0.843516	1.781203	0.285742
CAND2	3.242283	0.636492	16.51616	0.156753
RPL32	0.354693	0.092056	1.366639	0.132043
PTPRG	2.774201	0.68152	11.29269	0.154266
SHQ1	0.80796	0.327432	1.993698	0.643563
SLC25A26	0.277542	0.082494	0.933763	0.038389
UBA3	0.964278	0.556497	1.670866	0.896809

ARL6IP5	1.09678	0.623402	1.929619	0.748596
TMF1	0.790348	0.249457	2.504041	0.68924
LRIG1	0.92407	0.583338	1.463825	0.736531
LIMD1	0.397507	0.022251	7.101396	0.53052
ZNF660	1.046425	0.266507	4.108731	0.948151
NFKBIZ	1.005724	0.69186	1.461972	0.976144
MYH15	9.89595	1.080355	90.64599	0.042524
PHLDB2	0.412193	0.01655	10.2662	0.589008
ABHD10	0.752022	0.384614	1.470402	0.404827
PLA1A	2.518732	0.815807	7.776365	0.108268
RABL3	1.570775	0.589042	4.188725	0.366866
ADPRH	2.280264	1.092732	4.758352	0.028074
ATG3	0.642568	0.317394	1.300886	0.219067
NR1I2	1.280003	0.144609	11.32994	0.824403
SRPRB	0.515261	0.2531	1.048969	0.067526
AGTR1	3.398318	0.184043	62.74923	0.410932
MED12L	0.446505	0.068522	2.909542	0.399141
EIF2A	0.712533	0.384425	1.320681	0.2817
OSBPL11	1.365747	0.828405	2.251632	0.221723
TRPC1	0.754119	0.166823	3.408984	0.713892
LPP	1.163363	0.724066	1.869184	0.531689
TMEM44	0.460266	0.260218	0.814106	0.007658
AMT	0.554011	0.326932	0.938813	0.028193
TCTA	0.94859	0.373674	2.408044	0.911585
UCN2	0.628411	0.048918	8.072695	0.72136
STXBP5L	0.601251	0.070765	5.108488	0.641201
EAF2	0.768341	0.436422	1.352699	0.361169
ILDR1	1.770112	0.165875	18.88953	0.636405
TM4SF19	8.348233	0.049177	1417.201	0.417908
EIF2B5	0.40122	0.199115	0.808465	0.010626
DGKQ	1.00204	0.569141	1.764211	0.994365
FIP1L1	0.387826	0.111818	1.345126	0.135511
SLC26A1	0.106866	0.000483	23.63508	0.416907
LYAR	0.326554	0.153281	0.6957	0.003729
ATP10D	0.212948	0.025685	1.765525	0.151794
OCIAD2	0.663457	0.439605	1.001298	0.050726
SLC10A4	0.24346	0.043118	1.374651	0.109671
SCD5	1.934648	0.53313	7.020547	0.315619
PLAC8	0.862726	0.503632	1.477857	0.590801
ENOPH1	0.285787	0.128809	0.634071	0.002067
KLHL8	0.539496	0.270917	1.074338	0.079099
SNCA	1.006825	0.798065	1.270192	0.954252
CAMK2D	2.3421	0.243564	22.52149	0.461154
CISD2	0.911171	0.450782	1.841759	0.795574
DDIT4L	0.889913	0.615046	1.287621	0.536064
TIFA	1.146019	0.284176	4.621634	0.848082
SPATA5	0.267029	0.009051	7.877883	0.444479
CCNA2	0.828843	0.525557	1.307146	0.419302
METTL14	0.406481	0.13912	1.187662	0.099851
USP53	0.264818	0.030687	2.285258	0.226912
SETD7	0.121064	0.014341	1.021975	0.052381
NAF1	0.914778	0.247771	3.377384	0.893672
SFRP2	1.153665	0.214112	6.216111	0.867882
RPS3A	0.056306	0.008205	0.386402	0.003416
RNF175	0.551645	0.242601	1.254372	0.155829
PDGFC	1.010013	0.595074	1.714285	0.970556
CBR4	0.531089	0.268943	1.048752	0.068326
CYP4V2	1.379606	0.856919	2.221111	0.185356

ROPN1L	0.770849	0.457894	1.297698	0.3274
NDUFS6	2.58828	0.164509	40.72221	0.498809
NKD2	0.967906	0.706216	1.326568	0.839271
SRD5A1	1.631879	0.728193	3.657037	0.234231
MYO10	3.938422	0.608535	25.48938	0.150246
RPL37	0.545207	0.186273	1.595784	0.268283
SKP2	0.157908	0.037423	0.666296	0.011981
OSMR	0.178567	0.009903	3.219935	0.243006
PLK2	0.990249	0.31539	3.109148	0.986608
GZMA	0.932776	0.70605	1.232308	0.624287
PIK3R1	0.351237	0.092435	1.334637	0.1245
LHFPL2	0.888146	0.665341	1.185561	0.420866
SSBP2	0.764547	0.482355	1.21183	0.253286
IQGAP2	1.253337	0.667771	2.352383	0.4821
CRHBP	1.580617	1.002143	2.493007	0.048933
RASA1	0.445822	0.077587	2.561743	0.365189
LIX1	0.758876	0.096237	5.984135	0.793415
GIN1	2.596293	0.683277	9.865315	0.161277
PAM	0.852398	0.620797	1.170403	0.323517
BDP1	0.439605	0.10984	1.759403	0.245432
GTF2H2	1.363534	0.541424	3.433953	0.510542
SLC30A5	0.250513	0.054618	1.149024	0.074877
BTF3	0.219411	0.07843	0.613813	0.003855
FBXL17	0.799847	0.377047	1.696754	0.560537
SPATA9	0.263771	0.01573	4.42307	0.354238
TNFAIP8	0.773976	0.232677	2.574555	0.676081
FEM1C	0.647879	0.37167	1.129354	0.12579
COMMD1(	1.335466	0.764602	2.332546	0.309308
ATG12	0.27819	0.107387	0.720664	0.008427
MEGF10	0.749159	0.074596	7.523703	0.806167
YIPF5	1.496817	0.49687	4.509148	0.473458
ARHGAP2(	0.870345	0.325006	2.33073	0.782316
DDX46	0.442246	0.2418	0.808856	0.008082
TIMD4	1.160933	0.699134	1.927765	0.564131
RNF145	1.220916	0.629935	2.36633	0.554396
FBXO38	0.472519	0.196656	1.135357	0.093712
PCYOX1L	0.996448	0.502282	1.976795	0.991877
TNIP1	2.128016	0.835982	5.416926	0.113157
G3BP1	0.987827	0.481788	2.025374	0.973329
ZNF300	0.401218	0.079892	2.014907	0.267374
N4BP3	1.998537	0.111654	35.77262	0.638041
NHP2	0.582156	0.294531	1.150662	0.119643
RMND5B	1.367308	0.655794	2.850793	0.40399
KCNMB1	1.21733	0.850304	1.742781	0.282732
FAM50B	1.237722	0.783817	1.954482	0.360207
MYLK4	0.57915	0.269075	1.246549	0.162563
TBC1D7	0.357149	0.144045	0.885523	0.026257
FARS2	1.085196	0.5296	2.223661	0.823245
GFOD1	1.354535	0.911705	2.012456	0.133012
CDKAL1	0.809992	0.301145	2.178639	0.676359
PSD2	0.197276	0.034447	1.129768	0.068313
LRRTM2	0.027623	0.002095	0.364266	0.006384
ZMAT2	0.454868	0.153928	1.344162	0.154172
GFRA3	0.710486	0.032889	15.34832	0.827415
KLHL3	0.917622	0.493546	1.706085	0.785857
TRIM7	5.482945	1.163654	25.83473	0.031428
TRIM41	1.914411	0.550093	6.662456	0.307424
HIGD2A	2.918121	1.124933	7.569717	0.027664

FAM193B	1.225992	0.147735	10.17399	0.850314
PLA2G7	1.415268	0.909427	2.202467	0.123751
TNFRSF21	1.0869	0.789692	1.495965	0.609158
RNF44	0.71689	0.292841	1.754984	0.466226
DOK3	1.062972	0.664762	1.699721	0.798729
ABT1	0.552592	0.110653	2.759595	0.469764
DAAM2	0.104741	0.012039	0.911248	0.040937
PRIM2	0.811962	0.429918	1.533507	0.52083
FGD2	1.334219	0.938015	1.897775	0.108713
SCUBE3	0.324406	0.019964	5.27149	0.428718
ANO7	0.907728	0.05719	14.40762	0.94528
CRIP3	2.830737	0.343589	23.32166	0.333506
TCTE1	0.376967	0.03474	4.090563	0.422567
RPL7L1	0.49634	0.234254	1.051648	0.067473
NFKBIE	1.392871	0.770125	2.51919	0.273071
TPBG	1.112324	0.531481	2.327955	0.777556
IRAK1BP1	1.014924	0.519234	1.983828	0.965445
PHIP	0.741358	0.464859	1.18232	0.208865
PNRC1	0.902832	0.354716	2.29791	0.830195
PM20D2	0.607786	0.393406	0.938989	0.024857
RARS2	0.486197	0.194782	1.2136	0.122307
SCML4	0.787364	0.103178	6.008466	0.817654
RNF217	1.111789	0.701374	1.762361	0.6521
ARHGAP1ɛ	1.111861	0.491971	2.512816	0.798813
SHPRH	0.0943	0.018286	0.486296	0.004782
AIG1	0.514946	0.32904	0.805886	0.00368
DYNLT1	1.486771	0.810517	2.727259	0.200098
TIAM2	3.166929	0.332916	30.12603	0.315864
TMEM181	0.866753	0.426565	1.761187	0.692607
PNLDC1	0.215289	0.049919	0.928484	0.039449
WTAP	0.227284	0.043541	1.186427	0.078882
ZMYM4	0.752787	0.366857	1.544713	0.438752
VWDE	5.581061	0.332823	93.58797	0.232005
GNA12	2.709017	1.414679	5.187589	0.002643
SDK1	1.043912	0.037765	28.8564	0.979755
WASH2P	1.64894	0.751223	3.619436	0.212459
RBAK	0.919425	0.193011	4.379752	0.915996
CREB5	1.498618	0.537706	4.17674	0.43919
CDCA5	0.703367	0.469743	1.053183	0.087564
IGFBP3	3.227075	0.690246	15.0874	0.136524
PURB	0.674468	0.37282	1.220178	0.192897
MDH2	1.186205	0.359346	3.915673	0.779286
POMZP3	1.085601	0.537859	2.191148	0.818698
CCT6A	0.354952	0.128525	0.980278	0.045674
PSPH	0.560022	0.196664	1.594722	0.277534
ZNF92	1.341599	0.093781	19.1925	0.828619
ATXN7L1	0.5578	0.077773	4.000641	0.56143
TMEM168	0.686154	0.240356	1.958791	0.481584
ASB15	0.544415	0.038269	7.744802	0.653535
SLC12A9	1.571211	1.027685	2.402198	0.036975
TRIM4	1.044972	0.327097	3.33835	0.940826
MEPCE	0.876499	0.406232	1.891161	0.736896
TMEM209	0.650427	0.319282	1.32502	0.236106
AGBL3	2.180759	0.240307	19.79016	0.488392
ZC3HAV1L	0.046029	0.002925	0.724407	0.02858
TMEM140	1.212632	0.810532	1.814212	0.34826
TLK2	1.036046	0.379825	2.826017	0.944858
EPHA1	1.014579	0.485814	2.118855	0.969271

NOM1	0.365004	0.036933	3.607309	0.388527
NCAPG2	0.564283	0.284	1.12118	0.102376
RAB19	3.176125	0.115664	87.21601	0.494137
LUC7L2	0.233834	0.058635	0.932527	0.0395
SH3KBP1	0.669226	0.257777	1.737405	0.409303
LANCL3	0.407798	0.03587	4.636114	0.469543
CASK	0.214495	0.051595	0.891708	0.034207
SPIN2A	22.32252	1.425765	349.493	0.026919
MSN	1.923506	0.788741	4.690859	0.150376
HDAC8	0.322376	0.120272	0.864096	0.024428
SLC16A2	0.821278	0.265396	2.541477	0.732639
ZNF182	0.253728	0.053636	1.200276	0.083679
CHST7	1.595912	1.045853	2.43527	0.030167
NDUFB11	1.07503	0.548321	2.107687	0.833182
ZNF41	0.014939	0.000111	2.009918	0.092794
RAB41	0.115418	0.012801	1.040673	0.054301
ZMYM3	0.685094	0.183068	2.563824	0.57433
TAF1	0.758255	0.208254	2.760822	0.674689
GPR174	0.377511	0.083291	1.711051	0.20645
NONO	0.369214	0.140719	0.968732	0.042918
CCDC120	0.753441	0.251469	2.257432	0.613097
LPAR4	0.35778	0.193663	0.660976	0.001031
EBP	0.966657	0.45577	2.050212	0.929556
OGT	0.526305	0.272601	1.016127	0.055838
SNX12	1.955225	0.662541	5.770063	0.224606
ITGB1BP2	0.165643	0.015512	1.768829	0.13676
IL2RG	0.948691	0.566386	1.589048	0.841369
ZNF711	1.446091	0.547189	3.821675	0.456925
DIAPH2	0.415436	0.132771	1.299885	0.131217
NXF3	0.701273	0.407481	1.206886	0.20016
PRPS1	0.27123	0.131045	0.561376	0.000439
DOCK11	0.915962	0.552427	1.518727	0.73367
IGSF1	0.083595	0.002558	2.731921	0.163005
GPC3	1.535046	0.391128	6.024548	0.538997
RBMX	0.711591	0.380485	1.330831	0.28678
MCPH1	0.743099	0.267232	2.066357	0.569329
MFHAS1	1.42112	0.83104	2.430184	0.19919
FBXO25	7.698847	0.320978	184.6615	0.208029
NSDHL	0.447627	0.169377	1.182982	0.105003
ZNF185	0.993164	0.809165	1.219005	0.947686
CETN2	0.395616	0.194297	0.805532	0.010587
RPL10	0.351263	0.016078	7.674112	0.506124
CSGALNA(	1.319633	0.778431	2.237104	0.303063
ATP6V1B2	1.534209	0.868553	2.710022	0.140352
CCDC25	0.959886	0.237688	3.876424	0.954158
HMBOX1	0.789329	0.330217	1.88676	0.594671
CHRNA6	0.810716	0.370623	1.773393	0.599282
GNRH1	2.055805	0.500069	8.451499	0.31772
BIN3	0.775916	0.365489	1.647233	0.508903
DOK2	1.330744	0.942264	1.879387	0.104735
SLC25A37	1.001092	0.751159	1.334186	0.994057
CHMP7	6.565616	1.801594	23.92732	0.004342
DOCK5	0.389093	0.145435	1.040965	0.060108
STAR	0.373429	0.07186	1.940562	0.241402
ERLIN2	0.763378	0.233219	2.498707	0.655391
ST18	0.488916	0.1202	1.988676	0.317501
RGS20	0.019369	0.000409	0.916805	0.045059
TACC1	0.872311	0.507043	1.500712	0.621657

GOLGA7	0.247031	0.042201	1.44606	0.120935
GINS4	0.186471	0.044621	0.779263	0.021347
DNAJC5B	1.375586	0.179168	10.56122	0.75913
ADHFE1	0.520902	0.223325	1.214996	0.131223
MRPS28	1.405043	0.737164	2.678025	0.301442
LACTB2	0.563709	0.280507	1.132833	0.107459
TERF1	1.399346	0.278632	7.027804	0.683229
RPL7	0.900063	0.533833	1.517543	0.69281
ATP6V0D2	1.439239	0.149595	13.84682	0.752591
MTDH	0.712015	0.346676	1.46236	0.354977
LRP12	0.401447	0.052575	3.065353	0.378881
EBAG9	0.279938	0.043834	1.78776	0.178352
POLR2K	0.752914	0.326263	1.73749	0.505941
EIF3H	0.485913	0.240021	0.983713	0.044899
UTP23	1.076604	0.383551	3.021963	0.888526
NDUFB9	1.03683	0.556677	1.93113	0.909254
TATDN1	0.612602	0.279017	1.345012	0.221983
FAM83A	1.62561	0.956435	2.762976	0.072594
GSDMC	3.676633	0.803682	16.81962	0.093297
ZNF7	0.588356	0.226192	1.530393	0.276808
SLC39A4	0.304153	0.043721	2.11588	0.229108
VLDLR	1.513016	0.605031	3.783641	0.375888
AK3	0.393528	0.199134	0.777692	0.007288
UHRF2	0.629996	0.360157	1.102003	0.105337
NFIB	1.617203	0.506483	5.163735	0.417064
PLIN2	0.696513	0.494297	0.981455	0.038739
HAUS6	0.573112	0.047943	6.851016	0.660115
CDKN2B	3.106362	0.79452	12.14505	0.103246
CDKN2A	0.720529	0.346859	1.496754	0.379544
IFNK	3.264764	0.298513	35.70593	0.33233
ZCCHC7	0.73	0.367149	1.451452	0.369458
FBXO10	0.792243	0.059138	10.61332	0.860376
SIGMAR1	1.264683	0.564467	2.833508	0.56832
CBWD5	0.623467	0.313757	1.238892	0.177489
CEP78	0.25391	0.100821	0.639454	0.003628
AUH	0.483214	0.136492	1.710684	0.259497
ZNF462	2.31039	0.540514	9.875606	0.258531
UGCG	1.048538	0.724032	1.518485	0.801925
SNX30	0.786353	0.422902	1.462164	0.44757
STOM	0.966923	0.680136	1.374638	0.851363
GSN	1.680314	0.926286	3.048145	0.087643
MRRF	0.178295	0.044303	0.717542	0.015216
NR6A1	0.958285	0.033488	27.42182	0.980134
CRB2	0.409789	0.019344	8.681042	0.566868
ALAD	0.28377	0.072048	1.117659	0.071715
WDR31	0.64335	0.051628	8.017011	0.731833
POLE3	0.435902	0.200243	0.948899	0.036427
SURF4	1.111587	0.512748	2.409809	0.788725
GBGT1	0.794914	0.460107	1.373351	0.410657
SURF1	1.718259	0.614696	4.803046	0.302018
SURF2	2.522069	0.813981	7.814474	0.108878
SURF6	0.801539	0.418034	1.536873	0.505373
MED22	1.51762	0.530731	4.339619	0.436465
REXO4	0.619944	0.348694	1.102201	0.103414
RPL7A	0.613902	0.285476	1.320167	0.211678
GTF3C5	0.745988	0.448584	1.240565	0.258785
ASB6	0.976935	0.333352	2.863048	0.966071
PTGES2	1.386088	0.734894	2.614312	0.313221

CIZ1	0.634289	0.12695	3.169133	0.579132
SLC25A25	6.918372	1.633657	29.2986	0.008628
SH3GLB2	1.018071	0.549739	1.885385	0.954573
PTGES	0.778372	0.335473	1.805997	0.55959
LCN2	0.908927	0.758954	1.088535	0.299314
LRSAM1	0.427327	0.086456	2.112152	0.297021
GPR107	1.262933	0.088308	18.06179	0.863454
IDI2	0.493	0.071633	3.392957	0.472376
INPP5E	1.182875	0.529532	2.642323	0.682125
SEC16A	2.047279	0.999719	4.192529	0.05009
NOTCH1	1.325088	0.765415	2.293995	0.314784
NACC2	1.185478	0.805562	1.744569	0.388066
USP6NL	0.329422	0.083739	1.295913	0.112055
COMMD3	0.956011	0.493548	1.851811	0.89391
MSRB2	0.569663	0.292993	1.107589	0.097168
PDSS1	0.44654	0.152123	1.310767	0.142262
FAM171A1	1.284675	0.982851	1.679186	0.066748
RSU1	0.404871	0.147736	1.109552	0.078772
ST8SIA6	1.243185	0.615174	2.512312	0.544231
PARD3	0.863915	0.054112	13.79255	0.917575
ZEB1	0.804454	0.110094	5.878127	0.830209
NRBF2	2.427203	1.114842	5.28444	0.025495
POLR3A	0.517112	0.216943	1.232604	0.136728
HERC4	0.83436	0.302088	2.304478	0.726819
CAMK2G	0.444674	0.209356	0.94449	0.034983
GLUD1	1.149114	0.601887	2.193873	0.673566
ANKRD1	3.562316	0.509908	24.88701	0.200234
HTR7	1.433594	0.432098	4.756315	0.556103
RPP30	0.04571	0.002976	0.70201	0.026841
ADD3	0.919093	0.49751	1.69792	0.787612
DNAJB12	4.676642	0.794925	27.51325	0.087985
EIF4EBP2	1.058574	0.573304	1.954598	0.855643
TCF7L2	1.711301	1.025799	2.8549	0.039637
MKI67	0.875574	0.346972	2.209483	0.778438
LRRC27	0.155196	0.012363	1.948214	0.148933
MTG1	0.501381	0.178508	1.408242	0.190111
NKX6-2	0.023059	0.001599	0.332542	0.00563
PAOX	1.518314	0.707107	3.260155	0.284139
GSTO1	0.565988	0.257234	1.245334	0.15717
TAF5	0.743311	0.321885	1.716486	0.487245
PPRC1	0.643032	0.299493	1.380635	0.257372
ITPRIP	1.096608	0.758119	1.586227	0.624374
CNNM2	0.509128	0.084703	3.060223	0.460701
PDCD11	0.506225	0.126294	2.029108	0.336527
RGS10	1.127484	0.785347	1.618673	0.515475
BTBD10	1.534407	0.707551	3.327542	0.278346
ADM	1.192585	0.958755	1.483444	0.113717
GAS2	1.594955	0.525009	4.845405	0.410254
LIN7C	0.721998	0.325853	1.599741	0.422281
IMMP1L	0.266731	0.050537	1.4078	0.119473
TUT1	1.03505	0.494342	2.167179	0.927199
SCGB1A1	1.09097	0.052132	22.83099	0.95525
HSD17B12	0.758926	0.363874	1.58288	0.46204
APIP	0.241743	0.085045	0.687166	0.007725
DGKZ	1.870694	0.10084	34.70346	0.674255
EIF3M	0.822905	0.411955	1.643801	0.580866
TNKS1BP1	1.797971	0.510241	6.335629	0.361293
SERPING1	1.861253	0.974148	3.5562	0.060016

SSRP1	0.315481	0.100174	0.993552	0.048722
SLC43A1	1.125682	0.33717	3.758222	0.847371
PTPRJ	1.790175	0.548964	5.837775	0.33427
ARFGAP2	0.755128	0.364952	1.562447	0.449
CCDC81	1.185287	0.400063	3.511713	0.759036
SESN3	1.138236	0.712261	1.81897	0.588273
ENDOD1	0.880359	0.528097	1.467595	0.625057
CCDC82	0.896917	0.121224	6.636141	0.915151
KLHL35	0.890757	0.335522	2.364815	0.816367
SERPINH1	1.482399	0.977883	2.247208	0.063653
CAPN5	1.634033	0.739037	3.612896	0.22514
INTS4	0.110272	0.014135	0.860237	0.035413
PAK1	1.181419	0.81651	1.70941	0.376435
RPS3	0.722551	0.283023	1.844657	0.496784
ZC3H12C	1.248907	0.684979	2.277105	0.46827
TTC12	2.209603	0.582508	8.381601	0.243814
NCAM1	0.084238	0.006678	1.062615	0.055747
NPAT	1.168114	0.419575	3.25208	0.766124
ATM	0.776861	0.258641	2.333403	0.652737
AASDHPP	1.03859	0.439127	2.456395	0.931299
GLB1L2	1.621552	0.718361	3.660321	0.244562
P4HA3	0.050287	0.002605	0.970891	0.047757
ST14	1.398173	0.887376	2.202997	0.148494
HYOU1	2.473281	1.176175	5.200857	0.016947
MTA2	0.494358	0.239911	1.018671	0.056157
TMEM138	0.274472	0.098945	0.761382	0.013004
FADS1	1.327173	0.812903	2.166788	0.257746
ROM1	0.868935	0.221437	3.409758	0.84038
EML3	0.881099	0.463772	1.673961	0.699063
INCENP	0.587668	0.120355	2.869466	0.511146
ZP1	0.120548	0.00523	2.778351	0.18629
MS4A3	1.018461	0.83094	1.2483	0.860148
PLCH2	1.256372	0.547618	2.88243	0.590111
MS4A2	0.870912	0.443355	1.710788	0.688253
B3GAT3	2.275284	0.940177	5.506321	0.068279
EI24	1.432095	0.585991	3.499875	0.43086
CCDC15	0.891723	0.29004	2.741587	0.841492
CHEK1	0.374314	0.211203	0.663395	0.000764
FEZ1	1.078021	0.530271	2.191579	0.83559
ESAM	1.293875	0.978768	1.710429	0.070411
KIRREL3	0.262924	0.034355	2.012216	0.19825
MPZL2	2.628929	1.009383	6.84702	0.047806
SIDT2	1.340673	0.912703	1.969319	0.135082
TMEM25	1.78165	0.732419	4.333964	0.202885
TAGLN	1.60844	0.65043	3.977495	0.303553
DUSP15	27.05434	1.285479	569.3889	0.033878
COMMD7	0.723806	0.30936	1.693482	0.456087
DSN1	0.407615	0.171547	0.968536	0.042117
LSM14B	0.520471	0.231708	1.169103	0.113748
YTHDF1	1.255646	0.35865	4.396054	0.721782
CABLES2	0.852616	0.336745	2.158766	0.73657
GPHA2	0.076934	0.005545	1.067508	0.055968
TRPT1	0.214617	0.041206	1.117804	0.067595
NUDT22	1.775184	0.8538	3.690882	0.124358
FERMT3	1.96481	1.003012	3.848886	0.048984
PLCB3	18.73093	1.332582	263.2842	0.02979
MRPL49	1.077846	0.451544	2.572842	0.865896
FAU	5.990738	1.091032	32.8945	0.039377

TM7SF2	1.52027	0.91202	2.534178	0.108115
TBX6	0.336102	0.003976	28.41253	0.630076
PPP4C	0.996036	0.32204	3.080635	0.994499
ALDOA	2.504621	0.792021	7.9204	0.118047
DOC2A	1.463196	0.442837	4.834605	0.532508
HIRIP3	0.44264	0.228986	0.855642	0.015368
TAOK2	2.259148	0.427971	11.92547	0.336993
TMEM219	3.488323	1.601763	7.596879	0.001653
HMGA2	4.479217	0.175153	114.548	0.364605
CNKSR2	0.872985	0.072862	10.45953	0.914624
KLRF1	1.119096	0.719537	1.740531	0.617548
MKX	9.841221	0.95607	101.2997	0.054581
MPP7	0.78563	0.299292	2.062248	0.624137
ITGB1	1.268721	0.415294	3.875935	0.676159
CTF1	0.663895	0.164969	2.671748	0.564197
CWC15	0.370673	0.163147	0.842177	0.017779
FCGR1A	1.042486	0.781624	1.390407	0.777046
ARID5B	1.456133	0.925232	2.291665	0.104353
DCUN1D2	0.27284	0.043697	1.703575	0.164559
TMCO3	0.563286	0.319838	0.992036	0.04685
TMEM218	0.520359	0.136744	1.980152	0.338048
TIRAP	4.096717	0.23063	72.77056	0.336728
LATS2	1.287468	0.566698	2.924969	0.546174
SAP18	0.858308	0.406588	1.811892	0.688564
KIAA1328	0.415559	0.121492	1.421401	0.161654
FAM124A	0.937677	0.072869	12.06604	0.960626
MIA2	12.31968	0.324844	467.2227	0.175803
HNMT	1.496067	0.885122	2.528711	0.132509
PDCD4	0.362028	0.167225	0.78376	0.00993
ADRA2A	1.702763	0.494953	5.857934	0.39849
WDR17	0.032312	0.00072	1.450337	0.076993
CCDC102E	0.822511	0.039653	17.06109	0.899495
CD226	0.883428	0.355384	2.196061	0.789641
FSIP1	4.401154	0.977781	19.81032	0.053521
RGS18	1.116587	0.79377	1.57069	0.526477
PRSS23	1.020824	0.447346	2.329478	0.960949
MTMR12	1.538175	0.695571	3.401498	0.28759
CCT5	0.663653	0.262839	1.675685	0.385619
DOCK1	2.19933	1.507607	3.208432	4.30E-05
DIXDC1	1.600981	0.194558	13.17416	0.661646
DLAT	0.668134	0.277631	1.6079	0.368113
TIMM8B	1.046882	0.556845	1.968164	0.886885
IL18	0.378965	0.138992	1.03326	0.057956
TEX12	2.136768	0.256366	17.80962	0.482786
PTS	0.917452	0.491161	1.713732	0.786967
PIP4K2A	0.483793	0.282808	0.827614	0.008033
FOXO1	1.626727	1.069441	2.474413	0.022985
CRIM1	0.625425	0.218522	1.790011	0.3817
SEC24D	1.093235	0.587864	2.033062	0.778239
ABCB9	2.069991	0.819684	5.227456	0.123735
RILPL2	0.935717	0.439297	1.993107	0.863262
DHX37	1.059062	0.357262	3.13947	0.917567
UBC	0.511583	0.132367	1.977207	0.331205
ITPR1	1.156087	0.641153	2.084585	0.629658
SLC7A11	0.19491	0.021827	1.740499	0.143231
CACNA2D	1.115795	0.669883	1.858532	0.673833
DCP1B	4.423269	0.856563	22.84165	0.075878
CACNA1C	16.30266	0.233493	1138.263	0.19757

KCNA6	0.240393	0.033307	1.735023	0.157493
THRB	0.308672	0.015687	6.073856	0.439372
NGLY1	0.231144	0.117206	0.455845	2.37E-05
OXSM	0.954503	0.313784	2.903513	0.934617
UEVLD	0.660751	0.218316	1.999816	0.463328
TMEM86A	5.761128	0.663937	49.99056	0.112185
BTBD11	0.181474	0.05365	0.613846	0.006054
UBE3B	1.548111	0.50204	4.773814	0.446867
ANK3	0.870172	0.021319	35.51823	0.941421
IPMK	0.213715	0.031388	1.455139	0.114865
RAD9B	7.796612	0.941696	64.55072	0.056876
DLG5	1.905725	0.565438	6.422969	0.298229
SLC2A13	1.03253	0.123383	8.640684	0.97644
GXYLT1	0.405011	0.170591	0.961561	0.04048
TWF1	0.211546	0.033644	1.330164	0.097756
DIP2C	4.617445	0.225291	94.63671	0.320811
EIF4E	0.420997	0.034379	5.155478	0.498505
MAGI1	0.228775	0.003144	16.64682	0.500107
CSNK1G3	1.436465	0.343447	6.008006	0.619825
SRFBP1	0.581648	0.209069	1.618193	0.299269
FAM177A1	0.188879	0.021636	1.648873	0.131658
MBIP	0.745278	0.310834	1.786929	0.509945
EXT2	0.251945	0.087706	0.723737	0.010452
TMEM18	2.20287	0.911157	5.325799	0.079534
THRSP	0.761458	0.125465	4.62135	0.767069
NDUFC2	0.336563	0.093249	1.214751	0.096333
ME3	0.489139	0.131305	1.822142	0.286538
NUBPL	0.986532	0.562569	1.730001	0.962261
NEK7	0.165001	0.026045	1.045312	0.055759
FER	0.247362	0.066332	0.922445	0.037509
ANKRD50	0.499518	0.293479	0.850207	0.010528
UPF2	0.709997	0.336	1.500287	0.369587
CDC123	0.899918	0.412204	1.964693	0.791235
SCLT1	0.639346	0.16968	2.409023	0.508673
CCDC3	0.916774	0.596093	1.409971	0.692368
FRMD4A	1.051806	0.584322	1.893299	0.866258
PTPRO	1.660128	1.00931	2.730601	0.045883
EPS8	1.048138	0.565198	1.94373	0.881392
ACAD8	0.983027	0.288827	3.345753	0.978146
THYN1	0.427449	0.198633	0.919847	0.029733
VPS26B	1.513298	0.712045	3.216188	0.281456
NCAPD3	0.239447	0.085681	0.669165	0.006409
VTI1A	0.222212	0.030967	1.594554	0.134676
QDPR	0.538015	0.2167	1.33576	0.181548
FAM160B1	0.525923	0.182389	1.516509	0.234325
TEX9	0.332361	0.024639	4.483347	0.406672
MMAA	2.241725	0.397056	12.65647	0.360684
ZNF827	0.926086	0.645231	1.329191	0.677051
NR3C2	0.978703	0.196445	4.875955	0.979038
AKR1C2	2.569548	0.240821	27.41693	0.434625
DPYSL4	0.26921	0.024251	2.988486	0.28528
VENTX	1.268857	1.019126	1.579784	0.033222
ADAM8	1.118827	0.872568	1.434585	0.376027
ITIH2	3.152875	0.436738	22.76108	0.254882
KIN	0.158071	0.012208	2.046681	0.158004
PIGF	1.150706	0.452186	2.928275	0.768329
ANKAR	0.232026	0.022317	2.412309	0.221381
INPP1	0.739837	0.357196	1.532377	0.417318

MFSD6	0.945444	0.524247	1.705044	0.852083
RNF144A	0.51708	0.325546	0.821304	0.005208
ASAP2	2.352246	1.318869	4.195306	0.003761
ADAM17	0.900252	0.412886	1.962901	0.791618
FLI1	1.516736	0.812612	2.83098	0.19078
KCNJ1	0.028621	0.000381	2.150597	0.106854
TMEM45B	0.168399	0.022885	1.239155	0.080224
WWC2	0.133687	0.017814	1.003291	0.050375
ACSL1	1.097576	0.830336	1.450827	0.513121
SLC25A4	1.182063	0.504819	2.767869	0.700009
AMN1	0.881021	0.237145	3.273088	0.849954
BICD1	3.885475	0.191537	78.81983	0.376807
SAV1	1.307868	0.58507	2.923612	0.513145
CCDC122	0.261612	0.005828	11.7432	0.489661
SERP2	0.188861	0.02946	1.21075	0.078709
NBAS	0.896601	0.420582	1.911385	0.777485
ZNF385D	0.449364	0.224542	0.899287	0.02383
GUF1	0.422997	0.133171	1.343586	0.144537
SACS	0.411442	0.142404	1.188763	0.100892
PABPC3	1.550518	0.458184	5.247031	0.480722
CENPJ	0.39098	0.233969	0.653358	0.000338
FBXO4	0.77964	0.19738	3.079526	0.722471
CCL28	0.966393	0.27767	3.363402	0.957156
PARP8	3.053682	0.326282	28.57947	0.327884
DST	0.19748	0.001925	20.25908	0.492356
BEND6	1.048973	0.521468	2.110093	0.893341
TIAL1	0.362638	0.107372	1.224773	0.102373
BAG3	1.275246	0.874722	1.859166	0.206201
GLT1D1	0.837373	0.300698	2.331889	0.734114
SCHIP1	1.407535	1.089478	1.818444	0.008904
AP1S3	1.691112	0.342536	8.349087	0.518998
RABGAP1L	0.877497	0.358824	2.145899	0.774554
TUBA3E	1.568509	0.808213	3.044023	0.183336
FAM168B	0.398044	0.164672	0.96215	0.040791
PTPN14	1.187947	0.480612	2.936294	0.70913
MGAT5	0.890805	0.05338	14.86581	0.935826
TMEM163	2.171535	1.284875	3.670056	0.003778
GEMIN6	1.605842	0.973219	2.649691	0.063779
POU4F1	0.800804	0.610092	1.051133	0.109458
CYSLTR2	0.492001	0.119537	2.02503	0.325835
ARL11	0.477579	0.058562	3.894696	0.490074
SETBP1	1.550787	0.978935	2.45669	0.061586
PSTPIP2	0.596216	0.328019	1.083699	0.089827
SPC25	0.667019	0.202911	2.192662	0.504828
PDK1	0.107166	0.019887	0.577484	0.009352
PDE3B	0.748293	0.432751	1.293914	0.299381
TCF7L1	0.065629	0.004547	0.947219	0.045524
TGOLN2	0.57055	0.332354	0.979461	0.041828
KCNK13	10.59748	1.810061	62.04581	0.008844
UHMK1	0.594109	0.260463	1.35515	0.215861
ATG10	0.345181	0.125518	0.949265	0.039318
SPOCK1	0.938496	0.232755	3.784129	0.928899
FAM151B	0.46962	0.1122	1.965628	0.300782
CWF19L2	0.84694	0.371968	1.928409	0.692317
JMY	0.713064	0.340324	1.494045	0.370192
HOMER1	2.352132	0.20691	26.73873	0.490413
XRCC4	1.445821	0.692945	3.016686	0.325864
ZNF547	2.09103	0.301551	14.49974	0.455302

ZNF773	1.295482	0.587887	2.854755	0.520746
ZNF776	0.308929	0.091421	1.043927	0.058656
ZNF256	0.455428	0.272843	0.760198	0.002623
SUV39H2	13.24754	1.468619	119.4982	0.021311
DCLRE1C	0.313435	0.086137	1.140525	0.078333
OLAH	0.673741	0.106095	4.278502	0.675421
RPP38	3.750202	0.581843	24.17151	0.164428
NMT2	0.476468	0.221948	1.022859	0.057174
ZNF837	1.12556	0.493632	2.566458	0.778513
USP12	0.402519	0.163595	0.99038	0.04759
CCDC50	1.419339	0.578263	3.483748	0.444633
CAMK4	1.388488	0.112163	17.18843	0.7982
TRIM36	18.2927	1.090406	306.8793	0.043371
ZFP36L2	1.818433	1.036949	3.188873	0.036927
PAN3	0.615347	0.358298	1.056809	0.078455
PFKM	0.625325	0.304894	1.282517	0.200184
TMEM123	0.77012	0.46976	1.262528	0.300354
IGSF10	1.241252	0.758773	2.030523	0.389431
SPEF2	1.519011	0.065983	34.96938	0.793901
MBNL1	0.619416	0.273777	1.40142	0.250222
GPD1L	0.613434	0.318307	1.182193	0.144306
GJA1	1.384232	0.555829	3.447276	0.48491
SLC30A6	0.533638	0.060431	4.712308	0.571999
PELO	1.134022	0.607271	2.11768	0.693069
RASGRP3	0.975247	0.779309	1.220447	0.82662
SAR1B	2.165871	0.792502	5.919223	0.131914
CATSPER3	0.166277	0.018069	1.530145	0.113118
GPR180	0.553589	0.213235	1.437194	0.224426
TCTEX1D1	0.68652	0.479246	0.983439	0.040264
ANKRD22	0.657521	0.353138	1.224262	0.186172
FARP1	2.006605	0.063789	63.12198	0.692244
IFIT5	0.59153	0.143192	2.44362	0.468177
PANK1	0.676553	0.095152	4.810436	0.696217
PRDM8	1.160346	0.809451	1.663353	0.418279
BMP3	0.291841	0.045785	1.860226	0.192517
HHEX	1.060773	0.573709	1.961342	0.850769
UTRN	0.549451	0.174789	1.727205	0.305478
PTPRK	3.055753	0.182489	51.16823	0.437227
GGPS1	1.203732	0.371748	3.897723	0.757085
ZNF117	0.030818	0.00101	0.940634	0.046037
MARVELD:	1.346533	0.412529	4.395212	0.622046
RAD17	0.064166	0.009162	0.449397	0.005686
MED21	1.080984	0.658874	1.773522	0.757872
PLOD2	2.178816	0.372195	12.75472	0.387714
STK32B	0.826095	0.572289	1.192462	0.307682
CPB1	0.269065	0.029045	2.492501	0.247739
MR1	0.57562	0.216074	1.533453	0.269252
SRP19	0.85843	0.358751	2.054078	0.73166
CENPH	0.103808	0.022987	0.468787	0.003231
CDYL	4.059533	0.290699	56.69027	0.297627
CARHSP1	0.909634	0.533785	1.550126	0.727653
BANK1	0.353812	0.146652	0.853606	0.020765
TXNDC11	2.271965	0.973477	5.302461	0.057723
DAB2	1.894677	1.181636	3.037993	0.007983
ACMSD	0.105626	0.005473	2.038669	0.136661
BCL2L11	1.298501	0.691999	2.436574	0.415966
ANAPC1	0.216473	0.056172	0.834231	0.026196
CAST	1.640511	0.842609	3.19398	0.145342

SCOC	0.903718	0.522455	1.563209	0.717279
CLGN	0.129089	0.016402	1.015988	0.051788
CETN3	1.176398	0.532862	2.597127	0.687641
SMARCA5	0.724904	0.312945	1.679163	0.452866
SYCP2L	0.378895	0.062049	2.313675	0.293125
BMP6	1.012866	0.678485	1.512043	0.950136
RGPD3	3.385321	0.285238	40.17832	0.333982
RASSF3	0.292725	0.045623	1.878163	0.195192
HNRNPU	0.728887	0.357689	1.485303	0.383918
RANBP2	1.884321	0.50041	7.095516	0.348989
AHCTF1	0.684512	0.186919	2.506734	0.567087
MERTK	1.526325	0.908027	2.565637	0.110522
TMEM87B	1.308428	0.367861	4.653892	0.677966
NR4A2	0.942485	0.744888	1.192499	0.621706
RBMS1	1.108554	0.481321	2.553167	0.808695
SCN3A	0.128009	0.00255	6.425237	0.303532
CD96	0.875983	0.736009	1.042577	0.136072
ASAP1	0.888357	0.580879	1.358592	0.584958
FAM81B	5.536194	0.561249	54.60932	0.142818
INO80C	0.650951	0.325879	1.300293	0.223935
LPCAT1	0.926719	0.401868	2.137037	0.85831
NMRAL1	0.923221	0.487581	1.748095	0.806258
ING1	0.507748	0.050218	5.133765	0.565853
ADPRHL1	0.446378	0.024893	8.004457	0.583921
CMTM7	1.609043	0.778705	3.324775	0.19897
FBXL2	5.481956	1.647128	18.24499	0.005548
UBP1	0.929134	0.428152	2.016314	0.852491
RMND5A	0.879275	0.406549	1.901676	0.743747
CD8A	0.955116	0.682562	1.336502	0.78878
RPIA	0.303617	0.16311	0.565161	0.00017
PTPRD	1.204503	0.364925	3.975691	0.760063
CNKSR3	0.963819	0.519393	1.788524	0.906997
GTF2E1	1.594213	0.792565	3.206696	0.190884
CFDP1	0.621838	0.311282	1.242225	0.17843
ZDHHC7	1.112393	0.572526	2.161333	0.753291
JAZF1	1.080347	0.817444	1.427803	0.587001
CMIP	1.370508	0.367084	5.116786	0.639119
PID1	2.370385	1.365327	4.115295	0.002167
TRIP12	1.048674	0.569378	1.931436	0.878777
FBXO36	0.098412	0.005306	1.825378	0.119688
CEBPG	0.707382	0.3761	1.330469	0.28279
KCTD15	0.656397	0.400845	1.074873	0.094322
ZNF599	7.15544	0.165491	309.3852	0.305855
MCOLN2	0.948005	0.619861	1.449861	0.805432
LGI4	0.720501	0.052655	9.858901	0.806004
DDAH1	1.668034	0.399708	6.96092	0.48273
CHD1	0.725068	0.396392	1.326272	0.296733
DGKE	2.630462	0.533623	12.9667	0.234717
HS2ST1	0.530945	0.196933	1.431462	0.210893
MSI2	1.047047	0.708365	1.54766	0.817635
HS3ST3A1	1.260861	0.396868	4.005792	0.694306
GDPD1	0.496025	0.187131	1.314804	0.158632
NUS1	0.818177	0.233166	2.870976	0.754037
PPP2R5E	0.659541	0.292864	1.48531	0.314976
GRAP	1.69235	0.360547	7.943631	0.504845
SLC5A10	1.451222	0.563472	3.73762	0.440392
AK5	4.390668	1.476045	13.06055	0.007814
CABYR	0.324332	0.007844	13.41092	0.553232

IMPACT	0.143289	0.01301	1.578097	0.112455
TBCEL	0.816016	0.295141	2.256152	0.695171
JPH3	0.1914	0.019867	1.843942	0.152563
ANKH	0.911725	0.071945	11.55386	0.943136
UBASH3B	1.011445	0.58507	1.748543	0.967499
ROBO4	2.22602	0.690477	7.176435	0.180299
ROBO3	1.587922	1.130587	2.230253	0.007627
TBRG1	0.151357	0.016388	1.397932	0.095988
NRGN	1.171315	0.992983	1.381673	0.060599
GPR15	3.700522	0.394193	34.73896	0.252124
ABI3BP	0.031601	0.001753	0.569508	0.019203
ANGPT1	1.033061	0.788161	1.354055	0.813739
PITPNC1	1.25589	0.610467	2.583695	0.535884
CC2D1B	0.564391	0.102081	3.120433	0.512062
PRKCA	1.349061	0.95059	1.914565	0.093685
LRRK1	0.854841	0.217274	3.363286	0.822431
GAL3ST2	1.64898	0.165142	16.46543	0.670102
ABCA5	0.18911	0.009356	3.822431	0.277579
ENPP3	0.34967	0.116548	1.04909	0.060865
UCHL1	1.10964	0.680559	1.809249	0.676612
MIA3	0.239838	0.007676	7.493733	0.416186
DISP1	1.994136	0.569659	6.98063	0.280275
TNIK	0.321855	0.090108	1.149626	0.080933
TDH	0.417297	0.02932	5.939102	0.518899
FAM167A	3.334238	1.45178	7.657594	0.004529
NEIL2	1.910262	0.940922	3.878216	0.073226
OBSCN	2.162067	0.709191	6.591367	0.175176
LONRF1	0.594631	0.272629	1.29695	0.191395
TRIM11	1.729715	0.678526	4.409433	0.251106
ENAH	1.758734	0.240758	12.84751	0.577885
SH3RF1	0.822137	0.357705	1.889571	0.644616
GBP5	1.094344	0.733106	1.633584	0.659163
BUB3	0.839915	0.290291	2.430177	0.747577
MMP21	3.66157	0.068993	194.3244	0.52185
LY96	1.20401	0.944688	1.534518	0.133566
TMSB4Y	0.580194	0.263975	1.275216	0.175451
CXADR	0.311225	0.071143	1.3615	0.121106
BTG3	0.814978	0.510557	1.300909	0.39119
NCAM2	0.625808	0.060286	6.496284	0.694616
L3MBTL4	9.025112	0.771433	105.5861	0.079573
RABGEF1	1.341344	0.429142	4.192562	0.613517
MRPL39	1.02424	0.215446	4.869279	0.975978
GABPA	0.777397	0.202608	2.98283	0.713602
ADAMTS1	0.874752	0.606522	1.261606	0.473869
TSEN2	0.462261	0.201834	1.05872	0.068
SLFN13	0.281111	0.108875	0.725814	0.008738
XPC	1.713853	0.750687	3.912806	0.200858
FGD5	1.936572	0.894701	4.191691	0.093435
FLCN	2.33638	0.704695	7.746148	0.165243
DPH3	1.398774	0.563933	3.469507	0.469023
OXNAD1	0.10963	0.010956	1.096998	0.059947
PLCL2	0.958119	0.604505	1.518586	0.85553
CXXC1	0.651721	0.262681	1.616947	0.355763
SKA1	0.587841	0.242793	1.423254	0.238938
PPP4R1	3.313364	0.718158	15.28685	0.124638
CCDC144E	0.527943	0.072871	3.824877	0.52725
MPPE1	0.778585	0.196208	3.089554	0.721918
USP43	1.287881	0.26488	6.261844	0.753865

RAB6B	0.714615	0.362913	1.407152	0.331077
EME1	0.446282	0.12419	1.60374	0.216375
EPHB1	1.048702	0.314286	3.499283	0.938348
ACSS1	0.721445	0.430909	1.20787	0.214342
ANKRD40	1.18588	0.156102	9.008922	0.869111
ZNF18	0.948115	0.373736	2.405237	0.910686
VOPP1	1.28621	0.65936	2.509001	0.46033
APOOL	0.238521	0.061147	0.93042	0.039036
CYP2U1	4.469723	0.889107	22.47021	0.06917
FBXL18	1.489852	0.437	5.07931	0.524065
KLF10	0.87832	0.489113	1.577235	0.664012
PTPRN2	1.739706	0.794658	3.808655	0.16604
AZIN1	0.519487	0.256154	1.053531	0.069461
ATP6V1C1	0.778548	0.267561	2.26542	0.645981
OTUD6B	0.550522	0.199619	1.518268	0.248826
GTF3C6	0.117427	0.023461	0.587759	0.009141
TTC39B	0.506398	0.06645	3.859127	0.51139
AGPAT5	0.535371	0.315653	0.90803	0.020456
MMS19	0.972284	0.412343	2.2926	0.948794
PI4K2A	1.694861	0.530797	5.411777	0.373092
MARVELD	4.837616	1.250056	18.72119	0.022417
ZFYVE27	2.36776	0.472001	11.87771	0.294853
GOLGA7B	6.965925	0.50169	96.7214	0.148155
SLC25A28	0.731995	0.335123	1.598866	0.433827
HSPA13	1.24895	0.339212	4.59853	0.738171
SAMSN1	1.206756	0.745118	1.954402	0.444885
USP25	0.60821	0.162595	2.275102	0.460077
ZCCHC10	1.071109	0.182293	6.293594	0.939394
MOV10	1.602401	0.786336	3.265383	0.194231
RHOC	1.739797	1.263969	2.394752	0.000681
PPM1J	0.54663	0.060949	4.902539	0.589459
DBI	0.998674	0.609899	1.635272	0.995794
SLC16A1	0.762326	0.37151	1.564269	0.459313
HEATR3	0.474961	0.162998	1.383988	0.172435
TRIM74	0.22978	0.05552	0.95099	0.042427
OXA1L	0.843707	0.362714	1.962541	0.693157
SLC7A7	1.184676	0.984619	1.425381	0.072539
LARP1	0.314061	0.06568	1.501749	0.146879
CNOT8	0.602394	0.328394	1.105011	0.101552
LRGUK	2.333105	0.176747	30.79764	0.519875
MIER3	0.126131	0.012995	1.22421	0.074179
NUP205	0.440792	0.220347	0.881783	0.020581
ZKSCAN2	1.345059	0.359594	5.031182	0.659634
PIK3AP1	1.109196	0.746878	1.647278	0.607533
RBM45	0.572936	0.203427	1.613624	0.291757
TTN	0.004713	2.45E-05	0.906851	0.04589
VSIG4	3.109457	1.760709	5.491378	9.25E-05
PDIA4	1.174606	0.563775	2.447254	0.667405
OTOA	19.99152	0.181668	2199.945	0.21172
KCTD18	0.814357	0.245991	2.695938	0.736704
FAM126B	0.419745	0.094779	1.858918	0.252883
FZD7	1.411052	0.217098	9.171301	0.718425
RNF20	0.697495	0.326887	1.488283	0.351508
PPARGC1E	1.376531	0.755357	2.508533	0.296638
ELMO1	0.856918	0.275219	2.668085	0.789881
SLC26A2	0.786067	0.29789	2.074264	0.626811
LSM11	1.272649	0.151765	10.672	0.824146
MED7	1.545191	0.60134	3.970494	0.366147

RRAGA	1.212385	0.577992	2.543077	0.610365
RASA2	0.783115	0.210202	2.917518	0.715615
RMND1	3.6059	1.025699	12.67673	0.045552
SLA	1.061506	0.645267	1.746246	0.814193
TMBIM4	0.794243	0.303939	2.075488	0.638323
VBP1	0.612145	0.276456	1.355447	0.226244
RAB39B	0.734841	0.096061	5.621332	0.766628
CLIC2	1.840924	1.066087	3.178917	0.028555
AFF2	0.204801	0.059838	0.700949	0.011538
GRIP1	0.990154	0.167418	5.856019	0.991294
VPS37A	1.29383	0.623397	2.685279	0.489267
PSD3	1.452566	0.100088	21.08082	0.784442
GNA14	5.090642	0.942624	27.49202	0.058585
FAM161B	1.427798	0.112543	18.11396	0.783511
GNAQ	0.76618	0.493552	1.189404	0.235244
GPR61	0.400479	0.061755	2.597095	0.337367
ADK	0.732213	0.179142	2.992799	0.664359
BATF	0.976171	0.522467	1.823867	0.93972
DCK	1.09375	0.425689	2.810241	0.852349
ADAMTS3	0.156024	0.032425	0.750758	0.020471
ALX3	25.05183	0.966424	649.3985	0.052453
DPY19L4	0.586747	0.251978	1.366278	0.216351
ART3	0.855729	0.200172	3.658222	0.833516
WHAMM	0.745851	0.308948	1.800607	0.514345
CXCL13	2.92394	0.565461	15.11939	0.200586
N6AMT1	0.426606	0.123899	1.468878	0.176873
RWDD2B	2.261487	0.54591	9.368433	0.260474
USP16	1.008615	0.269994	3.767877	0.989821
CCT8	0.870012	0.48264	1.568293	0.643236
BACH1	0.397158	0.042211	3.736806	0.419446
TSPAN7	0.832739	0.646151	1.073206	0.157327
TIAM1	3.967057	1.242778	12.66319	0.019966
RPGR	0.167274	0.039654	0.705611	0.014902
PCGF6	0.375933	0.116311	1.215069	0.102152
ANKRD9	1.086572	0.71428	1.652906	0.698083
SFXN2	0.39268	0.092248	1.67155	0.205938
FUT6	0.916317	0.311653	2.694143	0.873808
TDRD9	1.005012	0.66223	1.525226	0.981257
FGF18	6.470493	0.581915	71.94737	0.128662
PCDH1	0.021305	0.00061	0.743745	0.033729
UQCRB	0.684305	0.260915	1.794734	0.440639
PTDSS1	0.759287	0.51913	1.110544	0.155757
RPL30	1.296768	0.562902	2.98739	0.541635
FAM122C	1.322315	0.155905	11.21528	0.797849
SUPV3L1	0.335747	0.112974	0.997805	0.04954
FAM122B	0.46518	0.212973	1.016055	0.054857
EEF1A1	1.460984	0.256435	8.323661	0.669352
FBXO43	0.156509	0.009252	2.647563	0.198707
HK1	2.001481	0.890121	4.500426	0.093266
TYSND1	2.315047	0.821797	6.521616	0.112161
PHF6	0.296388	0.026351	3.333639	0.324698
CD109	3.724441	1.094593	12.67271	0.035324
NODAL	0.385403	0.056307	2.637963	0.331272
PRG3	0.794642	0.590369	1.069596	0.129475
UBE2L6	0.792286	0.323409	1.940936	0.610535
ZDHHC5	1.107976	0.400311	3.066642	0.843514
MED19	1.7575	0.529145	5.837351	0.357201
ZFAND3	3.725375	1.612655	8.605941	0.00208

NPTN	1.138173	0.536104	2.416393	0.736161
SAMD8	2.652961	1.046321	6.72662	0.039845
RAB11FIP1	1.344741	0.716698	2.523139	0.356257
GLYATL2	0.432856	0.079783	2.348412	0.331805
UTP14A	0.896633	0.369564	2.175402	0.80934
AIFM1	0.200685	0.052479	0.767443	0.018939
MAPK13	0.674848	0.285093	1.597445	0.37104
BAG4	0.649938	0.244152	1.730151	0.388388
MS4A1	15.66707	0.650843	377.1372	0.090011
ATAD2	0.528712	0.287176	0.973397	0.040702
FBXO32	1.543136	0.453952	5.245633	0.487118
NSMCE2	0.563569	0.264491	1.200834	0.137335
ZNF689	0.880367	0.411204	1.88482	0.74287
PRR14	0.578073	0.284103	1.176222	0.130498
FBRS	1.336744	0.693825	2.57541	0.385692
FRRS1	0.628411	0.053193	7.423968	0.712321
PHKG2	0.838261	0.362021	1.940993	0.680459
SASS6	0.19598	0.029529	1.300679	0.091464
ITGAD	2.141506	0.272005	16.86015	0.469483
VPS8	0.38763	0.168129	0.8937	0.026171
GALK2	0.322258	0.083209	1.248072	0.101174
B3GNT7	1.508391	0.145415	15.64651	0.730543
MPV17L	2.204674	0.71496	6.798411	0.168827
BUB1B	0.584509	0.244382	1.398017	0.227468
PDE6D	0.447463	0.154255	1.297998	0.138886
EIF4A2	0.468599	0.239733	0.915956	0.026645
BRPF1	0.605535	0.273804	1.339179	0.215435
RPUSD3	0.59814	0.364411	0.981779	0.042083
TATDN2	0.719616	0.344812	1.501823	0.380724
GHRL	0.577441	0.37048	0.900015	0.0153
SEC13	0.5082	0.191504	1.348625	0.174042
EXOG	0.421796	0.03091	5.75577	0.517381
NTAN1	5.018706	0.997883	25.24085	0.050301
ZFYVE9	0.915725	0.084366	9.939422	0.942313
SMG1	0.84794	0.346335	2.076027	0.71806
FCHO2	0.982843	0.526588	1.834411	0.956652
RBPMS	2.788542	1.197899	6.491337	0.017368
TIMP4	0.804806	0.306815	2.111087	0.658964
CPT2	1.680449	0.821139	3.439016	0.155427
NECAP2	2.339166	1.210603	4.519811	0.01145
LRP8	0.875007	0.282241	2.712709	0.817087
PAXIP1	0.398938	0.178348	0.892364	0.025272
SSBP3	1.580218	0.744047	3.356089	0.233795
CLDN12	0.701802	0.278319	1.769643	0.453014
MMP14	0.643227	0.075444	5.484058	0.686543
FZD1	3.595378	1.333023	9.697311	0.011478
GATAD1	0.238021	0.084955	0.66687	0.006319
SUSD3	0.878682	0.643111	1.200541	0.416684
TMED6	0.096042	0.016906	0.545596	0.008204
DHRS4	0.957039	0.377974	2.423246	0.92619
DDX19B	0.413837	0.112905	1.516867	0.183096
ST3GAL2	2.690304	1.384551	5.227495	0.0035
IL34	7.735331	0.655572	91.27201	0.104239
DHRS1	1.89357	0.736198	4.870441	0.185308
CACNA1D	1.377802	0.153812	12.34192	0.774498
KIT	0.959077	0.740318	1.242479	0.751756
AASDH	0.629256	0.256759	1.542156	0.311147
ZNF19	12.21163	1.227774	121.4588	0.032757

CACNA2D	1.080791	0.804228	1.45246	0.606414
RNF111	0.597806	0.076958	4.643701	0.622795
CCNB2	0.775916	0.554157	1.086418	0.139587
FAM81A	3.22822	1.465173	7.112746	0.003641
MYO1E	5.624211	0.735775	42.99107	0.096055
APPL1	0.993661	0.444423	2.22167	0.98764
AFAP1L1	0.310191	0.041111	2.340476	0.256267
TSC22D3	1.033843	0.562123	1.901417	0.914742
DYRK1A	0.935204	0.389781	2.243843	0.880745
KCNJ15	3.362085	0.219906	51.40195	0.383502
ERG	0.107567	0.003412	3.391205	0.205381
ETS2	1.69663	1.124801	2.559165	0.011711
TSPAN18	0.554937	0.098465	3.127566	0.504448
LCA5L	3.310767	0.320603	34.18931	0.314892
SLC35B2	0.786262	0.421191	1.467762	0.45022
TMEM164	0.604657	0.107668	3.395717	0.567716
MX1	1.405103	1.104085	1.788192	0.005694
C2CD2	0.811231	0.486876	1.35167	0.421904
SLC38A10	1.929144	0.906476	4.105565	0.088166
ZNF618	0.629774	0.240683	1.647876	0.346095
SVOPL	0.777223	0.611693	0.987547	0.039159
SNX22	1.469643	0.544901	3.963749	0.446908
UBN2	0.680012	0.216635	2.134544	0.508761
BRAF	0.107551	0.018138	0.637726	0.014076
PSMG3	0.895797	0.365584	2.19499	0.809824
WDR19	0.553643	0.29823	1.027798	0.061055
SLC37A3	2.30352	0.574962	9.228781	0.238643
AP3S2	0.490753	0.138314	1.741243	0.270619
FMNL2	3.503501	1.248347	9.832619	0.017253
SPPL3	2.994766	0.782254	11.46511	0.109283
RAB28	0.259686	0.059208	1.138983	0.073866
TNFRSF14	0.745036	0.504204	1.100901	0.139562
PANK4	1.90593	0.658243	5.51858	0.234428
MEGF11	7.50971	0.651893	86.51079	0.105913
PEX10	1.040968	0.130511	8.30286	0.969768
RER1	2.044988	0.755622	5.534481	0.159033
RADIL	1.076405	0.273739	4.232679	0.916063
SKI	4.400477	1.098888	17.62163	0.036333
WIPI2	1.026057	0.26879	3.916789	0.969977
LDLRAP1	1.366763	0.679892	2.747553	0.380485
AGAP1	1.040428	0.017303	62.5593	0.984871
KRTCAP3	0.159048	0.025192	1.004129	0.050514
PAFAH2	0.706004	0.342644	1.45469	0.345249
MRPL17	1.31034	0.744876	2.305069	0.348293
DUSP2	1.266597	0.924823	1.734677	0.140779
UBXN11	2.773421	0.898767	8.558241	0.076011
PTPDC1	3.4975	0.191128	64.00166	0.398558
GALNT14	1.439351	0.75238	2.753571	0.27118
NCK1	1.004152	0.460259	2.19077	0.991694
HPD	1.983207	0.425615	9.241006	0.383185
RHPN1	1.476344	1.015503	2.146316	0.041296
TPRG1L	0.864634	0.343433	2.176823	0.757511
LRRC43	1.138135	0.15357	8.434936	0.899248
XKR8	1.655944	0.610015	4.495214	0.322226
CNNM4	0.9569	0.25875	3.53878	0.947359
EYA3	0.150101	0.034833	0.646813	0.010941
DZIP1L	1.19554	0.29092	4.913079	0.804383
TMSB15A	2.631613	0.775109	8.934729	0.120786

FANCC	0.194517	0.048175	0.785405	0.021495
MRAS	4.626319	1.070862	19.98653	0.040202
WASF2	2.631851	1.272324	5.444085	0.00907
ABHD3	0.851344	0.459002	1.579047	0.609624
FAIM	1.170968	0.407311	3.366388	0.769571
CLSTN2	0.655431	0.1614	2.661649	0.554626
COLEC12	0.701719	0.121855	4.040946	0.69169
RNF207	0.101928	0.010115	1.027154	0.052717
CUL4B	0.939792	0.290415	3.041201	0.917457
GPR153	0.678353	0.056394	8.159769	0.759751
SLC13A3	1.546594	0.325272	7.353713	0.583587
GPRASP2	0.924325	0.095799	8.918485	0.945755
RHBDL2	1.284731	0.623854	2.645702	0.496641
AUTS2	0.9262	0.632322	1.356659	0.693823
SHROOM⁴	1.047561	0.652639	1.681458	0.847384
CDC25C	0.616918	0.131183	2.901201	0.540864
MITD1	0.289936	0.124465	0.675395	0.00411
EIF5B	0.551948	0.236466	1.288335	0.169392
RIBC1	0.090598	0.007283	1.127047	0.061905
TSPAN33	1.091175	0.740337	1.608272	0.659306
AHCYL2	0.570753	0.22478	1.449238	0.23818
B4GALT5	1.018735	0.705466	1.471114	0.921133
CD1D	1.313806	0.915664	1.885065	0.13843
CD1A	3.466516	1.170783	10.26384	0.02479
SPATA2	0.952434	0.269596	3.364773	0.939672
CD1C	0.774572	0.411316	1.458642	0.428946
CD1E	1.455591	0.558403	3.794291	0.442497
HMHB1	1.991686	0.357268	11.10318	0.431924
NCF1	1.095408	0.935091	1.283212	0.259021
TSR2	0.592981	0.214676	1.637939	0.313405
PPP1R9A	1.296893	0.490172	3.43131	0.600495
ZC3H18	1.775924	0.647446	4.871303	0.264609
ZFAND2B	0.702968	0.294606	1.677372	0.427022
GDPD5	1.289189	0.882697	1.882875	0.18873
DYNC1I1	4.285265	0.717916	25.57889	0.110399
PFKFB1	0.023684	0.001131	0.495778	0.01586
ALAS2	0.983101	0.758224	1.274673	0.897665
TMED4	0.377682	0.154123	0.925519	0.033236
PPP1R15B	1.117583	0.555183	2.249695	0.755472
COPG2	0.87118	0.546366	1.389094	0.562364
ZSCAN12	1.225944	0.258275	5.819144	0.797672
TAGLN2	2.001716	1.140083	3.514541	0.015673
ELK4	0.335252	0.063023	1.78337	0.199993
SLC45A3	1.096861	0.83227	1.44557	0.511556
DUSP23	0.951351	0.446019	2.029217	0.89733
RNF166	1.493055	0.801389	2.781687	0.206751
NBL1	1.259452	0.919151	1.725745	0.15118
HTR6	0.189574	0.011201	3.208373	0.249224
F11R	2.08124	0.723934	5.983366	0.173711
USF1	0.712765	0.398027	1.276379	0.254679
SPATA2L	1.621276	1.041752	2.523187	0.032256
NIT1	3.947655	1.04678	14.88754	0.042614
DEDD	4.040413	0.976084	16.72494	0.054034
ZNF276	2.093638	0.514272	8.523361	0.302275
NPM2	1.063478	0.681751	1.658942	0.786167
FGF17	1.005576	0.132467	7.633457	0.99571
CDA	0.998704	0.798445	1.249189	0.990935
PINK1	0.981084	0.444784	2.164029	0.962262

B4GALT3	0.604937	0.251625	1.454338	0.261407
ADAMTS4	0.481299	0.157491	1.470871	0.199495
FAM160B2	1.488492	0.659298	3.360559	0.338396
NDUFS2	1.294746	0.400323	4.187543	0.666233
SLC5A11	29.21831	1.561193	546.8315	0.023945
FCER1G	1.106231	0.889039	1.376482	0.365304
APOA2	0.740504	0.042342	12.95043	0.836971
TOMM40L	3.635033	0.647564	20.40487	0.142571
MPZ	5.348212	0.687734	41.59075	0.109101
CACHD1	0.560986	0.170158	1.849491	0.34226
CDC42SE2	0.517638	0.189231	1.415988	0.199665
RAPGEF6	0.729664	0.404422	1.316471	0.295207
EPB41	0.942208	0.389013	2.28207	0.895068
ALG8	0.359743	0.107184	1.20741	0.097949
FBXW5	4.717008	1.086856	20.47205	0.038343
SYNJ1	1.83458	0.322828	10.42564	0.493641
IFNAR2	3.855957	0.950307	15.6459	0.058941
MRPL10	4.995356	0.84164	29.64877	0.076689
IFNGR2	1.373017	0.931969	2.022789	0.108809
GART	0.444945	0.173555	1.140708	0.091817
SON	0.387941	0.151437	0.9938	0.048504
DONSON	0.21481	0.073563	0.627262	0.004908
SV2A	1.310636	0.800628	2.145522	0.282049
STC1	0.916643	0.074855	11.22482	0.94571
CSRP1	1.6849	1.070989	2.650715	0.024032
C1QC	1.463163	1.14077	1.876667	0.002726
RCAN1	1.099225	0.302967	3.988214	0.885596
UBE2Z	1.866557	0.504801	6.901803	0.349584
SNF8	0.788627	0.440309	1.41249	0.424543
CCDC24	1.43202	0.981966	2.088342	0.062122
RUNX1	2.153419	0.897598	5.16625	0.085797
IGF2BP1	0.385295	0.135361	1.096711	0.073938
CBR1	1.261633	0.582107	2.734406	0.555937
CBR3	1.42359	0.641837	3.157515	0.384864
MORC3	1.042304	0.531321	2.044709	0.904072
CHAF1B	0.355303	0.186274	0.677714	0.001685
HLCS	0.366655	0.070025	1.91984	0.234911
SCUBE1	1.226028	0.729373	2.060872	0.44187
ARHGAP2?	1.939531	1.222156	3.077986	0.004933
ADPGK	0.283099	0.096206	0.833061	0.021926
PTMS	1.449346	1.075857	1.952495	0.014652
PADI4	0.976402	0.780514	1.221451	0.834423
ADIPOR1	0.977726	0.624575	1.530559	0.921525
CYB5R1	1.490449	0.703073	3.159616	0.297876
PSMD4	1.585838	0.347609	7.234803	0.551544
ATP13A2	1.418296	0.762848	2.636912	0.269402
PSMB4	1.057408	0.298001	3.752043	0.93116
IRX6	4.71503	1.597017	13.92065	0.004993
BTG2	1.518114	1.116359	2.064454	0.007773
HK2	0.977002	0.518797	1.839895	0.942567
C1R	0.698835	0.012678	38.51967	0.860948
ALDH4A1	0.516444	0.230728	1.155972	0.107969
STARD9	2.907328	0.448306	18.85442	0.263196
THEM4	0.133088	0.027994	0.632728	0.011232
UBR1	0.401027	0.159001	1.011455	0.052888
AMFR	1.7309	0.761949	3.932045	0.190015
MED8	0.787828	0.209873	2.95737	0.723826
RGL4	0.855241	0.653092	1.119961	0.255734

RSPRY1	0.465921	0.250245	0.867481	0.01603
CCDC17	0.161032	0.029039	0.892988	0.036666
GPBP1L1	0.794484	0.279529	2.258096	0.665982
NAE1	0.126487	0.039605	0.403962	0.000483
TMEM69	1.866981	0.610306	5.71126	0.273786
ACE	19.13892	0.20003	1831.221	0.204649
SPON2	0.986206	0.445684	2.182269	0.972657
CHCHD6	1.220018	0.575744	2.585255	0.60374
CTBP1	1.246643	0.488498	3.181424	0.644658
LRRC36	0.97281	0.220986	4.282449	0.97092
TPPP3	1.313978	0.6553	2.634729	0.441743
ZDHHC1	1.727974	0.992997	3.006953	0.052978
ATP6V0D1	1.489788	0.928005	2.391657	0.098823
AGRP	2.358216	0.654641	8.495009	0.18951
ZFYVE28	0.55728	0.080958	3.836079	0.552492
FAM131B	7.763345	0.586549	102.7527	0.119914
RGS12	2.96267	0.155876	56.31041	0.46976
PSKH1	0.872155	0.331582	2.29401	0.781608
ZYX	1.937004	1.19764	3.132813	0.007035
ABR	2.147138	0.878072	5.250368	0.093944
CCDC117	0.811328	0.301646	2.182201	0.678744
ZNF230	0.709754	0.198366	2.539504	0.598126
CCDC107	3.524171	1.679408	7.395331	0.000866
ZNF222	0.767903	0.164392	3.587011	0.737019
NPR2	1.020163	0.086822	11.987	0.98733
ZNF221	0.637298	0.033006	12.30521	0.765508
ZNF233	0.111655	0.010424	1.196021	0.069984
ZNF235	0.709771	0.164367	3.064929	0.646009
GNE	0.285781	0.121819	0.670426	0.003989
TNFRSF13(	1.051333	0.269592	4.099911	0.942526
PTGIR	3.491092	2.015735	6.04629	8.14E-06
CALM3	0.996671	0.515896	1.925493	0.992082
DFFA	0.628914	0.233703	1.692463	0.358521
CCDC28B	0.930362	0.470495	1.839706	0.835616
IQCC	0.748166	0.327425	1.709559	0.491372
BSDC1	0.591727	0.273805	1.278797	0.182037
ZBTB8A	1.332424	0.788893	2.250435	0.283165
ATAD3B	1.058139	0.358274	3.125143	0.918538
SSU72	0.642339	0.289786	1.423807	0.275743
UBE2J2	2.035392	0.23813	17.39729	0.516214
ZNF362	1.160767	0.565009	2.384704	0.68487
FNDC5	4.217985	0.204281	87.09256	0.351447
CPAMD8	3.383781	0.704473	16.25325	0.127896
NR2F6	1.041783	0.527045	2.05924	0.906274
ANKLE1	0.86488	0.382013	1.958097	0.7277
CCDC58	0.908478	0.614051	1.344075	0.631018
KALRN	0.168586	0.002015	14.10201	0.430543
CILP2	0.880846	0.179361	4.325854	0.875837
ABCG1	2.699806	0.86118	8.463918	0.088454
TFF3	0.848957	0.642725	1.121363	0.248812
UBASH3A	0.560749	0.179471	1.752032	0.319633
RSPH1	0.394493	0.106139	1.466231	0.164944
SLC37A1	0.633001	0.343969	1.164903	0.141706
PDE9A	3.092752	0.745336	12.8333	0.119915
WDR4	0.851924	0.264253	2.746513	0.788448
NDUFV3	0.867546	0.396028	1.900461	0.722494
PKNOX1	2.794066	0.410886	18.99992	0.29346
U2AF1	0.865192	0.398873	1.876679	0.713966

HSF2BP	0.060697	0.003396	1.084699	0.05682
RRP1B	0.798482	0.293494	2.172356	0.65943
PDXK	1.633436	1.013404	2.632821	0.043943
G6PD	2.15038	0.914953	5.053959	0.079071
CSTB	1.924693	1.277581	2.899577	0.001739
RRP1	1.327666	0.579546	3.041509	0.502771
AGPAT3	1.772668	0.706087	4.450376	0.222861
TRAPPC10	0.900272	0.062116	13.04805	0.938613
GAB3	0.308178	0.151258	0.627891	0.001188
ICOSLG	2.564883	1.265684	5.19768	0.008955
ZNF66	939.4279	7.356291	119968.7	0.005667
LRRC3	2.523566	0.64229	9.915118	0.184887
ITGB2	1.255796	0.738204	2.136298	0.400777
RALGDS	0.997199	0.336134	2.95836	0.995966
FTCD	0.055486	0.001293	2.380246	0.131611
LSS	2.535318	0.749052	8.581298	0.134788
VAV2	3.302133	1.539599	7.082415	0.002152
MCM3AP	0.379799	0.153431	0.940145	0.036311
PCNT	0.48374	0.221232	1.05773	0.068857
DIP2A	0.033492	0.003137	0.357611	0.004938
S100B	0.088479	0.008605	0.909768	0.0414
PRMT2	0.984561	0.305284	3.175269	0.979222
CLDND2	0.668348	0.358367	1.246458	0.205098
ADAMTS1	0.244294	0.00285	20.93981	0.534859
SLC2A6	1.222676	0.947214	1.578247	0.122689
ZNF761	0.488103	0.099194	2.4018	0.377667
ZNF714	0.294618	0.10706	0.810762	0.017975
GPSM1	1.228111	0.605697	2.490115	0.568849
HIPK4	0.028979	0.001973	0.425552	0.009789
TOR2A	1.441965	0.666375	3.120259	0.352718
ST6GALNA	1.833624	0.985739	3.410818	0.055543
SHKBP1	1.849654	1.124306	3.042962	0.015467
RDH13	1.668107	0.992998	2.802201	0.053185
ZER1	2.371419	1.027405	5.473622	0.043041
ZDHHC12	1.786908	0.991	3.222041	0.053619
PKN3	2.360315	1.323378	4.209746	0.003625
BRSK1	1.539036	1.151732	2.056584	0.003556
COX6B2	1.183554	0.035052	39.96308	0.925229
TAOK1	1.361225	0.678584	2.730588	0.385251
MED27	1.159715	0.439879	3.057517	0.764502
DEDD2	1.660974	0.901467	3.060385	0.103676
SIK3	1.788461	0.878322	3.641709	0.109076
MPZL3	0.280631	0.02998	2.626874	0.265455
NEK8	0.687046	0.27293	1.729502	0.425514
TLCD1	2.280452	0.665611	7.813057	0.189488
PCSK7	1.581674	0.58401	4.283648	0.367094
SAFB	0.803273	0.428057	1.507386	0.495164
CD3G	1.000685	0.580738	1.724305	0.998033
S100A1	3.160342	1.741118	5.736408	0.000155
CXCR5	1.262917	0.528688	3.016823	0.599309
ZBTB7B	1.679051	0.720791	3.911276	0.229704
FLAD1	0.333766	0.088956	1.2523	0.103846
SHC1	4.820004	0.604191	38.45212	0.1377
VPS11	0.454744	0.10772	1.919715	0.283533
NLRX1	0.693805	0.253715	1.897268	0.476319
ADAR	1.112458	0.26409	4.686146	0.884512
IL6R	1.911433	1.081257	3.379009	0.025832
UBE2Q1	0.698119	0.262818	1.854404	0.470924

CHRNB2	3.055596	0.356285	26.20561	0.308336
CRTC2	2.579707	1.074888	6.191238	0.033868
ANO10	1.444851	0.614961	3.39468	0.398453
FDPS	0.579485	0.228206	1.471488	0.251156
RUSC1	2.328276	0.930558	5.825391	0.070894
FAM189B	0.368354	0.09174	1.479019	0.15909
PAQR6	1.243265	0.429738	3.596862	0.687884
PMF1	1.420847	0.528841	3.817421	0.486066
SLC25A44	1.392806	0.569254	3.40781	0.467984
LMNA	1.341636	1.009926	1.782296	0.042548
CCR5	14.5889	0.529438	402.0038	0.11317
NBEAL2	1.14932	0.719345	1.836304	0.560491
CCDC12	1.173483	0.432482	3.184094	0.753433
PTH1R	1.218774	0.628612	2.363002	0.558092
UBQLN4	0.645059	0.274407	1.516362	0.314743
GPATCH4	0.340212	0.131684	0.878957	0.025989
FCRL3	0.952657	0.367649	2.468541	0.920473
FGFR4	1.048795	0.012869	85.47749	0.983071
NACC1	10.64994	2.375529	47.74565	0.002
HK3	1.096895	0.918136	1.310459	0.308228
IER2	1.549854	0.950284	2.527714	0.07915
ZNF394	0.800502	0.357995	1.789981	0.587849
CPSF4	0.383436	0.136885	1.074067	0.068151
LY6E	1.700918	1.224682	2.362345	0.001528
VPS28	1.166141	0.557077	2.441104	0.683437
PTGER1	1.205713	0.794102	1.830678	0.37996
RECQL4	0.558131	0.223117	1.396172	0.212555
LRRC14	0.58756	0.302542	1.14109	0.11636
ZNF333	0.793265	0.178814	3.519126	0.760606
PPP1R16A	0.71327	0.344452	1.476997	0.362918
ORAI2	1.009791	0.451496	2.258441	0.981072
ALKBH4	0.869085	0.298577	2.529695	0.796868
SH2B2	2.423721	1.294892	4.536613	0.005641
SQSTM1	1.048278	0.433188	2.53674	0.916719
MGAT4B	1.25362	0.426013	3.689004	0.681466
RPL8	0.807004	0.250994	2.594704	0.718959
MAML1	12.64499	0.91013	175.6846	0.058781
LRWD1	0.407555	0.187379	0.886444	0.023574
FBXL13	0.933113	0.362885	2.399383	0.885762
NAPEPLD	0.484045	0.159626	1.467801	0.199865
SCGB3A1	0.935028	0.590317	1.48103	0.774659
PSMC2	0.314801	0.122222	0.810817	0.016647
USP41	0.842387	0.044587	15.9154	0.90893
YDJC	0.882777	0.451645	1.725459	0.715379
CCDC116	2.576868	0.216571	30.66084	0.453755
DVL3	0.743351	0.387345	1.426557	0.372517
AP2M1	4.131264	1.246883	13.68801	0.020289
ABCF3	1.48651	0.477513	4.627544	0.49384
PCYT1A	0.453131	0.043492	4.721073	0.507975
U2AF1L4	0.482406	0.161098	1.444553	0.19268
BDH1	0.201791	0.019638	2.073546	0.178153
NPHS1	0.389673	0.018528	8.195608	0.54424
THAP8	5.072899	1.006667	25.56386	0.049066
COX7A1	2.428894	1.126335	5.23781	0.023612
ZNF382	72.00015	3.292605	1574.444	0.006586
LRRC56	1.32066	0.681705	2.558501	0.409746
PLXDC1	0.545864	0.048022	6.20485	0.62545
PGAP3	0.691537	0.308454	1.550389	0.370563

IKZF3	1.156891	0.219321	6.102454	0.863622
GRIN2C	0.195753	0.028007	1.36821	0.100186
FDXR	3.835663	0.328518	44.78382	0.283644
SAP30BP	3.065874	0.688592	13.65043	0.141479
ACOX1	0.33147	0.045491	2.415248	0.275832
PRPSAP1	0.436563	0.182332	1.045276	0.062806
CYGB	2.231611	1.222694	4.073046	0.008925
ZNF577	2.026364	0.589941	6.960276	0.261969
TMEM143	0.718028	0.251149	2.052818	0.536545
KLHL10	14.39464	0.499996	414.4146	0.119796
HCRT	0.650577	0.040318	10.49786	0.761913
ALDH16A1	1.775261	0.926191	3.402703	0.083812
ITGA5	1.531778	0.900458	2.605723	0.115684
SIGLEC11	1.755201	0.558467	5.516403	0.335604
ZNF385A	1.609159	1.165945	2.220853	0.003804
SIGLEC16	1.234435	0.808957	1.883698	0.328696
MPP3	6.371369	1.059565	38.31226	0.043055
NAGS	0.297113	0.031567	2.796479	0.288704
LSM12	3.918234	0.646797	23.7363	0.137311
ASB16	1.287053	0.321799	5.147635	0.721232
JOSD2	1.828813	1.141338	2.93038	0.012089
SHANK1	0.608629	0.022373	16.5572	0.76829
DBF4B	0.040534	0.001327	1.237708	0.066106
PLCD3	4.250422	1.141305	15.82933	0.031007
FMNL3	0.143736	0.010709	1.92922	0.14319
RACGAP1	0.531196	0.230002	1.226813	0.138524
LARP4	1.214993	0.381975	3.864676	0.741515
RAVER1	1.317061	0.703958	2.464135	0.388873
SYCE2	0.578953	0.137727	2.433697	0.455679
SPC24	0.669949	0.415552	1.080084	0.100215
LEMD2	0.661398	0.261884	1.670387	0.381809
ALOX15	2.654973	0.243682	28.92652	0.422955
TREML1	0.99878	0.616721	1.617522	0.996039
ZNF653	1.180603	0.57165	2.438247	0.653665
MED11	6.649309	1.558876	28.3623	0.010472
CXCL16	1.409234	0.919487	2.159834	0.115334
BCL6B	0.857519	0.158962	4.625887	0.85813
ASGR2	1.348318	0.884449	2.055474	0.164774
TNFSF13	2.370328	1.279163	4.392292	0.006101
SENP3	0.626925	0.158024	2.48719	0.506636
EIF4A1	1.298992	0.384692	4.386312	0.673522
RPL26	1.093583	0.571308	2.093308	0.787126
POLR3K	0.633992	0.254681	1.578231	0.327406
SNRNP25	0.57398	0.15013	2.194447	0.417164
WDR90	0.307284	0.081254	1.162084	0.0821
JMJD8	0.647324	0.291515	1.437418	0.285294
CCDC78	0.02301	0.000522	1.015129	0.050915
MSLNL	0.017478	0.000846	0.360912	0.008801
SPSB3	0.578045	0.255293	1.308834	0.188677
CCNF	0.998049	0.669579	1.487656	0.992351
TBC1D24	0.560228	0.150491	2.085538	0.387608
AMDHD2	2.34361	1.160133	4.734377	0.017597
NTN3	0.037576	0.001156	1.221683	0.064712
PAQR4	1.053336	0.561913	1.974533	0.871246
FLYWCH2	0.858356	0.273711	2.691795	0.793384
ZNF75A	0.543901	0.159203	1.858181	0.33129
ADCY9	1.54685	1.046639	2.286123	0.02862
CLPB	0.155998	0.02199	1.106644	0.063085

NEU3	0.047818	0.003144	0.727319	0.028581
ASRGL1	0.816752	0.423615	1.574743	0.54564
GNG3	1.011821	0.114237	8.961898	0.991575
UBXN1	6.022569	1.423801	25.475	0.014681
TTC9C	1.753396	0.743825	4.133226	0.199309
TAF6L	0.652795	0.296691	1.436312	0.28913
NXF1	0.903926	0.438927	1.861546	0.784053
STX5	2.535897	0.70273	9.151132	0.155265
SLC25A45	0.068713	0.004654	1.014411	0.05123
RPL29	0.789281	0.420747	1.480614	0.460974
ITIH3	0.660661	0.104295	4.184984	0.659864
SYVN1	2.582009	0.78925	8.44697	0.116743
ZFPL1	1.167414	0.475652	2.865234	0.735441
RPS6KA4	2.386754	1.451414	3.924858	0.000608
LRP5	1.594689	0.850596	2.989707	0.145576
TPCN2	0.938484	0.49217	1.789531	0.847122
PDZK1IP1	1.148526	0.792125	1.665284	0.465047
TAL1	1.060741	0.684757	1.643169	0.791721
CMPK1	0.539364	0.295123	0.985736	0.044786
BEND5	0.790768	0.522485	1.196806	0.266883
ZYG11B	0.75688	0.366319	1.563848	0.451866
MAGOH	0.397376	0.162882	0.969459	0.042547
ACOT11	1.02311	0.337695	3.099706	0.967776
FAM151A	0.786662	0.021641	28.59549	0.895864
PARS2	2.081271	0.678649	6.382815	0.199854
USP24	0.486146	0.272036	0.868775	0.014898
NOL9	0.859972	0.220551	3.3532	0.827988
KLHL21	1.102396	0.555987	2.185804	0.780139
ZSWIM5	2.964712	0.72683	12.09294	0.129737
GMEB1	0.740256	0.227459	2.40913	0.617395
JAK1	0.830026	0.511633	1.346559	0.45046
RAVER2	0.572018	0.350915	0.932434	0.025054
CTRC	4.169769	0.567613	30.63176	0.160508
LZIC	0.488798	0.1595	1.497956	0.2103
RBP7	1.803029	0.968176	3.357771	0.063166
FBLIM1	0.423699	0.005832	30.78181	0.694523
SLC25A34	0.233538	0.072521	0.752063	0.014789
AKR7A3	1.606875	0.480747	5.370904	0.441088
DHRS3	0.785785	0.60182	1.025984	0.076483
MATN1	2.700139	0.420249	17.34864	0.295298
LAPTM5	1.588268	0.85361	2.955209	0.144195
SDC3	2.588018	0.031504	212.6022	0.672476
PEF1	2.5312	1.022592	6.265426	0.044614
RBBP4	0.401981	0.140211	1.152466	0.089905
KIAA1522	2.581506	1.479979	4.502885	0.000835
TSSK3	0.849691	0.05819	12.40722	0.90522
TMCO4	0.848459	0.246482	2.920635	0.794432
UBXN10	0.163208	0.022851	1.165692	0.070743
CAMK2N1	1.600961	0.641407	3.996025	0.313269
ALPL	1.034271	0.798955	1.338896	0.798073
WNT4	0.176925	0.00723	4.329335	0.288373
TTLL10	1.418692	0.362572	5.551141	0.615355
SCNN1D	0.577274	0.29259	1.138949	0.113033
MXRA8	2.206496	0.101787	47.83137	0.614105
MEGF6	0.547114	0.338923	0.883189	0.013574
IL23R	6.068748	0.119664	307.7753	0.36805
NFIA	1.030239	0.723051	1.467937	0.869014
OMA1	0.534645	0.230626	1.239434	0.144402

MYSM1	0.629746	0.077569	5.112595	0.665154
TM2D1	0.191553	0.062575	0.586381	0.003791
USP1	0.99851	0.207754	4.799058	0.998515
FUBP1	0.51011	0.21274	1.223148	0.131415
NEXN	0.785541	0.450784	1.36889	0.394299
DNAJB4	0.798216	0.282264	2.25728	0.670889
TYW3	0.655353	0.341469	1.257762	0.203914
B3GALT2	3.55642	0.288074	43.9058	0.322455
FAM102B	0.713196	0.395984	1.284515	0.260201
GBP2	1.335816	1.008023	1.770201	0.043843
ATXN7L2	0.847794	0.381391	1.884559	0.685379
GBP4	1.132836	0.84164	1.524783	0.410662
ZNF326	0.498091	0.118529	2.093111	0.341335
GFI1	1.228728	0.788928	1.913703	0.362195
AGL	0.210025	0.010765	4.097644	0.303245
VCAM1	1.330896	0.693179	2.555304	0.39041
EXTL2	0.856806	0.414015	1.773165	0.677068
SLC30A7	0.691421	0.369603	1.29345	0.248195
ZNF281	0.640866	0.336358	1.221048	0.176129
ARPC5	0.82017	0.465621	1.444692	0.492515
NLRP3	1.495166	1.000716	2.233922	0.049593
ZNF496	0.05279	0.003728	0.747479	0.029615
TRIM58	1.00134	0.700061	1.432278	0.99415
SLAMF9	3.213183	0.41788	24.70698	0.262048
KCNJ9	0.981733	0.076533	12.59317	0.988701
IGSF8	3.195955	1.008903	10.12399	0.048265
DDR2	2.785881	0.144605	53.6714	0.497264
PEA15	1.710229	1.159189	2.523216	0.006842
PEX19	0.330865	0.085588	1.279061	0.108889
NCSTN	0.741373	0.319155	1.722157	0.486494
VANGL2	1.322984	0.791281	2.211966	0.285845
SLAMF6	0.712892	0.422133	1.203921	0.205577
OLFML2B	1.525464	1.011594	2.30037	0.043908
FCRLB	0.696791	0.357557	1.357873	0.288561
FCGR3B	1.538155	0.89887	2.632106	0.116188
KLHDC9	1.510991	0.463067	4.930374	0.493936
FLVCR1	0.481776	0.120424	1.927434	0.30191
ATF3	1.036256	0.606357	1.770946	0.896367
RBM15	0.633641	0.273845	1.466161	0.286429
DENND2D	0.494224	0.243105	1.004742	0.051547
IER5	1.248365	0.870014	1.791253	0.22854
SNED1	3.947635	0.0773	201.6016	0.49382
BPNT1	0.436434	0.193785	0.982919	0.045332
ACP6	1.405365	0.77987	2.532541	0.257415
KIF26B	1.156562	0.27932	4.788902	0.84098
TFB2M	0.673872	0.340808	1.332429	0.256451
KLHDC8A	0.269366	0.046307	1.56688	0.14427
PM20D1	0.619045	0.081184	4.720353	0.643581
PKDCC	0.838532	0.16591	4.238061	0.831303
OXER1	1.696075	0.563172	5.107977	0.347616
HAAO	2.42924	0.50965	11.57894	0.265282
B3GALNT2	0.420529	0.094478	1.871809	0.255512
MAPKAPK	2.833658	0.74435	10.78743	0.126738
IL24	0.821796	0.015134	44.62572	0.923284
PIGR	0.511967	0.04711	5.563752	0.582315
CAPN2	1.793757	0.962103	3.344305	0.066002
MRPL55	1.73838	0.664684	4.546468	0.259621
WDR26	0.864768	0.495736	1.508514	0.608797

REL	0.808884	0.406916	1.607933	0.545137
PUS10	0.849214	0.221203	3.260191	0.811778
PEX13	3.715756	0.715102	19.30751	0.118492
KIAA1841	2.645329	0.292644	23.91218	0.386474
TRIM17	0.455325	0.043437	4.772966	0.511666
RFTN2	0.733357	0.285064	1.886643	0.520057
DISC1	0.416326	0.051758	3.348772	0.410062
MEMO1	0.796348	0.303916	2.086664	0.643126
DPY30	0.625003	0.153569	2.543672	0.511634
ARL5A	0.606306	0.210866	1.74332	0.353118
DUSP19	1.041394	0.639837	1.694965	0.870358
NUP35	0.671776	0.263726	1.711185	0.404324
CCDC138	0.311421	0.084879	1.142604	0.078583
FBXO41	0.215351	0.061725	0.75133	0.016022
SMC6	0.710046	0.330599	1.525005	0.379959
CCDC74A	1.086475	0.401824	2.937672	0.870183
SLC16A14	1.418323	0.300552	6.693149	0.658887
SGCB	1.264815	0.191282	8.363343	0.807417
NOSTRIN	0.563938	0.051534	6.171162	0.638916
SGPP2	1.349535	0.609581	2.987698	0.459752
BBS5	2.927767	0.110747	77.40019	0.520262
SMARCAD	0.732092	0.393542	1.361884	0.324778
HPGDS	0.562099	0.340045	0.929159	0.024672
PDLIM5	0.15529	0.0274	0.880104	0.035356
RPRD2	0.546822	0.228625	1.307881	0.174877
ANKRD23	0.420252	0.039357	4.487429	0.473085
CTSS	1.159973	0.867281	1.551443	0.317206
MSX1	0.546983	0.161838	1.848697	0.331541
PACRGL	0.29415	0.026436	3.273023	0.319533
BNIPL	0.487069	0.006465	36.69547	0.744262
C1QTNF7	0.660255	0.066197	6.585409	0.723521
TNFAIP8L2	1.774977	1.097244	2.871326	0.019381
LYSMD1	0.014872	0.001275	0.173509	0.000787
SCNM1	1.523143	0.51459	4.508377	0.447264
TMOD4	0.286679	0.056293	1.459947	0.132492
VPS72	1.153858	0.654966	2.032761	0.620373
ERCC3	0.420077	0.167753	1.051932	0.064045
RNF149	1.287467	0.746124	2.221576	0.363987
IWS1	0.625146	0.342912	1.139672	0.125214
BOLA3	0.482619	0.174216	1.336968	0.161108
CDC42EP3	1.131943	0.575865	2.224991	0.719274
S100A11	1.332247	1.003362	1.768935	0.047348
DHX57	1.526646	0.064271	36.26268	0.7935
ARHGAP2	2.206386	0.664985	7.320673	0.195932
S100A9	1.118521	0.858242	1.457735	0.407217
S100A12	0.990606	0.843408	1.163495	0.908452
TGFA	0.678506	0.326356	1.41064	0.298966
TDRD10	3.416156	0.667711	17.4778	0.140207
CCNYL1	2.392099	0.705804	8.107265	0.161366
FZD5	1.85545	0.637605	5.399417	0.256712
DCAF16	0.551938	0.246343	1.236632	0.148755
NPPC	0.186412	0.009839	3.531949	0.263047
GNPDA2	0.745515	0.22555	2.464166	0.630189
PAQR3	0.571038	0.280904	1.160838	0.121639
ANTXR2	1.185152	0.753819	1.86329	0.461838
HELQ	0.453735	0.161997	1.270861	0.132626
MRPS18C	0.540955	0.210428	1.390656	0.202163
CGGBP1	0.314256	0.100656	0.981125	0.046287

GPR155	0.378956	0.033576	4.277028	0.432623
DAPL1	1.583089	0.976066	2.567625	0.062634
PMVK	1.218371	0.653281	2.272268	0.53452
PBXIP1	1.351786	0.69856	2.615843	0.370834
PYGO2	1.938551	0.74316	5.056761	0.176008
HIPK1	0.020792	0.000659	0.655821	0.02784
LENEP	1.202409	0.126752	11.40641	0.872426
DCST2	0.484738	0.026773	8.776253	0.624093
DCST1	6.938709	1.016752	47.35245	0.048051
COL6A3	3.50765	0.752906	16.34149	0.10994
YY1AP1	0.160336	0.01248	2.059867	0.159958
KBTBD8	2.057575	1.145821	3.694829	0.015705
LMOD3	1.015144	0.619757	1.662775	0.952396
SLC22A15	0.885281	0.666876	1.175215	0.39923
ATP1A1	1.570835	0.58941	4.186429	0.366539
SLC15A2	0.652768	0.284195	1.499345	0.31474
EIF4E3	0.979106	0.668581	1.433855	0.91361
PROK2	0.87132	0.7172	1.05856	0.165458
LRRC58	0.485805	0.220288	1.071356	0.07359
FSTL1	1.433893	0.748302	2.74762	0.27742
ELF3	1.213044	0.353974	4.157016	0.758589
TMEM183/	0.506939	0.096408	2.66562	0.422424
IGFBP7	0.796159	0.59961	1.057136	0.11506
TRIM46	1.410589	0.902008	2.205924	0.131579
KRTCAP2	0.423425	0.138303	1.296349	0.132241
CXCR1	1.025742	0.404226	2.602871	0.957337
ARPC2	0.444202	0.045291	4.356567	0.486049
CCT3	0.916989	0.383656	2.191728	0.84545
TMEM79	0.966895	0.467271	2.000737	0.927702
SSR2	0.63259	0.158101	2.531112	0.517442
RNF25	5.849214	1.705	20.06646	0.004981
STK36	0.785386	0.385746	1.599058	0.505442
NEK10	1.959089	0.279269	13.74311	0.498667
CCDC141	0.37859	0.029201	4.908348	0.457488
FEV	1.582602	0.802224	3.122106	0.185412
EOMES	0.941791	0.619838	1.430971	0.778723
CWC22	2.46964	0.719255	8.479775	0.150891
AZI2	0.587642	0.295713	1.167765	0.129185
TGFBR2	1.89494	0.947135	3.791218	0.070846
ANKZF1	3.042584	0.30795	30.06109	0.341034
HDAC11	10.55218	2.918185	38.15674	0.000327
TRAT1	0.63888	0.276822	1.474475	0.293728
FBLN2	1.884131	1.065366	3.332142	0.029434
GLB1L	0.663185	0.264405	1.663411	0.381376
STT3B	0.798521	0.445476	1.431358	0.44989
CHCHD4	0.632651	0.30095	1.329943	0.227134
FCRL1	1.334662	0.088589	20.10767	0.834764
SERPINI1	0.483962	0.12008	1.950522	0.307484
CLASP2	0.680765	0.380767	1.217123	0.194579
SUCLG1	0.464769	0.177073	1.219892	0.119647
NUAK2	1.557972	1.013171	2.395724	0.043429
SPTA1	0.918688	0.738758	1.14244	0.445717
PRKCI	0.159936	0.052239	0.489667	0.001324
MNDA	1.164777	0.913486	1.485194	0.218632
PYHIN1	0.719914	0.466544	1.110883	0.137587
IFI16	1.192708	0.824044	1.726307	0.350243
AIM2	1.021189	0.513731	2.02991	0.9523
EFHB	1.889956	0.226559	15.76604	0.556439

EIF5A2	0.516182	0.110731	2.406225	0.399796
RPL22L1	0.871176	0.548231	1.384357	0.559476
PPM1L	1.403033	0.1255	15.6853	0.783367
ICA1L	0.175212	0.013584	2.260022	0.181875
CTLA4	1.060325	0.457847	2.455598	0.891263
ICOS	1.182906	0.72287	1.93571	0.503832
RYBP	1.101289	0.628285	1.930393	0.736168
PPP4R2	0.903294	0.277494	2.94039	0.865877
CD200R1	4.412397	0.887558	21.93573	0.069649
GTPBP8	1.184652	0.363348	3.862408	0.7787
WDFY3	0.803714	0.078946	8.182247	0.853571
COX18	0.256362	0.024428	2.690404	0.256444
PTPN13	0.724103	0.074284	7.058364	0.781113
THOC7	0.252524	0.105202	0.606155	0.002066
ATXN7	0.409629	0.051243	3.274514	0.400049
PSMD6	0.266186	0.09014	0.786059	0.01659
ADAMTS9	0.278035	0.016096	4.80254	0.378571
PPM1K	0.735646	0.154899	3.493728	0.699334
GMPS	0.646819	0.346705	1.206717	0.170881
TIPARP	0.755615	0.502036	1.137277	0.17917
CCNL1	0.668729	0.449314	0.995293	0.047343
PTX3	1.322589	0.656887	2.662926	0.433612
HESX1	1.436324	0.739634	2.789254	0.284936
SLMAP	0.54621	0.228607	1.30506	0.173565
RPL9	1.171575	0.501435	2.737323	0.714576
RPP14	1.006483	0.205701	4.924658	0.993636
ABHD6	2.903971	1.413889	5.964433	0.003695
DNASE1L3	1.281989	0.56615	2.902935	0.551368
RBM47	1.221167	0.964605	1.545969	0.096818
APBB2	0.129067	0.004838	3.442888	0.22169
IL17RE	0.414871	0.033547	5.130593	0.49295
IL17RC	16.77873	1.384852	203.2894	0.026706
CRELD1	0.183823	0.033942	0.995549	0.049398
PRRT3	1.092701	0.636371	1.876256	0.747909
PCOLCE2	1.021757	0.821576	1.270712	0.846599
MTMR14	1.384098	0.527408	3.632345	0.509057
TTC14	0.881823	0.464274	1.674898	0.700804
CXCL3	3.119806	0.567244	17.15873	0.190835
CXCL5	1.223601	0.628516	2.382115	0.552714
PPBP	1.075705	0.932588	1.240786	0.316421
PF4	0.946728	0.154263	5.810175	0.952843
MTHFD2L	0.377759	0.123819	1.152505	0.087162
CXCL1	1.088384	0.820317	1.444051	0.55716
RCHY1	0.807845	0.117933	5.533759	0.827939
CPA3	1.09091	0.932126	1.276743	0.278287
GYG1	0.940066	0.575413	1.535809	0.805078
HPS3	0.648009	0.311919	1.346231	0.244833
TOPBP1	0.510628	0.2654	0.982445	0.044114
RYK	0.450452	0.1929	1.051876	0.065317
SNRK	0.926719	0.475572	1.805841	0.823078
TCF23	0.435268	0.025408	7.4566	0.56606
UCN	0.46302	0.211575	1.013295	0.05399
ZNF513	3.624855	1.334047	9.849408	0.011567
SLC4A1AP	0.503337	0.145631	1.739655	0.277955
PLB1	0.918721	0.460528	1.832781	0.809874
SPDYA	0.128015	0.000801	20.44809	0.427132
KIAA1143	1.018073	0.19484	5.319615	0.983061
KIF15	0.873824	0.446038	1.711892	0.694243

TGM4	0.061004	0.005056	0.736029	0.027723
WDR43	0.443023	0.174588	1.124181	0.086604
ZDHHC3	1.072351	0.405268	2.837471	0.888106
CDCP1	1.787041	0.435347	7.335559	0.420378
CLEC3B	1.40332	0.950293	2.072316	0.088451
LZTFL1	0.597722	0.311717	1.146143	0.121306
FYCO1	0.771918	0.387001	1.539679	0.46242
CCR1	1.453892	1.12849	1.873124	0.003791
LRRC2	0.294047	0.006127	14.11133	0.535426
FBXO40	4.769959	0.387682	58.68865	0.222459
DTX3L	1.149272	0.477585	2.765638	0.75616
ZNF148	0.64953	0.340119	1.240418	0.191125
NMNAT3	0.310025	0.15134	0.635097	0.001371
ZMYM6	0.386648	0.092749	1.611848	0.192038
YEATS2	0.706854	0.358013	1.395598	0.317511
ZC3H12A	1.193411	0.85036	1.674855	0.306522
MEAF6	0.599031	0.135208	2.653981	0.499834
SNIP1	0.878516	0.49262	1.566706	0.66079
DNALI1	0.257412	0.025995	2.549004	0.246013
POLR2H	0.419068	0.149254	1.176637	0.098707
LIPH	0.271125	0.024358	3.017795	0.288425
TMEM41A	1.371778	0.593375	3.171311	0.459725
RPN1	0.454446	0.183794	1.123653	0.08772
SENP2	0.417369	0.177099	0.983611	0.045744
HEYL	2.426573	1.149366	5.123047	0.020067
IFT122	0.29899	0.07205	1.240732	0.096337
RFC4	0.397925	0.21266	0.744591	0.003945
RPL39L	1.177963	0.724453	1.915373	0.509025
BAP1	0.363315	0.129608	1.018438	0.054201
TKT	1.294012	0.657475	2.546816	0.455612
PRKCD	1.550801	0.716722	3.355532	0.265197
RFT1	0.075966	0.010631	0.542819	0.010202
SFMBT1	0.100696	0.02817	0.359945	0.000412
GNL3	0.45539	0.199162	1.041265	0.062303
PBRM1	0.05979	0.005513	0.648399	0.020547
ARHGEF3	1.630043	1.103571	2.407676	0.014082
SLBP	0.561483	0.21599	1.459621	0.23637
LRPAP1	1.220812	0.807572	1.845511	0.344001
ZDHHC19	0.567032	0.201032	1.59938	0.283569
UBXN7	0.801668	0.174368	3.685715	0.7764
RNF168	1.930184	0.234583	15.88182	0.540828
PIGX	0.652835	0.302642	1.408242	0.27696
S100P	0.942889	0.779878	1.139973	0.543699
CLDN19	0.902795	0.04162	19.58275	0.948064
ERMAP	0.855994	0.342874	2.137016	0.739056
ZNF691	2.203811	0.701632	6.922123	0.176004
SGMS2	1.541349	0.819777	2.898054	0.179246
METAP1	0.378611	0.191246	0.749538	0.005314
DNAJB14	0.548571	0.142461	2.112374	0.382738
EMCN	0.061343	0.002451	1.535238	0.089314
BDH2	0.351608	0.060091	2.057355	0.246209
PGRMC2	0.902234	0.429027	1.897375	0.786188
CDC25A	0.452494	0.191625	1.068497	0.070476
CAMP	0.886852	0.761167	1.033292	0.12357
ZNF589	0.513222	0.170083	1.548635	0.236501
PLXNB1	1.00268	0.653819	1.537685	0.99021
CCDC51	1.31021	0.439984	3.901615	0.627466
ATRIP	0.552555	0.210448	1.450799	0.228424

SHISA5	2.588604	1.467287	4.566845	0.001025
SPRY1	0.849218	0.558962	1.290196	0.443722
APEH	1.04403	0.521047	2.091938	0.903286
INTU	1.922711	0.366344	10.0911	0.439619
RNF123	0.659201	0.291346	1.49151	0.31716
HSPA4L	0.334036	0.068459	1.629872	0.175131
MFSD8	0.051862	0.005226	0.514676	0.011497
CAMKV	1.109756	0.797303	1.544656	0.537048
MON1A	2.164371	0.857727	5.461531	0.102051
MST1R	1.432902	0.383004	5.360798	0.593112
RAD54L2	0.684187	0.266924	1.753726	0.42937
TEX264	1.10419	0.486856	2.504305	0.81249
GRM2	0.479572	0.083667	2.748862	0.409433
DUSP7	4.0243	1.323685	12.23478	0.014118
PPM1M	1.248057	0.867048	1.796494	0.233133
WDR82	1.55186	0.729106	3.303045	0.254193
NDST3	0.572898	0.051308	6.396954	0.650918
HMGB2	0.737304	0.464027	1.171521	0.197078
SAP30	1.059022	0.741842	1.511815	0.752193
SCRG1	1.518458	0.168323	13.69818	0.709748
MAD2L1	0.625485	0.336422	1.162919	0.138087
ANXA5	1.231364	1.00936	1.502196	0.040188
MAP9	1.207941	0.162927	8.955663	0.853367
FBXO8	2.194108	1.001314	4.807794	0.049618
HPGD	0.782558	0.488721	1.25306	0.307358
TMEM144	1.268817	0.795538	2.023659	0.317501
IL15	2.467049	0.560538	10.85801	0.232339
FAM160A1	0.269556	0.024436	2.973548	0.284491
ARFIP1	1.498582	0.363438	6.179175	0.575715
HHIP	0.500174	0.072117	3.469001	0.48322
ANAPC10	1.127581	0.576329	2.2061	0.725848
ABCE1	0.659588	0.384111	1.132634	0.131429
OTUD4	0.790578	0.150453	4.154213	0.781317
LSM6	1.043151	0.566499	1.920857	0.892121
TMEM184	1.733847	0.576732	5.212518	0.32711
ITGA2	1.187397	0.311678	4.523614	0.801279
MOCS2	0.215035	0.042139	1.097325	0.064562
EDIL3	1.604333	0.124654	20.64822	0.716881
TMEM161	0.430129	0.066969	2.762652	0.373961
ELOVL7	1.340422	0.450542	3.987934	0.598412
NDUFAF2	0.742519	0.367928	1.498483	0.405974
LMBRD2	0.303259	0.030572	3.008223	0.30811
NIPBL	0.393974	0.103431	1.500673	0.172225
SLC25A46	0.508455	0.248418	1.040691	0.064195
STARD4	1.238166	0.183272	8.364928	0.826516
PGGT1B	0.492561	0.113322	2.140944	0.344883
F2RL2	0.088924	0.001122	7.049859	0.278086
CCDC112	0.387552	0.179115	0.838546	0.016079
CMBL	0.892515	0.617452	1.290115	0.545246
PRRC1	0.737021	0.333803	1.627306	0.450209
F2RL1	1.394935	1.064179	1.828494	0.015934
AGGF1	0.396355	0.154244	1.018498	0.054618
WDR41	0.35017	0.175546	0.698498	0.002897
NDUFS4	0.385648	0.162439	0.915574	0.03078
SPINK1	0.0862	0.004795	1.549674	0.096353
GRPEL2	0.636248	0.201918	2.004833	0.440019
ARSK	0.450067	0.073151	2.769091	0.38911
RHOBTB3	0.714551	0.514549	0.992291	0.044839

TIGD6	2.473373	0.319364	19.15547	0.3859
SERINC5	0.081563	0.004231	1.572366	0.096878
CAGE1	3.258438	0.107717	98.56736	0.497108
CASP3	1.082194	0.570572	2.052577	0.808885
ERAP1	0.22783	0.022243	2.333627	0.212737
ERAP2	0.884834	0.681261	1.149238	0.359026
CMYA5	0.040274	0.0024	0.675907	0.025604
RICTOR	0.245785	0.035828	1.686107	0.153219
EBF1	1.116917	0.366382	3.40493	0.845842
ANKRA2	0.465862	0.189465	1.145472	0.096095
UBLCP1	1.023796	0.565322	1.854091	0.938134
UTP15	0.12834	0.029068	0.566648	0.006736
TLR3	0.585152	0.042566	8.04402	0.688598
NSA2	0.432982	0.170614	1.09882	0.078131
GFM2	0.232569	0.055419	0.975982	0.046241
TERT	0.079572	0.002882	2.197087	0.134906
CCDC127	2.580365	1.045732	6.3671	0.039686
ACSL6	1.240287	0.250539	6.140007	0.791876
IL3	1.821103	0.191019	17.36169	0.602329
CSF2	2.23654	0.174968	28.58879	0.535821
SHROOM1	0.00501	0.000129	0.194198	0.004536
GDF9	0.610233	0.029757	12.51423	0.748616
UQCRQ	1.153944	0.413072	3.223616	0.784718
LEAP2	0.708902	0.156033	3.220732	0.655971
GJB7	2.432031	0.222337	26.60275	0.46654
SLC35A1	1.4783	0.83797	2.607934	0.177136
TXLNB	0.355169	0.037206	3.390492	0.368508
CITED2	1.18947	0.778098	1.81833	0.422973
DCBLD1	0.851839	0.189663	3.825894	0.834269
SFXN1	0.719734	0.301743	1.716751	0.458398
SAMD3	0.89817	0.420407	1.918879	0.781566
PDSS2	0.428761	0.212794	0.863917	0.017827
STXBP5	0.501891	0.314102	0.801954	0.00394
IL31RA	0.416263	0.050196	3.451936	0.416763
ANKRD55	1.101837	0.379793	3.196599	0.858364
PI16	1.356227	0.280454	6.558486	0.704739
DAGLB	0.987586	0.546881	1.783432	0.966957
KIAA0895	1.266631	0.27224	5.893155	0.76317
STK17A	1.486085	0.115715	19.0852	0.761012
TRA2A	0.512738	0.260646	1.008648	0.052988
GALNT10	0.713808	0.114905	4.434265	0.717519
SAP30L	0.913269	0.418045	1.995148	0.819996
RPS14	0.892576	0.189843	4.196594	0.885584
MYOZ3	0.687086	0.389083	1.213333	0.195841
COG5	0.80099	0.169395	3.787518	0.779519
GPR85	0.663845	0.035549	12.39652	0.783828
SLU7	0.817641	0.343101	1.948514	0.649538
RP9	0.574659	0.287467	1.148769	0.116992
PTTG1	0.773802	0.476274	1.257196	0.300378
CAMLG	0.568813	0.276465	1.170302	0.12534
RELL2	2.273541	1.252782	4.126007	0.006911
KCNK5	1.599768	1.082123	2.365033	0.01849
ZNF12	0.517239	0.200943	1.331405	0.171748
SLC29A4	1.085183	0.512661	2.297079	0.830808
CDCA7L	0.587471	0.306922	1.124463	0.108311
SP8	0.409878	0.007667	21.91325	0.660423
MIOS	0.647707	0.209933	1.998374	0.449917
USP49	0.993831	0.537614	1.837193	0.984252

SYTL3	1.197734	0.66679	2.151454	0.545992
ZNF704	0.020146	0.000956	0.424424	0.012035
FABP5	0.48002	0.257404	0.895165	0.020984
TAGAP	1.089264	0.701971	1.690234	0.702895
COL1A2	0.480802	0.080266	2.880051	0.422677
SLC13A4	6.429557	0.649852	63.61324	0.111524
BRI3	1.450147	1.006972	2.088366	0.045792
LMTK2	1.896539	0.757254	4.749871	0.171826
CTSB	1.770872	1.17075	2.678616	0.006798
DLC1	0.033502	0.003763	0.298261	0.002331
ADCY1	2.050178	0.204072	20.59685	0.541945
RAD21	0.974561	0.446473	2.127272	0.948414
MED30	0.352686	0.156085	0.79692	0.012219
PHKG1	0.123196	0.003058	4.962596	0.266805
DEFA4	0.993415	0.861247	1.145867	0.927736
OSGIN2	0.637777	0.36436	1.116369	0.115353
DEFB1	1.012218	0.899373	1.139221	0.840415
OXR1	0.781648	0.43116	1.417047	0.417025
GPR146	2.520002	1.320152	4.810361	0.005079
UNCX	1.570272	0.165816	14.87042	0.694018
TMEM184/	0.319844	0.03201	3.195907	0.331729
NOS3	2.027224	0.560873	7.327215	0.281072
MICALL2	0.23302	0.058934	0.921345	0.037824
CA3	1.122587	0.296397	4.251733	0.86486
INTS1	0.754872	0.374442	1.521818	0.4318
CDK5	1.803543	0.774029	4.202383	0.171792
SLC4A2	2.476979	1.37668	4.456684	0.002473
FASTK	1.445106	0.553371	3.773833	0.452193
TMUB1	1.377658	0.754101	2.516824	0.297396
PHAX	0.63954	0.171367	2.386761	0.505881
ALDH7A1	1.204166	0.737752	1.965451	0.457339
FOXK1	3.923917	1.355694	11.35738	0.011696
COX6C	0.690218	0.255234	1.866524	0.465126
OSR2	0.078964	0.001066	5.851211	0.247792
YWHAZ	0.703442	0.369032	1.340887	0.285179
BAALC	1.116392	0.831424	1.499032	0.46404
FZD6	0.952467	0.575957	1.575105	0.849502
CTHRC1	0.842696	0.370823	1.915032	0.682804
SLC25A32	0.555162	0.147059	2.09579	0.385247
TP53INP1	0.695446	0.473193	1.022088	0.064493
INTS8	0.987302	0.426542	2.285276	0.976192
FREM1	4.797426	1.084221	21.22749	0.038779
GEM	1.793946	0.151614	21.2265	0.642946
TMEM67	1.966076	0.281578	13.72784	0.495361
SNAPC3	0.223825	0.048205	1.039255	0.056029
NUDT2	0.83077	0.409304	1.686227	0.607723
TMEM65	1.204358	0.453487	3.198497	0.709053
PSIP1	0.291977	0.089571	0.951764	0.041155
UBAP1	0.992343	0.478745	2.056931	0.983511
SYK	1.035214	0.619692	1.729355	0.894836
NIPSNAP3	0.097209	0.016504	0.572572	0.009987
ABCA1	1.235529	0.791321	1.929093	0.352175
NFIL3	0.767585	0.564495	1.043741	0.091617
LETM2	9.064628	0.654625	125.5183	0.10018
METTL2B	0.392061	0.095185	1.614876	0.194835
FXN	0.126632	0.006092	2.632397	0.181949
TMEM71	0.962156	0.643131	1.439433	0.851107
MAMDC2	1.153092	0.973226	1.3662	0.0997

CPA6	3.501711	1.00173	12.24081	0.049684
ALDH1A1	1.119415	0.84861	1.476638	0.4247
KDM1B	1.359073	0.497851	3.71011	0.549324
HGSNAT	1.845984	0.108704	31.34797	0.671397
GKAP1	0.450978	0.17353	1.172021	0.102212
KIF27	0.42796	0.041104	4.455748	0.477705
HNRNPK	0.536049	0.125849	2.283286	0.399049
TRPV6	3.357183	0.073992	152.3225	0.533795
ANKS6	19.94465	1.680107	236.7641	0.01774
FBP1	1.197154	0.96652	1.482822	0.099336
ZHX1	0.290947	0.058061	1.457957	0.133241
CYBB	1.080041	0.870818	1.339533	0.483375
DYNLT3	0.918046	0.379135	2.222977	0.849694
MID1IP1	1.824642	1.084574	3.069702	0.02346
NCF1C	1.101547	0.933689	1.299582	0.251561
KIAA1958	0.602783	0.252854	1.436983	0.253442
PCDH19	0.308734	0.022631	4.211729	0.378046
PIGA	0.357977	0.105767	1.211599	0.098655
OR1K1	0.343101	0.037969	3.100404	0.34086
STRBP	0.394637	0.185049	0.841606	0.016118
CLDN3	1.109719	0.099284	12.40362	0.932635
GAPVD1	0.662832	0.295123	1.488689	0.319184
ATP7A	1.044028	0.40558	2.687498	0.928833
ZNF367	1.407733	0.140684	14.08627	0.77104
HDX	1.314665	0.159518	10.83481	0.799321
NDUFB6	0.218686	0.080841	0.591573	0.002754
NOL6	0.541378	0.160654	1.824358	0.322178
AQP3	1.455106	0.450197	4.703124	0.530895
VCP	0.33674	0.1245	0.910793	0.032031
PIGO	0.677943	0.150917	3.045418	0.612089
STOML2	0.395444	0.175412	0.891476	0.02529
BRWD3	0.728595	0.220901	2.403116	0.603047
SLITRK5	1.701884	0.968755	2.989827	0.064378
MELK	0.635777	0.313431	1.289638	0.209448
OTUD1	1.336386	0.880821	2.027571	0.172781
ARHGAP1:	0.857349	0.331188	2.219425	0.751131
HECTD2	0.032816	0.002986	0.360599	0.005206
SLC7A3	1.053449	0.364385	3.045552	0.923416
FBXO33	0.83352	0.485746	1.430284	0.50863
LRRC18	3.295732	0.431599	25.1665	0.250207
WRN	0.1865	0.059733	0.582294	0.003841
TSHR	3.206617	0.011346	906.2663	0.685749
CFL2	2.281321	0.215291	24.17386	0.493468
SUGT1	0.585327	0.220857	1.551267	0.281471
GTF2A1	0.402768	0.050008	3.243904	0.392895
ZCCHC24	1.765602	0.6686	4.662501	0.251204
PGM2L1	1.241712	0.463571	3.326022	0.666722
SLC16A9	1.029783	0.754794	1.404957	0.853105
FOLR2	1.67	1.067037	2.613687	0.024841
INPPL1	0.738861	0.378505	1.442292	0.375177
PHOX2A	2.840303	0.242908	33.21138	0.405373
GJB2	3.708989	0.926334	14.85059	0.064047
CRYL1	3.716087	1.453225	9.502526	0.006139
REEP3	1.667036	0.388797	7.147719	0.491418
PCF11	0.772554	0.341079	1.749857	0.536167
RPL10L	0.792187	0.136484	4.598046	0.795146
RPL36AL	0.888873	0.553368	1.427792	0.626135
ZNF22	0.673473	0.380284	1.192705	0.175215

KLHDC2	0.39698	0.188013	0.838205	0.015401
RPUSD4	0.870525	0.406537	1.86407	0.721152
ARF6	1.874854	0.817847	4.297966	0.137568
TTC8	0.697191	0.234107	2.076295	0.517103
TMEM63C	0.189796	0.023577	1.527881	0.118374
AKR1E2	1.184978	0.174247	8.058528	0.862237
KBTBD6	3.559023	0.523442	24.19875	0.194266
FAAH2	0.393026	0.034917	4.423884	0.449607
NUDT5	1.03492	0.499386	2.144754	0.926443
DACT1	2.710062	0.801811	9.159808	0.108607
PRPF18	0.472613	0.190854	1.170335	0.105236
TAF3	3.562718	0.201307	63.05287	0.386152
VDAC2	1.872465	0.542941	6.457648	0.320688
COMTD1	2.093023	1.190198	3.680688	0.010332
SLC18A2	1.798589	0.569565	5.679637	0.31705
PDZD8	0.602348	0.407009	0.891438	0.011259
ZNF503	1.273626	0.8423	1.925826	0.251599
QSOX2	0.445719	0.218727	0.908281	0.026092
NSD1	0.279131	0.044572	1.74804	0.17279
PRDX3	1.104689	0.606719	2.011373	0.744695
ENOX2	0.186785	0.024437	1.427722	0.105916
GHITM	0.515479	0.240407	1.105286	0.088617
CLEC1B	1.004549	0.724583	1.392689	0.978275
SNAPC4	0.68733	0.320851	1.472405	0.334744
PMPCA	0.549814	0.259833	1.163425	0.117781
TSC1	0.233757	0.043106	1.267626	0.091981
GFI1B	0.906689	0.594479	1.382864	0.64923
HPRT1	0.133559	0.043466	0.410391	0.00044
ZMYND19	0.515837	0.218385	1.218432	0.131181
STOX1	2.906074	0.387901	21.77172	0.29914
RET	2.046985	0.224239	18.68605	0.525484
DDX21	0.734814	0.470296	1.14811	0.175944
BMS1	0.188121	0.060972	0.580424	0.003657
STK32C	2.979332	1.011412	8.776259	0.047642
FUNDC2	0.173916	0.032063	0.94335	0.042605
NDRG2	1.471179	0.876696	2.468776	0.143819
ZNF219	1.267475	0.849405	1.891317	0.245766
CASP7	0.550713	0.07114	4.263239	0.567798
METTL3	0.672592	0.3799	1.190787	0.173564
SALL2	3.031651	0.846322	10.85983	0.088444
PRAP1	2.995746	0.393479	22.80806	0.289426
TRUB1	0.885992	0.129777	6.0487	0.901704
ZFYVE1	67.42923	7.024326	647.2793	0.000263
FRAT1	1.382109	0.819587	2.330717	0.224842
UBTD1	1.478774	0.978437	2.234965	0.06338
E2F7	1.178246	0.396841	3.498283	0.767675
ISCA2	1.083122	0.465429	2.520583	0.853007
PACSIN3	1.213884	0.363955	4.048622	0.752474
TTC7B	5.064991	1.273875	20.13866	0.02124
SLC39A13	0.595858	0.080643	4.402711	0.611878
PSMC3	0.592804	0.25336	1.387026	0.227961
RAPSN	0.969236	0.076474	12.28421	0.980761
AGBL2	2.907496	0.49085	17.22222	0.239627
TC2N	0.611698	0.273841	1.366394	0.23066
CPSF2	1.411157	0.596425	3.338835	0.433149
MOAP1	0.719899	0.347803	1.490082	0.375923
IFI27L1	0.776258	0.399588	1.507995	0.454742
IFI27	1.207524	0.968439	1.505632	0.093917

CLMN	9.176108	1.074657	78.35148	0.042789
PDZRN4	1.18604	0.31823	4.420361	0.799351
CCDC38	0.724091	0.071944	7.287683	0.784059
PTER	3.057046	0.529558	17.64779	0.211569
C1QL3	0.051792	0.001036	2.588833	0.137978
CACNB2	1.102592	0.009789	124.1956	0.96768
ARL5B	1.032184	0.273561	3.894565	0.962709
TAF1D	0.691073	0.372738	1.28128	0.240762
ABTB2	0.50148	0.19485	1.290648	0.152438
AMOTL1	1.43132	0.108412	18.89708	0.785334
HTRA1	3.407432	1.528178	7.597672	0.002731
LIPC	1.090652	0.23337	5.097158	0.912169
CEP57	0.736994	0.403972	1.34455	0.319814
TCP11L2	1.127933	0.56533	2.250427	0.732652
SPRED1	2.143896	1.424649	3.226262	0.000255
GPR176	0.855899	0.101305	7.231269	0.88636
JAM3	1.237363	0.923596	1.657723	0.153488
CMTM5	1.252289	0.788992	1.987633	0.339845
ADAMTS1	0.412864	0.041594	4.098057	0.449983
GPT2	0.855839	0.553407	1.323545	0.484037
AMN	3.167683	0.174716	57.4317	0.435449
RAB8B	1.418302	0.831454	2.419351	0.199651
IKBIP	3.79578	1.148557	12.5444	0.028738
RPUSD2	1.296921	0.631113	2.66514	0.479264
HIF1AN	0.662139	0.248938	1.761195	0.408804
NDUFB8	0.37049	0.102661	1.337054	0.129426
ZFYVE19	0.97386	0.353867	2.680117	0.959102
SPINT1	1.686654	0.771103	3.689262	0.190518
FBN1	0.106433	0.012028	0.941839	0.044028
AVPR1A	0.250781	0.015714	4.002157	0.327735
DEPDC4	0.111811	0.00344	3.634501	0.217406
LRTM2	0.271143	0.015454	4.757212	0.371908
BRD7	0.484171	0.214328	1.09375	0.081082
CKB	1.365339	0.921619	2.02269	0.120442
TRMT61A	0.759802	0.31921	1.808526	0.534706
BTRC	0.133692	0.018672	0.95722	0.045124
POLL	2.509472	0.842597	7.473859	0.098457
BAG5	0.21268	0.041598	1.087377	0.062978
API5	0.793554	0.414405	1.519594	0.485431
ZNF319	1.063522	0.506341	2.233831	0.870795
HPS6	1.883867	1.038237	3.418254	0.037215
SENP8	2.945283	0.377898	22.95512	0.302501
NOLC1	0.266403	0.122349	0.580067	0.000863
ALKBH3	0.133328	0.051536	0.344935	3.26E-05
COPS2	1.164058	0.506271	2.676493	0.720637
SGPL1	4.587996	1.699493	12.38588	0.002642
FRS2	0.335429	0.020583	5.466354	0.443019
CCT2	0.63697	0.387096	1.048139	0.075907
PCBD1	1.537413	0.748708	3.156954	0.241353
ARIH1	0.450903	0.191121	1.063793	0.068949
COX11	0.747211	0.287842	1.939687	0.549353
ZNF202	0.627395	0.194796	2.020706	0.434694
STXBP4	1.398686	0.120247	16.26923	0.78869
CYYR1	0.71131	0.436586	1.158906	0.171374
CUL5	0.703967	0.339758	1.458597	0.344966
C2	1.037143	0.730467	1.472574	0.838421
PLEKHF1	1.416943	0.831523	2.41452	0.200009
SMPD1	1.993663	0.744776	5.33676	0.169624

APBB1	9.173126	0.1922	437.8061	0.261122
SYNPO2L	3.872028	0.491524	30.50229	0.198608
NUDT13	1.102848	0.122813	9.903426	0.930343
TRIM44	0.71073	0.407374	1.239983	0.229176
ILK	5.314964	1.443928	19.56389	0.011988
TAF10	1.778338	0.813724	3.886435	0.148965
TPP1	1.222572	0.739801	2.020384	0.432993
DCHS1	1.971044	1.026369	3.785204	0.041536
CYB5A	1.199755	0.507408	2.836792	0.678301
USP54	1.939172	0.722914	5.201708	0.188352
RAG1	2.290211	0.236926	22.13802	0.474057
WDR88	4.940146	0.796155	30.65363	0.08631
ATP9B	0.222239	0.053103	0.930075	0.039474
PPFIBP2	1.3609	0.951183	1.947102	0.091775
CYB5R2	0.079829	0.003445	1.849931	0.114942
SERPINB8	0.880632	0.544322	1.424731	0.604556
RIC3	1.761981	0.112492	27.59818	0.686568
IDH3A	1.476664	0.641071	3.401392	0.359884
CRABP1	1.07197	0.523659	2.194405	0.849204
PLD4	0.780429	0.259584	2.346327	0.658909
ZMAT1	0.598276	0.025751	13.89993	0.748908
XRRA1	1.429596	0.236101	8.656232	0.697305
TRIM66	0.867864	0.360251	2.090733	0.752064
RNF169	1.996238	0.344362	11.57202	0.440722
RPL27A	0.54362	0.029888	9.887628	0.68047
CDYL2	1.617524	0.31831	8.219597	0.562049
CENPN	0.483034	0.29858	0.781441	0.003029
ATMIN	1.139303	0.435929	2.977575	0.790185
TMEM41B	0.575209	0.257498	1.284921	0.177463
LEO1	0.656872	0.215377	2.003374	0.460098
ZNF143	0.689151	0.250694	1.894459	0.470552
TMX3	0.796771	0.446488	1.42186	0.441985
MFAP4	0.918644	0.653559	1.291249	0.625201
WEE1	0.376656	0.185506	0.764773	0.00689
MAPK7	2.245779	1.028931	4.901715	0.042196
PRKCB	1.04375	0.577651	1.88594	0.887189
NDST2	1.113665	0.406163	3.053578	0.834299
MCM7	0.27433	0.130472	0.576805	0.000647
CCDC68	3.619967	0.159829	81.98844	0.419022
CLEC4E	3.572701	0.742992	17.17946	0.112016
ZNF3	0.629297	0.100895	3.925032	0.619964
CLEC4D	1.165006	0.634781	2.138121	0.622027
ZSCAN21	0.568026	0.190198	1.696408	0.310969
RIMKLB	0.879177	0.383262	2.016774	0.761146
TK2	1.673538	0.794345	3.525834	0.175611
TMED3	0.463735	0.242764	0.885842	0.019963
SEC11C	1.025325	0.5356	1.962829	0.93983
TMEM135	1.258877	0.379365	4.177434	0.706781
IQCD	0.06199	0.002386	1.610395	0.094277
NDEL1	1.271298	0.556881	2.902229	0.568707
CENPV	0.49603	0.295274	0.833278	0.008071
RRAD	0.527632	0.029249	9.51816	0.664855
HSP90B1	0.647112	0.386402	1.083726	0.098058
BLCAP	1.789553	0.769965	4.159283	0.176228
CHRFAM7	0.842988	0.134684	5.276252	0.855166
ATF7IP2	0.734266	0.340528	1.583264	0.430755
TMPRSS5	0.047497	0.003237	0.696858	0.026181
COG1	0.595918	0.185482	1.914567	0.384692

PLEKHA7	1.494926	0.290244	7.69974	0.630671
ZNF606	1.987576	0.446659	8.844468	0.367144
B2M	1.12126	0.445143	2.82432	0.808141
ZNF592	1.559765	0.15141	16.06805	0.708725
NNMT	1.773474	0.964288	3.26169	0.065331
ACSM1	0.706617	0.091611	5.450308	0.739012
AP1G1	1.184838	0.669043	2.098283	0.560801
SLFN5	5.476732	0.77848	38.52972	0.087564
CATSPER2	0.913184	0.369664	2.255847	0.843962
SAAL1	0.295741	0.122869	0.711836	0.006559
YPEL4	0.962356	0.655891	1.412015	0.844484
PPIB	1.207601	0.916363	1.591401	0.180353
LDHC	1.448542	0.115463	18.17273	0.774006
LDHAL6A	0.390091	0.059798	2.54474	0.325204
FAM111A	0.552093	0.314433	0.969385	0.038619
KIF7	1.603764	0.588642	4.369478	0.355653
LDHD	0.655735	0.086838	4.951621	0.682459
PEX11A	1.272023	0.477356	3.389595	0.630404
TMEM170/	0.929375	0.43853	1.969623	0.848426
MESP1	1.238398	0.720624	2.128194	0.438942
ANPEP	1.551749	1.072957	2.244196	0.019594
RBPMS2	0.783903	0.614208	1.000482	0.050454
NAV2	1.031625	0.039555	26.9055	0.985071
ANKDD1A	0.569934	0.28052	1.157937	0.120061
DCTN5	0.681439	0.338776	1.370694	0.282079
TERF2IP	0.884878	0.431101	1.816303	0.738871
PLK1	0.607785	0.245122	1.507013	0.28249
CLPX	0.416637	0.160871	1.079039	0.071343
GPR182	1.087033	0.273102	4.326739	0.905745
ZBTB39	1.187247	0.322945	4.364685	0.796104
TAC3	0.390025	0.050685	3.001305	0.365814
MYO1A	0.260229	0.023806	2.844605	0.26993
NAB2	3.967573	1.963493	8.017158	0.000123
VPS39	1.606738	0.563995	4.577362	0.374662
STAT6	1.585699	0.779546	3.225521	0.20318
PATL1	0.980048	0.433886	2.213704	0.961336
STX3	0.481442	0.273248	0.848267	0.011426
MRPL16	0.623331	0.25606	1.517385	0.297725
PIP4K2C	0.723732	0.232351	2.254297	0.577001
MTMR10	0.49388	0.151082	1.61447	0.243072
YWHAB	0.917443	0.392278	2.145675	0.842442
SCG5	0.733948	0.124889	4.31326	0.732111
TSC22D4	2.216407	1.082415	4.538425	0.029515
MS4A7	1.247287	0.972536	1.599658	0.081754
MS4A14	1.333241	0.856804	2.074607	0.202342
DIS3L	0.397584	0.201729	0.783591	0.007712
CCNDBP1	0.719541	0.182433	2.837966	0.638272
EPB42	0.996351	0.831138	1.194405	0.968479
SMAD3	1.837436	1.008515	3.347664	0.046848
MAP1A	1.368481	0.97744	1.915964	0.06769
RCCD1	0.16362	0.043273	0.618665	0.00764
AKTIP	1.143768	0.552819	2.366425	0.717264
MAPRE2	1.287827	0.549306	3.01926	0.560655
MBD6	0.609325	0.319844	1.160807	0.131937
CNPY4	0.26829	0.044158	1.630044	0.152947
PDIA3	0.096661	0.006415	1.456465	0.091358
NUDT21	0.821084	0.396184	1.701679	0.595986
NKX3-1	1.246233	0.725319	2.141262	0.425402

DUSP18	1.782707	0.967768	3.283892	0.063618
TEF	1.462074	0.486409	4.394776	0.498738
MEI1	2.743774	0.379069	19.85995	0.317582
PBX3	1.067103	0.860702	1.323	0.553721
GNGT2	1.76496	0.713196	4.367781	0.219121
PHB	0.759171	0.38114	1.512147	0.433207
SNRPD1	0.195415	0.073957	0.51634	0.00099
TTC16	0.310809	0.062241	1.552073	0.154386
SAMD14	0.907836	0.495281	1.664038	0.754465
PIP5KL1	1.432849	0.288545	7.11521	0.660028
TMEM92	0.568806	0.029692	10.89638	0.708013
FAM102A	1.129902	0.519789	2.456147	0.757866
ACSF2	2.22093	1.184294	4.164955	0.012875
GOLGA2	0.429341	0.138846	1.327619	0.142118
TRUB2	0.983521	0.461596	2.095581	0.965658
COQ4	0.39519	0.106174	1.470942	0.166212
SLC27A4	0.743092	0.118706	4.651706	0.751015
URM1	1.297218	0.46542	3.615606	0.618789
CERCAM	0.759225	0.406121	1.419334	0.388177
DOLPP1	0.599592	0.268604	1.338439	0.211859
ENDOG	1.241922	0.756612	2.038523	0.391504
SP2	1.731482	0.923719	3.245608	0.086814
PRR15L	0.272624	0.047219	1.574034	0.146265
COQ7	0.222529	0.063623	0.778318	0.018659
GPRC5B	2.49296	0.28538	21.77747	0.40878
CRK	1.77063	0.599338	5.230994	0.301265
FBXO22	0.106762	0.014698	0.77546	0.027013
TBC1D2B	2.061334	1.164549	3.648707	0.013034
NOD2	1.329303	1.026298	1.721769	0.031035
SNX20	1.102048	0.282471	4.299593	0.888741
LOXHD1	0.905415	0.195069	4.202489	0.899046
KATNAL2	0.150881	0.013024	1.747901	0.130234
HDHD2	0.384492	0.208064	0.710521	0.002283
ZNF91	0.864977	0.542218	1.37986	0.542703
RNF214	0.411217	0.115896	1.459061	0.169045
DPEP2	1.248985	0.716525	2.177126	0.43292
POP5	0.32465	0.11644	0.905164	0.031522
CD3D	0.815651	0.562726	1.182257	0.281958
TBC1D16	1.473751	0.88685	2.449051	0.134504
ACAA2	0.414958	0.212416	0.810628	0.01004
STIM1	1.155443	0.622697	2.143977	0.646886
RRM1	0.464863	0.278961	0.77465	0.003282
TRIM68	0.502474	0.236579	1.067216	0.073342
FN3K	0.200863	0.021089	1.913078	0.162763
IRGQ	0.669956	0.045894	9.779999	0.76965
ZNF226	0.313454	0.09429	1.042038	0.058387
ZNF180	3.070734	0.268896	35.06717	0.36657
PPP2R3B	1.048831	0.381617	2.882595	0.926359
ZNF668	0.791818	0.377223	1.662083	0.537235
ZNF646	2.682966	1.230641	5.849234	0.01307
VKORC1	1.28892	0.554759	2.994656	0.555141
GNG8	1.076341	0.31009	3.73605	0.907759
LPO	5.420244	0.435575	67.44889	0.188884
CA4	0.995149	0.70237	1.409971	0.978178
TPM4	1.394014	0.775181	2.506864	0.267237
RAB8A	1.108744	0.423229	2.904606	0.833604
GPX4	2.030284	0.990492	4.16162	0.053128
MIDN	1.190646	0.658602	2.152496	0.563546

JSRP1	0.889238	0.580166	1.362961	0.590046
KLHL26	5.090481	1.623081	15.96531	0.005264
GATAD2A	1.467916	0.600691	3.587162	0.399802
MVD	2.457794	1.09221	5.530758	0.029772
CDT1	0.656552	0.434119	0.992955	0.046211
TRAPPC2L	0.932936	0.443173	1.963948	0.854969
ANKRD11	0.580337	0.165502	2.034963	0.395291
PROCA1	0.932958	0.197325	4.41105	0.930233
RPL13	1.159876	0.560733	2.399202	0.689199
ZNF641	1.405669	0.7684	2.571457	0.269145
CACNB3	1.328905	0.956012	1.847246	0.090599
DHRS13	0.857533	0.508701	1.445571	0.564033
TP53I13	0.928692	0.528373	1.632309	0.797106
CORO6	1.749459	0.842592	3.632371	0.133489
RHEBL1	0.325345	0.063382	1.670028	0.178476
TUBA1A	0.773621	0.314081	1.905527	0.576791
TUBA1C	0.49644	0.194336	1.268177	0.143339
ZNF610	2.886284	0.545815	15.26274	0.212245
ZNF528	0.0743	0.00156	3.538464	0.187215
ZNF701	1.332737	0.421366	4.215307	0.624908
SERTAD3	1.000418	0.238839	4.190415	0.999544
RAB4B	2.055506	1.009154	4.18678	0.047137
GPD1	2.507571	0.125125	50.25314	0.547802
CYP2S1	2.330567	1.534753	3.539031	7.19E-05
AXL	1.729297	0.238683	12.52906	0.587763
NFKBID	2.017831	0.732263	5.560358	0.174646
TMC4	1.04945	0.33195	3.317802	0.934499
LAIR1	1.792102	0.568869	5.645634	0.319031
TTYH1	1.630181	0.747955	3.553009	0.218929
LENG8	1.459612	0.556014	3.83168	0.4425
CDC42EP5	0.822716	0.199886	3.38623	0.786908
LAIR2	1.283276	0.807643	2.039017	0.291102
ZNF526	1.106362	0.48479	2.52488	0.810256
TRAPPC9	1.57741	0.923342	2.694799	0.095301
KIR3DL1	0.927587	0.394855	2.179075	0.863044
ZNF146	0.927705	0.3138	2.742632	0.89207
ZNF283	1.772364	0.241031	13.03264	0.573964
PPP1R14A	1.901918	1.22369	2.956054	0.004274
SPINT2	1.286391	0.998877	1.656664	0.051027
YIF1B	2.123097	1.017334	4.430738	0.044883
PSCA	0.354269	0.03684	3.406749	0.368887
ATCAY	0.387977	0.023023	6.538172	0.511172
DAPK3	1.912894	0.930244	3.933554	0.077836
EEF2	0.964034	0.426325	2.179936	0.929887
TMIGD2	0.657051	0.36266	1.190416	0.166015
CHAF1A	0.515885	0.235099	1.132022	0.0988
UBXN6	1.345355	0.839737	2.155416	0.217342
SEMA6B	1.721257	0.346126	8.559681	0.506967
ZNF444	1.934522	0.956337	3.913241	0.066393
NXN	1.088092	0.310408	3.814156	0.895045
GLOD4	0.231454	0.075834	0.706422	0.010157
MFSD3	0.763006	0.440576	1.321405	0.334376
GPT	0.125855	0.023684	0.668793	0.015016
KIFC2	1.000093	0.549452	1.820333	0.999757
SLC43A2	1.625341	1.166754	2.264174	0.004081
RILP	1.639967	0.600761	4.476811	0.334315
SERPINF2	1.284935	0.667838	2.472244	0.452734
WDR81	1.380144	0.845004	2.254188	0.198043

SRR	0.550047	0.183038	1.652942	0.286982
TSR1	0.458116	0.149927	1.399813	0.170753
HSD11B1L	0.663463	0.188921	2.329983	0.522065
CYB5D2	0.842654	0.286262	2.480476	0.755961
KLK1	0.89374	0.180957	4.414145	0.890351
ZNF83	0.800216	0.452788	1.414228	0.443029
KRT80	1.229712	0.217485	6.953086	0.815031
KRT1	0.915099	0.558589	1.499146	0.724625
OTUB1	1.781927	0.635666	4.995176	0.272006
RCOR2	1.663875	0.841956	3.288152	0.142925
ANGPTL4	0.113179	0.010772	1.189158	0.069432
CD320	0.726428	0.443209	1.190629	0.204856
SPRYD3	1.155538	0.81675	1.634855	0.414168
IGFBP6	1.076279	0.681345	1.700132	0.752664
ZNF558	0.697012	0.319626	1.519982	0.364196
NDUFV1	1.410993	0.676313	2.943756	0.358824
CDK2AP2	1.585749	0.928961	2.706895	0.091051
NUDT8	0.678738	0.152507	3.020755	0.610952
TBX10	0.275117	0.032021	2.363728	0.239574
PRDX2	0.955633	0.685714	1.3318	0.788713
ZNF232	1.649552	0.46442	5.858967	0.438955
MIS12	1.107072	0.586184	2.090827	0.753866
CD300C	1.151252	0.88448	1.498486	0.294984
CD300A	1.281601	0.867753	1.892821	0.21239
TMEM88	1.731565	0.77251	3.881267	0.182467
EVPL	1.414342	0.86056	2.32449	0.171452
SRP68	0.846101	0.28043	2.552817	0.76677
MGAT5B	0.088925	0.005724	1.381591	0.083806
TMC8	1.075729	0.552866	2.093081	0.829812
TK1	0.869895	0.583372	1.297142	0.494139
TMEM68	0.231699	0.028164	1.906163	0.173828
TMEM99	0.903558	0.250425	3.260121	0.876897
GHDC	1.047816	0.582894	1.883564	0.875956
ZNF598	2.085417	0.783913	5.547758	0.140946
RAB26	0.535799	0.060268	4.76343	0.575657
MLST8	1.121999	0.482764	2.607654	0.789064
E4F1	0.615678	0.278982	1.358722	0.22977
DNASE1L2	0.166743	0.027743	1.002168	0.050277
CASKIN1	0.136237	0.017123	1.083956	0.059595
ABCA3	1.153062	0.486759	2.731437	0.746183
KCTD5	2.338202	0.969382	5.639871	0.058659
SRRM2	1.023357	0.410237	2.552821	0.960517
ZNF597	0.441557	0.174309	1.118548	0.084757
NLRC3	0.29801	0.112766	0.787564	0.014622
SDHAF2	0.41918	0.141517	1.241636	0.116574
DDB1	0.155747	0.050737	0.478095	0.001156
VPS37C	1.864565	1.159319	2.998832	0.010179
VWCE	1.116149	0.711714	1.750407	0.632196
RAB3IL1	0.99032	0.752868	1.302662	0.944554
BEST1	1.311069	0.706481	2.43305	0.39059
FTH1	1.168609	0.851036	1.604688	0.335534
BSCL2	0.809326	0.396756	1.650909	0.56081
POLR2G	0.240599	0.097314	0.594857	0.002038
SLC3A2	1.107405	0.610466	2.008867	0.737062
ATG16L2	0.851897	0.521411	1.391855	0.522214
C2CD3	0.058855	0.010063	0.344233	0.00167
TTC21A	0.784288	0.228418	2.69291	0.699461
RPSA	0.994033	0.442851	2.231228	0.988425

CTNNB1	0.175656	0.031981	0.964784	0.045369
ULK4	0.205994	0.026316	1.612486	0.132356
FADD	2.083192	1.092388	3.972664	0.025863
LTBP3	3.40915	0.738936	15.72844	0.115917
NAALADL1	1.260744	0.918943	1.72968	0.150988
SAC3D1	1.180839	0.740659	1.882621	0.484882
BATF2	2.92758	0.821938	10.42747	0.097438
SF1	0.82901	0.387316	1.774409	0.629114
MAP4K2	1.057428	0.528153	2.117101	0.874732
CCDC88B	1.249605	0.712773	2.190756	0.436623
SCARA3	0.675415	0.257662	1.77048	0.424793
PBK	0.702775	0.337732	1.462379	0.345471
PNOC	1.136373	0.484434	2.665677	0.768851
COPS6	0.865376	0.281527	2.660054	0.800757
PAFAH1B2	1.504949	0.176382	12.84071	0.708631
ANKS3	2.268812	0.580192	8.872079	0.238992
NUDT16L1	1.508737	0.754661	3.016307	0.244595
KIAA1586	1.373235	0.599461	3.145783	0.453277
RAB4A	1.729183	0.699741	4.273114	0.235446
OR2B2	0.315779	0.054032	1.845493	0.200647
SETD5	1.348036	0.324639	5.597612	0.680967
VASN	1.069633	0.482197	2.372714	0.868471
THAP9	0.295962	0.056114	1.560999	0.151268
RNF187	2.561438	0.808823	8.111744	0.109774
HOOK3	1.405278	0.485845	4.064687	0.530097
MAPK1IP1	1.247202	0.126869	12.26079	0.849749
DDIT4	1.350912	1.040843	1.753352	0.023768
RBPJ	0.432794	0.113975	1.643427	0.218614
LMBRD1	1.88013	0.975845	3.622386	0.059175
ZCCHC4	1.052338	0.11615	9.534377	0.963814
PTGDR	1.015899	0.248478	4.153484	0.982484
TTC39C	1.240616	0.664484	2.316276	0.49851
GLYCTK	1.788845	0.626613	5.106765	0.277206
GNG4	1.690507	0.862984	3.311547	0.125912
UBTD2	4.895553	0.580605	41.27843	0.144248
POLR2J3	1.368736	0.435603	4.300796	0.591032
NKIRAS2	0.542535	0.078393	3.754717	0.535556
DNAJC7	1.035067	0.410116	2.612342	0.941832
KCNV2	3.000802	0.336153	26.78786	0.325178
IRF2BP2	0.884875	0.500938	1.563072	0.673513
NT5DC2	1.156067	0.740382	1.805135	0.523561
FOXI1	0.670793	0.005628	79.95762	0.869969
BMI1	0.910983	0.598244	1.38721	0.663906
THAP11	1.042186	0.360682	3.011383	0.939161
MMADHC	0.451966	0.234695	0.870378	0.017541
PDHB	0.469836	0.180014	1.226269	0.122773
PXK	0.506128	0.196221	1.305492	0.158968
PCMTD1	0.776067	0.428056	1.407012	0.403651
KCTD6	1.317776	0.487517	3.561997	0.586508
ACOX2	1.149566	0.73673	1.79374	0.539202
IRF2	3.867311	1.328276	11.25978	0.013116
CX3CR1	1.143708	0.9428	1.38743	0.173085
XIRP1	2.179312	0.10889	43.61662	0.610367
DEGS2	2.354569	0.677313	8.185277	0.177955
ARF4	0.591641	0.22551	1.552209	0.286188
FILIP1L	2.943902	0.534144	16.22513	0.215023
DTYMK	0.739437	0.150718	3.62775	0.709898
TAP1	1.116232	0.743425	1.675991	0.595944

ING5	0.66091	0.082154	5.316866	0.697056
ATG4B	0.737933	0.18682	2.914814	0.66458
MLKL	1.516894	0.624549	3.684207	0.357426
RFWD3	0.486387	0.223993	1.056158	0.068477
RHOH	0.822843	0.461319	1.467686	0.508981
KLHL30	1.195225	0.206798	6.908	0.842078
COG7	1.229302	0.310306	4.869987	0.768816
CDC40	0.691002	0.309895	1.540795	0.366327
STIP1	1.227406	0.543876	2.769978	0.621724
HR	0.742174	0.018503	29.7699	0.874217
RAB31	1.150364	0.933091	1.418229	0.189672
REEP4	2.765604	1.128878	6.775372	0.026073
TNXB	13.26232	0.259219	678.5341	0.197916
BMP1	17.25691	2.109748	141.1548	0.007903
ATXN2L	0.183914	0.027599	1.22558	0.080159
POLR3D	0.641634	0.276072	1.491259	0.302426
FEN1	0.519063	0.276922	0.972933	0.040799
HEXIM2	1.114698	0.462375	2.687321	0.808896
FNTA	0.594085	0.126074	2.799434	0.510281
SERINC2	1.292297	0.736617	2.267163	0.371273
COL3A1	2.070859	0.492078	8.714984	0.320791
ING2	0.512415	0.214109	1.226333	0.13317
CDKN2AIP	0.878277	0.487031	1.583822	0.66615
SNRNP48	0.256433	0.04565	1.440475	0.122229
SLC20A2	0.647616	0.229712	1.825792	0.411329
TMUB2	0.572272	0.153942	2.127397	0.40477
STAT3	0.87654	0.356961	2.152394	0.773735
ZSWIM1	2.686151	1.08247	6.66569	0.033101
ADAM9	0.378479	0.145394	0.985227	0.046541
AXIN2	1.337107	0.616971	2.897797	0.461626
NDUFS5	0.846094	0.464853	1.540003	0.58443
ZNF30	0.685424	0.253801	1.85108	0.456172
UGT3A2	0.159794	0.041979	0.60827	0.007169
SLC16A4	2.721424	0.071368	103.7736	0.589944
IL7R	0.973822	0.6907	1.372997	0.8797
TMEM208	3.435479	1.290863	9.143118	0.013467
AHCYL1	0.571396	0.260558	1.253056	0.162439
DNAJC21	0.805151	0.069978	9.263886	0.861956
PKIG	0.683386	0.184678	2.528814	0.568503
FAM178B	0.743989	0.487839	1.134637	0.169639
SEMA4C	1.785897	0.714606	4.463195	0.214631
CNNM3	0.558642	0.185749	1.680119	0.300019
GSTM4	0.974141	0.3569	2.658866	0.959214
TET2	0.715441	0.337922	1.514713	0.381585
TCTN2	0.621616	0.097126	3.978422	0.615683
SHOX2	0.062533	0.002344	1.668409	0.098034
TSPAN5	1.286245	0.913374	1.811334	0.149528
ABHD15	1.560399	0.865836	2.812131	0.138716
ZBTB5	0.620301	0.293111	1.312725	0.21183
CHTF8	1.674478	0.197696	14.18276	0.636284
ADAL	1.02232	0.212574	4.916591	0.978022
LCMT2	2.103764	1.00777	4.391697	0.047639
SNTB2	1.058275	0.533649	2.098654	0.871193
IL12A	0.973867	0.631794	1.501151	0.90453
ZNF507	0.207757	0.043518	0.991848	0.048812
STX18	0.186853	0.042945	0.812991	0.025356
GFM1	0.568601	0.271691	1.189978	0.134042
DDX19A	0.380253	0.12866	1.123832	0.080323

ANKRD49	1.257724	0.629546	2.512717	0.516076
SFTPB	0.272039	0.010949	6.759003	0.427082
USP39	0.19944	0.058045	0.685263	0.010463
TNIP2	5.602354	1.426303	22.0054	0.013562
TMEM150/	1.002732	0.467053	2.152803	0.994415
RNF181	1.234071	0.561979	2.709944	0.600249
VAMP5	1.706671	1.249074	2.331906	0.000789
LRRC28	0.460495	0.264172	0.802719	0.006238
MAT2A	0.472367	0.298853	0.746622	0.001323
ENHO	2.723751	0.819709	9.050551	0.10195
ZNF608	0.646649	0.225982	1.85039	0.41638
INPP5D	0.720532	0.348186	1.491061	0.377055
LETM1	0.45467	0.120731	1.712278	0.244016
TMEM129	2.846044	0.911719	8.884284	0.071729
PPIC	1.251191	0.487047	3.214229	0.641555
SPRY3	1.407609	0.143062	13.84972	0.769459
CEP120	1.001076	0.346239	2.894396	0.998415
STXBP6	1.867196	0.44528	7.829729	0.393231
MFF	0.611613	0.334544	1.11815	0.110226
LGALS9	2.063213	1.205852	3.530155	0.008216
SIGLEC7	0.8964	0.417477	1.924736	0.779081
E2F6	2.618741	0.402016	17.05854	0.313994
FEM1B	0.626828	0.342076	1.148614	0.130643
COMMD8	1.073474	0.636606	1.810143	0.790274
UQCRFS1	0.492375	0.170332	1.423302	0.1908
MAP2K1	1.112778	0.669505	1.849536	0.680176
HNRNPH1	0.449365	0.280182	0.720708	0.000904
IRS1	0.908554	0.093058	8.870518	0.934258
MECP2	3.321123	1.087394	10.14339	0.035114
UPF3A	0.398012	0.183543	0.863088	0.019659
ROR2	0.501346	0.030098	8.351117	0.630443
BRD7P3	0.24075	0.013961	4.151599	0.327009
AR	2.968645	0.721901	12.20785	0.131487
DHRSX	1.359148	0.346873	5.325525	0.659649
HSPBAP1	0.542931	0.269738	1.092815	0.087029
ASMTL	1.103233	0.60732	2.004087	0.747021
SLC25A6	1.860458	0.864195	4.005236	0.112539
CHST14	0.916304	0.488074	1.720259	0.785639
PARM1	0.611546	0.213216	1.754036	0.360331
CSNK1G1	0.0755	0.009068	0.62857	0.016878
FAM110B	0.82505	0.533037	1.277035	0.388238
AFAP1L2	1.042125	0.360439	3.013058	0.939282
ZNF354A	1.527901	0.357011	6.538957	0.567696
ATF5	1.29297	0.722905	2.312576	0.386407
UBE2V2	0.768542	0.233136	2.533536	0.665341
ZBTB43	0.92954	0.531332	1.626188	0.797919
XPO6	0.707511	0.284928	1.756833	0.455898
MN1	1.298871	0.979873	1.721719	0.068978
APEX2	1.211038	0.666421	2.200733	0.529809
NSMCE1	0.673933	0.266691	1.70304	0.404099
CCDC126	1.366408	0.650114	2.871912	0.410084
IL13	0.319357	0.019632	5.195086	0.422493
CD2BP2	1.333796	0.514573	3.457258	0.553374
RGS14	2.199584	1.237144	3.910755	0.007258
TBC1D10B	1.454965	0.779672	2.715146	0.238771
LMAN2	2.122565	0.758291	5.941359	0.151827
RAB24	1.241336	0.659461	2.33663	0.502926
PRELID1	1.588849	0.708446	3.56335	0.261204

THBS3	0.728945	0.355638	1.494105	0.387912
CA5B	1.215874	0.815737	1.812289	0.337132
EFNA1	1.788639	1.122027	2.851296	0.01453
CXCL10	1.147533	0.740157	1.779127	0.538498
SH3TC2	6.175037	0.098454	387.299	0.388608
CXCL11	0.043723	0.001113	1.717594	0.094693
ZRSR2	0.420323	0.150187	1.176343	0.098808
NMD3	0.796466	0.46684	1.358833	0.403743
ADRB2	1.478068	0.95538	2.28672	0.079268
B3GALNT1	0.516125	0.298855	0.891354	0.017667
GPRIN1	0.325629	0.032477	3.264937	0.34011
KCNAB1	0.414118	0.005469	31.35743	0.689652
MRPL1	1.164058	0.673252	2.012665	0.586598
SHE	11.29382	1.517221	84.0684	0.017933
PGM2	1.002494	0.486492	2.065797	0.994613
P2RY12	2.294431	0.760592	6.921472	0.14043
SLC33A1	1.602584	0.732784	3.50482	0.237504
SNUPN	1.399261	0.611635	3.201144	0.426244
CRADD	1.082891	0.511811	2.291183	0.835023
SIN3A	0.68187	0.348475	1.334233	0.263555
ARL13B	1.241167	0.141842	10.86066	0.845219
RNASE2	1.027659	0.822021	1.28474	0.810713
RNASE3	0.992779	0.844064	1.167695	0.930246
PTK2	1.521528	0.947566	2.443151	0.082376
PTAFR	1.209579	0.916966	1.595567	0.178136
PTPN9	3.356242	0.428527	26.28624	0.248903
RNASE6	1.122896	0.871571	1.446693	0.369908
SCN9A	1.394402	0.797087	2.439329	0.243957
SDC2	0.253403	0.034436	1.864681	0.177632
CD52	1.341368	1.066236	1.687495	0.012157
MMGT1	0.647335	0.172088	2.435049	0.519986
TM2D2	1.440753	0.669919	3.098535	0.349975
HTRA4	1.192505	0.142629	9.970419	0.870918
PLEKHA2	0.219279	0.031366	1.532967	0.126169
CLIC4	1.181053	0.61022	2.285874	0.621369
GPR183	1.884734	1.055479	3.365507	0.032154
CCDC8	1.34202	0.244047	7.379796	0.735173
ZEB2	1.46207	0.864923	2.471491	0.156139
PCBP1	0.735358	0.274367	1.970906	0.541126
HINT1	0.939559	0.455822	1.936658	0.865847
DTWD2	0.638583	0.186377	2.187975	0.47534
VPREB1	0.852208	0.660113	1.100204	0.219754
CLIC3	1.207571	0.852989	1.709551	0.287585
INO80E	1.835813	0.955543	3.527008	0.068233
DFFB	0.286282	0.100923	0.81208	0.01871
NFU1	0.552922	0.183388	1.667084	0.292653
CKAP2L	0.650482	0.394734	1.071928	0.091523
APLF	0.493883	0.01633	14.93718	0.685068
BOLA2B	0.267615	0.045287	1.581404	0.145856
RGPD8	0.759431	0.298404	1.932732	0.563675
HIC2	0.978539	0.501447	1.909548	0.949288
LUZP1	1.164206	0.774875	1.749154	0.464169
BUB1	0.62068	0.36083	1.06766	0.084817
SPNS1	1.526571	0.803739	2.899472	0.196202
LRRC45	1.340047	0.426415	4.211215	0.616357
AGPAT2	2.553761	1.265139	5.154921	0.008891
ASPSCR1	1.673602	0.708791	3.951721	0.240083
GP9	1.048723	0.849541	1.294605	0.658001

FASN	1.13101	0.743063	1.721502	0.565703
CNBP	0.376307	0.069109	2.04905	0.258344
MT1E	1.585491	1.092821	2.300268	0.015205
DUS1L	1.099545	0.517237	2.337419	0.805197
GPS1	2.026533	0.626841	6.551635	0.238076
RFNG	1.901223	0.68614	5.268094	0.216613
DCXR	1.37317	0.687645	2.742105	0.36881
ZNF32	0.715717	0.157167	3.259282	0.665431
LDB2	0.432585	0.07068	2.647554	0.36462
RAC3	1.35564	0.696571	2.638296	0.370449
NRG4	1.059853	0.722865	1.55394	0.765903
LIMS1	1.736432	0.549757	5.4846	0.347009
TAPT1	0.634612	0.29782	1.352268	0.238749
UGP2	0.942453	0.472924	1.87814	0.866217
HNRNPF	0.555681	0.176116	1.75329	0.316241
BTD	0.348769	0.110017	1.105645	0.073557
CSGALNA(	0.669879	0.395762	1.133858	0.13567
PCDH7	0.132278	0.000509	34.37312	0.475808
ROBO1	1.741991	0.023694	128.0707	0.800168
AVEN	0.564995	0.310257	1.028888	0.061923
P2RY1	0.269145	0.053898	1.344006	0.10968
CTNND2	0.085784	0.006645	1.107479	0.059871
TRIM56	1.339496	0.584213	3.071227	0.489941
AHSP	0.990507	0.845986	1.159717	0.905642
WNT10B	0.436527	0.099568	1.913822	0.271681
REPS2	0.332662	0.10909	1.014426	0.053017
SYAP1	0.804215	0.435735	1.4843	0.485895
ITGAM	1.13658	0.9067	1.424744	0.266812
PYDC1	10.89172	0.694773	170.7459	0.089014
TPST1	0.97031	0.579292	1.625263	0.908823
TOR1AIP2	0.998064	0.200482	4.968692	0.998112
S100G	0.108728	0.005363	2.204264	0.148408
TM4SF1	1.461206	1.215143	1.757095	5.55E-05
OTUD3	1.413934	0.254847	7.844749	0.691954
GUSB	0.535803	0.290895	0.986902	0.045256
BRD3	1.222523	0.585955	2.550643	0.592334
KLF13	1.900895	1.032764	3.498767	0.039062
ZFPM2	2.86716	0.224483	36.62024	0.417675
ZNF764	3.440045	0.94694	12.497	0.0605
ZNF747	1.124381	0.347061	3.642685	0.845025
ZNF768	1.658391	0.763538	3.601999	0.20117
TAS1R3	5.005245	0.5562	45.04218	0.150816
TMEM42	0.799885	0.347687	1.840208	0.599397
MAP3K2	0.716341	0.195064	2.630644	0.615221
PUSL1	1.622896	0.752922	3.498093	0.216563
SF3B5	1.754873	0.865268	3.559102	0.119034
ZNF35	1.045923	0.475036	2.302887	0.911222
IFFO2	2.106483	1.132568	3.917883	0.018615
NLGN2	1.064994	0.436139	2.600576	0.890049
MYO7B	0.270702	0.062167	1.178754	0.081707
CHD3	2.469751	0.322183	18.9323	0.384284
TMEM154	1.105598	0.728901	1.676974	0.636726
MYRIP	2.013543	0.042784	94.76402	0.721716
ALCAM	0.767947	0.453501	1.300423	0.325862
YWHAG	1.351128	0.660221	2.765056	0.41014
UBE2E3	1.239164	0.295278	5.200273	0.769497
CNTROB	0.498648	0.153323	1.621739	0.247502
TRAPPC1	1.270469	0.538775	2.995855	0.584421

KCNAB3	0.004215	0.00036	0.049392	1.33E-05
FAM153A	0.148599	0.005095	4.334154	0.267943
GPR37L1	0.743702	0.059887	9.235558	0.817794
TMEM192	0.229243	0.048571	1.08198	0.062823
ZNF778	3.496287	0.393974	31.02747	0.261129
NIPA1	0.806452	0.478068	1.360401	0.420066
FOXD4	0.070515	0.007275	0.683501	0.022119
GPR25	0.454231	0.160066	1.289001	0.138097
UBE2E1	0.486312	0.227515	1.039491	0.062881
HNRNPA3	0.332849	0.133343	0.83085	0.018423
SIK2	0.227277	0.035658	1.448621	0.116931
RNF150	3.428436	0.729798	16.10606	0.118544
CCDC144/	0.043162	0.000959	1.942062	0.105619
CHRNB1	0.503504	0.249197	1.017336	0.055867
GYPA	0.903313	0.68021	1.19959	0.48231
USP38	0.945772	0.450328	1.986296	0.882921
SLC16A5	1.18976	0.454706	3.113063	0.723299
NANP	0.727783	0.132421	3.999863	0.714752
PWWP2A	1.169599	0.363551	3.762776	0.792725
USP50	0.105528	0.003488	3.193053	0.196142
USP47	0.985558	0.393915	2.465825	0.975198
PDCD6IP	0.764892	0.220739	2.650453	0.672514
ZNF212	0.872794	0.384125	1.983125	0.745247
MRAP	0.093692	0.006407	1.3701	0.083647
FAM161A	0.259975	0.060697	1.113516	0.069509
ZNF282	1.537177	0.827694	2.854817	0.173441
GLB1	0.915656	0.319683	2.622683	0.86964
CRTAP	0.392419	0.107064	1.438326	0.158098
CNGB3	0.049013	0.003568	0.673207	0.024072
CMTM8	1.226165	0.865935	1.736251	0.250609
STX8	0.87456	0.43254	1.768289	0.70905
UBB	0.865418	0.385392	1.943342	0.726185
NFRKB	0.470085	0.168228	1.313578	0.149944
FABP4	1.14798	0.824072	1.599201	0.414539
PRDM10	0.502165	0.145613	1.731781	0.275471
B3GNT2	0.661233	0.358306	1.220266	0.185775
FOS	1.448721	1.002086	2.094425	0.048719
TMED10	0.97539	0.554413	1.716025	0.93111
SETMAR	0.696706	0.435147	1.115483	0.132355
SMAD1	11.04628	0.956957	127.5086	0.054266
EMX2	0.978729	0.360082	2.660254	0.966384
SP7	1.351265	0.137637	13.26621	0.796167
LRRN2	2.104372	0.749682	5.907014	0.157697
SLC30A1	1.177259	0.784072	1.767619	0.43132
DCLK2	1.417514	0.124461	16.14438	0.778628
ZNF804A	0.699853	0.240071	2.040207	0.513266
GPRC5C	3.709398	1.508632	9.120607	0.004293
TMEM182	0.239101	0.030875	1.851627	0.170663
KRT8	1.936241	0.988359	3.793184	0.054125
ADORA2B	1.100727	0.617038	1.963573	0.745193
MGMT	1.226469	0.867631	1.733717	0.247704
METTL7B	1.229997	0.942781	1.604711	0.127083
NFXL1	0.69786	0.308507	1.578596	0.387715
DENND5B	0.271999	0.099787	0.741411	0.010935
CD14	1.131628	0.977745	1.309729	0.097283
DNAJC18	0.864155	0.294891	2.532332	0.790115
RALGAPB	0.407169	0.171986	0.963954	0.041007
SLC23A1	0.410642	0.037499	4.496845	0.466094

KRT72	0.516267	0.213778	1.246771	0.141651
KISS1	1.058946	0.16452	6.815991	0.951926
NUDT9	0.25813	0.063655	1.046748	0.05796
HSD17B13	0.355403	0.027834	4.537954	0.425988
PA2G4	0.803382	0.384374	1.679152	0.560543
ELOVL6	0.390752	0.165964	0.920003	0.031491
PFKFB3	1.100498	0.788934	1.535104	0.572809
ARL6IP1	1.454241	0.773962	2.732455	0.244539
SERPINB9	2.516305	0.42635	14.85117	0.308304
SMAGP	0.825712	0.262621	2.596141	0.743166
IRX1	2.141268	0.716074	6.403006	0.173077
CDH2	0.946776	0.769093	1.165509	0.606042
EMB	1.63012	0.847014	3.137246	0.143497
STAT2	1.086209	0.58823	2.005763	0.791582
NUDCD2	1.012252	0.570426	1.796297	0.966807
IRF2BP1	1.756231	0.906734	3.4016	0.09498
HSPA4	0.403039	0.139663	1.163088	0.092848
COMMD5	1.920745	0.329537	11.1953	0.468007
GTSF1	0.970831	0.816925	1.153733	0.736761
DPY19L2P	0.084287	0.007688	0.924074	0.042909
ZNF16	0.732404	0.136786	3.921569	0.716029
ARMC10	0.391766	0.129297	1.187035	0.097556
RNF34	3.411423	0.66345	17.54134	0.141875
ACYP2	0.920721	0.208371	4.068355	0.913238
TRABD	0.561893	0.268987	1.173754	0.125101
ATF7	1.667769	0.227644	12.21846	0.614685
ZNF296	1.152835	0.737301	1.802559	0.532868
HOXB9	0.649845	0.235652	1.792046	0.404952
TTLL6	1.236532	0.44667	3.423137	0.682785
POLH	1.260245	0.259737	6.114725	0.77408
KIF5B	0.733248	0.372782	1.442274	0.368689
AKAP13	1.772409	0.883309	3.556439	0.107232
CDCA4	1.447372	0.566186	3.699993	0.440045
CHCHD7	0.635683	0.301635	1.339674	0.233599
HTRA3	0.458021	0.095913	2.18723	0.327651
FOXN2	0.914155	0.539597	1.548708	0.738608
LMOD2	28.56777	1.786393	456.8519	0.017779
USP32	0.300922	0.043814	2.066801	0.221894
CEL	0.754502	0.476616	1.194405	0.229377
PPM1D	0.823094	0.370776	1.827205	0.632308
GPR27	0.808837	0.320859	2.038953	0.652902
KBTBD2	0.987317	0.52622	1.852449	0.968288
TRIAP1	0.310146	0.11833	0.812902	0.017251
LSM3	1.00189	0.542324	1.850893	0.99519
KIAA0232	0.882296	0.311779	2.496787	0.813472
MTSS1	1.631438	1.121345	2.373571	0.010508
TMEM43	1.09805	0.616026	1.957246	0.751112
RNF139	0.211947	0.016308	2.754586	0.235775
RPS9	2.08319	0.738333	5.877676	0.165519
PLA2G1B	0.698312	0.308323	1.581584	0.389291
CYTL1	0.850293	0.703201	1.028153	0.094234
TSEN34	2.40772	1.153284	5.026615	0.019299
TRH	0.841644	0.69077	1.025472	0.087189
GSTA4	2.135387	0.897806	5.078914	0.086142
NDUFA3	1.487159	0.586949	3.76803	0.402773
OSCAR	1.239085	0.977875	1.570068	0.075941
PAQR8	1.277549	0.828995	1.968809	0.266976
NUDT6	1.960805	0.355754	10.80733	0.439404

TANC2	1.176935	0.451004	3.071316	0.739218
DNAJC24	0.598534	0.197537	1.813549	0.364153
ZNF160	0.920646	0.184681	4.589479	0.91965
ZNF415	2.570057	1.184324	5.57718	0.016943
CEACAM3	0.878562	0.48821	1.581023	0.66582
PDGFD	1.33059	0.674939	2.623153	0.409508
S1PR1	1.46707	0.911313	2.36175	0.114639
PKIA	0.994205	0.531454	1.859888	0.98549
XKR6	1.631817	0.194481	13.69196	0.651836
TSNARE1	0.05045	0.003144	0.809506	0.034927
FPR2	1.133055	0.753337	1.704168	0.548608
FPR1	1.130441	0.946593	1.349995	0.175772
FEZ2	1.148221	0.573599	2.298488	0.696303
SOX7	0.018514	0.000654	0.523734	0.019324
ALK	0.773975	0.200262	2.991264	0.710299
MTM1	1.078083	0.502424	2.313309	0.846955
INSR	0.444109	0.111002	1.776842	0.251223
MFN1	0.120801	0.0119	1.226262	0.073862
GIMAP8	1.45461	1.110342	1.90562	0.006537
NRTN	0.885274	0.113899	6.880717	0.907278
KCNMB3	0.239095	0.032103	1.780726	0.162497
FUT3	0.621577	0.074412	5.192119	0.660622
ATP6V0E2	1.72594	1.179944	2.524583	0.004912
PRKCE	1.556379	0.711182	3.406042	0.268281
JAGN1	1.100997	0.230564	5.257517	0.903993
TADA3	0.899458	0.327216	2.472453	0.837269
SOCS5	0.924363	0.09418	9.072474	0.946188
C1GALT1C	1.399532	0.576843	3.395534	0.457291
MORN4	1.801268	0.339306	9.562346	0.489601
ZNF672	0.631735	0.298281	1.337965	0.230315
ZNF692	0.194998	0.06897	0.551315	0.00205
NAIF1	2.174087	0.536001	8.818369	0.277012
RBKS	0.942318	0.529459	1.677112	0.839922
GRIK1	24.86917	0.309619	1997.535	0.150987
TMEM126A	0.34837	0.139509	0.86992	0.023919
TMEM126B	0.561728	0.267823	1.178159	0.126979
TRIM8	1.518212	0.866275	2.66078	0.1447
NETO2	1.179529	0.797873	1.743746	0.407762
CLDN20	0.303475	0.06034	1.526303	0.147927
CDC42BPG	1.987107	0.107514	36.72646	0.6445
SCAND1	1.703827	0.939945	3.088505	0.079108
JUNB	1.32723	1.043806	1.687612	0.020901
TMEM37	3.849648	0.650241	22.7912	0.137383
LRG1	1.207909	0.856254	1.703986	0.28194
SHCBP1	0.731179	0.201184	2.657379	0.634399
SOSTDC1	0.132046	0.013703	1.272455	0.079857
NPTX1	0.965737	0.291185	3.202942	0.954551
FAM98B	0.26156	0.005767	11.86296	0.490775
ZNF439	0.584299	0.327235	1.043302	0.069271
ZNF440	0.571548	0.048918	6.67782	0.655579
GAA	1.512583	1.078124	2.122119	0.016603
CANT1	3.175803	1.601067	6.299377	0.000943
ZDHHC16	0.409293	0.122973	1.362258	0.145372
CHST11	0.380824	0.063803	2.27304	0.289538
EXOSC1	0.365432	0.063901	2.089805	0.257845
PGAM1	1.049424	0.60227	1.828564	0.864795
CHD7	0.863062	0.603709	1.233833	0.419307
ESCO2	0.404985	0.124432	1.318091	0.133288

KRT19	1.892874	0.10104	35.46101	0.66953
CLCN5	2.420061	0.315284	18.57593	0.395369
TPPP	2.287416	0.594702	8.798143	0.22865
KCND3	11.59445	0.420273	319.8668	0.147669
APLN	9.693745	1.079722	87.03045	0.042513
PDE7B	1.824198	1.003538	3.315967	0.048661
MRPL36	0.880702	0.402455	1.927259	0.750533
ZNF581	2.382035	1.342566	4.226304	0.003008
NAT1	3.883305	0.940929	16.02678	0.060686
GLOD5	0.1778	0.020248	1.561313	0.119223
ZNF524	2.065224	1.105979	3.856447	0.02284
MCC	0.124985	0.007821	1.997287	0.141369
ZBTB26	1.057815	0.448835	2.49306	0.897756
DSEL	6.153657	0.261264	144.9396	0.259629
POLR1C	0.565908	0.27658	1.1579	0.11909
ASXL1	1.602189	0.621607	4.129636	0.329182
DLK2	0.165646	0.002093	13.10858	0.420155
ZNF562	0.402463	0.177409	0.913012	0.029427
ZNF318	1.04964	0.494458	2.228184	0.899616
ZNF561	1.507723	0.503612	4.513845	0.463007
WIPF2	0.856412	0.125939	5.823803	0.874078
HOPX	1.522488	0.973161	2.381897	0.065648
LRRC8C	1.283502	0.790136	2.084931	0.313288
RSL1D1	0.70846	0.482793	1.039606	0.078161
LRRC8D	4.298558	1.505621	12.27241	0.00644
PPID	0.904358	0.330191	2.476941	0.844957
COL24A1	0.83135	0.593179	1.16515	0.283512
ETFDH	0.587989	0.282821	1.222439	0.154995
RXFP1	2.108788	1.357762	3.275234	0.000895
LPAR3	0.327549	0.072936	1.470996	0.145291
PTGER4	1.029695	0.704328	1.505367	0.879959
TBCA	0.118465	0.033822	0.414935	0.000852
ECEL1	0.048614	0.002349	1.006	0.050454
BCL2L1	1.26022	0.903672	1.757445	0.172871
PLRG1	0.802409	0.458585	1.404015	0.440594
ZNF584	1.157332	0.24942	5.370126	0.851973
NMUR1	1.170135	0.330371	4.144477	0.807616
CLSTN1	1.406606	0.646567	3.06007	0.389606
CXXC5	1.470493	0.958752	2.255378	0.077238
ZNF274	0.277743	0.097045	0.7949	0.016949
PIK3CD	1.326591	0.766601	2.295644	0.312473
PTCRA	1.208513	0.683354	2.137258	0.514998
SLC25A33	1.723779	0.751773	3.952544	0.198416
ENC1	1.404668	0.835639	2.361177	0.199721
SPSB1	3.502792	0.822637	14.91489	0.089916
P2RY6	2.148608	0.193934	23.80465	0.533102
BPTF	0.439743	0.067624	2.859535	0.389752
S100Z	0.944007	0.488771	1.823246	0.863772
ZIK1	0.779014	0.332625	1.824469	0.565196
GPR82	4.822311	0.221976	104.7623	0.316513
GPR34	1.635831	0.489869	5.462574	0.423719
PLEKHG5	0.939073	0.00803	109.8218	0.979357
ATF7IP	12.57437	1.935892	81.67543	0.008004
RGS19	1.547776	0.997395	2.401868	0.051376
TCEA2	1.575009	0.562193	4.412459	0.387446
HDAC3	1.449106	0.519064	4.045569	0.47885
GPHN	0.627306	0.258574	1.521855	0.30241
VAT1L	0.282107	0.047809	1.664637	0.16233

TMEM51	1.337626	1.027681	1.74105	0.030542
CAMTA1	4.51668	0.767497	26.58042	0.095447
LGALS4	0.44148	0.071124	2.740336	0.380079
LRRC34	0.316402	0.084905	1.17909	0.086435
SPATA5L1	1.802662	0.866141	3.7518	0.115097
GATM	0.232484	0.073529	0.735069	0.012991
RASGRP4	4.493275	1.404581	14.37406	0.011322
SLFNL1	0.028142	0.001623	0.488022	0.014176
BCL2	0.406207	0.152972	1.078652	0.070604
UTF1	1.788951	0.97381	3.286418	0.060871
COL8A2	2.328497	1.296859	4.180794	0.004648
PWWP2B	2.709551	1.026974	7.148835	0.044037
ZNF540	0.166827	0.026615	1.045678	0.055842
ANGPTL7	0.094796	0.004704	1.910297	0.124157
FBXL14	5.313449	0.684704	41.23353	0.11012
EXOSC10	0.327937	0.115201	0.933523	0.036722
ZNF570	0.15811	0.025848	0.967152	0.045922
NINJ2	0.816004	0.583557	1.141042	0.234573
MLLT3	0.792157	0.139372	4.502438	0.792697
FAM90A1	0.529266	0.150771	1.857936	0.320666
RRM2	0.675181	0.389156	1.171433	0.162373
IFNB1	0.555561	0.112241	2.749861	0.47133
RPS21	1.629395	0.750489	3.537597	0.217094
C3AR1	1.180954	0.836338	1.667571	0.344779
PTEN	0.468165	0.27175	0.806545	0.006245
RPS7	0.775714	0.387734	1.55192	0.472875
RNASEH1	0.563025	0.272127	1.164886	0.121495
PRNP	0.898216	0.54317	1.485338	0.675739
FRMD5	0.155615	0.003331	7.268782	0.34284
CYP4F11	1.569951	0.333002	7.401592	0.568608
TLN2	0.944479	0.411586	2.167327	0.89278
ZNF217	1.401603	0.905676	2.169087	0.129696
SCG2	0.8854	0.538368	1.456129	0.63157
ATPAF2	0.56068	0.22048	1.425805	0.224352
CYP4F22	1.734248	0.400262	7.51413	0.461741
PPIH	0.567205	0.279208	1.152265	0.116873
ZNF57	0.653715	0.147568	2.895912	0.575638
JMJD1C	1.340434	0.610168	2.9447	0.465595
SYNPO	0.541175	0.066845	4.381335	0.564998
MAL	1.022572	0.737989	1.416895	0.893293
ZNF554	0.070428	0.006173	0.803463	0.032667
RAB33B	1.20822	0.759117	1.923017	0.425052
THOP1	1.283578	0.731988	2.250816	0.38364
LAMB2	1.536424	1.126033	2.096386	0.006756
USP19	2.574416	0.61836	10.71806	0.193797
ORMDL3	1.262052	0.604876	2.633225	0.535108
KLF11	1.173093	0.749737	1.835506	0.484594
SMN1	2.270812	0.44074	11.69983	0.326848
EIF2AK3	1.186654	0.356876	3.945769	0.780115
KRCC1	1.772646	0.910406	3.451507	0.092206
NME6	0.407458	0.140172	1.18441	0.099128
CYCS	0.169166	0.055001	0.520302	0.001937
CD8B	0.925486	0.0819	10.45822	0.950092
SLFN12	1.503105	0.596044	3.790538	0.387842
FRMD3	0.906214	0.609087	1.348285	0.627104
SNTB1	0.418061	0.208001	0.84026	0.014341
MTBP	0.088455	0.014927	0.524177	0.007552
MRPL13	0.694489	0.320721	1.503844	0.355031

MALT1	0.878746	0.304619	2.534948	0.811004
PRL	1.355634	0.804531	2.284241	0.253055
ISG20	1.450403	1.052431	1.998865	0.023072
MBOAT1	0.343582	0.115787	1.019539	0.054217
GPR22	0.167573	0.016428	1.709309	0.131672
CXCR6	1.032183	0.18863	5.648116	0.970861
CEBPB	1.399752	1.004912	1.949728	0.046707
AZU1	1.094245	0.914797	1.308894	0.32437
TPSAB1	1.200546	1.001511	1.439136	0.048122
PAIP1	0.591799	0.055805	6.275896	0.663253
CLEC7A	1.577938	1.139137	2.185767	0.006078
C1QTNF4	0.840217	0.667915	1.056967	0.137065
SERHL	22.9269	1.900337	276.6051	0.013691
NEGR1	0.534637	0.238519	1.19838	0.128382
ZNF131	0.671816	0.375257	1.20274	0.180672
MACROD2	0.156849	0.002955	8.325807	0.360648
DPAGT1	0.449612	0.197975	1.021095	0.056123
BSG	1.560775	0.643518	3.785468	0.324711
HINFP	0.430096	0.115862	1.596579	0.207371
SPTLC3	0.246405	0.064648	0.939168	0.04018
TP53RK	1.002015	0.600479	1.672054	0.993853
CLEC12A	0.978612	0.648877	1.475905	0.91786
BPGM	1.076145	0.703454	1.646289	0.735124
POP7	1.257503	0.607187	2.604327	0.537345
ALG14	1.181179	0.305295	4.569952	0.809389
SUCLG2	1.286454	0.969318	1.707348	0.081127
STARD5	1.934125	0.370664	10.09226	0.433878
CSDC2	2.148267	0.328818	14.03529	0.424582
IL16	1.471943	0.685437	3.160924	0.321503
GNB2	2.270824	1.321049	3.903442	0.003004
PDZD3	1.610888	0.144214	17.99387	0.698586
C2CD2L	1.196984	0.430222	3.330304	0.730545
GNG12	0.243238	0.01827	3.238375	0.284475
PRSS27	0.336799	0.04832	2.347554	0.27197
DNAJB7	0.131389	0.005385	3.205512	0.213037
CLP1	0.759019	0.272397	2.114965	0.597946
TTC36	0.288627	0.028117	2.96277	0.295636
RSPH9	4.3557	0.36215	52.38743	0.246224
GTPBP2	0.56435	0.175427	1.815517	0.337252
FGGY	1.206402	0.452669	3.21517	0.707521
IL17D	1.202386	0.659482	2.192223	0.54754
TCEAL1	0.680057	0.276565	1.672223	0.400943
ZNF24	0.768516	0.348951	1.692549	0.513361
MANEA	1.331383	0.093091	19.04142	0.832995
AGXT	1.209885	0.92547	1.581706	0.163476
AFF1	0.908522	0.358798	2.300492	0.839612
ACOT12	0.061059	0.00397	0.938978	0.04495
FIBP	1.136763	0.440024	2.936723	0.791235
BANP	1.864357	0.672078	5.171757	0.23146
PPP1CA	2.296538	1.083218	4.868908	0.030124
HCFC1	0.525886	0.228138	1.212232	0.131484
CTSW	0.767939	0.597545	0.986923	0.039132
NIPAL4	0.79833	0.011265	56.57581	0.917481
PDE3A	0.282715	0.039175	2.040263	0.210274
RASGRP1	1.040613	0.676827	1.59993	0.856058
KLHL6	0.997656	0.529461	1.879869	0.994206
CHCHD1	1.390695	0.535897	3.608961	0.497871
MRPL52	0.661553	0.36529	1.198095	0.172721

SMPDL3A	1.307717	0.924023	1.850737	0.130018
RND1	0.29731	0.024962	3.541175	0.337247
RAD9A	0.650543	0.276272	1.531849	0.325138
EFEMP2	0.466155	0.188178	1.154761	0.099134
OR10AD1	2.07396	0.359221	11.97399	0.414813
TMEM134	2.0922	1.019273	4.29453	0.044221
ZMAT3	0.829856	0.489984	1.405476	0.487818
ZNF738	1.003911	0.541554	1.861012	0.990109
SLFN11	0.93855	0.486662	1.810036	0.84989
CORO1B	1.646434	1.029124	2.634031	0.037553
FUT10	1.268211	0.091151	17.64498	0.859602
LRRC20	2.12514	0.787591	5.734222	0.136622
MUS81	0.61603	0.210974	1.798763	0.375556
TMEM217	2.22131	0.678794	7.2691	0.187022
ZNF596	1.975922	0.108252	36.06648	0.64581
CFL1	2.26531	0.791036	6.487224	0.127687
TMCC1	0.315274	0.026695	3.723501	0.359491
RAB43	1.690043	0.572743	4.986956	0.341863
CBWD1	1.725293	0.195615	15.21681	0.62341
RAB37	0.681187	0.331489	1.399793	0.296143
DCP2	0.804637	0.451396	1.434306	0.461116
SNX32	1.75609	0.314254	9.813249	0.521257
RPL38	16.69614	0.38596	722.2542	0.143015
CYP7B1	0.181263	0.029661	1.10772	0.06443
RARG	0.065304	0.005833	0.731083	0.02682
CES3	6.903961	1.100571	43.30906	0.039184
SSH3	0.794663	0.205233	3.076941	0.739321
CES2	0.759662	0.322478	1.789537	0.529496
PDP2	0.744785	0.107952	5.138421	0.764927
SP3	3.153695	0.994277	10.00303	0.05115
DMXL1	0.741538	0.347423	1.582732	0.439513
ZNF621	0.635466	0.356846	1.131628	0.12357
EGFL7	1.093848	0.757268	1.580028	0.632587
NADSYN1	0.773516	0.361603	1.654652	0.508012
DHCR7	1.401864	0.842406	2.332869	0.193604
NBEA	2.076512	0.596658	7.22676	0.250819
RNASEH2(	0.456665	0.185852	1.122092	0.087484
MYEOV	1.941501	0.370016	10.18719	0.432776
ANKRD13I	1.139552	0.311987	4.162294	0.84332
MYD88	1.422339	0.822662	2.459151	0.207251
MRGPRD	1.791736	0.172277	18.63461	0.625488
OXSR1	0.42701	0.200399	0.909871	0.027477
SLC22A13	0.185337	0.009021	3.807923	0.274408
PHF8	3.390942	0.069279	165.9735	0.538464
LCLAT1	0.370852	0.088722	1.550138	0.174054
KAT5	5.700077	1.242735	26.14464	0.025117
SH3RF3	1.422029	0.335465	6.027947	0.632804
GXYLT2	4.895249	0.488766	49.0285	0.176688
HPSE2	6.263059	0.799554	49.05976	0.080645
DCAKD	0.331453	0.173007	0.63501	0.000872
TADA2B	1.404703	0.771515	2.557551	0.266347
CCDC96	4.438108	0.594011	33.15897	0.146405
RELA	1.958135	0.779178	4.920948	0.152928
EVC2	4.988643	0.466784	53.31495	0.183637
ZNF680	0.971915	0.536136	1.761903	0.925224
RXFP4	2.149651	0.277861	16.6306	0.46347
HPSE	2.180463	1.244681	3.819788	0.006428
COQ2	0.600086	0.273045	1.318845	0.203691

HSPA6	1.133995	0.845875	1.520255	0.400469
TRMT112	2.345789	1.054334	5.21915	0.036651
LRRN3	1.84836	0.566894	6.026577	0.308337
ADCK5	2.021223	0.813	5.025021	0.129917
NOC3L	0.815795	0.426889	1.559005	0.537806
ESRRA	3.87881	1.751111	8.591784	0.000836
COMMD1	0.519428	0.185655	1.453261	0.212088
RAPH1	19.38906	1.649576	227.8983	0.01837
MTX1	1.017586	0.446417	2.319536	0.966921
PARP14	1.095266	0.630417	1.902878	0.746784
CYSLTR1	1.545676	0.897114	2.663109	0.116691
PARP15	0.852343	0.330426	2.198648	0.741062
CKS1B	0.487827	0.287156	0.828729	0.007936
ABCD2	0.477984	0.006047	37.78496	0.740592
ABLIM3	0.817587	0.339287	1.970158	0.65357
VANGL1	1.249948	0.31184	5.010164	0.752796
GLRX	1.101092	0.720956	1.681661	0.655805
IQCB1	0.530353	0.174994	1.607339	0.262258
GOLGB1	0.767698	0.456156	1.292014	0.319572
ZNF483	2.173077	0.411185	11.48452	0.360863
PLAC8L1	0.064953	0.006311	0.668441	0.021527
SLC2A14	1.200234	0.829048	1.737609	0.333623
GPR137	1.573706	0.795006	3.115135	0.193088
SNCG	0.123293	0.00657	2.313862	0.161756
MMRN2	0.184462	0.019229	1.769558	0.142858
TNKS	1.28546	0.175693	9.405076	0.804668
ZNF449	0.085481	0.008037	0.909161	0.041459
PPP1R3B	0.880009	0.054703	14.1567	0.928142
STOX2	0.829943	0.500003	1.377603	0.470944
MAP3K11	2.381675	1.366163	4.15205	0.002212
TRIB1	1.129308	0.914354	1.394796	0.258975
KCNK7	0.954751	0.080902	11.26737	0.970669
C1QB	1.337884	1.091148	1.640413	0.005132
C1QA	1.781784	1.152941	2.753616	0.009302
OLR1	0.963979	0.765556	1.213832	0.755054
GLIPR1L1	0.086639	0.007979	0.940731	0.044413
DAG1	1.689889	0.470858	6.064937	0.420982
ARV1	0.498141	0.250507	0.990569	0.046925
SAA1	0.184594	0.004621	7.373676	0.369162
EHBP1L1	1.35166	0.844437	2.163554	0.209304
THAP2	1.713048	0.1511	19.4211	0.663927
RNF26	1.38786	0.650304	2.961931	0.396766
PPP1R14B	1.194757	0.674948	2.114896	0.541382
SMARCC1	0.722748	0.457909	1.140759	0.163196
ZNF417	0.409189	0.059892	2.795639	0.362085
PTPRM	1.318703	0.756165	2.299732	0.329577
FKBP2	1.840458	0.528882	6.404619	0.337668
VEGFB	1.236856	0.317421	4.819506	0.759355
TNFRSF10	0.604527	0.180317	2.026719	0.414817
MST1	0.968241	0.558758	1.677812	0.908396
TNFRSF10	0.408298	0.055851	2.984833	0.377481
GMPPB	0.954241	0.240661	3.783644	0.946866
ZNF622	1.03513	0.370315	2.893464	0.947511
CSPG4	2.378275	0.761694	7.425806	0.135862
SNX33	2.767507	1.363717	5.616339	0.004816
CHD2	0.451086	0.083342	2.441496	0.3555
CCDC106	1.465442	0.914346	2.348697	0.112311
CCR9	1.142676	0.068772	18.98601	0.925891

SULT1B1	1.723403	0.811982	3.657862	0.156325
NUDT4	0.035578	0.000959	1.320012	0.070392
PC	5.563715	1.384795	22.35343	0.015573
NMNAT1	0.881645	0.230034	3.379061	0.854205
LRFN4	1.446856	0.856615	2.443794	0.167201
SLC19A1	1.525256	0.799178	2.910998	0.200482
HSPB7	1.705085	0.09242	31.45765	0.719757
RCE1	2.202332	0.856942	5.659971	0.101132
UQCRH	1.022984	0.492076	2.126697	0.951473
TAS1R1	0.208898	0.004608	9.470807	0.421008
EIF1AX	0.618188	0.2621	1.45806	0.271946
PSMD1	0.546636	0.220036	1.358011	0.19331
SUSD5	1.517213	0.135	17.05139	0.735576
HEG1	2.923077	1.343751	6.358604	0.006828
TOMM20	0.520118	0.29843	0.906488	0.021091
AGFG1	0.666703	0.281235	1.580503	0.357277
STAT5B	0.524012	0.208132	1.319304	0.170138
CD7	0.780429	0.6092	0.999784	0.049801
CNP	1.911557	0.259854	14.06193	0.52454
JUP	0.981312	0.626118	1.538007	0.934422
HAP1	0.007676	9.70E-05	0.60741	0.028998
EIF1	0.676139	0.392624	1.164383	0.158192
RNF213	1.035575	0.689938	1.554366	0.866027
TIGD3	0.646615	0.054085	7.730659	0.730537
PLK3	1.527874	0.61901	3.77118	0.357831
NET1	1.058943	0.583929	1.920371	0.850424
DPY19L1	0.844147	0.442511	1.61032	0.607143
PHOSPHO	1.180547	0.544213	2.560928	0.674423
ZNF791	1.120035	0.626293	2.00302	0.702301
PHC3	0.769887	0.345298	1.716565	0.522677
GPR160	0.734297	0.462092	1.166852	0.191227
CBX2	0.853546	0.412456	1.766347	0.669551
SPTBN2	0.519994	0.087109	3.104083	0.473145
GOLIM4	0.521456	0.219841	1.236876	0.139527
RBM4B	0.785186	0.438709	1.405298	0.415477
HOXB2	1.274442	1.030274	1.576476	0.02543
SLCO4C1	0.767188	0.303452	1.939607	0.575456
RBM4	0.719733	0.137774	3.759907	0.696623
UBXN2A	0.72679	0.383072	1.378913	0.328743
RAX2	1.583616	0.63393	3.956015	0.325042
TCAP	0.997359	0.055627	17.88212	0.998567
CCS	1.268938	0.620291	2.595883	0.514258
KLHL15	0.451101	0.242245	0.840027	0.012091
FBXO45	1.541761	0.408443	5.819728	0.522962
GNG5	1.651266	0.780637	3.492889	0.189488
SLC25A30	0.69449	0.109269	4.414027	0.699214
CD34	0.974838	0.733995	1.294708	0.860283
CTSF	1.450483	0.936433	2.246717	0.095756
MSRB3	0.770109	0.489551	1.211452	0.25843
LEMD3	1.094486	0.512328	2.338148	0.815669
TLR10	0.701918	0.183482	2.685222	0.605132
TLR1	1.188656	0.817665	1.727972	0.365261
TLR6	1.257646	0.596142	2.653186	0.547262
FAM174A	1.242938	0.450113	3.432238	0.674745
RGMB	0.649412	0.026339	16.01203	0.791788
FAM53A	1.460979	0.670712	3.18238	0.339873
CYB561D1	0.710526	0.310083	1.628104	0.419194
ZDHHC24	1.507455	0.773024	2.939651	0.228415

SELP	0.961404	0.464128	1.991472	0.915634
CTU2	2.992355	0.589803	15.18165	0.185907
MGA	0.178109	0.025657	1.236416	0.080934
ARL13A	1.814616	0.125262	26.28767	0.662195
PIGG	0.746477	0.201977	2.758866	0.6611
PRPF8	0.880343	0.409588	1.892158	0.744087
ADCY6	2.012628	1.062488	3.812439	0.031879
PITPNA	0.791931	0.283602	2.211392	0.656143
DDX23	0.672282	0.240094	1.882442	0.44974
ZNHIT2	2.431573	1.367212	4.324529	0.002489
ZBTB4	2.965039	1.213457	7.244965	0.017107
TNK1	0.797683	0.117376	5.421027	0.817166
ZHX3	0.629285	0.214053	1.850009	0.399882
PHLDA3	1.25796	0.528925	2.991851	0.603653
SLC16A11	0.298981	0.022816	3.917811	0.357707
SLC16A13	1.519907	0.129622	17.82199	0.7389
STAG3L3	0.675825	0.473407	0.964792	0.030982
SLC6A19	3.003005	0.208609	43.22942	0.419017
SNHG11	0.586541	0.165955	2.073037	0.407539
EXO1	0.303054	0.08228	1.116205	0.072702
RALGAPA1	0.50072	0.194463	1.289297	0.151742
LIG4	4.648604	0.435362	49.63574	0.203473
GTF2IRD2I	0.634547	0.213628	1.884821	0.412865
ABRA	0.074952	0.002695	2.084272	0.126738
ATP2A2	2.223716	0.863137	5.729002	0.097896
ZWILCH	0.166141	0.042787	0.645119	0.009508
RPL4	0.645111	0.327831	1.26946	0.204389
SNAPC5	0.264249	0.068038	1.026296	0.054547
ZCCHC12	0.513777	0.156684	1.684704	0.271715
GALNTL6	1.762414	0.238396	13.02916	0.578758
LINGO2	0.867179	0.076464	9.834662	0.90843
BBS1	1.40609	0.573266	3.448815	0.456573
DENND4A	0.727993	0.233757	2.267202	0.583883
PELI3	0.856377	0.215041	3.410421	0.825945
TTC9B	0.191216	0.009459	3.865324	0.2808
MYO1H	1.205124	0.123395	11.76973	0.872514
TMEM81	0.277409	0.034191	2.250758	0.229963
MRPL11	1.042554	0.374166	2.904908	0.93647
IL20RB	0.699292	0.066617	7.340616	0.765566
AKIRIN1	0.533114	0.233448	1.217449	0.135447
MSL2	1.136082	0.276553	4.667032	0.859524
ZNF497	0.662579	0.083513	5.256804	0.696891
CMKLR1	2.96221	0.748228	11.72729	0.121907
ANGEL2	0.658393	0.317516	1.365227	0.261322
UGT8	0.572025	0.014059	23.275	0.767679
KY	3.352619	0.148294	75.7958	0.447036
IQCK	1.185286	0.652333	2.153659	0.576919
ZNF266	0.619635	0.208624	1.840382	0.388831
SLC29A2	1.55412	0.979408	2.46607	0.061256
BRSK2	0.206798	0.031384	1.36266	0.10136
TMEM167A	1.275366	0.750272	2.167958	0.368893
SH3PXD2E	1.923493	0.61689	5.997541	0.25957
LARP7	1.181271	0.64693	2.15696	0.587624
FGFBP3	0.787757	0.148614	4.175665	0.779208
NR1D2	0.766679	0.249862	2.352491	0.642317
BRMS1	3.016833	0.931968	9.765663	0.065416
RPL15	0.93882	0.524195	1.681403	0.831852
HRAS	1.205245	0.370548	3.920185	0.756393

WDR49	1.668655	0.989624	2.813602	0.054753
SRP72	0.567519	0.281136	1.145628	0.113964
PCP2	0.561105	0.105003	2.998378	0.499177
RIN1	1.488485	0.768663	2.882393	0.238135
THAP6	0.740249	0.141887	3.862	0.721206
CEP135	0.539057	0.301982	0.962252	0.036607
FZD4	0.175412	0.009488	3.24292	0.242201
CD248	1.435792	0.894351	2.305022	0.134222
PDZK1	1.24622	0.04696	33.07232	0.895311
PDE12	0.674248	0.248406	1.830109	0.439126
GLMN	0.114744	0.019582	0.672376	0.016396
YIF1A	1.041531	0.524287	2.069068	0.907502
CNIH2	51.15503	1.002128	2611.28	0.049876
NLRP6	0.345065	0.018564	6.413989	0.475486
NDUFA11	2.095282	1.041536	4.215126	0.038072
RSRC1	0.478777	0.208411	1.099884	0.082632
RAB1B	2.699246	1.269829	5.737725	0.009856
PTDSS2	1.345229	0.676523	2.674915	0.397752
SEZ6L2	1.999595	0.57015	7.012854	0.279092
ASPHD1	5.231047	0.122001	224.2919	0.388206
KCTD13	1.127105	0.521142	2.437657	0.761114
P2RY14	1.708088	0.882844	3.304736	0.111854
AMZ1	0.231607	0.014537	3.689981	0.300392
GPR171	1.945712	0.18136	20.87446	0.58246
FUT1	0.398017	0.06504	2.435708	0.318879
DHX36	0.430424	0.175851	1.053529	0.064923
FBXW8	0.061516	0.003145	1.203186	0.066057
KLC2	25.86324	0.593658	1126.754	0.091186
SLC22A1	0.055743	0.00341	0.911274	0.042853
CTBP2	0.247553	0.024837	2.467361	0.234008
CHST2	1.674787	0.916673	3.05988	0.093538
ZDHHC14	1.067085	0.59826	1.903302	0.825931
ATR	1.368947	0.025566	73.30219	0.877113
UBE2C	0.754027	0.43397	1.310128	0.316524
GK5	0.504119	0.236396	1.075042	0.07628
VCPIP1	1.350089	0.556391	3.276009	0.506895
DES	0.305731	0.03199	2.921877	0.303497
PDIK1L	0.548201	0.283522	1.059966	0.073962
RAG2	0.064884	0.003702	1.137115	0.061204
TRAF6	0.311339	0.064672	1.498823	0.145595
ZNF654	1.223628	0.37968	3.943492	0.735352
MRPS22	0.57073	0.206529	1.577175	0.279515
PACS1	2.294847	1.262197	4.172344	0.006462
WFDC5	44.94171	1.787601	1129.871	0.02072
MARCKSL1	0.944639	0.700911	1.273119	0.708367
SH3BP5L	1.378371	0.655242	2.899548	0.397683
YPEL2	1.360571	0.822741	2.249983	0.230248
ABO	0.644079	0.151172	2.744136	0.551907
PSMD2	0.610135	0.16684	2.23127	0.455168
FAM131A	1.516585	0.624465	3.683199	0.357624
CSRP2	0.659852	0.350976	1.240552	0.196796
INHBC	7.112774	0.723272	69.94821	0.092533
PARL	0.469311	0.166254	1.324796	0.153073
DDIT3	0.912493	0.659652	1.262248	0.580154
PCCA	2.026419	1.249023	3.287668	0.004229
DCTN2	2.237375	0.495573	10.10112	0.295045
NPPA	1.004253	0.105266	9.580722	0.997058
ZNF408	1.100227	0.365694	3.31014	0.86504

CTDSP2	1.878533	0.875059	4.032741	0.105759
CKAP5	0.611388	0.220904	1.692114	0.343488
ARHGAP1	1.557031	0.745991	3.249828	0.238236
MED16	1.123506	0.598922	2.107566	0.716734
GOLGA8A	0.90166	0.446879	1.819266	0.772552
TP53I11	2.630306	1.021204	6.774853	0.045131
DOLK	3.126893	1.620166	6.03485	0.000678
PHYHD1	3.340187	0.641007	17.4052	0.15216
CATSPER1	0.862232	0.554395	1.341001	0.51065
CCNE2	0.509004	0.160511	1.614124	0.251446
CST6	0.179386	0.001447	22.24181	0.484769
ZNF519	0.344384	0.051177	2.31744	0.273116
LSM1	0.264623	0.069548	1.006858	0.051183
BANF1	1.370182	0.524124	3.58197	0.520647
APOF	0.252446	0.046775	1.362448	0.109512
TMEM9B	0.989306	0.385523	2.538695	0.98216
NRIP3	0.717113	0.404614	1.270967	0.25479
PTPN2	0.949001	0.183185	4.916353	0.950267
EIF1AD	0.555979	0.151269	2.043465	0.37675
SMAD2	0.645007	0.034192	12.16766	0.769832
EIF3F	1.260968	0.447328	3.554528	0.660996
ZNF25	0.74632	0.407562	1.366648	0.343144
ARL10	0.016713	0.000467	0.598134	0.024992
CLTB	2.366264	0.687339	8.146199	0.172082
LPL	0.80725	0.514465	1.26666	0.351565
RFESD	0.873422	0.608817	1.253031	0.462355
CCDC14	0.68804	0.445898	1.061676	0.091117
TBC1D10C	0.948735	0.512242	1.757175	0.867093
SART1	0.160307	0.021319	1.205418	0.075328
PPP2R2D	0.147716	0.040494	0.538845	0.003775
MCTP1	1.704	0.614853	4.722457	0.305463
POLD4	1.997934	0.966891	4.128429	0.061617
OR52W1	0.179786	0.016247	1.98944	0.161775
LRRC25	1.303104	1.005697	1.688462	0.045184
DPP10	1.501749	0.252269	8.939864	0.655044
CLCF1	1.117446	0.599435	2.083105	0.726748
TSGA10IP	1.892969	0.143658	24.94354	0.627625
GPR152	0.096757	0.005262	1.779256	0.115924
UBQLNL	0.269677	0.096421	0.754253	0.01251
KCNE3	1.99701	0.304497	13.09718	0.471043
CABP4	1.728134	0.583102	5.12165	0.323701
ALG10B	0.988253	0.209029	4.672293	0.988106
DRAP1	1.601917	0.816047	3.144596	0.170922
LONRF3	1.440103	0.482052	4.302227	0.513655
UCP3	0.004069	0.000149	0.110787	0.001095
UCP2	1.411264	0.952273	2.091487	0.086105
PAAF1	0.631871	0.355827	1.122066	0.117146
MRPL48	0.652821	0.324681	1.312596	0.231432
RAB6A	1.070878	0.504794	2.271779	0.858362
P2RY2	1.048426	0.496451	2.21411	0.901326
FOSL1	1.465962	0.696548	3.085277	0.313696
ERCC4	0.063748	0.003042	1.335703	0.076149
CCDC85B	1.377576	0.742167	2.556991	0.310073
TMEM70	1.221762	0.662479	2.253209	0.52127
RPS6KB2	1.368545	0.567129	3.302447	0.485138
TOM1L2	0.870636	0.127016	5.967805	0.887827
ZNF77	0.899795	0.345835	2.341089	0.828654
B3GNTL1	0.556052	0.32619	0.947895	0.031037

MLXIP	0.167439	0.013347	2.100537	0.166101
AURKAIP1	2.317068	0.955801	5.617069	0.062899
TTLL11	1.961287	0.248673	15.46869	0.522647
TOMM5	1.261664	0.666796	2.387232	0.474997
SLC35E3	0.881826	0.45324	1.715682	0.71113
ZNF169	0.11041	0.018124	0.67261	0.016842
RUVBL1	0.791227	0.294895	2.122927	0.641912
SFN	0.24355	0.01538	3.856786	0.316252
MSRA	0.314006	0.13761	0.716516	0.005925
ETV4	0.543076	0.023608	12.49264	0.702756
GAPT	0.960394	0.639212	1.442957	0.845738
BAIAP2	9.405497	2.79602	31.63903	0.000293
ZDHHC21	0.15319	0.022716	1.033087	0.054037
PLEKHF2	0.881334	0.276449	2.809737	0.830907
A2M	1.596414	0.622025	4.097164	0.330708
ARL4D	4.96489	0.757288	32.55056	0.094882
DOK7	0.292282	0.038967	2.192336	0.231526
LRRN1	2.312485	0.729265	7.332843	0.154515
UBE2O	1.417638	0.867035	2.317897	0.164163
ORAI3	0.828149	0.475625	1.441957	0.505139
UNC119B	0.890746	0.408876	1.940513	0.770881
DENND2C	0.609459	0.060375	6.152263	0.674643
ASCL3	3.924359	0.481585	31.97895	0.201487
TUBB6	1.44085	1.069468	1.941197	0.016324
LYSMD3	1.39875	0.439375	4.452921	0.570041
B3GALT6	1.605708	0.800689	3.220099	0.182247
ZNF613	2.220495	0.449907	10.95915	0.327393
JAKMIP2	0.744282	0.30704	1.804181	0.513281
MBLAC2	0.560078	0.255216	1.229104	0.148304
ZNF683	1.103059	0.627448	1.939186	0.733289
SLC35A4	2.017211	1.012335	4.019561	0.046063
IP6K1	2.948393	1.397106	6.222163	0.004546
SSNA1	1.937658	1.006083	3.731821	0.047918
CSTF3	0.311039	0.150734	0.641824	0.001579
YES1	0.937287	0.510685	1.720252	0.834414
CHMP6	1.14529	0.569968	2.301336	0.703193
DLEU1	0.346231	0.165839	0.722844	0.00474
UFSP1	2.664262	0.708477	10.01909	0.147058
TMEM39A	1.005167	0.472777	2.137077	0.989315
TCP11L1	0.484524	0.059348	3.955743	0.49882
GPX2	1.34011	0.074537	24.09413	0.84258
CCDC57	0.159027	0.029436	0.859146	0.03265
HSF5	1.325124	0.15717	11.1723	0.79579
SPHK1	1.997978	0.775734	5.145986	0.151608
BNIP3	0.812562	0.477617	1.382398	0.443927
MYPOP	1.812348	0.856898	3.833132	0.119739
ATAD5	8.23105	0.67265	100.7212	0.099016
ZNF404	0.717925	0.23952	2.151872	0.554059
RTTN	0.27328	0.120304	0.620778	0.001942
ACBD7	0.688537	0.05209	9.101214	0.776928
ANAPC2	2.33058	0.603906	8.994118	0.219447
ZBTB8OS	0.614506	0.132606	2.847662	0.533692
ZNF135	1.294053	0.706984	2.368616	0.403291
COX8A	2.077189	0.978786	4.408231	0.056896
TAC4	1.601153	0.943624	2.716857	0.081008
ZSCAN2	0.536626	0.195062	1.476289	0.227996
B3GNT4	2.7104	0.213137	34.46728	0.44218
CDC26	0.192791	0.068458	0.54294	0.001833

HSD11B2	0.22511	0.015195	3.334914	0.278269
CRLF3	0.753611	0.308314	1.842051	0.535035
RNPEP	0.780667	0.448472	1.358929	0.381295
EID2	0.574458	0.231528	1.425325	0.231858
EID2B	1.267135	0.605223	2.652956	0.530005
KCMF1	0.207156	0.003798	11.2983	0.440357
DNAJC30	0.825078	0.278652	2.443019	0.728464
SPRYD4	0.761239	0.356052	1.62753	0.481643
VPS37D	1.436755	0.391982	5.26623	0.58451
CLEC14A	0.965863	0.654033	1.426367	0.861383
CLK2	0.313204	0.066851	1.4674	0.140675
LPCAT4	2.20233	0.735587	6.593723	0.158213
SLCO3A1	3.530171	0.921209	13.52799	0.065734
ZNF575	5.490207	1.039463	28.99803	0.044905
WDR25	2.896682	1.012975	8.283294	0.047254
DIRAS1	0.622259	0.101351	3.820453	0.608403
PHLDB3	2.624275	0.436575	15.77466	0.291748
PRR15	10.30861	0.666147	159.5254	0.09506
GNG7	0.843123	0.614322	1.157139	0.290779
CNTD1	0.181837	0.010347	3.195609	0.243784
KBTBD11	1.149375	0.844281	1.564721	0.376417
B3GNT5	1.014942	0.588108	1.751563	0.957515
LMNB2	0.694512	0.348597	1.383681	0.299939
MEX3C	0.80801	0.442363	1.475895	0.487964
RNF152	2.646043	0.112945	61.99084	0.545379
MYO1D	0.959232	0.304015	3.026586	0.943402
FOXL1	2.523209	0.269861	23.59207	0.417079
LRRC37A	0.802183	0.205272	3.134857	0.751276
FOXC2	1.077255	0.554496	2.092852	0.826166
CCDC121	3.712421	0.735467	18.7392	0.112288
ACSF3	1.267061	0.868634	1.848242	0.219137
BOK	1.463473	0.865451	2.474725	0.15537
ZNF843	1.043094	0.256174	4.247283	0.953036
TTTY14	0.650113	0.251258	1.682125	0.374662
PFN4	2.035732	0.383792	10.79804	0.403702
CDK5R1	0.788467	0.164829	3.77166	0.766
ZNF285B	1.655944	0.059103	46.39627	0.766766
RUFY1	0.78422	0.162065	3.794774	0.762536
BASP1	1.188628	1.011442	1.396853	0.035896
LRRC37A3	1.647227	0.180041	15.07075	0.658565
IRX5	1.033188	0.769637	1.386988	0.827977
METRNL	1.619569	1.155128	2.270747	0.005169
FAM91A1	0.739896	0.234488	2.334642	0.60738
WSB2	1.524167	0.896693	2.590725	0.119448
SOX11	1.071868	0.046518	24.69791	0.965416
TYMS	0.600291	0.422009	0.853889	0.004533
PXMP2	0.831586	0.357786	1.93282	0.668235
TCEANC	2.21283	0.300914	16.2725	0.435248
PNMA1	1.197523	0.644775	2.224128	0.568238
ANKLE2	0.469942	0.236442	0.934038	0.031188
C8G	0.858008	0.098715	7.457613	0.889603
FUT2	1.221377	0.134493	11.09177	0.858992
EFCAB5	10.98055	0.111281	1083.491	0.306422
GCNT4	4.048046	0.277131	59.12979	0.306782
MUC20	0.577698	0.112644	2.962735	0.510644
THAP4	1.190121	0.361361	3.91959	0.774718
NFATC2IP	1.151827	0.187305	7.083141	0.878774
FAM89B	1.591284	0.82223	3.079657	0.167912

SHMT1	0.123686	0.026898	0.568752	0.007255
DPP7	1.852956	1.037023	3.310865	0.037276
SEC24C	5.197199	0.920068	29.35747	0.062089
SMCR8	0.593167	0.053774	6.54311	0.669818
MTHFR	1.494125	0.723483	3.08564	0.277834
DEAF1	1.613312	0.421615	6.173341	0.484829
MTX3	1.051183	0.58294	1.89554	0.868206
TMEM80	7.503637	1.280285	43.97815	0.025494
FBXO46	1.842743	0.72499	4.68379	0.199046
ZDHHC13	0.528812	0.151294	1.848339	0.318346
SLC38A9	0.674941	0.246205	1.850266	0.444834
ACER2	1.108765	0.252841	4.862186	0.891115
WDR73	0.385898	0.162617	0.915756	0.030807
POLE	0.587365	0.38471	0.896775	0.013717
SCN4B	1.214878	0.158069	9.337266	0.85161
RHOG	1.239465	0.685257	2.241894	0.477715
EPS8L2	0.260911	0.012453	5.466489	0.386706
ANO6	1.000625	0.581782	1.721006	0.998197
ZBTB34	0.571677	0.11664	2.801907	0.490498
TALDO1	0.741444	0.149094	3.687195	0.714709
ULK1	1.06567	0.336055	3.379363	0.913983
RPS6KA3	0.455009	0.117894	1.756093	0.253132
B3GNT8	1.913313	1.218844	3.003475	0.0048
PUS1	0.528038	0.151773	1.837117	0.315442
CHD9	0.607352	0.362385	1.017915	0.058415
TRIM72	0.608138	0.086902	4.25572	0.616357
MAN1B1	1.587886	0.670378	3.761134	0.293259
KCNA3	0.27469	0.032073	2.352585	0.238317
FZD8	2.164845	0.50126	9.349551	0.300799
FBXO39	1.568009	0.093557	26.27966	0.75448
TOP3A	0.614955	0.215813	1.752306	0.362798
CASKIN2	1.358787	0.217243	8.498784	0.743086
ZBTB38	4.583272	0.898749	23.37291	0.06702
FLJ13224	0.670594	0.041163	10.92464	0.778979
RPL13AP3	3.652577	0.207732	64.22359	0.375825
CCDC71	1.919786	1.18335	3.11453	0.008245
LRRN4CL	2.200996	0.038798	124.8627	0.7018
FLJ40194	1.639948	0.121689	22.10084	0.709329
TIMM22	0.734943	0.385852	1.399865	0.348878
HIC1	2.592015	0.79361	8.465799	0.114756
PPFIA3	0.846432	0.121188	5.911891	0.866488
MAGEF1	0.646483	0.272377	1.534419	0.322606
UMODL1	0.983229	0.734702	1.315824	0.909419
SAMD9L	0.991855	0.593028	1.658903	0.975137
PAWR	1.070085	0.419142	2.731969	0.887358
TGIF1	0.789753	0.16823	3.707474	0.764816
NAP1L5	0.681885	0.326233	1.42526	0.308717
CD19	1.306154	0.865884	1.970287	0.202879
OR2T8	0.355839	0.069157	1.830936	0.216348
NR2C2	0.439186	0.156713	1.230809	0.117587
ACOT4	1.630918	1.067214	2.492369	0.023784
ARIH2	0.321262	0.140376	0.735234	0.007187
RBM44	0.057104	0.004747	0.686879	0.024075
ZBTB33	0.895477	0.496553	1.614891	0.713656
ZBED2	0.291905	0.02453	3.473587	0.329809
IRX3	1.046417	0.918977	1.191531	0.493495
OR2B11	0.102138	0.006013	1.735031	0.114411
SLC25A22	0.438411	0.207482	0.926363	0.030745

RABEP2	1.075481	0.197693	5.850795	0.932895
ATOX1	1.34316	0.537696	3.355199	0.527636
TBL1XR1	0.873677	0.447714	1.704909	0.692178
CD163	1.353461	1.060952	1.726614	0.014842
ZNF491	0.223528	0.016807	2.972938	0.256483
RPLP2	7.937762	0.810827	77.70837	0.075108
JUN	1.068183	0.880182	1.296341	0.504257
CSTF2T	0.886207	0.399463	1.966043	0.766356
GBA	1.043773	0.527828	2.064046	0.901987
ACAD9	0.264258	0.116377	0.600053	0.00147
IL17RA	1.198514	0.840318	1.709396	0.317505
PNPLA2	1.79287	1.028106	3.126507	0.039623
MBOAT4	0.085189	0.005111	1.41982	0.086205
AGTRAP	1.117387	0.841199	1.484254	0.443553
CD163L1	0.718722	0.139538	3.701929	0.692897
THAP5	5.654582	0.418919	76.32568	0.191991
SUMO4	1.557018	0.284298	8.52735	0.609821
DNAJC28	1.413531	0.028951	69.01634	0.861508
NAALADL2	0.751665	0.041817	13.51109	0.846436
CD151	2.003087	1.03723	3.868336	0.038563
POLR2L	1.267792	0.425113	3.780872	0.670392
FAM20C	2.18333	1.077808	4.422802	0.030159
FLII	1.567012	0.753911	3.257055	0.228881
SOX12	0.319315	0.077891	1.309028	0.112768
HNRNPA0	0.656157	0.409141	1.052308	0.080395
FAM87B	0.178947	0.016822	1.903565	0.153769
CHID1	0.568555	0.059598	5.423897	0.623655
ZNF620	0.257607	0.024028	2.7618	0.26245
ZNF518A	0.797632	0.381134	1.669273	0.548446
TMEM187	1.440098	0.670738	3.091941	0.349519
ZNF619	7.407858	1.017026	53.9577	0.048084
AP3S1	1.000065	0.491577	2.034536	0.999857
GRB2	2.285163	1.062313	4.915658	0.034461
ZBTB41	0.335471	0.048842	2.304192	0.266597
UBE2N	0.821256	0.376908	1.789458	0.620206
ARL6IP6	0.636741	0.172641	2.348458	0.497859
ZNF354C	0.593856	0.027765	12.70189	0.73878
MAMDC4	0.275431	0.082853	0.915628	0.035398
CENPBD1	2.171593	0.87857	5.367605	0.093041
ODF3	0.678584	0.07713	5.970165	0.726723
BET1L	2.831454	0.730954	10.96804	0.131974
RPS27	1.263606	0.172523	9.254981	0.817859
RIC8A	1.52466	0.539443	4.309233	0.426244
IMP3	1.384267	0.650087	2.947598	0.399105
ASB8	0.596751	0.243982	1.459579	0.257927
ODF3B	2.157276	1.23198	3.777528	0.007149
DPY19L2	0.511469	0.301398	0.867957	0.012963
GPR150	0.150161	0.014009	1.609517	0.117185
TSPYL6	0.254143	0.057392	1.1254	0.071176
DMAP1	0.7031	0.29569	1.671849	0.425411
IMPDH2	0.810108	0.52964	1.239097	0.331434
ALS2CL	0.516149	0.005108	52.15954	0.778837
MLF1	1.962136	0.340965	11.29141	0.450309
NDUFAF3	0.302798	0.084369	1.086733	0.066893
GRAMD1C	0.755873	0.108906	5.246195	0.777067
STAP2	0.996822	0.045422	21.87604	0.998388
TSSK6	8.312317	1.592252	43.39427	0.012018
BOLA1	2.011015	0.694619	5.822158	0.197705

PDE4DIP	47.80287	0.702357	3253.492	0.072513
DDX10	0.363484	0.172132	0.767553	0.007963
NDUFV2	1.209943	0.452548	3.234932	0.704088
DALRD3	3.166344	0.536828	18.67589	0.203043
ZNF114	1.818247	0.180996	18.26574	0.611522
ZNF518B	1.582815	0.806254	3.107337	0.182126
LCORL	0.475585	0.011918	18.97755	0.692741
PARD6G	0.11136	0.010324	1.201161	0.070464
SH2B1	0.997161	0.377253	2.635716	0.995426
ZC3H12D	4.688972	0.308443	71.28209	0.26577
VN1R1	0.083794	0.018264	0.384443	0.001423
SH2D4B	0.05088	0.00097	2.668352	0.140436
RNF212	0.548041	0.101166	2.968867	0.485399
PRSS36	1.788297	0.420166	7.611299	0.431529
ZNF543	1.479092	0.527972	4.143614	0.456429
GALNT11	0.818094	0.472426	1.416683	0.473581
WDR6	0.807893	0.441353	1.478844	0.489211
GEN1	0.134562	0.00617	2.934803	0.202179
TMPRSS9	1.125752	0.750303	1.689075	0.567183
AQP11	0.222292	0.02793	1.769206	0.155352
TMEM11	1.534472	0.657622	3.580484	0.321949
ZNF354B	3.309837	0.541077	20.24669	0.195222
KCNG2	1.303875	0.094817	17.9302	0.842726
ZFAND2A	0.693346	0.406564	1.182419	0.17872
PLEKHM3	0.015844	0.000777	0.323067	0.00705
ZNF223	0.965219	0.534596	1.742711	0.906517
BEND3	0.326583	0.028215	3.780181	0.370433
NT5DC1	0.661127	0.250744	1.743168	0.402843
GLDC	2.48511	0.827548	7.462731	0.104681
TUBAL3	0.152696	0.027358	0.852257	0.032178
P4HTM	0.9425	0.451359	1.968069	0.874738
DTX3	1.469675	0.808546	2.671395	0.206619
KLHL11	1.172896	0.206901	6.649011	0.857031
CTXN1	1.126214	0.841846	1.506638	0.423414
SLC25A20	0.345336	0.1098	1.086127	0.068965
CA8	0.744361	0.340244	1.628456	0.459826
CD28	1.235605	0.414222	3.685756	0.704391
EPM2AIP1	0.52832	0.280479	0.995162	0.048271
MAF	1.309499	0.721995	2.375068	0.374726
CTNNBIP1	0.500594	0.153919	1.628095	0.250165
GTPBP6	0.670863	0.291088	1.546121	0.348721
ERN1	0.6668	0.306411	1.451064	0.307002
GPR35	1.747029	1.016437	3.002751	0.043491
ZNF713	1.122372	0.085195	14.78636	0.930068
PARP10	1.907066	0.74595	4.875528	0.177669
SUZ12	0.526834	0.304113	0.91267	0.02226
NSUN3	1.071393	0.355539	3.228574	0.90248
KCTD12	1.425544	1.047308	1.94038	0.024209
RPP25	1.385868	0.757297	2.536163	0.289896
GRINA	2.042915	1.299604	3.211363	0.001965
THBD	2.749014	0.971683	7.777306	0.056673
GP5	2.492503	0.225121	27.59654	0.456591
COX5A	0.430019	0.160255	1.153892	0.09379
STX19	0.352156	0.035696	3.474195	0.371516
ZHX2	1.072118	0.576728	1.993034	0.82577
CPNE7	27.54564	1.703944	445.2976	0.019527
CD300LB	1.388785	0.962243	2.004404	0.079367
MPI	0.726972	0.300748	1.757245	0.478891

TRIM73	0.534095	0.020805	13.71076	0.704857
OPLAH	1.540735	0.918379	2.584842	0.101541
TMEM52	0.271098	0.027885	2.635645	0.260666
MSC	1.223047	0.739827	2.021882	0.432428
APOLD1	1.969365	0.592922	6.541166	0.268495
EXOSC4	1.200391	0.741711	1.942723	0.457143
DPY19L3	0.380604	0.136424	1.061836	0.064987
TAF7	1.016356	0.589655	1.75184	0.953425
PFAS	0.554939	0.310046	0.993265	0.047399
ZNF552	0.367274	0.042899	3.14436	0.36057
GAK	0.508972	0.028404	9.120301	0.646465
ZBTB7A	2.677087	0.65624	10.92101	0.169829
TUFM	1.466511	0.789354	2.724577	0.225699
RMI1	0.983533	0.373759	2.588133	0.973169
FBXO34	1.145915	0.658661	1.993623	0.629745
EIF3K	1.833579	0.45642	7.366039	0.392832
MRFAP1L1	2.518838	0.606169	10.46663	0.203677
SNX18	1.135476	0.167234	7.70958	0.896561
AURKB	0.856828	0.583399	1.258409	0.430745
MRFAP1	0.81007	0.315934	2.077058	0.661063
TMEM107	1.034742	0.609018	1.758062	0.899509
RRS1	0.630766	0.280094	1.420473	0.265896
RCC2	0.246899	0.096822	0.629604	0.003404
OR13D1	0.779664	0.053081	11.45191	0.85594
IGSF22	6.315252	0.724876	55.01962	0.095188
DPM3	1.374183	0.80567	2.34386	0.243298
CYC1	0.904372	0.386835	2.114308	0.816556
PER1	0.947769	0.533767	1.68288	0.854701
HTR1F	1.182309	0.67258	2.078348	0.560656
TMTC2	3.544157	1.601065	7.845431	0.001803
FARSA	0.708053	0.424102	1.18212	0.186776
SPTY2D1	0.644542	0.308854	1.345084	0.24194
SAMD4B	1.190671	0.372936	3.801447	0.768263
GIMAP7	1.64572	1.143438	2.368643	0.007331
EDC3	0.731103	0.300777	1.777102	0.489475
FUCA1	1.581242	0.968616	2.58134	0.066885
PXT1	8.857596	0.565009	138.8598	0.12033
GGN	0.026696	0.000759	0.938893	0.046078
ZNF664	0.20429	0.063964	0.652465	0.007347
CALR	0.709289	0.369131	1.362905	0.302631
MAGED1	1.124268	0.574502	2.200129	0.732394
LDLRAD3	1.117397	0.634758	1.96701	0.700451
CDH4	0.912422	0.400459	2.0789	0.827321
RAD23A	1.288368	0.715973	2.318375	0.39794
GADD45G	1.289461	0.722654	2.30084	0.389515
MEIS3P1	1.405518	0.578031	3.417603	0.452724
DAND5	0.112592	0.007839	1.617199	0.108187
TMEM151,	3.121867	0.055418	175.8637	0.579926
PTPN11	0.291619	0.135102	0.629459	0.001695
NSUN7	3.109231	0.339366	28.48643	0.315504
WSCD1	0.059119	0.005982	0.584304	0.015534
CLK3	0.777464	0.280168	2.15746	0.628829
HLA-DQB:	1.210571	0.943153	1.553812	0.133506
GATA2	0.936113	0.692735	1.264998	0.667375
ARID3B	1.33343	0.730309	2.434634	0.348866
PACS2	1.913153	0.754011	4.854246	0.172058
ELMOD2	2.920683	0.726853	11.73607	0.130944
EGR3	1.800832	0.775484	4.181898	0.17117

GPC5	11.71704	2.60321	52.73839	0.001344
VWA1	22.33665	5.28646	94.37806	2.39E-05
GEMIN4	0.770974	0.370446	1.604555	0.486722
FJX1	1.986179	0.615171	6.412702	0.251174
KLHL28	0.880175	0.422233	1.834787	0.733438
MKRN3	0.240628	0.071804	0.806388	0.020959
SHARPIN	2.273446	1.053693	4.905183	0.036326
LBX2	0.837251	0.3896	1.799252	0.649039
DNHD1	0.200405	0.057645	0.696716	0.011459
SLITRK4	1.175739	0.922007	1.499296	0.191798
GCC1	1.212801	0.604322	2.433945	0.58723
RNF151	0.000111	8.06E-07	0.015296	0.000291
CIITA	1.135	0.705802	1.825193	0.60135
ZFPM1	0.893483	0.58783	1.358066	0.598031
ALOX15B	0.711746	0.144223	3.512504	0.676325
PLD6	0.795653	0.429793	1.472949	0.466925
CDC42EP4	1.004102	0.643943	1.565699	0.985591
ZBTB42	1.675977	1.022177	2.747956	0.040667
MAF1	1.665562	0.891741	3.110877	0.109488
FCER1A	0.954574	0.784923	1.160893	0.641467
FLJ37453	0.716733	0.217875	2.357806	0.583565
APOBEC3E	0.550853	0.292048	1.039002	0.065506
PIPOX	0.120689	0.010766	1.352901	0.086374
CDH5	2.537111	0.328657	19.58553	0.371937
MYADM	1.655923	0.859072	3.191908	0.13199
SERTAD2	1.008176	0.547458	1.856616	0.979149
AKAP5	1.255523	0.086548	18.21339	0.867565
NKPD1	6.383337	0.257684	158.128	0.257664
GIPC3	2.008758	0.157161	25.67505	0.591585
CITED4	1.28343	0.882738	1.866006	0.191285
ABCA13	0.893282	0.672582	1.186404	0.435725
TIGD5	0.870513	0.457889	1.654974	0.672258
PDXDC1	0.529796	0.232383	1.207848	0.130828
ZNF154	1.387986	0.698259	2.759011	0.349624
R3HDM2	0.286103	0.04839	1.691575	0.167525
B3GNT3	1.597457	0.190934	13.36519	0.665604
ITLN1	1.019336	0.643071	1.615754	0.935057
SEPHS2	0.767949	0.31931	1.846938	0.555395
GPBAR1	1.21479	0.977612	1.509509	0.079146
ZNF784	0.871622	0.405894	1.871736	0.724568
BBS10	1.337524	0.54463	3.284744	0.525813
FIZ1	1.890104	0.70752	5.049314	0.20414
PUF60	0.710812	0.33949	1.488275	0.365276
DCTPP1	0.551998	0.294055	1.036208	0.064419
ZNF771	1.794051	0.11568	27.82346	0.676042
TSHZ1	1.292156	0.613574	2.721217	0.499979
PSTK	0.538245	0.141766	2.043572	0.362817
SOCS4	0.333502	0.062199	1.788199	0.199975
ZADH2	0.583869	0.299754	1.137276	0.113693
ZNF48	3.123254	0.547727	17.80943	0.199766
TMEM150I	2.430373	0.609696	9.687961	0.20815
ANKRD18/	0.334903	0.027734	4.044138	0.389431
TMEM86B	1.006017	0.632379	1.600419	0.979794
TRNAU1AI	0.208808	0.022832	1.909622	0.165414
EXOC3	0.732425	0.279699	1.917942	0.526078
CSNK1A1L	8.976273	1.305282	61.72879	0.025696
LYNX1	6.201061	0.055204	696.5692	0.448764
MED14	0.486442	0.180126	1.313668	0.155108

FAHD1	0.291497	0.085595	0.992709	0.048648
RCC1	0.198764	0.054965	0.718772	0.01376
MYLPF	1.072014	0.26459	4.343372	0.922397
F2	0.840063	0.10888	6.481501	0.867231
PRKRA	0.457981	0.259918	0.806972	0.006892
ZNRF2	0.388319	0.111979	1.346602	0.135981
FGD6	0.126366	0.011842	1.348427	0.086807
OAZ2	1.499532	0.730606	3.077717	0.269429
PNPLA1	1.07826	0.227671	5.106688	0.924348
CCDC43	1.306111	0.421847	4.043948	0.64327
KCTD4	0.500286	0.020252	12.3586	0.672092
FZD2	1.705515	1.246517	2.333529	0.000845
TIGD2	1.457994	0.489098	4.346257	0.498655
HCLS1	0.765594	0.423817	1.382988	0.376003
ZNF609	1.253865	0.592063	2.655423	0.554579
PAK2	0.758695	0.24946	2.307454	0.626536
CCDC66	0.372381	0.156436	0.886419	0.025586
MCFD2	1.047679	0.409941	2.677533	0.922496
HARBI1	1.707906	0.214144	13.62139	0.613377
GAS1	0.955362	0.268251	3.402477	0.943825
ZNF571	0.704341	0.125191	3.962711	0.69087
KCNE1	0.817677	0.266502	2.508785	0.724909
NRIP1	1.300006	0.824074	2.050806	0.259302
BHLHA15	1.1687	0.093759	14.56778	0.903607
RNF182	1.103629	0.770734	1.580306	0.590367
TSPYL5	0.870921	0.410827	1.846286	0.718471
FUT7	2.075166	1.100435	3.913286	0.024091
SSTR2	1.17813	0.734018	1.890947	0.497105
ZNF594	0.597743	0.279179	1.27981	0.185234
PCGF5	1.214008	0.477428	3.086989	0.683811
PRF1	0.931968	0.698423	1.243607	0.632148
YOD1	0.629719	0.318005	1.246982	0.184591
TMEM64	0.85788	0.129052	5.702799	0.873977
CHRM4	1.387442	0.163776	11.75386	0.763892
SHISA2	1.165172	0.559095	2.428259	0.683253
S1PR5	0.989449	0.713534	1.372059	0.949297
GPR157	2.361492	0.138851	40.16274	0.552276
PIPSL	1.164617	0.408847	3.31746	0.775392
CHST13	1.032479	0.752615	1.416411	0.842933
SLC36A4	0.936947	0.591302	1.484638	0.781536
ZDHHC20	2.23634	0.589531	8.483386	0.236749
ZFP3	2.480234	1.096363	5.610884	0.029195
MAP3K15	0.320808	0.059541	1.728523	0.185808
PPA1	1.078236	0.729137	1.594479	0.705894
PSMG4	1.20859	0.371288	3.934112	0.753051
MAP6D1	1.108966	0.621945	1.977357	0.72595
ZNF443	2.618027	0.646907	10.59513	0.177235
SSR4	1.89255	0.754798	4.745306	0.173776
CAPS2	0.192384	0.015657	2.363914	0.197818
ZNF792	0.91302	0.52923	1.57513	0.743631
CUEDC1	1.375864	0.970508	1.950526	0.073156
SCRIB	0.590854	0.119818	2.913655	0.518055
KCTD2	13.39831	1.275981	140.6876	0.030533
D2HGDH	1.253638	0.779589	2.015943	0.350995
OXTR	0.384157	0.073622	2.004513	0.256384
FAM83H	1.184781	0.554637	2.530852	0.661495
ZNF572	1.174646	0.178428	7.733069	0.867048
ST20	2.122926	0.192772	23.37892	0.538543

PITPNB	1.015342	0.252734	4.079074	0.982879
TCEAL8	0.957179	0.521076	1.758271	0.887824
LRRC57	0.567434	0.263094	1.223825	0.148483
MRPL14	0.936681	0.419091	2.093509	0.873346
GPR137C	5.008951	0.653073	38.41772	0.121125
BBS12	1.640141	0.386742	6.955713	0.502085
ZFP82	0.392688	0.15634	0.98634	0.046675
NQO1	1.68213	0.370463	7.6379	0.500523
OR56B1	0.890938	0.110585	7.177885	0.913616
AEN	0.317961	0.101532	0.995735	0.049148
FKRP	1.077945	0.343776	3.380008	0.897576
TRAPPC5	1.442913	0.848171	2.454689	0.176206
RPH3AL	0.882193	0.211247	3.684144	0.863539
SLC25A42	0.513015	0.226178	1.163616	0.110194
FCRL6	1.210172	0.483149	3.03119	0.683861
SLC26A11	2.442226	0.400577	14.88968	0.333
HIGD1A	0.955373	0.5614	1.625826	0.866349
OR52N4	0.179899	0.005313	6.091554	0.339824
MAPK15	0.355913	0.087681	1.444716	0.148386
EHMT1	2.039507	0.121065	34.35829	0.620866
F2R	1.072334	0.467914	2.457504	0.868903
ZNF707	1.738507	0.632796	4.776273	0.283492
NPM1	0.688917	0.041259	11.50319	0.795307
PJA1	0.541893	0.10761	2.728808	0.457577
DHTKD1	0.674436	0.316376	1.437732	0.307789
PENK	2.778679	0.709682	10.87961	0.142235
ZNF746	1.459291	0.730431	2.915441	0.284454
POLR2A	2.092514	1.038055	4.218099	0.038981
FRAT2	1.122652	0.760146	1.658033	0.560892
TMEM102	1.833249	0.259614	12.94538	0.543361
CCL13	0.481685	0.072979	3.179279	0.448055
DDX60L	1.341747	0.765057	2.353139	0.305066
AATK	2.65508	0.171587	41.08371	0.484735
DDN	0.348193	0.080182	1.512031	0.159095
SAGE1	0.315031	0.081994	1.210387	0.092582
ZNF467	1.207849	0.915292	1.593918	0.182048
ZNF678	0.857425	0.091743	8.013475	0.892695
TMEM45A	1.40964	0.671905	2.957388	0.363793
RAP2B	2.746203	0.246747	30.56419	0.411244
ZBTB2	1.257836	0.517353	3.058167	0.612809
RNF135	1.857764	0.894909	3.856579	0.096509
ACBD4	2.324016	0.709562	7.611808	0.163576
SGSH	1.252891	0.962389	1.631083	0.093911
MAB21L2	9.141286	0.549422	152.0926	0.122954
FANCB	0.088893	0.022203	0.355902	0.000627
SETD2	0.735596	0.359318	1.505914	0.400896
TMIE	2.982735	0.466421	19.07443	0.248352
MEX3D	2.470183	0.977608	6.241567	0.055866
MRPS23	1.004214	0.504543	1.998732	0.990446
OR52H1	1.587877	0.108432	23.25287	0.735621
GPR135	1.092774	0.051211	23.31828	0.954692
P2RY13	1.054406	0.661164	1.681536	0.823948
ZFP41	0.342254	0.02196	5.334192	0.444157
PHLDA2	0.995774	0.155534	6.375243	0.996433
ATG9B	0.46412	0.053674	4.013228	0.485537
GPR88	0.624798	0.040143	9.724642	0.737006
PLAG1	2.094532	0.399615	10.97823	0.381722
YIPF6	0.529564	0.272734	1.028247	0.060424

ZBTB20	0.573007	0.352484	0.931495	0.02469
AMIGO1	0.181036	0.015651	2.094018	0.171231
GPR3	1.725294	0.472142	6.304545	0.409431
SIAH2	1.287886	0.810066	2.04755	0.284834
SLC9A9	1.553487	0.888324	2.716714	0.122417
LSM10	2.577279	1.215458	5.464911	0.013558
RELL1	0.904222	0.348544	2.345806	0.836014
RFX7	0.669528	0.387799	1.155929	0.1499
SLC35C1	2.250443	1.127694	4.491015	0.0214
TIGIT	0.351296	0.050352	2.45091	0.291204
RNF41	0.634205	0.144036	2.792478	0.547092
SLC2A4	0.349551	0.038132	3.204276	0.352458
CLDN7	2.012723	0.881429	4.596003	0.096838
ZNF329	0.763958	0.353695	1.650097	0.493178
ZNF101	1.780871	0.74569	4.253109	0.193842
ADO	1.980737	0.9772	4.01486	0.057962
PRKAG1	1.405986	0.664942	2.972886	0.372457
GINS3	0.390177	0.194879	0.781194	0.007881
OR52K2	1.260738	0.237627	6.688899	0.785522
CCDC149	2.751437	0.63547	11.91308	0.175863
MRPS11	0.859181	0.325868	2.265313	0.758968
SNRPE	1.099527	0.499673	2.419501	0.813597
RTKN2	192.0452	8.785092	4198.177	0.000836
CHST15	1.221215	1.011375	1.474594	0.037751
TRPC2	1.71375	0.908903	3.231301	0.095958
IDH2	0.865278	0.517294	1.447351	0.581421
TNRC18	0.712424	0.053365	9.510806	0.797605
TMEM30B	0.462857	0.084376	2.539067	0.375066
DEXI	0.744536	0.395868	1.400301	0.360034
NOP10	0.869587	0.338975	2.230791	0.77127
FAM89A	1.085366	0.718048	1.640586	0.697555
TDRKH	0.577962	0.042252	7.905839	0.681232
ZNF708	1.177607	0.493983	2.807299	0.712248
MRPL41	1.404332	0.862236	2.287249	0.172448
CREB3L2	0.682723	0.336101	1.386818	0.29117
P2RY8	1.119328	0.649494	1.929034	0.684793
TP53TG1	0.620481	0.257391	1.495763	0.287739
UNC5C	1.030745	0.131612	8.072486	0.976995
TSEN54	0.554147	0.307456	0.998776	0.049526
RGMA	2.174766	0.670326	7.055686	0.19572
UBA7	0.624795	0.371018	1.052156	0.076933
MRPS16	0.493	0.166513	1.459639	0.201578
LDOC1	1.318124	0.854322	2.033718	0.211898
ARL6IP4	1.299626	0.512601	3.295011	0.580863
EXT1	2.474221	1.02455	5.975078	0.044022
SHMT2	0.895748	0.4261	1.883043	0.771488
ATP6AP2	0.700578	0.384095	1.277835	0.245864
ZAR1	3.30767	0.269562	40.58682	0.349713
BACE2	1.324928	0.751162	2.336957	0.331183
UBE2E2	0.879191	0.618486	1.249791	0.473092
SYNM	1.081158	0.135753	8.610516	0.941242
IZUMO1	0.940825	0.04667	18.96625	0.96825
B4GALNT4	0.769707	0.163206	3.63008	0.740825
AP1S2	1.080895	0.744197	1.569927	0.682913
DCAF4L1	0.171765	0.014289	2.064813	0.164986
ZSCAN22	1.953447	0.44831	8.511873	0.372583
KCNJ14	0.08616	0.003969	1.870593	0.118486
FBXL6	2.069507	0.732099	5.850107	0.170127

C1S	1.74075	0.053874	56.24637	0.75458
GLTPD2	1.049749	0.045167	24.39789	0.975869
KBTBD3	0.962911	0.102166	9.07544	0.973659
CLN8	0.80667	0.215575	3.018514	0.749654
PLCXD1	1.379851	0.845697	2.251381	0.197394
NXPH4	0.05666	0.0004	8.032554	0.256085
CACNB4	1.666681	0.062516	44.43399	0.760401
TRAPPC6B	0.318815	0.102669	0.990003	0.048003
PGBD4	1.857138	0.389393	8.85727	0.437363
NPLOC4	1.253266	0.53355	2.943822	0.604362
CAPN12	1.081163	0.36017	3.245445	0.889336
EXOC7	0.284408	0.068747	1.176596	0.082653
KPNA2	0.741559	0.322163	1.70693	0.482099
NCF1B	1.110692	0.842056	1.465028	0.457411
BGN	1.068221	0.618096	1.846148	0.813107
CEP97	0.643505	0.059331	6.979443	0.717017
FES	0.645711	0.401589	1.038234	0.071056
GLRX5	1.072035	0.623406	1.843515	0.801441
FAM104B	0.215543	0.053019	0.87627	0.031989
MXRA7	0.859541	0.184859	3.996611	0.846934
LIMK2	0.593254	0.120327	2.92494	0.521235
MFSD5	2.880772	1.431453	5.797498	0.003025
ADI1	1.032745	0.686073	1.55459	0.87729
SPNS3	1.477323	1.058522	2.061821	0.02177
SATB1	0.622294	0.250648	1.544992	0.30661
CSF1R	1.589526	1.179493	2.1421	0.002331
EPHB3	1.839933	0.686142	4.933894	0.225695
EPGN	0.105299	0.010286	1.077974	0.057868
HS3ST4	0.418374	0.049891	3.508399	0.421904
TRAK1	0.810868	0.303856	2.163876	0.675486
PLCB1	3.420818	0.939506	12.45548	0.062136
SKA2	0.493871	0.148931	1.637731	0.248736
NDN	0.952676	0.772048	1.175564	0.651282
TTC3	0.340164	0.141286	0.818988	0.016155
GALR2	1.238111	0.781032	1.962685	0.363554
TSKU	1.298491	0.529837	3.18226	0.567917
ANXA2	1.395412	0.998808	1.949501	0.050823
RGS6	0.495111	0.048452	5.059363	0.553313
HOXB4	1.540707	1.010706	2.348635	0.044487
PAQR7	1.136841	0.684704	1.887542	0.62005
PAPPA	2.062589	0.264816	16.065	0.489401
MAFA	0.863286	0.116035	6.422748	0.885837
NGRN	0.74429	0.296319	1.8695	0.529691
RPS17	1.044514	0.236277	4.617505	0.954202
CRIP2	1.209133	0.962018	1.519724	0.103524
DDX28	1.921511	0.973638	3.792177	0.059708
ACBD3	1.103399	0.541929	2.246584	0.786211
VMO1	1.497644	0.544043	4.12272	0.434362
ALG12	0.958256	0.116857	7.857914	0.968318
LCK	1.046308	0.306629	3.570304	0.942375
COL18A1	0.88544	0.260581	3.008679	0.845424
RBM10	0.514588	0.113436	2.334374	0.389154
GLUD2	1.327654	0.112338	15.69073	0.82204
RPL35A	1.309908	0.629588	2.725369	0.470181
SLC25A18	0.412979	0.03575	4.77062	0.478704
ZNF721	0.704693	0.468105	1.060856	0.09356
CEP63	0.138248	0.025077	0.762154	0.023098
WFDC10B	4.970774	0.129685	190.5276	0.388701

EWSR1	0.407013	0.145164	1.141192	0.087472
ODF3L1	0.225203	0.025825	1.963847	0.177285
HMGN4	0.551329	0.248646	1.222473	0.142774
SPATA13	0.840886	0.428977	1.648317	0.613802
CNOT10	0.457316	0.207438	1.008194	0.052412
MTA1	1.118357	0.587398	2.129256	0.733492
ZNF662	1.759852	0.48269	6.416294	0.391785
CADM1	2.450832	1.416587	4.240174	0.00135
ZNF320	0.643732	0.187237	2.213185	0.484495
PYCR1	1.982489	0.596831	6.585225	0.26386
SPNS2	1.617316	1.067006	2.451449	0.023476
AP2A2	2.346065	1.058758	5.198564	0.035675
SLC8A1	0.901297	0.14954	5.432246	0.909721
SLC25A21	0.769379	0.231762	2.5541	0.668468
ABAT	2.175664	0.249944	18.93828	0.481377
SLC25A10	0.960425	0.394612	2.337525	0.929101
CAMK1D	7.34257	2.70886	19.90259	8.90E-05
LYSMD4	0.394932	0.133197	1.170982	0.09387
AFMID	0.757038	0.450165	1.273105	0.293941
GAS6	1.543491	0.984366	2.420203	0.058584
NEB	0.141136	0.020133	0.989379	0.048757
BEGAIN	0.847805	0.357049	2.013093	0.708255
GPC6	0.800043	0.158401	4.040818	0.787173
CSMD1	0.694843	0.288953	1.670884	0.416075
GPR19	1.997223	0.187267	21.30055	0.566775
RABIF	0.897989	0.197146	4.090299	0.889381
TMEM119	1.328043	0.573233	3.076755	0.508076
FANCF	0.253431	0.063093	1.017977	0.053009
CALN1	1.546011	0.149382	16.00024	0.714812
RUVBL2	1.26646	0.667604	2.402505	0.469615
PTTG1IP	1.006453	0.521	1.944239	0.984723
DDX41	1.441981	0.652207	3.188109	0.365903
DAZAP2	0.741891	0.352124	1.563094	0.432331
ZNF623	16.36884	0.597349	448.5467	0.09794
CCDC125	0.894424	0.486552	1.644212	0.719458
BCOR	0.878515	0.395681	1.950533	0.750281
JRKL	0.341911	0.082132	1.423367	0.140259
KIAA2026	0.400303	0.11376	1.408601	0.153795
FHL3	1.291258	0.780764	2.135531	0.319334
TMEM89	1.371743	0.225914	8.3292	0.731246
SF3A3	0.415881	0.224856	0.769189	0.005168
ASB7	0.344424	0.085887	1.381206	0.13253
TREX2	1.304093	0.094364	18.02241	0.842921
GPR132	1.36024	0.720758	2.567094	0.342394
MX2	2.002876	1.234016	3.250778	0.00494
EP400	0.890177	0.390369	2.029915	0.782085
MEX3B	0.959859	0.690638	1.334025	0.807277
PI4KAP2	0.587539	0.30327	1.138265	0.114992
PSMG1	0.602899	0.276284	1.315629	0.203745
KLRC4	0.044669	0.00267	0.747177	0.030561
SERHL2	4.26594	1.518751	11.98238	0.005905
PCBP3	0.801712	0.249617	2.574915	0.710464
SETD3	0.196721	0.04489	0.862083	0.03102
TNFAIP8L3	0.4579	0.050068	4.18779	0.489122
ZNRF3	1.915145	0.269648	13.6021	0.515926
SFXN4	0.902311	0.375068	2.170715	0.818473
FAM167B	0.232183	0.011667	4.620813	0.338601
MRPL54	1.764852	0.950367	3.277365	0.072055

ZNF438	1.390319	0.726514	2.660634	0.319671
CCR3	1.048975	0.202584	5.431575	0.954554
ZNF530	0.075448	0.005658	1.005992	0.050531
NDUFB1	3.681627	0.691113	19.61239	0.126738
KLHL25	2.74659	0.444321	16.97818	0.276982
TRMT12	0.656012	0.298136	1.443475	0.294761
BMP8A	1.966284	0.165645	23.34068	0.592202
EFHC2	0.911801	0.492806	1.687036	0.768671
NOG	1.144217	0.552103	2.371357	0.717103
UPP1	2.098486	1.004792	4.382642	0.048531
TRIM52	0.214748	0.070535	0.653807	0.006768
CMTM4	0.463844	0.146858	1.465028	0.190475
TMEM50A	0.569807	0.194732	1.667317	0.304537
ASCL2	1.220165	0.785426	1.895535	0.375974
TBK1	1.026911	0.533901	1.975172	0.936578
CBX6	1.508993	0.942109	2.416981	0.086924
MACC1	0.319148	0.0202	5.042348	0.417337
TBL3	1.224518	0.570728	2.627247	0.603043
KREMEN1	0.002829	4.06E-05	0.19722	0.006736
TRAIP	0.292398	0.079396	1.076839	0.064506
CHEK2	0.075332	0.017327	0.327516	0.000564
AIFM3	1.217619	0.294598	5.032607	0.785659
ZNF703	5.432231	1.460143	20.20975	0.011581
EMILIN3	0.05869	0.002852	1.207874	0.066126
OLFML1	1.374158	0.136236	13.86057	0.787516
RBM12B	0.873603	0.387342	1.970304	0.744697
CCR4	3.037429	0.745366	12.37779	0.121148
LIN9	1.021469	0.34763	3.001465	0.969189
BTBD9	0.583778	0.033839	10.07124	0.711071
NUDT14	1.325052	0.959722	1.829449	0.087236
PNMA3	1.036424	0.562487	1.909686	0.908657
IQGAP3	0.406596	0.064039	2.581547	0.339936
TOB2	2.851925	0.995473	8.170461	0.050996
SCN5A	0.340471	0.002649	43.76719	0.663679
ARSI	1.141042	0.06325	20.58444	0.928763
UTY	0.666442	0.20627	2.153224	0.497653
TTC32	0.727861	0.288628	1.835518	0.500901
DNAH2	1.117741	0.453057	2.757588	0.809102
SH2D1A	1.3056	1.010744	1.686473	0.041173
PRKX	0.938229	0.366227	2.403628	0.894318
KCNH8	2.88056	0.464354	17.86919	0.255886
SMTN	9.068909	1.388245	59.24395	0.021305
NPW	1.002312	0.75937	1.322978	0.986991
ST6GALNA	1.614813	0.230763	11.3	0.629265
PTP4A2	0.230442	0.043285	1.226837	0.08537
ACTG1	2.270353	0.636319	8.100503	0.206442
DENND5A	0.652492	0.319729	1.331585	0.240747
DIABLO	0.712628	0.245501	2.068585	0.53321
VPS33B	0.733002	0.307184	1.749088	0.483936
TBX1	0.304819	0.023327	3.983153	0.364938
ADAP2	1.170086	0.896572	1.527039	0.247564
FAM120C	4.445194	0.477452	41.38588	0.19002
BRD7P2	0.531355	0.179818	1.570136	0.252691
EIF3C	0.733768	0.319861	1.683277	0.464942
CLDN5	1.256334	0.851765	1.853064	0.249804
NIPSNAP1	0.865289	0.55046	1.36018	0.530669
LRTOMT	0.119851	0.011383	1.26189	0.077346
ADRA2C	0.894025	0.226866	3.52315	0.872802

NR2C2AP	0.349571	0.120599	1.013274	0.052907
CRELD2	1.321795	0.74961	2.330734	0.335012
SCFD2	0.484458	0.14673	1.599533	0.234358
UBE2F	0.43213	0.213636	0.874089	0.019576
KCNJ12	7.566101	0.980192	58.40271	0.052285
GPR173	0.011428	7.65E-05	1.707402	0.080026
PPP1R2	0.790416	0.451715	1.383076	0.409997
TSPYL2	0.652131	0.226281	1.879416	0.428584
PGP	1.553927	0.472523	5.110206	0.468017
SNRNP35	0.621162	0.110666	3.486541	0.588508
IRAK1	0.945974	0.382174	2.341513	0.904398
OLIG1	1.284574	0.954946	1.727983	0.097873
PCDH9	0.112146	0.00382	3.292011	0.204464
ACOT1	5.942391	1.311305	26.92891	0.020806
OAF	1.549952	0.996299	2.411273	0.051953
KCNK12	1.411614	0.913562	2.181194	0.12048
POU6F1	0.493001	0.060875	3.992627	0.50752
TM2D3	0.724498	0.19892	2.638742	0.625073
TSSC4	1.326048	0.634029	2.77338	0.453492
TACSTD2	0.91036	0.703686	1.177735	0.474726
CLECL1	0.976458	0.802937	1.187479	0.81138
ZDHHC23	0.536415	0.333109	0.863806	0.0104
SRPK3	0.061543	0.003449	1.098294	0.057934
EFNA5	0.605757	0.051583	7.113655	0.690004
PKP3	8.05292	0.185169	350.2183	0.278466
MAP7D2	0.261583	0.033561	2.038842	0.200548
CSF1	0.66707	0.088492	5.028489	0.694444
PLA2G6	2.313855	0.276457	19.36624	0.438989
MAML2	2.354424	0.232986	23.79251	0.468099
A3GALT2	2.262354	0.195143	26.22824	0.513758
SS18L1	0.412853	0.089626	1.901763	0.256304
TOP1MT	1.43351	0.831231	2.472178	0.195261
COPB2	0.5011	0.259927	0.966045	0.039102
LRRC19	0.002897	8.68E-05	0.096693	0.001093
THAP7	5.371295	1.230213	23.45189	0.025386
KNTC1	0.399674	0.219621	0.727339	0.002681
CCR10	2.517604	0.224653	28.21382	0.453935
WDR27	0.413078	0.173071	0.985917	0.04638
TXNRD2	1.340675	0.132903	13.52419	0.803664
FOXO4	1.640687	0.962261	2.797426	0.068964
PTP4A3	2.147003	1.407319	3.275464	0.000392
FOXD4L1	0.200381	0.022647	1.772964	0.148414
PROS1	1.480217	0.995149	2.201723	0.052872
GAST	0.517823	0.015691	17.08855	0.712197
HDDC3	0.895923	0.409	1.96254	0.783549
BEX5	1.36269	0.411588	4.511608	0.612415
ZFP1	0.884706	0.211145	3.70696	0.866913
CEND1	0.128434	0.010537	1.565465	0.107689
DUSP8	1.045567	0.469548	2.328222	0.913128
SOCS3	2.361018	1.545768	3.606235	7.03E-05
LPAR5	1.307235	0.898571	1.901756	0.161279
XPOT	0.331385	0.136895	0.80219	0.014341
PDE4B	0.587854	0.279549	1.23618	0.161248
SNN	1.789515	1.019409	3.141393	0.042671
KCNH7	0.336866	0.006608	17.17184	0.587505
NELL2	0.938055	0.539041	1.632432	0.821027
KRBA2	0.600556	0.158184	2.280057	0.453793
MED12	1.028681	0.504929	2.095709	0.937921

ZNF93	0.321521	0.089166	1.159357	0.082918
CDCA2	0.339756	0.112305	1.027865	0.055966
ZBTB40	0.50435	0.225857	1.126241	0.094935
EIF4ENIF1	0.978488	0.292816	3.269754	0.971817
LRRC26	1.369119	1.012486	1.85137	0.041294
ATL3	0.80462	0.389979	1.660123	0.556354
NDUFA12	0.998502	0.441473	2.258363	0.997128
TCTE3	14.76092	0.841921	258.7948	0.065444
UBE2G2	0.214927	0.072883	0.633806	0.005329
OSBP2	1.033424	0.842845	1.267096	0.751917
APOO	0.410685	0.14587	1.156252	0.091977
PRR16	0.592716	0.254028	1.382968	0.226306
TMED9	1.277863	0.448344	3.642145	0.646362
TMEM186	3.183521	0.759499	13.34407	0.113256
SDR42E1	0.453638	0.011735	17.53609	0.671632
RBM33	0.676591	0.418693	1.093345	0.110598
ARMCX2	0.957814	0.488972	1.876196	0.900012
BTBD6	1.089608	0.520258	2.282032	0.820012
RBM43	0.667467	0.115041	3.872639	0.652237
SUMO3	0.528741	0.181363	1.541477	0.243093
IMMP2L	0.519723	0.26107	1.034634	0.062456
JAG2	0.515973	0.034488	7.719421	0.631675
FMNL1	1.646208	0.694584	3.901618	0.257551
LCN12	0.889377	0.036892	21.44087	0.942444
WT1	1.607268	0.789817	3.27077	0.190514
ZFP90	1.046728	0.58453	1.874396	0.877898
MUC6	1.45822	0.362752	5.861872	0.595132
NOC4L	1.96402	0.390142	9.887096	0.413046
USP18	2.965909	1.403413	6.268019	0.004404
NDUFA6	1.049242	0.345416	3.187196	0.932425
CHRM5	1.086694	0.128455	9.193138	0.93917
SORCS2	0.639351	0.234911	1.740105	0.381246
TMEM121	0.810928	0.160942	4.085984	0.799492
TMEM106A	1.067004	0.671783	1.694738	0.783521
SIVA1	1.119981	0.640345	1.958877	0.691182
BRI3BP	0.425539	0.165849	1.09186	0.075541
DGAT1	1.723819	0.884467	3.359709	0.109737
ROBO2	3.290001	0.217045	49.87029	0.390569
AP3M1	1.437485	0.422662	4.888932	0.561201
F8	1.183181	0.01717	81.53308	0.937918
CA13	4.323075	0.639357	29.2309	0.133285
UBOX5	1.844892	0.167163	20.36114	0.617158
MAFF	1.248412	0.688727	2.262917	0.464701
BRF1	1.205529	0.158348	9.177879	0.856775
SEMA4B	1.376499	0.526147	3.601177	0.514902
CIB1	0.77359	0.312059	1.917719	0.579428
SLC24A3	1.344036	0.98218	1.839208	0.064659
INTS5	1.496707	0.736821	3.040268	0.264721
RPS27L	0.449734	0.153688	1.31605	0.144661
MANEAL	0.279651	0.050752	1.540932	0.143362
ANO9	0.793278	0.142423	4.418462	0.791553
FAF1	0.351011	0.141505	0.870705	0.023905
FAM43A	2.172599	1.531183	3.082705	1.38E-05
HSF1	0.984427	0.494924	1.958071	0.964317
PURA	1.407737	0.582122	3.404312	0.44783
INPP5J	1.518904	0.308385	7.481119	0.607373
MIXL1	2.060961	0.118128	35.95724	0.62008
MFSD6L	0.494105	0.133968	1.822378	0.289727

LRRC37B	0.094861	0.009641	0.933371	0.04348
DDX51	0.800361	0.342642	1.869525	0.606919
NOMO2	1.182188	0.285649	4.892597	0.817353
SIGIRR	3.10989	1.147457	8.428561	0.025724
NRBP2	1.789928	1.057026	3.030995	0.030286
IFITM2	1.753826	1.178994	2.608926	0.005561
TNFAIP2	1.61515	1.148884	2.270647	0.005806
ZNF445	0.597407	0.10688	3.339206	0.557389
PGBD2	1.511255	0.381625	5.984656	0.556479
RAB11B	1.271046	0.460061	3.511614	0.643674
PRMT3	0.446433	0.245508	0.811795	0.008208
GP1BA	0.824014	0.350846	1.935318	0.656804
PRPF39	0.656278	0.20592	2.091595	0.476357
PPIL6	0.849593	0.2107	3.425763	0.818774
ZNF74	0.39363	0.130125	1.190732	0.098766
CDNF	0.029987	0.001732	0.519235	0.015934
KLHL33	0.360257	0.024473	5.303149	0.45683
RBM11	0.076758	0.003319	1.774986	0.109176
GALNT17	0.385378	0.03665	4.052301	0.42701
ZBTB37	1.178043	0.081781	16.96947	0.904174
IL3RA	1.663093	1.062306	2.603656	0.026131
CCDC137	0.782308	0.287179	2.131097	0.631118
RGPD2	0.994999	0.326021	3.036683	0.992974
ARL15	2.368179	0.396484	14.14503	0.344435
CDK10	0.595586	0.136049	2.607319	0.491531
TMEM105	1.513312	0.747014	3.06569	0.250058
SOCS1	1.96324	1.35797	2.838289	0.000335
TCN2	1.525677	0.986379	2.359835	0.057652
GAS2L1	2.526616	0.81681	7.815513	0.10767
ATP6V0A2	0.422772	0.237151	0.753682	0.003516
HGS	0.734608	0.325498	1.657919	0.457704
TNFAIP8L1	1.435357	0.935332	2.202693	0.098126
MAPK11	2.669756	1.006861	7.079028	0.048415
SP140L	0.314803	0.089954	1.101681	0.070537
MRPL30	1.373438	0.632394	2.982841	0.422604
SMYD3	0.768634	0.533609	1.107174	0.1576
METTL7A	1.067828	0.696522	1.63707	0.763391
FAM174B	1.086186	0.782226	1.508261	0.621601
TMEM179I	2.283205	1.048117	4.973703	0.037685
GPRIN3	1.257246	0.695634	2.272267	0.448395
STAC3	1.779362	0.714007	4.434315	0.216123
MUC1	0.441879	0.241932	0.807075	0.007876
IRF7	2.593639	1.657479	4.058551	3.02E-05
BRCC3	0.656708	0.269945	1.597608	0.353885
PDE6G	0.932346	0.423669	2.051766	0.86181
NR2F2	5.643472	0.651271	48.9025	0.116246
DLK1	2.087075	1.153741	3.775444	0.014981
AHNAK2	0.692802	0.089654	5.353614	0.724998
OLFML2A	1.543755	1.145389	2.080673	0.004353
SP1	0.767147	0.270391	2.176535	0.618335
WASH3P	2.336769	0.899145	6.072982	0.081544
MRPL40	0.66166	0.36424	1.201937	0.175087
PDIA2	2.597391	0.152838	44.14098	0.509005
PCGF3	9.027309	1.92756	42.27745	0.005222
LMLN	0.332589	0.059775	1.850543	0.208712
P4HB	0.822332	0.411718	1.642458	0.57945
PSMD13	1.464425	0.484194	4.429091	0.499327
PBX1	1.43311	0.524452	3.916093	0.482926

NDUFA4L2	0.319974	0.026988	3.793714	0.366436
ZFP36L1	1.161375	0.844234	1.59765	0.357894
UBE2L3	0.468409	0.210777	1.040942	0.062677
BRWD1	0.332235	0.054006	2.043855	0.234528
SNAI3	2.619741	1.404458	4.886614	0.002464
ZBTB3	2.878283	1.656999	4.999708	0.000175
LYG2	0.649504	0.065792	6.41196	0.711831
PRAME	0.976089	0.556411	1.712313	0.932743
MYBL1	0.693043	0.257584	1.864666	0.467782
DRG1	0.594117	0.305318	1.15609	0.125291
ANKFY1	1.090887	0.383928	3.099632	0.870307
YTHDF3	0.918179	0.482914	1.745761	0.794569
ZNF696	1.193451	0.589848	2.414729	0.622834
ADARB2	2.009028	0.346376	11.65263	0.436656
IFIT1	3.704893	1.772747	7.742921	0.000497
KCNQ5	4.213478	0.228765	77.60533	0.333238
MORF4L1	0.860707	0.314871	2.352763	0.77001
NLRP9	0.179577	0.013037	2.473549	0.199426
WDR53	0.63721	0.178827	2.270558	0.486984
DMWD	1.60684	0.759656	3.398824	0.214682
PIGP	0.69192	0.349348	1.37042	0.290867
IKZF1	1.029876	0.65902	1.609429	0.897166
PCYT2	1.023786	0.36022	2.909716	0.964818
NAT8L	3.415624	0.274624	42.48166	0.339525
BCAP31	1.894252	0.668246	5.36957	0.229483
GNB1L	0.859927	0.481033	1.537265	0.610645
DNAH14	0.042533	0.001343	1.347421	0.073319
EVI2B	1.405836	0.823817	2.399048	0.211589
ZNF829	0.009474	4.03E-05	2.227864	0.09444
THNSL1	1.262568	0.480809	3.315411	0.635983
TRIM69	0.08964	0.002023	3.971712	0.212419
ATP6V0C	1.871066	0.919742	3.806381	0.083797
IFITM1	1.197206	0.912932	1.57	0.193139
LAMP1	0.713245	0.277077	1.836018	0.483623
TAS2R60	1.097223	0.072261	16.66054	0.946701
KLHDC8B	1.345103	0.940786	1.923182	0.104092
SETD4	0.566417	0.109925	2.918614	0.49681
PTCH1	0.925763	0.115271	7.434959	0.942149
RNPC3	0.383976	0.12895	1.143367	0.085558
ZNF267	5.259976	0.390199	70.90581	0.210983
IRS2	1.015898	0.785005	1.314703	0.904563
BICD2	0.303709	0.085858	1.07433	0.064496
CCIN	0.285349	0.030218	2.694588	0.273656
TMLHE	0.649884	0.16813	2.512034	0.532148
SDHAP3	13.50019	1.44982	125.7088	0.02224
RASA3	1.14713	0.443443	2.967476	0.777133
LRCH3	0.590997	0.196156	1.780616	0.349968
NDUFA13	1.495015	0.787828	2.837	0.218568
ZNF566	0.157601	0.010847	2.28988	0.175994
ZNF529	0.525803	0.284504	0.97176	0.04023
ZNF284	11.98716	0.143193	1003.484	0.27152
DLEU7	1.340333	0.306122	5.868552	0.697438
KRT73	0.754616	0.239952	2.373162	0.630079
AIDA	1.211074	0.698587	2.099523	0.495113
CD300LF	1.107727	0.809796	1.51527	0.522125
CYP2R1	0.943721	0.340035	2.619173	0.911444
LRRC70	2.460214	0.177239	34.14974	0.50237
ANKRD46	1.114819	0.666122	1.865757	0.679111

PIP5K1C	1.924821	1.149705	3.222511	0.012755
CYP4F2	0.552328	0.1586	1.923496	0.351109
ZBTB6	1.165154	0.190962	7.109174	0.868431
TAS2R42	1.266943	0.088892	18.0572	0.861441
POLR3C	0.244961	0.110221	0.544412	0.000556
WWOX	0.120331	0.008193	1.767324	0.122449
BCL9L	2.377181	1.279371	4.417005	0.006156
POLR1D	0.519714	0.130595	2.068244	0.353025
KIF18B	0.08586	0.003461	2.130016	0.134016
ZNRF1	0.359422	0.032286	4.001253	0.405283
CYP4F12	0.091307	0.007379	1.129817	0.0622
ZNF749	0.066846	0.005861	0.762403	0.029376
BTLA	1.454495	0.547169	3.86637	0.452592
ZNF17	1.750872	0.744916	4.1153	0.198937
GPAT2	1.73999	0.797156	3.797957	0.164306
TOR3A	0.730249	0.326171	1.634923	0.444575
PPP1CC	0.651398	0.314257	1.350229	0.249093
ZNF555	1.677192	0.460998	6.101917	0.432578
NAP1L3	7.230353	0.996532	52.45992	0.050403
PRELID2	0.358203	0.073448	1.746946	0.204116
BACE1	2.81393	0.942765	8.398911	0.063692
RGS9BP	0.074863	0.012751	0.439542	0.004103
SLC36A3	0.209171	0.006754	6.478234	0.371725
RXRA	1.535214	1.146076	2.056479	0.004052
ANKRD37	1.530944	0.988043	2.372154	0.056634
NUDT17	0.078925	0.007934	0.785124	0.030284
ZNF75D	0.453473	0.118849	1.730247	0.247071
KRT10	1.033002	0.55641	1.917819	0.918078
CD300E	0.628834	0.072597	5.446926	0.673657
NKRF	0.740282	0.292188	1.875567	0.526068
GLDN	0.791218	0.212704	2.94318	0.726794
FCAR	1.08736	0.708175	1.669578	0.701865
KPNA4	0.606482	0.356511	1.03172	0.065071
ZNF501	0.281365	0.018446	4.291778	0.361689
ZNF197	0.27535	0.022479	3.372797	0.313016
SPATA12	6.030019	0.498503	72.94058	0.157761
AQP7P1	1.339858	0.285916	6.278835	0.710464
RPS23	1.168738	0.703758	1.940934	0.546853
GNG2	0.721606	0.453104	1.149217	0.169382
BTN3A2	0.681107	0.388472	1.194183	0.180078
AKAP14	0.001543	1.39E-05	0.170668	0.007011
INSIG1	1.4964	0.806881	2.775145	0.200883
ZNF396	4.252811	0.098297	183.9975	0.451387
TMEM222	0.901845	0.360459	2.256357	0.825247
ARHGAP3(	1.297067	0.661402	2.54366	0.449087
CYP4F3	0.870883	0.503575	1.506106	0.620843
SMYD4	0.682664	0.296019	1.574326	0.370546
GPATCH8	0.846805	0.180893	3.964117	0.832777
CEACAM1	2.434704	0.323069	18.34835	0.387862
NF2	0.026866	0.00092	0.784224	0.035626
SPATC1	0.036321	0.000743	1.776259	0.094823
UBE2H	1.202739	0.72518	1.99479	0.474526
HPDL	0.741125	0.267965	2.049774	0.563816
KATNA1	0.273715	0.093259	0.803348	0.018346
ARAP1	1.754159	1.096375	2.806589	0.019095
KIF24	0.177281	0.026387	1.191056	0.075065
PDE2A	0.384645	0.048369	3.058824	0.366455
PRG2	0.979497	0.836596	1.146806	0.796811

PRR5	1.49803	0.840801	2.668996	0.170214
ZFP91	0.279636	0.10695	0.731146	0.009362
BCDIN3D	1.946442	1.020685	3.711853	0.043163
LYRM7	0.427004	0.230698	0.79035	0.00675
BCR	1.137242	0.367747	3.516871	0.823326
FSCN2	0.357105	0.015829	8.056221	0.517204
SPIN4	1.016139	0.642957	1.605921	0.945339
SPIN2B	1.388465	0.128561	14.99546	0.786908
HYAL3	0.700987	0.505025	0.972985	0.033698
CXCR3	2.197897	1.065007	4.535886	0.033144
ZNF397	0.667397	0.188563	2.362175	0.530632
TPCN1	1.020874	0.399526	2.608551	0.965573
LILRB4	1.853926	1.118633	3.072539	0.016625
TNFRSF4	1.746003	1.151746	2.646873	0.008652
HEXIM1	0.719715	0.107432	4.821556	0.734667
PDZD7	1.922505	0.44321	8.339224	0.382626
POFUT2	0.470173	0.113424	1.948988	0.298257
ERCC6L	1.261048	0.331165	4.801958	0.733861
TMEM17	0.724763	0.304734	1.723736	0.466479
TNFRSF18	2.728559	1.437371	5.17962	0.002145
RTN4RL2	10.01929	1.29263	77.66035	0.02741
ZDHHC17	0.665533	0.343374	1.289946	0.227851
P2RY4	0.289224	0.021098	3.964876	0.353029
ZNF395	0.853306	0.511919	1.422354	0.542843
ZACN	2.718482	0.101124	73.08036	0.551504
PPARA	0.120471	0.007114	2.039972	0.142626
KANK3	0.466661	0.052799	4.124526	0.493024
EMID1	0.787731	0.570702	1.087295	0.14678
ACTL7A	0.021531	0.001162	0.398808	0.00996
RHD	0.954011	0.693058	1.313219	0.772769
ESPN	1.078051	0.660876	1.758566	0.763402
GPR141	1.949377	0.495215	7.673577	0.339694
TMPRSS6	1.00087	0.074602	13.42773	0.999476
TMEM216	0.766179	0.343685	1.708049	0.514954
RPS19BP1	1.442938	0.688462	3.024235	0.331437
TEAD1	0.040571	0.001407	1.169544	0.061672
OR2AK2	0.504925	0.02127	11.9864	0.672378
PLCD1	1.414147	0.62361	3.206831	0.406807
ENTPD5	0.350509	0.041291	2.975389	0.336686
MITF	0.632055	0.048145	8.297646	0.726914
HEATR4	0.150218	0.016201	1.392883	0.095251
NAP1L1	0.895314	0.435638	1.84003	0.763515
LILRA5	1.169194	0.880225	1.553027	0.280507
CMC1	0.577259	0.2143	1.554962	0.277123
SLIT1	0.371557	0.054253	2.544637	0.313196
AKR1C1	0.267424	0.017926	3.989547	0.33882
SPATA21	0.480704	0.037095	6.229254	0.575188
RNF220	1.113698	0.514455	2.410944	0.78464
ZNF546	0.559337	0.040059	7.809929	0.665791
TSPYL4	0.936647	0.365063	2.403168	0.89171
MT1X	1.409371	1.126672	1.763003	0.002663
GCNT1	1.500708	0.778917	2.891356	0.225042
SESTD1	0.928076	0.558635	1.541838	0.773193
FNBP1	0.962382	0.493051	1.878463	0.91053
DYNC2H1	0.703903	0.230441	2.150137	0.537707
BCAM	0.054882	0.000957	3.146815	0.160011
RSBN1L	1.136771	0.531043	2.433414	0.741313
WDR86	1.173882	0.978363	1.408475	0.084592

EPOR	0.885834	0.621311	1.262976	0.502944
TAF9B	0.04788	0.003839	0.597113	0.018251
PCDHB13	0.38381	0.044788	3.28908	0.382287
FPR3	2.626503	1.189093	5.801494	0.016926
KCNJ11	0.369676	0.013876	9.848993	0.552387
COL4A1	2.762821	0.661039	11.54725	0.163714
GJA4	3.327965	0.81427	13.60158	0.094147
PTMA	1.120909	0.755411	1.663251	0.570794
HSPA14	0.095259	0.033443	0.271337	1.07E-05
SIRT7	2.225555	1.021804	4.847403	0.043981
IFT140	1.337033	0.640029	2.793089	0.439674
TLR5	1.366912	1.009478	1.850907	0.043284
USP7	0.803225	0.299087	2.157134	0.66376
NANOS3	0.738608	0.49105	1.110971	0.14575
NHLRC1	0.878132	0.062561	12.32586	0.923186
COX8C	0.183768	0.016654	2.027741	0.166697
PLEKHN1	0.549058	0.112138	2.688336	0.459442
ZNF385C	0.647815	0.345129	1.215964	0.176588
MAGEH1	0.590148	0.135734	2.565864	0.481858
TET3	0.468621	0.037552	5.847993	0.556152
ZNF286A	1.057368	0.291023	3.841707	0.932464
ISG15	2.100096	1.40993	3.1281	0.000262
EXD3	1.135377	0.524978	2.455496	0.746994
ZKSCAN4	0.545894	0.304755	0.977838	0.041819
DHRS4L2	1.00313	0.47644	2.112063	0.993435
SAMD11	1.917617	0.496398	7.407875	0.345048
VMAC	1.476046	0.270068	8.067258	0.653202
SPRY4	0.036889	0.000122	11.11225	0.257175
TRPV2	1.530717	0.925777	2.530948	0.097041
TMEM203	2.854888	0.93703	8.698104	0.064959
KBTBD12	1.108284	0.138464	8.870876	0.922821
DNAJB13	0.054429	0.004032	0.734715	0.028371
TCEA1	0.748564	0.30222	1.854104	0.531444
NHEJ1	0.089524	0.008388	0.955445	0.04575
FANCA	1.962661	0.046061	83.6283	0.724664
SECISBP2	0.545386	0.278102	1.069556	0.077685
OR52B6	1.608123	0.115596	22.37163	0.723586
SEMA4D	1.601811	0.939186	2.731937	0.083697
LIN28B	0.378941	0.027503	5.221152	0.468415
DNAH17	0.293914	0.018598	4.644874	0.384595
MCRS1	0.866548	0.275759	2.72305	0.806309
TMEM72	0.082638	0.002591	2.635342	0.158121
FANCM	0.267679	0.025891	2.767439	0.268787
ZNF70	0.292568	0.032216	2.656983	0.274896
CARD9	1.2187	0.862821	1.721363	0.261631
PEAR1	1.704091	0.876157	3.314392	0.116312
TMEM220	0.157421	0.029569	0.838083	0.030235
EIF4EBP1	1.863341	0.694493	4.999386	0.216473
P2RX2	0.574477	0.001171	281.9308	0.860813
TTC24	0.767644	0.054499	10.81259	0.844662
FAM122A	1.741926	0.548426	5.532761	0.346592
CLEC17A	0.670228	0.094159	4.770707	0.689456
LDLRAD2	1.704255	0.764018	3.801593	0.192777
PMS2CL	0.632264	0.023548	16.97625	0.784783
CYHR1	1.401145	0.465529	4.217149	0.548534
COL14A1	0.157973	0.014342	1.740016	0.131688
CPSF4L	0.904491	0.139606	5.860086	0.916143
KLHL17	1.228189	0.27518	5.481678	0.787691

PLA2G2C	0.233156	0.052635	1.032806	0.05518
ZSCAN23	1.862176	0.136882	25.33353	0.640623
RINL	1.046628	0.631206	1.735456	0.859796
MORN2	0.93711	0.435042	2.018596	0.868231
S100A3	1.30101	0.293964	5.757931	0.72879
UBQLN2	0.755098	0.362363	1.573486	0.453322
RILPL1	0.792759	0.407121	1.543684	0.49459
ZNF490	0.344424	0.048549	2.443453	0.286312
CLCN1	1.580653	0.277869	8.991522	0.60573
ARL4C	1.38462	0.87911	2.180811	0.160301
TREML4	1.339475	0.469216	3.823813	0.58499
RAB42	2.64423	0.147918	47.26902	0.508645
SCGB1C1	0.673126	0.167005	2.713079	0.577826
GPR89B	0.444253	0.151718	1.300838	0.138831
EYS	0.15332	0.025219	0.932113	0.04172
MAPK12	0.787549	0.49039	1.264776	0.323092
COL4A5	0.977385	0.731423	1.306059	0.877092
AGRN	1.21642	0.32101	4.609448	0.77317
FAM166A	0.84955	0.111983	6.445034	0.874688
ZNF626	0.364665	0.119058	1.116938	0.077341
HEPACAM	0.955306	0.365489	2.496956	0.925686
ZC3H6	0.240498	0.036747	1.574008	0.137092
PRKAR1B	0.614181	0.288324	1.308314	0.206433
DCUN1D3	0.999287	0.571736	1.746565	0.998002
POTEE	1.795482	0.880631	3.660732	0.107347
ZNF793	0.211364	0.010252	4.357774	0.314125
COMMD6	2.402609	0.70184	8.224849	0.162692
IL17REL	2.206105	0.20913	23.27212	0.510398
RUFY4	1.390534	0.032458	59.57123	0.863461
ZNF383	0.245153	0.062673	0.958941	0.043362
HES4	1.11726	0.868187	1.437789	0.388908
ZNF669	0.724777	0.316221	1.661189	0.446872
CENPP	0.443632	0.114578	1.717698	0.239307
PLSCR1	1.483684	0.72518	3.035546	0.280063
ZNF559	0.336098	0.14447	0.781906	0.011372
SBK1	1.229703	0.348809	4.335234	0.747726
SLC38A3	0.895826	0.081896	9.799018	0.928185
OR6N2	0.011767	0.000417	0.331681	0.009113
GTF2F2	0.590389	0.235785	1.478295	0.260472
PRR19	3.663117	0.354625	37.83836	0.275809
ZP3	1.417689	0.485526	4.139518	0.52321
PDCD1	2.039776	0.748857	5.556049	0.163231
CLEC2A	0.421546	0.038189	4.653139	0.480783
GPR21	3.272548	0.599031	17.87816	0.171166
TCTEX1D4	10.21849	1.89769	55.02355	0.006814
SELL	0.85206	0.622341	1.166574	0.3179
CHM	0.513724	0.152748	1.727767	0.281783
CERKL	0.071409	0.006475	0.78755	0.031165
IER5L	2.805167	1.701267	4.625352	5.29E-05
FAM83G	0.122542	0.009221	1.62843	0.111717
HBA2	1.256153	0.758836	2.079394	0.375177
DUSP28	0.171513	0.054365	0.541098	0.002633
NBR1	1.589537	0.073064	34.58099	0.76805
RALGAPA2	0.18393	0.023499	1.439653	0.106776
NDOR1	0.436361	0.075999	2.505451	0.352384
NKAIN2	1.099997	0.401229	3.015714	0.853057
PAQR9	0.860384	0.463328	1.597703	0.633941
CLN3	2.892114	1.26003	6.638195	0.012238

FAM72B	0.963574	0.233428	3.977568	0.959089
SUMO2	0.660479	0.278127	1.568465	0.34723
NANOS1	0.021665	0.000931	0.504408	0.017028
HMX3	0.509869	0.04623	5.623324	0.582335
DPYD	1.361861	0.929227	1.995921	0.113284
S100A16	1.645962	0.888739	3.048353	0.113003
PTAR1	0.792486	0.429554	1.462059	0.456674
CC2D2B	0.663338	0.043014	10.22965	0.768704
RHCE	0.922704	0.607317	1.401875	0.706192
PARVB	7.257394	2.501746	21.0532	0.000265
SLC4A5	0.626004	0.11764	3.331207	0.582898
UROS	0.476029	0.269666	0.84031	0.010467
ZDHHC9	0.847647	0.277333	2.590773	0.771842
QRFP	0.411409	0.026534	6.378762	0.525392
TMEM120I	0.230612	0.03033	1.753438	0.15637
RBM34	1.155586	0.546103	2.445284	0.705338
NOXA1	1.393791	0.846664	2.294479	0.191724
BCL2L15	0.626342	0.212821	1.843354	0.395604
FZD9	1.207518	0.707168	2.061888	0.489728
SPRED3	0.150734	0.001256	18.09277	0.438561
ZNF548	0.590159	0.303405	1.147931	0.120292
MTF1	1.061181	0.596366	1.888282	0.839945
TMCO2	1.225839	0.083433	18.01067	0.88194
TMEM201	0.503085	0.078063	3.242169	0.469889
NHLRC3	0.324065	0.11141	0.942626	0.0386
HMX2	6.903505	0.53096	89.75893	0.139879
ZDHHC11	2.982066	0.279691	31.79478	0.365546
CNR2	0.041017	0.002232	0.753688	0.031527
ENTPD8	0.010877	0.000191	0.62073	0.028447
RPL14	0.673676	0.387741	1.170471	0.161069
BEND4	2.692264	0.859717	8.431014	0.089048
ZNF563	1.225387	0.258161	5.816419	0.798112
TMC3	1.052065	0.116905	9.46789	0.963887
FBF1	2.994179	0.113481	79.00102	0.511336
KLRG2	0.259179	0.031035	2.164477	0.212438
ASTL	0.253319	0.023449	2.736631	0.258118
MSL1	0.653951	0.245833	1.739603	0.39486
LRRK2	1.141573	0.465908	2.797091	0.772139
TRMT2B	0.083074	0.010014	0.689137	0.021174
FAM120A(	0.48158	0.18707	1.239747	0.129895
NOC2L	1.621817	0.585221	4.494529	0.352486
ZNF292	0.264437	0.067044	1.042991	0.057454
HUS1B	0.364455	0.114052	1.164622	0.088594
KCTD21	1.99666	0.569336	7.002288	0.280097
ADAT2	0.063459	0.010275	0.391942	0.002995
KIR2DL4	1.015583	0.462873	2.228275	0.969234
ZNF567	2.642912	0.247123	28.26518	0.421501
NDUFA4	0.485183	0.129358	1.819771	0.28359
ALKBH2	0.862618	0.407132	1.827685	0.699666
RNFT1	0.605458	0.287261	1.27612	0.187164
RELN	0.215654	0.015455	3.009116	0.253968
FAM111B	0.0484	0.003507	0.668059	0.023749
LITAF	1.170455	0.603468	2.270155	0.641453
VSTM1	0.805019	0.628069	1.031822	0.086785
TMEM120,	1.811575	1.158909	2.831806	0.009133
ARID2	0.534753	0.223462	1.279682	0.159718
SF3B3	0.72426	0.33649	1.558895	0.409472
BLOC1S3	0.109583	0.00611	1.965238	0.133292

SP6	0.216122	0.032891	1.420113	0.110752
PLAC9	0.49423	0.070518	3.463822	0.478076
ZNF573	0.626851	0.331577	1.185068	0.150609
ZNF527	0.671725	0.042315	10.66314	0.777878
S100A13	0.326326	0.020553	5.181114	0.427286
ZNF33A	0.299974	0.082193	1.094792	0.068325
ZNF600	0.805865	0.369711	1.756555	0.587187
BTBD8	0.151785	0.00469	4.91258	0.287919
MAOA	0.861461	0.534731	1.387828	0.539928
TSPYL1	0.854253	0.420237	1.736516	0.663403
PNRC2	1.317059	0.593447	2.922998	0.498353
FHIT	0.546592	0.263495	1.133848	0.104687
ZKSCAN3	0.738158	0.25939	2.10061	0.569379
RRP7A	0.983979	0.562773	1.720437	0.95482
LIN54	0.605529	0.299021	1.226222	0.163477
FAM53B	3.225734	1.448143	7.185312	0.004155
ALG1L	2.58987	0.661749	10.1359	0.171653
GSPT2	1.208068	0.644285	2.265188	0.555637
CXCL17	5.938183	0.957098	36.84262	0.055764
HMGB1	0.65201	0.384307	1.106193	0.112792
SH2D5	2.464216	0.048734	124.6024	0.652311
ZFP92	1.427636	0.443649	4.594052	0.55048
NCR1	0.108388	0.010737	1.094109	0.059603
BLOC1S2	0.091102	0.007334	1.131697	0.062361
SYCP2	0.775774	0.153535	3.919794	0.758701
IL1RAP	1.114799	0.590127	2.105947	0.737736
PAX5	0.124225	0.006979	2.211183	0.155671
ZNF699	0.379755	0.020352	7.086099	0.516674
TDRD7	1.014151	0.593391	1.733262	0.959017
KIAA0895L	0.497792	0.121102	2.046181	0.333432
HLA-DRB1	1.074266	0.909892	1.268334	0.397839
MYT1	0.894516	0.406799	1.966966	0.781571
AKR1C3	0.989327	0.767496	1.275273	0.933983
SPATS2L	1.146946	0.7693	1.709976	0.501049
ZNF250	1.725306	0.415043	7.171979	0.45309
WDSUB1	0.442142	0.213302	0.916489	0.028203
ZNF79	0.363009	0.090891	1.449822	0.151505
S100A4	1.851619	1.140379	3.006449	0.012733
PLEKHG4	1.061491	0.67665	1.665209	0.795055
FAT4	5.560219	0.140202	220.5105	0.36089
ZNF681	2.579936	0.985478	6.754154	0.053585
ACADSB	0.658316	0.120155	3.606846	0.629987
STK40	3.03364	0.912127	10.08957	0.070303
TMEM63A	1.542762	0.889755	2.675023	0.122589
CTSE	0.455937	0.20214	1.02839	0.058423
SEMA4A	1.27092	0.940954	1.716594	0.118019
MPHOSPH	0.565937	0.227939	1.405132	0.219858
GREB1	6.782571	0.115891	396.9526	0.356525
SIRPB2	0.483411	0.065944	3.543712	0.474501
ZNF766	0.963852	0.298735	3.109816	0.950878
RYR1	2.50063	1.044832	5.984835	0.039546
SRGAP3	0.413684	0.149561	1.144249	0.08906
TUBB	0.706164	0.385157	1.294711	0.260647
LCOR	0.717793	0.406361	1.267903	0.253348
SUPT5H	1.082272	0.472622	2.478327	0.851635
XPNPEP3	1.372931	0.303839	6.20375	0.680424
ZNF107	2.335708	0.163374	33.39282	0.531934
PPIA	0.51974	0.250709	1.077462	0.078512

ZNF471	0.410409	0.096012	1.75432	0.229518
ZNF836	1.962546	0.417994	9.214444	0.392835
ZNF493	0.272006	0.069018	1.071999	0.0628
GTF2IRD2	0.769523	0.093662	6.322352	0.807379
SUPT3H	0.739352	0.301973	1.810234	0.508621
NIF3L1	0.699593	0.307478	1.591758	0.39436
ATP2A1	2.483733	0.148749	41.47195	0.526491
POM121	0.300228	0.089147	1.011102	0.05212
ZBTB44	0.403435	0.1832	0.888431	0.024217
GIMAP5	1.627583	1.146533	2.310468	0.006431
NLGN3	0.026272	0.002875	0.240027	0.001263
CD55	0.648144	0.347086	1.210334	0.173551
ZNF565	0.478422	0.081828	2.797166	0.413187
NTNG2	0.971692	0.54914	1.719389	0.921436
WDR5	1.074801	0.193347	5.974735	0.934313
LONP1	1.064808	0.541277	2.094707	0.855663
TRRAP	0.769814	0.351941	1.683844	0.512402
NUDT11	1.251514	0.459516	3.408556	0.660751
FUT4	0.580164	0.421591	0.798382	0.000831
ASB13	1.729943	0.987212	3.031471	0.055493
ZNF34	1.115684	0.506247	2.458783	0.785993
ZNF140	0.878065	0.259665	2.969202	0.834299
INCA1	5.180781	0.743152	36.11709	0.096847
PTPN1	1.02066	0.488557	2.132295	0.956616
EVL	0.821841	0.548906	1.23049	0.340703
THEM5	0.508569	0.068949	3.75121	0.5072
EPHB4	1.187114	0.762111	1.849126	0.448119
PRTN3	1.152792	0.971363	1.368106	0.103649
ZNF765	1.458497	0.166441	12.7806	0.733258
ZNF124	2.406522	0.526431	11.00115	0.257421
XRCC6	0.568894	0.182912	1.769382	0.329904
S100A5	0.687152	0.066237	7.128666	0.753252
TSC22D2	1.580022	0.75159	3.321584	0.227557
ASMT	0.647	0.038462	10.88385	0.7624
ZNF569	15.40476	1.184047	200.4199	0.036706
ARMCX4	1.211374	0.221183	6.634458	0.825084
YRDC	0.602068	0.240779	1.505469	0.27789
ZNF777	1.493822	0.72726	3.06837	0.274481
PIK3R4	0.743902	0.275738	2.006941	0.559049
ZNF775	1.579881	0.772062	3.232932	0.210618
ZNF605	0.570351	0.204102	1.593813	0.284199
TRAPPC2	0.074372	0.016082	0.343932	0.000881
MYL6B	0.807816	0.490521	1.330356	0.401754
ZNF799	0.563022	0.059066	5.366722	0.617528
SIAH1	0.699379	0.212712	2.299499	0.556002
IPO4	1.120119	0.578995	2.166975	0.736181
NCOR2	3.194282	1.473372	6.925226	0.003265
SULT1A1	1.814334	0.531007	6.199179	0.341978
ARL9	1.239106	0.7348	2.089528	0.421321
PRPF40A	1.239292	0.295842	5.191442	0.769107
GDAP2	0.514935	0.160324	1.653891	0.264915
TCEAL3	1.041812	0.687994	1.577588	0.846581
ANAPC7	0.461471	0.174688	1.219063	0.118687
TPK1	1.600784	0.880424	2.910541	0.122963
SLC6A9	1.272624	0.527715	3.06903	0.591425
AFAP1	20.02698	0.638857	627.8087	0.088185
NACA	0.45639	0.09621	2.164965	0.323379
MYO18A	1.418094	0.567704	3.542327	0.454545

MAN2A2	0.521511	0.267041	1.018473	0.056603
MME	1.44883	0.289634	7.24744	0.651718
FAM72A	0.521467	0.13379	2.032496	0.348199
CACNA1H	0.87112	0.2707	2.803286	0.817021
SULF2	1.282561	1.00008	1.644832	0.049926
HBG2	0.93567	0.820309	1.067255	0.321968
PLXNB2	3.048388	1.455657	6.383832	0.003121
XRCC2	1.318661	0.568523	3.05857	0.519312
MYO6	0.186246	0.017003	2.040131	0.168776
HDAC2	0.362787	0.126151	1.043306	0.059931
ZNF782	0.67034	0.085457	5.258248	0.703508
ZNF846	3.809953	0.270486	53.66551	0.321624
MMP1	0.735029	0.099525	5.428483	0.762837
TCF4	1.560709	1.043152	2.335053	0.030351
ZNF136	0.143137	0.018834	1.087835	0.060299
ZKSCAN5	1.171348	0.155813	8.805796	0.877875
ZNF502	0.376057	0.113889	1.241723	0.108553
TRAPPC4	1.162948	0.496215	2.72553	0.728298
TTC30B	2.144889	0.801175	5.742251	0.128822
TECPR2	0.903102	0.071475	11.41093	0.937228
TLR7	1.469356	1.080017	1.999051	0.014284
ZFP62	0.875549	0.235257	3.2585	0.842877
ERI2	0.483099	0.170147	1.371664	0.171807
TOMM7	0.953378	0.291264	3.120645	0.937101
HSH2D	0.768276	0.578881	1.019636	0.067954
TRPV1	0.012982	0.000187	0.900463	0.044597
ZNF33B	0.601466	0.330243	1.095439	0.096519
ZNF512B	0.87385	0.336921	2.266446	0.781541
AMZ2	0.530383	0.157719	1.783593	0.305434
ZNF431	0.582545	0.284261	1.193825	0.139937
NF1	0.190217	0.042521	0.850927	0.02992
VKORC1L1	0.471927	0.182266	1.221921	0.12185
ZNF418	0.301267	0.051951	1.747074	0.180956
DAPK1	2.016489	1.335369	3.045022	0.000852
HLA-DQA	1.096891	0.917176	1.311819	0.311072
COL27A1	0.594559	0.026299	13.44145	0.743819
GM2A	0.531864	0.218992	1.291729	0.16315
S100A2	1.909566	0.155938	23.3839	0.61279
ZNF700	0.757928	0.353278	1.62607	0.476672
CD47	0.711922	0.38438	1.318572	0.279906
TLE1	0.715652	0.445693	1.149128	0.166155
MAML3	0.695005	0.27291	1.769929	0.445545
STRN3	0.671737	0.381255	1.183538	0.168557
ZNF239	0.712068	0.248714	2.038651	0.526899
CHRNG	0.08504	0.005048	1.432544	0.087174
ZSCAN16	0.490411	0.205655	1.16945	0.108069
ADA	0.95128	0.683657	1.323666	0.766978
ARID5A	1.853393	0.686529	5.003527	0.223336
PPTC7	0.652603	0.362992	1.173279	0.153863
TOMM20L	0.552277	0.081704	3.733098	0.542567
RGPD4	5.610314	0.202197	155.6682	0.309074
NHLRC2	0.284502	0.133799	0.604951	0.001092
ZFP28	0.380896	0.039192	3.701865	0.405461
CBWD3	0.224115	0.088104	0.570095	0.001691
SCN8A	0.362695	0.04808	2.736003	0.325256
LAMB3	0.141077	0.002657	7.490701	0.333864
KPNA5	0.472933	0.157077	1.423918	0.183017
ANKRD36I	0.35645	0.144477	0.879419	0.025166

ARHGEF12	0.914602	0.322105	2.596975	0.866862
PDLIM7	1.82259	0.830162	4.001429	0.13464
FLNA	1.833373	1.167878	2.878088	0.008428
RPS26P11	1.827	0.75135	4.442575	0.183726
SRGAP1	1.093464	0.356245	3.356297	0.875913
FAM3C	0.760226	0.22865	2.527639	0.654715
SLC39A10	1.242716	0.761863	2.02706	0.384056
CASP4	0.296023	0.048856	1.793632	0.185386
AP2A1	3.150661	1.270551	7.812883	0.013259
ZNF585A	0.521049	0.16934	1.60324	0.255611
FUT11	1.459806	0.377373	5.64702	0.583634
ANXA4	0.92606	0.586509	1.46219	0.741684
LAGE3	0.96994	0.526071	1.78832	0.922108
WDR5B	1.01425	0.468623	2.195161	0.971348
WDR45	1.131016	0.245559	5.209323	0.874465
METTL9	1.106484	0.4617	2.651738	0.820496
ZNF138	3.65067	0.676076	19.71286	0.132323
ZNF429	0.214802	0.038531	1.197469	0.079362
ZNF470	2.005295	0.377312	10.65752	0.414288
SERTAD1	1.624106	1.054791	2.500704	0.027652
ZNF100	1.772788	0.037817	83.10551	0.770546
ZNF398	0.984569	0.171602	5.64899	0.98608
ANXA6	2.176823	0.979735	4.83657	0.056172
ZNF441	1.26706	0.143822	11.16272	0.831162
GMFB	1.105879	0.599848	2.038798	0.747107
SIGLEC15	7.146597	0.358271	142.5565	0.197813
ZNF420	2.781925	0.886428	8.730668	0.079539
ZNF763	1.263568	0.330129	4.836306	0.732644
ZMYM1	0.687972	0.310833	1.522701	0.356189
MAFG	4.769615	1.442468	15.77104	0.010456
ARRDC1	2.909452	1.431855	5.911852	0.003154
KIAA1671	2.086667	0.965494	4.509793	0.061392
IGF2R	1.165035	0.91228	1.487817	0.220882
GAL3ST4	0.384541	0.115163	1.284017	0.120285
DYNC1H1	1.56947	0.699984	3.51899	0.273904
PCBP2	1.042347	0.203332	5.343425	0.960332
ZGPAT	1.419219	0.227642	8.848042	0.707696
SLC25A29	0.757307	0.476286	1.204139	0.24005
PGAP1	0.318904	0.057438	1.770611	0.191306
SRC	6.313258	2.269062	17.56551	0.000417
ZNF682	0.741602	0.259552	2.118937	0.576782
ZNF772	5.95931	1.686987	21.05137	0.005569
ZNF257	1.628937	0.166679	15.91939	0.674843
ADAM32	0.074827	0.000333	16.80736	0.347992
ACSL5	0.613998	0.134342	2.806215	0.529275
LRRC8B	2.521828	0.172473	36.87301	0.499143
ABCB8	2.084575	0.657539	6.608655	0.212108
SND1	2.183287	1.033536	4.612072	0.040716
ZNF785	2.821931	0.861521	9.24329	0.086577
SULT1A2	2.107613	0.917559	4.841139	0.078887
NEK5	0.17814	0.013012	2.438913	0.196294
PSMD12	0.027979	0.001536	0.509478	0.015717
PIWIL2	0.803692	0.012012	53.77327	0.918834
SLC22A4	0.959528	0.590313	1.559671	0.867618
ENTPD4	1.111919	0.493767	2.503941	0.797844
C1D	1.643903	0.514254	5.255023	0.401839
TBC1D9B	0.986543	0.472064	2.061725	0.971262
SERPINA1	1.243605	0.935805	1.652645	0.132927

TPSB2	1.211976	0.993185	1.478966	0.058406
KANK2	1.611721	0.693488	3.745763	0.267302
GTF2E2	0.264447	0.111659	0.626301	0.002497
IL27	3.049602	0.43168	21.54389	0.263654
GUCA2A	0.727123	0.139475	3.790706	0.705249
ZNF165	0.488041	0.229985	1.035653	0.061664
SYNGAP1	0.785181	0.120742	5.105999	0.800137
FITM2	3.466271	0.069187	173.6591	0.533627
BLM	0.471095	0.193778	1.145282	0.096778
ZNF720	0.80243	0.105465	6.105298	0.831644
DDI2	0.024304	0.001	0.59058	0.022401
SVIL	1.862147	0.653563	5.305675	0.244499
TRIM33	0.259981	0.080233	0.842425	0.024715
LRP10	1.448635	0.881672	2.380186	0.143499
PELI1	1.100301	0.806383	1.501348	0.546636
ZNF655	0.35461	0.063179	1.990341	0.238827
MRPL21	1.88771	0.612335	5.819449	0.268684
LYPD2	1.756257	0.878618	3.510557	0.11099
UAP1L1	1.420958	0.833364	2.422855	0.1969
FBXL22	9.949526	0.829122	119.3951	0.069961
ZNF786	0.731706	0.342563	1.562902	0.41982
ZNF517	0.873151	0.05657	13.47706	0.922608
ZNF675	0.640824	0.117505	3.494781	0.607128
SLC22A5	1.723883	0.888885	3.343261	0.107087
DACT3	1.251381	0.442339	3.54017	0.672556
ADARB1	0.4597	0.149879	1.409963	0.174103
HTT	1.089533	0.497037	2.388315	0.830439
OR6N1	7.032605	0.497966	99.31917	0.148779
C5AR1	1.180938	0.960056	1.452638	0.115456
VEPH1	1.380615	0.122809	15.52087	0.793897
SHPK	0.373953	0.140909	0.992416	0.048239
IPP	1.12093	0.471214	2.666483	0.796261
MAP3K5	2.98467	1.425062	6.251135	0.003743
CYP2F1	0.727327	0.047049	11.24362	0.81973
GSTK1	1.254915	0.610039	2.581495	0.537232
HNRNPAB	0.45683	0.22001	0.948565	0.035587
STMN3	0.97894	0.641396	1.494122	0.921406
PDGFA	7.877556	0.408317	151.9798	0.171684
GYPE	1.019986	0.757888	1.372725	0.8961
SPN	0.667262	0.277556	1.604141	0.365999
ZNF695	2.347825	0.207219	26.60123	0.49075
ZNF628	2.012751	0.760736	5.325321	0.15881
SLC2A10	1.189553	0.361461	3.914773	0.775181
ZNF665	3.038352	0.417159	22.12964	0.272656
RPF2	0.494179	0.222844	1.095891	0.082808
SLC28A3	0.971694	0.155255	6.081556	0.975519
FAM177B	16.37313	1.694392	158.2157	0.015709
MIB2	2.366011	0.906969	6.17222	0.078346
MYO5A	0.670292	0.367753	1.221722	0.191512
GZMM	1.234504	0.820359	1.857725	0.312337
ATG7	0.959458	0.582832	1.579461	0.87073
SIPA1L1	0.446105	0.13272	1.499467	0.191887
TTC30A	0.833292	0.23233	2.988746	0.779587
ELANE	1.062834	0.852584	1.324931	0.587917
RAB40C	1.305464	0.602034	2.830795	0.499679
PIGN	0.253499	0.084672	0.758951	0.014169
ZNF624	14.80203	0.968838	226.1471	0.05272
HHLA3	0.709101	0.062004	8.109576	0.782172

HOXA4	0.854907	0.395201	1.849356	0.690485
TOPORS	1.208106	0.478735	3.048699	0.688939
BCO2	0.114794	0.007625	1.728282	0.117696
ENTPD6	1.93062	0.727534	5.123187	0.186455
KLKP1	0.503952	0.038984	6.514681	0.599729
FAR1	0.899861	0.534075	1.51617	0.691805
ZNF841	0.62242	0.21297	1.819066	0.386212
VN1R5	0.288143	0.0271	3.063732	0.30223
ZNF615	1.075147	0.465309	2.484247	0.865351
CDC42SE1	0.668788	0.388362	1.151702	0.146877
MPEG1	1.319738	0.895985	1.943903	0.160289
SERPINB2	1.096044	0.854655	1.405609	0.469961
DPP4	1.282552	0.38021	4.326397	0.688316
PDCD1LG2	0.124846	0.012372	1.259827	0.07771
ZNF433	1.473244	0.486429	4.462	0.493143
DNAH10	0.848149	0.345358	2.082929	0.719385
SPTAN1	1.922704	0.806566	4.583367	0.140226
NMB	2.121125	1.093735	4.113584	0.026075
KLHL14	2.433561	0.090674	65.31332	0.59622
FAM114A1	1.887834	0.211318	16.86519	0.569538
RPE	0.18467	0.03537	0.964182	0.045154
ZNF460	0.256634	0.048237	1.365362	0.110756
CR1L	1.67318	0.298882	9.366684	0.558071
PHF2	5.054139	1.467223	17.40998	0.010244
RPS26	1.324364	0.652241	2.689096	0.436918
PSAP	1.237966	0.822258	1.863842	0.306527
S100A10	1.185346	0.939005	1.496312	0.152574
LHFPL5	1.575173	0.24229	10.24049	0.634274
RPL37A	1.185956	0.296469	4.744143	0.809467
CFD	1.401608	1.018292	1.929217	0.038344
EME2	1.258372	0.10568	14.98387	0.855711
KLHDC1	0.211036	0.007363	6.048304	0.363505
ZNF81	0.089465	0.00849	0.942725	0.044532
TAF13	0.421274	0.090141	1.968819	0.27183
ZNF780A	0.53639	0.040492	7.105533	0.636564
ATAD3A	0.790436	0.389871	1.602553	0.514298
FAM118B	2.240713	0.650481	7.718591	0.201076
ZNF461	2.656584	0.347108	20.33209	0.346735
SLC9A8	2.191319	0.91901	5.225052	0.076816
OCLN	2.22719	0.023966	206.9769	0.729111
ZNF181	1.48997	0.619562	3.583196	0.373115
ZNF44	0.165384	0.013128	2.083475	0.163891
GPAA1	1.272823	0.676641	2.394296	0.454278
ADAMTSL1	1.52081	0.781239	2.960504	0.217364
SGTB	0.143883	0.012261	1.688422	0.122813
ZNF790	0.923521	0.172471	4.94514	0.925957
PRB3	0.160292	0.009455	2.717523	0.204902
MYO1C	9.181658	0.642191	131.2738	0.102331
NKIRAS1	1.518149	0.675794	3.410475	0.312012
SLC22A12	0.022026	0.000429	1.129566	0.057523
KIF13B	1.282841	0.574625	2.863918	0.54328
ADH5	0.869868	0.24524	3.085432	0.829131
TEAD4	1.454988	0.332247	6.371739	0.618723
SPG7	0.227417	0.060167	0.859584	0.029036
ZNF677	0.594529	0.04972	7.109057	0.681274
ZNF823	1.762272	0.728113	4.265274	0.208976
ZNF311	2.734815	0.638297	11.71745	0.175351
ZNF347	0.11338	0.007602	1.690985	0.114345

PLCG2	0.741726	0.404931	1.358643	0.333295
FCHSD1	130.6665	9.734468	1753.947	0.000236
ZNF71	0.230331	0.047546	1.115808	0.068175
S100A6	1.166178	0.805862	1.687599	0.414909
RPL12	0.934651	0.315551	2.7684	0.90291
DNM3	0.962195	0.332478	2.784602	0.943333
ZNF121	0.377465	0.172418	0.826366	0.014809
MPZL1	0.529321	0.23587	1.187863	0.122945
VPS13A	0.159214	0.042896	0.590934	0.00603
MBP	1.148373	0.609537	2.163543	0.668585
ELOVL2	0.495257	0.041098	5.968128	0.580059
LEKR1	0.037608	0.001675	0.844449	0.038785
SNHG12	0.594392	0.251336	1.405692	0.236191
CLEC9A	1.913843	0.903606	4.053529	0.090029
KEL	0.725592	0.52363	1.005451	0.053942
NOL8	0.610381	0.247492	1.505362	0.28378
IRAK4	0.634385	0.283448	1.419822	0.268216
MRPL42	0.642325	0.274336	1.503926	0.307815
ENTPD7	0.575766	0.19816	1.672925	0.310384
FCGR1B	1.075806	0.816282	1.417841	0.60392
ZNF335	2.461717	0.535077	11.32556	0.247317
RPS4X	0.858783	0.522762	1.410793	0.54777
ZNF273	0.201923	0.038092	1.070385	0.060103
ZNF84	0.398877	0.169799	0.937008	0.03492
MAK16	0.563624	0.251758	1.261814	0.163194
ZNF667	0.397252	0.129109	1.222299	0.107417
AVPR1B	0.699114	0.06805	7.182383	0.7633
SIRPA	1.428955	1.021511	1.998912	0.037138
GRK6	1.692595	0.912627	3.139153	0.094947
PRIM1	0.492195	0.267222	0.906573	0.022924
SULT1C4	1.975703	0.842841	4.631247	0.117209
CD2AP	0.931059	0.44738	1.93766	0.848505
NUP62CL	1.173083	0.287226	4.791084	0.824038
SFI1	0.721055	0.15554	3.342684	0.676018
ZNF649	0.32488	0.077366	1.364249	0.124611
ZNF248	0.409417	0.163903	1.022696	0.055887
LPAR1	7.573875	2.027913	28.28701	0.002599
HIBCH	0.343399	0.156471	0.753638	0.007693
ZNF544	0.598376	0.245117	1.460743	0.259418
TMEM229I	2.562783	0.688198	9.543566	0.160643
ZNF770	0.530096	0.247985	1.13314	0.101529
MIER1	0.564098	0.170276	1.868765	0.348849
MAN1A2	0.324727	0.070952	1.486176	0.147225
SVIP	0.573431	0.050701	6.485515	0.653183
ZNF251	1.082635	0.119653	9.795808	0.943674
DDRGK1	0.45239	0.120273	1.701598	0.240584
TFDP1	0.688545	0.394619	1.201398	0.188873
CLEC4C	1.570755	0.752922	3.27693	0.228762
ZNF607	0.032602	0.001971	0.539209	0.016783
ZNF334	0.764808	0.130604	4.478659	0.766211
HSD17B11	1.157951	0.735141	1.823936	0.526974
ZXDA	0.856631	0.090633	8.096546	0.892589
RPS6KL1	0.102861	0.012234	0.864852	0.036294
QRICH1	0.236415	0.029411	1.900365	0.175038
CSF2RA	1.669594	0.936387	2.976912	0.08235
DDX42	0.104721	0.028108	0.390158	0.000772
RPL23A	0.584219	0.300828	1.134576	0.112483
SLC29A3	2.840257	1.522426	5.298818	0.001034

STYX	3.287266	0.263444	41.01873	0.355421
UBL5	0.482958	0.161668	1.442758	0.192409
HELZ	1.296877	0.494919	3.398314	0.596867
TMEM116	1.248992	0.347912	4.483835	0.733147
UCKL1	1.303605	0.597501	2.844155	0.505343
CARD11	1.513483	0.945714	2.422119	0.08411
ZNF485	0.530151	0.110981	2.532499	0.426405
SDAD1	1.905176	1.004112	3.614831	0.04855
HYLS1	1.64559	0.578763	4.678885	0.350174
MYL4	0.950187	0.701169	1.287642	0.741754
ZNF442	1.682407	0.663496	4.266033	0.273154
ZNF813	0.453629	0.103993	1.978779	0.292876
HOXC4	0.50131	0.085779	2.929772	0.443314
PIM3	1.895252	1.122248	3.2007	0.016787
ASPH	0.451541	0.083249	2.449142	0.356713
SPRED2	1.787084	0.895155	3.567729	0.099771
WWP2	0.720425	0.198397	2.616032	0.618215
GFPT1	0.676557	0.218322	2.096574	0.49834
UVRAG	0.891717	0.256879	3.095459	0.856769
ZNF26	0.187205	0.068074	0.514816	0.001169
ITSN2	3.216731	0.236599	43.73375	0.380237
NTRK1	0.25503	0.04447	1.462583	0.1252
MT1F	1.53624	1.164055	2.027424	0.00242
ZNF69	0.792607	0.395453	1.588625	0.512347
TXNRD1	1.026223	0.340136	3.096218	0.963356
NRARP	5.420237	1.3351	22.00507	0.018067
ZNF583	0.596182	0.184578	1.925654	0.387263
ZNF568	22.4305	0.613548	820.0295	0.090279
ZXDB	0.697276	0.257723	1.886499	0.477673
ZNF480	1.126871	0.44709	2.840229	0.80008
ZNF587	0.034869	0.000809	1.502654	0.080485
TPM2	1.331669	0.79673	2.225778	0.274433
SH3BGRL2	1.069343	0.755694	1.513172	0.705052
ZNF808	0.061191	0.006108	0.613021	0.017493
ANKRD35	1.092679	0.547864	2.179275	0.801327
YTHDF2	0.663665	0.309303	1.424014	0.292569
NBR2	0.375324	0.160918	0.875402	0.023333
HLA-DRB5	0.991426	0.855055	1.149547	0.909202
ATL1	0.89781	0.379419	2.124464	0.806227
MAFK	0.528865	0.078398	3.567658	0.513079
ZNF43	0.931717	0.146909	5.909088	0.940181
GPN1	0.568793	0.294126	1.099958	0.093575
PLN	1.903639	0.168993	21.4438	0.602346
ZNF28	2.603427	0.248892	27.232	0.424378
ZNF511	1.552961	0.796093	3.029405	0.196675
ZNF627	1.434525	0.826932	2.488553	0.1992
KCNRG	0.31714	0.048427	2.076888	0.231028
WDHD1	0.272306	0.0314	2.361463	0.237881
ZNF789	0.08341	0.0215	0.323586	0.000329
CTNND1	0.293498	0.025874	3.329289	0.32251
SLC34A3	0.027805	0.001503	0.514362	0.016104
SH2D1B	0.782284	0.286605	2.135232	0.631745
ARC	1.367814	0.330258	5.66501	0.665755
NUDT16	1.277538	0.558868	2.920377	0.56148
TLK1	0.335202	0.14097	0.797051	0.013389
LRBA	1.639958	0.646834	4.157883	0.297349
MMP17	0.260731	0.00473	14.37093	0.511102
BAZ1A	0.599546	0.286617	1.254134	0.174274

AKR1C4	0.862441	0.439945	1.690673	0.666533
COPS8	0.230106	0.050875	1.040764	0.056382
CCDC69	1.727713	0.847868	3.520583	0.132178
MDM4	0.594038	0.278351	1.267756	0.178123
KLHL9	0.876171	0.495599	1.548985	0.649312
NCOA6	0.371761	0.158748	0.8706	0.022659
STK39	0.508491	0.236495	1.093313	0.083353
CALM1	1.16662	0.680796	1.999133	0.57493
TTC37	0.653845	0.364159	1.173976	0.154783
TUSC1	1.592535	1.04885	2.418046	0.028977
PAPSS2	1.718207	1.081167	2.730599	0.022012
SLC9A6	2.902431	0.521322	16.15912	0.223842
EIF1AY	0.950945	0.791279	1.14283	0.59172
IPO9	0.408433	0.156329	1.067091	0.067634
CEP290	0.221424	0.065171	0.752312	0.01569
DLL1	1.226303	0.879817	1.709242	0.228523
ANKRD13I	0.638912	0.161942	2.520716	0.522352
UNC13B	2.849476	0.752399	10.7915	0.123259
LDB1	0.418634	0.070028	2.502652	0.339858
PPP1R14C	13.97508	1.951723	100.0668	0.008646
CTR9	1.294627	0.526171	3.185391	0.574033
F5	1.079819	0.74981	1.555072	0.679851
ZNF652	0.192661	0.049402	0.751354	0.017708
SMURF1	2.654194	0.208709	33.754	0.451839
SLC5A3	1.530878	0.239587	9.781767	0.652699
GPATCH3	1.552059	0.682595	3.529013	0.294246
CDC42BPE	1.98056	1.023223	3.833594	0.042551
PLXNB3	11.04616	1.225156	99.5936	0.032278
OXCT2	1.137897	0.233132	5.553981	0.873111
RPL10A	0.768044	0.250463	2.355207	0.644361
RCSD1	1.034427	0.532969	2.007695	0.920313
FAM169A	1.860104	0.08679	39.86643	0.691451
ZNF830	5.140559	1.505879	17.54812	0.008963
GRIN3A	7.147528	1.183831	43.1541	0.032039
CNOT7	0.49271	0.195711	1.240416	0.13294
TMEM184I	4.504938	1.877353	10.81015	0.000751
SCAMP5	2.525599	1.279773	4.984205	0.007558
ZNF521	1.097743	0.885212	1.361301	0.395654
LRIG2	0.357664	0.133468	0.958459	0.040921
GK	0.668061	0.19957	2.236336	0.512883
FOXJ3	0.641826	0.275937	1.492883	0.303204
ZNF358	1.392604	0.904979	2.142973	0.132081
SFT2D1	0.674602	0.240701	1.890672	0.454084
CD247	0.978224	0.748318	1.278765	0.872043
INPP5F	0.848478	0.016194	44.45446	0.935164
ARHGAP1:	0.056056	0.001525	2.06113	0.117183
SUCNR1	0.65951	0.473838	0.917938	0.013604
HMGN2	0.334855	0.118678	0.944808	0.038712
UBE2J1	0.673513	0.361067	1.256332	0.214027
GJC2	1.277279	0.906652	1.799415	0.161648
OPA1	0.306882	0.112798	0.834911	0.020706
DENND4B	1.392561	0.731392	2.651416	0.313507
RYR3	0.317038	0.082843	1.213291	0.093422
ZNF277	0.818307	0.378956	1.767029	0.609687
KTI12	1.552975	0.737	3.272362	0.247072
TOX	0.800543	0.476938	1.343717	0.399845
CES1	1.700072	0.896544	3.223765	0.104065
CD3E	1.158197	0.780167	1.719401	0.466292

RUSC2	2.346255	1.220423	4.510661	0.010549
FICD	0.561068	0.240909	1.306707	0.180314
OSTC	0.713456	0.327795	1.552859	0.394839
TSEN15	0.661975	0.28269	1.550147	0.34199
RUNDC1	1.986808	0.928857	4.249747	0.076773
CCDC152	0.152667	0.016758	1.390774	0.095447
GRK5	2.236137	1.121753	4.457584	0.022232
TYW1	0.382864	0.11929	1.228816	0.106605
SFMBT2	1.611636	0.801918	3.238947	0.180211
PNMA5	0.032918	0.001798	0.602629	0.021369
ITPRIPL1	0.500617	0.025448	9.848098	0.648966
SMC5	2.13563	0.415144	10.98634	0.363898
PRMT6	0.837647	0.424255	1.653848	0.609753
SHISA4	0.64624	0.11375	3.67143	0.622311
CAPZA2	1.126615	0.630532	2.013001	0.687256
TOP1	1.233454	0.329041	4.623762	0.755638
PRC1	0.602708	0.387488	0.937466	0.024674
MAP3K3	2.423101	0.925779	6.342133	0.071409
L1CAM	0.447577	0.019238	10.4128	0.616593
SREBF2	2.293154	0.335107	15.69219	0.397677
RASGEF1A	1.11146	0.175855	7.024768	0.910558
RPL39	1.89083	0.650582	5.495448	0.241906
DZIP3	0.101587	0.011861	0.8701	0.036893
KIAA0753	0.750521	0.264848	2.126812	0.589187
DCLRE1A	0.331376	0.149221	0.73589	0.006661
ATG9A	2.733101	1.192356	6.264773	0.017518
NOS1AP	0.149662	0.018097	1.23773	0.078052
APRT	1.751968	0.927141	3.310598	0.084172
GPRASP1	4.891577	0.775625	30.84934	0.091115
TBKBP1	1.037592	0.393909	2.733107	0.940472
MAGEE1	2.926524	0.510018	16.79262	0.228346
ZFP2	0.273431	0.054739	1.365831	0.11409
L3MBTL3	0.868647	0.137009	5.507297	0.881206
MFAP3L	0.490637	0.031933	7.538462	0.609477
NAGA	1.967755	1.300021	2.978459	0.001371
SMG5	0.645347	0.27319	1.524484	0.318
TGM2	1.119942	0.625102	2.006506	0.703393
ARMCX6	0.908941	0.41202	2.00518	0.813038
PJA2	0.707411	0.340805	1.468377	0.352904
SGMS1	2.710157	0.711038	10.32989	0.144174
OR10Z1	0.263597	0.015673	4.433238	0.354504
MIR30E	3.18006	0.110112	91.84092	0.500176
MIR429	1.627459	0.131741	20.10482	0.704169
MIR345	3.242972	0.552783	19.02529	0.192477
MIR16-2	1.254082	0.199942	7.865902	0.809033
MIR340	0.814974	0.050539	13.14202	0.885322
MIR339	5.277075	0.863836	32.23703	0.071633
MIRLET7C	2.42631	0.369687	15.92421	0.355824
MIR425	0.131735	0.025365	0.684179	0.015887
MIR210	0.233458	0.006992	7.795006	0.416371
MIR324	0.560964	0.026552	11.85158	0.710322
MIR101-2	2.638876	0.447149	15.57349	0.284017
MIR26A1	8.695245	0.680166	111.1601	0.096209
MIR342	0.218529	0.041412	1.15317	0.073127
MIR326	0.329581	0.059168	1.835858	0.205275
MIR302C	1.208689	0.807676	1.808807	0.356791
MIR26B	2.434983	0.28321	20.9355	0.417532
MIR148B	0.773456	0.040615	14.72944	0.864331

MIR101-1	0.837692	0.044224	15.86754	0.906058
MIR373	0.59477	0.099514	3.554792	0.568955
MIR302D	0.358802	0.033671	3.823432	0.395861
MIRLET7G	1.535834	0.102816	22.94174	0.755784
MIR30D	0.94688	0.079713	11.24765	0.965519
MIR126	1.361965	0.116966	15.85891	0.805176
MIR374A	0.593161	0.061216	5.747479	0.652173
MIR367	0.118664	0.006292	2.237882	0.154909
MIR331	0.813196	0.058546	11.29525	0.877583
MIRLET7I	0.791366	0.118948	5.264999	0.808775
SNORD11	0.436576	0.072231	2.638721	0.366576
SNORD9	0.969522	0.076941	12.21683	0.980898
SNORA63	0.301015	0.126442	0.716614	0.006669
SNORA31	0.493312	0.241711	1.006812	0.05222
SNORD10	0.788197	0.439677	1.412978	0.424187
SNORD1B	0.109417	0.009726	1.230899	0.073176
VTRNA1-1	0.726641	0.507781	1.039831	0.080749
SNORA73	0.319427	0.138215	0.738223	0.007584
SNORA70	0.632763	0.417321	0.959427	0.031162
SNORA72	0.376412	0.174741	0.810833	0.012577
SNORA38	4.516049	0.506477	40.26777	0.176835
SNORA33	0.455765	0.25019	0.830256	0.010233
RNU4-1	1.070786	0.855891	1.339637	0.549563
SNORA38	0.536787	0.05575	5.168413	0.590278
SNORD46	0.086281	0.020886	0.356436	0.000711
SNORA74	1.479073	0.387769	5.641652	0.56662
RNY1	1.249799	0.862658	1.81068	0.238442
SNORA62	0.24611	0.105951	0.571681	0.001113
SNORA65	0.422605	0.094325	1.893408	0.260309
RNU105B	3.791803	0.378045	38.03192	0.257197
SNORA16	0.745751	0.086833	6.40474	0.789174
SNORA14	3.845554	0.219084	67.50058	0.35686
SNORD11	0.778924	0.046475	13.05469	0.862095
SNORA5C	0.52378	0.072989	3.758722	0.520133
SNORD11	0.053723	0.00511	0.564788	0.014854
SNORD48	0.371261	0.166283	0.828915	0.015613
SNORD11	5.116043	0.467817	55.94901	0.181055
SNORA23	0.148942	0.013111	1.691923	0.124581
VTRNA1-2	0.423711	0.026517	6.770371	0.543642
SNORD11	1.522909	0.141864	16.3484	0.728339
SNORD6	0.656662	0.199936	2.156716	0.488185
RNY3	0.175997	0.064437	0.480706	0.000702
RNU4-2	1.10874	0.862427	1.425402	0.420642
MIR146B	32.72699	1.509752	709.4252	0.026255
MIR582	1.493853	0.170527	13.08648	0.716998
ZNF525	0.639098	0.155891	2.620078	0.533993
INF2	1.816473	0.955685	3.452574	0.068511
EFCAB2	0.539636	0.183549	1.586533	0.262239
CHML	0.33072	0.074157	1.474926	0.146912
TATDN3	1.338678	0.455223	3.936661	0.59611
CR1	1.06059	0.417733	2.692752	0.901519
ECT2L	0.1321	0.014868	1.17365	0.069326
GPR52	0.457416	0.012232	17.10569	0.672077
FCGR3A	1.092733	0.269203	4.435554	0.901263
OR6K3	0.131492	0.010776	1.604446	0.111937
MSTO2P	0.48698	0.081726	2.90177	0.429455
SPRN	1.433704	0.096752	21.24509	0.793383
FANK1	0.097607	0.013315	0.715533	0.022062

DDO	0.626026	0.19511	2.008649	0.431045
SNHG5	1.348223	0.886647	2.05009	0.162316
GDI1	2.071356	0.874329	4.907212	0.097969
PCMTD2	0.816781	0.449424	1.484413	0.506701
SOX18	1.446859	1.153918	1.814168	0.001373
LIME1	0.928514	0.700901	1.230042	0.605205
SAMD13	0.631588	0.253538	1.573349	0.323761
EFCAB7	0.870938	0.190868	3.974123	0.858395
LCN8	1.977635	0.758304	5.157617	0.163238
LIPN	1.369076	0.415164	4.514769	0.605858
FOXO6	2.604849	0.076585	88.59701	0.594686
SYS1	1.408258	0.558473	3.551095	0.468156
INPP5B	0.639215	0.251609	1.623932	0.346838
RPA4	0.397051	0.118319	1.332404	0.134819
NEU4	0.961274	0.736883	1.253996	0.7709
MAFB	1.1785	1.001183	1.387222	0.048362
TRAF3IP1	0.576852	0.045886	7.251839	0.670129
BMP2KL	2.883262	0.252152	32.96906	0.394345
CHIC1	0.329768	0.006532	16.64742	0.579275
GIGYF2	4.40207	0.579305	33.45081	0.152043
RUFY2	0.00631	0.000289	0.137907	0.001287
NHSL2	1.692164	0.400959	7.141428	0.473998
GGTA1	9.726456	1.033525	91.53525	0.046724
PHACTR4	1.174962	0.217504	6.347192	0.851386
AGAP6	0.787082	0.327489	1.891662	0.592547
TIMM23B	0.274995	0.033189	2.278553	0.231452
ZDHHC18	1.696032	0.888975	3.235775	0.108958
ZDBF2	0.609684	0.038329	9.697905	0.725941
GGNBP1	7.5515	0.467421	121.9996	0.154385
DAXX	0.84368	0.208946	3.4066	0.811335
BMPR2	1.257652	0.670881	2.35763	0.474607
TCEA3	1.109276	0.728494	1.689091	0.628808
PFDN6	1.013063	0.543052	1.889868	0.96746
RING1	0.681885	0.21077	2.206036	0.522704
RXRB	0.521636	0.191143	1.423563	0.203907
COL11A2	1.401867	0.026655	73.72783	0.867303
HLA-DOA	1.150417	0.842408	1.571043	0.378134
BRD2	0.681408	0.296338	1.56685	0.366566
HLA-DMA	1.117141	0.871628	1.431807	0.381649
COL5A2	0.129329	0.01192	1.403228	0.092672
PSMB8	1.229719	0.592423	2.552585	0.578927
TAP2	1.175912	0.592768	2.332731	0.642896
SPIN3	0.858369	0.307649	2.394927	0.7705
HLA-DRA	1.138335	0.910526	1.423142	0.255436
NOTCH4	0.816355	0.354706	1.878838	0.633293
PBX2	0.715916	0.268693	1.907515	0.503893
AGER	19.18355	1.131227	325.3182	0.040822
RNF5	2.159068	0.627695	7.426503	0.222043
AGPAT1	6.708089	0.932189	48.2718	0.058728
PRRT1	0.010618	0.000844	0.133555	0.000434
FKBPL	1.651159	0.47745	5.710188	0.428274
MRPL38	1.386608	0.737966	2.605379	0.309761
STK19	0.699337	0.263706	1.854611	0.472338
SKIV2L	0.504017	0.206901	1.227799	0.131501
ZBTB12	0.083852	0.008015	0.877262	0.038519
SDHD	0.758536	0.379408	1.516511	0.434288
EHMT2	1.064655	0.288632	3.927121	0.925048
LAYN	1.072039	0.141431	8.126014	0.946334

SLC44A4	0.059895	0.007259	0.494223	0.008936
NEU1	2.153845	1.170291	3.96401	0.013693
HSPA1B	0.995146	0.805544	1.229375	0.964014
HSPA1A	1.105164	0.836881	1.459452	0.480928
HSPA1L	1.171536	0.611849	2.243195	0.63288
LSM2	0.715381	0.355283	1.440458	0.348272
CARD16	1.733696	1.011518	2.971478	0.045325
MBD5	0.373089	0.011699	11.89841	0.576762
MSH5	0.184406	0.032768	1.037758	0.055121
LY6G6C	0.34708	0.047166	2.554053	0.298732
LY6G6F	1.159353	0.61418	2.188444	0.648284
LY6G5C	2.533221	0.411511	15.59424	0.316151
CSNK2B	0.918527	0.382427	2.206148	0.849236
APOM	0.623354	0.246396	1.577016	0.318259
AIF1	1.349028	0.788449	2.308173	0.274588
NCR3	0.983316	0.508948	1.899823	0.960067
LST1	1.791216	1.203117	2.666786	0.004097
NFKBIL1	1.070637	0.227378	5.041217	0.931196
MCCD1	0.86862	0.083915	8.991197	0.905971
MICB	0.781859	0.398046	1.535759	0.474967
ZNF551	0.561693	0.208845	1.510682	0.253178
MICA	1.162706	0.324606	4.164702	0.816868
ZNF805	0.750499	0.273205	2.061637	0.577741
HLA-C	1.244323	0.676595	2.28843	0.481941
PSORS1C3	1.316914	0.194102	8.934794	0.778093
POU5F1	0.026482	0.000249	2.812838	0.127134
CCHCR1	0.167743	0.031346	0.89765	0.036967
DHX16	0.643639	0.200933	2.061736	0.4582
MRPS18B	2.07363	0.785939	5.471086	0.140658
PPP1R10	1.989974	1.214419	3.260817	0.006315
ABCF1	0.801322	0.303043	2.118893	0.655275
PRR3	0.516074	0.192769	1.381614	0.187975
LILRB3	1.12493	0.784418	1.613256	0.522196
DDR1	70.73612	6.120748	817.4815	0.000647
GNL1	1.273882	0.50178	3.234038	0.610579
HLA-E	1.493533	0.971482	2.29612	0.067533
TRIM39	0.299949	0.047896	1.878443	0.198291
ZNF468	4.546698	0.594393	34.77914	0.14461
TRIM15	1.989958	1.153039	3.434344	0.013458
ZNF616	1.245961	0.419213	3.703174	0.692339
TRIM10	1.058243	0.679018	1.649262	0.802546
RNF39	1.01142	0.082713	12.36772	0.992908
PPP1R11	0.976796	0.457422	2.085889	0.951637
HCG9	0.607742	0.077659	4.756063	0.635205
HLA-G	1.623337	1.028703	2.561696	0.037384
TBC1D8	1.857172	1.084632	3.179963	0.024069
HLA-F	1.29275	0.90099	1.854851	0.163332
ZFP57	1.407486	0.273961	7.231008	0.682286
CBY3	0.280589	0.061503	1.280093	0.100778
IL31	5.906015	0.307882	113.2935	0.23866
AKT1S1	7.076565	1.784715	28.05925	0.005367
GABBR1	1.725296	0.881338	3.377416	0.11152
SPDYC	0.801202	0.091119	7.044883	0.841616
TRIM27	0.948413	0.382152	2.353742	0.909075
RANBP17	0.083498	0.004933	1.413219	0.085374
TTC25	0.752611	0.401684	1.410121	0.37499
ATXN2	0.43046	0.204315	0.906912	0.026626
DCTN1	1.896917	0.309122	11.64036	0.489156

TCTN1	0.718904	0.388758	1.32942	0.292722
ZBTB48	1.202132	0.563462	2.564719	0.63395
NAT8B	1.12829	0.700117	1.818324	0.620075
ZNF155	0.784498	0.03016	20.40544	0.88393
FBXO48	0.658397	0.030516	14.20543	0.789707
CD177	0.410835	0.12784	1.320281	0.135309
ZNF783	0.978876	0.404982	2.366026	0.962184
ZNF425	1.147383	0.404114	3.257711	0.796237
TRIM13	0.658423	0.188414	2.300894	0.512702
SPIRE2	0.276587	0.036035	2.12292	0.216457
PKHD1L1	1.899105	0.189518	19.0304	0.58544
SLFN12L	8.621698	0.695357	106.9	0.093521
SLC35B4	0.952414	0.412473	2.199155	0.909086
SYCE1L	2.768613	0.92968	8.245007	0.067399
TMEM231	3.953186	0.984151	15.87935	0.052691
FAM71F2	0.247878	0.018965	3.23982	0.287513
CCNI2	1.23486	0.279239	5.460844	0.780918
CDKL4	0.222272	0.022047	2.240884	0.202106
SDHAF1	1.895642	1.027728	3.496508	0.040606
PSENEN	1.886683	0.75185	4.734418	0.176259
ZBTB10	0.135177	0.029516	0.619076	0.009948
CCDC144N	13.96535	0.776879	251.0441	0.073666
LGR4	0.615535	0.098374	3.851456	0.603988
PSMB10	1.261817	0.573442	2.776535	0.563304
E2F4	0.339985	0.126	0.917381	0.033152
PDE7A	0.516984	0.228559	1.16938	0.113142
TMEM170I	1.049785	0.778281	1.416003	0.750325
SNX2	1.179116	0.665505	2.08911	0.57235
NT5M	0.680561	0.425402	1.088767	0.108445
IPO7	0.50198	0.212259	1.187151	0.116572
PRR13	1.224829	0.366306	4.095497	0.741935
TECPR1	1.461792	0.85942	2.486372	0.161231
MT1H	2.237092	1.325223	3.776406	0.002578
MT1M	1.261048	0.670524	2.371644	0.4717
SAMD9	1.725321	0.954936	3.117207	0.070735
ATP6AP1L	0.241784	0.061769	0.946424	0.041445
C2CD4B	0.891599	0.654574	1.214452	0.466794
NAP1L4	0.79359	0.364825	1.726269	0.559858
TMSB4X	1.323191	0.695449	2.517559	0.393493
CPT1B	1.04225	0.364786	2.977871	0.938419
SMN2	0.429131	0.105531	1.745007	0.237188
SERF1B	0.664074	0.211533	2.084748	0.483094
HMGN1	0.226021	0.099345	0.514226	0.000392
STAG3L1	0.374122	0.148731	0.941079	0.036708
MUC19	0.180144	0.011671	2.780467	0.21961
EIF3CL	0.695	0.375396	1.286707	0.246948
LCMT1	0.395321	0.167749	0.931622	0.033846
MFSD2B	0.14732	0.029142	0.744732	0.020534
LIN52	0.292874	0.099588	0.861302	0.025664
DPF3	0.701396	0.084798	5.801541	0.742137
ITSN1	1.449025	0.352166	5.962174	0.607323
ITPRIPL2	1.406339	0.96474	2.050074	0.076181
DENND1C	0.924122	0.449211	1.901115	0.83023
CRLF2	0.372088	0.056919	2.43241	0.302052
CRYZL1	0.245335	0.092628	0.649796	0.004692
RP9P	1.284564	0.473522	3.484744	0.622855
CATSPER2	0.110632	0.010465	1.16961	0.067287
ARRDC5	1.435327	0.707198	2.913137	0.316983

LOH12CR2	0.330959	0.033431	3.276429	0.344475
KLRC2	0.578895	0.147081	2.278472	0.434243
KLRC3	1.083144	0.26671	4.398796	0.911064
RFPL3S	0.065479	0.003179	1.348779	0.077376
OLIG2	1.208344	0.908954	1.606347	0.19264
RNPS1	1.35921	0.483266	3.822842	0.560776
HSP90AB2	0.410366	0.008044	20.93488	0.657063
NYNRIN	1.471805	1.088912	1.989334	0.011936
DNAJC19	0.290813	0.104474	0.809504	0.018051
IFITM5	0.252429	0.011882	5.362575	0.377302
DEFA1	1.176106	0.924687	1.495886	0.186208
DOK6	6.57644	1.238512	34.92059	0.02703
ZDHHC11I	1.960909	0.157586	24.40036	0.600623
TMEM191(	13.41992	1.477657	121.8782	0.021064
HBA1	1.140823	0.814604	1.59768	0.443264
HBM	1.080407	0.928633	1.256987	0.31667
ATP10A	0.452146	0.085576	2.388954	0.350001
HCP5	1.431412	1.045502	1.959766	0.025249
HLA-H	1.451929	0.966226	2.181786	0.072716
HCG27	0.796551	0.325588	1.948765	0.618263
COL6A6	1.87753	0.542391	6.499214	0.320059
RAB12	0.578783	0.158833	2.109075	0.407192
HLA-A	2.60774	0.735235	9.249163	0.137856
LNP1	0.357068	0.050732	2.513157	0.300966
VGLL3	0.470678	0.02855	7.759697	0.598179
TRIM71	2.425479	0.835421	7.041895	0.10325
ANKRD28	0.8911	0.547339	1.450761	0.642892
COLQ	0.289706	0.03369	2.4912	0.259102
METTL6	0.458455	0.168629	1.246408	0.126433
SNORA2A	0.161472	0.01098	2.374663	0.18371
SNORA69	1.296879	0.103805	16.20237	0.840095
RNU6-1	1.281145	0.751714	2.183453	0.362407
SNORA22	0.176325	0.023753	1.308907	0.08974
SNORD10:	0.158467	0.023426	1.071953	0.058928
SNORA30	1.550893	0.156226	15.39609	0.707867
SNORD37	1.238063	0.240427	6.375327	0.798426
SNORA5A	0.511399	0.03338	7.834879	0.630094
SNORA75	0.232462	0.095143	0.567976	0.001369
SNORA70I	0.878062	0.153825	5.012146	0.883671
SNORD15/	0.916383	0.04121	20.37742	0.955998
SNORD63	1.138087	0.061918	20.91878	0.930607
SNORA54	0.577219	0.086076	3.870771	0.571403
SNORA36(	1.675535	0.127189	22.07282	0.694788
SNORD59/	0.140771	0.028938	0.684799	0.015138
SNORD51	0.171611	0.045294	0.650209	0.009505
SNORA7B	0.945533	0.523646	1.707322	0.852634
SNORA15	0.16871	0.016008	1.778088	0.138609
SNORA14I	0.166063	0.019418	1.420164	0.101083
SNORA37	0.261296	0.025409	2.687104	0.259031
SNORA2B	0.199147	0.03455	1.147883	0.070974
SNORA20	0.211607	0.042775	1.046823	0.056928
SNORD56I	8.974465	1.050988	76.63366	0.044919
SNORD15I	0.170983	0.059388	0.492271	0.001062
SNORA19	3.089488	0.207121	46.08397	0.413307
SNORA46	0.019892	0.000951	0.416081	0.011563
RNU1-3	0.95462	0.743802	1.225191	0.715284
SNORA66	0.099765	0.022608	0.440234	0.002341
MIR25	0.354987	0.142844	0.882191	0.025758

MIR647	5.980945	0.351905	101.6517	0.215935
MIR23B	1.647221	0.136567	19.86819	0.694433
MIR199B	0.365272	0.039774	3.354568	0.373372
MIR30B	0.255093	0.021144	3.077642	0.282285
MIR606	0.505892	0.035553	7.198402	0.614972
MIR593	0.183367	0.025731	1.306752	0.090466
MIR215	2.202657	0.27279	17.7855	0.458701
MIR181A2	5.907651	0.516644	67.55196	0.153074
MIR191	0.417927	0.019065	9.16157	0.579687
MIR554	0.365845	0.01584	8.449466	0.530185
MIR200A	0.173996	0.011022	2.746662	0.214154
MIR491	2.631547	0.398234	17.38936	0.315234
MIR149	1.75457	0.356187	8.642993	0.489517
MIR604	0.530864	0.112426	2.506692	0.423944
MIR181C	0.529658	0.082004	3.421017	0.504313
MIR193A	0.743919	0.041958	13.18962	0.840188
MIR194-1	0.089342	0.004761	1.676569	0.106412
MIR128-2	1.286992	0.072867	22.73109	0.863265
MIR562	10.3552	0.863989	124.1106	0.065096
MIR581	1.620229	0.145393	18.05551	0.694829
MIR651	7.868134	0.412524	150.0701	0.170273
MIR641	0.502215	0.019111	13.19765	0.679633
MIR505	0.29609	0.030635	2.861758	0.293009
MIR99A	3.041208	0.198684	46.55097	0.424275
MIR571	0.159931	0.039031	0.655313	0.010856
MIR570	0.051758	0.002608	1.027376	0.052107
MIR558	1.556866	0.079238	30.58924	0.770785
MIR128-1	0.348039	0.016482	7.349345	0.497628
MIR602	1.749721	0.170271	17.98027	0.637895
MIR184	0.929975	0.073035	11.84162	0.9554
MIR659	0.131801	0.00949	1.830512	0.131151
MIR573	1.285014	0.145485	11.35003	0.821498
MIR32	0.225978	0.016682	3.061218	0.263332
MIR597	10.50404	0.550084	200.5781	0.118102
MIR141	102.1489	4.103886	2542.568	0.00479
MIR627	0.932563	0.07032	12.36732	0.95778
MIR200C	3.655894	0.182778	73.12451	0.396378
MIR572	0.876487	0.118271	6.495477	0.897354
MIR623	2.554997	0.345127	18.91479	0.358408
MIR186	7.760745	0.827009	72.82774	0.072862
MIR222	2.902625	0.24155	34.87992	0.40089
MIR556	8.861244	0.561844	139.757	0.121073
MIR200B	1.237857	0.074542	20.55616	0.881676
MIR657	0.13053	0.021008	0.811023	0.028911
MIR181B2	4.02623	0.32443	49.96622	0.278396
MIR590	0.801664	0.099712	6.445188	0.835332
MIR575	2.581153	0.103296	64.4975	0.563625
MIR553	0.218469	0.010139	4.707471	0.331532
MIR199A1	2.380691	0.281585	20.12784	0.425808
MIR580	1.847071	0.225405	15.13575	0.567496
MIR93	0.354946	0.031551	3.993094	0.401601
MIR181A1	0.364007	0.037645	3.519791	0.382692
MIR617	0.353679	0.01882	6.646603	0.487407
MIR626	0.176241	0.012044	2.578927	0.204809
MIR586	0.499777	0.195883	1.275135	0.146677
MIR551A	4.36447	0.340907	55.87625	0.257337
MIR15B	7.61554	0.574839	100.8916	0.123564
MIR648	2.61466	0.177556	38.50297	0.48368

MIR26A2	6.045213	0.446571	81.83375	0.175889
MIR646	0.612945	0.059682	6.295077	0.68043
MIR599	0.292266	0.109887	0.77734	0.013716
MIR563	0.466986	0.025799	8.452709	0.606308
MIR545	5.32535	0.438453	64.68047	0.189256
MIR640	1.334452	0.095934	18.56245	0.829922
MIR596	5.314206	0.213893	132.0322	0.308175
MIR33B	9.21071	1.151008	73.70682	0.036393
MIR27B	1.234743	0.063513	24.00453	0.889232
MIR221	0.648703	0.385498	1.091614	0.103134
MIR610	3.025398	0.642255	14.2514	0.161508
MIR302A	1.252253	0.063675	24.6272	0.882342
MIR603	0.347651	0.019477	6.20541	0.472425
MIR33A	0.380392	0.019673	7.355041	0.522443
MIR634	0.532864	0.027535	10.31208	0.677102
MIR574	1.282172	0.743949	2.20978	0.37081
MIR624	1.497313	0.109766	20.42483	0.762059
MIR579	7.683852	0.935856	63.0883	0.057663
MIR30C1	2.30043	0.201921	26.20815	0.502139
MIR569	3.531382	0.227552	54.80345	0.367149
MIR629	0.19377	0.013172	2.850598	0.231568
MIR620	8.507737	1.06562	67.92441	0.043391
MIR638	0.48434	0.077652	3.020978	0.437618
MIR589	1.769618	0.117188	26.72251	0.680285
MIR181B1	0.085339	0.017163	0.424344	0.002634
MIR607	1.191699	0.341008	4.164561	0.783531
MIR613	0.340694	0.014711	7.890365	0.501841
MIR576	0.177741	0.010582	2.985446	0.2301
MIR182	1.046753	0.288701	3.79525	0.944569
MIR601	6.148748	0.510435	74.06835	0.152615
MIR301A	0.123437	0.006698	2.274662	0.159377
MIR643	0.010728	0.000279	0.412904	0.014896
MIR16-1	0.396368	0.024867	6.317941	0.51242
MIR130A	1.230443	0.794702	1.905104	0.352508
MIRLET7F2	1.452275	0.099333	21.23254	0.785132
MIR652	1.618181	0.059055	44.33998	0.775686
MIR140	19.05098	0.557768	650.7002	0.101861
MIR645	0.396657	0.050621	3.108134	0.378679
MIR618	1.312094	0.108354	15.88859	0.830965
MIR185	0.423216	0.073915	2.423206	0.334135
MIR591	2.077775	0.284085	15.19666	0.471316
MIR616	2.289914	0.436152	12.02267	0.32746
MIR548A3	12.71281	0.682831	236.6846	0.088335
MIR106B	0.075373	0.006175	0.920061	0.042844
MIR320A	6.916612	1.10911	43.13326	0.038372
SNORD94	0.096976	0.009954	0.944797	0.044553
SNORD96I	0.163391	0.029626	0.901123	0.037574
SNORA49	0.088759	0.007451	1.057348	0.055384
SNORD12(	0.692624	0.411662	1.165346	0.166502
SNORD83/	0.054924	0.005586	0.540036	0.012836
SNORD10!	0.269236	0.014343	5.053969	0.380461
SNORD41	0.576364	0.078195	4.248307	0.588748
MIR196A1	0.609586	0.04392	8.460636	0.712265
GPX3	1.509605	0.842561	2.704737	0.166297
GNRHR2	0.187647	0.017148	2.053336	0.170497
DIO1	17.17115	0.728837	404.5464	0.077774
AKR7L	0.314987	0.015764	6.293851	0.449623
STK38L	0.476504	0.144419	1.572201	0.223579

SACM1L	1.02108	0.543282	1.919085	0.948334
TSN	0.721574	0.386318	1.347774	0.305982
MIR454	1.724726	0.10776	27.60472	0.700039
MIR760	0.618565	0.056498	6.772367	0.694028
MIR766	0.918718	0.048804	17.29464	0.954856
MIR769	0.912572	0.057476	14.48928	0.94829
MIR765	3.326221	0.215795	51.26978	0.389139
SLC48A1	1.420521	0.728171	2.771163	0.303218
MIR301B	3.8568	0.40484	36.74266	0.240516
PRR22	0.354005	0.106751	1.173941	0.08955
TAS2R19	0.159632	0.010651	2.39258	0.184047
TAS2R50	0.089013	0.007205	1.099689	0.059311
TAS2R14	0.134188	0.014275	1.261385	0.078944
TAS2R13	1.379969	0.10293	18.501	0.807869
SNORD67	3.741431	0.270659	51.71931	0.324785
SNORD66	1.699932	0.112908	25.59394	0.701357
SNORD91/	0.979015	0.280465	3.417431	0.973474
SNORD17	0.184296	0.028573	1.188712	0.075371
SNORD89	0.775918	0.412498	1.459517	0.431261
SNORD72	0.413665	0.029558	5.789277	0.512052
SNORA74I	0.346477	0.033376	3.59678	0.374646
SNORA53	0.246152	0.025549	2.371569	0.225195
SNORD90	0.843363	0.065875	10.79715	0.895809
SNORD69	0.164287	0.038453	0.701897	0.014781
SNORA12	1.081391	0.780718	1.497862	0.637822
SNORD19	0.678651	0.048825	9.432993	0.772823
SNORD70	2.004947	0.196306	20.47733	0.557385
SNORD30	0.1808	0.056955	0.573941	0.003707
RNF208	0.887059	0.044902	17.52411	0.937246
PTTG3P	0.724362	0.468724	1.119422	0.146504
ZNF580	5.179938	1.140374	23.5289	0.033165
ZNF611	2.118917	0.345284	13.00325	0.417253
NUP62	1.320449	0.571751	3.049558	0.515114
DENND1B	0.095653	0.007847	1.165996	0.065828
SFT2D2	0.89946	0.210945	3.835251	0.886126
LPAL2	0.04901	0.000215	11.19008	0.276427
ZNF254	0.941544	0.223091	3.973741	0.934657
TCTEX1D2	0.850464	0.432839	1.671036	0.638339
CRYGS	0.556012	0.235832	1.310889	0.179809
CRIP1	1.487061	1.115168	1.982976	0.006886
KLHL23	0.502225	0.157427	1.602207	0.2446
FAM24B	1.071959	0.601268	1.911123	0.813785
TRIM59	0.342128	0.031233	3.747704	0.379826
MLLT11	0.836914	0.644791	1.086281	0.180899
GIMAP1	1.708248	1.197108	2.437633	0.00316
DNLZ	1.100958	0.655131	1.850179	0.716494
SUPT4H1	0.734061	0.268381	2.007761	0.547024
NRAS	0.450037	0.150297	1.347559	0.153616
LTC4S	0.63633	0.375626	1.077978	0.092807
ANKRD39	0.68757	0.226742	2.084977	0.508089
QTRT1	1.157751	0.596184	2.248279	0.665323
CHUK	0.382243	0.152812	0.95614	0.039798
MXD3	1.199512	0.800858	1.796609	0.377468
GSTM2	0.669425	0.442323	1.013129	0.057661
COG8	2.481869	0.875084	7.038957	0.087435
ARHGAP1!	0.679042	0.347868	1.325499	0.256695
LCAT	0.582172	0.22747	1.489975	0.259192
PVRIG	1.09849	0.765164	1.577022	0.610641

GPC2	0.47441	0.225662	0.997354	0.049189
SIPA1	1.707878	0.807424	3.612538	0.161413
SYNJ2BP	0.764308	0.375698	1.554885	0.458216
ARL2	0.690246	0.427488	1.114511	0.129402
SRA1	0.909315	0.114628	7.213391	0.928314
DNAJC9	0.422957	0.261362	0.684463	0.000459
VDAC1	0.79141	0.307259	2.038448	0.627947
ZBTB9	1.614591	0.943052	2.764327	0.080768
HEXA	0.158412	0.047363	0.529828	0.00278
NDUFS3	0.915003	0.498414	1.679787	0.774429
LEPROT	0.647859	0.341412	1.22937	0.184132
LBH	1.614228	0.447704	5.820215	0.46428
ADAT3	2.333232	1.137892	4.784262	0.020747
PPP1CB	0.730127	0.312611	1.705268	0.467373
GPSM3	1.488024	0.939621	2.356499	0.09018
LAT	0.87333	0.361843	2.10784	0.7632
NCKIPSD	1.074833	0.233738	4.942569	0.926139
ATF6B	1.99874	0.795977	5.018942	0.140425
S1PR3	1.656145	1.000494	2.741462	0.049776
CLIC1	1.332006	0.576743	3.076307	0.50204
DDAH2	1.20223	0.721626	2.002919	0.479431
RPS29	1.294338	0.324248	5.166756	0.714885
UGT2B11	0.733107	0.110889	4.846707	0.747326
ATP6V1G2	1.061516	0.191401	5.887183	0.945546
ZNF134	0.347516	0.128105	0.942722	0.037912
DDX47	0.613645	0.265999	1.415647	0.252213
ZNF845	0.918311	0.364659	2.312556	0.856487
KLRK1	6.111023	0.537662	69.45746	0.144402
EMP2	0.57993	0.073291	4.588796	0.605668
KCTD11	1.337298	0.390921	4.574748	0.643235
LTB4R	1.103754	0.702442	1.734341	0.668546
LTB4R2	1.149837	0.59142	2.235511	0.680634
DNASE1	0.31806	0.047169	2.144679	0.239434
CSNK1E	1.333773	0.597119	2.979219	0.482423
IRF9	2.58618	1.429039	4.680298	0.001692
GALT	0.248888	0.049519	1.250937	0.091375
HBG1	0.941578	0.824237	1.075624	0.375372
CLDN9	0.14254	0.018028	1.126969	0.064797
KRT18P17	3.42293	0.991224	11.82019	0.051652
NUDT19	0.322105	0.059677	1.738548	0.187832
TAX1BP3	1.459815	0.760644	2.801652	0.255369
AP1G2	0.703986	0.168338	2.944055	0.630649
ZNF90	3.028111	0.185695	49.37915	0.43664
MEF2B	0.570187	0.070307	4.624218	0.598849
GANC	0.259738	0.026225	2.57249	0.249194
TTLL3	0.347029	0.068176	1.766446	0.202419
REPIN1	0.07004	0.01502	0.326608	0.000713
MRPL23	1.596306	0.752388	3.386807	0.222979
UCA1	0.243241	0.080453	0.735414	0.012266
TSPAN4	1.168948	0.621826	2.197463	0.627871
CPNE1	1.176479	0.359332	3.851883	0.788253
ARL16	0.729148	0.329204	1.614973	0.436237
LYRM4	0.86835	0.409377	1.8419	0.712923
MYCBP	0.448916	0.153707	1.3111	0.143021
PRCD	0.167131	0.009623	2.902666	0.219332
ALG3	0.741467	0.353864	1.55363	0.428033
FIS1	1.625152	1.010692	2.613178	0.045086
MBLAC1	5.982116	0.865414	41.35095	0.069766

NEURL1B	1.89016	1.220494	2.927262	0.004334
PLIN5	1.322941	0.714162	2.450667	0.37362
HIGD1C	1.294344	0.091556	18.29847	0.848598
PPME1	1.036002	0.429966	2.496241	0.93717
STARD10	1.357111	0.725602	2.538237	0.33913
MEG3	1.508183	0.953933	2.384459	0.078719
IFRD2	0.743741	0.392882	1.407928	0.363214
CAPN14	0.483796	0.11365	2.05947	0.32588
ZBED1	2.470356	1.150528	5.304228	0.020361
HNRNPUL	0.759492	0.34845	1.655413	0.48892
POLR2J4	0.481361	0.006386	36.28122	0.740247
MTCP1	1.333195	0.615956	2.885611	0.465416
SMTNL1	1.234022	0.113215	13.45056	0.863018
ZSWIM7	0.640081	0.382492	1.071146	0.089443
LRRC69	0.465473	0.03587	6.040242	0.558719
PHB2	1.006821	0.496457	2.041842	0.984967
NEURL4	1.023598	0.37488	2.794904	0.9637
UBXN2B	0.913032	0.461345	1.806952	0.793911
CBWD6	0.486166	0.023892	9.892621	0.638965
FAM166B	1.281373	0.131546	12.48168	0.830957
PEX26	0.517042	0.082274	3.2493	0.481822
FASTKD5	0.73415	0.434515	1.24041	0.248143
GOLGA8B	0.756349	0.459248	1.245654	0.272627
HOMEZ	3.617065	0.288985	45.27279	0.318689
DDX3X	0.591972	0.273114	1.28309	0.184048
VPS16	0.596025	0.114559	3.100975	0.538564
MYL5	0.769548	0.388299	1.525125	0.452908
ZNF407	0.50267	0.044152	5.72287	0.579406
NPEPL1	1.375768	0.822378	2.301541	0.224326
SIAH3	5.155774	0.171642	154.8686	0.344772
GCGR	0.685954	0.301734	1.559431	0.368342
CELA2B	0.078292	0.007823	0.78352	0.030193
TMEM167I	0.541257	0.279209	1.049246	0.069121
FAM72D	0.592004	0.267578	1.30978	0.195696
TNFRSF25	1.353182	0.881929	2.076246	0.166134
ZC3H11B	0.243005	0.085867	0.687708	0.007691
TSTD1	0.931535	0.389012	2.230669	0.873523
PDZK1P1	0.130667	0.014352	1.189678	0.070942
CYB5RL	0.108515	0.015785	0.745968	0.02395
TTC34	0.49969	0.018908	13.20571	0.677946
MMP23A	2.659113	0.289015	24.46547	0.387741
ATAD3C	2.181322	0.248183	19.17204	0.481869
MIR942	1.780179	0.11782	26.89734	0.677203
MIR933	0.329668	0.024952	4.355598	0.399441
MIR874	1.090374	0.083785	14.19005	0.947309
MIR938	1.417957	0.119618	16.80851	0.781928
MIR875	0.182028	0.016451	2.014121	0.164816
MIR920	1.572464	0.110516	22.37363	0.73829
IFI30	1.25673	1.030092	1.533232	0.024311
CCDC7	0.048848	0.00106	2.250998	0.122395
FNIP1	0.162028	0.042604	0.616209	0.007577
CKLF	0.624826	0.303878	1.28475	0.201008
RPS10P3	1.270526	0.815453	1.979557	0.289938
ZNF579	2.14851	0.957254	4.822229	0.063731
RNASEK	2.076416	0.932073	4.625714	0.073803
NBPF1	0.378884	0.01244	11.54002	0.57767
LINGO3	11.58718	0.970447	138.3516	0.052836
VAMP2	1.408772	0.937225	2.11757	0.099316

MIR1250	0.306046	0.018951	4.942338	0.404166
MIR1272	0.293578	0.014865	5.797954	0.420675
MIR1286	0.668065	0.032428	13.76316	0.793845
MIR1296	1.693155	0.126008	22.75071	0.69117
SNORD11	4.866019	0.298549	79.31085	0.266523
MIR1302-!	0.957622	0.038364	23.90375	0.978955
SNORA11I	3.017107	0.326432	27.88613	0.33042
MIR548E	3.415445	0.171211	68.13378	0.421217
MIR548L	5.232799	0.60929	44.94112	0.131457
MIR548P	4.445289	0.350325	56.40651	0.249802
MIR1284	1.089721	0.051059	23.25719	0.956122
MIR1255A	0.034855	0.002197	0.552966	0.01731
MIR1302-8	2.851756	0.289348	28.10635	0.369364
MIR548I2	1.635566	0.146368	18.27637	0.689513
MIR663B	0.972251	0.12944	7.302808	0.978178
SNORA79	0.520607	0.093303	2.904859	0.456756
MIR1200	0.51789	0.031862	8.417891	0.643715
MIR548Q	1.47232	0.172934	12.53502	0.723327
MIR548K	0.788456	0.038567	16.11884	0.877317
MIR1288	0.887364	0.066591	11.82458	0.927936
MIR320B2	1.038696	0.049895	21.623	0.980444
MIR1301	1.160159	0.088456	15.21621	0.90993
MIR1202	1.813867	0.157561	20.88145	0.632902
MIR1277	0.587241	0.072313	4.768861	0.618382
MIR1271	0.437289	0.047052	4.06407	0.467097
MIR1827	21.16176	0.983564	455.3032	0.05125
MIR320C1	3.248461	0.181636	58.09708	0.4233
SNORD10(	0.476899	0.223025	1.019762	0.056198
MIR548O	0.635237	0.034115	11.82844	0.761031
SNORD11	1.947527	0.278038	13.6415	0.502124
MIR548H1	1.40371	0.144001	13.68328	0.770367
SNORD99	0.312102	0.161258	0.604051	0.000548
MIR1180	1.432864	0.09092	22.58137	0.798219
MIR1255B	0.747698	0.036784	15.19805	0.849932
MIR1249	2.182302	0.140815	33.82057	0.576791
MIR1276	0.944526	0.077217	11.5536	0.96437
SNORA77	0.340686	0.066515	1.744965	0.196366
MIR1290	0.919728	0.057489	14.71402	0.952829
MIR548N	0.115236	0.003962	3.35144	0.208889
RNU6ATA(	0.983445	0.457614	2.113491	0.965886
MIR1278	1.093672	0.06892	17.35515	0.94938
SNORA11	1.524108	0.189848	12.23563	0.691715
MIR1203	1.192312	0.043731	32.50798	0.916938
SNORD93	0.530378	0.026366	10.66921	0.678799
MIR548J	0.419807	0.016785	10.49985	0.597204
MIR1289-:	0.358289	0.032367	3.96608	0.402727
MIR1205	0.095901	0.003833	2.399212	0.153519
MIR548F2	0.732189	0.061056	8.78047	0.805735
MIR1183	10.46676	0.453232	241.7152	0.142665
MIR1256	0.877423	0.128921	5.971657	0.893686
PPP3R1	0.713173	0.425	1.196744	0.200575
FANCG	0.388796	0.204152	0.740441	0.00405
AP4M1	1.332775	0.451744	3.932068	0.602783
TAS2R41	0.534949	0.048818	5.861952	0.608546
PLXNA4	3.350411	0.181107	61.98137	0.416684
CEBPD	1.26311	0.980076	1.627881	0.071155
OR3A2	0.662933	0.067166	6.543175	0.724904
NPTXR	1.11434	0.105962	11.71887	0.928143

PPP2R2A	0.963781	0.52773	1.760131	0.904442
TRIM16	5.680896	1.880433	17.16231	0.002074
TIGD1	0.115587	0.013251	1.008212	0.050871
FXYD7	0.720656	0.131881	3.937979	0.705375
SLC12A8	2.419539	0.342746	17.08022	0.375554
KIR2DS4	0.236659	0.027786	2.015693	0.187302
APOL6	0.568115	0.089798	3.594249	0.548013
FADS3	0.750513	0.159339	3.535038	0.716625
CCNL2	1.744173	0.230454	13.20065	0.590104
UBA52	1.119859	0.336167	3.730535	0.853715
MYBPHL	11.09018	0.898863	136.8308	0.060546
PPT2	0.279317	0.067473	1.156282	0.078472
ZNF630	0.352694	0.075385	1.650097	0.185574
TIAF1	1.2296	0.399767	3.781993	0.718434
MIR1915	2.813019	0.446775	17.71153	0.270586
SNORD12I	0.180671	0.046747	0.698266	0.013114
SNORA36I	3.349534	0.190361	58.93735	0.408693
MIR1537	5.755518	0.347915	95.21287	0.221523
MIR1913	0.058675	0.002431	1.416354	0.080866
SNORD71	0.642071	0.118819	3.469604	0.606752
EXOSC6	0.717869	0.340142	1.515062	0.384417
VPS52	1.638899	0.61411	4.373794	0.32393
RPL23AP5	0.433665	0.165294	1.137766	0.089564
CDRT15	4.266569	0.359195	50.67888	0.250538
ZNF844	1.475365	0.646463	3.367094	0.355609
HBD	1.02288	0.844478	1.238971	0.817046
RFPL4A	1.217175	0.314695	4.707782	0.775824
HLA-DPB1	1.086095	0.816194	1.445249	0.570991
OR2L1P	4.011328	0.057916	277.8274	0.52058
ATXN1L	1.167715	0.176492	7.725877	0.872229
HLA-DPB2	1.26809	0.108214	14.85996	0.84998
TMEM183I	0.414222	0.105972	1.619097	0.205098
SRP14P1	0.571109	0.230877	1.412723	0.225418
INE1	7.9701	1.007161	63.07085	0.049215
BRI3P1	1.43509	0.98272	2.095697	0.06152
PLEKHM1	1.98676	1.082907	3.645018	0.026609
HSPA7	1.114791	0.826174	1.504235	0.477175
ZNF469	0.593214	0.100234	3.510806	0.564866
SLC26A6	2.320828	1.0543	5.108837	0.0365
DHRS4L1	0.114207	0.006271	2.080076	0.14283
MIAT	1.048774	0.558223	1.970407	0.882335
ERCC6	3.65743	0.446183	29.98052	0.227005
NOL7	0.644209	0.306212	1.355289	0.246535
GTF2H2B	1.101499	0.547876	2.214552	0.786156
TMEM191	0.946655	0.577348	1.552193	0.827985
NPY6R	0.032616	0.002888	0.368334	0.005649
TMEM185I	2.314886	1.062363	5.04413	0.034669
SMCR5	0.863068	0.446937	1.666646	0.660956
TAS2R46	2.008219	0.205979	19.57935	0.548435
SRRM5	0.437123	0.039302	4.861776	0.500755
FAM30A	1.342485	1.001008	1.800452	0.049219
PGAM4	1.353423	0.711514	2.574446	0.356273
HNRNPA3	0.557485	0.202556	1.534337	0.257972
LTA	1.325679	0.602156	2.918556	0.48381
WDR46	0.98859	0.426679	2.290503	0.978645
ZNF717	0.365552	0.073609	1.815386	0.218428
PARG	0.644052	0.021469	19.32073	0.799849
SCAMP4	2.305432	0.662542	8.022156	0.18922

LTB	1.106816	0.824444	1.485898	0.499457
POLR2J2	1.112357	0.635971	1.94559	0.708933
PPIAP19	11.32393	0.194545	659.1348	0.241825
ORM2	0.99334	0.596431	1.65438	0.979516
LYPLA2P1	0.971723	0.158792	5.946418	0.975241
CCT6P1	0.153123	0.025612	0.91547	0.039709
ARL17B	0.171731	0.022682	1.300203	0.088047
DHFR	0.521598	0.220728	1.232578	0.137973
RPL41	1.833497	0.262063	12.82788	0.541355
CCDC26	1.328594	0.534251	3.303992	0.541025
TTTY10	0.458463	0.082351	2.552346	0.373311
ORM1	0.99115	0.799795	1.228287	0.935264
HLA-DRB6	1.135084	0.929183	1.386612	0.214704
SPINK8	1.965263	0.538194	7.176335	0.306581
PATL2	0.578267	0.197267	1.695127	0.318196
ZNF492	0.576143	0.209748	1.582571	0.284824
XIST	0.933687	0.715917	1.217698	0.612594
ZNF688	2.975387	1.085702	8.154101	0.034021
EMX2OS	0.86022	0.261397	2.830855	0.804328
HBBP1	0.980546	0.715138	1.344456	0.902907
ACBD6	0.2754	0.113068	0.670794	0.004525
HERC2P4	0.050694	0.004417	0.581762	0.016618
DSCR9	12.92561	1.127685	148.1544	0.039732
ANKRD18I	0.596292	0.019398	18.32959	0.767366
PA2G4P4	0.131278	0.017744	0.97123	0.046751
PRB4	0.133565	0.01019	1.75078	0.125183
YY2	0.036905	0.002945	0.462439	0.010532
HSBP1	0.680511	0.343329	1.348841	0.270162
HCG18	0.692327	0.156034	3.071877	0.628615
HLA-DPA1	1.118449	0.925012	1.352337	0.247919
RPS18	0.564398	0.153349	2.077259	0.38959
SNORD62I	1.748602	0.596356	5.127155	0.308606
DLEU2	1.205031	0.421459	3.445414	0.727871
PRH1	0.282492	0.034554	2.309507	0.238331
TAPBP	1.176904	0.366221	3.78215	0.78449
ANXA2P2	0.079999	0.008348	0.766651	0.028495
MCTS1	0.739598	0.412651	1.325588	0.310953
DYTN	1.301674	0.088986	19.04061	0.847268
HLA-DQB1	1.491928	0.135723	16.39991	0.743594
TNF	1.0415	0.856324	1.26672	0.683938
SNHG7	1.293756	0.743598	2.250955	0.362036
RAB1C	0.318754	0.0261	3.892897	0.370537
GPX1	2.569652	1.53298	4.307368	0.000342
KRT8P15	1.815947	0.103715	31.79531	0.682929
MYCNOS	1.112545	0.579267	2.136762	0.748753
RPS28	5.430501	1.170161	25.20195	0.030724
UQCRHL	0.315607	0.094109	1.058435	0.061765
NUDT9P1	0.093115	0.009568	0.906203	0.040874
TRIM26	0.978217	0.456848	2.09459	0.95479
FAM133B	0.702043	0.286774	1.718647	0.438668
JRK	0.144088	0.017898	1.159987	0.068682
GAS5	0.118051	0.013913	1.001641	0.050176
HLA-B	1.267068	0.868592	1.848349	0.219191
MORF4	2.244088	0.15347	32.81367	0.554807
MIR155HG	1.287217	0.882554	1.877423	0.189803
APOC2	0.638285	0.450639	0.904068	0.011479
SUMO1P3	0.945548	0.281762	3.17311	0.927777
PPP1R3E	0.112394	0.030228	0.417909	0.001106

SNORA71I	0.492787	0.036361	6.678495	0.594639
NFAM1	5.830821	1.60412	21.19447	0.007414
CYCSP52	2.452232	0.391355	15.36568	0.338056
KIAA0040	2.656958	0.594935	11.86587	0.200606
IFITM4P	26.05547	3.205509	211.7877	0.002292
B3GALT4	1.931787	1.16893	3.192491	0.010201
EGOT	0.419061	0.109622	1.601977	0.203656
PNMA6A	0.981573	0.13734	7.015347	0.985212
ZBTB22	1.027114	0.36949	2.855183	0.959098
CLEC2L	1.506374	0.522313	4.344452	0.448373
ZBED5	0.742904	0.394828	1.397839	0.356803
SLFN14	0.07078	0.003934	1.273589	0.072502
BCYRN1	0.991173	0.424718	2.313122	0.983641
B3GNT9	1.843465	0.125207	27.14193	0.65578
CDKN2AIP	0.745353	0.271169	2.048726	0.568885
CECR7	1.628247	0.661146	4.00999	0.289079
RGL2	1.139987	0.518955	2.504206	0.744193
ID2B	3.230023	0.505732	20.62959	0.215218
HLA-DQA	0.523785	0.07378	3.718473	0.517848
PYY2	4.203271	0.138419	127.6378	0.409664
KIFC1	0.841434	0.574785	1.231784	0.374603
IRGM	0.829735	0.45428	1.515498	0.543661
PTENP1	0.556142	0.044715	6.917002	0.64824
LRRC37A2	0.614594	0.063661	5.933424	0.673908
OR2W3	1.232436	0.503532	3.016488	0.647224
PAGE2B	1.077601	0.169108	6.866746	0.936956
SNORD12:	0.3566	0.027817	4.571469	0.428218
SNORD11	0.181454	0.069892	0.471087	0.000454
SNORD12(	1.135645	0.057908	22.27139	0.933239
SNORA13	0.275989	0.078285	0.972984	0.045224
SNORD42I	0.268043	0.051133	1.405107	0.119329
MIR1976	0.790552	0.285428	2.189595	0.65115
SCARNA7	0.704759	0.113779	4.36537	0.70687
SNORD12	0.082092	0.005665	1.189602	0.06685
SCARNA1	0.319024	0.053647	1.897139	0.209117
SNORD12:	36.76121	1.359544	994.0001	0.03215
SNORA47	0.051509	0.006239	0.425256	0.005889
SNORD13	1.09935	0.861221	1.403323	0.446979
SNORD12	17.07152	0.996041	292.595	0.050321
SNORA59/	1.279785	0.359062	4.561473	0.703627
SNORA84	0.095455	0.011766	0.774407	0.027855
TXNDC5	1.131708	0.453245	2.825763	0.790998
RNF103	1.18984	0.658644	2.149446	0.564572
RBM14	0.846257	0.382592	1.871837	0.680234
ALKBH6	0.81902	0.310919	2.157459	0.686217
ASB14	0.196163	0.006607	5.823698	0.346446
NME1	0.546536	0.239127	1.249133	0.151997
TNFSF12	1.933022	1.141887	3.272282	0.014128
CDRT4	0.386138	0.198679	0.750472	0.005006
APOBEC3(	1.449723	0.695656	3.021174	0.321545
WBP1	0.653015	0.280405	1.520761	0.323137
MRPS17	0.414731	0.190709	0.901904	0.026389
DEFA3	1.137805	0.934644	1.385128	0.198285
ADSL	0.506425	0.256405	1.000236	0.05008
LILRA4	1.091385	0.365337	3.260335	0.87555
LILRA2	1.067805	0.825919	1.380531	0.61666
AMY2B	0.452149	0.179645	1.138017	0.091905
LY6G5B	0.044777	0.005117	0.391803	0.005006

PSMB9	1.312754	0.737098	2.337983	0.35543
PCDHGC3	0.358881	0.002837	45.3931	0.678165
COX19	0.668588	0.323913	1.380032	0.276236
DEFA1B	1.128197	0.941466	1.351963	0.19135
GHRLOS	0.09071	0.008384	0.981409	0.048222
ACAD11	0.546542	0.347049	0.860709	0.009125
PPIL3	0.267842	0.046196	1.552929	0.141801
RPL23AP7	0.605431	0.278657	1.315407	0.204973
KIR3DL2	1.216377	0.584035	2.533366	0.600783
RPL29P2	1.578474	0.87343	2.852638	0.130593
TNFRSF13	1.557099	0.539569	4.493509	0.412821
AQP1	1.095867	0.38271	3.13795	0.864575
ISY1	0.617666	0.262106	1.455561	0.270618
PCDHGC5	31.52385	0.438823	2264.587	0.113584
RDH14	0.667198	0.252583	1.762408	0.414198
PLCXD2	0.300378	0.032623	2.765727	0.288314
NSUN6	0.268583	0.101914	0.707817	0.007839
HLA-DOB	1.014317	0.42949	2.395494	0.974136
KRTAP5-8	0.536667	0.028899	9.96614	0.676293
CRCP	0.184444	0.024279	1.401165	0.102274
RPL36A	0.434885	0.126459	1.49554	0.186403
PDXP	0.569834	0.319859	1.015168	0.056279
RPP21	1.053882	0.445643	2.492279	0.904876
CD302	1.134224	0.704661	1.825649	0.604027
EGFL8	0.232568	0.012464	4.339421	0.328613
ARHGAP8	0.983088	0.028681	33.6966	0.992453
ARPC4	2.114105	0.887205	5.037662	0.091062
CORT	2.61867	0.154387	44.41729	0.505102
ARPC1A	1.249727	0.55038	2.837708	0.594179
SUMO1P1	0.380459	0.017378	8.329397	0.539396
PLEKHO2	2.153719	1.283756	3.613229	0.003659
PISD	1.025585	0.541855	1.941156	0.93814
PWP2	0.448549	0.132538	1.518028	0.197429
PI4KA	0.750148	0.092297	6.096834	0.78799
AMACR	0.371205	0.073989	1.862346	0.228477
SNHG3	0.986541	0.214685	4.533446	0.986105
ARFGAP3	0.219117	0.062348	0.770066	0.017913
BGLAP	1.088258	0.357805	3.309919	0.881532
PEG10	7.410382	0.20172	272.2281	0.276021
AK2P2	0.704582	0.238986	2.077254	0.525596
EIF6	1.330185	0.38932	4.544833	0.649011
PCDHGC4	45.72925	1.932249	1082.244	0.017885
MRPL20	0.756161	0.284353	2.010811	0.575403
SERPINB1(	0.87498	0.615541	1.243768	0.45671
HLA-DMB	1.127443	0.912868	1.392457	0.265445
DECR2	0.484059	0.217535	1.077131	0.075421
CNTF	0.035961	0.00169	0.765057	0.033037
ZNF702P	1.241021	0.468791	3.285333	0.663759
ZNF709	0.266648	0.027911	2.547421	0.251005
EIF4EBP3	0.610965	0.083339	4.479035	0.627845
ABCC13	1.383306	0.193746	9.87653	0.746292
MRPL33	1.01489	0.643771	1.599952	0.949255
MICAL3	1.280291	0.076679	21.37679	0.86342
STON1	1.54224	0.279347	8.514506	0.619195
PRAF2	1.711963	0.593904	4.934839	0.319566
KCTD7	0.630748	0.252436	1.576016	0.323962
EFNA4	2.550338	0.865283	7.516876	0.089585
INGX	0.226512	0.017602	2.914847	0.254609

AOX2P	0.390829	0.11197	1.364183	0.140741
UPK3B	1.521211	0.017245	134.1882	0.854373
IL10RB	1.18893	0.66715	2.118795	0.557188
CFB	1.048443	0.434778	2.528264	0.916109
ZNF487	0.855914	0.058634	12.49418	0.909438
WDR92	0.449872	0.226273	0.894427	0.022716
NME2	2.271011	0.925417	5.573153	0.073335
PLA2G4B	1.006695	0.545401	1.858145	0.982976
TTC4	0.877575	0.405807	1.897795	0.739996
RPLP0P2	1.331532	0.146527	12.09997	0.799268
JMJD7	3.71461	0.607183	22.72516	0.155591
APOBEC3D	0.917498	0.143936	5.848464	0.927405
RPL12P6	1.180896	0.759227	1.836758	0.460658
MRPS6	0.772572	0.309214	1.930272	0.580747
ZNF512	0.655651	0.379987	1.131298	0.12934
NFS1	0.157704	0.009483	2.622696	0.197839
DDOST	0.625228	0.210811	1.854316	0.397171
TMEM199	1.441891	0.489884	4.24396	0.506427
P2RY11	0.851632	0.535063	1.355501	0.498242
TMEM141	0.717137	0.375858	1.368297	0.313126
DBNDD2	2.061979	1.187953	3.579062	0.010108
ETV5	1.088427	0.596461	1.986171	0.782461
KRTAP5-7	0.297352	0.013506	6.546411	0.441978
RBM12	0.7623	0.233612	2.48746	0.652858
LILRA6	1.334722	0.629702	2.829087	0.451283
SCARF2	1.252861	0.199023	7.886821	0.810208
APOBEC3C	1.475806	0.537322	4.05344	0.450247
KRT18P34	3.656127	0.316651	42.21456	0.298969
ASPRV1	0.802876	0.102298	6.301303	0.834556
FCGR2C	0.899646	0.349052	2.318745	0.826708
UBE2V1	2.720022	0.09431	78.44901	0.559636
HBB	1.369772	0.657658	2.852964	0.400625
N4BP2L2	0.590688	0.132542	2.632463	0.489887
ZNF585B	0.14772	0.005159	4.229864	0.26384
CEBPA	0.895069	0.653335	1.226244	0.490091
SNHG6	0.795242	0.424572	1.489524	0.474278
DNM1P35	1.514457	0.125813	18.23003	0.743694
PGAM5	0.433046	0.112885	1.661247	0.222451
SNHG10	0.955786	0.563993	1.619748	0.866562
ZCCHC3	1.512558	0.750309	3.049189	0.247333
TWF2	2.576477	1.31685	5.040994	0.005715
MARS2	0.917898	0.294189	2.863933	0.882687
USP51	0.713339	0.068034	7.479338	0.778143
BCKDHA	0.715921	0.38485	1.331798	0.291325
INSL3	0.589293	0.240763	1.442358	0.246886
PCDHGB8P	4.711595	0.355319	62.47666	0.239856
POU5F2	0.278121	0.019548	3.956944	0.344846
PCP4L1	2.303343	0.16507	32.14033	0.53497
ABHD14A	0.865299	0.465753	1.607594	0.647098
ANP32C	1.216006	0.270926	5.457847	0.798499
CCDC153	0.311257	0.014073	6.883924	0.460032
ACTN3	0.078964	0.006493	0.960275	0.046397
FMN1	2.266174	0.202404	25.37277	0.506827
SNORA58	0.058258	0.005112	0.663969	0.022032
HAUS5	0.205667	0.067956	0.622443	0.005125
SLED1	0.779354	0.38762	1.56698	0.484203
TMEM150	0.616414	0.040469	9.389149	0.727685
NAIP	2.36479	0.732763	7.631706	0.14992

ZNF324B	1.228204	0.238519	6.324382	0.805816
ZNF564	1.519732	0.754783	3.059934	0.241153
PVT1	1.010601	0.466756	2.188114	0.978655
PDCD6	0.31757	0.118053	0.854286	0.023093
TMEM158	1.178426	0.941161	1.475505	0.152345
YJEFN3	0.309379	0.063279	1.512597	0.147369
PTTG2	0.632643	0.068836	5.814349	0.685807
ZNF718	0.199509	0.017753	2.242066	0.1916
GYPB	1.041921	0.868831	1.249493	0.657741
CHCHD10	2.095936	1.434168	3.063065	0.000132
GPR162	1.761468	1.102935	2.813193	0.017782
ATP6V1E2	0.330428	0.10237	1.066552	0.063998
GLI4	1.451322	0.618243	3.406967	0.392276
RNF138P1	2.484987	0.177885	34.71441	0.498666
ZNF674	0.17295	0.037167	0.804786	0.025299
ZNF345	0.288258	0.023533	3.53097	0.330519
SHANK3	1.14239	0.90124	1.448066	0.271152
ZNF550	0.287626	0.081659	1.013107	0.052417
RPL32P3	0.602376	0.082515	4.397486	0.617252
FOXD1	4.229431	0.87295	20.49153	0.073263
MALAT1	0.47845	0.03647	6.276859	0.574576
PRB1	1.086675	0.067816	17.41266	0.953168
SCARNA8	0.174085	0.023091	1.312433	0.089853
SCARNA6	6.71278	0.475545	94.75735	0.158641
SCARNA2:	1.708182	0.106664	27.35584	0.705152
SNORD79	0.489527	0.036503	6.56482	0.589683
SCARNA1:	0.627494	0.049849	7.898792	0.718374
SCARNA5	2.385918	0.405368	14.04305	0.336286
SNORD27	2.051951	0.361241	11.65565	0.417334
MIR2278	0.267067	0.024791	2.877084	0.276328
RNY4	0.908837	0.601842	1.372427	0.649432
SCARNA2(	1.020308	0.428122	2.431618	0.96381
MIR2276	7.124025	0.612555	82.85254	0.116776
SCARNA14	0.3716	0.088828	1.554532	0.175176
SCARNA2:	0.578401	0.095184	3.514747	0.552064
SCARNA3	0.271566	0.054227	1.359992	0.112765
SCARNA1	1.759144	0.132488	23.35754	0.668597
MIR2116	0.532873	0.155385	1.827428	0.316777
PCDHGA1	3.614487	0.56726	23.0309	0.173849
HOXA10	1.21926	0.888858	1.672477	0.218941
TMEM200I	12.39528	0.813291	188.9151	0.0701
PCDHGB6	1.570263	0.481953	5.116115	0.453991
SERPINE3	1.56866	0.165679	14.85221	0.694654
TUG1	0.565943	0.300559	1.065651	0.077895
TRNP1	1.438724	0.982841	2.106063	0.061353
PCDHGA5	10.85188	0.530679	221.9108	0.121506
NACA2	2.110995	0.900369	4.949412	0.085694
PCDHGA7	0.476634	0.018991	11.96243	0.652241
RNF5P1	1.341906	0.687551	2.619022	0.388704
SLC10A5	0.02365	0.00114	0.490764	0.015521
EIF5AL1	3.104451	0.343537	28.05408	0.313147
ALG11	0.311277	0.075815	1.278019	0.10533
PRKDC	0.424705	0.126047	1.431007	0.167056
PCDHGA6	0.646978	0.045193	9.261978	0.748451
PCDHGA8	0.620573	0.047216	8.156339	0.716585
PCDHGA1	0.018499	0.00055	0.622698	0.026149
PCDHGA1	1.138449	0.010279	126.0897	0.956944
PCDHGB2	0.356489	0.034864	3.645116	0.384536

PCDHGB4	2.810832	0.286965	27.53222	0.374711
CLDN23	1.454273	1.024797	2.063738	0.035983
ZNF260	0.955596	0.436532	2.091859	0.909536
LYN	1.514368	0.937669	2.445755	0.089731
PINX1	1.18928	0.239599	5.903159	0.832057
PCDHGB7	6.389236	0.169461	240.8951	0.316614
SIGLEC14	1.130584	0.924905	1.382001	0.230921
CHMP4A	0.524864	0.247869	1.111404	0.092177
SIGLEC12	1.247012	0.436767	3.560341	0.680038
FLJ20021	1.215751	0.613015	2.411118	0.576022
PABPC4L	0.760166	0.31623	1.82732	0.540016
OMP	0.633633	0.030838	13.01949	0.767338
FPGT	0.895437	0.121167	6.617367	0.913817
MEX3A	1.799311	0.673026	4.810394	0.241698
SLC22A18/	1.946577	0.949375	3.991216	0.069041
MPV17L2	1.91882	0.613832	5.998169	0.262409
SCARNA9	1.172702	0.847054	1.623544	0.337126
DPP3	1.276245	0.567107	2.872124	0.555594
ANKHD1-I	1.377238	0.28846	6.575558	0.688198
KRTAP5-9	0.036852	0.001944	0.698553	0.027881
CHMP1B	1.090002	0.585657	2.028669	0.785694
EID3	0.488569	0.189354	1.260599	0.138581
SNHG9	1.015428	0.54543	1.890426	0.961491
CARD17	17.38814	1.874473	161.2973	0.011977
EID1	0.337995	0.102448	1.11511	0.074901
TAS2R43	3.872553	0.306807	48.87981	0.295281
LY6G6E	3.569488	0.303935	41.9209	0.311348
FDXACB1	0.521207	0.169258	1.604989	0.256165
SNHG1	0.426862	0.260216	0.700231	0.000749
TIFAB	0.890162	0.226315	3.501268	0.867748
CYP2A6	0.469623	0.043541	5.065247	0.533353
CTSO	0.856337	0.442202	1.65832	0.645558
ZNF432	0.094762	0.012827	0.700088	0.020921
TAS2R30	8.289467	0.478174	143.7034	0.146202
ZNF10	0.716879	0.339525	1.51363	0.382717
ZNF486	0.782114	0.349876	1.748338	0.54932
HMBS	0.973702	0.715588	1.324918	0.865334
ZNF225	0.429574	0.084763	2.177045	0.307522
TAS2R31	0.045822	0.002325	0.903089	0.042664
POLG2	0.238634	0.106878	0.532815	0.000472
CLEC12B	0.673317	0.22848	1.984229	0.473185
ZNF350	0.463426	0.177627	1.209071	0.115965
ZNF253	4.190141	0.652005	26.92816	0.131202
CAPNS2	1.306989	0.128011	13.34429	0.821317
HP	0.967127	0.782765	1.194912	0.756748
LSM14A	0.54267	0.27597	1.067111	0.076445
NHLRC4	1.960586	0.769218	4.99715	0.158444
GATC	0.810416	0.214676	3.059374	0.756452
MGAM	0.609439	0.156122	2.379009	0.47604
FNTB	0.676114	0.282476	1.618299	0.37943
ZNF878	3.000632	0.124831	72.12778	0.498196
CNPY2	0.438026	0.212309	0.903717	0.025487
MRS2P2	0.350286	0.026072	4.70613	0.428698
CUX1	0.620128	0.089856	4.279712	0.627806
MAP1LC3I	6.723426	0.976176	46.30769	0.052932
CLEC5A	0.732362	0.533388	1.005561	0.054144
CHURC1	0.825461	0.55131	1.235941	0.351658
RTEL1	2.558978	0.393337	16.64824	0.325414

PDF	1.773981	0.51106	6.1578	0.366643
SPESP1	0.98993	0.185536	5.281781	0.990547
MC1R	0.26205	0.076054	0.902921	0.033858
TUBB3	1.057626	0.613094	1.824473	0.840393
ITGB3	1.123283	0.739707	1.705762	0.585459
THTPA	1.170556	0.243423	5.628893	0.844188
MRPL46	1.141174	0.44783	2.907973	0.782008
TYRO3P	0.551818	0.052441	5.80653	0.620516
RBM15B	0.806582	0.325224	2.000392	0.64277
HOXB7	1.194968	0.953561	1.497491	0.12187
MRC1	2.56464	0.090172	72.9429	0.581376
HSPB9	0.300105	0.021534	4.182379	0.370548
MT1L	1.19161	0.288767	4.917235	0.808468
CCPG1	0.587388	0.284035	1.214727	0.151216
EPPK1	0.235038	0.011006	5.019544	0.353897
BOP1	1.094285	0.602576	1.987235	0.767245
FIGNL2	1.983631	0.315111	12.48702	0.465586
PECAM1	1.226637	0.916949	1.640919	0.168837
UBE2MP1	1.412757	0.068303	29.22097	0.823096
GAN	0.080292	0.005068	1.272159	0.073583
HPR	0.07811	0.002239	2.724982	0.159478
PCDHGA9	6.750591	0.213737	213.2078	0.278346
PCDHGB3	0.055987	0.003168	0.989486	0.049163
CORO7	2.255338	1.234192	4.121362	0.008193
SPON1	1.412802	1.025458	1.946457	0.034538
MRPL12	1.28603	0.812025	2.036728	0.283564
ALOX12P2	1.729785	0.153352	19.5117	0.657569
GTF2I	0.176607	0.033518	0.93055	0.040869
ZNF234	0.751999	0.306111	1.847374	0.534245
IKBKE	0.967615	0.382791	2.445928	0.944529
SNORD43	0.999451	0.491648	2.031744	0.99879
SNORD3A	1.009519	0.77243	1.319379	0.9447
NBPF11	0.458108	0.074073	2.833179	0.401055
RNU4ATA(	1.15948	0.869401	1.546346	0.313798
DYNLL2	0.64947	0.378406	1.114705	0.117356
OTUD7B	0.820741	0.150205	4.484643	0.819648
DPRXP4	1.283296	0.26161	6.295054	0.758535
SNORD3C	1.008073	0.776819	1.308169	0.951778
SNORD92	0.373485	0.030453	4.580593	0.441262
SNORD53	0.964395	0.081	11.48219	0.977115
ANXA8	0.658466	0.164903	2.62929	0.554189
RBM8A	0.270297	0.052927	1.380383	0.115837
ARGFXP2	3.666233	0.212361	63.29428	0.371389
TIMM23	1.119339	0.449438	2.787749	0.808661
RNF115	0.098138	0.033312	0.289115	2.54E-05
RPL17	0.939361	0.306162	2.882132	0.912914
SEC22B	0.264446	0.100871	0.693282	0.006832
FSBP	2.996581	0.500227	17.95083	0.229531
TXNIP	0.769337	0.442319	1.33813	0.353121
SRGAP2	8.848965	1.131927	69.17779	0.037702
BAHCC1	0.91358	0.442251	1.887225	0.807091
RASSF5	0.703474	0.322338	1.535269	0.377065
STRADA	0.208725	0.031213	1.395775	0.106089
MIR744	0.827177	0.06985	9.795546	0.880405
NBPF15	16.3603	0.237297	1127.951	0.195671
NCOA4	0.835073	0.447708	1.557593	0.570932
MRPS21	1.115086	0.39296	3.164232	0.837803
GDF10	0.654959	0.078647	5.454366	0.695567

MYO15B	2.124268	0.168596	26.76521	0.560011
AARSD1	0.60639	0.274716	1.338506	0.21562
MIR212	0.445625	0.077272	2.569903	0.365918
MIR132	0.385383	0.024258	6.122575	0.499183
LCN6	1.045696	0.343149	3.18661	0.937355
S1PR2	0.783842	0.055638	11.04294	0.856801
ZNF224	0.358754	0.141708	0.90824	0.030537
LIN37	2.677318	0.733993	9.76581	0.135811
NDUFA7	0.780228	0.359757	1.692131	0.529808
NBPF12	6.571957	0.438906	98.40521	0.172699
CLEC4GP1	299.1861	2.789533	32088.65	0.016847
CT45A1	0.815985	0.512422	1.299381	0.391611
MAGIX	0.180698	0.014068	2.321061	0.189007
IKBKG	1.112197	0.515975	2.397365	0.786111
SPIB	1.522935	1.107328	2.094528	0.009683
TMEM185/	0.841449	0.234466	3.019782	0.791174
NBPF9	10.12786	0.353818	289.905	0.176097
KCNQ1OT	1.067135	0.338199	3.367179	0.911751
EGLN2	1.288648	0.313768	5.292485	0.724963
SNHG8	1.18514	0.408702	3.436626	0.7545
TERC	1.527368	0.676235	3.449769	0.308265
NBPF8	1.609665	0.802432	3.22896	0.180164
NBPF14	0.821679	0.327637	2.060685	0.675454
TAF15	0.412815	0.203581	0.83709	0.014168
RASL10B	7.900742	1.71815	36.33077	0.007925
SRXN1	1.715165	0.981902	2.996011	0.057988
NBPF10	2.117095	0.387202	11.57559	0.38686
MMP28	1.906035	1.005587	3.612785	0.048037
CCL5	0.941728	0.711738	1.246038	0.674303
LIX1L	0.924898	0.349816	2.445384	0.874944
SNORD11I	0.428448	0.121185	1.51477	0.188353
MIR98	1.120077	0.570106	2.200595	0.742076
ANKRD34/	0.601435	0.062461	5.791235	0.659932
SNORD14/	0.534164	0.304151	0.938122	0.02909
MIR139	2.264253	0.202861	25.27267	0.506721
GTF2H5	1.076488	0.585771	1.978292	0.812357
NUDT3	0.690661	0.243733	1.957113	0.486154
POM121C	0.763802	0.393321	1.48325	0.426194
CD24	1.082881	0.86953	1.348579	0.476946
SNORA28	0.364235	0.200189	0.66271	0.000942
ZNF595	25.29109	1.098176	582.4558	0.043541
DOC2B	1.984677	0.336392	11.70937	0.449102
MESTIT1	2.515586	0.157436	40.19522	0.514115
DCP1A	0.78586	0.349202	1.768533	0.560376
FAM106A	83.28828	1.003168	6915.033	0.049836
SNURF	0.632221	0.399883	0.999551	0.049776
DGCR11	1.094703	0.285055	4.204008	0.895143
ZNHIT3	1.041826	0.28165	3.853721	0.951044
CYFIP1	3.082744	1.216695	7.810757	0.017622
USP27X	1.712501	0.257987	11.36748	0.577498
TAF9	0.973441	0.594119	1.594944	0.914906
NOL12	0.530045	0.22866	1.228669	0.138905
ANTXRL	9.185588	1.025112	82.3081	0.047464
SOCS7	1.524494	0.105178	22.09667	0.75725
ADRA2B	3.491878	0.63441	19.21976	0.150716
ZNF658	4.394585	0.826229	23.37411	0.082545
SEBOX	0.033986	0.000859	1.344487	0.071512
PI4KAP1	0.675585	0.318653	1.432324	0.306375

CCL23	0.844518	0.656923	1.085685	0.187329
RNU11	1.004807	0.796538	1.267532	0.96772
ZNF280B	0.337054	0.049436	2.298038	0.266828
MLLT6	0.752067	0.419398	1.34861	0.338948
NUDT18	1.37882	0.716938	2.651757	0.335701
SNORD91I	0.436588	0.063526	3.000469	0.399391
ZNF2	3.384711	0.536209	21.36529	0.194631
LENG9	5.517055	0.671309	45.34113	0.112029
CCL4	2.69924	0.849126	8.580465	0.092414
MIR1299	2.488583	0.177047	34.97977	0.498988
CCL18	0.403811	0.035274	4.62272	0.465961
FCGBP	2.096605	0.914943	4.804401	0.080144
XKR5	0.228641	0.017666	2.959125	0.258679
MT1IP	0.73774	0.063576	8.560812	0.807857
AATF	0.663251	0.237636	1.85116	0.433009
ARHGAP2:	1.253825	0.379299	4.144698	0.710785
TUBGCP5	0.16438	0.050851	0.531376	0.00256
PRSS2	1.179898	0.78348	1.776891	0.428421
DUSP14	0.993555	0.647695	1.5241	0.976372
UHRF1	0.620621	0.359054	1.072738	0.087545
ORAI1	1.732834	1.041117	2.884125	0.034432
CCL4L2	1.457793	0.972058	2.186246	0.068317
CCL3L3	1.212741	1.021041	1.440433	0.028009
RN7SL1	0.355589	0.063623	1.987398	0.238923
PIK3R6	0.908334	0.539454	1.529456	0.717618
TADA2A	0.19405	0.036888	1.02079	0.052908
PIP4K2B	0.757979	0.197049	2.915687	0.686849
PCDHGB5	0.162053	0.009916	2.648348	0.201708
HERC2P2	0.199316	0.025896	1.5341	0.121388
RAB7B	0.775501	0.605164	0.993783	0.044511
DACH1	1.048736	0.603644	1.822014	0.865913
PADI6	0.204895	0.02341	1.793365	0.152073
GTF2IP1	0.657241	0.314754	1.372392	0.263876
STAG3L2	0.707974	0.49813	1.006216	0.054177
TYW1B	0.354661	0.168288	0.747436	0.006424
F8A3	3.639235	0.647518	20.45355	0.142498
PIGW	0.047895	0.005564	0.412314	0.005665
F8A1	1.109138	0.604056	2.036545	0.738309
PCGF2	1.009217	0.118765	8.57593	0.993295
MARCKS	0.970926	0.777721	1.212128	0.79438
ZNF670	5.89326	0.366975	94.64009	0.210477
PKD1L3	0.07987	0.007493	0.851405	0.036332
CCL3	1.235148	1.042219	1.463789	0.014802
PGM5P2	8.347964	0.312533	222.9799	0.205493
PSMB3	1.734664	0.566605	5.310683	0.334621
CISD3	1.102581	0.21114	5.757708	0.907814
RDM1	0.371061	0.031049	4.43443	0.433478
DDX52	0.63373	0.174766	2.298015	0.487679
ZNF8	0.293233	0.033303	2.581889	0.269011
SSTR3	2.340189	0.158152	34.62797	0.536266
MYO19	0.097768	0.017182	0.556307	0.008767
GGNBP2	0.73154	0.355965	1.503378	0.395
ZNF229	0.807409	0.064048	10.17848	0.868589
DHRS11	0.601228	0.343001	1.053859	0.075607
ACACA	0.654271	0.240876	1.777142	0.405344
TMEM191I	2.757164	0.62433	12.17617	0.180787
NR2E3	0.00306	3.02E-05	0.310221	0.014024
MRM1	0.856247	0.325443	2.252807	0.753189

MRPL45	0.843158	0.425715	1.669933	0.624637
OR2L8	0.416657	0.029829	5.819924	0.515196
FLJ42393	0.367554	0.038962	3.467343	0.382069
PLAC4	0.545023	0.109848	2.704199	0.457683
SNHG4	0.548114	0.210963	1.42408	0.217105
HSP90AB4	0.35059	0.046013	2.671268	0.311714
WASH5P	1.346996	0.910468	1.992819	0.136067
ADORA3	2.119688	0.805805	5.575883	0.127903
HYMAI	0.878303	0.10465	7.371376	0.904838
MIR1322	2.416074	0.229647	25.41903	0.462532
MIR1910	0.218168	0.013571	3.507252	0.282632
SNORD98	4.722644	0.483407	46.13783	0.181909
MIR924	7.489543	0.455984	123.0157	0.15853
EXOC3L2	1.399956	0.193566	10.12512	0.738926
MIR555	0.022621	0.001742	0.293751	0.003774
MIR130B	2.883107	0.214272	38.79322	0.424638
MIR628	3.333908	0.313922	35.40667	0.317858
MIR1281	2.444214	0.59174	10.09597	0.21685
MIR29A	8.75988	0.639662	119.9625	0.104093
SCO2	1.604625	1.076597	2.391631	0.02021
MIR718	2.44258	0.190331	31.34636	0.492799
TBCE	0.345772	0.128717	0.928846	0.035173
ARHGAP1:	0.397776	0.138942	1.13879	0.085835
TOP1P1	3.341869	0.542832	20.57376	0.19322
NPBWR1	0.161373	0.010267	2.536532	0.194378

Supplementray file 2. Results of univariate Cox regression analysis in TARGET-LAML

id	HR	HR.95L	HR.95H	pvalue
TSPAN6	1.273179	1.168387	1.38737	3.57E-08
DPM1	0.990395	0.881917	1.112216	0.870471
SCYL3	1.107698	1.00899	1.216063	0.031722
FGR	1.077213	1.033198	1.123104	0.000475
CFH	1.106723	1.052263	1.164001	8.19E-05
FUCA2	1.059742	0.966839	1.161571	0.215139
GCLC	1.08653	1.012974	1.165426	0.020319
NFYA	1.036553	0.96703	1.111075	0.310815
LAS1L	1.094153	0.989616	1.209732	0.07905
ENPP4	1.132443	1.074406	1.193614	3.59E-06
SEMA3F	0.945325	0.899498	0.993487	0.026576
ANKIB1	1.01041	0.94066	1.085331	0.776597
CYP51A1	1.046276	0.945147	1.158226	0.383084
KRIT1	1.021172	0.928745	1.122798	0.665136
RAD52	1.041106	0.9686	1.119039	0.274064
BAD	1.379633	1.209752	1.57337	1.59E-06
LAP3	1.135899	1.046006	1.233518	0.002452
CD99	0.841457	0.797796	0.887508	2.16E-10
HS3ST1	1.01201	0.918624	1.11489	0.809022
MAD1L1	1.115428	1.012402	1.228938	0.027158
LASP1	0.958239	0.89087	1.030703	0.251426
SNX11	1.036727	0.944151	1.138379	0.449788
TMEM176	0.915598	0.868342	0.965426	0.001109
M6PR	1.061442	0.975345	1.155139	0.167106
KLHL13	1.0108	0.950391	1.075049	0.732599
ICA1	1.062606	1.00828	1.119858	0.023334
DBNDD1	0.908013	0.833302	0.989423	0.027617
ALS2	0.904495	0.850091	0.962381	0.001517
CASP10	0.990018	0.919759	1.065643	0.789378
CFLAR	1.028089	0.948168	1.114745	0.502274
TFPI	0.929524	0.889017	0.971876	0.001305
RBM5	0.981845	0.917469	1.050738	0.596427
MTMR7	0.93792	0.871888	1.008952	0.085307
SLC7A2	0.912179	0.798144	1.042506	0.177326
ARF5	0.995095	0.89743	1.10339	0.925678
SARM1	1.321203	1.210755	1.441727	4.01E-10
POLDIP2	1.087081	0.981379	1.204169	0.10964
PLXND1	0.930166	0.882016	0.980945	0.0076
AK2	1.103155	1.035185	1.175588	0.00248
CD38	1.031258	0.980699	1.084424	0.230106
FKBP4	1.066216	1.001448	1.135173	0.044941
RBM6	1.038726	0.952777	1.132428	0.38857
CAMKK1	1.057337	0.982347	1.138051	0.137426
RECQL	1.079712	0.994035	1.172773	0.069044
HSPB6	0.924792	0.823154	1.038981	0.188102
NDUFAB1	1.114442	1.010044	1.229629	0.030842
PDK4	1.130976	1.069793	1.195659	1.44E-05
SLC22A16	0.970154	0.912075	1.03193	0.336029
ZMYND10	1.019396	0.934032	1.112562	0.666811
SLC25A13	0.983065	0.910665	1.061221	0.661672
ST7	0.95825	0.897068	1.023605	0.205198
CDC27	1.037637	0.9667	1.113781	0.306499
SLC4A1	0.988273	0.9626	1.014632	0.379752
HCCS	1.116307	0.989853	1.258915	0.072863
DVL2	1.025686	0.938601	1.120851	0.575314
UPF1	0.997309	0.929301	1.070295	0.940403

SKAP2	1.074958	1.022926	1.129637	0.004298
SLC25A5	1.045143	0.949575	1.15033	0.36682
HOXA11	1.096874	1.058976	1.136128	2.55E-07
POLR2J	1.067528	0.971275	1.17332	0.175284
DHX33	1.044091	0.971429	1.122188	0.24106
THSD7A	0.856685	0.804344	0.912433	1.52E-06
LIG3	1.012207	0.946584	1.08238	0.722747
RPAP3	1.036631	0.948872	1.132506	0.425381
ACSM3	0.859338	0.823364	0.896883	3.71E-12
CIAPIN1	1.20187	1.077634	1.34043	0.000957
SPPL2B	1.055983	0.97617	1.142323	0.174314
COPZ2	1.373616	1.230374	1.533535	1.61E-08
PRKAR2B	1.150023	1.101179	1.201034	2.74E-10
MSL3	0.973176	0.891275	1.062604	0.544389
CREBBP	0.906687	0.836171	0.98315	0.017724
MPO	0.907284	0.887704	0.927297	2.32E-18
WDR54	1.296804	1.177356	1.42837	1.35E-07
CROT	1.165898	1.065891	1.275288	0.000795
ABCB4	1.136899	1.026118	1.25964	0.014172
RHBDD2	0.907226	0.845369	0.973609	0.006887
IBTK	0.973496	0.904196	1.048108	0.475894
ZNF195	1.09359	1.005738	1.189117	0.036272
MYCBP2	1.030755	0.974283	1.0905	0.292021
FBXL3	0.96171	0.893028	1.035675	0.301726
ITGAL	1.042761	0.988618	1.099869	0.123766
PDK2	1.302981	1.190597	1.425973	8.89E-09
ITGA3	0.99199	0.899861	1.093551	0.871533
ZFX	0.9953	0.928911	1.066435	0.893605
LAMP2	1.026206	0.94263	1.117192	0.550622
ITGA2B	1.113327	1.080094	1.147583	3.84E-12
GDE1	0.944378	0.861724	1.034959	0.220705
CRLF1	1.290939	1.070866	1.556238	0.007407
OSBPL7	1.11046	1.016467	1.213145	0.020237
TMEM98	1.150042	1.056096	1.252346	0.001303
YBX2	1.152589	1.046501	1.269431	0.003945
MAP3K14	0.976574	0.900893	1.058613	0.56463
TMEM132/	1.081881	1.013861	1.154464	0.017526
AP2B1	0.989161	0.913529	1.071054	0.78828
ZNF263	0.960228	0.881562	1.045913	0.352049
SPATA20	1.025074	0.960551	1.093931	0.455311
TNFRSF12/	0.922824	0.876213	0.971915	0.002388
MAP3K9	1.134478	1.036457	1.241768	0.006207
RALA	0.921219	0.852866	0.995051	0.03697
BAIAP2L1	0.998427	0.930085	1.07179	0.965287
AGK	1.1002	0.999665	1.210845	0.050806
ALDH3B1	0.917838	0.867736	0.970833	0.002758
TTC22	1.342941	1.147091	1.57223	0.000246
PHTF2	0.957956	0.881881	1.040594	0.308946
FARP2	1.17137	1.080886	1.269429	0.000115
GGCT	1.110283	1.013159	1.216717	0.0251
DBF4	1.001045	0.921447	1.087519	0.980294
TBXA2R	0.893338	0.84624	0.943057	4.47E-05
IFRD1	0.911361	0.863685	0.961668	0.00071
COX10	1.082368	0.982777	1.19205	0.10801
GTF2IRD1	1.042469	0.964541	1.126694	0.294081
PAF1	0.861025	0.79141	0.936764	0.000504
VPS41	1.048662	0.982113	1.119719	0.155488
ELAC2	1.135336	1.04062	1.238671	0.004292

ARSD	1.006066	0.950672	1.064688	0.834224
PNPLA4	0.938953	0.847339	1.040473	0.229158
ADIPOR2	1.031173	0.960091	1.107518	0.39959
CDKL3	1.283982	1.064971	1.548032	0.008802
PRSS21	0.986738	0.95963	1.014612	0.347573
MARK4	0.878785	0.800858	0.964295	0.006384
PROM1	1.014736	0.983806	1.046639	0.354331
CCDC124	1.118215	1.01415	1.23296	0.024969
CEACAM2	1.114352	1.042014	1.191712	0.001568
PAFAH1B1	0.961809	0.89436	1.034346	0.293875
KIAA0100	0.994194	0.924076	1.069633	0.876009
GAS7	0.909354	0.865024	0.955956	0.000194
TRAPPC6A	1.046951	0.962153	1.139221	0.287019
MATK	0.951684	0.892174	1.015163	0.132792
CD79B	1.015241	0.955144	1.079121	0.627065
SCN4A	1.078579	0.92913	1.252067	0.320207
ST7L	1.005957	0.930075	1.08803	0.882012
TKTL1	1.032855	0.999016	1.06784	0.057167
RPUSD1	0.991667	0.911429	1.078969	0.845878
RHBDF1	0.943426	0.893025	0.996671	0.037615
LUC7L	1.034782	0.957609	1.118175	0.387251
CACNA2D	0.8705	0.813618	0.931359	5.76E-05
BAIAP3	0.821323	0.771206	0.874696	8.92E-10
PIGQ	1.032029	0.955613	1.114555	0.42185
TEAD3	1.074406	1.00064	1.15361	0.047976
DNAJC11	1.093135	1.007442	1.186116	0.032519
MYLIP	0.96172	0.89694	1.031179	0.272624
E2F2	1.032758	0.985577	1.082197	0.176684
PSMB1	1.040949	0.93412	1.159996	0.467583
SYN1	0.83921	0.770576	0.913956	5.66E-05
JARID2	0.94581	0.88523	1.010535	0.099011
CDKL5	1.176649	1.060555	1.305451	0.002146
CAMK1G	1.140542	1.025092	1.268994	0.015731
NADK	1.002807	0.939273	1.070638	0.933104
DLEC1	0.995699	0.93431	1.061122	0.8944
CYTH3	0.845601	0.787462	0.908034	3.94E-06
ADAM22	1.060279	0.976306	1.151474	0.16442
SYPL1	0.957945	0.877703	1.045522	0.335746
CYB561	1.024254	0.953497	1.100262	0.511723
SPAG9	0.923409	0.870457	0.979583	0.008178
CELSR3	0.980383	0.913178	1.052535	0.584516
AASS	1.201565	1.121329	1.287542	1.91E-07
PLEKHG6	0.958941	0.903186	1.018138	0.170125
SS18L2	1.260102	1.116909	1.421654	0.000172
MPND	1.00756	0.917282	1.106723	0.875046
MGST1	0.89009	0.853863	0.927853	3.97E-08
CRY1	1.083592	1.02092	1.150111	0.008264
PGLYRP1	0.989935	0.96379	1.01679	0.458859
NFIX	1.009669	0.957052	1.065179	0.724547
ST3GAL1	1.025835	0.949416	1.108405	0.51842
MMP25	0.965391	0.92086	1.012076	0.143804
IL32	1.056895	0.988282	1.130271	0.106142
PKD1	0.964717	0.907575	1.025458	0.248903
MED24	1.065549	0.968672	1.172114	0.191727
RHOBTB2	1.210605	1.115415	1.313919	4.78E-06
HEATR5B	1.004961	0.945408	1.068266	0.873837
SEC62	0.953289	0.868309	1.046586	0.315304
RPS20	0.863273	0.795253	0.93711	0.000446

CSDE1	0.894833	0.828676	0.966273	0.004576
UBE3C	0.98487	0.91704	1.057717	0.675405
REV3L	0.917869	0.860925	0.978579	0.008726
MASP2	0.970505	0.914998	1.02938	0.319096
FAM76A	1.209486	1.090334	1.34166	0.000325
TRAF3IP3	1.049053	0.981658	1.121076	0.157503
POMT2	0.983062	0.895549	1.079126	0.719501
VTA1	1.142712	1.031909	1.265413	0.01036
MLXIPL	1.215767	1.108761	1.3331	3.23E-05
BAZ1B	0.983518	0.904816	1.069066	0.696135
RANBP9	0.854296	0.793556	0.919685	2.85E-05
ETV7	1.233208	1.139151	1.33503	2.24E-07
DYRK4	1.338485	1.161498	1.542441	5.60E-05
ZNF207	0.93234	0.858441	1.0126	0.096359
UQCRC1	1.127016	1.01371	1.252986	0.026977
STARD3NL	0.847006	0.772218	0.929038	0.000431
CD9	1.010525	0.967348	1.055629	0.638412
NCAPD2	1.066508	0.994093	1.144198	0.072681
IFFO1	1.023268	0.952145	1.099703	0.531455
GIPR	1.004528	0.94077	1.072608	0.892583
PHF7	1.116848	1.024851	1.217104	0.011748
SEMA3G	1.208366	1.126961	1.295651	1.04E-07
NISCH	1.135777	1.05137	1.22696	0.001232
STAB1	0.988169	0.959904	1.017266	0.421508
FUZ	1.093161	0.987902	1.209636	0.084646
SLC6A13	1.111546	1.043135	1.184444	0.001102
IDS	0.987785	0.8988	1.08558	0.798592
PRSS3	0.892352	0.776015	1.02613	0.110034
ZNF200	1.087552	0.976764	1.210906	0.12575
CD4	1.076177	1.037098	1.116729	0.0001
LRRC23	1.120895	1.00618	1.248689	0.038283
BTK	0.946552	0.891041	1.005521	0.074845
HFE	1.136792	0.984517	1.312619	0.080584
SCMH1	0.800773	0.746976	0.858445	3.81E-10
FYN	1.016899	0.95767	1.079791	0.584159
HIVEP2	0.945435	0.894843	0.998888	0.045541
LYPLA2	1.17598	1.054667	1.311247	0.003522
CLCN6	0.924481	0.862102	0.991374	0.027593
MRC2	1.058218	1.013086	1.10536	0.01094
TSPAN9	0.866692	0.787248	0.954153	0.003537
BTBD7	1.009927	0.925204	1.102407	0.825124
APBA3	1.221436	1.093457	1.364393	0.000397
MKS1	1.076194	0.975584	1.18718	0.142552
ABHD5	0.985405	0.925778	1.048873	0.644325
AKAP8L	0.929708	0.856817	1.008799	0.080176
MBTD1	0.962691	0.899014	1.030878	0.276157
UTP18	0.926847	0.847624	1.013475	0.095644
RNF216	1.003057	0.934663	1.076455	0.932492
TTC19	0.925802	0.855475	1.001911	0.0558
PTBP1	0.963339	0.87885	1.055951	0.42516
DPF1	1.170996	1.030878	1.330159	0.015197
LARS2	1.017624	0.939044	1.102779	0.670048
PIK3C2A	0.962759	0.902134	1.027459	0.252763
PLAUR	1.032846	0.980718	1.087744	0.221297
ANLN	1.058377	0.99796	1.122453	0.05851
WIZ	1.037474	0.943941	1.140275	0.445357
RABGAP1	1.057242	0.982542	1.137622	0.136522
QPCTL	0.969422	0.894537	1.050575	0.448978

PPP5C	1.075743	0.996956	1.160758	0.059918
CEP68	0.994992	0.932736	1.061403	0.878948
MAP4K3	0.959896	0.900164	1.023591	0.211797
ZBTB32	1.094779	0.995866	1.203516	0.0609
TYROBP	0.985212	0.937177	1.035709	0.559098
TMEM159	1.088419	0.994304	1.191442	0.066333
BRCA1	1.084689	1.02123	1.152091	0.008218
ERCC1	1.166815	1.043439	1.304779	0.006816
CD22	1.024382	0.965841	1.08647	0.422359
SEMA3B	0.968281	0.902734	1.038587	0.367433
MBTPS2	1.053538	0.957431	1.159293	0.285238
PRICKLE3	1.102078	0.993046	1.223081	0.067448
LTF	1.006248	0.982816	1.030238	0.604399
EXTL3	1.078985	0.997126	1.167563	0.058963
ELOVL5	1.078681	0.993126	1.171606	0.072436
ALOX5	0.963945	0.923633	1.006017	0.092038
CALCOCO	1.111108	1.022377	1.207539	0.013097
UBR7	1.183038	1.08296	1.292364	0.000194
MAP4K5	0.941924	0.871816	1.01767	0.129493
EHD3	0.949932	0.898662	1.004127	0.069603
PSMC4	1.04113	0.930687	1.164679	0.481138
MAN2B2	1.034328	0.955761	1.119354	0.402378
SLC25A39	1.068126	1.005955	1.13414	0.031238
MVP	1.073539	0.993533	1.159989	0.072531
NUB1	0.949978	0.870785	1.036374	0.247895
PGM3	1.038377	0.966375	1.115743	0.304371
RWDD2A	1.075267	0.967469	1.195075	0.17818
CLK1	0.923664	0.863538	0.987976	0.020767
POLR3B	1.044735	0.966767	1.12899	0.268774
ANGEL1	1.182266	1.088694	1.283879	6.89E-05
RNF14	1.074683	0.987339	1.169754	0.095844
DNASE1L1	1.078694	0.962853	1.208472	0.191252
DDX11	1.086759	1.008457	1.171141	0.029207
HEBP1	0.982555	0.887486	1.087809	0.734648
GPRC5A	0.984244	0.883585	1.09637	0.772948
MAMLD1	1.090911	1.011756	1.17626	0.02357
CD6	0.97986	0.935278	1.026567	0.3918
TACC3	1.01588	0.932859	1.106289	0.717201
POLA2	1.254595	1.131854	1.390647	1.58E-05
ZC3H3	0.859416	0.791726	0.932893	0.000295
CAPN1	1.009827	0.923318	1.10444	0.830544
MDH1	1.163238	1.054914	1.282686	0.00243
SLC30A9	1.0782	0.987059	1.177756	0.09474
MTMR11	1.041901	0.995652	1.090298	0.076416
COX15	1.119475	1.030172	1.21652	0.007796
CCDC88C	0.903939	0.838721	0.974227	0.008208
YAF2	0.979185	0.916412	1.046258	0.533771
ZMYND11	0.968086	0.899736	1.041628	0.385273
WAS	0.984796	0.909772	1.066006	0.704722
DPEP1	1.057176	0.988186	1.130983	0.106351
BID	1.191844	1.072178	1.324866	0.00115
MATR3	1.006435	0.948243	1.068199	0.832819
NPC1L1	1.025514	0.916587	1.147386	0.660124
XYLT2	1.131683	1.048863	1.221042	0.001421
NUDCD3	1.086397	1.004226	1.175293	0.038919
GLT8D1	1.092976	1.003824	1.190045	0.040573
ATP2C1	0.944785	0.889004	1.004066	0.067359
SLC38A5	1.024931	0.959587	1.094724	0.463775

RALBP1	0.93987	0.858585	1.028851	0.179053
RUFY3	0.939436	0.87589	1.007593	0.080416
SLC11A1	0.998588	0.957868	1.041038	0.946956
WWTR1	1.316677	1.090409	1.589898	0.004241
AGPS	0.969776	0.901517	1.043203	0.409855
TTC27	1.035874	0.975019	1.100528	0.253878
ZNF582	1.037929	0.911758	1.18156	0.573458
VSIG2	1.15881	1.092554	1.229084	9.26E-07
PHLDB1	1.2661	1.173454	1.36606	1.16E-09
MARCO	0.951528	0.901257	1.004604	0.072799
PRDM11	0.997197	0.92546	1.074493	0.941249
CD74	0.974074	0.929718	1.020545	0.269292
HGF	0.872574	0.838765	0.907745	1.37E-11
ZRANB1	0.902933	0.840151	0.970407	0.005488
NCDN	1.060617	0.991015	1.135107	0.089255
ZFP64	1.190958	1.091629	1.299324	8.39E-05
MNAT1	1.023199	0.938611	1.115411	0.602412
SAMD4A	1.055652	0.929756	1.198596	0.403225
RUNX3	1.110349	1.060604	1.162428	7.61E-06
PLEKHB1	1.035386	0.965189	1.110688	0.33164
SERPINB1	0.93228	0.874059	0.994379	0.033065
SPAST	0.994531	0.909549	1.087454	0.904227
OSBPL5	0.976877	0.924455	1.032272	0.405803
AQR	1.042882	0.96137	1.131305	0.311927
FHL1	1.08152	1.032715	1.132632	0.00088
NLRP2	1.009114	0.95579	1.065413	0.743252
SLC45A4	1.144416	1.073918	1.219542	3.21E-05
RNF10	0.908853	0.839713	0.983686	0.017913
ZNF839	1.000326	0.918772	1.089118	0.994011
ZDHHC6	1.155893	1.049048	1.273621	0.003416
GRAMD1B	1.119084	1.056034	1.185899	0.000143
RNH1	0.967919	0.900826	1.040009	0.373659
NDUFS1	1.076398	0.987858	1.172874	0.092757
RB1CC1	0.953784	0.90196	1.008584	0.096898
ERP44	0.987433	0.885313	1.101332	0.82038
ALAS1	1.002022	0.931527	1.077852	0.956717
BIRC3	1.023756	0.965169	1.085898	0.434882
AKAP11	1.01086	0.948993	1.07676	0.737467
GLRX2	1.265471	1.127946	1.419764	6.04E-05
SNAPC1	1.039084	0.951094	1.135213	0.395737
DERA	0.931157	0.859224	1.009112	0.082063
STRAP	0.853548	0.779187	0.935006	0.000662
ABCC2	1.063888	0.973979	1.162097	0.169223
DEF6	1.076949	0.98643	1.175774	0.097936
PLEKHO1	0.941614	0.872978	1.015646	0.119252
GCLM	1.139744	1.070828	1.213095	3.95E-05
UBR2	1.008593	0.942095	1.079785	0.805769
EHD2	1.022686	0.974896	1.072819	0.358239
DEPDC1	1.151022	1.064608	1.24445	0.000412
CCDC28A	0.991016	0.901738	1.089134	0.851386
RRAGD	0.858243	0.807441	0.912242	9.09E-07
HSF2	0.907538	0.850747	0.96812	0.003254
PHF20	0.927426	0.850728	1.011039	0.087138
NR1H3	0.968133	0.896034	1.046033	0.412111
TYMP	0.995311	0.944381	1.048988	0.860783
NCAPH2	1.078307	0.981186	1.18504	0.117451
TOMM34	1.101796	0.990296	1.22585	0.07494
SEC63	0.951306	0.874466	1.034897	0.245356

KPNA6	0.94354	0.869948	1.023356	0.160702
VIM	1.016622	0.960182	1.076379	0.571604
FAS	1.017871	0.947788	1.093137	0.626492
RNASET2	0.868928	0.80441	0.938622	0.000358
CD44	0.914843	0.854798	0.979105	0.010182
AGPAT4	1.06782	1.002452	1.13745	0.041756
SLAMF7	1.021529	0.954113	1.093708	0.540878
BTN3A1	1.045205	0.977009	1.118162	0.19903
MIPEP	1.117992	1.032346	1.210743	0.006092
PRKCH	0.990391	0.936657	1.047208	0.734432
IFNGR1	0.997631	0.926406	1.074333	0.949962
B4GALT7	0.997588	0.907247	1.096924	0.960225
SH2D2A	1.020369	0.956482	1.088524	0.541032
VRK2	1.058747	0.971625	1.15368	0.192589
TNFRSF1B	1.016671	0.971461	1.063986	0.476215
VEZT	0.950588	0.881944	1.024575	0.185138
POU2F2	1.013136	0.955516	1.074231	0.66223
BRD9	1.108453	1.008565	1.218233	0.0326
SNX1	0.960494	0.891045	1.035357	0.292528
TBPL1	1.076578	0.979267	1.183558	0.126879
ARNTL2	1.101284	1.027233	1.180673	0.006598
BCLAF1	0.961066	0.893904	1.033275	0.282647
SLC39A9	1.102013	1.016739	1.194438	0.018081
ANK1	1.019052	0.985991	1.053221	0.262057
TFB1M	1.075707	0.997763	1.159739	0.057223
RABEP1	0.957309	0.884791	1.03577	0.277688
HMGB3	1.132158	1.040619	1.231749	0.003907
NUP160	1.052174	0.976517	1.133693	0.181607
BAK1	0.943556	0.866099	1.027939	0.183705
IKZF2	0.916677	0.873114	0.962413	0.000461
GRN	1.052657	1.003065	1.104701	0.037136
FAM13B	0.954109	0.892406	1.020077	0.168449
CENPQ	1.064298	0.977368	1.158959	0.151742
RANBP3	0.965783	0.888229	1.050109	0.414969
ARID4A	0.925496	0.863689	0.991726	0.028123
PNPLA6	1.096979	1.011837	1.189285	0.024741
IFT88	1.09122	0.988246	1.204925	0.084318
ALG1	1.216106	1.110128	1.332201	2.60E-05
ZCCHC8	0.974393	0.901653	1.053002	0.512277
ABCF2	1.162689	1.050096	1.287355	0.003725
CHPF2	0.946204	0.885093	1.011534	0.104526
LRRC7	1.166638	1.027444	1.324689	0.017424
FUT8	1.119352	1.05024	1.193013	0.000525
UBA6	0.943166	0.88679	1.003125	0.062779
GAB2	0.82373	0.778581	0.871498	1.56E-11
ATP6V0A1	1.065667	1.004569	1.13048	0.034747
PIAS1	0.95349	0.878365	1.03504	0.255349
SLC4A7	0.999669	0.946502	1.055823	0.990536
APBA2	1.098285	1.048817	1.150087	6.69E-05
MAP2K3	0.878198	0.817518	0.943381	0.000377
TMSB10	0.965158	0.913612	1.019612	0.205368
ASTE1	1.086916	0.998842	1.182756	0.053225
RNF19A	1.057208	0.982111	1.138047	0.138923
PEX3	1.081294	0.994942	1.175141	0.065688
GABARAPI	1.091767	0.991443	1.202243	0.074226
SH3YL1	1.024577	0.935103	1.122612	0.602526
FAM136A	1.109123	0.996634	1.234309	0.057674
VCL	1.051749	0.983016	1.125288	0.143412

DEPDC1B	1.157696	1.081904	1.238798	2.25E-05
DAPK2	1.059556	0.985063	1.139683	0.119864
NSMAF	0.941973	0.874477	1.014679	0.115066
STAP1	0.900611	0.852669	0.951249	0.000176
TIMP2	0.950284	0.892731	1.011548	0.109651
RFC1	0.97875	0.908694	1.054207	0.570821
TBC1D23	0.940303	0.881207	1.003362	0.063083
CUL3	0.917621	0.855635	0.984099	0.01599
MYOM2	1.158626	1.048819	1.27993	0.003753
CYP46A1	0.919032	0.821172	1.028554	0.1416
ZZZ3	1.018255	0.941291	1.101512	0.651891
USP2	1.044771	0.988047	1.104752	0.124106
TUBG2	1.228773	1.142326	1.321763	3.11E-08
RPL26L1	1.142811	1.022285	1.277548	0.018897
FLT4	0.837202	0.791426	0.885625	5.87E-10
NSUN2	1.020928	0.942713	1.105633	0.610536
FBXO42	0.944978	0.873652	1.022128	0.157547
MFAP3	1.088338	1.000013	1.184465	0.049965
MRI1	1.057886	0.976567	1.145975	0.167918
METTL1	1.111621	1.02378	1.206999	0.011752
AGA	1.034416	0.936397	1.142695	0.505302
PI4K2B	1.050219	0.96743	1.140092	0.242166
MAT2B	1.084054	0.997575	1.17803	0.057076
EDC4	0.949047	0.885053	1.017668	0.142033
TRIO	0.842065	0.804118	0.881803	2.74E-13
VCAN	1.02363	0.994899	1.053192	0.107864
CLEC16A	1.030523	0.956012	1.110842	0.432347
MSR1	1.082969	1.014619	1.155924	0.016562
CDH1	1.070084	1.005532	1.13878	0.032864
ZFYVE16	0.952476	0.886035	1.0239	0.186916
RAI14	1.268887	1.136391	1.41683	2.31E-05
PNKP	1.128775	1.025054	1.242992	0.013773
BEST2	0.875783	0.737975	1.039325	0.128912
PHLPP2	1.078494	1.003747	1.158808	0.039206
STAU2	1.092709	1.015398	1.175907	0.017879
CTNS	1.143982	1.043739	1.253853	0.004041
RTN4R	0.880148	0.83279	0.930199	6.07E-06
PHF23	1.011381	0.919851	1.112017	0.81513
INPP4A	1.041121	0.980845	1.105101	0.185386
RAB27B	1.138145	1.084874	1.194032	1.22E-07
PSMA4	1.235711	1.102706	1.384759	0.00027
MYO16	1.068147	0.988045	1.154743	0.097406
LSG1	1.031815	0.954604	1.115272	0.429977
PARP3	1.29314	1.198582	1.395157	3.24E-11
TNC	1.044235	0.960034	1.135821	0.312923
THAP3	1.01427	0.923411	1.11407	0.767294
TDP1	1.012181	0.932424	1.098761	0.77248
AIFM2	1.316036	1.21444	1.426132	2.09E-11
SPATA7	0.972771	0.861724	1.09813	0.655327
MED17	0.969213	0.901811	1.041653	0.395151
RETSAT	1.026911	0.951418	1.108394	0.495474
CAPG	1.147933	1.094871	1.203567	1.11E-08
AP2S1	1.139595	1.039558	1.24926	0.005311
TG	1.282255	1.15332	1.425604	4.26E-06
ADAM28	0.927307	0.878058	0.979318	0.006718
DCUN1D1	1.01602	0.947483	1.089514	0.655586
LCP2	1.159056	1.060097	1.267253	0.001188
TRIT1	1.018837	0.948703	1.094157	0.608056

ADRB1	1.015696	0.967582	1.066203	0.529358
CUL7	1.045891	0.964518	1.134129	0.277588
CTNNA1	1.014284	0.94727	1.086039	0.684245
PHKA2	0.979674	0.91387	1.050215	0.562675
CNTLN	1.129047	1.045281	1.219525	0.002029
HSPA5	0.900344	0.84716	0.956868	0.000727
DSG2	1.147951	1.086755	1.212593	7.95E-07
GEMIN8	0.97754	0.889877	1.073838	0.635593
OFD1	1.078076	1.000962	1.161131	0.047104
GPM6B	0.946139	0.893853	1.001483	0.05628
PREX2	1.082805	1.003674	1.168176	0.03991
WDR37	0.971483	0.902561	1.045667	0.440953
YTHDC2	1.012602	0.938026	1.093107	0.748327
CTPS2	1.030789	0.955159	1.112408	0.435411
ATP6V1H	0.986593	0.904599	1.076018	0.760435
POLR2B	0.981216	0.91365	1.053778	0.602409
ARAP2	0.967613	0.918671	1.019163	0.213791
TPR	1.020888	0.944835	1.103062	0.600722
CP	0.973949	0.871942	1.08789	0.640054
DTNBP1	1.046169	0.95562	1.145299	0.328486
XK	1.037547	0.997811	1.078865	0.064319
SCML1	1.068976	1.012405	1.128708	0.016199
WWC3	0.97563	0.915008	1.040267	0.450966
ARHGAP6	1.158013	1.072781	1.250016	0.000169
FAM184B	0.935224	0.863327	1.013107	0.10082
MAP4	0.94263	0.870412	1.020842	0.146292
GOPC	1.123723	1.039289	1.215016	0.003423
USP28	0.962023	0.887068	1.04331	0.349526
HDAC9	1.081534	1.024425	1.141827	0.004629
TSPAN17	1.194165	1.115362	1.278535	3.50E-07
NOP16	1.171805	1.058405	1.297354	0.002266
CC2D2A	1.078828	1	1.163871	0.050001
RRM2B	1.058985	0.975618	1.149476	0.170709
ZNF800	0.946877	0.875072	1.024573	0.174899
TNFRSF17	1.066769	0.964293	1.180136	0.209721
SNX29	0.905199	0.836997	0.978959	0.012701
MRPS10	1.053507	0.958351	1.158112	0.280504
RSF1	0.944082	0.871556	1.022643	0.158261
VPS13D	1.003099	0.944247	1.065619	0.920104
FAM120A	1.037352	0.951659	1.130761	0.4045
R3HDM1	1.013182	0.912967	1.124397	0.805341
COL9A2	0.997112	0.939973	1.057724	0.923468
KITLG	1.23434	1.071582	1.421817	0.00352
ERCC8	1.019005	0.924168	1.123573	0.705637
ADAMTS6	1.128771	1.059169	1.202947	0.000191
H6PD	1.042584	0.983783	1.104898	0.159142
VAMP3	0.967037	0.880174	1.062471	0.485161
PER3	1.244572	1.145403	1.352327	2.41E-07
UTS2	1.097068	1.020787	1.17905	0.011752
TNFRSF9	1.067974	0.98958	1.152579	0.090898
LTBP1	1.049244	1.012894	1.086899	0.007537
RCN1	0.898273	0.828685	0.973705	0.009116
ELN	1.080427	0.991384	1.177467	0.077939
RFC2	1.276982	1.154774	1.412124	1.90E-06
ARID1B	0.943967	0.878962	1.013779	0.113186
CLPTM1L	1.194905	1.091926	1.307595	0.000108
NEDD4L	1.268548	1.16827	1.377433	1.50E-08
FOXP3	1.089395	0.994505	1.193338	0.065554

PPP1R3F	1.128163	1.008526	1.261992	0.034997
HEXB	1.031513	0.959111	1.10938	0.403383
PTCD2	1.12827	1.029176	1.236904	0.010078
DKK3	1.08152	0.947193	1.234897	0.24679
ARHGEF5	1.259853	1.14339	1.388179	3.05E-06
NFE2L3	0.99829	0.952881	1.045862	0.942546
LIMA1	1.151941	1.067404	1.243173	0.000275
LETMD1	0.937782	0.845859	1.039695	0.222311
SLC4A8	1.00462	0.915952	1.101872	0.92211
LAMC3	0.819983	0.749045	0.89764	1.72E-05
PTGER3	1.056594	0.97978	1.139431	0.152854
MAPK9	1.049585	0.952543	1.156514	0.328219
COL23A1	1.031467	0.991078	1.073503	0.128458
BCAR1	1.218175	1.020599	1.453999	0.028831
FAM160A2	1.015654	0.937713	1.100074	0.702985
HERPUD1	1.004986	0.932467	1.083146	0.896434
HOMER3	0.929501	0.883412	0.977994	0.004839
RAD51	1.138114	1.050327	1.233239	0.001584
POLQ	1.091953	1.025825	1.162345	0.005782
PIK3CB	1.002988	0.935972	1.074803	0.932602
CYBA	0.956419	0.88525	1.033309	0.258715
THOC3	1.241832	1.123343	1.372819	2.30E-05
HEBP2	1.07253	0.970414	1.185391	0.170173
MPHOSPH	1.054455	0.984102	1.129837	0.132307
PLEKHA5	0.857043	0.802621	0.915155	4.05E-06
PRSS8	0.995193	0.894329	1.107433	0.929578
RRP12	0.958299	0.901618	1.018542	0.170893
FNIP2	1.091495	1.027274	1.159731	0.004659
TTC17	1.004966	0.9337	1.081672	0.894982
ALX4	0.882982	0.790911	0.98577	0.026756
FOXN3	1.004995	0.918159	1.100043	0.913949
AKR7A2	1.10521	1.03023	1.185648	0.005257
MRTO4	1.143185	1.044957	1.250648	0.003508
NNAT	1.038982	0.981634	1.099681	0.186809
USE1	0.968139	0.858186	1.092179	0.59859
MCF2L2	1.159714	0.972433	1.383065	0.099171
NRIP2	1.042833	0.971629	1.119255	0.245099
LAMA3	1.296399	1.17641	1.428627	1.62E-07
ANAPC4	1.114761	1.021568	1.216456	0.014727
KCNQ1	1.114369	1.059153	1.172464	2.96E-05
TRAPPC3	1.243365	1.103831	1.400538	0.000335
THRAP3	1.028221	0.949669	1.11327	0.492491
PHPT1	1.141144	1.045637	1.245373	0.00307
ENTPD2	1.196375	1.031429	1.387699	0.017846
LY75	0.977673	0.908907	1.051641	0.543974
ARID4B	0.97252	0.902701	1.047739	0.463509
OPN3	1.189411	1.08277	1.306556	0.000296
SDCCAG8	0.911987	0.848072	0.98072	0.012952
HHAT	1.07285	0.997799	1.153545	0.057378
KIF1B	1.086321	1.02011	1.15683	0.009865
FOXC1	1.029294	0.995785	1.06393	0.087299
TBC1D22A	0.986243	0.904315	1.075594	0.754235
SYNE2	0.983975	0.935468	1.034997	0.531108
PLEKHH1	1.094759	0.986789	1.214542	0.087463
ATP9A	1.093114	1.042991	1.145646	0.000201
FAM168A	1.027952	0.950145	1.112132	0.492402
RELT	0.996353	0.94105	1.054907	0.900216
GALC	0.970963	0.908982	1.037171	0.38128

NOP58	0.975455	0.899688	1.057601	0.546898
KCNH2	1.000566	0.960873	1.0419	0.978125
CUL1	0.921556	0.858663	0.989054	0.023504
FAM114A2	1.163922	1.064579	1.272537	0.000854
CYFIP2	1.023349	0.967495	1.082428	0.420238
EIF2AK2	0.983771	0.919586	1.052435	0.634552
USP36	0.921844	0.862745	0.98499	0.016069
PUM2	0.971753	0.914842	1.032204	0.352075
MRPL43	1.131109	1.012246	1.263931	0.029643
ITIH4	1.254047	1.041396	1.51012	0.016949
ZFR	0.972981	0.891298	1.062149	0.540372
ZNF280C	0.983909	0.898358	1.077608	0.726703
TRAF1	1.004882	0.950236	1.062671	0.864451
RC3H2	0.97514	0.906861	1.04856	0.496701
IL17RB	1.014432	0.938075	1.097004	0.719686
TRAF3IP2	0.861719	0.794027	0.935183	0.000363
GYG2	1.085838	1.01246	1.164534	0.021064
DCBLD2	0.889619	0.816796	0.968934	0.00727
SOAT1	0.952357	0.886873	1.022676	0.179256
PKP2	1.145105	1.078778	1.215509	8.55E-06
MSH4	1.269163	1.104149	1.458838	0.000796
GDI2	0.901791	0.832415	0.976949	0.011376
PRDM1	1.042108	0.979844	1.108328	0.189459
ATG5	0.953049	0.864183	1.051055	0.335595
TMCC3	0.988102	0.930698	1.049046	0.695071
MTA3	1.152251	1.06302	1.248972	0.000569
USP13	0.913443	0.850587	0.980944	0.012814
ATP11B	0.941798	0.889378	0.997308	0.040147
SEC61A1	0.984766	0.90506	1.071492	0.721491
PPP1R12A	0.968833	0.895148	1.048582	0.432722
CROCC	1.113021	1.034574	1.197417	0.004086
POLR3E	1.047382	0.973151	1.127275	0.217087
ATP2B4	1.165007	1.101036	1.232696	1.16E-07
ZC3H11A	0.931	0.819288	1.057945	0.272962
RIOK2	1.046287	0.94175	1.162429	0.39951
YIPF1	1.169228	1.042858	1.31091	0.007383
DGKG	0.949107	0.903208	0.997338	0.03889
FLYWCH1	1.028309	0.945245	1.118673	0.515948
UNKL	0.998661	0.936157	1.065338	0.967593
TBXAS1	0.965503	0.904067	1.031113	0.295299
PARP12	0.948004	0.887164	1.013015	0.114601
ALDH18A1	1.068572	1.007794	1.133015	0.026431
TARBP1	1.034762	0.976632	1.096353	0.246699
MXD1	0.963954	0.900034	1.032413	0.294304
DNAJC25	0.886186	0.815259	0.963284	0.004528
SLC2A3	0.903901	0.860804	0.949155	5.04E-05
PSD	1.03797	0.977521	1.102157	0.223486
CTDP1	0.931805	0.878779	0.98803	0.018137
STYK1	1.04988	0.962731	1.144919	0.270929
WNK1	1.071062	0.998642	1.148733	0.054615
CCAR1	0.991295	0.899257	1.092753	0.860404
OGFR	1.082962	0.994128	1.179734	0.067985
GNA15	0.921968	0.874589	0.971914	0.002542
CREB3L3	1.1789	1.063156	1.307245	0.001799
PIGV	1.024327	0.950354	1.104057	0.529684
PTPRU	1.109521	1.013878	1.214187	0.023845
SNRNP40	1.173662	1.070805	1.286399	0.000622
COL11A1	1.214944	1.122514	1.314986	1.42E-06

QSER1	0.967033	0.901792	1.036993	0.346881
ACAA1	0.898898	0.830566	0.972853	0.008236
BCAT1	0.970372	0.929684	1.01284	0.168767
HDAC7	0.972142	0.894894	1.056058	0.503609
LZTS1	1.014448	0.906142	1.135698	0.803349
SPAG4	0.982352	0.911625	1.058566	0.640463
NCKAP1	1.164033	1.066533	1.270445	0.000666
MRPS35	1.00745	0.914475	1.109878	0.880572
TNK2	0.936321	0.871297	1.006197	0.073175
MON2	1.009932	0.946845	1.077223	0.763939
GPBP1	0.929981	0.861358	1.004071	0.06344
DGAT2	1.114533	1.039838	1.194593	0.002186
CS	0.905356	0.812152	1.009257	0.072858
LTK	0.980237	0.94149	1.020579	0.332036
MRPS24	1.1523	1.023565	1.297225	0.019011
ELMO2	0.950071	0.877259	1.028926	0.208024
APPBP2	1.027549	0.946442	1.115606	0.517107
POLD1	1.000053	0.934884	1.069764	0.998775
SEZ6	0.996436	0.935763	1.061042	0.911299
EIF4B	0.917833	0.845308	0.99658	0.041196
SLC6A16	1.152213	1.063149	1.248737	0.000557
SPHK2	1.01665	0.924295	1.118233	0.73398
RPL18	0.857549	0.786015	0.935592	0.000544
CA11	1.080355	0.98446	1.185592	0.103163
ISOC2	1.103645	1.029271	1.183392	0.005597
U2AF2	0.964831	0.882054	1.055376	0.43404
EPN1	0.952426	0.864469	1.049332	0.324162
MED29	0.971336	0.885333	1.065695	0.538665
ZNF275	1.035111	0.961232	1.114668	0.361027
MTMR1	1.047506	0.971028	1.130007	0.230183
GPC1	1.110671	1.033608	1.19348	0.004224
ADCK1	1.044026	0.962031	1.13301	0.301879
HAGH	1.086929	1.005393	1.175077	0.036157
RNF4	0.993072	0.909901	1.083845	0.876196
CASP8	0.894646	0.834093	0.959594	0.001849
TM7SF3	0.96197	0.911282	1.015477	0.160355
DLX3	1.173896	1.093752	1.259913	8.84E-06
SPA17	1.645128	1.348993	2.006271	8.82E-07
TSPAN32	1.183525	1.128394	1.24135	4.42E-12
ST3GAL6	0.812252	0.768127	0.858913	2.95E-13
ATP2C2	1.058026	0.984652	1.136867	0.124008
NGFR	1.142795	1.013562	1.288505	0.029259
TAF2	1.030793	0.95361	1.114223	0.445004
HIPK2	1.038175	0.971574	1.109342	0.268082
TNPO3	0.993308	0.925739	1.065808	0.851804
RFXANK	1.063428	0.941324	1.20137	0.323026
TMEM161/	1.068806	0.983861	1.161084	0.115284
LPAR2	0.904457	0.838533	0.975563	0.009304
CTSA	1.016722	0.944289	1.094712	0.660082
SLC12A2	0.897946	0.844297	0.955003	0.000615
SNX24	1.284427	1.179155	1.399099	9.63E-09
CNN2	0.880904	0.820918	0.945273	0.000425
ABCA7	1.158561	1.094672	1.226179	3.67E-07
DDX20	0.996142	0.909229	1.091364	0.933869
BTBD1	0.97	0.889901	1.057308	0.488506
FAR2	1.072797	1.008455	1.141245	0.025964
POU1F1	1.189189	1.074034	1.316692	0.000855
CHI3L2	1.149985	1.063093	1.24398	0.00049

SBNO2	1.04038	0.970483	1.115311	0.264594
PMS1	1.082039	1.001335	1.169249	0.046186
HMG20B	0.890658	0.802853	0.988065	0.028765
CALCRL	1.165366	1.096356	1.23872	8.94E-07
TAF11	0.974168	0.882274	1.075633	0.60466
ANKS1A	0.931701	0.874195	0.99299	0.029527
AP3D1	1.045788	0.956013	1.143994	0.328246
ZNF76	1.034033	0.945318	1.131073	0.464624
SLC9A3R2	0.871467	0.827557	0.917707	1.83E-07
NTHL1	0.995469	0.9283	1.067498	0.898611
UHRF1BP1	0.941298	0.878244	1.008878	0.087245
GNAI3	1.032231	0.940234	1.133228	0.505383
IPO5	0.912485	0.843585	0.987013	0.022237
OAT	1.07243	1.012365	1.136058	0.017413
WDR3	1.135553	1.049078	1.229157	0.001658
PKN2	0.962889	0.897496	1.033046	0.29192
WDR18	1.088499	1.005298	1.178586	0.036598
TRAM2	1.116835	1.041271	1.197882	0.001992
NTN1	0.912456	0.860365	0.967702	0.002253
MCM10	1.145012	1.071162	1.223955	6.87E-05
DGKA	1.016787	0.944245	1.094901	0.659338
ERBB3	1.115321	0.983944	1.26424	0.087852
ANKRD44	0.994435	0.941598	1.050236	0.841205
ADAT1	1.069937	0.98437	1.162942	0.111938
PDIA5	1.150329	1.070621	1.235971	0.000132
TBC1D22B	1.050775	0.972896	1.134889	0.207454
NDUFB4	0.994697	0.901645	1.097352	0.915496
SPEN	0.907918	0.838463	0.983127	0.017357
MYLK	1.236206	1.140096	1.340419	2.82E-07
ZC3H15	0.911468	0.840415	0.988528	0.025182
MAP2K4	1.001603	0.92868	1.080251	0.966882
SLK	0.938055	0.868803	1.012826	0.102204
CYB5R4	0.901214	0.833523	0.974402	0.009031
COL17A1	0.941705	0.884121	1.003041	0.062089
GSTO2	1.01955	0.955307	1.088114	0.55985
SEC61A2	0.946932	0.875156	1.024595	0.175159
PRKCQ	1.014798	0.959409	1.073385	0.607976
TLE2	1.051798	0.939797	1.177146	0.379347
ASB1	1.092504	1.00678	1.185526	0.033834
FAM107B	0.973862	0.921335	1.029383	0.34914
ME1	0.925247	0.867225	0.98715	0.018703
TBC1D1	0.945829	0.87018	1.028054	0.190381
MTHFD2	1.099622	1.012071	1.194747	0.02487
SLC9A7	0.973606	0.917038	1.033664	0.381125
FOXJ2	0.941733	0.854402	1.037989	0.226642
YBX1	0.909489	0.830735	0.99571	0.040073
PDE4A	0.964519	0.910887	1.021309	0.215864
PPP2R5A	1.005086	0.919205	1.098992	0.911357
ELAVL1	1.019944	0.929159	1.119599	0.678012
TIE1	0.97062	0.923805	1.019808	0.237087
DIP2B	0.975783	0.910936	1.045247	0.484736
SMARCD1	0.950974	0.8716	1.037576	0.258289
NFYC	1.029546	0.921029	1.15085	0.608383
ZMYND12	1.168893	1.026909	1.330507	0.018185
SLC9A3	0.91077	0.812892	1.020433	0.107122
NGEF	0.950748	0.83633	1.08082	0.440119
ASPM	1.104702	1.042804	1.170274	0.000713
CD84	1.050038	0.995216	1.107881	0.074313

ELOVL1	1.009922	0.909097	1.121929	0.854025
SPI1	0.88121	0.822866	0.943691	0.000297
MPPED2	1.249068	1.160736	1.344122	2.80E-09
CLDN18	1.197912	1.065059	1.347337	0.002605
ZBTB11	0.923509	0.864043	0.987067	0.019114
ATXN3	0.967138	0.893537	1.046802	0.40802
GOLGA5	0.998352	0.911264	1.093764	0.971754
LRRC40	0.997553	0.923078	1.078038	0.950662
ISOC1	1.063568	0.98732	1.145704	0.10443
TRMT11	1.083906	0.998838	1.176219	0.053349
THUMPD1	0.918701	0.864257	0.976574	0.006519
KIF26A	0.931719	0.879368	0.987187	0.016528
ATG2B	1.023987	0.958369	1.094097	0.482981
ARFGEF1	0.943472	0.892448	0.997412	0.040236
ZFAT	0.995447	0.92411	1.07229	0.904253
MTFR1	0.995473	0.919881	1.077276	0.910337
STAG3	0.977075	0.906303	1.053374	0.545494
FECH	1.045188	0.993053	1.100059	0.090469
MYO9A	0.971005	0.905337	1.041437	0.4102
DDX3Y	1.008798	0.987316	1.030747	0.425099
PFKP	1.0313	0.967726	1.099051	0.342424
IDI1	0.958498	0.88088	1.042955	0.325208
SP100	1.057937	0.962989	1.162247	0.240433
KLF6	0.906009	0.849582	0.966184	0.002626
NEO1	1.108509	1.068996	1.149482	2.65E-08
TRAM1	0.979843	0.902194	1.064175	0.628809
PHKA1	1.216656	1.126744	1.313743	5.55E-07
TNFRSF1A	0.937784	0.868318	1.012806	0.101861
CACNB1	1.083008	1.010775	1.160403	0.023557
EVI5	1.001429	0.939559	1.067372	0.964996
STOML1	1.342789	1.161986	1.551724	6.48E-05
DHX29	1.036649	0.95065	1.130429	0.415302
DNTTIP2	0.991871	0.906187	1.085657	0.85945
TP53BP1	0.932414	0.876614	0.991765	0.026243
TRO	1.124349	1.065153	1.186835	2.16E-05
RRP15	1.114951	1.01384	1.226146	0.024875
RHOA	0.896475	0.807603	0.995126	0.0402
DHX8	1.051749	0.961403	1.150585	0.270887
PRKCZ	1.276559	1.181603	1.379145	5.97E-10
ZFY	1.014868	0.98349	1.047248	0.35703
IARS2	1.116616	1.031122	1.209198	0.006646
NAV3	0.943525	0.881293	1.010151	0.094947
IDH3G	1.079265	0.955766	1.218721	0.218593
ROGDI	1.13076	1.037927	1.231896	0.004929
PDZD4	1.084833	0.979919	1.20098	0.116633
ROCK1	0.967544	0.899087	1.041212	0.378167
CBFB	1.14349	1.050209	1.245056	0.002013
PDK3	0.996908	0.924522	1.074962	0.935825
HYAL2	0.858627	0.793247	0.929397	0.000162
HDAC4	0.985589	0.929524	1.045036	0.627135
RASSF1	0.862174	0.796504	0.933259	0.000244
FGFR3	0.955009	0.886064	1.029318	0.228541
IFI35	1.063313	0.975271	1.159303	0.163884
HEATR6	1.278328	1.169557	1.397215	6.23E-08
COASY	1.040794	0.934581	1.159077	0.466591
PLEKHH3	0.932053	0.876136	0.991539	0.025804
MEF2A	0.884616	0.825545	0.947915	0.000507
OTUD5	0.934039	0.846788	1.03028	0.172636

TFE3	0.863768	0.791957	0.942091	0.000943
TBC1D25	0.903125	0.818986	0.995908	0.041138
ACSL4	0.985244	0.915609	1.060175	0.690996
INPP5A	1.057023	0.985379	1.133875	0.121465
GPKOW	1.023612	0.91521	1.144853	0.682816
GRIPAP1	0.951715	0.870776	1.040176	0.275121
FTSJ1	1.066592	0.946823	1.201512	0.288773
PRR11	1.077382	0.991256	1.170992	0.07954
ATP11A	0.918137	0.863371	0.976377	0.006492
POLR1A	1.019656	0.953338	1.090587	0.570511
LAPTM4A	0.942651	0.869839	1.021558	0.149883
TTC7A	0.893376	0.837201	0.95332	0.000667
IP6K2	0.970357	0.895851	1.05106	0.460372
SRBD1	0.88979	0.831188	0.952524	0.000782
KIF2A	0.96441	0.895598	1.038508	0.337299
RASGRP2	1.104132	1.023157	1.191516	0.010802
PSME4	0.940297	0.890066	0.993362	0.027968
IFT80	1.134979	1.045431	1.232199	0.002532
SIRT2	1.078944	0.966101	1.204967	0.177629
PPP2R5B	1.041293	0.976459	1.110431	0.217333
PYGM	0.998254	0.946029	1.053362	0.949172
PITX1	1.150324	1.089953	1.214039	3.55E-07
MAST4	1.036742	0.973086	1.104562	0.264392
SDK2	1.019091	0.964975	1.076243	0.496954
NUP133	1.014903	0.943476	1.091737	0.691144
NUCKS1	1.113583	1.030076	1.203861	0.00683
VPS35	1.003413	0.928307	1.084596	0.931597
DNAJA2	0.956723	0.876445	1.044354	0.322462
BCL3	0.982342	0.921267	1.047467	0.586464
KCNAB2	1.008664	0.939675	1.082719	0.811368
GAL	1.103704	1.015812	1.199202	0.01978
CLEC2D	1.055797	0.997265	1.117764	0.062065
FUNDC1	1.060068	0.953742	1.178247	0.279386
RORA	1.026134	0.964534	1.091668	0.414079
DRD4	0.979347	0.907576	1.056794	0.590975
TGFBR3	1.065869	0.987796	1.150112	0.100261
PLA2G10	0.958809	0.878525	1.046429	0.345797
ATP1B3	0.977531	0.896761	1.065575	0.605517
NEDD4	0.966013	0.91098	1.024371	0.24793
PIGB	1.033478	0.952473	1.121372	0.429104
MAPK6	0.998099	0.923266	1.078998	0.961838
GNB5	1.175896	1.089517	1.269124	3.15E-05
RAB27A	0.892275	0.838258	0.949772	0.000347
LRP6	0.903803	0.855779	0.954522	0.000283
SCT	0.971452	0.914646	1.031786	0.346127
PHRF1	0.983043	0.91801	1.052684	0.624322
NUCB2	0.979494	0.91323	1.050565	0.56209
PFN2	1.207793	1.084165	1.345518	0.000611
SPTB	1.011831	0.975098	1.049946	0.533035
DAPP1	1.033837	0.967659	1.104542	0.324163
SLC44A1	1.209144	1.151556	1.269612	2.39E-14
SMG6	0.980514	0.914068	1.051789	0.582565
EXOC5	0.996554	0.914656	1.085787	0.937125
CLTCL1	1.08983	1.044319	1.137324	7.73E-05
FGF22	0.926059	0.765465	1.120346	0.429221
FSTL3	0.907727	0.855529	0.96311	0.001356
DGCR2	1.033285	0.965801	1.105485	0.342024
RNF126	0.859442	0.769947	0.959339	0.006937

MNT	0.965756	0.881463	1.05811	0.4546
ZXDC	0.970515	0.901947	1.044296	0.423383
JMJD6	1.026326	0.938871	1.121928	0.567421
POLB	0.880489	0.812421	0.95426	0.001932
ST6GALNA	1.08797	1.027043	1.15251	0.004137
WIPI1	0.979073	0.919839	1.042121	0.506554
GBA2	1.062088	0.980913	1.14998	0.13757
NDST1	0.916079	0.859768	0.976078	0.006769
ASNS	0.990593	0.861142	1.139502	0.894759
AP3M2	1.036681	0.958722	1.120979	0.366446
ST6GALNA	0.974702	0.882857	1.076102	0.611845
PABPC1	0.886443	0.808645	0.971726	0.010113
TESK2	1.009449	0.914533	1.114216	0.851922
CSNK2A2	0.918631	0.84404	0.999813	0.049496
EIF2B3	1.186743	1.099926	1.280413	1.00E-05
CAMK2A	0.999226	0.947948	1.053277	0.977012
TCOF1	1.018073	0.96193	1.077493	0.535989
CDC42	0.889771	0.815729	0.970534	0.008422
OSBPL3	1.026426	0.967928	1.088459	0.383661
SLC12A3	1.221544	1.102381	1.353589	0.000133
RAD18	1.018864	0.938385	1.106246	0.656211
ATP2B1	0.915179	0.860532	0.973295	0.004778
TRPM5	0.961174	0.88211	1.047325	0.365899
NCK2	0.952141	0.884822	1.024583	0.189911
MAP4K4	0.983918	0.905959	1.068586	0.700284
MGAT4A	0.992698	0.923006	1.067653	0.843575
RPL31	0.895567	0.831969	0.964027	0.003338
WDR1	0.993576	0.910154	1.084643	0.885467
SNX13	0.960843	0.896505	1.029798	0.258647
ARHGAP1(	1.070892	1.013806	1.131193	0.014265
RPS6KA2	0.825434	0.777934	0.875834	2.23E-10
ING3	0.980204	0.904181	1.062619	0.627384
VASH1	1.075689	1.005316	1.150987	0.034553
SEL1L	1.02281	0.953755	1.096864	0.527143
TRIP13	1.159178	1.077248	1.247339	7.83E-05
ATP6AP1	1.171643	1.058012	1.297479	0.002339
TCF3	0.967665	0.892826	1.048777	0.423513
TRIB2	0.979591	0.92922	1.032692	0.443926
DAZAP1	0.96741	0.877782	1.06619	0.504181
MBD3	1.057518	0.971924	1.150651	0.194058
HLTF	1.101864	1.031463	1.17707	0.003982
FAM50A	1.253727	1.125517	1.396541	3.99E-05
FAM3A	1.06313	0.945862	1.194936	0.304611
CPSF1	1.03781	0.964825	1.116316	0.318515
CYBRD1	0.961752	0.90685	1.019978	0.193466
PDCD2	1.098914	0.982142	1.22957	0.099849
RDH11	1.01093	0.919667	1.11125	0.821828
PRKACA	0.93902	0.849082	1.038485	0.220637
ACTN1	1.078861	1.014997	1.146744	0.014766
ZFYVE26	1.004093	0.937716	1.075168	0.906824
EPN2	0.920994	0.85253	0.994957	0.036775
PTPN18	0.999437	0.934412	1.068987	0.986903
LIMS2	1.204035	1.061217	1.366074	0.003948
LNX1	1.15657	1.029797	1.298949	0.014063
ALDH3A2	1.10864	1.036713	1.185556	0.002583
TFRC	1.038124	0.982595	1.096792	0.182214
SREBF1	0.99528	0.927284	1.068261	0.895734
TRPC5	0.933089	0.830277	1.048633	0.24495

AFF4	0.903125	0.848575	0.961182	0.001348
UBE2D1	1.00455	0.935897	1.07824	0.899971
MPP5	0.959512	0.883404	1.042177	0.326984
RHOBTB1	0.996094	0.93976	1.055805	0.895176
SMC1A	1.094093	1.009378	1.185918	0.028744
HSD17B10	1.244989	1.133859	1.367011	4.36E-06
MARK2	0.959817	0.883127	1.043166	0.334393
HMMR	1.058596	0.987208	1.135147	0.10992
CHFR	0.993956	0.914389	1.080447	0.886756
P4HA2	1.047459	0.948216	1.157088	0.361256
FCGR2B	1.032425	0.967496	1.101712	0.335612
NFATC3	1.00152	0.933046	1.07502	0.966462
TRNT1	0.945959	0.878118	1.01904	0.14341
ACADVL	1.032196	0.947938	1.123943	0.465779
STK10	1.153231	1.065876	1.247747	0.000389
FBXW11	0.956933	0.890481	1.028344	0.230597
ACAP1	1.00299	0.928522	1.08343	0.939542
CRMP1	0.88178	0.780137	0.996666	0.044072
EVC	0.923095	0.856833	0.99448	0.035239
DERL2	1.110903	1.00198	1.231667	0.045767
SIDT1	0.954604	0.881758	1.033468	0.251331
NDE1	0.973807	0.895102	1.059432	0.537047
TMEM38A	1.017573	0.952319	1.087299	0.606435
AP1M1	1.11259	1.010646	1.224817	0.02956
PVR	1.168808	1.098913	1.243149	7.12E-07
XRCC1	0.89848	0.801216	1.007551	0.06706
SCARB1	0.955879	0.896434	1.019266	0.16837
CYP2W1	1.174965	1.055476	1.307982	0.003212
MCM2	1.046301	0.983276	1.113365	0.153327
PANX2	0.981093	0.933676	1.030919	0.450135
TP63	1.107868	1.023707	1.198949	0.011047
ALPK1	1.018259	0.953444	1.08748	0.589733
LLGL2	1.081603	0.998004	1.172205	0.055969
PDE8A	0.889045	0.822919	0.960485	0.002861
CLCN4	1.098778	1.030559	1.171512	0.003972
NLE1	1.016948	0.928192	1.11419	0.718336
SDHA	1.068088	0.991345	1.150773	0.08337
SMARCE1	1.104243	1.017592	1.198272	0.017397
GSDMB	1.019408	0.950387	1.093441	0.59101
KDM5A	0.990863	0.929018	1.056825	0.780133
ADAM11	1.161972	1.065003	1.267771	0.000734
PPP2R3A	1.085012	0.998769	1.178702	0.053507
FERMT2	0.869821	0.760367	0.995031	0.042097
DHRS9	1.064434	1.013599	1.117819	0.012386
CD5L	1.029594	0.957872	1.106687	0.428559
PTGS2	1.044465	0.999779	1.091148	0.051167
IGF2BP2	0.962314	0.914401	1.012738	0.140422
MAP3K13	1.180232	1.071625	1.299845	0.000767
ST6GAL1	1.002982	0.942478	1.067371	0.925267
TBX21	1.041113	0.968739	1.118894	0.27308
FRY	0.92215	0.875934	0.970804	0.002005
PICALM	0.967035	0.897748	1.041668	0.376848
NSF	1.124573	1.044498	1.210787	0.001839
CLASP1	0.990921	0.930362	1.055422	0.776812
MRPS34	1.098514	1.012938	1.19132	0.02317
NOTCH3	0.916458	0.857447	0.97953	0.010199
CLNS1A	1.027788	0.935419	1.129276	0.568365
TEAD2	1.209324	1.129308	1.295009	5.28E-08

EED	0.973213	0.898367	1.054294	0.506032
TSG101	1.060841	0.947027	1.188332	0.307729
ATP2A3	0.958985	0.899066	1.022898	0.203294
MGLL	1.126417	1.066437	1.189769	2.01E-05
BCS1L	1.254379	1.156257	1.360828	4.94E-08
NUAK1	1.218739	1.114196	1.33309	1.54E-05
DPP8	1.035668	0.953451	1.124974	0.406283
SLC24A1	0.998846	0.93206	1.070418	0.973917
ZNF532	1.165935	1.115904	1.218209	6.85E-12
SCARF1	1.053905	0.998162	1.112761	0.058276
LMAN1	0.938887	0.871957	1.010953	0.094672
ZZEF1	0.960449	0.89924	1.025825	0.22972
ENO1	0.98528	0.908991	1.067972	0.718363
ANO8	1.120825	1.055886	1.189759	0.00018
TUBE1	1.299013	1.167036	1.445916	1.70E-06
ARHGEF1(	1.094551	1.044538	1.14696	0.000153
TXK	1.071997	1.015688	1.131428	0.011557
TACR2	1.043981	0.954471	1.141885	0.346653
ACTR6	1.099017	0.992078	1.217484	0.070656
TIPIN	1.124842	1.009727	1.253081	0.032705
SRI	1.194679	1.080381	1.321069	0.000527
EIF4G3	1.001434	0.921083	1.088794	0.973208
NUP37	1.182139	1.067547	1.309031	0.001298
SEMA3A	1.241633	1.126824	1.36814	1.23E-05
GTSE1	1.212095	1.128724	1.301624	1.22E-07
SEMA3C	1.141399	1.056998	1.232539	0.00074
TTC38	1.182713	1.101196	1.270264	4.11E-06
ACAT1	1.124941	1.031092	1.227332	0.008076
GRAMD4	1.010229	0.95386	1.069931	0.728277
CELSR1	1.047419	0.978941	1.120688	0.179276
WNT8B	1.106561	1.014029	1.207536	0.023048
ZNF638	1.008897	0.948108	1.073583	0.779966
SLC25A40	0.955718	0.890727	1.02545	0.207474
ADD2	1.047896	1.000684	1.097335	0.046698
RASAL2	1.097029	1.022546	1.176937	0.009838
ZNF37A	1.169918	1.069251	1.280062	0.00063
MARK3	1.004445	0.933724	1.080523	0.905227
SLC25A3	0.897058	0.811436	0.991714	0.033793
FNDC3B	0.880503	0.835255	0.928202	2.27E-06
FOSL2	0.943563	0.889761	1.000618	0.052459
CACNG4	1.096829	1.022351	1.176734	0.009993
FRYL	0.997906	0.940403	1.058924	0.944809
TMEM131	0.990153	0.913738	1.072958	0.809168
FSCN1	0.911578	0.875128	0.949546	8.73E-06
ACTB	1.051657	0.972451	1.137314	0.20741
PLD1	0.849012	0.800121	0.90089	6.34E-08
WDR62	1.102455	1.020813	1.190627	0.012966
DLG1	1.05747	0.969364	1.153583	0.208049
RAB7A	1.037031	0.914534	1.175936	0.570748
BCAP29	0.999507	0.873171	1.144122	0.994294
SEC31B	1.069194	0.999245	1.144039	0.052614
SART3	1.007655	0.933412	1.087803	0.845163
ARHGAP1!	0.993414	0.923693	1.068398	0.858738
TUBA3D	0.991966	0.911547	1.07948	0.851663
EXOSC7	1.039817	0.946271	1.142612	0.416924
KIFAP3	1.004169	0.939878	1.072859	0.901911
MKRN2	0.954965	0.875667	1.041444	0.29748
MCM6	1.10656	1.031304	1.187307	0.004837

REXO2	1.121116	1.04363	1.204354	0.001756
RBM7	0.936095	0.857871	1.021452	0.138011
RBMS2	0.928126	0.870474	0.989597	0.022633
BAZ2A	0.924843	0.865905	0.987792	0.020042
PTPN23	0.97866	0.890036	1.076108	0.656025
MLH1	1.064417	0.967722	1.170772	0.198885
UNG	1.067183	1.006317	1.13173	0.029997
FMO4	1.157537	1.05958	1.26455	0.001184
KLHL20	1.061984	0.974965	1.15677	0.167981
SLC46A1	1.510162	1.339518	1.702546	1.61E-11
PLXNA2	1.157068	1.066002	1.255914	0.000486
SPAG5	1.058344	0.987948	1.133757	0.106375
ANKRD13/	1.090366	0.98468	1.207396	0.096279
TPD52	1.142047	1.062432	1.227627	0.000315
ACACB	1.029972	0.966261	1.097884	0.364688
TRAF4	0.975472	0.918852	1.035581	0.415647
PAG1	0.937409	0.886323	0.99144	0.023781
GPATCH1	1.130636	1.033429	1.236986	0.007431
ICAM3	0.977758	0.903895	1.057656	0.574616
NT5C2	0.920747	0.855026	0.991519	0.028861
MCAM	0.842871	0.793389	0.895438	3.06E-08
GPC4	1.023669	0.970318	1.079953	0.391665
MBNL3	0.995522	0.934265	1.060796	0.889844
RAP1GAP	1.018582	0.97171	1.067715	0.443673
XAB2	0.93276	0.856731	1.015536	0.108585
ARHGEF1	1.069462	0.98319	1.163306	0.117605
STXBP2	0.943322	0.861485	1.032934	0.207618
MAP2K7	0.968369	0.889113	1.054691	0.460665
DGKD	0.969342	0.906063	1.037041	0.365995
RARB	1.052511	0.976263	1.134714	0.182252
TOP2B	1.014721	0.947542	1.086663	0.675839
TM9SF3	0.951415	0.888137	1.019201	0.156093
NFKB2	0.999915	0.946024	1.056876	0.9976
UBE2T	1.186291	1.104465	1.274179	2.80E-06
PPP1R12B	1.070927	0.999123	1.147892	0.052966
DNAJC10	1.172	1.075542	1.277108	0.000292
GTF3C1	0.98738	0.92066	1.058935	0.721997
IL4R	0.964171	0.908959	1.022737	0.225235
USP33	1.0145	0.946816	1.087021	0.682806
SNRPA	1.02223	0.922097	1.133236	0.675944
SPAG6	1.149374	1.109283	1.190913	1.52E-14
EXOSC5	1.084596	1.012596	1.161716	0.020497
DYNC1I2	1.001763	0.918901	1.092097	0.968105
APBB1IP	0.888615	0.83092	0.950315	0.000565
LRCH4	0.917727	0.846952	0.994415	0.036017
FAM76B	0.991739	0.914804	1.075145	0.840439
SIRT6	1.185029	1.048007	1.339966	0.006771
POLD3	1.134303	1.046373	1.229621	0.002206
CAPZB	1.01515	0.911358	1.130762	0.784669
GPR137B	1.051995	0.977049	1.13269	0.178877
NAALAD2	1.04676	0.940313	1.165256	0.4036
SLC25A43	0.984047	0.9154	1.057841	0.662919
UBE2A	0.885964	0.804425	0.975768	0.013974
FGFR1	0.855114	0.81237	0.900107	2.20E-09
SMC1B	1.037011	0.949147	1.133009	0.421075
FBLN1	0.898569	0.812724	0.993481	0.036831
CST7	0.926373	0.891521	0.962588	9.28E-05
PIAS2	0.966836	0.894262	1.0453	0.396919

AMPH	1.029168	0.932531	1.13582	0.567666
ARAF	0.949527	0.850836	1.059665	0.354987
MCCC1	1.018786	0.938353	1.106113	0.657365
LAMP3	1.304127	1.154274	1.473434	2.01E-05
ACER3	1.028065	0.935405	1.129904	0.565732
UBE2K	1.021193	0.929793	1.121579	0.661118
PIK3C3	1.045515	0.970245	1.126624	0.242979
N4BP2	1.015598	0.946349	1.089915	0.66752
TULP3	1.24172	1.11049	1.388457	0.000145
SYNJ2	1.11067	1.038622	1.187716	0.00216
PPP2R5C	0.99186	0.917542	1.072198	0.837047
GNB1	0.969391	0.883792	1.063281	0.509842
HOXA9	1.129473	1.099922	1.159818	2.24E-19
EDN1	1.190847	1.10622	1.281948	3.42E-06
MLLT10	0.948558	0.879376	1.023182	0.171677
ZCWPW1	0.937407	0.853717	1.029302	0.17552
P2RY10	0.937222	0.88438	0.993222	0.02855
ITM2A	0.907811	0.877108	0.939589	3.60E-08
VDAC3	1.054805	0.962907	1.155474	0.251285
PCM1	0.977463	0.918271	1.04047	0.474484
TNRC6C	1.046768	0.974934	1.123896	0.207631
CBFA2T2	1.041224	0.964934	1.123545	0.298097
ITCH	0.948312	0.887027	1.013832	0.119484
PKD2L2	0.986557	0.924915	1.052306	0.680957
TP53INP2	0.935328	0.885406	0.988064	0.016892
SDF4	0.995348	0.908505	1.090492	0.920253
MYH7B	1.031201	0.958018	1.109974	0.413339
TP73	1.270378	1.172353	1.376599	5.19E-09
TOLLIP	1.052366	0.962908	1.150135	0.260131
UBE2D4	1.125972	1.023064	1.239232	0.015256
RUNX1T1	0.93366	0.89236	0.976871	0.002942
THOC1	0.984258	0.92206	1.050653	0.633788
FKBP7	1.109809	1.000001	1.231675	0.049998
OSBPL6	1.055146	0.963282	1.155772	0.248081
SLC1A3	0.97966	0.917558	1.045965	0.538552
XRCC5	1.021796	0.938871	1.112046	0.61756
LXN	1.029899	0.959823	1.105092	0.412544
SP140	0.991878	0.935452	1.051708	0.784935
MKNK1	1.104683	0.998516	1.222138	0.053464
TNS1	1.016452	0.971387	1.063608	0.480639
REXO1	0.87677	0.812013	0.94669	0.000781
SAR1A	0.976258	0.906098	1.051851	0.527734
CDC14A	0.976514	0.91504	1.042118	0.473754
RAPGEF3	1.008987	0.927172	1.098022	0.835722
CEACAM1	1.046336	0.999298	1.095589	0.053602
SENP1	1.072034	0.978369	1.174667	0.135922
DUSP13	1.026455	0.954182	1.104202	0.48334
CIC	0.877215	0.814704	0.944522	0.000514
LIPE	1.215044	1.118622	1.319778	3.89E-06
FDFT1	1.016564	0.924437	1.117872	0.73465
PAFAH1B3	1.10803	1.015886	1.208531	0.020571
OPHN1	1.124284	1.049802	1.20405	0.000809
KIF22	1.183723	1.077624	1.300268	0.000431
PGM1	1.103608	1.031243	1.181051	0.004385
DDX1	1.111332	1.014276	1.217675	0.023576
DNM2	0.95773	0.87389	1.049614	0.355483
EPB41L2	1.032291	0.983653	1.083335	0.196836
STX7	0.990393	0.924849	1.060581	0.782289

RABL2B	1.149765	1.043251	1.267155	0.004899
KEAP1	1.074416	0.984902	1.172066	0.105835
DDX43	0.99466	0.913388	1.083163	0.902015
PTPRH	0.82359	0.758582	0.894169	3.72E-06
SLC35C2	0.9966	0.922343	1.076835	0.931294
CRYBG3	1.054452	0.995822	1.116533	0.069289
RFX3	0.968839	0.90273	1.03979	0.380003
RIF1	0.977314	0.918272	1.040152	0.470446
RAB21	0.976489	0.895144	1.065225	0.591867
SLC4A4	1.213883	1.131739	1.30199	5.91E-08
SMARCA2	0.872703	0.818118	0.930929	3.60E-05
SESN1	0.955725	0.89645	1.018921	0.165685
MID2	1.154082	1.010979	1.317441	0.03387
SRCAP	0.940515	0.841534	1.051137	0.279727
KCNN2	1.088231	1.026163	1.154054	0.004774
CNOT4	0.928104	0.858286	1.003602	0.061503
PSEN1	0.975753	0.900371	1.057445	0.549598
CPOX	1.043463	0.983221	1.107396	0.160842
CLDND1	0.966409	0.89365	1.045091	0.392227
HSP90AA1	1.032954	0.949094	1.124223	0.452941
RBL1	1.122249	1.047131	1.202756	0.001103
DLGAP4	0.845806	0.775206	0.922835	0.000166
IGSF9B	0.938476	0.842915	1.044872	0.246511
NDC80	1.043013	0.970359	1.121107	0.252961
AP4E1	1.015135	0.937293	1.099442	0.7121
RSBN1	1.021658	0.942127	1.107903	0.604319
MAGI3	1.083735	1.00633	1.167093	0.033431
CXCL2	1.062034	1.020885	1.104841	0.002834
COL4A4	1.112399	0.977055	1.266492	0.107556
TCF7	0.980823	0.934601	1.029332	0.431768
OSTM1	1.152457	1.067619	1.244036	0.000276
IMPG2	1.153008	1.033695	1.286092	0.010632
PCNP	1.009415	0.918458	1.10938	0.845782
EXD2	1.047847	0.970914	1.130875	0.229639
ARG2	1.039809	0.961876	1.124056	0.326057
MEF2C	1.164373	1.106193	1.225614	5.92E-09
PTPRC	0.994287	0.928665	1.064545	0.869357
UBA5	1.061159	0.963846	1.168297	0.226429
STK17B	0.966561	0.909437	1.027274	0.273854
CDC14B	1.250449	1.135616	1.376894	5.43E-06
ZNF510	1.117672	1.035687	1.206147	0.004209
ZNF506	0.933545	0.872581	0.998767	0.04596
JMJD4	1.250113	1.126471	1.387327	2.66E-05
DUSP12	1.063005	0.963628	1.17263	0.222424
AACS	1.138079	1.039234	1.246325	0.005269
PHLPP1	0.959338	0.897886	1.024996	0.219067
ATP8B1	1.01267	0.939526	1.091509	0.742037
IL12RB2	1.134268	1.087824	1.182695	3.50E-09
SMARCD3	1.00791	0.940414	1.080251	0.823701
WDR70	1.083103	0.994781	1.179266	0.065857
STRADB	1.019351	0.964283	1.077563	0.49879
BZW1	0.994626	0.917397	1.078356	0.896035
C1QTNF3	0.982489	0.904156	1.067609	0.676883
ME2	0.990184	0.905298	1.083029	0.829202
CCNT2	0.962329	0.901682	1.027057	0.247623
FAM135A	0.857206	0.795808	0.923341	4.84E-05
COL19A1	0.969927	0.892131	1.054508	0.474124
EPB41L3	1.188608	1.120023	1.261393	1.21E-08

COBLL1	0.936258	0.873822	1.003155	0.061415
DLG3	1.102545	1.008728	1.205087	0.031438
TRAF5	0.887623	0.836371	0.942015	8.55E-05
MRPL22	1.01448	0.901952	1.141046	0.810597
GEMIN5	1.065487	0.993409	1.142794	0.075909
NFE2L1	0.964005	0.89088	1.043132	0.362403
GSK3B	1.03905	0.953675	1.132068	0.381206
ITGB5	1.041611	0.98413	1.102449	0.159247
ERC1	0.978715	0.903604	1.060071	0.597443
XPO1	1.052495	0.976722	1.134146	0.179556
RNF13	1.011501	0.939874	1.088586	0.76024
PALB2	1.067005	0.974415	1.168394	0.161406
LYRM2	1.13303	1.013675	1.266438	0.02787
BCKDHB	1.059566	0.987065	1.137393	0.109612
ULK2	1.07079	1.001651	1.144701	0.044599
TNPO1	1.069244	0.999614	1.143724	0.051327
PLOD1	1.034439	0.970109	1.103035	0.301323
P2RX5	0.989821	0.931517	1.051774	0.741159
ITGAE	1.086864	1.01202	1.167243	0.022126
DIS3	0.954736	0.882256	1.03317	0.250185
PIBF1	0.981215	0.909976	1.05803	0.621916
TDRD3	1.022266	0.930278	1.123351	0.647138
NUFIP1	0.972058	0.885274	1.06735	0.55255
PDS5B	1.035123	0.959167	1.117093	0.374658
OXCT1	1.150362	1.064885	1.242701	0.000377
RRAGB	1.073314	0.993795	1.159196	0.071628
CYLD	0.982172	0.921294	1.047071	0.581614
SLC27A5	1.084208	0.927606	1.267248	0.309726
ZNF324	1.047151	0.960917	1.141124	0.293376
ZNF671	0.989981	0.919711	1.06562	0.788651
ZNF416	1.067796	0.985219	1.157295	0.11019
ZNF586	0.966946	0.906872	1.031	0.304375
ZNF446	1.008232	0.899903	1.129602	0.887583
ZNF264	0.982461	0.918682	1.050668	0.605372
RPS5	0.911086	0.846265	0.980871	0.013403
FAT1	1.074387	1.016003	1.136125	0.01184
YTHDC1	0.928318	0.86678	0.994226	0.03355
CHMP2B	1.021256	0.930801	1.120502	0.656682
SMAP2	0.902616	0.836216	0.974288	0.008586
PPIE	1.209016	1.093313	1.336964	0.000217
ZMPSTE24	1.136183	1.038115	1.243516	0.005569
STARD7	1.073437	0.984194	1.170772	0.109553
REST	1.022691	0.9535	1.096903	0.530161
HAL	0.917364	0.877369	0.959182	0.000149
SSH1	0.936065	0.864165	1.013947	0.105169
GSTP1	1.116271	1.034229	1.204822	0.004742
APLP2	0.948621	0.884539	1.017344	0.139382
WBP11	0.857483	0.78432	0.937471	0.000728
EIF3I	0.916714	0.809586	1.038019	0.170228
TXLNA	1.07442	0.996732	1.158163	0.060864
NCOA1	0.926292	0.853247	1.005591	0.06771
AGBL5	1.133586	1.026239	1.252161	0.013503
EFR3B	1.043577	0.923995	1.178635	0.492129
KIF3C	1.148168	1.062588	1.240641	0.000472
RAB10	1.087379	1.0072	1.173939	0.032069
HADHA	0.956924	0.868626	1.054197	0.3727
MAPRE3	1.20306	1.096622	1.319829	9.17E-05
CAD	1.044528	0.987008	1.1054	0.131692

CD59	1.159155	1.103261	1.217881	4.71E-09
CD82	1.118554	1.052951	1.188243	0.00028
BCORL1	0.943373	0.87171	1.020927	0.148134
ATRX	1.022583	0.947241	1.103917	0.567392
FCN1	1.041487	1.00615	1.078065	0.020994
MYNN	0.983023	0.903348	1.069726	0.691342
SCAMP1	1.035728	0.956599	1.121401	0.386644
PREP	1.084557	0.995467	1.181621	0.063442
HACE1	1.027887	0.955774	1.10544	0.458617
SEH1L	0.984268	0.913439	1.06059	0.677299
WDR47	1.012928	0.933542	1.099064	0.757726
WDFY1	1.017433	0.950265	1.089348	0.619912
OVGP1	1.018425	0.959975	1.080433	0.544906
SLC25A24	1.038447	0.967281	1.114848	0.297619
MAP3K4	1.019442	0.949008	1.095104	0.598093
PILRA	0.946412	0.888568	1.008021	0.086957
IGSF9	1.082348	1.013869	1.155453	0.017644
ABCB1	0.922678	0.880825	0.96652	0.00068
ZNF213	1.152058	1.035598	1.281614	0.009234
AKR1B1	1.189629	1.109861	1.275131	9.42E-07
CPNE3	1.125853	1.067585	1.187301	1.23E-05
RRN3	1.004246	0.931095	1.083144	0.912563
CTTN	1.063043	0.997898	1.132441	0.058127
WNT11	0.968265	0.902074	1.039313	0.372043
MTIF2	0.993857	0.900451	1.096953	0.902613
DDHD2	1.033619	0.952931	1.121139	0.425248
TTC39A	0.976119	0.899445	1.059331	0.562536
EPS15	0.955239	0.89398	1.020697	0.17568
MGST2	1.127657	1.043192	1.21896	0.002491
CHERP	0.893346	0.817984	0.975651	0.012136
ATG16L1	0.999937	0.930176	1.07493	0.998629
USP40	1.134587	1.054912	1.22028	0.000676
POMGNT1	1.155456	1.064461	1.254228	0.000555
RAD54L	1.052265	0.974722	1.135977	0.192088
MAST2	1.123246	1.038226	1.215228	0.003802
DNAJA1	0.976941	0.892885	1.06891	0.611296
B4GALT1	0.898486	0.837039	0.964444	0.00306
CHMP5	1.077465	0.964758	1.203338	0.185662
NFX1	0.954653	0.883944	1.031018	0.237221
IPO11	1.041208	0.963435	1.12526	0.30796
EIF2AK1	0.982183	0.905435	1.065437	0.664972
EPDR1	1.060618	1.004719	1.119627	0.033141
SNX10	1.107835	1.04256	1.177196	0.000949
SEPHS1	1.129205	1.019386	1.250854	0.019923
MRPL28	1.239513	1.085551	1.415311	0.001509
HBQ1	1.018061	0.975296	1.062702	0.413634
ITPKC	0.984419	0.905806	1.069855	0.711523
CEACAM6	1.008159	0.978322	1.038907	0.596011
FAT2	1.03917	0.905494	1.19258	0.584448
RBM22	0.862933	0.787166	0.945992	0.001666
TMED2	1.002813	0.915796	1.098099	0.95163
ZFAND6	0.94791	0.890528	1.008989	0.093134
PPEF1	1.302586	1.106615	1.533262	0.001484
LAT2	1.208519	1.146331	1.274082	2.12E-12
HUWE1	0.994646	0.928171	1.065881	0.879089
ZW10	1.035511	0.952777	1.125429	0.411447
ALG9	1.035302	0.934674	1.146764	0.506043
MYBPC2	1.026302	0.948269	1.110756	0.519925

ACOX3	1.074611	0.997351	1.157857	0.058719
MTMR2	0.8848	0.810819	0.965532	0.006009
PPP1R15A	0.894952	0.829745	0.965284	0.004036
HSD17B14	1.424741	1.291442	1.571798	1.63E-12
TRIP6	1.063452	1.003152	1.127376	0.038863
ACHE	1.026586	0.971876	1.084376	0.347713
FTL	0.902054	0.835901	0.973443	0.007987
BAX	0.975813	0.875272	1.087902	0.658968
NLK	1.020625	0.958264	1.087045	0.52565
PIGS	1.077852	0.971915	1.195336	0.155526
ADAMTS2	0.917754	0.870095	0.968024	0.001608
ATXN7L3	0.939824	0.870033	1.015212	0.114919
PGS1	1.049878	0.978291	1.126704	0.176743
PSMC5	1.091575	0.966096	1.233353	0.159619
UIMC1	0.935835	0.858999	1.019545	0.129234
CETP	0.817505	0.763702	0.875099	6.59E-09
MMP2	0.944012	0.909349	0.979996	0.002539
LPCAT2	1.012279	0.953377	1.07482	0.689885
OGFOD1	1.056455	0.959501	1.163206	0.263481
SH3BP2	1.104775	1.031749	1.18297	0.004294
NOP14	1.063221	0.958178	1.179781	0.248079
ADD1	0.957138	0.869656	1.05342	0.370362
L2HGDH	1.117236	1.011544	1.233971	0.028791
TXNDC16	1.106336	1.025308	1.193767	0.009215
NID2	1.027439	0.947058	1.114641	0.51488
GMCL1	0.95035	0.878787	1.027742	0.202344
SF3B2	1.000111	0.889178	1.124884	0.998526
GNAS	0.85506	0.765384	0.955244	0.005606
DNM1L	1.011945	0.929475	1.101731	0.784268
PHACTR3	1.141326	1.094495	1.190161	6.25E-10
ERGIC2	1.052518	0.980392	1.12995	0.15759
AURKA	1.044443	0.976775	1.1168	0.203244
CASS4	0.923368	0.867586	0.982737	0.012153
PIR	1.215767	1.139981	1.296592	2.69E-09
RFX2	1.196619	1.100973	1.300574	2.41E-05
METTL2A	1.17123	1.058094	1.296464	0.002293
SULT2B1	1.13315	1.006162	1.276166	0.039278
ALG6	1.145041	1.052346	1.2459	0.001663
CNOT3	0.900462	0.818152	0.991051	0.032052
GP6	1.031776	0.988283	1.077183	0.154563
PTPN4	1.052233	0.973844	1.136932	0.197407
DDX18	1.108851	1.017467	1.208444	0.018544
KHSRP	0.964549	0.886572	1.049383	0.401341
GNA11	0.989024	0.908588	1.076581	0.798723
EDEM2	0.971218	0.885236	1.065551	0.536903
DNMT3B	1.152519	1.100097	1.207438	2.28E-09
TPX2	1.091608	1.020787	1.167342	0.010434
FER1L4	1.01747	0.896082	1.155302	0.789321
PDRG1	0.96963	0.894564	1.050994	0.453149
EPB41L1	0.981571	0.845645	1.139346	0.80678
DOCK9	1.077754	1.003576	1.157415	0.039582
ANKRD10	0.999366	0.919601	1.086049	0.988069
TGDS	1.115373	1.016417	1.223962	0.021251
DOCK3	0.989019	0.899642	1.087274	0.819264
COQ9	1.222335	1.121688	1.332014	4.67E-06
TMEM40	1.114558	1.008944	1.231228	0.032739
KIF9	1.137504	1.020301	1.268171	0.020222
CRLS1	1.020366	0.904167	1.151497	0.743794

PPP1R13B	1.074389	0.993738	1.161585	0.071516
ATRN	0.98384	0.916933	1.055628	0.650259
SMOX	1.00038	0.955465	1.047405	0.987082
SIGLEC1	1.030031	0.976979	1.085964	0.272772
FKBP1A	1.206836	1.08964	1.336637	0.00031
NSFL1C	1.117292	1.001878	1.246002	0.046186
SLC4A11	0.962966	0.870873	1.064799	0.461862
ZNF343	1.143572	1.035192	1.263299	0.008271
EBF4	1.010587	0.950494	1.074478	0.736352
CPXM1	0.949122	0.913698	0.985918	0.00713
XRN2	0.924423	0.849566	1.005875	0.068151
DYNLL1	1.10804	1.038425	1.182322	0.001943
TESC	1.145891	1.090937	1.203615	5.60E-08
SNX5	0.935053	0.850703	1.027768	0.163878
RPL6	0.833568	0.770477	0.901825	5.81E-06
SIRPG	1.044559	0.969046	1.125957	0.254838
MAPKAPK	1.036339	0.941135	1.141172	0.467841
P2RX7	1.032688	0.972742	1.096328	0.291792
ESF1	1.024639	0.930839	1.127893	0.619263
RBBP9	1.155515	1.057724	1.262347	0.001356
ANAPC5	1.135128	1.039985	1.238976	0.004543
SLC23A2	1.019304	0.9489	1.094932	0.60056
KDM2B	0.981292	0.89107	1.080649	0.70114
TASP1	0.980508	0.910522	1.055873	0.602363
OAS1	1.011154	0.954502	1.071169	0.706129
RPLP0	0.870761	0.807936	0.938472	0.000292
PXN	0.895261	0.840483	0.95361	0.000594
SIRT4	1.113357	0.983633	1.260191	0.089344
KIF16B	1.12244	1.058065	1.190731	0.000127
TRMT6	1.188161	1.094426	1.289924	3.92E-05
PEBP1	0.981607	0.90401	1.065865	0.658614
BRAP	0.939829	0.86619	1.019729	0.136047
ERP29	0.941178	0.855706	1.035188	0.212022
FUS	0.984748	0.908172	1.067782	0.709813
IGBP1	0.853779	0.76967	0.947079	0.002812
FXYD5	0.924099	0.867775	0.984079	0.013887
ZNF302	1.068443	0.98136	1.163253	0.126962
GRAMD1A	0.865554	0.799511	0.937052	0.000363
CMTM1	1.117991	1.037551	1.204668	0.003416
KCNH4	1.12544	1.043006	1.214389	0.002327
GANAB	0.959182	0.894976	1.027995	0.238435
GMIP	0.91378	0.846804	0.986054	0.020256
RBM41	1.197145	1.093363	1.310778	0.000101
BIRC5	1.201176	1.125669	1.281748	3.14E-08
LAG3	1.161969	1.0842	1.245316	2.16E-05
MLF2	0.968694	0.864659	1.085247	0.583214
OTUB2	1.003108	0.94587	1.063809	0.917563
DDX24	0.963992	0.88907	1.045228	0.374335
ZBTB25	1.02475	0.943748	1.112703	0.560619
NECAP1	0.937256	0.871669	1.007778	0.080008
ARHGAP4	1.064532	0.991448	1.143003	0.084839
ANKRD24	1.350312	1.203754	1.514713	3.00E-07
DHX32	0.866174	0.81126	0.924805	1.71E-05
RCOR1	0.938973	0.8719	1.011205	0.095857
LTBP4	1.065746	1.006172	1.128847	0.030037
BLVRB	1.038151	0.987511	1.091387	0.142262
SLC9A1	0.892037	0.827097	0.962076	0.003052
SPTLC1	1.044649	0.938085	1.163319	0.426213

PAPOLA	1.040615	0.948089	1.14217	0.402053
CCNK	0.911895	0.840534	0.989315	0.026531
PCBP4	0.985671	0.913016	1.064108	0.711809
RGS1	1.034405	0.983914	1.087488	0.185231
YPEL3	0.869419	0.794313	0.951626	0.002401
MRPS33	1.025507	0.917197	1.146609	0.658294
NDUFB2	1.18091	1.06824	1.305462	0.001153
NUDC	1.16586	1.036601	1.311236	0.010482
MAEA	1.037986	0.934885	1.152457	0.484868
ICAM1	1.008981	0.959521	1.06099	0.727347
STRN4	0.937107	0.857895	1.023633	0.14942
IRAK3	0.960963	0.911866	1.012705	0.136712
LYZ	0.961886	0.932549	0.992146	0.013937
MUL1	1.035845	0.946678	1.133411	0.443184
TFAP4	1.042715	0.954666	1.138886	0.35275
PDCD7	0.992604	0.907982	1.085112	0.870297
SPG21	1.122971	1.015291	1.24207	0.024131
DNAJB11	0.910824	0.84584	0.9808	0.013387
FLT3LG	1.179824	1.057203	1.316668	0.003142
RAB11FIP3	0.952354	0.889322	1.019854	0.162334
GNPTG	1.148924	1.017653	1.297128	0.02492
ZNF268	1.107903	0.999823	1.227666	0.050396
GOLGA3	0.969729	0.904337	1.03985	0.388171
PABPC4	0.878008	0.797579	0.966548	0.007953
CD209	1.173851	1.04234	1.321956	0.008194
MCOLN1	1.082473	0.986008	1.188375	0.096095
USP48	1.002969	0.932791	1.078427	0.93616
EFNB1	1.019336	0.962839	1.079149	0.510345
PDPR	0.988563	0.915318	1.06767	0.769633
GLG1	1.110621	1.031457	1.19586	0.005421
KIF4A	1.131692	1.063376	1.204397	9.85E-05
TNRC6A	0.999993	0.934007	1.070641	0.999842
PLEKHG2	0.965118	0.897089	1.038305	0.341078
DLL3	0.83782	0.765624	0.916825	0.000119
NAT14	0.823302	0.76537	0.88562	1.76E-07
PITPNM2	0.943634	0.88061	1.011168	0.099958
EXOC1	1.045487	0.968091	1.129071	0.256976
RBM27	0.969123	0.898241	1.045598	0.418312
OSBPL8	0.982124	0.917621	1.05116	0.602767
DTX2	1.069122	0.977933	1.168814	0.141725
NLRC4	0.978065	0.928639	1.030121	0.401863
PUS7	1.052202	0.977555	1.132549	0.175314
NRCAM	0.772971	0.685011	0.872226	2.94E-05
LAMB1	0.995045	0.93797	1.055592	0.86908
DLD	0.948123	0.87984	1.021704	0.162438
WDR7	1.050242	0.984936	1.119878	0.134506
TXNL1	0.955365	0.858549	1.063099	0.402269
IL5RA	0.82153	0.776472	0.869201	8.45E-12
ABCC6	0.973135	0.911068	1.039429	0.418002
CMTM6	0.920315	0.854687	0.990982	0.027811
ITGA6	1.015068	0.962931	1.070028	0.57828
RAPGEF4	0.981293	0.877888	1.096877	0.739588
FH	1.180254	1.083209	1.285992	0.000153
SEL1L3	1.143335	1.091806	1.197296	1.25E-08
CDV3	0.921374	0.844967	1.004691	0.063738
MYO15A	0.997251	0.889002	1.118682	0.962553
ALKBH5	0.973386	0.899984	1.052775	0.500114
NLRP1	0.913629	0.864309	0.965763	0.001421

PITPNM3	1.019967	0.86454	1.203338	0.814689
SPAG7	0.994226	0.886622	1.11489	0.921075
ZC3HC1	1.099997	0.992575	1.219046	0.069093
ESR1	1.45877	1.279554	1.663087	1.64E-08
ANGPT2	1.075259	1.002908	1.152831	0.041186
TMEM101	1.106782	1.002128	1.222364	0.045294
CD200	0.952229	0.910493	0.995877	0.032303
CCDC80	1.077805	0.925312	1.255429	0.335721
CMA1	0.99458	0.870402	1.136475	0.936345
PSME1	1.064601	0.971217	1.166963	0.181398
PPP2R3C	0.978118	0.879359	1.087967	0.683695
HAUS4	1.004289	0.914388	1.103028	0.928731
JPH4	1.1583	1.03915	1.291112	0.007969
CEBPE	0.950346	0.910277	0.992178	0.02049
SLC7A8	1.030651	0.943985	1.125273	0.500523
OSGEP	1.120327	1.020042	1.230472	0.017563
SLC22A17	1.277431	1.192795	1.368072	2.55E-12
RNF31	1.143971	1.007077	1.299473	0.038601
SCFD1	0.956577	0.891452	1.02646	0.217193
HECTD1	0.983421	0.92284	1.047978	0.606296
HNRNPC	0.99227	0.896021	1.098858	0.8815
RPGRIP1	0.786683	0.683653	0.90524	0.000808
SUPT16H	1.134337	1.039178	1.23821	0.004808
TOX4	0.947532	0.871646	1.030024	0.205727
TGM1	1.045651	0.954224	1.145838	0.338955
TINF2	1.041672	0.943366	1.150221	0.419531
TBL1Y	1.042644	0.978738	1.110724	0.195661
SEMA6A	1.029963	0.944972	1.122597	0.501666
TRPM7	0.99279	0.924744	1.065843	0.841678
TYRO3	1.350957	1.21763	1.498883	1.39E-08
WDR76	1.076607	0.998592	1.160716	0.054447
CAPN3	1.0121	0.946884	1.081809	0.723395
SNAP23	0.93779	0.856738	1.026511	0.163732
PHGDH	1.092776	1.029231	1.160245	0.003701
COL9A3	0.918636	0.83022	1.016467	0.100252
EZR	0.923606	0.862869	0.988617	0.022031
MYL6	0.91416	0.815017	1.025363	0.125442
TEKT2	0.941001	0.871377	1.016188	0.121017
CLSPN	1.189444	1.108055	1.27681	1.61E-06
RFFL	0.929774	0.860885	1.004174	0.063752
UNC13D	0.984091	0.914944	1.058463	0.666154
MFSD11	1.163663	1.051124	1.288252	0.003492
DPYSL2	1.143414	1.100659	1.18783	5.48E-12
GPATCH2	1.049126	0.955921	1.151419	0.312356
NUP50	0.917261	0.854629	0.984482	0.016693
COMT	0.994924	0.926139	1.068816	0.889263
VNN3	1.0669	1.007752	1.129519	0.02606
ECHDC1	1.058191	0.97993	1.142702	0.149076
LRRFIP2	0.903897	0.838126	0.974831	0.00876
SEC22C	1.056704	0.968559	1.152871	0.214563
XYLB	1.161307	1.074901	1.254658	0.00015
HDAC6	1.038732	0.959654	1.124326	0.346906
CDC6	1.155456	1.072164	1.245218	0.000153
UPRT	1.060421	0.963147	1.167519	0.232062
CDC23	1.082645	0.990588	1.183257	0.079878
AAAS	1.142973	1.040254	1.255835	0.005413
CBX5	1.059002	0.991719	1.130851	0.086956
MSH2	1.068838	0.987169	1.157263	0.100686

MAP3K1	0.905837	0.851254	0.963921	0.001816
DHPS	1.025926	0.947892	1.110384	0.525994
HOOK2	1.161834	1.051635	1.28358	0.003176
ARCN1	0.996609	0.917938	1.082023	0.935477
TMEM38B	1.258907	1.161456	1.364536	2.13E-08
PSMD5	0.971087	0.895313	1.053274	0.479064
PTGS1	1.112648	1.054483	1.174023	9.76E-05
NUP188	1.042571	0.957427	1.135288	0.337509
CRAT	1.17176	1.075989	1.276055	0.000269
SH2D3C	1.130682	1.063176	1.202474	9.21E-05
NANS	0.931383	0.851373	1.018912	0.120867
TBC1D2	0.987547	0.928984	1.049803	0.687873
PDE6C	1.007983	0.882601	1.151178	0.906602
CWF19L1	1.01743	0.939031	1.102375	0.672759
SEMA4G	1.143289	0.989278	1.321276	0.069687
BTAF1	0.965439	0.915001	1.018658	0.198893
IKZF5	0.943939	0.872917	1.020739	0.148285
BLNK	1.12553	1.05462	1.201208	0.000368
SORBS1	1.020058	0.952532	1.092371	0.56983
BAMBI	1.073757	1.029655	1.119747	0.000882
IL11	0.88908	0.830038	0.952322	0.000798
WAC	0.9193	0.851462	0.992543	0.031449
CREM	1.01091	0.928133	1.10107	0.803406
NUBP2	1.182381	1.058095	1.321266	0.003111
TPSD1	0.869249	0.839155	0.900423	6.46E-15
HIVEP1	0.985475	0.923863	1.051196	0.6569
TREM2	0.99628	0.932638	1.064265	0.911889
KCNK16	0.910902	0.825443	1.005209	0.063369
CRISP3	1.074232	1.044125	1.105207	7.93E-07
FKBP5	1.025183	0.967429	1.086385	0.400525
SRPK1	1.013002	0.949114	1.081191	0.697522
BRPF3	0.985692	0.908042	1.069983	0.730672
MRPS18A	1.328915	1.183609	1.492061	1.49E-06
TMEM14A	1.375784	1.292783	1.464114	9.34E-24
EFHC1	1.03302	0.935875	1.140249	0.51911
NCR2	1.037853	0.982384	1.096455	0.184917
HSP90AB1	0.962439	0.883208	1.048777	0.382424
MLN	1.119429	0.994485	1.26007	0.061709
CDC5L	0.930832	0.860748	1.006622	0.072701
ITPR3	1.041217	0.982561	1.103375	0.172168
ZNF184	1.049275	0.985341	1.117358	0.133724
SIRT1	0.974845	0.904284	1.050911	0.506309
HNRNPH3	0.951688	0.864602	1.047546	0.311868
IFT74	1.074618	0.965508	1.196058	0.187703
JAK2	1.003015	0.938882	1.071528	0.928855
IL12RB1	1.10938	1.031882	1.192697	0.004963
ABL1	0.936754	0.866765	1.012395	0.099139
ACOT7	1.178886	1.09252	1.272079	2.24E-05
SH3GLB1	0.893261	0.823542	0.968883	0.006481
CDC7	1.135302	1.052515	1.224601	0.00102
SYDE2	1.166665	1.102199	1.234902	1.07E-07
PCSK5	1.361528	1.242375	1.492109	3.99E-11
SCD	1.10819	1.053078	1.166186	7.91E-05
TMED1	1.03317	0.924279	1.154889	0.565793
ABLIM1	1.066456	1.016759	1.118583	0.008228
ERMP1	1.09561	1.033208	1.16178	0.002274
RAB18	0.933799	0.864854	1.008239	0.080068
NRP1	0.906414	0.843363	0.97418	0.007561

HSD17B7P	1.041482	0.967913	1.120644	0.276851
PRTFDC1	1.130437	1.052221	1.214468	0.000804
TSPAN15	1.131434	1.068831	1.197703	2.12E-05
MAST3	1.05473	0.986232	1.127987	0.119876
MZF1	1.05544	0.974959	1.142564	0.182428
OCEL1	1.133426	1.035834	1.240213	0.006404
MYO9B	0.942262	0.873277	1.016695	0.125244
KCNK6	1.097637	1.03753	1.161227	0.001186
PSMD8	1.171701	1.032042	1.330258	0.014404
FBXL19	1.004174	0.942575	1.069799	0.897384
STX1B	1.212494	1.092601	1.345543	0.000287
HSD3B7	1.130703	1.046703	1.221444	0.001815
SETD1A	0.941715	0.871485	1.017605	0.128853
BCL7C	1.007563	0.883252	1.14937	0.910709
CIRBP	1.064866	0.951466	1.19178	0.273966
PRKY	1.024592	0.990531	1.059825	0.159008
IGFALS	1.080103	0.991412	1.176729	0.077956
HNRNPM	0.886453	0.8147	0.964527	0.005132
NDUFB7	1.06123	0.986369	1.141774	0.111331
TECR	1.109566	1.004694	1.225384	0.040131
TIMM13	1.108876	1.016703	1.209404	0.019591
CDC34	0.974571	0.887058	1.070717	0.591561
MTAP	1.047398	0.971766	1.128916	0.225892
POLR2E	1.163052	1.025434	1.31914	0.01873
POLRMT	1.060718	0.976998	1.151613	0.159958
HCN2	0.918522	0.827287	1.019819	0.111321
RASSF7	1.137096	1.047959	1.233814	0.002038
GADD45B	0.90442	0.850227	0.962067	0.001439
PALM	0.903162	0.863743	0.944381	7.70E-06
MADCAM1	1.241198	1.009923	1.525436	0.039994
MKNK2	0.865633	0.806903	0.928638	5.69E-05
ARVCF	0.956415	0.901145	1.015076	0.142297
TRMT2A	1.046177	0.9666	1.132307	0.263408
RANBP1	1.121051	1.018157	1.234344	0.020003
ZDHHC8	0.90977	0.845905	0.978458	0.010884
KLHL22	1.076938	0.996756	1.16357	0.060428
MED15	0.934984	0.859099	1.017571	0.119555
SERPIND1	1.018476	0.958515	1.082187	0.55429
SNAP29	1.087011	0.972544	1.21495	0.141676
CRKL	0.982766	0.909534	1.061894	0.659935
LZTR1	1.209121	1.09522	1.334866	0.000169
MMP11	1.33345	1.201516	1.479872	6.18E-08
CECR2	1.229521	1.092494	1.383734	0.00061
SMARCB1	1.107052	0.993914	1.233068	0.064462
DERL3	1.140668	1.015322	1.281487	0.026692
BCL2L13	0.95177	0.864343	1.04804	0.314651
DDTL	1.078224	1.007161	1.154302	0.03038
OSM	1.020263	0.970044	1.073082	0.435999
CABIN1	1.062532	0.979408	1.152712	0.14447
TBC1D10A	1.028746	0.956052	1.106968	0.448474
SUSD2	1.127829	1.018823	1.248498	0.020365
SF3A1	0.926662	0.843061	1.018553	0.114361
GGT5	0.930284	0.883786	0.97923	0.00574
RNF215	1.1729	1.052087	1.307585	0.004034
SEC14L2	1.143566	1.061998	1.231399	0.000381
SEC14L3	1.117417	1.004915	1.242514	0.040314
PPIL2	1.06629	0.978249	1.162254	0.144345
UPB1	1.101778	1.031235	1.177147	0.004092

YPEL1	1.113495	1.029216	1.204676	0.007427
SNRPD3	1.127931	1.031616	1.233239	0.008207
PES1	1.022904	0.931948	1.122737	0.63364
MAPK1	0.985684	0.908895	1.068961	0.727505
GGT1	1.086276	0.970023	1.21646	0.151869
PRODH	0.961668	0.885927	1.043885	0.350392
PPM1F	1.069219	0.998728	1.144687	0.054433
SLC35E4	1.102337	1.015666	1.196404	0.0197
TOP3B	1.089743	0.933306	1.272401	0.277043
CYTH4	1.083575	1.028593	1.141496	0.002519
MFNG	1.010474	0.942448	1.083409	0.76951
CARD10	1.029926	0.921808	1.150725	0.60229
LRP5L	0.884902	0.839788	0.932439	4.65E-06
SLC25A1	0.923122	0.859066	0.991953	0.029246
LGALS2	1.012924	0.97981	1.047157	0.448929
GGA1	0.955566	0.87391	1.044852	0.318634
HIRA	0.949033	0.863759	1.042725	0.27615
SH3BP1	1.13221	1.030653	1.243773	0.009607
SEZ6L	0.892178	0.822478	0.967784	0.005978
LGALS1	1.091425	1.045133	1.139768	7.61E-05
HPS4	1.00059	0.926213	1.080939	0.988068
PIK3IP1	0.94895	0.896915	1.004004	0.068592
SRRD	1.10346	1.006618	1.209619	0.035666
PATZ1	0.997842	0.922438	1.079411	0.957033
TRIOBP	1.014222	0.919103	1.119184	0.778671
TFIP11	1.064812	0.978856	1.158315	0.143651
GCAT	1.091854	1.02428	1.163885	0.007019
CRYBB1	1.225907	1.097389	1.369476	0.000313
ANKRD54	1.142909	1.042226	1.253318	0.004526
EIF3L	0.858069	0.793232	0.928206	0.000134
MICALL1	0.984841	0.916888	1.057831	0.67541
POLR2F	1.168278	1.035985	1.317465	0.011196
SOX10	1.01612	0.876163	1.178435	0.832496
CCDC134	1.032765	0.962854	1.107752	0.367328
DEPDC5	1.042058	0.966057	1.124038	0.286317
PICK1	1.02237	0.939542	1.112501	0.607788
TTC28	1.032101	0.974858	1.092705	0.277785
SLC16A8	1.13555	1.061583	1.214672	0.000217
CENPM	1.165535	1.081697	1.255872	5.77E-05
SLC5A4	0.937225	0.854721	1.027693	0.16791
KDELR3	0.992565	0.941366	1.046549	0.782412
CYP2D6	1.07074	0.993282	1.15424	0.074421
DDX17	1.016941	0.954618	1.083333	0.602625
DMC1	1.03358	0.937705	1.139258	0.506061
TCF20	1.029594	0.958841	1.105569	0.42204
HSCB	1.03667	0.925427	1.161285	0.534055
CBY1	1.200518	1.083498	1.330176	0.000478
TOMM22	1.037884	0.938441	1.147865	0.469317
XBP1	0.911918	0.845171	0.983936	0.017428
JOSD1	0.888277	0.819216	0.96316	0.004118
FBXO7	0.946097	0.8667	1.032767	0.21534
GTPBP1	0.931902	0.872366	0.995501	0.036275
POLDIP3	0.887507	0.811522	0.970607	0.008968
RAB36	1.059634	0.930095	1.207216	0.383936
TIMP3	1.103582	1.062474	1.146281	3.60E-07
SBF1	0.986114	0.927651	1.048262	0.653846
CYB5R3	0.971882	0.873618	1.081198	0.599972
DNAL4	1.055265	0.959515	1.160569	0.267688

LMF2	1.003616	0.915956	1.099667	0.938295
RHBDD3	1.113276	1.021254	1.213591	0.01478
PACSIN2	0.953245	0.881887	1.030376	0.227746
TTLL1	1.167534	1.071824	1.271791	0.000386
RASL10A	0.818359	0.762613	0.87818	2.57E-08
AP1B1	0.894809	0.828566	0.966347	0.004621
HMGXB4	1.058132	0.972443	1.151371	0.189714
TOM1	0.901977	0.838783	0.969931	0.005373
NEFH	0.935333	0.853086	1.02551	0.154573
CHKB	1.246757	1.102015	1.41051	0.00046
BIK	1.063724	1.002913	1.128224	0.039706
HMOX1	1.005754	0.96321	1.050177	0.794738
MCAT	1.056702	0.956268	1.167684	0.279082
THOC5	1.0442	0.947187	1.151149	0.38465
MCM5	1.103463	1.015751	1.198749	0.019818
APOBEC3H	1.235665	1.144092	1.334567	7.19E-08
ARSA	1.02341	0.951948	1.100235	0.530948
TSPO	1.014399	0.959528	1.072409	0.614349
RASD2	1.011747	0.933869	1.09612	0.775048
TTLL12	1.068232	1.006913	1.133285	0.028641
CBX7	1.08671	1.017642	1.160465	0.013066
PDGFB	0.981731	0.879407	1.095961	0.742674
ACR	1.068281	0.97605	1.169228	0.151639
CABP7	1.059035	0.933091	1.201979	0.374585
RPL3	0.857899	0.788651	0.933226	0.000358
ZMAT5	1.149899	1.011713	1.306961	0.032498
SYNGR1	0.848027	0.802579	0.896049	4.48E-09
ASCC2	1.096907	0.992122	1.212759	0.070985
MTMR3	0.968897	0.902725	1.03992	0.381342
APOL4	1.178801	1.109479	1.252454	1.04E-07
APOL1	1.144445	1.069135	1.225059	0.000102
PNPLA3	1.455895	1.304731	1.624573	1.87E-11
MYH9	0.93464	0.870494	1.003513	0.062419
CACNA1I	1.080628	0.955695	1.221893	0.216074
SAMM50	1.235649	1.101705	1.385879	0.000301
TXN2	1.11609	1.003939	1.240771	0.042081
FOXRED2	1.166552	1.093527	1.244455	3.00E-06
GRAP2	0.991124	0.93408	1.051652	0.768163
EIF3D	0.859494	0.777559	0.950062	0.003055
TNRC6B	0.98979	0.932482	1.05062	0.735927
SGSM3	1.08075	0.942222	1.239645	0.267177
NCF4	1.077853	1.001779	1.159704	0.04469
CSF2RB	0.995557	0.948556	1.044887	0.856786
SLC25A17	1.161955	1.065509	1.26713	0.000686
UPK3A	1.112356	1.054298	1.173611	9.89E-05
FAM118A	1.035978	0.966963	1.109919	0.314955
KCTD17	1.272694	1.182429	1.369851	1.32E-10
ST13	0.868144	0.789469	0.954659	0.003531
IL2RB	1.027897	0.971046	1.088076	0.343214
RBX1	1.10712	0.995062	1.231798	0.061616
EP300	0.943862	0.877956	1.014716	0.117724
L3MBTL2	1.160248	1.050724	1.281188	0.003303
CHADL	1.041391	0.967554	1.120863	0.279741
RANGAP1	1.0652	0.978159	1.159987	0.146438
ZC3H7B	1.029025	0.954859	1.10895	0.453453
PHF5A	0.987535	0.88317	1.104234	0.825795
ACO2	1.005792	0.91508	1.105496	0.904676
POLR3H	1.00327	0.916248	1.098556	0.943783

TRMU	1.114353	1.024836	1.211689	0.011272
PMM1	1.144924	1.046365	1.252767	0.003211
CERK	0.954163	0.891415	1.021328	0.176404
BRD1	1.023885	0.95719	1.095227	0.492184
ZBED4	1.002274	0.931258	1.078706	0.951691
MLC1	1.007056	0.963347	1.052748	0.756126
HDAC10	1.292229	1.090469	1.531318	0.003077
KCNK10	1.185441	1.083867	1.296533	0.000198
ABHD4	0.935889	0.877504	0.998158	0.043792
KHNYN	1.002049	0.939781	1.068441	0.950147
FKBP3	1.100927	0.993852	1.219538	0.065499
SDR39U1	1.145483	1.031321	1.272282	0.011222
CTSG	1.045802	1.01782	1.074555	0.001211
GZMH	1.052833	0.995597	1.113359	0.071036
GZMB	1.083259	1.029547	1.139773	0.002055
RBM23	0.98067	0.895245	1.074247	0.674661
PRMT5	1.130653	1.032316	1.238357	0.008168
COCH	0.93861	0.875078	1.006753	0.076438
AP4S1	0.998086	0.923017	1.07926	0.961697
POLE2	1.064732	0.984066	1.15201	0.118673
SOS2	0.914623	0.852135	0.981694	0.013449
CDKL1	1.063974	0.968792	1.168508	0.194673
NIN	0.978952	0.907409	1.056136	0.582728
PYGL	0.933425	0.883682	0.985968	0.013675
TRIM9	1.30282	1.143018	1.484964	7.43E-05
PSMC6	0.978488	0.899289	1.064662	0.613565
GNPNAT1	1.041626	0.939674	1.15464	0.437741
DDHD1	0.957584	0.893806	1.025914	0.217775
CDKN3	1.218733	1.123588	1.321935	1.85E-06
CGRRF1	1.225018	1.090174	1.376542	0.000647
ATP6V1D	1.063755	0.971875	1.164323	0.179926
PLEK2	1.005252	0.950778	1.062846	0.853803
PIGH	1.065384	0.971984	1.16776	0.17607
PSMA3	0.992403	0.8977	1.097096	0.881523
VTI1B	1.124191	1.004628	1.257984	0.041307
TIMM9	0.90765	0.816558	1.008903	0.072541
GSTZ1	0.978055	0.864115	1.10702	0.725502
KIAA0586	1.083996	0.996712	1.178924	0.059691
TMED8	1.009224	0.938299	1.085511	0.804928
AHSA1	1.145784	1.042478	1.259326	0.004759
DAAM1	1.119377	1.041866	1.202654	0.002069
SPTLC2	0.99876	0.932071	1.070222	0.971939
RIN3	0.927788	0.858782	1.002338	0.057336
LGMN	0.996092	0.937737	1.058077	0.898833
ALKBH1	1.138446	1.026031	1.263177	0.014508
SNW1	0.955607	0.86045	1.061288	0.39617
CHGA	1.019986	0.934666	1.113094	0.657039
ITPK1	1.085441	0.99673	1.182048	0.059475
DHRS7	0.942728	0.861875	1.031165	0.197348
PPM1A	0.965737	0.898257	1.038287	0.345506
ASB2	1.115888	1.031194	1.207538	0.006475
ERH	1.213373	1.107042	1.329918	3.58E-05
HIF1A	0.929961	0.875827	0.98744	0.017643
SLC10A1	1.00502	0.960144	1.051994	0.829886
EIF5	1.015895	0.940488	1.097347	0.688607
SLC8A3	1.084153	1.017335	1.155359	0.012792
DICER1	0.970408	0.912111	1.032431	0.341967
ZFYVE21	1.063365	0.961151	1.176449	0.233449

MTHFD1	1.061488	0.977726	1.152425	0.154782
TCL1A	1.068962	1.011304	1.129908	0.018408
ZC3H14	0.975201	0.866632	1.09737	0.676673
TELO2	0.977074	0.918407	1.039489	0.462885
VRK1	1.083296	0.995605	1.178711	0.063213
PSMC1	0.938527	0.831444	1.059402	0.3047
PAPLN	1.065676	0.994865	1.141527	0.069801
RPS6KA5	1.046566	0.969087	1.130238	0.24613
PSMB5	1.140192	1.038481	1.251865	0.005923
YY1	0.965542	0.886924	1.05113	0.418398
ACIN1	0.945308	0.876121	1.019959	0.146957
CCNB1IP1	0.912396	0.840457	0.990492	0.028673
TRIP11	1.054099	0.961537	1.155571	0.261209
APEX1	0.956481	0.876943	1.043234	0.315159
PABPN1	0.934873	0.862936	1.012808	0.09926
ARHGAP5	1.01018	0.966394	1.055951	0.654146
CINP	0.97997	0.851774	1.12746	0.777285
SRP54	1.019353	0.932736	1.114013	0.672252
CHD8	0.970531	0.89821	1.048676	0.449022
PCK2	0.961629	0.88846	1.040824	0.332538
PSMA6	1.086348	0.991382	1.190411	0.075979
NFKBIA	1.023594	0.953436	1.098914	0.519758
PSME2	1.264455	1.156977	1.381916	2.25E-07
BRMS1L	0.902772	0.825823	0.98689	0.02443
REC8	1.022769	0.964475	1.084586	0.452097
TM9SF1	1.007371	0.915843	1.108046	0.879887
SEC23A	0.918443	0.848148	0.994565	0.03625
GMPR2	0.949664	0.859463	1.049331	0.31044
PNN	0.893498	0.820427	0.973076	0.009683
RABGGTA	1.104086	0.978098	1.246303	0.109213
NFATC4	1.307153	1.187255	1.43916	4.85E-08
PLTP	0.91365	0.855232	0.976058	0.007389
PCIF1	1.095877	0.97189	1.235682	0.135037
GSS	1.077325	0.970314	1.196138	0.162899
MMP9	1.013556	0.981928	1.046203	0.405164
TRPC4AP	0.92673	0.858568	1.000304	0.050919
PYGB	0.875315	0.817134	0.937638	0.000148
ABHD12	1.034558	0.943572	1.134318	0.469469
PROCR	1.010753	0.92257	1.107364	0.818382
GINS1	1.117279	1.050049	1.188813	0.000461
NINL	1.160343	1.067374	1.26141	0.000483
CD40	1.042673	0.985454	1.103214	0.146743
ZMYND8	0.975047	0.916553	1.037275	0.423391
SGK2	1.133041	0.978567	1.3119	0.094872
IFT52	1.002547	0.90217	1.114093	0.962301
MYBL2	1.080966	1.015961	1.150129	0.013879
NDRG3	0.966971	0.895374	1.044294	0.39215
SLA2	0.973812	0.919786	1.03101	0.362153
NFATC2	1.020561	0.95773	1.087513	0.530153
PABPC1L	1.038674	0.972373	1.109496	0.259534
STK4	1.006722	0.936638	1.08205	0.855599
SALL4	1.270932	1.195231	1.351427	1.98E-14
ADNP	0.993188	0.927502	1.063526	0.844782
PFDN4	1.035257	0.945894	1.133064	0.451876
DOK5	1.195355	1.112934	1.283879	9.81E-07
CSTF1	1.003159	0.911366	1.104197	0.948641
RAE1	1.083064	0.974158	1.204145	0.14001
TPD52L2	0.961261	0.880999	1.048836	0.374469

DNAJC5	0.983768	0.922891	1.04866	0.615571
CTSZ	0.954669	0.904428	1.007702	0.092604
PRPF6	0.990422	0.896035	1.094751	0.850602
TUBB1	1.040129	0.989231	1.093646	0.124294
PSMA7	1.137982	1.00029	1.294629	0.049488
SLCO4A1	1.013543	0.952575	1.078412	0.670847
NTSR1	1.096402	1.031453	1.165441	0.003138
TCFL5	1.109537	1.023518	1.202784	0.011584
DIDO1	0.996139	0.93323	1.063288	0.907466
SLC17A9	0.887476	0.843304	0.933961	4.59E-06
BIRC7	1.061911	0.963202	1.170736	0.227518
ARFGAP1	0.975737	0.913265	1.042482	0.466871
AVP	0.991804	0.912171	1.078389	0.847182
COL20A1	0.909404	0.825379	1.001983	0.054869
EEF1A2	1.034982	0.973115	1.100782	0.274233
PTK6	1.189848	1.111894	1.273266	4.96E-07
GMEB2	0.936357	0.870664	1.007006	0.076422
CDC25B	1.157218	1.074179	1.246676	0.000121
ISM1	1.181943	1.021802	1.367182	0.02443
RNF24	0.960369	0.890689	1.0355	0.292691
ARFRP1	1.116269	1.025555	1.215006	0.010975
TRIB3	0.952896	0.89855	1.010528	0.107306
RASSF2	1.000482	0.937898	1.067243	0.988327
CSNK2A1	0.990478	0.898231	1.092198	0.84788
CDS2	0.905127	0.846622	0.967674	0.003458
HM13	1.002078	0.908858	1.104861	0.966758
SNPH	1.144356	1.07064	1.223147	7.21E-05
MYLK2	1.01401	0.872988	1.177812	0.855509
SIRPB1	1.049165	1.001592	1.098997	0.042646
SEC23B	1.109129	1.018675	1.207615	0.017022
FERMT1	1.233251	1.142775	1.330889	6.93E-08
PLCB4	1.191297	1.136965	1.248226	1.99E-13
MYL9	0.956061	0.888978	1.028207	0.226064
HCK	1.069331	1.016038	1.12542	0.010172
TM9SF4	1.029943	0.932661	1.137373	0.560013
CRNKL1	1.071952	0.979629	1.172977	0.130517
POFUT1	1.018385	0.936983	1.106859	0.668204
SAMHD1	1.067007	1.024648	1.111117	0.001701
KIF3B	0.989979	0.911005	1.075798	0.812307
NOP56	1.138968	1.04753	1.238388	0.002308
MANBAL	1.079152	0.992408	1.173477	0.074795
IDH3B	1.112484	0.98371	1.258116	0.089454
MAPRE1	0.900425	0.829875	0.976974	0.01175
JAG1	0.982609	0.940233	1.026895	0.435388
CDK5RAP1	1.025255	0.932039	1.127793	0.608071
SNTA1	1.095835	1.019138	1.178304	0.013435
OXT	0.924205	0.852778	1.001614	0.054775
E2F1	0.976106	0.910993	1.045874	0.492345
RPRD1B	0.967338	0.912058	1.025968	0.268698
PXMP4	1.168758	1.070757	1.275729	0.000483
CHMP4B	0.930661	0.850418	1.018474	0.118275
BPI	0.984548	0.957774	1.012071	0.268294
CST3	0.988709	0.950365	1.0286	0.573651
ASIP	0.941193	0.851304	1.040574	0.236657
ACTR5	1.055109	0.973367	1.143715	0.192282
AHCY	0.928617	0.854722	1.008901	0.080032
PPP1R16B	0.962122	0.925518	1.000173	0.051029
FAM83D	1.081221	1.013977	1.152925	0.017142

DHX35	1.029713	0.951822	1.113978	0.465637
DNTTIP1	0.962446	0.859981	1.077119	0.505114
MAP1LC3/	0.948228	0.900245	0.998767	0.044801
PIGU	1.067769	0.990734	1.150793	0.086108
TNNC2	1.114069	1.025927	1.209785	0.01021
ACOT8	1.196096	1.057943	1.352289	0.004244
ZNF516	0.946758	0.899629	0.996355	0.035717
ADNP2	0.935115	0.876077	0.998132	0.043783
USP14	0.944497	0.8749	1.019629	0.143687
VAPA	0.963656	0.876128	1.059929	0.446065
METTL4	1.155769	1.046502	1.276444	0.004277
LPIN2	1.009546	0.94474	1.078798	0.778965
SMCHD1	0.943128	0.891914	0.997283	0.039835
MYOM1	1.105408	1.025797	1.191198	0.008593
MYL12A	1.077125	0.974118	1.191023	0.147432
CEP76	1.033101	0.938116	1.137704	0.508111
CEP192	1.03076	0.972713	1.09227	0.305624
RNMT	0.972019	0.905182	1.043792	0.434924
SMAD7	1.045124	0.998407	1.094027	0.058542
RNF125	1.014005	0.951665	1.080428	0.667476
ANKRD12	0.970466	0.900704	1.045632	0.430916
POLI	1.100532	1.012519	1.196195	0.02429
MIB1	0.95829	0.901509	1.018648	0.171592
RBBP8	0.970305	0.891181	1.056455	0.487327
RIOK3	0.92131	0.858325	0.988916	0.023302
CSTF2	1.223476	1.110078	1.348457	4.82E-05
VSIG1	1.078163	0.923927	1.258147	0.339347
PSMD10	1.108259	1.009975	1.216108	0.030049
ATG4A	1.026418	0.945134	1.114693	0.535621
STS	1.045332	0.963977	1.133554	0.283508
TBL1X	0.918156	0.860478	0.9797	0.009894
GPR143	0.936744	0.82635	1.061887	0.307072
PGRMC1	1.236507	1.134382	1.347826	1.39E-06
POLA1	1.055944	0.983069	1.134222	0.135714
MID1	1.203604	1.108599	1.306752	9.99E-06
NKAP	1.048736	0.94699	1.161415	0.360767
RHOXF1	1.242343	1.111914	1.388072	0.000126
NXT2	1.168637	1.090332	1.252566	1.06E-05
ALG13	0.927622	0.851873	1.010106	0.083879
PRPS2	1.054067	0.975518	1.138941	0.182647
TLR8	1.038099	0.990264	1.088243	0.120307
MOSPD1	1.124377	1.038708	1.217112	0.003741
AMMECR1	1.075683	1.011546	1.143887	0.02002
CHRDL1	1.108321	1.040668	1.180371	0.001372
WDR13	0.965112	0.86403	1.07802	0.529292
SUV39H1	1.00528	0.910547	1.109868	0.916953
SRPX	1.17561	1.024515	1.348989	0.021166
XIAP	0.957293	0.886957	1.033207	0.262307
STAG2	0.943002	0.879946	1.010577	0.096514
ATP11C	1.019157	0.944311	1.099935	0.625839
MCF2	0.886354	0.756586	1.038379	0.135262
ABCD1	1.016305	0.945664	1.092222	0.659928
CCDC22	1.021687	0.929441	1.123089	0.656759
CACNA1F	1.241465	1.128565	1.365659	8.74E-06
SYP	1.26003	1.09447	1.450633	0.0013
PLP2	0.967198	0.9043	1.034471	0.33098
BMX	1.057129	0.996684	1.121239	0.0644
PLS3	1.255464	1.17368	1.342946	3.60E-11

RENBP	0.929572	0.858799	1.006178	0.07068
ELF4	0.988399	0.916319	1.066149	0.762626
SMARCA1	0.975436	0.912538	1.042669	0.464588
MTMR8	1.086129	1.016178	1.160895	0.014997
ASB9	1.102582	1.03145	1.17862	0.004105
ZC3H12B	1.088496	0.978046	1.21142	0.120345
RBBP7	1.008299	0.906963	1.120958	0.878448
KCND1	0.94021	0.865677	1.02116	0.143451
SLC25A14	1.246531	1.114029	1.394793	0.000121
FMR1	0.961733	0.896461	1.031758	0.276554
PIM2	1.054293	0.982728	1.131069	0.140435
SCML2	0.869439	0.823838	0.917564	3.58E-07
SLC35A2	0.988622	0.914786	1.068419	0.772636
PQBP1	0.97948	0.860239	1.11525	0.754247
PCSK1N	0.994104	0.90207	1.095528	0.905037
EMD	0.945158	0.849284	1.051855	0.301341
TAZ	1.000303	0.906907	1.103317	0.995165
PGK1	0.973598	0.87578	1.082343	0.620408
GATA1	1.01156	0.973854	1.050725	0.553173
MAGT1	0.944682	0.868414	1.027648	0.185179
SMS	0.927231	0.841885	1.021229	0.125136
PHEX	1.122552	0.972234	1.296112	0.11501
UBL4A	1.053108	0.966856	1.147055	0.23528
CD99L2	1.055186	0.977605	1.138923	0.167998
EEA1	1.054354	0.978497	1.136091	0.164726
RP2	0.945215	0.877277	1.018415	0.138743
USP11	1.069404	0.985716	1.160198	0.106542
PCYT1B	1.19429	1.12185	1.271408	2.68E-08
HTATSF1	1.018885	0.922042	1.1259	0.713501
CD40LG	1.035098	0.96515	1.110116	0.333884
TIMP1	0.992982	0.943867	1.044652	0.785519
GABRE	1.022225	0.971364	1.07575	0.398563
FGD1	1.033505	0.897415	1.190232	0.64733
PIN4	1.126579	1.002103	1.266517	0.046029
PORCN	1.136184	1.015707	1.270952	0.025584
MAGED2	1.016725	0.947125	1.091439	0.646633
RBM3	0.886293	0.813278	0.965863	0.005927
KLF8	1.257497	1.136385	1.391516	9.23E-06
SYTL4	1.186713	1.118441	1.259153	1.49E-08
ZDHHC15	1.31671	1.178854	1.470687	1.08E-06
CENPI	1.085671	1.010202	1.166777	0.025346
GLA	0.908496	0.825873	0.999384	0.048538
ARMCX3	0.969844	0.917144	1.025572	0.282742
BEX4	1.031087	0.980334	1.084469	0.234551
NDFIP2	0.958963	0.896613	1.025648	0.221842
TNFSF13B	1.025125	0.976117	1.076594	0.320792
FNDC3A	0.913698	0.856002	0.975284	0.006688
MLNR	0.912186	0.85235	0.976223	0.007928
CDADC1	0.993091	0.920426	1.071493	0.858072
CAB39L	1.005661	0.898054	1.126161	0.922119
KLF5	1.104348	1.053537	1.15761	3.63E-05
STK24	0.848624	0.789725	0.911914	7.73E-06
ACP5	1.008444	0.941425	1.080235	0.810594
DNAJC3	0.98873	0.924171	1.0578	0.742181
ARHGEF7	0.946545	0.892348	1.004035	0.067833
FGF9	1.18419	1.06638	1.315015	0.001567
PARP4	0.974513	0.916506	1.036192	0.409637
MRPS31	1.057105	0.954563	1.170661	0.286094

SLC25A15	1.086562	1.009698	1.169277	0.026569
KPNA3	0.930443	0.857701	1.009354	0.082601
FLT1	1.119207	0.996498	1.257026	0.057333
DGKH	1.20446	1.122194	1.292756	2.55E-07
KATNAL1	0.93225	0.86038	1.010123	0.086549
INTS6	1.007244	0.940347	1.078899	0.836917
DHRS12	0.965798	0.905175	1.030481	0.292722
TSC22D1	0.948663	0.892719	1.008113	0.08924
CLN5	1.032583	0.95392	1.117732	0.427727
OLFM4	1.041336	1.008689	1.07504	0.012692
MSLN	0.945345	0.920671	0.97068	3.11E-05
MGRN1	0.846236	0.779524	0.918657	6.75E-05
ZNF629	0.945373	0.882346	1.012903	0.11054
TRADD	1.126451	1.043289	1.216241	0.002343
HSF4	1.14946	1.054797	1.252618	0.00149
CORO1A	0.935141	0.873419	1.001225	0.054252
MAPK3	1.041465	0.937292	1.157215	0.449899
GDPD3	1.100211	1.013904	1.193865	0.021949
ELMO3	1.186774	1.093018	1.288571	4.54E-05
PHKB	1.021595	0.949377	1.099307	0.567878
LYRM1	0.990124	0.890037	1.101467	0.855166
NUTF2	0.994854	0.900791	1.098739	0.918909
NUP93	1.022601	0.930087	1.124317	0.644129
CENPT	1.219391	1.112916	1.336053	2.09E-05
TSNAXIP1	1.25072	1.074151	1.456314	0.003962
NFAT5	0.896839	0.847274	0.949303	0.000174
LONP2	1.035694	0.962091	1.114927	0.351095
N4BP1	0.980329	0.907005	1.05958	0.61645
ARL2BP	1.344987	1.164736	1.553133	5.41E-05
PLLP	1.445114	1.237089	1.688119	3.44E-06
CCL22	1.252067	1.14385	1.370521	1.09E-06
DHODH	1.079355	0.994038	1.171994	0.06912
CCL17	1.088725	0.927852	1.27749	0.297398
CTCF	1.060244	0.958458	1.172839	0.255954
ACD	1.127026	1.005011	1.263855	0.04081
POLR2C	1.030466	0.925361	1.147509	0.584554
PARD6A	1.152822	1.032546	1.287109	0.011417
ZNF821	0.931305	0.851441	1.018661	0.119759
MMP15	1.119998	1.061402	1.181829	3.57E-05
CYB5B	1.066823	0.977347	1.16449	0.147818
NME3	1.073816	0.988809	1.166131	0.09055
NDRG4	1.059866	0.953677	1.177878	0.2804
PSMD7	1.120426	0.992165	1.265269	0.06678
SETD6	1.040872	0.943293	1.148545	0.425097
SLC38A7	1.264593	1.134872	1.409142	2.13E-05
VAC14	0.977543	0.898392	1.063667	0.598029
HAS3	0.999388	0.883652	1.130283	0.992224
COG4	1.07638	0.997742	1.161216	0.05723
SMPD3	1.185423	1.093054	1.285597	3.96E-05
SLC7A6OS	1.035573	0.949426	1.129536	0.43022
SLC7A6	1.077193	0.999966	1.160383	0.050104
PLA2G15	1.076841	0.98814	1.173505	0.091423
ESRP2	1.007825	0.940939	1.079465	0.823957
WDR59	0.990669	0.915472	1.072041	0.815936
MON1B	0.964631	0.882593	1.054294	0.427155
AXIN1	1.015557	0.935157	1.10287	0.713738
HCFC1R1	0.886248	0.810719	0.968813	0.007881
MLYCD	1.027546	0.957724	1.102459	0.449135

MPG	0.930858	0.854244	1.014344	0.102054
HSDL1	0.953276	0.882347	1.029906	0.225136
TAF1C	1.030402	0.950969	1.11647	0.464348
NAGPA	1.072601	0.990971	1.160955	0.082672
WFDC1	0.777752	0.700475	0.863554	2.51E-06
COTL1	0.955393	0.901921	1.012035	0.120458
USP10	0.921239	0.850746	0.997575	0.043409
CRISPLD2	0.92707	0.877274	0.979693	0.007182
TSC2	0.965067	0.881165	1.056959	0.443536
ZNF500	1.122055	1.028318	1.224335	0.009672
NME4	0.872513	0.801226	0.950144	0.001713
ABCC1	0.982918	0.927497	1.04165	0.560645
NOMO3	1.019124	0.928275	1.118865	0.690888
LMF1	1.057914	0.96063	1.16505	0.252675
FOXF1	0.993049	0.880754	1.119662	0.909299
MTHFSD	1.069884	0.98226	1.165325	0.121284
CLCN7	0.952394	0.890471	1.018623	0.155015
HAGHL	0.823557	0.766225	0.885179	1.34E-07
SLC7A5	1.0156	0.963801	1.070183	0.562224
METRN	0.876853	0.815019	0.943378	0.000428
FBXO31	0.932401	0.859744	1.011199	0.090852
STUB1	0.956442	0.864954	1.057608	0.385318
RHBDL1	1.067659	0.98611	1.155951	0.106323
NUBP1	0.874562	0.790713	0.967302	0.009149
UBE2I	0.984894	0.89092	1.08878	0.766086
MEFV	1.040484	0.998012	1.084765	0.061991
CRYM	1.254698	1.165971	1.350177	1.33E-09
EEF2K	0.997737	0.934266	1.06552	0.946139
GSPT1	1.049725	0.966664	1.139923	0.24857
ZNF174	1.096751	0.991264	1.213463	0.07347
CLUAP1	1.080448	0.98181	1.188996	0.113164
UBFD1	0.985267	0.916357	1.059358	0.688252
PRSS33	0.770714	0.689472	0.86153	4.60E-06
EARS2	1.094304	1.014006	1.180961	0.020467
GGA2	0.993753	0.931642	1.060005	0.849073
AQP8	0.928181	0.837937	1.028144	0.153258
CPPED1	0.997918	0.936103	1.063814	0.949054
USP31	1.246103	1.157772	1.341174	4.49E-09
HMOX2	1.043209	0.949841	1.145754	0.376562
DNAJA3	1.154812	1.059352	1.258874	0.001077
BFAR	0.996608	0.891363	1.114279	0.952417
RRN3P2	1.009526	0.913379	1.115795	0.852705
RBL2	1.010743	0.942675	1.083725	0.763879
QPRT	0.941439	0.884647	1.001877	0.057317
XYLT1	1.09157	1.033058	1.153396	0.001827
PYCARD	1.089796	1.017938	1.166727	0.01348
RPGRIP1L	1.119082	0.975318	1.284037	0.108774
MAZ	0.926678	0.856078	1.0031	0.059646
STX4	0.970148	0.872209	1.079084	0.576724
CDIPT	1.199289	1.090143	1.319363	0.000189
BCKDK	1.237617	1.123438	1.3634	1.58E-05
NOMO1	1.035192	0.956651	1.120181	0.390268
IL21R	1.030713	0.970887	1.094226	0.321422
SYT17	1.095083	1.004963	1.193284	0.038177
TMC5	1.167947	1.062771	1.283532	0.001262
RNF40	0.913547	0.850444	0.981332	0.013287
AQP9	1.010155	0.973161	1.048554	0.595576
AAGAB	1.106317	1.007244	1.215134	0.034793

IQCH	1.203658	1.03178	1.404168	0.018377
LACTB	0.973264	0.897453	1.055479	0.512478
CORO2B	0.857351	0.778514	0.944172	0.001765
CSK	0.989912	0.906912	1.080507	0.820472
HERC1	0.993457	0.945018	1.044378	0.796863
TRIP4	1.053612	0.946836	1.172429	0.338099
MTFMT	1.201228	1.079567	1.336599	0.000765
ACSBG1	1.049003	0.915628	1.201807	0.49049
IGDCC4	0.869464	0.767185	0.985379	0.028478
RAB11A	1.075406	0.962101	1.202054	0.200614
CTSH	1.090897	1.022118	1.164305	0.008835
TTC23	1.013923	0.921606	1.115487	0.776505
CD276	1.059923	1.002387	1.120762	0.040983
FAH	1.024892	0.939054	1.118577	0.581678
RPAP1	1.06307	0.980679	1.152383	0.137289
HOMER2	0.885036	0.827924	0.946088	0.000333
EHD4	1.133351	1.063167	1.20817	0.000124
TMEM87A	0.939597	0.8741	1.010001	0.091024
CEP152	1.053193	0.977193	1.135103	0.175026
ATP8B4	0.998889	0.960635	1.038668	0.955524
DTWD1	1.046834	0.947597	1.156464	0.367742
TGM5	1.032918	0.978327	1.090555	0.242382
FAM189A1	1.163059	1.072134	1.261696	0.000276
GABPB1	0.956792	0.889661	1.028989	0.234036
BMF	0.946769	0.89871	0.997399	0.039595
DMXL2	0.994407	0.95233	1.038344	0.799314
SCG3	1.030707	0.924089	1.149627	0.587206
DNAJC17	1.132536	1.026143	1.24996	0.01341
EIF3J	0.877089	0.796114	0.9663	0.007964
SPG11	0.993193	0.930116	1.060549	0.838342
RHOV	1.037714	0.930883	1.156804	0.504221
VPS18	0.951713	0.879141	1.030275	0.221347
OIP5	1.087068	0.985787	1.198756	0.094312
SLC30A4	1.175047	1.083051	1.274857	0.000105
MYEF2	0.97824	0.926108	1.033307	0.431067
SGK3	0.97195	0.888609	1.063108	0.533929
CSPP1	0.977015	0.906547	1.05296	0.542646
ZDHHC2	0.93694	0.882915	0.994271	0.031589
BRF2	1.036164	0.936411	1.146543	0.49155
TRIM35	1.185551	1.100269	1.277443	7.87E-06
ZFAND1	0.937441	0.854919	1.027928	0.169416
CA2	1.022096	0.984911	1.060684	0.247738
FZD3	1.203588	1.101571	1.315054	4.12E-05
INTS9	1.046291	0.955906	1.145221	0.326259
RIPK2	0.863117	0.806947	0.923198	1.81E-05
NBN	0.957605	0.893083	1.026788	0.223535
DECR1	1.183151	1.085671	1.289384	0.000126
SFRP1	1.133864	0.964591	1.332843	0.127775
LAPTM4B	1.113563	1.072537	1.156158	1.95E-08
UBE2W	1.004456	0.933448	1.080866	0.905389
POP1	1.241161	1.138784	1.352742	8.71E-07
NIPAL2	1.106869	1.039858	1.178198	0.00144
IKBKB	1.016594	0.949529	1.088395	0.63647
PLAT	0.914146	0.781557	1.069228	0.261548
DKK4	0.993561	0.893302	1.105073	0.905254
STK3	1.060478	0.962212	1.168779	0.236592
GDAP1	1.466124	1.269797	1.692806	1.83E-07
RAB2A	0.960271	0.865501	1.065417	0.444452

EIF3E	0.894434	0.817261	0.978893	0.015378
NDRG1	0.922376	0.867751	0.98044	0.009483
IL7	1.209015	1.114138	1.311971	5.31E-06
ARMC1	1.067074	0.977647	1.164682	0.146017
TRPS1	1.086435	1.041091	1.133755	0.000138
SPAG1	1.19843	1.105947	1.298647	9.98E-06
CHRAC1	1.003476	0.903471	1.114549	0.948353
NCALD	0.952701	0.8802	1.031174	0.230205
SNX16	0.96405	0.887126	1.047645	0.388181
UBR5	0.952925	0.900361	1.008557	0.095788
GSDMD	1.002829	0.932181	1.078831	0.939587
EEF1D	1.014045	0.915198	1.123567	0.789834
SQLE	1.215758	1.125325	1.313458	7.27E-07
SH2D4A	0.975217	0.875997	1.085676	0.646666
INTS10	1.090988	0.994222	1.197173	0.06611
ERI1	1.034398	0.95019	1.12607	0.435021
SLC39A14	1.211342	1.133902	1.294071	1.28E-08
MTMR9	1.226567	1.125978	1.336142	2.90E-06
LEPROTL1	0.955467	0.873684	1.044905	0.318367
DCTN6	0.950727	0.864435	1.045633	0.297962
R3HCC1	1.159007	1.043759	1.286979	0.005755
GSR	1.028599	0.957578	1.104887	0.439835
TNFRSF10.	0.919287	0.864062	0.97804	0.007758
UBXN8	1.213785	1.102135	1.336746	8.31E-05
PPP2CB	0.96253	0.877892	1.055327	0.416083
ERICH1	1.000248	0.906554	1.103626	0.996051
TUSC3	1.214607	1.109375	1.329822	2.61E-05
KLHDC4	1.042918	0.959476	1.133617	0.323313
MCM4	1.106535	1.037589	1.180063	0.002042
KCTD9	0.977811	0.907876	1.053132	0.553411
ASAH1	0.95195	0.875663	1.034883	0.247915
BNIP3L	0.976213	0.913442	1.043298	0.477729
MAN2B1	0.920164	0.848343	0.998065	0.044785
KCNN4	0.963202	0.899611	1.031287	0.281974
TULP2	1.014277	0.965179	1.065873	0.575497
NUCB1	1.051716	0.954948	1.15829	0.305886
DHDH	0.994368	0.917349	1.077853	0.890778
GYS1	0.988387	0.914	1.068828	0.769828
MAP4K1	1.145043	1.053764	1.244227	0.001396
ECH1	1.166511	1.063481	1.279521	0.001096
HNRNPL	0.930488	0.846148	1.023234	0.137233
NFKBIB	0.877469	0.804955	0.956516	0.002976
LHB	0.971497	0.885501	1.065846	0.540881
SARS2	1.156138	1.025967	1.302824	0.017284
SNRNP70	1.037993	0.952753	1.130859	0.393705
CLPTM1	0.951858	0.86233	1.05068	0.327577
RELB	0.925721	0.873293	0.981297	0.009468
LIN7B	1.285838	1.14395	1.445325	2.50E-05
FCGRT	0.980243	0.927746	1.03571	0.477348
PIH1D1	1.193339	1.072572	1.327705	0.001167
CKM	1.000433	0.927283	1.079354	0.99108
ARHGEF18	0.986792	0.917941	1.060808	0.718624
PPP1R13L	1.344474	1.2072	1.497357	7.17E-08
PEX11G	0.986762	0.925971	1.051544	0.681236
ERCC2	1.238034	1.133404	1.352324	2.14E-06
DOT1L	0.987817	0.908545	1.074004	0.773952
PLEKHJ1	1.316757	1.1852	1.462917	3.00E-07
KLC3	1.167161	1.047838	1.300073	0.004966

CD37	1.10714	1.0135	1.209432	0.023985
SF3A2	0.880744	0.808635	0.959285	0.003571
AMH	1.118833	1.044639	1.198296	0.001339
LYL1	0.860967	0.7978	0.929136	0.000118
OAZ1	1.069991	0.94997	1.205176	0.265086
TRMT1	1.03466	0.945649	1.132049	0.457857
STX10	0.962053	0.860505	1.075586	0.496691
RETN	0.984471	0.952708	1.017293	0.349608
FCER2	0.965	0.917401	1.015069	0.16745
DMPK	1.022149	0.938827	1.112867	0.613586
TBC1D17	1.054041	0.963134	1.153529	0.252746
IL4I1	1.162285	1.100939	1.227049	5.45E-08
CCDC130	1.037595	0.929526	1.158228	0.510759
PTOV1	0.963291	0.876714	1.058418	0.436361
SGTA	0.802008	0.734477	0.875748	8.82E-07
KIR3DX1	1.170942	1.073517	1.277208	0.00037
LILRB1	1.064436	1.012302	1.119256	0.014802
MED25	0.945634	0.857424	1.042919	0.263204
LILRA1	1.068022	1.020666	1.117575	0.004456
SNAPC2	0.87631	0.790988	0.970835	0.011527
TIMM44	1.03258	0.945485	1.127697	0.475779
CCDC61	1.102569	1.028045	1.182496	0.006246
IL27RA	0.980251	0.912873	1.052602	0.583012
ASF1B	1.165614	1.091468	1.244796	4.88E-06
TNNT1	1.210014	1.154195	1.268531	2.55E-15
VRK3	1.030876	0.932341	1.139825	0.553019
FAM32A	1.139178	1.027581	1.262894	0.013242
MED26	0.94582	0.88102	1.015386	0.123975
OLFM2	1.017769	0.921323	1.124311	0.728784
RASAL3	1.019481	0.943196	1.101937	0.626812
AKAP8	1.021971	0.939847	1.11127	0.611119
ILVBL	1.086887	0.990739	1.192365	0.077887
ZNF419	1.059131	0.969427	1.157134	0.203265
SYDE1	0.968871	0.871226	1.07746	0.559582
AURKC	0.956629	0.873234	1.047988	0.340708
POP4	1.317857	1.160019	1.497171	2.23E-05
CCNE1	1.114173	1.029186	1.206179	0.007572
PDCD5	1.016145	0.926055	1.115	0.735263
ANKRD27	0.988845	0.931568	1.049645	0.712532
RPS16	0.901285	0.828364	0.980626	0.015759
TIMM50	1.027192	0.927547	1.137542	0.606323
FBL	0.925662	0.847257	1.011323	0.08715
DYRK1B	0.874617	0.803368	0.952185	0.002001
CLC	0.94134	0.917887	0.965393	2.65E-06
GPI	0.829463	0.767817	0.896059	2.08E-06
AKT2	0.981759	0.909549	1.059702	0.636721
PLD3	0.992413	0.944454	1.042807	0.763136
PRX	0.918313	0.856903	0.984124	0.015816
PIAS4	0.987727	0.906326	1.076439	0.778398
NUMBL	1.079677	0.99282	1.174133	0.073202
EBI3	0.883951	0.816488	0.956988	0.002324
SHD	0.90992	0.862606	0.959829	0.000531
TBCB	1.171179	1.060444	1.293478	0.00182
FSD1	1.057663	0.928105	1.205305	0.400419
POLR2I	1.222788	1.137019	1.315027	5.94E-08
CLIP3	0.88623	0.841807	0.932998	4.16E-06
SLC1A5	1.121445	1.058168	1.188507	0.00011
PRKD2	1.043616	0.965018	1.128616	0.285236

TJP3	1.155631	0.994882	1.342352	0.058382
APLP1	0.96398	0.91878	1.011404	0.134346
CCDC9	0.845925	0.775449	0.922806	0.000163
HNRNPUL	0.941504	0.84607	1.047702	0.268988
FZR1	0.98298	0.913927	1.05725	0.644128
BBC3	0.940399	0.881493	1.003242	0.062617
TGFB1	1.00625	0.931514	1.086982	0.874272
DENND3	0.983087	0.928013	1.04143	0.561993
CEACAM4	0.978878	0.939164	1.02027	0.312354
MRPL4	1.010123	0.913207	1.117324	0.844831
SIGLEC8	1.039354	0.92705	1.165264	0.508218
CD79A	0.999894	0.951994	1.050205	0.996631
RPS19	0.928115	0.852713	1.010184	0.084422
NKG7	1.0632	1.006716	1.122853	0.027787
ICAM5	0.893623	0.843842	0.946341	0.00012
ETFB	1.468326	1.308114	1.64816	7.21E-11
CD33	1.073435	1.011452	1.139217	0.019533
TYK2	1.057046	0.978945	1.141377	0.156599
CDC37	0.927637	0.825523	1.042382	0.206815
NAPA	1.058698	0.957958	1.170032	0.263539
RABAC1	1.224581	1.105609	1.356356	0.000102
PTPRS	1.020449	0.974323	1.068759	0.391041
CNFN	1.254529	1.165896	1.3499	1.31E-09
MEGF8	1.012852	0.949695	1.080209	0.697461
KDELR1	0.970022	0.879299	1.070105	0.5435
CYTH2	1.101569	1.002469	1.210465	0.0443
GRWD1	1.121197	1.029587	1.220958	0.008528
GRIN2D	1.219424	1.143705	1.300156	1.32E-09
CLEC11A	0.969532	0.93606	1.0042	0.084317
CCDC114	1.230481	1.092522	1.385862	0.00063
CARD8	1.051095	0.986777	1.119605	0.121917
LIG1	1.122453	1.031514	1.221409	0.007368
SIGLEC6	1.056902	1.000218	1.116799	0.049102
ZNF175	1.120733	1.04244	1.204907	0.002036
PLA2G4C	1.129594	1.025129	1.244704	0.013846
SIGLEC5	1.126976	1.014121	1.252389	0.02639
HAS1	0.937606	0.886911	0.991199	0.023106
RAB3D	1.084437	1.026461	1.145687	0.003833
DBP	0.992012	0.911259	1.079922	0.853131
TMEM205	1.207174	1.099511	1.325379	7.80E-05
CAPS	1.082216	0.985974	1.187853	0.096372
FAM83E	0.99591	0.897211	1.105466	0.938643
RASIP1	1.217854	1.12251	1.321296	2.15E-06
BCAT2	1.119486	1.030866	1.215724	0.007309
MIER2	0.977187	0.895419	1.066422	0.60474
PLEKHA4	1.218223	1.100584	1.348435	0.000139
PPP2R1A	0.971348	0.884772	1.066396	0.541645
TNPO2	1.005533	0.930826	1.086235	0.888598
GCDH	1.004191	0.920069	1.096004	0.925353
LILRB5	0.991248	0.857687	1.145607	0.905239
KLF1	0.994738	0.958749	1.032078	0.77901
DNASE2	0.889916	0.835847	0.947483	0.000266
MAST1	1.022142	0.920425	1.1351	0.682172
LENG1	1.030304	0.923178	1.14986	0.594059
PRPF31	1.260597	1.1103	1.43124	0.00035
TFPT	1.065177	0.944798	1.200894	0.302104
JAK3	0.951916	0.899932	1.006904	0.085462
RPL18A	0.916289	0.84976	0.988027	0.023017

SLC5A5	0.988228	0.872233	1.11965	0.852535
KCNN1	1.107664	1.03139	1.18958	0.004969
ARRDC2	0.968081	0.894804	1.047358	0.419217
RAB3A	1.152213	1.058884	1.253768	0.001011
ISYNA1	0.91161	0.854131	0.972957	0.005352
ELL	0.946137	0.882231	1.014673	0.120729
CRTC1	1.026462	0.948962	1.110292	0.51435
COPE	0.918627	0.82555	1.022197	0.119429
DDX49	0.977558	0.880897	1.084825	0.669174
ETV2	0.89943	0.810348	0.998306	0.046391
ARMC6	1.101124	1.01834	1.190639	0.015705
TMEM147	1.106275	1.017831	1.202404	0.017516
HAMP	1.03939	0.939029	1.150478	0.455844
USF2	0.986336	0.885392	1.098789	0.802775
LSR	1.090084	1.002548	1.185263	0.043431
FKBP8	0.927608	0.859745	1.000829	0.052551
ZNF14	0.950164	0.897243	1.006206	0.080398
SCN1B	0.846739	0.77259	0.928004	0.000374
PBX4	0.889187	0.845492	0.93514	4.92E-06
ERF	0.874818	0.816799	0.936958	0.000134
GSK3A	0.949415	0.846536	1.064797	0.375047
ATP13A1	1.089283	0.98854	1.200293	0.084136
ZNF574	0.927807	0.858746	1.002421	0.057605
GRIK5	0.857575	0.822332	0.894328	7.17E-13
SIPA1L3	1.006081	0.939468	1.077419	0.862284
ZNF85	1.034217	0.943649	1.133477	0.471811
ETHE1	0.953192	0.872905	1.040864	0.285594
CADM4	1.088236	0.970213	1.220616	0.148828
AVL9	1.003372	0.938545	1.072677	0.921309
RUNDC3B	1.053245	0.937865	1.18282	0.380855
GTPBP10	1.056165	0.95378	1.16954	0.293553
RASA4	0.827114	0.726147	0.94212	0.004269
CDK6	0.999511	0.948605	1.05315	0.985379
PMPCB	0.980157	0.889321	1.080271	0.686276
DNAJC2	0.930627	0.850839	1.017896	0.115926
TFPI2	1.134295	1.043593	1.23288	0.003042
BET1	1.066215	0.96179	1.181978	0.222786
NAMPT	0.940748	0.892939	0.991118	0.02172
PIK3CG	1.004179	0.946687	1.065163	0.889741
PON2	1.031851	0.980638	1.08574	0.227363
ITGB8	1.037356	0.953128	1.129026	0.395963
HBP1	0.927547	0.870013	0.988886	0.021333
DUS4L	1.073153	0.945707	1.217774	0.273717
SP4	0.976158	0.907442	1.050077	0.517028
WDR91	1.099572	1.027626	1.176556	0.005973
DNAH11	0.897759	0.764696	1.053977	0.187608
CBLL1	0.966761	0.871738	1.072141	0.521922
MTPN	0.930409	0.854274	1.013331	0.097732
MPP6	0.995983	0.932918	1.063312	0.90401
ZC3HAV1	0.93044	0.866017	0.999655	0.048909
TTC26	1.071639	0.974865	1.17802	0.151914
OGDH	1.024219	0.959271	1.093565	0.474032
ADAP1	1.077981	0.997459	1.165003	0.057996
TFEC	0.99164	0.942802	1.043008	0.744585
CAV2	0.94055	0.862438	1.025737	0.165891
CAV1	0.914952	0.867977	0.964469	0.000949
MET	1.118845	1.034669	1.209869	0.004893
RNF32	0.980026	0.899402	1.067877	0.645057

LMBR1	0.953771	0.889816	1.022323	0.181375
HOXA1	1.186917	1.121253	1.256426	3.61E-09
DNAJB6	0.919007	0.856209	0.986411	0.019344
HOXA2	1.160507	1.104055	1.219845	4.90E-09
HOXA3	1.152393	1.111993	1.194261	6.70E-15
LFNG	1.051604	1.007362	1.097789	0.021765
HOXA5	1.104723	1.07791	1.132202	1.95E-15
HOXA6	1.121065	1.09259	1.150283	3.16E-18
IQCE	1.283453	1.18149	1.394215	3.45E-09
TSPAN12	1.05502	0.939218	1.185099	0.366591
SSBP1	0.968488	0.882103	1.063331	0.501756
HOXA13	1.133538	1.062203	1.209664	0.000157
HIBADH	1.120543	1.038883	1.208623	0.003198
TAX1BP1	0.939547	0.866369	1.018907	0.13175
CPVL	1.092172	1.050079	1.135952	1.10E-05
CHN2	1.074062	0.970946	1.188129	0.165315
GRB10	0.971144	0.927299	1.017061	0.214143
ABHD11	1.346552	1.238996	1.463445	2.46E-12
FKBP14	1.072599	1.016902	1.131347	0.009995
PLEKHA8	1.04338	0.982183	1.10839	0.168509
STX1A	1.007256	0.958213	1.058809	0.776494
NOD1	0.941448	0.87821	1.009239	0.088992
CRHR2	0.939134	0.825869	1.067933	0.338239
EPHB6	0.992965	0.930574	1.059538	0.831142
CASP2	0.939295	0.87682	1.006221	0.074529
CHCHD2	0.938894	0.842117	1.046793	0.255949
CCL24	0.982298	0.912043	1.057966	0.637126
HSPB1	1.070432	1.023873	1.119109	0.002702
NPTX2	0.961671	0.916655	1.008898	0.110089
PDAP1	1.03539	0.921561	1.163279	0.558361
BUD31	0.934253	0.839261	1.039996	0.213823
PTCD1	1.069415	0.963469	1.18701	0.207376
CYP3A5	1.074983	0.978782	1.180638	0.130632
ZKSCAN1	1.018911	0.956665	1.085206	0.56023
EIF3B	1.01425	0.927645	1.108941	0.756019
SNX8	1.256382	1.138619	1.386326	5.49E-06
NUDT1	1.219922	1.108413	1.342649	4.81E-05
TAF6	1.076605	0.968191	1.197159	0.172875
WASL	0.982572	0.911258	1.059467	0.647425
AIMP2	1.005771	0.904312	1.118613	0.915531
TFR2	0.994159	0.94986	1.040524	0.801112
FSCN3	1.030928	0.939481	1.131277	0.520407
MOSPD3	1.17358	1.083705	1.270908	8.23E-05
PCOLCE	1.262669	1.113497	1.431825	0.000277
FBXO24	1.05714	0.956002	1.168977	0.278807
RBM28	1.054973	0.976102	1.140217	0.177065
USP42	0.906642	0.845651	0.972032	0.00581
IMPDH1	0.917668	0.854492	0.985515	0.01823
AGFG2	1.069715	0.983778	1.163159	0.114751
LSM5	1.044693	0.936953	1.164823	0.431096
SERPINE1	1.013872	0.967076	1.062933	0.567718
AP1S1	1.135199	1.065951	1.208945	7.86E-05
C1GALT1	1.00606	0.927856	1.090855	0.883653
PLOD3	0.965216	0.892748	1.043568	0.373977
RPA3	1.178994	1.058133	1.31366	0.002846
ZNHIT1	1.076701	0.948889	1.221729	0.251695
CLDN15	0.948016	0.881352	1.019723	0.151294
GLCCI1	1.048912	0.981196	1.121301	0.16078

PHF14	1.012961	0.932922	1.099867	0.759114
NRF1	0.947128	0.877237	1.022587	0.164868
TMEM106I	1.030602	0.947005	1.121578	0.484937
EZH2	0.984936	0.923488	1.050471	0.644202
ZNF862	1.048892	0.965305	1.139717	0.259922
SFRP4	1.164845	1.024004	1.325056	0.020301
MEST	0.871697	0.83712	0.907702	2.94E-11
ANKMY2	0.883749	0.817074	0.955864	0.002016
TSPAN13	1.033292	0.980182	1.089279	0.223809
RARRES2	0.787931	0.713654	0.86994	2.38E-06
AGR2	0.992709	0.922706	1.068022	0.844496
AHR	1.087246	1.027923	1.149993	0.003478
CHCHD3	1.03077	0.921868	1.152538	0.59475
GIMAP2	0.991601	0.926687	1.061062	0.807098
TMEM176I	0.937652	0.900225	0.976635	0.001951
PSMA2	0.994115	0.922173	1.07167	0.877608
MRPL32	1.051667	0.949128	1.165283	0.335824
BLVRA	0.927695	0.880069	0.977899	0.005253
STAG3L4	0.870844	0.788616	0.961647	0.00628
RHEB	0.873285	0.788723	0.966913	0.009121
PRKAG2	0.867073	0.795069	0.945598	0.001262
AEBP1	0.998649	0.951804	1.047799	0.956003
POLD2	1.121842	1.035726	1.215119	0.004782
BCL7B	0.873129	0.801044	0.951701	0.002029
YKT6	0.962638	0.883809	1.048498	0.382379
TBL2	1.09464	1.003677	1.193847	0.041064
CLIP2	1.101466	1.047716	1.157974	0.000153
EIF4H	0.921408	0.849158	0.999806	0.049458
LIMK1	1.123049	1.034273	1.219445	0.005744
FKTN	1.135584	1.036849	1.243721	0.00615
FSD1L	1.232868	1.118691	1.358699	2.42E-05
CNTNAP3	1.15056	1.049796	1.260995	0.002707
SPIN1	1.050877	0.973489	1.134417	0.203544
PRUNE2	1.185087	1.084285	1.295261	0.000181
MEGF9	0.984185	0.931059	1.040343	0.573399
TRIM14	0.971573	0.907724	1.039912	0.405669
CORO2A	0.973802	0.922616	1.027828	0.335233
TGFBR1	0.950184	0.889139	1.01542	0.131482
SEC61B	0.935735	0.854986	1.024112	0.149153
C5	0.998677	0.930464	1.071891	0.970747
OGN	1.063089	0.992818	1.138334	0.079535
ASPN	1.032534	0.970385	1.098664	0.312103
ECM2	1.045634	0.974674	1.121761	0.213302
TLE4	0.915127	0.857589	0.976526	0.00743
LHX6	0.893003	0.82095	0.97138	0.008378
PTGR1	0.976473	0.89969	1.059809	0.56883
SUSD1	0.994596	0.927728	1.066283	0.878711
AKNA	0.961364	0.899739	1.02721	0.243727
TNFSF8	0.907191	0.854718	0.962885	0.001355
DNM1	0.88307	0.837172	0.931484	4.96E-06
ENG	1.096753	1.020282	1.178954	0.012263
AK1	1.245744	1.121679	1.383532	4.04E-05
CDC37L1	1.025179	0.927894	1.132665	0.624959
TBC1D13	0.983885	0.906508	1.067867	0.697469
DOCK8	1.020357	0.960215	1.084265	0.515589
KANK1	1.11646	1.050258	1.186836	0.000412
TESK1	0.98039	0.896364	1.072294	0.664872
FUBP3	1.022931	0.936224	1.117669	0.615879

CREB3	1.05842	0.95317	1.175291	0.288029
RGP1	0.935867	0.867853	1.009212	0.085114
DDX58	1.034297	0.967702	1.105476	0.320662
EDF1	1.013078	0.909375	1.128607	0.813571
PIP5K1B	0.984706	0.926069	1.047055	0.622693
GLIS3	0.67985	0.604808	0.764202	1.00E-10
BAG1	0.944311	0.853348	1.044971	0.267536
RAPGEF1	0.971219	0.907282	1.039662	0.400623
NPDC1	1.031512	0.982768	1.082673	0.209053
APBA1	0.981885	0.894163	1.078213	0.701824
SETX	0.979795	0.919989	1.043489	0.525291
PTGDS	0.910044	0.821325	1.008347	0.071683
ABCA2	1.075074	1.016514	1.137007	0.011305
SHB	0.828337	0.763538	0.898635	5.85E-06
UBE2R2	0.877858	0.79893	0.964584	0.006726
EXOSC3	1.116599	1.020309	1.221976	0.016533
ZFAND5	0.881722	0.818101	0.950291	0.000987
DVL1	1.036457	0.952915	1.127322	0.403644
PDLIM1	0.954022	0.912442	0.997498	0.038439
CCNJ	0.94023	0.873542	1.012009	0.100603
DNTT	1.005587	0.97434	1.037835	0.7294
GATA3	1.069499	1.012703	1.129482	0.015807
HPS1	1.107486	0.9919	1.23654	0.069469
PHYH	1.167726	1.065165	1.280162	0.000947
RASSF4	1.076261	1.026243	1.128717	0.002471
DNMBP	1.008671	0.93848	1.084111	0.814516
RAB11FIP2	0.984184	0.907392	1.067475	0.700518
CXCL12	1.020631	0.979608	1.06337	0.329241
ERLIN1	0.949472	0.895817	1.006341	0.08064
EIF3A	0.893723	0.824694	0.96853	0.006151
CUBN	1.154164	1.065805	1.249847	0.000418
TRDMT1	1.149919	1.054944	1.253443	0.001493
DDX50	1.032799	0.93963	1.135207	0.503462
MAPK8	0.995929	0.916496	1.082246	0.923362
SEC23IP	1.086978	0.992321	1.190664	0.072793
ATE1	0.974597	0.898256	1.057425	0.536384
NSMCE4A	1.216376	1.096891	1.348876	0.000205
PLEKHA1	1.088345	0.989668	1.19686	0.080847
UNC5B	1.113999	1.056351	1.174793	6.83E-05
CDH23	1.232482	1.150982	1.319753	2.12E-09
SPOCK2	1.001715	0.959298	1.046008	0.938139
PPP3CB	1.003094	0.918752	1.095179	0.945039
BMPR1A	1.172199	1.066245	1.288682	0.001013
MINPP1	1.066419	1.013455	1.122151	0.013354
ACTA2	0.974942	0.899918	1.056221	0.534498
LIPA	1.144977	1.068	1.227503	0.000137
LZTS2	0.957296	0.897683	1.020867	0.183388
SFXN3	1.150293	1.076233	1.229449	3.73E-05
KAZALD1	1.361573	1.228453	1.50912	4.11E-09
FBXW4	1.143567	1.043193	1.253599	0.004208
NPM3	0.982602	0.907313	1.064138	0.666082
TNKS2	0.98735	0.923909	1.055146	0.707115
GBF1	1.017806	0.933236	1.110039	0.690068
ARHGAP2	1.055525	1.007594	1.105736	0.022666
CPEB3	1.142414	1.035856	1.259933	0.007696
FBXL15	1.180905	1.049355	1.328947	0.00579
CUEDC2	1.078906	0.970263	1.199714	0.160766
SUFU	1.053829	0.977078	1.13661	0.174169

ANKRD26	0.941602	0.850422	1.042557	0.246881
ACBD5	0.962081	0.883337	1.047844	0.37493
LHPP	1.195592	1.085627	1.316696	0.000285
LARP4B	1.020919	0.961909	1.083548	0.495539
GTPBP4	0.905329	0.836477	0.979848	0.013725
BCCIP	1.065042	0.954293	1.188644	0.260658
MTPAP	1.062028	0.96305	1.171179	0.227942
SH3PXD2A	0.958319	0.906164	1.013476	0.13593
PITRM1	1.088112	0.987176	1.199367	0.089109
MAP3K8	0.955665	0.888486	1.027923	0.22269
EBF3	0.949934	0.875368	1.030851	0.218149
GLRX3	1.04245	0.939245	1.156994	0.434453
SORCS1	0.872422	0.770989	0.987201	0.030446
XPNPEP1	0.874983	0.81194	0.942921	0.000465
SMC3	0.983085	0.90346	1.069728	0.692212
SHOC2	0.9829	0.91543	1.055344	0.634529
TFAM	1.098072	1.003183	1.201938	0.042471
CCDC6	1.094607	1.011279	1.184801	0.025247
CUL2	0.990822	0.914302	1.073745	0.822095
CCNY	0.888686	0.817757	0.965767	0.005424
UBE2S	0.972967	0.903776	1.047456	0.466543
RPL28	0.91224	0.852297	0.9764	0.008081
ZMIZ1	1.049638	0.972536	1.132853	0.2133
DNAJC12	1.039789	0.974105	1.109903	0.241218
PPIF	1.137852	1.046599	1.237061	0.002463
PBLD	0.955622	0.893472	1.022095	0.185837
TSPAN14	1.065739	0.995675	1.140733	0.066498
TBC1D12	1.179117	1.121599	1.239585	1.07E-10
KRT23	1.192228	1.11871	1.270576	6.15E-08
CRYBA1	0.987379	0.94006	1.03708	0.612226
NUFIP2	0.946868	0.888293	1.009306	0.093803
GIT1	0.981912	0.906269	1.063869	0.655398
RPL19	0.867975	0.798675	0.943289	0.000852
FBXL20	0.978011	0.909846	1.051283	0.546376
RUNDC3A	1.082216	1.021141	1.146944	0.00768
UBTF	1.20224	1.099543	1.314529	5.28E-05
CSF3	1.042474	0.962288	1.129341	0.308383
PSMD3	1.004847	0.905191	1.115474	0.927704
CASC3	1.016904	0.916451	1.128368	0.752097
RAPGEFL1	0.98987	0.915374	1.070429	0.798683
RGS9	0.928333	0.822305	1.048033	0.229446
RNF43	0.956147	0.871575	1.048925	0.342586
RAD51C	1.324644	1.180768	1.486051	1.65E-06
MTMR4	1.077939	1.004259	1.157023	0.037744
TRIM37	1.102049	1.01482	1.196775	0.020908
P2RX1	0.983797	0.910821	1.062619	0.67783
DHX40	0.939285	0.86381	1.021355	0.142768
TUBD1	1.056034	0.974632	1.144235	0.182814
KPNB1	0.99637	0.923058	1.075506	0.925704
GOSR2	1.113433	1.02752	1.20653	0.008726
PNPO	1.265182	1.168003	1.370446	8.00E-09
RPS6KB1	0.979636	0.907782	1.057178	0.596564
TRIM16L	1.082972	0.956421	1.226268	0.20868
CDK5RAP3	1.213792	1.107393	1.330414	3.48E-05
CBX1	1.160592	1.066659	1.262797	0.000543
RECQL5	1.041987	0.963928	1.126368	0.300551
PIGL	0.965799	0.885052	1.053913	0.434685
GALK1	1.00453	0.933959	1.080433	0.903213

INTS2	1.020989	0.947368	1.100331	0.586448
CAMTA2	1.072611	0.976361	1.17835	0.143947
MED13	0.982526	0.922816	1.046101	0.581592
HOXB6	1.078435	1.040494	1.117758	3.59E-05
ENO3	1.176383	1.07314	1.289558	0.000528
PFN1	1.054952	0.971906	1.145095	0.200977
RNF167	1.185457	1.059717	1.326117	0.002941
SLC25A11	1.124446	1.025881	1.23248	0.012215
RASD1	0.989459	0.94201	1.039299	0.672571
CHRNE	0.893541	0.819501	0.974271	0.010754
RAI1	0.980233	0.915798	1.0492	0.564941
NUP88	0.960234	0.889751	1.036301	0.29684
C1QBP	0.915305	0.845467	0.990912	0.028858
SLC6A4	1.113624	0.95569	1.297658	0.167846
BLMH	0.978661	0.89798	1.066591	0.623161
CPD	0.944504	0.891716	1.000416	0.051679
GOSR1	1.057918	0.967644	1.156613	0.216008
CCDC47	0.954003	0.888449	1.024393	0.194821
MED31	1.214307	1.105019	1.334404	5.45E-05
DRG2	1.104029	0.997303	1.222177	0.056404
FTSJ3	1.004609	0.920961	1.095855	0.917426
AKAP10	1.016229	0.944078	1.093894	0.668329
SMARCD2	0.924286	0.846776	1.008891	0.078088
ICAM2	1.142397	1.047263	1.246174	0.002691
SYNGR2	1.120188	1.008929	1.243716	0.03346
B9D1	1.10604	0.951272	1.285988	0.190047
UTP6	1.009809	0.929578	1.096965	0.817239
DDX5	0.997173	0.922635	1.077732	0.943064
CYTH1	0.928871	0.872576	0.988799	0.020718
PSMD11	1.054045	0.961685	1.155274	0.260607
LGALS3BP	1.009666	0.966647	1.054599	0.664999
CCL7	1.105638	1.051425	1.162646	9.04E-05
CCL2	1.12302	1.074979	1.173209	1.98E-07
CCL1	1.1097	1.054051	1.168286	7.33E-05
PEX12	1.180332	1.062429	1.31132	0.002017
DHX58	1.08577	1.004084	1.174101	0.039198
KAT2A	1.111051	1.040355	1.186551	0.001693
RAB5C	0.913457	0.834136	1.000321	0.050814
NAGLU	1.08319	0.978965	1.198511	0.121597
HSD17B1	1.180323	0.99696	1.39741	0.054277
MLX	0.961684	0.863752	1.070719	0.475852
CNTNAP1	0.99199	0.920965	1.068493	0.831971
ABI3	1.18069	1.116928	1.248092	4.52E-09
EZH1	0.998977	0.926828	1.076743	0.978647
DLX4	1.223686	1.119016	1.338146	9.65E-06
PPP1R9B	1.061828	0.985705	1.14383	0.113968
COL1A1	1.129748	1.062995	1.200692	8.64E-05
MRPL27	1.202811	1.083701	1.335012	0.000519
VAT1	0.86398	0.81529	0.915577	7.80E-07
LRRC59	0.95861	0.886826	1.036205	0.287145
RND2	1.118784	0.97578	1.282745	0.107706
ALOX12	0.978525	0.903172	1.060166	0.595448
HDAC5	0.974923	0.902411	1.053261	0.519547
ABCC3	1.117061	1.02632	1.215825	0.010438
MPP2	1.082418	0.999629	1.172064	0.051079
SMURF2	0.996803	0.924183	1.075128	0.933872
DUSP3	1.027067	0.957659	1.101506	0.454402
EFTUD2	1.039851	0.953495	1.134027	0.377018

HLF	1.144486	1.070592	1.22348	7.40E-05
SLC16A6	1.123008	1.050506	1.200513	0.000657
PRKAR1A	1.107016	1.02405	1.196703	0.010531
FAM20A	1.034694	0.970415	1.10323	0.297304
YWHAE	1.0133	0.914584	1.12267	0.800542
MMD	1.17599	1.092352	1.266032	1.66E-05
RANGRF	1.523134	1.243087	1.866271	4.93E-05
DPH1	1.206995	1.089327	1.337374	0.000325
MAP2K6	1.150859	1.065701	1.242822	0.000341
DHRS7B	1.14314	1.029834	1.268913	0.012007
WSB1	0.94851	0.885633	1.015852	0.130906
RCVRN	0.899661	0.805582	1.004726	0.060615
SLC9A3R1	0.955247	0.883958	1.032284	0.247263
MYH3	1.013767	0.91701	1.120733	0.789345
NAT9	0.99765	0.912301	1.090984	0.958878
TMEM104	0.957162	0.897681	1.020584	0.181053
VTN	1.04454	0.974064	1.120115	0.221461
TNFAIP1	1.011046	0.924888	1.105231	0.808981
IFT20	1.063586	0.968075	1.16852	0.199106
TMEM97	1.153193	1.068311	1.244819	0.000258
CDR2L	1.238329	1.126332	1.361462	9.89E-06
PMP22	0.847507	0.769315	0.933647	0.000808
UNC119	1.188689	1.100021	1.284505	1.24E-05
ALDOC	1.040323	0.958426	1.129218	0.34469
SUPT6H	0.879679	0.810925	0.954264	0.002019
RAB34	0.935399	0.899594	0.97263	0.000798
PHF12	0.885308	0.813825	0.963069	0.004568
TMEM33	1.042912	0.967877	1.123764	0.270061
GNRHR	1.020561	0.919849	1.132299	0.70103
SLAIN2	0.93724	0.858156	1.023611	0.149555
OCIAD1	1.037527	0.931613	1.155482	0.502493
DCUN1D4	1.0088	0.935906	1.087372	0.818895
USP46	1.024368	0.949268	1.105409	0.535419
CHIC2	0.959677	0.87538	1.052092	0.38026
NMU	1.067698	0.985561	1.15668	0.108748
PF4V1	1.043685	0.96502	1.128762	0.284882
NFKB1	0.937744	0.880609	0.998586	0.045062
AREG	0.96729	0.936581	0.999006	0.043342
MANBA	0.925221	0.85921	0.996304	0.039589
UBE2D3	0.910695	0.828626	1.000892	0.052202
ELF2	0.997825	0.919986	1.08225	0.958091
NDUFC1	1.194915	1.068505	1.336281	0.0018
TBC1D9	1.057344	1.010296	1.106583	0.016349
ZNF330	1.023452	0.916785	1.142529	0.679753
INPP4B	1.167909	1.110783	1.227973	1.31E-09
GAB1	0.94827	0.886808	1.013993	0.120294
KLHL2	0.994376	0.931639	1.061338	0.865308
RPL34	0.918843	0.850735	0.992403	0.031237
WFS1	1.050892	0.955411	1.155915	0.307066
GRPEL1	1.025545	0.937809	1.12149	0.580401
GAR1	1.039742	0.939325	1.150893	0.452013
FRG1	0.921817	0.820118	1.036128	0.172281
CLCN3	1.064966	1.001863	1.132043	0.043415
AADAT	0.87725	0.82174	0.936509	8.61E-05
GALNT7	0.957823	0.891906	1.028611	0.236202
DHX15	0.974091	0.910544	1.042074	0.445681
SEPSECS	1.015098	0.943893	1.091674	0.686328
SLC2A9	1.078041	1.016714	1.143068	0.011915

FBXW7	0.983414	0.904693	1.068985	0.694408
NEIL3	1.027379	0.967898	1.090516	0.374711
TBC1D19	1.070184	0.989847	1.15704	0.088447
CLNK	1.154989	1.053432	1.266337	0.002152
SH3D19	0.924211	0.867162	0.985012	0.01533
STIM2	1.145591	1.059557	1.23861	0.000644
MFSD10	0.886889	0.826338	0.951876	0.000878
GLRB	1.074092	0.987922	1.167777	0.0939
BST1	1.033865	0.984153	1.086089	0.185292
RAPGEF2	1.071903	1.008331	1.139482	0.026018
HGFAC	0.939614	0.827905	1.066396	0.334789
SNX25	1.009636	0.94835	1.074882	0.764068
LRP2BP	1.078956	0.988899	1.177215	0.087463
UFSP2	1.095823	0.99132	1.211342	0.073539
KLF3	0.847906	0.788182	0.912156	9.55E-06
KLHL5	0.94052	0.874643	1.011359	0.097902
NCAPG	1.067044	1.003717	1.134366	0.037633
UGDH	1.065915	0.99146	1.14596	0.084022
HTATIP2	1.14476	1.068699	1.226235	0.000116
CTSC	1.008556	0.931961	1.091446	0.83256
CCDC34	1.153277	1.057511	1.257715	0.001263
ZBTB16	0.920794	0.879771	0.96373	0.000387
ELP4	1.15991	1.042138	1.290992	0.006617
MTCH2	1.107451	0.991785	1.236606	0.06977
FNBP4	0.921405	0.854784	0.993219	0.032545
TECTA	1.268391	1.132083	1.421111	4.15E-05
CRTAM	1.056771	0.969879	1.151447	0.207187
HSPA8	0.96373	0.897088	1.035323	0.312258
VWA5A	1.187161	1.111341	1.268154	3.49E-07
DNAJC4	1.036573	0.932491	1.152272	0.505841
SIAE	0.969339	0.904132	1.03925	0.380795
LPXN	0.989587	0.925544	1.058062	0.759125
DTX4	1.06417	1.012814	1.11813	0.013721
ATG2A	1.011603	0.954765	1.071825	0.695789
EHD1	0.989243	0.92384	1.059276	0.756636
OSBP	0.933361	0.869842	1.001519	0.055141
UNC93B1	1.03173	0.972158	1.094952	0.30328
PUS3	1.057397	0.943595	1.184924	0.336735
DCPS	1.214273	1.113478	1.324192	1.13E-05
FOXRED1	1.167819	1.052852	1.295338	0.003346
NRXN2	1.066344	1.009706	1.126159	0.021064
MS4A6A	1.002126	0.967378	1.038121	0.90612
MS4A4A	1.034176	0.98406	1.086843	0.184854
ST3GAL4	1.142426	1.080836	1.207525	2.49E-06
CPT1A	1.089308	1.008594	1.176481	0.02942
CCND1	1.027233	0.967224	1.090966	0.381646
CCDC86	1.02811	0.94239	1.121627	0.532553
PRPF19	1.013448	0.934625	1.098918	0.746419
TMEM109	1.120792	1.042344	1.205144	0.002069
HPX	1.080157	0.945211	1.234369	0.257456
TRIM3	1.033258	0.945856	1.128736	0.468127
CHORDC1	1.040318	0.970311	1.115376	0.266117
FOLR1	1.218619	1.110642	1.337094	2.96E-05
FOLR3	1.010801	0.969142	1.05425	0.616881
PANX1	1.069531	0.993509	1.15137	0.073959
ARHGEF17	0.94477	0.894786	0.997547	0.04051
CEP164	1.169064	1.065297	1.282939	0.000989
RNF141	1.227922	1.117451	1.349314	1.97E-05

EIF4G2	0.965707	0.884749	1.054074	0.43474
IL10RA	0.997189	0.947985	1.048947	0.913187
BIRC2	1.112523	1.01859	1.215118	0.017826
UBE4A	1.078123	0.992521	1.171108	0.074732
DDX6	0.992233	0.928887	1.059898	0.8168
UPK2	1.000317	0.890606	1.123543	0.995738
CBL	0.950081	0.89019	1.014001	0.123211
HIPK3	0.924714	0.865699	0.987752	0.020006
FBXO3	1.031361	0.968346	1.098476	0.33706
PDHX	1.109538	1.019816	1.207153	0.015689
SLC1A2	0.916818	0.81886	1.026494	0.131966
COMMD9	1.005904	0.910003	1.11191	0.908329
SLC15A3	1.025856	0.979677	1.074212	0.277369
CD5	0.990609	0.941431	1.042356	0.716466
ACCS	0.95048	0.900877	1.002813	0.063277
MDK	0.904575	0.854902	0.957134	0.000501
AMBRA1	0.983158	0.921417	1.049035	0.607734
MADD	0.998414	0.930895	1.070831	0.964568
PTPMT1	1.059201	0.926424	1.211008	0.399991
SLC22A18	1.203099	1.109526	1.304564	7.61E-06
CD81	1.06503	0.994408	1.140668	0.071898
SLC35F2	0.8795	0.832144	0.929551	5.44E-06
SOX6	1.053136	0.982573	1.128767	0.143433
PITPNM1	0.896784	0.839488	0.957991	0.001221
RPS13	0.90769	0.837135	0.984191	0.018979
AIP	0.978254	0.885743	1.080428	0.664462
NUP98	0.926694	0.868933	0.988294	0.020419
NDUFS8	1.307978	1.159858	1.475015	1.20E-05
TCIRG1	1.026752	0.962636	1.095138	0.422283
CHKA	0.91962	0.862702	0.980294	0.010155
EXPH5	1.423595	1.305525	1.552342	1.29E-15
HPS5	1.015961	0.945938	1.091168	0.663855
GTF2H1	1.045018	0.961919	1.135297	0.297599
POU2AF1	1.000288	0.937295	1.067515	0.993073
VWF	1.128157	1.084783	1.173264	1.66E-09
PSMD9	1.039204	0.930605	1.160477	0.494696
PPFIBP1	1.083513	1.019427	1.151629	0.009923
PRPF40B	1.218949	1.105627	1.343885	6.98E-05
CD69	1.062761	1.011312	1.116829	0.016206
PRDM4	0.973935	0.892652	1.06262	0.552532
CLEC2B	1.050318	0.998825	1.104466	0.055606
COQ5	1.076403	0.98582	1.175309	0.100684
SELPLG	1.075449	1.025075	1.128299	0.002961
CORO1C	0.999857	0.926092	1.079497	0.997083
CAPRIN2	1.157498	1.089095	1.230198	2.53E-06
TSPAN11	1.320885	1.161455	1.5022	2.23E-05
KCTD10	0.980822	0.898995	1.070098	0.663078
SLC11A2	1.034915	0.961804	1.113583	0.358563
MLEC	0.97216	0.909623	1.038995	0.405232
MVK	1.153214	1.041558	1.276838	0.006076
CSRNP2	1.023677	0.942545	1.111793	0.578578
CAMKK2	0.980978	0.91556	1.05107	0.585461
BIN2	1.056474	0.984518	1.133689	0.1269
IL23A	1.047308	0.974469	1.12559	0.208834
PTGES3	1.027612	0.922495	1.144706	0.620807
BCL7A	1.170984	1.101744	1.244575	3.86E-07
RSRC2	0.930113	0.860903	1.004888	0.066304
CYP27B1	1.067122	0.976517	1.166135	0.151273

LIN7A	0.984541	0.941738	1.02929	0.492094
KRT18	0.977835	0.94372	1.013183	0.21605
GLI1	1.040226	0.965416	1.120833	0.300353
PPM1H	0.997299	0.943937	1.053677	0.923199
METAP2	0.948926	0.859866	1.04721	0.297141
LTA4H	0.965892	0.891196	1.046848	0.398074
ELK3	1.040563	0.965961	1.120926	0.29484
SLC6A12	1.025551	0.93674	1.122783	0.585115
WNT5B	0.794563	0.701053	0.900546	0.000319
MAGOHB	1.27786	1.148206	1.422155	7.06E-06
TRPV4	1.299444	1.164712	1.449762	2.73E-06
ITFG2	1.042949	0.953329	1.140994	0.358963
FOXM1	1.123929	1.038808	1.216025	0.003644
PRR4	1.048307	0.939084	1.170232	0.400698
PARP11	1.012803	0.936886	1.094871	0.748964
ARPC3	0.976398	0.890044	1.07113	0.613174
GPN3	0.975644	0.894363	1.064312	0.578503
VPS29	1.152258	1.043062	1.272886	0.005272
RAD51AP1	1.126759	1.053731	1.204849	0.000482
CUX2	1.139147	1.050771	1.234956	0.001567
SH2B3	1.023233	0.953153	1.098466	0.525769
MANSC1	1.037575	0.955816	1.126328	0.378409
DUSP16	0.964206	0.901701	1.031044	0.28645
CREBL2	1.053143	0.977356	1.134806	0.174187
ACAD10	1.063156	0.984838	1.147703	0.116732
ALDH2	1.166526	1.10335	1.23332	5.89E-08
CDKN1B	0.974551	0.903176	1.051566	0.506508
GPRC5D	1.005013	0.912256	1.107201	0.919388
GSG1	1.123456	0.949014	1.329962	0.176337
SCNN1A	1.06968	0.892465	1.282085	0.466065
LTBR	0.940861	0.871119	1.016188	0.120828
OGFOD2	1.175146	1.047144	1.318796	0.006091
CDK2AP1	1.072184	0.985035	1.167044	0.107101
OAS3	1.074577	1.02478	1.126794	0.002968
OAS2	1.056629	1.00522	1.110667	0.030422
ART4	1.031633	0.952923	1.116844	0.441836
MGP	0.866391	0.762558	0.984362	0.027669
RASAL1	0.873022	0.826552	0.922105	1.14E-06
ARHGDIB	0.986961	0.912906	1.067023	0.741545
GTF2H3	1.097351	1.007309	1.195441	0.033447
EIF2B1	1.2142	1.093649	1.348039	0.000275
DDX55	1.156079	1.050622	1.272121	0.00296
SLC38A1	0.97609	0.930618	1.023784	0.3201
VDR	1.18182	1.118953	1.248219	2.10E-09
RFC5	1.227176	1.112054	1.354215	4.64E-05
STX2	0.984392	0.919068	1.05436	0.65342
COPZ1	1.094203	0.978931	1.223048	0.112955
CAND1	0.980108	0.912739	1.052449	0.580255
IFNG	1.039187	0.957801	1.127489	0.355601
RAB5B	0.951009	0.885827	1.020986	0.165553
MDM1	1.089595	0.999147	1.18823	0.0523
NUP107	1.047541	0.968759	1.13273	0.244299
CNOT2	0.925972	0.861455	0.995321	0.036867
TIMELESS	1.135806	1.064425	1.211975	0.00012
CPSF6	0.958605	0.887504	1.035401	0.282288
KRR1	0.953541	0.886169	1.026036	0.203207
MRPL51	1.079055	0.988459	1.177955	0.089032
GAPDH	0.916993	0.844474	0.99574	0.039253

NOP2	0.968221	0.907288	1.033245	0.33015
CHD4	1.050751	0.963385	1.14604	0.263675
ACRBP	1.094363	1.012051	1.183371	0.023808
UHRF1BP1	0.964158	0.891024	1.043294	0.364457
COPS7A	1.158265	1.053696	1.273211	0.002339
ING4	1.046363	0.964865	1.134744	0.273326
GNB3	1.201563	1.069557	1.34986	0.001985
CDCA3	1.227078	1.130809	1.331543	9.15E-07
CHPT1	0.848293	0.78249	0.919628	6.50E-05
USP5	1.12415	1.033988	1.222174	0.006078
TPI1	1.093623	0.998638	1.197642	0.053537
GNPTAB	0.879082	0.828801	0.932415	1.80E-05
SPSB2	1.063548	0.985335	1.147968	0.113903
ENO2	1.200683	1.124035	1.282559	5.51E-08
ATN1	0.994509	0.938759	1.053569	0.851603
PTPN6	1.089131	1.003579	1.181977	0.040801
LPCAT3	1.328032	1.185094	1.488209	1.05E-06
NT5DC3	0.959722	0.913847	1.007899	0.099948
SUDS3	1.036112	0.961983	1.115954	0.348943
GOLT1B	1.086656	0.995164	1.186559	0.064035
LDHB	1.097651	1.009136	1.19393	0.029859
PRKAB1	1.023529	0.932437	1.12352	0.624827
CMAS	1.167001	1.078291	1.263008	0.000129
HCFC2	1.138239	1.045763	1.238892	0.002745
CLEC4A	0.958105	0.854556	1.074202	0.463321
RAB35	0.924589	0.85351	1.001588	0.054722
PHC1	0.963493	0.885855	1.047936	0.385599
COX6A1	1.143327	1.030689	1.268273	0.011367
RIC8B	1.180389	1.084708	1.28451	0.00012
FGFR1OP2	1.045102	0.958193	1.139893	0.319308
KLRB1	1.054262	0.992632	1.119719	0.085554
BTN3A3	1.056276	0.986524	1.130959	0.116247
DSE	0.953272	0.887629	1.023771	0.188643
RWDD1	1.0805	0.968631	1.205287	0.165008
MAK	1.005472	0.92828	1.089082	0.893486
TMEM14C	1.077077	0.994545	1.166458	0.067927
PAK1IP1	1.18627	1.092827	1.287703	4.49E-05
GCNT2	1.107847	1.037463	1.183005	0.002227
NEDD9	1.018498	0.94977	1.0922	0.607107
ASF1A	1.043703	0.962472	1.13179	0.300808
MCM9	1.028817	0.948235	1.116247	0.494801
RNGTT	1.038277	0.962048	1.120545	0.334302
MAN1A1	0.900113	0.861378	0.940589	2.74E-06
GABRR2	1.098468	1.014392	1.189514	0.020796
SERINC1	0.965861	0.901297	1.03505	0.325099
HDDC2	1.145972	1.040749	1.261833	0.005558
HINT3	0.991807	0.909631	1.081407	0.85211
NCOA7	0.928308	0.874397	0.985544	0.01481
SASH1	1.076933	1.001327	1.158248	0.045969
UST	1.075216	0.991562	1.165927	0.079276
FBXO5	1.144149	1.068833	1.224771	0.000106
MTRF1L	0.962804	0.857356	1.081222	0.521865
PPARD	1.037982	0.976719	1.103087	0.229738
FANCE	1.121538	1.042821	1.206196	0.002007
SLC26A8	0.99686	0.932114	1.066103	0.926859
MAPK14	0.992589	0.933126	1.055842	0.813438
RHAG	1.041882	1.012573	1.072039	0.004829
KCTD20	0.969473	0.891696	1.054033	0.467461

STK38	0.980731	0.920162	1.045288	0.549709
SOD2	1.127428	1.052694	1.207468	0.000609
MRPL18	1.214099	1.107639	1.330792	3.42E-05
MCM3	1.113677	1.047172	1.184405	0.00061
RNF8	1.251799	1.116288	1.403761	0.000122
PHACTR1	1.109563	1.027892	1.197724	0.007695
MDGA1	1.081059	1.002457	1.165823	0.043002
FBXO9	1.101355	1.013105	1.197292	0.023483
CD83	0.930129	0.88183	0.981074	0.007761
MDN1	1.000102	0.943429	1.060179	0.997265
BACH2	1.024828	0.974585	1.077661	0.33896
TREML2	1.045617	0.981672	1.113728	0.165918
ZNF451	0.973396	0.918553	1.031512	0.362114
BAG2	1.293344	1.18501	1.411583	8.25E-09
RAB23	1.456314	1.295186	1.637488	3.31E-10
KHDRBS2	1.185918	1.094071	1.285476	3.38E-05
FBXL4	1.0402	0.966682	1.11931	0.291935
CCNC	0.988387	0.909822	1.073736	0.782227
E2F3	0.982863	0.913612	1.057362	0.642859
PTP4A1	0.937135	0.877097	1.001282	0.054601
ASCC3	1.076896	1.003553	1.155599	0.039541
BVES	1.129106	1.072489	1.188712	3.72E-06
MED23	0.976506	0.916381	1.040577	0.463417
WASF1	0.991422	0.947808	1.037042	0.70741
GPLD1	1.127717	1.031599	1.232791	0.008183
ALDH5A1	1.077375	1.009825	1.149443	0.024076
VNN1	1.057038	1.016161	1.099559	0.005839
VNN2	1.018228	0.979539	1.058444	0.360738
SMAP1	0.980657	0.86527	1.11143	0.759736
RPS12	0.936566	0.881807	0.994726	0.033008
B3GAT2	1.268291	1.155343	1.392281	5.91E-07
GMNN	1.118557	1.025835	1.21966	0.011159
SOBP	1.246016	1.158274	1.340404	3.55E-09
SNX3	1.021185	0.90589	1.151154	0.731625
HBS1L	0.974837	0.908658	1.045835	0.477385
TRIM38	1.017759	0.949635	1.09077	0.618485
PEX7	1.215476	1.086461	1.359811	0.000653
ZBTB24	1.230896	1.115226	1.358563	3.69E-05
FIG4	1.079252	0.977834	1.191189	0.129832
PERP	1.402286	1.208809	1.626731	8.07E-06
SLC16A10	1.153591	1.038659	1.28124	0.007623
HECA	0.951506	0.891604	1.015433	0.134046
PHACTR2	1.040342	0.972521	1.112894	0.250203
EPM2A	0.993558	0.895952	1.101797	0.902505
SLC39A7	0.944155	0.874141	1.019777	0.143797
PHF1	0.916381	0.8488	0.989343	0.025478
CUTA	1.036067	0.93156	1.152298	0.513669
QKI	0.860004	0.800202	0.924276	4.11E-05
MDFI	0.988095	0.940946	1.037608	0.631174
TFEB	1.105534	1.034686	1.181233	0.002988
CCND3	1.22381	1.134876	1.319714	1.55E-07
BYSL	1.126984	1.045047	1.215345	0.001909
FAM120B	1.092069	1.019499	1.169804	0.012059
TBP	1.009298	0.911118	1.118058	0.859312
GUCA1B	0.968331	0.895223	1.047408	0.421687
PRPH2	1.072445	0.975472	1.179059	0.148066
PPP2R5D	1.031088	0.929597	1.14366	0.562535
MRPL2	1.406776	1.214474	1.629529	5.34E-06

PTK7	0.95361	0.908419	1.001049	0.055157
SRF	0.964466	0.880755	1.056133	0.434789
CUL9	1.013652	0.940964	1.091956	0.720971
DUSP22	0.965247	0.893954	1.042225	0.366251
EXOC2	1.085422	1.00547	1.171732	0.035754
COX7A2	1.053395	0.976983	1.135782	0.175769
TMEM30A	1.058802	0.980554	1.143293	0.144661
GMDS	1.011558	0.932567	1.097239	0.781771
SENP6	1.083195	1.008528	1.163389	0.028308
VEGFA	0.92498	0.878149	0.974308	0.003262
PRPF4B	0.970177	0.904521	1.0406	0.397088
TTK	1.085462	1.016129	1.159525	0.014888
SLC29A1	1.05126	0.975105	1.133362	0.192609
BTN2A1	0.991628	0.910259	1.08027	0.847381
LAMA4	1.249845	1.004582	1.554987	0.045395
CLIC5	1.195585	1.072795	1.33243	0.001234
LY86	1.021036	0.983363	1.060153	0.277777
HARS2	1.069842	0.976999	1.171508	0.144961
NUDT12	1.087957	0.983766	1.203181	0.100734
CEP72	1.145508	1.059511	1.238484	0.000645
MAN2A1	1.064601	0.993076	1.141277	0.077705
HMGCS1	1.115005	1.02947	1.207646	0.007514
DAP	0.891935	0.816245	0.974644	0.011485
BRD8	1.031649	0.94752	1.123248	0.472811
KIF20A	1.09317	1.033845	1.1559	0.001753
NNT	1.052013	0.972864	1.137601	0.203873
MRPS30	1.084439	0.978616	1.201706	0.121779
HSPA9	0.95444	0.874691	1.041459	0.294889
MRPS27	0.961673	0.880403	1.050446	0.385667
PFDN1	1.031497	0.919697	1.156888	0.596242
HBEGF	1.042105	0.997858	1.088314	0.062451
LOX	1.208466	1.122757	1.300718	4.54E-07
GZMK	1.065898	0.988014	1.149922	0.099254
CDH9	1.050409	0.975556	1.131006	0.192287
APBB3	1.067408	0.989289	1.151695	0.092521
TMCO6	1.112402	1.013768	1.220632	0.024537
SPARC	0.937909	0.904348	0.972715	0.000565
IK	1.055623	0.931156	1.196729	0.397747
HMGCR	1.046632	0.965686	1.134362	0.267099
FAF2	1.004028	0.925241	1.089524	0.923192
PDE8B	1.107201	0.901561	1.359746	0.331331
CLK4	1.068376	0.986248	1.157344	0.10509
HAVCR1	0.878296	0.817006	0.944185	0.000438
ITK	1.001621	0.952943	1.052785	0.949204
RNF130	0.8345	0.768718	0.905911	1.57E-05
THG1L	0.92307	0.835464	1.019862	0.115625
ARSB	0.97636	0.901305	1.057666	0.557741
CLINT1	0.893806	0.834047	0.957848	0.001474
THBS4	1.025585	0.967357	1.087318	0.396921
CNOT6	1.020267	0.949429	1.096391	0.584718
BTNL8	1.000873	0.897118	1.116629	0.987526
TTC1	1.086248	0.97938	1.204776	0.11743
MSH3	0.999459	0.925313	1.079546	0.989024
RASGRF2	1.072373	0.967841	1.188195	0.18178
CCNG1	0.891584	0.833206	0.954053	0.000896
POLR3G	0.955611	0.856293	1.066448	0.417397
LMNB1	1.048556	0.982904	1.118593	0.150645
ARRDC3	0.978049	0.925573	1.033501	0.43021

GOLPH3	0.985342	0.909074	1.068009	0.719412
SUB1	0.964558	0.871808	1.067175	0.484204
NPR3	0.937423	0.905352	0.970631	0.000275
FAM172A	1.080001	0.991651	1.176223	0.077156
SLC27A6	0.927172	0.860686	0.998793	0.046397
LNPEP	1.014197	0.957207	1.07458	0.632824
PDE4D	0.980119	0.921339	1.042649	0.524514
RAD1	1.140852	1.028763	1.265154	0.012511
BRIX1	1.098621	0.993598	1.214745	0.066552
PRLR	1.013502	0.952604	1.078293	0.671425
SLC12A7	1.004062	0.951678	1.05933	0.882127
RAD50	1.271605	1.092171	1.480519	0.001962
ST8SIA4	0.946754	0.873901	1.02568	0.18047
GNPDA1	1.065372	0.997599	1.13775	0.058988
PCDH12	1.129013	1.034931	1.231649	0.006269
SKP1	0.998056	0.923206	1.078974	0.960981
NUP155	1.059124	0.982782	1.141397	0.132337
PPP2CA	0.938384	0.861079	1.02263	0.14711
NR3C1	0.941433	0.877403	1.010136	0.093086
PPWD1	0.989378	0.920853	1.063003	0.770595
LIFR	0.939339	0.812148	1.08645	0.399229
TRIM23	1.026638	0.957083	1.101248	0.462661
SEC24A	0.90381	0.842232	0.969889	0.004967
TXNDC15	1.024448	0.940294	1.116134	0.580745
TTC33	1.101997	0.990439	1.226119	0.074497
TCERG1	0.957092	0.883634	1.036656	0.281751
DPYSL3	1.041908	0.992861	1.093379	0.095168
SMAD5	0.934076	0.871157	1.00154	0.055273
CSNK1A1	0.98183	0.907266	1.062523	0.649091
ERGIC1	0.922956	0.852851	0.998824	0.046683
PDGFRB	0.950638	0.891936	1.013203	0.119559
ATP6V0E1	1.075353	0.9927	1.164887	0.075009
BNIP1	0.965105	0.873536	1.066271	0.484962
CPEB4	1.016942	0.958969	1.07842	0.57481
HRH2	1.085973	1.036181	1.138157	0.000573
DBN1	1.028675	0.981579	1.078031	0.237054
ZNF346	1.017161	0.928763	1.113973	0.713754
UNC5A	1.054529	0.997466	1.114856	0.061403
EHHADH	1.147679	1.060535	1.241983	0.000629
SMC4	1.097027	1.025066	1.174041	0.00747
ACTR8	1.012347	0.927992	1.104369	0.782209
TBCCD1	1.157096	1.057996	1.265478	0.001403
CRBN	0.946915	0.868321	1.032622	0.217266
BCL6	0.973165	0.919518	1.029941	0.347097
HGD	1.066118	0.976634	1.1638	0.152324
ARL6	1.264803	1.113096	1.437186	0.000314
NPHP3	1.085167	0.966753	1.218085	0.165619
CD86	1.020576	0.977776	1.065249	0.351454
AMOTL2	1.225557	1.114406	1.347795	2.75E-05
NIT2	0.929038	0.846134	1.020065	0.122736
FAM162A	1.087271	0.97537	1.21201	0.131062
OGG1	1.281026	1.181997	1.388352	1.61E-09
KPNA1	1.061137	0.980342	1.14859	0.141939
PCCB	1.024903	0.934935	1.123528	0.599767
UBE3A	0.945973	0.878741	1.018348	0.139784
ARMC8	1.018604	0.946933	1.0957	0.620477
CEP70	1.072748	1.009749	1.139677	0.022957
RBP2	0.979253	0.917403	1.045272	0.528811

SLC25A36	0.917062	0.854086	0.984682	0.017069
GRK7	0.966807	0.859614	1.087367	0.573434
RNF7	0.985479	0.888522	1.093017	0.781931
TFDP2	1.000384	0.93452	1.07089	0.991178
XRN1	0.968753	0.914044	1.026737	0.284462
KAT2B	0.996372	0.932465	1.064659	0.914418
SERPINI2	1.061639	1.007906	1.118237	0.024
PDCD10	1.043365	0.949175	1.146903	0.379187
PFKFB4	0.988841	0.929687	1.051758	0.721417
COL7A1	0.984272	0.920636	1.052306	0.64202
PRKAR2A	0.993065	0.902903	1.092231	0.886058
HES1	1.121913	1.069245	1.177175	2.74E-06
USP4	1.004562	0.924473	1.091589	0.914493
ACAP2	1.002136	0.931152	1.078531	0.954601
ECT2	1.057022	0.989702	1.128921	0.098606
GNAT1	0.935264	0.845889	1.034082	0.191562
GNAI2	0.858655	0.788242	0.935358	0.000482
TFG	0.902554	0.830426	0.980947	0.015837
USP9Y	1.024729	0.987429	1.063437	0.196618
HYAL1	0.927844	0.850511	1.012209	0.091667
TUSC2	0.945862	0.854117	1.047461	0.284976
RPL24	0.875944	0.803621	0.954777	0.002591
CYB561D2	1.077624	0.981391	1.183295	0.117259
FXR1	0.873311	0.805707	0.946587	0.000983
CBLB	0.951221	0.880467	1.027661	0.204765
BBX	1.01458	0.94771	1.086169	0.677336
IFT57	0.992578	0.901852	1.092431	0.878929
GNB4	0.989221	0.918486	1.065403	0.77464
HHLA2	0.870075	0.771674	0.981023	0.023035
IQCG	1.182999	1.075206	1.301599	0.000566
GBE1	0.92668	0.859964	0.998572	0.045777
UMPS	1.155058	1.065642	1.251976	0.000454
NCBP2	1.070751	0.973103	1.178198	0.161175
SNX4	0.957065	0.874187	1.047801	0.342325
FRMD4B	1.134152	1.060956	1.212398	0.000217
SLC41A3	0.865063	0.780429	0.958875	0.005791
PLXNA1	1.096809	1.02715	1.171192	0.005778
ATP6V1A	1.063958	0.989289	1.144262	0.094938
ABTB1	0.977567	0.909126	1.051161	0.540103
PODXL2	1.040976	0.984525	1.100663	0.158037
UPK1B	0.951936	0.868754	1.043083	0.291049
CSPG5	1.02833	0.932869	1.133558	0.574118
KLHL18	1.02487	0.945592	1.110795	0.549812
SCAP	1.009594	0.938254	1.086359	0.798437
NEK11	1.054715	0.962249	1.156067	0.255147
MRPL3	1.020498	0.934143	1.114837	0.652859
PLSCR4	1.231572	1.141833	1.328365	6.81E-08
HEMK1	1.078489	0.994989	1.168996	0.066093
CISH	1.015496	0.942112	1.094595	0.687828
MAPKAPK	0.868308	0.800625	0.941712	0.000649
ACVR2B	1.14533	1.065586	1.231041	0.000229
WDR48	0.936276	0.880476	0.995614	0.035714
COMMD2	1.124056	1.016336	1.243194	0.022893
GORASP1	0.978344	0.896573	1.067573	0.62297
RRP9	1.191507	1.108264	1.281001	2.12E-06
ABCC5	1.083974	1.016085	1.156398	0.014544
ABHD14B	0.97745	0.890209	1.07324	0.632533
EIF1B	1.031541	0.936309	1.136461	0.529772

KLHL24	0.926538	0.875612	0.980425	0.00816
PLCH1	1.010606	0.934628	1.092761	0.791334
VIPR1	0.98221	0.898816	1.073341	0.691717
DNAH1	1.01573	0.955679	1.079555	0.615691
SSR3	1.033074	0.942398	1.132474	0.487547
ZBTB47	1.140502	1.0607	1.226307	0.000382
NKTR	0.964008	0.906815	1.024807	0.240117
CLCN2	1.123873	1.006875	1.254466	0.037332
FOXP1	1.021645	0.948866	1.100005	0.570085
EIF4G1	0.984695	0.908218	1.067613	0.708481
SPCS1	1.086435	0.99462	1.186724	0.065735
NEK4	0.986741	0.905083	1.075767	0.762007
SLC4A3	1.049479	0.973544	1.131336	0.207573
INO80D	0.955327	0.892667	1.022385	0.186711
EEF1B2	0.866491	0.808482	0.928661	5.05E-05
ADAM23	1.037772	0.990405	1.087404	0.119837
DGUOK	1.175469	1.053381	1.311708	0.003859
LMAN2L	1.073712	0.994247	1.159528	0.069849
RTKN	1.068729	0.988175	1.155849	0.096422
TTL	1.079632	0.984913	1.18346	0.10195
IL1A	1.229873	1.151536	1.31354	7.19E-10
CCL20	1.156943	1.063443	1.258663	0.000697
PIKFYVE	0.956322	0.899182	1.017094	0.155386
FAHD2A	1.168306	1.05097	1.298742	0.00397
NCL	1.040077	0.945474	1.144147	0.419313
ACTR1B	0.938235	0.871197	1.010432	0.091878
SLC35F5	0.973985	0.919118	1.032128	0.372916
ZAP70	0.916227	0.871901	0.962806	0.000544
ACTR3	0.99379	0.908494	1.087095	0.89178
STEAP3	1.009033	0.953525	1.067773	0.75542
EPB41L5	1.054086	0.974302	1.140403	0.189628
TFCP2L1	1.080863	0.992114	1.177552	0.075264
TP53I3	0.962648	0.880032	1.053019	0.405678
DNAJC27	1.104385	0.998929	1.220973	0.052497
POMC	1.021073	0.964988	1.080418	0.469376
STAM2	1.016498	0.933959	1.106331	0.704904
OTOF	1.156827	1.094527	1.222673	2.50E-07
GPD2	1.036041	0.971606	1.10475	0.279812
CENPA	1.176539	1.095579	1.26348	7.84E-06
CYTIP	0.881578	0.833978	0.931894	8.56E-06
ACVR1	1.127556	1.051908	1.208644	0.000704
TANC1	1.101847	1.048384	1.158037	0.000132
SLC30A3	1.129966	0.993215	1.285545	0.063379
MPV17	1.088643	0.984896	1.203317	0.096487
GTF3C2	1.051003	0.954349	1.157446	0.312182
EIF2B4	1.229174	1.090561	1.385405	0.000725
NRBP1	0.82874	0.7552	0.909441	7.43E-05
ITGA4	0.969791	0.91313	1.029968	0.317964
PSMD14	1.149868	1.026331	1.288275	0.016034
SNX17	0.992131	0.903286	1.089714	0.868905
ASB3	0.982392	0.90904	1.061663	0.653665
PPM1G	1.085305	0.97209	1.211705	0.145292
REEP6	1.208553	1.125723	1.297478	1.70E-07
PCSK4	1.075384	0.997996	1.158774	0.056481
APC2	1.003869	0.911553	1.105535	0.937461
IFIH1	0.989557	0.931088	1.051699	0.735498
RPS15	0.930421	0.861247	1.005151	0.067306
GCA	1.007539	0.953993	1.064091	0.787489

INO80B	1.32706	1.178542	1.494294	2.97E-06
MOGS	0.980848	0.906412	1.061397	0.631067
TTC31	1.078611	0.987505	1.178121	0.092819
NDUFS7	1.44805	1.265301	1.657195	7.51E-08
PCGF1	1.001937	0.907003	1.106807	0.969612
CLIP4	1.033268	0.978031	1.091625	0.243012
SPTBN1	0.927497	0.883207	0.974009	0.002571
AUP1	1.326205	1.154466	1.523491	6.61E-05
RTN4	0.894691	0.822307	0.973446	0.009732
HTRA2	1.117195	1.003831	1.243362	0.042356
LOXL3	0.928355	0.869853	0.990791	0.025185
DOK1	0.968311	0.90196	1.039543	0.373917
GALNT3	0.924377	0.877989	0.973215	0.002758
POLE4	1.150295	1.058344	1.250236	0.000988
TACR1	1.10162	0.976512	1.242756	0.115595
CCDC88A	0.918922	0.852403	0.990632	0.027422
MRPL19	1.05869	0.970622	1.154749	0.198075
LANCL1	1.111941	1.029831	1.200597	0.006709
WDR75	1.025684	0.949246	1.108277	0.521016
EFEMP1	1.06015	0.919166	1.222759	0.422397
FANCL	1.055466	0.986439	1.129324	0.117748
FN1	1.055122	0.987117	1.127811	0.114453
STAT1	1.032425	0.974583	1.093699	0.278025
GLS	0.968223	0.911737	1.028207	0.292359
PAPOLG	0.999507	0.913364	1.093776	0.991453
PECR	1.181635	1.103657	1.265122	1.66E-06
UNC50	1.049587	0.96594	1.140478	0.25339
IGFBP2	0.969271	0.933519	1.006391	0.103586
ELMOD3	1.225896	1.122212	1.339159	6.27E-06
IGFBP5	1.028948	0.95954	1.103377	0.423204
USP34	0.971975	0.915912	1.03147	0.348373
KCNJ13	0.97633	0.918874	1.037379	0.438874
CCT4	0.949146	0.855879	1.052576	0.322659
GGCX	0.962127	0.890981	1.038954	0.324613
EHBP1	1.091414	1.01495	1.173638	0.018256
TXNDC9	1.048656	0.935416	1.175605	0.415152
COQ10B	0.961081	0.887217	1.041095	0.3306
GNLY	1.063048	1.015045	1.113321	0.009504
SF3B1	0.963391	0.896451	1.035329	0.310078
ST3GAL5	0.890636	0.824971	0.961528	0.003037
CHST10	1.057308	0.98391	1.13618	0.128993
PDCL3	0.951314	0.876306	1.032744	0.233621
HSPE1	1.094237	1.016295	1.178156	0.016909
PLCD4	1.073496	0.965448	1.193635	0.190092
ZNF142	1.113562	1.014465	1.222339	0.0237
IL1R2	1.068847	1.025717	1.11379	0.001534
IL1R1	1.106735	1.045164	1.171933	0.000516
IL1RL1	0.854708	0.81431	0.89711	2.08E-10
IL18R1	1.009428	0.955056	1.066894	0.739764
IL18RAP	1.041694	0.995271	1.090283	0.079063
FHL2	1.087093	1.022903	1.15531	0.007162
UXS1	0.972498	0.890334	1.062244	0.535776
ABCB6	1.118793	1.014307	1.234043	0.024836
STK16	1.108819	0.999545	1.230039	0.051012
HDLBP	0.956281	0.880042	1.039124	0.291611
PPP1R7	1.276743	1.14474	1.423967	1.15E-05
PASK	1.12741	1.045418	1.215833	0.001852
STK25	1.193025	1.081141	1.316487	0.000443

TPO	0.722644	0.639096	0.817113	2.19E-07
PROC	0.887942	0.789143	0.999111	0.048297
ID2	1.163934	1.104525	1.226538	1.35E-08
TAF1B	1.146373	1.05952	1.240347	0.000678
HPCAL1	0.95357	0.883637	1.029039	0.221189
ODC1	1.012122	0.936788	1.093514	0.760118
BIRC6	0.964084	0.916969	1.01362	0.152496
NOL10	1.034382	0.942343	1.135411	0.477101
PLEKHB2	0.936597	0.873953	1.003731	0.063662
GORASP2	0.921444	0.840614	1.010047	0.080714
STRN	0.944589	0.870661	1.024794	0.17039
CEBPZ	1.059979	0.975072	1.152281	0.17151
PRKD3	1.023872	0.966653	1.084478	0.421362
QPCT	0.993424	0.947339	1.041752	0.785454
RAB3GAP1	0.948573	0.874536	1.028878	0.202893
SLC25A12	1.146902	1.053134	1.24902	0.001635
DLX2	1.092847	1.01764	1.173612	0.014661
LCT	1.207118	1.085373	1.342518	0.00052
SDC1	0.966893	0.871711	1.072469	0.52429
PLCL1	1.067501	0.994252	1.146146	0.071701
SLC1A4	1.061114	0.997812	1.128432	0.058734
SOS1	0.989083	0.922333	1.060662	0.758135
KYNU	1.05098	0.989898	1.115831	0.103608
WIPF1	0.824587	0.758756	0.89613	5.54E-06
COX7A2L	0.81864	0.73629	0.910199	0.000216
PNO1	1.078616	0.972862	1.195865	0.150606
PLEK	0.963573	0.89937	1.032359	0.291538
RND3	1.275746	1.153849	1.41052	2.01E-06
ATF2	0.947126	0.879213	1.020286	0.152444
THADA	1.034498	0.969372	1.104	0.306631
AAK1	1.004304	0.932678	1.081431	0.909422
TRAK2	1.070888	1.015317	1.1295	0.011767
TIA1	1.047027	0.968389	1.132051	0.248662
PCYOX1	1.079384	1.000128	1.16492	0.049617
KISS1R	0.839441	0.727949	0.968009	0.016078
EPAS1	0.94377	0.881808	1.010086	0.094855
ARID3A	0.917714	0.85739	0.982282	0.013312
SUMO1	0.978767	0.885331	1.082064	0.675029
GRIN3B	1.029523	0.945108	1.121477	0.505051
NFE2L2	0.953218	0.885773	1.025797	0.200657
MSH6	0.966189	0.895299	1.042691	0.376319
PLEKHA3	1.00442	0.931497	1.083052	0.9087
SPR	1.310773	1.226163	1.401221	1.88E-15
EPHA4	1.057594	0.983268	1.137539	0.132036
PARD3B	1.113966	1.023673	1.212223	0.012333
FARSB	1.111772	1.020936	1.210689	0.014833
ALMS1	1.023344	0.960847	1.089905	0.472933
BCL9	1.090917	1.026863	1.158967	0.004824
DHCR24	1.146853	1.092937	1.20343	2.45E-08
DNAJC16	1.04543	0.963679	1.134116	0.284879
MORN1	1.084465	0.984697	1.194341	0.099603
GPX7	0.881649	0.826627	0.940334	0.000128
CACYBP	1.158404	1.03985	1.290475	0.0076
SCP2	1.183323	1.078899	1.297855	0.000356
TPSG1	0.690626	0.610761	0.780936	3.56E-09
RALGPS2	0.996774	0.938293	1.0589	0.91658
ANGPTL1	1.001969	0.938696	1.069508	0.952866
FAM20B	1.07811	0.997214	1.165568	0.058778

TMEM59	1.10342	0.974905	1.248876	0.119307
LRRC42	0.97802	0.883943	1.082109	0.666679
MRPL37	1.088782	0.969842	1.222308	0.149546
ICMT	1.120084	1.057169	1.186744	0.000121
RPL22	0.908792	0.833538	0.990841	0.030114
CHD5	1.011068	0.935204	1.093085	0.782098
QSOX1	1.040831	0.965704	1.121804	0.29511
STXBP3	1.001061	0.922049	1.086843	0.979836
PHF13	1.035708	0.972017	1.103572	0.278589
ERRFI1	1.040578	0.950996	1.138597	0.386485
PARK7	1.176073	1.056269	1.309466	0.00309
AMPD2	0.976972	0.908093	1.051076	0.53227
MECR	1.144623	1.050158	1.247585	0.002115
KCNC4	1.097266	1.014954	1.186255	0.019646
EDEM3	1.074607	1.013571	1.139318	0.015875
WDR77	1.123966	1.033919	1.221856	0.006091
RAP1A	0.964467	0.88139	1.055374	0.431138
HDAC1	1.069247	0.966289	1.183174	0.194934
CAPZA1	1.056663	0.982198	1.136774	0.13935
S100PBP	1.003115	0.934691	1.076549	0.931235
RNF19B	1.015553	0.947291	1.088734	0.663766
SCAMP3	1.159947	1.041862	1.291416	0.006757
TRIM62	1.16242	1.068302	1.26483	0.000476
ASH1L	1.006503	0.942073	1.075338	0.847716
DLGAP3	0.810822	0.737568	0.891352	1.42E-05
SFPQ	0.922462	0.866532	0.982001	0.011435
RHOU	1.17747	1.11943	1.238518	2.38E-10
GON4L	0.985528	0.904324	1.074024	0.739688
ARHGEF2	0.976414	0.905963	1.052343	0.532171
MEF2D	0.94305	0.876192	1.015008	0.118075
DOCK7	1.009844	0.93205	1.094132	0.810711
SRM	1.01001	0.951403	1.072227	0.743989
DLEU2L	1.079261	0.948375	1.228211	0.247525
FBXO6	1.137731	1.045207	1.238446	0.002867
MAD2L2	1.221025	1.101146	1.353956	0.000152
DNAJC6	1.081741	1.031333	1.134613	0.00125
LEPR	1.095161	1.023266	1.172109	0.008695
IVNS1ABP	1.060295	1.003026	1.120834	0.03877
KIAA2013	1.039404	0.965807	1.118609	0.302336
MFN2	0.94201	0.877487	1.011277	0.098903
PRG4	1.148272	0.970677	1.358359	0.106788
MIIP	1.089609	0.990204	1.198993	0.078702
SMG7	0.963532	0.879133	1.056033	0.42703
NCF2	1.043333	0.994419	1.094652	0.083357
PDC	1.045016	0.943921	1.156938	0.396322
SLC35D1	1.034749	0.966648	1.107648	0.325404
PLA2G4A	1.106359	1.049896	1.165857	0.000156
GADD45A	0.954582	0.895076	1.018044	0.156948
PRDM2	0.89141	0.838863	0.94725	0.000209
RGS2	0.991248	0.940035	1.045251	0.745345
UCHL5	1.094591	0.995984	1.202961	0.060597
BCAS2	0.926529	0.848151	1.012149	0.090613
CTH	1.021311	0.957965	1.088846	0.518627
AGMAT	1.119014	1.024184	1.222623	0.012815
OLFML3	1.010589	0.916842	1.113921	0.832066
PLEKHM2	0.886205	0.824458	0.952575	0.001044
CRYZ	1.119876	1.041806	1.203797	0.002135
PHTF1	1.009889	0.948141	1.075659	0.759833

ZBTB17	0.9218	0.852701	0.996499	0.040542
CD58	0.961678	0.896426	1.031681	0.275727
TFAP2E	1.027262	0.951879	1.108614	0.489134
CD2	0.965309	0.910978	1.02288	0.23226
TTF2	1.09452	1.007881	1.188607	0.03183
NR5A2	0.998477	0.872663	1.142429	0.982301
KIF21B	1.073798	1.009322	1.142394	0.024218
TMEM9	1.2528	1.150749	1.363902	2.01E-07
MAP7D1	0.870402	0.8016	0.945109	0.000954
WARS2	0.984785	0.898359	1.079525	0.743547
OSCP1	1.029225	0.949894	1.115182	0.481507
MRPS15	1.00097	0.883168	1.134486	0.98789
EXOC8	1.103756	1.019005	1.195555	0.015443
GNPAT	1.094437	1.001796	1.195644	0.045529
TSNAX	0.949793	0.878585	1.026772	0.195143
RRAGC	1.012324	0.917009	1.117547	0.808184
NID1	0.95121	0.917589	0.986062	0.006441
LGALS8	1.015234	0.929888	1.108412	0.735769
NT5C1A	0.995571	0.869297	1.140186	0.94885
HPCAL4	1.002724	0.911496	1.103082	0.95543
MTR	1.050521	0.986444	1.11876	0.124809
BMP8B	1.263588	1.173917	1.360109	4.68E-10
SIPA1L2	1.016764	0.974705	1.060637	0.440521
RLF	0.930798	0.877291	0.987568	0.017591
KMO	1.161021	1.037802	1.29887	0.009103
ZNF684	1.269993	1.130299	1.426952	5.82E-05
KCNQ4	1.244555	1.101315	1.406425	0.000453
RIMS3	1.157847	1.087216	1.233066	5.02E-06
AKT3	1.073059	1.013457	1.136167	0.015589
ETV3	0.974214	0.915386	1.036824	0.41105
ACADM	1.080977	1.00237	1.165748	0.043236
SLAMF1	1.032625	0.970935	1.098235	0.307026
CD48	0.933292	0.878222	0.991814	0.026091
PADI2	1.02626	0.984142	1.07018	0.225388
SDHB	1.117522	0.980416	1.273802	0.09615
MFAP2	1.090404	0.978143	1.21555	0.118454
RPF1	1.056395	0.946092	1.179557	0.329528
KDM5B	0.937435	0.884734	0.993276	0.028634
UAP1	1.030116	0.965613	1.098928	0.368464
CTBS	1.108044	0.995091	1.233819	0.061448
KLHL12	1.089787	0.998106	1.189889	0.055151
SSX2IP	1.055994	0.990157	1.126208	0.097158
ZNHIT6	1.071474	0.979375	1.172235	0.1322
PLA2G2D	1.040907	0.935981	1.157596	0.459565
RBBP5	1.090788	1.001201	1.188391	0.046876
GBP3	1.054656	0.981557	1.133199	0.146496
GBP1	1.014237	0.962063	1.069241	0.599839
KIF17	0.926995	0.866842	0.991323	0.026791
GPR89A	1.058623	0.963018	1.16372	0.238138
CD160	1.017846	0.930564	1.113315	0.69898
ECE1	1.074875	1.022681	1.129732	0.004469
HMGCL	1.032629	0.94456	1.128909	0.480226
GALE	1.034787	0.950704	1.126307	0.42904
ID3	0.999894	0.946378	1.056436	0.99699
CR2	1.043954	0.948107	1.149491	0.381328
CD46	0.995564	0.921768	1.075268	0.909912
PRPF3	1.042645	0.965122	1.126394	0.289423
APH1A	0.996243	0.910686	1.089837	0.934514

SLC2A1	1.056349	1.003136	1.112384	0.037646
EBNA1BP2	1.145204	1.059012	1.23841	0.000683
CDC20	1.110964	1.043725	1.182535	0.000955
MPL	0.938803	0.896828	0.982743	0.006813
ARTN	1.057321	1.005789	1.111493	0.028787
IPO13	1.113957	1.038251	1.195182	0.002653
ATP6V0B	0.987819	0.887976	1.098888	0.821641
B4GALT2	0.988435	0.911853	1.071448	0.77739
ERI3	1.136984	1.033973	1.250259	0.008063
PTCH2	1.110743	1.010428	1.221016	0.029647
AKR1A1	1.131881	1.038306	1.23389	0.004897
PRDX1	1.183075	1.110219	1.260711	2.17E-07
PIK3R3	1.333295	1.222598	1.454014	7.79E-11
BLZF1	0.982183	0.914578	1.054785	0.621249
SLC19A2	0.975222	0.903226	1.052957	0.521391
FAAH	1.089053	1.032338	1.148885	0.00177
NSUN4	0.972335	0.88184	1.072118	0.573535
TMED5	0.92364	0.849402	1.004366	0.063161
DR1	1.049362	0.960965	1.14589	0.283207
CNN3	1.16569	1.092574	1.243699	3.50E-06
F3	1.12419	1.070603	1.180458	2.63E-06
ABCD3	1.03538	0.953311	1.124514	0.409276
VAMP4	0.999628	0.902339	1.107407	0.994321
DPH5	0.850184	0.770643	0.937936	0.001202
FASLG	1.097173	0.980782	1.227376	0.105059
PTBP2	0.954742	0.896432	1.016845	0.149745
TNFSF4	1.070966	1.010823	1.134688	0.020072
PRDX6	1.058934	0.947238	1.183802	0.313997
DARS2	1.003872	0.939121	1.073088	0.909547
IRF6	1.19212	1.08542	1.309309	0.000239
RCAN3	1.083498	1.008419	1.164166	0.028612
SYF2	0.876556	0.791102	0.971239	0.011816
SLC35A3	1.075896	0.969193	1.194347	0.169827
RCOR3	0.976426	0.900604	1.058631	0.562962
STMN1	1.055072	0.986385	1.128543	0.118565
MAN1C1	1.105264	1.028283	1.188009	0.006585
NEK2	1.143829	1.068902	1.224008	0.000101
RPS6KA1	1.220708	1.137041	1.310532	3.69E-08
DHDDS	1.045409	0.95131	1.148815	0.356127
NENF	1.049312	0.965063	1.140915	0.259658
NSL1	1.047344	0.944631	1.161224	0.379748
ARID1A	0.965164	0.887642	1.049456	0.406536
CENPF	1.111886	1.051766	1.175442	0.000184
RPA2	1.10536	1.005851	1.214714	0.037418
PPP1R8	1.071856	0.982089	1.169828	0.119952
STX12	0.944192	0.870191	1.024485	0.16788
SLC5A9	1.250717	1.12509	1.390371	3.44E-05
OSBPL9	0.908357	0.851122	0.96944	0.003796
TXNDC12	1.055281	0.931543	1.195455	0.397793
RCN2	0.97777	0.889601	1.074678	0.64104
MUC5B	1.024879	0.900013	1.167068	0.71084
CTSD	1.197449	1.118689	1.281753	2.09E-07
STAG1	0.974374	0.902639	1.05181	0.505833
STK11	1.043357	0.961724	1.131919	0.30722
MMP8	1.030088	1.002996	1.057912	0.029264
APOA1	1.210073	1.112466	1.316243	8.84E-06
KPTN	1.078229	0.993238	1.170493	0.072178
RPS25	0.945908	0.881018	1.015577	0.125109

KIF14	1.078417	1.00314	1.159344	0.040868
DDX59	1.004501	0.912528	1.105743	0.926974
ATF6	0.9599	0.889828	1.03549	0.289961
CRYGD	1.055934	1.008593	1.105499	0.020043
MREG	0.933584	0.87589	0.995078	0.034722
FASTKD2	1.084799	0.987868	1.191241	0.08831
NRP2	0.861306	0.773698	0.958834	0.006371
CREB1	1.096798	1.002481	1.199989	0.044013
KLF7	0.929969	0.86663	0.997938	0.043661
B4GALT6	0.967693	0.918676	1.019326	0.215629
CA14	1.036112	0.973606	1.102632	0.263813
SPCS2	1.062194	0.977735	1.153948	0.15349
USP35	0.891914	0.836421	0.951089	0.000483
CASP8AP2	1.08989	1.014575	1.170796	0.018472
HMGN3	0.916717	0.859644	0.977579	0.008016
ANKRD13(	1.013801	0.929387	1.105882	0.757316
PHF3	0.937848	0.877676	1.002145	0.057876
PLAGL1	0.877852	0.823352	0.935961	6.78E-05
FBXO30	0.968254	0.904347	1.036679	0.354447
TNFAIP3	0.986245	0.933745	1.041697	0.619706
AKAP7	1.18542	1.099128	1.278487	1.03E-05
RAB32	0.897419	0.853493	0.943605	2.37E-05
MYB	1.014194	0.955071	1.076978	0.645568
ALDH8A1	1.048118	0.981302	1.119483	0.16201
SGK1	1.023467	0.973367	1.076146	0.36503
RNF146	0.989036	0.921152	1.061923	0.761223
ARG1	0.99395	0.954036	1.035533	0.771655
PMFBP1	0.966404	0.879366	1.062057	0.47792
FBXL5	1.021963	0.942516	1.108107	0.598774
MED28	0.972834	0.904356	1.046498	0.459573
SLC16A7	1.011038	0.943213	1.08374	0.756687
ZNF430	0.989934	0.918994	1.066351	0.789728
VAMP8	1.003223	0.933496	1.078158	0.930236
DCLRE1B	1.091479	0.990728	1.202476	0.076488
FOXO3	0.908221	0.856707	0.962832	0.001232
ARMC2	1.073967	0.96241	1.198456	0.202219
GHRH	0.965804	0.867368	1.075412	0.525827
RPN2	1.075221	0.978227	1.181833	0.132688
TGIF2	0.9345	0.870056	1.003717	0.06314
OLFM3	1.0933	1.009285	1.184309	0.028779
PKD2	1.019037	0.959238	1.082563	0.541073
ABCG2	1.08777	1.010967	1.170408	0.024327
SPP1	1.01472	0.953182	1.080231	0.6471
STBD1	1.153606	1.01318	1.313494	0.030952
CCNI	0.869795	0.793703	0.953182	0.002822
RARRES1	1.015404	0.919571	1.121224	0.76248
MFSD1	0.991585	0.928929	1.058466	0.799679
RAB3GAP2	1.036532	0.962902	1.115793	0.339876
PPL	0.896905	0.841265	0.956224	0.000869
UBN1	0.902528	0.838092	0.971917	0.006654
KLF12	1.083628	1.027162	1.143197	0.003266
UCHL3	1.153123	1.047494	1.269405	0.003654
HS1BP3	1.051033	0.950465	1.162242	0.332075
WDR35	1.073731	1.006401	1.145565	0.031313
CCND2	0.923729	0.884715	0.964464	0.000314
ELL2	0.993372	0.947359	1.04162	0.783456
CYP20A1	1.096746	1.002943	1.199323	0.042932
NDUFB3	1.157858	1.064507	1.259394	0.000632

GTF3C3	0.9742	0.89462	1.06086	0.547728
SATB2	1.175571	1.084689	1.274068	8.14E-05
UBE2B	0.965001	0.889411	1.047015	0.391982
TRPM6	1.053982	0.97296	1.141751	0.197654
KLF9	0.963366	0.919157	1.009702	0.119437
TJP2	1.32089	1.179199	1.479607	1.53E-06
ITGB1BP1	1.167165	1.045379	1.303138	0.005973
CPSF3	1.000722	0.911727	1.098404	0.987882
PIGZ	0.939933	0.854992	1.033312	0.199887
SENP5	0.972794	0.91406	1.035301	0.385331
CCDC92	1.101876	1.013498	1.197961	0.022949
HEATR1	1.00247	0.937014	1.072499	0.942909
RAD23B	0.859134	0.792528	0.931338	0.000226
FKBP15	0.991133	0.922321	1.065079	0.808317
CTNNAL1	0.941945	0.883662	1.004073	0.066469
SET	1.018114	0.92865	1.116197	0.702048
GLE1	1.118585	1.028532	1.216522	0.008873
RAB14	0.976716	0.896947	1.063578	0.587832
TRIM32	1.133167	1.061433	1.20975	0.000179
FBXW2	0.98139	0.908949	1.059605	0.63112
PHF19	0.877789	0.802538	0.960096	0.004365
NEK6	1.059683	0.988721	1.135738	0.10117
BSPRY	0.996342	0.940638	1.055345	0.900642
PPP6C	0.954384	0.861787	1.056929	0.369906
NDUFA8	1.127573	1.053166	1.207237	0.000566
HDHD3	1.238497	1.132895	1.353944	2.55E-06
RBM18	1.024237	0.929151	1.129053	0.629993
SLC46A2	0.995669	0.902245	1.098766	0.931193
HSDL2	1.069671	0.985446	1.161094	0.107487
MAPKAP1	1.050378	0.966157	1.141941	0.249078
NR4A3	0.96462	0.922719	1.008423	0.111887
INVS	1.017723	0.941796	1.099771	0.656985
GALNT12	1.35141	1.250097	1.460934	3.61E-14
DENND1A	0.9282	0.863134	0.998172	0.044503
ALG2	1.010903	0.929671	1.099234	0.799706
CSF3R	0.849812	0.80658	0.895361	1.00E-09
KDSR	0.993317	0.904449	1.090917	0.888477
VPS4B	1.000779	0.91457	1.095115	0.986481
ONECUT2	1.307067	1.205008	1.41777	1.08E-10
ZBTB45	0.961594	0.872649	1.059604	0.429034
YLPM1	0.974949	0.894866	1.062199	0.561816
PROX2	1.037811	0.955801	1.126857	0.376891
VSX2	0.977001	0.876369	1.08919	0.674832
FCF1	0.998302	0.912837	1.091768	0.970304
PGF	0.964065	0.890869	1.043275	0.363673
IFI27L2	1.182883	1.113825	1.256222	4.44E-08
NEK9	1.084387	1.013461	1.160277	0.018906
ACYP1	1.2045	1.111612	1.30515	5.52E-06
NPC2	0.977676	0.90407	1.057276	0.571854
DNAL1	1.063393	0.951326	1.188661	0.279357
ACOT2	1.148628	1.051323	1.254938	0.002154
LTBP2	1.044251	0.934942	1.166341	0.442765
MLH3	1.054707	0.983318	1.131277	0.136351
TTLL5	1.040077	0.953196	1.134877	0.377285
FLVCR2	1.069943	0.9984	1.146612	0.055541
ABCD4	1.01338	0.93425	1.099213	0.748647
DLST	0.88205	0.824915	0.943143	0.00024
TGFB3	1.140044	1.024054	1.269172	0.016659

RBM25	0.978515	0.891155	1.074437	0.648959
ALDH6A1	1.102238	1.020685	1.190307	0.013064
GPR68	1.008806	0.93744	1.085606	0.814821
EIF2B2	1.163333	1.0411	1.299917	0.00756
COQ6	1.143837	1.008126	1.297816	0.037018
ZNF410	0.986779	0.922942	1.055031	0.696506
RHOQ	0.939638	0.869528	1.015401	0.115563
GPR75	0.969355	0.898046	1.046325	0.42465
SUPT7L	1.077389	0.9634	1.204865	0.191401
KLHL29	1.05932	0.983303	1.141214	0.129322
DNMT3A	1.040425	0.971436	1.114315	0.257596
TMEM214	1.08156	0.987779	1.184245	0.090216
ATAD2B	1.00126	0.942416	1.063778	0.967497
FKBP1B	1.11277	1.01045	1.225452	0.029917
ATL2	0.954147	0.887761	1.025498	0.202076
YPEL5	0.953091	0.88192	1.030007	0.225009
FAM98A	1.04215	0.951687	1.141212	0.37286
YIPF4	1.019535	0.947825	1.096669	0.603124
AFTPH	0.9753	0.902198	1.054326	0.529242
CNRIP1	1.010381	0.964211	1.058762	0.665185
BCL11A	1.108422	1.035527	1.186448	0.003019
CRIPT	1.015552	0.945635	1.090638	0.671542
EPCAM	0.952597	0.902311	1.005685	0.079244
SLC17A5	1.00886	0.934121	1.08958	0.822271
OGFRL1	0.945778	0.881998	1.01417	0.117589
IDE	1.090693	0.997559	1.192521	0.05661
ELOVL3	1.316907	1.201281	1.443663	4.33E-09
IFIT3	1.061537	1.020015	1.104749	0.003352
NKX2-3	1.080158	1.04929	1.111934	1.86E-07
IFIT2	1.055745	1.009102	1.104544	0.018623
GPAM	1.106473	1.006466	1.216416	0.036322
CUTC	1.073255	0.965756	1.19272	0.189222
CNNM1	1.240052	1.142457	1.345984	2.68E-07
MXI1	0.97812	0.909563	1.051845	0.550723
SMNDC1	0.973673	0.886337	1.069614	0.57792
HELLS	1.109315	1.042691	1.180195	0.001028
TCTN3	1.106799	1.009854	1.213051	0.030035
AVPI1	1.059716	0.985662	1.139333	0.116595
KCNIP2	1.097612	1.001485	1.202966	0.046404
GOT1	1.21773	1.116429	1.328223	8.78E-06
SFRP5	1.115787	1.052395	1.182998	0.000241
GNA13	0.909968	0.849829	0.974363	0.006842
HOXB8	1.069157	1.024438	1.115829	0.002158
HOXB5	1.063853	1.032268	1.096404	5.69E-05
HOXB3	1.053432	1.021004	1.086891	0.001103
DUSP1	0.958341	0.904748	1.015109	0.147273
PANK3	1.011017	0.941407	1.085774	0.76339
TEK	1.110829	1.014579	1.21621	0.023029
RCL1	1.100802	1.015861	1.192846	0.019075
INSL6	1.053403	0.983988	1.127715	0.134692
MLANA	0.9989	0.923984	1.079891	0.977928
CD274	1.217984	1.118125	1.32676	6.24E-06
NUP43	1.024728	0.963172	1.090219	0.439631
MTHFD1L	0.898469	0.840251	0.960721	0.001734
PCMT1	1.073776	0.980086	1.176422	0.12648
PLEKHG1	1.039953	0.976415	1.107626	0.223248
MYCT1	1.157006	1.108348	1.2078	2.88E-11
WDR55	1.132693	1.033429	1.241491	0.007752

ARAP3	0.993452	0.940905	1.048932	0.812691
SLC25A2	1.086018	0.973368	1.211705	0.139716
TNN	1.042254	0.951124	1.142115	0.375335
MRPS14	1.21524	1.093292	1.350791	0.000303
CENPL	1.00863	0.927628	1.096704	0.840567
ACAT2	1.214019	1.090587	1.351422	0.000392
TCP1	0.989149	0.898306	1.08918	0.82434
SNX19	1.052255	0.976408	1.133993	0.182049
KCNJ5	0.868368	0.742121	1.016093	0.078272
ARR3	0.969708	0.884733	1.062845	0.510932
PDZD11	1.186162	1.064955	1.321163	0.001907
SLC10A7	0.993788	0.912896	1.081848	0.885618
NUDCD1	1.072467	0.98571	1.166861	0.104046
ENY2	0.950592	0.858686	1.052334	0.328713
MASTL	0.945512	0.874185	1.022659	0.161495
KIAA1217	1.088111	1.033093	1.146059	0.001424
PLXDC2	1.039435	0.992518	1.088569	0.100743
EPC1	0.99538	0.907855	1.091344	0.921456
IQSEC3	1.000688	0.942672	1.062275	0.981992
CCDC77	1.007618	0.915744	1.108709	0.876369
TAF12	0.985055	0.877307	1.106036	0.798901
MTRF1	1.044108	0.951678	1.145516	0.361406
DNAJC15	1.110455	1.002356	1.23021	0.044962
UFM1	0.971335	0.895472	1.053624	0.483316
WBP4	0.94068	0.843668	1.048848	0.270825
ELF1	0.876525	0.817644	0.939646	0.000204
SMAD9	1.092024	0.94871	1.256988	0.220032
HSPH1	1.06859	1.011822	1.128543	0.017221
KBTBD7	1.079258	1.002526	1.161863	0.04266
ALG5	1.078095	0.960136	1.210545	0.203413
EXOSC8	1.082468	0.970818	1.206959	0.153653
ETF1	0.879649	0.820897	0.942606	0.000277
TGFBI	1.030866	0.990211	1.07319	0.138661
FAM53C	0.854055	0.799651	0.91216	2.63E-06
SIL1	0.992607	0.903232	1.090826	0.8775
PAIP2	0.952861	0.859617	1.056219	0.358102
MYOT	1.065591	0.982893	1.155246	0.123236
KDM3B	0.962464	0.892125	1.038349	0.323121
EGR1	0.987856	0.949461	1.027804	0.545783
SERP1	0.892574	0.798426	0.997823	0.045686
PLS1	1.309254	1.216475	1.409109	6.70E-13
ZFP30	0.948311	0.874839	1.027953	0.197084
NR2C1	0.948145	0.872461	1.030395	0.20965
UTP20	1.046833	0.974563	1.124462	0.209837
TMPO	1.072542	0.997425	1.153315	0.058707
ARL1	1.124371	1.023393	1.235312	0.014623
GLT8D2	1.048805	0.929051	1.183996	0.441111
SOCS2	1.169272	1.130858	1.208991	4.50E-20
NFYB	1.102712	0.994209	1.223055	0.064303
APAF1	1.050423	0.987753	1.11707	0.117036
DUSP4	1.086412	1.031714	1.144009	0.001663
CLU	1.049547	1.012626	1.087813	0.008129
TNFRSF10	0.939947	0.8806	1.003295	0.062729
SORBS3	1.042174	0.972484	1.116859	0.242071
PTK2B	0.994028	0.924979	1.068231	0.870459
CHRNA2	0.974721	0.898218	1.05774	0.539253
PPP3CC	0.985894	0.903481	1.075826	0.749758
PDLIM2	1.291383	1.18345	1.409159	9.34E-09

EPHX2	1.180171	1.10695	1.258237	4.00E-07
RNF170	1.080715	0.992485	1.176788	0.074039
NPPB	0.977165	0.901067	1.05969	0.576554
UBIAD1	1.160213	1.051012	1.28076	0.003214
TARDBP	0.993806	0.923137	1.069885	0.868874
TNFRSF8	1.052234	0.93708	1.181539	0.389234
ZNF706	0.990942	0.906896	1.082776	0.840515
LYPLA1	0.939802	0.863529	1.022812	0.150531
CRISPLD1	1.087092	1.017324	1.161645	0.013607
COPS5	1.11454	0.998813	1.243676	0.052535
RDH10	1.289828	1.185508	1.403328	3.33E-09
EPX	0.916201	0.891776	0.941295	2.18E-10
AKAP1	1.158103	1.084744	1.236422	1.10E-05
COIL	1.130848	1.035876	1.234527	0.006005
TRIM25	1.114253	1.036821	1.197469	0.003241
SCPEP1	1.071994	1.021936	1.124504	0.004382
SPOP	1.088095	0.99443	1.190582	0.066012
SLC35B1	1.070109	0.958935	1.194172	0.226001
TEX14	0.965552	0.912486	1.021703	0.224181
FAM117A	0.933004	0.869021	1.001698	0.055726
NCAPH	1.105471	1.027198	1.189709	0.007448
MND1	1.24241	1.09974	1.40359	0.000487
TRIM6	0.962825	0.886516	1.045702	0.368536
ADCY7	1.141811	1.083466	1.203297	7.21E-07
TSHZ3	1.018352	0.943954	1.098614	0.638474
ECHDC2	1.047439	0.97629	1.123773	0.196573
TAS2R10	1.130524	1.013495	1.261065	0.027779
PRB2	0.985988	0.86731	1.120906	0.829259
PYROXD1	1.002018	0.910989	1.102143	0.966907
PSPC1	0.981027	0.912192	1.055056	0.605804
ZNF549	1.052438	0.962758	1.150472	0.260699
A1BG	0.882767	0.792607	0.983182	0.023297
ZSCAN18	0.982643	0.918151	1.051666	0.613192
ZNF211	1.161181	1.062337	1.269221	0.000994
PDZRN3	1.066102	0.962159	1.181274	0.221353
LHX4	1.095141	1.006385	1.191724	0.035068
RNF2	1.054005	0.963764	1.152696	0.249426
SEC22A	1.176304	1.062613	1.30216	0.001742
CSTA	0.982808	0.944657	1.022499	0.390615
DPPA4	1.149913	1.099084	1.203094	1.40E-09
POPDC2	1.179742	1.044817	1.33209	0.007643
B4GALT4	0.966247	0.889278	1.049879	0.417542
CD80	1.095136	1.004534	1.19391	0.039146
KIF18A	1.049063	0.980337	1.122608	0.165897
MAPK8IP1	0.985652	0.918026	1.05826	0.690255
CRY2	0.987273	0.919784	1.059714	0.722934
PEX16	1.109506	0.992019	1.240908	0.068812
DEPDC7	0.805013	0.753465	0.860087	1.33E-10
CAT	1.083872	1.021948	1.149547	0.00729
PILRB	0.960523	0.899543	1.025637	0.22876
ZMYM2	0.916877	0.859058	0.978588	0.009021
GJB6	1.074841	1.013044	1.140409	0.016898
TBC1D15	0.945412	0.876928	1.019245	0.143435
HCRTR1	0.992865	0.931473	1.058304	0.825975
ZCCHC17	1.06654	0.965396	1.17828	0.205082
FABP3	0.978459	0.912044	1.04971	0.543707
KHDRBS1	0.931298	0.851788	1.018229	0.118002
TMEM39B	0.984424	0.896285	1.08123	0.742885

CCRL2	1.072723	0.992937	1.158921	0.075039
CCR2	1.031509	0.987047	1.077973	0.167589
POLR3GL	1.105131	1.005545	1.214581	0.038012
TNFSF10	1.118891	1.060812	1.18015	3.62E-05
ZNF639	0.937266	0.863996	1.01675	0.11876
PIK3CA	0.922755	0.864701	0.984708	0.015318
PDS5A	0.996037	0.922673	1.075233	0.918969
TMEM156	0.844045	0.780082	0.913253	2.48E-05
LIAS	1.076895	0.943468	1.229192	0.272334
ZSCAN20	1.294747	1.170517	1.432161	5.19E-07
CLCC1	0.919184	0.855453	0.987663	0.021529
GPSM2	0.935598	0.862725	1.014627	0.107617
GTDC1	1.041746	0.942716	1.151178	0.422274
CXCR4	1.005733	0.947726	1.067289	0.850408
ZRANB3	0.998036	0.917321	1.085854	0.96356
ACVR2A	1.219958	1.103331	1.348913	0.000105
POLK	0.975542	0.902261	1.054776	0.534281
FLT3	0.956324	0.921558	0.992402	0.018096
RPL21	0.872591	0.798828	0.953164	0.002491
MTIF3	0.982532	0.892661	1.081452	0.718809
GTF3A	0.987052	0.893021	1.090983	0.798604
RASL11A	0.972278	0.890458	1.061615	0.530765
UBL3	0.974599	0.898026	1.057701	0.537711
FYTTD1	1.017479	0.927917	1.115687	0.71243
XPNPEP2	0.790984	0.700697	0.892905	0.00015
SASH3	1.053672	0.980598	1.132192	0.153962
OCRL	0.980029	0.912065	1.053058	0.582227
MRPS2	1.017239	0.925865	1.117631	0.721894
FMOD	1.145805	1.020808	1.286107	0.020921
LAX1	0.917511	0.866032	0.972051	0.003477
KIAA1191	1.057138	0.955939	1.169049	0.279129
COPA	0.983192	0.913526	1.05817	0.651218
CD244	0.914934	0.869006	0.963289	0.000716
LY9	0.854606	0.795315	0.918317	1.85E-05
HS3ST2	1.238705	1.023843	1.498658	0.027638
RBBP6	0.965513	0.889542	1.047971	0.401268
ZC3H7A	0.951651	0.876699	1.033011	0.236412
SERAC1	1.112485	1.011776	1.223219	0.027682
ANXA11	0.879596	0.803214	0.963243	0.005641
ZNF205	1.15254	1.050908	1.264001	0.002577
RPL5	0.85674	0.788121	0.931334	0.000283
ODF2L	0.944016	0.871894	1.022104	0.155381
SPATA1	1.079007	0.976859	1.191838	0.133988
LRRC39	1.077537	0.968089	1.199358	0.171779
RWDD3	1.222148	1.096304	1.362437	0.000297
ZNF644	0.96494	0.912042	1.020907	0.214731
CCDC18	0.997605	0.922452	1.078881	0.952147
RPAP2	1.006452	0.922175	1.098431	0.885395
BBS9	1.023274	0.946559	1.106205	0.562834
PMS2	0.9936	0.908331	1.086874	0.888462
ZMIZ2	0.969993	0.884953	1.063205	0.51518
OCM	1.165899	1.050881	1.293506	0.003774
EEPD1	1.046557	0.977769	1.120186	0.18957
KIAA0087	1.207748	1.12515	1.296411	1.77E-07
KLHL7	0.956934	0.848064	1.079779	0.475001
HERPUD2	0.859597	0.791844	0.933148	0.000304
CBX3	1.040933	0.939899	1.152829	0.441234
HNRNPA2	0.979792	0.899554	1.067187	0.639567

WIPF3	0.826281	0.745678	0.915597	0.000269
NPY	1.110438	1.024986	1.203015	0.010347
FAM126A	1.042001	0.960498	1.13042	0.322131
HOXA7	1.180284	1.129752	1.233076	1.13E-13
INHBA	1.031885	0.977822	1.088937	0.252983
FKBP9	1.050934	0.98238	1.124272	0.148892
ARL4A	1.008798	0.94259	1.079656	0.800336
POLM	1.050288	0.970517	1.136617	0.223445
RAMP3	1.234197	1.129075	1.349107	3.61E-06
TWIST1	0.947669	0.87545	1.025845	0.183838
SMU1	0.993649	0.912131	1.082453	0.884022
GLIPR2	1.054003	0.997918	1.11324	0.059393
CLTA	1.067347	0.939042	1.213182	0.318552
RECK	1.398945	1.309376	1.494642	2.67E-23
SPINK4	0.923973	0.874267	0.976505	0.005068
ACO1	1.011235	0.931685	1.097576	0.789273
DCAF10	1.088634	0.999469	1.185753	0.051442
KIAA1549	0.809021	0.75832	0.863111	1.38E-10
TRIM24	0.91553	0.854679	0.980713	0.011905
CALD1	1.17067	1.093597	1.253175	5.76E-06
NUDT10	0.971136	0.900676	1.047108	0.44598
NEUROG3	1.030508	0.935336	1.135363	0.543298
PLAU	0.972633	0.923464	1.024419	0.294445
SRGN	1.024721	0.976419	1.075412	0.321545
BICC1	1.151885	1.052652	1.260473	0.002096
CISD1	1.103771	0.993514	1.226263	0.065947
EGR2	1.075272	1.009541	1.145283	0.024132
ECD	0.930295	0.856138	1.010876	0.088241
P4HA1	1.030783	0.951585	1.116573	0.45729
SLC25A16	1.109896	1.011128	1.21831	0.028329
ZWINT	1.165143	1.089739	1.245764	7.55E-06
VPS26A	1.017511	0.931642	1.111296	0.699562
RBM19	1.036486	0.95913	1.12008	0.365186
CIT	1.135394	1.056849	1.219778	0.000517
IFT81	1.076061	0.968059	1.196112	0.174328
ACADS	0.921981	0.845019	1.005952	0.067771
HVCN1	1.007737	0.948464	1.070715	0.80321
NME2P1	0.962995	0.892623	1.038914	0.330092
DDX54	1.002857	0.91897	1.094402	0.948957
MED13L	0.952912	0.892294	1.017647	0.150347
CDKN2C	1.120615	1.063284	1.181038	2.14E-05
RNF11	0.887648	0.821135	0.959547	0.002708
RASSF8	1.043214	0.96618	1.126391	0.279732
ITPR2	0.912193	0.866901	0.959852	0.000405
CCDC91	1.003897	0.931027	1.082471	0.919419
NECAB1	1.170967	1.091236	1.256523	1.15E-05
WWP1	1.02956	0.954533	1.110485	0.450488
ACOT9	1.031943	0.944515	1.127464	0.486335
PRDX4	1.124432	1.055002	1.198431	0.00031
PKN1	0.905238	0.832934	0.983819	0.019075
GIPC1	1.256941	1.161604	1.360103	1.33E-08
EBPL	0.939917	0.872449	1.012604	0.103016
ATP7B	1.065698	0.998476	1.137446	0.055608
ZC3H13	0.988522	0.895007	1.091809	0.819902
GUCY1B2	0.950901	0.863428	1.047236	0.306525
NLN	1.084607	1.008535	1.166418	0.028596
CENPK	1.091281	1.012747	1.175905	0.021885
OPTN	1.130903	1.071977	1.193067	6.61E-06

ATF1	1.064492	0.96613	1.172869	0.206441
TSFM	1.183828	1.026114	1.365783	0.020703
ARHGAP9	1.070249	0.98176	1.166715	0.123101
NCKAP1L	1.021597	0.949572	1.099086	0.566776
MMP19	0.999521	0.933807	1.069859	0.988981
PFDN5	1.062209	0.979881	1.151454	0.142594
SPATS2	1.098066	1.009736	1.194123	0.028786
ORMDL2	1.091544	0.979938	1.21586	0.111453
NR4A1	0.939735	0.894996	0.98671	0.012506
PDE1B	1.048129	0.982632	1.117991	0.153352
CDK2	1.199885	1.09439	1.31555	0.000104
LRP1	1.012141	0.975048	1.050644	0.52641
NFE2	1.028299	0.974074	1.085543	0.312677
IKZF4	1.091408	0.984374	1.210082	0.096733
SMUG1	1.229269	1.117922	1.351707	2.04E-05
TUBA1B	1.065903	0.992784	1.144407	0.07837
KBTBD4	1.006538	0.915508	1.106619	0.892822
SARDH	1.22165	1.142293	1.30652	5.15E-09
DBH	1.233848	1.117567	1.362228	3.17E-05
ATPAF1	1.2204	1.106746	1.345727	6.51E-05
STIL	1.0674	0.990762	1.149967	0.086197
HJURP	1.101539	1.041154	1.165426	0.000774
COL10A1	1.075912	0.9945	1.163988	0.068368
AMD1	0.982146	0.910925	1.058936	0.639045
USP45	1.175112	1.091325	1.265331	1.91E-05
MORF4L2	0.966307	0.887824	1.051729	0.427773
RAB9B	1.388801	1.259535	1.531335	4.43E-11
RAB9A	0.919626	0.829043	1.020107	0.113264
METTL8	1.074747	0.989914	1.16685	0.085739
TTC21B	1.015846	0.947543	1.089073	0.657978
NMI	1.085884	0.980781	1.20225	0.112662
TNFAIP6	1.041811	0.989908	1.096436	0.116198
BAZ2B	1.006483	0.939327	1.078441	0.854473
SLC36A1	1.042288	0.98159	1.10674	0.176059
LPGAT1	0.912266	0.84822	0.981149	0.013421
BATF3	1.240051	1.110309	1.384953	0.000136
G0S2	1.018466	0.98741	1.050499	0.246832
KCNJ2	1.351074	1.241772	1.469997	2.73E-12
RAP2C	0.956742	0.87649	1.044342	0.322509
EXOSC9	1.12889	1.018912	1.250738	0.020437
PLA2G12A	1.112319	1.020766	1.212084	0.015142
B9D2	1.149872	1.05072	1.25838	0.002403
PFKFB2	1.03029	0.974585	1.089178	0.2927
RAB38	1.109462	1.048217	1.174286	0.000337
MXD4	0.927036	0.862619	0.996264	0.039223
CKS2	0.982188	0.913268	1.056309	0.628265
ACSL3	0.972453	0.898424	1.052582	0.489285
CHPF	1.05498	0.985657	1.129179	0.122738
DNPEP	1.117551	1.004882	1.242852	0.040385
OBSL1	0.999435	0.951937	1.049303	0.981848
FAM124B	1.055581	1.000865	1.113289	0.046392
SLC12A4	1.075517	1.006384	1.1494	0.031739
GCNT7	0.98809	0.939112	1.039624	0.644157
PI3	1.114405	1.051815	1.180719	0.00024
SNX21	1.093494	0.9977	1.198485	0.056037
SLPI	0.987678	0.954621	1.02188	0.475346
TTPAL	1.069015	0.997203	1.145999	0.059969
PREX1	1.020574	0.950422	1.095904	0.57514

KCNS1	1.03022	0.967573	1.096924	0.352311
SDC4	0.9856	0.945977	1.026883	0.488414
NCOA3	0.948778	0.881212	1.021525	0.163028
PIGT	1.089906	0.973747	1.219921	0.134323
MATN4	1.081592	1.012684	1.155188	0.019531
NCOA5	1.124607	1.034949	1.222031	0.005599
VAPB	0.999713	0.911236	1.096782	0.995164
PARD6B	1.286071	1.182495	1.398719	4.28E-09
CHD6	0.960874	0.896121	1.030305	0.262182
PLCG1	0.962877	0.91563	1.012562	0.14057
TOX2	1.119054	1.052558	1.18975	0.00032
ARFGEF2	0.932194	0.879041	0.988561	0.019077
ZNFX1	0.988878	0.923926	1.058396	0.746949
ZNF831	1.040324	0.959339	1.128145	0.33904
CSE1L	1.030675	0.955479	1.11179	0.434394
RAB22A	0.947273	0.864702	1.037728	0.244385
STAU1	1.038075	0.944541	1.140872	0.437951
CDH26	0.918579	0.848187	0.994813	0.036816
SNAI1	0.974625	0.930262	1.021104	0.279556
MOCS3	1.216451	1.09838	1.347215	0.000169
STX16	1.060542	0.975968	1.152446	0.165664
PPP4R1L	0.932888	0.874415	0.995273	0.035427
PMEPA1	0.995219	0.934961	1.059361	0.880456
RNF114	0.945308	0.860943	1.037941	0.238313
DDX27	0.938672	0.852003	1.034157	0.200388
BCAS4	1.075681	1.001728	1.155094	0.044701
TP53TG5	1.023481	0.961312	1.089671	0.467895
ZBP1	1.114466	1.037612	1.197013	0.002952
NEURL2	1.119415	0.986862	1.269773	0.079379
MTRR	0.977116	0.894661	1.06717	0.606783
FASTKD3	1.201363	1.083741	1.331751	0.000484
PEPD	1.192548	1.100816	1.291924	1.62E-05
IQSEC2	1.045083	0.97291	1.122609	0.22714
VAMP7	0.945154	0.861257	1.037223	0.234299
IL9R	0.973469	0.8898	1.065005	0.557585
XG	0.890821	0.784126	1.012034	0.075703
STAMBP	1.074503	0.97389	1.18551	0.151991
NAGK	1.038378	0.963149	1.119484	0.326372
MCEE	1.001492	0.917097	1.093653	0.973517
PAIP2B	1.24553	1.145547	1.354241	2.71E-07
SNRNP27	1.210693	1.091682	1.342678	0.000293
MPHOSPH	1.13059	1.022369	1.250267	0.016807
IL17C	1.011676	0.937963	1.091181	0.763619
ATP8A1	1.016188	0.960796	1.074772	0.574449
USP22	0.931398	0.870572	0.996474	0.039163
POF1B	1.091018	0.989336	1.203151	0.080955
HIF3A	0.858112	0.765969	0.961339	0.008284
ZNF576	1.026849	0.944647	1.116204	0.533699
IRGC	1.027827	0.941757	1.121762	0.538481
ZNF45	1.072874	0.988178	1.16483	0.093639
LYPD3	1.092126	1.005284	1.186469	0.037103
CEACAM8	1.001707	0.973959	1.030245	0.905303
USP9X	0.972204	0.916352	1.03146	0.350383
CRISP2	1.165138	1.088267	1.24744	1.14E-05
F13A1	0.958527	0.926945	0.991185	0.013213
TRERF1	0.935414	0.880328	0.993948	0.031084
PACSIN1	0.968853	0.898565	1.044638	0.410238
BTN2A2	1.086794	1.013581	1.165295	0.019333

SIRT5	0.9915	0.913798	1.07581	0.837572
MRS2	1.039659	0.950401	1.137299	0.395767
WRNIP1	1.012285	0.935986	1.094805	0.76007
SNRPC	1.020351	0.932282	1.116739	0.661787
SERPINB6	1.066637	0.999872	1.13786	0.050456
XPO5	1.092524	1.010697	1.180976	0.025892
ABCC10	1.104575	1.028567	1.1862	0.006251
PEX6	1.04788	0.975181	1.125999	0.202348
NQO2	1.091646	1.00172	1.189645	0.045593
UNC5CL	1.023258	0.93877	1.115349	0.60104
AARS2	1.145291	1.063882	1.232929	0.000311
ZNF391	1.049691	0.960571	1.14708	0.28403
RPS10	1.090065	1.001183	1.186838	0.046899
MOCS1	1.297779	1.17289	1.435966	4.44E-07
MED20	1.205724	1.112143	1.307178	5.67E-06
OR2B6	1.015262	0.898984	1.146579	0.807183
TBCC	1.116794	1.012535	1.231788	0.027168
SPDEF	0.962704	0.851902	1.087918	0.542356
TCP11	1.000228	0.844436	1.184762	0.997895
MAD2L1BI	1.108468	1.001054	1.227408	0.047678
APOBEC2	1.017411	0.953188	1.085961	0.603864
KLHDC3	1.023279	0.940607	1.113218	0.592372
GNMT	1.043082	0.972667	1.118594	0.236883
DNAH8	0.833725	0.74535	0.932578	0.001468
TREM1	0.986612	0.947934	1.026867	0.50888
MEA1	1.195497	1.064042	1.343193	0.002661
CDKN1A	0.940996	0.896552	0.987644	0.013754
SOX4	0.975657	0.925848	1.028146	0.356649
GLO1	1.103931	1.006229	1.211121	0.036502
CPNE5	1.064002	0.981488	1.153453	0.131993
KCNK17	0.965936	0.930965	1.002221	0.06547
RREB1	0.879849	0.818111	0.946246	0.000564
SSR1	1.103626	1.007174	1.209315	0.034587
RIOK1	1.133551	1.029812	1.247741	0.010472
NRN1	1.204209	1.077154	1.346251	0.001089
SLC35B3	1.14908	1.051835	1.255316	0.002069
RPP40	1.075224	0.998389	1.157972	0.055195
ATXN1	0.983079	0.931596	1.037407	0.534053
NUP153	0.968138	0.907419	1.03292	0.327167
DEK	1.049641	0.95191	1.157407	0.331252
EEF1E1	1.223555	1.117491	1.339685	1.29E-05
RUNX2	0.971799	0.918606	1.028071	0.319233
LRRFIP1	0.957492	0.883699	1.037446	0.288437
RAB17	0.998432	0.898087	1.109988	0.976835
CXCL6	1.379511	1.209173	1.573845	1.71E-06
EREG	1.077327	1.048128	1.107339	1.08E-07
AHNAK	0.985432	0.943991	1.028692	0.503195
WNT1	1.035342	0.927631	1.155561	0.535473
SH3TC1	1.069242	1.009314	1.132729	0.022906
CNOT1	1.005911	0.931928	1.085769	0.879805
LRRC29	1.075519	0.964397	1.199444	0.190727
BBS2	1.15409	1.063105	1.252861	0.000625
MT1G	1.092556	1.044776	1.142522	0.000105
MT2A	1.013702	0.974125	1.054887	0.503
GOT2	1.093576	0.999637	1.196343	0.050932
DOK4	1.09881	1.027253	1.175352	0.006096
GPR18	1.005139	0.949609	1.063917	0.85968
CLYBL	1.21144	1.050966	1.396417	0.008155

TMTC4	1.011027	0.943086	1.083864	0.757327
RAP2A	0.99966	0.927941	1.076923	0.992862
SLC10A2	1.08639	0.975122	1.210355	0.132838
ABCC4	1.000403	0.948904	1.054698	0.98807
EFNB2	1.119635	0.980767	1.278167	0.094421
TM9SF2	1.017379	0.934053	1.108138	0.692702
IRF1	0.987574	0.932014	1.046445	0.672108
UPF3B	0.948444	0.859642	1.046419	0.291272
RNF113A	0.962275	0.852835	1.085759	0.532449
NDUFA1	1.024166	0.95607	1.097111	0.496362
BMP4	1.029653	0.87325	1.214068	0.72812
PTGER2	0.965164	0.917842	1.014925	0.166852
FAM193A	0.952739	0.878249	1.033547	0.243785
GRK4	1.159951	1.065747	1.262483	0.000596
HS3ST3B1	1.021878	0.956817	1.091363	0.519068
SLC25A35	1.299971	1.1849	1.426218	2.89E-08
MRPS7	1.188927	1.047491	1.34946	0.007407
GGA3	1.032701	0.93658	1.138686	0.518583
ARMC7	1.10428	1.003201	1.215543	0.042845
NUP85	1.129696	1.038985	1.228326	0.004297
SLC25A19	1.092998	1.013393	1.178856	0.021179
MIF4GD	1.039408	0.930952	1.160499	0.491806
NT5C	0.952786	0.86538	1.049021	0.32455
MSTO1	1.204176	1.089619	1.330778	0.00027
TTF1	1.10391	1.007778	1.209211	0.033449
GTF3C4	0.987218	0.912511	1.068042	0.74866
DDX31	1.041597	0.966836	1.122139	0.283508
PPP1R12C	1.086373	0.997083	1.183658	0.058329
MBOAT7	0.880214	0.818984	0.946021	0.000524
SRMS	1.024697	0.940626	1.116283	0.576456
OPRL1	1.099055	0.962125	1.255474	0.164154
SLC2A4RG	0.930222	0.85709	1.009593	0.083375
PPDPF	0.986992	0.940225	1.036085	0.597047
IL1B	1.035924	0.992882	1.080832	0.103092
CHCHD5	1.024968	0.929318	1.130463	0.621738
PAX8	1.085147	0.992384	1.186581	0.073087
INSIG2	1.094151	1.014153	1.180459	0.020193
POLR1B	1.115951	1.033968	1.204433	0.004832
CCDC93	1.008782	0.939237	1.083477	0.81039
PSD4	1.105385	1.033444	1.182334	0.003522
SLC25A23	1.263938	1.183045	1.350361	3.89E-12
PSPN	1.035927	0.940565	1.140957	0.473771
GTF2F1	0.903119	0.818042	0.997043	0.043526
ALKBH7	0.868152	0.79712	0.945515	0.001169
CLPP	1.046653	0.93494	1.171714	0.428487
TNFSF9	1.019415	0.973785	1.067183	0.410505
THOC2	0.978953	0.906974	1.056643	0.585114
MED1	0.980749	0.906874	1.060641	0.626603
RPL23	0.951306	0.874744	1.034569	0.243576
ATG4C	1.048406	0.975471	1.126794	0.198828
CD70	1.05347	1.009923	1.098896	0.01559
C3	1.125173	1.037389	1.220386	0.004432
SH2D3A	1.293042	1.189859	1.405172	1.39E-09
TRIP10	1.186547	1.111305	1.266884	3.10E-07
GPR108	1.30948	1.172976	1.461869	1.58E-06
TNFSF14	1.162392	1.081627	1.249186	4.21E-05
FOSB	0.980509	0.934877	1.028369	0.418227
OPA3	1.101869	1.011722	1.200047	0.025909

SNRPD2	1.030789	0.939222	1.131283	0.522887
RTN2	1.103784	1.014645	1.200755	0.021541
EML2	1.129243	1.036243	1.230589	0.005574
VASP	1.030228	0.948378	1.119142	0.480763
SYMPK	1.039593	0.954995	1.131685	0.369918
SDCBP2	1.012169	0.938385	1.091753	0.754131
PANK2	0.984798	0.911433	1.064069	0.698154
GNRH2	1.020476	0.949552	1.096697	0.581295
CD93	1.06579	1.024851	1.108366	0.001431
GZF1	1.001533	0.925479	1.083837	0.969676
NAPB	1.018475	0.947804	1.094414	0.617834
CENPB	1.001408	0.937403	1.069783	0.966702
PSMF1	1.142925	1.000474	1.305659	0.049189
DTD1	1.431838	1.320878	1.552119	2.73E-18
RBCK1	1.090452	0.976728	1.217418	0.123331
TMX4	0.957906	0.889506	1.031566	0.255222
STK35	0.971399	0.897336	1.051574	0.473283
SNRPB	0.992529	0.89811	1.096874	0.883103
NRSN2	0.949419	0.882736	1.021139	0.162426
RRBP1	0.991614	0.916173	1.073267	0.83477
BMP2	1.221957	1.163416	1.283442	1.22E-15
ZNF133	1.306975	1.208244	1.413773	2.39E-11
MKKS	1.10739	1.01557	1.207512	0.020899
BFSP1	1.309865	1.159767	1.47939	1.38E-05
DSTN	0.99921	0.923	1.081714	0.984432
SNRPB2	1.056378	0.952719	1.171316	0.29796
TBC1D20	0.997861	0.914805	1.088458	0.961484
ITPA	1.12535	1.033979	1.224795	0.006269
MCM8	1.23353	1.118864	1.359948	2.48E-05
FAM110A	1.031242	0.952642	1.116327	0.44693
SIRPD	1.118055	1.032406	1.21081	0.006065
MRPS26	1.177409	1.081762	1.281512	0.000158
S1PR4	0.832145	0.78192	0.885597	7.25E-09
NCLN	0.903562	0.833394	0.979639	0.013943
HNRNPR	1.008069	0.91586	1.109562	0.869568
ZNF436	0.971481	0.896077	1.053231	0.482764
MAX	0.970942	0.897827	1.050011	0.460367
ARMCX5	1.069744	0.971714	1.177665	0.169184
MMP24	1.033332	0.967723	1.103389	0.327244
NECAB3	0.90235	0.825436	0.986431	0.023789
ID1	1.039935	0.988479	1.09407	0.130433
RALY	0.906071	0.819155	1.00221	0.055231
DYNLRB1	1.031279	0.912211	1.165888	0.622685
EIF2S2	0.975556	0.876564	1.085727	0.650311
ERGIC3	0.962807	0.859685	1.078299	0.51199
ROMO1	1.063776	0.977322	1.157877	0.152844
CEP250	1.069268	0.969281	1.179568	0.181197
PLAGL2	0.948173	0.876085	1.026192	0.187136
AMOT	1.106232	1.015687	1.204849	0.020492
TMEM115	1.01124	0.933415	1.095554	0.784422
PSMB2	0.990665	0.880705	1.114354	0.875845
UROD	1.15153	1.074585	1.233985	6.37E-05
ST3GAL3	1.035646	0.969167	1.106685	0.300792
TMEM53	1.201372	1.095598	1.317358	9.56E-05
HECTD3	1.168752	1.06528	1.282274	0.000977
KLC1	0.970623	0.90129	1.045289	0.430368
XRCC3	0.95919	0.888394	1.035627	0.286831
TUBGCP3	0.976023	0.908044	1.049091	0.509974

MCF2L	1.055348	0.932877	1.193898	0.392023
PCID2	1.078364	0.987391	1.177719	0.09339
PROZ	1.411592	1.216906	1.637425	5.30E-06
LRFN3	1.188194	1.064715	1.325993	0.00207
CAPNS1	0.958004	0.882772	1.039647	0.303866
PDCD2L	1.323678	1.19418	1.467219	9.38E-08
RBM42	0.941419	0.845842	1.047797	0.269081
KIRREL2	0.961863	0.907915	1.019016	0.186724
UBA2	0.962606	0.879896	1.053091	0.405733
FFAR2	1.099176	1.052862	1.147528	1.67E-05
HCST	0.955664	0.900733	1.013945	0.133238
FFAR1	1.027675	0.965824	1.093486	0.388706
COX6B1	1.028798	0.945816	1.119061	0.508176
THRA	0.901238	0.831092	0.977305	0.011895
CCR7	1.00021	0.957484	1.044843	0.992473
NR1D1	1.103654	1.01039	1.205526	0.028566
FRMD8	1.033746	0.956589	1.117126	0.401702
PRDX5	1.109816	1.019817	1.207759	0.015747
BCL2L12	0.986405	0.890134	1.093088	0.793903
IRF3	1.187332	1.091352	1.291754	6.54E-05
PRMT1	0.972788	0.892268	1.060573	0.531404
RRAS	0.95797	0.892058	1.028752	0.237768
PRRG2	1.127448	1.012678	1.255224	0.028527
SCAF1	0.912941	0.840839	0.991226	0.030014
PRR12	0.924372	0.857722	0.996202	0.039435
TSKS	1.002785	0.93298	1.077814	0.939772
FLRT1	1.075487	0.965558	1.197931	0.185886
ASL	1.053558	0.954706	1.162646	0.299326
SBDS	0.899861	0.837658	0.966682	0.003887
CSN1S1	1.013289	0.923417	1.111907	0.78056
STAT5A	0.878443	0.820041	0.941004	0.000222
WNK4	1.227997	1.079405	1.397045	0.001802
BECN1	1.079666	0.965162	1.207755	0.180229
PRKCG	1.031676	0.959394	1.109404	0.400096
TRAP1	1.064338	0.985357	1.14965	0.112968
GLIS2	1.06298	0.977974	1.155376	0.15094
DNAJC8	1.087013	0.967393	1.221425	0.160721
AHDC1	0.975909	0.916243	1.039461	0.448695
IFI6	1.117947	1.069969	1.168076	6.30E-07
ZNF384	0.971365	0.86901	1.085776	0.609075
EMG1	1.217391	1.070246	1.384766	0.002764
UXT	0.92411	0.84159	1.01472	0.098176
CFP	1.021617	0.945538	1.103816	0.588065
ELK1	0.968125	0.878843	1.066477	0.511688
TIMM17B	1.212314	1.064521	1.380625	0.003701
KTN1	0.910974	0.839413	0.988635	0.025496
RHOJ	1.305394	1.048018	1.625978	0.017381
DLGAP5	1.085683	1.030347	1.143991	0.00207
HSPA2	0.994126	0.941584	1.049601	0.831618
ZBTB1	1.004477	0.931163	1.083563	0.908031
TRMT5	1.176102	1.062297	1.302099	0.001785
SGPP1	0.942886	0.898941	0.98898	0.015735
PLEKHG3	1.08636	1.031282	1.144379	0.001807
PZP	1.128945	1.054725	1.208388	0.000473
RHOT1	0.991829	0.908906	1.082317	0.853871
EVI2A	0.906524	0.840863	0.977314	0.010523
OMG	0.971782	0.916252	1.030678	0.34036
AIF1L	0.942172	0.901959	0.984177	0.007436

FAM78A	1.07982	1.010185	1.154255	0.023952
NUP214	0.952018	0.862061	1.051363	0.33158
CTAG2	0.990529	0.8261	1.187686	0.918163
AVPR2	1.108752	1.034999	1.187761	0.003288
SLC10A3	0.967014	0.878346	1.064634	0.494246
MAP2K2	1.043675	0.920904	1.182814	0.50318
HNRNPH2	1.034124	0.939747	1.13798	0.491947
ARMCX1	1.03839	0.975906	1.104874	0.234156
TIMM8A	1.116466	1.01257	1.231022	0.027062
ZC4H2	1.152481	1.051829	1.262765	0.002337
CANX	0.91457	0.847295	0.987188	0.021978
IPPK	1.077966	0.989236	1.174656	0.086709
ZNF484	1.104032	1.021995	1.192655	0.011997
OMD	1.081967	1.011176	1.157714	0.022496
FGD3	1.013223	0.941656	1.09023	0.725221
HIVEP3	1.286001	1.200614	1.377461	7.19E-13
PPCS	1.288479	1.15518	1.43716	5.39E-06
BCL11B	0.999103	0.952053	1.048478	0.970909
COX7C	1.024941	0.950276	1.105472	0.523248
TRAF2	1.054271	0.964856	1.151973	0.242494
ABHD8	1.076363	0.977803	1.184859	0.133138
ATP13A4	1.099231	0.975359	1.238834	0.120911
HELB	1.050875	0.989074	1.116538	0.108561
RAP1B	0.963263	0.896654	1.03482	0.305949
BEST3	1.063156	0.986281	1.146024	0.109768
RAB3IP	1.144108	1.051156	1.245281	0.001846
PTPRB	1.131873	1.053053	1.216591	0.000769
DYRK2	1.005846	0.937565	1.079099	0.870907
YEATS4	1.088334	0.978284	1.210764	0.119634
TAS2R3	1.020908	0.944888	1.103045	0.600198
TAS2R4	1.009554	0.920069	1.107743	0.840857
TAS2R5	0.995111	0.917759	1.078982	0.905507
LRRC61	1.01443	0.949834	1.083419	0.669538
IDUA	1.049492	0.97044	1.134983	0.226666
FGFRL1	1.037738	0.985439	1.092812	0.160314
TMEM175	1.130919	1.027467	1.244787	0.011952
PIN1	1.093246	0.964858	1.238718	0.161903
FBXL12	0.996487	0.897405	1.106509	0.947494
UBR4	0.972738	0.918848	1.029789	0.341846
HP1BP3	1.10265	0.994915	1.222051	0.062494
SIN3B	1.034162	0.952879	1.12238	0.42123
SLC35E1	0.929416	0.85952	1.004996	0.066501
EPS15L1	0.904155	0.827679	0.987696	0.025449
KLF2	0.9299	0.886416	0.975516	0.002935
F2RL3	1.084843	1.009063	1.166313	0.027513
GFER	1.12317	0.991106	1.272832	0.068761
SYNGR3	1.116374	1.001158	1.244851	0.047616
PKMYT1	1.070059	0.995694	1.149979	0.065395
WFIKKN1	0.995301	0.873853	1.133627	0.943441
WDR24	1.090716	0.999562	1.190183	0.051161
FBXL16	1.114157	0.991935	1.251438	0.068247
CHTF18	1.063729	0.990087	1.142848	0.09145
GNG13	1.442453	1.223255	1.700929	1.32E-05
MACF1	0.96399	0.91513	1.015458	0.166987
SMARCA4	1.038983	0.950892	1.135236	0.397547
TICAM1	0.910552	0.848993	0.976574	0.008699
IL17B	1.194177	1.049594	1.358677	0.007037
TUBA4A	1.017187	0.961308	1.076315	0.554433

VIL1	1.092914	0.948811	1.258903	0.218103
AAMP	1.041015	0.94304	1.149169	0.425422
PNKD	0.909669	0.840128	0.984965	0.01963
TNFRSF19	0.951401	0.879364	1.029338	0.214915
RNF6	0.968716	0.89065	1.053624	0.458426
ECHS1	1.136483	1.048783	1.231516	0.001794
AKAP9	0.918333	0.855963	0.985248	0.01759
GNG11	1.059382	0.996783	1.125912	0.063416
HIP1	1.123267	1.062599	1.1874	4.07E-05
PTPN12	1.087413	1.005213	1.176334	0.036653
POR	1.062464	0.971456	1.161998	0.184797
FGL2	1.039914	0.998393	1.083161	0.059753
STYXL1	1.000062	0.921819	1.084945	0.998818
STEAP4	1.200275	1.102542	1.306671	2.52E-05
GNAI1	0.902749	0.840868	0.969183	0.004744
PEX1	1.081658	0.989127	1.182846	0.085369
CASD1	0.996033	0.929334	1.067519	0.910496
ZNF780B	1.040888	0.972979	1.113537	0.244353
LRFN1	0.948689	0.88158	1.020908	0.159375
ZFP36	0.955088	0.902362	1.010895	0.112745
SRD5A3	1.130249	1.01995	1.252476	0.019439
SPINK2	1.091404	1.06422	1.119283	1.07E-11
PAICS	1.039857	0.964655	1.120922	0.307519
KDR	1.137076	1.021674	1.265512	0.018639
PPAT	1.100906	1.012297	1.197271	0.02474
TUBGCP6	1.009325	0.941937	1.081534	0.792343
ADM2	0.935458	0.873147	1.002215	0.057822
DGCR6L	1.125884	1.027953	1.233144	0.010656
DGCR8	1.069622	0.983375	1.163434	0.116621
ASPHD2	1.068087	0.980633	1.16334	0.130722
VPREB3	1.057483	1.002219	1.115794	0.04126
SDF2L1	1.115537	1.04118	1.195204	0.001893
YWHAH	0.929837	0.847	1.020775	0.126498
RFPL2	1.17225	1.019536	1.347838	0.025639
GNAZ	1.237923	1.151383	1.330968	7.82E-09
MGAT3	0.968448	0.916249	1.023621	0.256747
ADORA2A	1.044576	0.971609	1.123022	0.237842
ATF4	0.877965	0.805702	0.956709	0.00298
A4GALT	1.030542	0.973594	1.090821	0.299602
CDC42EP1	1.089704	1.034294	1.148083	0.001254
APOL3	1.017726	0.957132	1.082157	0.574786
TPST2	1.213554	1.121861	1.312741	1.37E-06
BAIAP2L2	1.246744	1.14297	1.359939	6.57E-07
MPST	0.925988	0.84251	1.017738	0.110665
GALR3	0.915603	0.807268	1.038477	0.16996
TST	1.070181	0.997278	1.148413	0.059532
IGLL1	1.001988	0.974533	1.030217	0.888566
APOL2	1.010324	0.94586	1.079181	0.760123
RAC2	1.11757	1.036303	1.205209	0.003905
LIF	0.949942	0.884696	1.019999	0.157201
APOBEC3/	1.032807	0.992316	1.07495	0.113667
APOBEC3F	1.054104	0.97477	1.139895	0.186879
RIBC2	1.143915	1.052529	1.243235	0.00155
KRT17	0.881586	0.845809	0.918877	2.49E-09
DOCK4	0.873734	0.817641	0.933674	6.69E-05
POT1	1.022607	0.934358	1.119191	0.627329
ATP6V1F	0.991491	0.91263	1.077166	0.839846
LSM8	1.116148	0.998413	1.247766	0.053356

PRKRIP1	0.920678	0.837481	1.012139	0.087216
VGF	1.020412	0.919014	1.132997	0.705132
PODXL	1.026898	0.956755	1.102183	0.462161
MKLN1	0.980469	0.921411	1.043313	0.53377
DNAJB9	0.959797	0.890547	1.034432	0.282842
FLNC	0.821477	0.740578	0.911215	0.000201
LRRC4	0.984086	0.930955	1.040249	0.571062
CALU	1.020192	0.941284	1.105714	0.626463
CCDC136	1.230456	1.159442	1.30582	8.06E-12
SMO	1.043801	0.97371	1.118938	0.226753
IRF5	1.110194	1.029553	1.197152	0.006589
LRRC17	1.13414	1.014134	1.268347	0.02739
KLHDC10	0.934688	0.862562	1.012846	0.099264
NDUFA5	1.03571	0.932027	1.150927	0.514421
OPN1SW	1.060117	0.954851	1.176988	0.273903
MRPS12	1.154095	1.058688	1.258099	0.001132
MYO1B	1.170826	1.087799	1.260189	2.64E-05
MTX2	1.025347	0.936531	1.122585	0.588178
GAD1	1.117565	1.06399	1.173839	9.23E-06
OSGEPL1	1.117224	1.026271	1.216237	0.010512
ORMDL1	1.121333	1.020787	1.231784	0.016885
HAT1	0.979061	0.884091	1.084232	0.684381
HERC2	1.021918	0.961207	1.086463	0.487801
SNRPN	1.042172	0.982736	1.105203	0.167985
PSMG2	1.015879	0.904153	1.141411	0.790999
TWSG1	0.92625	0.866854	0.989716	0.023472
ARHGAP2:	1.177123	1.111094	1.247077	3.09E-08
WDFY4	1.027481	0.974657	1.083168	0.314066
EIF2AK4	1.03187	0.941016	1.131494	0.504682
MYO5C	0.930662	0.879168	0.985173	0.013348
CGNL1	1.24277	1.147723	1.345688	8.60E-08
TMOD2	1.034253	0.968379	1.104607	0.315849
TTBK2	0.943799	0.872252	1.021214	0.150417
ELL3	0.862457	0.766771	0.970082	0.013655
INO80	0.914635	0.853505	0.980143	0.011463
DLL4	0.934815	0.836167	1.045102	0.236151
ALDH1A2	1.371616	1.208763	1.556409	9.58E-07
IVD	1.135425	1.053697	1.223492	0.000861
DUT	1.150323	1.0563	1.252714	0.001287
CHAC1	1.043906	0.987308	1.103748	0.13083
CLN6	0.987439	0.906966	1.075052	0.770711
ARPP19	0.952241	0.879324	1.031205	0.2286
VPS13C	0.989228	0.939079	1.042056	0.683267
LOXL1	1.067983	0.996798	1.144251	0.061647
ANAPC13	1.117604	1.013973	1.231826	0.025127
MBD4	1.05574	0.969497	1.149655	0.212212
COPB1	0.927949	0.861795	0.999182	0.047518
PSMA1	0.932722	0.843024	1.031965	0.176998
SUMF2	0.9403	0.861189	1.026679	0.169819
PALLD	1.122485	1.050098	1.199862	0.000681
SPCS3	0.990135	0.91166	1.075365	0.813967
MYOD1	0.954865	0.686453	1.328231	0.783871
SERGEF	1.129705	1.030772	1.238133	0.009104
TPH1	1.147268	0.974605	1.350519	0.098766
E2F8	1.18969	1.117006	1.267103	6.66E-08
DCTD	0.946708	0.863154	1.038349	0.245358
SOX15	0.842289	0.778541	0.911257	1.92E-05
RPAIN	0.968203	0.895339	1.046997	0.418239

USP6	1.064485	0.967186	1.171571	0.201334
SHBG	0.894285	0.791201	1.010799	0.073766
PLD2	1.061715	0.994522	1.133448	0.072606
CD68	1.045562	0.896917	1.218842	0.569039
TXNDC17	1.284414	1.144044	1.442006	2.25E-05
ATP1B2	1.064611	1.003018	1.129986	0.039488
FXR2	0.924421	0.850957	1.004227	0.062868
KIF1C	1.115023	1.040172	1.195261	0.002134
MPDU1	0.945472	0.857577	1.042376	0.260037
PHF20L1	1.008814	0.930562	1.093647	0.831309
LRRC6	0.972383	0.885646	1.067614	0.556873
CCNT1	0.954028	0.895817	1.01602	0.142875
PUS7L	1.110799	1.025877	1.202751	0.00961
KRI1	1.269703	1.156124	1.39444	5.91E-07
ILF3	0.952881	0.884314	1.026764	0.205241
SLC44A2	0.94963	0.888385	1.015097	0.128649
CDKN2D	1.069275	0.998864	1.144649	0.053946
MTUS1	0.997821	0.864845	1.151244	0.976158
SIGLEC9	0.996669	0.949049	1.046678	0.89374
NGDN	0.984494	0.890987	1.087814	0.758903
RIPK3	1.218025	1.14278	1.298226	1.34E-09
ADCY4	1.227741	1.151463	1.309072	3.62E-10
RAB2B	0.965942	0.888153	1.050543	0.418559
BCL2L2	0.961535	0.893668	1.034556	0.293581
PARP2	0.993833	0.899051	1.098609	0.903725
HEATR5A	0.99547	0.916217	1.081578	0.914573
SNX6	0.952437	0.863784	1.05019	0.328284
EAPP	1.041331	0.932876	1.162396	0.470458
NRL	1.138188	0.994818	1.30222	0.05952
RNASE1	1.070325	1.024486	1.118214	0.002341
NEDD8	1.250795	1.093022	1.431342	0.001142
DAD1	1.047069	0.975112	1.124337	0.205452
TEP1	1.0316	0.972376	1.094431	0.302381
EPB41L4A	1.1628	1.036218	1.304847	0.010318
REEP5	0.89093	0.837437	0.94784	0.000257
ITFG1	1.120122	1.009656	1.242675	0.032245
QRICH2	1.002129	0.942079	1.066006	0.94623
FOXJ1	1.238672	1.102776	1.391314	0.000306
SEC14L1	0.994572	0.922858	1.07186	0.88666
RHBDF2	1.092127	1.02075	1.168495	0.010603
AANAT	1.09649	1.00014	1.202122	0.049653
ARHGEF6	0.912845	0.8623	0.966354	0.001704
MAP7D3	1.012368	0.926538	1.106149	0.785668
ASH2L	1.111854	1.014821	1.218166	0.022862
CHRNA10	1.015726	0.913792	1.129031	0.772443
CDKN1C	0.951468	0.902913	1.002636	0.062677
RPS4Y1	1.010277	0.989956	1.031015	0.324022
KLF16	0.944637	0.875766	1.018923	0.14032
DOHH	0.973824	0.893981	1.060799	0.543383
SHC2	0.850866	0.702256	1.030925	0.09915
LBP	0.991409	0.890494	1.10376	0.874827
CBFA2T3	1.047631	0.985923	1.113201	0.133031
GAMT	0.987559	0.919178	1.061028	0.7324
PHF10	1.025385	0.941303	1.116978	0.56579
PRRG3	1.224708	1.08492	1.382508	0.001045
STARD8	1.069563	1.01604	1.125907	0.010245
GDPD2	1.061125	0.938994	1.199142	0.341605
SAT1	0.916868	0.861055	0.976298	0.006758

GNL3L	1.078342	0.996514	1.16689	0.061037
SH3BP4	1.081847	1.035758	1.129987	0.000398
MOSPD2	0.992154	0.923497	1.065915	0.829539
DOCK6	1.004063	0.942775	1.069334	0.899596
ECSIT	1.208814	1.071208	1.364096	0.002101
LDLR	1.078022	1.024846	1.133958	0.003604
ELOF1	1.23683	1.108144	1.38046	0.00015
TSPAN16	0.996602	0.943906	1.052241	0.902272
PRKCSH	0.859504	0.797553	0.926266	7.29E-05
CDC16	1.105988	1.02162	1.197324	0.012836
APOE	0.986093	0.927576	1.048301	0.653661
TOMM40	1.046316	0.960353	1.139975	0.300625
APOC1	1.062221	1.000332	1.127938	0.048746
GADD45G	0.979574	0.93737	1.023677	0.358359
XPO7	1.039576	0.9749	1.108542	0.236289
FAM98C	1.34255	1.200473	1.501442	2.45E-07
SAFB2	1.009487	0.925982	1.100524	0.830278
RPL36	0.947674	0.888996	1.010225	0.099348
ATP8B3	1.190279	1.107735	1.278975	2.03E-06
GTPBP3	1.233812	1.124466	1.353792	9.10E-06
PLVAP	1.083324	1.024059	1.146018	0.0053
BST2	1.222042	1.153111	1.295093	1.29E-11
SLC27A1	0.972615	0.90051	1.050495	0.479866
NSUN5	1.187983	1.082005	1.304341	0.000302
USHBP1	1.101107	0.948078	1.278837	0.207101
DDA1	1.005871	0.911941	1.109476	0.906833
MRPL34	1.228299	1.109475	1.35985	7.46E-05
PGLS	0.965876	0.869332	1.073142	0.518166
LSM7	1.031715	0.938833	1.133785	0.516557
TULP4	0.98106	0.923365	1.042359	0.536334
SNX9	0.974911	0.930009	1.021981	0.290895
RTN4IP1	1.124809	1.020255	1.240079	0.018137
QRSL1	1.037121	0.942937	1.140712	0.453038
RSPH3	1.069468	0.969907	1.179248	0.177947
MLLT1	0.946542	0.879544	1.018642	0.142424
FUT5	1.063419	0.971609	1.163906	0.181957
ACTN4	0.965092	0.889369	1.047262	0.394051
NDUFA10	1.002196	0.913735	1.099222	0.96289
ARPC1B	1.094262	0.99579	1.202471	0.061168
ZSWIM6	0.943767	0.889007	1.0019	0.057731
FCHO1	0.981723	0.902844	1.067493	0.666002
MAP1S	0.913521	0.843932	0.988847	0.025261
KLHDC7B	0.904808	0.851749	0.961172	0.001177
PXDN	0.949456	0.912738	0.987652	0.009955
SSBP4	1.00661	0.920087	1.101269	0.885754
GDF15	1.027537	0.971369	1.086953	0.343575
PGPEP1	1.031737	0.962746	1.105673	0.376266
LSM4	1.281884	1.162338	1.413724	6.64E-07
JUND	0.908162	0.836998	0.985376	0.020678
HRC	1.119092	1.042486	1.201328	0.001871
TRPM4	1.102149	1.066914	1.138548	4.44E-09
SULT4A1	1.200735	1.118653	1.28884	4.11E-07
ZNF557	1.178636	1.088008	1.276812	5.67E-05
CRB3	1.182559	0.966327	1.447176	0.103629
CAMSAP1	0.969208	0.895915	1.048497	0.435644
UBAC1	1.111043	1.023544	1.206022	0.01187
ZBTB46	0.976709	0.924566	1.031792	0.39984
SAMD10	0.97729	0.900972	1.060072	0.579752

LSP1	1.124394	1.067103	1.184761	1.11E-05
TNNT3	1.14254	1.073415	1.216117	2.85E-05
TNNI2	1.118546	1.058995	1.181446	5.98E-05
H19	0.95031	0.857069	1.053696	0.333399
COL5A1	1.186445	1.125203	1.251021	2.57E-10
ATXN10	1.086115	0.995088	1.18547	0.064355
TUBGCP2	0.959015	0.871993	1.054721	0.388548
CYP2E1	0.862067	0.768662	0.966823	0.011194
PNPLA7	1.033867	0.954591	1.119728	0.413215
HBZ	0.966617	0.911689	1.024855	0.255342
PAK4	1.058914	0.952587	1.177108	0.289021
MNX1	1.003323	0.913431	1.102061	0.944777
ZNF337	0.966478	0.881937	1.059124	0.465359
TAF4	1.003947	0.911881	1.105309	0.93602
LAMA5	0.854148	0.80322	0.908306	5.01E-07
OSBPL2	0.995728	0.923551	1.073546	0.911212
ADRM1	0.859735	0.784812	0.94181	0.001159
ASS1	0.982795	0.933363	1.034845	0.509827
EXOSC2	1.089008	1.006022	1.17884	0.034995
POMT1	1.102247	1.01618	1.195603	0.01893
UCK1	1.141284	1.035556	1.257806	0.007714
FIBCD1	0.699193	0.597378	0.818361	8.34E-06
CHMP2A	0.968829	0.853873	1.099261	0.623139
UBE2M	0.84104	0.754555	0.937438	0.001767
TRIM28	0.948961	0.88047	1.02278	0.170489
YIPF2	0.978729	0.892941	1.07276	0.645975
ATG4D	1.014263	0.943962	1.0898	0.699186
EIF2S3	0.90996	0.83738	0.988832	0.026095
TMEM160	1.005295	0.925592	1.091861	0.900281
ZC3H4	1.038662	0.968086	1.114383	0.290712
NPAS1	1.113	1.00947	1.227148	0.031621
GMFG	1.005885	0.91263	1.10867	0.905901
MAP3K10	1.193158	1.096434	1.298413	4.23E-05
LRRC47	1.092848	0.999264	1.195196	0.051914
SESN2	1.022515	0.962915	1.085803	0.467443
SMPDL3B	1.157009	1.076024	1.244089	8.18E-05
MED18	1.131048	1.035779	1.235081	0.006088
CLIP1	0.979881	0.899668	1.067247	0.640926
CCDC62	0.995434	0.903474	1.096755	0.926279
HIP1R	0.971047	0.923397	1.021156	0.252433
ZNF317	0.962781	0.884401	1.048109	0.381332
PPAN	1.020318	0.932282	1.116668	0.662186
EIF3G	0.935465	0.832944	1.050604	0.259984
ANGPTL6	0.842104	0.782657	0.906066	4.21E-06
DNMT1	1.132954	1.046647	1.226378	0.002017
ZNF426	1.029818	0.950418	1.11585	0.472921
SLC6A8	0.989057	0.944471	1.035749	0.64013
DKC1	1.110669	1.013606	1.217025	0.024473
PLXNA3	0.954898	0.896483	1.01712	0.151882
MPP1	1.004811	0.931364	1.084051	0.901368
ZNF331	0.960604	0.905782	1.018744	0.180056
ZNF236	1.016276	0.938965	1.099951	0.68921
LRP3	0.950521	0.908439	0.994552	0.028062
NOL11	1.071055	0.984177	1.165604	0.111739
UBE4B	0.983319	0.90153	1.072529	0.704204
CASZ1	1.034401	0.966922	1.106589	0.325775
PKDREJ	1.129313	0.997872	1.278067	0.054077
HABP4	1.07939	0.984746	1.183132	0.102753

SLC35D2	0.826827	0.74352	0.919468	0.000449
PRRG1	1.329872	1.200765	1.472861	4.47E-08
UBA1	0.989036	0.902461	1.083916	0.813529
PPIL4	0.983427	0.903776	1.070098	0.698168
ULBP2	1.059875	0.977905	1.148717	0.156796
AKAP12	0.993589	0.914952	1.078985	0.878487
SYNE1	1.114145	1.052388	1.179525	0.000203
ULBP3	1.374044	1.245516	1.515834	2.27E-10
LATS1	1.031565	0.948997	1.121318	0.465326
EPS8L1	1.151724	1.064025	1.246652	0.000473
LILRB2	1.092714	1.047501	1.139879	3.92E-05
TTLL9	0.95659	0.819047	1.11723	0.575242
RBM39	0.957733	0.890479	1.030067	0.245021
ZNF341	0.995631	0.892342	1.110876	0.937548
GGT7	1.123375	1.048132	1.204021	0.001006
ACSS2	1.058539	0.975223	1.148973	0.173792
EDA2R	1.130162	1.059374	1.20568	0.000209
ARHGEF9	1.105495	1.006004	1.214825	0.037126
HIGD1B	1.020861	0.957396	1.088533	0.528387
ATP6V1E1	0.99231	0.881193	1.117439	0.898625
ZNF227	1.06801	0.977066	1.167419	0.147334
ZNF428	0.918458	0.841853	1.002033	0.055588
ZNF141	0.984987	0.921086	1.053321	0.658483
CCL25	1.254882	1.140815	1.380353	3.02E-06
COX4I1	0.955226	0.869489	1.049418	0.339742
GINS2	1.221046	1.121376	1.329575	4.29E-06
CHMP1A	1.002075	0.91141	1.101758	0.965833
SH3BGRL	1.034838	0.950822	1.126279	0.427962
COX7B	0.926185	0.82901	1.034751	0.175125
F12	1.218704	1.131817	1.312261	1.60E-07
PRR7	1.071427	0.997144	1.151245	0.059844
NFATC1	0.967204	0.893411	1.047092	0.410212
CAP1	1.072166	0.979545	1.173546	0.130631
PPT1	1.111635	1.037719	1.190817	0.002573
RAB11FIP4	1.091475	1.021368	1.166393	0.009761
ABCB7	0.958979	0.87887	1.04639	0.346647
TRAF3	0.970926	0.903662	1.043197	0.420553
HAUS8	1.240505	1.112898	1.382744	9.97E-05
MRPS25	1.073294	0.989491	1.164195	0.088143
SH3BP5	1.152821	1.086831	1.222818	2.26E-06
HACL1	0.997787	0.90908	1.09515	0.962802
TBC1D5	1.01491	0.949029	1.085365	0.665593
CAPN7	1.057396	0.986795	1.133047	0.113436
RFTN1	1.086657	1.015218	1.163122	0.016608
SLC6A6	0.972385	0.909907	1.039152	0.408526
KCNC3	1.140576	1.088982	1.194614	2.56E-08
NAPSA	1.170291	1.067222	1.283314	0.000829
NAPSB	1.066569	1.030804	1.103575	0.000213
NR1H2	0.963159	0.868116	1.068608	0.478861
LRRC4B	1.016044	0.909147	1.135511	0.778991
PDLIM4	0.963413	0.910596	1.019294	0.195092
KIF3A	1.252703	1.137798	1.379211	4.43E-06
MGAT1	0.998293	0.918341	1.085206	0.968006
TUBG1	1.189022	1.108179	1.275763	1.44E-06
PSME3	1.116592	1.000701	1.245905	0.048553
RPL27	0.947904	0.893093	1.006078	0.078313
PSMC3IP	1.047985	0.958718	1.145564	0.302144
AOC3	0.987946	0.906569	1.076627	0.782153

ACLY	1.11838	1.03662	1.206588	0.003871
VPS25	1.241156	1.130644	1.362469	5.61E-06
RAMP2	1.051591	0.870111	1.270924	0.602744
AOC2	1.018619	0.94713	1.095504	0.619272
NDUFA2	1.17772	1.081382	1.28264	0.000172
ANKHD1	1.033505	0.94974	1.124657	0.444747
DIAPH1	0.861443	0.804462	0.92246	1.94E-05
NDFIP1	1.15677	1.080751	1.238135	2.68E-05
UBE2D2	0.917167	0.823132	1.021945	0.117198
EXOC4	1.016782	0.949292	1.08907	0.634839
ACAP3	1.020103	0.949643	1.095792	0.585719
PPFIA1	1.035289	0.953619	1.123954	0.408117
TMEM204	1.036102	0.964936	1.112517	0.328641
KREMEN2	1.050752	0.947141	1.165698	0.349962
THOC6	1.030361	0.949296	1.118348	0.474374
TRAF7	0.853371	0.786216	0.926261	0.00015
BARX1	1.366111	1.163663	1.603778	0.000138
NINJ1	1.018788	0.965115	1.075445	0.500262
CA6	1.056327	0.962907	1.15881	0.246096
NPHP4	1.004499	0.924721	1.09116	0.915326
MAP1B	1.180175	1.075011	1.295626	0.000503
IL13RA1	0.992995	0.947714	1.040439	0.767831
WDR44	0.93314	0.858518	1.014249	0.103682
CKMT2	1.057472	0.975845	1.145928	0.172759
ZCCHC9	1.118471	0.995938	1.25608	0.058595
TOP2A	1.116872	1.063455	1.172973	9.85E-06
STARD3	1.016854	0.920104	1.123777	0.743187
RARA	0.941416	0.876795	1.010799	0.09613
PPP1R1B	0.923716	0.803629	1.061748	0.264109
KHDRBS3	1.001008	0.910405	1.100627	0.983397
CHD1L	1.002077	0.927968	1.082105	0.957788
PEX11B	1.033755	0.949469	1.125524	0.444251
FMO5	1.136193	1.045865	1.234321	0.002519
PIAS3	1.27901	1.144182	1.429727	1.49E-05
PRKAB2	1.051131	0.982176	1.124927	0.149737
PDHA1	1.030411	0.93435	1.136347	0.548516
RAI2	1.163337	1.062037	1.2743	0.001135
MCCC2	1.102933	1.019838	1.192798	0.014226
ZNF304	1.01657	0.945166	1.093369	0.658283
ZSCAN5A	1.045445	0.939957	1.162772	0.412819
ZNF132	1.033148	0.95765	1.114599	0.399626
CHSY1	0.938698	0.879966	1.00135	0.054979
SNRPA1	0.980006	0.906392	1.0596	0.612212
LLGL1	1.009329	0.931002	1.094246	0.821741
THAP1	1.080203	0.963537	1.210994	0.18584
ACTR10	0.98636	0.886253	1.097774	0.801404
ABHD12B	0.897237	0.83656	0.962316	0.002404
GCH1	1.113334	1.048166	1.182554	0.000486
LGALS3	1.028226	0.980828	1.077914	0.247686
PODNL1	1.371266	1.158573	1.623007	0.000241
DNAJB1	1.004266	0.937212	1.076118	0.903899
ZSWIM4	0.939587	0.875746	1.008081	0.082605
FBXW9	0.96958	0.886641	1.060277	0.498349
RFX1	0.964267	0.884164	1.051627	0.410892
ZNF20	1.10664	0.990943	1.235846	0.072101
DCAF15	0.989431	0.904597	1.082222	0.816301
CC2D1A	1.214372	1.107607	1.331429	3.52E-05
TRIM21	1.154478	1.060664	1.256589	0.000894

SPATA6	1.183234	1.10667	1.265096	8.24E-07
LRRC41	1.013524	0.925835	1.10952	0.771083
CCT6B	1.066829	0.969389	1.174063	0.185576
DHX30	1.014387	0.925611	1.111677	0.759844
RAF1	0.963864	0.892496	1.040938	0.348384
PPARG	1.036032	0.978021	1.097485	0.228577
NUP210	1.137299	1.074387	1.203895	9.37E-06
FCRLA	1.037367	0.972562	1.106489	0.265001
HSD17B7	1.153415	1.052803	1.263642	0.002177
ENOSF1	1.063779	0.991636	1.14117	0.084426
EMILIN2	0.885584	0.841728	0.931724	2.75E-06
ARFIP2	1.070667	0.961294	1.192483	0.21425
TRIM5	0.97909	0.908835	1.054776	0.578054
CNGA4	1.037889	0.911535	1.181759	0.574466
TRIM22	0.990038	0.931051	1.052763	0.749402
RRP8	1.007729	0.937943	1.082708	0.833451
EFR3A	0.943064	0.887271	1.002365	0.05956
PTCD3	1.028845	0.95753	1.105472	0.437823
IMMT	1.006699	0.910896	1.112577	0.895895
MRPL35	1.10159	0.996866	1.217314	0.057646
ILKAP	0.990121	0.92202	1.063252	0.784795
PER2	1.012494	0.93453	1.096961	0.761349
RAMP1	0.924537	0.884315	0.966589	0.000545
SCLY	1.230378	1.073561	1.410101	0.002879
PTPRE	0.985404	0.932918	1.040843	0.598534
RAN	1.234876	1.106588	1.378037	0.000163
PRKAA1	1.093878	1.003915	1.191902	0.040443
CARD6	0.966332	0.902662	1.034492	0.324702
INPP5K	1.021096	0.928715	1.122667	0.666118
MYBBP1A	1.065311	0.99375	1.142025	0.074547
RPA1	1.139381	1.054754	1.230798	0.000921
SERPINF1	0.967956	0.911374	1.028052	0.289257
UBE2G1	0.995718	0.909819	1.089727	0.925724
EEFSEC	1.098245	1.010541	1.19356	0.027322
TBC1D14	0.970793	0.904794	1.041607	0.409277
TMEM128	1.123624	1.017626	1.240662	0.021134
COQ3	1.236752	1.125816	1.358619	9.36E-06
SEC61G	0.952404	0.874045	1.037787	0.2656
LANCL2	0.984013	0.906474	1.068185	0.700348
FIGNL1	1.108782	1.030171	1.193393	0.005919
GRSF1	0.923229	0.838934	1.015994	0.102019
ANKRD17	0.988837	0.923746	1.058515	0.74661
UTP3	1.222789	1.112863	1.343572	2.85E-05
ITGB4	0.802677	0.754874	0.853508	2.28E-12
WBP2	0.973713	0.869575	1.090322	0.644378
UNK	0.987767	0.897409	1.087223	0.801457
TRIM47	0.795541	0.728328	0.868956	3.80E-07
ZRANB2	1.078448	1.003668	1.1588	0.039416
EIF5A	1.059027	0.943629	1.188536	0.329922
KDM6B	0.924886	0.859908	0.994773	0.035643
CLEC10A	0.961454	0.922471	1.002083	0.062687
GUCY2D	1.158025	1.057643	1.267935	0.001517
GPS2	1.03312	0.894028	1.193852	0.658747
XAF1	1.076505	1.014741	1.142028	0.01447
DLG4	0.982632	0.912725	1.057893	0.641703
VPS13B	1.025881	0.967592	1.08768	0.391925
MATN2	0.971705	0.894066	1.056086	0.499312
REEP2	0.938702	0.836118	1.053871	0.284018

PCBD2	1.094683	0.999365	1.199092	0.05162
SDF2	1.026212	0.915142	1.150762	0.657976
FLOT2	1.044901	0.971778	1.123526	0.235397
ERAL1	1.186984	1.070909	1.315641	0.001096
PRMT7	1.139897	1.051583	1.235628	0.001461
NIP7	1.02719	0.933942	1.129749	0.580605
TERF2	0.954922	0.868863	1.049504	0.338448
VPS4A	0.983436	0.891636	1.084686	0.738322
HSPA12B	1.020718	0.944979	1.102527	0.602161
BTBD3	1.100278	1.018589	1.188518	0.015186
PCNA	1.139024	1.062846	1.220661	0.000228
NXT1	1.048894	0.955788	1.151069	0.314169
POLR3F	1.020409	0.930884	1.118544	0.666295
RIN2	1.146008	1.076619	1.219868	1.90E-05
PTPRA	1.035731	0.933887	1.148682	0.506194
DAP3	1.061939	0.927785	1.215491	0.383122
ARHGEF11	1.070818	1.024296	1.119452	0.002534
FCRL2	1.002551	0.93334	1.076895	0.944338
SYT11	0.95779	0.894056	1.026067	0.219627
IGHMBP2	0.970338	0.897899	1.048621	0.44687
ACY3	0.958278	0.919918	0.998237	0.04089
MMACHC	1.152233	1.059848	1.252672	0.00089
DPH2	1.159886	1.079085	1.246737	5.67E-05
TOE1	1.213035	1.101743	1.335569	8.38E-05
NASP	1.04374	0.950814	1.145748	0.368206
MUTYH	1.2265	1.081822	1.390527	0.001432
CTNNBL1	0.908199	0.83488	0.987958	0.024957
LPIN3	1.224668	1.136224	1.319998	1.16E-07
ZSWIM3	1.09416	1.008776	1.186771	0.029951
RBM38	1.00085	0.948679	1.055891	0.97518
VSTM2L	0.94306	0.828879	1.07297	0.373282
SERINC3	0.971516	0.900531	1.048098	0.455386
PPP1R3D	1.130445	1.042634	1.22565	0.002959
AP3B1	0.982422	0.914258	1.055669	0.628839
ZBED3	0.948769	0.883987	1.0183	0.145002
ANGPTL3	1.020821	0.924529	1.127142	0.683529
FBXO44	1.313882	1.220352	1.41458	4.31E-13
CASP9	0.972311	0.886176	1.066819	0.552983
DCTN4	0.925849	0.865135	0.990825	0.025992
PDE6A	0.9699	0.853632	1.102005	0.638999
MTUS2	0.96272	0.875017	1.059213	0.435637
ZMYM5	0.90753	0.832136	0.989756	0.028333
USPL1	0.973297	0.900096	1.052451	0.497471
XPO4	0.97608	0.90808	1.049171	0.511094
POMP	1.20172	1.092641	1.321687	0.000154
CDK8	0.910379	0.83846	0.988466	0.025334
ALOX5AP	1.065742	1.019826	1.113724	0.004601
WASF3	1.274101	1.145694	1.416899	7.84E-06
GPR12	0.663596	0.596143	0.73868	6.47E-14
MYH10	1.015776	0.958515	1.076459	0.596977
PEMT	1.049959	0.963631	1.144021	0.265424
SCO1	1.015132	0.93174	1.105987	0.731304
MPRIP	0.961678	0.895481	1.03277	0.282891
CHI3L1	0.990868	0.955677	1.027354	0.619016
MYBPH	0.964831	0.899276	1.035165	0.31863
PIK3C2B	1.072194	1.012138	1.135814	0.017778
DSTYK	0.98396	0.914792	1.058358	0.663697
CHIT1	1.135476	1.071877	1.202849	1.56E-05

SLC41A1	1.125008	1.058056	1.196197	0.000168
LGR6	1.090132	0.997608	1.191237	0.056517
TMCC2	0.9968	0.960404	1.034576	0.865897
CCNA1	0.977935	0.946512	1.010402	0.180579
COG6	1.074376	0.991237	1.164489	0.080848
RXFP2	0.934821	0.888462	0.9836	0.0094
EPSTI1	1.063049	1.014262	1.114183	0.010749
RFXAP	1.17863	1.073361	1.294224	0.000575
TPT1	0.846936	0.778344	0.921573	0.000116
RFC3	1.05644	0.979925	1.13893	0.152339
STARD13	0.998207	0.908025	1.097346	0.970373
MORC4	1.418299	1.300978	1.546201	2.15E-15
BEX2	1.105731	1.057685	1.155959	9.24E-06
TCEAL4	1.06689	1.002823	1.13505	0.040443
BEX1	0.989096	0.957551	1.02168	0.507352
FAM104A	1.155353	1.055669	1.264449	0.001708
SLC39A11	1.103349	1.0439	1.166185	0.000501
EPHB2	0.985466	0.878339	1.105659	0.803093
SRRM1	0.97063	0.876845	1.074446	0.565308
BTBD2	1.039256	0.956827	1.128787	0.361112
PRAM1	0.929197	0.878715	0.982581	0.00998
ZNF414	0.914098	0.822462	1.015943	0.095616
PDE6B	1.064391	0.97539	1.161514	0.161313
HSPBP1	1.068739	0.990011	1.153727	0.088601
CSNK1G2	0.958615	0.878205	1.046387	0.344373
CNDP2	1.013685	0.939471	1.093761	0.726052
MACROD1	0.996913	0.920354	1.079841	0.939553
WDR74	0.932099	0.865647	1.003652	0.062412
LGALS12	1.004093	0.961276	1.048817	0.854246
RTN3	0.966865	0.887284	1.053584	0.441954
MYH11	0.90534	0.843014	0.972275	0.006284
MED10	1.265401	1.14368	1.400077	5.08E-06
PDZD2	1.161904	1.071853	1.259522	0.000267
MORC2	1.097875	1.009282	1.194246	0.029616
MYO18B	0.982557	0.948145	1.018219	0.333349
SLC2A11	1.249961	1.116872	1.398908	0.000103
C1QTNF6	1.243931	1.129147	1.370383	9.92E-06
FAM83F	1.010695	0.920112	1.110194	0.824276
SEC14L4	1.030802	0.972502	1.092598	0.307121
GIMAP6	1.052772	0.999232	1.109182	0.053471
GIMAP4	0.979847	0.929385	1.033047	0.45042
ADCK2	1.159516	1.060112	1.26824	0.00121
MKRN1	0.926488	0.858648	0.999687	0.049064
AGAP3	0.929883	0.876273	0.986773	0.016419
KRBA1	0.934154	0.858756	1.016172	0.112665
ACTR3B	1.053881	0.955953	1.16184	0.291579
BTG1	0.903671	0.846957	0.964184	0.002192
ATP13A3	0.946943	0.892458	1.004753	0.07137
KRAS	0.953492	0.885981	1.026147	0.2037
IPO8	1.064823	0.990993	1.144153	0.086683
IMPA1	1.008927	0.923957	1.101711	0.843047
LRRCC1	1.22089	1.134449	1.313916	9.99E-08
E2F5	1.10867	1.011705	1.214929	0.027163
CA1	1.003274	0.974545	1.032849	0.825488
CCDC59	0.908687	0.827629	0.997684	0.044579
SWAP70	0.888049	0.828335	0.952067	0.000829
ARNTL	1.091276	1.028049	1.158392	0.004126
LYVE1	1.076291	0.977636	1.1849	0.13391

AMPD3	1.078654	1.019573	1.141158	0.008429
SBF2	0.965157	0.900375	1.0346	0.317099
MICAL2	1.068922	1.008175	1.13333	0.025568
RRAS2	0.944333	0.894486	0.996958	0.038445
HSD17B4	1.098114	1.019914	1.182309	0.013024
ZFC3H1	0.961093	0.90319	1.022707	0.210665
RNF122	0.912612	0.854429	0.974757	0.006516
DPF2	0.992592	0.894086	1.10195	0.889104
MEN1	1.087455	1.002566	1.179532	0.043201
NUMB	0.88312	0.810587	0.962143	0.004475
COX16	1.287689	1.145326	1.447747	2.34E-05
TTC9	0.896216	0.837688	0.958832	0.001473
MED6	0.987587	0.911611	1.069895	0.759739
EIF2S1	1.138633	1.042146	1.244053	0.004056
ADAM20	1.01667	0.918796	1.12497	0.748881
LOXL2	1.022547	0.929842	1.124495	0.645642
ELP3	1.063949	0.989138	1.144418	0.095641
ADAMDEC	1.08555	1.000337	1.17802	0.049062
MBD2	0.954034	0.856748	1.062367	0.39117
IER3IP1	1.069487	0.974865	1.173293	0.155213
MRPS36	1.027988	0.925108	1.14231	0.607902
CCNB1	1.155436	1.08088	1.235136	2.18E-05
CDK7	1.017383	0.918002	1.127523	0.742453
CD180	1.032908	0.986607	1.081382	0.166447
IRAK2	0.989156	0.942096	1.038567	0.661094
CAMK1	1.310426	1.203071	1.42736	5.67E-10
THUMPD3	1.088112	0.989298	1.196796	0.08213
VHL	1.087725	0.989866	1.195258	0.080429
ARL8B	0.906446	0.835462	0.983461	0.018236
EDEM1	0.916402	0.86163	0.974656	0.005497
MEIS2	1.023897	0.941551	1.113445	0.580907
GNAT2	1.023527	0.964222	1.08648	0.445098
GSTM1	1.057216	1.016527	1.099534	0.00546
PRPF38B	1.041705	0.938372	1.156417	0.443338
TSPAN2	1.110273	1.062412	1.16029	3.27E-06
TSHB	1.29001	1.143704	1.455033	3.38E-05
GSTM5	1.132702	1.054838	1.216313	0.000605
GSTM3	1.142683	1.073316	1.216533	2.99E-05
VAV3	1.007841	0.948803	1.070552	0.799819
PSRC1	1.195191	1.119266	1.276266	1.01E-07
PTPN22	1.075076	1.007295	1.147417	0.029352
SORT1	1.109911	1.066032	1.155596	4.04E-07
WNT2B	1.13135	0.991957	1.290331	0.065828
PTGFRN	0.996218	0.932963	1.06376	0.909853
NOTCH2	1.009057	0.952993	1.06842	0.757208
TRIM45	1.19241	1.096125	1.297154	4.19E-05
CEPT1	1.024346	0.952252	1.101898	0.51827
AP4B1	1.100887	0.996422	1.216304	0.058825
NAPG	0.972084	0.895767	1.054904	0.497328
SPIRE1	1.163065	1.090539	1.240415	4.26E-06
PPHLN1	0.967358	0.885561	1.056712	0.461596
FKBP11	1.164501	1.061622	1.27735	0.001251
ARF3	1.136713	1.050358	1.230167	0.001479
TMEM106	1.259558	1.172651	1.352906	2.51E-10
SLC38A2	0.915369	0.856754	0.977995	0.00882
YWHAQ	0.975415	0.882393	1.078245	0.626423
KIDINS220	1.064647	0.984483	1.151338	0.116783
GRHL1	1.321645	1.213717	1.43917	1.40E-10

ROCK2	1.03356	0.967658	1.10395	0.326125
RSAD2	1.044465	0.996472	1.094769	0.069879
MYCN	1.014733	0.981388	1.04921	0.390938
LPIN1	1.059669	0.990138	1.134083	0.094183
CMPK2	1.054316	1.000549	1.110972	0.047646
IAH1	1.082558	0.952166	1.230806	0.225732
LDHA	0.902525	0.831994	0.979034	0.013499
IL6ST	0.961797	0.911785	1.014551	0.152798
FST	0.940658	0.831628	1.063983	0.33042
NAV1	1.024869	0.966135	1.087173	0.414611
CDC73	1.000441	0.929186	1.077161	0.990659
TIMM17A	1.154253	1.044902	1.275046	0.004729
RPS15A	0.877421	0.793413	0.970324	0.010877
RBM17	1.023665	0.913076	1.147647	0.688439
IL2RA	1.090958	1.032696	1.152506	0.001878
ANKRD16	1.085009	0.991755	1.187031	0.075174
ECHDC3	1.052123	0.986855	1.121707	0.119948
IL15RA	0.951947	0.885745	1.023098	0.180557
CCNH	0.981153	0.910539	1.057244	0.617595
HRH4	0.912449	0.835214	0.996826	0.042315
KCTD1	0.985812	0.907062	1.071399	0.736573
CABLES1	1.032444	0.981441	1.086097	0.216742
DOCK2	0.913093	0.857748	0.97201	0.004374
EMP1	0.949776	0.911893	0.989232	0.01309
SOX5	1.213233	1.113231	1.322217	1.06E-05
KLRD1	1.064961	0.970338	1.168811	0.184934
KLRC1	1.211213	1.083028	1.35457	0.000787
PRH2	1.065727	0.982575	1.155915	0.124575
LRP4	0.785533	0.722497	0.85407	1.55E-08
MYBPC3	1.060854	0.974726	1.154593	0.171493
DDB2	1.160779	1.06359	1.26685	0.000832
ACP2	1.098307	1.00399	1.201484	0.040669
RAB33A	0.992324	0.941104	1.046331	0.775646
RBMX2	1.055113	0.936991	1.188126	0.375827
PIWIL4	0.949729	0.900809	1.001306	0.05593
PUM1	0.942585	0.88228	1.007011	0.079628
PHC2	0.902835	0.833804	0.977583	0.011781
CDCA8	1.115323	1.046838	1.188289	0.000736
GNL2	0.976925	0.885543	1.077738	0.641293
HOOK1	1.144067	1.064226	1.229898	0.000266
BTF3L4	0.993012	0.901484	1.093834	0.886978
PRPF38A	0.888887	0.8191	0.96462	0.004751
DSC2	0.975036	0.926532	1.026078	0.331504
RNF138	0.938467	0.86212	1.021576	0.142403
ELP2	0.992906	0.925077	1.065708	0.843675
DSC3	1.073506	0.924784	1.246145	0.351215
DTNA	1.059002	0.987068	1.136178	0.1102
DAGLA	0.906649	0.843835	0.974138	0.007468
SLC43A3	1.109395	1.026648	1.19881	0.008667
TIMM10	1.130744	1.03423	1.236263	0.006947
DHX34	1.01816	0.940452	1.102288	0.656829
APLNR	1.089571	0.926191	1.281772	0.300703
FADS2	1.101712	1.046188	1.160184	0.000241
TCN1	0.969251	0.937776	1.001783	0.06371
TMEM165	0.987459	0.916401	1.064027	0.740487
CLOCK	1.027002	0.947808	1.112814	0.515201
COL4A2	1.055095	0.920571	1.209278	0.440895
CLDN10	0.979144	0.89907	1.06635	0.628258

UBAC2	1.00397	0.915896	1.100514	0.932591
ARGLU1	0.994767	0.911583	1.085542	0.906258
BIVM	1.166895	1.086459	1.253285	2.28E-05
ERCC5	1.02907	0.943342	1.12259	0.518475
TPP2	0.97143	0.912094	1.034625	0.367369
CARS2	1.004859	0.917519	1.100513	0.916784
STT3A	1.099718	1.018954	1.186884	0.014589
ACRV1	1.14291	1.022398	1.277628	0.018795
ETS1	0.996714	0.948346	1.047548	0.896806
SLC37A2	1.097685	1.033377	1.165996	0.002479
KLB	0.9649	0.900442	1.033973	0.311113
TMED7	0.949388	0.883213	1.020521	0.158859
APC	1.01641	0.943733	1.094683	0.667188
WDR36	1.096962	1.018781	1.181144	0.01416
OSTF1	1.000529	0.927708	1.079066	0.989054
RFK	1.094534	0.998946	1.199267	0.052702
UBQLN1	0.942473	0.873531	1.016856	0.126346
ANXA1	0.927264	0.889991	0.966099	0.000309
AGTPBP1	0.846631	0.800879	0.894996	4.26E-09
GOLM1	1.018782	0.949639	1.09296	0.603808
PSAT1	1.185647	1.120327	1.254775	3.87E-09
ISCA1	1.005052	0.926095	1.090741	0.903915
ADAM19	0.998525	0.946122	1.053831	0.957207
HAVCR2	0.857282	0.81456	0.902246	3.55E-09
CCNJL	1.111569	1.033546	1.195482	0.004392
TAOK3	0.899253	0.828648	0.975874	0.010917
USP30	0.97761	0.900557	1.061256	0.588779
SDS	1.222191	1.129512	1.322474	6.14E-07
HNF1A	0.967494	0.832084	1.124939	0.667501
FBXO21	1.18667	1.109958	1.268683	5.18E-07
OASL	1.023588	0.979925	1.069197	0.29454
HRK	1.211878	1.097356	1.338353	0.000148
RNFT2	0.987761	0.923323	1.056696	0.720515
P2RX4	0.919018	0.852308	0.99095	0.028062
DTX1	0.987927	0.937765	1.040771	0.647761
TRAFD1	1.08026	0.988097	1.18102	0.089737
DMTF1	1.027609	0.958261	1.101977	0.44488
CCDC146	1.032592	0.942244	1.131602	0.492385
TMEM60	1.07678	0.98248	1.180131	0.113654
CD36	1.053933	1.011426	1.098226	0.012388
UGT2B28	0.759622	0.705027	0.818445	5.02E-13
PNPLA8	0.889347	0.840009	0.941582	5.65E-05
RINT1	0.990297	0.917668	1.068674	0.801896
SRPK2	0.948022	0.875101	1.027019	0.191173
TES	0.893451	0.850977	0.938044	5.80E-06
MDFIC	0.96228	0.910915	1.016542	0.169516
MTO1	1.022627	0.944841	1.106816	0.579367
ANKRD6	1.011169	0.913652	1.119094	0.830034
KHDC1	1.379786	1.254287	1.517842	3.68E-11
SYNCRIP	0.978551	0.90566	1.057308	0.58301
SNX14	0.981231	0.905038	1.063839	0.645934
NT5E	0.867275	0.819259	0.918104	9.57E-07
AKIRIN2	0.813854	0.744985	0.889089	4.97E-06
LCA5	1.093767	1.011308	1.18295	0.025018
MAP3K7	1.062619	0.967841	1.166677	0.202588
CGA	1.104014	1.002601	1.215686	0.044136
LMO2	1.048273	0.969262	1.133724	0.238354
PHF21A	0.864661	0.783517	0.954209	0.003825

NAT10	0.986168	0.91836	1.058983	0.701567
PRRG4	0.977617	0.919136	1.039819	0.471962
CAPRIN1	0.979168	0.904591	1.059893	0.602475
DNAJC14	0.973492	0.882461	1.073913	0.591718
CD63	0.951939	0.872382	1.038752	0.268671
AVIL	1.026137	0.942078	1.117695	0.554071
GDF11	1.03322	0.966594	1.104439	0.336593
GLS2	1.088602	0.911843	1.299625	0.347683
ITGA7	1.089373	1.043553	1.137205	9.45E-05
FAM186B	1.09799	0.997322	1.208819	0.05674
RDH5	1.208653	1.085858	1.345335	0.000527
AGAP2	1.134329	1.058382	1.215726	0.000364
BLOC1S1	1.304915	1.191456	1.429178	9.78E-09
CDK4	1.059496	0.977939	1.147856	0.157326
PPP1R1A	1.030098	0.938827	1.130243	0.531015
TROAP	1.188169	1.112024	1.269527	3.36E-07
TSPAN31	1.094279	0.968162	1.236824	0.149279
TFCP2	1.077584	1.003156	1.157535	0.040733
COQ10A	1.078418	0.97469	1.193184	0.14343
PAN2	0.991735	0.925761	1.062411	0.813208
ESPL1	1.111988	1.040912	1.187917	0.001634
ZC3H10	1.002661	0.921178	1.091352	0.950994
HNRNPA1	0.839089	0.768808	0.915796	8.47E-05
ACVR1B	0.915872	0.860653	0.974634	0.00561
OS9	0.940351	0.859866	1.028368	0.177911
KCNH3	1.010109	0.930367	1.096685	0.810547
LTV1	1.059306	0.965868	1.161783	0.221381
MAP7	1.086499	1.038382	1.136846	0.000331
CD164	1.107263	1.024893	1.196253	0.009784
AHI1	1.036706	0.962928	1.116137	0.338547
HEY2	1.173956	1.084417	1.270889	7.43E-05
PKIB	1.106749	1.039096	1.178808	0.001624
SMPD2	1.12425	1.024889	1.233243	0.013112
MICAL1	1.147209	1.070809	1.229061	9.40E-05
REPS1	0.90637	0.835401	0.983368	0.018122
STX11	0.975189	0.915216	1.039091	0.437844
TEC	1.021187	0.95997	1.086308	0.506234
SEMA4F	1.537602	1.397038	1.692309	1.42E-18
CCT7	1.051604	0.970024	1.140044	0.221984
EGR4	0.941219	0.834654	1.06139	0.323083
RAB11FIP5	1.198618	1.136384	1.26426	2.74E-11
SMYD5	1.040232	0.957142	1.130534	0.353066
DYSF	0.980945	0.943687	1.019674	0.330154
CCDC142	1.135768	1.050178	1.228334	0.001449
EMX1	1.021777	0.907645	1.15026	0.721475
KCNMB4	0.892994	0.823026	0.96891	0.006555
USP15	0.928626	0.861797	1.000637	0.051987
GNS	1.000377	0.940257	1.06434	0.990496
CPM	1.070284	1.013843	1.129867	0.013997
MDM2	1.120277	1.034062	1.21368	0.00544
KLHL36	0.98303	0.901457	1.071984	0.698568
MPHOSPH	1.254728	1.151601	1.367091	2.15E-07
KIAA0513	1.047782	0.994843	1.103537	0.077647
DYNC1LI2	1.01793	0.953803	1.086369	0.592442
FBXL8	1.20968	1.094041	1.33754	0.000205
FHOD1	1.079683	0.993667	1.173146	0.0703
CCDC102A	1.19303	1.118739	1.272254	7.43E-08
SLC9A5	1.165806	1.0739	1.265576	0.000251

AGT	1.046039	0.988179	1.107286	0.121052
KCNK1	1.124923	1.039122	1.217809	0.003638
URB2	1.115538	1.041353	1.195007	0.001845
EGLN1	0.906391	0.851534	0.964783	0.002032
COG2	1.197303	1.087638	1.318024	0.000239
ABCB10	1.051855	0.982822	1.125738	0.144381
TAF5L	1.023923	0.939903	1.115453	0.588382
GLUL	1.016898	0.947946	1.090865	0.639961
STX6	1.015609	0.943365	1.093386	0.680788
RNASEL	1.147362	1.071864	1.228177	7.55E-05
DHX9	0.984923	0.91405	1.061292	0.690109
KIAA1614	1.222889	1.112576	1.34414	3.02E-05
CEP350	0.993294	0.93217	1.058425	0.835494
NPL	1.075694	1.019171	1.135352	0.008061
PIGC	1.255015	1.125758	1.399113	4.20E-05
LAMC1	1.054021	1.008375	1.101733	0.019848
RC3H1	0.960103	0.897289	1.027314	0.238249
GPR55	1.027169	0.912499	1.156248	0.657159
SP110	1.041093	0.952456	1.137979	0.375064
MRPL44	1.012365	0.920974	1.112825	0.799043
CHRND	1.129523	1.050632	1.214338	0.000977
DOCK10	1.073579	1.017613	1.132624	0.009346
TTLL4	1.168387	1.08485	1.258358	3.93E-05
USP37	0.995382	0.923271	1.073124	0.903979
HTR2B	0.997298	0.944357	1.053207	0.922551
ITM2C	0.911375	0.869456	0.955314	0.000112
SERPINE2	1.098413	1.033214	1.167727	0.002643
DNAJB2	1.014413	0.936394	1.098934	0.725986
WNT10A	1.029568	0.908389	1.166911	0.648329
TMBIM1	0.906354	0.835505	0.983212	0.017901
CYP27A1	1.131113	1.079229	1.185491	2.71E-07
EIF4E2	1.091225	0.96953	1.228195	0.147883
ARMC9	1.289042	1.168899	1.421533	3.65E-07
CAB39	0.975727	0.90131	1.056288	0.543801
COX5B	1.112196	1.003846	1.232241	0.042015
REV1	0.985256	0.911068	1.065486	0.709984
TSGA10	0.929572	0.852343	1.013799	0.098885
MFSD9	1.091248	0.996893	1.194534	0.05842
TMEM127	0.951173	0.880148	1.027929	0.206131
EDAR	0.994327	0.904254	1.093371	0.906514
TGFBRAP1	0.844363	0.79277	0.899314	1.45E-07
GCC2	0.987173	0.913196	1.067143	0.745309
MRPS9	1.148175	1.02743	1.283111	0.014798
ANKRD36	1.016648	0.959089	1.077661	0.578726
EPC2	0.96875	0.900003	1.042748	0.397902
ARHGEF4	0.931414	0.795938	1.089948	0.375631
ISCU	1.017451	0.918055	1.127607	0.741515
ALDH1L2	1.31737	1.181883	1.468388	6.43E-07
STAB2	0.859467	0.716818	1.030504	0.101951
USP44	0.957503	0.890315	1.02976	0.242035
SCYL2	0.979842	0.90316	1.063035	0.624301
CKAP4	1.017771	0.975707	1.061647	0.413378
PLXNC1	1.166997	1.116307	1.219989	9.36E-12
APPL2	1.004059	0.937855	1.074937	0.907332
PWP1	1.03488	0.938142	1.141592	0.493519
DRAM1	0.886011	0.831384	0.944228	0.000193
SLC41A2	1.268822	1.153097	1.396161	1.06E-06
VILL	1.095033	1.00911	1.188272	0.029446

FLNB	0.974974	0.93074	1.021311	0.284692
NEK3	1.034285	0.957501	1.117227	0.391708
VPS36	0.986627	0.912621	1.066634	0.735047
RNASEH2E	1.008708	0.930877	1.093046	0.832399
CKAP2	1.053825	0.970573	1.144218	0.211812
TBC1D4	1.126103	1.063782	1.192076	4.34E-05
THSD1	1.070937	0.991596	1.156628	0.080974
LRCH1	0.91903	0.86156	0.980334	0.010382
SUCLA2	1.098021	0.998715	1.207201	0.05319
RCBTB1	0.919627	0.859068	0.984456	0.015921
MED4	1.039598	0.945155	1.143477	0.42419
PHF11	0.936282	0.852987	1.027711	0.166062
COG3	0.971236	0.887163	1.063277	0.527527
LMO7	1.27164	1.149572	1.40667	3.05E-06
ITM2B	0.780437	0.712591	0.854742	9.17E-08
SPRY2	0.965604	0.925811	1.007107	0.103075
NUDT15	1.009256	0.925373	1.100743	0.83514
EDNRB	1.161444	1.01762	1.325596	0.026492
RCBTB2	1.021643	0.955909	1.091896	0.528019
LCP1	1.043786	0.973778	1.118827	0.226351
SETDB2	1.028695	0.959434	1.102956	0.426313
SCRN1	1.050271	1.010835	1.091246	0.01201
TNS3	1.103298	1.052361	1.1567	4.58E-05
SPDYE1	1.019272	0.933669	1.112724	0.669747
CHST12	1.147131	1.087615	1.209903	4.42E-07
IGF2BP3	1.002761	0.959046	1.048468	0.903509
GPNMB	1.125594	1.04491	1.212508	0.001824
RAPGEF5	1.146492	1.055123	1.245772	0.001254
RAC1	0.893448	0.808837	0.986911	0.026451
KDELR2	0.983526	0.89061	1.086135	0.742847
IL6	1.11504	1.052102	1.181743	0.000239
ZDHHC4	0.992188	0.882469	1.115548	0.895644
AOAH	0.975322	0.929065	1.023883	0.313496
BZW2	0.899582	0.828753	0.976464	0.011432
TBRG4	1.014449	0.935054	1.100585	0.730094
DDX56	1.088401	0.994584	1.191067	0.065493
HUS1	1.03187	0.939662	1.133126	0.511254
NACAD	0.756169	0.670332	0.852997	5.46E-06
DBNL	1.088217	1.000652	1.183444	0.048246
CCM2	0.924044	0.852422	1.001684	0.054973
MYO1G	1.002077	0.935466	1.073433	0.952847
TTYH3	1.034395	0.964222	1.109676	0.345437
TTC5	1.157333	1.066039	1.256445	0.000491
ZFHX2	1.174096	1.049642	1.313306	0.004994
MTHFS	1.18524	1.053444	1.333525	0.004719
ADAMTS7	0.926258	0.855153	1.003276	0.060146
IREB2	0.981172	0.911998	1.055593	0.610362
ALPK3	0.993619	0.941639	1.048468	0.815377
TM6SF1	0.940189	0.884308	0.999603	0.048533
CIB2	1.11853	1.028333	1.216638	0.009021
CALCOCO	0.898012	0.832596	0.968567	0.00531
RSAD1	1.113632	1.030028	1.204021	0.006872
NMT1	1.037661	0.946435	1.137681	0.431044
MYCBPAP	1.102163	1.018066	1.193206	0.016301
VEZF1	0.998424	0.915548	1.088802	0.971544
CHAD	1.094963	0.992814	1.207621	0.069427
TACO1	1.175503	1.055838	1.308731	0.003158
TEX2	1.022275	0.949493	1.100636	0.558804

DCAF7	1.083592	1.014016	1.157941	0.017738
LIMD2	0.872777	0.802877	0.948762	0.001399
BRIP1	1.072415	0.998657	1.151621	0.054479
RTP4	1.097413	1.018454	1.182492	0.014689
ACTL6A	1.196208	1.100988	1.299664	2.30E-05
NDUFB5	1.082295	0.983266	1.191297	0.106249
MRPL47	0.907696	0.817385	1.007984	0.070105
ERMN	0.916235	0.866562	0.968755	0.002097
GALNT5	1.139548	1.000038	1.298521	0.049934
TANK	0.979122	0.905974	1.058176	0.594315
BLK	1.019234	0.96076	1.081267	0.527391
SKIL	0.998473	0.950281	1.04911	0.951725
HLX	1.077856	1.009144	1.151247	0.025695
VPS45	1.145797	1.063537	1.234418	0.000343
IL10	1.016899	0.925258	1.117616	0.728007
KCTD3	0.943955	0.883487	1.008562	0.087718
RPS6KC1	1.015304	0.949382	1.085803	0.657457
CBWD2	1.108621	1.010017	1.216852	0.030031
IL1RN	1.082481	1.039325	1.12743	0.000134
SMPD4	0.973028	0.892896	1.060351	0.532915
WDR33	1.026847	0.948866	1.111237	0.510893
CCDC115	1.222237	1.091615	1.368489	0.000501
SAP130	0.975841	0.889552	1.0705	0.604652
BIN1	0.945663	0.893171	1.001239	0.055179
IMP4	1.147534	1.026204	1.283208	0.015794
HS6ST1	1.011005	0.952241	1.073396	0.720172
GYPC	0.894291	0.855069	0.935312	1.05E-06
STAM	0.915012	0.861017	0.972392	0.004208
ABI1	0.896912	0.826754	0.973023	0.008844
YME1L1	0.939296	0.870296	1.013766	0.107673
DNAJC1	1.014181	0.944634	1.088848	0.69764
NIPSNAP3	1.027795	0.942232	1.121128	0.53644
LRRC8A	0.871959	0.813921	0.934136	9.67E-05
CDK9	1.106574	1.0163	1.204867	0.019683
TXN	0.995455	0.917824	1.079653	0.912448
ODF2	1.120349	1.026448	1.222841	0.010945
TOR1B	1.127657	1.050642	1.210318	0.000873
SMC2	1.125599	1.052577	1.203686	0.000546
KLF4	1.026674	0.991986	1.062575	0.133323
TOR1A	1.194208	1.080145	1.320316	0.00053
RALGPS1	1.258738	1.14428	1.384645	2.24E-06
ST6GALNA	1.126665	1.051848	1.206804	0.000669
TMOD1	1.015708	0.970381	1.063153	0.503407
DAB2IP	1.139691	1.031569	1.259147	0.010137
STXBP1	1.209382	1.117264	1.309095	2.56E-06
SLC2A8	1.123395	1.044743	1.20797	0.001679
ANGPTL2	1.241281	1.104557	1.394929	0.000283
CDK5RAP2	1.000065	0.928114	1.077595	0.998635
SLC31A2	0.984623	0.926826	1.046024	0.615606
SLC31A1	1.03505	0.954982	1.121832	0.401676
TLR4	1.052414	1.000061	1.107507	0.049727
ZNF189	0.995608	0.935687	1.059366	0.889458
STX17	1.106642	1.02496	1.194834	0.009593
PRPF4	1.050054	0.950699	1.159793	0.335517
FPGS	1.014895	0.929558	1.108065	0.741455
USP20	1.094835	1.026051	1.168229	0.006204
ATP6V1G1	0.958668	0.865442	1.061937	0.418709
TEX10	0.935936	0.867326	1.009974	0.088293

GARNL3	1.379882	1.217538	1.563872	4.60E-07
MRPL50	1.106081	1.002841	1.21995	0.043726
DPM2	1.158706	1.071646	1.252837	0.000219
WDR38	1.056806	0.981532	1.137853	0.142777
TSTD2	1.005911	0.927696	1.09072	0.886523
HEMGN	1.006848	0.9747	1.040056	0.680184
PSMB7	0.88278	0.79649	0.978418	0.017515
NR5A1	0.891342	0.810205	0.980605	0.018168
RABEPK	1.056948	0.960218	1.163422	0.258056
GOLGA1	1.048761	0.970713	1.133084	0.227574
XPA	0.9665	0.889013	1.050741	0.424215
NCBP1	1.018365	0.949253	1.092509	0.611781
ANP32B	0.945379	0.857267	1.042548	0.260491
PDCL	1.095849	1.003814	1.196323	0.040853
RPL35	0.952184	0.879814	1.030507	0.224419
LMX1B	0.94807	0.836526	1.074489	0.403719
ARPC5L	1.075575	0.988929	1.169812	0.089098
ENPP2	1.10921	1.044627	1.177786	0.000708
DSCC1	1.189481	1.095138	1.29195	3.86E-05
DERL1	0.984359	0.903763	1.072143	0.717577
MYC	0.940364	0.881057	1.003664	0.064324
RANBP6	0.969691	0.908627	1.034858	0.353686
POLR1E	0.980057	0.885027	1.085291	0.698674
PLAA	0.917416	0.844036	0.997176	0.042717
IL11RA	1.085467	1.011425	1.16493	0.0229
UBAP2	0.950423	0.864804	1.044519	0.29112
APTX	1.136663	1.007632	1.282218	0.037194
RNF38	0.88133	0.812029	0.956547	0.002501
TLN1	1.035887	0.963486	1.113729	0.340205
SIT1	1.013433	0.946571	1.085018	0.701585
DNAJB5	1.045916	0.978846	1.117582	0.184292
SPAG8	1.293812	1.166594	1.434903	1.07E-06
DCTN3	1.211102	1.070323	1.370399	0.002382
CD72	0.998691	0.936282	1.065261	0.968271
TMEM8B	1.438899	1.292594	1.601764	2.91E-11
GRHPR	0.888925	0.806455	0.979827	0.017779
ALDH1B1	1.124082	1.063011	1.188662	4.06E-05
DENND4C	0.883919	0.832873	0.938095	4.79E-05
RPS6	0.821135	0.75754	0.890069	1.66E-06
CNPY3	1.021532	0.936358	1.114453	0.631517
FOXP4	1.043669	0.977266	1.114585	0.20254
PPIL1	1.180342	1.10095	1.265458	3.06E-06
KLC4	0.99386	0.909208	1.086394	0.892138
KIF13A	0.919944	0.869505	0.97331	0.003728
PIM1	1.142982	1.081769	1.207658	1.95E-06
GMPR	0.980635	0.935289	1.02818	0.418225
YIPF3	1.105142	0.981602	1.244231	0.098342
TMEM14B	1.233007	1.104515	1.376446	0.000191
TMEM63B	1.021615	0.95826	1.089159	0.512665
FRS3	1.168907	1.059377	1.289762	0.001877
TJAP1	1.057918	0.940528	1.189959	0.348129
CAPN11	0.912091	0.849759	0.978997	0.010844
IRF4	1.023631	0.977307	1.072152	0.322918
SLC22A23	1.116744	1.059302	1.177301	4.16E-05
TUBB2A	1.001692	0.953707	1.052091	0.946187
LRRC1	1.16886	1.062618	1.285725	0.001331
GCM1	1.043035	0.944667	1.151646	0.40446
BPHL	1.283465	1.141163	1.443512	3.15E-05

RIPK1	1.037272	0.953322	1.128614	0.395422
TUBB2B	0.959837	0.85055	1.083167	0.506282
HMGA1	0.865308	0.810028	0.92436	1.75E-05
TCF19	1.127212	1.058185	1.200741	0.000204
FLOT1	1.041061	0.970515	1.116733	0.261012
IER3	1.006415	0.964863	1.049757	0.766276
MDC1	1.107399	0.995335	1.23208	0.060921
PGBD1	1.104067	1.0205	1.194476	0.013691
TPMT	1.126018	1.015756	1.248248	0.02399
RNF144B	1.033988	0.98604	1.084269	0.167692
NRM	1.035631	0.943639	1.136592	0.460713
MTCH1	1.007784	0.929984	1.092093	0.849965
VARS2	1.431998	1.149152	1.784463	0.001383
TAF8	1.097185	1.013098	1.18825	0.022617
FAM8A1	0.94515	0.883288	1.011343	0.102391
FGFBP2	1.010483	0.952335	1.072182	0.73018
CPEB2	1.166462	1.110837	1.224872	6.56E-10
FHDC1	1.046183	1.002488	1.091781	0.038066
TLR2	0.962459	0.916602	1.01061	0.124477
MYO7A	1.179607	1.093849	1.272089	1.79E-05
FCHSD2	1.0017	0.930343	1.078531	0.964059
ARRB1	1.017549	0.954404	1.084872	0.594573
SLCO2B1	1.060103	0.904963	1.241839	0.469689
ANKRD42	0.990241	0.902665	1.086313	0.835552
IL18BP	1.13036	1.024367	1.247321	0.01472
NUMA1	0.911393	0.846852	0.980852	0.013291
CCDC90B	1.065042	0.948301	1.196154	0.287419
SYTL2	0.912162	0.846857	0.982503	0.01528
RAB30	1.026644	0.965251	1.091941	0.403263
CREBZF	0.995559	0.930979	1.064618	0.896506
LRRC32	1.122237	1.049741	1.19974	0.000713
PRCP	1.045771	0.955092	1.145059	0.333498
NARS2	0.983642	0.918729	1.053142	0.635867
RNF121	1.087972	1.005335	1.177402	0.036441
MRPL15	0.97866	0.902423	1.061338	0.602162
PI15	1.243434	1.140089	1.356146	8.59E-07
GGH	1.276634	1.157949	1.407484	9.31E-07
SLCO5A1	0.825557	0.782545	0.870935	2.19E-12
TGS1	1.013996	0.929303	1.106408	0.754781
SDCBP	0.928335	0.857429	1.005104	0.066599
NEK1	1.07999	0.994571	1.172745	0.067178
DDX60	1.083443	1.023948	1.146396	0.005415
SORL1	0.936078	0.893615	0.980559	0.00529
BUD13	0.896466	0.819133	0.9811	0.017572
TRPC6	1.146054	1.087559	1.207696	3.39E-07
MMP7	1.269168	1.180904	1.36403	9.10E-11
DCUN1D5	1.200693	1.076277	1.339491	0.001049
SLC37A4	1.132058	1.0338	1.239655	0.007417
RDX	1.089085	1.005458	1.179668	0.036306
PPP2R1B	1.149699	1.066279	1.239646	0.000284
FDX1	0.940353	0.852077	1.037774	0.221418
FXYD6	1.187611	1.081625	1.303982	0.000312
TMPRSS13	1.203732	1.091838	1.327094	0.000195
CASP1	1.061066	0.99795	1.128175	0.058176
CASP5	0.98906	0.86898	1.125735	0.867713
ALKBH8	1.157658	1.065127	1.258227	0.000572
MAP2K5	0.951932	0.868984	1.042798	0.289585
CTDSPL2	0.941958	0.875492	1.01347	0.109246

SLTM	0.907014	0.82505	0.99712	0.04342
THBS1	1.041809	1.01179	1.072719	0.006039
MAPKBP1	1.05225	0.980608	1.129126	0.15688
NUSAP1	1.218903	1.078848	1.37714	0.00148
NDUFAF1	1.195935	1.084941	1.318284	0.000318
KIF23	1.103649	1.030985	1.181434	0.004538
ITGA11	0.776906	0.642808	0.938979	0.009021
RTF1	0.942032	0.858662	1.033496	0.206559
PARP6	0.947348	0.863731	1.039059	0.251273
RPLP1	0.902741	0.844443	0.965063	0.002664
PAQR5	0.974577	0.880196	1.079077	0.620232
TUBGCP4	1.129947	1.028173	1.241795	0.011184
ITPKA	0.946552	0.888834	1.008019	0.087051
UACA	1.329882	1.19874	1.475371	7.36E-08
SMAD6	1.171094	1.087562	1.261042	2.87E-05
PLCB2	1.07021	1.007662	1.13664	0.027219
TMEM62	0.999645	0.915702	1.091283	0.993669
PAK6	0.929893	0.854728	1.011669	0.09099
ADAM10	1.014386	0.946028	1.087683	0.688225
DUOX1	1.043466	0.961454	1.132473	0.308319
ZNF280D	0.946997	0.863124	1.03902	0.249744
BCL2L10	1.0737	1.012561	1.138531	0.017442
RSL24D1	0.871404	0.799409	0.949884	0.001757
SPTBN5	1.122773	1.016875	1.2397	0.021961
GCHFR	1.169603	1.049268	1.303739	0.004682
BCAR3	0.967976	0.903535	1.037014	0.354467
TTLL7	1.212018	1.10108	1.334134	8.64E-05
FNBP1L	0.957834	0.912352	1.005582	0.082617
GTF2B	0.93829	0.854906	1.029807	0.179787
RABGGTB	0.946574	0.87869	1.019701	0.148144
IFI44L	1.053208	1.012411	1.095648	0.010114
IFI44	1.009701	0.963712	1.057883	0.684824
SLC44A5	0.971635	0.891881	1.05852	0.510215
DBT	1.051749	0.962317	1.149492	0.265797
IFT172	1.02417	0.942889	1.112459	0.571338
CGREF1	1.389091	1.276738	1.511331	2.22E-14
HADHB	1.024982	0.928018	1.132076	0.626512
KHK	1.43249	1.327945	1.545266	1.46E-20
ADCY3	1.028675	0.961762	1.100244	0.410027
PPM1B	0.969073	0.899106	1.044485	0.411286
PNPT1	1.14132	1.054972	1.234735	0.000991
DYNC2LI1	1.041356	0.938587	1.155378	0.444621
THUMPD2	1.059157	0.977756	1.147335	0.158945
CYP1B1	1.123256	1.060863	1.189318	6.71E-05
RAB1A	0.931655	0.85954	1.009821	0.085031
ACTR2	1.07069	0.9951	1.152022	0.067478
PREB	1.144226	1.070635	1.222875	7.12E-05
SLC5A6	1.082118	1.009769	1.159651	0.025396
PREPL	1.108388	1.032293	1.190092	0.004571
SLC3A1	1.181	1.030237	1.353826	0.016965
EMILIN1	1.012294	0.975026	1.050986	0.523173
FBXO11	0.947468	0.880038	1.020065	0.151985
SIX3	0.749627	0.674896	0.832634	7.52E-08
CENPO	1.094183	1.004163	1.192272	0.039897
LRPPRC	0.985613	0.918614	1.057499	0.686613
DTNB	1.161502	1.052607	1.281663	0.002876
ACTR1A	1.049015	0.949104	1.159443	0.348738
CYP2C8	1.148919	1.037302	1.272546	0.00776

MYOF	1.106984	1.062087	1.15378	1.50E-06
LOXL4	0.946019	0.895349	0.999558	0.048188
STAMBPL1	1.013894	0.935934	1.098348	0.735348
CH25H	1.035385	0.952708	1.125237	0.412802
ATAD1	1.001983	0.928434	1.081359	0.959376
KIF11	1.110217	1.04116	1.183855	0.001418
DUSP5	0.942091	0.896574	0.989919	0.018226
CALHM2	1.077777	1.013156	1.146519	0.017584
ARL3	1.216404	1.11465	1.327446	1.11E-05
CEP55	1.157304	1.082669	1.237084	1.75E-05
KIF20B	1.007236	0.937682	1.08195	0.843435
ENTPD1	0.996027	0.936398	1.059452	0.89942
EXOC6	1.063689	0.981649	1.152585	0.131631
PLCE1	1.208225	1.084188	1.346453	0.00062
DBR1	1.106051	1.009889	1.211369	0.029855
DNAJC13	1.008609	0.951312	1.069358	0.773896
GPR87	1.156819	1.078281	1.241077	4.89E-05
ANXA7	1.034941	0.936418	1.14383	0.501021
FAM149B1	1.015328	0.925932	1.113355	0.746328
ASCC1	1.10907	1.001649	1.22801	0.046408
ADAMTS1	1.056607	0.998978	1.11756	0.054326
RPS24	0.890441	0.832802	0.952069	0.000678
TET1	1.020956	0.952271	1.094595	0.559448
DNA2	1.102905	1.025846	1.185752	0.008038
AOX1	0.87217	0.787358	0.966119	0.008784
ATIC	1.06502	0.986138	1.150213	0.108619
SMARCAL1	1.023619	0.956984	1.094895	0.496676
BARD1	1.066145	0.997446	1.139575	0.05947
STAT4	1.040491	0.987357	1.096484	0.137757
ASNSD1	1.056541	0.959804	1.163029	0.261612
METTL5	1.199034	1.074263	1.338296	0.001205
SSB	1.098885	0.99368	1.215229	0.066286
NAB1	0.975491	0.896185	1.061815	0.566259
PPIG	0.928216	0.839039	1.026871	0.148335
FASTKD1	1.0632	0.987478	1.14473	0.104017
IDH1	1.092062	1.028349	1.159723	0.004087
OLA1	0.968031	0.873593	1.072679	0.535015
CIR1	0.936786	0.856223	1.024929	0.154657
FAM117B	1.031816	0.956491	1.113072	0.418053
WDR12	1.100014	1.020393	1.185849	0.012898
ABI2	1.074353	0.997231	1.15744	0.059159
ITGAV	0.960278	0.916034	1.00666	0.092151
SLC40A1	1.105156	1.065894	1.145864	6.04E-08
SLC35A5	1.036676	0.966606	1.111824	0.313093
SENP7	1.000758	0.929523	1.077452	0.983954
COX17	0.995038	0.880482	1.1245	0.936472
PARP9	1.062292	0.991629	1.13799	0.085321
MNS1	1.097575	0.992588	1.213666	0.069534
USP8	0.978848	0.896071	1.069273	0.635342
SECISBP2L	0.94437	0.888927	1.00327	0.063711
TMOD3	0.944336	0.871566	1.023181	0.161558
SPPL2A	1.026349	0.940983	1.119459	0.557201
GLCE	1.211028	1.116649	1.313383	3.74E-06
SHF	0.916873	0.850461	0.988471	0.023684
APH1B	1.019473	0.938057	1.107956	0.649713
PARP16	0.964258	0.871779	1.066547	0.479232
PPCDC	1.196333	1.114422	1.284264	7.28E-07
SEMA7A	1.016198	0.965722	1.069313	0.536474

UBL7	1.068048	0.967121	1.179507	0.193651
ARHGAP24	1.004955	0.942153	1.071944	0.880668
FAM13A	1.001898	0.929754	1.07964	0.960333
HERC3	0.973214	0.895959	1.057131	0.519968
HERC6	1.067275	0.991073	1.149336	0.08494
HERC5	1.07139	1.015654	1.130184	0.011412
PCDH10	1.104729	1.040376	1.173062	0.001144
COPS4	0.938984	0.84267	1.046305	0.254208
HNRNPD	0.890921	0.803958	0.987291	0.027521
PRKG2	1.218872	1.121883	1.324245	2.89E-06
RASGEF1B	0.984724	0.928324	1.044551	0.608975
SEC31A	0.911612	0.837744	0.991993	0.031839
BBS7	1.223234	1.112492	1.344999	3.16E-05
KIAA1109	0.993039	0.935751	1.053834	0.817769
BMPR1B	1.138608	1.034652	1.253009	0.007876
RAP1GDS1	1.093761	1.007836	1.187012	0.031797
LARP1B	1.020309	0.921684	1.129488	0.698289
MMRN1	1.146377	1.109786	1.184175	1.54E-16
PDE5A	1.10275	1.029713	1.180968	0.005151
PRDM5	1.275575	1.156035	1.407477	1.25E-06
NAAA	1.032204	0.970431	1.097909	0.314087
NUP54	0.924626	0.857234	0.997316	0.042401
CXCL9	1.014131	0.922788	1.114516	0.770762
BMP2K	1.015249	0.951201	1.083609	0.648975
G3BP2	0.981389	0.893876	1.07747	0.69342
FRAS1	1.16676	1.099746	1.237859	3.22E-07
SCARB2	0.9069	0.837615	0.981916	0.015951
CCNG2	0.987885	0.913094	1.068803	0.761551
CNOT6L	1.009752	0.945227	1.078681	0.773322
USO1	0.969425	0.900276	1.043885	0.410831
ANXA3	1.040758	1.003747	1.079133	0.030585
PPA2	1.062516	0.95372	1.183725	0.271235
CENPE	1.133872	1.055671	1.217865	0.000569
GSTCD	1.136455	1.037976	1.244277	0.005676
INTS12	0.956863	0.852291	1.074265	0.455201
CASP6	0.992835	0.929081	1.060965	0.831838
LEF1	1.010561	0.966405	1.056734	0.644898
HADH	1.124929	1.020248	1.240352	0.018167
EGF	1.237257	1.141107	1.341507	2.50E-07
PAPSS1	0.952892	0.883263	1.02801	0.212614
SEC24B	0.903612	0.839371	0.972769	0.007066
PPP3CA	0.93881	0.871559	1.011251	0.095922
SLC39A8	0.981267	0.91593	1.051266	0.59065
FBN2	0.943982	0.898575	0.991683	0.021906
MAPK8IP3	0.911031	0.85543	0.970246	0.003731
RGS3	1.058152	0.996966	1.123093	0.062888
TTLL8	0.939932	0.856629	1.031335	0.190763
RNF185	1.080077	0.978009	1.192798	0.128279
PARVG	1.114001	1.037296	1.196378	0.003017
B4GALNT3	1.178328	1.067578	1.300567	0.00112
PDE6H	0.975351	0.935453	1.01695	0.241517
ERP27	0.979181	0.922919	1.038873	0.485911
ETV6	0.89706	0.837777	0.960537	0.001845
GABARAPI	0.930231	0.877062	0.986624	0.016022
KIF21A	1.06053	0.949014	1.185149	0.299847
CPNE8	1.162815	1.121568	1.205579	2.70E-16
YARS2	1.058267	0.962213	1.163911	0.2434
FGD4	1.029232	0.978031	1.083114	0.268417

ALG10	1.030144	0.951746	1.115	0.462121
AEBP2	0.958815	0.87191	1.054382	0.385623
ETNK1	1.042139	0.970893	1.118614	0.253286
ZCRB1	1.014388	0.908423	1.132713	0.799676
TMEM117	1.104485	1.014671	1.20225	0.021646
PRICKLE1	1.177605	1.08848	1.274027	4.67E-05
C1RL	1.135739	1.057115	1.220211	0.000506
NDUFA9	1.179195	1.052055	1.321699	0.004629
CLSTN3	1.037729	0.97236	1.107494	0.264585
KLRG1	1.238612	1.155624	1.327558	1.47E-09
VAMP1	1.031725	0.962988	1.105367	0.374625
TAPBPL	1.015807	0.926533	1.113683	0.738254
CD27	0.999749	0.928841	1.07607	0.994662
RBP5	1.018806	0.950415	1.092119	0.599221
PEX5	1.044093	0.956537	1.139663	0.334253
SLC38A4	0.973723	0.894321	1.060175	0.539508
AMIGO2	0.987883	0.916173	1.065206	0.751192
COL2A1	0.991813	0.921955	1.066965	0.825406
LLPH	1.107991	0.988467	1.241968	0.078274
INHBE	0.94669	0.835213	1.073046	0.391424
GLIPR1	0.953605	0.895051	1.015989	0.141743
PHLDA1	1.077798	1.029998	1.127816	0.001208
TMEM19	1.092369	0.99602	1.198039	0.060751
DUSP6	1.03197	0.988882	1.076935	0.148124
TMTC3	1.161949	1.053238	1.28188	0.002745
LUM	1.030255	0.956809	1.109339	0.429583
SNRPF	1.046312	0.966672	1.132514	0.262376
AMDHD1	0.896388	0.773526	1.038765	0.145864
NEDD1	1.008035	0.929147	1.093621	0.847361
SYCP3	0.97045	0.876583	1.074368	0.563324
GAS2L3	1.238475	1.165793	1.315689	4.17E-12
SLC15A4	0.997943	0.937176	1.06265	0.948778
TDG	1.106428	1.011165	1.210665	0.027688
SDSL	1.178665	1.106616	1.255404	3.26E-07
MMAB	1.324962	1.175248	1.493747	4.23E-06
GLTP	1.128485	1.026592	1.240491	0.012296
GIT2	0.977692	0.898578	1.063772	0.600266
TCHP	1.059114	0.964041	1.163563	0.231376
MTMR6	0.898691	0.839506	0.962049	0.002119
SLC46A3	1.259697	1.176327	1.348975	3.89E-11
SLC7A1	1.049194	0.983366	1.119429	0.146337
LNX2	1.063739	0.983254	1.150813	0.123739
SUOX	1.073427	0.987769	1.166512	0.094933
CCDC65	1.068554	0.995662	1.146782	0.06586
SLC39A5	1.074206	0.957043	1.205713	0.224438
TARBP2	1.093596	0.996916	1.199652	0.058151
DHH	0.999228	0.922577	1.082248	0.984872
ACVRL1	1.153609	1.067687	1.246445	0.000296
GPR84	1.0719	1.020078	1.126354	0.006029
N4BP2L1	0.973544	0.891087	1.063632	0.55266
SMARCC2	1.041714	0.956641	1.134353	0.347123
BRCA2	1.039956	0.968695	1.11646	0.279354
MAP3K12	0.980225	0.918383	1.04623	0.548021
ITGB7	1.008413	0.95702	1.062565	0.753598
GALNT6	1.027186	0.949163	1.111622	0.505741
CSAD	1.120334	1.033751	1.214169	0.005626
LMBR1L	0.943128	0.886076	1.003853	0.065892
ESYT1	0.931729	0.872751	0.994692	0.034049

TMBIM6	0.958106	0.868062	1.05749	0.395386
ANKRD52	1.014761	0.941116	1.094169	0.703057
ZNF740	1.024292	0.956628	1.096743	0.491239
WDFY2	0.969088	0.909943	1.032077	0.328428
HNRNPA1	0.961204	0.880594	1.049193	0.375936
ESD	0.942243	0.856321	1.036787	0.222672
RB1	1.041498	0.968468	1.120036	0.272997
SBNO1	1.036446	0.967158	1.110697	0.310572
MORN3	1.048175	0.964515	1.139093	0.267584
SETD1B	1.029703	0.950579	1.115413	0.473055
VPS33A	1.156753	1.046458	1.278673	0.004397
VPS37B	0.861719	0.797918	0.930621	0.000149
RHOF	1.05673	0.95306	1.171677	0.294925
DENR	1.032814	0.939125	1.135851	0.505751
DIAPH3	1.121145	1.038239	1.210672	0.00353
SLAIN1	0.99826	0.946103	1.053292	0.949284
RBM26	0.975297	0.900546	1.056253	0.538681
MBNL2	0.870179	0.821119	0.922169	2.64E-06
ABHD13	1.022495	0.933087	1.120471	0.633714
RAB20	0.969424	0.916975	1.024873	0.273858
GRTP1	1.001961	0.937433	1.07093	0.954008
CUL4A	0.983018	0.896844	1.077472	0.714437
CDH24	1.065187	0.998174	1.136699	0.056803
REM2	1.07187	1.007196	1.140697	0.028832
CBLN3	1.256605	1.125116	1.403461	5.11E-05
TSSK4	1.015331	0.949818	1.085364	0.654808
TMX1	1.105508	1.015725	1.203228	0.020287
FRMD6	1.00975	0.890649	1.144778	0.879563
PELI2	0.983379	0.925985	1.044331	0.584892
RTN1	1.138227	1.073569	1.206778	1.43E-05
SLC38A6	1.173925	1.058567	1.301855	0.002378
RDH12	1.023286	0.933888	1.121242	0.621645
RAB15	1.019334	0.953966	1.089182	0.571186
WDR89	1.060518	0.975924	1.152444	0.165942
ESR2	1.013106	0.91923	1.116569	0.792976
STON2	0.940535	0.901911	0.980812	0.004163
GPR65	0.942955	0.87408	1.017257	0.129057
PTGR2	1.0676	0.917605	1.242114	0.397106
JDP2	0.929479	0.875861	0.986378	0.015848
AK7	1.258184	1.100143	1.438929	0.000798
SLC24A4	1.013994	0.955497	1.076071	0.646682
FBLN5	0.906972	0.853158	0.964181	0.001755
WDR20	0.979093	0.910147	1.053261	0.570622
NIPA2	1.026899	0.93202	1.131436	0.59152
SLC12A6	0.978906	0.917078	1.044902	0.521864
MFAP1	0.973431	0.896619	1.056824	0.520806
TCF12	0.941386	0.873178	1.014921	0.115487
SORD	0.883388	0.807012	0.966993	0.0072
SERF2	1.019226	0.916531	1.133429	0.72525
ZSCAN29	1.053079	0.9759	1.136363	0.182935
LYSMD2	0.947542	0.872968	1.028486	0.197615
SLC27A2	0.868116	0.816436	0.923067	6.29E-06
HDC	0.860591	0.82905	0.89333	3.25E-15
BNIP2	0.918053	0.845741	0.996549	0.041097
GTF2A2	1.212986	1.092079	1.347279	0.000313
SRP14	0.982092	0.903918	1.067026	0.669381
BAHD1	0.929377	0.861214	1.002934	0.059487
DISP2	1.085732	0.9689	1.21665	0.156758

CDAN1	1.003583	0.938967	1.072645	0.916122
TLE3	1.088931	1.018409	1.164336	0.012633
ANP32A	1.018768	0.930768	1.115088	0.686652
COMMD4	1.462464	1.309161	1.633719	1.72E-11
UBE2Q2	0.926586	0.852913	1.006622	0.071259
PSTPIP1	1.106522	1.046533	1.169949	0.000372
ETFA	1.069288	0.973952	1.173955	0.159716
BCL2A1	1.004851	0.964068	1.04736	0.818917
HMG20A	1.019965	0.933462	1.114484	0.661975
SCAPER	1.055498	0.972499	1.145581	0.196145
TSPAN3	1.069406	0.969113	1.180079	0.181701
WDR61	1.344666	1.20441	1.501256	1.37E-07
NCOA2	1.011323	0.944032	1.08341	0.748592
NEIL1	1.134065	1.066645	1.205746	5.74E-05
MAN2C1	1.02833	0.958512	1.103234	0.436118
DNAJA4	1.096649	1.042408	1.153711	0.000364
TPM1	1.225959	1.153108	1.303413	7.14E-11
IGF1R	0.966709	0.924446	1.010905	0.137689
ARRDC4	1.077603	1.016182	1.142738	0.012558
PIF1	1.233147	1.138865	1.335235	2.41E-07
USP3	0.968461	0.90567	1.035605	0.348753
BBS4	1.079827	0.982614	1.186657	0.110584
PML	0.907218	0.831773	0.989506	0.027941
CYP1A1	0.978663	0.855449	1.119624	0.753406
ADAMTS1	1.137404	1.005393	1.286747	0.040814
LINS1	1.180388	1.075157	1.29592	0.0005
ULK3	1.149102	1.052335	1.254768	0.001958
PCSK6	0.797753	0.683875	0.930594	0.004036
SCAMP2	1.050759	0.945747	1.167431	0.356712
HAPLN3	1.071104	1.010162	1.135722	0.021547
POLG	1.023883	0.955664	1.096972	0.502276
RLBP1	0.989439	0.93638	1.045504	0.705758
FANCI	1.073864	1.002383	1.150443	0.042591
ABHD2	0.978416	0.917963	1.042851	0.502507
DET1	1.008381	0.900199	1.129564	0.885389
MFGE8	1.073277	1.010315	1.140161	0.021866
ZNF710	0.995089	0.924651	1.070892	0.895425
UNC45A	1.050541	0.964906	1.143776	0.255744
MCTP2	0.967933	0.924317	1.013607	0.165912
FURIN	0.947354	0.880747	1.018999	0.145952
IQGAP1	0.972943	0.91467	1.03493	0.384063
CRTC3	0.955591	0.883461	1.033611	0.25663
SEC11A	0.859871	0.775621	0.953273	0.004111
PMM2	1.102267	1.005661	1.208152	0.037471
ITGAX	1.104457	1.051558	1.160016	7.26E-05
TGFB1I1	0.901596	0.830275	0.979043	0.013751
ARMC5	1.066587	1.002164	1.135151	0.042565
PARN	1.054922	0.966686	1.151212	0.230251
FTO	0.998992	0.936464	1.065695	0.975608
UQCRC2	0.906922	0.824213	0.997931	0.045241
CDR2	0.91966	0.856436	0.987552	0.021185
IGSF6	1.007295	0.94476	1.073969	0.824104
ARHGAP1	0.920198	0.850099	0.996078	0.039671
NKD1	1.059553	0.982808	1.142292	0.131577
DHX38	0.964192	0.885966	1.049325	0.398293
TXNL4B	1.170913	1.039183	1.31934	0.009565
ZFHX3	0.921668	0.865228	0.981789	0.011406
CPNE2	1.021061	0.949295	1.098253	0.575115

NLRC5	1.023602	0.965778	1.084888	0.431705
KATNB1	1.047282	0.968417	1.13257	0.247454
KIFC3	1.120286	1.058893	1.185239	7.82E-05
ADAMTS1	1.068136	0.981537	1.162376	0.126521
NUDT7	1.085696	1.000693	1.177921	0.048085
GCSH	1.355629	1.133977	1.620606	0.000837
CMTM3	0.873275	0.815499	0.935144	0.000104
CMTM2	1.049475	0.985061	1.118101	0.13512
NOL3	1.155238	1.044462	1.277762	0.005019
MAP1LC3E	0.905943	0.834518	0.98348	0.018397
MBTPS1	0.906438	0.84081	0.977189	0.010416
ZCCHC14	1.026212	0.961896	1.094829	0.433312
OSGIN1	1.022377	0.927994	1.126359	0.65429
IRF8	1.091036	1.043027	1.141254	0.000148
RHOT2	1.087521	0.996007	1.187445	0.06138
RPL3L	1.092469	0.949093	1.257504	0.217918
RPS2	0.867598	0.805353	0.934654	0.000185
NDUFB10	1.065097	0.974903	1.163634	0.162429
PDPK1	0.992829	0.912488	1.080243	0.867236
TIGD7	1.20087	1.014694	1.421204	0.033198
DEF8	1.011969	0.942353	1.086727	0.743538
TCF25	0.966913	0.877563	1.06536	0.496412
GALNS	1.049532	0.967351	1.138694	0.245205
GAS8	1.186513	1.042804	1.350025	0.009424
MED9	1.115022	1.020879	1.217846	0.015558
NCOR1	0.968543	0.899909	1.042413	0.394044
COPS3	0.940783	0.847649	1.04415	0.251099
ZNF287	1.63182	1.405201	1.894986	1.37E-10
KSR1	0.951191	0.891562	1.014809	0.129791
RANBP10	0.964054	0.898848	1.033991	0.305595
DPEP3	0.996055	0.94489	1.04999	0.883192
GFOD2	1.01936	0.939569	1.105928	0.644739
NOB1	0.87551	0.801659	0.956165	0.003107
PRPSAP2	1.229002	1.108958	1.36204	8.42E-05
PCTP	1.074286	0.981305	1.176076	0.12081
TOM1L1	1.194971	1.109227	1.287343	2.75E-06
TOB1	0.889688	0.83954	0.942831	7.86E-05
VPS53	1.039826	0.959408	1.126985	0.341635
SGSM2	0.97479	0.898657	1.057374	0.538302
NPEPPS	0.9726	0.900016	1.051038	0.482644
SKAP1	0.952665	0.896104	1.012797	0.120476
LRRC46	1.118879	0.988821	1.266043	0.074806
SCRN2	1.06579	0.9794	1.1598	0.139586
SSH2	0.922624	0.858372	0.991687	0.02877
RHBDL3	1.046248	0.98407	1.112355	0.148105
ARSG	1.145771	1.050682	1.249465	0.002081
G6PC3	1.074158	0.98731	1.168647	0.096301
CLTC	0.997249	0.931588	1.067538	0.936814
BCAS3	1.117716	1.029885	1.213038	0.007695
PTRH2	1.015223	0.935143	1.10216	0.718557
SS18	0.971723	0.897373	1.052232	0.479993
TAF4B	0.967293	0.90612	1.032596	0.318446
AFG3L2	0.975135	0.88979	1.068667	0.590016
IMPA2	0.953621	0.896427	1.014464	0.132355
GNAL	1.142355	0.941096	1.386654	0.178306
SLC39A6	1.106406	1.01763	1.202927	0.017813
RPRD1A	1.056347	0.977509	1.141544	0.166006
GALNT1	0.906693	0.843071	0.975115	0.008319

ADCYAP1	0.960095	0.824362	1.118178	0.600529
ESCO1	1.029445	0.948733	1.117023	0.486044
OSBPL1A	1.011911	0.955733	1.071392	0.684508
PELP1	0.935823	0.853719	1.025824	0.156846
NPC1	0.957139	0.89256	1.026392	0.21904
SLC14A1	0.949795	0.898634	1.003868	0.068257
ARRB2	0.958108	0.892125	1.028971	0.239798
ZMYND15	1.129789	1.033843	1.234638	0.007039
MINK1	1.013871	0.94	1.093549	0.721162
SAT2	0.947788	0.858288	1.046622	0.289335
ASGR1	1.163429	1.05771	1.279714	0.001844
PIK3R5	1.073613	1.011387	1.139668	0.01972
TP53	1.076189	1.009492	1.147293	0.024489
ARHGDIA	0.874145	0.813836	0.938924	0.000226
TMC6	1.045816	0.969769	1.127826	0.244828
SLC16A3	1.052671	1.002526	1.105324	0.039275
CARD14	1.081828	0.954669	1.225923	0.217643
TTYH2	0.885172	0.820552	0.954881	0.001612
RAB40B	1.354285	1.221714	1.501241	7.93E-09
EIF4A3	0.968834	0.897774	1.045518	0.415269
CSNK1D	1.014283	0.948985	1.084075	0.676151
ANAPC11	1.178852	1.072156	1.296166	0.000675
TBCD	1.192386	1.100534	1.291904	1.69E-05
FN3KRP	1.217545	1.106945	1.339197	5.10E-05
NARF	1.110531	1.029514	1.197923	0.006677
RPTOR	0.959407	0.892042	1.031859	0.264578
FOXK2	0.977456	0.891054	1.072237	0.629174
TRIM65	1.012887	0.941878	1.089249	0.72988
CBX8	0.978267	0.892141	1.072707	0.64028
SECTM1	1.050957	1.002268	1.102012	0.040016
RNF157	1.13759	1.03042	1.255906	0.010663
ZNF750	1.106422	1.003095	1.220392	0.043203
CBX4	0.973449	0.907762	1.043889	0.450281
RNF165	1.148162	1.026505	1.284236	0.015617
DYM	0.919309	0.830505	1.017609	0.104549
ELAC1	1.19699	1.059575	1.352226	0.003852
MBD1	1.00493	0.911876	1.10748	0.920985
SMAD4	0.973495	0.908558	1.043074	0.445669
TNFRSF11.	1.111391	1.032535	1.19627	0.004914
ZCCHC2	1.118263	1.046967	1.194414	0.000883
PMAIP1	0.940734	0.883878	1.001248	0.054763
ERBB2	1.164215	1.032185	1.313133	0.013295
PNMT	0.91146	0.856643	0.969786	0.003396
ARL5C	0.947358	0.838997	1.069714	0.382891
IGFBP4	1.033887	0.982063	1.088446	0.204041
FKBP10	1.192996	1.12205	1.268428	1.69E-08
TXNL4A	1.029451	0.915006	1.158209	0.629292
SAMD1	1.021754	0.941014	1.109422	0.608374
BRD4	1.016326	0.927551	1.113597	0.7284
SLC39A3	1.059462	0.987327	1.136868	0.10839
NFIC	0.823897	0.760729	0.89231	1.94E-06
PRDM15	1.033933	0.948242	1.127367	0.449663
PFKL	1.001042	0.923451	1.085153	0.979812
FEM1A	1.103471	0.991972	1.227503	0.07004
VAV1	1.1795	1.096636	1.268625	8.91E-06
CIB3	1.159204	1.065758	1.260843	0.000571
SH3GL1	0.943956	0.863909	1.03142	0.202065
DUS3L	0.9883	0.90713	1.076734	0.787817

DPP9	0.939714	0.839848	1.051455	0.278059
CCDC97	1.050067	0.959744	1.148891	0.287058
TMEM91	1.144739	1.040771	1.259094	0.005393
ZFP14	0.984039	0.908677	1.065651	0.692248
SIRT3	1.029106	0.934112	1.13376	0.5615
IFITM3	1.003078	0.966579	1.040955	0.870913
COL6A1	1.009786	0.956906	1.065588	0.722714
IFNAR1	1.007647	0.925166	1.097482	0.861209
SOD1	1.159772	1.052358	1.278149	0.002798
COL6A2	0.975476	0.920293	1.033968	0.40333
SIK1	0.956301	0.897035	1.019483	0.171049
TRPM2	1.012303	0.962607	1.064564	0.633992
SCYL1	0.948724	0.862334	1.04377	0.279896
TMEM50B	1.120867	1.022278	1.228963	0.01514
APP	0.937495	0.907334	0.96866	0.00011
URB1	1.039591	0.981238	1.101414	0.187718
AKT1	0.959771	0.884828	1.041061	0.32224
EMP3	0.918464	0.856841	0.984519	0.016383
SAE1	1.173075	1.06029	1.297858	0.001968
NTN5	1.307181	1.133515	1.507454	0.00023
LMTK3	1.248013	1.127496	1.381412	1.90E-05
GEMIN7	1.111413	1.003172	1.231334	0.043328
ADAMTS1	1.104343	1.056366	1.154498	1.19E-05
RNPEPL1	1.071652	0.974231	1.178814	0.154711
CAPN10	1.085155	0.998788	1.178991	0.053446
MYO1F	1.010344	0.950109	1.074399	0.742811
NLRP12	1.073563	1.026393	1.122901	0.00196
ZNF787	1.004095	0.911956	1.105544	0.93368
CARM1	1.038108	0.93904	1.147627	0.464874
EVI5L	1.173693	1.076827	1.279272	0.000268
SLC47A1	1.08464	0.990558	1.187657	0.079253
PSMB6	1.103325	1.011372	1.203639	0.026784
GPR32	0.976811	0.909067	1.049603	0.522297
SIGLEC10	1.081951	1.016526	1.151588	0.013324
ZNF473	1.115592	1.021723	1.218085	0.01472
FAM71E1	1.167922	0.9981	1.366639	0.052838
RPS11	0.92244	0.858504	0.991137	0.027603
PTH2	1.117751	1.033956	1.208337	0.005113
RPL13A	0.870577	0.799045	0.948512	0.001533
NOSIP	1.110464	0.992994	1.241831	0.066252
RCN3	1.047603	0.986335	1.112678	0.130413
ZNF614	1.020166	0.936988	1.110728	0.645442
SLC2A5	1.065664	1.015336	1.118488	0.009979
RERE	0.908556	0.834153	0.989595	0.027814
PRDM16	1.084689	1.042467	1.128621	5.99E-05
PADI3	0.926945	0.841515	1.021047	0.124108
EPHA2	1.100797	1.015859	1.192837	0.019078
ARHGEF19	1.09037	0.998328	1.190897	0.054509
EFHD2	1.021804	0.941298	1.109196	0.606452
PEX14	1.015771	0.913675	1.129276	0.772173
PGD	0.947402	0.874728	1.026114	0.184546
SH3BGRL3	0.999776	0.946253	1.056326	0.993631
CNKSR1	0.969847	0.85084	1.1055	0.646685
RPL11	0.958142	0.907506	1.011603	0.122701
KIAA0319L	1.005006	0.926278	1.090425	0.904507
PLK4	1.05415	0.979025	1.135039	0.162113
MAP3K6	1.053271	0.984371	1.126993	0.132685
GPN2	1.0014	0.921528	1.088196	0.973678

SYTL1	1.022058	0.962394	1.085422	0.477114
WDTC1	0.914567	0.84624	0.98841	0.024182
NBPF3	1.078555	1.008475	1.153505	0.027372
HSPG2	0.925783	0.884397	0.969105	0.00095
ITGB3BP	1.150536	1.047481	1.26373	0.003402
SERBP1	1.049174	0.950515	1.158074	0.340733
BCL10	1.03959	0.948039	1.139982	0.409097
PRKACB	1.047899	0.981126	1.119217	0.16369
PIGK	1.064664	0.994065	1.140278	0.073469
TINAGL1	0.823363	0.745194	0.909732	0.000134
RPS8	0.895063	0.82294	0.973506	0.009698
KIF2C	1.140939	1.062277	1.225426	0.000297
PTPRF	1.142629	1.066869	1.223768	0.000139
BEST4	1.183815	1.056444	1.326542	0.003668
LMO4	1.098399	1.037796	1.16254	0.00119
MTF2	0.957303	0.894227	1.024828	0.209569
IGSF3	1.099344	1.008731	1.198096	0.030924
ZNF697	0.863636	0.80392	0.927788	6.07E-05
CTTNBP2N	1.140445	1.060572	1.226333	0.000389
PSMA5	1.23943	1.100786	1.395536	0.00039
FNDC7	0.97746	0.921333	1.037006	0.449879
CD53	0.883779	0.823673	0.948271	0.000586
CELSR2	1.227274	1.134325	1.327839	3.46E-07
ITGA10	1.028027	0.909373	1.162162	0.658675
GPR161	1.131219	1.004933	1.273374	0.041208
ALDH9A1	1.038128	0.946575	1.138535	0.426976
ATP1B1	0.959664	0.910441	1.011549	0.125393
TIPRL	1.211411	1.090418	1.34583	0.000354
NME7	1.067666	0.974215	1.170082	0.161217
POGK	1.099137	1.010812	1.195181	0.026997
CREG1	0.898492	0.8233	0.98055	0.016375
DCAF6	0.942786	0.875267	1.015513	0.120201
GPA33	0.942427	0.897363	0.989753	0.017694
TBX19	0.988429	0.907481	1.076597	0.78949
UCK2	1.027196	0.958497	1.100818	0.447405
TMCO1	1.151854	1.040246	1.275436	0.006552
XCL1	1.066125	0.983658	1.155506	0.119039
XCL2	1.069064	1.003437	1.138982	0.038816
POU2F1	0.93634	0.867422	1.010733	0.091743
ILDR2	1.061868	0.992484	1.136102	0.081657
MGST3	1.034897	0.935917	1.144344	0.503651
ADCY10	1.068125	0.939764	1.21402	0.31302
UFC1	0.927093	0.83926	1.024118	0.136047
PPOX	1.156349	1.071496	1.247922	0.000187
FCGR2A	0.98653	0.935711	1.040109	0.615267
NUF2	1.148886	1.070567	1.232934	0.000117
RGS5	1.217507	1.033076	1.434863	0.018863
SDHC	1.078877	1.000034	1.163935	0.049896
PFDN2	0.984247	0.904937	1.070507	0.711053
NR1I3	1.097682	1.027539	1.172613	0.005669
USP21	1.056854	0.959919	1.163578	0.259927
PRCC	1.030387	0.922319	1.151117	0.59644
FCRL5	1.000051	0.916251	1.091516	0.999082
MRPL24	1.091829	1.019334	1.169481	0.012203
PIGM	1.192242	1.10736	1.28363	3.07E-06
CASQ1	0.975303	0.91032	1.044924	0.477189
ISG20L2	1.066434	0.960085	1.184563	0.230133
CRABP2	1.022816	0.956733	1.093464	0.507969

HDGF	0.937099	0.850368	1.032675	0.189829
ABL2	0.964533	0.893374	1.041361	0.355752
XPR1	1.078615	0.985198	1.180891	0.101564
RGS16	1.131234	1.083253	1.18134	2.46E-08
TOR1AIP1	0.950161	0.877379	1.028981	0.208634
RGL1	1.047048	0.976591	1.122588	0.195834
LYPLAL1	1.062352	0.973842	1.158906	0.172959
LHX9	0.834743	0.676177	1.030494	0.09286
TUFT1	1.072038	1.009557	1.138387	0.023183
SF3B4	0.969788	0.893233	1.052905	0.464662
ECM1	0.999171	0.950309	1.050546	0.974147
ZNF687	0.972554	0.902665	1.047853	0.464513
TARS2	1.23015	1.114564	1.357724	3.88E-05
SNX27	1.045351	0.977339	1.118096	0.196296
SETDB1	0.983406	0.898038	1.076889	0.717979
ADAMTSL4	0.984116	0.927552	1.044129	0.596004
MCL1	0.944966	0.883991	1.010146	0.096247
CTSK	1.038846	0.95698	1.127715	0.362829
RFX5	0.973389	0.896407	1.056982	0.521112
PI4KB	0.952279	0.87353	1.038127	0.266865
PIP5K1A	0.985291	0.91469	1.061341	0.696083
ANP32E	1.127791	1.040208	1.222749	0.003549
ANXA9	0.970133	0.906146	1.038638	0.383754
SELENBP1	0.989069	0.957085	1.022121	0.512225
ENSA	0.890214	0.790827	1.002093	0.054186
SEMA6C	1.194535	1.114813	1.279959	4.56E-07
MRPL9	1.128106	1.009548	1.260587	0.033363
ARNT	1.022452	0.939586	1.112627	0.606624
POGZ	0.957342	0.879531	1.042037	0.313492
HORMAD1	0.942871	0.873518	1.01773	0.131274
GOLPH3L	1.033246	0.964935	1.106392	0.348683
GABPB2	1.139851	1.047517	1.240324	0.002389
DTL	1.04092	0.97232	1.114361	0.248921
DYRK3	1.034359	0.975757	1.09648	0.256276
INTS7	1.046977	0.985428	1.11237	0.137524
VASH2	1.262328	1.1574	1.376769	1.43E-07
TAF1A	1.100326	1.005383	1.204236	0.037842
SMYD2	0.879318	0.805604	0.959777	0.00399
SUSD4	1.273857	1.110187	1.461657	0.000561
DUSP10	0.921988	0.867334	0.980085	0.009183
TP53BP2	0.856584	0.80155	0.915395	4.90E-06
ATP8B2	1.011804	0.952551	1.074741	0.703112
ADAM15	0.963152	0.895373	1.036061	0.313247
JTB	1.332486	1.196026	1.484515	1.92E-07
RAB13	1.017522	0.943171	1.097733	0.653663
S100A8	1.020772	0.994019	1.048245	0.129201
TPM3	0.944028	0.849637	1.048906	0.283887
NUP210L	0.956718	0.901216	1.015637	0.14675
SNAPIN	1.181326	1.07089	1.303151	0.000876
SLC27A3	1.046403	0.980353	1.116903	0.172725
UBAP2L	0.941711	0.848452	1.04522	0.259011
SLC39A1	0.983738	0.873214	1.108251	0.787442
HAX1	1.04262	0.931283	1.167268	0.468835
CREB3L4	1.053912	0.969006	1.146258	0.220468
EFNA3	1.134289	0.951647	1.351985	0.159522
AQP10	1.132616	1.024489	1.252155	0.014993
GATAD2B	0.962013	0.892431	1.03702	0.312015
ILF2	1.012524	0.916646	1.118432	0.806283

RIT1	0.904667	0.849465	0.963455	0.001815
INTS3	1.073179	1.009132	1.14129	0.02448
PKLR	1.006464	0.955074	1.06062	0.809582
HCN3	1.216733	1.103014	1.342177	8.91E-05
ACTA1	1.111264	0.978539	1.261991	0.104022
GALNT2	0.936073	0.867414	1.010167	0.089189
TTC13	1.089598	1.016025	1.1685	0.016143
SCCPDH	0.883064	0.827164	0.942741	0.000194
LYST	0.998504	0.953385	1.045758	0.949392
CEP170	0.877084	0.819538	0.938671	0.000152
ACP1	1.035483	0.929227	1.153889	0.527914
SNAP47	1.29227	1.170556	1.42664	3.77E-07
SRP9	1.092072	0.993341	1.200616	0.06849
NVL	1.081409	0.991571	1.179386	0.07695
DEGS1	1.230955	1.109448	1.36577	8.90E-05
FBXO28	0.968062	0.878988	1.066164	0.509847
ARF1	0.856627	0.779963	0.940828	0.001216
CNIH4	1.075471	0.987433	1.171358	0.094972
ITPKB	0.979372	0.919801	1.042801	0.515039
GUK1	0.980456	0.880789	1.0914	0.718194
CDC42BPA	1.141013	1.08663	1.198118	1.19E-07
CNIH3	0.918218	0.806282	1.045695	0.198331
MBOAT2	1.076726	1.030258	1.125289	0.001022
PARP1	1.151205	1.06196	1.24795	0.000626
PYCR2	1.212824	1.101372	1.335555	8.74E-05
LBR	0.896946	0.842848	0.954515	0.000611
WNT9A	0.979569	0.894339	1.072922	0.65671
EPHX1	0.947875	0.8806	1.020289	0.154097
REN	1.103983	1.055282	1.154932	1.73E-05
SOX13	0.939841	0.853037	1.035477	0.209528
ETNK2	1.242698	1.107578	1.394301	0.000216
PPFIA4	1.090579	1.013886	1.173074	0.019773
PLEKHA6	1.203264	1.127871	1.283698	2.08E-08
PTPN7	1.106337	1.011824	1.209677	0.026558
SYT2	1.150517	1.058163	1.250931	0.001023
ARL8A	0.852877	0.794793	0.915206	9.77E-06
GDF7	0.988206	0.880506	1.109079	0.840301
PDIA6	1.0258	0.948477	1.109428	0.52409
RHOB	0.877973	0.841184	0.916371	2.54E-09
ATP6V1C2	0.991469	0.903079	1.088512	0.857293
GALM	1.050452	0.993608	1.110548	0.082908
EML4	1.012765	0.95112	1.078406	0.692194
CALM2	1.164084	1.061803	1.276217	0.001204
CHAC2	1.029853	0.956492	1.10884	0.435287
RPS27A	0.882843	0.820405	0.950032	0.00087
VPS54	1.033087	0.950426	1.122937	0.444267
ASXL2	1.032523	0.964953	1.104824	0.354012
ETAA1	1.149803	1.046318	1.263524	0.003721
SNRPG	0.989472	0.881089	1.111187	0.858085
ABHD1	1.30459	1.179243	1.443261	2.48E-07
MEIS1	1.108255	1.074307	1.143275	9.44E-11
CIAO1	1.043695	0.946557	1.1508	0.390876
ZNF514	1.027671	0.956524	1.10411	0.455869
SNRNP200	1.035166	0.955009	1.122052	0.400638
MRPS5	1.023704	0.921964	1.13667	0.660912
ANKRD53	1.157721	1.031262	1.299688	0.013081
TPRKB	1.062536	0.962292	1.173224	0.230245
EXOC6B	1.165697	1.083106	1.254586	4.33E-05

SFXN5	0.981598	0.911332	1.057281	0.624049
TEX261	1.13204	1.022344	1.253508	0.017084
DQX1	1.255994	1.120715	1.407603	8.85E-05
DUSP11	1.06812	0.974641	1.170566	0.15846
ST6GAL2	1.166258	1.10016	1.236327	2.38E-07
THNSL2	0.980453	0.895174	1.073857	0.670704
RALB	1.155487	1.063989	1.254853	0.000596
TMEM177	1.243551	1.138482	1.358317	1.30E-06
RABL2A	1.143269	1.038673	1.258398	0.006237
SLC20A1	1.072893	0.995879	1.155863	0.064127
FBLN7	1.100894	0.972936	1.245682	0.127322
ZC3H8	1.100328	0.986261	1.227587	0.086857
LIPT1	0.993941	0.892935	1.106374	0.911503
FAHD2B	1.277837	1.173842	1.391047	1.51E-08
LYG1	1.043861	0.963298	1.131163	0.294868
AFF3	1.065816	1.010337	1.124342	0.019438
UBXN4	0.965562	0.894403	1.042382	0.369591
SPOPL	0.925878	0.86438	0.991752	0.028081
POLR2D	1.108597	1.011415	1.215116	0.027635
AMMECR1	0.938312	0.864425	1.018516	0.128124
PKP4	0.98149	0.918668	1.048607	0.579844
SCRN3	1.142133	1.043512	1.250075	0.003922
CDCA7	0.926722	0.884639	0.970807	0.00133
DLX1	1.252114	1.158314	1.353509	1.52E-08
UBR3	1.048795	0.979009	1.123554	0.175065
PHOSPHO	1.114208	1.019178	1.218099	0.017425
FAM171B	1.050853	0.96827	1.140478	0.234905
HSPD1	1.038807	0.966504	1.116519	0.300972
CCDC150	1.198156	1.061724	1.352119	0.003379
PTH2R	0.889275	0.84713	0.933516	2.17E-06
NBEAL1	1.131435	1.042718	1.227699	0.003036
SPAG16	1.047523	0.918137	1.195142	0.49005
SUMF1	1.001653	0.92077	1.089641	0.969335
RHBDD1	1.122016	1.029646	1.222673	0.008628
HES6	1.012706	0.961706	1.066412	0.631998
ANKMY1	1.122263	1.0436	1.206856	0.001865
COPS7B	1.054364	0.956288	1.162498	0.287917
DIS3L2	1.054863	0.971153	1.145788	0.205481
CPNE9	1.100503	1.009335	1.199905	0.029965
FANCD2	1.089296	1.016607	1.167183	0.015207
VGLL4	0.888287	0.822139	0.959758	0.002698
RAB5A	0.95868	0.874343	1.051153	0.369105
CTDSP1	1.024034	0.939184	1.116551	0.590454
STK11IP	1.068176	0.983485	1.160159	0.117618
GMPPA	1.187398	1.081165	1.30407	0.000328
EAF1	1.113278	1.025639	1.208406	0.010315
CNTN4	1.050847	0.978755	1.128249	0.171385
DYNC1LI1	0.963553	0.858866	1.081001	0.526937
OSBPL10	1.060357	0.987675	1.138388	0.105736
SLC25A38	1.045772	0.950905	1.150103	0.356308
ITGA9	0.900499	0.866782	0.935527	7.33E-08
GOLGA4	0.946167	0.88818	1.007941	0.086372
CTDSPL	1.087629	1.027031	1.151804	0.004081
STAC	1.271065	1.201732	1.344398	5.24E-17
IQSEC1	1.054603	0.99366	1.119284	0.080024
CAND2	1.255392	1.160589	1.357939	1.37E-08
RPL32	0.886194	0.817058	0.961181	0.003553
PTPRG	1.042286	0.958216	1.133733	0.334424

SHQ1	1.114681	1.03295	1.202879	0.0052
SLC25A26	0.976608	0.874058	1.091191	0.67582
UBA3	1.071217	0.974558	1.177463	0.153913
ARL6IP5	1.129353	1.032254	1.235585	0.008
TMF1	0.971097	0.90358	1.043658	0.425036
LRIG1	1.087856	1.02119	1.158875	0.009059
LIMD1	1.001707	0.939363	1.068189	0.95851
ZNF660	1.002113	0.932547	1.076869	0.954135
NFKBIZ	1.027312	0.963365	1.095505	0.411219
MYH15	1.053197	0.929264	1.193659	0.417117
PHLDB2	1.179085	1.05101	1.322767	0.004985
ABHD10	1.063148	0.976725	1.157218	0.156904
PLA1A	1.152835	0.996857	1.333217	0.055171
RABL3	1.01871	0.928669	1.117483	0.694604
ADPRH	1.053025	0.980085	1.131394	0.158327
ATG3	1.066845	0.955368	1.191329	0.250512
NR1I2	1.117505	1.02781	1.215027	0.009254
SRPRB	1.052335	0.970609	1.140943	0.216189
AGTR1	1.135094	1.049308	1.227894	0.001576
MED12L	1.141011	1.102114	1.181281	9.04E-14
EIF2A	0.838661	0.771766	0.911354	3.35E-05
OSBPL11	0.992805	0.92108	1.070115	0.850289
TRPC1	1.227045	1.138245	1.322772	9.38E-08
LPP	1.068834	0.992427	1.151124	0.078561
TMEM44	0.9434	0.885712	1.004846	0.070326
AMT	1.222417	1.059327	1.410616	0.005981
TCTA	1.140949	1.030837	1.262823	0.010881
UCN2	0.90541	0.825303	0.993292	0.035522
STXBP5L	1.128441	1.024783	1.242585	0.013975
EAF2	1.092231	1.024507	1.164432	0.006907
ILDR1	1.083192	0.969012	1.210825	0.159699
TM4SF19	1.00628	0.870336	1.163458	0.932629
EIF2B5	1.197109	1.076492	1.331242	0.000899
DGKQ	1.13454	1.056475	1.218373	0.00052
FIP1L1	1.002997	0.905304	1.111232	0.954363
SLC26A1	1.059109	0.988799	1.134419	0.101302
LYAR	1.041658	0.960493	1.129681	0.324092
ATP10D	0.976703	0.919449	1.037522	0.444374
OCIAD2	1.015001	0.949722	1.084767	0.660653
SLC10A4	1.089699	0.989527	1.200011	0.080814
SCD5	1.196606	1.124736	1.273067	1.35E-08
PLAC8	1.081802	1.010631	1.157986	0.023542
ENOPH1	1.199808	1.072347	1.342421	0.001478
KLHL8	1.011169	0.930969	1.098277	0.792219
SNCA	1.114142	1.065992	1.164467	1.63E-06
CAMK2D	1.119162	1.06202	1.179378	2.55E-05
CISD2	1.089987	0.986995	1.203726	0.088853
DDIT4L	0.962442	0.916581	1.010597	0.124347
TIFA	1.096719	1.019828	1.179408	0.012797
SPATA5	1.099659	1.028635	1.175587	0.005292
CCNA2	1.113717	1.049316	1.182072	0.000394
METTL14	1.015568	0.927827	1.111605	0.737565
USP53	0.891621	0.829697	0.958167	0.001787
SETD7	1.012462	0.949265	1.079867	0.706453
NAF1	1.031342	0.918063	1.158598	0.603161
SFRP2	0.968591	0.865937	1.083413	0.576623
RPS3A	0.890066	0.818316	0.968107	0.006611
RNF175	0.989084	0.92057	1.062696	0.764417

PDGFC	0.864684	0.828469	0.902483	2.73E-11
CBR4	1.06076	0.98259	1.145149	0.130976
CYP4V2	1.153644	1.080771	1.231431	1.76E-05
ROPN1L	1.096035	1.018847	1.179069	0.013851
NDUFS6	1.076543	0.992446	1.167765	0.075526
NKD2	0.97866	0.927283	1.032883	0.433031
SRD5A1	1.04366	0.947726	1.149305	0.385046
MYO10	1.004659	0.875935	1.1523	0.947021
RPL37	0.926747	0.868145	0.989305	0.022455
SKP2	1.013795	0.922129	1.114573	0.77691
OSMR	0.958868	0.877413	1.047885	0.353765
PLK2	1.005996	0.95581	1.058817	0.818903
GZMA	1.06825	1.018789	1.120111	0.006342
PIK3R1	0.929939	0.874197	0.989235	0.021271
LHFPL2	0.892858	0.843188	0.945453	0.000104
SSBP2	1.041655	0.97923	1.108061	0.195561
IQGAP2	1.014861	0.957769	1.075357	0.617528
CRHBP	0.976338	0.923388	1.032325	0.399952
RASA1	1.018237	0.952975	1.08797	0.592814
LIX1	1.083392	0.941655	1.246464	0.262871
GIN1	1.10907	0.978581	1.25696	0.10503
PAM	0.886839	0.837797	0.938752	3.51E-05
BDP1	0.941244	0.873015	1.014806	0.11476
GTF2H2	1.047801	0.939629	1.168425	0.400971
SLC30A5	1.067294	0.96081	1.18558	0.224573
BTF3	0.863517	0.788999	0.945072	0.001438
FBXL17	0.986576	0.903226	1.077618	0.764105
SPATA9	1.119926	1.008777	1.243323	0.033685
TNFAIP8	0.941548	0.87495	1.013215	0.107571
FEM1C	0.900474	0.845709	0.958786	0.001058
COMMD1(	1.03889	0.966927	1.116208	0.297557
ATG12	1.016644	0.919957	1.123492	0.746139
MEGF10	1.016913	0.955118	1.082706	0.600046
YIPF5	1.036483	0.942145	1.140267	0.461762
ARHGAP2(	0.982488	0.924645	1.04395	0.568229
DDX46	1.013265	0.911108	1.126876	0.807979
TIMD4	0.923899	0.852745	1.00099	0.052897
RNF145	1.018205	0.93981	1.10314	0.658963
FBXO38	0.951395	0.889404	1.017707	0.14723
PCYOX1L	1.131424	1.0575	1.210515	0.000341
TNIP1	0.877776	0.810446	0.9507	0.001367
G3BP1	1.054062	0.97565	1.138777	0.181895
ZNF300	1.145381	1.059698	1.237993	0.000623
N4BP3	1.031199	0.984285	1.08035	0.195939
NHP2	1.111828	1.026126	1.204688	0.009594
RMND5B	1.204182	1.121692	1.292739	2.87E-07
KCNMB1	1.231005	1.126023	1.345774	4.88E-06
FAM50B	1.005113	0.924137	1.093186	0.905264
MYLK4	1.090197	1.020647	1.164488	0.010241
TBC1D7	1.055978	0.957603	1.164458	0.274976
FARS2	1.073125	0.995637	1.156643	0.064947
GFOD1	0.969256	0.912794	1.029212	0.307868
CDKAL1	1.005202	0.940335	1.074543	0.878845
PSD2	1.087675	0.954483	1.239453	0.207314
LRRTM2	1.034081	0.938213	1.139745	0.499589
ZMAT2	1.111615	0.997402	1.238906	0.055757
GFRA3	0.859284	0.758776	0.973106	0.016871
KLHL3	1.26228	1.158478	1.375384	1.04E-07

TRIM7	1.318964	1.195735	1.454894	3.17E-08
TRIM41	1.100725	0.989183	1.224845	0.07833
HIGD2A	0.99071	0.910724	1.077721	0.827969
FAM193B	0.962762	0.894608	1.036109	0.311042
PLA2G7	1.089233	0.993432	1.194273	0.068808
TNFRSF21	0.966505	0.923438	1.011581	0.142951
RNF44	0.861613	0.790256	0.939414	0.000733
DOK3	1.036656	0.962467	1.116563	0.342
ABT1	1.111189	1.000642	1.233948	0.048612
DAAM2	1.164428	1.038205	1.305997	0.009311
PRIM2	1.073049	0.993134	1.159395	0.074182
FGD2	1.052042	1.004967	1.101322	0.029849
SCUBE3	1.008641	0.940456	1.08177	0.809607
ANO7	0.977423	0.929292	1.028047	0.375439
CRIP3	1.216346	1.089454	1.358019	0.000494
TCTE1	1.169497	1.063864	1.285618	0.001188
RPL7L1	1.01042	0.932805	1.094493	0.799333
NFKBIE	0.982343	0.919177	1.049849	0.599324
TPBG	1.099667	1.036811	1.166334	0.001558
IRAK1BP1	1.091519	0.994611	1.19787	0.064884
PHIP	0.971619	0.909453	1.038034	0.393411
PNRC1	0.869085	0.808269	0.934477	0.00015
PM20D2	0.983222	0.916352	1.054972	0.637764
RARS2	1.035472	0.94874	1.130133	0.43481
SCML4	1.031128	0.95755	1.11036	0.417052
RNF217	1.02967	0.974856	1.087565	0.294835
ARHGAP1[illegible]	1.117271	1.043684	1.196047	0.001423
SHPRH	1.01836	0.950705	1.090831	0.60396
AIG1	1.083971	1.017068	1.155274	0.013115
DYNLT1	0.966266	0.890415	1.048579	0.410672
TIAM2	1.071795	0.977835	1.174783	0.138568
TMEM181	0.945865	0.887382	1.008202	0.087432
PNLDC1	1.023771	0.930469	1.12643	0.62991
WTAP	0.988997	0.906889	1.078538	0.802431
ZMYM4	1.001092	0.934907	1.071962	0.975052
VWDE	1.155204	1.077825	1.238138	4.53E-05
GNA12	1.000422	0.933469	1.072177	0.990479
SDK1	1.265421	1.151627	1.39046	9.76E-07
WASH2P	0.997653	0.903629	1.101461	0.962896
RBAK	1.031529	0.951841	1.117888	0.449204
CREB5	1.044944	0.982102	1.111807	0.164751
CDCA5	1.105504	1.034057	1.181887	0.003256
IGFBP3	1.081373	0.977192	1.19666	0.130133
PURB	0.978937	0.902694	1.06162	0.606847
MDH2	0.999392	0.912377	1.094706	0.989565
POMZP3	0.915843	0.839228	0.999451	0.048578
CCT6A	0.985156	0.899567	1.078889	0.747066
PSPH	1.04316	0.965	1.127651	0.28761
ZNF92	0.997713	0.931108	1.069084	0.94822
ATXN7L1	0.997841	0.919637	1.082696	0.958613
TMEM168	1.063516	0.98566	1.147522	0.112378
ASB15	1.001706	0.910218	1.10239	0.972172
SLC12A9	1.037355	0.977769	1.100572	0.224333
GIGYF1	1.019298	0.953005	1.090203	0.577478
TRIM4	1.0107	0.925363	1.103906	0.813064
MEPCE	0.946672	0.867504	1.033064	0.218724
TMEM209	1.236791	1.13168	1.351666	2.74E-06
AGBL3	1.034523	0.896114	1.19431	0.643252

ZC3HAV1L	0.954666	0.903597	1.008622	0.098147
TMEM140	0.965067	0.907299	1.026514	0.25888
TLK2	0.90885	0.844196	0.978456	0.011136
EPHA1	0.990384	0.930467	1.054159	0.761537
NOM1	1.08705	1.000253	1.18138	0.049308
NCAPG2	1.02833	0.963335	1.097709	0.40168
RAB19	1.055345	0.981425	1.134832	0.145971
LUC7L2	0.947527	0.879134	1.02124	0.158505
SH3KBP1	0.909501	0.838425	0.986604	0.022324
LANCL3	1.216918	1.113251	1.33024	1.55E-05
CASK	0.995364	0.90541	1.094255	0.9234
SPIN2A	0.977247	0.890124	1.072897	0.629027
MSN	0.922631	0.849293	1.002302	0.056707
HDAC8	1.028029	0.939721	1.124636	0.546347
SLC16A2	0.966232	0.866752	1.07713	0.535482
ZNF182	0.998787	0.913464	1.092079	0.978746
CHST7	1.008785	0.942711	1.079491	0.800214
NDUFB11	0.963323	0.887507	1.045616	0.371623
ZNF41	1.068322	0.984068	1.15979	0.114843
RAB41	1.095394	0.928874	1.291768	0.278815
ZMYM3	0.984602	0.920583	1.053073	0.650994
TAF1	1.023043	0.95643	1.094296	0.507217
GPR174	0.963526	0.916439	1.013033	0.146102
NONO	0.937573	0.847568	1.037135	0.210622
CCDC120	0.960897	0.878191	1.051391	0.385041
LPAR4	0.932823	0.867436	1.003139	0.060733
EBP	1.300804	1.173954	1.44136	5.07E-07
OGT	0.983544	0.929073	1.041208	0.568128
SNX12	0.998065	0.916668	1.086689	0.964401
ITGB1BP2	1.001808	0.934374	1.074107	0.959488
IL2RG	1.011755	0.941817	1.086886	0.749147
ZNF711	1.031619	0.996626	1.067841	0.077062
DIAPH2	0.950858	0.892446	1.013094	0.119282
NXF3	0.997457	0.954347	1.042514	0.910065
PRPS1	1.140203	1.033731	1.257642	0.00871
DOCK11	0.965355	0.907572	1.026819	0.262885
IGSF1	0.97529	0.920564	1.03327	0.395789
GPC3	1.221789	1.144494	1.304304	1.88E-09
RBMX	0.939412	0.855236	1.031872	0.191922
MCPH1	1.030009	0.931214	1.139285	0.565476
MFHAS1	1.135438	1.064385	1.211235	0.000117
FBXO25	1.126467	1.021692	1.241988	0.016812
NSDHL	1.075791	0.95474	1.212189	0.230332
ZNF185	0.968604	0.927631	1.011388	0.148042
CETN2	1.133915	1.040905	1.235236	0.004001
RPL10	0.844558	0.780984	0.913306	2.33E-05
CSGALNA(	0.985312	0.93207	1.041595	0.601608
ATP6V1B2	0.926429	0.864191	0.993149	0.031262
CCDC25	1.245235	1.137868	1.362732	1.87E-06
HMBOX1	0.969781	0.908112	1.035637	0.359995
CHRNA6	0.845275	0.762111	0.937514	0.001468
GNRH1	1.015606	0.944625	1.09192	0.675284
BIN3	0.984393	0.918542	1.054965	0.656116
DOK2	1.091425	1.020604	1.167162	0.010596
SLC25A37	1.022081	0.972352	1.074353	0.390769
CHMP7	1.030579	0.966988	1.098353	0.353969
DOCK5	0.998751	0.942252	1.058638	0.966453
STAR	0.902645	0.843543	0.965888	0.003032

ERLIN2	1.056942	0.974838	1.14596	0.179502
ST18	0.75084	0.69773	0.807993	1.92E-14
RGS20	0.965644	0.892213	1.045118	0.386289
TACC1	1.010864	0.934766	1.093158	0.786695
GOLGA7	1.116014	0.993005	1.25426	0.065452
GINS4	1.073032	0.988288	1.165042	0.093096
DNAJC5B	1.059634	0.998982	1.123969	0.054092
ADHFE1	1.223799	1.084932	1.38044	0.001014
MRPS28	1.010224	0.909943	1.121557	0.848755
LACTB2	1.213683	1.099298	1.33997	0.000126
TERF1	1.152532	1.049162	1.266087	0.003067
RPL7	0.867267	0.799098	0.941251	0.000651
ATP6V0D2	0.979975	0.820178	1.170906	0.823751
MTDH	0.961662	0.88253	1.047889	0.372247
LRP12	0.924504	0.873595	0.97838	0.006602
EBAG9	1.091086	0.970777	1.226306	0.143627
POLR2K	1.178266	1.055261	1.315608	0.003544
EIF3H	0.849322	0.774625	0.931223	0.000507
UTP23	1.111421	1.008768	1.22452	0.032636
NDUFB9	1.028789	0.92297	1.14674	0.608298
TATDN1	0.934499	0.823238	1.060797	0.294904
FAM83A	1.073381	1.015618	1.13443	0.012106
GSDMC	0.910509	0.823042	1.007271	0.068854
ZNF7	1.137767	1.031046	1.255534	0.010218
SLC39A4	1.118811	1.025567	1.220532	0.011453
VLDLR	0.949942	0.909008	0.992719	0.022304
AK3	1.018985	0.928463	1.118333	0.691942
UHRF2	0.978737	0.912096	1.050248	0.550276
NFIB	1.192496	1.085613	1.309902	0.000238
PLIN2	0.823764	0.78428	0.865237	1.03E-14
HAUS6	0.974387	0.900525	1.054308	0.518864
CDKN2B	1.0577	0.983473	1.137529	0.13077
CDKN2A	1.009405	0.951213	1.071157	0.757334
IFNK	0.949477	0.904658	0.996516	0.035603
ZCCHC7	0.995958	0.927405	1.069578	0.91136
FBXO10	1.118933	0.999984	1.252031	0.050032
SIGMAR1	1.138235	1.07376	1.20658	1.35E-05
CBWD5	0.931604	0.831077	1.044291	0.223958
CEP78	1.207008	1.100397	1.323947	6.67E-05
AUH	0.977626	0.918243	1.04085	0.479121
ZNF462	1.2546	1.145581	1.373995	1.01E-06
UGCG	1.009655	0.957646	1.064489	0.721755
SNX30	0.910501	0.850005	0.975303	0.007521
STOM	0.980474	0.922308	1.042308	0.527401
GSN	1.03259	0.976038	1.092418	0.264433
MRRF	0.940371	0.845479	1.045914	0.25729
NR6A1	1.060835	1.005881	1.118791	0.029553
CRB2	1.021485	0.949205	1.09927	0.57022
ALAD	1.145022	1.062903	1.233485	0.000362
WDR31	1.077883	0.984523	1.180097	0.104692
POLE3	1.095856	0.999769	1.201178	0.050579
SURF4	0.931383	0.862914	1.005285	0.068051
GBGT1	1.063719	0.997265	1.134601	0.060553
SURF1	1.092354	0.964265	1.237458	0.165096
SURF2	1.094409	0.994366	1.204519	0.065118
SURF6	1.104148	1.003352	1.21507	0.042511
MED22	1.046479	0.963949	1.136073	0.278391
REXO4	1.025845	0.936856	1.123286	0.581541

RPL7A	0.851945	0.781405	0.928853	0.000279
GTF3C5	1.1167	1.029726	1.21102	0.00763
ASB6	1.045319	0.959263	1.139096	0.311946
PTGES2	1.15144	1.045714	1.267855	0.00411
CIZ1	0.93145	0.839055	1.034019	0.182755
SLC25A25	0.91161	0.844204	0.984398	0.018217
SH3GLB2	1.190757	1.096155	1.293523	3.57E-05
PTGES	1.091116	1.048038	1.135965	2.21E-05
LCN2	1.007759	0.979167	1.037187	0.598667
LRSAM1	0.998091	0.918728	1.08431	0.963954
GPR107	0.959214	0.891154	1.032472	0.267454
IDI2	0.936168	0.867168	1.010658	0.091302
INPP5E	1.044234	0.951761	1.14569	0.360243
SEC16A	0.976942	0.910191	1.048588	0.518256
NOTCH1	1.078382	1.014397	1.146403	0.015606
NACC2	1.02127	0.970846	1.074313	0.415247
USP6NL	1.054498	0.975921	1.139401	0.179251
COMMD3	1.256673	1.179233	1.339199	1.92E-12
MSRB2	1.123498	1.045663	1.207127	0.001478
PDSS1	1.058277	0.976794	1.146557	0.165867
FAM171A1	0.886888	0.844254	0.931676	1.79E-06
RSU1	1.137179	1.05437	1.226492	0.000861
ST8SIA6	1.123622	1.077097	1.172157	6.58E-08
PARD3	0.940678	0.866166	1.0216	0.146383
ZEB1	0.998306	0.947242	1.052124	0.949546
NRBF2	1.026753	0.938309	1.123533	0.565654
POLR3A	1.097551	1.018734	1.182467	0.014361
HERC4	1.033283	0.962428	1.109354	0.366344
CAMK2G	1.082147	0.999536	1.171586	0.051355
GLUD1	0.956689	0.88082	1.039094	0.293589
ANKRD1	1.231636	1.019679	1.487652	0.030602
HTR7	1.098097	1.047573	1.151057	9.87E-05
RPP30	1.172208	1.057529	1.299324	0.002488
ADD3	1.016359	0.958113	1.078145	0.589961
DNAJB12	1.043037	0.947166	1.148611	0.391695
EIF4EBP2	1.033145	0.942295	1.132754	0.487475
TCF7L2	0.874957	0.820206	0.933362	5.09E-05
MKI67	1.087842	1.03352	1.14502	0.001275
LRRC27	1.150814	1.02459	1.292588	0.017798
MTG1	1.207158	1.083248	1.345242	0.000657
NKX6-2	1.011831	0.876116	1.168569	0.872827
PAOX	1.364163	1.182924	1.573171	1.96E-05
GSTO1	1.002811	0.911203	1.103628	0.954204
TAF5	1.199061	1.09622	1.311549	7.25E-05
PPRC1	1.028048	0.952852	1.109179	0.475367
ITPRIP	1.003237	0.9408	1.069818	0.921469
CNNM2	1.024148	0.951306	1.102568	0.526171
PDCD11	1.057809	0.984758	1.13628	0.123735
RGS10	1.040318	0.98245	1.101594	0.175861
BTBD10	1.082802	0.994627	1.178793	0.066409
ADM	0.977318	0.938474	1.017771	0.267546
GAS2	1.134229	1.010126	1.27358	0.033141
LIN7C	0.994915	0.926392	1.068508	0.888655
IMMP1L	1.072516	0.976476	1.178001	0.14357
TUT1	1.091128	0.99564	1.195774	0.061978
SCGB1A1	1.078369	0.971089	1.197502	0.158176
HSD17B12	0.992139	0.90976	1.081977	0.858372
APIP	1.102555	0.992404	1.224933	0.069067

DGKZ	0.978116	0.905879	1.056115	0.57191
EIF3M	0.96283	0.864647	1.072163	0.490043
TNKS1BP1	0.862879	0.809052	0.920288	7.20E-06
SERPING1	1.02872	0.983812	1.075678	0.213743
SSRP1	1.049404	0.960528	1.146502	0.285509
SLC43A1	1.00905	0.937719	1.085808	0.809667
PTPRJ	0.990934	0.943836	1.040382	0.713931
ARFGAP2	0.941103	0.868131	1.020207	0.140446
CCDC81	1.178469	1.009347	1.375929	0.037739
SESN3	0.975296	0.928099	1.024893	0.322954
ENDOD1	1.056325	0.997426	1.118702	0.061219
CCDC82	0.95164	0.880016	1.029093	0.214375
KLHL35	0.98707	0.910615	1.069943	0.7517
SERPINH1	1.081912	1.019225	1.148456	0.009731
CAPN5	1.388748	1.278992	1.507922	5.37E-15
INTS4	1.003275	0.928314	1.08429	0.934223
PAK1	1.105631	1.038212	1.177428	0.001759
RPS3	0.859329	0.790364	0.934312	0.000383
ZC3H12C	1.141164	1.088713	1.196143	3.79E-08
TTC12	1.145662	1.058196	1.240358	0.000791
NCAM1	1.088856	1.047785	1.131537	1.43E-05
NPAT	1.034548	0.970011	1.103379	0.301381
ATM	1.031957	0.978325	1.088529	0.248004
AASDHPP	1.15358	1.040615	1.278809	0.006586
GLB1L2	1.141149	1.02781	1.266986	0.013364
P4HA3	1.15178	0.97342	1.362821	0.099735
ST14	1.10051	1.04724	1.15649	0.000155
HYOU1	1.006545	0.942665	1.074754	0.84539
MTA2	0.982012	0.90392	1.06685	0.66767
TMEM138	1.144939	1.031535	1.270811	0.010979
FADS1	1.208838	1.129551	1.293691	4.27E-08
ROM1	1.063445	0.934964	1.209582	0.349095
EML3	1.026628	0.944496	1.115902	0.536763
INCENP	1.080785	1.009667	1.156913	0.025288
ZP1	1.017787	0.920521	1.125331	0.730835
MS4A3	0.930654	0.900095	0.962251	2.45E-05
PLCH2	1.040504	0.945655	1.144867	0.415546
MS4A2	0.80769	0.754716	0.864381	6.79E-10
B3GAT3	1.130294	1.023699	1.247988	0.015375
EI24	1.168607	1.068694	1.277861	0.000633
CCDC15	1.144731	1.063347	1.232344	0.000328
CHEK1	1.075762	0.992291	1.166255	0.076367
FEZ1	1.255887	1.173343	1.344238	5.08E-11
ESAM	0.972573	0.926325	1.021129	0.263228
KIRREL3	1.106398	1.041033	1.175867	0.001137
MPZL2	1.320774	1.212452	1.438774	1.86E-10
SIDT2	1.005128	0.936583	1.078689	0.887137
TMEM25	1.06992	0.989747	1.156588	0.089013
TAGLN	1.01206	0.947518	1.080997	0.721431
DUSP15	1.000595	0.887052	1.128672	0.992276
COMMD7	1.271726	1.151478	1.404532	2.10E-06
DSN1	1.147793	1.050917	1.253599	0.002185
LSM14B	0.913156	0.856422	0.973649	0.005504
YTHDF1	1.012055	0.92617	1.105903	0.791138
CABLES2	1.227875	1.121133	1.344779	9.68E-06
GPHA2	0.98607	0.918845	1.058213	0.696993
TRPT1	1.081356	0.953073	1.226905	0.22476
NUDT22	1.181098	1.075001	1.297667	0.000528

FERMT3	1.072662	0.977793	1.176734	0.137638
PLCB3	1.002268	0.933837	1.075713	0.949941
MRPL49	1.058775	0.955685	1.172985	0.274511
FAU	0.924751	0.835767	1.023209	0.129648
TM7SF2	1.09827	1.012996	1.190723	0.023021
TBX6	0.948269	0.863739	1.041072	0.264843
PPP4C	0.979869	0.888374	1.080786	0.684287
ALDOA	1.005446	0.920004	1.098822	0.904597
DOC2A	0.87222	0.792145	0.96039	0.005393
HIRIP3	1.24084	1.136199	1.355119	1.58E-06
TAOK2	1.033291	0.948671	1.125459	0.452519
TMEM219	1.052137	0.941287	1.17604	0.370928
HMGA2	1.239944	1.168289	1.315994	1.43E-12
CNKSR2	0.882724	0.7821	0.996295	0.043376
KLRF1	1.046029	0.983972	1.111999	0.149257
MKX	1.158611	1.045782	1.283614	0.004858
MPP7	1.172306	1.11018	1.237909	1.05E-08
ITGB1	0.914216	0.849986	0.983299	0.015818
CTF1	0.982842	0.888855	1.086767	0.735753
CWC15	1.034871	0.926459	1.15597	0.543796
FCGR1A	0.976836	0.923992	1.032702	0.408835
ARID5B	0.984419	0.931195	1.040684	0.579737
DCUN1D2	0.888407	0.823917	0.957944	0.002088
TMCO3	0.786719	0.72123	0.858154	6.31E-08
TMEM218	1.177123	1.077132	1.286396	0.000318
TIRAP	1.057209	0.955371	1.169903	0.281695
LATS2	0.917159	0.859775	0.978373	0.008711
SAP18	0.905514	0.814581	1.006598	0.066037
KIAA1328	1.120573	1.017389	1.234221	0.020902
FAM124A	1.161622	1.057583	1.275894	0.001751
MIA2	0.941902	0.847001	1.047436	0.269317
HNMT	1.180569	1.11853	1.246049	1.67E-09
PDCD4	0.900808	0.840249	0.965731	0.003261
ADRA2A	1.053507	0.988716	1.122543	0.107498
WDR17	1.205371	1.079523	1.345891	0.0009
CCDC102E	1.248381	1.070579	1.455713	0.004656
CD226	1.045522	0.972077	1.124517	0.230958
FSIP1	1.332371	1.114267	1.593166	0.001654
RGS18	1.012925	0.958241	1.070729	0.650164
PRSS23	1.253247	1.103299	1.423573	0.000517
MTMR12	1.058286	0.963982	1.161815	0.234188
CCT5	1.088361	1.001856	1.182335	0.045086
DOCK1	1.146968	1.100398	1.195509	8.94E-11
DIXDC1	1.043641	0.958153	1.136756	0.327274
DLAT	1.060965	0.971487	1.158685	0.188017
TIMM8B	1.171951	1.075376	1.277199	0.000299
IL18	0.961866	0.904377	1.02301	0.216283
TEX12	1.0155	0.876989	1.175888	0.837117
PTS	1.04036	0.945262	1.145026	0.41852
PIP4K2A	1.117124	1.04617	1.19289	0.000939
FOXO1	0.929736	0.882691	0.979288	0.005961
CRIM1	0.961627	0.907377	1.01912	0.186603
SEC24D	1.008338	0.935671	1.08665	0.827741
ABCB9	1.219068	1.08306	1.372155	0.001031
RILPL2	0.978771	0.883281	1.084585	0.682042
DHX37	1.029883	0.953073	1.112883	0.456524
UBC	0.787665	0.723708	0.857274	3.31E-08
ITPR1	0.994818	0.940693	1.052056	0.855553

SLC7A11	1.03854	0.983359	1.096817	0.174609
CACNA2D	1.057153	1.006819	1.110002	0.025548
DCP1B	1.20519	1.090847	1.331519	0.000243
CACNA1C	0.917907	0.826357	1.0196	0.110069
KCNA6	1.115877	1.031571	1.207074	0.006229
THRB	1.268993	1.1412	1.411096	1.09E-05
NGLY1	0.942151	0.869219	1.021201	0.14717
OXSM	1.016111	0.910096	1.134475	0.776194
UEVLD	1.075797	0.980745	1.180062	0.121619
TMEM86A	1.071641	0.987492	1.162961	0.097256
BTBD11	1.016372	0.966512	1.068805	0.526884
UBE3B	1.113339	1.028821	1.2048	0.007691
ANK3	1.1167	1.009681	1.235062	0.031762
IPMK	0.894433	0.828689	0.965393	0.004181
RAD9B	1.018773	0.928852	1.117399	0.693218
DLG5	1.074757	1.003083	1.151553	0.04062
SLC2A13	1.030833	0.938315	1.132473	0.526777
GXYLT1	0.993491	0.917335	1.075969	0.872496
TWF1	0.969238	0.896889	1.047423	0.429885
DIP2C	0.887656	0.74796	1.053442	0.172552
EIF4E	0.970341	0.891442	1.056222	0.486536
MAGI1	1.148692	1.046318	1.261082	0.003607
CSNK1G3	0.9854	0.913247	1.063254	0.704624
SRFBP1	1.065005	0.973454	1.165166	0.169663
FAM177A1	0.996669	0.882721	1.125326	0.95704
MBIP	0.91884	0.849671	0.993639	0.034026
EXT2	0.917083	0.848863	0.990787	0.028188
TMEM18	1.091705	0.970619	1.227897	0.143524
THRSP	1.105907	0.959594	1.274529	0.164433
NDUFC2	1.211558	1.108052	1.324734	2.53E-05
ME3	0.973237	0.922176	1.027124	0.323825
NUBPL	1.070222	0.973297	1.176798	0.161165
NEK7	1.038402	0.966869	1.115226	0.300775
FER	0.999647	0.930492	1.073941	0.992298
ANKRD50	0.961025	0.902181	1.023706	0.217502
UPF2	0.93305	0.856664	1.016246	0.1118
CDC123	0.913088	0.829849	1.004678	0.062283
SCLT1	1.080245	0.991074	1.17744	0.079092
CCDC3	1.059007	0.983009	1.140879	0.131315
FRMD4A	1.086331	1.008247	1.170462	0.029572
PTPRO	0.948814	0.859561	1.047335	0.29722
EPS8	0.97629	0.917075	1.039329	0.452277
ACAD8	1.136394	1.045282	1.235447	0.002713
THYN1	1.094089	0.990962	1.207949	0.075041
VPS26B	1.144855	1.037694	1.263082	0.006978
NCAPD3	1.123812	1.039784	1.214631	0.003241
VTI1A	0.998082	0.896121	1.111644	0.972144
QDPR	1.122423	1.032333	1.220376	0.006823
FAM160B1	0.943987	0.886417	1.005295	0.07258
TEX9	1.072999	0.933617	1.23319	0.320987
MMAA	1.269928	1.091285	1.477814	0.002006
ZNF827	1.010538	0.955967	1.068224	0.711314
NR3C2	1.102405	1.009084	1.204357	0.030746
AKR1C2	1.196111	1.096022	1.30534	5.91E-05
DPYSL4	1.243008	1.134475	1.361925	3.06E-06
VENTX	1.009083	0.964323	1.05592	0.696085
ADAM8	0.928716	0.883012	0.976786	0.004076
ITIH2	1.008964	0.916148	1.111184	0.856167

KIN	1.035575	0.950226	1.128589	0.425708
PIGF	1.076553	0.988398	1.172571	0.0906
ANKAR	1.021789	0.937642	1.113489	0.623016
INPP1	1.123124	1.018897	1.238012	0.019455
MFSD6	0.995996	0.925344	1.072042	0.914882
RNF144A	0.979035	0.919132	1.042841	0.5107
ASAP2	1.151976	1.088022	1.219688	1.20E-06
ADAM17	0.926391	0.870694	0.985651	0.015657
FLI1	0.989765	0.927155	1.056603	0.757653
KCNJ1	0.98624	0.900329	1.080348	0.765728
TMEM45B	1.033588	0.940634	1.135728	0.492018
WWC2	0.992617	0.934233	1.054649	0.81063
ACSL1	1.029131	0.977188	1.083836	0.277181
SLC25A4	1.214623	1.096798	1.345105	0.000188
AMN1	1.004995	0.927953	1.088434	0.902546
BICD1	1.025585	0.943601	1.114692	0.552306
SAV1	1.04774	0.959499	1.144095	0.29884
CCDC122	1.071411	0.919364	1.248605	0.377065
SERP2	0.889437	0.83348	0.94915	0.000409
NBAS	0.969734	0.915614	1.027052	0.294206
ZNF385D	1.059359	0.952609	1.178071	0.287299
GUF1	1.029548	0.943279	1.123706	0.514287
SACS	0.955612	0.896928	1.018135	0.160272
PABPC3	0.874533	0.795507	0.961409	0.00553
CENPJ	1.026546	0.959796	1.097938	0.445008
FBXO4	1.11386	1.008095	1.23072	0.034145
CCL28	1.111047	1.043581	1.182875	0.000986
PARP8	1.013446	0.964174	1.065236	0.599417
DST	1.051117	0.992169	1.113568	0.090461
BEND6	1.127803	1.041913	1.220773	0.002922
TIAL1	1.040193	0.9468	1.142797	0.411645
BAG3	1.150893	1.098991	1.205247	2.39E-09
GLT1D1	1.00886	0.967396	1.0521	0.680389
SCHIP1	1.130042	1.054958	1.210471	0.000492
AP1S3	1.150292	1.081481	1.223481	8.63E-06
RABGAP1L	1.047001	0.971186	1.128734	0.231066
TUBA3E	1.071018	0.924719	1.240463	0.3599
FAM168B	0.90765	0.847926	0.971581	0.005268
PTPN14	0.916473	0.871281	0.964009	0.000723
MGAT5	0.975751	0.91626	1.039105	0.444379
TMEM163	1.236804	1.14965	1.330565	1.19E-08
GEMIN6	1.224929	1.114873	1.345849	2.40E-05
POU4F1	0.909052	0.875562	0.943823	6.39E-07
CYSLTR2	0.973272	0.920038	1.029586	0.345168
ARL11	0.979457	0.918319	1.044666	0.527917
SETBP1	1.05448	0.985291	1.128527	0.125517
PSTPIP2	1.133598	1.057461	1.215217	0.000408
SPC25	1.172247	1.093204	1.257005	8.12E-06
PDK1	0.8941	0.841079	0.950464	0.000332
PDE3B	0.869636	0.822565	0.9194	8.67E-07
TCF7L1	0.963454	0.88282	1.051453	0.403789
TGOLN2	0.927566	0.85037	1.011769	0.089879
KCNK13	1.058815	0.929831	1.20569	0.388535
UHMK1	1.030034	0.957809	1.107705	0.424985
ATG10	1.041791	0.952215	1.139793	0.372111
SPOCK1	1.246779	1.170345	1.328205	8.31E-12
FAM151B	1.04	0.962144	1.124156	0.323198
CWF19L2	0.979384	0.895984	1.070547	0.646422

JMY	0.982346	0.934262	1.032905	0.486671
HOMER1	0.966528	0.889031	1.050781	0.424654
XRCC4	1.068033	0.984128	1.159093	0.114865
ZNF547	1.279037	1.122122	1.457894	0.000228
ZNF773	1.074327	0.967696	1.192708	0.178863
ZNF776	1.016911	0.950646	1.087794	0.625716
ZNF256	0.975411	0.922676	1.03116	0.379977
SUV39H2	1.045247	0.962862	1.13468	0.290752
DCLRE1C	0.98148	0.896644	1.074343	0.685269
OLAH	1.059449	0.963054	1.165492	0.235424
RPP38	1.188771	1.05637	1.337767	0.004102
NMT2	0.865011	0.808212	0.925802	2.85E-05
ZNF837	0.989086	0.897648	1.089838	0.824517
USP12	0.880848	0.813477	0.953799	0.001777
CCDC50	0.96787	0.923892	1.013942	0.168697
CAMK4	1.000424	0.946343	1.057595	0.988078
TRIM36	0.979055	0.870447	1.101215	0.72421
ZFP36L2	0.989869	0.923331	1.061202	0.774255
PAN3	0.982962	0.922342	1.047566	0.59672
PFKM	1.094378	1.022888	1.170865	0.008883
TMEM123	0.99879	0.920273	1.084005	0.976871
IGSF10	0.998206	0.95712	1.041056	0.933264
SPEF2	1.009467	0.918312	1.109671	0.845282
MBNL1	1.119232	1.032822	1.212872	0.006001
GPD1L	1.069694	1.001676	1.14233	0.044439
GJA1	0.994931	0.932722	1.061289	0.877398
SLC30A6	1.058637	0.985781	1.136879	0.117275
PELO	1.253497	1.143896	1.3736	1.30E-06
RASGRP3	0.989332	0.936545	1.045093	0.701434
SAR1B	1.029801	0.945658	1.121431	0.499537
CATSPER3	1.225986	1.06365	1.413099	0.004932
GPR180	1.196485	1.088421	1.315277	0.000204
TCTEX1D1	1.066463	1.019614	1.115465	0.004994
ANKRD22	0.95863	0.896752	1.024777	0.214594
FARP1	1.019479	0.891163	1.166271	0.778646
IFIT5	1.077365	1.01353	1.145221	0.016792
PANK1	1.127615	1.028074	1.236795	0.010861
PRDM8	1.047552	0.995446	1.102387	0.074324
BMP3	0.952392	0.895339	1.013081	0.121711
HHEX	0.977121	0.909791	1.049435	0.525193
UTRN	0.979228	0.929537	1.031575	0.429533
PTPRK	1.194063	1.093774	1.303548	7.42E-05
GGPS1	1.076884	0.967365	1.198801	0.175855
ZNF117	0.964638	0.899506	1.034487	0.312796
MARVELD	1.117015	0.970674	1.28542	0.122462
RAD17	1.036215	0.942905	1.138758	0.459976
MED21	0.956463	0.893352	1.024032	0.201212
PLOD2	1.176608	1.102439	1.255766	9.80E-07
STK32B	0.851682	0.809649	0.895896	5.06E-10
CPB1	1.141591	1.031239	1.263753	0.01068
MR1	1.146089	1.052119	1.248451	0.001784
SRP19	0.985855	0.897872	1.082459	0.765173
CENPH	1.116724	1.014034	1.229813	0.024889
CDYL	0.935426	0.869501	1.006349	0.073416
CARHSP1	1.066676	0.97489	1.167104	0.159722
BANK1	1.019704	0.965642	1.076793	0.482647
TXNDC11	0.916584	0.851053	0.987162	0.021371
DAB2	0.966022	0.90526	1.030863	0.296985

ACMSD	0.998674	0.873555	1.141714	0.984498
BCL2L11	1.074307	1.01519	1.136867	0.013065
ANAPC1	1.019492	0.946435	1.098188	0.610873
CAST	1.060112	0.989979	1.135213	0.09461
SCOC	1.094317	0.989803	1.209867	0.078433
CLGN	1.1796	1.089315	1.277369	4.79E-05
CETN3	1.113673	0.998795	1.241764	0.052593
SMARCA5	0.992432	0.920671	1.069785	0.842737
SYCP2L	0.988487	0.92961	1.051093	0.711688
BMP6	0.919607	0.855227	0.988833	0.023623
RGPD3	0.832557	0.685186	1.011626	0.06523
RASSF3	0.98149	0.920459	1.046568	0.568409
HNRNPU	0.981881	0.90116	1.069833	0.676133
RANBP2	0.935213	0.886299	0.986826	0.014532
AHCTF1	0.929571	0.877031	0.985258	0.013883
MERTK	0.964632	0.910516	1.021964	0.221549
TMEM87B	1.112542	1.027093	1.205099	0.008907
NR4A2	0.974072	0.923599	1.027302	0.333184
RBMS1	0.916454	0.839967	0.999907	0.049755
SCN3A	1.03217	0.927692	1.148415	0.560889
CD96	0.927698	0.894194	0.962458	6.37E-05
ASAP1	0.956936	0.893388	1.025003	0.209273
FAM81B	1.103815	1.021599	1.192646	0.012382
INO80C	0.948676	0.863706	1.042005	0.271109
LPCAT1	1.033415	0.973751	1.096735	0.278676
NMRAL1	1.08857	0.990646	1.196175	0.07764
ING1	0.918884	0.825731	1.022546	0.120865
ADPRHL1	0.86516	0.818521	0.914456	3.01E-07
CMTM7	0.896577	0.837763	0.95952	0.001612
FBXL2	1.033831	0.941339	1.135411	0.486568
UBP1	1.028617	0.947444	1.116744	0.501119
RMND5A	1.030328	0.953528	1.113313	0.449682
CD8A	0.981915	0.927465	1.039563	0.53067
RPIA	1.073462	0.986177	1.168472	0.101362
PTPRD	1.121226	1.036247	1.213174	0.004436
CNKSR3	1.098939	0.982827	1.22877	0.097737
GTF2E1	1.037637	0.954325	1.128223	0.386941
CFDP1	1.04583	0.926162	1.180961	0.469825
ZDHHC7	0.936295	0.868179	1.009755	0.087627
JAZF1	1.004307	0.947431	1.064597	0.885114
CMIP	0.917722	0.848044	0.993126	0.033075
PID1	1.188929	1.120181	1.261897	1.24E-08
TRIP12	0.966847	0.903561	1.034565	0.329003
FBXO36	1.163021	1.02274	1.322544	0.021289
CEBPG	0.98211	0.903325	1.067768	0.672223
KCTD15	0.925513	0.874363	0.979656	0.007618
ZNF599	1.197797	1.053741	1.361548	0.005769
MCOLN2	1.089156	1.044231	1.136013	7.07E-05
LGI4	0.96426	0.810403	1.147327	0.681547
DDAH1	1.107313	1.016059	1.206763	0.020178
CHD1	0.912654	0.861972	0.966317	0.001717
DGKE	1.132176	1.054366	1.215729	0.000633
HS2ST1	1.047203	0.9725	1.127645	0.221906
MSI2	1.08095	1.027181	1.137534	0.002788
HS3ST3A1	0.974615	0.854139	1.112084	0.702509
GDPD1	1.099007	1.013405	1.191841	0.0225
NUS1	0.989916	0.904477	1.083426	0.825818
PPP2R5E	0.966441	0.88058	1.060674	0.472089

GRAP	0.907002	0.822732	0.999905	0.049776
SLC5A10	0.869127	0.804952	0.938419	0.000338
AK5	0.91472	0.863318	0.969182	0.002521
CABYR	1.019197	0.943961	1.10043	0.626971
IMPACT	1.01097	0.941035	1.086103	0.765471
TBCEL	1.098837	1.026872	1.175845	0.006386
JPH3	1.091298	0.952505	1.250315	0.208084
ANKH	0.988593	0.923814	1.057914	0.740045
UBASH3B	0.959413	0.908184	1.013532	0.138902
ROBO4	0.951598	0.888128	1.019604	0.15892
ROBO3	1.124802	1.052818	1.201708	0.000492
TBRG1	1.051309	0.947806	1.166116	0.344029
NRGN	1.044897	0.998558	1.093387	0.057745
GPR15	1.035611	0.936996	1.144604	0.493122
ABI3BP	0.995508	0.894311	1.108155	0.934392
ANGPT1	1.042228	1.004014	1.081896	0.029996
PITPNC1	1.008802	0.930133	1.094126	0.832453
CC2D1B	1.01836	0.944461	1.098041	0.63597
PRKCA	1.027457	0.976905	1.080624	0.292683
LRRK1	0.870766	0.818978	0.925828	9.71E-06
GAL3ST2	0.992561	0.947913	1.039311	0.750494
ABCA5	1.295502	1.216601	1.379521	6.73E-16
ENPP3	0.830634	0.77247	0.893178	5.44E-07
UCHL1	1.152182	1.045712	1.269492	0.00419
MIA3	0.920361	0.846362	1.000831	0.052313
DISP1	1.053232	0.937863	1.182792	0.380927
TNIK	1.005206	0.951295	1.062171	0.853535
TDH	1.176094	1.064219	1.299729	0.001471
FAM167A	1.082608	0.997713	1.174726	0.056779
NEIL2	1.077338	0.97652	1.188565	0.137282
OBSCN	0.995992	0.934545	1.06148	0.901636
LONRF1	0.927676	0.866884	0.992731	0.029936
TRIM11	1.080783	1.0041	1.163323	0.038553
ENAH	1.218883	1.134092	1.310013	7.43E-08
SH3RF1	0.999352	0.938985	1.063599	0.983721
GBP5	1.02604	0.972888	1.082095	0.343537
BUB3	1.284422	1.181592	1.396202	4.12E-09
MMP21	1.047261	0.960024	1.142425	0.298059
LY96	1.008502	0.95486	1.065157	0.76145
TMSB4Y	1.074356	1.00855	1.144456	0.026152
CXADR	1.11639	1.038277	1.200379	0.002931
BTG3	0.948971	0.89964	1.001007	0.054477
NCAM2	1.158777	1.062562	1.263704	0.000862
L3MBTL4	1.062091	0.990851	1.138452	0.089034
RABGEF1	0.884094	0.818136	0.955371	0.001845
MRPL39	1.063473	0.968497	1.167764	0.197285
GABPA	0.985784	0.91005	1.067821	0.725551
ADAMTS1	1.02926	0.951723	1.113114	0.470471
TSEN2	1.069006	0.996653	1.146613	0.062014
SLFN13	1.062075	1.001858	1.125912	0.043147
XPC	1.05851	0.967637	1.157917	0.214379
FGD5	1.06702	1.012231	1.124774	0.015867
FLCN	1.114394	1.034094	1.20093	0.004531
DPH3	0.982444	0.890066	1.084411	0.725183
OXNAD1	1.017237	0.953048	1.085748	0.607326
PLCL2	0.985321	0.927686	1.046537	0.630623
CXXC1	1.188554	1.072884	1.316694	0.000944
SKA1	1.167796	1.093375	1.247282	3.89E-06

PPP4R1	0.945147	0.864246	1.033621	0.216581
CCDC144E	1.065447	1.010211	1.123702	0.019595
MPPE1	1.099579	1.001201	1.207623	0.047137
USP43	1.019498	0.940817	1.10476	0.637473
RAB6B	0.964203	0.905965	1.026185	0.251463
EME1	1.072195	0.99126	1.159738	0.081729
EPHB1	1.266453	1.131616	1.417357	3.91E-05
ACSS1	0.970144	0.908999	1.035402	0.36147
ANKRD40	1.022406	0.92651	1.128227	0.659238
ZNF18	1.088473	0.986486	1.201002	0.091237
VOPP1	1.07472	1.020244	1.132104	0.006626
APOOL	1.01777	0.931223	1.11236	0.697681
CYP2U1	1.206997	1.09237	1.333651	0.00022
FBXL18	1.028521	0.941223	1.123916	0.534327
KLF10	1.001006	0.94225	1.063425	0.97402
PTPRN2	1.056609	0.994572	1.122515	0.074477
AZIN1	1.013177	0.936669	1.095934	0.743841
ATP6V1C1	1.052483	0.982504	1.127446	0.145074
OTUD6B	1.061576	0.978108	1.152168	0.152668
GTF3C6	1.126688	1.029063	1.233575	0.009895
TTC39B	1.196475	1.109572	1.290184	3.12E-06
AGPAT5	0.993593	0.928081	1.06373	0.853477
MMS19	1.003321	0.921339	1.092598	0.939225
PI4K2A	1.057321	0.999094	1.118941	0.05378
MARVELD	1.05862	1.013249	1.106023	0.010807
ZFYVE27	0.964393	0.897744	1.035989	0.321052
GOLGA7B	1.232992	1.066728	1.425172	0.004597
SLC25A28	1.211561	1.10761	1.325268	2.75E-05
HSPA13	0.98563	0.911371	1.065939	0.717218
SAMSN1	0.917079	0.864387	0.972983	0.004142
USP25	0.973113	0.905615	1.045642	0.457423
ZCCHC10	1.082015	0.974915	1.200881	0.138276
MOV10	0.961185	0.893542	1.033948	0.287642
RHOC	0.976683	0.914218	1.043415	0.484141
PPM1J	0.959851	0.851917	1.08146	0.500774
DBI	0.970682	0.915208	1.029519	0.321657
SLC16A1	1.036836	0.982948	1.093678	0.184049
HEATR3	1.094086	1.021514	1.171814	0.010234
TRIM74	0.965048	0.906144	1.027781	0.268213
OXA1L	0.989672	0.879508	1.113635	0.863109
SLC7A7	0.950467	0.904089	0.999223	0.046546
LARP1	1.007972	0.937027	1.084288	0.831143
CNOT8	0.959367	0.882493	1.042937	0.330343
LRGUK	1.014893	0.919184	1.120568	0.769893
MIER3	0.959592	0.896681	1.026916	0.233167
NUP205	1.009293	0.941897	1.081511	0.793065
ZKSCAN2	0.942601	0.863463	1.028993	0.186442
PIK3AP1	1.034329	0.970204	1.102692	0.301307
RBM45	1.285123	1.151455	1.434309	7.58E-06
TTN	0.97954	0.925016	1.037278	0.479291
VSIG4	0.946518	0.907149	0.987596	0.011219
PDIA4	1.025114	0.962639	1.091644	0.439446
OTOA	1.322274	1.175774	1.487028	3.12E-06
KCTD18	1.080621	0.979492	1.192192	0.121952
FAM126B	0.985371	0.917402	1.058376	0.686115
FZD7	1.055022	0.982144	1.133308	0.142477
RNF20	1.062992	0.969997	1.164903	0.190939
PPARGC1E	1.034968	0.970841	1.103331	0.292257

ELMO1	0.898199	0.835537	0.96556	0.003616
SLC26A2	1.003048	0.944548	1.06517	0.920939
LSM11	1.03969	0.954336	1.132677	0.373172
MED7	1.044307	0.947191	1.15138	0.384011
RRAGA	1.01778	0.91402	1.133317	0.748032
RASA2	1.081506	1.009454	1.158701	0.025916
RMND1	1.033319	0.945828	1.128903	0.467767
SLA	1.076657	1.023996	1.132026	0.003893
TMBIM4	1.095547	1.007635	1.191128	0.032502
VBP1	1.050449	0.956264	1.15391	0.304474
RAB39B	1.163388	1.062613	1.273721	0.001062
CLIC2	1.08524	1.033373	1.13971	0.001061
AFF2	0.901799	0.863402	0.941903	3.22E-06
GRIP1	1.080018	1.004147	1.161622	0.038329
VPS37A	0.898774	0.83271	0.970079	0.006147
PSD3	0.951944	0.899526	1.007416	0.088331
GNA14	1.113607	1.046517	1.184998	0.000689
FAM161B	0.959813	0.861657	1.06915	0.456154
GNAQ	0.945723	0.878982	1.017531	0.135035
GPR61	1.003853	0.918264	1.09742	0.932592
ADK	1.028309	0.93695	1.128576	0.556492
BATF	1.313822	1.218842	1.416204	1.01E-12
DCK	1.045115	0.959608	1.138241	0.310949
ADAMTS3	0.936215	0.883309	0.99229	0.026369
ALX3	1.276781	1.166508	1.397479	1.15E-07
DPY19L4	1.029209	0.953967	1.110384	0.457306
ART3	0.978404	0.868294	1.102478	0.720043
WHAMM	0.949639	0.894397	1.008292	0.091048
CXCL13	1.111554	1.019466	1.211962	0.016535
N6AMT1	1.121014	1.032258	1.217403	0.006641
RWDD2B	1.191702	1.087657	1.3057	0.000168
USP16	0.93766	0.873373	1.006679	0.075689
CCT8	1.053453	0.958833	1.157411	0.278155
BACH1	0.9113	0.855132	0.971157	0.004214
TSPAN7	0.878105	0.832746	0.925934	1.56E-06
TIAM1	1.111813	1.052311	1.174679	0.000159
RPGR	0.972018	0.903114	1.046179	0.449315
PCGF6	1.103086	1.002424	1.213858	0.044479
ANKRD9	1.086409	1.024447	1.152118	0.005673
SFXN2	1.126585	1.040571	1.219709	0.003267
FUT6	1.115068	1.000414	1.242861	0.049132
TDRD9	1.012066	0.972272	1.053489	0.557868
FGF18	0.991038	0.875464	1.121869	0.886846
PCDH1	1.136649	1.000843	1.290883	0.048502
UQCRB	1.039575	0.928096	1.164445	0.502459
PTDSS1	0.959673	0.884084	1.041724	0.325415
RPL30	0.955059	0.89568	1.018374	0.160314
FAM122C	1.132326	1.014492	1.263848	0.026651
SUPV3L1	0.95087	0.877745	1.030088	0.217242
FAM122B	1.127832	1.027704	1.237715	0.01121
EEF1A1	0.821242	0.759607	0.887878	7.52E-07
FBXO43	1.181835	1.07718	1.296657	0.000413
HK1	1.027082	0.959591	1.099319	0.440978
TYSND1	1.117661	1.036368	1.20533	0.003888
PHF6	1.023885	0.951533	1.101739	0.527858
CD109	1.12407	1.079447	1.170538	1.52E-08
NODAL	1.034997	0.94954	1.128145	0.43401
PRG3	0.948136	0.922078	0.97493	0.00018

UBE2L6	1.10802	1.023176	1.199898	0.011614
ZDHHC5	1.012648	0.918863	1.116004	0.799909
MED19	1.012315	0.917002	1.117534	0.808319
ZFAND3	0.920354	0.855425	0.990212	0.026184
NPTN	0.955242	0.875535	1.042206	0.302992
SAMD8	0.990991	0.92252	1.064544	0.804328
RAB11FIP1	0.948326	0.891629	1.008628	0.091635
GLYATL2	1.21277	1.083466	1.357505	0.000798
UTP14A	1.001652	0.898714	1.116381	0.976198
AIFM1	1.120512	1.010375	1.242655	0.031124
MAPK13	1.046963	0.982374	1.115798	0.157772
BAG4	1.046089	0.958752	1.141381	0.311071
MS4A1	0.969191	0.920338	1.020636	0.23566
ATAD2	1.007652	0.942432	1.077385	0.823325
FBXO32	1.082723	1.015553	1.154337	0.015005
NSMCE2	1.059953	0.957219	1.173714	0.26298
ZNF689	1.082379	0.98135	1.193808	0.113329
PRR14	0.890141	0.801871	0.988127	0.028953
FBRS	0.869714	0.812652	0.930783	5.54E-05
FRRS1	1.03377	0.970032	1.101696	0.306364
PHKG2	1.046345	0.935818	1.169926	0.426399
SASS6	1.052227	0.973846	1.136917	0.197413
ITGAD	1.174708	1.042364	1.323855	0.008283
VPS8	1.070048	1.002202	1.142487	0.042788
GALK2	1.007976	0.943879	1.076426	0.812666
B3GNT7	1.071264	1.030644	1.113485	0.000482
MPV17L	1.302312	1.226019	1.383352	9.84E-18
BUB1B	1.057896	0.992977	1.127061	0.081537
PDE6D	1.164978	1.053011	1.28885	0.003058
EIF4A2	0.972843	0.895469	1.056902	0.51495
BRPF1	1.067963	0.989883	1.152202	0.08961
RPUSD3	1.033912	0.949449	1.12589	0.443093
TATDN2	1.02421	0.930192	1.127731	0.6263
GHRL	1.025661	0.963309	1.09205	0.428476
SEC13	0.978391	0.876254	1.092434	0.697758
EXOG	1.18824	1.095068	1.28934	3.48E-05
NTAN1	0.921401	0.836003	1.015522	0.099029
ZFYVE9	0.901319	0.84116	0.965781	0.0032
SMG1	0.958171	0.9037	1.015926	0.152473
FCHO2	0.908235	0.859025	0.960263	0.000708
RBPMS	0.930759	0.880701	0.983661	0.010958
TIMP4	1.113649	1.029226	1.204997	0.007448
CPT2	1.206168	1.065359	1.365589	0.003081
NECAP2	1.072672	0.971796	1.184019	0.163859
LRP8	1.111788	1.032697	1.196936	0.004885
PAXIP1	1.160802	1.047011	1.28696	0.004616
SSBP3	1.039809	0.955374	1.131705	0.3663
CLDN12	0.945032	0.862121	1.035917	0.22752
MMP14	0.991611	0.956119	1.028421	0.650552
FZD1	1.224597	1.141959	1.313216	1.32E-08
GATAD1	1.013399	0.935238	1.098093	0.745167
SUSD3	1.127569	1.0688	1.189568	1.10E-05
TMED6	0.962815	0.879053	1.054559	0.414494
DHRS4	1.131713	1.025158	1.249344	0.014189
DDX19B	0.886271	0.802157	0.979205	0.017645
ST3GAL2	0.996124	0.926899	1.070518	0.91583
IL34	1.05324	0.93036	1.19235	0.412488
DHRS1	1.00164	0.90296	1.111103	0.975301

CACNA1D	0.950993	0.840421	1.076114	0.425581
KIT	0.94544	0.911211	0.980956	0.002864
AASDH	1.038698	0.948099	1.137953	0.414851
ZNF19	1.230657	1.046098	1.447776	0.012294
CACNA2D	0.998236	0.938669	1.061584	0.955159
RNF111	0.956867	0.887407	1.031764	0.251501
CCNB2	1.104422	1.028821	1.185579	0.006045
FAM81A	1.118237	1.015967	1.230801	0.022391
MYO1E	1.203907	1.123924	1.289582	1.22E-07
APPL1	0.886818	0.826028	0.952083	0.000916
AFAP1L1	0.886146	0.802022	0.979092	0.017542
TSC22D3	1.047991	0.989266	1.110202	0.111122
DYRK1A	0.99136	0.924516	1.063037	0.807508
KCNJ15	1.126107	1.031989	1.22881	0.007652
ERG	0.995233	0.951251	1.041247	0.835833
ETS2	1.124091	1.054043	1.198793	0.000366
TSPAN18	1.140585	1.055956	1.231995	0.000825
LCA5L	0.997939	0.873931	1.139544	0.975689
SLC35B2	0.976997	0.903738	1.056194	0.55842
TMEM164	1.028707	0.959905	1.10244	0.422927
MX1	1.078528	1.032347	1.126776	0.00071
C2CD2	0.941434	0.881149	1.005844	0.073873
SLC38A10	1.081567	1.020374	1.14643	0.008322
ZNF618	1.033631	0.966882	1.104988	0.331468
SVOPL	0.973608	0.871935	1.087138	0.634582
SNX22	1.034201	0.958349	1.116058	0.386876
UBN2	1.007424	0.938494	1.081417	0.837924
BRAF	0.950723	0.887197	1.018798	0.1521
PSMG3	1.291478	1.168185	1.427785	5.84E-07
WDR19	1.015754	0.940226	1.09735	0.691728
SLC37A3	1.119053	1.035081	1.209839	0.004709
AP3S2	1.016627	0.928821	1.112733	0.720497
FMNL2	0.96492	0.893909	1.041572	0.359865
SPPL3	0.890605	0.814448	0.973883	0.011078
RAB28	1.103112	0.997361	1.220077	0.056318
TNFRSF14	1.015205	0.942811	1.093157	0.689309
PANK4	1.03428	0.960838	1.113335	0.369773
MEGF11	1.064512	0.990308	1.144277	0.089926
PEX10	1.037688	0.942504	1.142484	0.451057
RER1	1.19515	1.068738	1.336513	0.001775
RADIL	1.012734	0.932072	1.100376	0.765087
SKI	1.009837	0.950007	1.073435	0.753415
WIPI2	0.922768	0.848347	1.003717	0.061002
LDLRAP1	1.149912	1.07501	1.230033	4.81E-05
AGAP1	0.98893	0.89837	1.08862	0.820299
KRTCAP3	1.021669	0.95599	1.091861	0.527158
PAFAH2	0.8939	0.824481	0.969163	0.006541
MRPL17	1.235359	1.11341	1.370665	6.73E-05
DUSP2	0.947984	0.899208	0.999406	0.047478
UBXN11	1.018943	0.946669	1.096734	0.617132
PTPDC1	1.154101	1.054702	1.262867	0.001815
GALNT14	0.911451	0.864969	0.960431	0.000517
NCK1	0.912575	0.832714	1.000096	0.05024
HPD	1.078861	0.990848	1.174692	0.080426
RHPN1	1.053851	1.003364	1.106878	0.036256
TPRG1L	1.083492	1.001587	1.172096	0.045555
LRRC43	0.914179	0.840201	0.994671	0.037154
XKR8	0.9845	0.907229	1.068352	0.707975

CNNM4	0.930811	0.859042	1.008576	0.079881
EYA3	1.052773	0.978701	1.132451	0.167098
DZIP1L	1.221602	1.139542	1.309571	1.68E-08
TMSB15A	1.251589	1.170174	1.338668	6.18E-11
FANCC	1.151154	1.047215	1.265411	0.003552
MRAS	1.116894	1.05512	1.182285	0.00014
WASF2	0.861074	0.787111	0.941986	0.001098
ABHD3	0.96716	0.893448	1.046955	0.409071
FAIM	1.066259	0.984364	1.154966	0.115613
CLSTN2	1.10828	1.071012	1.146845	3.84E-09
COLEC12	0.81044	0.707435	0.928441	0.002441
RNF207	1.187684	1.071475	1.316498	0.00106
CUL4B	0.974033	0.89425	1.060934	0.546239
GPR153	0.911565	0.867967	0.957352	0.000213
SLC13A3	1.19719	1.096633	1.306968	5.80E-05
GPRASP2	1.305285	1.168988	1.457473	2.19E-06
RHBDL2	1.14094	0.998005	1.304345	0.053517
AUTS2	0.944993	0.882463	1.011953	0.105278
SHROOM4	1.113753	1.039128	1.193737	0.002329
CDC25C	1.082849	1.000637	1.171815	0.048181
MITD1	1.140712	1.030782	1.262366	0.010885
EIF5B	0.935397	0.847386	1.03255	0.185291
RIBC1	1.126082	0.980153	1.293736	0.093567
TSPAN33	0.91849	0.866073	0.974079	0.004569
AHCYL2	1.007825	0.936395	1.084703	0.83538
B4GALT5	1.014764	0.951272	1.082495	0.656617
CD1D	1.092118	1.047042	1.139133	4.17E-05
CD1A	1.108372	0.980269	1.253215	0.100599
SPATA2	0.87519	0.8069	0.949259	0.001299
CD1C	0.921271	0.871494	0.973891	0.00381
CD1E	0.854173	0.761266	0.958419	0.0073
HMHB1	1.121443	1.004544	1.251946	0.041282
NCF1	1.038924	0.993487	1.086439	0.094213
TSR2	1.033609	0.925583	1.154244	0.557251
PPP1R9A	1.152065	1.074244	1.235522	7.28E-05
ZC3H18	0.976734	0.890605	1.071193	0.617213
ZFAND2B	1.299355	1.172848	1.439507	5.43E-07
GDPD5	1.114105	1.040833	1.192536	0.001852
DYNC1I1	1.093442	1.023516	1.168146	0.008066
PFKFB1	1.246525	1.05864	1.467754	0.008203
ALAS2	0.980412	0.954859	1.006649	0.142061
TMED4	1.10669	0.990687	1.236275	0.072757
PPP1R15B	0.992315	0.923267	1.066527	0.833939
COPG2	0.984821	0.924573	1.048996	0.634878
ZSCAN12	0.915212	0.845755	0.990372	0.027792
TAGLN2	1.170226	1.079494	1.268585	0.000135
ELK4	0.993967	0.92118	1.072504	0.876057
SLC45A3	0.852393	0.815207	0.891276	2.26E-12
DUSP23	1.091495	1.031034	1.155502	0.002603
RNF166	1.019757	0.93833	1.108251	0.64495
NBL1	0.914165	0.867546	0.963288	0.000778
HTR6	1.139167	0.995367	1.303741	0.058423
F11R	0.903653	0.85161	0.958877	0.000816
USF1	1.039405	0.958305	1.127368	0.351103
SPATA2L	0.972844	0.898359	1.053505	0.498133
NIT1	1.022383	0.936738	1.115858	0.619953
DEDD	1.002622	0.880703	1.141419	0.968424
ZNF276	1.026788	0.953721	1.105453	0.482753

NPM2	1.055161	0.986143	1.129009	0.119787
FGF17	1.115285	1.002922	1.240238	0.04403
CDA	0.99194	0.956966	1.028192	0.658578
PINK1	1.043919	0.955422	1.140613	0.341609
B4GALT3	1.150183	1.020809	1.295953	0.021548
ADAMTS4	1.080083	0.964052	1.210079	0.183982
FAM160B2	1.130835	1.026255	1.246073	0.013014
NDUFS2	1.229464	1.086309	1.391484	0.001073
SLC5A11	1.017694	0.883719	1.17198	0.807587
FCER1G	1.034678	0.992575	1.078567	0.107759
APOA2	1.047502	1.000507	1.096705	0.047522
TOMM40L	1.174658	1.084524	1.272282	7.75E-05
MPZ	0.954736	0.882051	1.033412	0.251592
CACHD1	1.140902	1.070336	1.21612	5.20E-05
CDC42SE2	0.973037	0.903568	1.047846	0.469517
RAPGEF6	1.045409	0.963402	1.134397	0.286679
EPB41	0.949098	0.893659	1.007975	0.088888
ALG8	1.114478	1.032008	1.203538	0.005724
FBXW5	1.058678	0.95881	1.168947	0.259348
SYNJ1	1.053497	0.979648	1.132914	0.159886
IFNAR2	1.106515	1.006324	1.216682	0.036605
MRPL10	1.107805	0.991876	1.237285	0.069474
IFNGR2	1.006962	0.929585	1.090781	0.864951
GART	1.044875	0.97165	1.123618	0.236353
SON	0.953296	0.887651	1.023797	0.18888
DONSON	1.055396	0.968918	1.149593	0.21643
SV2A	1.046549	0.99808	1.097371	0.060037
STC1	1.076375	0.960167	1.206647	0.206722
CSRP1	1.212604	1.121206	1.311452	1.43E-06
C1QC	1.010917	0.966105	1.057807	0.638813
RCAN1	1.114519	1.043753	1.190084	0.001198
UBE2Z	1.03615	0.945208	1.135842	0.448639
SNF8	1.248081	1.098137	1.418499	0.00069
CCDC24	1.244538	1.124998	1.376779	2.18E-05
RUNX1	0.906273	0.846319	0.970475	0.00483
IGF2BP1	1.107447	1.06413	1.152528	5.35E-07
CBR1	1.149595	1.080811	1.222756	9.48E-06
CBR3	1.408747	1.212273	1.637063	7.76E-06
MORC3	0.974286	0.903144	1.051031	0.500697
CHAF1B	1.214493	1.105291	1.334485	5.29E-05
HLCS	1.106468	1.01404	1.20732	0.023012
SCUBE1	1.054214	1.009988	1.100375	0.015757
ARHGAP27	1.034681	0.959154	1.116155	0.378004
ADPGK	0.934605	0.863142	1.011986	0.095634
PTMS	1.1369	1.072731	1.204908	1.50E-05
PADI4	0.998692	0.960497	1.038407	0.94757
ADIPOR1	0.903825	0.830955	0.983085	0.018387
CYB5R1	1.308493	1.169702	1.463752	2.60E-06
PSMD4	1.146942	1.02584	1.282339	0.016037
ATP13A2	1.199768	1.128113	1.275974	6.77E-09
PSMB4	1.096141	0.958143	1.254014	0.181178
IRX6	0.856345	0.775656	0.945429	0.002131
BTG2	1.108006	1.037243	1.183598	0.00232
HK2	0.857269	0.81424	0.902572	4.59E-09
C1R	1.281188	1.166738	1.406866	2.10E-07
ALDH4A1	0.979879	0.917079	1.04698	0.547534
STARD9	1.046488	0.984836	1.111999	0.142447
THEM4	1.097052	1.023477	1.175917	0.00892

UBR1	1.021356	0.955999	1.09118	0.531127
AMFR	0.945535	0.881317	1.014432	0.118602
MED8	1.134345	1.007228	1.277505	0.037642
RGL4	1.041919	0.980562	1.107115	0.184818
RSPRY1	1.044418	0.960059	1.136191	0.311829
CCDC17	0.981015	0.926453	1.038791	0.511516
GPBP1L1	1.013714	0.929561	1.105486	0.758048
NAE1	1.150226	1.052585	1.256924	0.001986
TMEM69	0.980971	0.893406	1.077118	0.687143
ACE	1.003895	0.922454	1.092526	0.928246
SPON2	1.07235	1.010804	1.137644	0.020542
CHCHD6	1.150942	1.04355	1.269386	0.004909
CTBP1	1.142048	1.044295	1.248952	0.003622
LRRC36	0.958444	0.809001	1.135493	0.623596
TPPP3	0.924741	0.890631	0.960158	4.50E-05
ZDHHC1	0.844703	0.78059	0.914081	2.78E-05
ATP6V0D1	0.927289	0.848108	1.013864	0.097394
AGRP	0.916412	0.854733	0.982542	0.014074
ZFYVE28	1.017921	0.933142	1.110403	0.688905
FAM131B	1.102666	0.963753	1.261602	0.154863
RGS12	1.04927	0.976324	1.127666	0.190802
PSKH1	1.073087	0.992415	1.160317	0.076891
ZYX	0.826773	0.77123	0.886316	8.27E-08
ABR	1.108395	1.049804	1.170256	0.000204
CCDC117	1.042085	0.951366	1.141454	0.375026
ZNF230	1.067668	0.968915	1.176487	0.186079
CCDC107	1.281728	1.135393	1.446923	6.00E-05
ZNF222	0.938909	0.87457	1.007982	0.081777
NPR2	1.475653	1.336004	1.629898	1.71E-14
ZNF221	1.066784	0.984926	1.155445	0.112495
ZNF233	0.992574	0.907912	1.085131	0.869842
ZNF235	0.995343	0.910501	1.08809	0.918201
GNE	0.985293	0.90602	1.071503	0.729193
TNFRSF13	1.001809	0.947905	1.058778	0.948939
PTGIR	0.938522	0.889158	0.990626	0.021357
CALM3	1.063316	0.972297	1.162857	0.178743
DFFA	1.063732	0.969582	1.167025	0.191326
CCDC28B	1.1161	1.006533	1.237593	0.037207
IQCC	1.090385	0.999312	1.189758	0.051835
BSDC1	0.90114	0.844197	0.961924	0.001775
ZBTB8A	0.965331	0.907985	1.026298	0.258802
ATAD3B	0.862627	0.804038	0.925486	3.83E-05
SSU72	1.042304	0.929641	1.16862	0.477756
UBE2J2	1.031063	0.939519	1.131526	0.519029
ZNF362	1.048603	0.960695	1.144555	0.288074
FNDC5	1.079757	0.951363	1.225478	0.23482
CPAMD8	0.974261	0.894463	1.061177	0.549786
NR2F6	1.03493	0.957041	1.119158	0.389759
ANKLE1	1.014558	0.94821	1.085547	0.675334
CCDC58	1.108679	0.992983	1.237855	0.066544
KALRN	1.064168	0.969984	1.167497	0.188374
CILP2	0.83446	0.782503	0.889867	3.44E-08
ABCG1	1.038511	0.983669	1.096412	0.172215
TFF3	0.948022	0.890441	1.009327	0.095
UBASH3A	1.017783	0.952985	1.086986	0.599468
RSPH1	1.106646	0.998239	1.226827	0.054048
SLC37A1	1.112645	1.039177	1.191307	0.002194
PDE9A	1.104979	1.027094	1.188771	0.007433

WDR4	1.079226	1.009851	1.153367	0.024504
NDUFV3	1.089529	0.99314	1.195274	0.069629
PKNOX1	1.149152	1.047144	1.261096	0.003376
U2AF1	0.979253	0.930217	1.030874	0.423791
HSF2BP	1.163621	0.987035	1.371801	0.071144
RRP1B	1.068092	0.989768	1.152614	0.09002
PDXK	0.998206	0.932435	1.068616	0.958813
G6PD	1.065702	0.983027	1.15533	0.122473
CSTB	1.040655	0.965401	1.121776	0.298091
RRP1	1.031513	0.955894	1.113116	0.424447
AGPAT3	1.013128	0.947552	1.083242	0.702455
TRAPPC10	0.94478	0.875343	1.019725	0.144717
GAB3	1.047298	0.979259	1.120064	0.177526
ICOSLG	0.914891	0.846668	0.988611	0.02447
ZNF66	0.993996	0.930289	1.062067	0.858581
LRRC3	1.136315	1.031911	1.251283	0.009356
ITGB2	1.070861	1.017706	1.126793	0.008399
RALGDS	1.152318	1.067547	1.24382	0.000276
FTCD	0.978746	0.857858	1.11667	0.749431
LSS	1.053196	0.979067	1.132939	0.163969
VAV2	1.004753	0.951028	1.061512	0.86571
MCM3AP	0.983034	0.917802	1.052902	0.625231
PCNT	1.016641	0.943615	1.095318	0.664331
DIP2A	1.004391	0.94052	1.072599	0.896021
S100B	0.999301	0.954324	1.046397	0.976244
PRMT2	1.09641	1.002661	1.198923	0.043566
CLDND2	1.200872	1.107458	1.302165	9.41E-06
ADAMTS1	1.072712	0.969809	1.186533	0.172519
SLC2A6	0.961319	0.911103	1.014302	0.149531
ZNF761	1.141458	1.044308	1.247645	0.003554
ZNF714	1.026509	0.949534	1.109723	0.51062
GPSM1	0.947057	0.902568	0.99374	0.026707
HIPK4	0.907638	0.810198	1.016797	0.094429
TOR2A	1.130537	1.021574	1.251122	0.017656
ST6GALNA	1.104737	1.02078	1.195598	0.013512
SHKBP1	1.111105	1.007907	1.224869	0.034148
RDH13	1.104024	1.034694	1.178	0.002784
ZER1	0.999713	0.924216	1.081377	0.994285
ZDHHC12	1.028888	0.948124	1.116531	0.49474
PKN3	0.992202	0.926629	1.062415	0.822439
BRSK1	1.180034	1.084404	1.284098	0.000123
COX6B2	0.893831	0.792728	1.007829	0.066858
TAOK1	1.033201	0.96818	1.102589	0.324686
MED27	1.145851	1.019039	1.288444	0.022899
DEDD2	0.957849	0.887959	1.03324	0.265259
SIK3	0.90478	0.837227	0.977783	0.011489
MPZL3	1.088639	1.009849	1.173576	0.026715
NEK8	1.165613	1.065699	1.274894	0.000803
TLCD1	1.049223	0.971321	1.133373	0.222191
PCSK7	1.054354	0.976593	1.138307	0.175724
SAFB	0.923385	0.853377	0.999136	0.047542
CD3G	1.06832	1.012742	1.126948	0.015332
S100A1	1.37221	1.199826	1.569362	3.84E-06
CXCR5	1.007247	0.884155	1.147475	0.913541
ZBTB7B	1.023787	0.966261	1.084739	0.425593
FLAD1	1.20395	1.078002	1.344613	0.000994
SHC1	0.919163	0.840591	1.005079	0.06448
VPS11	0.980327	0.888702	1.081399	0.691467

NLRX1	0.956788	0.885462	1.03386	0.263772
ADAR	1.020624	0.947311	1.09961	0.59144
IL6R	1.068331	1.014741	1.12475	0.011827
UBE2Q1	1.06867	0.958594	1.191385	0.231116
CHRNB2	1.035203	0.950855	1.127033	0.42496
CRTC2	0.902565	0.830803	0.980525	0.015299
ANO10	1.100734	1.016655	1.191765	0.017913
FDPS	1.130353	1.012031	1.262509	0.029859
RUSC1	1.105048	0.99494	1.227342	0.062148
FAM189B	1.070399	0.98434	1.163983	0.111639
PAQR6	1.003345	0.936596	1.074851	0.924259
PMF1	1.031148	0.931112	1.141931	0.555789
SLC25A44	1.021344	0.949182	1.098991	0.572142
LMNA	0.968194	0.922482	1.016172	0.190245
CCR5	1.059371	0.996856	1.125807	0.063098
NBEAL2	1.070545	1.011726	1.132784	0.018064
CCDC12	1.085104	0.980607	1.200737	0.1139
PTH1R	0.865426	0.796715	0.940063	0.000616
UBQLN4	1.051425	0.97181	1.137562	0.21196
GPATCH4	1.20085	1.093994	1.318142	0.000118
FCRL3	0.989812	0.920787	1.064011	0.781267
FGFR4	1.155532	1.038613	1.285613	0.007906
NACC1	0.940826	0.874841	1.011789	0.10016
HK3	1.04117	1.00779	1.075655	0.015236
IER2	0.960418	0.891815	1.034298	0.285472
ZNF394	0.941951	0.871656	1.017915	0.130725
CPSF4	1.093656	0.996991	1.199693	0.057942
LY6E	1.015023	0.964252	1.068468	0.56898
VPS28	1.061549	0.929748	1.212034	0.377209
PTGER1	0.923218	0.862177	0.988581	0.022078
RECQL4	1.104412	1.033492	1.180199	0.003359
LRRC14	1.172837	1.078598	1.275311	0.000191
ZNF333	0.980623	0.900268	1.06815	0.653743
PPP1R16A	1.169044	1.057004	1.292959	0.002378
ORAI2	0.906457	0.844149	0.973364	0.006872
ALKBH4	0.917798	0.843711	0.99839	0.045772
SH2B2	0.972661	0.911475	1.037954	0.403029
SQSTM1	0.927162	0.867282	0.991178	0.026412
MGAT4B	0.928359	0.861146	1.000818	0.052546
RPL8	0.897448	0.824078	0.977351	0.012904
MAML1	0.945505	0.874962	1.021735	0.156643
LRWD1	0.957674	0.886941	1.034048	0.269279
FBXL13	0.949734	0.884708	1.019539	0.154091
NAPEPLD	1.08391	0.999171	1.175835	0.052381
SCGB3A1	1.043973	0.969579	1.124075	0.253904
PSMC2	1.002456	0.904531	1.110981	0.962701
USP41	1.003239	0.912126	1.103453	0.94693
YDJC	1.0339	0.948996	1.1264	0.445739
CCDC116	1.123087	0.960386	1.313351	0.146012
DVL3	0.98713	0.892713	1.091533	0.800631
AP2M1	1.061526	0.952058	1.18358	0.282275
ABCF3	0.952371	0.879662	1.03109	0.228446
PCYT1A	1.140265	1.038922	1.251492	0.005709
U2AF1L4	1.083046	0.96295	1.21812	0.183396
BDH1	0.969052	0.895137	1.04907	0.437402
NPHS1	0.937698	0.862934	1.01894	0.129171
THAP8	1.002711	0.921637	1.090918	0.949811
COX7A1	0.956699	0.905904	1.010343	0.111768

ZNF382	0.966455	0.879747	1.061709	0.476812
LRRC56	1.185877	1.098893	1.279747	1.15E-05
PLXDC1	1.150371	1.029173	1.285842	0.013655
PGAP3	1.136635	1.042279	1.239534	0.003774
IKZF3	1.003975	0.952516	1.058214	0.882519
GRIN2C	0.999783	0.937271	1.066464	0.994744
FDXR	1.214652	1.123723	1.312939	9.67E-07
SAP30BP	0.956516	0.874367	1.046383	0.331866
ACOX1	1.059884	0.980793	1.145354	0.141608
PRPSAP1	1.177119	1.064526	1.301621	0.001478
CYGB	0.916648	0.845299	0.99402	0.035287
ZNF577	1.005181	0.921332	1.096662	0.907422
TMEM143	1.074254	0.968546	1.1915	0.175337
KLHL10	1.191611	1.020822	1.390974	0.026346
HCRT	1.156979	1.010279	1.324982	0.035049
ALDH16A1	1.124812	1.035411	1.221932	0.005377
ITGA5	0.964719	0.905946	1.027304	0.262714
SIGLEC11	1.075387	0.954007	1.212211	0.234275
ZNF385A	1.009747	0.953257	1.069584	0.741233
SIGLEC16	0.97024	0.889967	1.057752	0.492911
MPP3	1.32536	1.21475	1.44604	2.37E-10
NAGS	1.047601	0.919168	1.193979	0.485884
LSM12	0.981153	0.888486	1.083485	0.706999
ASB16	1.109469	1.025348	1.200491	0.009817
JOSD2	1.13779	1.037944	1.247241	0.005875
SHANK1	0.926776	0.887905	0.967349	0.000504
DBF4B	1.118131	1.010081	1.237739	0.031287
PLCD3	0.810782	0.752984	0.873015	2.71E-08
FMNL3	1.164769	1.091057	1.243462	4.82E-06
RACGAP1	1.075167	1.0058	1.149318	0.033178
LARP4	1.060178	0.980686	1.146113	0.14169
RAVER1	0.897675	0.828229	0.972944	0.008598
SYCE2	0.93897	0.874254	1.008477	0.083936
SPC24	1.156155	1.084917	1.232069	7.75E-06
LEMD2	0.998069	0.929042	1.072224	0.957846
ALOX15	0.853176	0.801672	0.907989	5.79E-07
TREML1	1.071886	1.005357	1.142818	0.033722
ZNF653	1.077991	0.970551	1.197324	0.160928
MED11	1.20672	1.0918	1.333737	0.000233
CXCL16	0.994552	0.953864	1.036976	0.797697
BCL6B	0.909826	0.855044	0.968118	0.002858
ASGR2	1.088459	1.037642	1.141764	0.000511
TNFSF13	1.15127	1.061343	1.248816	0.000687
SENP3	1.038797	0.91966	1.173368	0.54025
EIF4A1	1.016567	0.948137	1.089935	0.643994
RPL26	0.985774	0.913001	1.064349	0.714235
POLR3K	1.27743	1.176462	1.387064	5.60E-09
SNRNP25	1.331383	1.215237	1.458629	7.96E-10
WDR90	1.051207	0.975482	1.13281	0.190472
JMJD8	1.087698	0.978968	1.208503	0.117726
CCDC78	0.914283	0.844252	0.990123	0.027517
MSLNL	0.882273	0.784293	0.992495	0.037034
SPSB3	1.014489	0.856527	1.201581	0.867705
CCNF	1.157298	1.086563	1.232637	5.63E-06
TBC1D24	1.016779	0.942099	1.097379	0.668995
AMDHD2	0.988336	0.924609	1.056454	0.730081
NTN3	1.043144	0.931133	1.16863	0.466117
PAQR4	1.062362	0.990779	1.139115	0.089189

FLYWCH2	1.200732	1.093188	1.318857	0.000133
ZNF75A	1.137663	1.036341	1.248892	0.006728
ADCY9	0.999822	0.961341	1.039843	0.992905
CLPB	1.047445	0.974857	1.125439	0.205861
NEU3	1.025816	0.964415	1.091125	0.4183
ASRGL1	1.174388	1.102494	1.25097	6.12E-07
GNG3	1.053291	0.983313	1.128249	0.138819
UBXN1	0.91512	0.814993	1.027547	0.133532
TTC9C	1.059428	0.9633	1.165148	0.234231
TAF6L	0.979734	0.889716	1.078859	0.677138
NXF1	0.826058	0.771922	0.883992	3.29E-08
STX5	0.831738	0.774488	0.893219	4.12E-07
SLC25A45	1.164496	1.061302	1.277724	0.001297
RPL29	0.915329	0.856444	0.978262	0.009113
ITIH3	1.079931	0.968324	1.204401	0.167086
SYVN1	0.885525	0.81341	0.964034	0.00503
ZFPL1	1.112115	0.955164	1.294856	0.171003
RPS6KA4	0.996072	0.93113	1.065543	0.908904
LRP5	0.945281	0.892471	1.001216	0.055044
TPCN2	0.954852	0.892995	1.020993	0.176383
PDZK1IP1	1.038222	0.989829	1.08898	0.123517
TAL1	1.016962	0.979859	1.05547	0.375096
CMPK1	0.920171	0.842254	1.005296	0.065336
BEND5	1.028466	0.946712	1.117281	0.506575
ZYG11B	1.090112	1.008194	1.178686	0.030411
MAGOH	1.089233	0.964071	1.230643	0.169927
ACOT11	0.979212	0.925034	1.036563	0.469443
FAM151A	1.019628	0.935598	1.111204	0.657799
PARS2	1.075638	0.984346	1.175398	0.107117
USP24	0.948237	0.896372	1.003102	0.064018
NOL9	1.069757	0.990104	1.155819	0.087628
KLHL21	0.974397	0.918763	1.033399	0.387208
ZSWIM5	1.221916	1.065914	1.400751	0.004028
GMEB1	0.949465	0.870434	1.035672	0.242206
JAK1	0.924155	0.857399	0.996109	0.03922
RAVER2	0.91573	0.858461	0.97682	0.007546
CTRC	1.040947	0.962597	1.125675	0.314823
LZIC	1.12207	1.022045	1.231885	0.015619
RBP7	0.929035	0.87074	0.991231	0.025991
FBLIM1	1.037005	0.871052	1.234577	0.682987
SLC25A34	1.003523	0.923321	1.090692	0.934042
AKR7A3	1.115073	1.039796	1.1958	0.002256
DHRS3	0.971529	0.92909	1.015908	0.205004
MATN1	0.988317	0.891786	1.095297	0.822673
LAPTM5	1.028639	0.949408	1.114482	0.489903
SDC3	1.040555	0.948065	1.142069	0.402571
PEF1	0.986152	0.885985	1.097644	0.798597
RBBP4	1.070555	0.989996	1.157669	0.087624
KIAA1522	0.943987	0.882458	1.009805	0.093694
TSSK3	0.945564	0.872633	1.02459	0.171693
TMCO4	1.048512	0.971961	1.131091	0.220682
UBXN10	1.166683	1.088356	1.250648	1.38E-05
CAMK2N1	1.280975	1.120891	1.463922	0.000277
ALPL	1.089862	1.042806	1.139042	0.000133
WNT4	0.984489	0.928218	1.044173	0.602674
TTLL10	1.030343	0.945846	1.122388	0.493545
SCNN1D	1.109881	1.025292	1.201449	0.009952
MXRA8	1.079325	0.996767	1.168721	0.06008

MEGF6	0.943982	0.901864	0.988067	0.013307
IL23R	1.18319	1.08119	1.294812	0.000255
NFIA	1.13593	1.069369	1.206634	3.52E-05
OMA1	1.019253	0.96003	1.082129	0.532379
MYSM1	0.985269	0.912339	1.064028	0.705252
TM2D1	1.15273	1.051982	1.263126	0.002319
USP1	1.10786	1.022362	1.200507	0.012431
FUBP1	0.951051	0.883142	1.024182	0.184242
NEXN	1.143962	1.046551	1.25044	0.003057
DNAJB4	1.017717	0.93863	1.103467	0.670483
TYW3	1.00086	0.910729	1.099911	0.985757
B3GALT2	0.991246	0.943317	1.041609	0.728033
FAM102B	1.069404	0.993854	1.150698	0.072648
GBP2	0.953335	0.899971	1.009863	0.103944
ATXN7L2	1.08594	0.999079	1.180352	0.052586
GBP4	1.015048	0.971961	1.060046	0.499736
ZNF326	0.977016	0.909276	1.049801	0.525906
GFI1	0.959867	0.912702	1.00947	0.111086
AGL	1.073347	0.996845	1.155719	0.060627
VCAM1	1.032522	0.965335	1.104386	0.351191
EXTL2	1.099374	1.029257	1.174268	0.004839
SLC30A7	1.004094	0.935163	1.078106	0.910356
ZNF281	1.020345	0.944437	1.102355	0.609608
ARPC5	1.16068	1.076783	1.251115	9.92E-05
NLRP3	1.016261	0.967494	1.067488	0.520295
ZNF496	0.896072	0.83471	0.961944	0.00243
TRIM58	0.999869	0.958823	1.042671	0.995108
SLAMF9	1.137869	1.05458	1.227735	0.000868
KCNJ9	1.030123	0.923944	1.148504	0.59284
IGSF8	0.977223	0.908943	1.050631	0.532975
DDR2	0.966838	0.850377	1.099248	0.606564
PEA15	1.090628	0.999904	1.189584	0.050253
PEX19	1.2011	1.078105	1.338128	0.000886
NCSTN	1.049616	0.957003	1.151191	0.304201
VANGL2	1.088648	1.028449	1.152371	0.003428
SLAMF6	0.966589	0.911109	1.025446	0.259835
OLFML2B	1.090996	1.013521	1.174393	0.020486
FCRLB	1.050239	0.958827	1.150366	0.291415
FCGR3B	1.008395	0.971298	1.046909	0.66199
KLHDC9	1.006984	0.900017	1.126663	0.903322
FLVCR1	1.105097	1.020804	1.19635	0.013564
ATF3	0.916244	0.867897	0.967285	0.001564
RBM15	0.99531	0.926261	1.069506	0.898022
DENND2D	1.090388	1.016259	1.169925	0.015999
IER5	0.941028	0.87845	1.008065	0.083418
SNED1	1.024729	0.947776	1.107931	0.539666
BPNT1	1.079449	1.007431	1.156615	0.029997
ACP6	1.242172	1.153348	1.337838	1.01E-08
KIF26B	0.924163	0.878464	0.972239	0.002303
TFB2M	0.925427	0.857931	0.998232	0.044883
KLHDC8A	1.1008	1.033626	1.17234	0.002795
PM20D1	1.226301	1.100916	1.365966	0.00021
PKDCC	1.291296	1.164472	1.431933	1.25E-06
OXER1	1.004743	0.93833	1.075856	0.892135
HAAO	0.943465	0.880371	1.01108	0.099366
B3GALNT2	1.10134	1.003962	1.208164	0.040985
MAPKAPK	0.884656	0.819762	0.954688	0.001617
IL24	0.971977	0.913689	1.033983	0.367681

PIGR	1.11165	0.980303	1.260595	0.098968
CAPN2	0.998961	0.943979	1.057146	0.971292
MRPL55	0.976762	0.875422	1.089833	0.673965
WDR26	0.956694	0.894829	1.022835	0.194287
REL	0.92176	0.86556	0.981609	0.011139
PUS10	0.945619	0.8723	1.025101	0.17449
PEX13	1.049068	0.963522	1.142209	0.269707
KIAA1841	1.004852	0.920559	1.096863	0.91378
TRIM17	1.178102	1.077771	1.287773	0.000307
RFTN2	1.064725	0.956722	1.184921	0.250455
DISC1	0.921476	0.864031	0.98274	0.01277
MEMO1	0.916526	0.84814	0.990425	0.027585
DPY30	1.089843	0.972574	1.221252	0.138554
ARL5A	0.955855	0.881563	1.036408	0.274092
DUSP19	1.201676	1.087103	1.328324	0.000326
NUP35	1.14985	1.05486	1.253394	0.001504
CCDC138	0.976657	0.904555	1.054507	0.546094
FBXO41	0.952464	0.895445	1.013114	0.122028
SMC6	1.050506	0.964327	1.144386	0.259231
CCDC74A	1.076485	0.983398	1.178385	0.110229
SLC16A14	1.274876	1.175537	1.382609	4.43E-09
SGCB	1.285813	1.179593	1.401599	1.10E-08
NOSTRIN	1.059672	0.984818	1.140215	0.120982
SGPP2	1.069235	0.992547	1.151848	0.077909
BBS5	1.159264	1.013381	1.326147	0.031266
SMARCAD	0.995392	0.925583	1.070466	0.900926
HPGDS	0.890678	0.856696	0.926008	5.44E-09
PDLIM5	1.191128	1.115738	1.271611	1.58E-07
RPRD2	0.949036	0.86025	1.046985	0.296592
ANKRD23	1.182701	1.046448	1.336695	0.00721
CTSS	0.951718	0.901516	1.004715	0.07348
MSX1	1.134897	1.065868	1.208396	7.74E-05
PACRGL	1.139168	1.034107	1.254903	0.008307
BNIPL	0.994913	0.933055	1.060872	0.876251
C1QTNF7	1.134326	1.011624	1.271912	0.030942
TNFAIP8L2	1.077161	1.017439	1.14039	0.010649
LYSMD1	1.287383	1.160128	1.428596	1.97E-06
SCNM1	1.100314	0.975206	1.241471	0.120595
TMOD4	1.077683	0.986044	1.177838	0.098942
VPS72	1.115662	1.036121	1.201309	0.003729
ERCC3	1.053425	0.965869	1.148918	0.239764
RNF149	0.953239	0.892061	1.018612	0.157046
IWS1	1.037094	0.939081	1.145338	0.472093
BOLA3	1.287574	1.149182	1.442633	1.32E-05
CDC42EP3	1.136305	1.068106	1.208859	5.20E-05
S100A11	1.013135	0.972032	1.055976	0.536878
DHX57	1.135251	1.035419	1.244708	0.006911
ARHGAP25	0.909278	0.846906	0.976244	0.008713
S100A9	1.012802	0.986069	1.040259	0.351316
S100A12	1.006269	0.980484	1.032733	0.637005
TGFA	1.05879	0.986177	1.13675	0.115031
TDRD10	0.949488	0.855413	1.05391	0.330233
CCNYL1	1.16439	1.059786	1.279319	0.00153
FZD5	1.042591	0.979752	1.109461	0.188498
DCAF16	1.044411	0.969938	1.124601	0.249624
NPPC	1.032096	0.961343	1.108055	0.383262
GNPDA2	0.991674	0.905494	1.086055	0.856951
PAQR3	1.079335	0.986325	1.181117	0.096818

ANTXR2	0.974714	0.916675	1.036429	0.413564
HELQ	1.119906	1.022309	1.226821	0.014924
MRPS18C	1.298868	1.147326	1.470426	3.61E-05
CGGBP1	1.139274	1.032704	1.256842	0.009263
GPR155	1.090039	1.007537	1.179296	0.031797
DAPL1	1.228307	1.138829	1.324815	9.89E-08
PMVK	1.070799	0.997159	1.149878	0.059877
PBXIP1	0.971322	0.907176	1.040003	0.40387
PYGO2	1.095032	1.015772	1.180478	0.017876
HIPK1	0.950629	0.884494	1.021709	0.168758
LENEP	1.068475	0.952011	1.199187	0.260679
DCST2	1.091727	1.014989	1.174267	0.018272
DCST1	1.104821	1.003738	1.216085	0.041733
COL6A3	1.062655	0.953574	1.184213	0.27146
YY1AP1	0.923124	0.848666	1.004116	0.062288
KBTBD8	1.149234	1.083892	1.218514	3.20E-06
LMOD3	1.104898	0.987091	1.236765	0.082901
SLC22A15	1.075811	1.034717	1.118538	0.000236
ATP1A1	0.999812	0.917795	1.089158	0.996559
SLC15A2	1.052975	1.002807	1.105653	0.03822
EIF4E3	1.003256	0.951898	1.057385	0.903495
PROK2	1.012701	0.982099	1.044256	0.420151
LRRC58	1.021853	0.947539	1.101996	0.574694
FSTL1	1.152341	1.067811	1.243563	0.000264
ELF3	1.144161	1.033865	1.266224	0.009217
TMEM183,	0.975043	0.88817	1.070413	0.595541
IGFBP7	1.07391	1.019653	1.131053	0.007023
TRIM46	0.953793	0.893925	1.017671	0.152618
KRTCAP2	1.13638	1.043716	1.23727	0.00322
CXCR1	1.028217	0.98228	1.076303	0.232768
ARPC2	1.020046	0.912887	1.139785	0.725971
CCT3	1.126245	1.03291	1.228014	0.007069
TMEM79	1.194367	1.091719	1.306667	0.000107
SSR2	1.057346	0.93463	1.196175	0.375669
RNF25	1.271583	1.150742	1.405113	2.41E-06
STK36	1.154687	1.07321	1.242349	0.000117
NEK10	1.009341	0.927086	1.098895	0.830255
CCDC141	1.026417	0.94742	1.112001	0.523397
FEV	1.020529	0.930525	1.119238	0.666188
EOMES	1.015591	0.914643	1.12768	0.772101
CWC22	1.018472	0.931315	1.113785	0.68842
AZI2	0.8615	0.782276	0.948747	0.002454
TGFBR2	0.906775	0.853406	0.963483	0.001567
ANKZF1	1.068101	0.989219	1.153273	0.092366
HDAC11	1.260734	1.124063	1.414023	7.57E-05
TRAT1	0.871705	0.827198	0.918608	2.82E-07
FBLN2	0.981148	0.931053	1.033938	0.476604
GLB1L	1.036875	0.958219	1.121987	0.368311
STT3B	0.994891	0.927589	1.067076	0.886029
CHCHD4	1.100432	1.020811	1.186262	0.012508
FCRL1	1.000795	0.95411	1.049765	0.973992
SERPINI1	1.204777	1.139291	1.274026	6.44E-11
CLASP2	1.024926	0.959381	1.094949	0.46529
SUCLG1	1.078218	0.959551	1.21156	0.205547
NUAK2	1.008378	0.956986	1.062529	0.754591
SPTA1	1.012315	0.978696	1.047089	0.477526
PRKCI	0.955605	0.885975	1.030707	0.239422
MNDA	0.961587	0.926663	0.997827	0.037969

PYHIN1	0.897497	0.845456	0.952742	0.000388
IFI16	0.978896	0.914703	1.047593	0.537645
AIM2	1.100275	1.021804	1.184772	0.011363
EFHB	1.146378	0.932348	1.409541	0.19511
EIF5A2	1.101203	1.018434	1.190699	0.0156
RPL22L1	0.987991	0.909405	1.073367	0.775106
PPM1L	1.080872	1.009113	1.157733	0.026501
ICA1L	1.028404	0.950197	1.113047	0.487654
CTLA4	1.07325	1.002276	1.149249	0.042857
ICOS	1.022412	0.97091	1.076645	0.400629
RYBP	0.944531	0.878631	1.015374	0.121982
PPP4R2	0.927384	0.862168	0.997532	0.042724
CD200R1	0.975524	0.898266	1.059427	0.556098
GTPBP8	1.193934	1.079301	1.320741	0.000578
WDFY3	1.007063	0.963251	1.052869	0.756451
COX18	1.014556	0.928731	1.108312	0.748631
PTPN13	1.01553	0.928107	1.111188	0.737218
THOC7	1.07405	0.961566	1.199693	0.205651
ATXN7	0.941927	0.872599	1.016764	0.125092
PSMD6	0.9649	0.876334	1.062416	0.466982
ADAMTS9	1.142001	1.016783	1.282639	0.025036
PPM1K	1.072397	0.999611	1.150483	0.05128
GMPS	1.006295	0.91374	1.108224	0.898569
TIPARP	0.938101	0.881046	0.998851	0.045948
CCNL1	0.950841	0.893294	1.012095	0.113528
PTX3	0.914291	0.876512	0.953698	3.16E-05
HESX1	0.943615	0.87524	1.017331	0.130474
SLMAP	0.960185	0.896744	1.028114	0.24403
RPL9	0.867042	0.799071	0.940796	0.000614
RPP14	0.962792	0.886676	1.045442	0.366856
ABHD6	1.21087	1.111446	1.319187	1.20E-05
DNASE1L3	0.978971	0.868247	1.103816	0.728555
RBM47	1.032669	0.988027	1.079327	0.153941
APBB2	1.240856	1.156587	1.331265	1.81E-09
IL17RE	1.009176	0.962213	1.058431	0.707157
IL17RC	1.110034	1.02885	1.197624	0.007061
CRELD1	1.046552	0.930387	1.177222	0.448462
PRRT3	0.955591	0.888075	1.02824	0.224348
PCOLCE2	1.135966	1.057826	1.219879	0.000455
MTMR14	0.977791	0.901194	1.060898	0.589464
TTC14	1.100258	1.030797	1.174401	0.004084
CXCL3	1.099376	1.053879	1.146837	1.12E-05
CXCL5	1.154477	1.067245	1.248839	0.000339
PPBP	1.059068	1.027829	1.091257	0.000172
PF4	1.077346	1.043943	1.111818	3.55E-06
MTHFD2L	1.068215	0.982241	1.161715	0.123216
CXCL1	1.119546	1.068611	1.172908	2.00E-06
RCHY1	1.075711	0.987758	1.171495	0.093556
CPA3	0.892556	0.866601	0.919288	4.38E-14
GYG1	0.969839	0.899922	1.045189	0.422428
HPS3	1.053773	0.977471	1.136032	0.17201
TOPBP1	1.002225	0.929804	1.080287	0.953677
RYK	0.867901	0.812769	0.926773	2.33E-05
SNRK	0.94919	0.885445	1.017525	0.141517
TCF23	1.077052	0.989739	1.172069	0.08528
UCN	1.119284	1.038667	1.206157	0.00313
ZNF513	0.936474	0.8634	1.015733	0.113338
SLC4A1AP	1.03676	0.928293	1.1579	0.521996

PLB1	1.028131	0.956027	1.105673	0.454573
SPDYA	0.967386	0.895521	1.045018	0.399841
KIAA1143	1.016094	0.922107	1.119661	0.747146
KIF15	1.110568	1.041916	1.183744	0.001277
TGM4	0.95337	0.870804	1.043766	0.301525
WDR43	0.997518	0.918699	1.083098	0.952806
ZDHHC3	1.021625	0.920445	1.133928	0.687632
CDCP1	1.197568	1.130793	1.268286	7.32E-10
CLEC3B	1.177147	1.036049	1.33746	0.012294
LZTFL1	1.110553	1.006573	1.225273	0.036565
FYCO1	0.947387	0.885903	1.013139	0.11441
CCR1	1.105941	1.063581	1.149988	4.34E-07
LRRC2	1.237167	1.108673	1.380553	0.000142
FBXO40	1.041586	0.913629	1.187464	0.542355
DTX3L	1.053074	0.986881	1.123706	0.118458
ZNF148	1.019909	0.946081	1.099499	0.607107
NMNAT3	1.163016	1.081012	1.251241	5.17E-05
ZMYM6	1.050907	0.955965	1.155279	0.304047
YEATS2	0.929767	0.860228	1.004927	0.066351
ZC3H12A	0.989566	0.931307	1.051469	0.734753
MEAF6	1.036168	0.919569	1.167551	0.559684
SNIP1	0.946424	0.879443	1.018507	0.141474
DNALI1	1.042847	0.926768	1.173465	0.485915
POLR2H	1.319917	1.19136	1.462347	1.10E-07
LIPH	1.207837	1.126724	1.294789	1.02E-07
TMEM41A	1.068723	0.983725	1.161066	0.115976
RPN1	1.018864	0.92682	1.12005	0.698864
SENP2	0.953238	0.881705	1.030574	0.228865
HEYL	1.343036	1.208773	1.492212	4.06E-08
IFT122	0.968557	0.896212	1.046741	0.419891
RFC4	1.168324	1.063304	1.283718	0.001207
RPL39L	0.961857	0.899672	1.028341	0.254107
BAP1	0.994838	0.911815	1.085421	0.907335
TKT	0.853743	0.795231	0.91656	1.27E-05
PRKCD	1.148542	1.068242	1.234879	0.00018
RFT1	1.163796	1.074616	1.260376	0.000192
SFMBT1	1.050752	0.986127	1.119612	0.12636
GNL3	1.090932	0.996483	1.194333	0.059605
PBRM1	1.045645	0.964755	1.133318	0.27725
ARHGEF3	1.119259	1.048231	1.195099	0.000757
SLBP	0.955928	0.871975	1.047964	0.336531
LRPAP1	1.119921	1.018751	1.231138	0.019051
ZDHHC19	1.143793	1.064666	1.228801	0.00024
UBXN7	0.921068	0.866136	0.979483	0.008774
RNF168	1.102972	1.022311	1.189997	0.011424
PIGX	1.124479	1.009222	1.252898	0.033475
S100P	0.923096	0.894653	0.952444	5.41E-07
CLDN19	0.856295	0.778926	0.941349	0.001323
ERMAP	1.060149	0.989516	1.135824	0.096842
ZNF691	1.154381	1.061217	1.255725	0.000826
SGMS2	1.073463	1.013168	1.137345	0.016238
METAP1	0.996103	0.913681	1.085961	0.929397
DNAJB14	0.992379	0.92268	1.067342	0.836861
EMCN	1.170283	0.952045	1.438548	0.135364
BDH2	1.120849	1.01087	1.242795	0.030377
PGRMC2	1.202454	1.094538	1.32101	0.000122
CDC25A	1.108084	1.0363	1.184841	0.00267
CAMP	1.015381	0.98783	1.0437	0.276807

ZNF589	1.065757	0.987029	1.150765	0.103839
PLXNB1	1.035716	0.982595	1.091709	0.19143
CCDC51	1.222047	1.113673	1.340967	2.31E-05
ATRIP	1.180351	1.063428	1.31013	0.001837
SHISA5	1.110824	1.015274	1.215366	0.022004
SPRY1	0.965769	0.926094	1.007144	0.103659
APEH	1.121789	1.036417	1.214193	0.004432
INTU	1.188385	1.079369	1.308413	0.000439
RNF123	1.078414	1.00633	1.155661	0.032458
HSPA4L	1.123545	1.007557	1.252886	0.036136
MFSD8	1.004894	0.9296	1.086287	0.902215
CAMKV	1.115679	1.028293	1.210492	0.008528
MON1A	1.270501	1.152122	1.401043	1.61E-06
MST1R	1.206164	1.069546	1.360233	0.002242
RAD54L2	1.024763	0.955281	1.0993	0.494704
TEX264	1.190296	1.081683	1.309815	0.000359
GRM2	1.403534	1.262837	1.559907	3.18E-10
DUSP7	1.302156	1.224849	1.384342	2.79E-17
PPM1M	1.09731	1.021797	1.178403	0.010689
WDR82	1.03814	0.959662	1.123035	0.350657
NDST3	0.977368	0.905829	1.054557	0.555012
HMGB2	0.939328	0.867958	1.016566	0.120556
SAP30	0.871863	0.828645	0.917334	1.25E-07
SCRG1	0.953356	0.876431	1.037031	0.265778
MAD2L1	1.129831	1.038308	1.229421	0.004623
ANXA5	1.014642	0.979538	1.051003	0.418449
MAP9	1.31633	1.179057	1.469584	1.00E-06
FBXO8	0.990751	0.90801	1.081032	0.834572
HPGD	0.952907	0.900654	1.008193	0.09366
TMEM144	1.177201	1.082769	1.279869	0.000131
IL15	1.192959	1.097758	1.296416	3.21E-05
FAM160A1	0.939757	0.815781	1.082575	0.389359
ARFIP1	1.108718	1.032047	1.191084	0.004761
HHIP	1.133512	1.005569	1.277734	0.040282
ANAPC10	0.958301	0.869028	1.056745	0.393265
ABCE1	1.022929	0.943994	1.108464	0.580065
OTUD4	0.929234	0.86545	0.99772	0.043086
LSM6	1.21123	1.084886	1.352288	0.000651
TMEM184	1.145773	1.043841	1.257659	0.004202
ITGA2	1.219579	1.125427	1.321606	1.28E-06
MOCS2	1.046149	0.949484	1.152656	0.36174
EDIL3	1.020924	0.966193	1.078757	0.461355
TMEM161	1.117226	1.027622	1.214642	0.009356
ELOVL7	1.103655	1.040471	1.170676	0.001042
NDUFAF2	1.197571	1.054659	1.359848	0.005423
LMBRD2	1.122185	1.034891	1.216842	0.00527
NIPBL	0.964149	0.903216	1.029193	0.27304
SLC25A46	1.087911	0.995851	1.188483	0.06179
STARD4	1.134286	1.071588	1.200653	1.40E-05
PGGT1B	1.056916	0.977668	1.142589	0.163918
F2RL2	1.017033	0.957641	1.080109	0.582222
CCDC112	1.045974	0.971465	1.126197	0.233207
CMBL	0.960069	0.905127	1.018345	0.175311
PRRC1	1.050337	0.967091	1.14075	0.243735
F2RL1	1.059324	0.996392	1.126231	0.065141
AGGF1	1.094016	0.987571	1.211936	0.085345
WDR41	1.023504	0.93961	1.114889	0.594428
NDUFS4	0.900858	0.812763	0.998501	0.046752

SPINK1	1.085292	0.974017	1.20928	0.138081
GRPEL2	1.261633	1.171568	1.358622	7.74E-10
ARSK	1.214248	1.08575	1.357954	0.00067
RHOBTB3	0.988509	0.938114	1.041611	0.665085
TIGD6	1.298019	1.144543	1.472077	4.85E-05
SERINC5	0.890732	0.842104	0.942167	5.35E-05
CAGE1	1.073122	0.935582	1.230883	0.313234
CASP3	0.923066	0.864702	0.98537	0.016297
ERAP1	0.982225	0.910865	1.059176	0.641192
ERAP2	0.949534	0.89349	1.009093	0.095246
CMYA5	1.158171	1.026188	1.307129	0.017372
RICTOR	0.961557	0.911524	1.014337	0.150476
EBF1	1.040762	0.980212	1.105054	0.191408
ANKRA2	1.112124	1.006973	1.228255	0.035986
UBLCP1	1.103248	1.011092	1.203803	0.027256
UTP15	1.103277	1.004537	1.211722	0.039918
TLR3	1.104461	0.958849	1.272185	0.168385
NSA2	0.92343	0.847747	1.00587	0.067877
GFM2	1.040611	0.955415	1.133405	0.36102
TERT	1.17373	1.08485	1.269892	6.69E-05
CCDC127	1.083516	0.957741	1.225808	0.202623
ACSL6	1.07705	0.995164	1.165674	0.065796
IL3	1.531834	1.248294	1.879779	4.44E-05
CSF2	1.220689	1.137549	1.309905	3.01E-08
SHROOM1	1.163832	1.057401	1.280977	0.001931
GDF9	0.986571	0.908908	1.070871	0.746562
UQCRQ	1.202094	1.082316	1.335127	0.000588
LEAP2	0.992645	0.940532	1.047646	0.788468
GJB7	1.001543	0.922301	1.087592	0.970761
SLC35A1	1.061811	0.973743	1.157845	0.174575
TXLNB	0.981928	0.880751	1.094728	0.742378
CITED2	0.888868	0.833975	0.947374	0.000292
DCBLD1	1.256499	1.161257	1.359553	1.37E-08
SFXN1	1.197945	1.120647	1.280574	1.11E-07
SAMD3	1.022697	0.914605	1.143564	0.693741
PDSS2	1.116578	1.034831	1.204782	0.004475
STXBP5	0.928104	0.881638	0.97702	0.004412
IL31RA	1.028995	0.974719	1.086294	0.301221
ANKRD55	0.955225	0.868395	1.050736	0.346132
PI16	1.018655	0.910049	1.140223	0.747957
DAGLB	0.844211	0.790014	0.902126	5.66E-07
KIAA0895	1.076668	0.99303	1.167349	0.073382
STK17A	0.947105	0.881451	1.017648	0.138161
TRA2A	0.92301	0.859807	0.99086	0.02685
GALNT10	1.031906	0.955562	1.114348	0.423204
SAP30L	0.970855	0.901854	1.045136	0.431676
RPS14	0.913998	0.845945	0.987525	0.022729
MYOZ3	0.956242	0.863139	1.059387	0.391927
COG5	0.964641	0.899641	1.034337	0.311808
GPR85	1.269405	1.140175	1.413283	1.33E-05
SLU7	0.91411	0.830119	1.0066	0.06782
RP9	1.013671	0.917338	1.12012	0.789849
PTTG1	1.259237	1.155002	1.372879	1.71E-07
CAMLG	0.947967	0.860908	1.043829	0.276946
RELL2	1.086738	1.011591	1.167467	0.022894
KCNK5	1.075252	1.027629	1.125082	0.001694
ZNF12	0.960059	0.895621	1.029133	0.250197
SLC29A4	1.023507	0.938938	1.115693	0.597469

CDCA7L	0.891365	0.838374	0.947704	0.000235
SP8	1.09117	1.008076	1.181112	0.030849
MIOS	0.999082	0.9173	1.088156	0.983191
USP49	1.079361	1.004101	1.160262	0.038364
SYTL3	0.941124	0.89174	0.993242	0.027345
ZNF704	0.832731	0.783119	0.885486	5.20E-09
FABP5	0.989781	0.912932	1.073099	0.803286
TAGAP	1.028357	0.959886	1.101711	0.426385
COL1A2	1.108469	1.031045	1.191706	0.005311
SLC13A4	1.030128	0.945207	1.122678	0.498905
BRI3	0.848103	0.779026	0.923305	0.000144
LMTK2	0.957043	0.900755	1.016849	0.155697
CTSB	1.037761	0.966272	1.114539	0.308767
DLC1	0.967058	0.923477	1.012696	0.154519
ADCY1	1.169481	1.043114	1.311157	0.007287
RAD21	0.950616	0.878046	1.029183	0.211304
MED30	0.855121	0.777656	0.940302	0.001236
PHKG1	0.9302	0.88107	0.982069	0.008961
DEFA4	1.009481	0.987042	1.032429	0.41065
OSGIN2	0.909065	0.836392	0.988051	0.024914
DEFB1	1.015047	0.983585	1.047514	0.35254
OXR1	0.962834	0.89199	1.039304	0.331394
GPR146	1.139599	1.040556	1.248069	0.004848
UNCX	0.928223	0.868455	0.992105	0.02828
TMEM184/	0.975345	0.919358	1.034741	0.407851
NOS3	1.134785	0.970057	1.327486	0.11409
MICALL2	0.897655	0.846927	0.951421	0.000275
CA3	1.163991	1.093423	1.239114	1.95E-06
INTS1	1.033027	0.961462	1.109919	0.375048
CDK5	0.999312	0.923322	1.081556	0.986391
SLC4A2	1.00282	0.932215	1.078773	0.939738
FASTK	1.071015	0.983947	1.165787	0.112764
TMUB1	0.87549	0.797861	0.960671	0.005002
PHAX	1.119606	1.018825	1.230356	0.018901
ALDH7A1	1.011906	0.900225	1.137443	0.842761
FOXK1	0.975953	0.916072	1.039749	0.451198
COX6C	1.177972	1.058904	1.310429	0.00259
OSR2	1.189606	1.076087	1.315101	0.000691
YWHAZ	0.972533	0.882741	1.071458	0.573092
BAALC	0.936869	0.904939	0.969925	0.000228
FZD6	1.144701	1.0859	1.206686	5.09E-07
CTHRC1	1.096563	1.022395	1.176111	0.009885
SLC25A32	1.063328	0.936193	1.207728	0.344595
TP53INP1	0.971968	0.92392	1.022515	0.271684
INTS8	0.987245	0.912818	1.067739	0.748203
FREM1	1.049377	0.997766	1.103658	0.061064
GEM	1.197603	1.118157	1.282693	2.62E-07
TMEM67	1.23572	1.133753	1.346858	1.46E-06
SNAPC3	1.050386	0.975157	1.131419	0.194809
NUDT2	0.959196	0.864394	1.064396	0.432686
TMEM65	1.142914	1.070146	1.220631	6.90E-05
PSIP1	1.023027	0.934379	1.120087	0.622516
UBAP1	0.908049	0.843083	0.978022	0.010874
SYK	1.058668	0.98786	1.134552	0.106494
NIPSNAP3	1.062555	0.975928	1.156872	0.161994
ABCA1	1.030403	0.98492	1.077985	0.193503
NFIL3	1.026559	0.965238	1.091776	0.404214
LETM2	0.99029	0.911378	1.076035	0.817857

METTL2B	1.048854	0.95674	1.149837	0.309145
FXN	1.045529	0.958504	1.140454	0.315316
TMEM71	0.974914	0.920452	1.032597	0.386349
MAMDC2	0.929763	0.896263	0.964516	0.0001
CPA6	0.929943	0.844708	1.02378	0.138655
ALDH1A1	1.146654	1.108082	1.186569	4.56E-15
KDM1B	1.040581	0.966479	1.120366	0.291254
HGSNAT	0.931753	0.8632	1.00575	0.069844
GKAP1	1.06189	0.970131	1.162328	0.192809
KIF27	1.017382	0.914158	1.132262	0.752222
HNRNPK	0.999226	0.906138	1.101877	0.987616
TRPV6	1.188734	1.024176	1.379732	0.022954
ANKS6	1.229077	1.137835	1.327634	1.60E-07
FBP1	1.03648	0.99563	1.079007	0.080729
ZHX1	0.949602	0.873796	1.031984	0.223119
CYBB	0.998216	0.964951	1.032628	0.917762
DYNLT3	1.103675	1.020599	1.193513	0.013487
MID1IP1	0.917018	0.857442	0.980734	0.011485
NCF1C	1.058017	1.010697	1.107552	0.015703
KIAA1958	0.878666	0.822276	0.938923	0.000132
PCDH19	1.187322	1.107051	1.273413	1.53E-06
PIGA	0.895217	0.840781	0.953178	0.000544
OR1K1	1.06379	0.930488	1.21619	0.365325
STRBP	0.976076	0.903227	1.0548	0.540628
CLDN3	1.294146	1.18578	1.412416	7.51E-09
GAPVD1	0.965702	0.903734	1.03192	0.30236
ATP7A	1.023842	0.955322	1.097277	0.504964
ZNF367	1.124162	1.047798	1.206091	0.001111
HDX	1.076052	0.972321	1.190851	0.156413
NDUFB6	1.117218	1.009326	1.236643	0.032426
NOL6	1.173603	1.081087	1.274036	0.000133
AQP3	1.05565	0.998389	1.116194	0.056999
VCP	0.971426	0.889954	1.060358	0.516567
PIGO	1.005711	0.918376	1.10135	0.902219
STOML2	1.148436	1.034541	1.27487	0.009399
BRWD3	0.965301	0.89787	1.037796	0.339148
SLITRK5	1.31994	1.223685	1.423767	6.71E-13
MELK	1.137347	1.062609	1.217343	0.000206
OTUD1	0.941856	0.881902	1.005887	0.074254
ARHGAP1:	0.975839	0.919026	1.036163	0.42419
HECTD2	1.150598	1.01846	1.29988	0.024206
SLC7A3	1.236497	1.136702	1.345053	7.64E-07
FBXO33	0.995211	0.915779	1.081534	0.909949
LRRC18	1.024775	0.968255	1.084594	0.397849
WRN	1.015037	0.950433	1.084033	0.656453
TSHR	0.929422	0.830929	1.039589	0.20032
CFL2	1.021657	0.941504	1.108635	0.607261
SUGT1	1.067129	0.974189	1.168936	0.162264
GTF2A1	1.070499	0.992661	1.15444	0.076942
ZCCHC24	1.083121	1.020154	1.149974	0.008977
PGM2L1	1.004975	0.930165	1.085801	0.899938
SLC16A9	1.139612	1.064726	1.219764	0.000164
FOLR2	1.124512	1.05208	1.201931	0.000551
INPPL1	0.997735	0.925324	1.075812	0.952959
PHOX2A	1.039165	0.94008	1.148693	0.452407
GJB2	1.064432	0.945367	1.198494	0.302216
CRYL1	1.12243	1.024548	1.229662	0.013105
REEP3	1.06831	1.015112	1.124295	0.011229

PCF11	0.966306	0.912977	1.02275	0.236675
RPL10L	0.801425	0.686597	0.935457	0.005023
RPL36AL	0.982783	0.922131	1.047424	0.593091
ZNF22	1.042865	0.965342	1.126614	0.286893
KLHDC2	0.992692	0.90703	1.086444	0.873434
RPUSD4	1.056541	0.966404	1.155085	0.226714
ARF6	1.006121	0.9075	1.11546	0.907705
TTC8	1.11523	1.036782	1.199614	0.003383
TMEM63C	1.283818	1.165998	1.413544	3.64E-07
AKR1E2	1.020364	0.965235	1.078642	0.476855
KBTBD6	1.077752	0.990725	1.172424	0.081324
FAAH2	1.053016	0.967274	1.146358	0.233214
NUDT5	0.983678	0.901497	1.073351	0.711599
DACT1	1.149915	1.060957	1.246332	0.000673
PRPF18	1.286114	1.14509	1.444506	2.17E-05
TAF3	1.203686	1.068474	1.356008	0.002293
VDAC2	0.944481	0.850029	1.04943	0.288008
COMTD1	1.236921	1.12108	1.364731	2.25E-05
SLC18A2	0.936771	0.877922	0.999565	0.048483
PDZD8	0.986208	0.935826	1.039303	0.603705
ZNF503	1.09539	1.048859	1.143985	3.89E-05
QSOX2	0.950444	0.888823	1.016336	0.137238
NSD1	1.041259	0.969095	1.118796	0.269896
PRDX3	1.1351	1.055538	1.220659	0.000631
ENOX2	1.140143	1.060838	1.225376	0.000363
GHITM	1.142812	1.018205	1.282668	0.023436
CLEC1B	1.003428	0.944452	1.066087	0.911832
SNAPC4	1.052747	0.966214	1.14703	0.240157
PMPCA	1.011593	0.930801	1.099398	0.786069
TSC1	1.040533	0.958592	1.129478	0.342402
GFI1B	1.066695	1.018083	1.117628	0.006667
HPRT1	1.180751	1.069422	1.30367	0.001008
ZMYND19	1.073936	0.986792	1.168775	0.098527
STOX1	1.281874	1.140523	1.440743	3.10E-05
RET	1.039039	0.980654	1.100901	0.194327
DDX21	0.922281	0.854639	0.995278	0.037364
BMS1	0.986962	0.902959	1.07878	0.772459
STK32C	1.201142	1.114585	1.294421	1.56E-06
FUNDC2	1.002822	0.873433	1.151379	0.968104
NDRG2	1.168371	1.097764	1.243519	9.94E-07
ZNF219	0.819641	0.769513	0.873033	6.53E-10
CASP7	0.895524	0.827662	0.968949	0.006061
METTL3	0.993997	0.926976	1.065863	0.865751
SALL2	1.042802	0.977926	1.111982	0.200947
PRAP1	1.1003	0.990462	1.222318	0.074855
TRUB1	1.134945	1.034081	1.245646	0.007682
ZFYVE1	0.971775	0.899842	1.049458	0.465586
FRAT1	1.00662	0.941335	1.076433	0.84707
UBTD1	0.947706	0.885151	1.014682	0.123168
E2F7	1.225936	1.150417	1.306413	3.40E-10
ISCA2	1.202123	1.073521	1.346131	0.001428
PACSIN3	1.030085	0.932391	1.138014	0.559874
TTC7B	0.889917	0.82648	0.958223	0.001995
SLC39A13	1.057045	0.96617	1.156467	0.226438
PSMC3	1.10868	0.9829	1.250556	0.093108
RAPSN	0.954561	0.898716	1.013875	0.130546
AGBL2	1.006937	0.878427	1.154248	0.920951
TC2N	1.020336	0.969121	1.074257	0.443553

CPSF2	1.122575	1.040405	1.211234	0.00287
MOAP1	1.114148	1.021853	1.214779	0.014288
IFI27L1	1.211828	1.088356	1.349308	0.000458
IFI27	1.044487	1.003756	1.08687	0.031979
CLMN	0.916241	0.85728	0.979257	0.009948
PDZRN4	1.177312	1.097503	1.262926	5.17E-06
CCDC38	1.116802	0.994592	1.254028	0.061727
PTER	1.160277	1.072821	1.254863	0.000201
C1QL3	1.158229	1.061933	1.263257	0.000911
CACNB2	1.187455	1.111725	1.268345	3.22E-07
ARL5B	0.954747	0.900192	1.012609	0.122932
TAF1D	0.937373	0.872774	1.006754	0.075866
ABTB2	1.068248	1.009619	1.130281	0.021884
AMOTL1	1.093073	1.035957	1.153337	0.001154
HTRA1	1.317599	1.152498	1.506351	5.39E-05
LIPC	1.266926	1.156453	1.387952	3.72E-07
CEP57	0.923229	0.84572	1.007841	0.0742
TCP11L2	0.998464	0.944411	1.055611	0.956826
SPRED1	1.103055	1.024235	1.18794	0.009514
GPR176	1.437956	1.268413	1.63016	1.39E-08
JAM3	1.049579	0.970128	1.135537	0.228265
CMTM5	1.149518	1.092583	1.20942	7.60E-08
ADAMTS1	0.933068	0.841359	1.034772	0.189377
GPT2	1.087061	1.036527	1.140058	0.000588
AMN	0.910499	0.85902	0.965062	0.001591
RAB8B	0.964909	0.907837	1.025569	0.250829
IKBIP	1.019661	0.93018	1.117751	0.677784
RPUSD2	1.231394	1.114622	1.3604	4.23E-05
HIF1AN	1.027996	0.958139	1.102946	0.441901
NDUFB8	1.24973	1.124944	1.388357	3.27E-05
ZFYVE19	1.201955	1.101011	1.312155	3.96E-05
SPINT1	1.114602	1.059787	1.172253	2.48E-05
FBN1	1.044816	0.991996	1.100447	0.097649
AVPR1A	1.021301	0.904558	1.153111	0.733613
DEPDC4	1.109377	0.958712	1.28372	0.163383
LRTM2	1.139492	1.021998	1.270494	0.01868
BRD7	1.060624	0.956722	1.175809	0.263182
CKB	0.910375	0.868383	0.954398	9.73E-05
TRMT61A	1.061071	0.978141	1.151033	0.153385
BTRC	1.046151	0.972027	1.125928	0.228864
POLL	1.03172	0.930736	1.143661	0.552387
BAG5	1.063856	0.973396	1.162724	0.172177
API5	1.079309	0.991698	1.174661	0.077235
ZNF319	1.028367	0.950888	1.112159	0.483994
HPS6	1.013266	0.942913	1.088869	0.719628
SENP8	1.009356	0.891838	1.142361	0.882771
NOLC1	1.026099	0.954173	1.103446	0.487155
ALKBH3	1.005596	0.909712	1.111586	0.913088
COPS2	0.92863	0.855706	1.007769	0.075981
SGPL1	1.118466	1.037476	1.205779	0.003509
FRS2	1.011485	0.932921	1.096666	0.78192
CCT2	0.972408	0.892277	1.059735	0.523682
PCBD1	1.126235	1.046109	1.212499	0.001594
ARIH1	0.960776	0.895152	1.031211	0.267635
COX11	1.249036	1.125575	1.38604	2.82E-05
ZNF202	1.175019	1.078638	1.280012	0.000221
STXBP4	1.193765	1.080245	1.319214	0.000513
CYYR1	1.129508	1.056877	1.20713	0.000329

CUL5	0.993622	0.913229	1.081093	0.881842
C2	1.066932	0.9985	1.140054	0.055419
PLEKHF1	1.087921	1.01529	1.165748	0.01683
SMPD1	1.141287	1.048802	1.241928	0.002176
APBB1	1.081888	1.024781	1.142178	0.004446
SYNPO2L	1.528067	1.330593	1.754849	1.91E-09
NUDT13	0.94368	0.888983	1.001742	0.057063
TRIM44	1.111723	1.029227	1.200832	0.007097
ILK	1.046322	0.932816	1.173639	0.439589
TAF10	0.983301	0.865947	1.11656	0.795096
TPP1	1.018172	0.949378	1.09195	0.613882
DCHS1	1.010771	0.965946	1.057676	0.643437
CYB5A	1.620566	1.451386	1.809466	9.32E-18
USP54	1.069842	0.990519	1.155517	0.085872
RAG1	1.146604	1.080961	1.216234	5.41E-06
WDR88	1.079552	1.006508	1.157896	0.032239
ATP9B	0.979438	0.908651	1.055741	0.587267
PPFIBP2	1.063617	1.007679	1.122661	0.025256
CYB5R2	1.230932	1.135756	1.334083	4.18E-07
SERPINB8	0.927399	0.880111	0.977227	0.004762
RIC3	1.029219	0.895265	1.183217	0.6856
IDH3A	1.113635	1.019986	1.215882	0.016327
CRABP1	1.145978	1.070587	1.226677	8.69E-05
PLD4	1.050016	1.010724	1.090836	0.012137
ZMAT1	0.954874	0.895602	1.018067	0.157861
XRRA1	1.035121	0.969259	1.105458	0.303436
TRIM66	1.028027	0.960166	1.100684	0.427597
RNF169	1.013205	0.946861	1.084197	0.704187
RPL27A	0.908956	0.846988	0.975459	0.008057
CDYL2	0.945813	0.89866	0.99544	0.032751
CENPN	1.055536	0.948605	1.17452	0.321303
ATMIN	1.07855	0.99258	1.171967	0.074387
TMEM41B	0.97543	0.911049	1.04436	0.475173
LEO1	0.972322	0.887459	1.0653	0.546917
ZNF143	0.982489	0.916776	1.052912	0.616954
TMX3	1.064644	0.985696	1.149915	0.111056
MFAP4	0.841396	0.799358	0.885645	4.01E-11
WEE1	1.086017	1.007919	1.170166	0.030226
MAPK7	0.880737	0.823459	0.941999	0.000214
PRKCB	0.992537	0.933158	1.055694	0.81188
NDST2	1.04904	0.957136	1.149768	0.306101
MCM7	1.079484	0.99267	1.173891	0.073779
CCDC68	1.305485	1.19938	1.420977	7.12E-10
CLEC4E	0.981099	0.936511	1.027811	0.421359
ZNF3	1.075723	0.983941	1.176066	0.108677
CLEC4D	1.030053	0.99082	1.070839	0.135042
ZSCAN21	0.998632	0.897515	1.111141	0.979944
RIMKLB	1.034999	0.965667	1.10931	0.330845
TK2	1.114573	1.028242	1.208151	0.008363
TMED3	1.127922	1.012451	1.256563	0.028924
SEC11C	1.284551	1.187633	1.389378	3.94E-10
TMEM135	1.095954	1.007743	1.191886	0.032345
IQCD	1.052873	0.962175	1.152121	0.262282
NDEL1	0.868015	0.80729	0.933307	0.000131
CENPV	0.96385	0.887468	1.046807	0.382092
RRAD	0.985731	0.93665	1.037384	0.581271
HSP90B1	0.940166	0.870478	1.015432	0.116362
BLCAP	1.145215	1.043438	1.25692	0.004298

CHRFAM7.	1.079042	1.00186	1.16217	0.044533
ATF7IP2	0.993215	0.916034	1.076898	0.868971
TMPRSS5	0.986088	0.897935	1.082896	0.769371
COG1	1.045336	0.956072	1.142934	0.330268
PLEKHA7	1.30716	1.180875	1.446949	2.38E-07
ZNF606	1.056609	0.977312	1.142339	0.16654
B2M	0.982587	0.896851	1.076519	0.706092
ZNF592	0.933278	0.858824	1.014187	0.103552
NNMT	1.052798	0.963952	1.149833	0.252712
ACSM1	0.871798	0.838942	0.905942	2.57E-12
AP1G1	0.948536	0.891828	1.00885	0.09299
SLFN5	1.06175	1.003145	1.123779	0.038607
CATSPER2	1.070042	0.979958	1.168407	0.131361
SAAL1	1.100923	0.990629	1.223498	0.074236
YPEL4	1.000504	0.950557	1.053076	0.984604
PPIB	0.946789	0.858967	1.043591	0.270941
LDHC	0.943941	0.846703	1.052346	0.298289
LDHAL6A	1.15522	1.018228	1.310642	0.025062
FAM111A	1.073221	1.009858	1.14056	0.022851
KIF7	1.055756	0.987305	1.128953	0.11265
LDHD	1.099366	1.029509	1.173964	0.004681
PEX11A	1.185394	1.067948	1.315756	0.001399
TMEM170.	1.015189	0.929544	1.108725	0.737449
MESP1	1.572418	1.366861	1.808887	2.42E-10
ANPEP	0.947603	0.912223	0.984356	0.005569
RBPMS2	1.03155	0.989434	1.07546	0.144146
NAV2	1.162477	1.014267	1.332345	0.0305
ANKDD1A	1.042588	0.97363	1.116429	0.232266
DCTN5	1.015038	0.929487	1.108462	0.739702
TERF2IP	1.117433	1.000647	1.247848	0.048672
PLK1	1.078396	0.998964	1.164145	0.053186
CLPX	0.972266	0.886886	1.065865	0.548664
GPR182	1.079614	0.911466	1.278782	0.375182
ZBTB39	1.018182	0.942635	1.099784	0.646884
TAC3	1.047679	0.957148	1.146772	0.312435
MYO1A	0.95846	0.854964	1.074484	0.466777
NAB2	0.898066	0.845274	0.954156	0.000505
VPS39	1.01723	0.932232	1.109979	0.701181
STAT6	1.006831	0.931344	1.088437	0.864063
PATL1	0.894889	0.824056	0.97181	0.0083
STX3	0.934067	0.868838	1.004193	0.064793
MRPL16	1.045813	0.951478	1.149501	0.353031
PIP4K2C	1.38576	1.281652	1.498324	2.67E-16
MTMR10	0.919857	0.865178	0.977991	0.007546
YWHAB	1.161574	1.050934	1.283862	0.00336
SCG5	1.05587	0.980406	1.137141	0.150738
TSC22D4	0.8675	0.793392	0.948532	0.00181
MS4A7	1.009646	0.963699	1.057784	0.686236
MS4A14	0.956516	0.901275	1.015143	0.142983
DIS3L	1.039595	0.960291	1.125448	0.337492
CCNDBP1	1.107047	1.0054	1.218971	0.038494
EPB42	0.991959	0.957759	1.02738	0.651974
SMAD3	1.126778	1.062958	1.194429	6.01E-05
MAP1A	1.066689	1.019648	1.115901	0.005024
RCCD1	1.101081	1.018459	1.190406	0.015539
AKTIP	1.084487	1.009389	1.165171	0.026746
MAPRE2	0.973916	0.894291	1.06063	0.543618
MBD6	0.918891	0.836197	1.009764	0.078743

CNPY4	1.188032	1.086922	1.298547	0.000147
PDIA3	0.851244	0.789293	0.918057	2.95E-05
NUDT21	1.017946	0.926176	1.118809	0.712136
NKX3-1	1.232311	1.156363	1.313248	1.22E-10
DUSP18	1.137061	1.027461	1.258353	0.012998
TEF	1.140162	1.038277	1.252045	0.006024
MEI1	1.186683	1.08059	1.303193	0.000341
PBX3	1.070224	1.030704	1.111258	0.000407
GNGT2	1.334989	1.231166	1.447567	2.66E-12
PHB	1.176995	1.083507	1.27855	0.000114
SNRPD1	1.144474	1.029709	1.272031	0.012315
TTC16	1.177143	1.015929	1.363939	0.029987
SAMD14	1.197844	1.115201	1.286613	7.45E-07
PIP5KL1	1.079489	1.003306	1.161457	0.040526
TMEM92	1.136062	1.040684	1.240182	0.004354
FAM102A	1.018415	0.96699	1.072575	0.490045
ACSF2	1.105558	1.019863	1.198453	0.014779
GOLGA2	0.927753	0.851708	1.010588	0.085688
TRUB2	1.130885	1.039714	1.23005	0.00413
COQ4	1.077314	0.969542	1.197065	0.166116
SLC27A4	1.120338	1.051424	1.193768	0.000451
URM1	1.031653	0.924863	1.150773	0.576202
CERCAM	0.961531	0.909104	1.016981	0.170269
DOLPP1	1.152712	1.065817	1.246692	0.000379
ENDOG	1.057693	0.979341	1.142314	0.15319
SP2	0.965857	0.877528	1.063076	0.477736
PRR15L	1.196455	1.091877	1.311051	0.000121
COQ7	1.06101	0.966037	1.165319	0.215799
GPRC5B	0.794023	0.628853	1.002576	0.052581
CRK	0.987354	0.913961	1.06664	0.746735
FBXO22	1.075231	0.993653	1.163507	0.071576
TBC1D2B	0.915735	0.864585	0.969911	0.002684
NOD2	1.048978	0.997766	1.102819	0.061155
SNX20	1.135	1.058459	1.217076	0.000378
LOXHD1	0.99466	0.95048	1.040894	0.81734
KATNAL2	1.07245	0.977469	1.17666	0.139322
HDHD2	1.037705	0.941392	1.143872	0.456439
ZNF91	0.942282	0.892966	0.994322	0.030192
RNF214	0.995891	0.896398	1.106426	0.938882
DPEP2	1.004361	0.938097	1.075306	0.900559
POP5	1.062306	0.97959	1.152007	0.143913
CD3D	0.954987	0.911675	1.000357	0.051786
TBC1D16	0.969978	0.911045	1.032724	0.340533
ACAA2	1.020426	0.942571	1.104712	0.61753
STIM1	0.944244	0.871491	1.023071	0.160796
RRM1	1.11277	1.026264	1.206567	0.009658
TRIM68	1.168997	1.074844	1.271397	0.000268
FN3K	1.119564	1.05748	1.185293	0.000104
IRGQ	0.920292	0.871197	0.972155	0.002982
ZNF226	1.178016	1.075508	1.290294	0.00042
ZNF180	1.121979	1.023007	1.230526	0.014576
PPP2R3B	1.185908	1.077149	1.305647	0.000512
ZNF668	1.02503	0.947596	1.108792	0.537323
ZNF646	1.031958	0.959922	1.109399	0.394182
VKORC1	1.400378	1.249918	1.568948	6.37E-09
GNG8	1.009558	0.936736	1.088041	0.803335
LPO	0.823136	0.784305	0.863888	2.92E-15
CA4	0.991516	0.935697	1.050665	0.7732

TPM4	0.972348	0.913427	1.03507	0.379286
RAB8A	1.140642	1.034703	1.257428	0.008148
GPX4	0.979266	0.900767	1.064606	0.623104
MIDN	0.932094	0.867445	1.001562	0.055186
JSRP1	0.973843	0.88621	1.070142	0.581696
KLHL26	0.914225	0.83955	0.995542	0.039139
GATAD2A	0.892471	0.82294	0.967876	0.005979
MVD	1.260306	1.135658	1.398635	1.34E-05
CDT1	0.936372	0.87067	1.007032	0.076532
TRAPPC2L	1.136192	1.007674	1.281102	0.037089
ANKRD11	0.978721	0.8982	1.06646	0.623401
PROCA1	1.068656	0.972012	1.174908	0.169753
RPL13	0.875007	0.809934	0.945309	0.000708
ZNF641	1.080862	1.001326	1.166715	0.046157
CACNB3	1.285285	1.182297	1.397245	3.87E-09
DHRS13	1.094031	1.005262	1.19064	0.037387
TP53I13	1.106186	1.012608	1.208411	0.025235
CORO6	0.922255	0.851513	0.998874	0.046853
RHEBL1	1.187513	1.112921	1.267105	2.08E-07
TUBA1A	0.991304	0.932881	1.053387	0.778099
TUBA1C	1.042434	0.962496	1.129011	0.307292
ZNF610	1.062616	0.970378	1.163623	0.189885
ZNF528	1.096527	1.021212	1.177396	0.011145
ZNF701	1.125788	1.027901	1.232997	0.010683
SERTAD3	1.003552	0.924882	1.088913	0.932156
RAB4B	1.238269	1.086272	1.411534	0.001382
GPD1	1.012916	0.908351	1.129518	0.817429
CYP2S1	0.955116	0.914109	0.997962	0.04026
AXL	0.951686	0.893591	1.013558	0.12334
NFKBID	0.980247	0.920759	1.043579	0.53225
TMC4	0.935376	0.864393	1.012188	0.097093
LAIR1	0.964614	0.905145	1.02799	0.267132
TTYH1	1.122969	1.01003	1.248538	0.031993
LENG8	0.936236	0.882994	0.992688	0.02741
CDC42EP5	0.94442	0.82777	1.077508	0.395241
LAIR2	1.047042	0.964999	1.136059	0.269518
ZNF526	1.012646	0.933059	1.099021	0.763482
TRAPPC9	1.042143	0.967326	1.122746	0.277481
KIR3DL1	1.141317	0.984768	1.322752	0.07908
ZNF146	0.999453	0.930618	1.073381	0.988017
ZNF283	1.137181	1.038981	1.244663	0.005273
PPP1R14A	0.956227	0.898949	1.017155	0.155538
SPINT2	1.221518	1.140552	1.308231	1.08E-08
YIF1B	1.120473	1.025458	1.224293	0.011869
PSCA	1.066742	0.963696	1.180807	0.212575
ATCAY	0.932141	0.827354	1.050198	0.248106
DAPK3	0.91072	0.843222	0.983622	0.0173
EEF2	0.845712	0.785292	0.910781	9.38E-06
TMIGD2	0.868033	0.831337	0.906349	1.35E-10
CHAF1A	1.097451	1.010585	1.191785	0.027089
UBXN6	0.940743	0.848736	1.042724	0.244723
SEMA6B	1.04688	0.999724	1.096261	0.051387
ZNF444	1.123857	1.023127	1.234505	0.014802
NXN	1.116118	1.034891	1.20372	0.004378
GLOD4	1.085188	0.982029	1.199184	0.108682
MFSD3	1.101432	1.038212	1.168502	0.001358
GPT	1.092686	1.020524	1.169951	0.010997
KIFC2	1.076276	1.011074	1.145683	0.021146

SLC43A2	1.062703	1.004244	1.124565	0.035146
RILP	1.023726	0.941574	1.113045	0.582727
SERPINF2	0.959693	0.87662	1.050637	0.373125
WDR81	0.997449	0.937286	1.061475	0.935873
SRR	1.012629	0.916294	1.119092	0.805638
TSR1	1.079734	0.986979	1.181206	0.094138
HSD11B1L	0.990231	0.91314	1.073829	0.812336
CYB5D2	1.037269	0.938254	1.146734	0.474702
KLK1	1.40917	1.287479	1.542363	9.80E-14
ZNF83	1.061185	0.986372	1.141671	0.111361
KRT80	0.781374	0.687804	0.887675	0.00015
KRT1	0.970227	0.922333	1.020608	0.241913
OTUB1	1.138046	0.996645	1.299509	0.056091
RCOR2	1.078623	1.003802	1.159021	0.039073
ANGPTL4	0.954171	0.888846	1.024298	0.194809
CD320	0.978241	0.906462	1.055705	0.571542
SPRYD3	1.112749	1.027236	1.205381	0.008829
IGFBP6	0.907778	0.839383	0.981746	0.015481
ZNF558	1.107219	1.01234	1.21099	0.025862
NDUFV1	1.179197	1.054353	1.318825	0.00389
CDK2AP2	0.976326	0.901652	1.057184	0.555071
NUDT8	1.113745	1.029566	1.204808	0.007218
TBX10	1.154091	1.052875	1.265038	0.002212
PRDX2	1.02465	0.979521	1.071859	0.289326
ZNF232	1.274505	1.172061	1.385904	1.40E-08
MIS12	1.131383	1.022769	1.251532	0.016523
CD300C	1.055198	1.009078	1.103425	0.018457
CD300A	1.165807	1.100376	1.235129	1.93E-07
TMEM88	0.930567	0.865576	1.000438	0.051402
EVPL	0.861283	0.818733	0.906043	7.61E-09
SRP68	0.989827	0.897582	1.091552	0.837685
MGAT5B	1.24286	1.152833	1.339917	1.45E-08
TMC8	1.05528	0.98203	1.133993	0.142666
TK1	1.146602	1.061159	1.238925	0.000535
TMEM68	1.17043	1.038202	1.319497	0.010085
TMEM99	1.432178	1.243807	1.649079	5.97E-07
GHDC	1.066889	0.98167	1.159506	0.127406
ZNF598	0.861142	0.802857	0.923658	2.90E-05
RAB26	0.965679	0.914043	1.020231	0.212908
MLST8	0.990972	0.887286	1.106775	0.872233
E4F1	1.118381	1.023095	1.222541	0.013797
DNASE1L2	1.083281	0.977417	1.200612	0.127353
CASKIN1	0.804619	0.699218	0.925909	0.002409
ABCA3	1.128484	1.048459	1.214617	0.001278
KCTD5	1.07871	0.996711	1.167455	0.060342
SRRM2	0.897585	0.825068	0.976476	0.011943
ZNF597	0.991593	0.90985	1.080679	0.847472
NLRC3	1.041261	0.984517	1.101275	0.157305
SDHAF2	1.12955	1.003519	1.271408	0.043574
DDB1	1.003822	0.922391	1.092441	0.929583
VPS37C	1.043694	0.974115	1.118243	0.224397
VWCE	0.897689	0.83932	0.960117	0.001652
RAB3IL1	0.955023	0.896294	1.017599	0.155261
BEST1	1.028723	0.960253	1.102075	0.420348
FTH1	0.938179	0.883829	0.995871	0.036096
BSCL2	1.064334	0.966533	1.17203	0.204869
POLR2G	1.147183	1.01519	1.296338	0.027686
SLC3A2	0.975504	0.888937	1.070502	0.600924

ATG16L2	1.029344	0.96819	1.094361	0.35471
C2CD3	1.044924	0.964021	1.132616	0.285172
TTC21A	0.94175	0.884929	1.002218	0.058735
RPSA	0.877718	0.809745	0.951397	0.001517
CTNNB1	0.941791	0.885409	1.001764	0.056908
ULK4	1.060786	0.990717	1.135812	0.090555
FADD	0.950691	0.870089	1.038759	0.263271
LTBP3	0.942742	0.885678	1.003482	0.064192
NAALADL1	0.960519	0.896649	1.028939	0.251233
SAC3D1	1.122353	1.049306	1.200485	0.000775
BATF2	1.292135	1.150198	1.451587	1.58E-05
SF1	0.866168	0.791348	0.948063	0.001827
MAP4K2	0.983998	0.907628	1.066794	0.695543
CCDC88B	1.041524	0.979118	1.107907	0.196857
SCARA3	0.983471	0.901777	1.072566	0.706404
PBK	1.15215	1.081561	1.227346	1.13E-05
PNOC	1.157444	1.048907	1.277213	0.00361
COPS6	1.247753	1.125419	1.383385	2.62E-05
PAFAH1B2	0.9729	0.904393	1.046596	0.460832
ANKS3	1.031668	0.956307	1.112969	0.420486
NUDT16L1	1.149397	1.032543	1.279475	0.010915
KIAA1586	1.019878	0.95394	1.090375	0.56381
RAB4A	0.847142	0.769905	0.932128	0.000672
OR2B2	0.94934	0.844798	1.066819	0.382466
SETD5	0.933944	0.871675	1.000661	0.052234
VASN	0.978661	0.925093	1.035331	0.452627
THAP9	0.948081	0.862808	1.041782	0.267546
RNF187	1.113027	1.016836	1.218317	0.020233
HOOK3	1.020308	0.93793	1.109922	0.639735
MAPK1IP1	0.964189	0.871301	1.06698	0.480449
DDIT4	1.119839	1.067173	1.175104	4.12E-06
RBPJ	0.999325	0.926142	1.078292	0.986119
LMBRD1	0.947016	0.869164	1.031842	0.213577
ZCCHC4	1.045473	0.954592	1.145008	0.337857
PTGDR	1.04688	0.943154	1.162013	0.389463
TTC39C	1.131001	1.04205	1.227546	0.003224
GLYCTK	1.039865	0.969322	1.115542	0.275426
GNG4	0.968949	0.837722	1.120733	0.670962
UBTD2	1.064234	0.992088	1.141627	0.082179
POLR2J3	1.042748	0.953108	1.14082	0.361376
NKIRAS2	0.881015	0.792961	0.978847	0.018379
DNAJC7	0.942728	0.875575	1.015032	0.117762
KCNV2	0.991121	0.89295	1.100085	0.866908
IRF2BP2	1.108506	1.012557	1.213546	0.025739
NT5DC2	1.064831	1.009519	1.123173	0.020996
FOXI1	0.961148	0.859407	1.074932	0.487569
BMI1	1.183257	1.11422	1.256572	4.11E-08
THAP11	1.043936	0.955934	1.14004	0.338583
MMADHC	0.97809	0.884206	1.081942	0.666989
PDHB	1.049228	0.955291	1.152401	0.315296
PXK	0.863118	0.803931	0.926661	4.88E-05
PCMTD1	0.984368	0.912667	1.061702	0.683043
KCTD6	1.064879	0.980271	1.15679	0.136695
ACOX2	1.050396	0.936968	1.177556	0.399066
IRF2	1.049151	0.982849	1.119925	0.149706
CX3CR1	1.093323	1.0513	1.137027	8.13E-06
XIRP1	0.918801	0.849599	0.993641	0.034036
DEGS2	0.996422	0.922013	1.076836	0.927877

ARF4	0.836583	0.76258	0.917768	0.000159
FILIP1L	0.983231	0.926983	1.042891	0.573657
DTYMK	1.213904	1.107313	1.330757	3.57E-05
TAP1	1.0572	0.987692	1.131598	0.108922
ING5	1.031068	0.951924	1.116792	0.452752
ATG4B	1.042576	0.947869	1.146747	0.390839
MLKL	1.054448	0.981427	1.132901	0.147631
RFWD3	1.002896	0.933476	1.077478	0.937025
RHOH	0.944735	0.897469	0.99449	0.029934
KLHL30	1.02543	0.906724	1.159677	0.689114
COG7	0.996977	0.913437	1.088158	0.945947
CDC40	0.955086	0.885837	1.02975	0.231464
STIP1	1.032398	0.934522	1.140525	0.530391
HR	0.854574	0.757582	0.963984	0.010566
RAB31	1.054112	1.009029	1.101209	0.018128
REEP4	1.070852	0.981303	1.168574	0.12445
TNXB	1.030998	0.970196	1.095611	0.324946
BMP1	1.050553	0.976872	1.129792	0.183762
ATXN2L	0.859192	0.784515	0.940977	0.00107
POLR3D	1.009336	0.934072	1.090665	0.814185
FEN1	1.232028	1.135525	1.336732	5.33E-07
HEXIM2	1.00352	0.940892	1.070316	0.914896
FNTA	0.984932	0.902344	1.075079	0.734016
SERINC2	1.10998	1.053383	1.169618	9.32E-05
COL3A1	1.090773	0.986905	1.205573	0.088794
ING2	1.074966	0.963763	1.199	0.194467
CDKN2AIF	1.067063	0.985343	1.15556	0.110325
SNRNP48	1.052165	0.955688	1.15838	0.300068
SLC20A2	1.075619	0.987985	1.171026	0.092728
TMUB2	0.984422	0.882271	1.098399	0.778794
STAT3	0.988103	0.915994	1.065888	0.756888
ZSWIM1	1.109916	1.004754	1.226085	0.040039
ADAM9	0.924464	0.876477	0.975078	0.003878
AXIN2	1.070922	0.971778	1.180182	0.166849
NDUFS5	0.999244	0.933027	1.07016	0.982749
ZNF30	1.054523	0.963212	1.15449	0.250615
UGT3A2	0.944048	0.893634	0.997307	0.039756
SLC16A4	1.0225	0.965499	1.082866	0.447096
IL7R	1.000832	0.957149	1.046509	0.970861
TMEM208	1.201875	1.061769	1.36047	0.003641
AHCYL1	1.085234	0.997482	1.180705	0.057254
DNAJC21	1.005139	0.909656	1.110644	0.91983
PKIG	0.985465	0.924179	1.050816	0.654924
FAM178B	1.069505	1.007013	1.135875	0.028708
SEMA4C	1.038723	0.978238	1.102949	0.214544
CNNM3	1.066783	0.989324	1.150308	0.092786
GSTM4	1.265507	1.160058	1.380541	1.13E-07
TET2	0.92377	0.864704	0.986872	0.018675
TCTN2	1.086576	0.989542	1.193125	0.081913
SHOX2	1.184301	1.107299	1.266658	8.17E-07
TSPAN5	1.063468	1.008591	1.121331	0.022821
ABHD15	1.156052	1.085146	1.231591	7.11E-06
ZBTB5	0.955248	0.889274	1.026117	0.209887
CHTF8	1.111242	0.88087	1.401863	0.373543
ADAL	1.227874	1.101062	1.36929	0.000223
LCMT2	1.312612	1.199149	1.436811	3.70E-09
SNTB2	1.110841	1.021145	1.208416	0.014402
IL12A	1.142041	1.055391	1.235805	0.00097

ZNF507	1.113983	1.038042	1.195478	0.002732
STX18	0.904125	0.822726	0.993577	0.036276
GFM1	1.124249	1.024566	1.233631	0.013426
DDX19A	1.015811	0.92164	1.119605	0.751971
ANKRD49	1.054797	0.96421	1.153894	0.244242
SFTPB	0.944606	0.824959	1.081606	0.40954
USP39	0.938591	0.849857	1.03659	0.211029
TNIP2	0.986272	0.902931	1.077306	0.758944
TMEM150,	0.971785	0.897268	1.052491	0.48198
RNF181	0.949637	0.855132	1.054587	0.33394
VAMP5	0.876843	0.818189	0.939702	0.000199
LRRC28	0.925866	0.856145	1.001264	0.053814
MAT2A	0.972355	0.924153	1.023071	0.279832
ENHO	0.789692	0.699737	0.891211	0.00013
ZNF608	0.873449	0.829475	0.919753	2.84E-07
INPP5D	0.948306	0.879228	1.022811	0.168981
LETM1	1.088749	1.009421	1.174312	0.027602
TMEM129	1.002322	0.928777	1.081691	0.952435
PPIC	1.283307	1.15813	1.422015	1.90E-06
SPRY3	1.064091	0.943247	1.200416	0.312492
CEP120	1.04143	0.953854	1.137047	0.365043
STXBP6	1.167139	1.073201	1.269301	0.000306
MFF	1.082969	0.964343	1.216187	0.178119
LGALS9	0.98368	0.916211	1.056118	0.649921
SIGLEC7	1.070625	1.013567	1.130895	0.014597
E2F6	1.082206	0.992354	1.180193	0.074033
FEM1B	0.985943	0.906705	1.072106	0.740505
COMMD8	1.003488	0.931769	1.080728	0.926662
UQCRFS1	0.925873	0.834384	1.027393	0.146815
MAP2K1	0.954467	0.883257	1.031417	0.23879
HNRNPH1	0.970954	0.904767	1.041982	0.413189
IRS1	1.06348	0.973647	1.161601	0.171671
MECP2	0.98189	0.91237	1.056707	0.625696
UPF3A	1.293877	1.155037	1.449407	8.64E-06
ROR2	0.998114	0.902946	1.103312	0.970545
BRD7P3	1.020252	0.957942	1.086615	0.5329
AR	0.860507	0.80314	0.921972	1.97E-05
DHRSX	1.005666	0.915462	1.104759	0.90619
HSPBAP1	0.979724	0.911322	1.053261	0.579084
ASMTL	1.004696	0.918348	1.099162	0.918618
SLC25A6	0.94068	0.860833	1.027933	0.176625
CHST14	1.020989	0.952377	1.094545	0.558392
PARM1	1.094691	0.93513	1.281478	0.260352
CSNK1G1	1.013321	0.939727	1.092678	0.730857
FAM110B	1.045626	0.984079	1.111021	0.149457
AFAP1L2	1.326686	1.215133	1.448479	2.82E-10
ZNF354A	1.147654	1.046804	1.258219	0.003339
ATF5	1.104186	1.01778	1.197927	0.017131
UBE2V2	1.149622	1.048419	1.260595	0.003021
ZBTB43	0.983821	0.929881	1.040889	0.570734
XPO6	1.017625	0.939505	1.102242	0.668119
MN1	0.921434	0.88745	0.956719	1.98E-05
APEX2	1.209184	1.095664	1.334466	0.000159
NSMCE1	1.102163	0.99891	1.21609	0.052595
CCDC126	1.1458	1.049263	1.25122	0.002439
IL13	1.027991	0.911328	1.159589	0.653303
CD2BP2	1.071143	0.978617	1.172416	0.135956
RGS14	1.119075	1.04605	1.197197	0.001085

TBC1D10B	0.974169	0.895487	1.059764	0.542484
LMAN2	1.079586	0.974213	1.196356	0.143908
RAB24	1.332096	1.206398	1.47089	1.42E-08
PRELID1	1.140003	1.041204	1.248178	0.004612
THBS3	1.024725	0.948077	1.10757	0.538056
CA5B	1.115189	1.016569	1.223376	0.021009
EFNA1	0.983676	0.924892	1.046195	0.600612
CXCL10	1.066304	1.013378	1.121995	0.013452
SH3TC2	1.367463	1.255977	1.488845	5.49E-13
CXCL11	1.143591	1.054547	1.240152	0.001178
ZRSR2	1.005109	0.892762	1.131595	0.932842
NMD3	0.932011	0.85512	1.015818	0.108994
ADRB2	1.032553	0.986348	1.080923	0.170234
B3GALNT1	1.056899	0.978043	1.142111	0.161877
GPRIN1	1.328145	1.221666	1.443905	2.82E-11
KCNAB1	0.998711	0.936119	1.065488	0.968837
MRPL1	1.00454	0.917831	1.09944	0.921663
SHE	0.955355	0.854384	1.068257	0.422904
PGM2	1.043699	0.968165	1.125126	0.264467
P2RY12	1.201249	1.144883	1.260391	7.56E-14
SLC33A1	1.048211	0.963415	1.140471	0.273956
SNUPN	1.141353	1.021177	1.275671	0.019852
CRADD	1.222214	1.13679	1.314058	5.70E-08
SIN3A	0.988489	0.910881	1.07271	0.781381
ARL13B	1.288344	1.146736	1.447439	2.00E-05
RNASE2	0.980731	0.951998	1.01033	0.199652
RNASE3	0.980363	0.952885	1.008634	0.171537
PTK2	1.073644	1.019299	1.130887	0.007335
PTAFR	1.066992	1.023537	1.112292	0.002239
PTPN9	1.13363	1.037824	1.238279	0.005368
RNASE6	0.999119	0.961747	1.037943	0.96386
SCN9A	1.185216	1.119145	1.255189	6.39E-09
SDC2	1.087738	1.041755	1.13575	0.000136
CD52	0.992301	0.956927	1.028983	0.676449
MMGT1	0.883597	0.812661	0.960724	0.003751
TM2D2	1.072235	0.959826	1.197809	0.217086
HTRA4	1.035889	0.950817	1.128574	0.419979
PLEKHA2	0.946804	0.879252	1.019547	0.14779
CLIC4	0.973346	0.917025	1.033127	0.374359
GPR183	0.937933	0.890256	0.988162	0.016069
CCDC8	0.867712	0.772601	0.974531	0.016598
ZEB2	1.004158	0.94714	1.064608	0.889356
PCBP1	0.912301	0.836751	0.994673	0.037427
HINT1	0.884662	0.81048	0.965634	0.006096
DTWD2	1.039681	0.95803	1.128291	0.351078
VPREB1	1.081604	1.034145	1.131242	0.000611
CLIC3	1.137711	1.061145	1.219801	0.000284
INO80E	1.153769	1.035015	1.286148	0.009852
DFFB	1.007879	0.941447	1.078998	0.821517
NFU1	0.975737	0.862007	1.104472	0.697678
CKAP2L	1.188304	1.109836	1.27232	7.43E-07
APLF	0.975397	0.904338	1.05204	0.518629
BOLA2B	1.212783	1.050385	1.400288	0.008535
RGPD8	0.938657	0.85675	1.028395	0.17417
HIC2	1.052614	0.983437	1.126656	0.139298
LUZP1	0.868393	0.823442	0.915797	1.95E-07
BUB1	1.092724	1.023894	1.16618	0.007555
SPNS1	1.02795	0.93489	1.130273	0.56911

LRRC45	1.103092	1.030314	1.181011	0.00484
AGPAT2	0.92165	0.856079	0.992243	0.030253
ASPSCR1	1.103647	1.004271	1.212856	0.040512
GP9	1.059474	1.014357	1.106597	0.009268
FASN	1.011998	0.952012	1.075764	0.70204
CNBP	1.062443	0.957291	1.179145	0.25466
MT1E	1.152807	1.105603	1.202027	2.63E-11
DUS1L	1.100564	0.988924	1.224806	0.079109
GPS1	1.117406	1.007081	1.239817	0.03635
RFNG	1.044435	0.931799	1.170687	0.455229
DCXR	1.319099	1.210915	1.436948	2.25E-10
ZNF32	1.178665	1.071206	1.296903	0.000751
LDB2	1.276107	1.096891	1.484603	0.00159
RAC3	0.992023	0.925088	1.063801	0.822206
NRG4	1.119508	1.066299	1.175371	5.53E-06
LIMS1	1.042399	0.962072	1.129432	0.310148
TAPT1	0.983888	0.927328	1.043899	0.590776
UGP2	1.019745	0.944287	1.101234	0.618141
HNRNPF	0.970336	0.885564	1.063222	0.518524
BTD	0.979556	0.905625	1.059522	0.605919
CSGALNA(	0.929945	0.86728	0.997136	0.041298
PCDH7	1.143623	1.065216	1.227802	0.000213
ROBO1	0.930415	0.88273	0.980675	0.007211
AVEN	0.836579	0.760239	0.920586	0.000257
P2RY1	1.062628	1.010371	1.117588	0.018227
CTNND2	1.164748	1.083753	1.251796	3.37E-05
TRIM56	1.047272	0.974176	1.125852	0.210855
AHSP	0.984428	0.957034	1.012606	0.275732
WNT10B	1.10971	1.044596	1.178884	0.00074
REPS2	0.991251	0.91156	1.077908	0.837176
SYAP1	0.925171	0.847456	1.010014	0.08232
ITGAM	1.078996	1.034001	1.125948	0.000468
PYDC1	0.974583	0.916947	1.035843	0.407816
TPST1	0.995738	0.94381	1.050522	0.875795
TOR1AIP2	0.922249	0.863728	0.984735	0.015527
S100G	0.986452	0.888958	1.094638	0.797245
TM4SF1	0.95806	0.912717	1.005655	0.083271
OTUD3	1.069655	0.985752	1.1607	0.106174
GUSB	0.984513	0.90166	1.07498	0.727851
BRD3	1.044454	0.950201	1.148056	0.367396
KLF13	1.014751	0.947087	1.087249	0.677482
ZFPM2	1.126215	1.044891	1.213867	0.001882
ZNF764	1.01739	0.926708	1.116945	0.717392
ZNF747	1.125661	1.0181	1.244585	0.020887
ZNF768	1.04163	0.968569	1.120202	0.27166
TAS1R3	1.027841	0.944691	1.118309	0.523468
TMEM42	1.18715	1.077313	1.308185	0.000533
MAP3K2	0.935935	0.8767	0.999171	0.047165
PUSL1	1.120183	1.005721	1.247672	0.039047
SF3B5	0.995001	0.917583	1.078951	0.903477
ZNF35	0.967533	0.906858	1.032267	0.31785
IFFO2	1.083234	1.023322	1.146654	0.005885
NLGN2	1.101235	1.031404	1.175793	0.003913
MYO7B	1.047721	0.992407	1.106118	0.092074
CHD3	1.156256	1.070111	1.249336	0.000238
TMEM154	1.056162	0.997811	1.117924	0.059511
MYRIP	1.140781	1.049613	1.239867	0.001939
ALCAM	1.047676	0.992372	1.106061	0.09233

YWHAG	0.981328	0.919552	1.047253	0.569906
UBE2E3	1.007014	0.950996	1.066333	0.810823
CNTROB	0.937249	0.871507	1.00795	0.080717
TRAPPC1	1.078058	0.987667	1.176722	0.092526
KCNAB3	1.113215	1.010069	1.226895	0.030625
FAM153A	1.166079	0.993383	1.368798	0.060274
GPR37L1	0.970237	0.895716	1.050957	0.458678
TMEM192	1.091817	0.992083	1.201576	0.072282
ZNF778	1.084672	0.989092	1.189488	0.08418
NIPA1	1.165867	1.107626	1.22717	4.37E-09
FOXD4	1.167394	1.049697	1.298288	0.004311
GPR25	1.1299	1.069092	1.194167	1.51E-05
UBE2E1	0.873793	0.817794	0.933627	6.54E-05
HNRNPA3	0.940148	0.857741	1.030471	0.187286
SIK2	1.009569	0.929649	1.09636	0.820946
RNF150	1.365379	1.269786	1.468168	4.12E-17
CCDC144/	0.999405	0.934847	1.06842	0.986053
CHRNB1	0.988841	0.912451	1.071626	0.784416
GYPA	1.015461	0.973556	1.05917	0.475498
USP38	1.006638	0.933859	1.085089	0.862814
SLC16A5	0.969401	0.900752	1.043282	0.406941
NANP	1.157683	1.050818	1.275415	0.003046
PWWP2A	1.058587	0.966767	1.159128	0.218743
USP50	0.93805	0.858763	1.024657	0.155793
USP47	0.975536	0.907134	1.049096	0.504283
PDCD6IP	1.001466	0.915554	1.09544	0.974466
ZNF212	1.023931	0.947269	1.106797	0.551433
MRAP	1.073467	0.942393	1.222771	0.285984
FAM161A	1.136476	1.020853	1.265194	0.01944
ZNF282	0.938752	0.870032	1.0129	0.103207
GLB1	1.043311	0.961138	1.13251	0.311071
CRTAP	0.924326	0.853551	1.000969	0.052852
CNGB3	1.320906	1.149157	1.518323	8.99E-05
CMTM8	0.953886	0.88726	1.025515	0.201264
GABARAP	0.955427	0.871069	1.047955	0.333648
STX8	1.136336	1.028011	1.256074	0.012404
UBB	0.985997	0.893484	1.088088	0.779061
NFRKB	0.999988	0.921588	1.085059	0.999779
FABP4	1.047457	0.960011	1.142868	0.297217
PRDM10	0.946612	0.878873	1.019571	0.147525
B3GNT2	1.006236	0.935297	1.082556	0.867633
FOS	0.99969	0.945877	1.056565	0.991249
TMED10	1.144405	1.048178	1.249465	0.002613
SETMAR	1.033966	0.947972	1.127761	0.450886
SMAD1	0.993389	0.933613	1.056991	0.834065
EMX2	1.075129	0.978466	1.181342	0.131792
SP7	1.104432	1.033045	1.180753	0.003573
LRRN2	1.087426	1.015304	1.164671	0.016677
SLC30A1	1.043917	0.978252	1.113991	0.19476
DCLK2	1.048884	0.980827	1.121663	0.163205
ZNF804A	0.879059	0.823258	0.938642	0.000117
GPRC5C	1.080318	1.002502	1.164175	0.042818
TMEM182	1.19833	1.076142	1.334392	0.000976
KRT8	1.014837	0.957893	1.075166	0.617161
ADORA2B	1.008134	0.938013	1.083498	0.825675
MGMT	0.949232	0.877303	1.027059	0.195013
METTL7B	1.129098	1.090311	1.169265	9.91E-12
NFXL1	0.999916	0.924714	1.081233	0.998316

DENND5B	0.930979	0.867101	0.999563	0.048608
CD14	1.010701	0.979769	1.04261	0.50209
DNAJC18	1.025195	0.937661	1.1209	0.584766
RALGAPB	1.00025	0.929569	1.076305	0.99467
SLC23A1	0.971311	0.906535	1.040715	0.408436
KRT72	1.062272	0.988585	1.141453	0.099566
KISS1	1.179878	1.091713	1.275163	2.99E-05
NUDT9	1.118148	1.011651	1.235857	0.028758
HSD17B13	0.895273	0.828086	0.967911	0.005446
PA2G4	1.088665	0.977775	1.212132	0.121163
ELOVL6	1.1024	1.033169	1.176269	0.003219
PFKFB3	0.981765	0.930538	1.035813	0.500909
ARL6IP1	1.0012	0.921843	1.087388	0.977298
SERPINB9	0.967779	0.921865	1.015979	0.1866
SMAGP	0.987817	0.920638	1.059898	0.733017
IRX1	0.875303	0.834966	0.917589	3.15E-08
CDH2	1.089565	0.988279	1.201231	0.084866
EMB	1.046182	0.987681	1.108149	0.124107
STAT2	1.071257	1.002191	1.145082	0.042935
NUDCD2	0.963582	0.873144	1.063388	0.460672
IRF2BP1	0.982683	0.913121	1.057543	0.640957
HSPA4	1.035042	0.94303	1.136031	0.468396
COMMD5	1.130709	1.026847	1.245075	0.012459
GTSF1	0.947095	0.912626	0.982865	0.004057
DPY19L2P	1.069241	1.003981	1.138744	0.037195
ZNF16	1.13813	1.015974	1.274975	0.025514
ARMC10	1.011684	0.934511	1.095229	0.774167
RNF34	1.054708	0.972982	1.143299	0.195537
ACYP2	1.112467	0.975915	1.268126	0.110692
TRABD	1.089253	1.00109	1.18518	0.047114
ATF7	0.960533	0.871811	1.058284	0.415452
SOCS6	0.899506	0.842889	0.959926	0.001408
ZNF296	1.039163	0.957672	1.127589	0.356544
HOXB9	1.036606	1.004062	1.070205	0.027173
TTLL6	1.0872	0.989482	1.194568	0.081872
POLH	1.071048	0.994223	1.15381	0.070698
KIF5B	0.969541	0.894288	1.051126	0.453022
AKAP13	0.955994	0.897762	1.018003	0.160464
CDCA4	1.043916	0.96281	1.131855	0.297627
CHCHD7	1.269665	1.159176	1.390685	2.75E-07
HTRA3	1.096756	1.052216	1.143182	1.26E-05
FOXN2	1.010924	0.931282	1.097377	0.795245
LMOD2	0.979989	0.924897	1.038363	0.493509
USP32	1.03564	0.964578	1.111937	0.33426
CEL	1.214775	1.125376	1.311276	6.09E-07
PPM1D	0.986667	0.914676	1.064323	0.728404
GPR27	1.019408	0.981595	1.058677	0.318899
KBTBD2	0.986873	0.914004	1.065552	0.735647
TRIAP1	0.952281	0.868873	1.043695	0.295794
LSM3	1.054349	0.95699	1.161613	0.284337
KIAA0232	0.980774	0.911933	1.054811	0.601083
MTSS1	0.924936	0.879759	0.972433	0.002257
TMEM43	0.995212	0.916944	1.080162	0.908578
RNF139	0.983476	0.907168	1.066201	0.68595
RPS9	0.935064	0.841121	1.0395	0.213925
PLA2G1B	0.959918	0.859464	1.072113	0.468253
CYTL1	0.930745	0.904526	0.957724	8.53E-07
TSEN34	1.290662	1.153228	1.444474	8.92E-06

TRH	0.897206	0.873653	0.921394	1.33E-15
GSTA4	1.336005	1.240316	1.439076	2.18E-14
NDUFA3	1.134309	1.052761	1.222173	0.000931
OSCAR	0.950876	0.914729	0.988453	0.010856
PAQR8	1.129828	1.070069	1.192925	1.07E-05
NUDT6	1.228748	1.098281	1.374714	0.000322
TANC2	1.169363	1.095618	1.248072	2.51E-06
DNAJC24	1.196321	1.071834	1.335265	0.001387
ZNF160	1.02601	0.952387	1.105324	0.49911
ZNF415	1.025175	0.932926	1.126546	0.605286
CEACAM3	1.067052	1.018615	1.117792	0.00618
PDGFD	1.241448	1.170422	1.316783	6.24E-13
S1PR1	0.936969	0.892437	0.983723	0.00878
PKIA	1.138116	1.06027	1.221677	0.000345
XKR6	1.16351	1.055134	1.283018	0.002399
TSNARE1	1.055956	0.988072	1.128504	0.108272
FPR2	1.050674	0.996975	1.107264	0.064779
FPR1	1.000418	0.966108	1.035948	0.981253
FEZ2	1.017229	0.937124	1.104181	0.683137
SOX7	1.116962	0.962575	1.296113	0.145007
ALK	1.008931	0.882365	1.15365	0.896561
MTM1	0.940441	0.866791	1.020349	0.139992
INSR	1.040148	0.983465	1.100098	0.168573
MFN1	0.986694	0.921679	1.056296	0.700119
GIMAP8	1.037781	0.974341	1.105352	0.2492
NRTN	1.034591	0.937808	1.141362	0.497387
KCNMB3	0.983899	0.875099	1.106226	0.786023
FUT3	1.022333	0.955405	1.09395	0.522572
ATP6V0E2	1.33178	1.219855	1.453976	1.58E-10
PRKCE	1.091427	1.026768	1.160158	0.004989
JAGN1	1.149098	1.040033	1.2696	0.006306
TADA3	1.227084	1.11753	1.347377	1.80E-05
SOCS5	0.90538	0.832235	0.984953	0.020738
C1GALT1C	1.115794	1.03393	1.204139	0.004829
MORN4	1.527676	1.374622	1.697771	3.63E-15
ZNF672	0.999533	0.926174	1.078702	0.99041
ZNF692	1.113989	1.038255	1.195248	0.002656
NAIF1	1.086191	0.977399	1.207093	0.124681
RBKS	1.066344	0.990333	1.148189	0.088663
GRIK1	0.939592	0.856105	1.03122	0.189376
TMEM126A	0.868091	0.79317	0.950088	0.002128
TMEM126I	1.130417	1.014826	1.259175	0.025923
TRIM8	1.222869	1.134109	1.318577	1.66E-07
NETO2	0.928766	0.86603	0.996047	0.038362
CLDN20	0.995664	0.941182	1.053299	0.879687
CDC42BPC	1.062076	0.987454	1.142338	0.105171
SCAND1	0.986928	0.888435	1.09634	0.806224
JUNB	0.942821	0.879511	1.010689	0.096879
TMEM37	0.94493	0.779874	1.144918	0.563058
LRG1	1.11984	1.062787	1.179956	2.21E-05
SHCBP1	1.067464	0.997767	1.142029	0.058083
SOSTDC1	0.91685	0.811896	1.03537	0.16164
NPTX1	0.908284	0.82672	0.997896	0.045089
FAM98B	1.026678	0.95892	1.099223	0.449777
ZNF439	0.942683	0.890813	0.997573	0.040941
ZNF440	0.971974	0.908546	1.039829	0.409023
GAA	1.025051	0.969191	1.08413	0.386821
CANT1	1.026276	0.951233	1.107239	0.5032

ZDHHC16	1.047848	0.971044	1.130726	0.228819
CHST11	0.958968	0.888128	1.035458	0.284593
EXOSC1	1.115157	0.962124	1.292532	0.147823
PGAM1	1.062186	0.969631	1.163575	0.194643
CHD7	0.963042	0.913919	1.014806	0.158613
ESCO2	1.230298	1.135944	1.332489	3.56E-07
KRT19	0.895562	0.678755	1.181622	0.43543
CLCN5	1.351147	1.25356	1.456332	3.60E-15
TPPP	0.929011	0.85578	1.008509	0.078797
KCND3	0.990326	0.874301	1.121747	0.878471
APLN	0.810197	0.734775	0.89336	2.42E-05
PDE7B	1.164835	1.057581	1.282967	0.001962
MRPL36	1.230684	1.119579	1.352815	1.71E-05
ZNF581	0.845955	0.773436	0.925274	0.000254
NAT1	1.244464	1.116627	1.386936	7.66E-05
GLOD5	0.959914	0.898407	1.025632	0.225945
ZNF524	0.918118	0.835313	1.009132	0.076483
MCC	1.004589	0.92421	1.091959	0.914314
ZBTB26	0.990371	0.92109	1.064863	0.79371
DSEL	1.237648	1.125383	1.361113	1.11E-05
POLR1C	1.205353	1.09532	1.32644	0.000131
ASXL1	0.955406	0.882255	1.034622	0.261654
DLK2	1.079851	0.989027	1.179015	0.08656
ZNF562	1.002233	0.930534	1.079456	0.953036
ZNF318	1.095032	1.015241	1.181094	0.018682
ZNF561	0.977905	0.897565	1.065436	0.609477
WIPF2	1.056641	0.956412	1.167375	0.278581
HOPX	1.164742	1.111896	1.220101	1.22E-10
LRRC8C	0.993099	0.922794	1.068761	0.853345
RSL1D1	0.881217	0.804948	0.964711	0.006185
LRRC8D	1.056781	0.984256	1.134651	0.127888
PPID	1.158667	1.036691	1.294996	0.009463
COL24A1	0.988551	0.938519	1.041251	0.663909
ETFDH	1.186839	1.081747	1.302141	0.000293
RXFP1	1.036205	0.981968	1.093438	0.194779
LPAR3	1.103841	1.045886	1.165007	0.00033
PTGER4	0.97279	0.917555	1.03135	0.354984
TBCA	0.990736	0.905614	1.08386	0.839093
ECEL1	1.101998	0.965768	1.257445	0.14913
BCL2L1	1.034019	0.976635	1.094774	0.250818
PLRG1	1.138028	1.034851	1.251493	0.007666
ZNF584	1.124788	1.021554	1.238454	0.016661
NMUR1	1.035354	0.990498	1.082241	0.12418
CLSTN1	0.952518	0.890241	1.01915	0.158509
CXXC5	1.031794	0.973829	1.09321	0.288694
ZNF274	0.895181	0.831491	0.96375	0.003277
PIK3CD	0.980555	0.916406	1.049194	0.569461
PTCRA	1.001014	0.933444	1.073475	0.977324
SLC25A33	0.967169	0.885673	1.056164	0.457313
ENC1	1.111392	1.043063	1.184197	0.001105
SPSB1	1.061751	0.997977	1.129599	0.057974
P2RY6	1.106577	1.020517	1.199894	0.014222
BPTF	1.013774	0.94075	1.092466	0.719857
S100Z	0.935178	0.897361	0.97459	0.001462
ZIK1	1.079908	1.008605	1.156252	0.027397
GPR82	1.11284	1.017006	1.217704	0.019966
GPR34	0.81094	0.751404	0.875194	7.18E-08
PLEKHG5	0.900123	0.81381	0.995591	0.040767

ATF7IP	0.978652	0.909958	1.052531	0.561132
RGS19	1.058947	0.991712	1.130741	0.087027
TCEA2	1.168327	1.04972	1.300336	0.004394
HDAC3	1.033608	0.929109	1.14986	0.543287
GPHN	1.02671	0.962823	1.094837	0.421303
VAT1L	1.101065	1.043447	1.161865	0.000447
TMEM51	0.943258	0.886361	1.003806	0.065728
CAMTA1	1.068969	0.946327	1.207504	0.283413
LGALS4	1.102177	0.998359	1.21679	0.053926
LRRC34	0.942726	0.85142	1.043823	0.256472
SPATA5L1	1.114064	1.018586	1.218491	0.018137
GATM	1.019073	0.959798	1.082007	0.536623
RASGRP4	1.049445	0.983725	1.119556	0.143557
SLFNL1	1.058394	0.993561	1.127458	0.078466
BCL2	1.026387	0.965609	1.090991	0.403005
UTF1	1.01913	0.954882	1.087701	0.568432
COL8A2	1.063012	0.997657	1.132648	0.059091
PWWP2B	1.001649	0.934048	1.074142	0.963136
ZNF540	1.06269	0.971949	1.161904	0.181817
ANGPTL7	1.028443	0.964987	1.096071	0.388072
FBXL14	1.056636	0.978772	1.140693	0.158369
EXOSC10	1.010138	0.924667	1.10351	0.82305
ZNF570	1.106694	1.00391	1.22	0.041507
NINJ2	1.220233	1.141146	1.304802	5.82E-09
MLLT3	1.047062	0.992207	1.104949	0.093933
FAM90A1	1.183637	1.066228	1.313976	0.001561
RRM2	1.111324	1.04745	1.179093	0.000474
IFNB1	1.096609	1.012892	1.187245	0.022839
RPS21	0.923531	0.863093	0.988202	0.021243
C3AR1	0.929532	0.884181	0.977209	0.004192
PTEN	0.965141	0.901596	1.033164	0.307218
RPS7	0.836523	0.770209	0.908546	2.28E-05
RNASEH1	1.084737	0.994214	1.183503	0.067331
PRNP	0.911818	0.854933	0.972489	0.004973
FRMD5	1.061224	0.99024	1.137297	0.092514
CYP4F11	0.696274	0.60547	0.800697	3.82E-07
TLN2	1.13723	1.058349	1.22199	0.000455
ZNF217	0.956726	0.890634	1.027722	0.225794
SCG2	1.079728	1.00198	1.163508	0.044235
ATPAF2	0.938728	0.862179	1.022072	0.145139
CYP4F22	1.229241	1.116695	1.353129	2.52E-05
PPIH	1.003202	0.894785	1.124755	0.956312
ZNF57	1.096596	0.932105	1.290115	0.26612
JMJD1C	0.962598	0.908841	1.019535	0.193558
SYNPO	0.976264	0.859832	1.108462	0.710827
MAL	1.007117	0.950733	1.066844	0.809362
ZNF554	1.058755	0.963395	1.163553	0.235785
RAB33B	0.892463	0.823173	0.967587	0.005797
THOP1	1.071669	0.972937	1.18042	0.160434
LAMB2	0.918464	0.88543	0.95273	5.34E-06
USP19	1.024963	0.948988	1.10702	0.530344
ORMDL3	0.997265	0.924552	1.075695	0.943466
KLF11	0.994633	0.948444	1.04307	0.824444
SMN1	0.903215	0.816816	0.998752	0.047223
EIF2AK3	0.924038	0.870257	0.981144	0.009818
KRCC1	0.993826	0.925578	1.067106	0.864523
NME6	1.117292	1.026752	1.215816	0.010104
CYCS	0.944262	0.87498	1.019029	0.140178

CD8B	1.007845	0.929863	1.092368	0.849159
SLFN12	1.075021	1.003791	1.151306	0.038628
FRMD3	1.061163	0.999514	1.126615	0.051891
SNTB1	1.082446	1.014515	1.154927	0.016587
MTBP	1.087255	0.995659	1.187278	0.062451
MRPL13	1.066827	0.954149	1.192811	0.25602
MALT1	1.01345	0.947318	1.084198	0.697991
PRL	1.027209	0.975621	1.081524	0.307184
ISG20	0.95281	0.899054	1.00978	0.102789
MBOAT1	1.001977	0.943029	1.064609	0.949105
GPR22	1.000278	0.945086	1.058692	0.992351
CXCR6	0.988495	0.933725	1.046477	0.690701
CEBPB	1.005894	0.947903	1.067433	0.846192
AZU1	0.947356	0.919661	0.975885	0.000354
TPSAB1	0.896488	0.870575	0.923172	2.84E-13
PAIP1	0.94568	0.867733	1.030629	0.203177
CLEC7A	0.983734	0.946098	1.022866	0.409933
C1QTNF4	0.92453	0.894828	0.955217	2.48E-06
SERHL	1.108881	0.975114	1.260998	0.115087
NEGR1	1.05699	1.008132	1.108215	0.02171
ZNF131	0.968761	0.88938	1.055228	0.466872
MACROD2	0.818096	0.725891	0.922013	0.000999
DPAGT1	1.132278	1.047972	1.223366	0.00165
BSG	0.976608	0.899502	1.060323	0.572696
HINFP	1.083111	0.979312	1.197912	0.120365
SPTLC3	0.995384	0.911554	1.086923	0.917902
TP53RK	1.22187	1.117863	1.335553	1.01E-05
CLEC12A	0.960291	0.922139	1.000022	0.050124
BPGM	1.006796	0.959994	1.055881	0.78033
POP7	1.215055	1.11151	1.328247	1.82E-05
ALG14	1.832134	1.477744	2.271514	3.38E-08
SUCLG2	1.040868	0.989275	1.095151	0.122531
STARD5	1.158257	1.045872	1.28272	0.004784
CSDC2	1.004105	0.892952	1.129095	0.945432
IL16	1.078718	1.001966	1.161349	0.044206
GNB2	0.890216	0.817509	0.96939	0.007471
PDZD3	0.936667	0.790771	1.109482	0.44884
C2CD2L	1.078833	0.974342	1.19453	0.144322
GNG12	1.170427	0.974508	1.405735	0.092243
PRSS27	1.170909	1.019504	1.344798	0.025523
DNAJB7	1.121151	1.00885	1.245953	0.033705
CLP1	1.027831	0.941956	1.121536	0.537455
TTC36	0.963432	0.889932	1.043003	0.357536
RSPH9	1.140436	1.052448	1.235779	0.001337
GTPBP2	0.976168	0.90261	1.055721	0.546221
FGGY	1.053924	0.945971	1.174196	0.340807
IL17D	0.936458	0.78292	1.120105	0.472418
TCEAL1	1.142357	1.047081	1.246302	0.002741
ZNF24	1.081935	1.000107	1.170458	0.04969
MANEA	1.090864	1.000358	1.189559	0.04906
AGXT	0.754803	0.675201	0.84379	7.53E-07
AFF1	0.986263	0.918953	1.058503	0.70133
ACOT12	1.172278	1.048616	1.310524	0.005197
FIBP	1.226779	1.103943	1.363283	0.000146
BANP	1.044634	0.954069	1.143796	0.3453
PPP1CA	1.074431	0.977013	1.181563	0.138763
HCFC1	0.958834	0.886467	1.037108	0.293747
CTSW	1.018338	0.97659	1.061869	0.394861

NIPAL4	0.962041	0.891122	1.038604	0.321941
PDE3A	1.100554	1.024114	1.1827	0.009088
RASGRP1	1.010801	0.957109	1.067504	0.699666
KLHL6	0.970182	0.907353	1.037362	0.375527
CHCHD1	1.146175	1.040336	1.26278	0.005781
MRPL52	1.186496	1.058684	1.329738	0.003276
SMPDL3A	1.105809	1.049382	1.16527	0.000167
RND1	1.047125	0.977434	1.121784	0.190049
RAD9A	1.184133	1.077373	1.301473	0.000455
EFEMP2	0.977605	0.912888	1.046911	0.516907
OR10AD1	1.077122	0.970947	1.194907	0.160581
TMEM134	1.11128	0.976364	1.264839	0.1101
ZMAT3	0.94108	0.886299	0.999246	0.047188
ZNF738	0.987129	0.921919	1.056952	0.710252
SLFN11	0.973288	0.920472	1.029134	0.341532
CORO1B	0.933201	0.857726	1.015317	0.10812
FUT10	1.040094	0.964411	1.121716	0.307804
LRRC20	1.232172	1.149494	1.320798	3.83E-09
MUS81	1.073745	0.981619	1.174517	0.120036
TMEM217	1.106603	1.011726	1.210379	0.02677
ZNF596	1.248688	1.082354	1.440584	0.002327
CFL1	1.038862	0.955509	1.129486	0.371617
TMCC1	1.003587	0.919985	1.094785	0.935698
RAB43	0.840036	0.776261	0.909051	1.51E-05
CBWD1	1.15378	1.034696	1.28657	0.010064
RAB37	1.035773	0.976283	1.098887	0.244167
DCP2	0.954986	0.891896	1.022539	0.18657
SNX32	1.046994	0.98779	1.109748	0.122033
RPL38	0.948234	0.886152	1.014665	0.123913
CYP7B1	1.133122	1.080196	1.188641	3.04E-07
RARG	1.063564	0.98669	1.146427	0.107419
CES3	1.17101	1.073702	1.277137	0.000362
SSH3	0.965002	0.893249	1.04252	0.366163
CES2	1.084813	0.990737	1.187821	0.078595
PDP2	1.078251	0.99686	1.166287	0.059912
SP3	0.960211	0.889417	1.036641	0.298781
DMXL1	1.004612	0.928087	1.087447	0.909374
ZNF621	1.02414	0.952427	1.101253	0.51957
EGFL7	0.927854	0.895356	0.961531	3.85E-05
NADSYN1	0.996031	0.915029	1.084204	0.926788
DHCR7	0.975914	0.914112	1.041894	0.465125
NBEA	1.160176	1.076883	1.249911	9.28E-05
RNASEH2(	1.135532	1.04802	1.230351	0.001895
MYEOV	1.325442	1.151228	1.526019	8.90E-05
ANKRD13I	1.081372	1.001729	1.167347	0.045047
MYD88	1.000511	0.92372	1.083686	0.989994
MRGPRD	0.971774	0.874514	1.07985	0.594618
OXSR1	0.901003	0.833021	0.974533	0.009202
SLC22A13	1.030731	0.915799	1.160086	0.615818
PHF8	0.95518	0.883084	1.033161	0.252114
LCLAT1	1.148137	1.062262	1.240955	0.000496
KAT5	1.0231	0.92911	1.1266	0.642298
SH3RF3	0.992912	0.935595	1.053742	0.814629
GXYLT2	1.012699	0.952697	1.076481	0.685516
HPSE2	1.132265	1.079718	1.187369	3.00E-07
DCAKD	1.109429	1.018495	1.208483	0.017315
TADA2B	1.006239	0.935869	1.081899	0.866474
CCDC96	1.139969	1.004814	1.293303	0.041896

RELA	0.853661	0.782649	0.931116	0.000356
EVC2	0.909842	0.842936	0.982058	0.015327
ZNF680	1.004994	0.935811	1.079292	0.891106
RXFP4	0.976917	0.933851	1.021969	0.309979
HPSE	1.071303	1.009401	1.137002	0.023323
COQ2	1.054195	0.968281	1.147733	0.223674
HSPA6	1.034031	0.992056	1.077782	0.113481
TRMT112	1.132801	1.031755	1.243744	0.008904
LRRN3	0.989032	0.920411	1.062768	0.763702
ADCK5	1.169637	1.082926	1.263292	6.69E-05
NOC3L	1.116801	1.040197	1.199047	0.002312
ESRRA	1.008367	0.935724	1.086649	0.827103
COMMD1	1.183158	1.054057	1.32807	0.00433
RAPH1	1.169378	1.000876	1.366248	0.048725
MTX1	1.191388	1.049165	1.35289	0.006936
PARP14	1.044354	0.987948	1.10398	0.125538
CYSLTR1	0.96288	0.896575	1.034089	0.298745
PARP15	1.064387	0.998946	1.134114	0.053929
CKS1B	1.176799	1.080268	1.281955	0.000193
ABCD2	1.226828	1.119276	1.344713	1.26E-05
ABLIM3	1.157784	1.029301	1.302304	0.01464
VANGL1	1.090044	1.025377	1.158791	0.005726
GLRX	1.120825	1.019306	1.232455	0.018538
IQCB1	0.974156	0.895047	1.060256	0.544558
GOLGB1	1.000326	0.929948	1.076029	0.993022
ZNF483	1.141725	1.015109	1.284133	0.027104
PLAC8L1	1.208502	1.04327	1.399904	0.011581
SLC2A14	1.064163	1.004208	1.127698	0.035562
GPR137	1.155596	1.024451	1.303529	0.018622
SNCG	1.237016	1.102907	1.387433	0.00028
MMRN2	1.236954	1.048275	1.459593	0.011793
TNKS	0.966264	0.90363	1.03324	0.315551
ZNF449	1.076975	0.957069	1.211904	0.218192
PPP1R3B	1.002919	0.939854	1.070216	0.929906
STOX2	0.949551	0.88182	1.022485	0.170361
MAP3K11	0.980016	0.904308	1.062063	0.622654
TRIB1	0.960451	0.911256	1.012303	0.132538
KCNK7	0.979885	0.919616	1.044104	0.530398
C1QB	1.019778	0.975791	1.065749	0.383975
C1QA	1.031544	0.991328	1.073391	0.12585
OLR1	0.975	0.934199	1.017582	0.245709
GLIPR1L1	1.024249	0.917285	1.143686	0.67028
DAG1	1.094588	1.01617	1.179056	0.017177
ARV1	1.159186	1.078544	1.245858	5.94E-05
SAA1	0.993777	0.896624	1.101456	0.905325
EHBP1L1	1.039785	0.966862	1.118208	0.292981
THAP2	1.055593	0.945961	1.17793	0.333538
RNF26	1.095406	0.996226	1.204461	0.059854
PPP1R14B	0.890567	0.821321	0.965652	0.005012
SMARCC1	0.938441	0.867769	1.01487	0.111729
ZNF417	1.045049	0.969647	1.126315	0.248811
PTPRM	0.935113	0.884258	0.988893	0.018701
FKBP2	1.118008	0.981249	1.273827	0.093813
VEGFB	0.965793	0.87601	1.064779	0.484463
TNFRSF10	0.912886	0.862022	0.966752	0.001834
MST1	1.068304	0.998125	1.143416	0.056671
TNFRSF10	1.071003	1.017977	1.126792	0.008105
GMPPB	1.027932	0.953874	1.107738	0.47022

ZNF622	1.101544	1.001656	1.211392	0.046142
CSPG4	1.051877	1.005278	1.100637	0.028695
SNX33	1.028608	0.966654	1.094531	0.373501
CHD2	0.919193	0.869741	0.971456	0.002823
CCDC106	1.065166	0.978216	1.159845	0.146214
CCR9	0.999461	0.935099	1.068252	0.987327
SULT1B1	0.984403	0.92952	1.042525	0.591202
NUDT4	1.030961	0.956666	1.111026	0.424266
PC	1.135479	1.052445	1.225063	0.001041
NMNAT1	1.064913	0.977926	1.159637	0.148021
LRFN4	1.071734	1.008823	1.138568	0.024795
SLC19A1	1.099158	1.018528	1.186171	0.015005
HSPB7	1.21658	1.10584	1.338411	5.67E-05
RCE1	1.045451	0.945528	1.155933	0.385842
UQCRH	1.053792	0.957636	1.159602	0.283153
TAS1R1	0.981754	0.920644	1.046919	0.574386
EIF1AX	1.007703	0.921637	1.101805	0.866223
PSMD1	1.058959	0.966267	1.160543	0.2203
SUSD5	0.941748	0.826384	1.073217	0.368029
HEG1	1.185724	1.118003	1.257548	1.37E-08
TOMM20	0.970821	0.892423	1.056106	0.49063
AGFG1	0.953092	0.890445	1.020147	0.166064
STAT5B	0.972129	0.90693	1.042016	0.424859
CD7	1.042945	0.992971	1.095433	0.09327
CNP	0.998335	0.929376	1.072412	0.963615
JUP	0.904334	0.870911	0.939039	1.66E-07
HAP1	0.892562	0.798165	0.998122	0.04627
EIF1	0.89307	0.820377	0.972205	0.009036
RNF213	1.027771	0.970904	1.08797	0.345563
TIGD3	1.009849	0.926818	1.100319	0.822844
PLK3	1.004579	0.942966	1.070217	0.887507
NET1	0.938445	0.883256	0.997082	0.039931
DPY19L1	1.058178	0.990524	1.130454	0.093442
PHOSPHO	1.036782	0.994183	1.081207	0.091522
ZNF791	0.953098	0.886433	1.024775	0.194132
PHC3	0.950658	0.883624	1.022777	0.175002
GPR160	0.956789	0.90161	1.015346	0.144984
CBX2	0.905565	0.863475	0.949708	4.41E-05
SPTBN2	0.967228	0.905902	1.032706	0.318763
GOLIM4	0.981523	0.917388	1.050141	0.588552
RBM4B	1.019629	0.95219	1.091845	0.577687
HOXB2	1.0421	1.004883	1.080695	0.026251
SLCO4C1	1.017532	0.96406	1.073971	0.52801
RBM4	1.012359	0.951833	1.076734	0.696156
UBXN2A	0.905704	0.831153	0.986942	0.023829
RAX2	1.02201	0.872745	1.196803	0.786953
TCAP	0.976723	0.917431	1.039846	0.461053
CCS	1.069073	0.974899	1.172344	0.155711
KLHL15	0.890924	0.835441	0.950092	0.000431
FBXO45	1.113469	1.019824	1.215713	0.016489
GNG5	0.958496	0.863029	1.064524	0.428426
SLC25A30	1.065528	0.988932	1.148057	0.095405
CD34	0.995932	0.969989	1.02257	0.762148
CTSF	1.080855	1.020079	1.145252	0.008457
MSRB3	0.976356	0.93486	1.019694	0.280215
LEMD3	0.999611	0.928167	1.076553	0.991792
TLR10	0.900204	0.826969	0.979924	0.015166
TLR1	0.962174	0.903385	1.024788	0.23062

TLR6	0.905631	0.849481	0.965493	0.002403
FAM174A	0.994495	0.888777	1.112788	0.923307
RGMB	1.083419	0.931918	1.25955	0.297174
FAM53A	0.901041	0.813954	0.997446	0.044509
CYB561D1	1.078925	1.004404	1.158974	0.037497
ZDHHC24	0.992744	0.908567	1.08472	0.872028
SELP	1.090599	1.045866	1.137246	4.94E-05
CTU2	1.155878	1.049203	1.273401	0.003366
MGA	0.973833	0.919791	1.031051	0.362703
ARL13A	1.085882	0.98073	1.202307	0.112847
PIGG	1.019078	0.941544	1.102996	0.63973
PRPF8	0.928351	0.858134	1.004314	0.063928
ADCY6	1.104019	1.046948	1.164202	0.000258
PITPNA	0.998562	0.913291	1.091794	0.974789
DDX23	1.129321	1.032966	1.234665	0.007523
ZNHIT2	1.064337	0.96736	1.171037	0.200834
ZBTB4	0.89907	0.837602	0.965048	0.003234
TNK1	1.123424	1.020838	1.236318	0.017215
ZHX3	1.092678	1.010039	1.182079	0.027181
PHLDA3	1.117953	1.051803	1.188263	0.00034
SLC16A11	1.103477	0.940189	1.295123	0.228152
SLC16A13	1.230217	1.129395	1.34004	2.04E-06
STAG3L3	0.967396	0.904369	1.034815	0.334871
SLC6A19	0.995465	0.939172	1.055132	0.878363
SNHG11	1.238261	1.088028	1.409237	0.001202
EXO1	1.148098	1.072757	1.22873	6.66E-05
RALGAPA1	0.9855	0.914698	1.061783	0.700997
LIG4	1.059971	0.981596	1.144605	0.137276
GTF2IRD2E	0.950449	0.84958	1.063295	0.374642
ABRA	0.966186	0.859249	1.086432	0.565437
ATP2A2	0.934608	0.875936	0.997211	0.040915
ZWILCH	1.117005	1.014291	1.230121	0.024559
RPL4	0.841723	0.778714	0.90983	1.42E-05
SNAPC5	1.00689	0.908791	1.115579	0.895545
ZCCHC12	0.986349	0.892321	1.090286	0.788009
GALNTL6	0.997386	0.883038	1.126541	0.966393
LINGO2	1.214417	1.137666	1.296345	5.47E-09
BBS1	1.030966	0.858802	1.237644	0.743568
DENND4A	0.972512	0.913763	1.035039	0.380649
PELI3	1.267821	1.163737	1.381215	5.66E-08
TTC9B	1.262279	1.097994	1.451144	0.00106
MYO1H	1.038029	0.904828	1.190839	0.594266
TMEM81	1.019209	0.924515	1.123602	0.702137
MRPL11	1.091647	0.993634	1.199328	0.067714
IL20RB	0.957512	0.875043	1.047754	0.344756
AKIRIN1	0.913436	0.842653	0.990165	0.027798
MSL2	0.963579	0.896787	1.035346	0.311422
ZNF497	0.988777	0.896471	1.090588	0.821424
CMKLR1	1.022909	0.935549	1.118426	0.618987
ANGEL2	1.069385	0.987677	1.157853	0.098085
UGT8	0.942374	0.855914	1.037568	0.226727
KY	1.156813	1.043849	1.282001	0.00546
IQCK	1.001109	0.842733	1.189248	0.989939
ZNF266	0.980321	0.91406	1.051386	0.577794
SLC29A2	1.221436	1.133141	1.316611	1.74E-07
BRSK2	1.174049	1.046676	1.316922	0.006171
TMEM167A	1.058228	0.971272	1.152969	0.195775
SH3PXD2E	1.089275	1.027658	1.154587	0.003998

LARP7	0.991895	0.890407	1.104952	0.88253
FGFBP3	1.170271	1.073801	1.275409	0.000341
NR1D2	1.010229	0.950034	1.074238	0.745422
BRMS1	1.027187	0.908933	1.160825	0.667305
RPL15	0.879945	0.809226	0.956844	0.002772
HRAS	1.160953	1.0497	1.283997	0.003688
WDR49	1.039452	0.998356	1.08224	0.060108
SRP72	0.99641	0.907513	1.094015	0.939867
PCP2	1.010347	0.945462	1.079684	0.761165
RIN1	0.976257	0.911297	1.045848	0.49399
THAP6	1.126363	1.023218	1.239907	0.015167
CEP135	1.015376	0.937064	1.100234	0.709429
FZD4	1.011552	0.87209	1.173315	0.879388
CD248	0.875703	0.813323	0.942869	0.000431
PDZK1	1.061568	0.948222	1.188463	0.29969
PDE12	1.051034	0.95834	1.152694	0.290681
GLMN	0.999538	0.926351	1.078508	0.990499
YIF1A	1.093076	0.990026	1.206852	0.078146
CNIH2	1.017993	0.944348	1.097382	0.641607
NLRP6	1.071293	1.006665	1.14007	0.030066
NDUFA11	1.181595	1.077274	1.296017	0.000403
RSRC1	1.091631	1.008615	1.181479	0.029815
RAB1B	0.984015	0.885659	1.093294	0.764245
PTDSS2	0.925784	0.844663	1.014696	0.099321
SEZ6L2	1.164557	1.05889	1.280769	0.001695
ASPHD1	1.12702	0.971966	1.306811	0.113323
KCTD13	1.119558	1.014803	1.235126	0.024251
P2RY14	1.116531	1.065442	1.170071	3.98E-06
AMZ1	1.031712	0.963103	1.105208	0.373902
GPR171	1.151524	1.099782	1.205701	1.80E-09
FUT1	0.974327	0.906348	1.047405	0.480929
DHX36	0.968127	0.901088	1.040155	0.376321
FBXW8	1.072501	0.993657	1.157601	0.072394
KLC2	1.165521	1.082712	1.254664	4.63E-05
SLC22A1	1.17972	1.09316	1.273134	2.13E-05
CTBP2	0.985119	0.916875	1.058443	0.682313
CHST2	1.086262	1.030661	1.144863	0.002025
ZDHHC14	1.060054	0.979738	1.146954	0.146848
ATR	1.033567	0.958791	1.114175	0.388867
UBE2C	1.138479	1.06797	1.213644	7.01E-05
GK5	1.019246	0.940618	1.104448	0.641642
VCPIP1	1.046659	0.972445	1.126536	0.224243
DES	0.963702	0.897849	1.034385	0.305917
PDIK1L	1.039996	0.965388	1.120369	0.301822
RAG2	1.199668	1.100084	1.308267	3.83E-05
TRAF6	0.989675	0.917815	1.067162	0.787278
ZNF654	1.048871	0.975422	1.127851	0.197695
MRPS22	0.929959	0.834062	1.036882	0.190971
PACS1	0.816011	0.751294	0.886303	1.42E-06
WFDC5	0.962619	0.874186	1.059998	0.438416
MARCKSL1	0.906871	0.856039	0.960721	0.000895
SH3BP5L	0.96294	0.90719	1.022115	0.214574
YPEL2	0.904645	0.84888	0.964074	0.002022
ABO	1.078307	1.037006	1.121252	0.000155
PSMD2	1.057892	0.957741	1.168515	0.267404
FAM131A	0.978291	0.891648	1.073354	0.642745
CSRP2	0.942372	0.890154	0.997653	0.041276
INHBC	0.968974	0.862006	1.089216	0.597442

PARL	1.200408	1.077924	1.336811	0.00088
DDIT3	0.909302	0.84915	0.973715	0.006474
PCCA	1.144042	1.080899	1.210872	3.39E-06
DCTN2	0.975758	0.870334	1.093952	0.67399
NPPA	0.978757	0.900915	1.063324	0.611577
ZNF408	0.976146	0.897205	1.062032	0.574696
CTDSP2	0.913051	0.848668	0.982319	0.014764
CKAP5	1.07683	1.006284	1.152322	0.032261
ARHGAP1	1.095966	1.000018	1.201119	0.049955
MED16	1.029937	0.948899	1.117896	0.480515
GOLGA8A	1.078031	1.030086	1.128207	0.001208
TP53I11	1.061156	1.003706	1.121895	0.036598
DOLK	1.057182	0.973342	1.148244	0.187159
PHYHD1	0.819485	0.747249	0.898704	2.35E-05
CATSPER1	0.939554	0.868569	1.016341	0.119809
CCNE2	1.162131	1.066473	1.266369	0.000607
CST6	1.039193	0.932873	1.15763	0.485096
ZNF519	1.129724	1.019522	1.251837	0.019851
LSM1	1.210524	1.059848	1.382622	0.004848
BANF1	1.123745	1.045328	1.208046	0.001572
APOF	1.189242	1.055456	1.339986	0.004422
TMEM9B	1.065826	0.963223	1.179357	0.21705
NRIP3	1.036313	0.980273	1.095557	0.20856
PTPN2	0.951336	0.868104	1.042547	0.28553
EIF1AD	1.08377	0.994943	1.180527	0.065218
SMAD2	1.122248	1.032852	1.219382	0.006466
EIF3F	0.855987	0.77693	0.943089	0.001661
ZNF25	1.126304	1.026141	1.236243	0.012314
ARL10	1.013436	0.944717	1.087154	0.70948
CLTB	1.094286	0.981457	1.220087	0.104621
LPL	1.068525	1.013526	1.126509	0.013961
RFESD	1.119484	1.03224	1.214103	0.006402
CCDC14	1.102387	1.029534	1.180395	0.0052
TBC1D10C	1.009677	0.938716	1.086001	0.795629
SART1	0.885287	0.800738	0.978763	0.017355
PPP2R2D	0.934479	0.850992	1.026157	0.155838
MCTP1	0.929206	0.875924	0.985729	0.014808
POLD4	1.10136	0.976473	1.24222	0.115893
OR52W1	0.986126	0.884491	1.099439	0.801235
LRRC25	0.985553	0.942372	1.030713	0.524371
DPP10	0.981605	0.905686	1.063888	0.65122
CLCF1	0.972205	0.906952	1.042153	0.426492
TSGA10IP	0.922816	0.79206	1.075157	0.302828
GPR152	0.940284	0.863293	1.024142	0.157756
UBQLNL	1.052236	0.948983	1.166725	0.333918
KCNE3	0.96255	0.907676	1.02074	0.202482
CABP4	1.074	0.997444	1.156432	0.058475
ALG10B	1.071639	0.977554	1.174778	0.140009
DRAP1	1.053058	0.960896	1.154061	0.268581
LONRF3	0.95511	0.909588	1.00291	0.06528
UCP3	1.107181	1.013882	1.209065	0.023395
UCP2	1.009749	0.935401	1.090006	0.803654
PAAF1	1.140698	1.040534	1.250505	0.004996
MRPL48	1.093325	0.972509	1.22915	0.135335
RAB6A	1.000309	0.905185	1.10543	0.995161
P2RY2	0.962217	0.919042	1.007422	0.100116
FOSL1	0.960132	0.905664	1.017877	0.17215
ERCC4	1.204209	1.081735	1.340549	0.000685

CCDC85B	0.956256	0.882243	1.036477	0.276475
TMEM70	1.061155	0.965442	1.166357	0.218415
RPS6KB2	1.010815	0.920397	1.110115	0.821993
TOM1L2	1.06477	0.991272	1.143719	0.085479
ZNF77	1.034589	0.952632	1.123598	0.419351
B3GNTL1	1.044072	0.978401	1.114151	0.193198
MLXIP	0.959161	0.883666	1.041106	0.318825
AURKAIP1	1.095643	0.980929	1.223772	0.105506
TTLL11	1.162033	1.063077	1.270201	0.000943
TOMM5	1.153145	1.047977	1.268866	0.003496
SLC35E3	1.004476	0.913725	1.10424	0.926352
ZNF169	1.112608	1.024966	1.207743	0.010802
RUVBL1	1.127638	1.038816	1.224055	0.004108
SFN	1.01713	0.94562	1.094047	0.647924
MSRA	0.890096	0.816392	0.970454	0.00829
ETV4	1.174611	1.064186	1.296494	0.001398
GAPT	0.953321	0.903833	1.005518	0.07881
BAIAP2	0.940248	0.871784	1.014089	0.110207
ZDHHC21	0.977178	0.911003	1.048161	0.518759
PLEKHF2	0.920435	0.84835	0.998645	0.04631
A2M	1.077734	1.016557	1.142593	0.012049
ARL4D	1.121515	1.046347	1.202082	0.001196
DOK7	0.949772	0.874829	1.031136	0.219133
LRRN1	1.051517	0.970297	1.139535	0.220657
UBE2O	1.089818	1.022437	1.16164	0.008257
ORAI3	0.96392	0.885975	1.048721	0.393005
UNC119B	0.901091	0.842649	0.963586	0.002333
DENND2C	1.179809	1.102138	1.262953	1.95E-06
ASCL3	0.916006	0.800821	1.047758	0.200704
TUBB6	0.940287	0.894983	0.987885	0.014536
LYSMD3	0.956318	0.889549	1.028099	0.226459
B3GALT6	1.080895	1.01335	1.152943	0.01814
ZNF613	1.032076	0.937269	1.136474	0.520743
JAKMIP2	1.263325	1.171307	1.362572	1.38E-09
MBLAC2	1.080702	0.993217	1.175892	0.071554
ZNF683	0.88097	0.79811	0.972432	0.011915
SLC35A4	1.130111	1.044608	1.222612	0.00231
IP6K1	0.938964	0.881684	0.999964	0.04987
SSNA1	1.063828	0.962784	1.175477	0.224315
CSTF3	1.046738	0.965316	1.135029	0.268904
YES1	0.967451	0.923722	1.01325	0.160862
CHMP6	1.002583	0.926711	1.084667	0.94877
DLEU1	0.957778	0.876556	1.046526	0.340016
UFSP1	1.167191	1.071749	1.271133	0.000382
TMEM39A	0.934822	0.865929	1.009196	0.084421
TCP11L1	0.914619	0.846935	0.987712	0.022896
GPX2	1.020458	0.954897	1.090519	0.550014
CCDC57	0.981046	0.91581	1.050931	0.585728
HSF5	1.097327	0.998188	1.206313	0.054553
SPHK1	0.99452	0.930194	1.063295	0.872041
BNIP3	1.004477	0.933452	1.080906	0.904962
MYPOP	1.023794	0.928893	1.128391	0.635647
ATAD5	1.04895	0.97327	1.130514	0.210995
ZNF404	1.113739	1.008419	1.230059	0.033555
RTTN	1.019111	0.951427	1.09161	0.589268
ACBD7	1.298861	1.177569	1.432647	1.72E-07
ANAPC2	1.010889	0.93842	1.088955	0.77537
ZBTB8OS	1.369671	1.194454	1.570589	6.66E-06

ZNF135	1.083221	0.998924	1.174633	0.053124
COX8A	1.091675	1.015614	1.173432	0.017292
TAC4	1.049332	0.993274	1.108554	0.085607
ZSCAN2	1.021523	0.928251	1.124167	0.66291
B3GNT4	0.957016	0.898045	1.019858	0.175743
CDC26	1.028788	0.911352	1.161355	0.646283
HSD11B2	0.966294	0.896591	1.041415	0.369395
CRLF3	0.975508	0.906002	1.050347	0.510856
RNPEP	1.176682	1.078751	1.283503	0.000243
EID2	1.02516	0.91948	1.142987	0.654406
EID2B	1.252429	1.116555	1.404838	0.000122
KCMF1	0.905108	0.8222	0.996376	0.041949
DNAJC30	1.190163	1.049418	1.349784	0.006705
SPRYD4	1.083819	0.990354	1.186105	0.080238
VPS37D	1.117038	0.982841	1.26956	0.090092
CLEC14A	0.85619	0.798034	0.918584	1.52E-05
CLK2	1.181621	1.078433	1.294682	0.000344
LPCAT4	1.002444	0.931014	1.079354	0.948401
SLCO3A1	0.997623	0.932537	1.067253	0.94489
ZNF575	0.949183	0.856248	1.052205	0.321188
WDR25	1.037858	0.931632	1.156196	0.499991
DIRAS1	1.015407	0.949532	1.085852	0.655044
PHLDB3	1.107487	1.018359	1.204416	0.017082
PRR15	0.990632	0.91142	1.076729	0.824821
GNG7	1.012343	0.94823	1.080791	0.713246
CNTD1	1.086113	0.986481	1.195808	0.092434
KBTBD11	1.00402	0.961623	1.048286	0.855381
B3GNT5	0.912594	0.857709	0.970992	0.003851
LMNB2	1.078917	0.999471	1.164677	0.051606
MEX3C	0.897304	0.842288	0.955913	0.000789
RNF152	1.085226	0.978285	1.203858	0.1223
MYO1D	1.069958	1.011605	1.131676	0.018117
FOXL1	0.84711	0.764578	0.938551	0.001511
LRRC37A	0.942318	0.846597	1.048863	0.277005
FOXC2	0.916369	0.799366	1.050497	0.210161
CCDC121	1.044361	0.95265	1.144901	0.354663
ACSF3	1.136893	1.04894	1.23222	0.00179
BOK	1.260723	1.131001	1.405322	2.89E-05
ZNF843	1.20314	1.042879	1.388028	0.011225
TTTY14	1.017657	0.962228	1.07628	0.54019
PFN4	0.977342	0.908254	1.051685	0.540064
CDK5R1	1.045611	0.985407	1.109494	0.140455
ZNF285B	0.986688	0.869512	1.119655	0.835415
RUFY1	1.022432	0.940405	1.111613	0.603122
BASP1	1.073722	1.033215	1.115817	0.000289
LRRC37A3	1.045368	0.955315	1.143909	0.334373
IRX5	1.03361	0.987731	1.08162	0.153564
METRNL	1.05606	0.997279	1.118306	0.06194
FAM91A1	0.972598	0.914592	1.034284	0.375859
WSB2	0.955729	0.878645	1.039575	0.29126
SOX11	1.152969	1.086525	1.223475	2.60E-06
TYMS	1.055494	0.976571	1.140796	0.173178
PXMP2	1.256767	1.117469	1.41343	0.000137
TCEANC	1.117456	1.01503	1.230216	0.023567
PNMA1	1.067613	0.991951	1.149046	0.081075
ANKLE2	0.941935	0.865961	1.024575	0.163273
C8G	1.229452	1.107463	1.36488	0.000107
FUT2	1.261155	1.127877	1.410182	4.67E-05

EFCAB5	0.861123	0.751269	0.98704	0.031769
GCNT4	1.224405	1.13548	1.320295	1.42E-07
MUC20	1.127958	1.012129	1.257043	0.029403
THAP4	0.983831	0.889548	1.088108	0.751136
NFATC2IP	1.063457	0.970854	1.164893	0.185629
FAM89B	1.150012	1.028824	1.285474	0.013889
SHMT1	1.141726	1.046946	1.245086	0.002722
DPP7	1.082587	0.992426	1.180939	0.07368
SEC24C	0.974241	0.907157	1.046286	0.473412
SMCR8	0.952429	0.89375	1.014961	0.133035
MTHFR	1.026712	0.957701	1.100696	0.457758
DEAF1	1.178233	1.075745	1.290484	0.000412
MTX3	1.1003	1.038817	1.165423	0.001122
TMEM80	1.155848	1.05085	1.271338	0.002876
FBXO46	1.025225	0.937414	1.121263	0.585551
ZDHHC13	1.144531	1.060983	1.234659	0.000482
SLC38A9	1.033845	0.951212	1.123658	0.433551
ACER2	1.131994	1.03335	1.240053	0.007694
WDR73	1.020021	0.924971	1.12484	0.691215
POLE	1.036666	0.972326	1.105263	0.270679
SCN4B	1.251198	1.09561	1.428881	0.000941
RHOG	0.8557	0.790436	0.926352	0.000118
EPS8L2	1.235723	1.071438	1.425198	0.003638
ANO6	1.041538	0.967141	1.121657	0.281775
ZBTB34	0.971421	0.908535	1.038661	0.395819
TALDO1	1.072986	0.978407	1.176708	0.134577
ULK1	0.954399	0.896655	1.015861	0.142713
RPS6KA3	0.983292	0.91893	1.052163	0.625688
B3GNT8	1.082792	1.010684	1.160045	0.023685
PUS1	1.118272	1.022852	1.222593	0.01403
CHD9	0.984113	0.916369	1.056866	0.659881
TRIM72	0.964719	0.892132	1.043212	0.368129
MAN1B1	1.011039	0.925835	1.104085	0.806909
KCNA3	1.082537	1.033758	1.133617	0.000748
FZD8	1.163805	1.099246	1.232155	1.89E-07
FBXO39	1.230048	1.113341	1.358988	4.68E-05
TOP3A	1.064386	0.979922	1.156129	0.139095
CASKIN2	0.96327	0.892658	1.039467	0.335333
ZBTB38	1.237506	1.156946	1.323675	5.48E-10
FLJ13224	0.950956	0.909304	0.994516	0.027765
RPL13AP3	0.992607	0.873769	1.127606	0.909189
CCDC71	1.002907	0.933653	1.077299	0.936622
LRRN4CL	1.000212	0.889739	1.124401	0.997172
FLJ40194	1.281181	1.121577	1.463498	0.000262
TIMM22	1.155125	1.038394	1.284978	0.007976
HIC1	0.962031	0.907488	1.019852	0.193647
PPFIA3	1.417982	1.306628	1.538826	5.79E-17
MAGEF1	1.163102	1.099172	1.230749	1.62E-07
UMODL1	0.879097	0.831635	0.929267	5.35E-06
SAMD9L	1.063554	1.005652	1.12479	0.030984
PAWR	1.187841	1.093989	1.289744	4.15E-05
TGIF1	0.771009	0.713873	0.832718	3.59E-11
NAP1L5	1.137241	1.039537	1.244129	0.005016
CD19	0.958436	0.908702	1.010891	0.118398
OR2T8	1.076375	0.99295	1.16681	0.073762
NR2C2	0.985606	0.923207	1.052222	0.663931
ACOT4	1.297549	1.196312	1.407352	3.29E-10
ARIH2	0.999034	0.912366	1.093934	0.983344

RBM44	1.00563	0.926959	1.090978	0.892548
ZBTB33	1.116028	1.039841	1.197797	0.002343
ZBED2	0.906744	0.85985	0.956196	0.000302
IRX3	1.070098	1.028484	1.113395	0.000815
OR2B11	1.180389	1.0507	1.326086	0.005225
SLC25A22	1.037922	0.943581	1.141695	0.443949
RABEP2	1.02079	0.938899	1.109823	0.629614
ATOX1	0.968753	0.871667	1.076652	0.555729
TBL1XR1	0.985528	0.92328	1.051972	0.661445
CD163	1.020412	0.982371	1.059927	0.297219
ZNF491	1.032065	0.936025	1.13796	0.526524
RPLP2	0.903619	0.840081	0.971961	0.00644
JUN	0.922919	0.877656	0.970517	0.00177
CSTF2T	1.069457	0.991413	1.153645	0.082404
GBA	1.064806	0.983908	1.152355	0.119336
ACAD9	1.077432	0.99658	1.164843	0.060947
IL17RA	1.018169	0.969859	1.068884	0.467847
PNPLA2	0.823494	0.753358	0.900159	1.90E-05
MBOAT4	1.011439	0.921565	1.110078	0.810665
AGTRAP	1.046044	0.98906	1.106311	0.115242
CD163L1	1.267965	1.159887	1.386115	1.76E-07
THAP5	1.022501	0.941514	1.110454	0.597138
SUMO4	1.013759	0.948723	1.083253	0.686249
DNAJC28	0.981281	0.867806	1.109595	0.763132
NAALADL2	1.119882	0.984498	1.273883	0.085014
CD151	0.900878	0.845068	0.960373	0.001378
POLR2L	1.0261	0.959152	1.09772	0.454187
FAM20C	1.093737	1.048258	1.141188	3.55E-05
FLII	0.966419	0.889422	1.050081	0.420039
SOX12	0.968782	0.898257	1.044844	0.410831
HNRNPA0	0.920603	0.842796	1.005593	0.066333
FAM87B	1.006668	0.920609	1.100772	0.884113
CHID1	1.117047	1.01945	1.223988	0.017647
ZNF620	1.084011	0.991373	1.185306	0.076749
ZNF518A	1.034091	0.971077	1.101195	0.296004
TMEM187	1.127339	1.027024	1.237452	0.01171
ZNF619	1.030251	0.954021	1.112571	0.44734
AP3S1	0.892294	0.814383	0.977658	0.014498
GRB2	1.100371	1.002437	1.207872	0.044311
ZBTB41	0.998008	0.91952	1.083194	0.961939
UBE2N	1.023973	0.921565	1.137762	0.65947
ARL6IP6	1.079402	0.972561	1.197979	0.150779
ZNF354C	1.262081	1.161906	1.370892	3.46E-08
MAMDC4	1.032125	0.974173	1.093525	0.283508
CENPBD1	1.079469	0.989275	1.177885	0.085842
ODF3	1.029398	0.957211	1.107029	0.43476
BET1L	1.081738	0.977644	1.196914	0.128012
RPS27	0.956441	0.907651	1.007854	0.095489
RIC8A	1.048588	0.962834	1.141981	0.275755
IMP3	1.293055	1.174568	1.423494	1.59E-07
ASB8	1.10097	1.000859	1.211095	0.047972
ODF3B	0.902695	0.852898	0.955399	0.000406
DPY19L2	0.908663	0.866942	0.952392	6.50E-05
GPR150	1.19768	1.11094	1.291192	2.57E-06
TSPYL6	0.961329	0.84998	1.087265	0.530063
DMAP1	1.141416	1.034643	1.259207	0.0083
IMPDH2	0.988465	0.905822	1.078649	0.794529
ALS2CL	0.933561	0.880118	0.99025	0.02227

MLF1	1.177894	1.099001	1.262451	3.68E-06
NDUFAF3	1.12531	1.01974	1.241809	0.01883
GRAMD1C	1.048127	0.911894	1.204713	0.508189
STAP2	1.004958	0.911986	1.107408	0.920461
TSSK6	1.013809	0.932995	1.101623	0.746255
BOLA1	0.99053	0.917756	1.069074	0.806916
PDE4DIP	1.235192	1.139069	1.339426	3.22E-07
DDX10	1.034794	0.960901	1.114369	0.365553
NDUFV2	0.952741	0.904438	1.003624	0.068197
DALRD3	0.976846	0.892471	1.069197	0.611258
ZNF114	1.225067	1.10015	1.364167	0.000216
ZNF518B	1.11555	1.038985	1.197758	0.002577
LCORL	0.977144	0.896067	1.065558	0.600861
PARD6G	1.027822	0.928701	1.137522	0.595852
SH2B1	1.060057	0.96957	1.15899	0.200145
ZC3H12D	1.046011	0.973587	1.123822	0.219156
VN1R1	1.094369	0.977517	1.225189	0.11752
SH2D4B	0.976123	0.872627	1.091893	0.672581
RNF212	1.134034	1.015011	1.267014	0.026194
PRSS36	1.201097	1.098109	1.313744	6.17E-05
ZNF543	1.038863	0.966002	1.117221	0.304113
GALNT11	0.987602	0.91537	1.065534	0.7475
WDR6	1.028795	0.962741	1.099382	0.401766
GEN1	1.076787	0.986285	1.175593	0.098603
TMPRSS9	1.066396	0.998649	1.138739	0.054907
AQP11	1.061804	0.986908	1.142383	0.108086
TMEM11	1.00421	0.899645	1.120927	0.94031
ZNF354B	1.040037	0.957889	1.12923	0.349732
KCNG2	0.838468	0.783934	0.896795	2.83E-07
ZFAND2A	0.96161	0.88059	1.050084	0.383362
PLEKHM3	1.006415	0.931423	1.087446	0.871415
ZNF223	1.180064	1.015111	1.37182	0.031146
BEND3	1.074845	0.979132	1.179914	0.129322
NT5DC1	1.047024	0.949073	1.155084	0.359166
GLDC	1.159608	1.031046	1.3042	0.013515
TUBAL3	1.177737	1.103678	1.256766	7.94E-07
P4HTM	1.143055	1.052355	1.241572	0.001526
DTX3	1.223705	1.157903	1.293247	8.14E-13
KLHL11	1.012889	0.94363	1.087233	0.723044
CTXN1	0.860329	0.782902	0.945414	0.001769
SLC25A20	1.153976	1.064848	1.250564	0.000479
CA8	1.027197	0.94655	1.114715	0.520087
CD28	0.974269	0.923711	1.027594	0.337669
EPM2AIP1	1.011262	0.938619	1.089527	0.768411
MAF	1.093966	1.01458	1.179563	0.019463
CTNNBIP1	0.943319	0.869385	1.02354	0.161146
GTPBP6	1.069805	0.971062	1.17859	0.172049
ERN1	1.0281	0.947284	1.115811	0.507045
GPR35	1.068414	1.002518	1.138642	0.041611
ZNF713	0.967908	0.87595	1.06952	0.521906
PARP10	1.117807	1.046699	1.193746	0.000897
SUZ12	0.962737	0.88862	1.043036	0.352843
NSUN3	1.098421	0.999442	1.207202	0.05137
KCTD12	1.024783	0.986759	1.064273	0.20444
RPP25	1.096541	1.021646	1.176927	0.010672
GRINA	1.153602	1.057308	1.258666	0.001313
THBD	1.089715	1.043525	1.13795	0.000101
GP5	1.154131	1.077874	1.235784	3.95E-05

COX5A	0.984587	0.901028	1.075896	0.731393
STX19	1.166108	1.017674	1.336191	0.026955
ZHX2	1.03347	0.975845	1.094498	0.260728
CPNE7	0.919697	0.877123	0.964338	0.000537
CD300LB	1.078162	1.032837	1.125477	0.000594
MPI	1.092893	0.991141	1.20509	0.074829
TRIM73	0.929991	0.829671	1.042441	0.212671
OPLAH	1.204832	1.135339	1.27858	7.87E-10
TMEM52	0.894971	0.798467	1.003138	0.056632
MSC	1.143565	1.055801	1.238624	0.000992
APOLD1	1.123513	1.024835	1.231693	0.013029
EXOSC4	1.351765	1.241574	1.471736	3.72E-12
DPY19L3	0.97844	0.908041	1.054298	0.567262
TAF7	0.973882	0.88784	1.068262	0.57495
PFAS	1.024572	0.96013	1.093339	0.46392
ZNF552	0.984923	0.922762	1.05127	0.647851
GAK	0.957925	0.891112	1.029749	0.243906
ZBTB7A	0.930823	0.85231	1.016568	0.110829
TUFM	1.051028	0.949776	1.163074	0.335571
RMI1	1.116079	1.039081	1.198783	0.002603
FBXO34	1.048999	0.973775	1.130035	0.20767
EIF3K	0.969547	0.869845	1.080677	0.576443
MRFAP1L1	1.202019	1.09758	1.316396	7.26E-05
SNX18	0.979375	0.924208	1.037834	0.481092
AURKB	1.128355	1.055754	1.205949	0.000372
MRFAP1	1.026999	0.91466	1.153136	0.652178
TMEM107	1.023598	0.972324	1.077576	0.373713
RRS1	1.164319	1.068124	1.269177	0.000544
RCC2	0.9565	0.892945	1.024579	0.204873
OR13D1	0.937889	0.837845	1.049878	0.265188
IGSF22	1.175967	1.018698	1.357515	0.026906
DPM3	1.083085	0.996179	1.177572	0.061448
CYC1	1.162953	1.038708	1.30206	0.008825
PER1	0.921952	0.867441	0.979889	0.008968
HTR1F	1.140836	1.09456	1.189067	4.47E-10
TMTC2	0.943049	0.899636	0.988557	0.014745
FARSA	0.993894	0.925733	1.067073	0.865817
SPTY2D1	0.884981	0.828762	0.945015	0.000263
SAMD4B	0.87721	0.807325	0.953145	0.001982
GIMAP7	1.042268	0.987594	1.099969	0.132095
EDC3	1.044692	0.96537	1.130533	0.277833
FUCA1	1.150732	1.077232	1.229247	3.06E-05
PXT1	1.005134	0.890106	1.135026	0.934186
GGN	0.970982	0.899634	1.047988	0.449505
ZNF664	1.154715	1.076592	1.238507	5.70E-05
CALR	0.831455	0.781769	0.884299	4.33E-09
MAGED1	1.028288	0.971817	1.08804	0.333061
LDLRAD3	1.168789	1.119487	1.220263	1.31E-12
CDH4	1.019966	0.946567	1.099056	0.603889
RAD23A	1.023493	0.909899	1.151268	0.69885
GADD45G	0.997738	0.932439	1.067611	0.947723
MEIS3P1	1.008914	0.942175	1.08038	0.799384
DAND5	0.976201	0.846911	1.125228	0.739671
TMEM151/	1.011093	0.916704	1.115202	0.825377
PTPN11	0.99206	0.912736	1.078279	0.851286
NSUN7	1.249258	1.129776	1.381376	1.43E-05
WSCD1	1.143136	1.026287	1.273289	0.015032
CLK3	0.977486	0.900597	1.060939	0.585908

HLA-DQB1	0.96517	0.92914	1.002598	0.067803
GATA2	0.981772	0.945383	1.019561	0.33974
ARID3B	1.071065	1.00157	1.145381	0.044877
PACS2	1.117382	1.021882	1.221807	0.014899
ELMOD2	1.110355	1.018756	1.210191	0.017174
EGR3	1.09564	1.042622	1.151355	0.000307
GPC5	1.251482	1.150652	1.361147	1.66E-07
VWA1	0.970544	0.897648	1.049359	0.452929
GEMIN4	1.026865	0.946523	1.114026	0.523625
FJX1	1.292667	1.185386	1.409657	6.35E-09
KLHL28	0.955403	0.876939	1.040888	0.29675
MKRN3	1.055604	0.996435	1.118286	0.06597
SHARPIN	1.232991	1.079583	1.408198	0.002005
LBX2	1.069186	0.98413	1.161594	0.113713
DNHD1	0.986228	0.925497	1.050945	0.668908
SLITRK4	1.131857	1.077827	1.188594	6.93E-07
GCC1	0.928607	0.861347	1.001119	0.053505
RNF151	0.980093	0.903465	1.063219	0.628307
CIITA	0.957196	0.915339	1.000968	0.055169
ZFPM1	1.053891	0.990832	1.120964	0.095435
ALOX15B	1.019846	0.9314	1.116691	0.671144
PLD6	1.125897	1.033249	1.226853	0.006799
CDC42EP4	0.98162	0.927372	1.039041	0.522445
ZBTB42	0.973071	0.907888	1.042935	0.440327
MAF1	0.917686	0.833687	1.010148	0.079462
FCER1A	0.99874	0.965164	1.033485	0.942402
FLJ37453	1.026746	0.928806	1.135014	0.605831
APOBEC3B	1.114164	1.043812	1.189259	0.00116
PIPOX	1.026953	0.948313	1.112115	0.5129
CDH5	1.236627	1.102034	1.387658	0.000303
MYADM	1.016915	0.94848	1.090287	0.637011
SERTAD2	0.944331	0.870414	1.024526	0.168411
AKAP5	1.000575	0.916137	1.092796	0.989799
NKPD1	0.992118	0.916036	1.07452	0.845879
GIPC3	0.903242	0.845972	0.96439	0.002328
CITED4	0.902747	0.855005	0.953154	0.000224
ABCA13	1.026044	0.985994	1.06772	0.205648
TIGD5	1.064407	0.980937	1.15498	0.134126
PDXDC1	1.014528	0.939791	1.095208	0.711813
ZNF154	1.020612	0.964409	1.08009	0.480208
R3HDM2	1.118762	1.002527	1.248474	0.044958
B3GNT3	1.184691	1.039446	1.350231	0.011094
ITLN1	1.077608	1.021785	1.136481	0.005886
SEPHS2	1.084541	0.996162	1.180761	0.061303
GPBAR1	0.921767	0.858861	0.989281	0.023897
ZNF784	0.973435	0.891795	1.062549	0.546882
BBS10	1.088282	1.010283	1.172302	0.025775
FIZ1	0.964427	0.865987	1.074056	0.509646
PUF60	1.082816	0.986148	1.18896	0.095392
DCTPP1	1.118368	1.037915	1.205056	0.003314
ZNF771	1.256299	1.089607	1.448493	0.001681
TSHZ1	1.050299	0.971793	1.135147	0.215681
PSTK	1.011257	0.917039	1.115155	0.822498
SOCS4	1.036583	0.95737	1.12235	0.375693
ZADH2	1.043103	0.955288	1.13899	0.346962
ZNF48	1.119025	1.032666	1.212606	0.006062
TMEM150I	1.126334	1.069367	1.186337	7.04E-06
ANKRD18A	0.998676	0.955223	1.044105	0.953445

TMEM86B	1.156672	1.052331	1.271359	0.002549
TRNAU1AI	1.014689	0.917125	1.122632	0.777391
EXOC3	1.128514	1.022542	1.24547	0.01626
CSNK1A1L	1.062809	0.962462	1.173619	0.228651
LYNX1	1.476735	1.342063	1.624921	1.35E-15
MED14	0.979964	0.904567	1.061646	0.620257
FAHD1	1.251024	1.141463	1.371101	1.67E-06
RCC1	0.935063	0.867726	1.007625	0.078279
MYLPF	1.073297	0.969817	1.18782	0.171479
F2	0.959262	0.844804	1.089228	0.521158
PRKRA	0.956737	0.875885	1.045052	0.326214
ZNRF2	0.97329	0.909304	1.04178	0.435225
FGD6	0.972954	0.910124	1.04012	0.420804
OAZ2	0.894613	0.821652	0.974052	0.010298
PNPLA1	1.062242	0.986383	1.143935	0.110203
CCDC43	1.102272	0.994976	1.221137	0.062381
KCTD4	0.986794	0.931829	1.045001	0.649374
FZD2	1.135761	1.075174	1.199761	5.33E-06
TIGD2	1.073293	0.991173	1.162217	0.081571
HCLS1	1.068318	0.987351	1.155926	0.100301
ZNF609	0.92613	0.873837	0.981552	0.009657
PAK2	1.000079	0.926114	1.07995	0.998396
CCDC66	1.054928	0.97073	1.14643	0.207675
MCFD2	1.005826	0.924408	1.094414	0.892708
HARBI1	1.071221	0.975924	1.175823	0.147817
GAS1	1.288201	1.208086	1.373628	1.07E-14
ZNF571	0.960524	0.885723	1.041643	0.330229
KCNE1	1.085965	1.026471	1.148908	0.00412
NRIP1	1.060542	1.008657	1.115097	0.021632
BHLHA15	0.977864	0.867468	1.102309	0.714179
RNF182	1.067484	1.018696	1.118609	0.006219
TSPYL5	1.174667	1.11821	1.233976	1.50E-10
FUT7	1.138969	1.084232	1.19647	2.24E-07
SSTR2	0.961668	0.86274	1.071939	0.480377
ZNF594	1.073069	0.9953	1.156916	0.066175
PCGF5	1.087304	0.994455	1.188822	0.066081
PRF1	0.991089	0.946384	1.037907	0.703885
YOD1	0.98742	0.93239	1.045697	0.665227
TMEM64	0.964325	0.906647	1.025671	0.248313
CHRM4	0.959834	0.884689	1.041362	0.324345
SHISA2	1.058277	0.969156	1.155593	0.206965
S1PR5	1.048116	0.975437	1.12621	0.199955
GPR157	1.331348	1.224556	1.447453	1.97E-11
PIPSL	1.134956	0.967047	1.332018	0.1212
CHST13	0.987956	0.943701	1.034286	0.604297
SLC36A4	0.985415	0.926232	1.048378	0.641971
ZDHHC20	0.89731	0.833448	0.966065	0.004022
ZFP3	1.210986	1.121254	1.307899	1.10E-06
MAP3K15	0.919968	0.833028	1.015981	0.099572
PPA1	1.157789	1.069177	1.253746	0.00031
PSMG4	1.234991	1.121293	1.360216	1.84E-05
MAP6D1	1.002411	0.855754	1.174202	0.976195
ZNF443	1.016112	0.949343	1.087577	0.644871
SSR4	1.033781	0.93705	1.140497	0.507449
CAPS2	1.081636	0.972533	1.202979	0.148018
ZNF792	0.996114	0.942771	1.052475	0.889715
CUEDC1	1.173181	1.095321	1.256576	5.15E-06
SCRIB	1.036067	0.969239	1.107503	0.297633

KCTD2	0.966228	0.885946	1.053785	0.4376
D2HGDH	1.075251	1.001483	1.154453	0.04541
OXTR	1.375407	1.188831	1.591265	1.82E-05
FAM83H	1.121092	1.054089	1.192353	0.000278
ZNF572	1.154632	1.038083	1.284266	0.008087
ST20	1.029676	0.954168	1.111159	0.451695
PITPNB	0.953937	0.879018	1.035241	0.258463
TCEAL8	1.082343	0.99729	1.174649	0.058096
LRRC57	0.928495	0.853342	1.010268	0.084933
MRPL14	1.126169	1.024876	1.237474	0.013476
GPR137C	1.107563	0.976754	1.25589	0.111121
BBS12	1.303629	1.164994	1.458761	3.80E-06
ZFP82	1.09495	0.988414	1.212969	0.082417
NQO1	1.071909	0.973631	1.180108	0.156978
OR56B1	0.894466	0.787079	1.016506	0.087435
AEN	1.059381	0.987372	1.136642	0.108245
FKRP	1.10531	1.000855	1.220666	0.048061
TRAPPC5	1.086223	0.981761	1.2018	0.108898
RPH3AL	0.964196	0.879114	1.057513	0.439193
SLC25A42	1.017483	0.946456	1.09384	0.638756
FCRL6	1.108874	1.014953	1.211486	0.022099
SLC26A11	1.012726	0.959156	1.069287	0.648362
HIGD1A	1.31922	1.217607	1.429312	1.25E-11
OR52N4	0.95474	0.873115	1.043995	0.30975
MAPK15	0.999415	0.90357	1.105427	0.990927
EHMT1	0.973847	0.898438	1.055586	0.519286
F2R	1.068124	1.016523	1.122344	0.00909
ZNF707	1.177861	1.072299	1.293815	0.000633
NPM1	0.868908	0.799217	0.944675	0.000987
PJA1	1.079275	0.987422	1.179673	0.092751
DHTKD1	0.98824	0.928154	1.052216	0.711663
PENK	1.081959	1.017366	1.150654	0.012136
ZNF746	0.939803	0.865647	1.020313	0.138754
POLR2A	0.94048	0.867826	1.019217	0.13467
FRAT2	1.005492	0.939956	1.075598	0.873451
TMEM102	0.916597	0.844536	0.994807	0.037106
CCL13	1.268367	1.067834	1.50656	0.006781
DDX60L	1.092236	1.029085	1.159261	0.00369
AATK	1.072085	0.999871	1.149515	0.050425
DDN	1.018623	0.899722	1.153237	0.770772
SAGE1	1.060824	1.021116	1.102076	0.002417
ZNF467	0.932128	0.881108	0.986102	0.014395
ZNF678	1.071536	0.99337	1.155853	0.073804
TMEM45A	1.145765	1.02219	1.284279	0.019445
RAP2B	1.02362	0.947669	1.105658	0.552842
ZBTB2	0.97809	0.897979	1.065347	0.611369
RNF135	0.951957	0.883109	1.026173	0.19864
ACBD4	1.202974	1.101434	1.313875	4.00E-05
SGSH	0.994747	0.946116	1.045878	0.836838
MAB21L2	1.052267	0.973976	1.136851	0.196525
FANCB	1.045252	0.965882	1.131143	0.27202
SETD2	0.943308	0.882167	1.008688	0.08783
TMIE	0.846877	0.742416	0.966036	0.013346
MEX3D	1.171796	1.087499	1.262627	3.15E-05
MRPS23	1.263918	1.129377	1.414486	4.53E-05
OR52H1	1.006514	0.923437	1.097064	0.882568
GPR135	1.089525	1.014597	1.169986	0.018344
P2RY13	1.030561	0.988138	1.074806	0.160443

ZFP41	1.15102	1.045389	1.267325	0.004186
PHLDA2	1.015743	0.966608	1.067376	0.536934
ATG9B	1.211709	1.041154	1.410204	0.013101
GPR88	1.15295	1.0806	1.230145	1.68E-05
PLAG1	1.175575	1.101919	1.254155	9.59E-07
YIPF6	1.037676	0.948597	1.13512	0.419316
ZBTB20	1.01445	0.942388	1.092021	0.702753
AMIGO1	0.988883	0.909113	1.075654	0.794478
GPR3	0.965163	0.89073	1.045816	0.38652
SIAH2	0.989596	0.914385	1.070994	0.795388
SLC9A9	1.175493	1.117395	1.236612	4.05E-10
LSM10	1.157088	1.028356	1.301935	0.015324
RELL1	0.956276	0.896108	1.020483	0.177519
RFX7	0.939742	0.879189	1.004465	0.067418
SLC35C1	1.0895	1.004759	1.181387	0.037997
TIGIT	1.05197	0.952453	1.161886	0.317692
RNF41	0.98263	0.885913	1.089906	0.740299
SLC2A4	1.053827	0.993678	1.117617	0.080383
CLDN7	1.063753	0.953925	1.186226	0.266321
ZNF329	0.997729	0.929352	1.071136	0.949941
ZNF101	1.074271	0.997261	1.157228	0.059067
ADO	1.10118	1.004067	1.207685	0.040743
PRKAG1	1.11878	0.991079	1.262937	0.069517
GINS3	1.276492	1.16703	1.396221	9.46E-08
OR52K2	1.00702	0.936552	1.08279	0.850095
CCDC149	1.013298	0.938633	1.093903	0.735152
MRPS11	1.29002	1.141811	1.457466	4.32E-05
SNRPE	1.047931	0.971796	1.13003	0.223776
RTKN2	1.251696	1.123603	1.394393	4.59E-05
CHST15	0.996898	0.952702	1.043145	0.893186
TRPC2	1.033707	0.970573	1.100947	0.302531
IDH2	1.003597	0.915783	1.099831	0.938737
TNRC18	0.945875	0.891433	1.003641	0.065797
TMEM30B	1.348421	1.174879	1.547596	2.11E-05
DEXI	0.958423	0.895614	1.025637	0.219461
NOP10	1.011729	0.93111	1.099328	0.783146
FAM89A	0.899022	0.838207	0.964249	0.002895
TDRKH	1.417212	1.299213	1.545928	3.80E-15
ZNF708	0.98578	0.914307	1.062841	0.709191
MRPL41	1.11391	1.013955	1.22372	0.024521
CREB3L2	0.895976	0.828442	0.969015	0.006011
P2RY8	0.984519	0.932268	1.039698	0.574952
TP53TG1	1.637589	1.346645	1.991392	7.73E-07
UNC5C	1.109529	1.064098	1.1569	1.10E-06
TSEN54	1.074366	1.000868	1.153261	0.047261
RGMA	0.900938	0.813877	0.997312	0.044234
UBA7	1.025586	0.959783	1.0959	0.455229
MRPS16	1.190039	1.071802	1.321319	0.001119
LDOC1	1.207568	1.072476	1.359676	0.001834
ARL6IP4	1.153753	1.007671	1.321013	0.038396
EXT1	1.085453	1.017319	1.158151	0.013171
SHMT2	0.982974	0.898008	1.075979	0.709668
ATP6AP2	1.114709	1.003177	1.23864	0.043494
ZAR1	1.036414	0.938272	1.144821	0.481023
BACE2	1.110581	1.038462	1.187708	0.002201
UBE2E2	0.856691	0.80771	0.908642	2.61E-07
SYNM	1.19538	1.057017	1.351854	0.004463
IZUMO1	1.20463	1.057217	1.372597	0.005183

B4GALNT4	1.070735	0.992562	1.155065	0.077236
AP1S2	1.055801	0.982639	1.134409	0.138345
DCAF4L1	1.026653	0.940764	1.120383	0.555126
ZSCAN22	1.048275	0.960225	1.144399	0.292229
KCNJ14	1.090232	0.999066	1.189718	0.052503
FBXL6	1.122843	1.026862	1.227794	0.011041
C1S	1.192218	1.070984	1.327176	0.001312
GLTPD2	1.025903	0.949441	1.108524	0.517551
KBTBD3	1.065518	0.919699	1.234457	0.398021
CLN8	0.940142	0.886956	0.996517	0.037767
PLCXD1	1.008574	0.936826	1.085817	0.820613
NXPH4	1.148005	0.982306	1.341654	0.082653
CACNB4	0.81026	0.732229	0.896606	4.65E-05
TRAPPC6B	1.04012	0.94836	1.140758	0.403847
PGBD4	1.115966	1.019719	1.221296	0.017111
NPLOC4	1.019005	0.934455	1.111204	0.670109
CAPN12	1.070019	0.963005	1.188924	0.208102
EXOC7	1.076909	0.970629	1.194827	0.162221
KPNA2	0.953573	0.878653	1.034882	0.25483
NCF1B	1.056441	1.009292	1.105793	0.018424
BGN	1.078731	0.983358	1.183353	0.108575
CEP97	1.072321	0.989449	1.162134	0.088853
FES	1.043438	0.979569	1.111471	0.187023
GLRX5	1.050233	0.975453	1.130746	0.193428
FAM104B	1.199679	1.08219	1.329924	0.000536
MXRA7	1.017791	0.958073	1.08123	0.567586
LIMK2	1.033736	0.974615	1.096443	0.269497
MFSD5	1.125254	1.037614	1.220297	0.004338
ADI1	1.124107	1.019776	1.239111	0.018572
SPNS3	1.054475	1.013558	1.097045	0.008617
SATB1	0.990125	0.923735	1.061287	0.779296
CSF1R	1.025948	0.984606	1.069026	0.222203
EPHB3	1.148253	1.085746	1.214359	1.29E-06
EPGN	1.049977	1.012367	1.088984	0.008783
HS3ST4	1.024383	0.962702	1.090017	0.447069
TRAK1	0.909608	0.838567	0.986667	0.022402
PLCB1	0.935571	0.887112	0.986678	0.01412
SKA2	1.309134	1.210692	1.41558	1.44E-11
NDN	1.060266	1.012905	1.109842	0.012076
TTC3	0.944252	0.884532	1.008003	0.085283
GALR2	0.97481	0.892112	1.065175	0.572724
TSKU	1.09422	0.96183	1.244833	0.17116
ANXA2	0.980876	0.934363	1.029705	0.435978
RGS6	1.158478	1.061774	1.26399	0.00094
HOXB4	1.055341	1.023449	1.088227	0.000581
PAQR7	0.890381	0.833689	0.950929	0.000542
PAPPA	1.099855	1.024195	1.181105	0.00886
MAFA	0.972032	0.90706	1.041659	0.4216
NGRN	1.209895	1.092744	1.339605	0.000246
RPS17	1.119685	1.019743	1.229422	0.017798
CRIP2	0.88823	0.838296	0.941138	5.94E-05
DDX28	1.106777	1.003635	1.220518	0.042086
ACBD3	0.929989	0.846457	1.021766	0.130649
VMO1	1.058424	0.95853	1.16873	0.26161
ALG12	1.038337	0.944013	1.142085	0.438795
LCK	1.022133	0.969297	1.077848	0.418863
COL18A1	1.162232	1.097402	1.230892	2.84E-07
RBM10	0.996602	0.906304	1.095898	0.944005

GLUD2	0.864199	0.745312	1.002049	0.053254
RPL35A	0.929126	0.85825	1.005855	0.069405
SLC25A18	0.9863	0.862126	1.12836	0.840758
ZNF721	0.871005	0.81372	0.932321	6.92E-05
CEP63	1.009292	0.904943	1.125674	0.868068
WFDC10B	0.791407	0.65403	0.957641	0.016177
EWSR1	0.933152	0.857055	1.016007	0.110917
ODF3L1	1.118647	1.04896	1.192963	0.000634
HMGN4	1.092015	1.003062	1.188856	0.042306
SPATA13	1.03464	0.961706	1.113105	0.361213
CNOT10	1.023735	0.923805	1.134475	0.654427
MTA1	0.946792	0.871732	1.028316	0.194493
ZNF662	1.011937	0.948926	1.079134	0.717527
CADM1	1.046794	0.999253	1.096596	0.053799
ZNF320	1.03947	0.958179	1.127658	0.351478
PYCR1	1.071234	1.007296	1.13923	0.028418
SPNS2	0.986437	0.945075	1.02961	0.53209
AP2A2	0.902983	0.834094	0.977562	0.01172
SLC8A1	1.171341	1.105937	1.240612	6.86E-08
SLC25A21	1.144697	1.022113	1.281982	0.019365
ABAT	1.087478	0.997997	1.184982	0.055595
SLC25A10	1.200314	1.090146	1.321616	0.000201
CAMK1D	1.010961	0.953184	1.07224	0.716559
LYSMD4	1.050984	0.969703	1.139078	0.225957
AFMID	1.01683	0.942	1.097604	0.668692
GAS6	1.111708	1.042254	1.18579	0.001294
NEB	0.991105	0.887213	1.107164	0.874353
BEGAIN	1.138813	1.035581	1.252335	0.007338
GPC6	1.016373	0.930174	1.110561	0.719473
CSMD1	1.127477	1.036734	1.226162	0.005068
GPR19	1.169505	0.990083	1.381441	0.065376
RABIF	1.137661	1.035229	1.250228	0.00738
TMEM119	1.105688	1.000099	1.222426	0.049774
FANCF	1.104087	1.020388	1.194652	0.013826
CALN1	1.074029	1.013615	1.138044	0.015615
RUVBL2	1.079399	0.994099	1.172019	0.068904
PTTG1IP	1.000451	0.924596	1.082529	0.991055
DDX41	1.038026	0.940869	1.145217	0.456671
DAZAP2	1.008626	0.912631	1.114718	0.866333
ZNF623	1.078837	1.007862	1.15481	0.028852
CCDC125	0.967404	0.903205	1.036167	0.34421
BCOR	1.151191	1.068195	1.240636	0.000226
JRKL	1.026633	0.944618	1.11577	0.536077
KIAA2026	0.980793	0.901515	1.067043	0.651999
FHL3	0.848389	0.800537	0.899102	2.85E-08
TMEM89	1.075934	0.964903	1.199742	0.187825
SF3A3	0.980058	0.900467	1.066684	0.641118
ASB7	0.959234	0.88332	1.041673	0.322471
TREX2	1.312083	1.148775	1.498605	6.20E-05
GPR132	1.067438	1.008326	1.130015	0.024755
MX2	1.124744	1.058773	1.194827	0.000138
EP400	0.970142	0.901527	1.043978	0.417957
MEX3B	1.097867	1.050797	1.147045	2.96E-05
PI4KAP2	1.032286	0.94433	1.128436	0.484341
PSMG1	1.041153	0.940265	1.152867	0.438029
KLRC4	0.998116	0.92313	1.079194	0.962258
SERHL2	1.036804	0.960246	1.119464	0.35576
PCBP3	1.08306	1.023669	1.145896	0.005555

SETD3	0.959691	0.882627	1.043485	0.33538
TNFAIP8L3	0.94943	0.838748	1.074718	0.411899
ZNRF3	1.186449	1.075665	1.308644	0.00063
SFXN4	1.023707	0.943353	1.110904	0.574268
FAM167B	1.029886	0.919504	1.153518	0.61068
MRPL54	1.00104	0.916879	1.092927	0.981483
ZNF438	0.944189	0.880609	1.01236	0.106401
CCR3	0.989297	0.8713	1.123273	0.868107
ZNF530	1.101013	1.007885	1.202746	0.032831
NDUFB1	1.232997	1.132174	1.342797	1.49E-06
KLHL25	0.892461	0.817649	0.974118	0.010865
TRMT12	1.143446	1.050012	1.245195	0.002056
BMP8A	0.992807	0.913172	1.079387	0.865624
EFHC2	1.049236	0.995681	1.105672	0.072171
NOG	1.028522	0.980346	1.079065	0.250567
UPP1	1.008519	0.957199	1.062591	0.750216
TRIM52	1.071257	0.976166	1.17561	0.146685
CMTM4	1.105453	1.043595	1.170977	0.000644
TMEM50A	1.063014	0.940866	1.201021	0.326487
ASCL2	1.12945	1.069233	1.193059	1.33E-05
TBK1	1.000682	0.929666	1.077124	0.985507
CBX6	1.140169	1.072968	1.211579	2.31E-05
MACC1	1.029286	0.975553	1.085978	0.291341
TBL3	1.107102	1.007461	1.216598	0.034477
KREMEN1	1.125702	1.054315	1.201922	0.000397
TRAIP	1.242876	1.127042	1.370616	1.32E-05
CHEK2	1.093146	1.012317	1.180427	0.023067
AIFM3	1.044457	0.982662	1.110138	0.162148
ZNF703	1.004554	0.958441	1.052886	0.849685
EMILIN3	1.207736	1.109383	1.314808	1.33E-05
OLFML1	1.204449	1.04796	1.384306	0.008801
RBM12B	1.046602	0.978489	1.119456	0.184632
CCR4	1.076989	1.00375	1.155573	0.039006
LIN9	1.176034	1.061587	1.302818	0.001909
BTBD9	0.969923	0.903669	1.041036	0.39759
NUDT14	1.291904	1.207551	1.382149	1.05E-13
PNMA3	1.129745	1.035788	1.232226	0.005893
IQGAP3	1.098114	1.036214	1.163712	0.001569
TOB2	0.933866	0.868095	1.00462	0.066318
SCN5A	0.969143	0.841118	1.116655	0.664587
ARSI	0.98766	0.945313	1.031904	0.578661
UTY	1.013648	0.986149	1.041914	0.33403
TTC32	0.99709	0.911523	1.090688	0.949235
DNAH2	1.059219	0.964628	1.163085	0.228044
SH2D1A	1.021803	0.975891	1.069875	0.35781
PRKX	0.92105	0.859362	0.987166	0.020063
KCNH8	0.931043	0.848387	1.021752	0.131988
SMTN	1.193019	1.089761	1.306061	0.000133
NPW	0.845227	0.808808	0.883285	7.28E-14
ST6GALNA	1.300738	1.165095	1.452173	2.88E-06
PTP4A2	0.939653	0.862771	1.023387	0.152954
ACTG1	0.918469	0.850778	0.991545	0.029456
DENND5A	0.838506	0.785973	0.89455	9.52E-08
DIABLO	0.974687	0.915141	1.038107	0.425353
VPS33B	1.198301	1.043113	1.376577	0.010575
TBX1	0.950363	0.886836	1.018441	0.149222
ADAP2	1.0547	0.998158	1.114445	0.058175
FAM120C	1.046271	0.95762	1.143129	0.316667

BRD7P2	1.0309	0.867319	1.225332	0.729937
EIF3C	0.850261	0.765328	0.944619	0.002519
CLDN5	1.113377	1.022254	1.212623	0.013694
NIPSNAP1	1.032572	0.956484	1.114713	0.411799
LRTOMT	1.099167	1.009574	1.196711	0.029286
ADRA2C	0.886378	0.837416	0.938204	3.18E-05
NR2C2AP	1.191445	1.074937	1.320581	0.000849
CRELD2	1.144586	1.04723	1.250993	0.002906
SCFD2	1.089803	1.026453	1.157061	0.004886
UBE2F	1.079138	0.983282	1.184338	0.108552
KCNJ12	1.147428	1.028267	1.280397	0.013962
GPR173	1.097208	1.002146	1.201288	0.044821
PPP1R2	0.956061	0.876907	1.04236	0.308174
TSPYL2	0.934829	0.884455	0.988071	0.017099
PGP	1.112088	1.009299	1.225345	0.031792
SNRNP35	1.110265	0.975256	1.263964	0.113829
IRAK1	1.151777	1.047833	1.266032	0.003409
OLIG1	0.997744	0.952974	1.044617	0.923186
PCDH9	1.181189	1.113735	1.252728	2.85E-08
ACOT1	1.19487	1.077161	1.325442	0.000766
OAF	0.953382	0.903019	1.006555	0.084704
KCNK12	1.017777	0.900983	1.14971	0.776916
POU6F1	1.027856	0.943209	1.120101	0.530927
TM2D3	1.069852	0.977352	1.171106	0.143347
TSSC4	0.950566	0.863205	1.04677	0.302687
TACSTD2	0.929502	0.885656	0.975518	0.003023
CLECL1	1.057011	1.011115	1.104991	0.014365
ZDHHC23	1.078124	1.009632	1.151261	0.02469
SRPK3	1.149494	1.045012	1.264424	0.004163
EFNA5	1.097404	1.007859	1.194905	0.032337
PKP3	1.072394	0.953834	1.205691	0.242303
MAP7D2	1.034285	0.93479	1.14437	0.513601
CSF1	1.120265	1.073105	1.169497	2.28E-07
PLA2G6	1.069603	0.988315	1.157576	0.095213
MAML2	0.896667	0.840692	0.956369	0.000912
A3GALT2	0.956433	0.877865	1.042031	0.30842
SS18L1	1.102678	1.020669	1.191277	0.013182
TOP1MT	1.016073	0.954915	1.081148	0.614657
COPB2	1.038516	0.953049	1.131648	0.388417
LRRC19	1.04724	0.941001	1.165474	0.397694
THAP7	1.263253	1.146596	1.391779	2.28E-06
KNTC1	1.051578	0.991412	1.115396	0.09432
CCR10	0.975117	0.855476	1.11149	0.70596
WDR27	1.053372	0.986005	1.125342	0.123074
TXNRD2	1.092659	0.9965	1.198097	0.059381
FOXO4	0.951495	0.892579	1.0143	0.127361
PTP4A3	1.12167	1.067214	1.178906	6.13E-06
FOXD4L1	1.067475	0.963236	1.182995	0.212948
PROS1	1.225728	1.164962	1.289664	4.31E-15
GAST	0.940296	0.852921	1.036622	0.21603
HDDC3	1.300651	1.14414	1.478573	5.86E-05
BEX5	1.187872	1.08047	1.30595	0.00037
ZFP1	1.036115	0.95306	1.126408	0.405292
CEND1	1.124855	0.985629	1.283747	0.080944
DUSP8	0.933783	0.895704	0.97348	0.001259
SOCS3	1.040474	0.992554	1.090708	0.099088
LPAR5	0.885605	0.831001	0.943798	0.000183
XPOT	1.003264	0.927979	1.084657	0.934736

PDE4B	0.91389	0.866344	0.964045	0.000956
SNN	0.943107	0.886262	1.003598	0.064784
KCNH7	0.947636	0.826434	1.086612	0.441115
NELL2	0.986907	0.934787	1.041934	0.634018
KRBA2	0.978307	0.910877	1.05073	0.54725
MED12	0.950351	0.885854	1.019544	0.155559
ZNF93	0.91904	0.840248	1.00522	0.064875
CDCA2	1.11585	1.047624	1.188519	0.000661
ZBTB40	0.99104	0.924982	1.061816	0.798171
EIF4ENIF1	1.062987	0.975524	1.158292	0.163221
LRRC26	0.972878	0.86301	1.096733	0.652901
ATL3	1.033954	0.962408	1.11082	0.361422
NDUFA12	1.158327	1.059861	1.265941	0.001184
TCTE3	1.107333	1.007	1.217661	0.035385
UBE2G2	1.119269	1.020856	1.227169	0.016415
OSBP2	1.012038	0.969977	1.055924	0.580602
APOO	1.29208	1.14409	1.459212	3.65E-05
PRR16	1.138949	1.079276	1.201921	2.15E-06
TMED9	1.052326	0.960801	1.15257	0.271937
TMEM186	1.178967	1.07223	1.296328	0.000673
SDR42E1	1.19301	1.060541	1.342025	0.003295
RBM33	0.902905	0.839717	0.970848	0.005794
ARMCX2	1.373724	1.245448	1.515213	2.18E-10
BTBD6	1.165174	1.069038	1.269956	0.000502
RBM43	1.156141	1.059565	1.26152	0.001114
SUMO3	1.032025	0.934138	1.14017	0.535267
IMMP2L	1.004242	0.929027	1.085546	0.915139
JAG2	1.182981	1.090328	1.283509	5.39E-05
FMNL1	0.922637	0.852158	0.998945	0.047032
LCN12	1.298581	1.094624	1.54054	0.002726
WT1	0.981489	0.937705	1.027318	0.422298
ZFP90	1.117845	1.0314	1.211535	0.006671
MUC6	0.917923	0.845563	0.996475	0.040928
NOC4L	1.108053	0.995433	1.233414	0.060619
USP18	1.155442	1.095666	1.21848	9.77E-08
NDUFA6	1.174399	1.06182	1.298915	0.001768
CHRM5	0.998251	0.871078	1.14399	0.979908
SORCS2	1.12562	1.049026	1.207807	0.000998
TMEM121	1.067792	0.986008	1.15636	0.106664
TMEM106	0.910785	0.850518	0.975324	0.007467
SIVA1	1.038701	0.934441	1.154593	0.481704
BRI3BP	1.055726	1.000541	1.113954	0.047734
DGAT1	1.142186	1.039086	1.255515	0.005881
ROBO2	1.035065	0.954356	1.122599	0.405379
AP3M1	0.966358	0.886246	1.053712	0.43832
F8	1.260951	1.091545	1.456648	0.001633
CA13	1.269922	1.176832	1.370376	7.65E-10
UBOX5	1.021259	0.935196	1.115242	0.639544
MAFF	0.939569	0.890299	0.991565	0.023318
BRF1	1.067179	0.98683	1.154071	0.103523
SEMA4B	1.000181	0.934441	1.070546	0.995836
CIB1	1.066484	0.961758	1.182615	0.222248
SLC24A3	0.939244	0.902534	0.977446	0.00206
INTS5	1.0236	0.945366	1.108309	0.565291
RPS27L	1.1084	0.985885	1.246141	0.085052
MANEAL	1.136288	1.044796	1.235792	0.002853
ANO9	1.097992	1.022432	1.179136	0.010176
FAF1	1.069917	0.976588	1.172166	0.146715

FAM43A	1.120254	1.054581	1.190016	0.000229
HSF1	0.910156	0.82462	1.004565	0.06155
PURA	1.155763	1.073497	1.244334	0.000122
INPP5J	1.023715	0.929702	1.127233	0.633448
MIXL1	1.216546	1.066436	1.387786	0.003531
MFSD6L	0.997102	0.916673	1.084587	0.946069
LRRC37B	0.902926	0.835226	0.976114	0.010231
DDX51	1.12106	1.027957	1.222596	0.009786
NOMO2	0.97318	0.902018	1.049956	0.482856
SIGIRR	1.094194	0.996042	1.202019	0.060483
NRBP2	1.30016	1.203736	1.404309	2.45E-11
IFITM2	1.103125	1.045432	1.164002	0.000342
TNFAIP2	1.085576	1.034192	1.139512	0.000904
ZNF445	1.002021	0.941264	1.0667	0.949548
PGBD2	1.117491	1.040344	1.200358	0.002337
RAB11B	0.957071	0.853935	1.072664	0.45072
PRMT3	1.111165	1.02371	1.206091	0.011727
GP1BA	1.125946	1.063772	1.191754	4.26E-05
PRPF39	0.998786	0.922646	1.08121	0.97605
PPIL6	1.294449	1.134336	1.477161	0.000128
ZNF74	1.086138	1.010821	1.167067	0.024228
CDNF	0.939327	0.850593	1.037319	0.216355
KLHL33	1.048625	0.973913	1.129068	0.208018
RBM11	1.301507	1.168516	1.449633	1.65E-06
GALNT17	1.043012	0.960417	1.132709	0.317084
ZBTB37	1.034588	0.968009	1.105747	0.316379
IL3RA	1.025827	0.947646	1.110457	0.528409
CCDC137	1.241864	1.109237	1.39035	0.000171
RGPD2	1.002089	0.937448	1.071188	0.951089
ARL15	1.081838	1.010857	1.157803	0.023096
CDK10	1.080112	0.99767	1.169367	0.057122
TMEM105	1.154577	1.094068	1.218433	1.67E-07
SOCS1	1.036094	0.986213	1.088498	0.158981
TCN2	0.992347	0.942786	1.044512	0.768825
GAS2L1	0.948712	0.894898	1.005761	0.077204
ATP6V0A2	0.98319	0.916308	1.054954	0.637187
HGS	0.977349	0.900418	1.060852	0.583868
TNFAIP8L1	0.900906	0.836754	0.969976	0.005627
MAPK11	0.995245	0.937567	1.05647	0.875652
SP140L	0.975172	0.9012	1.055216	0.532204
MRPL30	1.031458	0.936944	1.135506	0.527602
SMYD3	1.020676	0.960652	1.084451	0.508097
METTL7A	1.020459	0.9582	1.086762	0.528335
FAM174B	1.020943	0.939306	1.109675	0.625943
TMEM179I	1.114743	0.999308	1.243512	0.051469
GPRIN3	1.022614	0.966796	1.081655	0.434884
STAC3	1.073141	1.006051	1.144706	0.032104
MUC1	1.114978	1.027528	1.20987	0.009012
IRF7	0.981294	0.911853	1.056022	0.614062
BRCC3	1.125909	1.021342	1.241181	0.017099
PDE6G	0.942353	0.882498	1.006267	0.076168
NR2F2	1.596584	1.165404	2.187293	0.00358
DLK1	0.931232	0.87163	0.994909	0.034753
AHNAK2	1.128388	1.040855	1.223283	0.003369
OLFML2A	1.123894	1.078027	1.171713	3.93E-08
SP1	0.955074	0.891122	1.023616	0.193642
WASH3P	0.942926	0.863234	1.029974	0.19209
MRPL40	1.03777	0.918595	1.172406	0.551388

PDIA2	1.024607	0.93379	1.124257	0.607708
PCGF3	1.002246	0.927792	1.082675	0.954565
LMLN	1.171628	1.050738	1.306427	0.004362
P4HB	0.910443	0.851364	0.973622	0.006127
PSMD13	1.123966	0.983139	1.284965	0.087082
PBX1	1.154442	1.106324	1.204652	3.80E-11
NDUFA4L2	1.174112	1.032836	1.334712	0.014132
ZFP36L1	1.030092	0.980468	1.082228	0.239224
UBE2L3	0.977236	0.862124	1.107718	0.718763
BRWD1	0.975529	0.916605	1.03824	0.435741
SNAI3	1.050497	0.972665	1.134557	0.209735
ZBTB3	1.032037	0.946644	1.125132	0.474225
LYG2	1.053559	0.959572	1.156751	0.2738
PRAME	0.953809	0.926804	0.981602	0.00125
MYBL1	1.224456	1.109584	1.351219	5.61E-05
DRG1	0.976424	0.871122	1.094454	0.681964
ANKFY1	1.014121	0.946987	1.086014	0.68823
YTHDF3	0.915093	0.856596	0.977585	0.008474
ZNF696	1.128776	1.039683	1.225504	0.003881
ADARB2	0.995618	0.88907	1.114935	0.939386
IFIT1	1.06259	1.011765	1.115968	0.015195
KCNQ5	0.877405	0.831009	0.926392	2.38E-06
MORF4L1	0.928685	0.845819	1.01967	0.120783
NLRP9	1.144855	1.012127	1.294988	0.031421
WDR53	1.144183	1.017264	1.286938	0.02475
DMWD	0.999458	0.926249	1.078453	0.988853
PIGP	0.880729	0.780633	0.99366	0.039086
IKZF1	1.023301	0.949961	1.102302	0.543819
PCYT2	1.070087	0.976877	1.17219	0.14516
NAT8L	1.087472	0.999692	1.182959	0.050843
BCAP31	1.066276	0.958733	1.185882	0.236792
GNB1L	0.957931	0.888019	1.033348	0.266328
DNAH14	1.042428	0.958904	1.133228	0.329485
EVI2B	0.900922	0.852786	0.951774	0.000196
ZNF829	1.109634	0.998151	1.233569	0.05414
THNSL1	1.112312	1.021657	1.211011	0.014131
TRIM69	1.017514	0.944152	1.096577	0.649279
ATP6V0C	0.965311	0.909577	1.02446	0.244608
IFITM1	1.048409	1.000836	1.098244	0.046018
LAMP1	0.947525	0.867282	1.035193	0.232525
TAS2R60	0.997278	0.936923	1.061522	0.931817
KLHDC8B	1.087881	1.022874	1.157018	0.007376
SETD4	1.199637	1.091644	1.318313	0.000156
PTCH1	1.070494	0.994911	1.151818	0.068241
RNPC3	1.068928	0.987514	1.157054	0.099123
ZNF267	1.044861	0.976108	1.118457	0.206361
IRS2	0.887323	0.842388	0.934656	6.53E-06
BICD2	0.969614	0.899639	1.045033	0.419436
CCIN	1.012326	0.944693	1.084803	0.728398
TMLHE	0.994981	0.908502	1.089692	0.913633
SDHAP3	1.236869	1.155478	1.323993	9.30E-10
RASA3	1.091138	1.018909	1.168487	0.012559
LRCH3	0.98878	0.919789	1.062947	0.759791
NDUFA13	1.022611	0.942638	1.10937	0.590466
ZNF566	1.05267	0.958377	1.15624	0.283702
ZNF529	0.90903	0.848065	0.974378	0.007086
ZNF284	1.265274	1.120451	1.428816	0.000148
DLEU7	0.906692	0.815041	1.008648	0.071609

KRT73	1.094783	0.979297	1.223888	0.111353
AIDA	1.007409	0.93061	1.090547	0.855226
CD300LF	1.122622	1.063768	1.184732	2.55E-05
CYP2R1	0.916491	0.842318	0.997196	0.042848
LRRC70	0.990569	0.901481	1.088461	0.843766
ANKRD46	1.131438	1.032467	1.239896	0.008191
PIP5K1C	0.97756	0.904046	1.057052	0.569364
CYP4F2	0.849096	0.798629	0.902753	1.67E-07
ZBTB6	1.084879	0.997868	1.179478	0.056143
TAS2R42	1.000763	0.863945	1.159247	0.991889
POLR3C	1.026324	0.919734	1.145266	0.642342
WWOX	1.137708	1.048665	1.234313	0.001918
BCL9L	0.991975	0.926189	1.062433	0.817976
POLR1D	0.891217	0.779	1.019598	0.093483
KIF18B	1.119056	1.05469	1.187351	0.000198
ZNRF1	1.021243	0.95267	1.094752	0.553354
CYP4F12	1.162939	1.028772	1.314602	0.0158
ZNF749	1.049875	0.964096	1.143286	0.263068
BTLA	0.954739	0.892459	1.021366	0.178394
ZNF17	1.072893	0.987944	1.165147	0.094569
GPAT2	1.124222	1.037055	1.218716	0.004461
TOR3A	1.050227	0.951684	1.158973	0.329635
PPP1CC	1.13782	1.030312	1.256545	0.010783
ZNF555	0.980548	0.917071	1.048418	0.565098
NAP1L3	1.220246	1.14396	1.30162	1.51E-09
PRELID2	0.938076	0.872252	1.008868	0.08505
BACE1	1.075763	0.997028	1.160715	0.059672
RGS9BP	1.105963	1.002382	1.220248	0.044709
SLC36A3	0.900012	0.807906	1.002619	0.055815
RXRA	1.116872	1.05456	1.182865	0.000161
ANKRD37	1.119922	1.030981	1.216535	0.007305
NUDT17	1.015311	0.9186	1.122203	0.766068
ZNF75D	1.091381	1.005116	1.18505	0.037394
KRT10	1.140601	1.063599	1.223178	0.000225
CD300E	1.027168	0.993446	1.062035	0.115516
NKRF	0.953962	0.863156	1.054321	0.355746
GLDN	1.133078	1.057189	1.214415	0.000412
FCAR	0.994283	0.961588	1.028089	0.736783
KPNA4	0.991357	0.916146	1.072743	0.829273
ZNF501	1.21018	1.083006	1.352287	0.000758
ZNF197	1.132229	1.040396	1.232169	0.004008
SPATA12	1.047936	0.962056	1.141482	0.283149
AQP7P1	1.063897	0.989636	1.14373	0.093395
RPS23	0.909712	0.841783	0.983122	0.016854
GNG2	0.867784	0.822565	0.915489	2.06E-07
BTN3A2	1.060947	0.996102	1.130013	0.065975
AKAP14	0.965899	0.891431	1.046589	0.396677
INSIG1	1.04771	0.971412	1.130001	0.226997
ZNF396	1.251179	1.068254	1.465426	0.005458
TMEM222	1.02631	0.926781	1.136528	0.617796
ARHGAP3(	0.99345	0.923076	1.069189	0.860835
CYP4F3	1.045333	1.006996	1.085129	0.020037
SMYD4	1.015428	0.944539	1.091637	0.678406
GPATCH8	1.041268	0.950451	1.140764	0.385106
CEACAM1	1.179011	1.086532	1.279362	7.77E-05
NF2	1.080307	1.000613	1.166347	0.048195
SPATC1	1.261144	1.185067	1.342105	2.70E-13
UBE2H	0.90398	0.852491	0.958578	0.000741

HPDL	1.096663	1.009063	1.191867	0.029827
KATNA1	0.948012	0.857848	1.047652	0.295087
ARAP1	1.025281	0.964886	1.089457	0.420246
KIF24	1.054918	0.985894	1.128774	0.121501
PDE2A	0.892562	0.83013	0.95969	0.002126
PRG2	0.875359	0.815649	0.93944	0.000222
PRR5	1.089543	1.016955	1.167313	0.014773
ZFP91	0.945701	0.878676	1.017839	0.136613
BCDIN3D	1.18761	1.048418	1.345283	0.006864
LYRM7	1.027992	0.955	1.106563	0.462534
BCR	0.912805	0.862303	0.966265	0.00168
FSCN2	1.104349	1.017194	1.19897	0.017961
SPIN4	1.066228	0.994316	1.143341	0.071866
SPIN2B	0.989816	0.885908	1.105912	0.856453
HYAL3	0.979463	0.923058	1.039314	0.492897
CXCR3	1.075644	1.011826	1.143488	0.019455
ZNF397	1.03283	0.959147	1.112174	0.392318
TPCN1	1.055757	0.987688	1.128517	0.110568
LILRB4	1.082688	1.042571	1.124348	3.72E-05
TNFRSF4	1.041175	0.993896	1.090703	0.088804
HEXIM1	1.05215	0.981316	1.128098	0.15284
PDZD7	1.129171	1.052267	1.211695	0.000737
POFUT2	0.980415	0.892009	1.077584	0.681645
ERCC6L	1.16076	1.066745	1.263061	0.000542
TMEM17	1.103965	0.992721	1.227674	0.067977
TNFRSF18	1.049683	1.002442	1.099151	0.039039
RTN4RL2	1.002154	0.909665	1.104046	0.965266
ZDHHC17	0.975455	0.906829	1.049276	0.50435
P2RY4	0.997414	0.845395	1.176769	0.975513
ZNF395	0.947742	0.893788	1.004954	0.072699
ZACN	1.058082	0.963123	1.162404	0.239283
PPARA	1.136102	1.046051	1.233906	0.002458
KANK3	0.961595	0.883122	1.047042	0.367258
EMID1	1.013209	0.970679	1.057601	0.54866
ACTL7A	1.070616	0.938574	1.221235	0.309616
RHD	1.025277	0.979599	1.073084	0.28303
ESPN	1.042738	0.97889	1.110749	0.194237
GPR141	1.007575	0.96118	1.056209	0.7537
TMPRSS6	1.355052	1.157282	1.586619	0.00016
TMEM216	0.914497	0.836823	0.999381	0.048424
RPS19BP1	1.020618	0.906258	1.149409	0.736429
TEAD1	0.838521	0.738055	0.952663	0.006836
OR2AK2	1.132592	1.029509	1.245997	0.01055
PLCD1	1.073293	0.990679	1.162796	0.083486
ENTPD5	1.04497	0.946234	1.154009	0.385044
MITF	1.07617	0.997893	1.160587	0.05675
HEATR4	1.119499	1.018122	1.230969	0.019763
NAP1L1	0.856515	0.785471	0.933985	0.000455
LILRA5	1.06282	1.02398	1.103133	0.001339
CMC1	1.135075	0.996662	1.29271	0.056188
SLIT1	1.161979	1.05714	1.277216	0.00186
AKR1C1	1.109098	1.037527	1.185606	0.002347
SPATA21	0.960912	0.904812	1.02049	0.193906
RNF220	1.089139	1.017702	1.165592	0.013629
ZNF546	0.991283	0.917046	1.07153	0.825527
TSPYL4	1.103264	1.012611	1.202034	0.024677
MT1X	1.197026	1.127547	1.270787	3.75E-09
GCNT1	1.093416	1.031759	1.158758	0.002564

SESTD1	1.014495	0.965003	1.066525	0.572796
FNBP1	1.011382	0.929025	1.101039	0.793974
DYNC2H1	1.2642	1.169929	1.366067	3.04E-09
BCAM	1.180871	1.110142	1.256106	1.32E-07
RSBN1L	0.954342	0.883783	1.030534	0.23307
WDR86	1.037097	0.965765	1.113699	0.316411
EPOR	1.074804	1.019315	1.133314	0.007645
TAF9B	1.00799	0.930026	1.092489	0.846372
PCDHB13	1.116485	1.001748	1.244364	0.046424
FPR3	1.091845	0.973399	1.224704	0.133671
KCNJ11	0.964019	0.855385	1.08645	0.548029
COL4A1	1.323399	1.172112	1.494212	6.07E-06
GJA4	1.18865	1.114431	1.267811	1.49E-07
PTMA	0.96537	0.88303	1.055387	0.438441
HSPA14	0.961319	0.883505	1.045986	0.359663
SIRT7	1.171403	1.057792	1.297216	0.002371
IFT140	1.035592	0.958874	1.118447	0.373159
TLR5	1.106069	1.027549	1.19059	0.00729
USP7	0.960413	0.887785	1.038983	0.314044
NANOS3	0.94295	0.852144	1.043431	0.255523
NHLRC1	1.170314	1.059696	1.292479	0.001906
COX8C	1.10428	1.006232	1.211881	0.036535
PLEKHN1	1.115671	0.99062	1.256507	0.071139
ZNF385C	0.934387	0.864468	1.00996	0.087228
MAGEH1	1.254393	1.161586	1.354616	7.50E-09
TET3	0.942135	0.877904	1.011066	0.098025
ZNF286A	1.226897	1.108549	1.35788	7.78E-05
ISG15	1.091803	1.038397	1.147956	0.000598
EXD3	1.04062	0.953403	1.135816	0.372643
ZKSCAN4	1.036147	0.955925	1.123101	0.387784
DHRS4L2	1.167783	1.052587	1.295587	0.003421
SAMD11	0.799206	0.737387	0.866209	4.85E-08
VMAC	1.021976	0.951364	1.09783	0.551784
SPRY4	0.947164	0.855037	1.049218	0.298468
TRPV2	1.011093	0.944395	1.082501	0.751369
TMEM203	1.072228	0.979744	1.173443	0.129693
KBTBD12	1.198298	1.076427	1.333968	0.000947
DNAJB13	1.025023	0.947217	1.109219	0.539469
TCEA1	0.948387	0.86948	1.034455	0.231832
NHEJ1	1.127832	1.003212	1.267932	0.044046
FANCA	1.073058	0.994775	1.157501	0.068088
SECISBP2	1.000722	0.933738	1.072511	0.983705
OR52B6	0.999029	0.911305	1.095198	0.983475
SEMA4D	1.109048	1.036585	1.186577	0.00268
LIN28B	1.034087	0.975026	1.096726	0.26396
DNAH17	1.067181	0.973676	1.169665	0.1646
MCRS1	1.164707	1.052025	1.289458	0.003315
TMEM72	1.153524	1.07669	1.235841	4.89E-05
FANCM	1.064169	0.976585	1.159608	0.155816
ZNF70	0.977966	0.918254	1.04156	0.488211
CARD9	0.990682	0.934649	1.050074	0.75266
PEAR1	0.981513	0.927828	1.038303	0.515551
TMEM220	0.96042	0.879484	1.048804	0.368596
EIF4EBP1	1.079655	1.011765	1.152101	0.020725
P2RX2	1.072445	0.973417	1.181547	0.157095
TTC24	0.975824	0.921208	1.033679	0.404972
FAM122A	1.098757	0.990216	1.219196	0.07595
CLEC17A	0.931919	0.866271	1.002541	0.058508

LDLRAD2	1.026061	0.958217	1.098709	0.461055
PMS2CL	0.961999	0.887044	1.043289	0.349248
CYHR1	1.097063	0.998634	1.205193	0.053425
COL14A1	0.868898	0.797276	0.946954	0.001366
CPSF4L	0.973033	0.867729	1.091117	0.639942
KLHL17	1.176582	1.091026	1.268848	2.43E-05
PLA2G2C	1.198971	1.101085	1.305559	2.97E-05
ZSCAN23	0.81759	0.730745	0.914756	0.00044
RINL	1.217184	1.131621	1.309216	1.26E-07
MORN2	1.192881	1.092098	1.302964	9.00E-05
S100A3	1.301837	1.168679	1.450166	1.66E-06
UBQLN2	0.965754	0.881058	1.058591	0.456817
RILPL1	0.942469	0.860142	1.032676	0.203905
ZNF490	1.030876	0.941319	1.128953	0.511955
CLCN1	1.095652	0.993493	1.208317	0.067365
ARL4C	1.126512	1.073486	1.182156	1.28E-06
TREML4	1.114715	1.045285	1.188758	0.000934
RAB42	1.135969	1.025714	1.258076	0.014391
SCGB1C1	0.979569	0.888035	1.080537	0.680025
GPR89B	1.16283	1.057904	1.278163	0.001768
EYS	0.9766	0.868618	1.098006	0.692061
MAPK12	0.972996	0.910828	1.039407	0.416425
COL4A5	1.033872	0.992273	1.077215	0.111893
AGRN	0.866844	0.821094	0.915142	2.40E-07
FAM166A	0.963607	0.92399	1.004922	0.083496
ZNF626	0.879973	0.793034	0.976442	0.01599
HEPACAM	1.029591	0.974884	1.087367	0.295173
ZC3H6	0.990054	0.912342	1.074387	0.8106
PRKAR1B	1.009904	0.933396	1.092682	0.806315
DCUN1D3	1.0169	0.951127	1.087221	0.623271
POTEE	0.894054	0.740152	1.079958	0.245278
ZNF793	1.083268	0.979507	1.198021	0.119491
COMMD6	0.939306	0.867196	1.017413	0.124447
IL17REL	1.023943	0.939224	1.116304	0.591285
RUFY4	1.200787	1.131271	1.274575	1.81E-09
ZNF383	1.102099	1.011263	1.201095	0.026749
HES4	0.983908	0.93921	1.030734	0.494057
ZNF669	0.982177	0.903274	1.067972	0.673837
CENPP	1.13804	1.035295	1.25098	0.007396
PLSCR1	1.058833	0.988725	1.133912	0.101933
ZNF559	1.100462	1.019405	1.187965	0.014195
SBK1	1.053607	0.9808	1.131818	0.152913
SLC38A3	0.87636	0.775818	0.989932	0.033777
OR6N2	1.130823	0.984839	1.298446	0.081275
GTF2F2	0.983455	0.884376	1.093634	0.758134
PRR19	0.89767	0.78509	1.026393	0.114348
ZP3	0.934191	0.84508	1.032699	0.183223
PDCD1	1.034459	0.996711	1.073638	0.074059
CLEC2A	1.194794	1.142346	1.249649	7.80E-15
GPR21	1.074638	1.014342	1.138518	0.014553
TCTEX1D4	1.011492	0.960735	1.064932	0.663554
SELL	0.988369	0.950498	1.027748	0.557253
CHM	1.031016	0.954402	1.113781	0.438149
CERKL	1.036177	0.90787	1.182617	0.598257
IER5L	0.993686	0.928997	1.06288	0.853679
FAM83G	1.019614	0.949036	1.095441	0.595607
HBA2	0.97592	0.953024	0.999366	0.044183
DUSP28	1.137694	1.032139	1.254043	0.009412

NBR1	1.15047	1.06081	1.247708	0.000709
RALGAPA2	0.937254	0.884292	0.993389	0.029001
NDOR1	0.995829	0.919213	1.078831	0.918495
NKAIN2	1.236165	1.170285	1.305754	3.26E-14
PAQR9	1.083591	1.004146	1.169322	0.038785
CLN3	1.300565	1.169141	1.446764	1.33E-06
FAM72B	1.216104	1.103578	1.340104	7.83E-05
SUMO2	1.103012	0.979817	1.241695	0.104687
NANOS1	1.006577	0.924678	1.095731	0.879659
HMX3	1.050028	1.014432	1.086872	0.005532
DPYD	0.984926	0.936933	1.035377	0.551222
S100A16	0.991857	0.953689	1.031554	0.683012
PTAR1	1.012535	0.938339	1.092598	0.748342
CC2D2B	0.974401	0.869306	1.092201	0.656064
RHCE	1.009167	0.965429	1.054887	0.686466
PARVB	1.090801	1.010321	1.177692	0.026247
SLC4A5	1.070664	0.915621	1.251959	0.392286
UROS	1.156438	1.053608	1.269305	0.002221
ZDHHC9	0.96161	0.880814	1.049818	0.381996
QRFP	1.049406	0.948968	1.160476	0.347475
TMEM120I	1.061625	0.973314	1.157948	0.17716
RBM34	1.080589	0.994061	1.174648	0.068746
NOXA1	1.095553	1.033188	1.161683	0.002275
BCL2L15	1.051154	0.968527	1.14083	0.232332
FZD9	1.139729	0.985357	1.318287	0.078185
SPRED3	0.962341	0.874214	1.059351	0.433414
ZNF548	0.999058	0.926627	1.077151	0.980427
MTF1	0.985494	0.910787	1.066329	0.716389
TMCO2	0.921576	0.822327	1.032804	0.160088
TMEM201	1.062011	0.973454	1.158625	0.17563
NHLRC3	0.96794	0.909292	1.030371	0.306886
HMX2	1.079117	1.023279	1.138003	0.004972
ZDHHC11	1.026228	0.92198	1.142263	0.635711
CNR2	0.960939	0.899621	1.026436	0.236267
ENTPD8	1.189072	1.0559	1.33904	0.00427
RPL14	0.847444	0.778183	0.92287	0.000142
BEND4	1.099807	1.039729	1.163356	0.000902
ZNF563	1.107249	1.013505	1.209663	0.023997
TMC3	0.968942	0.848795	1.106097	0.640433
FBF1	1.298373	1.153296	1.461701	1.57E-05
KLRG2	0.764245	0.692877	0.842965	7.65E-08
ASTL	0.947909	0.898345	1.000207	0.050889
MSL1	0.992681	0.916015	1.075764	0.857843
LRRK2	0.996781	0.955795	1.039525	0.880371
TRMT2B	1.103979	1.00908	1.207804	0.031001
FAM120A(	1.098977	0.98881	1.221418	0.079916
NOC2L	1.00559	0.925428	1.092697	0.895356
ZNF292	0.977422	0.922105	1.036058	0.442332
HUS1B	1.048008	0.96446	1.138794	0.268617
KCTD21	1.006206	0.910839	1.111558	0.90308
ADAT2	1.07848	0.999605	1.163579	0.051204
KIR2DL4	1.210116	1.099322	1.332075	9.91E-05
ZNF567	1.021539	0.936903	1.113822	0.629137
NDUFA4	1.073217	0.980254	1.174996	0.126377
ALKBH2	1.087495	0.984855	1.200833	0.097265
RNFT1	1.113625	1.012029	1.225422	0.027459
RELN	1.03619	0.936302	1.146735	0.491847
FAM111B	1.165201	1.093341	1.241783	2.51E-06

LITAF	1.007728	0.943531	1.076294	0.818692
VSTM1	0.891783	0.862402	0.922164	2.07E-11
TMEM120A	1.02404	0.935978	1.120387	0.604601
ARID2	1.000785	0.941358	1.063963	0.979961
SF3B3	1.021804	0.944955	1.104902	0.588722
BLOC1S3	1.192102	1.07838	1.317815	0.000592
SP6	1.193368	1.072944	1.327309	0.001125
PLAC9	0.954305	0.901457	1.010252	0.107599
ZNF573	1.064262	0.986807	1.147796	0.106206
ZNF527	1.08753	0.988706	1.196233	0.084296
S100A13	1.448971	1.285836	1.632803	1.16E-09
ZNF33A	0.988475	0.921352	1.060489	0.746633
ZNF600	1.127816	1.023833	1.24236	0.014801
BTBD8	1.092463	0.999637	1.19391	0.050944
MAOA	1.130309	1.03787	1.230983	0.004896
TSPYL1	1.053777	0.978476	1.134873	0.166129
PNRC2	1.030389	0.945535	1.122858	0.49478
FHIT	0.950335	0.871801	1.035943	0.247048
ZKSCAN3	1.01106	0.94664	1.079864	0.743329
RRP7A	1.114992	1.021451	1.2171	0.014904
LIN54	1.062765	0.972353	1.161583	0.179621
FAM53B	1.056325	0.987286	1.130192	0.112078
ALG1L	1.121162	0.992617	1.266353	0.065666
GSPT2	1.081643	1.005735	1.16328	0.034516
CXCL17	1.170969	1.069617	1.281924	0.000633
HMGB1	1.102104	0.993394	1.22271	0.066526
SH2D5	0.9664	0.879472	1.061919	0.477271
ZFP92	1.023616	0.954202	1.098078	0.514734
NCR1	1.08046	1.006064	1.160358	0.033499
BLOC1S2	1.161652	1.036539	1.301866	0.00996
SYCP2	1.132425	1.061287	1.208332	0.000172
IL1RAP	0.948725	0.891847	1.00923	0.095178
PAX5	0.990268	0.945018	1.037684	0.681936
ZNF699	1.072403	0.975123	1.179387	0.149659
TDRD7	1.040309	0.977623	1.107016	0.212671
KIAA0895L	1.127497	1.052908	1.20737	0.00059
HLA-DRB1	0.979994	0.942729	1.018731	0.306907
MYT1	1.187819	1.039686	1.357058	0.011321
AKR1C3	0.9763	0.931696	1.023039	0.314755
SPATS2L	1.367618	1.270443	1.472226	8.42E-17
ZNF250	1.10165	1.006815	1.205419	0.035044
WDSUB1	0.912199	0.843717	0.986239	0.021001
ZNF79	1.070901	0.990667	1.157633	0.084714
S100A4	1.084003	1.034538	1.135834	0.000712
PLEKHG4	1.101292	1.036204	1.170468	0.001908
FAT4	1.231321	1.13005	1.341667	2.01E-06
ZNF681	0.943775	0.861625	1.033759	0.212984
ACADSB	1.070376	0.99165	1.155353	0.081011
STK40	0.925629	0.85373	1.003582	0.061029
TMEM63A	1.029885	0.968264	1.095428	0.349555
CTSE	1.039367	0.98892	1.092387	0.128247
SEMA4A	1.035102	0.975691	1.09813	0.252645
MPHOSPH	1.09032	0.991934	1.198464	0.073115
GREB1	1.094025	1.014826	1.179406	0.019087
SIRPB2	0.923651	0.882941	0.966238	0.000554
ZNF766	1.041799	0.95938	1.131299	0.330145
RYR1	1.111993	1.017599	1.215142	0.019005
SRGAP3	1.203449	1.137459	1.273267	1.22E-10

TUBB	1.06463	0.979385	1.157294	0.141356
LCOR	1.026165	0.952357	1.105692	0.497652
SUPT5H	0.953673	0.865255	1.051126	0.339304
XPNPEP3	1.065707	0.965732	1.176033	0.205445
ZNF107	0.974224	0.902851	1.051239	0.501125
PPIA	0.927912	0.83676	1.028995	0.156137
ZNF471	0.922067	0.835307	1.017838	0.107554
ZNF836	1.103451	0.999583	1.218112	0.050974
ZNF493	1.017702	0.954536	1.085048	0.591449
GTF2IRD2	1.015499	0.909437	1.133931	0.784643
SUPT3H	1.150175	1.061496	1.246261	0.000631
NIF3L1	1.092511	1.008586	1.183418	0.030037
ATP2A1	0.994316	0.91255	1.083408	0.896412
POM121	0.838273	0.774498	0.9073	1.24E-05
ZBTB44	1.047619	0.972064	1.129047	0.223195
GIMAP5	1.109662	1.013524	1.214918	0.024417
NLGN3	0.947367	0.888123	1.010563	0.100788
CD55	0.895501	0.82753	0.969056	0.006136
ZNF565	0.930556	0.856949	1.010487	0.086926
NTNG2	0.871874	0.836144	0.909131	1.34E-10
WDR5	1.095135	1.000303	1.198958	0.049239
LONP1	0.943391	0.873721	1.018617	0.13656
TRRAP	0.964173	0.904427	1.027865	0.263617
NUDT11	1.288847	1.164939	1.425934	8.64E-07
FUT4	0.943175	0.896617	0.99215	0.023508
ASB13	0.947401	0.885764	1.013328	0.115435
ZNF34	0.871698	0.785912	0.966848	0.009382
ZNF140	1.03883	0.957467	1.127107	0.359945
INCA1	1.08882	1.014098	1.169049	0.018981
PTPN1	1.028499	0.942141	1.122771	0.530006
EVL	1.117921	1.047425	1.19316	0.000796
THEM5	1.087784	1.012233	1.168975	0.021962
EPHB4	0.9027	0.848537	0.960321	0.001185
PRTN3	0.967327	0.942393	0.99292	0.012659
ZNF765	1.179584	1.068747	1.301915	0.001036
ZNF124	1.047678	0.992173	1.106288	0.093536
XRCC6	0.97852	0.880633	1.087287	0.686367
S100A5	1.081033	0.979829	1.192691	0.120269
TSC22D2	1.050095	0.977383	1.128216	0.181837
ASMT	1.003413	0.875363	1.150193	0.960991
ZNF569	1.178037	1.063561	1.304835	0.001681
ARMCX4	1.098492	0.980331	1.230896	0.105696
YRDC	1.066574	0.980784	1.159867	0.131952
ZNF777	1.036268	0.954728	1.124773	0.394211
PIK3R4	0.947884	0.889268	1.010364	0.100301
ZNF775	0.90078	0.825951	0.982388	0.018199
ZNF605	1.059051	0.965301	1.161906	0.225054
TRAPPC2	1.048837	0.965616	1.139231	0.258291
MYL6B	0.999669	0.896254	1.115016	0.995261
ZNF799	0.988363	0.911424	1.071798	0.777121
SIAH1	0.908907	0.848958	0.97309	0.006078
IPO4	1.030507	0.929245	1.142803	0.569062
NCOR2	1.055486	0.960291	1.160118	0.262813
SULT1A1	0.985404	0.90091	1.077822	0.747854
ARL9	1.197275	1.082444	1.324288	0.000465
PRPF40A	0.948061	0.873991	1.028407	0.198764
GDAP2	1.059674	0.984561	1.140517	0.122305
TCEAL3	1.041108	0.963886	1.124517	0.30559

ANAPC7	1.065146	0.983513	1.153555	0.120825
TPK1	0.952645	0.874564	1.037697	0.266195
SLC6A9	1.051971	1.002388	1.104007	0.039705
AFAP1	0.87064	0.764778	0.991155	0.036235
NACA	0.814625	0.746407	0.889077	4.33E-06
MYO18A	1.003421	0.940897	1.070101	0.917127
MAN2A2	0.962349	0.899041	1.030115	0.268996
MME	1.169287	1.113744	1.2276	3.01E-10
FAM72A	1.356925	1.178216	1.56274	2.27E-05
CACNA1H	0.716186	0.655964	0.781936	9.41E-14
SULF2	0.986541	0.940878	1.034419	0.575192
HBG2	1.02512	0.995403	1.055725	0.098339
PLXNB2	1.016042	0.958264	1.077304	0.594185
XRCC2	1.101511	1.027743	1.180573	0.006262
MYO6	1.196094	1.144865	1.249616	1.08E-15
HDAC2	1.002642	0.910065	1.104636	0.957428
ZNF782	1.050402	0.980143	1.125697	0.16388
ZNF846	1.054285	0.968196	1.148029	0.223871
MMP1	1.297002	1.064941	1.579631	0.009723
TCF4	1.179038	1.121564	1.239457	1.05E-10
ZNF136	0.981106	0.918765	1.047677	0.569035
ZKSCAN5	1.006145	0.911716	1.110355	0.903026
ZNF502	1.166346	1.05954	1.283919	0.001688
TRAPPC4	0.998959	0.899694	1.109177	0.984444
TTC30B	1.088417	0.998377	1.186578	0.054469
TECPR2	1.001108	0.927702	1.080323	0.977258
TLR7	1.040345	0.973136	1.112196	0.245729
ZFP62	1.066087	0.986789	1.151758	0.104647
ERI2	1.018319	0.939419	1.103847	0.659082
TOMM7	0.940929	0.872546	1.014671	0.113735
HSH2D	1.108861	1.054334	1.166208	5.91E-05
TRPV1	1.123423	1.02529	1.230948	0.012578
ZNF33B	1.023255	0.946845	1.105832	0.56153
ZNF512B	0.995747	0.930539	1.065524	0.901831
AMZ2	1.133563	1.010122	1.272089	0.033075
ZNF431	0.988066	0.91866	1.062716	0.746641
NF1	0.975847	0.913305	1.042671	0.469384
VKORC1L1	1.081274	1.011461	1.155907	0.021756
ZNF418	0.998717	0.941168	1.059784	0.966172
DAPK1	1.019655	0.969213	1.072721	0.452099
HLA-DQA	0.941418	0.900968	0.983683	0.007056
COL27A1	1.082279	0.972716	1.204183	0.146507
GM2A	0.982709	0.91555	1.054794	0.62914
S100A2	1.106858	0.971287	1.261353	0.127773
ZNF700	1.004988	0.94392	1.070007	0.876385
CD47	0.962233	0.903203	1.025121	0.233317
TLE1	1.005531	0.952151	1.061904	0.842892
MAML3	0.968246	0.901408	1.04004	0.376584
STRN3	1.018584	0.952994	1.088687	0.58768
ZNF239	1.196606	1.091968	1.311272	0.000121
CHRNG	1.141186	1.053977	1.235611	0.00113
ZSCAN16	0.986427	0.906739	1.073117	0.750494
ADA	0.95594	0.899844	1.015533	0.144176
ARID5A	0.962694	0.901545	1.027989	0.256159
PPTC7	0.919173	0.857972	0.98474	0.016513
TOMM20L	0.917645	0.854259	0.985734	0.018602
RGPD4	0.90803	0.746002	1.105248	0.336012
NHLRC2	1.002316	0.930611	1.079545	0.951294

ZFP28	1.112964	1.006741	1.230396	0.036508
CBWD3	0.959311	0.842646	1.092128	0.530075
SCN8A	1.308707	1.164753	1.470452	6.04E-06
LAMB3	1.045705	0.989927	1.104627	0.110052
KPNA5	0.967708	0.892651	1.049077	0.425527
ANKRD36I	1.004474	0.942035	1.071051	0.891555
ARHGEF12	1.025707	0.979793	1.073772	0.277346
PDLIM7	1.079253	1.003838	1.160334	0.039055
FLNA	1.055853	0.990458	1.125566	0.095703
RPS26P11	1.040723	0.971128	1.115307	0.258338
SRGAP1	1.267571	1.172934	1.369842	2.11E-09
FAM3C	0.937289	0.862139	1.018989	0.128807
SLC39A10	1.008169	0.944212	1.076458	0.807775
CASP4	0.843647	0.769301	0.925177	0.000304
AP2A1	0.962536	0.881378	1.051167	0.395536
ZNF585A	1.136004	1.012709	1.27431	0.0296
FUT11	1.009625	0.930489	1.095491	0.818089
ANXA4	1.092573	1.010893	1.180852	0.025532
LAGE3	1.175931	1.075637	1.285577	0.000367
WDR5B	1.01489	0.950224	1.083957	0.659927
WDR45	1.11907	0.994368	1.25941	0.062003
METTL9	0.982609	0.886417	1.089239	0.738551
ZNF138	0.980985	0.900917	1.068169	0.658545
ZNF429	1.047203	0.96366	1.137989	0.276897
ZNF470	1.304255	1.147613	1.482278	4.72E-05
SERTAD1	0.951913	0.893206	1.014478	0.129167
ZNF100	1.010006	0.937261	1.088397	0.794048
ZNF398	1.048738	0.967339	1.136985	0.24833
ANXA6	0.962799	0.913456	1.014808	0.157848
ZNF441	1.071854	0.988049	1.162766	0.094816
GMFB	1.016246	0.935743	1.103673	0.701933
SIGLEC15	0.951314	0.889681	1.017218	0.144169
ZNF420	1.146299	1.043618	1.259082	0.00435
ZNF763	0.986815	0.880995	1.105346	0.818606
ZMYM1	1.03727	0.965589	1.114272	0.316567
MAFG	0.983722	0.921784	1.049823	0.620871
ARRDC1	0.906716	0.831891	0.98827	0.025849
KIAA1671	1.300073	1.127324	1.499294	0.000309
IGF2R	1.095133	1.045906	1.146677	0.000108
GAL3ST4	1.020831	0.954897	1.091318	0.545039
DYNC1H1	0.970141	0.912442	1.031489	0.332566
PCBP2	0.936835	0.852839	1.029103	0.173385
ZGPAT	1.076142	0.970481	1.193307	0.164012
SLC25A29	0.848829	0.801886	0.89852	1.64E-08
PGAP1	1.026171	0.951016	1.107267	0.505583
SRC	1.09464	1.039971	1.152183	0.000541
ZNF682	0.971861	0.89991	1.049565	0.46705
ZNF772	1.013381	0.94256	1.089524	0.719138
ZNF257	0.917472	0.839382	1.002827	0.057726
ADAM32	1.044391	0.939208	1.161354	0.422581
ACSL5	1.197044	1.104983	1.296776	1.06E-05
LRRC8B	0.921791	0.8679	0.979029	0.008061
ABCB8	1.161321	1.057026	1.275907	0.001839
SND1	0.91383	0.846894	0.986057	0.020247
ZNF785	1.078071	0.999412	1.16292	0.051805
SULT1A2	0.862483	0.753698	0.986971	0.031507
NEK5	1.238123	1.080443	1.418814	0.002118
PSMD12	0.961296	0.894411	1.033182	0.283362

PIWIL2	1.037018	0.913567	1.177151	0.574057
SLC22A4	0.958259	0.907624	1.011718	0.123715
ENTPD4	0.977735	0.917952	1.041412	0.484264
C1D	0.994626	0.892029	1.109025	0.922726
TBC1D9B	1.002975	0.927228	1.08491	0.940894
SERPINA1	0.951513	0.919732	0.984393	0.004137
TPSB2	0.886767	0.857737	0.91678	1.48E-12
KANK2	1.127957	1.060921	1.199228	0.000117
GTF2E2	1.039282	0.948709	1.138503	0.40756
IL27	1.170267	1.08258	1.265056	7.60E-05
GUCA2A	0.864188	0.739281	1.010198	0.066866
ZNF165	0.967578	0.905067	1.034407	0.333426
SYNGAP1	0.981847	0.913144	1.055718	0.620611
FITM2	1.154988	1.059083	1.259576	0.001123
BLM	1.104074	1.023598	1.190877	0.010348
ZNF720	1.038381	0.949691	1.135353	0.408353
DDI2	0.967544	0.905847	1.033442	0.326367
SVIL	0.972233	0.909396	1.039411	0.408772
TRIM33	0.961848	0.89281	1.036225	0.306028
LRP10	1.066834	0.979769	1.161636	0.136378
PELI1	1.030438	0.976726	1.087103	0.272304
ZNF655	0.932656	0.863663	1.00716	0.075402
MRPL21	1.066041	0.943704	1.204236	0.303812
LYPD2	0.931868	0.83833	1.035843	0.191054
UAP1L1	1.019008	0.957939	1.08397	0.550396
FBXL22	1.065463	0.984975	1.152529	0.1136
ZNF786	0.979226	0.909755	1.054001	0.576062
ZNF517	1.065327	0.968175	1.172227	0.194611
ZNF675	0.959492	0.879015	1.047338	0.354883
SLC22A5	1.265088	1.157918	1.382177	1.92E-07
DACT3	1.088276	1.004755	1.17874	0.037856
ADARB1	1.183974	1.111394	1.261295	1.68E-07
HTT	0.987659	0.92905	1.049965	0.690736
OR6N1	1.082292	0.941036	1.244753	0.267748
C5AR1	0.95362	0.920055	0.988409	0.009385
VEPH1	1.051801	0.898817	1.230824	0.528847
SHPK	1.326052	1.164261	1.510327	2.13E-05
IPP	1.310118	1.196287	1.43478	5.73E-09
MAP3K5	1.060666	0.995035	1.130627	0.070726
CYP2F1	1.265898	1.099368	1.457653	0.001051
GSTK1	1.01591	0.928466	1.11159	0.731049
HNRNPAB	1.10676	1.006597	1.216891	0.036099
STMN3	0.93314	0.872432	0.998073	0.043784
PDGFA	1.087336	1.022875	1.15586	0.007246
GYPE	1.026919	0.982387	1.07347	0.240248
SPN	1.026752	0.973434	1.08299	0.331883
ZNF695	1.010406	0.911315	1.120272	0.844163
ZNF628	0.859452	0.79218	0.932436	0.00027
SLC2A10	1.22692	1.154487	1.303898	4.49E-11
ZNF665	1.123999	0.980187	1.28891	0.094236
RPF2	0.949722	0.854915	1.055042	0.336351
SLC28A3	0.914881	0.867897	0.964409	0.000942
FAM177B	1.041521	0.914268	1.186486	0.540622
MIB2	0.956186	0.880584	1.038278	0.286373
MYO5A	0.935574	0.874323	1.001116	0.053894
GZMM	0.977815	0.916465	1.043272	0.497386
ATG7	1.029538	0.967504	1.095549	0.358574
SIPA1L1	0.86232	0.807852	0.92046	8.60E-06

TTC30A	1.274235	1.125465	1.442669	0.00013
ELANE	0.933069	0.911147	0.955518	1.12E-08
RAB40C	0.934952	0.863499	1.012317	0.097285
PIGN	1.093028	1.013581	1.178703	0.020871
ZNF624	1.033866	0.947305	1.128337	0.455342
HHLA3	1.022237	0.940891	1.110616	0.603168
HOXA4	1.166091	1.110879	1.224048	5.34E-10
TOPORS	0.943779	0.88065	1.011433	0.101394
BCO2	1.061077	0.938379	1.199819	0.344374
ENTPD6	1.018236	0.935553	1.108225	0.675779
KLKP1	1.07671	0.959584	1.208132	0.208446
FAR1	1.038995	0.975369	1.106772	0.235436
ZNF841	1.062498	1.006054	1.122109	0.029504
VN1R5	0.939891	0.850408	1.038789	0.224579
ZNF615	1.078139	0.994389	1.168943	0.068216
CDC42SE1	0.985368	0.916248	1.059702	0.691195
MPEG1	1.035454	1.005035	1.066793	0.022016
SERPINB2	0.988499	0.952844	1.025489	0.537152
DPP4	1.254795	1.142226	1.378458	2.21E-06
PDCD1LG2	1.306104	1.145287	1.489502	6.79E-05
ZNF433	0.970474	0.911863	1.032852	0.3457
DNAH10	1.243425	1.106173	1.397707	0.000261
SPTAN1	0.945386	0.881525	1.013874	0.115525
NMB	0.96553	0.882417	1.056472	0.444991
KLHL14	1.091665	1.002093	1.189244	0.04466
FAM114A1	0.880564	0.803001	0.96562	0.006859
RPE	1.040488	0.98028	1.104393	0.191873
ZNF460	0.983102	0.935148	1.033515	0.50417
CR1L	1.06827	1.014739	1.124624	0.011809
PHF2	1.047771	0.958312	1.145582	0.305445
RPS26	0.975383	0.927153	1.026121	0.335375
PSAP	1.019757	0.954235	1.089779	0.56366
S100A10	0.984743	0.951873	1.018748	0.374753
LHFPL5	0.981015	0.916916	1.049596	0.578242
RPL37A	0.889717	0.829832	0.953922	0.001013
CFD	0.928058	0.900547	0.956409	1.16E-06
EME2	1.127822	1.038325	1.225033	0.004351
KLHDC1	0.979505	0.902143	1.063501	0.621788
ZNF81	1.069959	0.991442	1.154694	0.082045
TAF13	0.99868	0.922943	1.080631	0.973808
ZNF780A	1.089868	1.011781	1.173982	0.023285
ATAD3A	0.914643	0.841402	0.994261	0.03616
FAM118B	1.046557	0.965349	1.134597	0.269495
ZNF461	0.977338	0.902492	1.058391	0.572819
SLC9A8	1.014694	0.939465	1.095947	0.710533
OCLN	1.093368	1.033997	1.156149	0.001727
ZNF181	1.16273	1.031794	1.310282	0.013382
ZNF44	0.952358	0.888353	1.020973	0.169067
GPAA1	1.179957	1.077957	1.29161	0.000334
ADAMTSL1	1.260207	1.150913	1.379879	5.83E-07
SGTB	0.998272	0.934154	1.06679	0.95927
ZNF790	0.956369	0.880608	1.038648	0.289402
PRB3	0.970799	0.895388	1.052561	0.472555
MYO1C	0.970959	0.917254	1.027809	0.310039
NKIRAS1	0.906016	0.844747	0.971729	0.005732
SLC22A12	1.020307	0.929657	1.119796	0.671944
KIF13B	0.93892	0.879487	1.002369	0.058886
ADH5	0.969382	0.882327	1.065026	0.517166

TEAD4	1.012593	0.937745	1.093415	0.749423
SPG7	0.981136	0.911787	1.055758	0.610608
ZNF677	1.125183	1.026945	1.23282	0.011394
ZNF823	0.937417	0.863125	1.018103	0.12501
ZNF311	1.362668	1.20191	1.544927	1.36E-06
ZNF347	1.079866	0.989977	1.177916	0.083133
PLCG2	1.042082	0.976551	1.11201	0.21353
FCHSD1	1.06642	0.992411	1.145948	0.079707
ZNF71	1.082048	0.995289	1.17637	0.064426
S100A6	1.093659	1.038725	1.151499	0.000662
RPL12	0.912884	0.848585	0.982055	0.01445
DNM3	1.201929	1.137422	1.270095	6.36E-11
ZNF121	1.040596	0.97473	1.110912	0.232952
MPZL1	1.036673	0.968979	1.109096	0.295867
VPS13A	1.049009	0.989168	1.11247	0.110367
MBP	1.16605	1.075328	1.264427	0.000201
ELOVL2	1.080184	0.964963	1.209162	0.180167
LEKR1	0.998619	0.872768	1.142618	0.98396
SNHG12	0.999908	0.937877	1.066041	0.997749
CLEC9A	1.091013	1.021686	1.165045	0.00931
KEL	1.022691	0.987225	1.059431	0.212773
NOL8	1.053561	0.970398	1.143849	0.21361
IRAK4	1.094515	1.006374	1.190375	0.035006
MRPL42	1.192579	1.08472	1.311163	0.000271
ENTPD7	1.056561	0.992465	1.124797	0.084872
FCGR1B	0.970992	0.898718	1.049077	0.455711
ZNF335	0.940306	0.877056	1.008117	0.083199
RPS4X	0.893884	0.831968	0.960409	0.002192
ZNF273	1.001108	0.92517	1.083279	0.978056
ZNF84	1.027547	0.950611	1.11071	0.493735
MAK16	1.116347	1.015427	1.227297	0.022807
ZNF667	0.969347	0.904254	1.039126	0.380047
AVPR1B	0.831155	0.747477	0.924201	0.000636
SIRPA	1.042544	0.985913	1.102428	0.143714
GRK6	1.0295	0.955104	1.109691	0.447441
PRIM1	1.102033	1.010986	1.201279	0.027223
SULT1C4	0.958684	0.902876	1.017942	0.167948
CD2AP	0.956394	0.895133	1.021846	0.1868
NUP62CL	1.239647	1.171031	1.312284	1.42E-13
SFI1	1.056356	0.981096	1.137389	0.145983
ZNF649	0.991114	0.912281	1.076759	0.832836
ZNF248	1.062116	0.98461	1.145723	0.119049
LPAR1	1.01172	0.945311	1.082795	0.736585
HIBCH	1.14235	1.060566	1.23044	0.000446
ZNF544	0.948876	0.894834	1.006182	0.079432
TMEM229I	1.216927	1.13636	1.303206	1.94E-08
ZNF770	1.009918	0.947076	1.07693	0.763349
MIER1	0.967182	0.894929	1.045268	0.399596
MAN1A2	1.02342	0.943403	1.110224	0.577308
SVIP	1.194284	1.09665	1.300609	4.50E-05
ZNF251	1.048294	0.958401	1.146618	0.302501
DDRGK1	1.058258	0.942119	1.188714	0.339731
TFDP1	1.024619	0.951804	1.103005	0.517868
CLEC4C	1.046357	0.980921	1.116158	0.169038
ZNF607	1.05924	0.963849	1.164072	0.231988
ZNF334	1.293301	1.207121	1.385634	2.67E-13
HSD17B11	0.760966	0.706446	0.819694	5.94E-13
ZXDA	0.934938	0.86765	1.007444	0.077505

RPS6KL1	0.886283	0.832162	0.943924	0.000173
QRICH1	0.933392	0.851944	1.022628	0.138966
CSF2RA	1.085248	1.021867	1.152559	0.00771
DDX42	0.917565	0.848869	0.99182	0.030246
RPL23A	0.958449	0.905927	1.014016	0.139969
SLC29A3	1.306924	1.19474	1.429643	5.05E-09
STYX	1.018553	0.936813	1.107426	0.666684
UBL5	1.129962	1.005785	1.26947	0.039678
HELZ	0.98771	0.918653	1.061959	0.738086
TMEM116	1.102143	0.991264	1.225425	0.072214
UCKL1	1.009092	0.897049	1.135129	0.880192
CARD11	1.020211	0.962365	1.081533	0.501663
ZNF485	1.168679	1.077177	1.267954	0.000179
SDAD1	1.171402	1.082299	1.267841	8.88E-05
HYLS1	1.101079	1.000197	1.212137	0.049532
MYL4	1.024385	0.989423	1.060583	0.173893
ZNF442	1.165668	1.045761	1.299323	0.005642
ZNF813	1.08101	0.989346	1.181167	0.084882
HOXC4	1.278117	1.175689	1.389469	8.53E-09
PIM3	0.956428	0.902416	1.013673	0.13308
ASPH	0.946295	0.884208	1.012741	0.11087
SPRED2	1.012093	0.950173	1.078047	0.709019
WWP2	1.039234	0.936353	1.153418	0.469347
GFPT1	0.935888	0.870011	1.006754	0.075205
UVRAG	0.947122	0.879343	1.020126	0.15157
ZNF26	1.066408	0.970057	1.172328	0.183269
ITSN2	1.001585	0.928574	1.080337	0.967277
NTRK1	0.89337	0.850148	0.93879	8.34E-06
MT1F	1.196596	1.135904	1.26053	1.40E-11
ZNF69	0.976632	0.915446	1.041909	0.473817
TXNRD1	0.952256	0.888045	1.021111	0.169611
NRARP	0.951797	0.886709	1.021663	0.171639
ZNF583	1.460245	1.250815	1.70474	1.64E-06
ZNF568	1.195386	1.064086	1.342889	0.002644
ZXDB	0.915437	0.8496	0.986377	0.020332
ZNF480	1.120429	1.020224	1.230476	0.017368
ZNF587	1.058074	0.981863	1.1402	0.138854
TPM2	1.135428	1.060518	1.215629	0.000265
SH3BGRL2	1.067694	1.004293	1.135099	0.035984
ZNF808	0.974444	0.909635	1.04387	0.460966
ANKRD35	1.125918	1.009994	1.255147	0.032408
YTHDF2	0.999885	0.920085	1.086606	0.99783
NBR2	0.969375	0.902226	1.041522	0.395761
HLA-DRB5	1.00102	0.966672	1.036588	0.954377
ATL1	0.969594	0.874708	1.074774	0.556778
MAFK	0.958538	0.905536	1.014642	0.144532
ZNF43	0.988501	0.934513	1.045607	0.686488
GPN1	1.050776	0.93924	1.175557	0.386986
PLN	1.026747	0.96327	1.094406	0.417555
ZNF28	1.061555	0.981725	1.147876	0.134247
ZNF511	0.984578	0.867426	1.117552	0.809973
ZNF627	1.112873	1.029453	1.203054	0.007143
KCNRG	1.165335	1.034766	1.31238	0.011615
WDHD1	1.101646	1.024301	1.184831	0.009149
ZNF789	1.020088	0.954215	1.090509	0.559261
CTNND1	1.014017	0.949338	1.083103	0.67892
SLC34A3	1.14195	1.023452	1.274169	0.017564
SH2D1B	1.118969	1.027542	1.218532	0.009746

ARC	0.797337	0.755915	0.841029	8.76E-17
NUDT16	1.013994	0.944925	1.088111	0.699432
TLK1	0.952051	0.883809	1.025563	0.195386
LRBA	1.042522	0.987775	1.100304	0.130271
MMP17	0.939508	0.88451	0.997926	0.042621
BAZ1A	1.040683	0.963196	1.124404	0.312445
AKR1C4	1.087801	0.976485	1.211807	0.126528
COPS8	1.053999	0.951428	1.167629	0.314038
CCDC69	0.960059	0.888024	1.037938	0.305707
MDM4	0.966127	0.90674	1.029404	0.287044
KLHL9	1.031488	0.951669	1.118001	0.450582
NCOA6	0.955619	0.860112	1.061732	0.398122
STK39	1.044276	0.973451	1.120254	0.226648
CALM1	1.083952	0.985393	1.19237	0.097435
TTC37	1.018284	0.951262	1.090029	0.601953
TUSC1	1.017784	0.960275	1.078737	0.552497
PAPSS2	0.95534	0.889273	1.026314	0.211455
SLC9A6	1.121701	1.02697	1.22517	0.010738
EIF1AY	1.018561	0.98349	1.054882	0.303608
IPO9	1.127935	1.039106	1.224359	0.004021
CEP290	1.089685	0.983119	1.207802	0.101897
DLL1	1.057078	1.001177	1.1161	0.045241
ANKRD13I	0.931675	0.865403	1.003022	0.060132
UNC13B	1.2033	1.127346	1.284371	2.65E-08
LDB1	0.960171	0.892158	1.03337	0.278243
PPP1R14C	1.159392	1.052113	1.27761	0.002832
CTR9	1.119877	1.034078	1.212796	0.00537
F5	0.951289	0.897477	1.008327	0.092794
ZNF652	1.098993	1.022169	1.181592	0.01068
SMURF1	0.953143	0.898771	1.010805	0.109298
SLC5A3	0.946516	0.899345	0.996162	0.035082
GPATCH3	0.961328	0.88475	1.044535	0.351751
CDC42BPE	0.999318	0.947248	1.05425	0.980054
PLXNB3	1.036466	0.955827	1.123909	0.386096
OXCT2	1.474883	1.312671	1.657141	6.30E-11
RPL10A	0.889851	0.820477	0.965091	0.004833
RCSD1	0.966436	0.909983	1.026391	0.266255
FAM169A	1.278123	1.198374	1.363179	8.32E-14
ZNF830	1.113837	1.012575	1.225226	0.026627
GRIN3A	1.118909	1.01536	1.233018	0.023353
CNOT7	1.043796	0.949524	1.147428	0.374799
TMEM184I	0.931485	0.857839	1.011453	0.091224
SCAMP5	1.218487	1.140247	1.302095	5.35E-09
ZNF521	1.031766	1.00467	1.059593	0.021272
LRIG2	0.986194	0.912938	1.065328	0.724076
GK	0.980793	0.915781	1.050419	0.579411
FOXJ3	0.925275	0.854109	1.002371	0.057175
ZNF358	1.129042	1.052434	1.211227	0.00071
SFT2D1	1.120269	1.009204	1.243557	0.033011
CD247	1.013594	0.962582	1.067311	0.608305
INPP5F	1.097178	1.011093	1.190592	0.026109
ARHGAP1:	1.090308	1.020475	1.164919	0.010464
SUCNR1	0.899297	0.864361	0.935645	1.52E-07
HMGN2	1.185384	1.076461	1.305329	0.000544
UBE2J1	0.914677	0.84056	0.99533	0.03859
GJC2	0.936346	0.872708	1.004624	0.067026
OPA1	0.973465	0.90755	1.044166	0.452168
DENND4B	1.03899	0.954584	1.130859	0.376273

RYR3	1.027699	0.972006	1.086583	0.336474
ZNF277	0.843706	0.767111	0.927949	0.000465
KTI12	1.08122	0.979907	1.193008	0.119798
TOX	0.98609	0.934249	1.040807	0.611185
CES1	1.03927	1.010047	1.069338	0.008122
CD3E	0.982664	0.932501	1.035525	0.513004
RUSC2	1.028051	0.979094	1.079455	0.266451
FICD	1.024782	0.959559	1.094437	0.465632
OSTC	0.936169	0.854382	1.025785	0.15732
TSEN15	1.27983	1.151976	1.421873	4.33E-06
RUNDC1	0.995209	0.918534	1.078285	0.906542
CCDC152	1.266375	1.145406	1.40012	4.02E-06
GRK5	0.929507	0.869036	0.994187	0.033187
TYW1	0.937501	0.864469	1.016703	0.118841
SFMBT2	0.933036	0.881198	0.987924	0.017476
PNMA5	1.033513	0.952988	1.120842	0.425757
ITPRIPL1	1.133641	1.043734	1.231291	0.002927
SMC5	0.918111	0.851276	0.990194	0.026726
PRMT6	1.24478	1.149607	1.347832	6.83E-08
SHISA4	1.146918	1.083222	1.214361	2.58E-06
CAPZA2	1.055603	0.958923	1.162031	0.269543
TOP1	0.953153	0.887901	1.023201	0.184821
PRC1	1.034732	0.952107	1.124526	0.421336
MAP3K3	0.924528	0.857566	0.996719	0.040791
L1CAM	0.838198	0.723893	0.970551	0.018296
SREBF2	1.011674	0.937024	1.092271	0.76664
RASGEF1A	1.086897	0.999104	1.182405	0.052488
RPL39	0.875023	0.81674	0.937466	0.000147
DZIP3	1.048492	0.968475	1.13512	0.242362
KIAA0753	0.981822	0.902099	1.06859	0.671138
DCLRE1A	1.115795	1.029908	1.208845	0.007339
ATG9A	0.96382	0.904074	1.027515	0.259054
NOS1AP	1.21974	1.141514	1.303326	4.26E-09
APRT	1.054334	0.959313	1.158767	0.272216
GPRASP1	1.001443	0.935893	1.071584	0.966696
TBKBP1	0.958454	0.89827	1.02267	0.199676
MAGEE1	1.133877	1.031029	1.246984	0.009602
ZFP2	1.024723	0.912139	1.151202	0.680873
L3MBTL3	1.122774	1.048449	1.202367	0.00092
MFAP3L	1.13906	1.047573	1.238536	0.002304
NAGA	1.082424	1.024562	1.143554	0.004719
SMG5	0.994526	0.921763	1.073033	0.887394
TGM2	1.144737	1.09197	1.200054	1.98E-08
ARMCX6	1.081499	0.973432	1.201563	0.144664
PJA2	0.900481	0.841826	0.963224	0.002286
SGMS1	1.061107	0.989696	1.137671	0.095201
OR10Z1	1.071754	0.996209	1.153029	0.063153
MIR30E	0.99633	0.934481	1.062273	0.910479
MIR429	0.943977	0.862691	1.032923	0.209516
MIR345	0.960458	0.907969	1.015981	0.159415
MIR16-2	1.033755	0.98749	1.082188	0.155296
MIR340	0.960459	0.918877	1.003924	0.074011
MIR339	1.152647	1.092583	1.216013	1.96E-07
MIRLET7C	1.020014	0.926218	1.123307	0.68722
MIR425	0.987441	0.951899	1.02431	0.499203
MIR210	0.996479	0.943002	1.052987	0.900248
MIR324	1.029537	0.974591	1.087582	0.298233
MIR101-2	1.134625	1.019733	1.262463	0.020412

MIR26A1	1.141331	1.053756	1.236184	0.001173
MIR342	1.1482	1.077316	1.223748	2.13E-05
MIR326	1.0269	0.979113	1.07702	0.274931
MIR302C	0.998096	0.931918	1.068973	0.956574
MIR26B	1.040515	0.983949	1.100333	0.163741
MIR148B	1.032292	0.967461	1.101467	0.336876
MIR101-1	1.078218	1.021575	1.138001	0.006233
MIR373	1.101387	1.035649	1.171298	0.002101
MIR302D	0.974995	0.907894	1.047054	0.486381
MIRLET7G	1.00788	0.963493	1.054313	0.732664
MIR30D	1.013878	0.948108	1.084209	0.687125
MIR126	0.949059	0.902109	0.998452	0.043403
MIR374A	0.987463	0.934728	1.043173	0.652313
MIR367	1.013426	0.935757	1.097542	0.743044
MIR331	1.00628	0.952259	1.063366	0.824016
MIRLET7I	1.040791	0.978816	1.106691	0.201815
SNORD114	0.958028	0.878238	1.045068	0.333836
SNORD9	1.034899	0.975316	1.098121	0.25686
SNORA63	1.006373	0.920036	1.100812	0.889595
SNORA31	0.994285	0.933636	1.058874	0.858341
SNORD104	1.082521	1.014799	1.154763	0.016143
SNORD1B	1.078549	1.020804	1.13956	0.007073
VTRNA1-1	1.073507	1.01897	1.130964	0.007667
SNORA73I	1.022803	0.996601	1.049693	0.088599
SNORA70	1.080637	0.968548	1.205698	0.165137
SNORA72	1.023184	0.957727	1.093115	0.496839
SNORA38I	1.047455	0.988156	1.110314	0.118938
SNORA33	1.003319	0.9433	1.067157	0.91614
RNU4-1	1.03142	0.997541	1.06645	0.069449
SNORA38	1.015293	0.972829	1.05961	0.486271
SNORD46	1.05408	1.003558	1.107146	0.03558
SNORA74/	0.977323	0.933992	1.022666	0.321525
RNY1	1.102334	1.037585	1.171123	0.001607
SNORA62	1.053618	0.956002	1.1612	0.292383
SNORA65	1.064078	1.012378	1.118419	0.014524
RNU105B	0.950812	0.902688	1.001501	0.056991
SNORA16I	1.008725	0.956871	1.06339	0.746971
SNORA14/	1.085399	1.011753	1.164406	0.02226
SNORD11:	0.891518	0.815621	0.974478	0.011424
SNORA5C	0.997421	0.966376	1.029464	0.872834
SNORD11	0.962877	0.920091	1.007653	0.102844
SNORD48	1.013442	0.933279	1.100491	0.750796
SNORD114	0.953541	0.869078	1.046214	0.314759
SNORA23	1.015974	0.988221	1.044505	0.262084
VTRNA1-2	0.971615	0.901667	1.046989	0.450013
SNORD11:	0.860546	0.764603	0.968527	0.012767
SNORD6	0.946496	0.899643	0.99579	0.033768
RNY3	1.128222	1.065764	1.19434	3.30E-05
RNU4-2	1.028485	0.993294	1.064924	0.113838
MIR146B	0.938837	0.876293	1.005846	0.072772
MIR582	1.203827	1.106523	1.309689	1.60E-05
ZNF525	1.105955	1.023955	1.194522	0.0104
INF2	1.063795	0.992502	1.140208	0.080584
EFCAB2	0.974249	0.902016	1.052266	0.506845
CHML	1.049381	0.979046	1.124767	0.17329
TATDN3	1.064686	0.956305	1.185349	0.252498
CR1	0.921581	0.877779	0.967568	0.001013
ECT2L	1.006467	0.927838	1.09176	0.876568

GPR52	1.032344	0.980261	1.087195	0.22814
FCGR3A	1.001246	0.958159	1.04627	0.955763
OR6K3	1.081742	0.927117	1.262154	0.318091
MSTO2P	1.062374	0.984624	1.146263	0.118673
SPRN	1.129171	1.000342	1.274591	0.049357
FANK1	0.847645	0.748635	0.95975	0.009102
DDO	0.937006	0.886988	0.989845	0.020092
SNHG5	0.827684	0.763337	0.897456	4.65E-06
GDI1	1.059874	0.952452	1.179411	0.286197
PCMTD2	1.106485	1.022923	1.196875	0.011549
SOX18	0.931564	0.845112	1.026861	0.153709
LIME1	1.119062	1.005298	1.245699	0.039727
SAMD13	1.090766	0.985551	1.207213	0.093204
EFCAB7	1.092998	0.979658	1.219451	0.111378
LCN8	0.741903	0.644339	0.85424	3.32E-05
LIPN	0.873202	0.800014	0.953086	0.002399
FOXO6	1.044892	0.947831	1.151893	0.377331
SYS1	1.065585	0.97546	1.164036	0.158866
INPP5B	1.042316	0.968822	1.121385	0.266597
RPA4	0.974162	0.902637	1.051354	0.501056
NEU4	0.981184	0.945282	1.018449	0.317914
MAFB	1.045022	1.009616	1.081669	0.012274
TRAF3IP1	1.194051	1.073775	1.327799	0.00106
BMP2KL	0.993301	0.928879	1.062191	0.844244
CHIC1	1.318907	1.224454	1.420646	2.86E-13
GIGYF2	1.007704	0.934143	1.087058	0.8427
RUFY2	0.987966	0.913644	1.068333	0.761568
NHSL2	1.139496	1.082975	1.198968	4.88E-07
GGTA1	0.859367	0.800329	0.922759	3.00E-05
PHACTR4	1.001192	0.920137	1.089387	0.977942
AGAP6	0.956368	0.864806	1.057624	0.384925
TIMM23B	1.00168	0.912084	1.100078	0.971988
ZDHHC18	0.987114	0.925193	1.053179	0.694762
ZDBF2	1.054551	0.985503	1.128436	0.124217
GGNBP1	1.101839	1.00303	1.210381	0.043066
DAXX	1.135151	1.02161	1.261311	0.018394
BMPR2	1.104685	1.047177	1.165351	0.000262
TCEA3	0.998535	0.880634	1.132222	0.981759
PFDN6	1.054296	0.941843	1.180175	0.358209
RING1	1.076663	0.968342	1.1971	0.172147
HSD17B8	1.046655	0.954348	1.147891	0.333036
RXRB	1.083166	0.997541	1.176141	0.057253
COL11A2	1.220145	1.096427	1.357823	0.000265
HLA-DOA	0.955021	0.913967	0.99792	0.040088
BRD2	0.918046	0.843706	0.998936	0.04718
HLA-DMA	0.971862	0.922598	1.023758	0.282233
COL5A2	1.135133	0.988922	1.302961	0.071606
PSMB8	1.2481	1.129151	1.379581	1.44E-05
TAP2	1.102881	1.020526	1.191882	0.013394
SPIN3	1.09194	0.958525	1.243924	0.185878
HLA-DRA	0.984283	0.946711	1.023346	0.424991
NOTCH4	0.991227	0.905824	1.084682	0.847992
PBX2	0.912933	0.84943	0.981183	0.013273
AGER	1.02414	0.946418	1.108244	0.553612
RNF5	1.075647	0.974309	1.187525	0.148616
AGPAT1	1.084031	0.990182	1.186776	0.08074
PRRT1	1.12815	0.949262	1.34075	0.171048
FKBPL	1.012067	0.93337	1.097398	0.771497

MRPL38	1.226932	1.106828	1.360069	9.98E-05
STK19	0.969514	0.857031	1.096759	0.622671
SKIV2L	1.072273	0.988376	1.163292	0.093212
ZBTB12	0.997587	0.93127	1.068626	0.94512
SDHD	1.060379	0.967189	1.162547	0.211613
EHMT2	1.02482	0.942805	1.113968	0.564563
LAYN	1.099835	0.999586	1.210139	0.051002
SLC44A4	1.049222	0.965746	1.139913	0.25598
NEU1	0.932275	0.857226	1.013895	0.101481
HSPA1B	1.022529	0.986072	1.060334	0.229067
HSPA1A	1.021715	0.982277	1.062736	0.284794
HSPA1L	1.025406	0.940277	1.118242	0.570467
LSM2	0.990013	0.906532	1.081181	0.823288
CARD16	1.056429	0.978703	1.140328	0.159171
MBD5	1.04488	0.957866	1.139798	0.322363
MSH5	1.116255	1.019204	1.222547	0.017795
LY6G6C	0.950734	0.903838	1.000063	0.050285
LY6G6F	1.109567	0.995736	1.23641	0.059756
LY6G5C	1.138312	1.036562	1.250051	0.006696
CSNK2B	1.153144	1.003924	1.324543	0.043868
APOM	0.988711	0.898876	1.087524	0.815294
AIF1	1.090472	1.026049	1.158941	0.00531
NCR3	1.230899	1.138507	1.330789	1.81E-07
LST1	1.112256	1.058414	1.168837	2.64E-05
NFKBIL1	1.067076	0.949812	1.198817	0.274375
MCCD1	1.025545	0.957353	1.098595	0.472443
MICB	0.943716	0.859684	1.035961	0.223422
ZNF551	0.997928	0.935926	1.064037	0.949459
MICA	0.972791	0.887023	1.066851	0.558007
ZNF805	1.030348	0.95574	1.110781	0.435649
HLA-C	0.939034	0.866451	1.017697	0.12538
PSORS1C3	1.131406	1.019669	1.255387	0.019959
POU5F1	1.479615	1.284769	1.704012	5.38E-08
CCHCR1	1.105114	1.011395	1.207518	0.027067
DHX16	1.004799	0.913819	1.104836	0.92125
MRPS18B	1.076012	0.981047	1.18017	0.120169
PPP1R10	0.993905	0.936952	1.054319	0.839084
ABCF1	1.005327	0.915363	1.104133	0.911553
PRR3	1.125888	1.012965	1.251401	0.027889
LILRB3	1.026396	0.973754	1.081884	0.332104
DDR1	0.891275	0.810991	0.979506	0.016853
GNL1	0.902411	0.837911	0.971875	0.006648
HLA-E	1.023396	0.936807	1.117988	0.608147
TRIM39	1.024861	0.935215	1.123099	0.599022
ZNF468	1.022773	0.945357	1.106528	0.574994
TRIM15	1.114189	1.030426	1.204761	0.006696
ZNF616	1.090733	1.0041	1.184841	0.039699
TRIM10	1.010907	0.971663	1.051737	0.591264
RNF39	1.003289	0.926065	1.086953	0.935957
PPP1R11	1.092503	0.980717	1.217031	0.108184
HCG9	1.088875	0.987572	1.200569	0.08746
HLA-G	0.985829	0.87919	1.105403	0.806964
TBC1D8	1.040124	0.983323	1.100206	0.169747
HLA-F	1.119763	1.053986	1.189645	0.00025
ZFP57	1.011219	0.962303	1.062621	0.659206
CBY3	1.103395	0.986946	1.233584	0.083801
IL31	0.971103	0.887474	1.062613	0.523348
AKT1S1	0.900343	0.8023	1.010366	0.074318

GABBR1	1.140023	1.093589	1.188429	6.54E-10
SPDYC	1.061421	0.976763	1.153418	0.159853
TRIM27	1.041872	0.961786	1.128627	0.314811
RANBP17	1.06999	0.993021	1.152925	0.075718
TTC25	0.956808	0.908976	1.007156	0.09152
ATXN2	0.976051	0.893635	1.066067	0.590182
DCTN1	1.020391	0.935817	1.112609	0.647477
TCTN1	1.054523	0.948793	1.172035	0.324699
ZBTB48	1.048553	0.963789	1.140773	0.270297
NAT8B	0.871557	0.782784	0.970397	0.012134
ZNF155	1.078766	0.978138	1.189745	0.129131
FBXO48	1.004439	0.901539	1.119083	0.935987
CD177	1.028069	0.994051	1.063252	0.106869
ZNF783	1.039716	0.962102	1.123591	0.325151
ZNF425	0.976151	0.859819	1.108221	0.709273
TRIM13	0.956561	0.868459	1.0536	0.367669
SPIRE2	1.081868	0.943284	1.240813	0.260537
PKHD1L1	1.208932	1.090115	1.340699	0.000325
SLFN12L	1.150476	1.026984	1.288818	0.01554
SLC35B4	1.155219	1.074301	1.242232	9.85E-05
SYCE1L	0.96155	0.876256	1.055146	0.408057
TMEM231	1.446552	1.29599	1.614605	4.59E-11
FAM71F2	1.041887	0.977619	1.110381	0.206532
CCNI2	1.171653	0.983194	1.396235	0.076638
CDKL4	1.039486	0.944732	1.143744	0.427127
SDHAF1	1.090211	0.995833	1.193533	0.061543
PSENEN	1.018642	0.930308	1.115364	0.689829
ZBTB10	0.89616	0.847828	0.947247	0.000106
CCDC144N	1.120501	1.07301	1.170093	2.62E-07
LGR4	1.092266	1.038576	1.148732	0.0006
PSMB10	1.085828	0.978834	1.204518	0.119765
E2F4	1.150065	1.033296	1.280029	0.01048
PDE7A	1.085252	1.021959	1.152464	0.00762
TMEM170I	0.996677	0.943028	1.053378	0.906146
SNX2	0.97902	0.886152	1.08162	0.676686
NT5M	1.047587	0.978244	1.121846	0.183367
IPO7	0.978595	0.912373	1.049624	0.545025
PRR13	1.132315	1.007856	1.272142	0.036466
TECPR1	1.050773	0.969603	1.138738	0.227276
MT1H	1.080336	1.00468	1.16169	0.036979
MT1M	1.161317	1.057558	1.275255	0.001737
SAMD9	1.072553	1.009656	1.139367	0.023108
ATP6AP1L	1.087462	0.966907	1.223047	0.16193
C2CD4B	0.963182	0.913265	1.015828	0.167097
NAP1L4	0.996305	0.932367	1.064628	0.912903
TMSB4X	0.853959	0.786219	0.927536	0.000181
CPT1B	1.332727	1.16786	1.520869	2.02E-05
SMN2	0.949729	0.867865	1.039316	0.26208
SERF1B	0.952919	0.834948	1.087558	0.47449
HMGN1	0.956791	0.867918	1.054764	0.374522
STAG3L1	0.921451	0.840448	1.010261	0.081418
MUC19	1.056096	0.948318	1.176124	0.320337
EIF3CL	0.940346	0.859276	1.029064	0.181176
LCMT1	0.934246	0.839023	1.040277	0.214958
MFSD2B	0.984538	0.932693	1.039264	0.572345
LIN52	1.103303	1.013882	1.200611	0.022628
DPF3	1.220792	1.1297	1.319229	4.60E-07
ITSN1	0.972989	0.886976	1.067343	0.562009

ITPRIPL2	0.981126	0.926613	1.038847	0.513576
DENND1C	0.978155	0.892859	1.0716	0.635177
CRLF2	1.01328	0.96869	1.059921	0.565594
CRYZL1	0.977162	0.889873	1.073014	0.628458
RP9P	1.017229	0.938227	1.102883	0.678775
CATSPER2	0.949502	0.896074	1.006115	0.079492
ARRDC5	0.997724	0.931112	1.069101	0.94846
LOH12CR2	1.134065	0.979965	1.312396	0.091341
KLRC2	1.077216	0.99351	1.167975	0.071513
KLRC3	1.054733	0.956256	1.163351	0.286628
RFPL3S	0.987522	0.918418	1.061826	0.734439
OLIG2	0.933316	0.858355	1.014825	0.106208
RNPS1	0.950987	0.856908	1.055394	0.34437
HSP90AB2	1.102379	0.981207	1.238516	0.100875
NYNRIN	1.20096	1.146792	1.257686	7.45E-15
DNAJC19	1.045844	0.929028	1.177347	0.458237
IFITM5	1.116579	1.050598	1.186703	0.000388
DEFA1	1.072222	1.00436	1.144669	0.036583
DOK6	1.170422	1.094149	1.252011	4.72E-06
ZDHHC11I	0.928096	0.852258	1.010683	0.086228
TMEM191(	1.487779	1.203784	1.838775	0.000237
HBA1	0.982538	0.957262	1.008482	0.185244
HBM	0.986935	0.958423	1.016295	0.379247
ATP10A	1.066067	1.010545	1.124638	0.019059
HCP5	1.073191	1.01539	1.134281	0.012396
HLA-H	1.027926	0.952365	1.109481	0.479539
HCG27	1.006066	0.945706	1.070278	0.84807
COL6A6	1.125932	1.076664	1.177455	2.04E-07
RAB12	0.997981	0.931901	1.068747	0.953889
HLA-A	1.007085	0.923393	1.098363	0.873274
LNP1	0.964243	0.871364	1.067023	0.481052
VGLL3	1.115044	0.983456	1.264238	0.089207
TRIM71	0.871095	0.834054	0.909781	4.82E-10
ANKRD28	0.968722	0.911308	1.029754	0.308014
COLQ	1.171784	1.07424	1.278186	0.00035
METTL6	1.248597	1.138074	1.369853	2.67E-06
SNORA2A	0.991063	0.94332	1.041223	0.721572
SNORA69	1.034909	0.952832	1.124056	0.415701
RNU6-1	1.076222	1.013441	1.142893	0.016606
SNORA22	1.073251	1.008649	1.141991	0.025626
SNORD10:	0.970535	0.933508	1.009031	0.131825
SNORA30	1.012186	0.966058	1.060518	0.610774
SNORD37	0.987858	0.941585	1.036404	0.6177
SNORA5A	1.003052	0.965199	1.04239	0.876605
SNORA75	1.094635	1.015496	1.179942	0.018197
SNORA70I	0.988351	0.936959	1.042561	0.667129
SNORD15/	1.083713	0.995453	1.179798	0.063621
SNORD63	1.009172	0.918656	1.108606	0.848976
SNORA54	0.987297	0.948818	1.027337	0.52851
SNORA36(	1.018701	0.94994	1.092438	0.603319
SNORD59/	0.982894	0.933425	1.034985	0.51257
SNORD51	1.105726	1.011355	1.208902	0.027242
SNORA7B	1.062002	1.003667	1.123728	0.036893
SNORA15	1.04283	0.945562	1.150105	0.401195
SNORA14I	1.010457	0.967628	1.055181	0.637812
SNORA37	1.024415	0.974548	1.076834	0.343438
SNORA2B	1.001072	0.946841	1.058409	0.969918
SNORA20	1.115723	1.039423	1.197623	0.002447

SNORD56I	0.983483	0.946729	1.021664	0.391412
SNORD15I	1.013179	0.964741	1.06405	0.600387
SNORA19	0.996302	0.945868	1.049424	0.888822
SNORA46	1.014761	0.967167	1.064698	0.549933
RNU1-3	0.935354	0.876165	0.998542	0.0451
SNORA66	1.013213	0.948819	1.081977	0.695198
MIR25	1.065813	1.00441	1.13097	0.035264
MIR647	0.94273	0.881489	1.008226	0.085266
MIR635	1.013379	0.967437	1.061502	0.5745
MIR23B	1.057732	0.980675	1.140843	0.145857
MIR199B	0.969088	0.912696	1.028963	0.304639
MIR30B	1.085865	1.006996	1.170911	0.03226
MIR606	0.97642	0.941021	1.01315	0.205319
MIR593	0.987423	0.94567	1.031019	0.565844
MIR215	1.057631	1.000494	1.118032	0.047998
MIR181A2	1.0373	0.988317	1.088711	0.137863
MIR191	1.002574	0.961718	1.045166	0.903608
MIR554	1.043917	1.000692	1.089009	0.046366
MIR200A	1.001361	0.914048	1.097014	0.976692
MIR491	0.984211	0.935924	1.03499	0.535231
MIR149	1.063957	0.977797	1.157708	0.150195
MIR604	1.015729	0.976172	1.056889	0.441276
MIR181C	0.951908	0.855047	1.059741	0.368017
MIR193A	1.088753	0.99597	1.19018	0.06133
MIR194-1	1.058929	1.001766	1.119354	0.043149
MIR128-2	0.922182	0.831577	1.02266	0.124707
MIR562	1.043845	0.987673	1.103212	0.128394
MIR581	1.023532	0.971706	1.078123	0.380306
MIR651	0.906956	0.823489	0.998882	0.047403
MIR641	0.996959	0.956333	1.039311	0.885912
MIR505	0.996697	0.954946	1.040274	0.879556
MIR99A	0.97625	0.863886	1.10323	0.700037
MIR571	0.997712	0.956197	1.041029	0.915864
MIR570	1.010068	0.952387	1.071243	0.738446
MIR558	0.942639	0.900092	0.987197	0.012184
MIR128-1	1.013576	0.954564	1.076237	0.659497
MIR602	0.974417	0.90521	1.048916	0.490539
MIR184	1.220762	1.08386	1.374956	0.001013
MIR659	1.100746	1.027457	1.179262	0.006324
MIR573	0.996815	0.951555	1.044229	0.892981
MIR32	0.975263	0.931275	1.021329	0.287465
MIR597	0.995138	0.949171	1.04333	0.839913
MIR141	0.930237	0.834363	1.037127	0.192547
MIR627	0.987018	0.916393	1.063085	0.730118
MIR200C	1.026354	0.955634	1.102309	0.475143
MIR572	0.988446	0.901923	1.083269	0.803633
MIR623	0.98565	0.946093	1.026862	0.489196
MIR186	1.026256	0.976496	1.078552	0.306772
MIR222	0.911125	0.869125	0.955155	0.000111
MIR556	1.21193	1.141028	1.287238	4.12E-10
MIR200B	1.020739	0.933335	1.116329	0.653117
MIR657	1.084434	1.016103	1.157359	0.014645
MIR181B2	1.026505	0.979664	1.075587	0.272301
MIR590	0.973729	0.927146	1.022652	0.28714
MIR575	1.045014	0.97309	1.122254	0.226207
MIR553	1.020402	0.974396	1.06858	0.390871
MIR199A1	0.99743	0.957877	1.038617	0.900812
MIR580	1.079659	1.014398	1.149118	0.015981

MIR93	1.043896	0.992532	1.097918	0.09516
MIR181A1	0.945462	0.900069	0.993143	0.02548
MIR617	1.019428	0.965992	1.075821	0.483642
MIR626	0.966889	0.916087	1.020507	0.221414
MIR586	1.083282	1.021717	1.148556	0.00737
MIR551A	0.964539	0.905721	1.027176	0.260712
MIR15B	1.024354	0.979127	1.071669	0.296306
MIR648	0.962573	0.91893	1.008289	0.107118
MIR26A2	1.000583	0.94982	1.054058	0.98251
MIR646	1.015714	0.971837	1.061572	0.488929
MIR599	1.038554	0.980797	1.099712	0.19505
MIR563	1.046864	0.98184	1.116195	0.161571
MIR545	1.009548	0.958044	1.063822	0.722072
MIR640	0.99589	0.963488	1.029383	0.807225
MIR596	1.01729	0.931245	1.111285	0.703815
MIR33B	1.012159	0.960466	1.066633	0.65138
MIR27B	1.117033	1.042813	1.196535	0.001605
MIR221	0.911651	0.874932	0.949911	1.03E-05
MIR610	0.983405	0.902373	1.071714	0.7029
MIR302A	1.013051	0.944275	1.086836	0.717735
MIR603	1.192201	1.101609	1.290244	1.30E-05
MIR33A	0.991229	0.947277	1.03722	0.703405
MIR634	1.043296	0.973618	1.117961	0.229426
MIR574	0.962724	0.902111	1.027409	0.25222
MIR624	1.03043	0.987371	1.075367	0.168701
MIR579	0.97115	0.924594	1.020049	0.24282
MIR30C1	1.036597	0.972134	1.105334	0.272544
MIR569	1.000578	0.945679	1.058665	0.983977
MIR629	1.040387	1.001945	1.080305	0.039293
MIR620	0.999071	0.959217	1.040581	0.9643
MIR638	0.975757	0.918154	1.036974	0.429233
MIR589	0.982176	0.930653	1.036551	0.512991
MIR181B1	0.964254	0.927819	1.00212	0.063995
MIR607	0.947224	0.868908	1.032599	0.218168
MIR613	1.009409	0.947872	1.07494	0.770437
MIR576	0.960194	0.922268	0.999681	0.048213
MIR182	1.042601	0.947512	1.147233	0.392556
MIR601	1.008257	0.964801	1.05367	0.714494
MIR301A	1.180677	1.101901	1.265086	2.43E-06
MIR643	0.989767	0.942498	1.039407	0.680373
MIR16-1	1.084218	1.013402	1.159982	0.018963
MIR130A	1.023689	0.968978	1.081489	0.40346
MIRLET7F2	1.038906	0.968483	1.11445	0.286534
MIR652	1.029167	0.979834	1.080983	0.251336
MIR140	1.044864	0.979508	1.114582	0.182958
MIR645	0.998456	0.957998	1.040623	0.941645
MIR618	1.030853	0.988428	1.0751	0.15644
MIR185	1.055366	1.003999	1.109362	0.034284
MIR591	1.003099	0.956983	1.051437	0.897487
MIR616	0.941572	0.898436	0.986778	0.011861
MIR548A3	0.96233	0.91279	1.014559	0.154464
MIR106B	1.031377	0.982169	1.083051	0.215482
MIR320A	0.977727	0.934513	1.02294	0.328772
SNORD94	0.983577	0.932654	1.037279	0.541505
SNORD96I	1.071308	1.017278	1.128207	0.009087
SNORA49	1.024076	0.980056	1.070074	0.288555
SNORD12(	1.004189	0.965604	1.044315	0.83438
SNORD83/	0.975292	0.931247	1.02142	0.288652

SNORD10!	1.13823	1.064552	1.217007	0.000149
SNORD41	1.023575	0.952848	1.099552	0.523576
MIR196A1	1.085792	0.998047	1.18125	0.055556
GPX3	0.953672	0.897428	1.013441	0.126145
GNRHR2	1.044402	0.94335	1.156278	0.402736
DIO1	1.039402	0.94897	1.138452	0.405331
AKR7L	1.116667	1.030497	1.210043	0.007078
STK38L	0.903848	0.834141	0.979381	0.013558
SACM1L	1.058445	0.986712	1.135392	0.112657
TSN	1.081282	0.984538	1.187532	0.102232
MIR454	1.190721	1.10032	1.288549	1.47E-05
MIR760	1.027483	0.971434	1.086766	0.343478
MIR766	0.937263	0.894551	0.982014	0.006476
MIR769	1.088511	1.005805	1.178017	0.03542
MIR765	1.094187	1.025539	1.167432	0.006473
SLC48A1	1.1702	1.07874	1.269414	0.000153
MIR301B	1.121381	1.038004	1.211455	0.003659
PRR22	1.124371	0.998335	1.266319	0.0533
TAS2R19	1.000681	0.947516	1.056828	0.980515
TAS2R50	0.999157	0.929801	1.073686	0.981659
TAS2R14	1.031345	0.958062	1.110234	0.411808
TAS2R13	1.030015	0.950026	1.116738	0.473367
SNORD67	1.030838	0.976822	1.087842	0.268723
SNORD66	1.032538	0.972363	1.096437	0.295947
SNORD91/	1.159254	1.08551	1.238009	1.05E-05
SNORD17	1.013278	0.98159	1.045989	0.415809
SNORD89	1.043393	0.973822	1.117936	0.227619
SNORD72	1.028157	0.974195	1.085108	0.312736
SNORA74I	1.025179	0.997626	1.053493	0.073615
SNORA53	1.012369	0.989194	1.036087	0.29814
SNORD90	1.034108	0.964618	1.108604	0.344662
SNORD69	1.113328	1.045676	1.185356	0.00079
SNORA12	1.042084	1.010903	1.074227	0.007824
SNORD19	1.024877	0.978467	1.073488	0.298675
SNORD70	1.014423	0.974729	1.055734	0.481963
SNORD30	1.049258	0.965696	1.140051	0.25613
RNF208	1.078769	0.989517	1.176071	0.085289
PTTG3P	0.958444	0.902961	1.017335	0.162994
ZNF580	0.915928	0.832801	1.007352	0.070442
ZNF611	1.11602	1.027171	1.212555	0.009505
NUP62	1.20933	1.105186	1.323287	3.52E-05
DENND1B	0.96003	0.900464	1.023538	0.211994
SFT2D2	0.971066	0.910268	1.035925	0.373446
LPAL2	1.120247	1.03431	1.213324	0.005297
ZNF254	0.968566	0.906829	1.034505	0.341878
TCTEX1D2	1.223607	1.080332	1.385883	0.001493
CRYGS	1.173513	1.06276	1.295807	0.001559
CRIP1	1.131049	1.059879	1.206997	0.000204
KLHL23	1.010284	0.945424	1.079593	0.762485
FAM24B	0.95949	0.896883	1.026466	0.229673
TRIM59	1.148668	1.051946	1.254284	0.002013
MLLT11	0.991941	0.923589	1.065352	0.824213
GIMAP1	1.091203	0.994978	1.196735	0.063873
DNLZ	0.970405	0.881448	1.068339	0.540268
SUPT4H1	0.933206	0.821718	1.059821	0.286905
NRAS	0.944634	0.874397	1.020513	0.148497
LTC4S	0.99379	0.945629	1.044405	0.805862
ANKRD39	1.284156	1.137776	1.449369	5.12E-05

QTRT1	1.253232	1.137817	1.380355	4.67E-06
CHUK	0.986927	0.909817	1.070573	0.751228
MXD3	1.160864	1.064715	1.265695	0.000721
GSTM2	1.079441	1.009374	1.154372	0.025586
COG8	1.027987	0.939885	1.124347	0.545986
ARHGAP19	1.048525	0.969036	1.134535	0.238792
LCAT	1.122716	1.043972	1.2074	0.00181
PVRIG	0.981706	0.869173	1.108808	0.766287
GPC2	1.03373	0.968027	1.103893	0.322123
SIPA1	1.097663	1.022137	1.178769	0.010409
SYNJ2BP	1.102144	1.023129	1.187261	0.010395
ARL2	1.122434	1.046279	1.204133	0.001273
SRA1	1.003489	0.899896	1.119008	0.95004
DNAJC9	1.065396	0.967379	1.173343	0.198286
VDAC1	1.015517	0.929959	1.108947	0.731671
ZBTB9	1.074096	0.988922	1.166605	0.089944
HEXA	0.97726	0.902621	1.058072	0.570411
NDUFS3	1.149798	1.020785	1.295116	0.021519
LEPROT	1.034321	0.954046	1.12135	0.412972
LBH	0.973723	0.917936	1.0329	0.376363
ADAT3	1.00672	0.910255	1.113409	0.89631
PPP1CB	1.002424	0.928753	1.08194	0.950425
GPSM3	0.925626	0.840935	1.018847	0.114432
LAT	1.099955	0.999938	1.209976	0.05015
NCKIPSD	1.007643	0.932038	1.089381	0.848258
ATF6B	1.029859	0.925243	1.146305	0.59035
S1PR3	0.981637	0.913094	1.055325	0.61577
CLIC1	0.938907	0.852512	1.034056	0.200551
DDAH2	0.906422	0.843824	0.973664	0.007125
RPS29	0.952414	0.896694	1.011596	0.112936
UGT2B11	0.777755	0.734611	0.823434	6.04E-18
ATP6V1G2	1.179739	1.028684	1.352976	0.018054
ZNF134	0.954348	0.888729	1.024811	0.198569
DDX47	1.014264	0.940403	1.093927	0.713506
ZNF845	1.140466	1.049973	1.238758	0.001833
KLRK1	1.046281	0.937054	1.16824	0.421254
EMP2	1.229958	1.114186	1.35776	4.07E-05
KCTD11	1.053824	0.963937	1.152092	0.249111
LTB4R	1.172613	1.088916	1.262744	2.50E-05
LTB4R2	1.149598	1.063955	1.242135	0.000416
DNASE1	1.039875	0.977526	1.106201	0.215179
CSNK1E	0.898778	0.833203	0.969514	0.005764
IRF9	1.018262	0.960898	1.079051	0.54072
GALT	1.209788	1.101396	1.328848	6.99E-05
HBG1	1.049831	0.998901	1.103358	0.055285
CLDN9	0.978609	0.908675	1.053925	0.567594
KRT18P17	0.768823	0.599989	0.985167	0.037701
NUDT19	0.996001	0.925225	1.072191	0.915153
TAX1BP3	1.044088	0.927563	1.175251	0.474878
AP1G2	1.12216	1.031695	1.220558	0.007198
ZNF90	0.976418	0.888029	1.073604	0.622049
MEF2B	1.069672	0.90635	1.262426	0.425593
GANC	1.007287	0.940945	1.078305	0.834558
TTLL3	1.073071	0.995313	1.156903	0.066128
REPIN1	0.970102	0.900226	1.045401	0.426124
MRPL23	0.967781	0.892662	1.049221	0.426944
UCA1	0.891817	0.781441	1.017784	0.089421
TSPAN4	0.888795	0.823227	0.959587	0.00257

CPNE1	1.022961	0.941946	1.110945	0.589704
ARL16	1.117637	0.997348	1.252435	0.055587
LYRM4	1.016861	0.912331	1.133367	0.762569
MYCBP	1.195058	1.09459	1.304747	6.97E-05
PRCD	0.776025	0.668489	0.900861	0.000863
ALG3	1.085683	1.003416	1.174695	0.040877
FIS1	1.044214	0.938483	1.161858	0.427011
MBLAC1	1.080468	0.971867	1.201204	0.152149
NEURL1B	1.014288	0.965685	1.065338	0.571207
PLIN5	1.093639	1.011601	1.182329	0.024456
HIGD1C	1.061982	0.939554	1.200363	0.335913
PPME1	1.153772	1.056346	1.260184	0.001484
STARD10	1.071227	0.994624	1.153731	0.069131
MEG3	0.964082	0.915805	1.014904	0.162846
IFRD2	1.098811	1.013294	1.191546	0.022642
CAPN14	1.077618	0.974471	1.191682	0.145338
ZBED1	1.018607	0.940418	1.103297	0.650955
HNRNPUL	1.080932	0.988932	1.18149	0.086393
POLR2J4	1.103291	0.990855	1.228485	0.073062
MTCP1	1.155255	0.981981	1.359102	0.081744
SMTNL1	1.006132	0.949124	1.066565	0.837241
ZSWIM7	0.981719	0.883923	1.090334	0.730381
LRRC69	1.097197	1.004969	1.19789	0.038396
PHB2	0.883134	0.797836	0.977551	0.016482
NEURL4	1.036377	0.968008	1.109574	0.304818
UBXN2B	1.119811	1.030811	1.216496	0.007403
CBWD6	0.896918	0.785569	1.02405	0.10771
FAM166B	0.926252	0.845772	1.014391	0.098559
PEX26	1.042825	0.967581	1.12392	0.27244
FASTKD5	1.001211	0.931431	1.076219	0.973801
GOLGA8B	1.099595	1.048073	1.153649	0.000105
HOMEZ	1.049378	0.962996	1.143508	0.271473
DDX3X	0.940247	0.875518	1.009761	0.090448
VPS16	1.183982	1.073333	1.306037	0.000742
MYL5	1.143361	1.043262	1.253064	0.004157
ZNF407	1.008292	0.948035	1.072379	0.792822
NPEPL1	1.101365	1.005329	1.206575	0.038067
SIAH3	1.036269	0.926273	1.159327	0.533762
GCGR	0.83531	0.732278	0.95284	0.00738
CELA2B	0.970522	0.89845	1.048377	0.447262
TMEM167I	0.949658	0.872081	1.034137	0.23485
FAM72D	1.269156	1.11213	1.448352	0.000405
TNFRSF25	0.979847	0.925136	1.037794	0.48738
ZC3H11B	0.823073	0.711705	0.951868	0.008665
TSTD1	0.937504	0.866494	1.014334	0.108314
PDZK1P1	1.230976	1.099698	1.377927	0.000304
CYB5RL	1.070738	0.983784	1.165378	0.113735
TTC34	1.305933	1.126823	1.513513	0.000391
MMP23A	0.879756	0.74705	1.036036	0.124632
ATAD3C	0.855533	0.727881	1.005573	0.058417
MIR942	1.058537	1.005838	1.113998	0.029009
MIR933	0.98023	0.943973	1.017879	0.299072
MIR874	1.092052	1.01502	1.174931	0.018304
MIR938	0.97194	0.930471	1.015257	0.20078
MIR875	1.029829	0.971646	1.091497	0.321886
MIR920	1.085876	1.021397	1.154426	0.008344
IFI30	1.011498	0.9653	1.059907	0.631715
CCDC7	1.092911	1.012066	1.180214	0.02346

FNIP1	0.960075	0.905274	1.018193	0.174236
CKLF	1.164491	1.073091	1.263677	0.000261
RPS10P3	0.921685	0.853061	0.995828	0.038841
ZNF579	0.876294	0.806535	0.952086	0.001808
RNASEK	1.048819	0.957266	1.149128	0.306403
NBPF1	1.11876	1.02901	1.216338	0.008533
LINGO3	0.967779	0.918731	1.019446	0.217132
VAMP2	0.875297	0.7996	0.958161	0.003901
MIR1250	1.048338	0.995618	1.103849	0.072952
MIR1272	1.044084	0.983736	1.108135	0.155562
MIR1286	0.935202	0.886584	0.986486	0.013913
MIR1296	1.000485	0.960275	1.042379	0.981501
SNORD11:	1.037379	0.982576	1.095239	0.185099
MIR1302-!	1.083825	1.03562	1.134274	0.000525
SNORA11I	1.024349	0.97029	1.081419	0.384485
MIR548E	0.99225	0.951057	1.035226	0.719112
MIR548L	0.997212	0.956267	1.03991	0.896169
MIR548P	0.98236	0.916828	1.052576	0.613377
MIR1284	0.996689	0.953823	1.041481	0.882436
MIR1255A	0.945147	0.906391	0.98556	0.00827
MIR1302-8	1.111062	1.019854	1.210428	0.015961
MIR548I2	0.995813	0.930927	1.065222	0.902867
MIR663B	0.985248	0.961412	1.009675	0.234275
SNORA79	1.030901	0.963806	1.102667	0.375446
MIR1200	0.987819	0.952498	1.02445	0.509452
MIR548Q	1.003569	0.954809	1.054818	0.888514
MIR548K	0.990345	0.951963	1.030276	0.630487
MIR1288	0.980888	0.9264	1.038582	0.508134
MIR320B2	1.02166	0.961344	1.08576	0.490078
MIR1301	0.995182	0.944698	1.048364	0.855726
MIR1202	0.991779	0.94824	1.037317	0.718544
MIR1277	1.021103	0.969388	1.075576	0.43098
MIR1271	1.088744	1.014911	1.167949	0.017642
MIR1827	1.102666	1.047952	1.160235	0.000167
MIR320C1	0.968585	0.919927	1.019816	0.22483
SNORD10(	1.009059	0.960528	1.060042	0.719905
MIR548O	0.988547	0.939236	1.040448	0.659071
SNORD11:	1.015673	0.967976	1.065721	0.526279
MIR548H1	1.011037	0.951835	1.073922	0.72143
SNORD99	1.000809	0.955113	1.048691	0.972945
MIR1180	0.977446	0.894986	1.067504	0.61194
MIR1255B:	0.974473	0.906496	1.047549	0.483383
MIR1249	1.040456	0.983729	1.100454	0.165611
MIR1276	0.953045	0.907014	1.001412	0.056899
SNORA77	1.176381	1.105593	1.251701	2.89E-07
MIR1290	1.004827	0.964484	1.046857	0.817847
MIR548N	1.004954	0.918149	1.099967	0.914613
RNU6ATA(	1.088486	1.029503	1.150849	0.002855
MIR1278	1.007582	0.965936	1.051025	0.725788
SNORA11	1.040079	0.979615	1.104274	0.198451
MIR1203	0.998905	0.948378	1.052123	0.966986
SNORD93	1.079165	1.00949	1.153649	0.025265
MIR548J	1.047555	0.999377	1.098055	0.053112
MIR1289-:	1.02221	0.954616	1.094589	0.529135
MIR1205	1.016486	0.969704	1.065526	0.496371
MIR548F2	1.024337	0.863838	1.214656	0.782128
MIR1183	0.982816	0.926559	1.04249	0.56439
MIR1256	1.003662	0.963976	1.044981	0.85907

PPP3R1	0.943021	0.873365	1.018233	0.134014
FANCG	1.282399	1.123308	1.464021	0.000233
AP4M1	1.059268	0.947245	1.184538	0.312677
TAS2R41	1.025521	0.961835	1.093424	0.441065
PLXNA4	1.18539	1.090857	1.288115	6.05E-05
CEBPD	0.941589	0.905109	0.979538	0.002832
OR3A2	0.957316	0.850737	1.077246	0.468835
NPTXR	1.12792	1.032377	1.232306	0.007686
PPP2R2A	0.941685	0.877436	1.010638	0.095622
TRIM16	1.138437	1.037768	1.248871	0.006055
TIGD1	1.143016	1.051983	1.241927	0.001595
FXYD7	0.985884	0.870798	1.11618	0.822389
SLC12A8	1.360576	1.228059	1.507392	3.88E-09
KIR2DS4	1.141557	1.023362	1.273402	0.017593
APOL6	1.015211	0.953254	1.081195	0.638444
FADS3	1.013014	0.947132	1.083478	0.706283
CCNL2	1.035369	0.963953	1.112075	0.340498
UBA52	0.91698	0.840199	1.000776	0.052068
MYBPHL	1.113864	1.016951	1.220012	0.020239
PPT2	0.981806	0.912581	1.056282	0.622575
ZNF630	1.093871	0.986459	1.212978	0.088863
TIAF1	1.077059	1.002741	1.156886	0.041852
DCDC2B	1.043486	0.966123	1.127044	0.278779
MIR1915	1.123394	1.082585	1.165741	7.14E-10
SNORD12I	0.972854	0.925971	1.022111	0.274784
SNORA36I	1.031551	0.944526	1.126596	0.489695
MIR1537	0.993983	0.959657	1.029536	0.736418
MIR1913	0.891834	0.840567	0.946227	0.000151
SNORD71	0.967688	0.930237	1.006646	0.102886
EXOSC6	1.079754	0.991607	1.175737	0.077397
VPS52	1.065115	0.982474	1.154708	0.125798
RPL23AP5	1.100439	1.008813	1.200387	0.030945
CDRT15	1.093272	0.993029	1.203633	0.069155
ZNF844	0.997321	0.923067	1.077549	0.945827
HBD	0.99814	0.969552	1.027572	0.900081
RFPL4A	1.01454	0.93638	1.099226	0.724149
HLA-DPB1	0.957386	0.918101	0.998353	0.041642
OR2L1P	1.058601	0.981916	1.141274	0.137728
ATXN1L	0.920873	0.861845	0.983943	0.014733
HLA-DPB2	1.072462	0.982581	1.170565	0.117235
TMEM183I	0.948472	0.855221	1.05189	0.316389
SRP14P1	1.065253	0.952369	1.191517	0.268707
INE1	0.996582	0.930473	1.067388	0.922122
BRI3P1	1.021692	0.936163	1.115035	0.63044
PLEKHM1	0.951285	0.877648	1.031099	0.22439
HSPA7	1.053777	1.013992	1.095124	0.00764
ZNF469	0.940813	0.874644	1.011989	0.101073
SLC26A6	1.112276	1.040932	1.188509	0.001655
DHRS4L1	1.170375	0.996018	1.375254	0.055941
MIAT	1.052927	0.975094	1.136973	0.188084
ERCC6	1.137595	1.042264	1.241646	0.00389
NOL7	1.029617	0.92008	1.152194	0.611054
GTF2H2B	0.947298	0.876049	1.024342	0.174745
TMEM191	1.095308	0.943195	1.271953	0.232732
NPY6R	1.081829	0.981458	1.192464	0.11337
TMEM185I	1.127037	1.041912	1.219117	0.002839
SMCR5	1.072087	0.989873	1.16113	0.087282
TAS2R46	0.995489	0.939918	1.054345	0.877385

SRRM5	0.987677	0.891022	1.094816	0.813446
FAM30A	1.085581	1.047368	1.125188	7.08E-06
PGAM4	0.956112	0.878303	1.040813	0.300058
HNRNPA3	0.76204	0.614883	0.944415	0.013051
LTA	1.006055	0.934477	1.083117	0.872628
WDR46	1.0701	0.964634	1.187098	0.200611
ZNF717	0.987298	0.917571	1.062323	0.732285
PARG	1.037319	0.966461	1.113373	0.310123
SCAMP4	0.932508	0.848765	1.024512	0.145521
LTB	1.051896	0.99226	1.115116	0.089316
POLR2J2	1.01496	0.938983	1.097084	0.708362
PPIAP19	1.059322	0.981718	1.14306	0.137645
ORM2	0.994609	0.943873	1.048072	0.839646
LYPLA2P1	0.994983	0.834941	1.185702	0.955172
CCT6P1	0.991851	0.916575	1.073309	0.838992
ARL17B	0.996722	0.913462	1.08757	0.941183
DHFR	1.075587	1.004718	1.151455	0.036143
RPL41	0.926423	0.875492	0.980316	0.008071
CCDC26	1.015627	0.939469	1.097958	0.696614
TTTY10	1.046884	0.977073	1.121684	0.193174
ORM1	1.010937	0.970859	1.052669	0.598165
HLA-DRB6	0.97785	0.942102	1.014955	0.238496
SPINK8	1.070034	0.994124	1.151742	0.07139
PATL2	1.001291	0.929971	1.07808	0.972707
ZNF492	0.889732	0.812428	0.974392	0.011757
XIST	0.996755	0.974957	1.01904	0.773286
ZNF688	1.193668	1.058006	1.346725	0.004028
EMX2OS	0.991664	0.885714	1.110288	0.884548
HBBP1	1.104024	1.045918	1.165358	0.000334
ACBD6	0.999808	0.900344	1.110259	0.997132
HERC2P4	1.14178	1.062455	1.227027	0.000307
DSCR9	1.108072	1.010316	1.215287	0.029424
ANKRD18I	1.18982	1.13262	1.249909	4.71E-12
PA2G4P4	0.894683	0.813177	0.98436	0.022406
PRB4	0.984935	0.920975	1.053336	0.657675
YY2	1.044539	0.89415	1.220223	0.582738
HSBP1	1.128862	1.016364	1.253812	0.023635
HCG18	1.048796	0.971184	1.13261	0.224536
HLA-DPA1	0.959378	0.924256	0.995834	0.029303
RPS18	0.939306	0.883103	0.999086	0.046699
SNORD62I	0.935332	0.884505	0.989081	0.019022
DLEU2	1.079533	0.988504	1.178945	0.088625
PRH1	1.022621	0.952007	1.098473	0.540048
TAPBP	1.050406	0.95439	1.156081	0.314665
ANXA2P2	0.980439	0.923464	1.040929	0.517807
MCTS1	1.081474	0.958358	1.220406	0.204018
DYTN	1.096822	1.032835	1.164773	0.002583
HLA-DQB1	0.954409	0.905854	1.005566	0.079838
TNF	1.04147	0.998135	1.086687	0.06095
SNHG7	0.856598	0.79573	0.922123	3.86E-05
RAB1C	0.910166	0.805982	1.027816	0.12911
GPX1	0.99984	0.931383	1.073329	0.996474
KRT8P15	1.0814	0.998635	1.171025	0.054062
MYCNOS	1.078973	1.00892	1.153891	0.02647
RPS28	0.932807	0.869934	1.000225	0.050743
UQCRHL	1.090068	0.963814	1.23286	0.169713
NUDT9P1	1.162521	1.035991	1.304505	0.010426
TRIM26	0.946274	0.868471	1.031047	0.20712

FAM133B	0.981078	0.890205	1.081226	0.700079
JRK	1.034156	0.9708	1.101646	0.297774
GAS5	0.863291	0.793162	0.939619	0.000672
HLA-B	0.973956	0.897428	1.057009	0.527352
MORF4	0.863	0.761992	0.977396	0.020344
MIR155HG	1.114614	1.048612	1.184771	0.000494
APOC2	1.071005	0.963637	1.190337	0.203116
SUMO1P3	0.980112	0.917682	1.046789	0.549689
PPP1R3E	1.057776	0.973245	1.149649	0.186241
SNORA71I	1.011066	0.956993	1.068194	0.694739
NFAM1	0.973331	0.936307	1.011819	0.171894
CYCSP52	1.003964	0.94712	1.064221	0.89416
KIAA0040	1.005883	0.937535	1.079214	0.870217
IFITM4P	1.190738	1.107595	1.280124	2.28E-06
B3GALT4	1.057072	0.973654	1.147637	0.185715
EGOT	1.020518	0.971623	1.071874	0.417488
PNMA6A	1.140393	1.019076	1.276151	0.022065
ZBTB22	0.963051	0.886983	1.045643	0.369817
CLEC2L	0.824404	0.749602	0.906671	6.93E-05
ZBED5	1.08568	0.996187	1.183214	0.061079
SLFN14	1.039164	0.979962	1.101942	0.19927
BCYRN1	1.043025	1.008409	1.07883	0.014435
B3GNT9	1.022882	0.941639	1.111134	0.592094
CDKN2AIP	1.054297	0.936606	1.186776	0.381296
CECR7	1.128619	1.038835	1.226162	0.004226
RGL2	1.07789	0.998123	1.164032	0.055868
ID2B	1.004733	0.904424	1.116167	0.929888
HLA-DQA	0.975115	0.944503	1.006719	0.121507
PYY2	1.088887	0.986127	1.202355	0.092232
KIFC1	1.075006	1.014716	1.138877	0.014047
IRGM	0.931115	0.869543	0.997047	0.040885
PTENP1	1.094381	0.980098	1.221991	0.108997
LRRC37A2	0.980943	0.903354	1.065196	0.647197
OR2W3	1.008464	0.963115	1.055948	0.71958
PAGE2B	1.006928	0.91004	1.114131	0.893601
SNORD12:	0.997986	0.909895	1.094606	0.965901
SNORD11	0.985176	0.92831	1.045526	0.622479
SNORD12(	1.033207	0.982675	1.086338	0.20165
SNORA13	1.082813	1.021045	1.148317	0.007933
SNORD42I	1.164538	1.074711	1.261872	0.0002
MIR1976	1.131249	1.042739	1.227271	0.003009
SCARNA7	1.034737	0.997351	1.073525	0.068962
SNORD12	1.027868	0.966469	1.093168	0.381754
SCARNA18	1.069745	1.013776	1.128804	0.013934
SNORD12:	1.005159	0.956236	1.056585	0.839828
SNORA47	0.980382	0.932241	1.03101	0.440575
SNORD13	1.019469	0.980055	1.060469	0.337809
SNORD12	0.986917	0.935165	1.041534	0.631805
SNORA59/	1.030078	0.978813	1.084027	0.255211
SNORA84	0.955807	0.886527	1.030501	0.239055
TXNDC5	0.930736	0.8508	1.018183	0.117192
RNF103	0.978602	0.917484	1.04379	0.510924
RBM14	0.966756	0.915791	1.020557	0.221122
ALKBH6	1.279367	1.108434	1.476659	0.00076
ASB14	0.934013	0.87681	0.994948	0.034256
NME1	1.196128	1.105477	1.294214	8.44E-06
TNFSF12	0.962183	0.89183	1.038086	0.319687
CDRT4	1.028562	0.936336	1.129871	0.556839

APOBEC3(	1.133096	1.022668	1.255448	0.016921
WBP1	1.157009	1.057933	1.265363	0.001408
MRPS17	1.138907	1.039959	1.24727	0.005033
DEFA3	1.018608	0.99882	1.038789	0.06547
ADSL	1.054393	0.970914	1.145049	0.208185
LILRA4	1.05744	0.972419	1.149895	0.191559
LILRA2	0.974656	0.925984	1.025885	0.326008
AMY2B	1.05085	0.948317	1.16447	0.343697
LY6G5B	1.001781	0.929643	1.079516	0.96278
PSMB9	1.153706	1.07489	1.238301	7.49E-05
PCDHGC3	0.936176	0.870348	1.006983	0.076246
COX19	1.026165	0.937924	1.122708	0.573427
DEFA1B	1.104223	1.025739	1.188712	0.008401
GHRLOS	1.039803	0.965927	1.119328	0.299266
ACAD11	1.021427	0.886841	1.176437	0.768687
PPIL3	0.943045	0.845999	1.051225	0.289893
RPL23AP7	1.00484	0.907103	1.113108	0.926321
KIR3DL2	1.157507	1.026387	1.305377	0.017099
RPL29P2	1.01244	0.859912	1.192022	0.882033
TNFRSF13	0.901171	0.771716	1.052342	0.188453
AQP1	0.974412	0.931443	1.019364	0.259964
ISY1	1.064294	0.961513	1.178063	0.229155
PCDHGC5	0.895078	0.802029	0.998922	0.04779
RDH14	1.096118	0.988801	1.215082	0.080858
PLCXD2	1.019611	0.954316	1.089375	0.565184
NSUN6	1.070878	0.99583	1.151582	0.064711
HLA-DOB	1.041916	0.973101	1.115597	0.238867
KRTAP5-8	1.055016	0.935592	1.189682	0.382243
CRCP	0.955263	0.880477	1.0364	0.271167
RPL36A	1.061531	0.975598	1.155033	0.165631
PDXP	1.11768	1.020822	1.223727	0.016148
RPP21	1.126385	0.99693	1.27265	0.056056
CD302	0.889038	0.822354	0.961128	0.00311
EGFL8	1.042877	0.954416	1.139537	0.353243
ARHGAP8	1.122031	1.026554	1.226388	0.011163
ARPC4	1.19297	1.071707	1.327954	0.001254
CORT	1.063208	0.932839	1.211797	0.358456
ARPC1A	1.125784	1.00786	1.257505	0.035849
SUMO1P1	1.070946	1.020296	1.124111	0.005558
PLEKHO2	0.979736	0.918103	1.045508	0.536883
PISD	1.06	0.968318	1.160364	0.20679
PWP2	1.170933	1.059174	1.294483	0.002048
PI4KA	1.010009	0.930771	1.095992	0.811175
AMACR	1.161353	0.969508	1.39116	0.104417
SNHG3	0.942199	0.86909	1.021457	0.148519
ARFGAP3	0.950209	0.873936	1.033139	0.231572
BGLAP	1.068898	0.973513	1.173628	0.162386
PEG10	1.135863	1.017456	1.268049	0.023325
AK2P2	1.027368	0.928724	1.136489	0.600109
EIF6	1.05128	0.95422	1.158212	0.311625
PCDHGC4	0.907007	0.816998	1.006932	0.067188
MRPL20	1.201493	1.048608	1.37667	0.008206
SERPINB1(	0.964429	0.9274	1.002936	0.069804
HLA-DMB	0.947639	0.902802	0.994703	0.029649
DECR2	1.063574	0.952553	1.187536	0.273181
CNTF	0.983463	0.918187	1.05338	0.634166
ZNF702P	0.91117	0.799754	1.038106	0.162125
ZNF709	0.994151	0.881464	1.121245	0.923867

EIF4EBP3	0.932109	0.851888	1.019884	0.125727
ABCC13	1.036259	0.926064	1.159568	0.534658
MRPL33	1.034507	0.97633	1.09615	0.250639
MICAL3	0.967462	0.902068	1.037598	0.35426
STON1	1.222275	1.080738	1.382349	0.001391
PRAF2	1.178347	1.037908	1.337788	0.011258
KCTD7	0.958405	0.90128	1.019151	0.17543
EFNA4	1.19356	1.095657	1.300212	5.08E-05
INGX	1.087701	0.94809	1.247871	0.230366
AOX2P	0.951883	0.911289	0.994286	0.026578
UPK3B	1.122348	1.018063	1.237316	0.020355
IL10RB	1.049922	0.942372	1.169747	0.376961
CFB	1.084921	0.964985	1.219764	0.17268
ZNF487	1.0534	0.940038	1.180432	0.370508
WDR92	1.116481	1.013391	1.230058	0.025809
NME2	1.181875	1.056846	1.321695	0.003399
PLA2G4B	1.255037	1.038892	1.516151	0.018492
TTC4	1.477121	1.216801	1.793133	8.02E-05
RPLP0P2	1.129081	0.96433	1.321979	0.131398
JMJD7	1.144063	1.048075	1.248842	0.002611
APOBEC3D	0.972651	0.851425	1.111137	0.683052
RPL12P6	0.933719	0.851811	1.023504	0.14319
MRPS6	0.958444	0.884137	1.038996	0.302612
ZNF512	1.062178	0.984938	1.145476	0.117357
NFS1	1.206325	1.099398	1.32365	7.46E-05
DDOST	0.981992	0.903175	1.067688	0.670334
TMEM199	1.189515	1.067705	1.32522	0.001641
P2RY11	1.051549	0.972161	1.137421	0.20947
TMEM141	1.156759	1.034874	1.292998	0.010365
DBNDD2	1.04898	0.906708	1.213576	0.520212
ETV5	0.977328	0.921447	1.036598	0.445212
KRTAP5-7	0.973878	0.852308	1.112788	0.697219
RBM12	0.999568	0.923132	1.082332	0.991497
LILRA6	1.009369	0.959548	1.061777	0.718033
SCARF2	1.135928	1.02543	1.258332	0.01465
APOBEC3C	1.068334	0.983577	1.160395	0.117039
KRT18P34	1.094836	0.987462	1.213887	0.085363
ASPRV1	1.145388	1.044505	1.256014	0.003907
FCGR2C	1.043744	1.001281	1.088009	0.043345
UBE2V1	0.988855	0.927227	1.054578	0.732821
HBB	0.965436	0.940258	0.991287	0.00908
N4BP2L2	1.02768	0.95921	1.101038	0.437659
ZNF585B	1.100673	1.013844	1.194937	0.022143
CEBPA	0.933799	0.890465	0.979243	0.004726
SNHG6	0.992702	0.944299	1.043586	0.773969
DNM1P35	1.429465	1.282442	1.593342	1.10E-10
PGAM5	0.962973	0.900547	1.029726	0.26987
SNHG10	1.237371	1.126392	1.359285	8.90E-06
ZCCHC3	1.043624	0.952576	1.143375	0.35925
TWF2	1.132638	1.032151	1.242909	0.0086
MARS2	1.091787	1.016235	1.172956	0.01639
USP51	1.125468	1.02372	1.237328	0.01449
BCKDHA	1.20761	1.08306	1.346482	0.000682
INSL3	0.93314	0.872542	0.997948	0.043392
PCDHGB8P	0.969741	0.885187	1.062373	0.509188
POU5F2	1.030961	0.967552	1.098526	0.346462
PCP4L1	1.034582	0.939359	1.139459	0.490122
ABHD14A	1.301639	1.178197	1.438015	2.15E-07

ANP32C	1.089237	0.896959	1.322734	0.388368
CCDC153	0.851043	0.732815	0.988346	0.034552
ACTN3	0.927268	0.797118	1.078667	0.327777
FMN1	1.180982	1.092714	1.276381	2.70E-05
SNORA58	1.065957	1.007954	1.127297	0.025255
HAUS5	1.210167	1.103065	1.327669	5.47E-05
SLED1	1.033789	0.989521	1.080037	0.136703
TMEM150	1.215665	1.112716	1.32814	1.52E-05
NAIP	1.076513	1.009392	1.148098	0.024797
ZNF324B	1.127245	1.032884	1.230226	0.007245
ZNF564	1.017127	0.950881	1.087989	0.621152
PVT1	1.038361	0.969252	1.112397	0.284066
PDCD6	1.067402	0.986264	1.155215	0.105865
TMEM158	1.044371	0.986352	1.105803	0.136559
YJEFN3	0.983172	0.909345	1.062993	0.670024
PTTG2	1.009853	0.925356	1.102066	0.825935
ZNF718	0.967177	0.914897	1.022445	0.239163
GYPB	1.017087	0.977503	1.058273	0.402868
CHCHD10	1.032683	0.97499	1.093789	0.272883
GPR162	0.988678	0.933548	1.047063	0.697293
ATP6V1E2	1.195802	1.054977	1.355426	0.005156
GLI4	1.172834	1.078217	1.275755	0.000203
RNF138P1	0.884283	0.819866	0.953763	0.001439
ZNF674	1.057883	0.962745	1.162422	0.241874
ZNF345	0.986284	0.911818	1.066832	0.730236
SHANK3	0.975817	0.940303	1.012671	0.195578
ZNF550	1.014832	0.942181	1.093084	0.697663
RPL32P3	1.020797	0.951761	1.094841	0.56453
FOXD1	1.260646	1.1292	1.407393	3.74E-05
MALAT1	0.95066	0.904927	0.998704	0.044271
PRB1	0.999187	0.922046	1.082782	0.984165
SCARNA8	0.98675	0.935164	1.041181	0.626343
SCARNA6	1.01208	0.981174	1.043959	0.447931
SCARNA2	1.057191	0.997411	1.120554	0.061114
SNORD79	0.994696	0.935887	1.057201	0.864201
SCARNA1	1.024225	0.959611	1.09319	0.471554
SCARNA5	1.015099	0.992789	1.03791	0.18627
SNORD27	1.021891	0.965736	1.08131	0.452692
MIR2278	1.026892	0.983585	1.072105	0.227405
RNY4	1.038697	0.976434	1.104931	0.228657
SCARNA2	1.037608	0.979926	1.098686	0.205844
MIR2276	0.993767	0.955417	1.033656	0.755492
SCARNA1	1.07492	0.997372	1.158498	0.058614
SCARNA2	1.046049	1.020428	1.072314	0.000373
SCARNA3	1.013879	0.97284	1.056649	0.513229
SCARNA1	1.039689	0.992091	1.08957	0.103551
MIR2116	1.082377	1.028624	1.13894	0.00232
PCDHGA1	0.977585	0.888236	1.075922	0.642959
HOXA10	1.159087	1.122658	1.196697	1.29E-19
TMEM200	0.932336	0.810799	1.072091	0.325535
PCDHGB6	0.985827	0.889454	1.092642	0.785656
SERPINE3	0.981587	0.897843	1.073143	0.682939
TUG1	1.056091	0.968881	1.151152	0.214588
TRNP1	1.074381	0.995778	1.159189	0.064196
PCDHGA5	0.975219	0.883082	1.076968	0.620194
NACA2	0.966389	0.886138	1.053907	0.439555
PCDHGA7	0.93198	0.854196	1.016846	0.113135
RNF5P1	1.038687	0.962886	1.120456	0.326219

SLC10A5	0.930428	0.859383	1.007346	0.075183
EIF5AL1	1.044695	0.930081	1.173433	0.46084
ALG11	1.080065	0.988236	1.180428	0.089332
PRKDC	1.06613	1.006218	1.129609	0.030004
PCDHGA6	0.925572	0.855682	1.001169	0.053509
PCDHGA8	0.942981	0.837242	1.062073	0.333287
PCDHGA1	1.01548	0.930347	1.108403	0.730952
PCDHGA1	0.938567	0.858881	1.025645	0.161337
PCDHGB2	0.958965	0.903053	1.018338	0.171604
PCDHGB4	0.895952	0.797083	1.007085	0.06553
CLDN23	1.172339	1.085927	1.265627	4.70E-05
ZNF260	1.031575	0.948067	1.122438	0.470445
LYN	0.948939	0.877294	1.026434	0.190685
PINX1	1.202482	1.076225	1.34355	0.001122
PCDHGB7	0.938931	0.861116	1.023779	0.153419
SIGLEC14	1.019084	0.974296	1.065932	0.40972
CHMP4A	1.106033	0.988165	1.237961	0.079621
SIGLEC12	1.070129	1.024603	1.117678	0.002245
FLJ20021	0.868201	0.788489	0.955971	0.004023
PABPC4L	1.042354	0.956331	1.136115	0.345208
OMP	1.331633	1.195334	1.483474	2.01E-07
FPGT	1.173371	1.081618	1.272908	0.000119
MEX3A	1.044607	0.979689	1.113827	0.182493
SLC22A18,	1.293919	1.115112	1.501397	0.000684
MPV17L2	1.111661	1.016221	1.216064	0.020817
SCARNA9	1.043475	0.980537	1.110452	0.180006
DPP3	1.082222	1.001708	1.169208	0.045152
ANKHD1-I	1.000164	0.91188	1.096995	0.99723
KRTAP5-9	0.956298	0.860403	1.06288	0.407194
CHMP1B	1.072234	0.972597	1.182077	0.161039
EID3	0.942801	0.893426	0.994904	0.031865
SNHG9	1.013926	0.967652	1.062412	0.561732
CARD17	1.090152	0.96162	1.235863	0.177487
EID1	1.178728	1.088928	1.275932	4.76E-05
TAS2R43	0.994425	0.940495	1.051448	0.844205
LY6G6E	1.097626	1.001978	1.202404	0.045236
FDXACB1	1.25469	1.089984	1.444283	0.001578
SNHG1	0.931662	0.864209	1.004379	0.06489
TIFAB	1.034564	0.98115	1.090885	0.208987
CYP2A6	0.987262	0.913153	1.067386	0.747454
CTSO	1.016076	0.947783	1.089289	0.65326
ZNF432	1.071403	0.990813	1.158548	0.083872
TAS2R30	0.996192	0.949987	1.044644	0.874879
ZNF10	0.952481	0.886061	1.02388	0.186811
ZNF486	0.911981	0.857668	0.969734	0.003272
HMBS	1.03721	0.987553	1.089364	0.144405
ZNF225	1.229527	1.09802	1.376785	0.000343
TAS2R31	1.004464	0.953418	1.058244	0.867069
POLG2	1.009874	0.939258	1.0858	0.790499
CLEC12B	0.980141	0.912535	1.052756	0.582264
ZNF350	1.06104	0.971006	1.159423	0.190324
ZNF253	1.011436	0.938914	1.08956	0.764521
CAPNS2	1.039652	0.962745	1.122701	0.321342
HP	0.961109	0.913407	1.011302	0.1267
LSM14A	0.930148	0.84904	1.019005	0.119819
NHLRC4	0.972546	0.904043	1.04624	0.45507
GATC	1.001578	0.925396	1.084033	0.968836
MGAM	0.998554	0.960968	1.037609	0.941054

FNTB	0.977325	0.904721	1.055755	0.560322
ZNF878	0.939229	0.882457	0.999654	0.048741
CNPY2	1.219311	1.085969	1.369025	0.000792
MRS2P2	0.974884	0.926542	1.025748	0.326954
CUX1	0.88115	0.81392	0.953933	0.00178
MAP1LC3E	1.06949	0.97511	1.173005	0.154084
CLEC5A	0.900164	0.865141	0.936606	2.05E-07
CHURC1	1.091329	1.007277	1.182394	0.032575
RTEL1	1.260996	1.107742	1.435452	0.000452
PDF	0.959641	0.861068	1.069499	0.456302
SPESP1	0.987877	0.905262	1.078032	0.78429
MC1R	0.94414	0.860135	1.036349	0.226661
TUBB3	0.869445	0.769745	0.982058	0.024366
ITGB3	1.123542	1.084877	1.163585	7.06E-11
THTPA	1.103255	1.003677	1.212713	0.04175
MRPL46	1.533265	1.331912	1.765058	2.68E-09
TYRO3P	0.985431	0.927272	1.047237	0.636316
RBM15B	0.927731	0.858618	1.002407	0.057552
HOXB7	1.131484	1.080807	1.184537	1.27E-07
MRC1	1.031734	0.987915	1.077497	0.158281
HSPB9	1.017846	0.948583	1.092167	0.62276
MT1L	1.134489	1.064564	1.209006	0.000101
CCPG1	0.999094	0.92008	1.084894	0.982799
EPPK1	1.017823	0.923075	1.122297	0.723065
BOP1	1.078808	1.000574	1.163159	0.048278
FIGNL2	1.24372	1.117637	1.384026	6.35E-05
PECAM1	1.150295	1.083566	1.221134	4.39E-06
UBE2MP1	0.821096	0.733973	0.918561	0.000573
GAN	1.080722	0.996068	1.17257	0.06214
HPR	1.112607	0.979292	1.26407	0.101292
PCDHGA9	0.966543	0.876051	1.066382	0.497461
PCDHGB3	0.91951	0.858107	0.985308	0.017326
CORO7	1.045296	0.955151	1.143948	0.335677
SPON1	1.108436	1.035177	1.186878	0.003168
MRPL12	1.126903	1.023384	1.240894	0.015094
ALOX12P2	1.018818	0.966958	1.073459	0.484291
GTF2I	0.964816	0.891489	1.044174	0.374473
ZNF234	1.121425	1.037403	1.212253	0.003926
IKBKE	0.98225	0.920135	1.048558	0.591039
SNORD43	0.998599	0.914376	1.090578	0.975112
SNORD3A	1.022644	0.984061	1.062739	0.253817
NBPF11	1.04436	0.964072	1.131334	0.28757
RNU4ATA(	1.004244	0.962037	1.048303	0.846709
DYNLL2	0.943156	0.858462	1.036206	0.222806
OTUD7B	1.054695	0.974714	1.141239	0.185685
DPRXP4	1.013574	0.944735	1.087429	0.707131
SNORD3C	1.027629	0.993455	1.062979	0.114238
SNORD92	1.014901	0.970192	1.061671	0.519905
SNORD53	0.97127	0.926373	1.018343	0.227355
ANXA8	0.801806	0.718531	0.894733	7.88E-05
RBM8A	1.155965	1.024696	1.30405	0.018441
ARGFXP2	0.961029	0.908441	1.016662	0.166222
TIMM23	1.067763	0.963824	1.182911	0.209552
RNF115	0.924981	0.847963	1.008995	0.078733
RPL17	1.017445	0.923802	1.120581	0.725531
SEC22B	1.116552	1.021041	1.220996	0.015676
FSBP	1.26084	1.097628	1.448321	0.001049
TXNIP	0.93633	0.886472	0.988992	0.018451

SRGAP2	1.101448	1.025352	1.183192	0.00816
BAHCC1	1.058229	1.015833	1.102395	0.006669
RASSF5	0.998197	0.930429	1.070901	0.959876
STRADA	0.964593	0.900077	1.033733	0.307427
MIR744	0.968608	0.919416	1.020432	0.230383
NBPF15	1.071448	0.984128	1.166516	0.111592
NCOA4	0.956821	0.889772	1.028922	0.233737
MRPS21	1.128539	1.015626	1.254004	0.02456
GDF10	1.252261	1.149544	1.364156	2.58E-07
MYO15B	0.959242	0.905768	1.015873	0.155072
AARSD1	1.144428	1.025633	1.276983	0.015839
MIR212	1.030345	0.96664	1.098248	0.358613
MIR132	1.030903	0.960767	1.10616	0.397204
LCN6	0.840104	0.76644	0.920848	0.000198
S1PR2	1.138436	1.063249	1.218939	0.0002
ZNF224	1.126266	1.029037	1.232682	0.009841
LIN37	1.031805	0.869936	1.223793	0.71914
NDUFA7	1.035657	0.957388	1.120325	0.382196
NBPF12	1.016843	0.94573	1.093304	0.651604
CLEC4GP1	1.204402	1.119762	1.29544	5.66E-07
CT45A1	1.082536	1.023418	1.14507	0.005643
MAGIX	1.227456	1.092101	1.379587	0.000586
IKBKG	1.064548	0.960597	1.179747	0.232812
SPIB	1.021472	0.952991	1.094875	0.548478
TMEM185/	0.98406	0.880877	1.099329	0.776164
NBPF9	1.085451	0.991193	1.188672	0.076876
KCNQ1OT	1.038595	0.974455	1.106957	0.244285
EGLN2	1.002951	0.947017	1.062189	0.919834
SNHG8	0.865134	0.797799	0.938153	0.000458
TERC	1.156533	1.043876	1.281348	0.005416
NBPF8	1.010059	0.948832	1.075237	0.753746
NBPF14	1.025233	0.943197	1.114404	0.558123
TAF15	0.963415	0.885811	1.047818	0.384394
RASL10B	1.149138	1.002857	1.316757	0.045388
SRXN1	1.204522	1.040338	1.394617	0.012814
NBPF10	1.022192	0.932764	1.120192	0.638435
MMP28	0.845967	0.790723	0.90507	1.21E-06
CCL5	1.03567	0.988238	1.085379	0.142839
LIX1L	1.04796	0.965919	1.13697	0.260046
SNORD11I	1.014596	0.966494	1.065092	0.558725
MIR98	1.037522	0.979379	1.099117	0.210623
ANKRD34/	1.069496	0.941717	1.214612	0.300692
SNORD14/	1.053244	0.983631	1.127784	0.13704
MIR139	0.949829	0.872924	1.03351	0.232148
GTF2H5	1.215298	1.074345	1.374744	0.001935
NUDT3	0.576201	0.474375	0.699885	2.75E-08
POM121C	0.894827	0.821617	0.974561	0.010722
CD24	0.995165	0.965254	1.026003	0.755588
SNORA28	1.01464	0.976777	1.05397	0.453848
ZNF595	1.034158	0.980884	1.090326	0.213241
DOC2B	0.887505	0.841778	0.935715	9.79E-06
MESTIT1	0.839478	0.750385	0.93915	0.002238
DCP1A	0.909695	0.853754	0.9693	0.003468
FAM106A	1.107404	1.030662	1.18986	0.005366
SNURF	1.047775	0.969943	1.131852	0.236007
DGCR11	1.088455	1.005468	1.178291	0.036195
ZNHIT3	1.272979	1.103711	1.468207	0.000915
CYFIP1	1.044827	0.982302	1.111332	0.163674

USP27X	1.22369	1.113968	1.34422	2.53E-05
TAF9	0.967922	0.87227	1.074062	0.539119
NOL12	1.178211	1.080245	1.28506	0.000213
ANTXRL	1.132226	0.987871	1.297675	0.074326
SOCS7	1.009276	0.937438	1.086618	0.806396
ADRA2B	0.896803	0.818842	0.982187	0.018909
ZNF658	1.398123	1.253146	1.559873	1.97E-09
SEBOX	1.115453	0.965976	1.288061	0.136643
PI4KAP1	1.020468	0.961551	1.082994	0.504283
CCL23	1.062516	1.028886	1.097245	0.00022
RNU11	1.031677	0.979398	1.086746	0.239849
ZNF280B	1.147317	1.044289	1.260509	0.004201
MLLT6	1.114358	1.02469	1.211871	0.011412
NUDT18	1.096299	1.017616	1.181066	0.015541
SNORD91I	1.125408	1.040841	1.216846	0.003034
ZNF2	1.153105	1.041393	1.2768	0.006142
LENG9	1.00999	0.934387	1.091709	0.80228
CCL4	1.116387	1.064511	1.17079	5.76E-06
MIR1299	1.002416	0.964179	1.042169	0.903216
CCL18	1.027369	0.924669	1.141475	0.615331
FCGBP	0.973888	0.891489	1.063904	0.55747
XKR5	1.188655	1.052737	1.342122	0.005279
MT1IP	0.97638	0.845892	1.126996	0.743986
AATF	1.067487	0.948564	1.20132	0.278497
ARHGAP2:	0.918045	0.86334	0.976217	0.006375
TUBGCP5	1.116595	1.031028	1.209264	0.006706
PRSS2	1.229069	1.1786	1.281699	5.35E-22
DUSP14	1.003622	0.930458	1.082538	0.925418
UHRF1	1.020969	0.967309	1.077606	0.451233
ORAI1	0.990896	0.919304	1.068062	0.811076
CCL4L2	1.095456	1.047429	1.145686	6.73E-05
CCL3L3	1.064738	1.025656	1.105309	0.00101
RN7SL1	1.017887	0.995499	1.040778	0.11819
PIK3R6	0.969397	0.912742	1.029568	0.311739
TADA2A	1.101658	1.013023	1.198048	0.023679
PIP4K2B	1.069502	0.998822	1.145184	0.054082
PCDHGB5	0.974542	0.884555	1.073683	0.601882
HERC2P2	1.064455	1.009064	1.122887	0.021968
RAB7B	0.957	0.916449	0.999344	0.046628
DACH1	1.03916	1.003734	1.075837	0.029964
PADI6	1.083335	0.972564	1.206723	0.145819
GTF2IP1	0.962086	0.855271	1.08224	0.519758
STAG3L2	0.949083	0.892469	1.009287	0.09584
TYW1B	0.985423	0.917517	1.058355	0.686878
F8A3	0.887635	0.741864	1.062049	0.192829
PIGW	1.163695	1.061256	1.276022	0.001262
F8A1	0.982862	0.915227	1.055496	0.634647
PCGF2	1.300035	1.205042	1.402515	1.22E-11
MARCKS	1.096296	1.056642	1.137438	1.00E-06
ZNF670	1.075728	0.976272	1.185316	0.140267
PKD1L3	1.087941	0.994283	1.190422	0.066486
CCL3	1.084977	1.03361	1.138896	0.000981
PGM5P2	0.986511	0.91993	1.057912	0.703266
PSMB3	1.248083	1.124834	1.384836	2.95E-05
CISD3	1.275576	1.158746	1.404186	6.83E-07
RDM1	1.547885	1.37803	1.738676	1.75E-13
DDX52	1.006713	0.932384	1.086968	0.864247
ZNF8	0.968538	0.905336	1.036152	0.35316

SSTR3	1.031868	0.92065	1.156521	0.589801
MYO19	1.112212	1.021416	1.21108	0.014379
GGNBP2	0.957955	0.880566	1.042145	0.317578
ZNF229	1.103058	0.99338	1.224844	0.066408
DHRS11	1.005766	0.938659	1.07767	0.870377
ACACA	1.000968	0.939156	1.066847	0.976276
TMEM191I	1.088826	0.959321	1.235813	0.187781
NR2E3	1.050929	0.925454	1.193417	0.443833
MRM1	1.068018	1.002059	1.138319	0.043052
MRPL45	0.887916	0.814714	0.967696	0.006769
OR2L8	1.10746	1.020258	1.202115	0.014718
FLJ42393	0.987693	0.927916	1.051321	0.69746
TMEM75	1.032367	0.963635	1.106001	0.364846
PLAC4	1.062306	0.983655	1.147246	0.123546
SNHG4	0.902912	0.844383	0.965499	0.00282
HSP90AB4	0.989092	0.928853	1.053238	0.732276
WASH5P	1.006756	0.885081	1.145157	0.918398
ADORA3	0.834268	0.707039	0.984391	0.031848
HYMAI	0.930236	0.859473	1.006825	0.073219
MIR1322	1.029165	0.98462	1.075725	0.202874
MIR1910	1.020785	0.972944	1.070979	0.400914
SNORD98	0.985055	0.914207	1.061394	0.692559
MIR924	1.068114	0.983101	1.160479	0.119425
EXOC3L2	1.043332	0.989938	1.099607	0.1135
MIR555	1.155458	1.064783	1.253855	0.00053
MIR130B	0.890152	0.816913	0.969956	0.0079
MIR628	0.984669	0.93218	1.040114	0.580422
MIR1281	0.99428	0.936292	1.05586	0.851587
MIR29A	0.989046	0.935831	1.045286	0.696271
SCO2	1.042743	0.952948	1.141	0.362301
MIR718	0.999117	0.941893	1.059817	0.97657
TBCE	1.033265	0.91358	1.16863	0.602375
ARHGAP1:	1.0374	0.951608	1.130927	0.404445
TOP1P1	1.042798	0.925815	1.174562	0.490008
NPBWR1	1.101023	0.99507	1.218259	0.06229

Supplementary file 3. Results of univariate Cox regression analysis in TCGA-LAML

id	HR	HR.95L	HR.95H	pvalue
TSPAN6	1.308024	0.919863	1.85998	0.134934
DPM1	1.172525	0.606693	2.266082	0.635898
SCYL3	0.989455	0.596158	1.642218	0.967289
FGR	1.193546	1.06726	1.334776	0.00193
CFH	1.03815	0.89135	1.209127	0.630287
FUCA2	1.174529	0.806424	1.710662	0.401738
GCLC	1.384228	0.985245	1.944783	0.060893
NFYA	0.644811	0.462227	0.899517	0.009782
LAS1L	0.982545	0.637264	1.514905	0.936465
ENPP4	1.275568	1.038335	1.567002	0.020434
SEMA3F	1.01047	0.750738	1.360059	0.945224
ANKIB1	0.667029	0.444891	1.000083	0.050047
CYP51A1	1.375975	0.749159	2.527244	0.303519
KRIT1	0.475548	0.275084	0.822098	0.007782
RAD52	0.857186	0.521834	1.408049	0.542817
BAD	1.733881	1.040857	2.888335	0.034536
LAP3	1.529674	1.141949	2.049042	0.004373
CD99	0.798002	0.612203	1.04019	0.095199
HS3ST1	1.052227	0.814456	1.359412	0.696872
MAD1L1	0.757041	0.454855	1.259987	0.284238
LASP1	1.417485	0.839442	2.393571	0.191823
SNX11	1.488601	0.960261	2.307637	0.075294
TMEM176,	0.988252	0.871848	1.120199	0.853375
M6PR	0.976399	0.57892	1.646781	0.92864
KLHL13	0.973209	0.816041	1.160647	0.762508
ICA1	1.011627	0.854395	1.197794	0.893298
DBNDD1	0.858823	0.630125	1.170525	0.335379
ALS2	0.721027	0.575179	0.903859	0.00456
CASP10	1.713509	1.147327	2.559091	0.0085
CFLAR	1.003017	0.660818	1.52242	0.98871
TFPI	1.066773	0.906434	1.255474	0.436674
RBM5	0.862391	0.549399	1.353694	0.519868
MTMR7	0.539141	0.361944	0.803087	0.002377
SLC7A2	0.910097	0.643776	1.286593	0.593813
ARF5	1.208865	0.819371	1.783509	0.339097
SARM1	1.345895	1.018075	1.779273	0.037002
POLDIP2	2.572155	1.335496	4.95395	0.004727
PLXND1	1.059395	0.811101	1.383696	0.67197
AK2	1.46612	1.054894	2.037653	0.022717
CD38	0.967887	0.803026	1.166594	0.731901
FKBP4	0.982245	0.749266	1.287667	0.896815
RBM6	0.784236	0.435201	1.413199	0.418574
CAMKK1	1.148464	0.841603	1.567213	0.382808
RECQL	1.057716	0.739198	1.513483	0.758887
HSPB6	1.176226	0.81279	1.702173	0.389382
NDUFAB1	1.490506	0.844864	2.629546	0.168221
PDK4	1.146207	1.009031	1.302032	0.035887
SLC22A16	0.729984	0.575173	0.926462	0.009652
ZMYND10	0.992281	0.700013	1.406578	0.965281
SLC25A13	0.747685	0.483508	1.156203	0.191085
ST7	1.081429	0.864297	1.353109	0.493601
CDC27	0.770268	0.500439	1.185585	0.235515
SLC4A1	0.905971	0.820559	1.000275	0.05064
HCCS	2.002266	1.197059	3.349097	0.008162
DVL2	1.030984	0.69516	1.529041	0.879392
UPF1	1.759156	1.137902	2.719591	0.011048

SKAP2	1.170731	0.994339	1.378413	0.058513
SLC25A5	1.402847	0.910235	2.162056	0.125078
HOXA11	1.096112	0.941566	1.276024	0.236624
POLR2J	1.425908	0.85599	2.375278	0.172965
DHX33	0.764036	0.512005	1.140125	0.187555
THSD7A	0.963904	0.833298	1.114979	0.620676
LIG3	0.563919	0.372847	0.852908	0.006654
RPAP3	1.079239	0.674197	1.727622	0.750737
ACSM3	0.811364	0.70375	0.935433	0.003985
CIAPIN1	1.811119	0.990923	3.310198	0.053566
SPPL2B	0.909541	0.709505	1.165974	0.454332
COPZ2	1.122538	0.900984	1.398571	0.302795
PRKAR2B	1.20174	1.031589	1.399955	0.018313
MSL3	1.41666	0.909662	2.206232	0.123306
CREBBP	0.977678	0.628508	1.52083	0.920231
MPO	0.877387	0.818684	0.940298	0.000214
WDR54	1.458565	1.043484	2.03876	0.027169
CROT	0.893476	0.61418	1.299781	0.555886
ABCB4	1.286943	0.741975	2.23218	0.369281
RHBDD2	1.127246	0.854149	1.487661	0.397441
IBTK	0.687311	0.449564	1.050787	0.083409
ZNF195	0.656385	0.48289	0.892213	0.007184
MYCBP2	0.927629	0.654858	1.314018	0.67241
FBXL3	0.901655	0.552859	1.470503	0.678269
ITGAL	1.433612	1.167971	1.759669	0.000571
PDK2	1.191421	0.870212	1.631191	0.274535
ITGA3	1.088823	0.820365	1.445131	0.555766
ZFX	0.873263	0.558715	1.364897	0.552014
LAMP2	0.646447	0.39518	1.057477	0.082317
ITGA2B	1.074775	0.935185	1.235199	0.309672
GDE1	1.1988	0.684604	2.099201	0.525856
CRLF1	1.057127	0.524977	2.128698	0.876379
OSBPL7	1.187032	0.794771	1.772895	0.402202
TMEM98	0.933823	0.682204	1.278247	0.669066
YBX2	0.854811	0.554546	1.317659	0.477375
MAP3K14	1.453573	1.045284	2.021339	0.026201
TMEM132/	1.136206	0.791577	1.630877	0.488639
AP2B1	1.068456	0.697301	1.637167	0.761048
ZNF263	1.016612	0.562223	1.838238	0.956524
SPATA20	1.267853	0.973942	1.65046	0.077776
TNFRSF12/	0.992469	0.780786	1.261543	0.950754
MAP3K9	1.244809	0.884444	1.752005	0.209197
RALA	0.832632	0.560426	1.23705	0.364515
BAIAP2L1	0.744994	0.515692	1.076255	0.116781
AGK	0.787886	0.462051	1.343495	0.381275
ALDH3B1	1.173511	0.940428	1.464363	0.156691
TTC22	1.266061	0.948149	1.690568	0.109806
PHTF2	1.121308	0.756242	1.662605	0.568866
FARP2	1.563409	1.041606	2.346613	0.031029
GGCT	1.164362	0.756751	1.791525	0.488827
DBF4	0.762519	0.472556	1.230405	0.266731
TBXA2R	1.163004	1.002441	1.349285	0.046357
IFRD1	0.701174	0.495584	0.992053	0.04496
COX10	1.147154	0.619069	2.12571	0.662675
GTF2IRD1	0.914521	0.702911	1.189837	0.505751
PAF1	2.550544	1.218606	5.338288	0.012969
VPS41	1.015119	0.660499	1.560133	0.94544
ELAC2	1.574458	0.932918	2.657164	0.089147

ARSD	0.995799	0.763977	1.297966	0.975163
PNPLA4	1.475556	0.970158	2.244239	0.06901
ADIPOR2	1.237789	0.742299	2.064022	0.413531
CDKL3	0.497874	0.236043	1.050144	0.06703
PRSS21	0.98683	0.915906	1.063246	0.727551
MARK4	0.920525	0.560728	1.511188	0.743346
PROM1	1.036318	0.953743	1.126043	0.399758
CCDC124	1.950503	1.36656	2.783969	0.000233
CEACAM2	1.440663	1.129655	1.837296	0.003256
PAFAH1B1	0.725104	0.402555	1.306099	0.284366
KIAA0100	0.986715	0.680702	1.430299	0.943714
GAS7	1.104335	0.911223	1.338373	0.311549
TRAPPC6A	0.995042	0.718846	1.377358	0.976097
MATK	0.934653	0.741437	1.178221	0.56736
CD79B	1.391767	1.10339	1.755514	0.005263
SCN4A	1.526465	1.094368	2.129169	0.012735
ST7L	0.76093	0.412515	1.403621	0.381793
TKTL1	1.217282	0.944067	1.569566	0.129485
RPUSD1	1.491871	1.038851	2.142443	0.030282
RHBDF1	1.130884	0.955203	1.338875	0.153318
LUC7L	0.569826	0.329231	0.986243	0.044489
CACNA2D	0.929748	0.749302	1.153648	0.508188
BAIAP3	1.166909	0.955694	1.424805	0.129736
PIGQ	0.953149	0.702891	1.29251	0.757486
TEAD3	0.989476	0.718725	1.362221	0.948283
DNAJC11	1.243078	0.686708	2.250217	0.47236
MYLIP	0.993429	0.704662	1.400532	0.96999
E2F2	1.128949	0.880044	1.448252	0.339869
PSMB1	1.784095	0.968496	3.286533	0.063273
SYN1	0.856001	0.6026	1.21596	0.385301
JARID2	0.589817	0.364231	0.95512	0.031819
CDKL5	1.172052	0.890667	1.542335	0.257059
CAMK1G	5.301362	0.161121	174.4302	0.349395
NADK	1.341704	1.038664	1.733159	0.024424
DLEC1	0.856926	0.561172	1.308551	0.474683
CYTH3	0.778951	0.557067	1.089213	0.144184
ADAM22	0.783307	0.606854	1.011067	0.060731
SYPL1	0.776031	0.528197	1.140151	0.196437
CYB561	1.400851	1.038513	1.889611	0.027284
SPAG9	0.770416	0.478154	1.241315	0.283845
CELSR3	1.055024	0.832246	1.337435	0.658049
AASS	0.962211	0.805924	1.148806	0.670129
PLEKHG6	0.648863	0.475615	0.885217	0.006347
SS18L2	2.414363	1.287187	4.528594	0.006021
MPND	0.829668	0.604351	1.138988	0.248091
MGST1	0.883785	0.788099	0.991088	0.034594
CRY1	1.259438	0.929793	1.705953	0.136274
PGLYRP1	0.985682	0.847565	1.146307	0.851484
NFIX	1.092803	0.845772	1.411985	0.497277
ST3GAL1	1.17004	0.782662	1.749153	0.443993
MMP25	0.883842	0.725804	1.076292	0.219263
IL32	1.166648	0.935775	1.454482	0.170698
PKD1	0.899965	0.692034	1.170371	0.431686
MED24	1.230578	0.791287	1.913748	0.357089
RHOBTB2	2.166647	1.543622	3.041131	7.84E-06
HEATR5B	1.075947	0.700041	1.653704	0.738534
SEC62	0.901133	0.592812	1.369809	0.626098
RPS20	1.276672	0.805645	2.02309	0.29839

CSDE1	0.917868	0.60044	1.403108	0.692257
UBE3C	0.722154	0.431826	1.207676	0.214705
REV3L	0.618514	0.429675	0.890347	0.009742
MASP2	0.835567	0.575358	1.213459	0.345345
FAM76A	1.345623	0.729974	2.480501	0.341444
TRAF3IP3	1.224477	0.876105	1.711374	0.235779
POMT2	0.954571	0.654323	1.392593	0.809332
VTA1	1.079786	0.650423	1.792582	0.76661
MLXIPL	1.21267	0.882822	1.665758	0.233854
BAZ1B	1.062767	0.661344	1.707846	0.801404
RANBP9	0.689184	0.395217	1.201808	0.189507
ETV7	1.266991	0.964401	1.66452	0.089201
DYRK4	2.561907	1.255468	5.227825	0.009734
ZNF207	0.539697	0.308657	0.94368	0.030519
UQCRC1	1.970608	1.303733	2.978597	0.001289
STARD3NL	0.659204	0.37395	1.162054	0.149664
CD9	0.989345	0.864199	1.132613	0.876624
NCAPD2	1.076592	0.76035	1.524366	0.67747
IFFO1	0.927148	0.659236	1.303938	0.663758
GIPR	1.051358	0.788121	1.402517	0.733394
PHF7	0.687492	0.452619	1.044245	0.078924
SEMA3G	0.851825	0.633471	1.145444	0.288543
NISCH	1.059931	0.783078	1.434665	0.706298
STAB1	1.024641	0.946947	1.10871	0.545148
FUZ	1.046517	0.643949	1.700754	0.854394
SLC6A13	0.81861	0.325426	2.059214	0.670654
IDS	0.84046	0.605078	1.167409	0.299875
PRSS3	0.576054	0.362903	0.914398	0.019307
ZNF200	0.903616	0.577798	1.41316	0.656886
CD4	1.251775	1.073569	1.459563	0.004158
LRRC23	0.779772	0.488677	1.244266	0.296794
BTK	1.527776	0.977901	2.386846	0.062632
HFE	1.290635	0.972331	1.713139	0.077434
SCMH1	0.75295	0.57097	0.992931	0.044408
FYN	1.032462	0.808016	1.319251	0.798385
HIVEP2	0.860614	0.700241	1.057716	0.153675
LYPLA2	1.91867	1.238193	2.973118	0.003545
CLCN6	1.174175	0.738428	1.867057	0.497431
MRC2	0.847195	0.748066	0.95946	0.009007
TSPAN9	1.037722	0.788598	1.365546	0.791502
BTBD7	0.844652	0.504974	1.41282	0.520059
APBA3	1.293978	0.853838	1.961003	0.224361
MKS1	0.880779	0.566473	1.369476	0.572942
ABHD5	0.974635	0.717682	1.323584	0.869303
AKAP8L	1.098339	0.70209	1.718225	0.681199
MBTD1	0.517371	0.351215	0.762134	0.000855
UTP18	0.746761	0.46899	1.189048	0.218554
RNF216	0.835716	0.492838	1.41714	0.505376
TTC19	0.724544	0.420332	1.248928	0.246118
PTBP1	1.016091	0.615083	1.678538	0.950302
DPF1	0.752937	0.523667	1.082584	0.125605
LARS2	0.95476	0.583946	1.561047	0.853578
PIK3C2A	0.819556	0.514933	1.304389	0.401334
PLAUR	1.022245	0.862373	1.211755	0.79984
ANLN	0.817105	0.630676	1.058642	0.126345
WIZ	0.926471	0.62208	1.379805	0.707066
RABGAP1	1.247031	0.879684	1.767776	0.214992
QPCTL	0.950114	0.669604	1.348135	0.77438

PPP5C	1.764405	1.029112	3.02506	0.03899
CEP68	0.848473	0.57533	1.251292	0.407112
MAP4K3	0.675826	0.46016	0.99257	0.045717
ZBTB32	1.254023	0.909604	1.728856	0.16708
TYROBP	1.128465	0.95431	1.334401	0.157614
TMEM159	1.273325	0.821037	1.974765	0.280482
BRCA1	0.946006	0.704981	1.269435	0.711429
ERCC1	1.716449	1.079647	2.728852	0.022375
CD22	1.169157	0.958686	1.425836	0.122754
SEMA3B	0.808651	0.511412	1.278647	0.363606
MBTPS2	0.654567	0.40763	1.051097	0.079476
PRICKLE3	1.592469	1.033796	2.453053	0.034794
LTF	0.981419	0.896798	1.074024	0.683496
EXTL3	1.249187	0.831958	1.875657	0.283338
ELOVL5	1.018949	0.792851	1.309523	0.883414
ALOX5	0.995674	0.883468	1.122131	0.943344
CALCOCO	0.933122	0.634256	1.372817	0.725293
UBR7	1.195309	0.742179	1.925091	0.463122
MAP4K5	0.626176	0.416754	0.940834	0.024224
EHD3	0.986543	0.813291	1.196701	0.89063
PSMC4	2.184279	1.27609	3.738824	0.004386
MAN2B2	1.132439	0.782527	1.638817	0.509546
SLC25A39	1.03266	0.768612	1.387421	0.83109
MVP	1.551315	1.231312	1.954483	0.000195
NUB1	1.336504	0.833184	2.143877	0.228965
PGM3	0.911568	0.621276	1.337499	0.635975
RWDD2A	0.997446	0.697161	1.427072	0.988835
CLK1	0.627255	0.39839	0.987599	0.044026
POLR3B	0.707871	0.424893	1.179314	0.184626
ANGEL1	1.738614	1.090434	2.772086	0.020142
RNF14	0.861275	0.562662	1.318367	0.491752
DNASE1L1	2.209972	1.497677	3.261034	6.48E-05
DDX11	1.556969	1.066009	2.274044	0.021982
HEBP1	1.153562	0.716188	1.85804	0.556942
GPRC5A	0.162911	0.006137	4.324408	0.278066
MAMLD1	0.877969	0.672105	1.146888	0.339756
CD6	0.991468	0.817216	1.202874	0.930757
TACC3	1.523321	0.96165	2.413047	0.072918
POLA2	1.404954	0.857213	2.302692	0.177408
ZC3H3	0.896125	0.670507	1.197663	0.458621
CAPN1	1.797663	1.18762	2.721066	0.005555
MDH1	1.580903	0.963583	2.59371	0.069815
SLC30A9	0.908171	0.552433	1.492986	0.704109
MTMR11	1.064405	0.948368	1.194641	0.28923
COX15	1.44337	0.855343	2.43565	0.169237
CCDC88C	1.153632	0.773431	1.720732	0.483575
YAF2	0.790664	0.469789	1.330704	0.376532
ZMYND11	0.953947	0.694607	1.310116	0.770853
WAS	1.988237	1.402118	2.819367	0.000115
DPEP1	1.155051	0.969748	1.375761	0.10617
BID	1.593981	0.999143	2.542953	0.050422
MATR3	0.548313	0.368818	0.815164	0.002977
NPC1L1	1.362638	0.730512	2.541757	0.330667
XYLT2	0.830223	0.546627	1.260953	0.382897
NUDCD3	0.83879	0.504785	1.393798	0.497466
GLT8D1	0.832237	0.550334	1.258544	0.384172
ATP2C1	0.696168	0.469955	1.03127	0.070858
SLC38A5	1.198173	0.890975	1.611289	0.23162

RALBP1	0.92849	0.593511	1.452531	0.74521
RUFY3	0.659621	0.512247	0.849395	0.001259
SLC11A1	1.10616	0.996408	1.228002	0.058429
WWTR1	1.062932	0.772621	1.462328	0.707672
AGPS	0.928975	0.664687	1.298345	0.666219
TTC27	0.915668	0.658102	1.274039	0.601118
ZNF582	0.986295	0.613098	1.586661	0.954634
VSIG2	1.206224	1.030139	1.412408	0.019871
PHLDB1	0.848924	0.59069	1.22005	0.376091
MARCO	1.079641	0.962207	1.211407	0.192152
PRDM11	0.856391	0.632023	1.16041	0.317235
CD74	1.349687	1.139216	1.599042	0.000527
HGF	0.803982	0.721099	0.896392	8.48E-05
ZRANB1	0.646779	0.402384	1.039612	0.071934
NCDN	1.38556	0.941168	2.039782	0.098397
ZFP64	0.956546	0.554493	1.650119	0.873125
MNAT1	0.462585	0.268883	0.795826	0.005353
SAMD4A	1.250089	0.942897	1.657363	0.120824
RUNX3	1.167539	0.982819	1.386976	0.077944
PLEKHB1	1.128638	0.776756	1.639926	0.525574
SERPINB1	0.824693	0.617484	1.101434	0.191704
SPAST	0.752875	0.446487	1.269512	0.286965
OSBPL5	0.846009	0.674941	1.060434	0.146818
AQR	0.89534	0.539011	1.487228	0.669393
FHL1	1.242384	1.065501	1.44863	0.005612
NLRP2	1.13235	0.989621	1.295665	0.070577
SLC45A4	1.31242	0.982061	1.753909	0.066119
RNF10	1.31896	0.796902	2.183021	0.281535
ZNF839	0.635302	0.371557	1.086264	0.097394
ZDHHC6	1.259972	0.65189	2.435272	0.491876
GRAMD1B	1.162118	0.957225	1.410867	0.128961
RNH1	1.11671	0.833853	1.495516	0.458861
NDUFS1	0.86854	0.523928	1.439819	0.584712
RB1CC1	0.718569	0.485398	1.06375	0.098698
ERP44	1.470219	0.830937	2.601332	0.18556
ALAS1	1.162772	0.797659	1.695008	0.432881
BIRC3	1.185498	0.902702	1.556888	0.221034
AKAP11	0.842246	0.598107	1.18604	0.325594
GLRX2	0.736215	0.416023	1.302844	0.293006
SNAPC1	0.818358	0.528774	1.266532	0.368338
DERA	0.862196	0.575987	1.290622	0.471275
STRAP	1.094489	0.647538	1.84994	0.736001
ABCC2	0.965551	0.596356	1.563309	0.886614
DEF6	1.9073	1.215076	2.993879	0.005004
PLEKHO1	1.333909	0.974404	1.826054	0.072155
GCLM	1.216014	0.944057	1.566315	0.129964
UBR2	0.747377	0.459085	1.216709	0.241563
EHD2	1.062854	0.93375	1.209808	0.356241
DEPDC1	0.947987	0.732958	1.226098	0.684036
CCDC28A	1.411923	0.764433	2.607848	0.270506
RRAGD	0.750557	0.590305	0.954314	0.019203
HSF2	0.623197	0.421159	0.922157	0.018015
PHF20	0.878494	0.503291	1.533408	0.648523
NR1H3	1.03642	0.812755	1.321635	0.773032
TYMP	1.137895	0.988275	1.310166	0.072496
NCAPH2	1.439288	0.95562	2.167756	0.081383
TOMM34	1.606685	0.987432	2.614293	0.056256
SEC63	0.795728	0.481645	1.314627	0.372374

KPNA6	1.462568	0.65807	3.250576	0.350796
VIM	1.098811	0.90105	1.339977	0.351975
FAS	0.956253	0.659535	1.386461	0.813425
RNASET2	0.990299	0.706351	1.388391	0.954905
CD44	0.80007	0.622938	1.027569	0.080643
AGPAT4	1.107914	0.895685	1.370429	0.344887
SLAMF7	0.995607	0.817771	1.212117	0.965025
BTN3A1	0.912091	0.673668	1.234894	0.551707
MIPEP	1.241831	0.751979	2.050781	0.39742
PRKCH	1.158478	0.947426	1.416545	0.151674
IFNGR1	1.381254	1.093252	1.745127	0.006784
B4GALT7	0.976721	0.633335	1.506284	0.91513
SH2D2A	1.287254	1.01838	1.627115	0.034659
VRK2	1.275284	0.781605	2.080781	0.330304
TNFRSF1B	1.197713	1.057608	1.356377	0.004478
VEZT	0.859912	0.546309	1.353535	0.514357
POU2F2	1.240076	1.048811	1.46622	0.011817
BRD9	1.250945	0.675953	2.315047	0.475885
SNX1	1.002505	0.586511	1.713551	0.992702
TBPL1	0.897599	0.5421	1.486228	0.674567
ARNTL2	1.221792	0.961106	1.553186	0.101844
BCLAF1	0.712609	0.460465	1.102824	0.128338
SLC39A9	1.098472	0.64537	1.869685	0.729259
ANK1	0.95418	0.838259	1.086132	0.477872
TFB1M	1.417569	0.814342	2.46764	0.217278
RABEP1	0.729008	0.484588	1.09671	0.129287
HMGB3	0.815801	0.650104	1.023729	0.078831
NUP160	0.913807	0.596614	1.399637	0.678608
BAK1	2.25285	1.445206	3.511839	0.000336
IKZF2	0.885316	0.770521	1.017214	0.085598
GRN	1.047153	0.894592	1.225731	0.566306
FAM13B	0.658325	0.431211	1.005055	0.052793
CENPQ	0.875948	0.60308	1.272278	0.486753
RANBP3	1.26752	0.659389	2.436512	0.477092
ARID4A	0.773701	0.472722	1.266312	0.307406
PNPLA6	1.676777	1.264464	2.223535	0.000331
IFT88	0.675015	0.424277	1.073935	0.097138
ALG1	1.61193	0.934811	2.779512	0.085894
ZCCHC8	0.528602	0.270893	1.03148	0.06161
ABCF2	0.895122	0.542839	1.476023	0.664156
CHPF2	0.99352	0.684622	1.441791	0.972704
LRRC7	1.242731	0.529611	2.916064	0.617521
FUT8	0.758303	0.538645	1.067536	0.112862
UBA6	0.454285	0.280608	0.735456	0.001327
GAB2	0.701041	0.512887	0.95822	0.025906
ATP6V0A1	1.14207	0.9148	1.425801	0.240639
PIAS1	0.835683	0.463363	1.507168	0.550789
SLC4A7	0.813319	0.664782	0.995045	0.044618
APBA2	1.098255	0.909543	1.326121	0.3299
MAP2K3	1.640633	1.113199	2.417966	0.012353
TMSB10	1.240215	0.889258	1.729681	0.204641
ASTE1	0.696795	0.409819	1.184724	0.182197
RNF19A	0.877396	0.558995	1.377155	0.569592
PEX3	0.700181	0.489103	1.002353	0.05152
GABARAPI	1.355036	0.696004	2.638093	0.371415
SH3YL1	0.571262	0.39763	0.820713	0.002456
FAM136A	1.102283	0.66998	1.813529	0.701457
VCL	1.620959	1.203153	2.183852	0.001493

DEPDC1B	1.029114	0.797296	1.328335	0.825574
DAPK2	1.195098	0.905726	1.576923	0.207683
NSMAF	0.678461	0.435142	1.057837	0.086924
STAP1	0.919245	0.758515	1.114033	0.390502
TIMP2	0.958239	0.716236	1.282011	0.773938
RFC1	0.932915	0.632801	1.375362	0.725861
TBC1D23	0.810631	0.472034	1.392108	0.446701
CUL3	0.803165	0.454234	1.420138	0.450984
MYOM2	1.054416	0.779061	1.427094	0.731491
CYP46A1	1.101774	0.915786	1.325536	0.304226
ZZZ3	0.789083	0.506164	1.230137	0.295717
USP2	1.283092	0.921961	1.785678	0.139367
TUBG2	1.310818	1.020177	1.68426	0.034332
RPL26L1	1.522879	0.908744	2.55205	0.110333
FLT4	0.811714	0.660907	0.996933	0.046673
NSUN2	1.446289	0.876375	2.386824	0.148829
FBXO42	1.031399	0.54605	1.948144	0.924092
MFAP3	0.856449	0.586299	1.251074	0.422875
MRI1	1.183577	0.812436	1.724263	0.379975
METTL1	1.244484	0.812866	1.905282	0.314168
AGA	0.876324	0.598977	1.282092	0.496496
PI4K2B	1.275368	0.841343	1.933296	0.251789
MAT2B	0.845897	0.546942	1.308259	0.451911
EDC4	1.103247	0.777942	1.564582	0.581468
TRIO	0.977957	0.778188	1.229009	0.848375
VCAN	1.047505	0.972425	1.128382	0.221301
CLEC16A	1.52688	0.867833	2.686419	0.142049
MSR1	1.151595	1.001552	1.324116	0.047508
CDH1	0.925935	0.751141	1.141405	0.470969
ZFYVE16	0.537286	0.37906	0.761557	0.000482
RAI14	1.133686	0.86639	1.483446	0.360413
PNKP	1.285093	0.814167	2.028408	0.281427
BEST2	0.611244	0.301325	1.239921	0.172548
PHLPP2	1.056282	0.75109	1.485483	0.752967
STAU2	0.581181	0.36093	0.935835	0.025561
CTNS	1.481594	0.929508	2.361593	0.098401
RTN4R	0.839118	0.716826	0.982274	0.029073
PHF23	1.037606	0.642208	1.676447	0.880122
INPP4A	1.144428	0.783528	1.671561	0.485227
RAB27B	1.246875	1.085882	1.431736	0.00176
PSMA4	1.669763	0.96093	2.901469	0.068973
MYO16	1.100126	0.772472	1.56676	0.596839
LSG1	0.890673	0.553598	1.432986	0.633229
PARP3	1.856059	1.42855	2.411505	3.65E-06
TNC	0.998089	0.822993	1.210438	0.984494
THAP3	1.374145	0.776511	2.431743	0.275101
TDP1	0.921007	0.559667	1.515641	0.746109
AIFM2	1.85184	1.363762	2.514597	7.89E-05
SPATA7	0.522412	0.330816	0.824972	0.005347
MED17	0.603379	0.363232	1.002296	0.051044
RETSAT	1.495264	0.924503	2.418396	0.101012
CAPG	1.102947	0.942539	1.290655	0.22172
AP2S1	1.571127	1.04253	2.367741	0.03085
TG	1.812477	0.978457	3.3574	0.058661
ADAM28	1.190018	1.007026	1.406261	0.041136
DCUN1D1	0.81652	0.483216	1.379723	0.448843
LCP2	1.406649	0.892655	2.216602	0.14141
TRIT1	0.739992	0.406485	1.34713	0.324567

ADRB1	1.170022	0.995734	1.374815	0.056388
CUL7	0.941056	0.637433	1.3893	0.759858
CTNNA1	1.105268	0.870354	1.403586	0.411657
PHKA2	1.124154	0.773421	1.633939	0.539633
CNTLN	1.350776	0.989179	1.844557	0.058555
HSPA5	0.87901	0.675748	1.143414	0.336488
DSG2	1.032394	0.910496	1.170612	0.618978
GEMIN8	0.892101	0.565316	1.407787	0.623753
OFD1	1.575708	1.012962	2.451086	0.043686
GPM6B	1.04869	0.817048	1.346006	0.70891
PREX2	1.058373	0.828937	1.351314	0.649055
WDR37	1.168763	0.716586	1.90627	0.532109
YTHDC2	0.640101	0.412784	0.9926	0.046245
CTPS2	0.852658	0.622067	1.168725	0.321779
ATP6V1H	2.153376	1.173853	3.950263	0.013221
POLR2B	0.938539	0.585542	1.504343	0.792156
ARAP2	1.005483	0.790649	1.278692	0.964438
TPR	0.925463	0.628423	1.362906	0.694894
CP	0.897708	0.68579	1.17511	0.432188
DTNBP1	0.746133	0.427222	1.303103	0.303304
XK	0.939793	0.810841	1.089253	0.409582
SCML1	0.967619	0.766336	1.221772	0.782064
WWC3	0.974064	0.78483	1.208925	0.811544
ARHGAP6	1.259218	1.011741	1.567228	0.038969
FAM184B	0.455345	0.214379	0.967163	0.040674
MAP4	0.831122	0.517823	1.333976	0.44352
GOPC	0.754959	0.502889	1.133379	0.175105
USP28	0.985293	0.669349	1.450367	0.940128
HDAC9	1.151998	0.973488	1.363242	0.099521
TSPAN17	1.219804	0.894874	1.662716	0.208691
NOP16	1.082076	0.727686	1.609058	0.696787
CC2D2A	0.958536	0.740119	1.241412	0.748239
RRM2B	1.000674	0.651193	1.537715	0.997547
ZNF800	0.708982	0.448057	1.121854	0.141865
TNFRSF17	0.927965	0.722706	1.191521	0.557785
SNX29	1.049568	0.708971	1.55379	0.809019
MRPS10	1.408548	0.759741	2.611426	0.27678
RSF1	0.86757	0.534973	1.406946	0.564691
VPS13D	0.911838	0.615636	1.350551	0.645151
FAM120A	1.635038	0.963229	2.775403	0.068578
R3HDM1	0.898936	0.50951	1.586004	0.713023
COL9A2	1.152825	0.966	1.375782	0.114909
KITLG	1.135524	0.828309	1.556683	0.429743
ERCC8	0.710234	0.437807	1.152179	0.165712
ADAMTS6	1.088413	0.847162	1.398368	0.507554
H6PD	0.85195	0.609418	1.191002	0.348567
VAMP3	1.437078	0.909743	2.270084	0.120075
PER3	0.992423	0.790117	1.24653	0.947864
UTS2	1.017241	0.766817	1.349447	0.905629
TNFRSF9	1.318079	0.867858	2.00186	0.195229
LTBP1	0.961723	0.849903	1.088255	0.536001
RCN1	0.662502	0.478504	0.917253	0.013128
ELN	1.148845	0.961891	1.372135	0.125719
RFC2	1.598049	0.959136	2.662564	0.071895
ARID1B	0.713714	0.483069	1.054482	0.090345
CLPTM1L	1.106212	0.644423	1.898915	0.714258
NEDD4L	0.985766	0.723587	1.342941	0.927591
FOXP3	1.53703	0.974782	2.42358	0.064308

PPP1R3F	1.116936	0.614819	2.029129	0.716562
HEXB	1.131684	0.881797	1.452386	0.331156
PTCD2	0.977491	0.642947	1.486109	0.915175
DKK3	0.56295	0.045287	6.997871	0.654987
ARHGEF5	1.742903	1.251955	2.426375	0.000998
NFE2L3	1.119744	0.834777	1.50199	0.450382
LIMA1	1.367901	0.986865	1.896058	0.060027
LETMD1	1.153964	0.634712	2.098012	0.638698
SLC4A8	1.330616	0.93421	1.895227	0.113456
LAMC3	0.640004	0.461114	0.888296	0.007628
PTGER3	0.826243	0.653946	1.043935	0.109684
MAPK9	1.073005	0.597711	1.926246	0.813408
COL23A1	0.909659	0.820565	1.008426	0.071796
BCAR1	1.225755	0.609212	2.466262	0.56824
FAM160A2	1.095501	0.752801	1.594209	0.633707
HERPUD1	1.046828	0.784685	1.396547	0.755654
HOMER3	0.882336	0.75717	1.028194	0.108767
RAD51	1.507681	1.014908	2.239714	0.042028
POLQ	1.012351	0.759091	1.350109	0.933399
PIK3CB	0.693257	0.499769	0.961655	0.028226
CYBA	1.352976	1.016596	1.800659	0.038188
THOC3	1.536503	0.889184	2.655064	0.123781
HEBP2	1.503161	0.967626	2.335089	0.069751
MPHOSPH	0.718801	0.474829	1.088127	0.118588
PLEKHA5	0.714667	0.573837	0.890059	0.002699
PRSS8	0.85463	0.442892	1.649143	0.639514
RRP12	1.064285	0.842944	1.343745	0.600468
FNIP2	1.274103	0.987107	1.644541	0.062842
TTC17	0.794007	0.508518	1.239773	0.310303
ALX4	0.518258	0.265168	1.01291	0.05455
FOXN3	1.129784	0.755821	1.688774	0.551858
AKR7A2	1.7994	1.262616	2.564391	0.001154
MRTO4	1.466964	0.944998	2.277236	0.087668
NNAT	0.924467	0.599862	1.424725	0.721917
USE1	1.333029	0.808602	2.197577	0.259734
MCF2L2	1.113562	0.621572	1.994973	0.717672
NRIP2	0.738353	0.501433	1.087214	0.124434
LAMA3	1.586432	1.211522	2.07736	0.000794
ANAPC4	0.71267	0.427774	1.187305	0.193358
KCNQ1	1.273931	1.077495	1.506179	0.004604
TRAPPC3	3.72678	1.578657	8.797917	0.002684
THRAP3	1.269722	0.675039	2.388297	0.458804
PHPT1	1.002161	0.735748	1.365042	0.989075
ENTPD2	1.452812	0.739885	2.852688	0.277966
LY75	1.093858	0.778734	1.536499	0.604837
ARID4B	0.850553	0.532626	1.358251	0.497896
OPN3	1.284085	0.88455	1.864081	0.188553
SDCCAG8	0.712242	0.463539	1.09438	0.121519
HHAT	1.126323	0.829582	1.529207	0.445785
KIF1B	1.118409	0.900788	1.388604	0.310776
FOXC1	1.023904	0.918476	1.141434	0.670047
TBC1D22A	1.435375	0.847151	2.432035	0.17914
SYNE2	1.016964	0.821615	1.258758	0.877165
PLEKHH1	1.069813	0.672022	1.70307	0.776046
ATP9A	0.839668	0.71001	0.993003	0.04115
FAM168A	1.409466	0.979574	2.028019	0.064487
RELT	1.393848	1.116245	1.74049	0.003385
GALC	0.932378	0.774988	1.121733	0.457953

NOP58	0.953787	0.624271	1.457234	0.826812
KCNH2	0.949326	0.82979	1.086082	0.448842
CUL1	0.701684	0.509312	0.966718	0.030234
FAM114A2	0.991635	0.607101	1.619731	0.973231
CYFIP2	1.161041	0.932186	1.44608	0.182519
EIF2AK2	1.244004	0.893269	1.732453	0.196342
USP36	0.951729	0.644956	1.404418	0.803193
PUM2	0.571995	0.346088	0.94536	0.02932
MRPL43	1.620859	0.857487	3.063817	0.1371
ITIH4	1.001045	0.496714	2.017441	0.997668
ZFR	0.861062	0.525555	1.410752	0.552615
ZNF280C	0.584478	0.383621	0.890499	0.012427
TRAF1	1.139159	0.858383	1.511777	0.366864
RC3H2	0.982612	0.653898	1.476573	0.932728
IL17RB	0.800556	0.53255	1.203437	0.284811
TRAF3IP2	0.69473	0.481751	1.001864	0.051177
GYG2	0.818087	0.659213	1.01525	0.068367
DCBLD2	0.712224	0.543082	0.934046	0.01416
SOAT1	1.149669	0.832335	1.587991	0.397361
PKP2	1.122253	0.90996	1.384074	0.281014
MSH4	0.638532	0.365349	1.11598	0.115315
GDI2	0.906838	0.611039	1.345832	0.627339
PRDM1	1.26972	0.942537	1.710479	0.116252
ATG5	0.962478	0.501145	1.848495	0.90856
TMCC3	1.071394	0.837197	1.371105	0.583712
MTA3	0.96123	0.759726	1.216179	0.741831
USP13	0.736203	0.538318	1.006831	0.055195
ATP11B	1.045992	0.736055	1.486436	0.801979
SEC61A1	1.353703	0.794844	2.305499	0.264949
PPP1R12A	0.813371	0.466569	1.417953	0.466331
CROCC	1.231375	0.941262	1.610906	0.128925
POLR3E	0.820277	0.530228	1.268992	0.373521
ATP2B4	1.002124	0.764981	1.312782	0.987713
ZC3H11A	0.142696	0.039321	0.517845	0.00307
RIOK2	0.772413	0.497615	1.198961	0.249687
YIPF1	1.494822	0.822626	2.716291	0.187094
DGKG	0.760446	0.593878	0.973733	0.029933
FLYWCH1	1.220655	0.883955	1.685605	0.225946
UNKL	0.923134	0.626945	1.359253	0.685366
TBXAS1	1.003566	0.763837	1.318535	0.979607
PARP12	1.143312	0.847073	1.543152	0.381418
ALDH18A1	0.926979	0.668659	1.285095	0.649144
TARBP1	0.958605	0.719908	1.276445	0.772306
MXD1	0.834252	0.649711	1.071211	0.155409
DNAJC25	0.824534	0.506975	1.341003	0.436853
SLC2A3	0.928097	0.782561	1.100699	0.391196
PSD	1.052511	0.705589	1.570009	0.801942
CTDP1	0.792728	0.556329	1.12958	0.19859
STYK1	0.866494	0.715693	1.049069	0.141854
WNK1	0.967098	0.650569	1.437632	0.868634
CCAR1	0.940416	0.51795	1.707467	0.840015
OGFR	1.685029	1.191067	2.383847	0.003201
GNA15	0.899389	0.725386	1.115133	0.33374
CREB3L3	1.044287	0.797277	1.367826	0.752987
PIGV	0.953255	0.620869	1.463585	0.826777
PTPRU	1.039538	0.727308	1.485808	0.831501
SNRNP40	2.055324	1.025144	4.120745	0.042363
COL11A1	1.209228	0.847444	1.725464	0.294923

QSER1	0.895551	0.617402	1.29901	0.561002
ACAA1	1.290572	0.848641	1.962639	0.233012
BCAT1	1.122013	0.976085	1.289758	0.105348
HDAC7	1.574772	1.098753	2.257018	0.013407
LZTS1	0.496238	0.258493	0.952645	0.035225
SPAG4	0.790899	0.54996	1.137392	0.205699
NCKAP1	1.277355	0.858491	1.900586	0.227281
MRPS35	1.148111	0.710908	1.854191	0.572237
TNK2	1.131405	0.817697	1.565467	0.456163
MON2	0.578489	0.371381	0.901096	0.015498
GPBP1	0.701533	0.426797	1.153122	0.162094
DGAT2	1.259879	0.990419	1.602651	0.059896
CS	1.988833	0.984987	4.015744	0.05514
LTK	0.830199	0.732475	0.940961	0.003587
MRPS24	0.848107	0.434955	1.653701	0.628701
ELMO2	1.083415	0.712683	1.646998	0.707724
APPBP2	0.703835	0.441088	1.123093	0.140733
POLD1	1.175888	0.853585	1.619887	0.321518
SEZ6	0.848974	0.593979	1.213438	0.368965
EIF4B	0.97671	0.674263	1.414822	0.900808
SLC6A16	1.538953	1.095585	2.161746	0.012901
SPHK2	1.100326	0.688443	1.75863	0.68945
RPL18	1.127592	0.748227	1.699302	0.566059
CA11	1.043492	0.760173	1.432405	0.792241
ISOC2	1.15868	0.806751	1.66413	0.425235
U2AF2	1.343094	0.83831	2.15183	0.219979
EPN1	1.648697	1.111683	2.445123	0.012901
MED29	2.215884	1.207348	4.066883	0.010224
ZNF275	1.754892	1.162976	2.648073	0.007379
MTMR1	1.617429	1.041481	2.51188	0.03228
GPC1	1.140737	0.862228	1.509208	0.356529
ADCK1	1.371713	0.858712	2.191186	0.185979
HAGH	1.132876	0.737244	1.740816	0.569224
RNF4	1.296594	0.648412	2.592728	0.462559
CASP8	1.39329	0.771148	2.517362	0.271804
TM7SF3	0.73045	0.567698	0.939861	0.014597
DLX3	0.518444	0.23986	1.120588	0.09483
SPA17	0.881929	0.489716	1.588266	0.675508
TSPAN32	1.194737	0.997031	1.431647	0.053886
ST3GAL6	0.796905	0.644175	0.985846	0.036503
ATP2C2	1.358681	0.709567	2.601606	0.355075
NGFR	1.094228	0.853987	1.402051	0.476474
TAF2	0.792072	0.450009	1.394144	0.419046
HIPK2	1.119942	0.859659	1.459033	0.401244
TNPO3	0.687532	0.449518	1.051569	0.083982
RFXANK	1.709977	1.066562	2.741539	0.025912
TMEM161/	1.178661	0.816821	1.700791	0.379644
LPAR2	1.111116	0.844255	1.462329	0.452131
CTSA	1.087913	0.833316	1.420297	0.535616
SLC12A2	0.733982	0.551333	0.977141	0.034145
SNX24	1.202962	0.939641	1.540075	0.142639
CNN2	0.957372	0.754549	1.214713	0.719863
ABCA7	1.255845	1.005509	1.568504	0.044601
DDX20	1.059468	0.626447	1.791807	0.829399
BTBD1	1.095694	0.682667	1.75861	0.705004
FAR2	1.177937	0.835233	1.661255	0.350522
POU1F1	0.773706	0.423973	1.411932	0.403172
CHI3L2	1.03875	0.709077	1.521699	0.845267

SBNO2	1.270415	0.932603	1.73059	0.129127
PMS1	0.884034	0.550277	1.420223	0.610337
HMG20B	1.315996	0.857146	2.020481	0.209374
CALCRL	1.337461	1.158689	1.543817	7.13E-05
TAF11	0.966503	0.448344	2.083507	0.930722
ANKS1A	0.818992	0.583748	1.149037	0.247753
AP3D1	1.3387	0.818787	2.188748	0.244868
ZNF76	1.030381	0.67374	1.575809	0.890183
SLC9A3R2	1.07469	0.909754	1.269528	0.396795
NTHL1	1.166017	0.840125	1.618326	0.358428
UHRF1BP1	0.828326	0.520454	1.318317	0.426969
GNAI3	1.223428	0.719946	2.079012	0.456028
IPO5	1.05539	0.746504	1.492087	0.760252
OAT	1.168532	0.935943	1.458922	0.169018
WDR3	0.981338	0.677948	1.420498	0.920478
PKN2	0.645737	0.408213	1.021467	0.061596
WDR18	1.485048	1.038917	2.122755	0.030051
TRAM2	0.983164	0.654392	1.477114	0.934843
NTN1	1.075935	0.888358	1.303119	0.453974
MCM10	1.147279	0.857504	1.534977	0.354972
DGKA	1.622544	1.133662	2.322252	0.008151
ERBB3	0.510073	0.083851	3.10281	0.464907
ANKRD44	0.860613	0.625262	1.184551	0.35709
ADAT1	0.993131	0.545379	1.808483	0.982019
PDIA5	0.884549	0.653801	1.196735	0.426353
TBC1D22B	1.667189	1.010496	2.750649	0.04541
NDUFB4	1.084594	0.59718	1.969832	0.789689
SPEN	0.974865	0.596542	1.593116	0.919085
MYLK	1.186418	0.964449	1.459473	0.105781
ZC3H15	1.019098	0.620099	1.674829	0.940506
MAP2K4	0.792264	0.480628	1.305965	0.36116
SLK	1.055943	0.744788	1.49709	0.759895
CYB5R4	0.880876	0.601518	1.289973	0.514592
COL17A1	0.966991	0.641282	1.458129	0.872742
GSTO2	1.219219	0.741906	2.003614	0.434176
SEC61A2	1.056005	0.733663	1.519971	0.769324
PRKCQ	0.989672	0.784975	1.247749	0.93003
TLE2	0.7582	0.52591	1.09309	0.138054
ASB1	1.252169	0.811729	1.931589	0.309245
FAM107B	1.114073	0.890174	1.394288	0.345341
ME1	0.997739	0.857979	1.160265	0.976546
TBC1D1	1.45051	1.055662	1.993043	0.021785
MTHFD2	0.828657	0.553397	1.240831	0.361546
SLC9A7	1.201784	0.994471	1.452315	0.057096
FOXJ2	0.899848	0.517249	1.565448	0.708741
YBX1	1.196038	0.73785	1.93875	0.467608
PDE4A	1.173795	0.987872	1.394709	0.068564
PPP2R5A	1.374199	0.822824	2.295053	0.224469
ELAVL1	0.848808	0.429597	1.677093	0.637076
TIE1	1.017414	0.850835	1.216607	0.849902
DIP2B	0.903707	0.618333	1.320785	0.601009
SMARCD1	1.204752	0.757753	1.915435	0.431055
NFYC	0.915166	0.504496	1.660132	0.770479
ZMYND12	0.871976	0.594247	1.279506	0.483806
SLC9A3	0.697611	0.511081	0.95222	0.023306
NGEF	0.798508	0.42453	1.501934	0.485137
ASPM	0.86027	0.680789	1.087068	0.207421
CD84	1.117503	0.91671	1.362278	0.2716

ELOVL1	2.140014	1.312426	3.489461	0.00229
SPI1	1.362742	1.041923	1.782344	0.023832
MPPED2	1.000361	0.722883	1.38435	0.998261
CLDN18	0.646737	0.34357	1.21742	0.176894
ZBTB11	0.66204	0.422293	1.037899	0.072207
ATXN3	0.510633	0.295922	0.881131	0.015752
GOLGA5	0.941701	0.59724	1.484831	0.795991
LRRC40	1.025898	0.598804	1.757614	0.92584
ISOC1	0.722085	0.475205	1.097226	0.12718
TRMT11	0.952327	0.559283	1.621588	0.857253
THUMPD1	0.658758	0.47883	0.906296	0.010334
KIF26A	0.710516	0.572857	0.881257	0.001868
ATG2B	0.716539	0.486582	1.055173	0.091412
ARFGEF1	0.451645	0.308538	0.661128	4.34E-05
ZFAT	1.475732	0.981632	2.218534	0.061367
MTFR1	0.605861	0.388111	0.945779	0.027433
STAG3	0.632573	0.451805	0.885666	0.007652
FECH	0.982977	0.751893	1.285082	0.900072
MYO9A	0.622568	0.419219	0.924554	0.018837
DDX3Y	0.986319	0.92435	1.052442	0.677342
PFKP	1.299882	0.976828	1.729776	0.071997
IDI1	1.637795	1.098976	2.440793	0.015367
SP100	1.82398	1.10043	3.023275	0.019745
KLF6	0.923062	0.692456	1.230467	0.585155
NEO1	1.110587	0.929311	1.327222	0.24865
TRAM1	0.906824	0.562688	1.461432	0.68791
PHKA1	1.089351	0.928107	1.278608	0.295047
TNFRSF1A	1.134556	0.701813	1.834132	0.606466
CACNB1	1.359471	1.078295	1.713965	0.009388
EVI5	1.009269	0.785577	1.296657	0.942466
STOML1	0.895519	0.514917	1.557445	0.695923
DHX29	1.107399	0.694616	1.765483	0.668152
DNTTIP2	1.161569	0.760764	1.773538	0.487914
TP53BP1	0.710722	0.518025	0.9751	0.034325
TRO	1.113734	0.957799	1.295057	0.161604
RRP15	0.809204	0.54445	1.202702	0.295061
RHOA	1.852598	1.099889	3.120422	0.020456
DHX8	1.181419	0.717621	1.944971	0.512183
PRKCZ	1.142022	0.895358	1.45664	0.284769
ZFY	0.980857	0.885008	1.087086	0.712565
IARS2	1.308991	0.84575	2.025963	0.226966
NAV3	0.843245	0.696019	1.021614	0.081589
IDH3G	1.552458	1.056406	2.28144	0.025134
ROGDI	1.100284	0.891768	1.357556	0.372685
PDZD4	1.063393	0.842697	1.341887	0.604534
ROCK1	0.896513	0.575558	1.396447	0.629003
CBFB	1.229252	0.812403	1.859988	0.328678
PDK3	0.952707	0.661356	1.37241	0.794755
HYAL2	0.860669	0.626502	1.182361	0.354406
HDAC4	0.936547	0.701012	1.25122	0.657366
RASSF1	0.763583	0.502401	1.160545	0.206635
FGFR3	0.891985	0.715613	1.111825	0.309192
IFI35	1.779256	1.240008	2.55301	0.001762
HEATR6	1.156419	0.729825	1.832366	0.536023
COASY	1.608169	0.965616	2.678299	0.067922
PLEKHH3	1.124344	0.890538	1.419534	0.324465
MEF2A	0.853566	0.639512	1.139267	0.282445
OTUD5	1.851369	1.082604	3.166038	0.024456

TFE3	1.696574	1.188555	2.421732	0.003599
TBC1D25	1.056515	0.709727	1.572753	0.78652
ACSL4	1.188846	0.835543	1.691539	0.336356
INPP5A	1.184352	0.832693	1.684524	0.346536
GPKOW	2.120778	1.166214	3.856668	0.013743
GRIPAP1	1.199225	0.715907	2.008837	0.490048
FTSJ1	2.224586	1.231026	4.020046	0.008087
PRR11	1.188372	0.889442	1.587768	0.243035
ATP11A	0.809094	0.611551	1.070449	0.137999
POLR1A	1.098208	0.720818	1.673185	0.662781
LAPTM4A	0.870098	0.527571	1.43501	0.58568
TTC7A	0.905515	0.640813	1.279558	0.573706
IP6K2	0.802233	0.494918	1.300373	0.371231
SRBD1	0.854764	0.553713	1.319495	0.478691
KIF2A	0.792212	0.550356	1.140352	0.2101
RASGRP2	1.207004	0.863464	1.687226	0.270927
PSME4	0.909599	0.561635	1.473144	0.700112
IFT80	1.274887	0.907824	1.790365	0.160981
SIRT2	1.676464	1.085852	2.588319	0.019719
PPP2R5B	1.354133	0.949086	1.932044	0.094565
PYGM	0.876232	0.598212	1.283464	0.497481
PITX1	1.214171	1.045186	1.410479	0.011151
MAST4	1.075155	0.869965	1.328743	0.502423
SDK2	1.004661	0.900692	1.120631	0.933511
NUP133	0.941132	0.607732	1.457435	0.785699
NUCKS1	1.059526	0.716784	1.566155	0.771826
VPS35	1.009583	0.62419	1.632928	0.96899
DNAJA2	1.14822	0.645622	2.042078	0.637997
BCL3	1.261234	1.020682	1.558479	0.03159
KCNAB2	1.612768	1.122662	2.316834	0.00971
GAL	1.047236	0.720264	1.52264	0.809025
CLEC2D	0.953045	0.710232	1.27887	0.748561
FUNDC1	1.390767	0.840629	2.300933	0.199099
RORA	0.881477	0.665052	1.168333	0.380137
DRD4	0.967863	0.745931	1.255826	0.805835
TGFBR3	1.148757	0.88589	1.489624	0.295539
PLA2G10	0.910035	0.63299	1.308335	0.610773
ATP1B3	1.38858	0.949452	2.030806	0.090544
NEDD4	0.833213	0.665314	1.043483	0.112006
PIGB	0.736197	0.408705	1.326106	0.307745
MAPK6	0.846614	0.54421	1.317055	0.460203
GNB5	1.000448	0.777514	1.287302	0.997224
RAB27A	0.816746	0.629975	1.058889	0.126504
LRP6	0.966774	0.844249	1.107081	0.625053
SCT	0.949044	0.745064	1.208868	0.67185
PHRF1	1.183325	0.744868	1.879873	0.475998
NUCB2	0.639655	0.498526	0.820737	0.000443
PFN2	1.001294	0.693209	1.446303	0.994501
SPTB	0.931594	0.796268	1.089918	0.376263
DAPP1	1.266685	1.01405	1.582261	0.037261
SLC44A1	1.21879	1.031942	1.439469	0.019792
SMG6	0.795227	0.525461	1.203488	0.278445
EXOC5	0.724536	0.467288	1.123402	0.149878
CLTCL1	0.91721	0.807708	1.041558	0.182778
FGF22	0.565744	0.247646	1.292434	0.176577
FSTL3	0.73237	0.568793	0.942989	0.01573
DGCR2	1.038389	0.668927	1.611911	0.866665
RNF126	1.632436	1.123729	2.371433	0.010104

MNT	1.191022	0.755867	1.876699	0.45114
ZXDC	1.044011	0.671723	1.62263	0.848191
JMJD6	0.993667	0.707786	1.395017	0.970721
POLB	0.846684	0.53	1.352593	0.486228
ST6GALNA	1.043246	0.769315	1.414716	0.785293
WIPI1	0.932015	0.731632	1.18728	0.56864
GBA2	1.410789	0.932788	2.133736	0.103027
NDST1	0.805382	0.592504	1.094744	0.166977
ASNS	0.672308	0.457188	0.988647	0.043592
AP3M2	0.852494	0.578993	1.255189	0.418802
ST6GALNA	0.914078	0.650815	1.283834	0.604207
PABPC1	1.227282	0.772371	1.950127	0.386057
TESK2	1.214208	0.725497	2.032124	0.460101
CSNK2A2	0.722439	0.409606	1.274196	0.261441
EIF2B3	1.12348	0.692418	1.8229	0.637289
CAMK2A	11.91446	3.807951	37.27842	2.07E-05
TCOF1	1.393366	0.859055	2.260004	0.178851
CDC42	1.369811	0.780371	2.404474	0.273022
OSBPL3	1.14734	0.878873	1.497816	0.312202
SLC12A3	1.657228	1.163696	2.360072	0.005104
RAD18	0.897641	0.583138	1.381763	0.623661
ATP2B1	0.989519	0.745444	1.313509	0.941877
TRPM5	1.771706	0.887405	3.537214	0.104945
NCK2	1.176599	0.770743	1.796168	0.451159
MAP4K4	0.6323	0.466099	0.857764	0.003219
MGAT4A	0.811035	0.618996	1.062653	0.128716
RPL31	0.991212	0.659047	1.490789	0.966188
WDR1	1.952314	1.201167	3.173187	0.006943
SNX13	0.837019	0.57003	1.229058	0.364043
ARHGAP1(	0.775858	0.627853	0.958753	0.018773
RPS6KA2	0.828559	0.703062	0.976457	0.024815
ING3	0.775849	0.504662	1.192763	0.247422
VASH1	1.295009	0.977343	1.715927	0.071804
SEL1L	0.726063	0.485301	1.08627	0.119379
TRIP13	1.192612	0.857061	1.659535	0.29605
ATP6AP1	1.41359	0.95959	2.082387	0.079901
TCF3	0.880741	0.647766	1.197508	0.417866
TRIB2	0.871461	0.716924	1.05931	0.167143
DAZAP1	0.771153	0.396006	1.501689	0.444726
MBD3	1.244924	0.786511	1.970521	0.349783
HLTF	0.827162	0.6218	1.10035	0.192505
FAM50A	1.441324	1.055441	1.968292	0.021485
FAM3A	1.335776	0.866676	2.058785	0.18963
CPSF1	1.230299	0.898367	1.684874	0.196392
CYBRD1	0.947606	0.699312	1.284056	0.728476
PDCD2	1.322755	0.651859	2.684139	0.438497
RDH11	1.148648	0.694911	1.898648	0.588865
PRKACA	1.359056	0.974677	1.89502	0.070491
ACTN1	0.915427	0.708167	1.183345	0.499892
ZFYVE26	1.011199	0.639198	1.599696	0.962046
EPN2	0.952846	0.717603	1.265208	0.738466
PTPN18	1.195004	0.889801	1.604893	0.236417
LIMS2	0.973102	0.624127	1.517204	0.904226
LNX1	1.144472	0.745693	1.75651	0.536971
ALDH3A2	1.107071	0.861489	1.42266	0.426687
TFRC	0.853477	0.67471	1.079608	0.186431
SREBF1	1.598011	1.200858	2.126511	0.001302
TRPC5	0.787721	0.45	1.3789	0.403562

AFF4	0.649236	0.430292	0.979584	0.039566
UBE2D1	1.179704	0.909153	1.530766	0.213723
MPP5	0.586144	0.364142	0.943491	0.027845
RHOBTB1	0.99928	0.840457	1.188117	0.993494
SMC1A	1.126543	0.757125	1.676209	0.55674
HSD17B10	2.026524	1.289571	3.184623	0.002194
MARK2	1.578083	0.941039	2.646381	0.083707
HMMR	0.839894	0.660125	1.068618	0.155641
CHFR	0.997916	0.697121	1.428497	0.990903
P4HA2	0.719356	0.447639	1.156006	0.173519
FCGR2B	1.156905	0.948198	1.41155	0.151026
NFATC3	0.771787	0.502222	1.18604	0.237339
TRNT1	0.51865	0.343714	0.782621	0.001762
ACADVL	1.210524	0.863295	1.697415	0.267995
STK10	1.883264	1.290342	2.74864	0.001033
FBXW11	1.115569	0.637528	1.952063	0.701648
ACAP1	1.439679	0.989983	2.093648	0.056485
CRMP1	0.728203	0.533992	0.993048	0.045066
EVC	0.955384	0.78858	1.157472	0.641064
DERL2	0.693392	0.344607	1.395189	0.304695
SIDT1	1.066012	0.839529	1.353594	0.599878
NDE1	1.197771	0.741437	1.934966	0.460851
TMEM38A	0.975864	0.760794	1.251732	0.84747
AP1M1	1.368733	0.861118	2.175581	0.184323
PVR	1.023817	0.849915	1.233302	0.804271
XRCC1	1.618599	0.91771	2.854784	0.096243
SCARB1	1.080769	0.765661	1.525559	0.658735
CYP2W1	0.964458	0.681718	1.364464	0.838014
MCM2	1.308599	1.006645	1.701127	0.04449
PANX2	1.041945	0.860145	1.26217	0.674482
TP63	1.100634	0.85094	1.423596	0.465143
ALPK1	0.849225	0.584316	1.234235	0.391591
LLGL2	0.905543	0.695752	1.178593	0.460573
PDE8A	0.585402	0.407985	0.83997	0.003654
CLCN4	0.993776	0.792381	1.246358	0.956908
NLE1	1.033031	0.722895	1.47622	0.858396
SDHA	2.45569	1.320811	4.565691	0.004521
SMARCE1	0.637435	0.381789	1.064262	0.085102
GSDMB	0.754741	0.581786	0.979113	0.034097
KDM5A	0.866277	0.52137	1.439351	0.579491
ADAM11	1.023413	0.782605	1.338319	0.86573
PPP2R3A	0.67805	0.441481	1.041386	0.075943
FERMT2	0.895917	0.508845	1.577428	0.703358
DHRS9	1.240587	1.085153	1.418284	0.001597
CD5L	0.951153	0.798936	1.132371	0.573548
PTGS2	1.063424	0.923538	1.224499	0.392789
IGF2BP2	1.366707	1.06275	1.757598	0.014926
MAP3K13	1.6969	1.08947	2.643	0.019336
ST6GAL1	1.108818	0.796466	1.543666	0.540611
TBX21	1.159297	0.949933	1.414806	0.1458
FRY	0.838323	0.686568	1.02362	0.083481
PICALM	0.98544	0.647335	1.500138	0.945459
NSF	1.124629	0.734362	1.722298	0.589111
CLASP1	0.67479	0.40355	1.128339	0.133711
MRPS34	1.854132	1.212315	2.835737	0.004398
NOTCH3	0.922706	0.779352	1.092427	0.350408
CLNS1A	0.809213	0.481352	1.36039	0.424448
TEAD2	0.925051	0.620516	1.379043	0.702159

EED	1.117717	0.629411	1.98486	0.704071
TSG101	1.383911	0.742084	2.580853	0.306856
ATP2A3	1.120626	0.845172	1.485854	0.428796
MGLL	1.177644	1.001803	1.384351	0.047502
BCS1L	1.336974	0.865514	2.065247	0.190546
NUAK1	1.700766	0.810459	3.569095	0.160237
DPP8	0.822306	0.472099	1.432299	0.489564
SLC24A1	0.653501	0.447224	0.95492	0.027926
ZNF532	1.340649	1.141506	1.574535	0.000353
SCARF1	1.137575	0.917821	1.409945	0.239211
LMAN1	0.677647	0.488696	0.939653	0.019639
ZZEF1	0.918034	0.637642	1.321723	0.645579
ENO1	2.208462	1.481273	3.292645	0.000101
ANO8	1.100466	0.87599	1.382464	0.410806
TUBE1	0.775362	0.508422	1.182455	0.237358
ARHGEF1(	1.165879	1.025857	1.325014	0.018722
TXK	0.941804	0.763299	1.162054	0.57602
TACR2	1.173203	0.810279	1.698679	0.397608
ACTR6	0.980247	0.596319	1.611358	0.937291
TIPIN	1.06416	0.679445	1.666706	0.785888
SRI	1.159952	0.737014	1.825596	0.52137
EIF4G3	0.84996	0.549664	1.314316	0.464788
NUP37	1.309873	0.77803	2.205271	0.309814
SEMA3A	1.18818	0.776287	1.818622	0.427234
GTSE1	1.029008	0.77956	1.358277	0.840011
SEMA3C	1.229015	1.015471	1.487465	0.034208
TTC38	1.495863	1.086546	2.059374	0.013556
ACAT1	1.185532	0.795333	1.767167	0.403367
GRAMD4	1.005107	0.734187	1.376	0.974639
CELSR1	0.97534	0.774696	1.22795	0.831727
WNT8B	1.090926	0.729285	1.631898	0.671896
ZNF638	0.655794	0.432791	0.993703	0.046618
SLC25A40	0.626622	0.436813	0.898908	0.011122
ADD2	0.961145	0.833141	1.108815	0.586809
RASAL2	0.81301	0.644885	1.024966	0.079887
ZNF37A	0.833268	0.564784	1.229381	0.357976
MARK3	0.695494	0.391014	1.237072	0.216498
SLC25A3	1.560272	0.851862	2.857799	0.149664
FNDC3B	0.552201	0.406656	0.749837	0.000142
FOSL2	1.077814	0.866307	1.340959	0.501378
CACNG4	1.21524	1.012653	1.458356	0.036162
FRYL	0.535106	0.341542	0.83837	0.006342
TMEM131	0.944633	0.530763	1.681226	0.846449
FSCN1	0.983476	0.847353	1.141466	0.826482
ACTB	1.439206	1.020271	2.030159	0.038052
PLD1	0.764842	0.624532	0.936676	0.009525
WDR62	1.075762	0.786742	1.470957	0.647334
DLG1	0.549448	0.337035	0.895734	0.016326
RAB7A	1.829625	0.93574	3.577412	0.077425
BCAP29	0.523763	0.240374	1.141254	0.103638
SEC31B	1.038026	0.734278	1.467424	0.832659
SART3	1.236456	0.625586	2.443825	0.541474
ARHGAP1!	0.949141	0.595998	1.511528	0.825978
TUBA3D	1.059527	0.749712	1.497372	0.743176
EXOSC7	0.940896	0.510054	1.735668	0.845386
KIFAP3	1.016742	0.739926	1.397117	0.918445
MKRN2	0.679106	0.404179	1.141042	0.143846
MCM6	1.17139	0.868614	1.579706	0.299834

REXO2	0.899982	0.663907	1.220003	0.497207
RBM7	0.914888	0.508023	1.647603	0.766949
RBMS2	0.882337	0.655845	1.187046	0.408196
BAZ2A	1.05971	0.646857	1.736064	0.817879
PTPN23	1.109134	0.745533	1.650064	0.609307
MLH1	1.180229	0.651682	2.137455	0.584475
UNG	1.190003	0.844539	1.67678	0.320102
FMO4	1.043328	0.698604	1.558155	0.8358
KLHL20	0.904737	0.503833	1.624643	0.737489
SLC46A1	1.501519	0.989755	2.277896	0.055936
PLXNA2	1.065628	0.808681	1.404215	0.65161
SPAG5	1.019596	0.746804	1.392034	0.902769
ANKRD13/	1.587949	0.984288	2.561834	0.058083
TPD52	1.251765	1.003712	1.561121	0.046278
ACACB	0.843941	0.665335	1.070493	0.161963
TRAF4	0.970927	0.76529	1.231819	0.808027
PAG1	1.059428	0.840636	1.335163	0.624755
GPATCH1	0.914457	0.504803	1.656551	0.768005
ICAM3	1.469012	1.110129	1.943916	0.007124
NT5C2	0.558902	0.367941	0.848973	0.00638
MCAM	0.809155	0.596765	1.097137	0.17282
GPC4	1.095808	0.939343	1.278336	0.244457
MBNL3	0.821994	0.594969	1.135647	0.234583
RAP1GAP	0.962511	0.751249	1.233183	0.762494
XAB2	1.446718	0.984393	2.126177	0.060123
ARHGEF1	1.607152	1.15319	2.239819	0.005085
STXBP2	1.245178	0.950148	1.631817	0.111987
MAP2K7	1.161823	0.667602	2.02191	0.595703
DGKD	1.03913	0.754366	1.43139	0.814281
RARB	0.846834	0.571889	1.253963	0.40651
TOP2B	0.670566	0.469894	0.956935	0.027625
TM9SF3	0.766341	0.482999	1.215901	0.258497
NFKB2	1.364759	1.082426	1.720734	0.008545
UBE2T	1.130999	0.813975	1.571498	0.463242
PPP1R12B	0.934345	0.582284	1.499271	0.778356
DNAJC10	1.832227	1.111963	3.019036	0.017479
GTF3C1	1.513151	0.901991	2.538411	0.116606
IL4R	1.130073	0.887269	1.439321	0.321773
USP33	0.686135	0.417543	1.127502	0.137169
SNRPA	1.503126	0.938703	2.406925	0.089768
SPAG6	1.074262	0.958643	1.203825	0.217581
EXOSC5	1.395455	0.945969	2.058517	0.092971
DYNC1I2	0.945533	0.557631	1.603268	0.835323
APBB1IP	1.577412	1.027744	2.421061	0.037055
LRCH4	0.865488	0.608041	1.231938	0.422563
FAM76B	0.855191	0.499759	1.463408	0.568178
SIRT6	1.798757	1.196333	2.704538	0.004781
POLD3	1.328201	0.843326	2.091859	0.220692
CAPZB	2.513919	1.467304	4.307076	0.000792
GPR137B	1.070467	0.854572	1.340905	0.553507
NAALAD2	0.971067	0.650613	1.449356	0.885745
SLC25A43	1.075409	0.675712	1.711533	0.759119
UBE2A	1.409784	0.675504	2.942234	0.360243
FGFR1	0.862385	0.750325	0.991181	0.037098
SMC1B	0.932677	0.717581	1.21225	0.602343
FBLN1	0.800207	0.635531	1.007552	0.057965
CST7	0.90585	0.806386	1.017581	0.095659
PIAS2	0.645232	0.430364	0.967376	0.033965

AMPH	1.208667	0.95432	1.530804	0.115926
ARAF	1.467341	0.887804	2.425186	0.134717
MCCC1	1.037065	0.653867	1.644837	0.877096
LAMP3	1.718552	1.114647	2.649648	0.014233
ACER3	1.425084	1.035066	1.962062	0.029915
UBE2K	0.840362	0.481625	1.466306	0.5403
PIK3C3	0.820297	0.514337	1.308262	0.405555
N4BP2	0.720902	0.505476	1.028141	0.070802
TULP3	0.765074	0.459887	1.272788	0.302474
SYNJ2	1.111657	0.897728	1.376565	0.331725
PPP2R5C	1.553084	0.883145	2.731229	0.126385
GNB1	1.871412	0.994393	3.52193	0.052072
HOXA9	1.191805	1.087711	1.30586	0.000168
EDN1	2.090877	1.26055	3.468142	0.00428
MLLT10	0.650175	0.387723	1.09028	0.102626
ZCWPW1	0.848757	0.615347	1.170703	0.317593
P2RY10	1.005001	0.795858	1.269106	0.966574
ITM2A	0.874776	0.78801	0.971096	0.012063
VDAC3	1.074952	0.646939	1.786138	0.780262
PCM1	0.630191	0.414597	0.957895	0.03067
TNRC6C	0.774016	0.570215	1.050659	0.100382
CBFA2T2	0.605307	0.343236	1.067479	0.082853
ITCH	0.761672	0.442582	1.310816	0.325681
PKD2L2	0.776869	0.540146	1.117337	0.173315
TP53INP2	1.069852	0.939394	1.218427	0.308837
SDF4	1.232836	0.829104	1.833164	0.301091
MYH7B	0.872337	0.600747	1.26671	0.472965
TP73	1.050712	0.828109	1.333154	0.683829
TOLLIP	1.488521	0.976821	2.268272	0.064192
UBE2D4	0.81564	0.470211	1.414828	0.46836
RUNX1T1	0.951987	0.804451	1.126582	0.56685
THOC1	0.556795	0.363289	0.853373	0.007193
FKBP7	0.712052	0.499776	1.01449	0.060066
OSBPL6	1.094048	0.742988	1.610983	0.648916
SLC1A3	1.023258	0.8443	1.240149	0.814663
XRCC5	1.210968	0.764117	1.919136	0.415188
LXN	1.135344	0.876143	1.471228	0.337065
SP140	1.016687	0.793109	1.303292	0.896085
MKNK1	1.079427	0.775674	1.50213	0.65032
TNS1	1.052939	0.875518	1.266314	0.583744
REXO1	0.962408	0.661139	1.40096	0.841471
SAR1A	1.07838	0.604694	1.923128	0.798212
CDC14A	0.876693	0.508544	1.511355	0.635781
RAPGEF3	1.263744	0.973655	1.640261	0.078525
CEACAM1	1.070196	0.901599	1.270322	0.437955
SENP1	1.077568	0.657714	1.765437	0.766782
DUSP13	0.678054	0.255001	1.802963	0.436178
CIC	1.332745	0.949672	1.870339	0.096652
LIPE	1.142024	0.830532	1.570341	0.413785
FDFT1	1.104067	0.715695	1.703191	0.654438
PAFAH1B3	1.437231	0.99637	2.073159	0.052318
OPHN1	1.068618	0.832026	1.372485	0.603227
KIF22	1.236411	0.815748	1.874	0.317232
PGM1	1.255889	0.96143	1.640533	0.094638
DDX1	1.181953	0.750256	1.862049	0.470987
DNM2	1.179295	0.787054	1.767018	0.424096
EPB41L2	1.25713	1.08648	1.454584	0.00211
STX7	1.036465	0.702071	1.530128	0.85699

RABL2B	0.698765	0.433492	1.126372	0.141171
KEAP1	1.548732	1.041788	2.302361	0.030593
DDX43	1.105484	0.822731	1.485411	0.505824
PTPRH	1.084032	0.834014	1.409	0.5464
SLC35C2	0.734665	0.512493	1.053151	0.093324
CRYBG3	1.049648	0.876198	1.257432	0.599024
RFX3	0.794704	0.543278	1.162489	0.236373
RIF1	0.635403	0.430726	0.93734	0.022244
RAB21	1.188561	0.687024	2.056227	0.536784
SLC4A4	0.976106	0.737476	1.29195	0.86573
SMARCA2	0.896679	0.578776	1.389195	0.62537
SESN1	1.493822	1.150339	1.939867	0.002608
MID2	0.907412	0.653306	1.260352	0.562183
SRCAP	0.767482	0.421604	1.397116	0.386575
KCNN2	2.497696	1.384933	4.504542	0.002348
CNOT4	0.532295	0.298293	0.949864	0.032839
PSEN1	0.970765	0.612978	1.537388	0.899345
CPOX	0.997621	0.724803	1.373128	0.988342
CLDND1	0.818583	0.44693	1.499293	0.516779
HSP90AA1	1.136889	0.795083	1.625639	0.481953
RBL1	1.026313	0.719397	1.464168	0.886076
DLGAP4	0.751195	0.492648	1.145431	0.183802
IGSF9B	0.810038	0.659998	0.994187	0.043827
NDC80	0.934049	0.698854	1.248397	0.64482
AP4E1	0.87171	0.547388	1.388189	0.563035
RSBN1	0.7019	0.411066	1.198505	0.194752
MAGI3	0.798471	0.572957	1.112748	0.183826
CXCL2	1.066639	0.920593	1.235854	0.390508
COL4A4	1.33318	0.73628	2.413985	0.342459
TCF7	0.893924	0.704599	1.134119	0.355756
OSTM1	0.987611	0.691032	1.411477	0.94545
IMPG2	1.113912	0.684409	1.812951	0.664219
PCNP	1.058542	0.617353	1.815027	0.836167
EXD2	0.872594	0.647979	1.17507	0.369442
ARG2	1.230432	0.843088	1.795734	0.282344
MEF2C	1.253546	1.043873	1.505334	0.015532
PTPRC	1.297578	0.971663	1.732811	0.077534
UBA5	0.684552	0.431614	1.085718	0.107292
STK17B	0.88626	0.674849	1.163901	0.385181
CDC14B	0.759135	0.51999	1.108263	0.153439
ZNF510	0.937654	0.640908	1.371796	0.740193
ZNF506	0.704646	0.512902	0.968071	0.030756
JMJD4	1.87818	1.05564	3.34163	0.03202
DUSP12	0.901919	0.520559	1.56266	0.712781
AACS	0.955579	0.643219	1.419627	0.82199
PHLPP1	1.028311	0.77611	1.362465	0.845816
ATP8B1	0.782284	0.598687	1.022184	0.07199
IL12RB2	1.096382	0.956063	1.257295	0.187868
SMARCD3	0.93359	0.77868	1.119318	0.457892
WDR70	0.854322	0.45296	1.611326	0.62672
STRADB	1.023954	0.716408	1.463527	0.896649
BZW1	0.760056	0.471592	1.224968	0.259876
C1QTNF3	0.482072	0.322524	0.720547	0.000373
ME2	0.997872	0.620623	1.604436	0.992986
CCNT2	0.621859	0.376004	1.02847	0.064227
FAM135A	0.835181	0.630122	1.106971	0.210219
COL19A1	0.739951	0.433229	1.26383	0.270166
EPB41L3	1.16196	1.014741	1.330539	0.029881

COBLL1	1.051164	0.897394	1.231282	0.536339
DLG3	1.140972	0.882088	1.475835	0.315176
TRAF5	1.02344	0.821219	1.275455	0.836568
MRPL22	0.747348	0.364825	1.530953	0.42606
GEMIN5	0.832074	0.598109	1.15756	0.275118
NFE2L1	1.317671	0.803578	2.160659	0.274263
GSK3B	0.810557	0.48325	1.359551	0.426058
ITGB5	1.073089	0.848313	1.357425	0.556386
ERC1	0.824838	0.519015	1.310864	0.415228
XPO1	0.799992	0.490172	1.305638	0.371921
RNF13	1.275865	0.887933	1.833282	0.187743
PALB2	1.083502	0.695592	1.687738	0.722837
LYRM2	1.527354	0.83489	2.794153	0.169323
BCKDHB	0.661	0.456675	0.956743	0.028211
ULK2	1.141801	0.92654	1.407073	0.213451
TNPO1	0.814617	0.535619	1.238942	0.337845
PLOD1	1.242898	0.934976	1.65223	0.134375
P2RX5	0.833755	0.672045	1.034375	0.098384
ITGAE	1.096467	0.801479	1.500028	0.564642
DIS3	1.014135	0.618298	1.66339	0.955663
PIBF1	0.604271	0.405184	0.901179	0.013503
TDRD3	0.559578	0.329803	0.949437	0.031372
NUFIP1	0.87435	0.548992	1.392531	0.571747
PDS5B	0.729159	0.44791	1.187007	0.20393
OXCT1	1.375713	1.055209	1.793566	0.01842
RRAGB	0.974303	0.690727	1.374299	0.882077
CYLD	1.306652	0.798725	2.13758	0.286851
SLC27A5	1.049714	0.625769	1.760873	0.854147
ZNF324	0.969658	0.574894	1.635496	0.90803
ZNF671	0.901543	0.649165	1.252039	0.536211
ZNF416	0.974394	0.675286	1.405989	0.889727
ZNF586	0.824963	0.537852	1.265339	0.37797
ZNF446	0.799596	0.515585	1.240056	0.317821
ZNF264	0.850777	0.583323	1.240858	0.401328
RPS5	1.176536	0.849618	1.629246	0.32768
FAT1	1.000749	0.741175	1.351232	0.996099
YTHDC1	0.712608	0.392654	1.293277	0.265182
CHMP2B	1.107772	0.736073	1.667168	0.62361
SMAP2	1.445214	1.113477	1.875786	0.005643
PPIE	1.159167	0.73209	1.835386	0.528735
ZMPSTE24	0.971885	0.602063	1.568874	0.907083
STARD7	1.752967	1.017577	3.019814	0.043098
REST	0.775943	0.533512	1.128537	0.184417
HAL	0.882247	0.762513	1.020782	0.092266
SSH1	1.06595	0.67495	1.683458	0.784147
GSTP1	1.77349	1.247212	2.521839	0.001423
APLP2	1.073188	0.850615	1.354001	0.55143
WBP11	0.998325	0.588413	1.693797	0.99504
EIF3I	1.844108	1.05683	3.217864	0.031197
TXLNA	0.98351	0.556526	1.738087	0.954359
NCOA1	0.856734	0.553073	1.327118	0.488622
AGBL5	0.860571	0.548548	1.350078	0.513403
EFR3B	0.823619	0.6259	1.083798	0.165922
KIF3C	1.05857	0.757557	1.47919	0.738807
RAB10	1.105057	0.689948	1.769915	0.677654
HADHA	1.306613	0.826655	2.065235	0.252225
MAPRE3	1.098443	0.822406	1.46713	0.524867
CAD	1.179457	0.839185	1.657701	0.341902

CD59	1.036336	0.871315	1.23261	0.686712
CD82	1.136148	0.87272	1.47909	0.342919
BCORL1	1.212239	0.863765	1.701298	0.265695
ATRX	0.932232	0.635968	1.366512	0.71912
FCN1	1.066388	0.972107	1.169814	0.173522
MYNN	0.691019	0.411719	1.159791	0.161849
SCAMP1	0.624177	0.417905	0.932264	0.0213
PREP	1.263016	0.673001	2.370292	0.467224
HACE1	0.559622	0.380713	0.822604	0.003141
SEH1L	0.671245	0.418715	1.076076	0.09783
WDR47	0.750252	0.445543	1.263354	0.279816
WDFY1	1.154084	0.794788	1.675805	0.451423
OVGP1	1.23217	0.884075	1.717324	0.217743
SLC25A24	1.226606	0.945367	1.591513	0.124257
MAP3K4	0.738822	0.454762	1.200318	0.221505
PILRA	1.025225	0.866923	1.212432	0.770956
IGSF9	1.215768	0.690979	2.139128	0.497945
ABCB1	1.006849	0.877291	1.15554	0.92263
ZNF213	1.448039	0.972788	2.15547	0.068147
AKR1B1	1.556501	1.196522	2.024781	0.000977
CPNE3	1.27017	1.010553	1.596484	0.04037
RRN3	1.306092	0.85442	1.996532	0.217456
CTTN	1.055972	0.840421	1.326809	0.640122
WNT11	0.964759	0.757801	1.228238	0.770883
MTIF2	0.89512	0.569817	1.406136	0.630645
DDHD2	0.865463	0.575864	1.3007	0.486966
TTC39A	1.187174	0.736314	1.914106	0.481433
EPS15	0.843015	0.572511	1.241328	0.387052
MGST2	1.707791	1.209659	2.411053	0.002352
CHERP	0.962497	0.65015	1.424902	0.848557
ATG16L1	0.89376	0.50442	1.583614	0.700355
USP40	1.027024	0.756913	1.393527	0.864021
POMGNT1	1.256207	0.794357	1.986585	0.329344
RAD54L	1.154372	0.777272	1.714423	0.47685
MAST2	1.419699	0.960966	2.097417	0.078408
DNAJA1	1.241236	0.786125	1.959825	0.353747
B4GALT1	1.609596	1.116871	2.319695	0.010687
CHMP5	1.428536	0.8545	2.388198	0.173748
NFX1	1.520338	0.881702	2.621555	0.131798
IPO11	0.778063	0.507024	1.19399	0.250757
EIF2AK1	1.020279	0.652612	1.595081	0.92983
EPDR1	0.880961	0.697991	1.111895	0.285964
SNX10	1.186756	0.988694	1.424495	0.066075
SEPHS1	0.958051	0.544495	1.685711	0.88183
MRPL28	2.160246	1.346482	3.465819	0.001406
HBQ1	1.023359	0.824034	1.270899	0.834523
ITPKC	1.520371	0.933487	2.476227	0.092296
CEACAM6	1.028328	0.900329	1.174525	0.680432
FAT2	0.626272	0.420538	0.932656	0.021274
RBM22	0.959047	0.544686	1.688625	0.884815
TMED2	0.773172	0.439412	1.360443	0.372231
ZFAND6	0.856755	0.492322	1.490953	0.584418
PPEF1	1.315283	0.441418	3.919115	0.622746
LAT2	1.232116	1.004404	1.511454	0.045273
HUWE1	1.123005	0.737481	1.710063	0.588723
ZW10	0.968007	0.595241	1.574216	0.895729
ALG9	0.696238	0.415906	1.165521	0.168418
MYBPC2	1.380985	0.944864	2.018408	0.095501

ACOX3	1.233644	0.722973	2.105025	0.441206
MTMR2	0.720251	0.447442	1.159391	0.176676
PPP1R15A	0.905792	0.662589	1.238263	0.53508
HSD17B14	0.990885	0.765106	1.28329	0.944667
TRIP6	1.267647	1.014621	1.583772	0.036821
ACHE	0.853423	0.694462	1.04877	0.131769
FTL	1.29792	0.974167	1.72927	0.074882
BAX	1.30794	0.85727	1.995528	0.212957
NLK	0.943227	0.689793	1.289774	0.714296
PIGS	1.12722	0.658691	1.929016	0.662201
ADAMTS2	0.97256	0.858876	1.101291	0.660877
ATXN7L3	1.290651	0.776482	2.145291	0.325039
PGS1	0.945464	0.619115	1.443838	0.795165
PSMC5	1.458531	0.860867	2.471128	0.160604
UIMC1	0.910408	0.502908	1.6481	0.756578
CETP	0.975344	0.790072	1.204063	0.816332
MMP2	0.875833	0.78926	0.971901	0.012536
LPCAT2	0.788304	0.614444	1.011359	0.061329
OGFOD1	1.275277	0.734823	2.213228	0.387311
SH3BP2	1.372488	1.061666	1.77431	0.015662
NOP14	1.264233	0.747132	2.139229	0.382285
ADD1	1.483391	0.847495	2.596416	0.167395
L2HGDH	1.102423	0.708257	1.715953	0.665783
TXNDC16	1.029586	0.718604	1.475148	0.873737
NID2	0.927523	0.669746	1.284513	0.650639
GMCL1	0.440061	0.281036	0.689069	0.000334
SF3B2	2.145266	1.107189	4.156621	0.023717
GNAS	1.648551	0.970178	2.801261	0.064597
DNM1L	0.933122	0.548828	1.586503	0.79825
PHACTR3	1.170608	0.991681	1.38182	0.062709
ERGIC2	0.582221	0.367088	0.923433	0.021536
AURKA	0.935605	0.710597	1.231861	0.635326
CASS4	1.30388	1.049256	1.620294	0.016679
PIR	1.022799	0.834272	1.253928	0.828316
RFX2	1.139602	0.855122	1.518721	0.37248
METTL2A	0.840527	0.495841	1.424824	0.518826
SULT2B1	1.170876	0.970849	1.412117	0.098852
ALG6	0.589968	0.351533	0.990127	0.045768
CNOT3	1.276205	0.768703	2.11876	0.34571
GP6	1.157663	0.969336	1.382578	0.106058
PTPN4	0.76843	0.535199	1.103298	0.153495
DDX18	0.92089	0.587154	1.444322	0.719662
KHSRP	1.191769	0.753193	1.885725	0.45365
GNA11	0.859098	0.650847	1.133983	0.28361
EDEM2	1.159317	0.710421	1.891858	0.554092
DNMT3B	1.151205	0.960762	1.379398	0.126979
TPX2	1.051185	0.810989	1.362521	0.70607
FER1L4	0.972944	0.721017	1.312895	0.857622
PDRG1	1.909251	1.163316	3.133489	0.010515
EPB41L1	0.69546	0.411992	1.173964	0.173968
DOCK9	1.076088	0.850162	1.362053	0.541928
ANKRD10	0.8638	0.548576	1.360159	0.527346
TGDS	0.923533	0.569011	1.498938	0.747504
DOCK3	1.014141	0.825397	1.246045	0.893684
COQ9	1.754006	0.966206	3.184143	0.064753
TMEM40	1.286615	0.942408	1.75654	0.112618
KIF9	0.692999	0.4497	1.067927	0.096492
CRLS1	0.574745	0.273451	1.208011	0.143924

PPP1R13B	0.870333	0.653064	1.159886	0.343251
ATRN	1.134597	0.717881	1.79321	0.588705
SMOX	1.029075	0.819796	1.29178	0.804855
SIGLEC1	1.148699	1.015033	1.299966	0.028066
FKBP1A	2.340197	1.33683	4.096649	0.002919
NSFL1C	1.024414	0.619364	1.694357	0.925146
SLC4A11	0.936913	0.743688	1.180342	0.58028
ZNF343	1.121293	0.673428	1.867013	0.659873
EBF4	0.734146	0.58929	0.91461	0.005853
CPXM1	1.163459	0.967472	1.39915	0.107703
XRN2	0.863826	0.551475	1.353088	0.522618
DYNLL1	1.096438	0.694293	1.731512	0.692905
TESC	1.021959	0.877125	1.19071	0.780572
SNX5	1.12431	0.673286	1.87747	0.654246
RPL6	1.066739	0.73146	1.555699	0.737177
SIRPG	1.007866	0.799993	1.269753	0.946993
MAPKAPK	0.519108	0.281321	0.957885	0.035939
P2RX7	1.170988	0.959027	1.429798	0.121303
ESF1	0.95461	0.65457	1.39218	0.809327
RBBP9	1.05886	0.633998	1.768434	0.826999
ANAPC5	0.853458	0.534711	1.362213	0.506544
SLC23A2	0.93078	0.634516	1.365374	0.713672
KDM2B	0.810685	0.493434	1.331911	0.40738
TASP1	0.556133	0.367647	0.841253	0.00546
OAS1	1.335049	1.127436	1.580893	0.000806
RPLP0	1.265192	0.863636	1.853455	0.227267
PXN	1.412076	1.071963	1.860101	0.014119
SIRT4	0.841162	0.555806	1.273025	0.413269
KIF16B	1.397553	1.120779	1.742676	0.002953
TRMT6	1.583353	1.039018	2.412863	0.032513
PEBP1	1.411807	0.867678	2.297166	0.164982
BRAP	1.083962	0.54036	2.174428	0.820431
ERP29	1.080971	0.655305	1.783138	0.760448
FUS	0.962185	0.576861	1.604893	0.882596
IGBP1	0.935489	0.618195	1.415638	0.75238
FXYD5	0.956984	0.66826	1.370451	0.81035
ZNF302	0.950768	0.678742	1.331818	0.769071
GRAMD1A	0.843522	0.568608	1.251351	0.397737
CMTM1	1.229212	0.934193	1.617398	0.140529
KCNH4	1.220702	0.946473	1.574385	0.12449
GANAB	0.856763	0.555093	1.322379	0.485108
GMIP	1.330871	0.980498	1.806448	0.06671
RBM41	0.978415	0.657097	1.456855	0.914445
BIRC5	1.035808	0.773445	1.387168	0.813371
LAG3	1.20205	1.013767	1.425302	0.034237
MLF2	1.611441	1.024964	2.533496	0.038755
OTUB2	1.122985	0.710446	1.775073	0.619522
DDX24	1.282688	0.809954	2.031336	0.288523
ZBTB25	0.778446	0.469184	1.291556	0.332272
NECAP1	0.756229	0.429154	1.332578	0.333716
ARHGAP4	1.482752	1.167261	1.883514	0.001251
ANKRD24	1.432859	1.001053	2.050925	0.049331
DHX32	0.769901	0.577287	1.026781	0.075067
RCOR1	0.917656	0.560024	1.503675	0.733068
LTBP4	1.008041	0.853284	1.190865	0.924969
BLVRB	1.087156	0.908129	1.301478	0.362691
SLC9A1	1.706134	1.096189	2.655466	0.01794
SPTLC1	1.139083	0.651785	1.990701	0.647534

PAPOLA	0.726033	0.430672	1.223955	0.229541
CCNK	0.989126	0.561193	1.743377	0.969839
PCBP4	0.908368	0.742009	1.112025	0.351769
RGS1	0.963838	0.81438	1.140726	0.668338
YPEL3	1.149056	0.827331	1.595891	0.407105
MRPS33	1.106506	0.679195	1.802656	0.684423
NDUFB2	0.915103	0.515563	1.624269	0.761848
NUDC	2.439977	1.367179	4.354577	0.002543
MAEA	1.251124	0.718227	2.17941	0.428838
ICAM1	1.127096	0.913401	1.390786	0.264649
STRN4	1.35535	0.882096	2.08251	0.165293
IRAK3	0.928242	0.744413	1.157467	0.508428
LYZ	1.052192	0.963427	1.149135	0.257888
MUL1	2.454429	1.343122	4.485238	0.003512
TFAP4	0.785949	0.48556	1.272173	0.326958
PDCD7	1.019617	0.606735	1.713465	0.941526
SPG21	1.799027	1.128211	2.8687	0.013638
DNAJB11	0.571501	0.372656	0.876446	0.010334
FLT3LG	1.194927	0.742759	1.92236	0.46289
RAB11FIP3	0.880407	0.6591	1.176023	0.388524
GNPTG	1.182896	0.76254	1.834976	0.453382
ZNF268	0.905785	0.596648	1.375094	0.642244
GOLGA3	0.746681	0.47778	1.166923	0.199731
PABPC4	1.279561	0.733195	2.23307	0.385582
CD209	1.050491	0.762714	1.446849	0.762975
MCOLN1	1.563156	1.160444	2.105623	0.003293
USP48	0.757319	0.497768	1.152207	0.1942
EFNB1	1.027519	0.828554	1.274263	0.804733
PDPR	0.953222	0.676792	1.342557	0.783957
GLG1	1.304877	0.899591	1.892755	0.160814
KIF4A	0.991647	0.765451	1.284684	0.949366
TNRC6A	0.77347	0.45384	1.318208	0.34501
PLEKHG2	1.078554	0.850401	1.367919	0.532876
DLL3	0.519191	0.342349	0.787383	0.002036
NAT14	0.890313	0.671541	1.180355	0.419379
PITPNM2	0.89538	0.697249	1.149813	0.386496
EXOC1	1.01941	0.654094	1.588758	0.93233
RBM27	0.92617	0.602359	1.424052	0.72677
OSBPL8	0.874933	0.602139	1.271313	0.483415
DTX2	0.988898	0.657519	1.487286	0.957242
NLRC4	1.077177	0.922214	1.258178	0.34818
PUS7	0.803702	0.583352	1.107285	0.181346
NRCAM	0.522299	0.203991	1.337292	0.17572
LAMB1	0.607534	0.364945	1.011377	0.055307
DLD	0.910995	0.60269	1.37701	0.658314
WDR7	0.96296	0.606387	1.529208	0.872921
TXNL1	0.710712	0.372582	1.355708	0.300027
IL5RA	0.913344	0.751944	1.109387	0.360908
ABCC6	0.88663	0.702686	1.118726	0.31045
CMTM6	0.884151	0.608312	1.285069	0.518694
ITGA6	0.912424	0.7484	1.112396	0.364688
RAPGEF4	0.764031	0.505597	1.154562	0.201358
FH	1.655195	1.056865	2.592263	0.027694
SEL1L3	1.541608	1.245051	1.908803	7.17E-05
CDV3	1.065687	0.647756	1.753265	0.802235
MYO15A	1.183605	0.460169	3.04436	0.726556
ALKBH5	2.1831	1.16284	4.098522	0.015124
NLRP1	1.100505	0.916553	1.321375	0.304779

PITPNM3	1.042885	0.570184	1.907472	0.891578
SPAG7	1.593315	0.89451	2.838037	0.113768
ZC3HC1	1.016991	0.554644	1.864746	0.956564
ESR1	1.158106	0.7876	1.702907	0.455551
ANGPT2	1.12114	0.789566	1.591957	0.522693
TMEM101	0.844908	0.493655	1.446089	0.538784
CD200	0.958353	0.859845	1.068146	0.442073
CCDC80	0.994391	0.696471	1.419748	0.975302
CMA1	0.570644	0.336056	0.968989	0.037841
PSME1	1.828311	1.177296	2.839319	0.007215
PPP2R3C	0.957235	0.569814	1.608068	0.868838
HAUS4	1.20146	0.810561	1.780874	0.360707
JPH4	1.854355	1.418722	2.423753	6.19E-06
CEBPE	0.887202	0.773856	1.017151	0.08614
SLC7A8	0.925186	0.727456	1.176661	0.52617
OSGEP	1.067219	0.6261	1.81913	0.811034
SLC22A17	1.001111	0.797501	1.256704	0.992363
RNF31	0.938097	0.484182	1.817554	0.849806
SCFD1	0.519243	0.287305	0.938419	0.029973
HECTD1	0.694201	0.429449	1.122172	0.136341
HNRNPC	1.255501	0.607453	2.594904	0.539045
RPGRIP1	0.925545	0.65651	1.30483	0.658816
SUPT16H	1.135977	0.739262	1.745582	0.560792
TOX4	1.694087	0.859001	3.341012	0.128176
TGM1	1.289452	0.781953	2.126326	0.319173
TINF2	2.372381	1.284639	4.381143	0.005775
TBL1Y	0.622507	0.386786	1.001884	0.050913
SEMA6A	0.990899	0.695296	1.412176	0.959658
TRPM7	0.740701	0.46664	1.175721	0.202924
TYRO3	0.8175	0.553757	1.206858	0.310628
WDR76	1.099911	0.775883	1.55926	0.592768
CAPN3	0.822186	0.608097	1.111649	0.203302
SNAP23	0.720052	0.422977	1.225776	0.226288
PHGDH	1.266403	1.049591	1.528001	0.013696
COL9A3	1.024534	0.743432	1.411925	0.882244
EZR	1.249815	0.930019	1.679575	0.139185
MYL6	1.703849	1.106202	2.624387	0.015609
TEKT2	0.72006	0.565733	0.916487	0.007618
CLSPN	1.207644	0.889182	1.640165	0.227059
RFFL	0.479037	0.277704	0.826334	0.008152
UNC13D	1.416484	1.037345	1.934196	0.028477
MFSD11	0.980392	0.555053	1.731669	0.945606
DPYSL2	1.180492	1.01011	1.379614	0.036938
GPATCH2	0.871251	0.478674	1.585796	0.651962
NUP50	0.814999	0.494732	1.342593	0.421846
COMT	0.828413	0.572671	1.198363	0.317638
VNN3	1.136584	0.967747	1.334877	0.118668
ECHDC1	0.981986	0.61771	1.561085	0.938737
LRRFIP2	0.987378	0.60483	1.611883	0.959486
SEC22C	1.656488	1.005411	2.729186	0.047575
XYLB	1.291739	0.985401	1.69331	0.063813
HDAC6	0.901975	0.615485	1.321817	0.596741
CDC6	1.120645	0.818818	1.533729	0.47681
UPRT	1.079741	0.557172	2.092423	0.820205
CDC23	0.724883	0.475978	1.103949	0.13383
AAAS	1.880548	1.088033	3.250326	0.023687
CBX5	1.017813	0.726324	1.426281	0.918313
MSH2	0.918281	0.650701	1.295894	0.627611

MAP3K1	0.639028	0.485323	0.841413	0.001423
DHPS	0.863268	0.542323	1.374148	0.535317
HOOK2	1.147536	0.712778	1.847475	0.571117
ARCN1	1.303235	0.822185	2.065742	0.259784
TMEM38B	1.681539	1.278218	2.212121	0.000204
PSMD5	1.055111	0.626218	1.777749	0.840277
PTGS1	1.267044	1.043294	1.538781	0.016964
NUP188	1.091412	0.691394	1.722866	0.707256
CRAT	1.234305	0.966751	1.575906	0.091276
SH2D3C	1.296087	1.024965	1.638927	0.03032
NANS	1.680349	0.823188	3.430045	0.154001
TBC1D2	1.263114	1.004144	1.588872	0.046011
PDE6C	0.903757	0.55456	1.472838	0.684662
CWF19L1	1.381215	0.751512	2.538553	0.298324
SEMA4G	0.821462	0.559389	1.206316	0.31577
BTAF1	0.96418	0.66258	1.403067	0.848855
IKZF5	0.839894	0.502821	1.402929	0.505053
BLNK	1.178707	1.034324	1.343244	0.013657
SORBS1	1.000238	0.756272	1.322907	0.998667
BAMBI	0.866097	0.710084	1.056389	0.156007
IL11	1.93598	0.637241	5.88164	0.243944
WAC	1.077995	0.570733	2.036104	0.816952
CREM	0.9044	0.490939	1.666071	0.747182
NUBP2	1.258671	0.831643	1.904966	0.276568
TPSD1	0.95104	0.857388	1.05492	0.342564
HIVEP1	1.113535	0.766034	1.618673	0.57312
TREM2	0.977842	0.82125	1.164291	0.801311
KCNK16	0.903264	0.647784	1.259503	0.54864
CRISP3	1.109733	0.924841	1.331587	0.262838
FKBP5	1.427539	1.148217	1.774811	0.001355
SRPK1	0.898406	0.575755	1.40187	0.636983
BRPF3	0.831374	0.534816	1.292374	0.411946
MRPS18A	2.722712	1.465804	5.057402	0.001523
TMEM14A	1.318208	1.03954	1.671578	0.022609
EFHC1	0.638277	0.420742	0.968282	0.034725
NCR2	1.198832	1.040161	1.381706	0.012295
HSP90AB1	1.179816	0.820677	1.696119	0.371928
MLN	1.281777	0.766852	2.142464	0.343566
CDC5L	1.058333	0.649405	1.724759	0.820019
ITPR3	1.129774	0.952748	1.339693	0.160534
ZNF184	0.790923	0.577298	1.083599	0.144248
SIRT1	0.694406	0.413055	1.167397	0.168822
HNRNPH3	0.583408	0.348489	0.976689	0.040396
IFT74	0.712865	0.431752	1.177008	0.185854
JAK2	1.03601	0.77669	1.381912	0.809804
IL12RB1	1.730883	1.347771	2.222897	1.72E-05
ABL1	0.893861	0.582074	1.372655	0.60817
ACOT7	1.655416	1.222753	2.241174	0.00111
SH3GLB1	1.013683	0.609717	1.685296	0.958212
CDC7	0.939028	0.660848	1.334305	0.725612
SYDE2	1.07578	0.731608	1.581863	0.710394
PCSK5	1.096049	0.76726	1.565731	0.614251
SCD	1.196441	1.029015	1.391107	0.019709
TMED1	1.009895	0.628232	1.623427	0.967571
ABLIM1	1.116539	0.953961	1.306823	0.169772
ERMP1	1.062872	0.776897	1.454114	0.702979
RAB18	0.841225	0.497361	1.42283	0.519058
NRP1	0.933959	0.778878	1.119917	0.460827

HSD17B7P	0.908882	0.701717	1.177205	0.46914
PRTFDC1	1.091699	0.918185	1.298004	0.320489
TSPAN15	1.326777	1.008624	1.745287	0.043244
MAST3	1.281446	0.964228	1.703024	0.087462
MZF1	0.805472	0.52539	1.234865	0.321058
OCEL1	1.751209	1.206937	2.540923	0.003174
MYO9B	1.210287	0.793601	1.845755	0.375421
KCNK6	1.044201	0.840176	1.297772	0.696576
PSMD8	2.084584	1.261332	3.445159	0.004161
FBXL19	1.371131	0.933948	2.012963	0.107147
STX1B	1.279011	0.914287	1.789229	0.150782
HSD3B7	1.185231	0.943303	1.489205	0.144596
SETD1A	1.220426	0.808871	1.841381	0.342515
BCL7C	1.01255	0.599903	1.70904	0.962753
CIRBP	1.616394	1.005957	2.597257	0.047199
PRKY	0.986131	0.90302	1.07689	0.755871
IGFALS	0.747346	0.509904	1.095353	0.135427
HNRNPM	1.057317	0.618073	1.808717	0.838771
NDUFB7	1.654452	1.115986	2.452729	0.012203
TECR	1.604835	1.001011	2.572896	0.049512
TIMM13	1.099844	0.781297	1.548268	0.585445
CDC34	1.128556	0.740287	1.720464	0.574011
MTAP	0.872843	0.553448	1.37656	0.558495
POLR2E	1.954633	1.259536	3.033332	0.002798
POLRMT	1.181195	0.848547	1.644247	0.323747
HCN2	1.365862	0.958444	1.946467	0.084506
RASSF7	1.415648	1.042671	1.922042	0.025895
GADD45B	0.987369	0.801705	1.21603	0.904796
PALM	0.795405	0.685626	0.92276	0.002521
MADCAM1	0.974893	0.535151	1.775978	0.933778
MKNK2	1.396634	0.968153	2.014749	0.073962
ARVCF	0.870724	0.70613	1.073683	0.195346
TRMT2A	1.459574	0.945026	2.254283	0.088191
RANBP1	1.558281	0.955322	2.541803	0.075589
ZDHHC8	1.060479	0.800008	1.405756	0.683029
KLHL22	1.084531	0.718767	1.636424	0.69903
MED15	1.522725	1.014208	2.286209	0.04256
SERPIND1	0.816714	0.56023	1.190623	0.292456
SNAP29	1.823382	1.043329	3.186649	0.034955
CRKL	1.228531	0.695676	2.169526	0.47811
LZTR1	1.779132	1.056256	2.996729	0.030334
MMP11	1.094119	0.813963	1.4707	0.551161
CECR2	0.57917	0.308869	1.086017	0.088622
SMARCB1	1.188166	0.696891	2.025765	0.5265
DERL3	0.946587	0.698517	1.282757	0.723327
BCL2L13	1.260548	0.680652	2.334502	0.461472
DDTL	1.597812	0.89286	2.859355	0.114497
OSM	1.126451	0.947468	1.339244	0.177422
CABIN1	1.418216	0.956475	2.102863	0.082116
TBC1D10A	1.117227	0.76226	1.637495	0.569848
SUSD2	1.010489	0.718421	1.421296	0.952195
SF3A1	1.934816	1.057368	3.540406	0.032281
GGT5	0.980729	0.836824	1.149381	0.810058
RNF215	1.551727	0.937862	2.567388	0.087219
SEC14L2	1.280445	0.911716	1.798302	0.153699
SEC14L3	0.845574	0.594618	1.202445	0.350444
PPIL2	1.265629	0.785137	2.040174	0.333546
UPB1	1.292511	0.942526	1.772455	0.111255

YPEL1	0.708082	0.532861	0.940921	0.017323
SNRPD3	1.412901	0.818101	2.440149	0.215045
PES1	1.916742	1.103006	3.330807	0.021016
MAPK1	1.429493	0.906604	2.25396	0.124061
GGT1	1.363058	0.870569	2.134154	0.175729
PRODH	0.897541	0.744611	1.081881	0.256714
PPM1F	1.479492	1.123688	1.947957	0.005257
SLC35E4	1.520865	1.150246	2.010901	0.003259
TOP3B	0.523538	0.224806	1.219239	0.133516
CYTH4	1.399781	1.165515	1.681135	0.000319
MFNG	1.905779	1.388353	2.616046	6.60E-05
CARD10	1.336432	0.868049	2.057545	0.187763
LRP5L	0.812489	0.603027	1.094709	0.172223
SLC25A1	1.432057	1.12309	1.826021	0.003778
LGALS2	1.119239	1.023258	1.224222	0.013794
GGA1	1.823286	1.135299	2.928192	0.012957
HIRA	2.165761	1.03067	4.550942	0.04138
SH3BP1	1.709634	1.212593	2.410412	0.002215
SEZ6L	0.93444	0.773166	1.129354	0.482994
LGALS1	1.297314	1.128732	1.491075	0.000247
HPS4	0.818183	0.613447	1.091249	0.17204
PIK3IP1	1.342227	1.058369	1.702217	0.015186
SRRD	0.966677	0.613101	1.52416	0.884016
PATZ1	1.040689	0.669011	1.618858	0.859573
TRIOBP	1.343011	0.902382	1.998795	0.14604
TFIP11	1.030703	0.654069	1.624215	0.896305
GCAT	0.934338	0.753398	1.158733	0.536287
CRYBB1	1.461527	1.082707	1.972888	0.013172
ANKRD54	1.095929	0.707579	1.697422	0.681541
EIF3L	0.986484	0.72538	1.341575	0.930871
MICALL1	1.285285	0.94237	1.752982	0.112946
POLR2F	1.359174	0.727423	2.539589	0.335971
SOX10	0.79012	0.523234	1.193135	0.262614
CCDC134	0.949058	0.615575	1.463203	0.812878
DEPDC5	1.136508	0.655094	1.971701	0.648951
PICK1	1.695292	1.19192	2.411247	0.003317
TTC28	1.06617	0.880469	1.291038	0.511692
SLC16A8	1.420675	1.136057	1.776598	0.002082
CENPM	1.282676	0.957331	1.718589	0.09535
SLC5A4	0.982842	0.825092	1.170753	0.84627
KDELR3	0.836177	0.584383	1.196459	0.327705
CYP2D6	0.964676	0.708667	1.313169	0.819219
DDX17	0.724259	0.463851	1.130862	0.155892
DMC1	0.860138	0.63335	1.168134	0.33465
TCF20	0.959519	0.612489	1.503171	0.85682
HSCB	1.366603	0.757031	2.467008	0.300037
CBY1	0.964768	0.615313	1.512688	0.875791
TOMM22	1.406822	0.77826	2.543042	0.258469
XBP1	0.932119	0.641226	1.354977	0.712648
JOSD1	1.521669	0.919156	2.519134	0.102636
FBXO7	1.526598	0.902107	2.583397	0.114995
GTPBP1	1.305699	0.820645	2.077453	0.260275
POLDIP3	1.901208	1.001866	3.607861	0.049337
RAB36	2.517543	1.437831	4.408045	0.001235
TIMP3	1.067536	0.926507	1.230033	0.365977
SBF1	1.183513	0.877623	1.596018	0.269441
CYB5R3	1.638916	1.137788	2.360763	0.007973
DNAL4	0.968036	0.587539	1.59495	0.898535

LMF2	1.252496	0.93759	1.673169	0.127559
RHBDD3	1.151367	0.812202	1.632163	0.428555
PACSIN2	1.647941	0.966283	2.810469	0.066649
TTLL1	1.302686	0.849194	1.998353	0.225816
RASL10A	0.884293	0.672542	1.162714	0.378595
AP1B1	1.25596	0.933257	1.690247	0.132559
HMGXB4	1.439303	0.794635	2.606975	0.229551
TOM1	1.044333	0.800472	1.362485	0.749193
NEFH	0.95225	0.763664	1.187407	0.663915
CHKB	1.31266	0.762433	2.25997	0.326369
BIK	0.924314	0.785206	1.088067	0.344281
HMOX1	1.153015	1.02222	1.300546	0.020466
MCAT	1.084687	0.716059	1.643084	0.701229
THOC5	0.921605	0.51067	1.66322	0.786375
MCM5	1.67416	1.184851	2.36554	0.003482
APOBEC3H	1.307322	1.050863	1.626369	0.016161
ARSA	1.140828	0.900138	1.445877	0.275813
TSPO	1.281582	1.006688	1.631541	0.044001
RASD2	2.445276	0.744004	8.036752	0.140786
TTLL12	1.338844	0.934506	1.918127	0.111674
CBX7	1.051942	0.72852	1.518945	0.787043
PDGFB	1.375746	1.025915	1.844868	0.0331
ACR	0.831324	0.631319	1.094692	0.188296
CABP7	0.668883	0.263534	1.697707	0.397428
RPL3	1.150689	0.826965	1.601138	0.404987
ZMAT5	1.627139	0.848986	3.118521	0.142451
SYNGR1	0.865757	0.677282	1.10668	0.249828
ASCC2	1.598465	0.898938	2.842342	0.110227
MTMR3	0.758011	0.467542	1.228939	0.261104
APOL4	1.05916	0.919786	1.219654	0.424619
APOL1	1.510266	1.076917	2.117994	0.016874
PNPLA3	1.36436	0.913783	2.037113	0.128734
MYH9	1.550224	1.096332	2.192032	0.013127
CACNA1I	1.16481	0.81161	1.671718	0.407894
SAMM50	1.936629	1.011631	3.707411	0.046057
TXN2	2.97384	1.643085	5.382389	0.000318
FOXRED2	1.206712	0.876208	1.661881	0.249865
GRAP2	0.919287	0.753534	1.1215	0.406764
EIF3D	1.246068	0.807018	1.923978	0.320916
TNRC6B	0.840248	0.578364	1.220713	0.361033
SGSM3	0.991242	0.611325	1.607266	0.971546
NCF4	1.510928	1.113873	2.049518	0.007972
CSF2RB	1.136284	0.954819	1.352236	0.150102
SLC25A17	0.973635	0.53208	1.78162	0.930936
UPK3A	1.223589	1.015111	1.474883	0.034231
FAM118A	1.11201	0.864414	1.430525	0.408711
KCTD17	1.657669	1.277093	2.151657	0.000146
ST13	0.779758	0.508911	1.194754	0.253183
IL2RB	1.036162	0.870865	1.232834	0.688696
RBX1	1.712188	0.819416	3.577656	0.152641
EP300	0.98836	0.602481	1.621387	0.963023
L3MBTL2	1.725167	0.932806	3.190588	0.082168
CHADL	1.012571	0.662001	1.548788	0.954056
RANGAP1	1.752248	1.086896	2.8249	0.021339
ZC3H7B	1.147151	0.73433	1.792049	0.546386
PHF5A	1.815445	0.986355	3.341432	0.055387
ACO2	2.065766	1.176577	3.626951	0.011532
POLR3H	1.482351	0.899702	2.442324	0.122322

TRMU	1.152487	0.709222	1.872795	0.566692
PMM1	1.739321	1.267603	2.386583	0.000606
CERK	0.974767	0.634879	1.496618	0.907001
BRD1	0.84253	0.520497	1.363804	0.48562
ZBED4	0.70851	0.483198	1.038882	0.077627
MLC1	0.930112	0.783812	1.10372	0.406683
HDAC10	1.088736	0.632097	1.875261	0.759255
KCNK10	1.45354	1.097688	1.924754	0.00904
ABHD4	0.796136	0.63932	0.991417	0.041651
KHNYN	1.221394	0.795593	1.875083	0.360492
FKBP3	1.283557	0.733627	2.245717	0.381758
SDR39U1	1.120705	0.708782	1.772025	0.625907
CTSG	0.978415	0.909054	1.053069	0.5608
GZMH	1.2404	1.057048	1.455557	0.008296
GZMB	1.257178	1.073068	1.472876	0.004613
RBM23	1.058821	0.587498	1.908265	0.849167
PRMT5	1.182742	0.751464	1.86154	0.468296
COCH	0.910761	0.743859	1.115111	0.365441
AP4S1	0.606209	0.360644	1.018982	0.058892
POLE2	0.911698	0.649433	1.279877	0.593233
SOS2	0.660256	0.413764	1.053591	0.08168
CDKL1	1.118333	0.709955	1.761617	0.629521
NIN	0.887878	0.643497	1.225067	0.469039
PYGL	0.977721	0.721596	1.324757	0.884414
TRIM9	1.158349	0.895745	1.497942	0.262449
PSMC6	0.777515	0.475536	1.271258	0.315767
GNPNAT1	0.912872	0.598661	1.391999	0.67194
DDHD1	0.826722	0.5638	1.212256	0.329879
CDKN3	1.032099	0.762506	1.39701	0.837925
CGRRF1	0.997529	0.543039	1.8324	0.993638
ATP6V1D	0.946808	0.550087	1.629645	0.843606
PLEK2	0.976502	0.71475	1.334111	0.881275
PIGH	1.228653	0.699656	2.157616	0.473526
PSMA3	0.944174	0.506754	1.759165	0.856423
VTI1B	1.068347	0.609513	1.872586	0.817398
TIMM9	0.913673	0.580509	1.438046	0.696441
GSTZ1	1.048371	0.660251	1.664643	0.841295
KIAA0586	0.75092	0.470175	1.199301	0.230463
TMED8	0.742435	0.475748	1.158615	0.189658
AHSA1	1.497214	0.932413	2.404139	0.09485
DAAM1	1.181051	0.876945	1.590616	0.273298
SPTLC2	1.223873	0.897147	1.669586	0.202316
RIN3	1.514475	1.093844	2.096856	0.012409
LGMN	1.091117	0.893159	1.332951	0.393247
ALKBH1	1.053694	0.564964	1.965206	0.869365
SNW1	1.250868	0.683969	2.287635	0.467389
CHGA	1.097952	0.757485	1.591449	0.621726
ITPK1	1.818953	1.301401	2.542329	0.000462
DHRS7	0.72223	0.499446	1.044389	0.083778
PPM1A	0.639029	0.416287	0.980954	0.040569
ASB2	1.824095	1.348307	2.467778	9.70E-05
ERH	1.359878	0.841344	2.197993	0.209558
HIF1A	0.848077	0.601691	1.195355	0.346714
SLC10A1	0.944303	0.68687	1.298221	0.724182
EIF5	0.836884	0.518	1.352074	0.466892
SLC8A3	1.152751	0.959763	1.384546	0.128352
DICER1	0.736408	0.503352	1.077371	0.115006
ZFYVE21	0.872619	0.572053	1.331106	0.5271

MTHFD1	1.327608	0.892604	1.974608	0.161796
TCL1A	0.97811	0.805763	1.18732	0.822909
ZC3H14	0.69999	0.415135	1.180306	0.18087
TELO2	1.240952	0.942912	1.633197	0.123439
VRK1	1.147308	0.730442	1.802082	0.55084
PSMC1	1.249621	0.681176	2.292434	0.471646
PAPLN	0.8185	0.635564	1.05409	0.120707
RPS6KA5	0.865189	0.546387	1.370003	0.536903
PSMB5	1.634331	1.067714	2.501643	0.023721
YY1	1.180699	0.619022	2.25202	0.614131
ACIN1	0.731314	0.423597	1.262567	0.261382
CCNB1IP1	1.229795	0.893618	1.69244	0.204227
TRIP11	0.782792	0.519758	1.17894	0.241164
APEX1	1.122298	0.75044	1.678418	0.574206
PABPN1	0.836712	0.56258	1.244422	0.378722
ARHGAP5	0.809668	0.69018	0.949843	0.009553
CINP	2.208453	1.025157	4.757579	0.04303
SRP54	0.827479	0.437264	1.565922	0.560636
CHD8	1.055686	0.621122	1.794288	0.841292
PCK2	1.206267	0.848678	1.714524	0.295859
PSMA6	0.93373	0.57747	1.509778	0.779729
NFKBIA	1.292151	0.997671	1.673551	0.052101
PSME2	2.081149	1.400542	3.092502	0.000287
BRMS1L	0.734766	0.507848	1.063076	0.101965
REC8	1.323945	1.027364	1.706143	0.030114
TM9SF1	0.924972	0.446226	1.917355	0.833899
SEC23A	0.851708	0.518632	1.398691	0.525946
GMPR2	1.24991	0.699608	2.23307	0.4512
PNN	1.040439	0.647059	1.672973	0.870056
RABGGTA	1.669563	1.123398	2.481257	0.011226
NFATC4	1.368382	0.941338	1.989159	0.100337
PLTP	0.969602	0.794119	1.183863	0.761856
PCIF1	1.476915	0.932276	2.339733	0.096668
GSS	1.794708	1.055382	3.051952	0.030854
MMP9	0.931352	0.804963	1.077587	0.3392
TRPC4AP	0.943476	0.560084	1.589308	0.826896
PYGB	0.959473	0.680512	1.35279	0.813411
ABHD12	1.35708	0.855688	2.152266	0.194415
PROCR	0.805606	0.532286	1.219271	0.306625
GINS1	1.048432	0.763101	1.440452	0.770429
NINL	1.194506	0.745385	1.914239	0.460104
CD40	1.200043	0.970188	1.484355	0.092768
ZMYND8	0.909554	0.655938	1.261229	0.569756
SGK2	0.799744	0.539696	1.185093	0.26543
IFT52	0.73685	0.470791	1.153266	0.181527
MYBL2	1.336639	1.038205	1.720858	0.024398
NDRG3	0.619995	0.410152	0.937198	0.023351
SLA2	1.018351	0.831985	1.246464	0.860031
NFATC2	1.049198	0.834546	1.319061	0.6809
PABPC1L	1.191118	0.887948	1.597797	0.243218
STK4	1.31538	0.82342	2.101268	0.251378
SALL4	0.8524	0.696344	1.04343	0.121646
ADNP	0.837496	0.541678	1.294863	0.425064
PFDN4	0.768971	0.502093	1.177701	0.227086
DOK5	1.864779	1.120907	3.102311	0.016419
CSTF1	1.18081	0.718369	1.940942	0.51217
RAE1	1.616138	0.82936	3.149297	0.158455
TPD52L2	1.465704	0.99385	2.161582	0.05375

DNAJC5	1.428135	0.989749	2.060692	0.056794
CTSZ	1.259608	1.024762	1.548275	0.028358
PRPF6	1.546433	0.958809	2.494195	0.073858
TUBB1	1.117129	0.941415	1.325639	0.204605
PSMA7	2.072017	1.289259	3.330018	0.002617
SLCO4A1	1.414185	1.121477	1.783291	0.003402
NTSR1	0.953462	0.808708	1.124128	0.570554
TCFL5	0.77778	0.509435	1.187476	0.2444
DIDO1	1.026563	0.651712	1.61702	0.909963
SLC17A9	0.910317	0.734513	1.128199	0.390766
BIRC7	0.849734	0.485043	1.488628	0.569218
ARFGAP1	1.186487	0.81213	1.733405	0.376653
AVP	1.005363	0.811238	1.245941	0.96103
COL20A1	1.141237	0.782019	1.665461	0.493322
EEF1A2	1.03105	0.847667	1.254105	0.759596
PTK6	1.326773	1.038983	1.69428	0.02342
GMEB2	1.140935	0.79593	1.635486	0.472979
CDC25B	1.485388	1.057966	2.085489	0.022287
ISM1	0.611901	0.311229	1.203044	0.154436
RNF24	0.880327	0.581713	1.332232	0.546531
ARFRP1	1.549759	0.898546	2.672933	0.115188
TRIB3	1.142511	0.908532	1.436747	0.254491
RASSF2	1.177143	0.890644	1.555802	0.25175
CSNK2A1	0.874972	0.454068	1.686039	0.689829
CDS2	1.15841	0.719593	1.864822	0.544958
HM13	2.27177	1.180976	4.370059	0.013959
SNPH	1.478523	1.090873	2.003929	0.011715
MYLK2	0.933599	0.630668	1.382038	0.731372
SIRPB1	1.077857	0.961533	1.208254	0.198184
SEC23B	1.672136	1.1172	2.502721	0.012469
FERMT1	1.088375	0.916852	1.291987	0.33312
PLCB4	0.984003	0.833605	1.161537	0.848879
MYL9	1.087279	0.875327	1.350555	0.449432
HCK	1.278427	1.097273	1.489489	0.001629
TM9SF4	1.524367	0.847768	2.740956	0.159047
CRNKL1	1.045991	0.635056	1.722838	0.859814
POFUT1	1.262306	0.746062	2.13577	0.385305
SAMHD1	1.173932	1.03418	1.332569	0.01315
KIF3B	1.60112	0.950181	2.697996	0.077058
NOP56	1.143525	0.754609	1.732884	0.52714
MANBAL	1.788987	0.977054	3.275636	0.059464
IDH3B	1.448401	0.894564	2.345125	0.131865
MAPRE1	0.987197	0.611124	1.594698	0.958
JAG1	0.972131	0.846979	1.115776	0.687704
CDK5RAP1	1.080704	0.563817	2.071454	0.815141
SNTA1	1.234676	0.868144	1.755959	0.240751
OXT	1.072314	0.845027	1.360734	0.565647
E2F1	0.991198	0.750097	1.309795	0.950426
RPRD1B	1.040471	0.583056	1.856734	0.893194
PXMP4	1.133776	0.591195	2.174323	0.705497
CHMP4B	1.972265	1.285289	3.026425	0.001879
BPI	0.994424	0.891845	1.108801	0.919813
CST3	1.022619	0.899809	1.162189	0.731864
ASIP	0.92922	0.656267	1.3157	0.679086
ACTR5	0.919102	0.597895	1.41287	0.700588
AHCY	1.416965	0.94534	2.123879	0.091458
PPP1R16B	1.104231	0.91703	1.329646	0.295513
FAM83D	0.990978	0.768067	1.278582	0.944423

DHX35	1.183865	0.633504	2.212356	0.596759
DNTTIP1	3.552182	1.901851	6.63459	6.99E-05
MAP1LC3/	1.137923	0.915446	1.414469	0.244406
PIGU	1.188054	0.79381	1.778098	0.402268
TNNC2	1.65693	1.135757	2.417258	0.008777
ACOT8	1.347754	0.819214	2.217298	0.240029
ZNF516	0.822368	0.667722	1.012831	0.065767
ADNP2	1.079518	0.703945	1.655467	0.725783
USP14	0.810484	0.494555	1.32823	0.404437
VAPA	1.027699	0.617344	1.710823	0.916317
METTL4	0.865253	0.49967	1.498315	0.60541
LPIN2	0.801028	0.562438	1.140828	0.218812
SMCHD1	0.67473	0.446295	1.020089	0.062091
MYOM1	1.029358	0.718961	1.473763	0.874438
MYL12A	1.680579	1.112889	2.53785	0.013565
CEP76	0.541678	0.318791	0.9204	0.023413
CEP192	0.636656	0.433601	0.934803	0.021224
RNMT	0.653362	0.415923	1.02635	0.064734
SMAD7	0.939153	0.71107	1.240396	0.658302
RNF125	0.8272	0.636019	1.075847	0.15714
ANKRD12	0.957344	0.635709	1.441711	0.834697
POLI	0.749923	0.47983	1.172048	0.206532
MIB1	0.527843	0.390625	0.713262	3.18E-05
RBBP8	1.182383	0.833024	1.678257	0.348471
RIOK3	1.113453	0.723154	1.714404	0.625535
CSTF2	1.866017	1.049439	3.31798	0.033646
VSIG1	0.957471	0.630671	1.453612	0.83834
PSMD10	1.08215	0.65548	1.786549	0.757586
ATG4A	1.473421	0.890748	2.437245	0.131194
STS	1.173087	0.9566	1.438566	0.125105
TBL1X	0.990816	0.79446	1.235702	0.934745
GPR143	0.674714	0.389965	1.167385	0.159526
PGRMC1	1.709611	1.121497	2.606131	0.012666
POLA1	1.007112	0.642807	1.577883	0.975321
MID1	1.14097	0.436353	2.983394	0.787994
NKAP	0.912422	0.499279	1.667432	0.765752
RHOXF1	0.932708	0.678255	1.282622	0.668219
NXT2	1.147469	0.856736	1.536862	0.356144
ALG13	0.795369	0.49806	1.270152	0.337733
PRPS2	0.987934	0.626645	1.557521	0.958317
TLR8	1.100852	1.005665	1.205049	0.037306
MOSPD1	0.923155	0.632717	1.346913	0.678259
AMMECR1	1.119725	0.837698	1.496702	0.44499
CHRDL1	1.112181	0.977369	1.265587	0.1068
WDR13	1.207727	0.826695	1.764381	0.329115
SUV39H1	1.305619	0.848391	2.009261	0.225339
SRPX	0.994554	0.749251	1.320168	0.969853
XIAP	0.867783	0.567554	1.326832	0.512724
STAG2	0.85195	0.644024	1.127006	0.26169
ATP11C	1.027823	0.750171	1.408239	0.864375
MCF2	1.003484	0.647884	1.55426	0.98757
ABCD1	1.576909	1.181212	2.105161	0.002003
CCDC22	1.743328	1.119801	2.714049	0.013856
CACNA1F	1.433443	1.032107	1.990839	0.031672
SYP	1.792581	1.053514	3.050122	0.031382
PLP2	1.126922	0.890691	1.425808	0.319479
BMX	0.818381	0.669827	0.999883	0.049866
PLS3	0.957111	0.764085	1.1989	0.702874

RENBP	1.541343	1.183667	2.0071	0.00132
ELF4	1.397383	1.02205	1.910552	0.036027
SMARCA1	0.915703	0.741911	1.130205	0.412157
MTMR8	0.952465	0.70493	1.286923	0.751117
ASB9	1.041143	0.866512	1.250967	0.666893
ZC3H12B	0.838117	0.60886	1.153697	0.278764
RBBP7	1.494927	0.885671	2.523293	0.132222
KCND1	1.014069	0.699561	1.469975	0.941205
SLC25A14	0.841462	0.473041	1.496823	0.556936
FMR1	1.001684	0.623214	1.609996	0.994454
PIM2	1.133335	0.833806	1.540463	0.42412
SCML2	0.993587	0.810379	1.218214	0.95067
SLC35A2	1.009497	0.652821	1.561045	0.966102
PQBP1	1.871571	1.102092	3.1783	0.020355
PCSK1N	1.523198	1.079582	2.149102	0.016577
EMD	1.737258	1.14776	2.629527	0.009012
TAZ	1.4153	0.940335	2.13017	0.095901
PGK1	1.499632	0.9536	2.358323	0.079383
GATA1	1.011932	0.882831	1.159911	0.86475
MAGT1	0.88187	0.537709	1.446313	0.618466
SMS	0.835485	0.506094	1.379259	0.4822
PHEX	1.413384	1.030666	1.938216	0.031758
UBL4A	1.831952	1.18328	2.836224	0.006636
CD99L2	1.0796	0.729447	1.597833	0.701804
EEA1	0.949779	0.655314	1.376561	0.785526
RP2	1.147885	0.826032	1.595145	0.411341
USP11	1.117484	0.797601	1.565659	0.518542
PCYT1B	0.911717	0.71274	1.166243	0.461885
HTATSF1	1.513882	0.93099	2.461723	0.094584
CD40LG	0.995951	0.780227	1.271321	0.974014
TIMP1	1.033818	0.870111	1.228326	0.705338
GABRE	0.822772	0.71561	0.945983	0.006145
FGD1	0.943678	0.550379	1.618027	0.833099
PIN4	1.075701	0.544675	2.124444	0.833542
PORCN	1.285213	0.898349	1.838677	0.169664
MAGED2	0.997052	0.730996	1.359945	0.985129
RBM3	1.16417	0.74218	1.826097	0.50809
KLF8	1.012103	0.750684	1.364559	0.937103
SYTL4	1.328083	1.139627	1.547704	0.000279
ZDHHC15	1.126298	0.836596	1.516319	0.433064
CENPI	0.820768	0.582528	1.156443	0.258862
GLA	1.199208	0.799644	1.798426	0.379622
ARMCX3	0.820463	0.568338	1.184437	0.2908
BEX4	0.941203	0.768248	1.153095	0.558597
NDFIP2	0.764205	0.613191	0.952408	0.016663
TNFSF13B	1.127432	0.906195	1.402682	0.28185
FNDC3A	0.513312	0.348038	0.75707	0.000769
MLNR	0.796839	0.624304	1.017057	0.068134
CDADC1	0.72119	0.517829	1.004416	0.053126
CAB39L	0.807995	0.501426	1.301999	0.381118
KLF5	1.115373	0.910605	1.366187	0.29139
STK24	1.187168	0.833918	1.690057	0.341047
ACP5	1.013525	0.792151	1.296764	0.91491
DNAJC3	0.87129	0.58995	1.286798	0.488602
ARHGEF7	0.953825	0.628169	1.448308	0.824436
FGF9	0.859809	0.300143	2.463065	0.778489
PARP4	0.848186	0.58666	1.226297	0.381359
MRPS31	0.913642	0.529167	1.577463	0.745842

SLC25A15	1.064058	0.714791	1.583987	0.7597
KPNA3	0.911651	0.566154	1.46799	0.703536
FLT1	0.806777	0.500543	1.300367	0.378011
DGKH	1.074879	0.846791	1.364403	0.552931
KATNAL1	0.82448	0.576174	1.179796	0.291138
INTS6	0.969018	0.54053	1.737177	0.915843
DHRS12	1.261511	0.892529	1.783033	0.188198
TSC22D1	0.99705	0.771692	1.28822	0.981969
CLN5	0.648662	0.336091	1.251929	0.196974
OLFM4	0.980876	0.825804	1.165068	0.825935
MSLN	0.9101	0.804108	1.030064	0.135936
MGRN1	1.192821	0.816488	1.742614	0.36194
ZNF629	0.890592	0.599778	1.322412	0.565657
TRADD	1.75874	1.133578	2.728676	0.011754
HSF4	1.039971	0.765055	1.413676	0.802418
CORO1A	1.447857	1.110615	1.887503	0.00623
MAPK3	1.481726	0.99271	2.211635	0.054335
GDPD3	0.926811	0.653516	1.314395	0.669833
ELMO3	1.098814	0.743796	1.623286	0.636001
PHKB	0.848907	0.521608	1.381581	0.509767
LYRM1	1.174784	0.798954	1.727407	0.412838
NUTF2	1.324223	0.781787	2.243025	0.296291
NUP93	1.878928	1.030383	3.426271	0.039628
CENPT	1.102511	0.754095	1.611908	0.614556
TSNAXIP1	0.824515	0.474055	1.434063	0.494409
NFAT5	0.56004	0.401526	0.781132	0.000638
LONP2	0.822324	0.503652	1.342627	0.434173
N4BP1	1.389437	0.781984	2.468766	0.262097
ARL2BP	2.185422	1.172097	4.074809	0.013912
PLLP	1.867407	0.807019	4.321096	0.144547
CCL22	2.539634	1.652695	3.90256	2.12E-05
DHODH	0.678119	0.420599	1.093308	0.110957
CCL17	1.066134	0.456644	2.489119	0.882319
CTCF	0.899512	0.507621	1.593951	0.716753
ACD	1.304617	0.875583	1.943877	0.191234
POLR2C	2.148445	1.098966	4.200144	0.02536
PARD6A	1.358135	0.916423	2.012748	0.127227
ZNF821	1.264042	0.725884	2.20118	0.407699
MMP15	0.951564	0.814327	1.111931	0.532111
CYB5B	0.754471	0.399594	1.424512	0.384944
NME3	1.2875	0.985512	1.682025	0.063889
NDRG4	0.816217	0.448282	1.48614	0.506569
PSMD7	1.395841	0.832573	2.340181	0.205888
SETD6	0.679809	0.393589	1.174169	0.166317
SLC38A7	1.187484	0.742547	1.89903	0.473166
VAC14	1.286671	0.801407	2.065771	0.296731
HAS3	0.578513	0.377456	0.886666	0.012002
COG4	1.513544	0.971991	2.356828	0.066619
SMPD3	1.371591	1.015452	1.852636	0.039405
SLC7A6OS	0.991468	0.55825	1.760873	0.976673
SLC7A6	1.336747	0.85857	2.081245	0.198828
PLA2G15	1.409858	0.999781	1.988135	0.050146
ESRP2	0.764333	0.545501	1.070952	0.118371
WDR59	0.776837	0.52739	1.144267	0.201264
MON1B	1.416529	0.834529	2.404414	0.197088
AXIN1	1.131456	0.787546	1.625545	0.504092
HCFC1R1	1.465708	1.01105	2.124822	0.043595
MLYCD	0.510867	0.340678	0.766076	0.001158

MPG	1.358855	0.986865	1.871063	0.060251
HSDL1	0.603311	0.443838	0.820084	0.001254
TAF1C	0.979841	0.692786	1.385839	0.908339
NAGPA	1.243093	0.847339	1.823687	0.265789
WFDC1	0.811825	0.608635	1.08285	0.156074
COTL1	1.32591	1.100556	1.597409	0.002997
USP10	1.21296	0.719877	2.043781	0.46829
CRISPLD2	1.085944	0.935056	1.26118	0.280048
TSC2	1.121676	0.746176	1.68614	0.580871
ZNF500	1.085182	0.67871	1.735087	0.732801
NME4	1.107188	0.812471	1.508811	0.519043
ABCC1	1.101611	0.80679	1.504168	0.542545
NOMO3	0.795899	0.498498	1.270727	0.33892
LMF1	0.884458	0.618837	1.264089	0.500421
FOXF1	0.477758	0.142914	1.597137	0.230303
MTHFSD	0.909876	0.533843	1.550784	0.728465
CLCN7	1.151975	0.854521	1.552971	0.353225
HAGHL	0.922274	0.713404	1.192297	0.536866
SLC7A5	0.936485	0.782118	1.121319	0.475208
METRN	0.799519	0.601439	1.062836	0.123462
FBXO31	0.887252	0.584395	1.347064	0.574446
STUB1	1.50203	1.044936	2.159074	0.027993
RHBDL1	0.98424	0.755151	1.282826	0.906452
NUBP1	1.569686	0.895742	2.750694	0.115191
UBE2I	1.150724	0.630561	2.099981	0.64736
MEFV	1.13473	1.018323	1.264444	0.022094
CRYM	1.384657	0.888644	2.157527	0.150366
EEF2K	0.711999	0.458051	1.106736	0.131213
GSPT1	1.037653	0.631981	1.703729	0.883845
ZNF174	1.02924	0.591558	1.790752	0.91876
CLUAP1	0.828809	0.556631	1.234076	0.355251
UBFD1	0.705034	0.458898	1.083188	0.110658
PRSS33	0.872407	0.350178	2.173452	0.769457
EARS2	0.842317	0.539517	1.315063	0.450267
GGA2	1.391015	0.949286	2.038293	0.090458
AQP8	0.855779	0.425636	1.720622	0.662073
CPPED1	1.164597	0.922481	1.470259	0.200051
USP31	1.099971	0.76108	1.589761	0.612109
HMOX2	1.812847	1.111213	2.957501	0.017207
DNAJA3	1.792533	0.99019	3.245005	0.053929
BFAR	1.102203	0.538577	2.255669	0.789988
RRN3P2	1.209484	0.809155	1.807876	0.353722
RBL2	0.774316	0.535641	1.11934	0.173718
QPRT	0.907111	0.734293	1.120601	0.365964
XYLT1	0.919368	0.729391	1.158825	0.476566
PYCARD	1.720254	1.284803	2.30329	0.00027
RPGRIP1L	0.57132	0.374263	0.872132	0.009489
MAZ	0.99355	0.734872	1.343286	0.966459
STX4	1.759733	1.060883	2.918947	0.028607
CDIPT	1.649698	1.0225	2.661618	0.040254
BCKDK	2.477864	1.658086	3.70295	9.56E-06
NOMO1	0.971495	0.633972	1.488712	0.894355
IL21R	1.283448	0.963868	1.708988	0.087623
SYT17	1.48604	1.116814	1.977334	0.006567
TMC5	0.588029	0.209563	1.649995	0.313132
RNF40	1.317968	0.842601	2.061521	0.226424
AQP9	1.075307	0.968081	1.194408	0.175512
AAGAB	1.718127	1.009464	2.924284	0.046078

IQCH	0.810448	0.472473	1.390186	0.445239
LACTB	1.187742	0.838804	1.681836	0.332301
CORO2B	0.714118	0.561241	0.908638	0.006154
CSK	2.027055	1.401233	2.932383	0.000176
HERC1	1.095878	0.767701	1.564344	0.61413
TRIP4	1.239976	0.632197	2.432059	0.531442
MTFMT	1.665561	0.942123	2.944515	0.07928
ACSBG1	1.125913	0.853931	1.484522	0.400541
IGDCC4	0.458391	0.266561	0.788268	0.0048
RAB11A	1.249708	0.725971	2.151283	0.421186
CTSH	1.146221	0.920588	1.427156	0.222401
TTC23	0.80212	0.636674	1.010558	0.061365
CD276	1.20817	1.027959	1.419974	0.021759
FAH	1.136352	0.799457	1.615216	0.476189
RPAP1	1.000971	0.642292	1.559948	0.99658
HOMER2	0.919596	0.729336	1.159487	0.478483
EHD4	1.293864	1.03277	1.620966	0.025068
TMEM87A	0.558392	0.387618	0.804403	0.001756
CEP152	0.962985	0.643743	1.440543	0.854361
ATP8B4	0.867999	0.715634	1.052805	0.150588
DTWD1	0.889559	0.559628	1.414001	0.620655
TGM5	0.872763	0.714712	1.065766	0.181841
FAM189A1	0.848915	0.358901	2.007958	0.709224
GABPB1	0.984982	0.64079	1.514051	0.945001
BMF	1.36286	1.031424	1.800799	0.029436
DMXL2	0.92891	0.800805	1.077509	0.330066
SCG3	1.130452	0.485946	2.62976	0.775909
DNAJC17	1.906978	1.003219	3.624898	0.048865
EIF3J	1.024483	0.604195	1.737133	0.928461
SPG11	0.799136	0.515529	1.238764	0.316062
RHOV	1.091709	0.519219	2.295424	0.816999
VPS18	1.285753	0.850059	1.94476	0.233846
OIP5	0.754809	0.473761	1.202582	0.236534
SLC30A4	0.816463	0.537708	1.23973	0.341328
MYEF2	1.124877	0.953017	1.327728	0.1642
SGK3	1.034102	0.688024	1.554258	0.871857
CSPP1	0.465801	0.287569	0.754498	0.001904
ZDHHC2	1.025188	0.77736	1.352025	0.860148
BRF2	1.251057	0.754272	2.07504	0.385601
TRIM35	1.663455	0.955183	2.896914	0.072182
ZFAND1	0.890857	0.611426	1.297994	0.547302
CA2	0.991755	0.864587	1.137629	0.905872
FZD3	0.942545	0.717799	1.237661	0.670286
INTS9	1.492904	0.834683	2.670189	0.176753
RIPK2	1.351345	0.90578	2.016088	0.140173
NBN	0.953728	0.607583	1.497075	0.836837
DECR1	1.295968	0.812306	2.067611	0.276697
SFRP1	0.849735	0.517246	1.395949	0.520283
LAPTM4B	1.026861	0.912411	1.155666	0.660207
UBE2W	0.588615	0.376687	0.919778	0.019957
POP1	1.143832	0.728759	1.795314	0.559036
NIPAL2	0.952736	0.73959	1.227309	0.707862
IKBKB	0.945887	0.618514	1.446537	0.79743
PLAT	0.562645	0.291604	1.085614	0.086345
DKK4	0.701943	0.371687	1.325642	0.275287
STK3	0.526595	0.291127	0.952513	0.033934
GDAP1	1.837193	1.094261	3.084527	0.021409
RAB2A	1.227056	0.685563	2.19625	0.490873

EIF3E	0.809551	0.56593	1.158046	0.247415
NDRG1	0.813164	0.618375	1.069312	0.138791
IL7	1.242401	1.049852	1.470265	0.011531
ARMC1	0.78268	0.494789	1.238078	0.294991
TRPS1	1.137686	0.991857	1.304955	0.065308
SPAG1	1.656166	1.217566	2.252761	0.001309
CHRAC1	2.126955	1.189226	3.804103	0.010953
NCALD	1.324467	1.049438	1.671572	0.017967
SNX16	0.702552	0.45079	1.094922	0.118901
UBR5	0.779986	0.526584	1.155328	0.215107
GSDMD	1.165861	0.816811	1.664072	0.397924
EEF1D	0.903314	0.582832	1.400019	0.649221
SQLE	1.28376	0.940642	1.752039	0.115418
SH2D4A	1.131458	0.915799	1.397902	0.252322
INTS10	1.094845	0.686302	1.746586	0.703757
ERI1	1.286821	0.824144	2.009246	0.267334
SLC39A14	1.13949	0.883809	1.469139	0.313821
MTMR9	0.923422	0.534355	1.595771	0.775299
LEPROTL1	0.822008	0.507442	1.331574	0.425792
DCTN6	0.975384	0.524298	1.814567	0.937277
R3HCC1	1.514708	0.8735	2.626605	0.139297
GSR	1.188374	0.813708	1.735551	0.37179
TNFRSF10/	1.026183	0.761519	1.38283	0.865145
UBXN8	0.893832	0.532213	1.501158	0.671358
PPP2CB	0.960151	0.499937	1.844014	0.902799
ERICH1	0.988136	0.679651	1.436639	0.950161
TUSC3	1.304951	0.701711	2.426779	0.400422
KLHDC4	0.906466	0.596325	1.377906	0.645791
MCM4	1.211879	0.902867	1.626651	0.200689
KCTD9	1.13137	0.66624	1.921225	0.647781
ASAH1	0.928753	0.654773	1.317375	0.678557
BNIP3L	0.98816	0.680769	1.43435	0.950047
MAN2B1	1.127431	0.820052	1.550026	0.460219
KCNN4	1.091995	0.800932	1.488831	0.577909
TULP2	0.806597	0.619543	1.050126	0.110348
NUCB1	1.200938	0.877575	1.643451	0.252614
DHDH	1.142474	0.65208	2.001669	0.641554
GYS1	1.208218	0.775448	1.882515	0.403171
MAP4K1	2.250635	1.494665	3.388958	0.000103
ECH1	1.811712	1.216274	2.698653	0.003467
HNRNPL	0.973319	0.51938	1.824	0.932745
NFKBIB	1.656665	1.109477	2.473724	0.013593
LHB	1.075177	0.684477	1.688889	0.753067
SARS2	1.163172	0.664909	2.034818	0.596304
SNRNP70	0.985088	0.676976	1.433431	0.937425
CLPTM1	1.392753	0.862037	2.250205	0.175913
RELB	1.297242	0.995271	1.690834	0.054242
LIN7B	0.740253	0.503595	1.088125	0.125952
FCGRT	1.019277	0.86325	1.203505	0.821794
PIH1D1	1.232193	0.759521	1.999023	0.397687
CKM	1.118049	0.843196	1.482495	0.438249
ARHGEF18	1.245546	0.822514	1.886148	0.299691
PPP1R13L	1.451995	1.065391	1.978888	0.018228
PEX11G	0.909183	0.701564	1.178245	0.471628
ERCC2	1.45537	0.953534	2.221316	0.081961
DOT1L	1.117265	0.750293	1.663725	0.585197
PLEKHJ1	1.832829	1.131022	2.970114	0.0139
KLC3	1.1013	0.464456	2.611357	0.826615

CD37	2.112876	1.483271	3.00973	3.41E-05
SF3A2	1.377879	0.976631	1.94398	0.067954
AMH	0.955878	0.752132	1.214815	0.712164
LYL1	1.145252	0.81219	1.614896	0.439211
OAZ1	2.095643	1.372979	3.198677	0.000606
TRMT1	1.293575	0.903891	1.851261	0.159291
STX10	1.61658	1.082641	2.413848	0.018867
RETN	0.917888	0.837646	1.005818	0.066406
FCER2	1.085183	0.941605	1.250654	0.258901
DMPK	1.09769	0.831451	1.449182	0.510774
TBC1D17	1.077675	0.737549	1.574653	0.699039
IL4I1	1.392832	1.147527	1.690576	0.000802
CCDC130	1.241137	0.818037	1.88307	0.309789
PTOV1	0.864401	0.603701	1.237679	0.426236
SGTA	1.319945	0.829697	2.09987	0.241262
KIR3DX1	1.249886	1.004187	1.555702	0.045784
LILRB1	1.175856	1.035026	1.335848	0.012814
MED25	0.968115	0.663359	1.412879	0.866578
LILRA1	1.185246	1.055343	1.331138	0.004112
SNAPC2	1.640285	1.132143	2.376497	0.008895
TIMM44	1.142798	0.706264	1.849149	0.586702
CCDC61	0.834315	0.49875	1.395654	0.490163
IL27RA	1.620423	1.100351	2.386304	0.014517
ASF1B	1.385576	1.024323	1.874235	0.034355
TNNT1	1.124816	0.983381	1.286593	0.086249
VRK3	2.190833	1.003814	4.781509	0.048893
FAM32A	1.268038	0.710593	2.262787	0.42158
MED26	1.458694	0.879485	2.419356	0.143604
OLFM2	0.803525	0.560533	1.151852	0.233833
RASAL3	1.226735	0.914855	1.644937	0.172131
AKAP8	1.010848	0.606959	1.683499	0.96693
ILVBL	1.351606	0.878237	2.080122	0.170779
ZNF419	0.848053	0.549815	1.308064	0.456031
SYDE1	0.744786	0.576369	0.962415	0.024268
AURKC	0.636722	0.393537	1.030184	0.06594
POP4	1.846424	0.894652	3.810734	0.097148
CCNE1	1.096068	0.819962	1.465148	0.535607
PDCD5	0.683063	0.393638	1.185289	0.175267
ANKRD27	0.674274	0.511222	0.88933	0.005265
RPS16	1.388388	0.868792	2.218736	0.170088
TIMM50	1.509798	0.81463	2.798192	0.19064
FBL	1.131927	0.752684	1.702253	0.551676
DYRK1B	1.082186	0.781649	1.498276	0.634192
CLC	1.033713	0.953994	1.120094	0.418077
GPI	0.893496	0.653167	1.222254	0.48114
AKT2	0.809948	0.525771	1.24772	0.339024
PLD3	1.267746	1.018515	1.577964	0.033651
PRX	1.082103	0.899114	1.302334	0.403814
PIAS4	0.877423	0.508009	1.515467	0.639078
NUMBL	0.901151	0.680892	1.19266	0.466695
EBI3	1.390856	1.140503	1.696165	0.00112
SHD	0.898893	0.796512	1.014433	0.084042
TBCB	1.826842	1.161866	2.872407	0.009062
FSD1	1.326928	0.860347	2.046545	0.200706
POLR2I	1.404127	1.042244	1.891662	0.025611
CLIP3	0.908027	0.764304	1.078777	0.272449
SLC1A5	1.425935	0.985747	2.062689	0.059599
PRKD2	1.282461	0.934605	1.759787	0.123309

TJP3	0.866984	0.536341	1.401462	0.560218
APLP1	1.163252	0.80024	1.690937	0.428163
CCDC9	0.958988	0.617353	1.489679	0.852167
HNRNPUL	1.969475	1.027011	3.776818	0.041331
FZR1	1.030267	0.705819	1.503857	0.877197
BBC3	1.298208	0.913267	1.845402	0.145842
TGFB1	1.877843	1.380692	2.554006	5.92E-05
DENND3	0.946159	0.710016	1.260841	0.705586
CEACAM4	1.014402	0.865824	1.188476	0.85954
MRPL4	1.69679	1.137328	2.531456	0.009586
SIGLEC8	0.821006	0.580818	1.160521	0.264036
CD79A	1.144605	0.945099	1.386225	0.166933
RPS19	1.283166	0.854139	1.927689	0.229865
NKG7	1.183628	0.974409	1.437771	0.089369
ICAM5	0.992608	0.857955	1.148394	0.920542
ETFB	2.613619	1.465488	4.661247	0.001135
CD33	1.017106	0.834002	1.240411	0.866981
TYK2	1.395399	0.981899	1.983035	0.063156
CDC37	1.678634	1.136686	2.478972	0.009213
NAPA	1.2717	0.87267	1.853188	0.210916
RABAC1	1.369982	0.903911	2.076368	0.137865
PTPRS	0.916834	0.784487	1.07151	0.274999
CNFN	0.983699	0.741315	1.305335	0.909342
MEGF8	0.965578	0.680367	1.370351	0.84453
KDELR1	1.213068	0.68597	2.145186	0.506643
CYTH2	0.657425	0.434973	0.993644	0.046567
GRWD1	1.039448	0.673657	1.603863	0.861207
GRIN2D	1.117314	0.830224	1.503678	0.46413
CLEC11A	0.830047	0.751232	0.917131	0.000253
CCDC114	0.913296	0.608243	1.371345	0.66189
CARD8	0.867646	0.552589	1.362329	0.537397
LIG1	1.186602	0.815675	1.726209	0.370984
SIGLEC6	1.036714	0.878483	1.223446	0.669593
ZNF175	0.964558	0.698881	1.331231	0.826248
PLA2G4C	1.186461	0.920298	1.529601	0.187122
SIGLEC5	1.059743	0.79858	1.406315	0.687721
HAS1	1.391955	0.703937	2.752432	0.341747
RAB3D	1.363105	1.046488	1.775514	0.021624
DBP	0.902527	0.531993	1.53114	0.703733
TMEM205	1.52846	1.087749	2.14773	0.014501
CAPS	1.072918	0.765862	1.503081	0.682413
FAM83E	1.43256	0.797746	2.572533	0.228801
RASIP1	1.119741	0.75879	1.652396	0.568914
BCAT2	1.175616	0.789831	1.749833	0.425281
MIER2	1.085369	0.753424	1.563565	0.660057
PLEKHA4	1.199182	0.894785	1.607132	0.224051
PPP2R1A	1.815609	1.0318	3.194841	0.03859
TNPO2	1.053457	0.715922	1.55013	0.791588
GCDH	0.765533	0.513778	1.140649	0.189124
LILRB5	1.140228	0.858744	1.513977	0.364301
KLF1	0.90066	0.776219	1.045051	0.167857
DNASE2	0.922249	0.730867	1.163746	0.495194
MAST1	0.689879	0.497267	0.957098	0.026251
LENG1	1.872067	0.973176	3.601234	0.060312
PRPF31	1.788427	1.032119	3.098936	0.038202
TFPT	1.683999	1.001483	2.831654	0.04935
JAK3	0.821483	0.64791	1.041557	0.104429
RPL18A	1.452386	1.009951	2.08864	0.044074

SLC5A5	1.369042	1.075867	1.742107	0.010627
KCNN1	1.132654	0.727655	1.763068	0.581127
ARRDC2	1.727294	1.211934	2.461804	0.002501
RAB3A	1.76626	1.22882	2.538756	0.002118
ISYNA1	1.129189	0.897644	1.42046	0.299407
ELL	1.348627	0.866239	2.099647	0.185436
CRTC1	1.456088	0.978071	2.167729	0.064206
COPE	1.323014	0.927822	1.886532	0.122067
DDX49	1.101733	0.743462	1.632654	0.629247
ETV2	0.868863	0.542089	1.392617	0.559209
ARMC6	1.474691	0.99351	2.18892	0.053898
TMEM147	1.265719	0.766878	2.089048	0.356671
HAMP	0.803478	0.463357	1.39326	0.435928
USF2	1.512693	0.984677	2.323848	0.058829
LSR	1.276127	0.96425	1.688877	0.088129
FKBP8	1.408398	1.034967	1.916567	0.02936
ZNF14	0.682818	0.458577	1.016711	0.06033
SCN1B	0.879831	0.635275	1.218533	0.441015
PBX4	1.004413	0.753388	1.33908	0.976058
ERF	1.221901	0.855381	1.745469	0.270705
GSK3A	1.974713	0.982867	3.967463	0.055951
ATP13A1	1.639884	1.053143	2.553519	0.028587
ZNF574	1.009393	0.720089	1.414928	0.956732
GRIK5	1.063592	0.909488	1.243807	0.440122
SIPA1L3	0.721715	0.505168	1.031088	0.073171
ZNF85	0.701113	0.522816	0.940216	0.017706
ETHE1	1.04349	0.696624	1.56307	0.83641
CADM4	1.068268	0.736615	1.549244	0.727695
AVL9	0.524453	0.318292	0.864145	0.011308
RUNDC3B	0.633764	0.370998	1.082639	0.09505
GTPBP10	0.557842	0.369506	0.842174	0.005482
RASA4	0.777999	0.376387	1.60814	0.498026
CDK6	0.713409	0.568216	0.895702	0.00363
PMPCB	0.77748	0.497196	1.215768	0.269838
DNAJC2	0.715395	0.471161	1.086233	0.116002
TFPI2	10.53528	0.733911	151.2337	0.083209
BET1	0.795908	0.509356	1.24367	0.316156
NAMPT	1.063004	0.910112	1.241581	0.440608
PIK3CG	1.125256	0.784389	1.61425	0.521551
PON2	0.930207	0.802359	1.078427	0.337525
ITGB8	0.824449	0.600052	1.132761	0.233687
HBP1	0.571881	0.380162	0.860284	0.007311
DUS4L	0.587352	0.324059	1.064566	0.079472
SP4	0.757733	0.531486	1.080291	0.125237
WDR91	1.428791	1.009273	2.022687	0.044219
DNAH11	0.653409	0.416343	1.02546	0.064223
CBLL1	0.708716	0.439229	1.143544	0.1584
MTPN	1.120611	0.78004	1.609879	0.537855
MPP6	1.064202	0.887233	1.276469	0.502492
ZC3HAV1	0.707416	0.410123	1.220212	0.213341
TTC26	0.591533	0.398984	0.877006	0.008971
OGDH	1.334397	0.941627	1.890999	0.104841
ADAP1	1.233126	0.982576	1.547565	0.070562
TFEC	0.939844	0.745277	1.185205	0.600118
CAV2	0.823798	0.648946	1.045762	0.111303
CAV1	0.950351	0.805876	1.120727	0.545004
MET	0.84498	0.404929	1.763248	0.653572
RNF32	0.63228	0.371831	1.075161	0.090566

LMBR1	0.653577	0.407961	1.047067	0.076945
HOXA1	1.293868	1.02533	1.632738	0.029952
DNAJB6	0.622193	0.446987	0.866076	0.004923
HOXA2	1.185867	1.011234	1.390657	0.035958
HOXA3	1.190722	1.064439	1.331987	0.002276
LFNG	1.261468	1.065838	1.493005	0.006901
HOXA5	1.135112	1.04952	1.227685	0.001533
HOXA6	1.154302	1.052605	1.265824	0.002292
IQCE	1.443047	1.052242	1.978998	0.022847
TSPAN12	0.983809	0.478773	2.021584	0.964568
SSBP1	0.591176	0.348081	1.004045	0.051772
HOXA13	1.020101	0.766418	1.357754	0.891487
HIBADH	0.752084	0.485359	1.165385	0.2023
TAX1BP1	0.744605	0.531426	1.0433	0.086594
CPVL	1.124227	0.997009	1.267679	0.055996
CHN2	0.904822	0.639417	1.280389	0.572324
GRB10	0.986521	0.839487	1.159306	0.869094
ABHD11	1.681288	1.242424	2.275174	0.000762
FKBP14	0.927664	0.760354	1.131788	0.459327
PLEKHA8	0.836217	0.668671	1.045744	0.116907
STX1A	1.204843	0.821398	1.767288	0.340397
NOD1	0.722043	0.506407	1.029502	0.071967
CRHR2	1.276343	0.788263	2.066635	0.321033
EPHB6	0.852131	0.638578	1.137101	0.276996
CASP2	0.670707	0.453624	0.991676	0.0453
CHCHD2	1.643315	0.976442	2.765637	0.061456
CCL24	0.939906	0.754391	1.171041	0.58063
HSPB1	1.221722	0.996958	1.49716	0.053533
NPTX2	1.048143	0.928367	1.183371	0.447587
PDAP1	1.385589	0.720309	2.665324	0.328539
BUD31	0.982024	0.56572	1.704681	0.948602
PTCD1	0.672685	0.398248	1.136239	0.138231
CYP3A5	0.537245	0.343793	0.839552	0.006376
ZKSCAN1	0.814878	0.572833	1.159196	0.254934
EIF3B	1.025723	0.665263	1.581492	0.908467
SNX8	1.940377	1.287673	2.923929	0.001532
NUDT1	1.529298	1.060647	2.205024	0.022886
TAF6	1.064495	0.647909	1.748936	0.805123
WASL	0.675491	0.433473	1.052635	0.083038
AIMP2	1.258959	0.720358	2.200265	0.418833
TFR2	0.974475	0.825733	1.15001	0.759624
FSCN3	1.040848	0.532382	2.034938	0.906826
MOSPD3	1.271343	0.934617	1.729385	0.126204
PCOLCE	0.909712	0.671713	1.232039	0.54087
FBXO24	0.55434	0.291136	1.055494	0.07256
RBM28	0.662495	0.44237	0.992155	0.045696
USP42	0.584498	0.325272	1.050313	0.072525
IMPDH1	1.186877	0.881581	1.597898	0.258801
AGFG2	0.909313	0.605123	1.366414	0.6473
LSM5	0.941074	0.574444	1.5417	0.809441
SERPINE1	0.844446	0.727649	0.979989	0.026007
AP1S1	1.186116	0.814642	1.726981	0.373224
C1GALT1	0.632836	0.430837	0.929544	0.019679
PLOD3	0.970113	0.733213	1.283555	0.831785
RPA3	1.003372	0.630006	1.598009	0.988688
ZNHIT1	0.978207	0.57665	1.659392	0.934872
CLDN15	0.811549	0.580657	1.134254	0.221535
GLCCI1	1.043154	0.834762	1.30357	0.710215

PHF14	0.69522	0.478021	1.01111	0.057151
NRF1	0.588257	0.310649	1.113944	0.10337
TMEM106I	0.97879	0.698782	1.370999	0.900767
EZH2	0.756537	0.521821	1.096828	0.14095
ZNF862	1.213345	0.87879	1.675265	0.240024
SFRP4	0.742123	0.468974	1.174365	0.202807
MEST	0.887477	0.784753	1.003649	0.057179
ANKMY2	0.716657	0.487822	1.052837	0.089583
TSPAN13	1.002678	0.863617	1.16413	0.971993
RARRES2	0.874132	0.665889	1.147497	0.332562
AGR2	0.986015	0.81522	1.192594	0.884619
AHR	1.156514	0.9889	1.352537	0.068722
CHCHD3	1.212383	0.723432	2.031804	0.464751
GIMAP2	1.080783	0.843794	1.384332	0.538478
TMEM176I	0.967987	0.870784	1.07604	0.546769
PSMA2	0.48791	0.276014	0.862479	0.013551
MRPL32	0.642497	0.356001	1.159556	0.141956
BLVRA	1.069806	0.913782	1.252469	0.401498
STAG3L4	0.473903	0.27339	0.821478	0.0078
RHEB	0.698264	0.398666	1.223011	0.209128
PRKAG2	0.66105	0.419872	1.040763	0.073868
AEBP1	0.855271	0.73189	0.999452	0.049199
POLD2	1.090839	0.744571	1.598141	0.65543
BCL7B	1.326636	0.829587	2.121494	0.238002
YKT6	1.071464	0.6211	1.848391	0.804053
TBL2	0.673392	0.369147	1.228391	0.197304
CLIP2	1.010269	0.859185	1.187921	0.901619
EIF4H	1.09712	0.651391	1.847848	0.727491
LIMK1	1.559918	1.09668	2.21883	0.013386
FKTN	0.703979	0.457037	1.084346	0.111259
FSD1L	0.954376	0.646661	1.408516	0.814097
CNTNAP3	0.652338	0.287792	1.478656	0.306231
SPIN1	0.778457	0.547706	1.106425	0.162665
PRUNE2	1.177699	0.897462	1.545442	0.238123
MEGF9	1.110348	0.941455	1.309539	0.213737
TRIM14	1.046303	0.709037	1.543998	0.819652
CORO2A	1.008037	0.821	1.237683	0.939066
TGFBR1	0.921099	0.635459	1.335133	0.664334
SEC61B	1.276008	0.838353	1.942138	0.255424
C5	0.89451	0.675727	1.184128	0.435986
OGN	0.926746	0.676439	1.269678	0.635791
ASPN	0.827454	0.64493	1.061634	0.136333
ECM2	0.955443	0.721571	1.265117	0.750327
TLE4	1.20758	0.910279	1.601981	0.190856
LHX6	1.078813	0.923722	1.259944	0.338066
PTGR1	0.867373	0.713344	1.054661	0.153744
SUSD1	1.006785	0.704557	1.438656	0.970383
AKNA	1.259589	0.871293	1.82093	0.219715
TNFSF8	1.217458	0.997785	1.485495	0.052609
DNM1	0.816669	0.705377	0.945521	0.00674
ENG	1.217318	0.994537	1.490002	0.056541
AK1	1.927653	1.262571	2.943078	0.002367
CDC37L1	0.756412	0.453545	1.261527	0.284738
TBC1D13	1.426074	0.95655	2.126063	0.081518
DOCK8	1.089033	0.765366	1.549574	0.635521
KANK1	0.758771	0.593287	0.970413	0.027861
TESK1	1.004686	0.736048	1.371372	0.976504
FUBP3	0.766992	0.361398	1.627778	0.489596

CREB3	3.758782	1.931495	7.31477	9.71E-05
RGP1	1.516997	0.897434	2.564288	0.119726
DDX58	1.156812	0.802229	1.668121	0.435388
EDF1	1.655676	0.990044	2.768828	0.054628
PIP5K1B	0.957687	0.781	1.174347	0.677784
GLIS3	0.594223	0.397464	0.888384	0.011188
BAG1	1.631974	0.867776	3.069156	0.128543
RAPGEF1	1.248876	0.88525	1.761867	0.205594
NPDC1	1.010649	0.900654	1.134077	0.857013
APBA1	0.885032	0.725416	1.079768	0.228735
SETX	0.966672	0.681121	1.371936	0.849504
PTGDS	1.018028	0.837362	1.237673	0.857743
ABCA2	1.002647	0.837892	1.199798	0.97697
SHB	1.622269	1.037134	2.537527	0.034031
UBE2R2	1.346536	0.737659	2.457991	0.332541
EXOSC3	0.856733	0.521098	1.408548	0.542153
ZFAND5	0.83547	0.564527	1.236451	0.368774
DVL1	1.182866	0.851271	1.643626	0.317029
PDLIM1	1.146096	0.996144	1.31862	0.056656
CCNJ	0.754616	0.482228	1.180863	0.217832
DNTT	0.991911	0.926153	1.062338	0.816482
GATA3	0.99817	0.820943	1.213658	0.98535
HPS1	1.822896	1.202199	2.76406	0.004698
PHYH	1.242759	0.849316	1.818462	0.263129
RASSF4	1.067209	0.919482	1.238671	0.392171
DNMBP	1.301916	0.961301	1.763221	0.088209
RAB11FIP2	0.658371	0.390185	1.11089	0.117353
CXCL12	0.917796	0.836959	1.00644	0.068229
ERLIN1	0.80016	0.564942	1.133312	0.209362
EIF3A	0.772593	0.550382	1.084521	0.135949
CUBN	1.022535	0.730276	1.431757	0.896763
TRDMT1	0.950988	0.617491	1.464603	0.819582
DDX50	1.010664	0.635876	1.606352	0.964213
MAPK8	0.715567	0.446493	1.146796	0.164294
SEC23IP	1.071227	0.62641	1.831912	0.801554
ATE1	0.738371	0.466528	1.168614	0.195391
NSMCE4A	1.756175	0.9489	3.250241	0.072979
PLEKHA1	0.997881	0.694155	1.434502	0.990861
UNC5B	1.120324	0.873399	1.437057	0.37111
CDH23	1.3147	1.050559	1.645254	0.016803
SPOCK2	1.153478	0.954065	1.394571	0.140377
PPP3CB	0.60033	0.335654	1.073712	0.085395
BMPR1A	0.799044	0.567004	1.126045	0.19994
MINPP1	0.969409	0.74878	1.255046	0.813584
ACTA2	1.052442	0.736353	1.504216	0.7791
LIPA	1.466351	1.12944	1.903763	0.004055
LZTS2	1.554539	1.121114	2.155528	0.008157
SFXN3	1.552198	1.210624	1.990144	0.000526
KAZALD1	1.41893	1.09992	1.830463	0.007083
FBXW4	2.55129	1.563871	4.162159	0.000176
NPM3	1.174779	0.824054	1.674775	0.373288
TNKS2	0.967171	0.603088	1.551051	0.889832
GBF1	1.113341	0.641716	1.931583	0.702516
ARHGAP21	0.958125	0.819544	1.120138	0.591502
CPEB3	1.232046	0.816551	1.858961	0.320076
FBXL15	1.511761	1.072391	2.131144	0.01833
CUEDC2	1.995286	1.183341	3.364345	0.009556
SUFU	1.857941	1.095352	3.151448	0.021574

ANKRD26	0.679737	0.477456	0.967717	0.03219
ACBD5	0.698092	0.378233	1.288444	0.250377
LHPP	1.410941	0.976179	2.039333	0.066998
LARP4B	1.097628	0.605828	1.988664	0.758689
GTPBP4	1.103695	0.718049	1.696463	0.652826
BCCIP	1.150914	0.694384	1.907594	0.585612
MTPAP	0.669672	0.378918	1.18353	0.167579
SH3PXD2A	1.036005	0.861016	1.246557	0.707871
PITRM1	1.461782	0.842019	2.537718	0.177342
MAP3K8	0.989713	0.769002	1.273769	0.935981
EBF3	0.774476	0.606528	0.98893	0.04044
GLRX3	1.022594	0.552177	1.893774	0.943349
SORCS1	0.997982	0.749886	1.328159	0.988945
XPNPEP1	1.309718	0.787316	2.178743	0.298772
SMC3	0.967123	0.660974	1.415073	0.863322
SHOC2	0.985626	0.592461	1.639701	0.955541
TFAM	1.002497	0.674478	1.490042	0.99016
CCDC6	1.823417	1.198564	2.774027	0.005016
CUL2	1.101943	0.622133	1.951797	0.739273
CCNY	1.235548	0.820397	1.860781	0.311344
UBE2S	0.958553	0.648424	1.417011	0.831908
RPL28	1.651184	1.103627	2.470408	0.014702
ZMIZ1	1.404548	1.055916	1.868288	0.01961
DNAJC12	1.043988	0.907911	1.20046	0.545751
PPIF	1.875768	1.352853	2.600804	0.000162
PBLD	0.482885	0.278941	0.835941	0.009323
TSPAN14	1.314809	0.995531	1.736484	0.053805
TBC1D12	1.368129	1.131074	1.654866	0.001244
KRT23	1.119755	0.885856	1.415412	0.344076
CRYBA1	0.748324	0.5036	1.11197	0.151362
NUFIP2	0.732986	0.468142	1.147662	0.174496
GIT1	1.01541	0.693019	1.487777	0.937457
RPL19	1.29517	0.844942	1.985301	0.235295
FBXL20	0.510865	0.324093	0.805271	0.003819
RUNDC3A	0.833849	0.589888	1.178705	0.303514
UBTF	1.351735	0.76398	2.39167	0.300556
CSF3	0.460452	0.161572	1.312207	0.146654
PSMD3	1.832475	1.048921	3.201351	0.033357
CASC3	0.732583	0.373826	1.435638	0.364659
RAPGEFL1	0.730796	0.481985	1.108049	0.139724
RGS9	1.387568	0.802414	2.39944	0.241119
RNF43	0.837474	0.61365	1.142938	0.263611
RAD51C	1.021707	0.610232	1.710637	0.934912
MTMR4	0.741336	0.534043	1.029092	0.07368
TRIM37	1.093365	0.683516	1.748965	0.709586
P2RX1	1.010311	0.769916	1.325766	0.941018
DHX40	0.860621	0.549169	1.348708	0.51256
TUBD1	0.713805	0.449638	1.133175	0.152784
KPNB1	1.324082	0.810602	2.162829	0.262177
GOSR2	0.879916	0.5346	1.448281	0.61484
PNPO	1.877747	1.214106	2.904141	0.004626
RPS6KB1	0.614714	0.352589	1.071712	0.086206
TRIM16L	1.27395	0.853756	1.900952	0.235745
CDK5RAP3	0.912529	0.645722	1.289578	0.603944
CBX1	1.177593	0.835946	1.658869	0.349773
RECQL5	1.076188	0.656785	1.763409	0.770728
PIGL	0.744625	0.514371	1.077951	0.118223
GALK1	1.659866	1.123733	2.451788	0.010893

INTS2	0.768507	0.492451	1.199314	0.246227
CAMTA2	1.320745	0.934298	1.867035	0.115217
MED13	0.728508	0.472663	1.122837	0.151268
HOXB6	1.185985	1.050897	1.338438	0.0057
ENO3	1.400189	0.958011	2.046457	0.082136
PFN1	1.809423	1.207652	2.711055	0.004046
RNF167	1.714077	1.07226	2.740061	0.024356
SLC25A11	1.34948	0.854762	2.13053	0.198301
RASD1	1.157427	1.012768	1.322749	0.031856
CHRNE	0.999554	0.728718	1.371047	0.99779
RAI1	1.029554	0.74797	1.417145	0.858203
NUP88	1.132703	0.673866	1.903963	0.638164
C1QBP	1.286363	0.870748	1.900356	0.20594
SLC6A4	0.83416	0.420909	1.653142	0.603352
BLMH	1.159714	0.80865	1.663188	0.42056
CPD	0.825255	0.630705	1.079817	0.161468
GOSR1	0.623871	0.341537	1.139599	0.124819
CCDC47	1.037615	0.667738	1.612376	0.869583
MED31	0.770637	0.454325	1.307174	0.33385
DRG2	1.3034	0.805026	2.110307	0.281124
FTSJ3	1.107746	0.701431	1.749424	0.660738
AKAP10	0.90787	0.586284	1.405851	0.664868
SMARCD2	1.608256	0.914432	2.82852	0.099058
ICAM2	1.511368	1.006079	2.270431	0.046684
SYNGR2	1.110706	0.788544	1.56449	0.548019
B9D1	1.543744	1.04961	2.270506	0.027388
UTP6	1.118149	0.662603	1.886887	0.675727
DDX5	0.932009	0.629663	1.379532	0.724901
CYTH1	1.442571	1.010721	2.058939	0.043517
PSMD11	1.05645	0.507265	2.200202	0.883363
LGALS3BP	1.108383	0.995951	1.233507	0.059346
CCL7	1.329518	0.743772	2.37656	0.336513
CCL2	1.221238	0.905921	1.646307	0.189661
CCL1	1.022222	0.886716	1.178437	0.761951
PEX12	1.212808	0.866297	1.697919	0.261058
DHX58	1.445034	1.00803	2.071489	0.045125
KAT2A	1.106038	0.805568	1.51858	0.533186
RAB5C	1.922668	1.201918	3.07563	0.006386
NAGLU	0.897954	0.58886	1.369294	0.617079
HSD17B1	0.561938	0.225531	1.400135	0.215943
MLX	1.586519	0.961837	2.616911	0.070673
CNTNAP1	1.283131	0.921917	1.785871	0.139413
ABI3	1.298918	1.1106	1.519167	0.001066
EZH1	0.91102	0.616576	1.346075	0.639876
DLX4	0.905805	0.701829	1.169065	0.447259
PPP1R9B	1.594717	1.059382	2.40057	0.025326
COL1A1	0.903236	0.745761	1.093962	0.297786
MRPL27	1.217254	0.613104	2.416731	0.574221
VAT1	0.848091	0.66667	1.078882	0.179693
LRRC59	1.139661	0.765631	1.696415	0.519487
RND2	0.791681	0.464961	1.347981	0.389639
ALOX12	0.955777	0.681797	1.339857	0.792982
HDAC5	1.303455	0.90581	1.875664	0.153518
ABCC3	1.203396	1.000249	1.447801	0.049692
MPP2	1.125498	0.82303	1.539126	0.459093
SMURF2	0.422432	0.264166	0.675517	0.000321
DUSP3	1.094369	0.78924	1.517463	0.588692
EFTUD2	1.207647	0.666111	2.189441	0.53425

HLF	1.102426	0.922617	1.317278	0.283093
SLC16A6	1.030927	0.824832	1.288518	0.788961
PRKAR1A	0.799733	0.515639	1.24035	0.318263
FAM20A	1.018963	0.754116	1.376825	0.902643
YWHAE	1.464615	0.879264	2.439649	0.142722
MMD	1.453248	1.076562	1.961735	0.014611
RANGRF	1.148913	0.707832	1.864851	0.574311
DPH1	1.078222	0.727573	1.597866	0.707464
MAP2K6	1.092831	0.789027	1.51361	0.593233
DHRS7B	1.354451	0.828143	2.215244	0.226773
WSB1	0.789599	0.532061	1.171796	0.240857
RCVRN	1.007494	0.716486	1.416697	0.965758
SLC9A3R1	1.473307	1.027605	2.112322	0.035022
MYH3	0.8748	0.613543	1.247305	0.459894
NAT9	1.061392	0.66855	1.685069	0.800547
TMEM104	1.47734	1.105773	1.973762	0.008286
VTN	1.03989	0.755856	1.430658	0.81009
TNFAIP1	1.174585	0.722156	1.91046	0.516743
IFT20	0.708095	0.465805	1.076412	0.10623
TMEM97	1.254829	0.98528	1.598119	0.065801
CDR2L	0.86165	0.595762	1.246203	0.428998
PMP22	0.831279	0.640673	1.078593	0.164343
UNC119	1.463526	1.127907	1.89901	0.004162
ALDOC	1.369769	1.088058	1.72442	0.007398
SUPT6H	1.269584	0.759599	2.121966	0.362416
RAB34	1.202863	1.009957	1.432616	0.038354
PHF12	0.933148	0.533408	1.632459	0.808411
TMEM33	0.912632	0.574308	1.450262	0.698854
GNRHR	0.641405	0.459023	0.896251	0.009277
SLAIN2	0.704445	0.41294	1.201731	0.198573
OCIAD1	0.78769	0.419648	1.478514	0.457589
DCUN1D4	0.611849	0.407638	0.91836	0.01774
USP46	0.678576	0.434725	1.059209	0.087864
CHIC2	0.43592	0.235	0.808624	0.008444
NMU	0.973943	0.811713	1.168598	0.776408
PF4V1	0.899034	0.6296	1.283771	0.558153
NFKB1	1.476896	0.957205	2.278739	0.078019
AREG	1.136077	1.043407	1.236977	0.003296
MANBA	0.632709	0.410197	0.975922	0.038435
UBE2D3	0.818597	0.453408	1.477922	0.506667
ELF2	0.378584	0.224921	0.63723	0.000256
NDUFC1	2.514668	1.381697	4.576656	0.002543
TBC1D9	1.172775	0.987355	1.393016	0.069518
ZNF330	1.083201	0.657758	1.783822	0.753512
INPP4B	1.143237	0.970185	1.347157	0.109929
GAB1	0.941347	0.689439	1.285299	0.703656
KLHL2	1.360924	1.014701	1.82528	0.039648
RPL34	0.811628	0.537051	1.226586	0.321876
WFS1	1.208932	1.023776	1.427574	0.025286
GRPEL1	1.248926	0.794406	1.963501	0.335586
GAR1	0.878059	0.561593	1.372856	0.568489
FRG1	1.012435	0.578077	1.773162	0.965526
CLCN3	1.222618	0.888582	1.682224	0.217034
AADAT	0.984636	0.859672	1.127766	0.823077
GALNT7	0.613382	0.447445	0.840857	0.00239
DHX15	0.632815	0.398856	1.004008	0.052019
SEPSECS	0.70977	0.441918	1.139969	0.15617
SLC2A9	1.172373	0.959687	1.432196	0.119452

FBXW7	0.976587	0.633908	1.504512	0.914435
NEIL3	0.922024	0.728825	1.166438	0.4986
TBC1D19	0.691734	0.451377	1.060082	0.090629
CLNK	1.258945	1.080138	1.467351	0.003216
SH3D19	0.971073	0.728079	1.295166	0.841661
STIM2	2.371586	1.498081	3.754416	0.000229
MFSD10	0.958271	0.775748	1.183739	0.692568
GLRB	0.813894	0.56716	1.167966	0.263808
BST1	1.042631	0.891195	1.2198	0.60211
RAPGEF2	1.10994	0.830943	1.482612	0.480084
HGFAC	0.886625	0.507883	1.547805	0.672079
SNX25	1.162764	0.936778	1.443265	0.17142
LRP2BP	1.210324	0.889889	1.646143	0.22379
UFSP2	0.955021	0.608057	1.499968	0.841641
KLF3	1.096831	0.74921	1.605743	0.634602
KLHL5	1.025318	0.74471	1.411659	0.878201
NCAPG	0.980572	0.736181	1.306093	0.893289
UGDH	0.618444	0.428304	0.892992	0.010354
HTATIP2	1.494869	1.187125	1.882391	0.00063
CTSC	0.846563	0.57483	1.246749	0.399029
CCDC34	0.97289	0.733553	1.290316	0.848706
ZBTB16	0.868964	0.734642	1.027845	0.10113
ELP4	0.905232	0.479574	1.708691	0.758714
MTCH2	1.256491	0.607318	2.599578	0.538206
FNBP4	1.086127	0.606412	1.945329	0.781136
TECTA	0.880832	0.428577	1.810329	0.729929
CRTAM	1.411774	1.05805	1.883754	0.019108
HSPA8	1.260076	0.87857	1.807245	0.208981
VWA5A	1.415642	1.150728	1.741545	0.001009
DNAJC4	1.585453	1.047951	2.398644	0.029133
SIAE	1.061549	0.82743	1.36191	0.638465
LPXN	1.293365	0.995	1.6812	0.054542
DTX4	1.189739	1.004258	1.409477	0.044527
ATG2A	1.339752	0.910213	1.971995	0.138082
EHD1	1.265881	0.904875	1.770913	0.168696
OSBP	0.951896	0.508539	1.781783	0.877509
UNC93B1	1.503581	1.204184	1.877418	0.000318
PUS3	1.059714	0.685791	1.637515	0.793927
DCPS	1.27158	0.762455	2.120672	0.357219
FOXRED1	1.476434	0.899345	2.423829	0.123436
NRXN2	1.128127	0.980536	1.297934	0.091949
MS4A6A	1.126575	1.021904	1.241966	0.016599
MS4A4A	1.174577	1.047485	1.31709	0.005888
ST3GAL4	0.896411	0.734059	1.094671	0.283412
CPT1A	1.346858	0.938342	1.933226	0.106346
CCND1	1.067386	0.89317	1.275585	0.473196
CCDC86	1.273074	0.863577	1.876751	0.222746
PRPF19	2.233369	1.353027	3.686501	0.001676
TMEM109	2.064369	1.380764	3.086421	0.000412
HPX	1.47959	0.737547	2.968201	0.27006
TRIM3	1.121357	0.81938	1.534626	0.474284
CHORDC1	0.686808	0.399629	1.18036	0.173893
FOLR1	1.535268	0.936758	2.516174	0.088984
FOLR3	1.131573	0.971736	1.317701	0.111623
PANX1	1.385687	0.999025	1.922003	0.050687
ARHGEF17	0.926533	0.776887	1.105004	0.395876
CEP164	1.632651	0.992287	2.686269	0.05367
RNF141	1.102173	0.739176	1.643431	0.633166

EIF4G2	0.820984	0.522528	1.289912	0.39219
IL10RA	1.122939	0.96508	1.306618	0.133587
BIRC2	1.194391	0.733925	1.943755	0.474651
UBE4A	0.962058	0.647512	1.429403	0.848154
DDX6	0.840103	0.54095	1.304693	0.437894
UPK2	2.274918	1.038248	4.984604	0.04
CBL	1.097301	0.795921	1.512801	0.57088
HIPK3	0.958342	0.674634	1.361359	0.812209
FBXO3	0.759383	0.482333	1.195567	0.234589
PDHX	1.061978	0.634726	1.776824	0.818878
SLC1A2	0.388064	0.10496	1.434774	0.155944
COMMD9	1.517735	0.944898	2.43785	0.084428
SLC15A3	1.211359	1.058509	1.386282	0.005333
CD5	1.057026	0.869752	1.284623	0.577248
ACCS	0.756149	0.616411	0.927565	0.007335
MDK	0.922266	0.775103	1.097371	0.361583
AMBRA1	0.97268	0.614156	1.540501	0.906011
MADD	1.283024	0.804596	2.045937	0.295203
PTPMT1	1.685318	0.832392	3.41221	0.14699
SLC22A18	1.211826	0.938975	1.563964	0.139898
CD81	1.159025	0.895536	1.500038	0.262073
SLC35F2	0.892903	0.719751	1.107711	0.303054
SOX6	0.776012	0.519997	1.158075	0.214429
PITPNM1	1.230513	0.939107	1.612342	0.132494
RPS13	1.132025	0.710638	1.803283	0.601659
AIP	1.694481	1.084099	2.648526	0.02065
NUP98	0.89607	0.550387	1.458869	0.65901
NDUFS8	1.850565	1.308492	2.617203	0.000501
TCIRG1	1.308089	1.080776	1.583212	0.005824
CHKA	0.971386	0.640673	1.472812	0.891257
EXPH5	1.201437	0.932982	1.547136	0.15493
HPS5	1.103537	0.692439	1.758701	0.678639
GTF2H1	1.043499	0.667355	1.631649	0.851902
POU2AF1	1.122018	0.884503	1.423313	0.342789
VWF	1.172312	1.028426	1.336329	0.017336
PSMD9	0.818201	0.4012	1.668629	0.581065
PPFIBP1	1.027974	0.787016	1.342706	0.83956
PRPF40B	0.736383	0.448174	1.209932	0.227122
CD69	1.084713	0.933683	1.260173	0.287796
PRDM4	1.217792	0.715557	2.072537	0.467663
CLEC2B	1.292254	1.084024	1.540484	0.004238
COQ5	1.085304	0.624024	1.887565	0.771886
SELPLG	1.604691	1.237093	2.08152	0.000367
CORO1C	1.546502	1.09183	2.190513	0.014105
CAPRIN2	0.951676	0.767866	1.179487	0.651021
TSPAN11	0.794729	0.461301	1.369161	0.407757
KCTD10	1.355541	0.734916	2.500274	0.330105
SLC11A2	0.788174	0.530081	1.171931	0.239559
MLEC	0.740255	0.487497	1.124066	0.158183
MVK	1.984152	1.184725	3.323016	0.009208
CSRNP2	0.63679	0.400407	1.012721	0.056577
CAMKK2	0.870911	0.572919	1.323895	0.517727
BIN2	1.810508	1.266954	2.58726	0.001118
IL23A	1.146303	0.780379	1.683812	0.486439
PTGES3	0.940925	0.548555	1.61395	0.824949
BCL7A	1.046559	0.84148	1.301619	0.68257
RSRC2	0.67183	0.413393	1.091833	0.108415
CYP27B1	1.0401	0.750425	1.441593	0.813383

LIN7A	0.839112	0.74237	0.948461	0.005007
KRT18	0.979576	0.875592	1.09591	0.718546
GLI1	1.116981	0.806854	1.54631	0.504982
PPM1H	0.797268	0.665147	0.955633	0.01425
METAP2	1.167462	0.763024	1.786271	0.475514
LTA4H	1.227095	0.965862	1.558982	0.093821
ELK3	1.342709	0.978836	1.841849	0.067652
SLC6A12	1.119779	0.864778	1.449972	0.390862
WNT5B	1.284667	0.915555	1.802589	0.147207
MAGOHB	0.735037	0.436469	1.237841	0.247028
TRPV4	1.267598	0.988095	1.626164	0.062079
ITFG2	0.799443	0.488827	1.307432	0.372459
FOXM1	1.091409	0.779866	1.527407	0.61
PRR4	0.726285	0.507479	1.039432	0.080375
PARP11	0.901631	0.588811	1.380644	0.633856
ARPC3	1.553368	0.97173	2.483149	0.065747
GPN3	1.134023	0.804502	1.598515	0.47273
VPS29	1.104039	0.616412	1.977414	0.73925
RAD51AP1	1.018165	0.764505	1.355989	0.901998
CUX2	1.075044	0.878391	1.315722	0.482665
SH2B3	1.516144	1.110213	2.070497	0.008856
MANSC1	1.372038	1.119907	1.680934	0.002265
DUSP16	0.843487	0.604012	1.177907	0.317808
CREBL2	1.083512	0.742417	1.58132	0.677536
ACAD10	0.894347	0.519745	1.538938	0.686782
ALDH2	1.076643	0.899294	1.288968	0.421318
CDKN1B	1.081782	0.723082	1.618422	0.702118
GPRC5D	1.14727	0.913826	1.44035	0.236571
GSG1	0.369282	0.188165	0.724735	0.003781
SCNN1A	2.724078	1.417601	5.234618	0.002637
LTBR	1.347545	0.949059	1.913345	0.095385
OGFOD2	0.745531	0.42111	1.319884	0.313632
CDK2AP1	1.184479	0.748165	1.875242	0.470138
OAS3	1.310549	1.087207	1.57977	0.004553
OAS2	1.331279	1.121244	1.580658	0.00109
ART4	0.841391	0.622597	1.137076	0.26104
MGP	0.742756	0.518509	1.063987	0.10486
RASAL1	1.040847	0.828732	1.307255	0.730609
ARHGDIB	1.435097	0.914518	2.252008	0.116118
GTF2H3	1.194282	0.742943	1.919811	0.463506
EIF2B1	1.442986	0.789674	2.636795	0.233163
DDX55	0.814234	0.478709	1.384929	0.448259
SLC38A1	1.171476	1.012032	1.356041	0.03399
VDR	1.317003	1.121718	1.546285	0.000772
RFC5	1.514457	0.927444	2.473013	0.097133
STX2	0.887157	0.684975	1.149017	0.364229
COPZ1	1.787955	0.812141	3.936242	0.148973
CAND1	0.897084	0.564325	1.426057	0.646067
IFNG	1.116271	0.833985	1.494104	0.459616
RAB5B	0.55752	0.367432	0.845947	0.006026
MDM1	0.676414	0.463769	0.98656	0.042333
NUP107	0.792349	0.52153	1.203799	0.275386
CNOT2	0.711209	0.377262	1.340761	0.292121
TIMELESS	1.251445	0.895507	1.748857	0.188977
CPSF6	0.666432	0.436522	1.017433	0.060121
KRR1	0.781924	0.473889	1.290189	0.335659
MRPL51	1.691433	0.949508	3.013082	0.074409
GAPDH	1.153619	0.802175	1.659035	0.440776

NOP2	0.590529	0.370299	0.941737	0.026962
CHD4	0.507254	0.276375	0.931005	0.028473
ACRBP	1.374235	0.968468	1.950009	0.074992
UHRF1BP1	1.068688	0.729877	1.564775	0.732756
COPS7A	1.579511	0.970281	2.571272	0.065972
ING4	0.878583	0.518678	1.488223	0.630238
GNB3	0.84003	0.643002	1.097431	0.20117
CDCA3	0.947683	0.696521	1.289412	0.732326
CHPT1	0.763091	0.472198	1.233187	0.269564
USP5	2.255929	1.373941	3.7041	0.001302
TPI1	1.975292	1.261916	3.091948	0.002906
GNPTAB	0.922642	0.67911	1.253507	0.606605
SPSB2	1.232499	0.832377	1.82496	0.296563
ENO2	1.057108	0.874326	1.278101	0.566386
ATN1	1.037235	0.853498	1.260527	0.71324
PTPN6	1.661441	1.253441	2.202246	0.000414
LPCAT3	2.385871	1.485296	3.832488	0.000323
NT5DC3	1.077664	0.877817	1.323009	0.474787
SUDS3	0.577969	0.300386	1.11206	0.100617
GOLT1B	0.805944	0.474705	1.368312	0.424379
LDHB	1.003768	0.751683	1.340391	0.979667
PRKAB1	1.192483	0.735488	1.933431	0.475252
CMAS	1.227961	0.811162	1.858924	0.331704
HCFC2	1.017297	0.612453	1.689753	0.947187
CLEC4A	1.057357	0.863992	1.293998	0.58834
RAB35	1.542358	0.970583	2.45097	0.066711
PHC1	0.98727	0.713335	1.366402	0.938412
COX6A1	1.777999	1.065808	2.966088	0.027521
RIC8B	0.809753	0.544552	1.204109	0.297209
FGFR1OP2	0.896533	0.618938	1.298631	0.563446
KLRB1	0.888	0.677128	1.164542	0.39049
BTN3A3	1.000757	0.770675	1.299529	0.99547
DSE	0.735967	0.578465	0.936353	0.012588
RWDD1	1.211494	0.655382	2.239485	0.540516
MAK	0.661837	0.480385	0.911828	0.011585
TMEM14C	0.94623	0.597014	1.499716	0.814043
PAK1IP1	1.00396	0.643657	1.56595	0.986097
GCNT2	1.012688	0.817757	1.254086	0.90798
NEDD9	1.343414	1.012066	1.783245	0.041056
ASF1A	0.886221	0.618576	1.26967	0.510252
MCM9	0.731978	0.394872	1.356876	0.321778
RNGTT	1.238747	0.669311	2.292646	0.495459
MAN1A1	0.904166	0.774446	1.055614	0.202311
GABRR2	1.156428	0.90994	1.469686	0.234711
SERINC1	1.100383	0.701753	1.725456	0.676829
HDDC2	1.947213	1.175347	3.225973	0.009675
HINT3	0.956738	0.586442	1.56085	0.859432
NCOA7	0.94274	0.747992	1.188192	0.617469
SASH1	1.25811	1.006938	1.571936	0.043299
UST	1.51931	1.195839	1.93028	0.000617
FBXO5	0.95073	0.631193	1.432029	0.808969
MTRF1L	1.376312	0.732769	2.585034	0.320627
PPARD	1.267748	0.995776	1.614001	0.054153
FANCE	1.327641	0.873041	2.018955	0.185129
SLC26A8	0.862613	0.677745	1.097907	0.229774
MAPK14	0.859478	0.564608	1.308343	0.479977
RHAG	0.976697	0.869158	1.097542	0.691982
KCTD20	1.038912	0.666478	1.619464	0.866157

STK38	0.72915	0.494217	1.075763	0.111404
SOD2	1.318906	1.030539	1.687965	0.027882
MRPL18	2.328397	1.364331	3.973693	0.001941
MCM3	1.356813	0.946277	1.945455	0.096989
RNF8	1.813455	1.096262	2.999848	0.020458
PHACTR1	1.335942	0.961	1.857171	0.084838
MDGA1	0.974924	0.825852	1.150905	0.764215
FBXO9	0.906123	0.557925	1.47163	0.690322
CD83	1.149691	0.915497	1.443794	0.23003
MDN1	0.925868	0.66076	1.297342	0.654508
BACH2	1.064316	0.85303	1.327936	0.580898
TREML2	1.789009	1.420717	2.252773	7.58E-07
ZNF451	0.673981	0.418215	1.086166	0.105127
BAG2	1.110358	0.844222	1.460392	0.454007
RAB23	1.046616	0.706182	1.551166	0.820446
KHDRBS2	1.271275	1.003089	1.611163	0.047091
FBXL4	0.624343	0.402353	0.96881	0.035613
CCNC	0.742677	0.468485	1.177344	0.205698
E2F3	0.803951	0.470606	1.373415	0.424487
PTP4A1	0.393956	0.130547	1.188852	0.098332
ASCC3	0.977872	0.642242	1.4889	0.916916
BVES	1.753438	1.343619	2.288256	3.56E-05
MED23	0.734633	0.435352	1.239653	0.248006
WASF1	0.905915	0.784068	1.046697	0.180017
GPLD1	0.910247	0.643718	1.287131	0.594727
ALDH5A1	0.939688	0.735556	1.200471	0.618618
VNN1	1.196333	1.080046	1.32514	0.000591
VNN2	1.133525	1.012053	1.269578	0.030226
SMAP1	1.244001	0.615122	2.515826	0.543442
RPS12	1.313928	0.896409	1.925914	0.161685
B3GAT2	0.43277	0.199495	0.938822	0.034028
GMNN	1.293945	0.865861	1.933675	0.208661
SOBP	1.523218	0.982882	2.360603	0.059739
SNX3	1.204669	0.60203	2.410556	0.598795
HBS1L	0.842305	0.498206	1.424066	0.521835
TRIM38	1.370096	0.911137	2.060242	0.130319
PEX7	1.688618	0.865423	3.294843	0.124498
ZBTB24	1.160288	0.69351	1.941237	0.571278
FIG4	0.922075	0.52303	1.62557	0.779135
PERP	1.362479	0.66859	2.776513	0.39445
SLC16A10	0.909187	0.634855	1.302064	0.603382
HECA	1.3044	0.907229	1.875447	0.15145
PHACTR2	1.010558	0.723327	1.411848	0.950915
EPM2A	0.707855	0.416872	1.20195	0.200885
SLC39A7	0.992219	0.618372	1.592081	0.974171
PHF1	1.027418	0.710131	1.486469	0.88587
CUTA	1.455362	0.929223	2.279408	0.101153
QKI	1.058794	0.734117	1.527065	0.759789
MDFI	0.883215	0.783787	0.995255	0.041548
TFEB	1.54307	1.260454	1.889053	2.64E-05
CCND3	1.876008	1.40272	2.508987	2.22E-05
BYSL	1.566506	1.002253	2.448426	0.048857
FAM120B	0.976203	0.608264	1.566708	0.920514
TBP	0.720838	0.366225	1.418822	0.343414
GUCA1B	0.926985	0.654613	1.312686	0.669275
PRPH2	1.033725	0.68024	1.570898	0.876546
PPP2R5D	1.267694	0.696345	2.307834	0.437755
MRPL2	1.306458	0.732563	2.329949	0.365126

PTK7	0.904976	0.766281	1.068774	0.239456
SRF	1.491977	0.943186	2.360082	0.08727
CUL9	0.914762	0.614205	1.362394	0.661121
DUSP22	1.033083	0.71632	1.489923	0.861698
EXOC2	0.839463	0.527353	1.336291	0.460657
COX7A2	1.685512	0.994105	2.8578	0.052621
TMEM30A	0.94941	0.643505	1.400732	0.793605
GMDS	1.284775	0.809999	2.037837	0.287029
SENP6	0.745772	0.423442	1.313463	0.309741
VEGFA	0.869644	0.728886	1.037584	0.121043
PRPF4B	0.604452	0.372	0.982156	0.042087
TTK	0.940944	0.704251	1.257186	0.680515
SLC29A1	1.226265	0.861767	1.744935	0.25707
BTN2A1	0.945159	0.597504	1.495094	0.809512
LAMA4	0.872566	0.568337	1.33965	0.533162
CLIC5	1.214568	0.586592	2.514823	0.600643
LY86	1.12544	1.006247	1.258753	0.038547
HARS2	0.962999	0.611895	1.515566	0.870558
NUDT12	1.109164	0.855747	1.437627	0.433705
CEP72	1.096421	0.692696	1.73545	0.694406
MAN2A1	0.878131	0.58225	1.32437	0.535322
HMGCS1	1.310321	0.909349	1.888099	0.147027
DAP	1.08496	0.735561	1.600327	0.680921
BRD8	0.809114	0.547976	1.194696	0.286745
KIF20A	0.829734	0.658284	1.045837	0.113999
NNT	1.023095	0.669628	1.563141	0.915918
MRPS30	0.831578	0.521988	1.324785	0.43761
HSPA9	0.921225	0.631875	1.343076	0.669703
MRPS27	0.960865	0.643024	1.435814	0.845547
PFDN1	1.442391	0.811661	2.563251	0.211795
HBEGF	1.020529	0.893328	1.165842	0.764797
LOX	0.94766	0.751664	1.19476	0.649291
GZMK	1.007625	0.786399	1.291085	0.952111
CDH9	0.909808	0.775214	1.067771	0.247198
APBB3	0.976945	0.679551	1.40449	0.89978
TMCO6	0.880641	0.52698	1.471648	0.627566
SPARC	0.92724	0.832197	1.033138	0.170962
IK	1.123497	0.649672	1.942897	0.676911
HMGCR	1.107031	0.710148	1.725719	0.653509
FAF2	1.224863	0.670479	2.237637	0.509438
PDE8B	0.589385	0.300376	1.156467	0.124229
CLK4	0.932809	0.604644	1.439082	0.753192
HAVCR1	1.190956	0.848589	1.671454	0.312226
ITK	1.008585	0.810873	1.254505	0.938791
RNF130	0.621433	0.398088	0.970084	0.036293
THG1L	1.121725	0.719249	1.749418	0.612442
ARSB	0.857379	0.575804	1.276647	0.44872
CLINT1	0.487919	0.318366	0.747771	0.000987
THBS4	0.800558	0.603814	1.061409	0.12215
CNOT6	0.694497	0.445764	1.082023	0.107071
BTNL8	0.766584	0.478433	1.228283	0.269113
TTC1	1.547813	0.807109	2.96828	0.188537
MSH3	0.807202	0.541837	1.202529	0.29228
RASGRF2	0.724708	0.486755	1.078986	0.11283
CCNG1	1.109942	0.83152	1.481589	0.479024
POLR3G	0.654907	0.470442	0.911701	0.012154
LMNB1	0.780795	0.551525	1.105374	0.162981
ARRDC3	0.971209	0.775542	1.216242	0.799108

GOLPH3	1.137293	0.728425	1.775661	0.571415
SUB1	0.794361	0.461803	1.366405	0.405471
NPR3	1.027097	0.940275	1.121936	0.552958
FAM172A	0.686139	0.414817	1.134928	0.14237
SLC27A6	0.834674	0.65426	1.064837	0.145841
LNPEP	0.775138	0.530865	1.131811	0.187217
PDE4D	0.898916	0.671549	1.203263	0.473828
RAD1	1.185185	0.585266	2.400046	0.636969
BRIX1	0.962755	0.590099	1.57075	0.879207
PRLR	1.068275	0.913074	1.249857	0.409606
SLC12A7	1.208244	0.97377	1.499178	0.085706
RAD50	0.742833	0.386936	1.426077	0.371659
ST8SIA4	0.771087	0.562136	1.057706	0.10695
GNPDA1	1.138213	0.917704	1.411707	0.238675
PCDH12	1.449781	1.077403	1.950862	0.014199
SKP1	0.78058	0.479813	1.269878	0.318425
NUP155	1.011235	0.645162	1.58502	0.961141
PPP2CA	0.922306	0.568396	1.496574	0.743304
NR3C1	0.897296	0.598169	1.346007	0.600429
PPWD1	0.748444	0.452502	1.237936	0.259066
LIFR	0.80438	0.593726	1.089774	0.160004
TRIM23	0.684155	0.438429	1.067604	0.094557
SEC24A	0.645574	0.394135	1.057419	0.082175
TXNDC15	0.740348	0.410956	1.333758	0.316819
TTC33	0.903334	0.586324	1.391742	0.644794
TCERG1	0.508137	0.304579	0.847737	0.009528
DPYSL3	1.165776	1.006801	1.349852	0.040308
SMAD5	0.73579	0.553753	0.977668	0.03437
CSNK1A1	0.856171	0.508631	1.441179	0.558913
ERGIC1	1.162768	0.835409	1.618404	0.371354
PDGFRB	0.976628	0.793755	1.201633	0.823095
ATP6V0E1	1.333496	0.82758	2.148688	0.237033
BNIP1	0.903036	0.527856	1.544882	0.709672
CPEB4	0.959037	0.700065	1.313811	0.794523
HRH2	1.032026	0.897704	1.186446	0.657689
DBN1	1.10434	0.912948	1.335855	0.306753
ZNF346	1.099764	0.69432	1.741964	0.685291
UNC5A	1.08263	0.91661	1.27872	0.349905
EHHADH	1.11709	0.841854	1.482313	0.442964
SMC4	1.234942	0.932581	1.635334	0.140802
ACTR8	0.929429	0.602457	1.43386	0.740762
TBCCD1	0.979213	0.568025	1.688056	0.939736
CRBN	0.625626	0.40324	0.970657	0.036361
BCL6	1.112327	0.964406	1.282936	0.143696
HGD	1.436668	1.022382	2.018828	0.036844
ARL6	0.769005	0.494496	1.195903	0.243668
NPHP3	0.808702	0.450852	1.450586	0.476325
CD86	1.143123	1.015471	1.286821	0.026823
AMOTL2	0.983679	0.696659	1.388951	0.92552
NIT2	0.832553	0.50199	1.380795	0.477732
FAM162A	2.479064	1.367778	4.493244	0.00277
OGG1	0.996822	0.746552	1.330993	0.982786
KPNA1	0.799216	0.463569	1.377887	0.419961
PCCB	0.852785	0.570803	1.274068	0.436889
UBE3A	0.763208	0.457126	1.274236	0.301472
ARMC8	0.845787	0.521329	1.372179	0.497518
CEP70	0.958113	0.779251	1.17803	0.684841
RBP2	0.816568	0.595	1.120646	0.209586

SLC25A36	0.646741	0.427716	0.977923	0.038849
GRK7	1.200003	0.550131	2.617571	0.646819
RNF7	1.50036	0.832106	2.705281	0.177375
TFDP2	0.909229	0.703942	1.174384	0.466111
XRN1	0.709707	0.489856	1.028227	0.069862
KAT2B	1.131982	0.761139	1.683508	0.540424
SERPINI2	1.285509	1.106375	1.493646	0.001037
PDCD10	1.506025	0.852302	2.661158	0.158614
PFKFB4	1.060338	0.853815	1.316816	0.596056
COL7A1	1.282128	0.809835	2.029859	0.289066
PRKAR2A	1.435967	0.883438	2.334064	0.144311
HES1	1.162594	0.879837	1.536222	0.289335
USP4	1.116491	0.698771	1.783921	0.644898
ACAP2	0.874921	0.571284	1.339942	0.53894
ECT2	0.839361	0.576504	1.222069	0.360907
GNAT1	0.216696	0.010937	4.293573	0.315545
GNAI2	1.545705	1.066788	2.239625	0.021353
TFG	0.799523	0.420159	1.521419	0.4955
USP9Y	1.013268	0.907958	1.130792	0.813888
HYAL1	0.888737	0.593388	1.33109	0.567112
TUSC2	1.114629	0.637107	1.95006	0.703747
RPL24	0.909688	0.544986	1.518448	0.71728
CYB561D2	1.359732	0.88487	2.089426	0.160935
FXR1	0.503926	0.313425	0.810214	0.004675
CBLB	0.966498	0.561424	1.663838	0.902145
BBX	1.127916	0.818496	1.554306	0.461884
IFT57	1.640215	1.116964	2.408588	0.011595
GNB4	0.74102	0.458523	1.197563	0.221018
HHLA2	1.210593	0.959162	1.527933	0.107631
IQCG	1.461296	0.965878	2.210823	0.072555
GBE1	0.788647	0.518937	1.198533	0.266183
UMPS	1.323829	0.822423	2.130927	0.248079
NCBP2	0.70118	0.407525	1.206437	0.199794
SNX4	0.826161	0.486949	1.40167	0.478927
FRMD4B	1.060885	0.854269	1.317474	0.5928
SLC41A3	0.94083	0.600547	1.473925	0.790016
PLXNA1	1.30595	1.05559	1.615688	0.013965
ATP6V1A	1.439385	1.005573	2.060346	0.046554
ABTB1	1.142116	0.870331	1.498772	0.337886
PODXL2	1.136423	0.99336	1.300089	0.062474
UPK1B	1.440211	0.947591	2.188926	0.08765
CSPG5	0.646653	0.341659	1.223909	0.180488
KLHL18	1.20026	0.763619	1.886573	0.428869
SCAP	1.074911	0.712581	1.621475	0.730544
NEK11	1.198315	0.795721	1.804601	0.386451
MRPL3	0.994618	0.628222	1.574707	0.981634
PLSCR4	1.038399	0.876015	1.230885	0.664079
HEMK1	0.804881	0.531089	1.219819	0.306187
CISH	1.122569	0.904536	1.393158	0.294015
MAPKAPK	1.231638	0.757798	2.001766	0.400476
ACVR2B	0.877348	0.674719	1.14083	0.328763
WDR48	0.60191	0.386545	0.937266	0.024659
COMMD2	1.235428	0.778974	1.95935	0.368933
GORASP1	0.753899	0.451846	1.257868	0.279433
RRP9	1.538671	0.993351	2.383355	0.053596
ABCC5	0.909748	0.635584	1.302174	0.605193
ABHD14B	1.596944	1.01249	2.51877	0.044078
EIF1B	1.31728	0.868885	1.997073	0.194295

KLHL24	0.636574	0.442878	0.914985	0.01469
PLCH1	0.818835	0.680735	0.98495	0.033933
VIPR1	1.196554	0.968906	1.477688	0.095595
DNAH1	1.017546	0.745592	1.388696	0.912702
SSR3	1.269236	0.760811	2.117425	0.361216
ZBTB47	1.458828	1.055218	2.016816	0.022301
NKTR	0.604548	0.417651	0.875079	0.00765
CLCN2	0.988109	0.654572	1.4916	0.9546
FOXP1	0.737822	0.483275	1.126443	0.159004
EIF4G1	1.39204	0.871298	2.224009	0.166465
SPCS1	1.553387	0.89674	2.690871	0.116144
NEK4	0.793041	0.512542	1.227049	0.297782
SLC4A3	1.206742	0.99428	1.464604	0.057183
INO80D	0.503121	0.319095	0.793279	0.003109
EEF1B2	0.983148	0.713658	1.354404	0.917186
ADAM23	0.960404	0.579043	1.59293	0.875638
DGUOK	2.450117	0.931776	6.442615	0.069261
LMAN2L	0.801526	0.467931	1.372945	0.420422
RTKN	0.841259	0.67508	1.048345	0.123687
TTL	1.209873	0.73009	2.00495	0.459748
IL1A	1.101878	0.701396	1.731025	0.673784
CCL20	0.688439	0.325302	1.456948	0.329044
PIKFYVE	0.564047	0.377915	0.841854	0.005071
FAHD2A	0.995108	0.625686	1.582647	0.983473
NCL	1.111061	0.743324	1.660726	0.607568
ACTR1B	2.019536	1.245726	3.274014	0.004354
SLC35F5	0.401028	0.25314	0.635314	9.92E-05
ZAP70	0.995273	0.846428	1.170293	0.954286
ACTR3	1.271147	0.783676	2.06184	0.33095
STEAP3	0.932035	0.750132	1.158048	0.525187
EPB41L5	0.984795	0.73726	1.31544	0.917382
TFCP2L1	1.162479	0.878793	1.537744	0.291533
TP53I3	1.266563	0.891486	1.799447	0.18721
DNAJC27	0.789068	0.462798	1.345356	0.384175
POMC	1.028454	0.882362	1.198734	0.719653
STAM2	0.735222	0.435839	1.240254	0.248951
OTOF	1.616565	1.08618	2.40594	0.017912
GPD2	1.042429	0.73596	1.476518	0.815028
CENPA	0.782148	0.587383	1.041495	0.092627
CYTIP	1.270199	1.03256	1.562529	0.023627
ACVR1	1.367483	0.985849	1.896853	0.060848
TANC1	0.953757	0.801061	1.13556	0.594815
SLC30A3	0.328451	0.109221	0.987725	0.047485
MPV17	1.189431	0.611673	2.312911	0.609168
GTF3C2	1.083539	0.52487	2.23685	0.828248
EIF2B4	1.267327	0.65266	2.460881	0.484107
NRBP1	0.914449	0.551878	1.515222	0.728513
ITGA4	0.606979	0.446449	0.825232	0.001444
PSMD14	1.546195	0.898296	2.661393	0.115751
SNX17	1.862673	1.056902	3.282755	0.031446
ASB3	0.638519	0.339802	1.199838	0.163353
PPM1G	2.016478	1.107297	3.672169	0.021835
REEP6	0.972117	0.778061	1.214573	0.803426
PCSK4	0.887375	0.717261	1.097835	0.271168
APC2	0.915726	0.590789	1.41938	0.693787
IFIH1	1.264703	0.909262	1.759091	0.163035
RPS15	1.285598	0.944079	1.750661	0.110783
GCA	1.047874	0.82564	1.329926	0.700591

INO80B	1.042468	0.628282	1.729702	0.872103
MOGS	0.939217	0.603909	1.460697	0.780775
TTC31	1.337046	0.756	2.364673	0.318058
NDUFS7	1.204425	0.692569	2.094579	0.510011
PCGF1	1.339199	0.806514	2.22371	0.258958
CLIP4	1.339461	1.12491	1.594933	0.001033
SPTBN1	0.931328	0.724768	1.196758	0.578165
AUP1	1.440085	0.888844	2.333194	0.138514
RTN4	1.808435	0.976026	3.350768	0.059721
HTRA2	1.238553	0.765751	2.00328	0.383177
LOXL3	0.942089	0.740102	1.199201	0.628009
DOK1	1.091187	0.764933	1.556594	0.630173
GALNT3	0.778151	0.616089	0.982843	0.035273
POLE4	1.653787	1.135198	2.409282	0.00878
TACR1	0.725963	0.423158	1.245448	0.24486
CCDC88A	0.83453	0.565718	1.231074	0.36181
MRPL19	0.891806	0.577292	1.377672	0.605824
LANCL1	1.002438	0.708814	1.417695	0.989015
WDR75	0.93785	0.563362	1.561276	0.805101
EFEMP1	1.160245	0.874766	1.538891	0.302331
FANCL	0.93953	0.62479	1.412821	0.764429
FN1	1.119766	0.943319	1.329216	0.196012
STAT1	1.089742	0.823008	1.442922	0.5485
GLS	0.764543	0.524494	1.114459	0.162612
PAPOLG	0.718769	0.466819	1.106702	0.133729
PECR	1.090935	0.876539	1.357771	0.43562
UNC50	0.68244	0.348382	1.336821	0.265382
IGFBP2	0.94636	0.853466	1.049365	0.295619
ELMOD3	1.050318	0.692251	1.593597	0.817469
IGFBP5	0.801767	0.673387	0.954622	0.01308
USP34	0.693376	0.48678	0.987656	0.042481
KCNJ13	0.691779	0.471807	1.01431	0.059134
CCT4	0.9544	0.638973	1.425537	0.819652
GGCX	0.649526	0.365081	1.155591	0.142105
EHBP1	0.774196	0.522867	1.146332	0.201247
TXNDC9	1.013753	0.598623	1.716766	0.959469
COQ10B	1.263162	0.707705	2.254579	0.429326
GNLY	1.062657	0.924439	1.221541	0.392651
SF3B1	0.85732	0.583262	1.26015	0.433425
ST3GAL5	0.962493	0.746654	1.240725	0.767937
CHST10	0.937399	0.701566	1.25251	0.661954
PDCL3	0.932693	0.579962	1.499955	0.773773
HSPE1	1.171134	0.795954	1.723158	0.422703
PLCD4	0.931977	0.536079	1.620246	0.802842
ZNF142	1.289121	0.805836	2.062246	0.289408
IL1R2	1.198935	1.082241	1.328212	0.000515
IL1R1	1.060428	0.891492	1.261377	0.507534
IL1RL1	0.853541	0.726774	1.002418	0.053543
IL18R1	0.957936	0.787173	1.165744	0.667922
IL18RAP	1.037905	0.867742	1.241436	0.683846
FHL2	1.232269	0.996777	1.523396	0.053591
UXS1	0.823513	0.543152	1.248589	0.360489
ABCB6	0.811297	0.502722	1.309278	0.391777
STK16	1.899585	1.151482	3.133719	0.011997
HDLBP	1.042776	0.695762	1.562862	0.839223
PPP1R7	3.322565	1.764258	6.25727	0.000201
PASK	0.947922	0.601463	1.493949	0.817756
STK25	1.543017	0.984251	2.418997	0.058656

TPO	0.897281	0.687515	1.171048	0.425005
PROC	1.060603	0.675071	1.666313	0.798522
ID2	1.153397	0.983947	1.35203	0.07835
TAF1B	0.932723	0.574613	1.514012	0.778098
HPCAL1	1.487762	1.019634	2.170815	0.03932
ODC1	1.146467	0.820314	1.602298	0.423546
BIRC6	0.677295	0.46928	0.977516	0.037393
NOL10	0.970868	0.552209	1.706933	0.918206
PLEKHB2	1.205523	0.739096	1.966303	0.453978
GORASP2	1.316206	0.6845	2.530896	0.410149
STRN	0.900756	0.574491	1.412313	0.648757
CEBPZ	1.027811	0.7035	1.501628	0.887228
PRKD3	0.985387	0.805813	1.204978	0.885962
QPCT	0.981609	0.856037	1.1256	0.790397
RAB3GAP1	1.073261	0.674142	1.708675	0.765706
SLC25A12	1.629378	0.967745	2.743358	0.066266
DLX2	1.271311	0.510201	3.167831	0.606329
LCT	1.43125	1.162033	1.762839	0.000745
SDC1	0.950646	0.785879	1.149957	0.602239
PLCL1	0.914608	0.713445	1.172491	0.481234
SLC1A4	0.727917	0.537562	0.985677	0.04005
SOS1	0.80133	0.548998	1.169641	0.251025
KYNU	1.205114	1.014739	1.431205	0.033441
WIPF1	1.095709	0.685846	1.750506	0.702183
COX7A2L	0.355726	0.186555	0.678302	0.001697
PNO1	1.199265	0.725447	1.982555	0.478639
PLEK	1.154109	0.767795	1.734797	0.490655
RND3	1.225556	0.870395	1.72564	0.244043
ATF2	0.595214	0.338179	1.047611	0.072064
THADA	0.654968	0.399519	1.073747	0.093378
AAK1	0.780961	0.564861	1.079734	0.134701
TRAK2	1.099791	0.819537	1.475881	0.526193
TIA1	0.676992	0.464161	0.987413	0.042791
PCYOX1	0.725475	0.504664	1.0429	0.083074
KISS1R	0.83797	0.51684	1.358628	0.4734
EPAS1	0.800618	0.645545	0.992942	0.042924
ARID3A	0.96565	0.66727	1.397455	0.852953
SUMO1	1.203172	0.666752	2.171156	0.539132
GRIN3B	1.049213	0.716043	1.537403	0.805335
NFE2L2	1.165573	0.659278	2.060679	0.5982
MSH6	0.865879	0.558018	1.343588	0.520593
PLEKHA3	0.594552	0.340437	1.03835	0.067598
SPR	1.032503	0.81314	1.311044	0.792946
EPHA4	0.86012	0.571434	1.294648	0.470157
PARD3B	0.971986	0.679766	1.389825	0.876239
FARSB	1.20854	0.761345	1.918407	0.421735
ALMS1	0.691058	0.489183	0.976241	0.036049
BCL9	1.281034	1.047555	1.56655	0.015843
DHCR24	1.098984	0.935664	1.290811	0.250205
DNAJC16	0.821612	0.494398	1.36539	0.448334
MORN1	0.828058	0.484267	1.415915	0.490615
GPX7	1.13486	0.869756	1.480768	0.35135
CACYBP	1.45655	0.831995	2.549939	0.188098
SCP2	1.222664	0.76454	1.955304	0.401357
TPSG1	1.058404	0.715838	1.564905	0.776039
RALGPS2	0.887323	0.700788	1.123509	0.320802
ANGPTL1	0.742401	0.576523	0.956007	0.020962
FAM20B	0.814213	0.503576	1.316472	0.401812

TMEM59	1.35079	0.688239	2.651163	0.382123
LRRC42	1.926638	1.055224	3.517677	0.032764
MRPL37	2.131066	1.225217	3.706641	0.00738
ICMT	1.514057	0.912258	2.512852	0.108561
RPL22	0.899301	0.640417	1.262837	0.540045
CHD5	0.811549	0.644188	1.022389	0.076386
QSOX1	1.037062	0.808239	1.330668	0.774788
STXBP3	0.714258	0.438096	1.164505	0.177239
PHF13	0.859401	0.632301	1.168068	0.333171
ERRFI1	0.823896	0.565584	1.200182	0.312851
PARK7	2.297428	1.279982	4.123631	0.005319
AMPD2	0.994456	0.721511	1.370656	0.972909
MECR	1.236397	0.727415	2.10152	0.433011
KCNC4	0.885467	0.670963	1.168547	0.390096
EDEM3	0.704533	0.491102	1.01072	0.057164
WDR77	1.755588	0.92044	3.348493	0.087577
RAP1A	1.374051	0.915743	2.06173	0.124827
HDAC1	1.805467	1.01303	3.217781	0.045084
CAPZA1	1.482097	0.92746	2.368417	0.099949
S100PBP	0.812538	0.521959	1.264885	0.35792
RNF19B	1.405562	0.996865	1.981816	0.052131
SCAMP3	1.716693	1.028274	2.866001	0.038773
TRIM62	1.303388	0.891729	1.905087	0.17124
ASH1L	0.647067	0.44398	0.943051	0.023509
DLGAP3	1.154611	0.933903	1.427479	0.184114
SFPQ	0.701815	0.441237	1.116279	0.13481
RHOU	1.064023	0.907374	1.247716	0.44503
GON4L	1.038212	0.592898	1.817994	0.895621
ARHGEF2	1.158848	0.674086	1.992223	0.593831
MEF2D	1.330331	0.974058	1.816913	0.072702
DOCK7	0.672629	0.477842	0.946818	0.023013
SRM	1.587803	1.116707	2.257636	0.010034
DLEU2L	0.684137	0.434068	1.078271	0.101983
FBXO6	1.506885	1.079741	2.103005	0.015905
MAD2L2	1.223155	0.858826	1.742038	0.264229
DNAJC6	1.079162	0.910978	1.278397	0.378128
LEPR	0.960474	0.76513	1.205692	0.72813
IVNS1ABP	1.075273	0.84367	1.370455	0.557603
KIAA2013	1.413333	0.901386	2.216044	0.131672
MFN2	1.278282	0.764926	2.13616	0.348697
PRG4	0.893493	0.5356	1.490534	0.66624
MIIP	1.315863	0.917635	1.886912	0.135549
SMG7	1.106613	0.62597	1.956313	0.727475
NCF2	1.203676	1.058867	1.368289	0.004589
PDC	1.212978	0.807061	1.823053	0.35299
SLC35D1	0.757634	0.492091	1.166469	0.20745
PLA2G4A	1.578436	1.27946	1.947273	2.04E-05
GADD45A	1.618904	1.152104	2.274837	0.005507
PRDM2	1.026299	0.716172	1.470723	0.887544
RGS2	1.074587	0.949176	1.216568	0.255893
UCHL5	1.142684	0.703656	1.855632	0.589763
BCAS2	1.582065	0.936465	2.672742	0.086417
CTH	0.616585	0.464727	0.818067	0.000802
AGMAT	1.295629	0.897021	1.871367	0.167389
OLFML3	0.964661	0.797698	1.166571	0.710603
PLEKHM2	1.787214	1.21089	2.637838	0.003463
CRYZ	0.959	0.685714	1.341201	0.806751
PHTF1	1.069403	0.678275	1.686076	0.772695

ZBTB17	1.136801	0.799455	1.616497	0.475326
CD58	0.637343	0.462823	0.877669	0.005793
TFAP2E	0.9934	0.726354	1.358627	0.966936
CD2	1.134369	0.936724	1.373717	0.196791
TTF2	0.982607	0.646885	1.492561	0.934435
NR5A2	1.071492	0.791874	1.449845	0.654483
KIF21B	1.405026	1.07912	1.829361	0.011554
TMEM9	1.176472	0.884093	1.565544	0.264906
MAP7D1	1.765835	1.192108	2.61568	0.00456
WARS2	0.979574	0.597942	1.60478	0.934692
OSCP1	0.946948	0.759687	1.180368	0.627752
MRPS15	1.474321	0.835636	2.60116	0.180214
EXOC8	1.647966	1.021816	2.657811	0.040515
GNPAT	1.102075	0.638911	1.901	0.726774
TSNAX	1.11075	0.715971	1.723205	0.639226
RRAGC	1.599356	0.840085	3.044859	0.152855
NID1	0.990569	0.887872	1.105144	0.865255
LGALS8	1.43066	0.819225	2.498445	0.20803
NT5C1A	1.547319	0.141526	16.91701	0.72056
HPCAL4	0.84868	0.495832	1.452626	0.549612
MTR	0.931529	0.63598	1.364423	0.715676
BMP8B	1.445747	1.07997	1.935411	0.013253
SIPA1L2	1.042246	0.885725	1.226427	0.618219
RLF	0.784837	0.499675	1.232739	0.29294
KMO	1.495085	1.096754	2.038085	0.010953
ZNF684	1.173456	0.672819	2.046609	0.573014
KCNQ4	0.955529	0.563244	1.621031	0.866045
RIMS3	1.332874	0.993043	1.788999	0.055687
AKT3	1.199744	1.003789	1.433952	0.045336
ETV3	0.992453	0.597376	1.648814	0.976665
ACADM	1.131812	0.729602	1.75575	0.58046
SLAMF1	1.303559	1.005439	1.690074	0.045403
CD48	1.036252	0.866288	1.239562	0.696839
PADI2	1.010765	0.8852	1.154141	0.874294
SDHB	2.484819	1.514236	4.077518	0.000316
MFAP2	1.338463	1.021031	1.754583	0.034802
RPF1	2.108843	1.086434	4.093406	0.027458
KDM5B	0.61919	0.480206	0.798399	0.000219
UAP1	0.997095	0.752029	1.322022	0.983871
CTBS	0.83386	0.559449	1.24287	0.372264
KLHL12	1.690074	1.021535	2.796135	0.041061
SSX2IP	1.004603	0.715896	1.40974	0.978805
ZNHIT6	1.058379	0.682584	1.641067	0.799851
PLA2G2D	1.415292	0.931637	2.150035	0.103515
RBBP5	0.95298	0.586171	1.549328	0.845993
GBP3	1.21079	0.906324	1.617538	0.195541
GBP1	1.122308	0.962577	1.308546	0.140738
KIF17	1.081886	0.930846	1.257435	0.304943
GPR89A	0.645981	0.34921	1.194957	0.163793
CD160	0.677283	0.470681	0.974573	0.035845
ECE1	1.316261	1.087437	1.593235	0.004799
HMGCL	0.959659	0.65363	1.408971	0.83355
GALE	1.543678	0.972286	2.450863	0.06565
ID3	1.085562	0.840811	1.401558	0.528816
CR2	1.003925	0.74419	1.354311	0.979542
CD46	0.955391	0.611728	1.492121	0.840997
PRPF3	0.90045	0.474491	1.708799	0.748359
APH1A	1.993896	1.143889	3.475531	0.014927

SLC2A1	0.969464	0.765287	1.228113	0.797163
EBNA1BP2	1.403785	0.905556	2.176135	0.129413
CDC20	0.974631	0.770995	1.232052	0.829857
MPL	0.991706	0.866584	1.134894	0.903663
ARTN	1.004457	0.865044	1.16634	0.953479
IPO13	1.8699	1.19919	2.91574	0.005756
ATP6V0B	1.060836	0.759202	1.482308	0.729349
B4GALT2	0.840462	0.659768	1.070644	0.159348
ERI3	1.359715	0.800663	2.309118	0.255456
PTCH2	1.281599	0.890254	1.844973	0.181996
AKR1A1	1.375837	0.880946	2.148745	0.160708
PRDX1	1.359324	1.022539	1.807032	0.034566
PIK3R3	1.013529	0.774209	1.326828	0.922102
BLZF1	0.689337	0.452382	1.050408	0.08343
SLC19A2	0.851534	0.62471	1.160715	0.309185
FAAH	1.201684	0.965023	1.496384	0.100631
NSUN4	1.343657	0.780011	2.314601	0.287067
TMED5	0.855442	0.540541	1.353794	0.504997
DR1	1.405512	0.841291	2.348133	0.193607
CNN3	1.02623	0.855787	1.230619	0.779935
F3	0.894071	0.777135	1.028602	0.117433
ABCD3	1.056024	0.691396	1.61295	0.800851
VAMP4	0.698035	0.446997	1.090058	0.113929
DPH5	0.767319	0.500125	1.177261	0.225232
FASLG	1.074372	0.806953	1.430411	0.62327
PTBP2	0.537475	0.325318	0.887992	0.015364
TNFSF4	1.196193	0.986706	1.450156	0.068194
PRDX6	1.446956	0.766569	2.73124	0.254353
DARS2	1.031114	0.635364	1.673365	0.901296
IRF6	0.882433	0.783177	0.994268	0.039938
RCAN3	0.969934	0.718205	1.309895	0.842165
SYF2	1.239724	0.714642	2.150611	0.444526
SLC35A3	0.422248	0.24722	0.721195	0.001596
RCOR3	0.442313	0.252808	0.773871	0.004261
STMN1	0.769527	0.587722	1.007572	0.056766
MAN1C1	1.268576	0.961291	1.674087	0.092762
NEK2	0.981619	0.749271	1.286018	0.892913
RPS6KA1	1.920265	1.310662	2.813401	0.000813
DHDDS	1.118469	0.593473	2.107887	0.729141
NENF	1.586513	1.037222	2.426696	0.033295
NSL1	0.779468	0.415386	1.462664	0.437847
ARID1A	0.984819	0.549324	1.765567	0.959039
CENPF	0.847462	0.678148	1.059049	0.145544
RPA2	2.161095	1.165691	4.006492	0.014415
PPP1R8	1.29057	0.75067	2.218779	0.356192
STX12	1.396485	0.939952	2.074757	0.098255
SLC5A9	1.205463	0.863579	1.682697	0.272171
OSBPL9	0.811992	0.480944	1.37091	0.435756
TXNDC12	0.918094	0.514592	1.63799	0.772342
RCN2	0.674037	0.437386	1.038729	0.073815
MUC5B	0.43659	0.222927	0.855036	0.015665
CTSD	1.352244	1.12285	1.628501	0.001465
STAG1	0.681655	0.448041	1.03708	0.073468
STK11	1.245819	0.819989	1.892788	0.30303
MMP8	1.037786	0.947929	1.136161	0.422161
APOA1	1.259935	0.768194	2.066454	0.36003
KPTN	1.246416	0.88388	1.757651	0.209084
RPS25	1.052449	0.711911	1.555884	0.797719

KIF14	0.908605	0.68848	1.19911	0.498325
DDX59	0.748107	0.42369	1.320925	0.317091
ATF6	1.032846	0.624045	1.709445	0.899957
CRYGD	1.152338	1.006673	1.319081	0.039742
MREG	1.07524	0.878776	1.315627	0.481007
FASTKD2	1.036792	0.640851	1.677361	0.882974
NRP2	1.149893	0.802649	1.647363	0.446389
CREB1	0.587365	0.348865	0.988913	0.045295
KLF7	0.984084	0.710588	1.362844	0.923064
B4GALT6	1.01018	0.870624	1.172106	0.893782
CA14	1.688849	1.066985	2.67315	0.025306
SPCS2	0.68047	0.349423	1.325152	0.2576
USP35	0.836085	0.611187	1.143739	0.262774
CASP8AP2	0.915242	0.635113	1.318926	0.634727
HMGN3	1.026794	0.679138	1.552418	0.900231
ANKRD13(	0.961944	0.559392	1.654181	0.888441
PHF3	0.73897	0.463811	1.177371	0.20306
PLAGL1	0.811249	0.623807	1.055015	0.118653
FBXO30	0.719491	0.460249	1.124755	0.148678
TNFAIP3	1.054367	0.827652	1.343185	0.668226
AKAP7	1.34315	1.084944	1.662807	0.00676
RAB32	0.929859	0.748233	1.155572	0.511903
MYB	0.590362	0.450438	0.773753	0.000134
ALDH8A1	1.122663	0.75231	1.675336	0.571057
SGK1	1.078056	0.922427	1.259942	0.344729
RNF146	0.668652	0.456394	0.979627	0.038866
ARG1	0.878602	0.705614	1.093999	0.247314
PMFBP1	0.879671	0.580912	1.33208	0.544799
FBXL5	0.951865	0.689906	1.313291	0.763873
MED28	0.578038	0.354694	0.942017	0.02783
SLC16A7	0.780223	0.579929	1.049694	0.101097
ZNF430	0.691695	0.417975	1.144666	0.151502
VAMP8	1.267938	0.80852	1.988407	0.301095
DCLRE1B	1.669854	1.026437	2.716592	0.038918
FOXO3	0.841657	0.5484	1.291733	0.430272
ARMC2	1.319888	0.80603	2.16134	0.270025
GHRH	0.841607	0.509393	1.390483	0.500856
RPN2	0.995803	0.689553	1.438066	0.982103
TGIF2	0.822935	0.582641	1.162332	0.26867
OLFM3	0.599997	0.1994	1.805402	0.363426
PKD2	0.793041	0.630412	0.997624	0.047672
ABCG2	1.000469	0.729562	1.371971	0.997679
SPP1	0.972083	0.815825	1.15827	0.751492
STBD1	1.727155	1.068081	2.79292	0.025843
CCNI	0.711325	0.391994	1.290793	0.262552
RARRES1	0.944846	0.62837	1.420713	0.785154
MFSD1	1.021341	0.68735	1.517623	0.916766
RAB3GAP2	1.242304	0.72237	2.136466	0.432851
PPL	0.753207	0.513055	1.105771	0.147972
UBN1	1.389933	0.78386	2.464616	0.259887
KLF12	0.914101	0.768822	1.086832	0.309127
UCHL3	0.844759	0.498153	1.432527	0.531271
HS1BP3	1.374072	0.91882	2.054891	0.121711
WDR35	0.998151	0.829153	1.201594	0.984398
CCND2	0.791297	0.675283	0.927243	0.003806
ELL2	1.026679	0.815428	1.292658	0.822754
CYP20A1	0.994815	0.574249	1.723395	0.985206
NDUFB3	1.604392	0.98156	2.622433	0.059332

GTF3C3	0.718934	0.45787	1.128849	0.151723
SATB2	1.192098	0.871335	1.630943	0.27188
UBE2B	0.669347	0.400149	1.119647	0.126161
TRPM6	0.29742	0.153001	0.578155	0.000349
KLF9	1.182024	0.989177	1.412467	0.065736
TJP2	1.047043	0.674656	1.624977	0.837576
ITGB1BP1	1.108568	0.72311	1.699499	0.636353
CPSF3	1.162487	0.742433	1.820199	0.510455
PIGZ	0.934955	0.682103	1.281538	0.675905
SENP5	0.640479	0.426336	0.962183	0.031904
CCDC92	1.065042	0.7789	1.456303	0.693043
HEATR1	0.931322	0.647247	1.340078	0.701544
RAD23B	1.059389	0.671049	1.672463	0.80441
FKBP15	1.134315	0.846988	1.519114	0.397749
CTNNAL1	1.2185	0.956291	1.552605	0.10994
SET	0.982168	0.609689	1.582207	0.941041
GLE1	1.345878	0.716441	2.528313	0.355806
RAB14	1.510264	0.840728	2.713001	0.167745
TRIM32	1.403492	1.154214	1.706608	0.00068
FBXW2	0.931089	0.568692	1.524423	0.776527
PHF19	1.141057	0.765993	1.69977	0.516377
NEK6	1.354771	1.051364	1.745737	0.018917
BSPRY	1.140937	0.916905	1.419706	0.237143
PPP6C	1.212319	0.620986	2.366748	0.572697
NDUFA8	1.843844	1.207761	2.814927	0.004591
HDHD3	1.383681	0.929946	2.058799	0.109213
RBM18	1.196833	0.744006	1.925267	0.458816
SLC46A2	1.081421	0.885177	1.321173	0.443584
HSDL2	1.369937	0.897368	2.091369	0.144766
MAPKAP1	2.047436	1.027877	4.078304	0.041533
NR4A3	0.907021	0.712712	1.154305	0.427561
INVS	0.795922	0.535328	1.183372	0.259342
GALNT12	1.287435	1.058465	1.565937	0.011451
DENND1A	1.137828	0.800133	1.618048	0.472291
ALG2	1.665209	0.972264	2.852025	0.063239
CSF3R	0.809149	0.669556	0.977846	0.028391
KDSR	0.668011	0.406754	1.097073	0.110949
VPS4B	0.836395	0.498722	1.402696	0.498267
ONECUT2	1.299788	0.933293	1.810201	0.120788
ZBTB45	1.437042	0.969439	2.13019	0.071009
YLPM1	0.866052	0.518619	1.446239	0.58254
PROX2	0.896392	0.612249	1.312405	0.573902
VSX2	0.226341	0.011232	4.561009	0.332248
FCF1	0.912809	0.504573	1.65134	0.762943
PGF	1.129657	0.889548	1.434577	0.317329
IFI27L2	1.577454	1.229877	2.023261	0.000332
NEK9	1.324255	0.811032	2.162247	0.261566
ACYP1	0.893789	0.592595	1.34807	0.592293
NPC2	1.251903	0.925075	1.694198	0.145548
DNAL1	0.558812	0.327886	0.952375	0.032407
ACOT2	1.734175	1.230796	2.443428	0.001649
LTBP2	0.831794	0.620086	1.115782	0.219099
MLH3	0.881349	0.623437	1.245956	0.47459
TTLL5	0.686196	0.400681	1.175163	0.170079
FLVCR2	1.281802	1.020307	1.610316	0.032953
ABCD4	0.874863	0.547134	1.398899	0.576679
DLST	1.475218	0.825162	2.637384	0.189638
TGFB3	0.880356	0.585487	1.32373	0.540324

RBM25	0.603674	0.376652	0.967529	0.035983
ALDH6A1	0.72351	0.469927	1.113934	0.141585
GPR68	1.018638	0.746874	1.389288	0.907152
EIF2B2	1.138372	0.629256	2.0594	0.668302
COQ6	0.979177	0.473118	2.026531	0.954783
ZNF410	0.482567	0.255535	0.911309	0.024686
RHOQ	1.085171	0.688092	1.711395	0.725098
GPR75	0.855754	0.582274	1.257679	0.42782
SUPT7L	0.729972	0.41673	1.27867	0.271123
KLHL29	0.952113	0.774236	1.170856	0.641884
DNMT3A	0.638684	0.448329	0.909861	0.013023
TMEM214	1.204673	0.774291	1.874279	0.408989
ATAD2B	0.609703	0.390904	0.950969	0.029137
FKBP1B	0.60759	0.423184	0.872352	0.006935
ATL2	0.481992	0.264325	0.878905	0.017262
YPEL5	0.917889	0.625727	1.346465	0.661192
FAM98A	1.063896	0.667422	1.695891	0.794592
YIPF4	0.698637	0.443408	1.100777	0.122096
AFTPH	1.76376	1.061954	2.929364	0.028366
CNRIP1	0.972027	0.810749	1.165387	0.759221
BCL11A	0.919381	0.714737	1.182617	0.512914
CRIPT	0.663135	0.396076	1.110261	0.118244
EPCAM	0.933775	0.806161	1.081591	0.360784
SLC17A5	1.229241	0.782971	1.929871	0.369799
OGFRL1	1.093458	0.88618	1.349218	0.404756
IDE	0.794538	0.500359	1.261674	0.329659
ELOVL3	1.165596	0.908844	1.494882	0.227414
IFIT3	1.361361	1.118823	1.656478	0.002059
NKX2-3	1.146428	1.038859	1.265137	0.006562
IFIT2	1.314973	1.073936	1.610109	0.008039
GPAM	0.89101	0.600455	1.322162	0.566585
CUTC	1.021215	0.632376	1.649146	0.931583
CNNM1	1.248587	1.066074	1.462347	0.005896
MXI1	1.078001	0.730943	1.589846	0.704768
SMNDC1	1.121722	0.560988	2.242935	0.745255
HELLS	1.141891	0.887135	1.469803	0.302932
TCTN3	1.058838	0.645149	1.737797	0.821069
AVPI1	1.408372	1.03654	1.913588	0.028566
KCNIP2	0.708206	0.44432	1.128816	0.146908
GOT1	1.717964	1.081784	2.728272	0.021843
SFRP5	1.265945	1.007782	1.590243	0.042705
GNA13	0.959581	0.650265	1.416031	0.835371
HOXB8	1.189085	1.034867	1.366285	0.014544
HOXB5	1.176786	1.058919	1.307772	0.002502
HOXB3	1.111323	1.014147	1.21781	0.023767
DUSP1	1.024658	0.836611	1.254972	0.813847
PANK3	0.852859	0.561433	1.295558	0.455602
TEK	1.055334	0.782941	1.422495	0.723665
RCL1	1.076095	0.767237	1.509285	0.670914
INSL6	0.895904	0.676282	1.186846	0.443617
MLANA	0.808605	0.589843	1.108502	0.186851
CD274	1.338023	1.001176	1.788202	0.049079
NUP43	0.764662	0.464712	1.258218	0.290975
MTHFD1L	0.928709	0.701183	1.230064	0.60598
PCMT1	1.106476	0.718344	1.704322	0.646188
PLEKHG1	0.894476	0.695864	1.149775	0.384024
MYCT1	1.090464	0.969205	1.226894	0.149896
WDR55	0.879094	0.549626	1.406059	0.590733

ARAP3	0.987004	0.846956	1.15021	0.866945
SLC25A2	0.547991	0.238884	1.257073	0.155639
TNN	0.802865	0.595479	1.082477	0.149824
MRPS14	1.285565	0.676955	2.44134	0.442687
CENPL	1.27466	0.754521	2.153364	0.36435
ACAT2	1.512864	0.977278	2.341973	0.063329
TCP1	1.08306	0.701806	1.671429	0.718527
SNX19	1.139163	0.781339	1.660856	0.498212
KCNJ5	0.659938	0.31142	1.398491	0.278074
ARR3	1.107357	0.77223	1.58792	0.579236
PDZD11	1.684527	0.955306	2.97039	0.071551
SLC10A7	0.473878	0.256494	0.875497	0.017102
NUDCD1	0.906332	0.608172	1.350667	0.628972
ENY2	1.433645	0.806158	2.549548	0.220058
MASTL	1.390298	0.845301	2.286673	0.194298
KIAA1217	0.810659	0.644769	1.019231	0.072351
PLXDC2	0.969518	0.827266	1.13623	0.702176
EPC1	0.665903	0.356203	1.244871	0.202735
IQSEC3	1.057917	0.907902	1.232721	0.47053
CCDC77	0.73154	0.438598	1.220139	0.231044
TAF12	1.404378	0.797755	2.472282	0.239236
MTRF1	0.795419	0.499295	1.267169	0.335368
DNAJC15	1.459782	1.035717	2.057477	0.030743
UFM1	0.638774	0.395556	1.031543	0.066808
WBP4	1.015004	0.596801	1.726259	0.956169
ELF1	0.907345	0.565553	1.455697	0.686843
SMAD9	0.663488	0.400822	1.098284	0.110626
HSPH1	1.098459	0.779737	1.547461	0.591221
KBTBD7	1.134524	0.803969	1.600988	0.4726
ALG5	0.676517	0.368845	1.240833	0.206685
EXOSC8	0.955382	0.521156	1.751406	0.882652
ETF1	0.968703	0.663266	1.414794	0.869312
TGFBI	1.006839	0.89511	1.132515	0.909575
FAM53C	1.044735	0.662608	1.647233	0.850582
SIL1	1.062653	0.719708	1.569014	0.759872
PAIP2	0.78453	0.463124	1.328991	0.366866
MYOT	1.341629	0.916654	1.963628	0.130488
KDM3B	0.678648	0.441488	1.043206	0.077204
EGR1	0.899797	0.802385	1.009036	0.070903
SERP1	0.955089	0.524613	1.738797	0.880514
PLS1	1.154396	0.962183	1.385006	0.122321
ZFP30	0.678446	0.474678	0.969686	0.033264
NR2C1	0.523077	0.303365	0.901917	0.019735
UTP20	0.909875	0.63771	1.298196	0.602485
TMPO	0.880635	0.595977	1.301254	0.523417
ARL1	0.723633	0.433301	1.208501	0.216382
GLT8D2	0.750873	0.340883	1.65397	0.477013
SOCS2	1.192839	1.052164	1.352323	0.005884
NFYB	0.587394	0.349748	0.986515	0.044295
APAF1	0.990066	0.726208	1.349794	0.94966
DUSP4	0.961968	0.731439	1.265152	0.781479
CLU	1.014585	0.918111	1.121195	0.776388
TNFRSF10	1.308919	0.976694	1.754151	0.071529
SORBS3	1.30672	1.065387	1.60272	0.01023
PTK2B	1.505719	1.031903	2.197096	0.033766
CHRNA2	1.144066	0.723228	1.809784	0.565169
PPP3CC	1.250973	0.859806	1.820101	0.241825
PDLIM2	1.758687	1.251149	2.472111	0.001155

EPHX2	1.175541	0.900142	1.535199	0.23503
RNF170	0.96931	0.567044	1.656947	0.909278
NPPB	1.275562	0.851738	1.910279	0.237536
UBIAD1	1.380831	0.834146	2.285805	0.209558
TARDBP	1.109695	0.606052	2.031875	0.735917
TNFRSF8	1.178325	0.95468	1.454361	0.126495
ZNF706	1.215749	0.674183	2.192351	0.516075
LYPLA1	1.140758	0.758962	1.714618	0.526464
CRISPLD1	1.2818	1.077247	1.525195	0.005129
COPS5	1.024569	0.552504	1.899973	0.938598
RDH10	1.481103	1.076977	2.036874	0.015686
EPX	0.874641	0.756218	1.011608	0.071157
AKAP1	1.329546	0.887229	1.992375	0.167529
COIL	0.770016	0.469993	1.261561	0.299486
TRIM25	1.288424	0.85713	1.936736	0.222987
SCPEP1	1.103503	0.965111	1.261739	0.149713
SPOP	2.058138	1.055721	4.012361	0.034077
SLC35B1	0.672537	0.340302	1.329131	0.253724
TEX14	1.151017	0.928022	1.427597	0.200515
FAM117A	0.980619	0.76523	1.256635	0.877086
NCAPH	1.079279	0.800206	1.455681	0.617211
MND1	0.751746	0.460157	1.228106	0.254504
TRIM6	0.67362	0.531318	0.854033	0.001102
ADCY7	1.242954	1.012964	1.525164	0.03722
TSHZ3	0.914331	0.774202	1.079824	0.291345
ECHDC2	1.012811	0.844565	1.214573	0.890763
TAS2R10	0.826283	0.616021	1.108313	0.202812
PRB2	0.761572	0.391675	1.480801	0.422081
PYROXD1	0.847368	0.513816	1.397452	0.516424
PSPC1	1.025695	0.508095	2.070577	0.943567
ZNF549	0.717583	0.479727	1.073372	0.10624
A1BG	0.581599	0.412148	0.82072	0.00204
ZSCAN18	0.972422	0.723576	1.306849	0.852888
ZNF211	0.930499	0.622211	1.391535	0.725724
PDZRN3	0.657207	0.331545	1.302755	0.22922
LHX4	0.624643	0.442123	0.882511	0.007612
RNF2	1.30712	0.843288	2.026072	0.231024
SEC22A	1.160046	0.647572	2.078081	0.617699
CSTA	1.0543	0.93783	1.185235	0.375994
DPPA4	1.122446	1.013984	1.242511	0.025894
POPDC2	1.484553	0.889168	2.478609	0.13084
B4GALT4	1.476823	0.848024	2.571869	0.168345
CD80	1.371542	0.979392	1.920709	0.065949
KIF18A	1.11669	0.802564	1.553767	0.512537
MAPK8IP1	1.068178	0.697163	1.63664	0.761922
CRY2	1.716532	1.038475	2.837317	0.0351
PEX16	1.7519	1.160465	2.644764	0.007627
DEPDC7	0.759012	0.626651	0.919331	0.004799
CAT	1.212537	0.963795	1.525475	0.099937
PILRB	0.844813	0.57792	1.23496	0.384001
ZMYM2	0.482932	0.308789	0.755283	0.001423
GJB6	0.706149	0.463798	1.075138	0.104765
TBC1D15	1.014626	0.650967	1.581441	0.948872
HCRTR1	1.062067	0.79771	1.414032	0.680089
ZCCHC17	3.177377	1.614532	6.253034	0.000817
FABP3	0.82566	0.559419	1.218612	0.334783
KHDRBS1	1.120733	0.593193	2.117426	0.725483
TMEM39B	1.357686	0.797796	2.310505	0.259652

CCRL2	1.329983	0.997748	1.772847	0.051824
CCR2	1.098824	0.994414	1.214196	0.064314
POLR3GL	1.955833	1.150256	3.325595	0.013256
TNFSF10	1.055482	0.890243	1.251392	0.534201
ZNF639	0.703001	0.454506	1.087359	0.113285
PIK3CA	0.53227	0.322601	0.87821	0.013576
PDS5A	0.801318	0.500007	1.284202	0.357326
TMEM156	1.490973	1.080658	2.057083	0.015002
LIAS	0.810783	0.482119	1.363499	0.429008
ZSCAN20	0.820704	0.505185	1.333285	0.424805
CLCC1	1.029126	0.754463	1.403779	0.856173
GPSM2	0.760093	0.5587	1.034083	0.080712
GTDC1	0.891323	0.539204	1.473386	0.653693
CXCR4	1.105551	0.894306	1.366694	0.353686
ZRANB3	0.717461	0.485739	1.059725	0.095222
ACVR2A	1.261856	0.935013	1.702952	0.12835
POLK	0.794249	0.518409	1.216859	0.28993
FLT3	1.115388	0.918904	1.353886	0.269359
RPL21	1.205222	0.767781	1.891894	0.417158
MTIF3	0.817965	0.52627	1.271336	0.371844
GTF3A	1.236537	0.780295	1.959546	0.366077
RASL11A	1.482679	1.097513	2.003018	0.010281
UBL3	1.095934	0.777578	1.544631	0.600846
FYTTD1	0.740526	0.487382	1.125152	0.159288
XPNPEP2	0.908723	0.644376	1.281515	0.585253
SASH3	1.493851	1.098341	2.031783	0.010536
OCRL	0.923916	0.600562	1.42137	0.718799
MRPS2	1.333975	0.854761	2.081856	0.204472
FMOD	1.026004	0.790614	1.331478	0.846908
LAX1	0.86511	0.698788	1.071019	0.183473
KIAA1191	1.216617	0.676525	2.187882	0.512571
COPA	1.267828	0.752253	2.136766	0.37291
CD244	0.923847	0.701128	1.217314	0.573583
LY9	0.922299	0.717515	1.185528	0.527765
HS3ST2	0.575367	0.203268	1.628628	0.297778
RBBP6	1.026138	0.573561	1.835827	0.930721
ZC3H7A	1.037405	0.600704	1.791581	0.895196
SERAC1	0.641033	0.403176	1.019215	0.060174
ANXA11	1.995189	1.17548	3.386513	0.0105
ZNF205	0.874706	0.620148	1.233756	0.44554
RPL5	1.026229	0.719611	1.463493	0.886314
ODF2L	0.909645	0.634037	1.305055	0.607088
SPATA1	0.836511	0.592121	1.18177	0.31125
LRRC39	0.875798	0.552082	1.389324	0.573227
RWDD3	0.774913	0.512032	1.172758	0.227745
ZNF644	0.761275	0.499714	1.159742	0.204098
CCDC18	0.778939	0.50174	1.209282	0.26562
RPAP2	0.751562	0.531465	1.062809	0.106221
BBS9	0.560975	0.312709	1.006344	0.052531
PMS2	0.748184	0.445829	1.255591	0.272078
ZMIZ2	0.939933	0.656148	1.346456	0.735513
OCM	0.866912	0.650821	1.154751	0.328898
EEPD1	0.774802	0.579375	1.036148	0.08534
KIAA0087	1.109735	0.964177	1.277268	0.146657
KLHL7	0.744402	0.448279	1.23614	0.253991
HERPUD2	0.859198	0.482634	1.529566	0.606049
CBX3	0.862577	0.56969	1.306043	0.484892
HNRNPA2	0.629482	0.37385	1.05991	0.081667

WIPF3	0.885994	0.634512	1.23715	0.477322
NPY	1.205325	0.792065	1.834202	0.383335
FAM126A	1.017344	0.730506	1.416811	0.918951
HOXA7	1.2661	1.105328	1.450257	0.000661
INHBA	1.194504	1.003314	1.422126	0.045809
FKBP9	0.947633	0.730744	1.228896	0.68502
ARL4A	0.994575	0.803394	1.231251	0.960167
POLM	0.966237	0.612419	1.52447	0.882638
RAMP3	1.119747	0.794514	1.578115	0.518246
TWIST1	0.553223	0.391132	0.782485	0.000818
SMU1	1.644814	0.848136	3.189832	0.140872
GLIPR2	1.190613	0.978024	1.449411	0.082116
CLTA	3.467843	1.740232	6.910538	0.000408
RECK	1.312374	1.105218	1.558358	0.001927
SPINK4	0.83806	0.625955	1.122038	0.235394
ACO1	1.038079	0.673805	1.599287	0.865419
DCAF10	0.751245	0.441245	1.279038	0.292117
KIAA1549	0.719338	0.589175	0.878259	0.001218
TRIM24	0.573131	0.414333	0.792788	0.000772
CALD1	1.061094	0.804846	1.398927	0.674125
NUDT10	0.833663	0.705123	0.985634	0.033227
NEUROG3	1.385544	0.638429	3.006962	0.409452
PLAU	0.981843	0.797071	1.209449	0.863235
SRGN	1.03332	0.897551	1.189625	0.648349
BICC1	0.81684	0.584765	1.141019	0.235478
CISD1	1.71939	1.068579	2.766574	0.02553
EGR2	1.1567	0.99205	1.348678	0.063157
ECD	0.892969	0.462573	1.723821	0.735872
P4HA1	1.03878	0.693163	1.556723	0.853751
SLC25A16	0.816193	0.436772	1.525216	0.524335
ZWINT	1.333648	0.962544	1.847829	0.083539
VPS26A	1.462575	0.854305	2.503937	0.165763
RBM19	1.056033	0.669235	1.66639	0.814781
CIT	0.88537	0.642147	1.220716	0.457513
IFT81	0.653692	0.442719	0.965201	0.032509
ACADS	1.439149	0.993678	2.084329	0.054054
HVCN1	1.122277	0.905155	1.391481	0.292987
NME2P1	1.04058	0.695318	1.557283	0.846662
DDX54	1.456605	0.944797	2.245665	0.088593
MED13L	0.764275	0.524944	1.112722	0.160717
CDKN2C	1.034126	0.821196	1.302267	0.775438
RNF11	1.115244	0.686537	1.811656	0.659482
RASSF8	1.049234	0.788248	1.396631	0.741888
ITPR2	0.567199	0.434752	0.739997	2.93E-05
CCDC91	0.95877	0.568355	1.617368	0.874602
NECAB1	1.041014	0.836796	1.295069	0.718273
WWP1	0.836801	0.531293	1.317987	0.442067
ACOT9	1.752505	1.233327	2.490236	0.001749
PRDX4	1.527209	1.17753	1.980729	0.001414
PKN1	1.89707	1.270486	2.832674	0.001746
GIPC1	1.210224	0.932264	1.571059	0.151817
EBPL	0.921711	0.672957	1.262414	0.611469
ATP7B	0.912551	0.756204	1.101223	0.339891
ZC3H13	0.99694	0.649478	1.53029	0.988817
GUCY1B2	0.89976	0.647676	1.249957	0.528851
NLN	1.030367	0.694671	1.528288	0.881769
CENPK	0.866381	0.628363	1.194557	0.381467
OPTN	1.272207	1.059983	1.52692	0.009722

ATF1	1.177003	0.711635	1.946695	0.525544
TSFM	0.528355	0.238699	1.169502	0.11555
ARHGAP9	1.646335	1.164966	2.326607	0.004724
NCKAP1L	1.464765	0.912899	2.350244	0.113601
MMP19	1.094276	0.944746	1.267474	0.229451
PFDN5	1.406716	0.87437	2.263172	0.159546
SPATS2	1.107787	0.768964	1.595902	0.582622
ORMDL2	2.224721	1.231933	4.017577	0.00801
NR4A1	1.120265	0.968387	1.295962	0.126561
PDE1B	1.485739	1.138762	1.938439	0.003528
CDK2	0.822528	0.531601	1.272669	0.380334
LRP1	1.061279	0.960485	1.17265	0.242761
NFE2	0.979532	0.728144	1.317709	0.89129
IKZF4	1.139174	0.733285	1.76973	0.56209
SMUG1	1.029374	0.529303	2.0019	0.932016
TUBA1B	1.236092	0.930207	1.642561	0.14396
KBTBD4	1.272522	0.732997	2.209167	0.391827
SARDH	1.193349	0.957668	1.487029	0.115337
DBH	1.135756	0.837757	1.539756	0.412304
ATPAF1	2.009545	1.120127	3.60519	0.019264
STIL	1.014129	0.735036	1.399193	0.931916
HJURP	0.904686	0.701832	1.166172	0.439373
COL10A1	0.672871	0.417569	1.084264	0.103606
AMD1	0.97821	0.652534	1.466429	0.915065
USP45	0.807905	0.603843	1.080928	0.150985
MORF4L2	0.82337	0.5109	1.326947	0.424762
RAB9B	1.033615	0.818855	1.304701	0.78084
RAB9A	1.352864	0.793972	2.305172	0.266358
METTL8	0.927944	0.594467	1.448492	0.742041
TTC21B	0.716369	0.446388	1.149639	0.166927
NMI	1.261447	0.818012	1.945265	0.293267
TNFAIP6	0.989965	0.81796	1.198139	0.917506
BAZ2B	0.791301	0.538546	1.162681	0.233166
SLC36A1	0.831582	0.67915	1.018228	0.074243
LPGAT1	0.895826	0.654269	1.226564	0.492602
BATF3	1.611657	1.15873	2.241626	0.004581
G0S2	1.012282	0.892773	1.14779	0.848955
KCNJ2	1.168198	0.937945	1.454975	0.16514
RAP2C	1.257788	0.798582	1.981052	0.322392
EXOSC9	0.941722	0.582223	1.523197	0.806655
PLA2G12A	0.951154	0.659918	1.370921	0.788316
B9D2	0.941085	0.645352	1.372338	0.752393
PFKFB2	1.123801	0.918973	1.374283	0.255581
RAB38	0.91806	0.770422	1.09399	0.33921
MXD4	1.146286	0.815527	1.611194	0.431873
CKS2	1.155248	0.808452	1.650807	0.428118
ACSL3	1.393075	0.908618	2.135837	0.128399
CHPF	1.086887	0.872499	1.353954	0.457332
DNPEP	1.52749	0.838462	2.782744	0.166281
OBSL1	0.968025	0.838864	1.117074	0.656497
FAM124B	1.35314	1.075489	1.702471	0.00985
SLC12A4	1.300962	0.931747	1.816483	0.122378
GCNT7	0.742626	0.508291	1.084996	0.123986
PI3	1.15074	0.927298	1.428023	0.202415
SNX21	1.346233	0.926824	1.955435	0.118528
SLPI	0.98624	0.89932	1.081562	0.768499
TTPAL	1.059455	0.642602	1.746718	0.820891
PREX1	1.507283	1.11701	2.033913	0.00728

KCNS1	1.210236	0.765783	1.912646	0.413836
SDC4	1.158246	0.971197	1.38132	0.102102
NCOA3	1.099099	0.740598	1.63114	0.638992
PIGT	0.951937	0.652851	1.38804	0.797969
MATN4	1.052092	0.871774	1.269707	0.596537
NCOA5	1.199615	0.671781	2.142178	0.538414
VAPB	1.421984	0.738692	2.73732	0.29208
PARD6B	1.04509	0.728732	1.498784	0.81053
CHD6	0.653876	0.438771	0.974437	0.036871
PLCG1	1.050672	0.866703	1.273691	0.614749
TOX2	0.994117	0.687885	1.436676	0.974946
ARFGEF2	0.818256	0.536175	1.248739	0.352364
ZNFX1	1.300202	0.821051	2.058979	0.263014
ZNF831	1.225013	0.911254	1.646802	0.17883
CSE1L	1.023479	0.699854	1.496754	0.904743
RAB22A	0.707788	0.403585	1.241282	0.22788
STAU1	1.558486	0.771128	3.149776	0.216461
CDH26	0.709402	0.552535	0.910805	0.007088
SNAI1	1.000879	0.849632	1.17905	0.991618
MOCS3	1.188465	0.705695	2.001499	0.516176
STX16	1.378388	0.816703	2.326371	0.229466
PPP4R1L	1.09199	0.842757	1.414931	0.505574
PMEPA1	0.856347	0.662574	1.106791	0.236101
RNF114	0.803405	0.449655	1.435457	0.459773
DDX27	1.109835	0.642349	1.917546	0.708765
BCAS4	0.99052	0.758165	1.294084	0.944323
TP53TG5	0.881952	0.565705	1.374991	0.57928
ZBP1	1.561004	1.16738	2.087352	0.002666
NEURL2	0.978108	0.602373	1.588211	0.928687
MTRR	1.019523	0.591221	1.758101	0.944556
FASTKD3	1.011944	0.611879	1.673582	0.963107
PEPD	1.408302	1.007962	1.967649	0.04481
IQSEC2	1.089013	0.88286	1.343304	0.425809
VAMP7	1.046489	0.612501	1.78798	0.867944
IL9R	0.76546	0.515352	1.136948	0.185464
XG	0.848882	0.567243	1.270355	0.425717
STAMBP	0.817784	0.433819	1.541588	0.534013
NAGK	1.115983	0.788951	1.578574	0.535126
MCEE	0.756089	0.449457	1.271913	0.292064
PAIP2B	1.009351	0.791839	1.286613	0.940082
SNRNP27	1.130725	0.590663	2.16458	0.710772
MPHOSPH	0.910319	0.596612	1.388979	0.662947
IL17C	1.116513	0.789948	1.578082	0.532427
ATP8A1	1.081487	0.905065	1.292298	0.3886
USP22	0.977044	0.642703	1.485312	0.913462
POF1B	0.956992	0.493863	1.854428	0.896373
HIF3A	1.467873	1.032353	2.087126	0.032576
ZNF576	1.157185	0.585374	2.287555	0.674584
IRGC	1.112179	0.768264	1.610049	0.573236
ZNF45	0.854663	0.572041	1.276918	0.44329
LYPD3	2.211743	1.445728	3.383628	0.000253
CEACAM8	0.992223	0.882085	1.116113	0.896519
USP9X	1.068337	0.699443	1.631789	0.759701
CRISP2	0.787472	0.519644	1.193341	0.259933
F13A1	0.986273	0.880817	1.104355	0.810663
TRERF1	0.802625	0.649197	0.992314	0.04223
PACSIN1	1.26592	1.111251	1.442116	0.00039
BTN2A2	1.236425	0.909688	1.680517	0.175279

SIRT5	0.736971	0.456936	1.188626	0.210774
MRS2	0.897657	0.549756	1.465718	0.666042
WRNIP1	0.949377	0.627997	1.435225	0.805393
SNRPC	1.340062	0.780783	2.299954	0.288195
SERPINB6	1.407072	1.117124	1.772275	0.003723
XPO5	0.938642	0.596	1.47827	0.784664
ABCC10	1.310107	0.841402	2.039905	0.231855
PEX6	1.106093	0.759059	1.611787	0.599652
NQO2	1.057286	0.756107	1.478433	0.744697
UNC5CL	1.220066	0.829775	1.793935	0.311891
AARS2	1.328772	0.813762	2.16972	0.25587
ZNF391	1.023653	0.850433	1.232154	0.804791
RPS10	1.054917	0.669839	1.661369	0.817539
MOCS1	1.143085	0.818601	1.59619	0.432446
MED20	2.708696	1.424226	5.151592	0.00238
OR2B6	1.142704	0.875804	1.490941	0.325672
TBCC	2.286971	1.458134	3.586939	0.000315
SPDEF	1.712441	0.756266	3.877543	0.197046
TCP11	0.70146	0.420092	1.171282	0.175237
MAD2L1BI	1.86856	0.973413	3.586881	0.060248
APOBEC2	1.17362	0.780658	1.764387	0.441534
KLHDC3	1.350092	0.805132	2.26391	0.255063
GNMT	1.061114	0.803203	1.401841	0.676304
DNAH8	0.619488	0.292945	1.310028	0.210124
TREM1	1.083114	0.944137	1.242549	0.254487
MEA1	2.58428	1.337583	4.992962	0.004719
CDKN1A	1.175044	0.9916	1.392425	0.062524
SOX4	0.849053	0.703544	1.024657	0.088006
GLO1	1.339867	0.919074	1.953318	0.128212
CPNE5	1.102327	0.81698	1.487338	0.523855
KCNK17	1.004028	0.899052	1.121262	0.94312
RREB1	0.541786	0.330372	0.888488	0.015164
SSR1	0.699981	0.430983	1.136875	0.149433
RIOK1	1.022035	0.620042	1.684653	0.931882
NRN1	1.102775	0.771773	1.575738	0.591093
SLC35B3	0.947681	0.589586	1.52327	0.824375
RPP40	1.032732	0.749179	1.423606	0.844092
ATXN1	0.856513	0.709654	1.033763	0.106538
NUP153	0.738671	0.446105	1.22311	0.239102
DEK	1.298996	0.859856	1.962409	0.213983
EEF1E1	0.961664	0.636078	1.453908	0.852952
RUNX2	0.962773	0.749658	1.236472	0.766323
LRRFIP1	1.336258	0.872381	2.046795	0.182727
RAB17	2.494763	1.597615	3.89571	5.81E-05
CXCL6	1.320713	0.80495	2.166945	0.270853
EREG	1.080815	0.995271	1.173712	0.064704
AHNAK	1.117382	0.920867	1.355834	0.260748
WNT1	1.068606	0.659947	1.730318	0.787277
SH3TC1	1.458707	1.205524	1.765062	0.000104
CNOT1	0.906372	0.642088	1.279436	0.576215
LRRC29	1.017972	0.698972	1.482558	0.926013
BBS2	0.882141	0.582915	1.334967	0.553019
MT1G	1.107237	0.922593	1.328835	0.273777
MT2A	1.04253	0.864748	1.256863	0.662388
GOT2	1.69498	1.024988	2.802917	0.039769
DOK4	1.028571	0.826354	1.280272	0.800868
GPR18	0.870963	0.654898	1.158314	0.342264
CLYBL	1.443765	0.859041	2.426495	0.165627

TMTC4	0.548608	0.341166	0.882182	0.013242
RAP2A	0.919015	0.669745	1.26106	0.600877
SLC10A2	1.114698	0.960999	1.292978	0.151449
ABCC4	1.001902	0.82231	1.220715	0.984962
EFNB2	0.542671	0.286004	1.029677	0.061418
TM9SF2	1.15928	0.66354	2.025394	0.60364
IRF1	1.130546	0.847553	1.508029	0.403868
UPF3B	1.079709	0.663342	1.757424	0.757662
RNF113A	1.964805	1.204618	3.204715	0.006815
NDUFA1	1.753898	1.126158	2.73155	0.012934
BMP4	0.532344	0.347752	0.81492	0.003707
PTGER2	1.09206	0.84827	1.405914	0.494446
FAM193A	0.819852	0.453055	1.483613	0.511576
GRK4	0.974498	0.671681	1.413837	0.89178
HS3ST3B1	0.672231	0.551204	0.819831	8.80E-05
SLC25A35	1.174465	0.836481	1.649012	0.353014
MRPS7	2.025687	1.057647	3.879752	0.033254
GGA3	0.910486	0.596764	1.389134	0.663512
ARMC7	1.408973	0.860406	2.307288	0.173044
NUP85	0.864702	0.549199	1.361454	0.530211
SLC25A19	1.111803	0.683073	1.809626	0.669804
MIF4GD	1.05678	0.730208	1.529406	0.769659
NT5C	0.991962	0.702733	1.400233	0.963401
MSTO1	0.955044	0.56221	1.622364	0.8649
TTF1	1.032842	0.634779	1.680525	0.896483
GTF3C4	0.977821	0.611598	1.563338	0.925364
DDX31	1.001489	0.677709	1.479959	0.99404
PPP1R12C	1.539171	1.008231	2.349707	0.045723
MBOAT7	1.096278	0.786927	1.527239	0.58685
SRMS	1.513603	0.830232	2.759462	0.176132
OPRL1	1.343164	0.795793	2.267033	0.269294
SLC2A4RG	1.053872	0.828941	1.339837	0.668383
PPDPF	1.514974	1.160698	1.977384	0.00224
IL1B	0.987408	0.830235	1.174336	0.886088
CHCHD5	1.336505	0.84893	2.104113	0.210329
PAX8	1.100628	0.895587	1.352611	0.362004
INSIG2	0.861374	0.577023	1.28585	0.46538
POLR1B	0.991364	0.656037	1.498088	0.967155
CCDC93	0.930034	0.601746	1.437423	0.744029
PSD4	1.657201	1.085879	2.529116	0.019183
SLC25A23	1.032057	0.855632	1.24486	0.74148
PSPN	0.66519	0.385453	1.147941	0.14309
GTF2F1	1.170568	0.718019	1.908348	0.527676
ALKBH7	0.98321	0.725431	1.332589	0.913084
CLPP	1.622044	1.001774	2.626368	0.049163
TNFSF9	1.161098	0.970321	1.389383	0.102893
THOC2	0.823485	0.499521	1.357557	0.446391
MED1	0.862669	0.586583	1.268698	0.452867
RPL23	1.076011	0.699914	1.654205	0.738468
ATG4C	0.990691	0.650601	1.508557	0.965229
CD70	1.059304	0.918448	1.221763	0.428714
C3	1.179865	0.899818	1.547071	0.231543
SH2D3A	1.42709	1.024548	1.987789	0.035431
TRIP10	1.153178	0.912968	1.456589	0.23173
GPR108	1.483617	0.966139	2.278263	0.071458
TNFSF14	1.246137	1.003324	1.547712	0.046594
FOSB	0.866355	0.737246	1.018075	0.08144
OPA3	1.702545	0.907994	3.19238	0.097107

SNRPD2	1.459863	0.812561	2.622818	0.205648
RTN2	1.424409	1.120827	1.810217	0.003819
EML2	1.404558	0.898	2.196863	0.136604
VASP	1.555905	1.111932	2.177147	0.00991
SYMPK	1.208219	0.720203	2.026921	0.473651
SDCBP2	1.15733	0.635061	2.109108	0.633231
PANK2	1.529208	0.826649	2.828864	0.175936
GNRH2	0.875972	0.675975	1.13514	0.316632
CD93	1.122973	0.996407	1.265616	0.057307
GZF1	0.822082	0.515266	1.311592	0.411097
NAPB	0.873934	0.545112	1.401108	0.5758
CENPB	2.008647	1.295861	3.113501	0.001815
PSMF1	2.637605	1.395432	4.985521	0.002829
DTD1	2.059578	1.305609	3.248954	0.001893
RBCK1	1.572934	1.015601	2.436117	0.042426
TMX4	0.610639	0.428568	0.870063	0.006324
STK35	1.295558	0.775388	2.164685	0.322825
SNRPB	1.442822	0.983764	2.116091	0.060629
NRSN2	1.062726	0.827491	1.364832	0.633658
RRBP1	1.328913	0.908067	1.944801	0.143302
BMP2	1.610914	1.178413	2.202153	0.002797
ZNF133	1.394853	0.923405	2.106999	0.113806
MKKS	1.171456	0.705435	1.945339	0.54085
BFSP1	2.367292	1.36543	4.104254	0.002145
DSTN	1.194576	0.808296	1.765457	0.372348
SNRPB2	1.6849	0.996436	2.849042	0.051578
TBC1D20	0.86853	0.528881	1.426303	0.57757
ITPA	2.337501	1.272946	4.292336	0.006177
MCM8	1.167355	0.772765	1.76343	0.462217
FAM110A	1.461167	1.094333	1.950968	0.010137
SIRPD	1.109322	0.973516	1.264072	0.119442
MRPS26	1.321469	0.884969	1.973269	0.17301
S1PR4	0.973429	0.695555	1.362314	0.875217
NCLN	1.011558	0.735642	1.390961	0.943624
HNRNPR	1.007892	0.586524	1.731977	0.977297
ZNF436	0.948599	0.644853	1.395419	0.788725
MAX	0.537093	0.330934	0.87168	0.011876
ARMCX5	0.90706	0.575485	1.429677	0.674341
MMP24	0.848482	0.576972	1.247758	0.4037
NECAB3	0.850731	0.61315	1.180368	0.333287
ID1	1.219174	1.028076	1.445794	0.022709
RALY	1.146127	0.700292	1.8758	0.587397
DYNLRB1	1.500334	0.816946	2.755388	0.190851
EIF2S2	1.215679	0.707799	2.087989	0.479139
ERGIC3	1.078298	0.674214	1.724566	0.753039
ROMO1	1.327008	0.937959	1.87743	0.110006
CEP250	1.101259	0.682636	1.776601	0.692629
PLAGL2	1.070391	0.679059	1.68724	0.769542
AMOT	1.076651	0.872249	1.328952	0.491737
TMEM115	1.821926	1.147953	2.891592	0.010914
PSMB2	2.485682	1.272495	4.855514	0.007691
UROD	1.453785	1.085805	1.946474	0.011977
ST3GAL3	0.619465	0.432334	0.887595	0.009061
TMEM53	1.00951	0.694333	1.467755	0.960468
HECTD3	1.841928	1.188109	2.855544	0.006325
KLC1	0.935265	0.587317	1.48935	0.777999
XRCC3	1.200304	0.835829	1.723714	0.322778
TUBGCP3	0.781658	0.484357	1.261445	0.313064

MCF2L	0.733339	0.526274	1.021875	0.066931
PCID2	1.023918	0.629119	1.66647	0.924225
PROZ	1.091249	0.772974	1.540574	0.619663
LRFN3	0.904126	0.706679	1.156739	0.422712
CAPNS1	1.733045	1.178812	2.547858	0.005164
PDCD2L	1.418302	0.888089	2.265066	0.143447
RBM42	1.712134	1.111983	2.636194	0.014607
KIRREL2	1.0701	0.717588	1.595782	0.739661
UBA2	1.064971	0.672211	1.687211	0.7886
FFAR2	1.252839	1.066263	1.472062	0.006148
HCST	1.258413	1.016984	1.557156	0.034436
FFAR1	1.305108	0.737506	2.309552	0.360505
COX6B1	1.894461	1.180228	3.040923	0.008138
THRA	0.682327	0.445501	1.045049	0.078853
CCR7	1.032478	0.843988	1.263065	0.755978
NR1D1	1.309345	0.921813	1.859796	0.132251
FRMD8	1.525445	1.060351	2.194539	0.02286
PRDX5	2.040855	1.383018	3.011594	0.000326
BCL2L12	1.195531	0.767065	1.863329	0.430253
IRF3	1.435845	0.897502	2.297098	0.131323
PRMT1	1.127871	0.778683	1.633646	0.524393
RRAS	1.279221	1.056149	1.549409	0.011778
PRRG2	1.783074	1.046924	3.036852	0.033275
SCAF1	1.394454	0.922429	2.108022	0.114795
PRR12	1.075353	0.710654	1.62721	0.73103
TSKS	1.021225	0.842558	1.237779	0.830509
FLRT1	0.962346	0.673302	1.375474	0.833191
ASL	1.080677	0.783873	1.48986	0.63579
SBDS	1.18396	0.811316	1.727761	0.381212
CSN1S1	1.057537	0.867197	1.289655	0.580562
STAT5A	0.971934	0.704289	1.341289	0.862475
WNK4	0.729993	0.365242	1.459006	0.37305
BECN1	1.204219	0.71189	2.037031	0.488383
PRKCG	1.255483	0.76067	2.07217	0.373493
TRAP1	1.602601	1.071925	2.395999	0.021536
GLIS2	0.536788	0.319798	0.901009	0.01855
DNAJC8	3.808824	1.910772	7.592293	0.000145
AHDC1	1.015898	0.800801	1.288771	0.896613
IFI6	1.203912	1.029697	1.407604	0.01997
ZNF384	1.213493	0.72897	2.020061	0.456761
EMG1	1.78126	0.77556	4.091094	0.173564
UXT	1.119402	0.663029	1.889904	0.672942
CFP	1.111587	0.913239	1.353015	0.291459
ELK1	1.282326	0.812114	2.024789	0.285972
TIMM17B	1.63351	1.04521	2.552936	0.031236
KTN1	0.821779	0.53494	1.262422	0.3702
RHOJ	0.881754	0.439277	1.769931	0.723355
DLGAP5	0.913748	0.723494	1.154033	0.448902
HSPA2	1.521071	1.048167	2.207337	0.027274
ZBTB1	0.957876	0.601341	1.525803	0.856226
TRMT5	0.910252	0.620043	1.336293	0.6312
SGPP1	0.955493	0.819968	1.113417	0.559644
PLEKHG3	0.892441	0.753658	1.05678	0.186981
PZP	1.265075	1.014944	1.576851	0.036444
RHOT1	0.703962	0.450056	1.101113	0.12406
EVI2A	1.001516	0.777747	1.289668	0.990629
OMG	0.667145	0.481356	0.924642	0.015081
AIF1L	0.937646	0.832942	1.055513	0.286564

FAM78A	1.556534	1.034949	2.340984	0.033591
NUP214	1.141024	0.600562	2.167864	0.687041
CTAG2	1.249158	0.862292	1.809592	0.239411
AVPR2	1.328807	1.051685	1.678951	0.017207
SLC10A3	1.570104	1.114236	2.212481	0.009934
MAP2K2	1.452808	0.980573	2.152468	0.062582
HNRNPH2	1.393232	0.84355	2.301103	0.195188
ARMCX1	0.94044	0.784245	1.127744	0.507552
TIMM8A	0.929625	0.526646	1.640957	0.801278
ZC4H2	0.936593	0.688698	1.273718	0.676239
CANX	0.64119	0.440595	0.933111	0.020254
IPPK	0.920523	0.51828	1.634951	0.777513
ZNF484	0.834282	0.556775	1.250103	0.379887
OMD	0.802982	0.612409	1.052858	0.112435
FGD3	1.283203	0.906379	1.816689	0.159784
HIVEP3	1.44894	1.131903	1.854776	0.003246
PPCS	2.156832	1.079853	4.307922	0.029435
BCL11B	0.908315	0.733444	1.124879	0.378102
COX7C	1.053936	0.632782	1.755392	0.840058
TRAF2	1.119212	0.729574	1.716942	0.605962
ABHD8	1.468194	1.038174	2.076333	0.029869
ATP13A4	0.897761	0.595134	1.354276	0.607134
HELB	0.767185	0.555561	1.05942	0.107521
RAP1B	0.80919	0.498558	1.313365	0.391548
BEST3	0.758394	0.534843	1.075383	0.120643
RAB3IP	1.009136	0.767862	1.326223	0.947986
PTPRB	1.001802	0.767798	1.307123	0.989418
DYRK2	0.863263	0.573365	1.299736	0.481266
YEATS4	1.134491	0.719401	1.789084	0.587178
TAS2R3	0.737674	0.581606	0.935621	0.01212
TAS2R4	0.707724	0.536899	0.9329	0.014176
TAS2R5	0.72951	0.558171	0.953444	0.020943
LRRC61	1.267521	1.042265	1.541458	0.017567
IDUA	0.982383	0.759863	1.270068	0.892114
FGFRL1	0.942472	0.801218	1.108629	0.4745
TMEM175	1.231801	0.891744	1.701536	0.205932
PIN1	1.347631	0.832392	2.181798	0.224869
FBXL12	1.620345	0.94977	2.764373	0.076582
UBR4	1.039514	0.728123	1.484076	0.831067
HP1BP3	0.852606	0.469939	1.546875	0.599826
SIN3B	1.230725	0.828984	1.827158	0.303149
SLC35E1	0.942824	0.537219	1.654666	0.837452
EPS15L1	0.949498	0.582394	1.548003	0.835388
KLF2	1.176509	0.985176	1.405002	0.072648
F2RL3	1.306785	1.019171	1.675564	0.034885
GFER	1.273175	0.834333	1.942839	0.262708
SYNGR3	1.224255	0.634617	2.361739	0.54615
PKMYT1	1.145071	0.869147	1.508592	0.335544
WFIKKN1	0.712148	0.478858	1.059091	0.093651
WDR24	1.126317	0.803266	1.57929	0.490364
FBXL16	0.893777	0.602303	1.326305	0.577083
CHTF18	1.133322	0.850659	1.50991	0.392555
GNG13	1.574158	0.852512	2.906672	0.147055
MACF1	0.861765	0.640603	1.15928	0.325509
SMARCA4	1.097238	0.688719	1.748073	0.696143
TICAM1	1.668499	1.208024	2.304497	0.00189
IL17B	1.224781	0.740773	2.025031	0.429323
TUBA4A	1.219527	1.032537	1.44038	0.019439

VIL1	1.026081	0.373808	2.816535	0.960143
AAMP	1.71985	0.963841	3.068851	0.06646
PNKD	1.112906	0.785086	1.577611	0.547924
TNFRSF19	0.855087	0.658759	1.109926	0.239469
RNF6	1.07099	0.67306	1.704187	0.772284
ECHS1	2.096824	1.341993	3.276225	0.001147
AKAP9	0.5847	0.441157	0.774948	0.000189
GNG11	1.168524	1.013718	1.346971	0.031725
HIP1	1.380969	1.131734	1.685093	0.00148
PTPN12	0.988552	0.703615	1.388879	0.947082
POR	1.356929	0.998315	1.844365	0.051272
FGL2	1.10052	0.987512	1.22646	0.08316
STYXL1	0.970125	0.599034	1.571102	0.901866
STEAP4	1.087805	0.843059	1.403602	0.517512
GNAI1	1.060535	0.888485	1.265902	0.51519
PEX1	0.485701	0.322157	0.732268	0.000566
CASD1	0.613795	0.431026	0.874063	0.006804
ZNF780B	0.769783	0.569156	1.041131	0.089446
LRFN1	0.937285	0.680087	1.29175	0.69229
ZFP36	1.161752	0.938483	1.438139	0.168546
SRD5A3	0.72502	0.483984	1.086099	0.118896
SPINK2	1.181733	1.067307	1.308426	0.001311
PAICS	0.992775	0.723732	1.361834	0.964137
KDR	0.844586	0.58394	1.221573	0.369692
PPAT	1.023073	0.722633	1.448423	0.897677
TUBGCP6	1.069041	0.781298	1.462756	0.676456
ADM2	0.835771	0.650331	1.074089	0.161041
DGCR6L	1.313683	0.943886	1.828358	0.105753
DGCR8	0.985505	0.633594	1.532875	0.948347
ASPHD2	1.251701	0.923201	1.697089	0.148325
VPREB3	1.135423	0.946123	1.362598	0.172311
SDF2L1	1.019513	0.798306	1.302016	0.876931
YWHAH	1.815401	1.153845	2.856261	0.009914
RFPL2	0.945197	0.551544	1.61981	0.837514
GNAZ	1.362694	1.069628	1.736058	0.012253
MGAT3	0.908936	0.757529	1.090605	0.304406
ADORA2A	0.801111	0.480721	1.335031	0.394749
ATF4	0.956716	0.633021	1.445934	0.833678
A4GALT	0.991337	0.829989	1.18405	0.923522
CDC42EP1	1.236429	1.055986	1.447706	0.00837
APOL3	1.245527	1.025377	1.512945	0.026933
TPST2	1.389544	0.978502	1.973253	0.065988
BAIAP2L2	1.432915	1.063036	1.931492	0.018214
MPST	0.973483	0.654073	1.448874	0.894622
GALR3	0.831185	0.527191	1.310472	0.426042
TST	1.150529	0.873104	1.516104	0.31923
IGLL1	0.931472	0.841108	1.031545	0.172739
APOL2	1.285355	0.901405	1.832848	0.16556
RAC2	2.203807	1.51852	3.198355	3.21E-05
LIF	1.156233	0.898812	1.487381	0.258587
APOBEC3A	1.073602	0.958076	1.203058	0.221463
APOBEC3F	1.30344	0.90274	1.881999	0.15736
RIBC2	1.167991	0.901653	1.513004	0.239607
KRT17	0.868201	0.737692	1.021798	0.089035
DOCK4	0.742712	0.550305	1.002393	0.051855
POT1	0.677428	0.419773	1.093231	0.11073
ATP6V1F	1.452887	1.017149	2.075292	0.040031
LSM8	0.728281	0.450807	1.176541	0.195104

PRKRIP1	0.886828	0.548459	1.433954	0.624229
VGF	1.067567	0.603079	1.889799	0.822455
PODXL	0.913312	0.659376	1.265042	0.585386
MKLN1	0.713922	0.501826	1.015658	0.060988
DNAJB9	0.648492	0.449167	0.93627	0.020811
FLNC	0.682487	0.495352	0.94032	0.019475
LRRC4	0.739815	0.550435	0.994354	0.045771
CALU	0.71372	0.49412	1.030917	0.072229
CCDC136	1.07615	0.90891	1.274162	0.394413
SMO	0.822564	0.683122	0.99047	0.039302
IRF5	1.358798	1.032397	1.788395	0.028711
LRRC17	0.868967	0.601614	1.255129	0.454056
KLHDC10	0.683613	0.448637	1.04166	0.076723
NDUFA5	0.644093	0.389465	1.065194	0.086546
OPN1SW	0.719148	0.431972	1.197239	0.204889
MRPS12	1.959275	1.262601	3.040358	0.002699
MYO1B	1.06721	0.864351	1.317679	0.545358
MTX2	0.90628	0.562529	1.46009	0.685896
GAD1	0.973648	0.735718	1.288526	0.851821
OSGEPL1	0.783436	0.509832	1.203871	0.265502
ORMDL1	1.040233	0.62734	1.72488	0.878496
HAT1	1.413258	0.844714	2.364467	0.187743
HERC2	0.909238	0.652473	1.267047	0.574128
SNRPN	0.997004	0.795472	1.249594	0.979225
PSMG2	0.898494	0.439126	1.838407	0.769505
TWSG1	0.980268	0.714472	1.344945	0.901713
ARHGAP2:	1.199904	1.018588	1.413494	0.029234
WDFY4	1.328191	0.995109	1.772762	0.05402
EIF2AK4	1.487539	0.916052	2.415555	0.108388
MYO5C	0.932512	0.809866	1.073733	0.331465
CGNL1	1.331196	0.941086	1.88302	0.105922
TMOD2	0.975828	0.780016	1.220796	0.830451
TTBK2	0.789177	0.509036	1.22349	0.289903
ELL3	0.604231	0.303209	1.204103	0.152137
INO80	1.043446	0.587731	1.852513	0.884542
DLL4	0.675937	0.281282	1.624318	0.381276
ALDH1A2	1.398358	0.754834	2.590511	0.286479
IVD	1.129979	0.729342	1.750693	0.584342
DUT	1.229247	0.80763	1.870967	0.335513
CHAC1	1.317869	1.000854	1.735295	0.049293
CLN6	0.798256	0.486855	1.308836	0.371777
ARPP19	1.134287	0.662109	1.943196	0.646406
VPS13C	0.691057	0.506563	0.942745	0.019698
LOXL1	1.036837	0.844029	1.27369	0.730384
ANAPC13	1.347515	0.792414	2.291474	0.270875
MBD4	1.051616	0.616873	1.792744	0.853291
COPB1	0.915754	0.565753	1.48228	0.720214
PSMA1	0.87379	0.47392	1.611048	0.665585
SUMF2	0.937577	0.582151	1.510005	0.790943
PALLD	1.149839	0.869367	1.520797	0.327731
SPCS3	0.677029	0.404999	1.131775	0.136807
MYOD1	0.175737	0.001177	26.22842	0.495985
SERGEF	1.097814	0.658245	1.830921	0.720653
TPH1	1.119619	0.721524	1.737361	0.61425
E2F8	1.057063	0.806826	1.384912	0.687221
DCTD	0.930583	0.522046	1.658828	0.807282
SOX15	0.91408	0.658168	1.269496	0.591907
RPAIN	0.648485	0.408851	1.028572	0.065729

USP6	0.777761	0.598252	1.011133	0.060481
SHBG	0.657617	0.351426	1.230586	0.189867
PLD2	1.180981	0.989065	1.410138	0.065997
CD68	1.364245	0.888399	2.094965	0.155826
TXNDC17	1.250839	0.783904	1.995906	0.347853
ATP1B2	0.953723	0.801459	1.134914	0.593404
FXR2	1.042937	0.631148	1.723397	0.869686
KIF1C	1.155089	0.903226	1.477184	0.250598
MPDU1	1.772236	1.147057	2.738155	0.009935
PHF20L1	0.85131	0.489126	1.481682	0.569114
LRRC6	1.003291	0.682404	1.475071	0.986668
CCNT1	0.868454	0.589372	1.279688	0.47579
PUS7L	0.766129	0.514177	1.141539	0.190418
KRI1	1.663865	1.087762	2.545084	0.01888
ILF3	0.882653	0.571165	1.364014	0.574061
SLC44A2	1.126249	0.904564	1.402263	0.287739
CDKN2D	1.28051	1.001657	1.636991	0.048475
MTUS1	1.769724	0.876135	3.574703	0.111537
SIGLEC9	1.100103	0.985151	1.228469	0.090212
NGDN	0.956394	0.564886	1.619248	0.868189
RIPK3	1.416064	1.055978	1.898938	0.020136
ADCY4	1.185363	0.968998	1.45004	0.098188
RAB2B	0.756574	0.391594	1.461728	0.406431
BCL2L2	1.063456	0.598302	1.890249	0.833943
PARP2	1.474066	0.860509	2.525099	0.157679
HEATR5A	0.559881	0.379532	0.825931	0.003455
SNX6	1.011058	0.676967	1.510027	0.957147
EAPP	1.340749	0.72844	2.467751	0.346174
NRL	1.262773	0.562203	2.836333	0.572007
RNASE1	1.098123	0.952086	1.266562	0.198586
NEDD8	2.31643	1.204353	4.45538	0.011831
DAD1	1.015649	0.656732	1.570721	0.944352
TEP1	1.225885	0.850009	1.767972	0.275658
EPB41L4A	1.307857	0.660408	2.590049	0.441384
REEP5	0.687829	0.530567	0.891703	0.004723
ITFG1	0.646572	0.377819	1.106495	0.111656
QRICH2	0.967077	0.675143	1.385243	0.85512
FOXJ1	1.154456	0.643662	2.070604	0.629905
SEC14L1	1.56673	1.047803	2.342657	0.028709
RHBDF2	1.510777	1.214145	1.87988	0.000216
AANAT	1.321353	1.020391	1.711083	0.034598
ARHGEF6	0.752496	0.542914	1.042983	0.087768
MAP7D3	1.250326	0.843447	1.853484	0.266017
ASH2L	1.30641	0.742302	2.29921	0.354065
CHRNA10	0.797686	0.535366	1.188539	0.266566
CDKN1C	0.917508	0.771436	1.091239	0.330508
RPS4Y1	0.994885	0.940065	1.052903	0.859256
KLF16	1.226492	0.866855	1.735332	0.248906
DOHH	1.032322	0.754378	1.412671	0.842447
SHC2	0.78578	0.490671	1.258378	0.315666
LBP	0.967694	0.815418	1.148407	0.706972
CBFA2T3	0.88581	0.682353	1.149932	0.362457
GAMT	1.14826	0.884962	1.489897	0.29819
PHF10	0.722937	0.432043	1.209691	0.216757
PRRG3	0.229551	0.023525	2.239853	0.205459
STARD8	1.310653	1.07112	1.603751	0.008609
GDPD2	2.398509	1.307929	4.398438	0.00469
SAT1	1.276681	1.064601	1.531009	0.008405

GNL3L	1.253379	0.702409	2.236533	0.444635
SH3BP4	1.035473	0.904056	1.185994	0.61469
MOSPD2	0.985859	0.707587	1.373566	0.932924
DOCK6	1.085604	0.880956	1.33779	0.440892
ECSIT	1.555066	0.973107	2.485061	0.064894
LDLR	1.322273	1.096421	1.594649	0.003464
ELOF1	2.017057	1.201363	3.386586	0.007958
TSPAN16	1.093109	0.8808	1.356595	0.419091
PRKCSH	1.03966	0.695167	1.554867	0.849785
CDC16	1.130816	0.711222	1.797954	0.603322
APOE	0.990314	0.850295	1.15339	0.900409
TOMM40	1.505727	1.00905	2.24688	0.045062
APOC1	1.07848	0.877792	1.32505	0.472023
GADD45G	1.336475	0.981207	1.820375	0.065823
XPO7	1.061871	0.742284	1.519055	0.74245
FAM98C	1.484189	0.994986	2.213917	0.05295
SAFB2	0.926968	0.521611	1.647336	0.796021
RPL36	1.225925	0.83314	1.80389	0.301312
ATP8B3	0.9681	0.72246	1.297259	0.828124
GTPBP3	1.199561	0.770473	1.867614	0.420496
PLVAP	1.113981	0.922757	1.344833	0.26129
BST2	1.477331	1.117313	1.953352	0.006175
SLC27A1	0.805301	0.562654	1.152592	0.236543
NSUN5	1.288089	0.847255	1.958294	0.236233
USHBP1	0.816718	0.29654	2.249368	0.695293
DDA1	1.534891	0.931131	2.530136	0.092927
MRPL34	2.286455	1.375149	3.801679	0.001433
PGLS	1.43549	0.930761	2.213921	0.101972
LSM7	1.133227	0.773704	1.659812	0.520667
TULP4	0.808437	0.638191	1.024098	0.077969
SNX9	1.00191	0.821388	1.222107	0.984978
RTN4IP1	1.240934	0.724951	2.124167	0.431214
QRSL1	1.018116	0.616565	1.681185	0.944064
RSPH3	0.980313	0.567394	1.693731	0.943184
MLLT1	0.909156	0.605889	1.364217	0.645538
FUT5	3.183425	0.291426	34.77444	0.3425
ACTN4	1.536624	1.056455	2.235034	0.024624
NDUFA10	0.893843	0.516756	1.546099	0.688118
ARPC1B	1.053868	0.67604	1.642856	0.816832
ZSWIM6	1.046374	0.779329	1.404924	0.763009
FCHO1	1.231362	0.856104	1.771109	0.261769
MAP1S	1.139496	0.811205	1.600645	0.451344
KLHDC7B	1.040385	0.823385	1.314575	0.7401
PXDN	0.909664	0.815647	1.014517	0.088938
SSBP4	1.616717	1.178682	2.21754	0.002886
GDF15	1.188533	0.960046	1.471399	0.112822
PGPEP1	1.427146	0.910081	2.237981	0.121264
LSM4	2.583936	1.662605	4.015823	2.45E-05
JUND	1.274295	0.972217	1.67023	0.079112
HRC	1.826656	1.239576	2.691784	0.002322
TRPM4	1.207777	1.004606	1.452038	0.044551
SULT4A1	1.445026	0.964675	2.164563	0.074176
ZNF557	1.067073	0.686312	1.659078	0.773116
CRB3	2.13529	0.703724	6.479049	0.1804
CAMSAP1	1.157414	0.734029	1.825007	0.529234
UBAC1	1.381767	0.985709	1.936961	0.060596
ZBTB46	1.232041	0.972632	1.560637	0.083645
SAMD10	1.141351	0.866504	1.503377	0.346918

LSP1	1.365152	1.163424	1.601858	0.000136
TNNT3	1.345178	1.114146	1.624117	0.002041
TNNI2	1.343478	1.153778	1.564369	0.000144
H19	1.379895	0.997719	1.908464	0.051635
COL5A1	1.085876	0.955724	1.233752	0.20596
ATXN10	0.956585	0.552297	1.656817	0.87416
TUBGCP2	1.757253	1.119109	2.759283	0.014334
CYP2E1	0.600013	0.444151	0.81057	0.000873
PNPLA7	1.047715	0.813187	1.349883	0.718459
HBZ	0.891194	0.50437	1.574689	0.691649
PAK4	0.813932	0.54659	1.212033	0.310868
MNX1	1.004099	0.7373	1.367442	0.979292
ZNF337	0.827059	0.551986	1.239211	0.357379
TAF4	0.72511	0.39263	1.339134	0.304438
LAMA5	0.941858	0.753442	1.177391	0.59889
OSBPL2	0.992207	0.551132	1.786278	0.979193
ADRM1	1.123428	0.853562	1.478615	0.406352
ASS1	0.832791	0.706354	0.981859	0.029416
EXOSC2	1.03573	0.613637	1.748161	0.895422
POMT1	0.88525	0.642064	1.220543	0.457004
UCK1	1.796201	1.123859	2.870769	0.014364
FIBCD1	0.563843	0.293662	1.082605	0.08516
CHMP2A	1.796489	1.159668	2.783015	0.008709
UBE2M	1.642714	1.035947	2.604871	0.03485
TRIM28	1.17514	0.801435	1.723101	0.408552
YIPF2	1.070497	0.764356	1.499254	0.691825
ATG4D	1.203612	0.889093	1.629392	0.230425
EIF2S3	0.936489	0.576252	1.521924	0.791126
TMEM160	1.160793	0.848869	1.587335	0.350406
ZC3H4	1.271275	0.683412	2.364811	0.448492
NPAS1	0.896618	0.637099	1.26185	0.531364
GMFG	1.359718	0.80879	2.285924	0.24633
MAP3K10	1.186945	0.84518	1.66691	0.322589
LRRC47	1.857997	1.061121	3.253309	0.030194
SESN2	1.429712	1.067394	1.915015	0.016514
SMPDL3B	1.27685	1.030327	1.582356	0.025553
MED18	1.619441	0.985384	2.661489	0.057188
CLIP1	0.908301	0.571869	1.442657	0.683686
CCDC62	0.95905	0.689909	1.333187	0.803519
HIP1R	0.813735	0.652871	1.014236	0.066627
ZNF317	1.369118	0.72523	2.584673	0.332528
PPAN	1.052014	0.742591	1.490367	0.775397
EIF3G	1.729731	1.069258	2.798173	0.02556
ANGPTL6	1.014845	0.798941	1.289094	0.903897
DNMT1	1.320759	0.844426	2.065785	0.222835
ZNF426	0.937213	0.614464	1.429487	0.763372
SLC6A8	0.908186	0.733592	1.124332	0.376628
DKC1	1.082169	0.725703	1.61373	0.698507
PLXNA3	0.941857	0.757826	1.170579	0.589168
MPP1	1.43394	1.011572	2.03266	0.042909
ZNF331	1.146935	0.805164	1.633778	0.447579
ZNF236	0.674991	0.41586	1.09559	0.111713
LRP3	0.966636	0.833843	1.120575	0.652664
NOL11	0.900944	0.586811	1.38324	0.633464
UBE4B	0.973958	0.613733	1.545614	0.910832
CASZ1	1.122277	0.76707	1.641971	0.552403
PKDREJ	0.924293	0.661559	1.291371	0.644525
HABP4	1.024296	0.720936	1.455307	0.893429

SLC35D2	1.142367	0.787099	1.657988	0.483721
PRRG1	0.871207	0.663024	1.144757	0.322365
UBA1	1.891203	1.189712	3.006314	0.007049
PPIL4	0.942935	0.52272	1.700961	0.84523
ULBP2	1.252362	0.805999	1.945923	0.316927
AKAP12	0.665416	0.474765	0.932628	0.018035
SYNE1	1.173931	0.875008	1.574973	0.284858
ULBP3	1.642656	1.248275	2.161638	0.000395
LATS1	0.836202	0.521138	1.341744	0.458409
EPS8L1	1.391349	1.011059	1.914678	0.042613
LILRB2	1.170131	1.058689	1.293304	0.002092
TTLL9	1.790752	0.9252	3.466052	0.08377
RBM39	0.627939	0.376405	1.047562	0.074747
ZNF341	1.131112	0.695251	1.84022	0.619785
GGT7	1.212075	0.982505	1.495286	0.072618
ACSS2	1.214717	0.895983	1.646837	0.210336
EDA2R	0.914942	0.770052	1.087094	0.312209
ARHGEF9	1.045199	0.67551	1.617207	0.842654
HIGD1B	0.633138	0.439426	0.912244	0.014172
ATP6V1E1	2.007583	1.14412	3.522699	0.01513
ZNF227	0.62952	0.410583	0.965201	0.033805
ZNF428	1.053896	0.665313	1.669435	0.823015
ZNF141	0.703052	0.508294	0.972433	0.033265
CCL25	1.367375	1.06843	1.749965	0.012925
COX4I1	1.49953	0.954991	2.354567	0.078422
GINS2	1.230508	0.83842	1.805957	0.289303
CHMP1A	1.564193	1.002549	2.44048	0.048703
SH3BGRL	1.479005	0.950127	2.302276	0.083029
COX7B	1.715793	0.997442	2.951497	0.051092
F12	1.661622	1.325769	2.082555	1.04E-05
PRR7	1.452125	1.176093	1.792942	0.000525
NFATC1	1.400247	0.992535	1.975438	0.055201
CAP1	1.979381	1.377672	2.843891	0.000222
PPT1	1.312692	0.943519	1.826314	0.106335
RAB11FIP4	1.286281	0.983911	1.681572	0.065573
ABCB7	0.838293	0.501141	1.40227	0.501604
TRAF3	1.460251	0.994599	2.143913	0.05332
HAUS8	2.044524	1.145263	3.649886	0.015577
MRPS25	1.072518	0.688088	1.671726	0.757209
SH3BP5	1.306612	1.030565	1.6566	0.027202
HACL1	0.706396	0.442355	1.128044	0.145546
TBC1D5	0.923894	0.587413	1.45312	0.731912
CAPN7	1.103768	0.667588	1.824935	0.70035
RFTN1	1.672546	1.12728	2.481557	0.010615
SLC6A6	1.459154	1.066691	1.996015	0.018086
KCNC3	1.295416	1.081288	1.551947	0.004989
NAPSA	0.968836	0.698772	1.343275	0.849391
NAPSB	1.20706	1.075408	1.35483	0.001404
NR1H2	1.663367	1.061559	2.606344	0.026373
LRRC4B	0.863306	0.586397	1.270975	0.456359
PDLIM4	0.617243	0.36683	1.038596	0.069167
KIF3A	0.882446	0.595475	1.307714	0.533186
MGAT1	1.505941	1.018846	2.225908	0.040013
TUBG1	1.310384	0.926332	1.853661	0.126625
PSME3	1.906326	1.069035	3.399401	0.028804
RPL27	1.137355	0.717964	1.801728	0.583459
PSMC3IP	1.10567	0.676758	1.806415	0.688371
AOC3	1.110219	0.759194	1.623548	0.589743

ACLY	1.305336	0.79565	2.141522	0.291455
VPS25	2.271835	1.301085	3.96687	0.003908
RAMP2	0.934594	0.684402	1.276245	0.670455
AOC2	1.182478	0.810082	1.726065	0.385093
NDUFA2	1.804982	1.062078	3.067534	0.029069
ANKHD1	0.462419	0.277267	0.771213	0.003122
DIAPH1	0.930251	0.678077	1.276207	0.654034
NDFIP1	1.452254	1.177724	1.790777	0.000483
UBE2D2	1.27197	0.703178	2.300851	0.426323
EXOC4	0.973255	0.594157	1.594234	0.914261
ACAP3	1.158701	0.863206	1.55535	0.326769
PPFIA1	0.931038	0.580645	1.492876	0.76676
TMEM204	1.154957	0.927094	1.438825	0.198852
KREMEN2	0.68967	0.461083	1.031581	0.070512
THOC6	1.308695	0.87717	1.952509	0.187523
TRAF7	1.409252	0.937667	2.118014	0.098872
BARX1	0.694693	0.1573	3.068016	0.630733
NINJ1	1.250666	1.047343	1.493461	0.013475
CA6	1.043778	0.684371	1.591932	0.842301
NPHP4	0.977596	0.696172	1.372786	0.895927
MAP1B	1.052539	0.592502	1.869761	0.861348
IL13RA1	1.116522	0.965173	1.291603	0.138072
WDR44	1.276911	0.716208	2.276576	0.407349
CKMT2	0.731133	0.543446	0.983641	0.038552
ZCCHC9	1.007262	0.585334	1.733331	0.979157
TOP2A	0.887167	0.720185	1.092867	0.260471
STARD3	1.103819	0.649367	1.876313	0.715177
RARA	1.26571	0.963327	1.66301	0.090699
PPP1R1B	0.835601	0.179169	3.897046	0.819172
KHDRBS3	0.920172	0.679099	1.246824	0.591446
CHD1L	0.791013	0.504586	1.240028	0.306748
PEX11B	2.224803	1.045765	4.733137	0.037881
FMO5	1.308199	0.952942	1.795897	0.096552
PIAS3	0.972183	0.666123	1.418867	0.883725
PRKAB2	1.123893	0.76933	1.641866	0.54587
PDHA1	1.370105	0.774012	2.425269	0.279809
RAI2	1.045121	0.193015	5.659023	0.959159
MCCC2	1.162659	0.71389	1.893535	0.544763
ZNF304	0.829198	0.578391	1.188762	0.308148
ZSCAN5A	0.667031	0.411856	1.080307	0.09977
ZNF132	0.943508	0.678214	1.312574	0.729924
CHSY1	0.684882	0.45866	1.022684	0.064269
SNRPA1	1.070053	0.654971	1.748188	0.786895
LLGL1	1.095195	0.793408	1.511772	0.58034
THAP1	1.271627	0.759304	2.129627	0.361053
ACTR10	1.851535	0.865041	3.96303	0.112611
ABHD12B	0.791357	0.614095	1.019786	0.070523
GCH1	1.108884	0.919066	1.337906	0.280621
LGALS3	1.094478	0.955488	1.253686	0.192627
PODNL1	1.77662	1.114193	2.832882	0.01577
DNAJB1	1.534849	0.895977	2.629266	0.118757
ZSWIM4	0.747282	0.527114	1.059412	0.101867
FBXW9	1.106675	0.801335	1.528361	0.538314
RFX1	1.187525	0.787601	1.79052	0.412021
ZNF20	0.533744	0.347574	0.819631	0.00412
DCAF15	1.288575	0.865153	1.919229	0.212272
CC2D1A	1.744126	1.138064	2.67294	0.010659
TRIM21	1.594988	1.078589	2.358623	0.019336

SPATA6	1.002949	0.811699	1.239259	0.97824
LRRC41	1.184783	0.690527	2.032811	0.538166
CCT6B	1.023055	0.647476	1.616496	0.922205
DHX30	1.153334	0.707971	1.878861	0.566682
RAF1	0.783149	0.523908	1.17067	0.233375
PPARG	0.946633	0.819434	1.093577	0.456314
NUP210	1.676635	1.268864	2.21545	0.000278
FCRLA	1.128354	0.913297	1.394052	0.263003
HSD17B7	1.365131	0.770768	2.417824	0.285875
ENOSF1	0.797966	0.607691	1.047819	0.104404
EMILIN2	0.937131	0.788114	1.114326	0.462425
ARFIP2	1.342881	0.780164	2.311475	0.287323
TRIM5	0.753193	0.490741	1.156006	0.194728
CNGA4	1.519771	0.983992	2.347278	0.059133
TRIM22	1.291762	0.883963	1.88769	0.185934
RRP8	0.819632	0.481099	1.39638	0.464353
EFR3A	1.346923	0.955573	1.898547	0.089038
PTCD3	0.732581	0.473658	1.133041	0.161937
IMMT	1.390406	0.766969	2.52061	0.277532
MRPL35	1.190408	0.731825	1.936354	0.482572
ILKAP	0.737218	0.39688	1.369406	0.334575
PER2	0.961373	0.618792	1.493616	0.860892
RAMP1	0.943941	0.839263	1.061674	0.336043
SCLY	0.601895	0.378429	0.957319	0.032018
PTPRE	1.11837	0.883338	1.415939	0.352678
RAN	1.313326	0.827063	2.085483	0.248004
PRKAA1	0.791786	0.493856	1.269449	0.332368
CARD6	1.640661	1.091382	2.466385	0.017294
INPP5K	1.451317	0.905792	2.325392	0.121481
MYBBP1A	1.255085	0.889322	1.771281	0.196139
RPA1	1.383456	0.896554	2.134787	0.142491
SERPINF1	1.072448	0.87747	1.310751	0.494484
UBE2G1	0.673196	0.370728	1.222439	0.193568
EEFSEC	1.479779	0.946561	2.313371	0.085604
TBC1D14	1.16019	0.800594	1.681303	0.432461
TMEM128	0.816323	0.434687	1.53302	0.527918
COQ3	1.199213	0.798995	1.799902	0.380569
SEC61G	0.776498	0.498637	1.209192	0.262972
LANCL2	1.615426	0.958923	2.721387	0.071493
FIGNL1	0.940626	0.690554	1.281259	0.697883
GRSF1	0.68588	0.405393	1.160433	0.15991
ANKRD17	0.842073	0.527797	1.343484	0.470807
UTP3	1.41123	0.872664	2.282172	0.160147
ITGB4	0.792965	0.607042	1.035831	0.088809
WBP2	1.426641	0.884143	2.302006	0.145518
UNK	0.673923	0.408726	1.111191	0.121927
TRIM47	1.017817	0.814945	1.271191	0.876263
ZRANB2	0.768543	0.500601	1.179899	0.228736
EIF5A	1.387656	0.922503	2.087351	0.115781
KDM6B	1.021008	0.710959	1.466271	0.910357
CLEC10A	1.033922	0.931657	1.147412	0.530148
GUCY2D	0.976852	0.758473	1.258106	0.856042
GPS2	1.097451	0.630512	1.910191	0.742263
XAF1	0.999175	0.806988	1.237132	0.993957
DLG4	1.109977	0.847221	1.454223	0.449028
VPS13B	0.680038	0.459603	1.006197	0.053723
MATN2	0.81095	0.549373	1.197074	0.291589
REEP2	0.528182	0.27143	1.027799	0.060212

PCBD2	0.456056	0.284767	0.730378	0.001085
SDF2	1.428894	0.697189	2.928529	0.329663
FLOT2	1.348032	0.949296	1.914252	0.09509
ERAL1	1.642553	0.893413	3.019859	0.110218
PRMT7	1.216817	0.766837	1.930845	0.404836
NIP7	1.084076	0.66111	1.77765	0.749023
TERF2	2.147685	1.175025	3.925491	0.012987
VPS4A	1.289144	0.69555	2.389321	0.419811
HSPA12B	0.952498	0.717537	1.264398	0.736311
BTBD3	1.231491	0.819264	1.851137	0.31667
PCNA	1.358968	0.948501	1.947067	0.094567
NXT1	1.208804	0.817386	1.787658	0.342165
POLR3F	0.586458	0.309565	1.111018	0.101626
RIN2	0.980427	0.814957	1.179493	0.833989
PTPRA	2.05797	1.03248	4.102009	0.040287
DAP3	1.160487	0.60779	2.215783	0.651957
ARHGEF11	1.039311	0.906453	1.191642	0.580586
FCRL2	1.010036	0.77041	1.324195	0.942386
SYT11	1.395017	0.991913	1.961938	0.05571
IGHMBP2	1.105297	0.702665	1.738638	0.664893
ACY3	1.123214	0.999208	1.26261	0.051571
MMACHC	1.630129	1.016414	2.614406	0.04261
DPH2	1.418818	0.9009	2.234482	0.131143
TOE1	1.543792	0.915442	2.603433	0.103394
NASP	0.970982	0.628369	1.5004	0.894488
MUTYH	1.241775	0.61701	2.499154	0.543973
CTNNBL1	1.283271	0.832385	1.978393	0.258775
LPIN3	1.203385	0.858124	1.68756	0.283224
ZSWIM3	1.316947	0.863078	2.009494	0.201608
RBM38	1.182255	0.962967	1.45148	0.109719
VSTM2L	1.202775	0.827561	1.748108	0.333135
SERINC3	0.951361	0.583877	1.550135	0.841343
PPP1R3D	1.274915	0.868571	1.871358	0.214839
AP3B1	0.864762	0.550318	1.358876	0.528622
ZBED3	0.877424	0.719066	1.070657	0.197862
ANGPTL3	0.486349	0.301152	0.785434	0.003203
FBXO44	1.198312	0.937079	1.53237	0.149309
CASP9	1.881089	1.078067	3.282259	0.026107
DCTN4	1.091466	0.722962	1.647802	0.67709
PDE6A	1.297754	0.673302	2.501351	0.436287
MTUS2	0.768585	0.60414	0.977791	0.032128
ZMYM5	0.739443	0.423141	1.292184	0.289187
USPL1	1.049898	0.612156	1.800662	0.859579
XPO4	0.659567	0.41264	1.054257	0.082006
POMP	1.790042	1.100847	2.910715	0.018909
CDK8	0.623203	0.352541	1.101662	0.103764
ALOX5AP	1.167622	1.02278	1.332975	0.021831
WASF3	1.258454	0.874383	1.811228	0.215937
GPR12	0.641464	0.485402	0.847703	0.001799
MYH10	0.906458	0.712134	1.153809	0.424995
PEMT	0.990862	0.738277	1.329862	0.951241
SCO1	0.748789	0.424824	1.319805	0.317113
MPRIP	0.869111	0.6246	1.209341	0.405252
CHI3L1	0.949095	0.829821	1.085513	0.445771
MYBPH	1.105297	0.939596	1.30022	0.227005
PIK3C2B	1.027261	0.805077	1.310761	0.828756
DSTYK	0.887008	0.578829	1.359267	0.58194
CHIT1	1.057385	0.756193	1.478542	0.744268

SLC41A1	1.127156	0.951095	1.33581	0.167179
LGR6	1.070675	0.87663	1.307674	0.503266
TMCC2	0.92214	0.798851	1.064457	0.268321
CCNA1	0.928216	0.840045	1.025641	0.143523
COG6	0.636272	0.39544	1.023774	0.062442
RXFP2	1.001051	0.891305	1.124309	0.985859
EPSTI1	1.240681	1.067579	1.44185	0.00491
RFXAP	1.144717	0.723122	1.81211	0.564135
TPT1	0.793557	0.522866	1.204385	0.277347
RFC3	1.213775	0.841709	1.750306	0.299591
STARD13	1.134258	0.85274	1.508714	0.386756
MORC4	1.105037	0.835038	1.462338	0.484709
BEX2	1.153713	1.015273	1.311029	0.028352
TCEAL4	0.927937	0.69603	1.237112	0.610229
BEX1	1.032576	0.944783	1.128528	0.479508
FAM104A	1.180081	0.726708	1.916301	0.503237
SLC39A11	0.689261	0.526317	0.90265	0.006846
EPHB2	1.131674	0.854864	1.498116	0.387426
SRRM1	1.339207	0.769345	2.331174	0.301708
BTBD2	1.197687	0.848464	1.690647	0.305056
PRAM1	1.139631	0.912989	1.422535	0.24796
ZNF414	1.424561	1.021448	1.986762	0.037069
PDE6B	1.090096	0.733773	1.619451	0.669265
HSPBP1	1.264723	0.867552	1.843721	0.222018
CSNK1G2	1.098021	0.744576	1.619242	0.637062
CNDP2	1.171925	0.846407	1.622634	0.339291
MACROD1	1.142798	0.88882	1.46935	0.297929
WDR74	1.363223	0.98581	1.885128	0.060993
LGALS12	0.941754	0.841724	1.053671	0.294893
RTN3	0.97538	0.588518	1.616545	0.922959
MYH11	0.696174	0.487576	0.994015	0.046261
MED10	1.780405	0.969293	3.270263	0.062967
PDZD2	0.916493	0.695865	1.207072	0.534865
MORC2	1.251971	0.738333	2.122933	0.404257
MYO18B	0.981772	0.834834	1.154573	0.82401
SLC2A11	1.041644	0.689765	1.573032	0.846175
C1QTNF6	1.184819	0.888653	1.579689	0.247851
FAM83F	0.704582	0.358842	1.383438	0.30909
SEC14L4	0.878949	0.705701	1.09473	0.24935
GIMAP6	1.198203	0.993702	1.44479	0.058252
GIMAP4	1.03845	0.911913	1.182546	0.569289
ADCK2	1.371566	0.914868	2.056247	0.126191
MKRN1	0.891043	0.592221	1.340644	0.579931
AGAP3	1.045506	0.821463	1.330653	0.717612
KRBA1	1.010192	0.769918	1.325451	0.941665
ACTR3B	0.759497	0.493244	1.169472	0.211621
BTG1	0.991966	0.765829	1.284878	0.951275
ATP13A3	0.62884	0.40731	0.970857	0.03631
KRAS	0.918081	0.569961	1.478826	0.725291
IPO8	0.916312	0.584629	1.43617	0.703063
IMPA1	0.895928	0.576984	1.391179	0.624509
LRRCC1	1.137568	0.873882	1.480819	0.338066
E2F5	1.310866	0.911346	1.885529	0.144442
CA1	0.949994	0.849364	1.062546	0.369193
CCDC59	0.842923	0.499914	1.421283	0.52148
SWAP70	1.382228	1.004908	1.901223	0.046584
ARNTL	1.140806	0.914572	1.423003	0.242756
LYVE1	1.21782	0.979736	1.51376	0.075814

AMPD3	0.826809	0.609828	1.120995	0.220747
SBF2	1.20332	0.943519	1.534658	0.135839
MICAL2	1.128913	0.934387	1.363937	0.208884
RRAS2	1.005279	0.825528	1.22417	0.95822
HSD17B4	0.908872	0.61087	1.352249	0.637392
ZFC3H1	0.74771	0.472805	1.182455	0.213761
RNF122	1.003918	0.674959	1.493204	0.984599
DPF2	1.234068	0.682504	2.231378	0.486461
MEN1	1.355941	0.870418	2.11229	0.178194
NUMB	1.027353	0.689407	1.530958	0.89452
COX16	1.474928	0.88697	2.452633	0.134211
TTC9	1.015821	0.834342	1.236774	0.875775
MED6	0.689541	0.335278	1.418126	0.312296
EIF2S1	1.255326	0.789579	1.995802	0.336423
ADAM20	0.593996	0.314798	1.120816	0.10786
LOXL2	1.115546	0.670532	1.855904	0.673741
ELP3	1.351022	0.768092	2.376359	0.296385
ADAMDEC	1.257576	1.063946	1.486444	0.007219
MBD2	1.35235	0.915522	1.997606	0.129387
IER3IP1	0.689874	0.437675	1.087396	0.109804
MRPS36	1.019411	0.639926	1.623936	0.935502
CCNB1	0.873472	0.673449	1.132906	0.307955
CDK7	0.803592	0.430165	1.501192	0.492838
CD180	1.249424	1.082374	1.442255	0.002359
IRAK2	0.908045	0.726429	1.135068	0.396871
CAMK1	1.076547	0.821788	1.410284	0.592401
THUMPD3	1.130219	0.629655	2.02872	0.681714
VHL	1.315245	0.706848	2.447301	0.38709
ARL8B	1.173883	0.720773	1.911836	0.519435
EDEM1	0.996356	0.654553	1.516647	0.986414
MEIS2	1.026891	0.689609	1.529134	0.896076
GNAT2	0.990232	0.711547	1.378068	0.953581
GSTM1	1.031718	0.928133	1.146862	0.562979
PRPF38B	0.998789	0.513472	1.942809	0.997151
TSPAN2	1.07722	0.927694	1.250846	0.329267
TSHB	1.022688	0.774432	1.350528	0.874351
GSTM5	1.08888	0.906289	1.308258	0.363216
GSTM3	1.066331	0.863241	1.317201	0.551329
VAV3	1.290059	0.983931	1.691431	0.065364
PSRC1	1.290152	1.015394	1.639258	0.03707
PTPN22	1.002239	0.768272	1.307458	0.986844
SORT1	1.223751	1.091471	1.372062	0.000541
WNT2B	0.958805	0.543253	1.692227	0.884607
PTGFRN	0.911174	0.759357	1.093344	0.317161
NOTCH2	1.221798	0.981465	1.520981	0.073045
TRIM45	1.198847	0.816786	1.759622	0.354286
CEPT1	0.695934	0.452827	1.069557	0.098274
AP4B1	1.258151	0.791128	2.000872	0.331969
NAPG	0.822094	0.477607	1.415054	0.479557
SPIRE1	0.931034	0.759249	1.141687	0.492291
PPHLN1	1.17756	0.609784	2.273998	0.626416
FKBP11	1.458629	0.91042	2.33694	0.116481
ARF3	2.230019	1.370876	3.627596	0.001235
TMEM106	1.444498	1.064511	1.960125	0.018208
SLC38A2	0.734997	0.542711	0.995412	0.046626
YWHAQ	1.093504	0.655052	1.825428	0.732432
KIDINS220	0.99668	0.656682	1.512713	0.987537
GRHL1	0.628647	0.244555	1.615983	0.335234

ROCK2	0.931617	0.646332	1.342824	0.704148
RSAD2	1.113699	0.911556	1.360669	0.29198
MYCN	0.925696	0.820687	1.044142	0.208816
LPIN1	1.294438	0.979298	1.71099	0.069831
CMPK2	1.077658	0.835882	1.389368	0.563952
IAH1	1.421156	0.73406	2.751386	0.297069
LDHA	1.230618	0.791412	1.913569	0.356878
IL6ST	1.086621	0.8813	1.339777	0.436897
FST	0.755515	0.563323	1.013279	0.061223
NAV1	0.922233	0.781848	1.087825	0.336626
CDC73	0.983524	0.595715	1.623797	0.948219
TIMM17A	1.281881	0.715416	2.296872	0.403982
RPS15A	0.985555	0.602233	1.612864	0.953832
RBM17	1.622642	0.848477	3.103169	0.143396
IL2RA	1.328435	1.180061	1.495464	2.60E-06
ANKRD16	1.01519	0.673139	1.531051	0.942672
ECHDC3	1.421469	1.178842	1.714034	0.000231
IL15RA	1.585023	1.213275	2.070674	0.000731
CCNH	0.565794	0.342217	0.935438	0.02641
HRH4	0.704738	0.523233	0.949205	0.021276
KCTD1	0.636908	0.478731	0.847348	0.001954
CABLES1	0.879901	0.732532	1.056918	0.171293
DOCK2	1.033946	0.70843	1.509033	0.86261
EMP1	1.056344	0.920946	1.211648	0.433493
SOX5	1.360048	0.815278	2.268836	0.238882
KLRD1	1.167905	0.91971	1.483078	0.202901
KLRC1	1.010044	0.702797	1.451612	0.956929
PRH2	0.679666	0.476709	0.96903	0.032859
LRP4	0.865832	0.689717	1.086917	0.214369
MYBPC3	0.847376	0.581548	1.234716	0.388557
DDB2	1.269902	0.852804	1.890997	0.239524
ACP2	1.449664	1.059115	1.984229	0.020418
RAB33A	1.181376	0.976501	1.429234	0.086299
RBMX2	1.992215	1.077631	3.683005	0.027918
PIWIL4	0.904124	0.756399	1.080699	0.268156
PUM1	0.817141	0.47296	1.411786	0.469155
PHC2	1.434747	1.004091	2.050111	0.047436
CDCA8	1.040573	0.766357	1.412909	0.798845
GNL2	1.240766	0.693647	2.219429	0.467168
HOOK1	1.037259	0.840954	1.279389	0.732538
BTF3L4	0.497461	0.296529	0.834547	0.008166
PRPF38A	0.912951	0.529615	1.573746	0.743059
DSC2	0.904715	0.785652	1.041823	0.164266
RNF138	0.890651	0.593808	1.335887	0.575571
ELP2	0.703953	0.458613	1.080541	0.108349
DSC3	0.033744	7.41E-06	153.6718	0.430398
DTNA	1.066341	0.883253	1.287382	0.503931
DAGLA	0.979262	0.819108	1.17073	0.8181
SLC43A3	1.455232	1.00451	2.108191	0.047283
TIMM10	1.536323	0.973646	2.424175	0.065009
DHX34	1.286518	0.872114	1.897835	0.20404
APLNR	0.979049	0.733135	1.307448	0.885916
FADS2	1.211222	0.965652	1.519242	0.097393
TCN1	0.899025	0.794989	1.016676	0.089813
TMEM165	0.435134	0.242648	0.780314	0.005232
CLOCK	0.686208	0.46372	1.015443	0.059657
COL4A2	0.796974	0.516781	1.229084	0.304548
CLDN10	0.852746	0.720974	1.0086	0.06289

UBAC2	1.230933	0.699018	2.167605	0.471728
ARGLU1	0.787906	0.520413	1.192891	0.259968
BIVM	1.021411	0.810951	1.286491	0.857184
ERCC5	0.748306	0.438595	1.276716	0.287454
TPP2	0.51179	0.344813	0.759625	0.000886
CARS2	1.19188	0.661188	2.148522	0.559318
STT3A	0.836404	0.513379	1.362681	0.473161
ACRV1	1.043714	0.705173	1.544782	0.830649
ETS1	1.037135	0.835255	1.28781	0.741308
SLC37A2	1.122665	0.968522	1.301339	0.124658
KLB	0.643515	0.362697	1.141756	0.131859
TMED7	0.779989	0.537576	1.131717	0.190736
APC	0.831744	0.584596	1.183377	0.305808
WDR36	0.812635	0.523935	1.260415	0.354203
OSTF1	1.218939	0.897152	1.656143	0.205523
RFK	1.034123	0.698946	1.530033	0.86668
UBQLN1	0.971062	0.590951	1.595666	0.907744
ANXA1	1.018565	0.829611	1.250556	0.860527
AGTPBP1	0.663008	0.515973	0.851944	0.001316
GOLM1	0.868872	0.643776	1.172674	0.358214
PSAT1	1.299826	1.085875	1.555932	0.004266
ISCA1	1.110452	0.701495	1.75782	0.65483
ADAM19	1.084873	0.878852	1.339189	0.448369
HAVCR2	1.202172	1.004936	1.438119	0.044028
CCNJL	0.397057	0.217628	0.72442	0.002606
TAOK3	1.265893	0.818376	1.958129	0.289424
USP30	0.620032	0.368559	1.043088	0.071701
SDS	1.666156	1.162239	2.388558	0.005467
HNF1A	0.569004	0.139414	2.322339	0.431994
FBXO21	1.237232	0.878622	1.742209	0.222851
OASL	1.343831	1.095096	1.649063	0.004657
HRK	4.395182	1.723955	11.20541	0.001932
RNFT2	1.174268	0.931471	1.480353	0.174056
P2RX4	0.887846	0.566197	1.392221	0.604265
DTX1	1.050394	0.841372	1.311344	0.664078
TRAFD1	1.330806	0.83419	2.123071	0.230445
DMTF1	0.712953	0.461078	1.102421	0.12814
CCDC146	0.620533	0.436101	0.882962	0.00801
TMEM60	1.066838	0.682026	1.668768	0.776839
CD36	1.120785	1.000155	1.255964	0.049689
UGT2B28	0.935413	0.788802	1.109274	0.442702
PNPLA8	0.876467	0.606903	1.26576	0.481952
RINT1	0.631465	0.375479	1.061973	0.08305
SRPK2	0.641014	0.43624	0.94191	0.023529
TES	1.009029	0.825078	1.233992	0.93025
MDFIC	1.000195	0.859514	1.163903	0.997986
MTO1	1.14084	0.65112	1.998887	0.645166
ANKRD6	1.058669	0.788819	1.420833	0.70411
KHDC1	1.469265	1.106953	1.950163	0.007737
SYNCRIP	0.764659	0.48115	1.215219	0.256267
SNX14	0.883095	0.485506	1.606277	0.683784
NT5E	0.866373	0.717858	1.045613	0.134897
AKIRIN2	1.415259	0.949043	2.110501	0.088485
LCA5	0.995633	0.734743	1.349159	0.977479
MAP3K7	0.975527	0.563108	1.689998	0.929577
CGA	4.056403	0.026221	627.5326	0.586175
LMO2	1.42761	0.991186	2.056194	0.055824
PHF21A	0.891379	0.506668	1.568199	0.689937

NAT10	1.223048	0.807119	1.853316	0.342378
PRRG4	1.150859	0.913873	1.449299	0.232329
CAPRIN1	0.870101	0.566835	1.335617	0.524517
DNAJC14	1.134482	0.675986	1.903957	0.632909
CD63	1.005403	0.740785	1.364547	0.972414
AVIL	0.804106	0.560238	1.154126	0.237004
GDF11	0.713031	0.534575	0.951059	0.02137
GLS2	0.940631	0.486375	1.819146	0.855683
ITGA7	1.259143	1.071361	1.479839	0.005166
FAM186B	0.855745	0.630087	1.162221	0.318556
RDH5	1.58627	1.080951	2.327814	0.018386
AGAP2	1.334297	0.961256	1.852106	0.084746
BLOC1S1	1.984776	1.339402	2.941115	0.000635
CDK4	1.340117	0.851566	2.108954	0.205716
PPP1R1A	1.401706	0.889475	2.20892	0.145604
TROAP	0.967059	0.744596	1.255988	0.801713
TSPAN31	0.921038	0.449665	1.886541	0.822099
TFCP2	0.918095	0.521248	1.617077	0.767326
COQ10A	0.630681	0.433262	0.918056	0.016116
PAN2	0.801873	0.571808	1.124504	0.200607
ESPL1	1.092537	0.825581	1.445815	0.535829
ZC3H10	1.004946	0.548989	1.839591	0.98724
HNRNPA1	0.900654	0.61392	1.321308	0.592581
ACVR1B	0.740646	0.551428	0.994791	0.04608
OS9	0.974934	0.641377	1.481963	0.90542
KCNH3	1.215325	0.945716	1.561795	0.127549
LTV1	0.92225	0.55456	1.53373	0.75513
MAP7	1.19867	1.036965	1.385592	0.01425
CD164	1.012366	0.744842	1.375976	0.937432
AHI1	0.738867	0.506311	1.07824	0.11657
HEY2	1.133182	0.653708	1.964332	0.655994
PKIB	1.228509	1.031437	1.463235	0.021058
SMPD2	1.314706	0.816565	2.116736	0.260164
MICAL1	1.317466	1.033238	1.679879	0.02617
REPS1	0.750198	0.418592	1.344498	0.334278
STX11	1.192411	1.023766	1.388838	0.023706
TEC	0.86351	0.669463	1.113802	0.258465
SEMA4F	1.138318	0.862265	1.502749	0.360607
CCT7	1.611181	0.96197	2.698528	0.06989
EGR4	1.058575	0.866873	1.292671	0.576543
RAB11FIP5	1.147392	0.944959	1.393191	0.165042
SMYD5	1.108354	0.72991	1.683012	0.629301
DYSF	1.131514	0.980539	1.305736	0.090837
CCDC142	1.268074	0.769047	2.090915	0.351962
EMX1	0.215427	0.066511	0.697762	0.010463
KCNMB4	0.908246	0.728188	1.132827	0.393279
USP15	0.954965	0.60842	1.498897	0.841214
GNS	1.1368	0.90312	1.430944	0.274809
CPM	1.144936	0.993054	1.320047	0.062325
MDM2	1.287373	0.841979	1.968374	0.243609
KLHL36	0.910865	0.583881	1.420966	0.680721
MPHOSPH	1.253252	0.750777	2.092018	0.387864
KIAA0513	1.225866	1.038824	1.446586	0.015914
DYNC1LI2	0.888196	0.59035	1.336314	0.569431
FBXL8	1.010568	0.72465	1.409296	0.950602
FHOD1	1.192705	0.844323	1.684834	0.317383
CCDC102A	1.385269	1.118272	1.716015	0.002852
SLC9A5	1.237713	0.855339	1.791025	0.257984

AGT	0.948999	0.734756	1.225713	0.688434
KCNK1	1.295136	1.044401	1.606066	0.018489
URB2	1.140146	0.769678	1.688933	0.512981
EGLN1	1.130693	0.868776	1.471573	0.360908
COG2	1.011893	0.602454	1.699596	0.964357
ABCB10	0.770941	0.541317	1.09797	0.149326
TAF5L	0.985688	0.549127	1.769317	0.961479
GLUL	1.058343	0.769968	1.454722	0.726811
STX6	0.642417	0.413823	0.997284	0.0486
RNASEL	1.000971	0.665601	1.505319	0.996282
DHX9	1.095421	0.71026	1.689447	0.680128
KIAA1614	0.593957	0.390168	0.904188	0.015111
CEP350	0.846579	0.570629	1.255976	0.407931
NPL	1.027597	0.875632	1.205935	0.738826
PIGC	1.202077	0.523881	2.758238	0.664045
LAMC1	0.943448	0.840061	1.059558	0.325588
RC3H1	0.661928	0.392245	1.117026	0.12224
GPR55	1.585341	1.154595	2.176786	0.004391
SP110	1.373956	0.964936	1.956353	0.078069
MRPL44	1.627599	0.933362	2.838211	0.085999
CHRND	1.940772	0.771867	4.879851	0.15868
DOCK10	0.961828	0.767202	1.205829	0.735819
TTLL4	1.073766	0.745627	1.546312	0.702099
USP37	0.701661	0.456953	1.077413	0.105403
HTR2B	0.673716	0.46572	0.974605	0.036037
ITM2C	0.898947	0.766858	1.053789	0.188908
SERPINE2	1.032387	0.867265	1.228947	0.720014
DNAJB2	1.645331	1.104688	2.450569	0.014294
WNT10A	1.122645	0.765756	1.645865	0.553401
TMBIM1	1.907554	1.281205	2.840108	0.001472
CYP27A1	1.091603	0.974612	1.222637	0.12968
EIF4E2	1.896183	1.066061	3.372707	0.02943
ARMC9	0.891887	0.614395	1.29471	0.547382
CAB39	1.338815	0.879628	2.037709	0.173354
COX5B	2.306599	1.485151	3.582396	0.000199
REV1	0.637425	0.400525	1.014444	0.057502
TSGA10	0.722145	0.551371	0.945811	0.018047
MFSD9	1.168567	0.690138	1.978662	0.562084
TMEM127	1.557119	1.027858	2.358905	0.036652
EDAR	0.667587	0.37568	1.186308	0.168344
TGFBRAP1	0.982553	0.717542	1.345442	0.912607
GCC2	0.779088	0.532868	1.139078	0.197727
MRPS9	1.057498	0.655676	1.705573	0.818685
ANKRD36	0.727816	0.524229	1.010466	0.057726
EPC2	0.712133	0.437914	1.158067	0.171177
ARHGEF4	0.29585	0.059864	1.4621	0.135181
ISCU	1.131433	0.712165	1.797532	0.601104
ALDH1L2	1.137508	0.809223	1.598971	0.458345
STAB2	1.004639	0.657058	1.536091	0.982954
USP44	0.95411	0.776322	1.172615	0.655244
SCYL2	0.845705	0.528094	1.354336	0.485476
CKAP4	0.991232	0.862415	1.13929	0.901323
PLXNC1	1.263506	1.039073	1.536415	0.019073
APPL2	0.620362	0.387316	0.993631	0.046972
PWP1	1.109176	0.692633	1.776225	0.666251
DRAM1	1.043732	0.798328	1.364573	0.75429
SLC41A2	1.365716	1.018669	1.830996	0.037195
VILL	1.022554	0.69644	1.501372	0.909386

FLNB	1.117689	0.963302	1.29682	0.142375
NEK3	0.994273	0.709067	1.394199	0.973438
VPS36	0.610659	0.377573	0.987634	0.044358
RNASEH2E	0.366514	0.222215	0.604518	8.44E-05
CKAP2	0.917708	0.642049	1.31172	0.637511
TBC1D4	1.0406	0.847268	1.278047	0.704313
THSD1	1.030937	0.720849	1.474414	0.867449
LRCH1	0.769498	0.486233	1.217785	0.263265
SUCLA2	1.129627	0.64461	1.979579	0.670225
RCBTB1	0.93743	0.695835	1.262907	0.670892
MED4	1.05941	0.605751	1.852824	0.839641
PHF11	1.222625	0.728569	2.05171	0.446652
COG3	0.709473	0.434552	1.158323	0.169961
LMO7	0.813707	0.533335	1.241469	0.33884
ITM2B	1.144763	0.740088	1.770711	0.543518
SPRY2	0.81392	0.711794	0.930699	0.002614
NUDT15	1.173497	0.739756	1.861552	0.496775
EDNRB	1.135649	0.694228	1.857746	0.612453
RCBTB2	1.109688	0.875721	1.406164	0.388965
LCP1	1.347402	1.001238	1.813247	0.049053
SETDB2	1.016229	0.676045	1.527592	0.938295
SCRN1	1.130101	1.001541	1.275164	0.04715
TNS3	1.300352	1.086357	1.556501	0.004199
SPDYE1	0.657103	0.441229	0.978595	0.038787
CHST12	1.01041	0.840666	1.214428	0.91212
IGF2BP3	1.855275	1.308994	2.629533	0.000515
GPNMB	1.274525	0.976062	1.664253	0.074753
RAPGEF5	1.046703	0.710971	1.540972	0.817074
RAC1	1.225308	0.766179	1.959569	0.396335
KDELR2	0.713863	0.422472	1.206235	0.207891
IL6	1.340381	0.596589	3.01149	0.478128
ZDHHC4	1.021513	0.544273	1.917218	0.947169
AOAH	0.939392	0.811417	1.087551	0.402736
BZW2	1.131644	0.76797	1.667536	0.531811
TBRG4	1.225409	0.766521	1.959016	0.395778
DDX56	0.952814	0.594735	1.526487	0.840694
HUS1	0.751112	0.431716	1.306805	0.311097
NACAD	0.805254	0.442732	1.46462	0.477906
DBNL	1.544251	1.030589	2.313931	0.035205
CCM2	0.869405	0.586757	1.288209	0.485438
MYO1G	1.406619	1.08151	1.829458	0.01095
TTYH3	1.388346	1.103464	1.746777	0.005107
TTC5	0.901153	0.545213	1.489467	0.684773
ZFHX2	0.734408	0.508735	1.060188	0.099364
MTHFS	1.391178	0.617065	3.136423	0.426039
ADAMTS7	0.633068	0.456519	0.877894	0.006132
IREB2	0.813406	0.508514	1.301104	0.388843
ALPK3	0.918797	0.741203	1.138942	0.439639
TM6SF1	0.728816	0.59978	0.885613	0.001464
CIB2	1.262666	0.999317	1.595416	0.050673
CALCOCO	0.902934	0.681085	1.197045	0.477855
RSAD1	1.043511	0.634545	1.716058	0.866729
NMT1	1.474508	0.813227	2.673513	0.200892
MYCBPAP	1.062816	0.841373	1.342542	0.609307
VEZF1	1.001495	0.635014	1.579479	0.994874
CHAD	1.212686	0.795832	1.847885	0.369548
TACO1	2.298965	1.062276	4.975393	0.034572
TEX2	1.149408	0.82116	1.608868	0.417038

DCAF7	1.172239	0.737882	1.862281	0.501021
LIMD2	1.200279	0.861738	1.671817	0.280232
BRIP1	0.955731	0.714356	1.278666	0.760471
RTP4	1.405848	1.138405	1.736122	0.001556
ACTL6A	0.812653	0.524363	1.259442	0.353382
NDUFB5	1.218545	0.741143	2.003462	0.4359
MRPL47	1.232691	0.68912	2.205027	0.480769
ERMN	1.387721	1.045472	1.842009	0.023346
GALNT5	1.26167	0.961872	1.654909	0.093126
TANK	0.826545	0.507052	1.347349	0.444801
BLK	1.057136	0.869715	1.284946	0.576819
SKIL	0.843026	0.650647	1.092288	0.196345
HLX	1.322992	1.050384	1.666349	0.01743
VPS45	1.082225	0.651472	1.797792	0.760253
IL10	1.47975	1.18107	1.853962	0.000657
KCTD3	1.160211	0.82466	1.632296	0.393574
RPS6KC1	1.211599	0.74911	1.959621	0.433966
CBWD2	1.322453	0.807389	2.166095	0.266937
IL1RN	1.212351	1.06223	1.383688	0.004303
SMPD4	1.224005	0.792993	1.889285	0.361413
WDR33	0.778724	0.374733	1.618249	0.502755
CCDC115	1.672915	0.859263	3.257029	0.130089
SAP130	1.082953	0.644308	1.820228	0.763574
BIN1	1.159964	0.960211	1.401272	0.12383
IMP4	1.436774	0.803362	2.569599	0.221784
HS6ST1	0.93	0.679762	1.272357	0.649981
GYPC	0.87019	0.735149	1.030037	0.106094
STAM	0.787766	0.542417	1.144094	0.210226
ABI1	1.28035	0.663026	2.472444	0.461703
YME1L1	1.122488	0.625672	2.013801	0.698405
DNAJC1	1.392114	1.075751	1.801516	0.0119
NIPSNAP3	1.118794	0.717801	1.743798	0.62009
LRRC8A	0.949415	0.686662	1.31271	0.753513
CDK9	1.337642	0.907018	1.972714	0.142209
TXN	1.316668	0.838147	2.068388	0.23256
ODF2	0.955422	0.576276	1.584016	0.859673
TOR1B	1.059892	0.660921	1.699704	0.809253
SMC2	1.049808	0.773411	1.424982	0.755199
KLF4	1.094535	0.976833	1.22642	0.11967
TOR1A	0.883455	0.571905	1.364726	0.576512
RALGPS1	1.1049	0.763899	1.598122	0.596288
ST6GALNA	1.453722	1.138045	1.856963	0.002742
TMOD1	1.211028	1.004853	1.459507	0.044346
DAB2IP	0.895322	0.638231	1.255975	0.522005
STXBP1	0.820099	0.665332	1.010868	0.063075
SLC2A8	1.362025	1.078454	1.720158	0.009484
ANGPTL2	0.865495	0.621129	1.206001	0.393443
CDK5RAP2	0.946704	0.628754	1.425438	0.793093
SLC31A2	0.838846	0.623459	1.128645	0.24578
SLC31A1	1.197729	0.893236	1.606021	0.227985
TLR4	1.121704	0.973518	1.292446	0.112129
ZNF189	0.899504	0.601463	1.345232	0.606021
STX17	0.825708	0.548469	1.243083	0.358875
PRPF4	1.797119	0.931207	3.468227	0.080552
FPGS	1.301099	0.919482	1.8411	0.137271
USP20	0.979734	0.748947	1.281639	0.881247
ATP6V1G1	1.218393	0.68001	2.183029	0.506771
TEX10	0.741999	0.505044	1.090129	0.128433

GARNL3	1.364333	1.006356	1.849647	0.045417
MRPL50	1.258286	0.844449	1.874931	0.258862
DPM2	1.945801	1.19075	3.179628	0.00789
WDR38	1.344228	0.760987	2.374479	0.30818
TSTD2	0.860073	0.586788	1.260635	0.439705
HEMGN	0.989733	0.87985	1.11334	0.863541
PSMB7	1.376294	0.738173	2.566046	0.314962
NR5A1	0.878406	0.694786	1.110554	0.278556
RABEPK	0.908001	0.488256	1.688592	0.760449
GOLGA1	1.014547	0.624276	1.648801	0.953516
XPA	1.010896	0.66785	1.53015	0.959136
NCBP1	0.925116	0.602859	1.419634	0.721657
ANP32B	1.498614	0.881161	2.548733	0.135428
PDCL	0.931653	0.58126	1.493268	0.768664
RPL35	1.422462	0.887602	2.279624	0.143069
LMX1B	1.165897	0.812215	1.673592	0.405278
ARPC5L	1.74944	1.221997	2.50454	0.00225
ENPP2	1.319093	1.099795	1.582119	0.002832
DSCC1	0.986085	0.733378	1.325871	0.926096
DERL1	1.154247	0.555235	2.399498	0.700839
MYC	0.997855	0.802387	1.240941	0.984598
RANBP6	0.999141	0.676417	1.475838	0.996554
POLR1E	1.136079	0.786264	1.641529	0.496871
PLAA	0.914657	0.517492	1.616638	0.75886
IL11RA	0.909376	0.688989	1.20026	0.502305
UBAP2	0.918165	0.531893	1.584956	0.759211
APTX	1.359365	0.580282	3.184441	0.479637
RNF38	0.737622	0.433744	1.254395	0.261296
TLN1	1.550396	1.154472	2.0821	0.00356
SIT1	1.087593	0.86908	1.361047	0.463095
DNAJB5	2.006377	1.13868	3.535277	0.015983
SPAG8	1.051328	0.669575	1.650735	0.827862
DCTN3	1.45258	0.881229	2.394371	0.143162
CD72	1.153842	0.941358	1.414289	0.168199
TMEM8B	1.139273	0.817964	1.586797	0.440514
GRHPR	1.070681	0.705049	1.625925	0.748669
ALDH1B1	1.192422	0.846286	1.680132	0.314436
DENND4C	0.745145	0.557785	0.995439	0.046492
RPS6	1.091511	0.755217	1.577556	0.641241
CNPY3	1.436159	0.951882	2.166814	0.084534
FOXP4	1.178582	0.898199	1.54649	0.235858
PPIL1	1.204541	0.82468	1.759372	0.335673
KLC4	1.042413	0.664159	1.636092	0.856673
KIF13A	0.951429	0.751208	1.205016	0.679601
PIM1	1.306328	1.061058	1.608294	0.011784
GMPR	0.933888	0.764596	1.140662	0.502685
YIPF3	1.52983	0.857392	2.72965	0.150109
TMEM14B	1.224044	0.680411	2.202024	0.499834
TMEM63B	1.149493	0.870675	1.517597	0.325645
FRS3	1.429282	0.963391	2.120476	0.075955
TJAP1	1.192195	0.723807	1.963686	0.489909
CAPN11	1.002822	0.826812	1.2163	0.977172
IRF4	1.110318	0.903728	1.364134	0.319124
SLC22A23	0.856542	0.647776	1.13259	0.27729
TUBB2A	0.981643	0.816433	1.180285	0.84379
LRRC1	1.000255	0.665661	1.503031	0.999022
GCM1	0.56873	0.045747	7.070452	0.660746
BPHL	0.735422	0.476789	1.134348	0.164574

RIPK1	0.920426	0.545288	1.553647	0.756234
TUBB2B	1.673858	0.598002	4.685267	0.326639
HMGA1	1.462361	1.107285	1.9313	0.007404
TCF19	1.434876	1.019742	2.019009	0.038251
FLOT1	1.411542	1.108261	1.797817	0.005224
IER3	1.170183	0.993482	1.378312	0.05988
MDC1	0.997066	0.599549	1.658149	0.990968
PGBD1	0.785885	0.60422	1.022169	0.072419
TPMT	1.437861	0.982159	2.105001	0.061847
RNF144B	1.25893	1.06026	1.494828	0.008596
NRM	1.750806	1.185615	2.585429	0.004862
MTCH1	1.984989	1.312633	3.001738	0.001158
VARS2	1.009126	0.466049	2.185039	0.981613
TAF8	1.428446	0.801387	2.546157	0.226598
FAM8A1	0.758261	0.481356	1.194458	0.232652
FGFBP2	1.172162	0.990238	1.387509	0.064897
CPEB2	1.155431	0.874	1.527484	0.310401
FHDC1	0.955872	0.786885	1.161148	0.64933
TLR2	1.010259	0.870498	1.17246	0.89312
MYO7A	1.353745	1.150382	1.593058	0.000266
FCHSD2	0.697597	0.462499	1.052199	0.085921
ARRB1	1.268202	0.829305	1.939379	0.272932
SLCO2B1	1.099625	0.814209	1.485091	0.535649
ANKRD42	0.601406	0.352852	1.025045	0.061617
IL18BP	1.287689	0.81766	2.027913	0.275186
NUMA1	1.061713	0.695545	1.620649	0.781392
CCDC90B	0.908037	0.4935	1.670781	0.756495
SYTL2	0.860408	0.638428	1.15957	0.323382
RAB30	0.931117	0.650518	1.332753	0.696491
CREBZF	0.686313	0.43474	1.083466	0.106128
LRRC32	1.384277	1.119232	1.712088	0.002711
PRCP	1.046626	0.712366	1.537729	0.816417
NARS2	0.622748	0.411784	0.941792	0.024824
RNF121	0.735886	0.437415	1.238017	0.247884
MRPL15	1.485069	0.955747	2.307546	0.078633
PI15	1.135313	0.971043	1.327373	0.111503
GGH	1.156942	0.832232	1.608343	0.385753
SLCO5A1	0.936983	0.804548	1.091219	0.402492
TGS1	1.189363	0.762289	1.855706	0.444827
SDCBP	1.020586	0.733066	1.420877	0.90393
NEK1	0.911572	0.596422	1.393248	0.668831
DDX60	1.129385	0.895226	1.424791	0.304727
SORL1	0.70987	0.57789	0.871992	0.001094
BUD13	1.27853	0.696601	2.346593	0.427746
TRPC6	1.083684	0.742987	1.580606	0.676444
MMP7	1.202558	1.075477	1.344655	0.001208
DCUN1D5	0.984738	0.526758	1.840899	0.961572
SLC37A4	1.038767	0.72056	1.497498	0.8385
RDX	0.966742	0.64297	1.453552	0.870874
PPP2R1B	1.064449	0.693534	1.633737	0.775079
FDX1	1.012413	0.599861	1.708697	0.963152
FXYD6	1.288736	1.064054	1.56086	0.009455
TMPRSS13	1.20534	0.845148	1.719041	0.302495
CASP1	1.32179	1.097118	1.592471	0.003335
CASP5	1.100526	0.910591	1.330078	0.321693
ALKBH8	0.966961	0.644731	1.45024	0.870945
MAP2K5	0.787477	0.388854	1.594734	0.506926
CTDSPL2	0.663587	0.409766	1.074633	0.095451

SLTM	0.886194	0.536416	1.464052	0.637148
THBS1	1.174938	1.063386	1.298193	0.001538
MAPKBP1	1.034878	0.776261	1.379656	0.815233
NUSAP1	1.043756	0.476598	2.28584	0.914729
NDUFAF1	1.3468	0.647428	2.801656	0.425643
KIF23	0.933649	0.702262	1.241274	0.636579
ITGA11	0.795097	0.442615	1.428284	0.44296
RTF1	1.297604	0.728524	2.311215	0.376401
PARP6	0.878386	0.547902	1.408212	0.590261
RPLP1	1.533769	1.047211	2.246394	0.028028
PAQR5	0.939472	0.685673	1.287213	0.697576
TUBGCP4	1.045536	0.671071	1.628957	0.84396
ITPKA	0.739166	0.595605	0.91733	0.006086
UACA	1.182173	0.90312	1.547451	0.223143
SMAD6	1.58231	1.020515	2.453374	0.040295
PLCB2	1.499239	1.157651	1.94162	0.002143
TMEM62	0.873581	0.485383	1.572251	0.652159
PAK6	1.077517	0.848282	1.3687	0.540709
ADAM10	0.980555	0.672306	1.430136	0.918776
DUOX1	0.895841	0.611873	1.311599	0.571751
ZNF280D	0.500891	0.307725	0.815312	0.005412
BCL2L10	0.946947	0.810345	1.106576	0.492814
RSL24D1	0.789698	0.515632	1.209433	0.277643
SPTBN5	1.363324	0.785705	2.365588	0.270358
GCHFR	1.46463	0.911992	2.352151	0.114378
BCAR3	0.857703	0.650617	1.130704	0.276286
TTLL7	0.963152	0.736365	1.259786	0.784027
FNBP1L	0.853209	0.741802	0.981347	0.026168
GTF2B	1.971815	0.92495	4.203529	0.078754
RABGGTB	1.055493	0.63808	1.745966	0.833419
IFI44L	1.144085	1.01921	1.284259	0.022452
IFI44	1.177916	0.938597	1.478256	0.157625
SLC44A5	0.858134	0.695196	1.05926	0.15442
DBT	0.749186	0.480001	1.169331	0.203628
IFT172	0.767319	0.57803	1.018595	0.066878
CGREF1	1.215733	0.987749	1.496338	0.065238
HADHB	0.805656	0.48978	1.325253	0.394769
KHK	1.355203	0.896688	2.048175	0.149173
ADCY3	1.241197	0.980831	1.570679	0.072045
PPM1B	0.862383	0.525784	1.414466	0.557567
PNPT1	0.920468	0.57905	1.463192	0.726006
DYNC2LI1	0.751374	0.543455	1.038842	0.083734
THUMPD2	1.225528	0.762501	1.969728	0.400906
CYP1B1	1.046039	0.910491	1.201766	0.524993
RAB1A	1.16996	0.659687	2.074931	0.591297
ACTR2	1.333646	0.935061	1.902135	0.111987
PREB	1.753142	1.108217	2.77338	0.016438
SLC5A6	1.402544	0.882084	2.230093	0.152803
PREPL	0.589239	0.371991	0.933361	0.024207
SLC3A1	0.576404	0.279481	1.188781	0.135766
EMILIN1	0.95505	0.852383	1.070084	0.428009
FBXO11	0.727158	0.440162	1.201281	0.213515
SIX3	0.758237	0.631454	0.910475	0.003031
CENPO	1.125385	0.728571	1.738321	0.594392
LRPPRC	1.001941	0.697619	1.439018	0.991623
DTNB	1.057172	0.751717	1.486747	0.749299
ACTR1A	2.364726	1.349446	4.143869	0.002638
CYP2C8	1.17766	0.978943	1.416716	0.082871

MYOF	1.205709	1.072238	1.355795	0.001777
LOXL4	0.710987	0.578887	0.873231	0.001144
STAMBPL1	0.646891	0.455055	0.919598	0.015225
CH25H	1.499732	1.011475	2.223679	0.043723
ATAD1	0.939257	0.608645	1.449455	0.777101
KIF11	0.981213	0.720371	1.336506	0.904256
DUSP5	1.214505	0.996876	1.479645	0.053743
CALHM2	1.272213	1.005822	1.609156	0.044602
ARL3	1.163264	0.755599	1.790873	0.492109
CEP55	1.071756	0.836884	1.372545	0.582957
KIF20B	1.224951	0.883633	1.698107	0.223384
ENTPD1	0.919606	0.686227	1.232355	0.574705
EXOC6	0.920629	0.560321	1.512628	0.744102
PLCE1	0.845476	0.488362	1.463728	0.548888
DBR1	1.094822	0.65007	1.843855	0.733387
DNAJC13	0.844339	0.591393	1.205473	0.351672
GPR87	0.995204	0.727005	1.362343	0.976059
ANXA7	1.35964	0.743213	2.487334	0.318797
FAM149B1	0.803986	0.520116	1.242786	0.326187
ASCC1	2.734537	1.441082	5.188944	0.002084
ADAMTS1	1.020671	0.865682	1.203408	0.807632
RPS24	0.92724	0.586519	1.465891	0.746487
TET1	1.123462	0.919986	1.371941	0.253487
DNA2	1.055166	0.74051	1.503527	0.766305
AOX1	0.970885	0.773237	1.219055	0.799174
ATIC	1.388726	0.927415	2.0795	0.110901
SMARCAL1	0.995135	0.624158	1.586607	0.983652
BARD1	1.088443	0.754859	1.569442	0.649924
STAT4	1.160006	0.945178	1.423662	0.155493
ASNSD1	1.006266	0.622022	1.627871	0.979693
METTL5	1.793393	0.919731	3.496954	0.086459
SSB	1.143946	0.73625	1.777403	0.549744
NAB1	1.037578	0.689358	1.561698	0.859644
PPIG	1.074348	0.653215	1.766988	0.777569
FASTKD1	1.120826	0.704814	1.782387	0.629849
IDH1	1.421162	1.013607	1.992589	0.041515
OLA1	0.98879	0.613667	1.593219	0.963057
CIR1	1.052636	0.626874	1.767568	0.846192
FAM117B	0.993318	0.71055	1.388616	0.968714
WDR12	0.993262	0.645648	1.528029	0.975458
ABI2	0.868297	0.691638	1.090079	0.223678
ITGAV	1.004212	0.813659	1.239392	0.968768
SLC40A1	1.092648	0.954912	1.25025	0.197449
SLC35A5	1.191801	0.779475	1.822239	0.417967
SENP7	0.618033	0.41999	0.90946	0.014628
COX17	1.241644	0.677961	2.273994	0.483271
PARP9	1.286351	0.975126	1.696908	0.074793
MNS1	0.962676	0.690396	1.342339	0.82256
USP8	0.909354	0.548362	1.507989	0.71272
SECISBP2L	0.788829	0.525051	1.185127	0.253396
TMOD3	1.088452	0.672309	1.762179	0.730248
SPPL2A	1.08022	0.676928	1.723782	0.746235
GLCE	1.169806	0.812294	1.684668	0.399337
SHF	0.804775	0.628432	1.0306	0.08523
APH1B	0.882274	0.559271	1.391826	0.590225
PARP16	1.184146	0.740857	1.892674	0.479944
PPCDC	1.895143	1.248521	2.876658	0.002679
SEMA7A	1.104588	0.921053	1.324695	0.283301

UBL7	2.13437	1.356952	3.357182	0.001035
ARHGAP24	1.099872	0.875461	1.381808	0.413581
FAM13A	1.197944	0.828004	1.733169	0.337856
HERC3	1.058158	0.622369	1.799091	0.834641
HERC6	1.128427	0.817207	1.55817	0.463026
HERC5	1.003359	0.805771	1.249399	0.976091
PCDH10	0.953416	0.646185	1.406723	0.81004
COPS4	0.995686	0.584553	1.695982	0.987306
HNRNPD	1.047441	0.50927	2.154322	0.899752
PRKG2	0.951177	0.775644	1.166433	0.630585
RASGEF1B	1.077777	0.891725	1.302648	0.438523
SEC31A	0.588041	0.350526	0.986494	0.044275
BBS7	1.501725	0.985954	2.287308	0.058216
KIAA1109	0.85332	0.60386	1.205835	0.368617
BMPR1B	1.07507	0.786645	1.469246	0.64969
RAP1GDS1	1.260685	0.747883	2.125101	0.384559
LARP1B	0.427452	0.26229	0.696616	0.000648
MMRN1	1.141461	1.0055	1.295806	0.040881
PDE5A	0.878541	0.623087	1.238728	0.460089
PRDM5	1.09429	0.645395	1.855405	0.738019
NAAA	1.202215	0.921327	1.56874	0.174958
NUP54	0.721178	0.432046	1.203802	0.21115
CXCL9	1.012439	0.817724	1.253521	0.909681
BMP2K	0.760749	0.526113	1.100028	0.146143
G3BP2	0.97754	0.59672	1.601396	0.928128
FRAS1	0.474678	0.082714	2.724072	0.403249
SCARB2	1.035402	0.676304	1.585169	0.872804
CCNG2	0.799598	0.576905	1.108254	0.179331
CNOT6L	0.840855	0.563134	1.25554	0.39676
USO1	0.708826	0.479658	1.047485	0.084142
ANXA3	1.017541	0.877801	1.179526	0.817543
PPA2	1.088589	0.620075	1.9111	0.767532
CENPE	0.903686	0.697113	1.171473	0.444393
GSTCD	1.061259	0.689408	1.633677	0.787054
INTS12	1.097997	0.626862	1.923225	0.743745
CASP6	0.682299	0.494857	0.940739	0.019662
LEF1	0.937727	0.775243	1.134268	0.507802
HADH	1.264674	0.862802	1.853729	0.228755
EGF	1.201693	0.997051	1.448336	0.053736
PAPSS1	0.801243	0.526664	1.218976	0.300645
SEC24B	0.670725	0.399029	1.127417	0.131722
PPP3CA	0.916043	0.537591	1.560916	0.747085
SLC39A8	0.854471	0.645439	1.1312	0.271889
FBN2	0.875716	0.746343	1.027514	0.1037
MAPK8IP3	1.128569	0.836132	1.523284	0.429289
RGS3	0.923968	0.74225	1.150175	0.479105
TTLL8	0.74418	0.37745	1.467225	0.39361
RNF185	1.25639	0.684241	2.306961	0.461642
PARVG	1.435602	0.964677	2.136419	0.074641
B4GALNT3	1.170166	0.868865	1.575949	0.300879
PDE6H	0.836315	0.614506	1.138187	0.255625
ERP27	0.814398	0.574701	1.154069	0.248374
ETV6	0.602781	0.417544	0.870197	0.006889
GABARAPI	0.922036	0.7407	1.147767	0.467543
KIF21A	1.016481	0.780872	1.323179	0.903292
CPNE8	1.265941	1.130051	1.418172	4.70E-05
YARS2	0.884159	0.462642	1.689725	0.709469
FGD4	1.176143	1.02037	1.355697	0.025212

ALG10	0.775971	0.525232	1.146411	0.202742
AEBP2	0.585046	0.321842	1.063502	0.078739
ETNK1	0.810612	0.503131	1.306005	0.388222
ZCRB1	1.606057	0.933765	2.762385	0.086843
TMEM117	1.172332	0.857954	1.601907	0.318203
PRICKLE1	1.142604	0.936496	1.394072	0.189012
C1RL	1.800199	1.28602	2.519959	0.000613
NDUFA9	0.727063	0.362896	1.456673	0.368645
CLSTN3	1.380783	1.047369	1.820335	0.022127
KLRG1	1.41335	1.127977	1.77092	0.002643
VAMP1	0.76575	0.560668	1.045848	0.093326
TAPBPL	1.649668	1.130881	2.406447	0.009365
CD27	1.124146	0.858651	1.471733	0.394587
RBP5	1.282118	0.93552	1.757127	0.122234
PEX5	1.138529	0.729095	1.777888	0.568315
SLC38A4	0.876513	0.673612	1.14053	0.326525
AMIGO2	0.993043	0.775495	1.271618	0.955869
COL2A1	0.675565	0.516669	0.883329	0.004147
LLPH	0.920343	0.505091	1.676985	0.786271
INHBE	2.867745	0.799215	10.29005	0.106062
GLIPR1	1.113046	0.876667	1.413162	0.379241
PHLDA1	0.994706	0.802775	1.232524	0.96129
TMEM19	1.434755	0.876557	2.348418	0.151032
DUSP6	1.094379	0.937949	1.276899	0.251804
TMTC3	0.955708	0.567844	1.608501	0.864573
LUM	0.913737	0.766151	1.089752	0.315527
SNRPF	1.311597	0.802821	2.142801	0.27879
AMDHD1	1.092201	0.779299	1.530738	0.608589
NEDD1	1.014148	0.642751	1.600147	0.951855
SYCP3	0.750571	0.531907	1.059129	0.102467
GAS2L3	1.233556	0.963086	1.579983	0.096487
SLC15A4	1.256219	0.797999	1.977554	0.32448
TDG	0.964254	0.597705	1.555593	0.881417
SDSL	1.436199	1.146885	1.798494	0.00161
MMAB	1.72238	0.986218	3.008051	0.055981
GLTP	1.741491	1.151007	2.634901	0.00865
GIT2	0.831981	0.504975	1.370746	0.470256
TCHP	1.210889	0.66693	2.19851	0.529462
MTMR6	0.871698	0.589192	1.289661	0.492027
SLC46A3	1.037008	0.774895	1.387784	0.806881
SLC7A1	1.053918	0.776599	1.430266	0.736054
LNX2	1.134111	0.773465	1.662919	0.519261
SUOX	0.940255	0.605944	1.459013	0.783463
CCDC65	1.151642	0.774066	1.713393	0.486094
SLC39A5	0.826867	0.427875	1.597919	0.571679
TARBP2	1.294926	0.788081	2.127742	0.307709
DHH	0.450504	0.183732	1.104624	0.081418
ACVRL1	1.159846	0.917665	1.465941	0.214634
GPR84	1.024796	0.843043	1.245733	0.805765
N4BP2L1	0.700741	0.46339	1.059666	0.091928
SMARCC2	1.148962	0.657333	2.008287	0.625996
BRCA2	1.060625	0.753625	1.492687	0.735673
MAP3K12	0.917365	0.619813	1.357761	0.666362
ITGB7	1.389017	1.160049	1.663179	0.00035
GALNT6	1.095819	0.750702	1.599597	0.635404
CSAD	0.911903	0.588361	1.413361	0.679978
LMBR1L	0.933549	0.63786	1.366311	0.723457
ESYT1	0.884287	0.628394	1.244385	0.480473

TMBIM6	1.778494	0.994842	3.179442	0.052075
ANKRD52	1.082811	0.660795	1.774345	0.7522
ZNF740	1.571339	0.937585	2.633475	0.086282
WDFY2	1.124331	0.756223	1.671623	0.562505
HNRNPA1	0.882507	0.627267	1.241606	0.473025
ESD	0.978233	0.59994	1.595061	0.9297
RB1	1.129355	0.824871	1.546233	0.447921
SBNO1	0.668172	0.418399	1.067053	0.091367
MORN3	0.737715	0.547392	0.994211	0.045706
SETD1B	1.037646	0.625581	1.721135	0.886184
VPS33A	1.051836	0.513102	2.15622	0.890248
VPS37B	1.445833	0.914681	2.285422	0.114516
RHOF	1.520015	0.968584	2.385386	0.068586
DENR	1.260761	0.758862	2.094607	0.370991
DIAPH3	1.121159	0.791432	1.588256	0.51984
SLAIN1	0.929574	0.785067	1.100681	0.396906
RBM26	0.700605	0.404938	1.212154	0.203338
MBNL2	1.029382	0.802027	1.321187	0.820096
ABHD13	0.777426	0.47744	1.2659	0.311486
RAB20	1.13007	0.85582	1.492206	0.38859
GRTP1	0.638936	0.447542	0.91218	0.013664
CUL4A	0.976546	0.599591	1.590488	0.924025
CDH24	0.806707	0.662751	0.981931	0.032211
REM2	1.600903	1.099186	2.331624	0.01417
CBLN3	1.752478	1.201194	2.556773	0.003601
TSSK4	0.816296	0.544439	1.223901	0.32598
TMX1	0.888982	0.582769	1.356092	0.584939
FRMD6	0.629342	0.473241	0.836933	0.001453
PELI2	0.867867	0.67231	1.120307	0.276644
RTN1	1.244455	1.045296	1.481561	0.013978
SLC38A6	0.820257	0.49979	1.346208	0.433127
RDH12	1.089816	0.681008	1.744031	0.719952
RAB15	0.851479	0.612953	1.182827	0.337695
WDR89	0.967644	0.677339	1.382372	0.856579
ESR2	0.927124	0.64552	1.331578	0.682064
STON2	0.988083	0.872306	1.119227	0.850455
GPR65	1.132003	0.939388	1.364113	0.192606
PTGR2	0.642974	0.406637	1.01667	0.058859
JDP2	1.264956	0.984407	1.62546	0.066191
AK7	0.696684	0.366279	1.325135	0.270557
SLC24A4	1.016735	0.876816	1.178983	0.826098
FBLN5	0.953288	0.760219	1.195391	0.678656
WDR20	0.694743	0.367607	1.313002	0.262091
NIPA2	1.427534	0.78895	2.582995	0.239408
SLC12A6	1.139483	0.830797	1.562861	0.417929
MFAP1	1.62732	0.980616	2.700519	0.059535
TCF12	0.736811	0.502802	1.079731	0.117229
SORD	0.733808	0.470086	1.14548	0.173139
SERF2	1.994009	1.251212	3.177778	0.003702
ZSCAN29	0.963992	0.633164	1.467677	0.864231
LYSMD2	1.113021	0.692522	1.788849	0.658271
SLC27A2	0.817902	0.675053	0.990979	0.040122
HDC	0.895394	0.808919	0.991114	0.032989
BNIP2	0.729047	0.465222	1.142484	0.16796
GTF2A2	1.177738	0.755565	1.8358	0.470078
SRP14	2.567549	1.444494	4.563747	0.001313
BAHD1	1.058259	0.664422	1.685543	0.811543
DISP2	0.855982	0.549859	1.332532	0.491046

CDAN1	0.799752	0.525482	1.217175	0.297042
TLE3	1.242123	0.881214	1.750846	0.21573
ANP32A	1.173376	0.634569	2.16968	0.610194
COMMD4	1.416704	0.907667	2.211219	0.125159
UBE2Q2	0.554224	0.340906	0.901026	0.017298
PSTPIP1	1.11506	0.916362	1.356841	0.276743
ETFA	1.458845	0.845469	2.517215	0.17483
BCL2A1	1.24503	1.087242	1.425718	0.001526
HMG20A	0.942913	0.598735	1.484939	0.799746
SCAPER	0.897622	0.571526	1.409778	0.639127
TSPAN3	0.673671	0.395824	1.146554	0.14542
WDR61	1.074191	0.647959	1.780802	0.781403
NCOA2	1.058859	0.759538	1.476138	0.735821
NEIL1	1.121319	0.786065	1.599558	0.527521
MAN2C1	1.01767	0.721122	1.436168	0.920612
DNAJA4	1.107388	0.900127	1.362372	0.334658
TPM1	1.115797	0.881038	1.413109	0.363297
IGF1R	0.867979	0.746623	1.009061	0.06539
ARRDC4	1.184804	0.911943	1.539307	0.204171
PIF1	0.893358	0.682387	1.169553	0.411957
USP3	1.150823	0.805278	1.644642	0.440625
BBS4	0.897965	0.571964	1.409775	0.640028
PML	1.111952	0.785757	1.573562	0.549177
CYP1A1	2.378364	0.685606	8.250534	0.172187
ADAMTS1	0.546995	0.298941	1.000879	0.050334
LINS1	1.028558	0.618613	1.710167	0.913563
ULK3	1.543829	1.038657	2.294702	0.031751
PCSK6	1.216742	0.878348	1.685505	0.238061
SCAMP2	2.219972	1.345238	3.663497	0.001806
HAPLN3	1.114484	0.889817	1.395876	0.345351
POLG	1.452026	0.88407	2.384856	0.140688
RLBP1	1.220946	0.921599	1.617526	0.164213
FANCI	1.019336	0.724265	1.434621	0.912542
ABHD2	1.30775	0.953242	1.794098	0.096286
DET1	0.568538	0.367172	0.880336	0.011365
MFGE8	1.037614	0.875186	1.230188	0.670764
ZNF710	1.249961	0.860035	1.816675	0.242179
UNC45A	1.855338	1.174606	2.930581	0.00805
MCTP2	0.898665	0.753957	1.071145	0.232973
FURIN	1.115928	0.82398	1.511318	0.478436
IQGAP1	1.062794	0.848685	1.330919	0.595707
CRTC3	1.021726	0.704595	1.481595	0.909748
SEC11A	1.211776	0.756813	1.940244	0.42383
PMM2	0.65869	0.396852	1.093285	0.106317
ITGAX	1.324186	1.145759	1.530399	0.000143
TGFB1I1	0.951319	0.723206	1.251384	0.721256
ARMC5	0.926268	0.74917	1.145229	0.479289
PARN	1.101977	0.571617	2.124415	0.771852
FTO	0.710036	0.502273	1.003739	0.052521
UQCRC2	1.23166	0.705609	2.149896	0.463492
CDR2	0.720607	0.483736	1.073466	0.107107
IGSF6	1.111294	0.947487	1.30342	0.194639
ARHGAP1	1.696091	1.058568	2.717562	0.028049
NKD1	0.720864	0.5498	0.945152	0.01788
DHX38	1.080612	0.698304	1.672227	0.727832
TXNL4B	0.840539	0.466647	1.514004	0.562881
ZFHX3	0.729112	0.587144	0.905406	0.004245
CPNE2	1.029644	0.778207	1.362321	0.837957

NLRC5	1.04065	0.823287	1.3154	0.738892
KATNB1	1.097228	0.822715	1.463335	0.527647
KIFC3	1.144408	0.934	1.402215	0.193166
ADAMTS1	0.660717	0.481838	0.906002	0.010089
NUDT7	0.801835	0.602347	1.067388	0.130241
GCSH	0.655241	0.373479	1.149572	0.14049
CMTM3	1.431736	0.93064	2.202643	0.10249
CMTM2	1.215942	1.000222	1.478188	0.049741
NOL3	0.932245	0.684189	1.270235	0.656683
MAP1LC3E	1.193596	0.750918	1.89724	0.454186
MBTPS1	0.670267	0.467378	0.961231	0.029637
ZCCHC14	0.884229	0.680223	1.149417	0.357887
OSGIN1	0.733087	0.439111	1.223874	0.235075
IRF8	1.17789	1.042799	1.330481	0.008432
RHOT2	1.168124	0.83758	1.629114	0.359856
RPL3L	2.231898	1.272579	3.914388	0.005096
RPS2	1.198738	0.836755	1.717315	0.323014
NDUFB10	2.201778	1.320742	3.670535	0.002471
PDPK1	1.131324	0.635	2.015581	0.675397
TIGD7	1.096577	0.648616	1.85392	0.730762
DEF8	1.026742	0.758221	1.390359	0.864527
TCF25	1.142179	0.69031	1.889838	0.604856
GALNS	0.835525	0.623054	1.120453	0.230031
GAS8	0.774595	0.558295	1.074695	0.126317
MED9	1.876339	1.075944	3.272147	0.026559
NCOR1	0.892904	0.559389	1.425265	0.634954
COPS3	0.910404	0.580869	1.426888	0.682235
ZNF287	1.135464	0.790557	1.630848	0.491626
KSR1	1.160167	0.885961	1.51924	0.280204
RANBP10	1.004794	0.665895	1.516172	0.981821
DPEP3	1.067309	0.908969	1.253231	0.426586
GFOD2	0.697956	0.41882	1.163132	0.167578
NOB1	1.020006	0.688665	1.510765	0.921269
PRPSAP2	1.396774	0.778061	2.507487	0.262989
PCTP	1.303333	0.929093	1.828317	0.125009
TOM1L1	1.110901	0.883497	1.396837	0.368126
TOB1	1.072701	0.809342	1.421757	0.625363
VPS53	0.8098	0.526055	1.246591	0.337798
SGSM2	1.074429	0.774374	1.490749	0.667453
NPEPPS	0.523425	0.28225	0.970679	0.039936
SKAP1	1.095101	0.903914	1.326726	0.353398
LRRC46	1.090213	0.713825	1.665065	0.689345
SCRN2	1.147777	0.784401	1.67949	0.477922
SSH2	1.041139	0.769326	1.408987	0.793965
RHBDL3	1.038379	0.836533	1.288929	0.73273
ARSG	0.993856	0.728023	1.356758	0.969046
G6PC3	1.222895	0.824064	1.814751	0.317731
CLTC	1.096501	0.72033	1.669115	0.667392
BCAS3	0.656997	0.385593	1.119432	0.122343
PTRH2	0.910876	0.537025	1.544983	0.729133
SS18	0.654018	0.371564	1.151187	0.141044
TAF4B	0.942089	0.718818	1.234709	0.665553
AFG3L2	1.169967	0.697708	1.961885	0.551723
IMPA2	1.044926	0.631394	1.729301	0.864243
GNAL	1.286286	0.643933	2.569415	0.475756
SLC39A6	0.831108	0.566805	1.218657	0.343472
RPRD1A	0.837071	0.490822	1.427578	0.513775
GALNT1	0.571919	0.410673	0.796475	0.000944

ADCYAP1	1.061795	0.808676	1.394142	0.666063
ESCO1	0.790122	0.472661	1.320805	0.368871
OSBPL1A	1.030944	0.833801	1.274699	0.778381
PELP1	1.071938	0.67915	1.691896	0.765446
NPC1	1.230944	0.810028	1.870579	0.330464
SLC14A1	0.711074	0.590275	0.856594	0.000331
ARRB2	1.249313	0.973357	1.603504	0.080479
ZMYND15	1.153802	0.894278	1.488639	0.271133
MINK1	1.670457	1.123828	2.482966	0.011173
SAT2	0.955098	0.671824	1.357813	0.797998
ASGR1	1.23008	0.886032	1.707724	0.216052
PIK3R5	1.47584	1.13419	1.920405	0.003764
TP53	1.127047	0.747822	1.698581	0.567679
ARHGDIA	1.423209	1.018822	1.988105	0.038518
TMC6	1.183686	0.870208	1.610089	0.282689
SLC16A3	1.275407	1.031606	1.576826	0.024612
CARD14	1.267361	0.711743	2.256719	0.420898
TTYH2	1.245761	0.95381	1.627075	0.106774
RAB40B	0.832958	0.573149	1.210539	0.337941
EIF4A3	1.37731	0.82555	2.297841	0.220247
CSNK1D	1.229224	0.766463	1.971382	0.391798
ANAPC11	1.210486	0.800775	1.829823	0.364884
TBCD	0.967381	0.642181	1.457262	0.873954
FN3KRP	1.241789	0.688025	2.241255	0.472267
NARF	0.800383	0.528571	1.21197	0.292879
RPTOR	0.773336	0.556802	1.074078	0.125129
FOXK2	0.92722	0.549834	1.563631	0.776863
TRIM65	0.88835	0.526172	1.499825	0.657734
CBX8	0.778529	0.518908	1.168044	0.226466
SECTM1	1.153071	1.027258	1.294293	0.015684
RNF157	1.036766	0.744714	1.443351	0.830636
ZNF750	0.680183	0.459305	1.007282	0.054386
CBX4	0.969544	0.653299	1.438874	0.877963
RNF165	1.074096	0.811071	1.422418	0.617933
DYM	0.670612	0.396599	1.133941	0.135979
ELAC1	1.198593	0.611062	2.351032	0.598192
MBD1	1.020837	0.612988	1.700045	0.936834
SMAD4	0.627769	0.365096	1.079425	0.092261
TNFRSF11,	1.130513	0.916416	1.394629	0.252147
ZCCHC2	1.372993	0.949215	1.985966	0.092334
PMAIP1	1.030518	0.812012	1.307821	0.804717
ERBB2	1.243396	0.78321	1.973969	0.355602
PNMT	1.033329	0.806784	1.323488	0.795136
ARL5C	1.103933	0.905692	1.345567	0.327527
IGFBP4	0.918487	0.794757	1.061481	0.249421
FKBP10	0.794174	0.618078	1.020443	0.071585
TXNL4A	1.095648	0.510469	2.351651	0.814667
SAMD1	1.263915	0.83965	1.902558	0.261685
BRD4	1.358308	0.695588	2.652432	0.369789
SLC39A3	0.873834	0.665001	1.148247	0.333103
NFIC	0.796027	0.475362	1.333003	0.385811
PRDM15	0.998598	0.659031	1.513129	0.994722
PFKL	1.82646	1.276723	2.612906	0.000977
FEM1A	0.989228	0.538233	1.818121	0.972178
VAV1	1.718106	1.076446	2.742255	0.023283
CIB3	0.790724	0.613022	1.019938	0.070614
SH3GL1	1.243511	0.866449	1.784663	0.237088
DUS3L	1.013608	0.734729	1.398341	0.934382

DPP9	1.322436	0.844709	2.070343	0.221695
CCDC97	1.687113	1.012604	2.810924	0.044638
TMEM91	1.250565	0.821471	1.903796	0.297043
ZFP14	0.73306	0.504393	1.065394	0.103548
SIRT3	0.861531	0.55125	1.346458	0.512972
IFITM3	1.117803	0.994889	1.255903	0.060965
COL6A1	0.997136	0.868604	1.144687	0.967508
IFNAR1	1.059145	0.675672	1.660254	0.802165
SOD1	1.632508	1.065597	2.501023	0.02433
COL6A2	0.986574	0.843095	1.15447	0.866129
SIK1	1.364994	1.02864	1.811334	0.031116
TRPM2	1.10291	0.898693	1.353532	0.348465
SCYL1	1.53979	1.010424	2.346493	0.044621
TMEM50B	0.466495	0.266394	0.8169	0.007643
APP	0.905843	0.827049	0.992144	0.033186
URB1	1.12865	0.781929	1.629112	0.518088
AKT1	1.26033	0.844515	1.880879	0.257351
EMP3	1.344033	1.08009	1.672474	0.008034
SAE1	1.630609	0.927895	2.865504	0.089169
NTN5	1.033244	0.699307	1.526644	0.869576
LMTK3	1.033232	0.676109	1.578987	0.879909
GEMIN7	1.320413	0.857513	2.033195	0.206948
ADAMTS1	1.045682	0.904329	1.209128	0.546621
RNPEPL1	1.642895	1.214086	2.223158	0.001295
CAPN10	1.333433	0.871318	2.040639	0.185017
MYO1F	1.306281	1.024005	1.666369	0.031482
NLRP12	1.130794	1.005177	1.27211	0.040766
ZNF787	1.310683	0.869618	1.975451	0.196169
CARM1	1.408031	0.940292	2.108443	0.096691
EVI5L	1.43777	0.960302	2.152638	0.077858
SLC47A1	1.102165	0.793071	1.531727	0.562386
PSMB6	1.488083	0.93768	2.361563	0.091625
GPR32	0.930884	0.776953	1.115313	0.437397
SIGLEC10	1.191808	0.996109	1.425956	0.055192
ZNF473	0.872236	0.535233	1.421431	0.583275
FAM71E1	0.765298	0.461033	1.270368	0.300912
RPS11	1.256043	0.781309	2.019233	0.346635
PTH2	1.106495	0.74152	1.651109	0.620214
RPL13A	1.199791	0.809332	1.778625	0.364511
NOSIP	1.480008	0.943809	2.320833	0.087634
RCN3	1.065031	0.889456	1.275264	0.493052
ZNF614	0.916231	0.628457	1.335778	0.649232
SLC2A5	1.348528	1.145848	1.587059	0.00032
RERE	1.281858	0.841518	1.952613	0.247518
PRDM16	1.227633	1.097255	1.373504	0.000343
PADI3	1.264028	1.083161	1.475095	0.00294
EPHA2	1.168165	0.932816	1.462892	0.175709
ARHGEF19	1.227301	0.853808	1.764175	0.268602
EFHD2	1.511398	1.157026	1.974306	0.002446
PEX14	1.399817	0.850486	2.303961	0.185848
PGD	1.474752	1.046793	2.077674	0.02632
SH3BGRL3	1.689665	1.305546	2.1868	6.72E-05
CNKSR1	1.027393	0.710443	1.485743	0.885828
RPL11	1.124426	0.690633	1.830689	0.637236
KIAA0319L	1.046869	0.604646	1.812524	0.870086
PLK4	1.000595	0.729824	1.371825	0.997051
MAP3K6	1.401112	1.116175	1.758787	0.003644
GPN2	2.250161	1.135973	4.457169	0.020043

SYTL1	1.179618	0.930944	1.494718	0.171447
WDTC1	1.006383	0.634548	1.596109	0.978427
NBPF3	0.788509	0.626873	0.991823	0.042345
HSPG2	0.94452	0.805908	1.106974	0.480882
ITGB3BP	0.730561	0.456604	1.16889	0.19047
SERBP1	1.055752	0.693292	1.607709	0.800391
BCL10	1.573212	0.925515	2.674183	0.094131
PRKACB	1.02936	0.743795	1.424562	0.861434
PIGK	0.938438	0.59553	1.478792	0.784205
TINAGL1	1.064105	0.805847	1.40513	0.661338
RPS8	1.455649	0.94963	2.231305	0.084922
KIF2C	1.164023	0.849467	1.595059	0.344689
PTPRF	0.815333	0.641003	1.037076	0.09624
BEST4	2.035095	1.139035	3.636071	0.016413
LMO4	1.018336	0.848578	1.222053	0.845174
MTF2	0.685215	0.473319	0.991971	0.045212
IGSF3	0.875525	0.51981	1.47466	0.617261
ZNF697	0.790141	0.612731	1.018918	0.069446
CTTNBP2N	1.234883	0.985607	1.547206	0.066663
PSMA5	1.951338	1.113269	3.420306	0.019559
FNDC7	1.430985	0.518821	3.946865	0.488749
CD53	1.206115	0.876488	1.659708	0.249907
CELSR2	1.366483	1.003195	1.861329	0.047682
ITGA10	1.268139	0.738643	2.177205	0.389006
GPR161	0.864417	0.583508	1.28056	0.467449
ALDH9A1	1.168262	0.740698	1.842634	0.503553
ATP1B1	0.864927	0.729962	1.024846	0.093653
TIPRL	0.955176	0.553729	1.647668	0.869056
NME7	0.791676	0.517802	1.210405	0.280845
POGK	1.064464	0.61533	1.841424	0.823219
CREG1	1.565939	0.999335	2.453798	0.05034
DCAF6	0.770214	0.482002	1.230764	0.274949
GPA33	1.054827	0.917	1.213371	0.454983
TBX19	0.822505	0.533551	1.267947	0.376219
UCK2	0.728137	0.514278	1.030928	0.073731
TMCO1	0.922466	0.469136	1.81385	0.815033
XCL1	1.087427	0.765839	1.544057	0.63939
XCL2	1.16591	0.932861	1.45718	0.177294
POU2F1	0.880443	0.52571	1.474539	0.628419
ILDR2	1.183372	0.964572	1.451803	0.106494
MGST3	1.051036	0.673959	1.639085	0.826222
ADCY10	1.101715	0.632017	1.920479	0.732614
UFC1	1.164184	0.662717	2.045102	0.596927
PPOX	1.005019	0.713983	1.414687	0.977106
FCGR2A	1.024168	0.888468	1.180594	0.741933
NUF2	0.908035	0.672815	1.225489	0.528257
RGS5	0.994791	0.587192	1.685327	0.98451
SDHC	1.376233	0.700587	2.703473	0.353913
PFDN2	1.609397	0.98333	2.634069	0.058346
NR1I3	1.091847	0.728452	1.636525	0.670434
USP21	1.17971	0.67203	2.070912	0.564864
PRCC	1.984381	1.064638	3.698691	0.030997
FCRL5	0.98821	0.767693	1.272071	0.926654
MRPL24	1.91201	1.093973	3.34175	0.022891
PIGM	1.079823	0.705722	1.652233	0.723423
CASQ1	0.816736	0.495035	1.347495	0.428092
ISG20L2	2.167281	1.16625	4.02753	0.014429
CRABP2	1.299759	0.959986	1.759788	0.089919

HDGF	1.172278	0.71639	1.918277	0.527006
ABL2	0.78734	0.49815	1.244411	0.305964
XPR1	1.08978	0.687466	1.727532	0.71455
RGS16	1.024846	0.766294	1.370634	0.868589
TOR1AIP1	0.756511	0.470901	1.215348	0.248647
RGL1	1.274921	0.997212	1.629968	0.052661
LYPLAL1	0.622789	0.381336	1.017124	0.058475
LHX9	0.045805	2.14E-06	980.0384	0.544457
TUFT1	1.24138	0.944634	1.631347	0.120827
SF3B4	1.548573	0.934844	2.565215	0.089447
ECM1	0.997794	0.856449	1.162464	0.977389
ZNF687	1.496922	0.919882	2.435939	0.104415
TARS2	1.197439	0.704283	2.035915	0.505807
SNX27	0.91948	0.608441	1.389523	0.69028
SETDB1	0.678925	0.389267	1.184121	0.172417
ADAMTSL4	1.073347	0.90079	1.27896	0.428627
MCL1	0.956261	0.730729	1.251401	0.744516
CTSK	0.771256	0.510397	1.165439	0.217533
RFX5	1.620309	1.016072	2.583874	0.04267
PI4KB	1.717195	0.986135	2.990217	0.056053
PIP5K1A	1.190855	0.59107	2.399266	0.625035
ANP32E	0.865543	0.592576	1.264251	0.455075
ANXA9	1.09196	0.739333	1.612775	0.658387
SELENBP1	0.908767	0.795989	1.037524	0.157045
ENSA	2.227382	0.970966	5.109582	0.058703
SEMA6C	0.975876	0.764096	1.246352	0.844888
MRPL9	1.738345	0.928576	3.254275	0.083928
ARNT	0.912596	0.534548	1.55801	0.737511
POGZ	0.924288	0.549771	1.553935	0.766447
HORMAD1	1.084713	0.894772	1.314974	0.407719
GOLPH3L	0.865095	0.569823	1.313371	0.49632
GABPB2	1.08736	0.631209	1.873152	0.762788
DTL	1.13163	0.843431	1.518308	0.409622
DYRK3	1.004873	0.82356	1.226105	0.961807
INTS7	1.25805	0.794453	1.992176	0.327663
VASH2	0.675002	0.43394	1.049979	0.081227
TAF1A	0.822879	0.525402	1.288784	0.39441
SMYD2	0.928226	0.654355	1.316722	0.676294
SUSD4	0.962517	0.493164	1.878562	0.910845
DUSP10	0.817655	0.625812	1.068308	0.140044
TP53BP2	0.681219	0.430665	1.077541	0.100848
ATP8B2	1.072238	0.852638	1.348396	0.550829
ADAM15	1.608716	1.199315	2.15787	0.001509
JTB	1.466538	0.923579	2.328695	0.104591
RAB13	1.102815	0.839991	1.447875	0.481058
S100A8	1.085922	1.007375	1.170594	0.031415
TPM3	1.817422	1.064178	3.103825	0.028688
NUP210L	0.797152	0.482738	1.31635	0.375672
SNAPIN	2.454275	1.390714	4.331204	0.001948
SLC27A3	1.011498	0.785648	1.302274	0.929335
UBAP2L	1.249062	0.674581	2.312776	0.479234
SLC39A1	1.750891	0.802094	3.822016	0.159638
HAX1	1.905605	0.952817	3.811152	0.068259
CREB3L4	0.834848	0.600192	1.161248	0.283688
EFNA3	1.285248	0.821985	2.009601	0.271164
AQP10	1.287191	0.839461	1.97372	0.247034
GATAD2B	1.003309	0.541491	1.858991	0.991625
ILF2	1.079544	0.676237	1.723385	0.748428

RIT1	1.213029	0.802746	1.833005	0.359222
INTS3	1.476606	0.90424	2.411266	0.119313
PKLR	0.921459	0.787826	1.077758	0.306201
HCN3	1.152395	0.833433	1.593426	0.390934
ACTA1	1.598752	0.845153	3.024314	0.149107
GALNT2	0.850548	0.576734	1.25436	0.41413
TTC13	1.079039	0.728664	1.59789	0.704128
SCCPDH	0.683444	0.502004	0.930462	0.015614
LYST	0.844148	0.70269	1.014084	0.070216
CEP170	0.540365	0.363093	0.804184	0.002411
ACP1	0.907309	0.504679	1.631157	0.745159
SNAP47	1.936472	1.240264	3.023489	0.003647
SRP9	1.035345	0.633091	1.693184	0.889918
NVL	0.97319	0.498395	1.900298	0.936558
DEGS1	1.690426	1.031995	2.768948	0.037065
FBXO28	0.764454	0.416582	1.402823	0.385857
ARF1	1.426639	0.950588	2.141096	0.086285
CNIH4	1.260562	0.901645	1.762353	0.175613
ITPKB	1.249764	0.822122	1.899852	0.296779
GUK1	1.38531	0.991782	1.934987	0.055932
CDC42BPA	0.977015	0.834607	1.143722	0.772361
CNIH3	0.477546	0.272317	0.837444	0.009909
MBOAT2	1.116595	0.959023	1.300056	0.155348
PARP1	1.539295	1.015869	2.332416	0.041929
PYCR2	1.974645	1.115657	3.495	0.019509
LBR	0.686522	0.505044	0.933211	0.016338
WNT9A	1.122784	0.660018	1.910014	0.669214
EPHX1	0.767924	0.562799	1.047813	0.095833
REN	1.269737	0.914456	1.763049	0.153873
SOX13	0.908702	0.720517	1.146036	0.418721
ETNK2	0.964883	0.585685	1.589592	0.888386
PPFIA4	1.182681	0.980486	1.426571	0.079438
PLEKHA6	1.201567	0.996384	1.449004	0.0546
PTPN7	1.406588	1.069059	1.850683	0.014811
SYT2	1.109317	0.751674	1.637126	0.601358
ARL8A	1.310286	0.919236	1.867692	0.135093
GDF7	0.799309	0.500101	1.277533	0.349141
PDIA6	0.791529	0.569286	1.100534	0.164441
RHOB	0.990396	0.853007	1.149914	0.899214
ATP6V1C2	0.514531	0.310022	0.853948	0.010147
GALM	1.421611	1.10836	1.823396	0.005604
EML4	1.24719	0.854457	1.820433	0.252292
CALM2	1.275875	0.803882	2.024996	0.301268
CHAC2	1.248604	0.820386	1.900341	0.300162
RPS27A	0.964072	0.62783	1.480393	0.86721
VPS54	0.689959	0.415786	1.144924	0.150941
ASXL2	0.926299	0.61343	1.398741	0.715794
ETAA1	0.76719	0.494281	1.19078	0.237396
SNRPG	1.554501	0.869266	2.779901	0.136873
ABHD1	0.912614	0.599585	1.38907	0.669639
MEIS1	1.159397	1.043476	1.288195	0.005927
CIAO1	1.168787	0.625818	2.182845	0.624582
ZNF514	0.711242	0.516541	0.979334	0.036804
SNRNP200	1.12059	0.760318	1.651575	0.565074
MRPS5	1.729295	0.839111	3.563846	0.13767
ANKRD53	1.021155	0.651818	1.599767	0.927178
TPRKB	1.086371	0.64287	1.835832	0.756959
EXOC6B	0.918243	0.695012	1.213175	0.548385

SFXN5	0.950169	0.630383	1.432178	0.807103
TEX261	1.797139	1.004533	3.215136	0.048245
DQX1	1.216573	0.668255	2.214796	0.521318
DUSP11	1.348964	0.702406	2.590671	0.368636
ST6GAL2	0.949899	0.720489	1.252354	0.715524
THNSL2	0.962409	0.765615	1.209787	0.7427
RALB	1.337493	0.933071	1.917204	0.113447
TMEM177	1.34343	0.90441	1.995557	0.143657
RABL2A	0.745769	0.490239	1.134489	0.170546
SLC20A1	1.239258	0.719065	2.135774	0.439869
FBLN7	0.795718	0.427716	1.480347	0.470628
ZC3H8	0.905494	0.584592	1.402552	0.656557
LIPT1	0.662577	0.428651	1.024162	0.063952
FAHD2B	1.266388	0.933551	1.717891	0.129014
LYG1	0.772476	0.514278	1.160303	0.213617
AFF3	1.058451	0.898663	1.246651	0.496294
UBXN4	0.899177	0.521622	1.550009	0.702075
SPOPL	0.978887	0.672639	1.424569	0.911246
POLR2D	0.983179	0.557482	1.733942	0.953268
AMMECR1	0.727023	0.39169	1.349441	0.312372
PKP4	0.889137	0.692358	1.141844	0.357225
SCRN3	0.770024	0.500513	1.18466	0.234444
CDCA7	0.900268	0.763172	1.061992	0.21261
DLX1	0.810756	0.492167	1.335574	0.410079
UBR3	0.873925	0.564258	1.353536	0.546014
PHOSPHO	0.812462	0.589099	1.120514	0.205433
FAM171B	1.087515	0.902771	1.310066	0.377139
HSPD1	1.040783	0.744839	1.454315	0.814847
CCDC150	0.948283	0.540089	1.664986	0.853315
PTH2R	0.936708	0.815244	1.076268	0.356157
NBEAL1	0.709561	0.488877	1.029865	0.071052
SPAG16	1.264154	0.939744	1.700555	0.12133
SUMF1	1.11344	0.65038	1.906192	0.695269
RHBDD1	1.269634	0.768502	2.097547	0.351339
HES6	0.829451	0.686791	1.001745	0.052154
ANKMY1	1.20028	0.777275	1.853492	0.410254
COPS7B	1.141722	0.614551	2.121108	0.674932
DIS3L2	0.710955	0.408313	1.237916	0.227945
CPNE9	1.031679	0.697667	1.525601	0.875835
FANCD2	1.140245	0.79104	1.643606	0.48175
VGLL4	0.837492	0.542655	1.292521	0.423129
RAB5A	0.910616	0.472901	1.753476	0.779415
CTDSP1	1.456921	0.929989	2.282414	0.10037
STK11IP	1.453326	0.961956	2.195691	0.075777
GMPPA	1.402995	0.842665	2.335918	0.192978
EAF1	1.294611	0.875692	1.913934	0.195496
CNTN4	1.498303	0.164846	13.61821	0.719548
DYNC1LI1	1.07676	0.564581	2.053576	0.822359
OSBPL10	0.876256	0.633486	1.212061	0.424835
SLC25A38	0.739074	0.511898	1.067067	0.106626
ITGA9	0.999944	0.886593	1.127787	0.999268
GOLGA4	0.726998	0.488433	1.082085	0.116137
CTDSPL	1.065051	0.900309	1.259936	0.462293
STAC	0.932516	0.719543	1.208526	0.597376
IQSEC1	1.205604	0.959029	1.515576	0.109239
CAND2	1.172491	0.873331	1.574128	0.289694
RPL32	1.080502	0.702202	1.662606	0.724744
PTPRG	0.652599	0.474294	0.897934	0.008763

SHQ1	0.958664	0.587774	1.563586	0.865691
SLC25A26	0.937036	0.513217	1.710848	0.832321
UBA3	1.322737	0.752201	2.326018	0.331439
ARL6IP5	1.984419	1.350192	2.916561	0.000486
TMF1	0.686157	0.439836	1.070425	0.09691
LRIG1	0.988117	0.761865	1.28156	0.928208
LIMD1	1.014381	0.694706	1.481157	0.941068
ZNF660	0.682086	0.520719	0.893459	0.005471
NFKBIZ	0.9013	0.726141	1.11871	0.345922
MYH15	4.051081	1.904863	8.615452	0.000279
PHLDB2	0.813954	0.555266	1.193161	0.291463
ABHD10	0.979991	0.667803	1.438123	0.917738
PLA1A	1.052494	0.506655	2.186385	0.890904
RABL3	0.804297	0.484415	1.335413	0.399858
ADPRH	1.049873	0.837334	1.316362	0.67324
ATG3	1.455616	1.063174	1.992919	0.019174
NR1I2	0.984877	0.781685	1.240887	0.897156
SRPRB	0.920948	0.57623	1.471887	0.730676
AGTR1	0.979407	0.675482	1.420079	0.912589
MED12L	1.094283	0.958936	1.248734	0.181055
EIF2A	0.631246	0.391753	1.017149	0.058744
OSBPL11	1.091285	0.835987	1.424549	0.520574
TRPC1	0.962045	0.744286	1.243515	0.767604
LPP	1.363674	0.911355	2.040485	0.131418
TMEM44	0.899086	0.670589	1.205439	0.477053
AMT	1.275379	0.693288	2.346199	0.43414
TCTA	2.299874	1.461825	3.618368	0.000316
UCN2	1.705796	0.514199	5.658778	0.382753
STXBP5L	1.122538	0.796432	1.58217	0.509178
EAF2	1.37439	0.979222	1.929029	0.065979
ILDR1	1.306615	0.862754	1.978829	0.206637
TM4SF19	1.060425	0.508592	2.211009	0.875642
EIF2B5	1.264499	0.688775	2.321453	0.448985
DGKQ	1.067739	0.790489	1.44223	0.669168
FIP1L1	0.906601	0.452763	1.815355	0.781946
SLC26A1	1.054129	0.754369	1.473003	0.757479
LYAR	1.376017	0.923378	2.050538	0.116813
ATP10D	0.778548	0.599253	1.011488	0.060871
OCIAD2	1.185493	0.925815	1.518006	0.177364
SLC10A4	1.038712	0.741046	1.455946	0.825515
SCD5	1.143641	0.965165	1.35512	0.121046
PLAC8	0.90279	0.652076	1.2499	0.537826
ENOPH1	1.411297	0.832854	2.391488	0.200448
KLHL8	0.881953	0.600232	1.295899	0.522309
SNCA	0.945588	0.765915	1.167411	0.602821
CAMK2D	1.201395	0.981154	1.471074	0.075762
CISD2	1.029875	0.559603	1.895348	0.924641
DDIT4L	0.796787	0.656294	0.967354	0.021715
TIFA	1.27835	0.964708	1.693962	0.087302
SPATA5	0.876887	0.568256	1.353141	0.552798
CCNA2	0.955663	0.735032	1.24252	0.734898
METTL14	0.660351	0.359596	1.212649	0.180828
USP53	0.971259	0.75434	1.250555	0.821091
SETD7	1.149341	0.942325	1.401834	0.169539
NAF1	0.637008	0.371912	1.091063	0.100479
SFRP2	1.015469	0.514131	2.005669	0.964742
RPS3A	0.861163	0.610947	1.213855	0.393421
RNF175	1.04176	0.857952	1.264948	0.679551

PDGFC	0.955303	0.785741	1.161456	0.646478
CBR4	0.651493	0.415516	1.021485	0.061856
CYP4V2	1.155632	0.924916	1.443901	0.203008
ROPN1L	0.804435	0.645629	1.002302	0.052444
NDUFS6	1.751876	1.071261	2.864914	0.025465
NKD2	0.944473	0.785894	1.13505	0.54241
SRD5A1	1.201382	0.900723	1.602399	0.211855
MYO10	0.907649	0.546347	1.507883	0.7083
RPL37	1.154464	0.743117	1.79351	0.522796
SKP2	0.881412	0.574968	1.351181	0.562506
OSMR	0.634026	0.437774	0.918255	0.015898
PLK2	1.232082	1.01606	1.494031	0.033841
GZMA	1.062027	0.879979	1.281737	0.530481
PIK3R1	0.782865	0.534381	1.146895	0.208943
LHFPL2	0.818808	0.674904	0.993395	0.042646
SSBP2	0.750772	0.605731	0.930543	0.008865
IQGAP2	0.867625	0.663364	1.134781	0.299841
CRHBP	1.135228	0.987128	1.305546	0.075351
RASA1	0.708738	0.473505	1.060833	0.09433
LIX1	0.847396	0.485822	1.478075	0.559645
GIN1	0.825006	0.540108	1.260182	0.373458
PAM	0.882501	0.720821	1.080446	0.226051
BDP1	0.642801	0.436451	0.946712	0.025274
GTF2H2	1.158275	0.766111	1.751184	0.486001
SLC30A5	0.678558	0.396376	1.161627	0.157434
BTF3	0.756692	0.494307	1.158356	0.199382
FBXL17	0.92156	0.546815	1.553126	0.759043
SPATA9	0.52189	0.372151	0.731878	0.000164
TNFAIP8	0.728781	0.535034	0.992687	0.044801
FEM1C	0.598972	0.401349	0.893902	0.012107
COMMD1(	0.68467	0.424167	1.105161	0.120983
ATG12	0.580672	0.362076	0.931242	0.024098
MEGF10	0.889274	0.713018	1.109101	0.297783
YIPF5	1.039014	0.632654	1.706383	0.879817
ARHGAP2(	1.072641	0.842945	1.364928	0.568444
DDX46	0.817887	0.501936	1.332717	0.419672
TIMD4	1.119761	0.948025	1.322608	0.182983
RNF145	1.613746	1.15969	2.245579	0.004528
FBXO38	0.893027	0.632752	1.260362	0.51983
PCYOX1L	1.326911	0.940652	1.871778	0.10709
TNIP1	1.53528	0.986239	2.389974	0.057618
G3BP1	0.568413	0.344554	0.937714	0.026983
ZNF300	1.062069	0.867824	1.299792	0.558996
N4BP3	1.236692	0.867291	1.763431	0.240603
NHP2	1.83849	1.125254	3.003807	0.015053
RMND5B	1.712298	1.231504	2.380799	0.001383
KCNMB1	1.489813	1.22581	1.810674	6.18E-05
FAM50B	1.123272	0.9122	1.383182	0.273679
MYLK4	0.942883	0.733073	1.212742	0.646971
TBC1D7	1.121189	0.61077	2.058164	0.712054
FARS2	0.895535	0.488387	1.642107	0.721343
GFOD1	1.298089	1.025992	1.642348	0.029723
CDKAL1	0.690753	0.41982	1.136535	0.145333
PSD2	1.186623	0.802926	1.753679	0.390563
LRRTM2	0.737892	0.496941	1.095673	0.131817
ZMAT2	0.827442	0.498713	1.372853	0.463406
GFRA3	0.43268	0.262872	0.712181	0.000984
KLHL3	1.282178	0.983585	1.671418	0.066122

TRIM7	1.067859	0.807327	1.412465	0.645443
TRIM41	2.162736	1.096419	4.266096	0.026045
HIGD2A	1.80772	1.240487	2.63433	0.002059
FAM193B	0.791523	0.551141	1.13675	0.205534
PLA2G7	1.012204	0.821482	1.247206	0.909334
TNFRSF21	1.057958	0.897044	1.247738	0.503313
RNF44	0.834432	0.540033	1.289323	0.414892
DOK3	1.178984	0.885282	1.570124	0.259999
ABT1	1.370135	0.726364	2.584474	0.330763
DAAM2	1.158591	0.927491	1.447275	0.194689
PRIM2	1.120901	0.636654	1.973473	0.692504
FGD2	1.204624	1.063039	1.365066	0.00352
SCUBE3	0.799955	0.575841	1.111291	0.183255
ANO7	0.975374	0.863282	1.102021	0.688926
CRIP3	1.617388	1.23729	2.114253	0.000435
TCTE1	1.322165	0.827929	2.111437	0.242272
RPL7L1	1.049449	0.622251	1.769934	0.856377
NFKBIE	1.396381	0.949801	2.052934	0.089501
TPBG	0.877809	0.751837	1.024887	0.09916
IRAK1BP1	0.635915	0.463784	0.871931	0.00494
PHIP	0.603199	0.390883	0.930839	0.022387
PNRC1	0.837749	0.50468	1.390633	0.493555
PM20D2	0.971657	0.7618	1.239324	0.816845
RARS2	0.780494	0.361498	1.685129	0.527978
SCML4	0.975754	0.774094	1.229948	0.835391
RNF217	1.145471	0.991966	1.322731	0.064301
ARHGAP1[illegible]	1.343382	0.960083	1.879708	0.085017
SHPRH	0.595393	0.415476	0.85322	0.004733
AIG1	0.911956	0.76976	1.080419	0.286593
DYNLT1	1.06413	0.759178	1.491577	0.718264
TIAM2	1.020519	0.62063	1.678066	0.936202
TMEM181	0.707936	0.500219	1.001908	0.051271
PNLDC1	0.893882	0.558937	1.429546	0.639591
WTAP	0.9413	0.479852	1.846499	0.860319
ZMYM4	0.690175	0.407142	1.169964	0.168502
VWDE	1.130099	0.961986	1.327591	0.136659
GNA12	1.227884	0.92511	1.629749	0.155285
SDK1	0.785502	0.457888	1.347519	0.380603
WASH2P	1.307563	0.885659	1.930449	0.177305
RBAK	0.690116	0.446155	1.067476	0.095602
CREB5	1.163734	0.997316	1.357922	0.054126
CDCA5	1.127822	0.805715	1.578698	0.483294
IGFBP3	0.929493	0.738459	1.169946	0.533372
PURB	0.757108	0.462987	1.238074	0.267478
MDH2	1.604805	0.998264	2.579877	0.050844
POMZP3	0.653618	0.427729	0.998801	0.049355
CCT6A	0.8723	0.604031	1.259714	0.466232
PSPH	0.979756	0.666205	1.440881	0.917229
ZNF92	0.667516	0.47264	0.94274	0.021749
ATXN7L1	0.654501	0.409756	1.045429	0.076058
TMEM168	0.682407	0.459199	1.014111	0.058673
ASB15	1.180002	0.391989	3.552153	0.768476
SLC12A9	1.127035	0.8794	1.444403	0.344797
GIGYF1	0.724118	0.486194	1.078472	0.112227
TRIM4	0.751716	0.472691	1.195446	0.227914
MEPCE	1.122564	0.686229	1.836342	0.645212
TMEM209	0.948404	0.615922	1.460365	0.809919
AGBL3	0.847946	0.506977	1.418235	0.529673

ZC3HAV1L	0.695713	0.546123	0.886277	0.00331
TMEM140	1.233489	0.840644	1.809915	0.283426
TLK2	0.615166	0.342656	1.104397	0.103659
EPHA1	1.119137	0.798605	1.568319	0.513267
NOM1	0.704209	0.440272	1.126371	0.143365
NCAPG2	1.024621	0.743548	1.411943	0.881811
RAB19	0.895884	0.600319	1.336969	0.590406
LUC7L2	0.468779	0.297706	0.738154	0.001073
SH3KBP1	1.053114	0.695056	1.595626	0.807145
LANCL3	1.01041	0.764793	1.33491	0.9419
CASK	0.717564	0.504315	1.020987	0.065106
SPIN2A	0.542518	0.367091	0.80178	0.002152
MSN	1.819767	1.127191	2.937879	0.01429
HDAC8	0.597769	0.375684	0.95114	0.029904
SLC16A2	0.762725	0.604684	0.962072	0.022234
ZNF182	0.817966	0.487962	1.371146	0.445841
CHST7	1.54708	1.170695	2.044474	0.002155
NDUFB11	1.30586	0.856539	1.990883	0.214879
ZNF41	0.870335	0.519584	1.457865	0.597735
RAB41	1.211386	0.641941	2.285965	0.553937
ZMYM3	0.938109	0.652233	1.349284	0.730454
TAF1	0.812654	0.515803	1.280348	0.371089
GPR174	0.85203	0.716504	1.013191	0.070032
NONO	0.960924	0.5745	1.607268	0.879286
CCDC120	1.024149	0.721094	1.454569	0.893955
LPAR4	0.839362	0.713638	0.987234	0.034417
EBP	2.17715	1.401855	3.381222	0.000532
OGT	0.823252	0.601581	1.126606	0.224304
SNX12	1.51613	0.773312	2.972475	0.225682
ITGB1BP2	0.678803	0.40529	1.136897	0.140923
IL2RG	1.055957	0.859301	1.297618	0.604583
ZNF711	0.877283	0.790131	0.974049	0.014187
DIAPH2	0.856944	0.652494	1.125456	0.26695
NXF3	0.993674	0.881623	1.119966	0.917202
PRPS1	1.682212	0.976433	2.898139	0.060926
DOCK11	1.11125	0.78816	1.566784	0.547298
IGSF1	1.166789	0.819096	1.662072	0.392821
GPC3	1.567566	1.180624	2.081326	0.001884
RBMX	0.819939	0.495781	1.356044	0.439277
MCPH1	0.886347	0.550002	1.428377	0.62022
MFHAS1	1.334596	0.953805	1.86741	0.092179
FBXO25	0.688655	0.463055	1.024166	0.065468
NSDHL	1.267481	0.748602	2.146011	0.377643
ZNF185	1.086123	0.94698	1.24571	0.237556
CETN2	0.943133	0.615831	1.444391	0.787761
RPL10	1.207205	0.818121	1.781329	0.342797
CSGALNA(	1.011484	0.86137	1.187758	0.88921
ATP6V1B2	1.080284	0.782872	1.490684	0.638329
CCDC25	1.37318	0.845802	2.22939	0.199621
HMBOX1	0.903018	0.581723	1.401769	0.649345
CHRNA6	0.714824	0.556402	0.918352	0.008633
GNRH1	0.827172	0.503473	1.358989	0.45383
BIN3	1.072667	0.710397	1.619678	0.738649
DOK2	1.399042	1.175794	1.664677	0.000153
SLC25A37	0.920105	0.729481	1.160542	0.482065
CHMP7	1.669898	0.936722	2.976934	0.082148
DOCK5	0.754231	0.625635	0.909259	0.003103
STAR	0.740874	0.592203	0.926869	0.008677

ERLIN2	1.037714	0.675051	1.595213	0.865998
ST18	0.889141	0.766033	1.032032	0.122274
RGS20	0.753195	0.427513	1.326984	0.326649
TACC1	1.230903	0.789382	1.919378	0.359381
GOLGA7	0.908068	0.528524	1.560169	0.72692
GINS4	1.460815	0.9336	2.285753	0.09708
DNAJC5B	1.400737	1.033455	1.898547	0.029851
ADHFE1	1.455888	1.021139	2.075731	0.037935
MRPS28	1.118867	0.658412	1.901338	0.678023
LACTB2	1.195549	0.850317	1.680946	0.30427
TERF1	0.769465	0.42838	1.382129	0.380504
RPL7	0.951882	0.690767	1.3117	0.763076
ATP6V0D2	0.910569	0.417806	1.984498	0.813667
MTDH	1.049636	0.660327	1.66847	0.837678
LRP12	0.841755	0.668059	1.060612	0.144039
EBAG9	0.906778	0.541991	1.517087	0.709391
POLR2K	1.50673	0.873081	2.600258	0.140898
EIF3H	1.002861	0.655777	1.533648	0.989483
UTP23	1.072672	0.724074	1.589098	0.726451
NDUFB9	1.587381	1.076088	2.341612	0.019823
TATDN1	0.802529	0.478107	1.34709	0.40514
FAM83A	1.158147	0.921187	1.456061	0.208723
GSDMC	0.930484	0.781308	1.108144	0.418997
ZNF7	0.66072	0.378422	1.15361	0.144997
SLC39A4	1.333123	0.960805	1.849716	0.085309
VLDLR	1.065929	0.894353	1.27042	0.475832
AK3	1.252741	0.824196	1.904109	0.291492
UHRF2	0.530494	0.311251	0.904171	0.019793
NFIB	0.951187	0.630317	1.435401	0.811597
PLIN2	0.929301	0.755658	1.142845	0.487198
HAUS6	0.757658	0.450399	1.274528	0.29564
CDKN2B	1.086881	0.87832	1.344965	0.443433
CDKN2A	0.97285	0.797598	1.186611	0.785926
IFNK	1.034543	0.833009	1.284835	0.758696
ZCCHC7	1.031329	0.694502	1.531514	0.878468
FBXO10	0.719897	0.508418	1.01934	0.064023
SIGMAR1	1.930592	1.403021	2.656542	5.36E-05
CBWD5	0.514844	0.275842	0.960929	0.037057
CEP78	0.963857	0.638548	1.454895	0.860897
AUH	0.516682	0.327828	0.81433	0.004444
ZNF462	1.106129	0.883464	1.384915	0.379109
UGCG	0.949313	0.752627	1.197398	0.660569
SNX30	0.854619	0.632686	1.1544	0.305815
STOM	1.156507	0.896991	1.491107	0.262077
GSN	1.334147	1.065728	1.670171	0.01189
MRRF	0.821446	0.491706	1.37231	0.452533
NR6A1	1.172775	0.992786	1.385397	0.060822
CRB2	0.952886	0.674717	1.345736	0.784077
ALAD	1.297803	0.90499	1.861117	0.156422
WDR31	0.760921	0.551408	1.05004	0.096352
POLE3	1.25382	0.729958	2.153636	0.412486
SURF4	1.059716	0.72904	1.54038	0.761178
GBGT1	1.026581	0.737343	1.429278	0.876531
SURF1	1.706141	0.990972	2.937434	0.053949
SURF2	0.964842	0.639881	1.454834	0.864373
SURF6	1.358481	0.748348	2.466061	0.313902
MED22	1.279417	0.820222	1.99569	0.277355
REXO4	1.50796	0.875202	2.59819	0.13894

RPL7A	1.141049	0.783982	1.660743	0.490791
GTF3C5	1.190309	0.896833	1.579821	0.227772
ASB6	1.139437	0.707243	1.835743	0.591646
PTGES2	1.364429	0.889438	2.09308	0.15465
CIZ1	1.299416	0.846396	1.99491	0.231116
SLC25A25	1.389198	0.940176	2.052669	0.098886
SH3GLB2	1.350864	0.966129	1.888808	0.078665
PTGES	1.109711	0.781595	1.575571	0.560508
LCN2	1.007076	0.877846	1.155329	0.919849
LRSAM1	1.678387	0.942256	2.989615	0.078741
GPR107	1.002227	0.600133	1.673729	0.993215
IDI2	0.92938	0.560108	1.542107	0.77682
INPP5E	0.894515	0.60617	1.320022	0.574471
SEC16A	1.072717	0.639715	1.798805	0.790125
NOTCH1	1.074794	0.828698	1.393973	0.586666
NACC2	1.116195	0.940191	1.325148	0.209278
USP6NL	1.171608	0.786194	1.745962	0.436499
COMMD3	1.410856	1.067808	1.864114	0.015455
MSRB2	1.435929	0.901758	2.286525	0.127432
PDSS1	1.215731	0.770213	1.918953	0.401564
FAM171A1	0.925642	0.81728	1.048373	0.223858
RSU1	1.769384	1.112921	2.813066	0.015855
ST8SIA6	1.093256	0.973412	1.227855	0.132304
PARD3	0.859489	0.691897	1.067674	0.171235
ZEB1	0.940176	0.782627	1.12944	0.509755
NRBF2	1.374738	0.868939	2.174956	0.173905
POLR3A	1.005756	0.614804	1.645313	0.981766
HERC4	0.819138	0.464222	1.445401	0.491109
CAMK2G	0.845727	0.605879	1.180523	0.324778
GLUD1	1.616563	1.045238	2.500176	0.030863
ANKRD1	1.771582	0.654796	4.793099	0.260107
HTR7	1.360345	1.175953	1.57365	3.46E-05
RPP30	2.241007	1.06209	4.728521	0.034168
ADD3	1.270358	0.975509	1.654325	0.075742
DNAJB12	2.015073	1.20107	3.380752	0.007956
EIF4EBP2	1.189177	0.697291	2.028052	0.524678
TCF7L2	1.018107	0.822283	1.260566	0.869219
MKI67	0.97197	0.780899	1.209793	0.799048
LRRC27	1.696971	1.068297	2.695607	0.025106
MTG1	0.94752	0.525926	1.707071	0.857562
NKX6-2	0.588732	0.281139	1.232865	0.160067
PAOX	1.624917	0.993471	2.657707	0.053129
GSTO1	1.636349	1.068525	2.505919	0.023527
TAF5	1.09621	0.600727	2.000369	0.764687
PPRC1	0.976811	0.672712	1.418379	0.901878
ITPRIP	1.195612	0.892922	1.600911	0.230318
CNNM2	0.659625	0.378143	1.150637	0.142733
PDCD11	0.999896	0.669143	1.494138	0.999595
RGS10	1.345538	1.101352	1.643862	0.003675
BTBD10	1.318506	0.738791	2.353112	0.349486
ADM	1.139497	0.943738	1.375862	0.17451
GAS2	1.193588	0.843345	1.689289	0.318007
LIN7C	0.793218	0.491418	1.280367	0.342988
IMMP1L	0.541274	0.344789	0.849729	0.007639
TUT1	0.909928	0.533646	1.551532	0.72883
SCGB1A1	1.291972	0.987709	1.689962	0.061525
HSD17B12	1.009265	0.651972	1.562361	0.967006
APIP	1.068935	0.633799	1.802814	0.802609

DGKZ	0.919314	0.677107	1.24816	0.589748
EIF3M	0.880276	0.558649	1.38707	0.582558
TNKS1BP1	0.89852	0.669493	1.205896	0.475967
SERPING1	0.887328	0.7784	1.0115	0.073638
SSRP1	1.573242	0.953153	2.596738	0.076344
SLC43A1	0.825264	0.603479	1.128558	0.229119
PTPRJ	1.026037	0.883112	1.192094	0.736988
ARFGAP2	1.416637	0.822602	2.439648	0.209179
CCDC81	0.918475	0.520299	1.621366	0.769304
SESN3	0.897254	0.756972	1.063533	0.211349
ENDOD1	1.117619	0.933776	1.337658	0.225237
CCDC82	0.649581	0.415883	1.014602	0.057927
KLHL35	0.807753	0.509866	1.27968	0.363109
SERPINH1	0.964701	0.806781	1.153532	0.693571
CAPN5	1.233848	0.926012	1.644017	0.151278
INTS4	0.676699	0.393148	1.164755	0.158683
PAK1	1.379157	1.087689	1.74873	0.007958
RPS3	1.250223	0.823278	1.898578	0.294787
ZC3H12C	1.013508	0.851531	1.206297	0.879957
TTC12	0.947954	0.751159	1.196307	0.652557
NCAM1	1.082577	0.922254	1.270771	0.331919
NPAT	0.919839	0.625641	1.35238	0.670906
ATM	0.935757	0.654428	1.338023	0.715905
AASDHPP	0.973112	0.626293	1.511987	0.903514
GLB1L2	1.287679	1.011024	1.640038	0.040482
P4HA3	0.730538	0.30294	1.761691	0.484492
ST14	1.210598	1.060678	1.381709	0.004607
HYOU1	0.880016	0.652531	1.186807	0.402252
MTA2	2.310025	1.307433	4.081446	0.003939
TMEM138	0.926117	0.519074	1.652353	0.794987
FADS1	1.474898	1.092337	1.99144	0.011198
ROM1	1.820116	0.944192	3.508633	0.073699
EML3	1.537892	1.009585	2.342657	0.045029
INCENP	1.044532	0.719736	1.515899	0.818652
ZP1	1.395018	0.638031	3.050128	0.404232
MS4A3	0.898189	0.822741	0.980557	0.016459
PLCH2	1.230471	0.880776	1.719005	0.224072
MS4A2	0.903798	0.78183	1.044793	0.171457
B3GAT3	1.444507	0.966065	2.159897	0.073171
EI24	2.227536	1.380495	3.594304	0.001035
CCDC15	0.914244	0.660119	1.266199	0.589493
CHEK1	1.037536	0.753683	1.428293	0.821238
FEZ1	1.43028	1.004314	2.036914	0.047275
ESAM	1.011171	0.868208	1.177676	0.886413
KIRREL3	0.827578	0.668074	1.025164	0.083192
MPZL2	1.352948	1.111058	1.6475	0.002631
SIDT2	1.275119	0.9838	1.652701	0.066277
TMEM25	1.007924	0.797262	1.274249	0.947396
TAGLN	1.183544	0.893767	1.567273	0.23955
DUSP15	0.813044	0.557458	1.185812	0.282433
COMMD7	2.759814	1.559647	4.883523	0.00049
DSN1	1.350761	0.861579	2.117689	0.190009
LSM14B	0.969992	0.640092	1.469921	0.88577
YTHDF1	0.94831	0.555183	1.619812	0.845946
CABLES2	1.599785	1.064504	2.404231	0.023777
GPHA2	1.24028	0.616831	2.493866	0.545691
TRPT1	0.966636	0.658155	1.419703	0.86263
NUDT22	1.610438	1.034624	2.506719	0.034795

FERMT3	1.953302	1.402477	2.720464	7.46E-05
PLCB3	1.325977	0.953027	1.844873	0.094044
MRPL49	3.016751	1.65899	5.485737	0.000296
FAU	1.388726	0.959121	2.010757	0.082045
TM7SF2	0.988014	0.661584	1.475508	0.95301
TBX6	1.210472	0.812392	1.803614	0.347838
PPP4C	1.631748	1.023896	2.600462	0.039468
ALDOA	1.161882	0.752547	1.793867	0.498359
DOC2A	0.712222	0.566528	0.895385	0.003657
HIRIP3	2.201253	1.346795	3.597813	0.001646
TAOK2	1.411784	0.852856	2.337012	0.179912
TMEM219	1.636176	0.921002	2.906697	0.093096
HMGA2	1.163833	0.961186	1.409205	0.120093
CNKSR2	1.19386	0.820025	1.73812	0.355175
KLRF1	1.156926	0.97122	1.37814	0.102501
MKX	1.403115	0.545392	3.609755	0.482364
MPP7	1.288118	1.074739	1.543862	0.006144
ITGB1	0.795927	0.576899	1.098112	0.164529
CTF1	0.953746	0.697793	1.303583	0.766429
CWC15	1.192802	0.621554	2.289061	0.596029
FCGR1A	1.110852	0.942194	1.309701	0.210842
ARID5B	1.311585	1.045493	1.645402	0.019052
DCUN1D2	0.61246	0.400326	0.937005	0.023828
TMCO3	0.781813	0.517141	1.181943	0.243108
TMEM218	0.847817	0.467726	1.536784	0.586431
TIRAP	1.140302	0.728324	1.785316	0.565962
LATS2	1.041811	0.717637	1.512423	0.829474
SAP18	1.064103	0.573333	1.974968	0.843894
KIAA1328	0.81792	0.522865	1.279474	0.378632
FAM124A	1.125649	0.93611	1.353564	0.208337
MIA2	0.633457	0.321161	1.249427	0.187701
HNMT	1.194283	1.053687	1.35364	0.005465
PDCD4	0.591126	0.365125	0.957013	0.032459
ADRA2A	0.955863	0.818348	1.116485	0.568947
WDR17	1.101967	0.856821	1.417252	0.44946
CCDC102E	1.168962	0.744818	1.834638	0.497229
CD226	1.161771	0.897478	1.503894	0.254868
FSIP1	1.46639	0.650323	3.306508	0.356136
RGS18	0.837402	0.65177	1.075904	0.165201
PRSS23	1.15566	0.763278	1.749758	0.494242
MTMR12	1.579604	1.051245	2.373516	0.027771
CCT5	1.23801	0.831181	1.843966	0.293568
DOCK1	1.247436	1.116021	1.394325	9.92E-05
DIXDC1	0.746606	0.556859	1.001009	0.050793
DLAT	1.030608	0.630883	1.683598	0.904166
TIMM8B	1.671717	1.030361	2.71229	0.037425
IL18	1.085519	0.821277	1.43478	0.564242
TEX12	0.633684	0.342867	1.171169	0.145456
PTS	0.721741	0.438748	1.187266	0.199127
PIP4K2A	1.580252	0.966635	2.583392	0.068054
FOXO1	1.056301	0.882803	1.263897	0.549627
CRIM1	0.829701	0.645097	1.067132	0.145963
SEC24D	0.80975	0.569451	1.151451	0.240053
ABCB9	1.22996	0.775503	1.950736	0.379096
RILPL2	1.292521	0.81966	2.038175	0.269509
DHX37	1.485909	0.968824	2.278975	0.069551
UBC	1.541719	1.089357	2.181927	0.014567
ITPR1	1.2429	0.901598	1.713402	0.184326

SLC7A11	1.402399	1.062526	1.850987	0.016928
CACNA2D	1.028238	0.874146	1.209492	0.736747
DCP1B	1.028967	0.612288	1.729207	0.914143
CACNA1C	0.653341	0.481873	0.885825	0.006134
KCNA6	0.948557	0.794628	1.132305	0.558826
THRB	0.949469	0.711178	1.267603	0.725077
NGLY1	0.61535	0.38894	0.973558	0.038037
OXSM	1.09177	0.692456	1.721356	0.705467
UEVLD	0.90833	0.643589	1.281973	0.584422
TMEM86A	0.88957	0.647993	1.22121	0.469177
BTBD11	0.855059	0.725822	1.007307	0.061085
UBE3B	0.958086	0.587208	1.563211	0.863893
ANK3	1.137089	0.854247	1.513581	0.378643
IPMK	0.948914	0.689532	1.305867	0.74755
RAD9B	0.992272	0.601536	1.636817	0.975765
DLG5	0.968684	0.788604	1.189884	0.761735
SLC2A13	0.853633	0.630071	1.156521	0.30706
GXYLT1	0.864491	0.571955	1.306648	0.489624
TWF1	0.796181	0.496322	1.277205	0.344527
DIP2C	0.740579	0.439952	1.246631	0.258351
EIF4E	0.634028	0.355318	1.131357	0.123015
MAGI1	1.063862	0.703963	1.607758	0.768888
CSNK1G3	1.135939	0.78777	1.637987	0.494897
SRFBP1	0.772161	0.509914	1.16928	0.221978
FAM177A1	0.574604	0.279381	1.181791	0.132074
MBIP	0.809686	0.576778	1.136642	0.222512
EXT2	0.705723	0.474925	1.048681	0.084574
TMEM18	0.821273	0.485093	1.390432	0.46358
THRSP	0.584846	0.332062	1.030061	0.063253
NDUFC2	0.791178	0.482516	1.29729	0.353217
ME3	1.150922	0.963604	1.374652	0.120925
NUBPL	0.740531	0.455689	1.203423	0.225313
NEK7	0.812575	0.512535	1.288258	0.377394
FER	0.831449	0.636116	1.086763	0.176697
ANKRD50	0.714952	0.562766	0.908291	0.006003
UPF2	1.161404	0.738564	1.826328	0.517079
CDC123	1.643886	0.991092	2.726652	0.054191
SCLT1	0.761999	0.483973	1.199741	0.240536
CCDC3	1.227365	1.046688	1.43923	0.011682
FRMD4A	0.947459	0.713275	1.25853	0.709458
PTPRO	1.418405	1.127862	1.783794	0.0028
EPS8	1.254726	1.057789	1.488327	0.00919
ACAD8	0.904297	0.556781	1.468717	0.684345
THYN1	1.523175	0.861709	2.692395	0.147658
VPS26B	1.563829	0.877952	2.78553	0.129001
NCAPD3	1.180582	0.787885	1.769007	0.421078
VTI1A	1.117164	0.557474	2.238769	0.754746
QDPR	1.753233	0.98058	3.134702	0.058249
FAM160B1	0.750816	0.527369	1.068937	0.111814
TEX9	0.960034	0.635629	1.450007	0.846284
MMAA	1.102783	0.689388	1.764072	0.683143
ZNF827	1.094273	0.925747	1.293478	0.29107
NR3C2	0.822811	0.564192	1.199979	0.311046
AKR1C2	1.299306	0.929258	1.816713	0.125778
DPYSL4	1.383997	0.961922	1.991271	0.079978
VENTX	1.221656	1.084148	1.376604	0.001016
ADAM8	1.113244	0.956511	1.295659	0.165853
ITIH2	1.3817	0.93762	2.036106	0.102182

KIN	1.083708	0.618544	1.898687	0.778738
PIGF	0.721172	0.454582	1.144104	0.165066
ANKAR	0.794962	0.524541	1.204795	0.27939
INPP1	1.670564	1.111913	2.509893	0.013484
MFSD6	1.260477	0.937738	1.694292	0.125035
RNF144A	0.884337	0.674035	1.160255	0.374995
ASAP2	1.067134	0.880135	1.293863	0.508596
ADAM17	0.856079	0.571372	1.282651	0.451288
FLI1	0.955563	0.634521	1.439037	0.827744
KCNJ1	0.907371	0.700532	1.175281	0.461483
TMEM45B	0.696188	0.356149	1.360881	0.289628
WWC2	0.758029	0.627744	0.915354	0.003987
ACSL1	1.103089	0.934315	1.302351	0.246844
SLC25A4	1.576171	1.052553	2.360274	0.027204
AMN1	0.881532	0.547507	1.419339	0.603838
BICD1	0.937219	0.668363	1.314226	0.707004
SAV1	1.334808	0.9561	1.863521	0.089834
CCDC122	0.972129	0.701814	1.34656	0.86498
SERP2	0.992438	0.840428	1.171941	0.928693
NBAS	0.877095	0.616523	1.247796	0.465926
ZNF385D	0.94937	0.773063	1.165886	0.620113
GUF1	0.826859	0.502757	1.359892	0.453877
SACS	0.842194	0.616576	1.150371	0.280373
PABPC3	1.095832	0.749138	1.602973	0.637227
CENPJ	0.74068	0.566556	0.968318	0.028134
FBXO4	1.005529	0.604832	1.671686	0.983038
CCL28	1.103031	0.902845	1.347604	0.337201
PARP8	1.033568	0.84646	1.262037	0.745915
DST	0.945091	0.787246	1.134584	0.544709
BEND6	0.928372	0.781236	1.103219	0.398563
TIAL1	0.820958	0.418245	1.611427	0.566409
BAG3	1.348175	1.134793	1.601679	0.000678
GLT1D1	1.001663	0.86623	1.15827	0.982119
SCHIP1	1.300567	1.104983	1.53077	0.001575
AP1S3	1.313166	1.061579	1.624378	0.012051
RABGAP1L	0.838263	0.523477	1.342342	0.462706
TUBA3E	0.976008	0.587096	1.622549	0.925394
FAM168B	1.239196	0.659239	2.329363	0.505406
PTPN14	0.82801	0.698374	0.98171	0.029822
MGAT5	1.00284	0.631674	1.592099	0.990406
TMEM163	1.272188	0.997631	1.622305	0.052277
GEMIN6	1.293286	0.782534	2.137403	0.315703
POU4F1	0.87395	0.748004	1.021103	0.089708
CYSLTR2	1.097009	0.946818	1.271024	0.217761
ARL11	0.913671	0.649647	1.284997	0.603852
SETBP1	1.157973	1.002817	1.337135	0.045686
PSTPIP2	1.31728	1.005884	1.725076	0.045221
SPC25	0.947026	0.69737	1.286058	0.727384
PDK1	0.776458	0.570807	1.056201	0.107034
PDE3B	0.686689	0.560471	0.841331	0.000287
TCF7L1	1.350407	0.675718	2.698759	0.395118
TGOLN2	0.856274	0.541664	1.353617	0.506633
KCNK13	1.049522	0.76346	1.442769	0.765938
UHMK1	1.031306	0.69356	1.533527	0.878962
ATG10	0.821645	0.488335	1.382454	0.459299
SPOCK1	1.239185	1.018917	1.507071	0.031742
FAM151B	0.997529	0.797413	1.247865	0.98272
CWF19L2	1.006345	0.643454	1.573897	0.977886

JMY	0.899667	0.740096	1.093642	0.288516
HOMER1	0.908505	0.657541	1.255254	0.56075
XRCC4	1.061321	0.710933	1.584401	0.770964
ZNF547	0.959946	0.648751	1.420415	0.83798
ZNF773	0.864933	0.533914	1.401179	0.555509
ZNF776	0.906866	0.631057	1.303222	0.597205
ZNF256	1.129458	0.881106	1.447811	0.336611
SUV39H2	0.725666	0.496217	1.06121	0.098208
DCLRE1C	0.94549	0.586028	1.525441	0.818348
OLAH	1.230702	1.03184	1.467889	0.020969
RPP38	1.12648	0.596641	2.126837	0.713401
NMT2	0.574433	0.425163	0.776109	0.000305
ZNF837	0.877746	0.658459	1.170062	0.373949
USP12	0.71203	0.473024	1.0718	0.103597
CCDC50	1.092982	0.933855	1.279223	0.268075
CAMK4	0.826466	0.619624	1.102355	0.19467
TRIM36	0.715859	0.526227	0.973828	0.033265
ZFP36L2	1.288724	0.923425	1.798533	0.135826
PAN3	0.80997	0.5526	1.187209	0.279995
PFKM	1.232317	0.928881	1.634876	0.147497
TMEM123	0.895839	0.61048	1.314584	0.574026
IGSF10	1.136851	1.020099	1.266966	0.020347
SPEF2	0.793615	0.577192	1.091188	0.154789
MBNL1	0.877098	0.546228	1.408389	0.587324
GPD1L	1.428791	0.971454	2.10143	0.069858
GJA1	0.979944	0.842558	1.139733	0.792649
SLC30A6	0.759711	0.466237	1.237912	0.269938
PELO	1.669433	1.077214	2.587236	0.021865
RASGRP3	1.033695	0.871193	1.226508	0.704114
SAR1B	0.686371	0.393736	1.196499	0.184423
CATSPER3	0.677633	0.366807	1.251848	0.213987
GPR180	0.811041	0.518628	1.268323	0.358595
TCTEX1D1	1.047695	0.928585	1.182083	0.449248
ANKRD22	0.910249	0.736901	1.124376	0.382986
FARP1	1.098814	0.628588	1.9208	0.740883
IFIT5	1.446841	1.115676	1.876304	0.005347
PANK1	1.143668	0.86423	1.51346	0.347658
PRDM8	1.02537	0.82883	1.268517	0.817501
BMP3	1.032093	0.857548	1.242165	0.738239
HHEX	0.929753	0.628968	1.37438	0.71492
UTRN	0.948465	0.703298	1.279095	0.728774
PTPRK	0.848556	0.470151	1.531525	0.585695
GGPS1	1.444445	0.83151	2.509194	0.191856
ZNF117	0.722072	0.550916	0.946402	0.018322
MARVELD:	1.217645	0.907574	1.633651	0.189109
RAD17	0.638137	0.389362	1.045862	0.074738
MED21	0.545759	0.377227	0.789585	0.00131
PLOD2	0.847378	0.599812	1.197125	0.347533
STK32B	0.949801	0.823079	1.096033	0.480865
CPB1	0.950099	0.461291	1.956873	0.889563
MR1	1.616697	1.084496	2.410067	0.018366
SRP19	0.466668	0.275432	0.790682	0.004612
CENPH	1.145911	0.82174	1.597965	0.422107
CDYL	0.929108	0.497787	1.734159	0.817364
CARHSP1	0.961141	0.569732	1.621451	0.881915
BANK1	1.121548	0.92893	1.354107	0.232811
TXNDC11	1.894377	1.083539	3.311986	0.024997
DAB2	1.174231	0.916923	1.503744	0.203124

ACMSD	0.608838	0.337071	1.09972	0.099998
BCL2L11	1.531096	1.187002	1.974939	0.001038
ANAPC1	0.850259	0.580351	1.245696	0.405135
CAST	1.174202	0.944483	1.459794	0.148247
SCOC	0.886787	0.601182	1.308075	0.544627
CLGN	1.006531	0.863038	1.173883	0.933886
CETN3	0.846295	0.572179	1.251734	0.403345
SMARCA5	1.039954	0.694558	1.557111	0.849136
SYCP2L	0.851198	0.680297	1.065033	0.158847
BMP6	1.018111	0.797089	1.300419	0.885704
RGPD3	0.251074	0.05539	1.138072	0.073095
RASSF3	1.290291	0.951706	1.749333	0.100753
HNRNPU	0.949584	0.570367	1.580927	0.842334
RANBP2	0.995427	0.699987	1.415562	0.979647
AHCTF1	0.82046	0.528381	1.273995	0.378102
MERTK	0.922857	0.751963	1.13259	0.442283
TMEM87B	1.300105	0.805972	2.097184	0.282028
NR4A2	0.940558	0.813516	1.087441	0.407828
RBMS1	0.860305	0.569008	1.300729	0.475603
SCN3A	1.137326	0.958109	1.350067	0.141337
CD96	0.919132	0.827057	1.021459	0.117412
ASAP1	1.072806	0.830645	1.385566	0.590294
FAM81B	1.535689	1.060226	2.224376	0.023248
INO80C	0.699508	0.457641	1.069203	0.098766
LPCAT1	1.457521	1.051407	2.020501	0.023772
NMRAL1	1.442495	0.876004	2.375323	0.149942
ING1	0.831526	0.434953	1.589675	0.576841
ADPRHL1	0.838798	0.561221	1.253664	0.39125
CMTM7	1.018457	0.765522	1.354964	0.900083
FBXL2	1.077051	0.66482	1.744892	0.763005
UBP1	1.375533	0.776233	2.437528	0.274728
RMND5A	1.195136	0.774461	1.844314	0.42064
CD8A	1.033501	0.84398	1.265582	0.749866
RPIA	1.167693	0.732376	1.861756	0.514815
PTPRD	0.932561	0.753758	1.153779	0.520301
CNKSR3	1.107492	0.758525	1.617007	0.597
GTF2E1	1.033192	0.677751	1.575043	0.879352
CFDP1	0.725147	0.39205	1.34125	0.305718
ZDHHC7	1.556865	1.092106	2.219406	0.014405
JAZF1	0.990807	0.798614	1.229253	0.933102
CMIP	1.255535	0.795422	1.981801	0.328497
PID1	1.12183	0.931866	1.350518	0.224565
TRIP12	0.855112	0.546055	1.33909	0.493981
FBXO36	0.748645	0.443683	1.263222	0.27812
CEBPG	1.138403	0.749768	1.728484	0.542947
KCTD15	0.73606	0.615258	0.880581	0.000807
ZNF599	0.667444	0.451768	0.986086	0.042323
MCOLN2	1.324591	1.081335	1.62257	0.006622
LGI4	1.355407	0.734898	2.499841	0.330204
DDAH1	1.13566	0.928089	1.389655	0.216719
CHD1	0.590552	0.398259	0.87569	0.008783
DGKE	1.059401	0.834325	1.345197	0.635831
HS2ST1	0.975811	0.676214	1.408145	0.895889
MSI2	0.955807	0.808498	1.129957	0.596618
HS3ST3A1	0.406955	0.203803	0.812609	0.010832
GDPD1	1.131202	0.897244	1.426163	0.29704
NUS1	1.034158	0.617586	1.731713	0.89839
PPP2R5E	0.819642	0.491223	1.367634	0.44642

GRAP	1.670391	1.173453	2.377774	0.004402
SLC5A10	1.225553	0.896735	1.674945	0.201916
AK5	1.031108	0.880142	1.207968	0.704483
CABYR	1.127733	0.793746	1.602254	0.502311
IMPACT	1.049994	0.758935	1.452677	0.768343
TBCEL	0.892597	0.566394	1.40667	0.624418
JPH3	0.473472	0.23152	0.968276	0.040532
ANKH	0.835861	0.635936	1.098638	0.198621
UBASH3B	1.094155	0.90202	1.327216	0.361077
ROBO4	1.156983	0.996288	1.343596	0.055977
ROBO3	1.32738	1.118444	1.575348	0.001191
TBRG1	1.066475	0.648677	1.753367	0.799718
NRGN	1.278269	1.110566	1.471296	0.000623
GPR15	1.117139	0.869947	1.43457	0.385338
ABI3BP	1.02608	0.583754	1.803571	0.928711
ANGPT1	1.112468	0.978707	1.264511	0.102961
PITPNC1	1.066778	0.799662	1.423121	0.660223
CC2D1B	1.319378	0.824598	2.111039	0.247785
PRKCA	0.99151	0.84273	1.166555	0.918131
LRRK1	1.35165	1.005254	1.817409	0.04608
GAL3ST2	1.408851	1.027932	1.930927	0.033068
ABCA5	1.181786	0.950997	1.468583	0.131883
ENPP3	0.878203	0.695738	1.108521	0.27441
UCHL1	1.053473	0.862281	1.287058	0.610179
MIA3	0.65385	0.428456	0.997815	0.048826
DISP1	0.958355	0.566336	1.621731	0.874071
TNIK	0.924916	0.756708	1.130515	0.445983
TDH	1.971053	1.025767	3.787462	0.04172
FAM167A	0.819375	0.587553	1.142663	0.240385
NEIL2	0.87426	0.573266	1.333291	0.532582
OBSCN	0.998041	0.823905	1.208982	0.984008
LONRF1	0.641664	0.412592	0.997916	0.048928
TRIM11	1.433216	0.859317	2.390395	0.167883
ENAH	0.971353	0.793168	1.189567	0.778632
SH3RF1	0.836922	0.646089	1.08412	0.177574
GBP5	1.021801	0.838149	1.245693	0.831055
BUB3	1.326144	0.718549	2.447514	0.366617
MMP21	0.746953	0.430412	1.29629	0.299593
LY96	1.152657	1.004448	1.322734	0.043055
TMSB4Y	0.942016	0.785565	1.129626	0.519176
CXADR	0.946957	0.61695	1.453484	0.803119
BTG3	1.111875	0.903456	1.368375	0.31667
NCAM2	1.133709	0.780701	1.646335	0.50969
L3MBTL4	1.155104	0.993434	1.343085	0.060885
RABGEF1	0.659383	0.397396	1.094085	0.106978
MRPL39	1.579138	0.986971	2.526597	0.056745
GABPA	0.837716	0.542118	1.294491	0.425166
ADAMTS1	0.816211	0.677059	0.983962	0.033213
TSEN2	0.804703	0.54711	1.183576	0.269686
SLFN13	0.973945	0.814783	1.164198	0.77182
XPC	1.254885	0.761976	2.066649	0.3724
FGD5	1.11476	0.954871	1.30142	0.169023
FLCN	0.874541	0.569721	1.342451	0.539812
DPH3	0.937221	0.617984	1.421368	0.760261
OXNAD1	0.894708	0.597553	1.339632	0.589045
PLCL2	1.21821	0.960728	1.544699	0.103257
CXXC1	1.119334	0.757041	1.655007	0.572075
SKA1	1.019248	0.749101	1.386818	0.903421

PPP4R1	0.893562	0.544426	1.466597	0.656199
CCDC144E	0.925905	0.768617	1.115379	0.417688
MPPE1	1.231058	0.79881	1.897201	0.346187
USP43	1.19513	0.898451	1.589776	0.220794
RAB6B	0.786286	0.615285	1.004812	0.054657
EME1	0.8728	0.61063	1.24753	0.455381
EPHB1	1.166337	0.841098	1.617342	0.356273
ACSS1	1.116822	0.755051	1.651929	0.580134
ANKRD40	1.415071	0.845826	2.367421	0.186084
ZNF18	0.87881	0.495104	1.559889	0.659017
VOPP1	1.380261	1.038121	1.835163	0.026598
APOOL	0.560284	0.305659	1.027021	0.060968
CYP2U1	1.202404	0.982131	1.472079	0.074209
FBXL18	0.918251	0.533624	1.580111	0.758114
KLF10	1.154774	0.93143	1.431674	0.189445
PTPRN2	1.258221	1.048712	1.509584	0.013444
AZIN1	1.067225	0.676425	1.683805	0.779748
ATP6V1C1	0.953389	0.601859	1.510238	0.83884
OTUD6B	0.693928	0.433743	1.110187	0.127512
GTF3C6	1.52625	0.868849	2.681063	0.141321
TTC39B	1.211037	0.942495	1.556093	0.134405
AGPAT5	0.814856	0.623529	1.064891	0.133744
MMS19	1.055467	0.652335	1.707728	0.82596
PI4K2A	1.353674	1.077929	1.699958	0.009169
MARVELD	1.099962	0.911041	1.328058	0.321711
ZFYVE27	1.181641	0.72071	1.937362	0.508206
GOLGA7B	1.3496	0.783633	2.324328	0.279732
SLC25A28	1.760672	1.187128	2.611315	0.004909
HSPA13	0.86658	0.591503	1.269581	0.46237
SAMSN1	1.087426	0.822757	1.437235	0.555874
USP25	1.370412	0.894277	2.100054	0.147927
ZCCHC10	0.712794	0.458522	1.10807	0.132562
MOV10	1.07296	0.778106	1.479546	0.667518
RHOC	1.423431	1.12568	1.799941	0.003191
PPM1J	1.317879	1.036982	1.674866	0.024015
DBI	1.034916	0.772118	1.38716	0.818382
SLC16A1	1.095301	0.901521	1.330734	0.359485
HEATR3	0.972673	0.624401	1.515201	0.902492
TRIM74	0.731836	0.535112	1.00088	0.050647
OXA1L	1.858033	1.02622	3.364083	0.040814
SLC7A7	1.111006	0.987021	1.250565	0.081232
LARP1	0.551153	0.334099	0.909222	0.01967
CNOT8	0.693018	0.453198	1.059744	0.09061
LRGUK	0.803993	0.558846	1.156678	0.239745
MIER3	0.850414	0.59141	1.222846	0.381925
NUP205	0.901133	0.598059	1.357795	0.6187
ZKSCAN2	1.063714	0.663246	1.705983	0.797736
PIK3AP1	1.288204	0.970445	1.710009	0.07971
RBM45	1.285061	0.751001	2.198907	0.36012
TTN	0.732729	0.533965	1.005482	0.054092
VSIG4	1.022081	0.910937	1.146787	0.710007
PDIA4	0.722004	0.516258	1.009746	0.057003
OTOA	1.795935	1.279386	2.521041	0.000715
KCTD18	0.800627	0.412195	1.555096	0.511532
FAM126B	0.782007	0.472095	1.295363	0.339611
FZD7	0.913805	0.681426	1.22543	0.547124
RNF20	1.03472	0.660386	1.621241	0.881581
PPARGC1E	1.289645	0.922126	1.803641	0.137211

ELMO1	0.964532	0.66805	1.392595	0.847187
SLC26A2	0.709042	0.52579	0.956164	0.02421
LSM11	0.868666	0.564923	1.335721	0.521288
MED7	0.787237	0.486217	1.274621	0.330543
RRAGA	2.601293	1.380791	4.900618	0.003092
RASA2	1.035375	0.684315	1.566533	0.869307
RMND1	0.761143	0.440505	1.315169	0.328008
SLA	1.319015	1.065359	1.633066	0.011054
TMBIM4	0.768228	0.514716	1.146602	0.196901
VBP1	1.039509	0.66793	1.617804	0.863674
RAB39B	1.191466	0.911302	1.557761	0.200242
CLIC2	1.239159	1.064618	1.442315	0.005634
AFF2	0.783107	0.678925	0.903277	0.000789
GRIP1	0.79686	0.597778	1.062244	0.121561
VPS37A	0.521022	0.329856	0.822977	0.005185
PSD3	1.062601	0.885918	1.274522	0.512834
GNA14	1.139333	0.864985	1.500698	0.353385
FAM161B	1.011723	0.570266	1.794924	0.968216
GNAQ	0.920932	0.589477	1.438757	0.71746
GPR61	0.181815	0.038306	0.862963	0.031917
ADK	1.481766	0.935859	2.346112	0.093499
BATF	2.333146	1.577199	3.451417	2.23E-05
DCK	1.071625	0.795913	1.442847	0.648513
ADAMTS3	0.829761	0.668017	1.030668	0.09162
ALX3	0.551561	0.114511	2.656671	0.458201
DPY19L4	0.703242	0.469963	1.052314	0.086897
ART3	0.928988	0.459983	1.876199	0.837268
WHAMM	1.498494	0.848484	2.646466	0.163387
CXCL13	1.086306	0.662127	1.782227	0.743118
N6AMT1	0.942668	0.651186	1.364622	0.754417
RWDD2B	1.678424	1.054023	2.672718	0.029138
USP16	0.648505	0.406091	1.035628	0.069773
CCT8	1.267876	0.81301	1.977231	0.295157
BACH1	0.811608	0.567379	1.160965	0.253112
TSPAN7	0.979508	0.868638	1.104529	0.735499
TIAM1	1.022142	0.843772	1.238219	0.822892
RPGR	0.584434	0.4137	0.82563	0.002312
PCGF6	0.841907	0.463936	1.527813	0.571405
ANKRD9	1.310511	1.024714	1.676018	0.031203
SFXN2	0.989414	0.692761	1.413099	0.953332
FUT6	1.32497	0.947736	1.852358	0.09977
TDRD9	0.797038	0.698961	0.908876	0.000709
FGF18	0.99012	0.667051	1.469659	0.960701
PCDH1	0.90977	0.577142	1.434104	0.683825
UQCRB	1.165031	0.693481	1.957226	0.563881
PTDSS1	1.206313	0.824701	1.764508	0.333709
RPL30	0.932472	0.601753	1.444952	0.754383
FAM122C	1.218331	0.69418	2.138251	0.491393
SUPV3L1	0.961121	0.569301	1.62261	0.882016
FAM122B	0.898613	0.618345	1.305913	0.575124
EEF1A1	1.017429	0.744129	1.391106	0.91379
FBXO43	1.06504	0.782683	1.449258	0.688472
HK1	1.44558	1.094782	1.908783	0.009363
TYSND1	1.477396	0.965772	2.260056	0.071957
PHF6	1.014844	0.709689	1.451212	0.935642
CD109	1.17107	1.04641	1.310581	0.00596
NODAL	0.843807	0.539801	1.319024	0.456198
PRG3	0.998023	0.81368	1.224129	0.984843

UBE2L6	1.547431	1.047846	2.285207	0.028169
ZDHHC5	1.654845	0.902304	3.035021	0.103578
MED19	0.87022	0.419331	1.805931	0.709015
ZFAND3	1.63877	0.811612	3.30893	0.16828
NPTN	1.354667	0.90375	2.030564	0.141586
SAMD8	1.232076	0.858831	1.767532	0.257023
RAB11FIP1	1.083786	0.856675	1.371106	0.502467
GLYATL2	1.044734	0.78554	1.389451	0.763566
UTP14A	1.3755	0.822646	2.299895	0.22414
AIFM1	2.494999	1.231583	5.054488	0.011141
MAPK13	0.978865	0.709849	1.349833	0.896338
BAG4	1.163442	0.711713	1.901888	0.546031
MS4A1	0.955881	0.774749	1.179361	0.673798
ATAD2	1.087788	0.752639	1.57218	0.654313
FBXO32	1.189271	0.876342	1.613941	0.265851
NSMCE2	0.914136	0.499345	1.673479	0.771056
ZNF689	1.334632	0.823449	2.163149	0.241377
PRR14	0.967908	0.688987	1.359744	0.850813
FBRS	1.135465	0.7791	1.654832	0.508566
FRRS1	1.220958	0.903672	1.649647	0.193513
PHKG2	0.797627	0.520545	1.222197	0.299058
SASS6	0.970819	0.603845	1.560814	0.902704
ITGAD	1.242171	0.935636	1.649134	0.133655
VPS8	0.898288	0.569292	1.41741	0.644837
GALK2	0.564354	0.366544	0.868914	0.009374
B3GNT7	1.000839	0.850702	1.177473	0.991932
MPV17L	1.404574	1.160614	1.699814	0.000483
BUB1B	0.980811	0.740347	1.299377	0.892597
PDE6D	1.237374	0.578047	2.648734	0.583352
EIF4A2	1.064058	0.638042	1.774522	0.811925
BRPF1	0.963936	0.6794	1.367638	0.836953
RPUSD3	0.814427	0.565408	1.17312	0.270269
TATDN2	0.934753	0.54111	1.61476	0.808847
GHRL	0.802453	0.59487	1.082471	0.149565
SEC13	0.81351	0.484347	1.366371	0.435325
EXOG	1.110025	0.706005	1.745248	0.651191
NTAN1	1.227923	0.792961	1.901472	0.357445
ZFYVE9	0.876207	0.710667	1.080307	0.216106
SMG1	0.782943	0.520418	1.1779	0.240298
FCHO2	0.767421	0.636435	0.925365	0.005566
RBPMS	1.097327	0.906923	1.327704	0.33948
TIMP4	1.276251	1.052999	1.546837	0.012906
CPT2	1.748334	0.833461	3.667446	0.139404
NECAP2	1.921067	1.13686	3.246221	0.01472
LRP8	1.349314	0.918004	1.98327	0.12736
PAXIP1	0.714865	0.431775	1.183562	0.191949
SSBP3	0.79799	0.562645	1.131774	0.205629
CLDN12	0.689935	0.527795	0.901886	0.006618
MMP14	0.859612	0.758333	0.974418	0.018023
FZD1	1.282442	0.99118	1.659291	0.058415
GATAD1	0.773757	0.477975	1.252576	0.296647
SUSD3	1.232361	1.040198	1.460024	0.015708
TMED6	0.877558	0.51441	1.49707	0.631738
DHRS4	1.862381	1.120266	3.096108	0.016491
DDX19B	1.163829	0.586053	2.311218	0.664705
ST3GAL2	1.209894	0.813323	1.799831	0.347079
IL34	0.913186	0.540862	1.541815	0.733983
DHRS1	1.397097	0.850701	2.294439	0.186455

CACNA1D	0.515387	0.179597	1.479003	0.217823
KIT	0.907556	0.798909	1.030978	0.135956
AASDH	0.81587	0.522431	1.274129	0.370914
ZNF19	0.631592	0.244541	1.631254	0.342537
CACNA2D	1.171954	0.997591	1.376792	0.05353
RNF111	0.737532	0.418182	1.30076	0.292958
CCNB2	0.949318	0.723668	1.245329	0.707219
FAM81A	1.412192	1.089135	1.831073	0.009208
MYO1E	1.28019	1.021298	1.60471	0.032131
APPL1	0.879918	0.594622	1.302098	0.522317
AFAP1L1	0.719666	0.509305	1.016915	0.062199
TSC22D3	1.224578	0.985562	1.521559	0.067446
DYRK1A	1.168663	0.661624	2.064273	0.591302
KCNJ15	1.024267	0.751404	1.396217	0.879425
ERG	0.898616	0.776259	1.040259	0.152303
ETS2	1.703472	1.283831	2.26028	0.000223
TSPAN18	1.068597	0.840573	1.358477	0.58797
LCA5L	0.666949	0.396292	1.122457	0.127254
SLC35B2	1.078795	0.670033	1.736926	0.754952
TMEM164	1.309547	0.851133	2.014859	0.219918
MX1	1.303551	1.124712	1.510826	0.00043
C2CD2	0.843272	0.647465	1.098294	0.206056
SLC38A10	1.483901	1.029742	2.138365	0.034244
ZNF618	1.041414	0.802627	1.351241	0.760078
SVOPL	0.735389	0.512783	1.054633	0.09476
SNX22	0.747747	0.527203	1.060551	0.103046
UBN2	0.538844	0.373859	0.776636	0.000915
BRAF	0.499436	0.296788	0.840452	0.008935
PSMG3	1.496479	1.026571	2.181485	0.036052
WDR19	0.587875	0.429231	0.805155	0.000931
SLC37A3	0.991057	0.770354	1.274991	0.944283
AP3S2	1.205742	0.693309	2.09692	0.507548
FMNL2	1.171224	0.937465	1.463271	0.16409
SPPL3	0.543789	0.253834	1.164963	0.117076
RAB28	0.468167	0.285143	0.76867	0.0027
TNFRSF14	1.029653	0.830703	1.27625	0.789658
PANK4	1.59553	0.956624	2.661147	0.073444
MEGF11	1.033113	0.679486	1.570778	0.878883
PEX10	1.251755	0.779286	2.010676	0.353077
RER1	2.272596	1.098964	4.699603	0.026792
RADIL	0.925313	0.670406	1.277144	0.636846
SKI	1.388739	0.943815	2.043404	0.09561
WIPI2	0.879518	0.489339	1.580808	0.667808
LDLRAP1	1.572822	1.159777	2.132968	0.003573
AGAP1	1.052384	0.832779	1.329899	0.66896
KRTCAP3	0.803856	0.503428	1.283567	0.360495
PAFAH2	0.680066	0.419112	1.1035	0.11848
MRPL17	1.589008	0.877301	2.878086	0.126503
DUSP2	1.140387	0.929287	1.399442	0.208469
UBXN11	1.2946	1.055749	1.587488	0.01309
PTPDC1	0.949766	0.714999	1.261617	0.72201
GALNT14	1.010457	0.867595	1.176845	0.893592
NCK1	0.694636	0.425379	1.134325	0.145327
HPD	1.134304	0.837077	1.537069	0.416301
RHPN1	1.175964	1.011368	1.367347	0.035126
TPRG1L	1.079338	0.650672	1.790414	0.76748
LRRC43	0.732613	0.52713	1.018196	0.06394
XKR8	1.266011	0.769649	2.082487	0.352948

CNNM4	0.987922	0.656675	1.486261	0.953498
EYA3	1.189467	0.729994	1.938143	0.486095
DZIP1L	0.900903	0.660377	1.229036	0.510187
TMSB15A	1.047504	0.805897	1.361544	0.728662
FANCC	1.209677	0.769785	1.900946	0.409136
MRAS	0.868891	0.712862	1.059071	0.164029
WASF2	1.982343	1.190511	3.30084	0.008532
ABHD3	0.67373	0.457026	0.993187	0.046099
FAIM	0.634303	0.455641	0.883022	0.006997
CLSTN2	1.082894	0.866035	1.354055	0.484882
COLEC12	0.772693	0.555857	1.074116	0.124905
RNF207	1.232459	0.928662	1.63564	0.147777
CUL4B	0.736033	0.480658	1.12709	0.158635
GPR153	0.818406	0.698075	0.959478	0.01352
SLC13A3	1.360745	0.951817	1.94536	0.091188
GPRASP2	0.895352	0.62942	1.27364	0.538714
RHBDL2	0.903156	0.573576	1.422115	0.660129
AUTS2	0.711418	0.573909	0.881875	0.00189
SHROOM4	1.010765	0.847907	1.204904	0.904917
CDC25C	0.851233	0.627046	1.155572	0.301698
MITD1	0.823971	0.490501	1.384151	0.464409
EIF5B	1.101713	0.735764	1.649675	0.638159
RIBC1	1.03272	0.573262	1.860424	0.914625
TSPAN33	1.031106	0.814428	1.305431	0.799107
AHCYL2	0.761811	0.516688	1.123225	0.169641
B4GALT5	0.984542	0.708227	1.368661	0.926149
CD1D	1.162645	1.033655	1.307733	0.012017
CD1A	1.059706	0.817054	1.374421	0.662047
SPATA2	0.895088	0.556061	1.440816	0.648157
CD1C	1.080425	0.952387	1.225677	0.229381
CD1E	1.086923	0.936656	1.261298	0.272225
HMHB1	1.21429	0.928421	1.588182	0.156286
NCF1	1.122208	0.998608	1.261106	0.052798
TSR2	3.229586	1.616258	6.453319	0.000902
PPP1R9A	1.135415	0.942599	1.367673	0.181084
ZC3H18	1.105351	0.580437	2.104968	0.760538
ZFAND2B	0.943247	0.666123	1.335662	0.742
GDPD5	0.970542	0.792838	1.188076	0.771986
DYNC1I1	1.850111	1.184274	2.890304	0.006871
PFKFB1	1.19193	0.538946	2.636068	0.664611
ALAS2	0.928779	0.843465	1.022723	0.132861
TMED4	0.635829	0.352022	1.148446	0.133321
PPP1R15B	1.003437	0.62337	1.615228	0.98873
COPG2	0.788498	0.583329	1.065831	0.122262
ZSCAN12	0.610753	0.439388	0.84895	0.00334
TAGLN2	1.844002	1.372005	2.478376	4.98E-05
ELK4	0.806102	0.517095	1.256635	0.341338
SLC45A3	0.816847	0.684747	0.974431	0.024592
DUSP23	1.571532	1.105984	2.233045	0.01167
RNF166	1.258636	0.930991	1.701589	0.134868
NBL1	0.898955	0.754733	1.070736	0.232507
HTR6	1.282961	0.781276	2.106798	0.324814
F11R	1.070835	0.763235	1.502403	0.692018
USF1	1.648468	1.05581	2.573804	0.027887
SPATA2L	1.422781	1.048606	1.930473	0.023525
NIT1	1.730826	0.953479	3.141923	0.07133
DEDD	3.428392	1.576949	7.45355	0.001874
ZNF276	1.187517	0.782	1.803319	0.420064

NPM2	0.960404	0.807412	1.142386	0.648144
FGF17	0.959946	0.78202	1.178355	0.695921
CDA	1.090789	0.984578	1.208457	0.09639
PINK1	1.178827	0.772905	1.797935	0.444932
B4GALT3	1.172067	0.689926	1.991142	0.557069
ADAMTS4	1.0621	0.742906	1.518438	0.741124
FAM160B2	0.993605	0.705979	1.398414	0.970652
NDUFS2	2.517679	1.336686	4.742107	0.004259
SLC5A11	1.332138	0.849285	2.089511	0.211781
FCER1G	1.135719	0.994975	1.296371	0.059384
APOA2	1.101526	0.930897	1.30343	0.260133
TOMM40L	2.060431	1.392606	3.048512	0.000298
MPZ	0.940942	0.637527	1.388759	0.759233
CACHD1	1.058408	0.886791	1.263238	0.529415
CDC42SE2	0.68782	0.445705	1.061456	0.090925
RAPGEF6	0.892377	0.572882	1.390053	0.614583
EPB41	0.667119	0.452468	0.9836	0.041009
ALG8	0.749202	0.411075	1.365451	0.345753
FBXW5	1.507638	1.087189	2.090688	0.013851
SYNJ1	1.266082	0.809082	1.981212	0.301761
IFNAR2	0.923983	0.567151	1.505324	0.750872
MRPL10	1.558393	0.86101	2.820628	0.142756
IFNGR2	1.096193	0.833852	1.44107	0.510496
GART	0.989375	0.642363	1.523845	0.961339
SON	0.991154	0.632305	1.553659	0.969094
DONSON	1.193542	0.669113	2.129004	0.549047
SV2A	1.102728	0.939866	1.293812	0.2304
STC1	0.557314	0.140631	2.208611	0.405331
CSRP1	2.357028	1.58746	3.499666	2.12E-05
C1QC	1.06132	0.955938	1.17832	0.264677
RCAN1	1.375242	1.107156	1.708242	0.003976
UBE2Z	1.100931	0.562871	2.153332	0.778767
SNF8	0.985034	0.488931	1.984516	0.966345
CCDC24	1.177281	0.845502	1.63925	0.333888
RUNX1	0.933211	0.684082	1.273068	0.662652
IGF2BP1	1.65899	0.000867	3173.645	0.895539
CBR1	2.207665	1.649349	2.954976	1.02E-07
CBR3	1.070352	0.713663	1.605314	0.742344
MORC3	1.047633	0.631308	1.738509	0.857098
CHAF1B	1.511768	0.993951	2.299351	0.053408
HLCS	1.273485	0.765483	2.118617	0.351904
SCUBE1	1.077368	0.941314	1.233087	0.279291
ARHGAP27	1.540102	1.136608	2.086835	0.005335
ADPGK	1.238797	0.725546	2.115121	0.432721
PTMS	1.086946	0.931514	1.268314	0.289643
PADI4	1.053465	0.904067	1.22755	0.504459
ADIPOR1	1.107136	0.672926	1.821523	0.688683
CYB5R1	1.528166	1.003617	2.326876	0.048065
PSMD4	2.728462	1.427463	5.215202	0.002392
ATP13A2	1.7189	1.300581	2.271767	0.000141
PSMB4	2.078865	1.13656	3.802419	0.017527
IRX6	0.870029	0.515905	1.467226	0.601557
BTG2	1.15073	0.917455	1.443319	0.224506
HK2	0.68067	0.523078	0.885742	0.004197
C1R	1.217877	0.945382	1.568916	0.127179
ALDH4A1	0.933766	0.71458	1.220185	0.615631
STARD9	0.939386	0.789253	1.118076	0.48157
THEM4	1.185843	0.926197	1.518276	0.176408

UBR1	0.666549	0.428465	1.036928	0.071998
AMFR	1.088859	0.64521	1.837563	0.749849
MED8	1.732519	1.011076	2.968741	0.045495
RGL4	1.030789	0.781218	1.360089	0.830242
RSPRY1	0.711702	0.40721	1.243878	0.232527
CCDC17	0.901806	0.607413	1.338882	0.608232
GPBP1L1	0.726473	0.390081	1.352957	0.313846
NAE1	1.070185	0.696543	1.644256	0.756888
TMEM69	1.589478	0.831704	3.037668	0.160821
ACE	1.120274	0.882895	1.421477	0.349885
SPON2	1.186643	0.947999	1.485361	0.135225
CHCHD6	1.092212	0.738229	1.615932	0.65896
CTBP1	1.479309	0.826798	2.646783	0.187101
LRRC36	1.143077	0.72868	1.793139	0.56049
TPPP3	1.107499	0.968573	1.266353	0.135427
ZDHHC1	0.889466	0.640445	1.235311	0.484575
ATP6V0D1	1.411968	1.048615	1.901225	0.023045
AGRP	1.272024	0.929628	1.740528	0.132613
ZFYVE28	0.87606	0.65929	1.164103	0.361605
FAM131B	1.164446	0.86111	1.574636	0.322766
RGS12	0.966336	0.725086	1.287854	0.815237
PSKH1	1.075456	0.714061	1.619757	0.72773
ZYX	1.258518	0.957986	1.653331	0.098606
ABR	1.191617	0.936856	1.515655	0.153153
CCDC117	1.042085	0.652683	1.663808	0.862901
ZNF230	0.959838	0.624113	1.476159	0.851937
CCDC107	1.342247	0.828646	2.17418	0.231643
ZNF222	0.696545	0.491387	0.987359	0.042211
NPR2	1.188318	0.918587	1.537251	0.189015
ZNF221	0.775383	0.601959	0.99877	0.048896
ZNF233	0.663947	0.469508	0.938911	0.020531
ZNF235	0.566709	0.372922	0.861196	0.007818
GNE	0.707661	0.377187	1.32768	0.281435
TNFRSF13	1.049647	0.793495	1.388488	0.734264
PTGIR	1.045512	0.901805	1.212118	0.555228
CALM3	1.344028	0.907203	1.991189	0.14039
DFFA	1.468381	0.844479	2.553223	0.17349
CCDC28B	1.375899	0.856488	2.210302	0.187026
IQCC	1.181015	0.688357	2.026269	0.5458
BSDC1	0.852763	0.521101	1.395515	0.52621
ZBTB8A	0.902774	0.728326	1.119006	0.350498
ATAD3B	1.249307	0.870647	1.792652	0.226996
SSU72	1.751469	0.826199	3.712962	0.143755
UBE2J2	1.692753	0.790215	3.626116	0.175672
ZNF362	1.15112	0.71045	1.865123	0.567612
FNDC5	0.736017	0.44064	1.229397	0.241615
CPAMD8	1.141389	0.885043	1.471984	0.308203
NR2F6	1.10248	0.748283	1.624334	0.621715
ANKLE1	1.184801	0.910215	1.542221	0.207448
CCDC58	1.03955	0.665066	1.624896	0.864851
KALRN	1.161321	0.811135	1.662691	0.41405
CILP2	0.823357	0.674102	1.005658	0.056822
ABCG1	1.175072	0.984163	1.403014	0.074507
TFF3	0.668518	0.453488	0.985508	0.041983
UBASH3A	0.997694	0.787376	1.26419	0.98475
RSPH1	1.218433	0.912022	1.627788	0.181278
SLC37A1	1.27392	0.978808	1.65801	0.071758
PDE9A	1.113795	0.90336	1.373251	0.313111

WDR4	1.205326	0.80194	1.81162	0.369036
NDUFV3	1.541365	0.819795	2.898049	0.179229
PKNOX1	0.883111	0.516337	1.510421	0.649865
U2AF1	0.906457	0.719524	1.141956	0.404587
HSF2BP	1.180923	0.672641	2.073289	0.562527
RRP1B	1.286456	0.854096	1.937683	0.228084
PDXK	1.204183	0.895952	1.618454	0.218078
G6PD	1.748508	1.330829	2.297276	6.02E-05
CSTB	1.70476	1.207676	2.406444	0.002423
RRP1	1.385681	0.879519	2.183137	0.159596
AGPAT3	1.552772	1.156454	2.084909	0.003425
TRAPPC10	1.067	0.679994	1.674262	0.777844
GAB3	1.183721	0.841163	1.665783	0.33323
ICOSLG	1.491131	0.956755	2.323969	0.077614
ZNF66	0.682581	0.531707	0.876266	0.002732
LRRC3	1.209568	0.873967	1.674038	0.251176
ITGB2	1.336954	1.136102	1.573315	0.000472
RALGDS	1.226502	0.801295	1.877344	0.347209
FTCD	0.720566	0.504574	1.029016	0.071446
LSS	1.316187	0.949757	1.823991	0.098878
VAV2	1.262881	1.024379	1.556912	0.028852
MCM3AP	1.130849	0.715491	1.787331	0.59853
PCNT	0.925723	0.641761	1.33533	0.679677
DIP2A	0.94983	0.655037	1.377292	0.786012
S100B	0.917563	0.819281	1.027635	0.136654
PRMT2	1.341121	0.86549	2.078136	0.189019
CLDND2	1.279554	0.968177	1.691072	0.083157
ADAMTS1	0.941244	0.660844	1.340619	0.737204
SLC2A6	1.147489	0.968241	1.359921	0.112391
ZNF761	0.832258	0.570965	1.213127	0.339555
ZNF714	0.939384	0.73255	1.204617	0.622146
GPSM1	1.023382	0.852684	1.228252	0.803939
HIPK4	1.129954	0.726691	1.756998	0.587496
TOR2A	1.512328	1.042158	2.194615	0.029457
ST6GALNA	1.830456	1.183233	2.831708	0.006612
SHKBP1	1.439398	1.05772	1.958806	0.020508
RDH13	1.120021	0.83622	1.500139	0.447097
ZER1	1.31413	0.92357	1.86985	0.128986
ZDHHC12	1.356002	0.974266	1.887309	0.071011
PKN3	1.031193	0.826179	1.287081	0.785931
BRSK1	0.831244	0.64368	1.073462	0.156589
COX6B2	0.622817	0.327774	1.183442	0.148257
TAOK1	0.772109	0.535046	1.114209	0.166953
MED27	2.849304	1.356541	5.984733	0.005687
DEDD2	1.682981	1.134048	2.497623	0.009752
SIK3	1.126095	0.69137	1.834169	0.633276
MPZL3	1.574865	1.185609	2.09192	0.001717
NEK8	1.417081	0.952288	2.108733	0.085632
TLCD1	1.121922	0.779811	1.614122	0.535335
PCSK7	1.369292	0.811435	2.310675	0.239085
SAFB	1.071042	0.56255	2.039161	0.834522
CD3G	0.993469	0.801296	1.231732	0.952366
S100A1	1.269063	0.769275	2.093557	0.35085
CXCR5	1.038068	0.355052	3.035009	0.945583
ZBTB7B	1.237337	1.007605	1.519448	0.042127
FLAD1	1.614585	0.920747	2.831271	0.094559
SHC1	1.439668	0.872268	2.376154	0.154037
VPS11	1.369318	0.823863	2.275903	0.22531

NLRX1	1.191323	0.826866	1.716423	0.347423
ADAR	1.415418	0.8838	2.266813	0.148208
IL6R	1.389751	1.029835	1.875452	0.031381
UBE2Q1	4.057234	1.746314	9.426225	0.001129
CHRNB2	1.034605	0.654435	1.635621	0.884251
CRTC2	1.482494	0.965141	2.27717	0.072187
ANO10	1.166047	0.812714	1.672996	0.404252
FDPS	1.940412	0.927797	4.058215	0.078257
RUSC1	1.72496	1.026987	2.897298	0.03934
FAM189B	1.583146	1.059742	2.365057	0.024878
PAQR6	1.072669	0.851762	1.350867	0.551017
PMF1	1.493428	0.817952	2.726718	0.19164
SLC25A44	1.183003	0.668952	2.092072	0.563422
LMNA	1.185961	1.039723	1.352767	0.011081
CCR5	1.21243	1.046659	1.404455	0.010232
NBEAL2	1.219468	0.955117	1.556983	0.111474
CCDC12	1.642655	0.987422	2.732686	0.055976
PTH1R	1.0086	0.742242	1.370543	0.956351
UBQLN4	1.179405	0.693763	2.005002	0.542204
GPATCH4	1.364499	0.892878	2.085232	0.150912
FCRL3	1.146415	0.914561	1.437046	0.235918
FGFR4	1.239932	0.743762	2.067102	0.409535
NACC1	1.48994	1.002214	2.215016	0.048736
HK3	1.114189	1.016702	1.221024	0.020639
IER2	1.043779	0.80414	1.354832	0.747474
ZNF394	0.799656	0.487004	1.313029	0.376902
CPSF4	0.748035	0.454841	1.230225	0.252753
LY6E	1.33695	1.08299	1.650464	0.006899
VPS28	1.23895	0.851063	1.803621	0.263449
PTGER1	0.823705	0.658591	1.030214	0.089287
RECQL4	1.26026	0.955872	1.661575	0.101006
LRRC14	1.191734	0.77826	1.824878	0.419761
ZNF333	0.611469	0.36087	1.036092	0.067522
PPP1R16A	1.017528	0.751369	1.37797	0.910576
ORAI2	0.802986	0.567231	1.136725	0.215971
ALKBH4	0.794159	0.514584	1.225627	0.297874
SH2B2	1.007539	0.781781	1.29849	0.95373
SQSTM1	1.347353	0.936005	1.939477	0.108685
MGAT4B	1.511199	1.052752	2.169287	0.025176
RPL8	1.532744	1.020743	2.301562	0.039499
MAML1	0.846865	0.520208	1.378641	0.503807
LRWD1	0.789517	0.541392	1.151358	0.219535
FBXL13	0.835999	0.592369	1.179828	0.308144
NAPEPLD	0.787338	0.534302	1.160207	0.226763
SCGB3A1	0.833713	0.595314	1.167582	0.289899
PSMC2	1.17662	0.662915	2.088403	0.578482
USP41	0.752422	0.603262	0.938463	0.011624
YDJC	1.202132	0.85763	1.685017	0.285277
CCDC116	0.804456	0.492304	1.314532	0.385152
DVL3	0.758528	0.505209	1.138866	0.182578
AP2M1	1.874515	1.178018	2.982814	0.00802
ABCF3	1.365559	0.777286	2.399054	0.278516
PCYT1A	1.58238	0.921814	2.716304	0.09598
U2AF1L4	1.05537	0.730117	1.525516	0.774358
BDH1	0.659437	0.489239	0.888842	0.006265
NPHS1	7.964304	0.508829	124.659	0.139266
THAP8	0.964724	0.589194	1.579603	0.886487
COX7A1	0.870867	0.625763	1.211975	0.412265

ZNF382	0.741701	0.563887	0.975586	0.032621
LRRC56	1.052121	0.825701	1.340629	0.681121
PLXDC1	1.108133	0.70862	1.732889	0.652642
PGAP3	0.952665	0.6569	1.381596	0.7982
IKZF3	1.093867	0.872293	1.371724	0.437231
GRIN2C	1.11485	0.826627	1.503567	0.476233
FDXR	1.24248	0.866135	1.782351	0.238271
SAP30BP	1.41959	0.796206	2.531049	0.235017
ACOX1	0.762628	0.475559	1.222983	0.260761
PRPSAP1	0.946176	0.537748	1.664812	0.847811
CYGB	1.045816	0.837667	1.305686	0.692386
ZNF577	0.805874	0.59501	1.091465	0.163171
TMEM143	0.925971	0.590546	1.451915	0.737517
KLHL10	0.865428	0.361106	2.074087	0.745866
HCRT	0.758307	0.444148	1.294679	0.310727
ALDH16A1	1.864324	1.21614	2.857981	0.004267
ITGA5	0.943315	0.699068	1.2729	0.702694
SIGLEC11	1.220126	0.964413	1.543641	0.097319
ZNF385A	1.377575	1.138158	1.667354	0.001007
SIGLEC16	1.189722	0.998249	1.417921	0.052332
MPP3	1.102443	0.917262	1.325008	0.298578
NAGS	1.256728	0.670668	2.354914	0.475731
LSM12	0.728306	0.409532	1.29521	0.280443
ASB16	0.845603	0.543879	1.314713	0.456395
JOSD2	1.37918	0.970291	1.960379	0.073154
SHANK1	0.814388	0.72064	0.920332	0.001
DBF4B	0.831937	0.551276	1.255486	0.380848
PLCD3	1.490341	1.13443	1.957915	0.004158
FMNL3	1.041148	0.810722	1.337065	0.75205
RACGAP1	0.951243	0.65995	1.371109	0.788726
LARP4	0.973355	0.620425	1.527052	0.906437
RAVER1	1.024721	0.744425	1.410555	0.880942
SYCE2	0.721892	0.477153	1.092162	0.122918
SPC24	1.086469	0.809142	1.458849	0.581264
LEMD2	1.403396	0.784781	2.509642	0.25314
ALOX15	0.764333	0.490203	1.191759	0.235672
TREML1	1.097456	0.924317	1.303026	0.288432
ZNF653	1.081034	0.668166	1.749018	0.750934
MED11	1.320506	0.816858	2.134687	0.25659
CXCL16	1.153966	1.008098	1.320939	0.037807
BCL6B	1.019706	0.777741	1.336948	0.887714
ASGR2	1.111	0.975953	1.264735	0.111418
TNFSF13	1.570694	1.184513	2.082782	0.001712
SENP3	1.563903	0.817956	2.990126	0.17628
EIF4A1	0.874364	0.632811	1.208121	0.415724
RPL26	0.912282	0.599208	1.388931	0.668597
POLR3K	1.80776	0.95932	3.406575	0.067026
SNRNP25	1.981602	1.338345	2.934032	0.000637
WDR90	1.100664	0.815719	1.485147	0.530355
JMJD8	1.24809	0.869405	1.791719	0.22962
CCDC78	0.873821	0.635709	1.201121	0.405993
MSLNL	0.581198	0.341198	0.990015	0.045837
SPSB3	1.447459	0.530072	3.952551	0.470584
CCNF	0.902729	0.666575	1.222548	0.508385
TBC1D24	0.583353	0.375073	0.907291	0.01677
AMDHD2	1.074076	0.772032	1.494289	0.671432
NTN3	0.741402	0.469246	1.171406	0.199815
PAQR4	1.019294	0.740929	1.402238	0.906521

FLYWCH2	1.315498	0.896404	1.930531	0.16117
ZNF75A	1.114351	0.758516	1.637116	0.581171
ADCY9	0.934066	0.79259	1.100796	0.415672
CLPB	1.419713	0.873811	2.30666	0.157
NEU3	0.814802	0.586423	1.132121	0.222281
ASRGL1	1.150884	0.946667	1.399156	0.158528
GNG3	1.007832	0.67689	1.500575	0.969358
UBXN1	1.545015	0.93383	2.556216	0.090367
TTC9C	0.88465	0.521803	1.49981	0.649077
TAF6L	1.087969	0.694273	1.704916	0.712968
NXF1	1.376237	0.832229	2.275851	0.213362
STX5	1.163808	0.690123	1.96262	0.569394
SLC25A45	1.339671	0.940108	1.909055	0.10562
RPL29	1.219767	0.796034	1.869055	0.361586
ITIH3	0.882514	0.541192	1.439103	0.616416
SYVN1	1.138458	0.760493	1.70427	0.528733
ZFPL1	0.894759	0.515812	1.552104	0.692336
RPS6KA4	1.523016	1.115012	2.080316	0.008188
LRP5	0.941456	0.778678	1.138261	0.533366
TPCN2	0.948036	0.613035	1.466104	0.810409
PDZK1IP1	1.034674	0.855554	1.251295	0.725252
TAL1	0.994066	0.870903	1.134646	0.929722
CMPK1	0.764148	0.49434	1.181215	0.226087
BEND5	0.949189	0.730819	1.232807	0.695842
ZYG11B	0.953265	0.600949	1.512132	0.838885
MAGOH	1.271064	0.748498	2.158462	0.374669
ACOT11	0.874841	0.71743	1.066789	0.186449
FAM151A	0.772894	0.591738	1.009511	0.058691
PARS2	1.072386	0.71768	1.602401	0.733062
USP24	0.828341	0.547158	1.254023	0.373402
NOL9	1.505171	0.77105	2.938249	0.230865
KLHL21	1.021394	0.712177	1.464869	0.9084
ZSWIM5	0.905252	0.637319	1.285827	0.578264
GMEB1	0.742015	0.392187	1.403885	0.359044
JAK1	1.064842	0.717915	1.57942	0.754774
RAVER2	0.894704	0.687632	1.164132	0.407435
CTRC	1.43025	0.807074	2.534608	0.220286
LZIC	1.340628	0.785772	2.287285	0.282168
RBP7	0.875171	0.711959	1.075797	0.205453
FBLIM1	0.844971	0.582222	1.226296	0.375371
SLC25A34	1.406677	0.871591	2.270265	0.162349
AKR7A3	1.505403	1.012793	2.237613	0.043091
DHRS3	1.022225	0.88141	1.185537	0.771294
MATN1	1.078366	0.635824	1.828924	0.779544
LAPTM5	1.41897	1.084547	1.856513	0.010716
SDC3	1.096399	0.918907	1.308176	0.307072
PEF1	3.057159	1.782945	5.242013	4.87E-05
RBBP4	0.87355	0.536271	1.422955	0.587095
KIAA1522	1.113604	0.874577	1.417959	0.382745
TSSK3	0.83364	0.518245	1.34098	0.453119
TMCO4	1.707451	1.134591	2.569551	0.010304
UBXN10	1.238527	0.939442	1.632831	0.129273
CAMK2N1	0.91747	0.651556	1.29191	0.621828
ALPL	0.924277	0.776981	1.099498	0.373983
WNT4	0.869006	0.569681	1.325606	0.514606
TTLL10	0.886089	0.703324	1.116346	0.304831
SCNN1D	0.903032	0.718857	1.134394	0.380794
MXRA8	1.31359	0.956784	1.803457	0.091648

MEGF6	0.793838	0.657418	0.958567	0.016403
IL23R	0.830452	0.423174	1.629713	0.589126
NFIA	1.004721	0.83266	1.212338	0.960802
OMA1	0.932382	0.616697	1.409665	0.739916
MYSM1	0.672465	0.429263	1.053456	0.08317
TM2D1	0.7184	0.360391	1.432049	0.347387
USP1	1.247969	0.869082	1.792036	0.230179
FUBP1	0.790528	0.51237	1.219695	0.288072
NEXN	1.31321	1.087689	1.585491	0.004592
DNAJB4	1.187948	0.823092	1.714537	0.357575
TYW3	0.998347	0.688028	1.448629	0.993052
B3GALT2	0.762604	0.525396	1.106907	0.153966
FAM102B	0.936285	0.683011	1.28348	0.682467
GBP2	1.200022	0.991906	1.451803	0.060609
ATXN7L2	1.068536	0.710027	1.608066	0.750586
GBP4	1.08367	0.948408	1.238223	0.237502
ZNF326	0.702736	0.444635	1.110661	0.130903
GFI1	0.937762	0.815181	1.078775	0.368619
AGL	1.003315	0.674632	1.492135	0.98696
VCAM1	0.941512	0.799684	1.108494	0.469386
EXTL2	0.858175	0.621351	1.185263	0.353232
SLC30A7	0.568198	0.318984	1.012115	0.054974
ZNF281	0.892461	0.553653	1.438602	0.640464
ARPC5	1.31437	0.943568	1.83089	0.105992
NLRP3	1.174529	0.970131	1.421992	0.099126
ZNF496	0.89962	0.673104	1.202364	0.474759
TRIM58	0.941582	0.781011	1.135165	0.528046
SLAMF9	0.925026	0.714127	1.198208	0.554987
KCNJ9	0.807069	0.402502	1.618281	0.545938
IGSF8	1.084927	0.781202	1.506738	0.62666
DDR2	0.95166	0.67438	1.342948	0.777974
PEA15	1.666503	1.220256	2.275941	0.001319
PEX19	1.652462	0.80668	3.385024	0.169816
NCSTN	1.263634	0.737377	2.165476	0.394536
VANGL2	0.796208	0.665901	0.952015	0.012446
SLAMF6	1.029115	0.837634	1.264369	0.784678
OLFML2B	0.898386	0.763128	1.057618	0.198061
FCRLB	0.907557	0.622691	1.322741	0.613785
FCGR3B	0.98131	0.849845	1.13311	0.797104
KLHDC9	1.944387	1.136953	3.325237	0.01515
FLVCR1	0.90522	0.551833	1.484912	0.693336
ATF3	1.097268	0.903904	1.331997	0.347995
RBM15	1.084842	0.681275	1.72747	0.731539
DENND2D	1.255812	0.898987	1.754267	0.181684
IER5	1.631005	1.177281	2.259594	0.003269
SNED1	1.071389	0.859217	1.335955	0.540272
BPNT1	1.464786	0.868743	2.469774	0.152124
ACP6	1.167686	0.95681	1.425039	0.127134
KIF26B	0.989716	0.821055	1.193024	0.913645
TFB2M	0.886307	0.589237	1.333148	0.562285
KLHDC8A	1.185674	0.898745	1.564207	0.22829
PM20D1	1.247552	0.955243	1.629308	0.104417
PKDCC	1.165032	0.832774	1.629853	0.372553
OXER1	0.956826	0.759845	1.204871	0.707464
HAAO	1.016611	0.785356	1.315961	0.900437
B3GALNT2	1.052448	0.682046	1.624008	0.817334
MAPKAPK	1.472669	1.076838	2.014003	0.015374
IL24	1.165732	0.822889	1.651413	0.388152

PIGR	1.454755	0.896117	2.361645	0.12945
CAPN2	1.171719	0.97374	1.40995	0.093315
MRPL55	1.378736	0.98447	1.9309	0.061639
WDR26	0.927985	0.558834	1.540987	0.772706
REL	1.137367	0.774447	1.670358	0.511551
PUS10	1.016969	0.592684	1.744989	0.951293
PEX13	0.775588	0.461885	1.302353	0.336552
KIAA1841	0.665521	0.447131	0.990577	0.04479
TRIM17	1.086563	0.764088	1.545135	0.643982
RFTN2	0.861694	0.563787	1.317015	0.491622
DISC1	0.859951	0.617068	1.198433	0.372927
MEMO1	0.691106	0.455787	1.047917	0.081931
DPY30	1.321513	0.667866	2.614892	0.423339
ARL5A	0.817567	0.505782	1.321549	0.411036
DUSP19	0.986108	0.735038	1.322937	0.925656
NUP35	0.681182	0.410106	1.131436	0.138081
CCDC138	0.767787	0.535419	1.101	0.150778
FBXO41	1.236595	0.847016	1.805359	0.27135
SMC6	1.197116	0.735475	1.948519	0.469157
CCDC74A	1.127997	0.832959	1.527538	0.436251
SLC16A14	0.917413	0.687478	1.224254	0.55819
SGCB	1.135261	0.857938	1.502228	0.374675
NOSTRIN	0.891584	0.635792	1.250286	0.505932
SGPP2	1.181523	0.853301	1.635994	0.31511
BBS5	1.034255	0.652197	1.640124	0.886156
SMARCAD	0.625483	0.427851	0.914403	0.015443
HPGDS	0.941925	0.855905	1.03659	0.220768
PDLIM5	1.37372	1.061182	1.778307	0.015916
RPRD2	1.10901	0.592384	2.076194	0.746393
ANKRD23	0.958934	0.565163	1.627062	0.876468
CTSS	1.123161	0.975894	1.29265	0.1053
MSX1	0.775234	0.482045	1.246745	0.293616
PACRGL	0.43559	0.264079	0.718492	0.001135
BNIPL	0.740456	0.412373	1.329563	0.314337
C1QTNF7	0.999197	0.624389	1.598996	0.997329
TNFAIP8L2	1.305381	0.976531	1.744973	0.071925
LYSMD1	1.129491	0.73917	1.725921	0.573515
SCNM1	4.107207	2.056476	8.202939	6.26E-05
TMOD4	0.83288	0.572732	1.211192	0.338511
VPS72	1.103474	0.620418	1.962637	0.737516
ERCC3	0.897078	0.56665	1.420187	0.643092
RNF149	0.977087	0.689024	1.385582	0.896516
IWS1	1.015612	0.595712	1.731486	0.954615
BOLA3	1.259658	0.596071	2.661993	0.545396
CDC42EP3	1.342221	1.116425	1.613684	0.001737
S100A11	1.127321	0.967418	1.313653	0.124651
DHX57	0.944279	0.618924	1.440666	0.790233
ARHGAP2!	1.216147	0.767227	1.927738	0.405076
S100A9	1.087987	1.010794	1.171074	0.02471
S100A12	1.078764	1.002245	1.161124	0.043413
TGFA	0.718065	0.574933	0.896831	0.003501
TDRD10	1.005414	0.618704	1.633831	0.982611
CCNYL1	1.400701	0.896865	2.18758	0.138493
FZD5	1.088152	0.785147	1.508093	0.611915
DCAF16	0.723852	0.463279	1.130986	0.155796
NPPC	0.85251	0.310241	2.342606	0.757017
GNPDA2	0.832205	0.545831	1.268827	0.393357
PAQR3	0.809801	0.516562	1.269506	0.357736

ANTXR2	1.125923	0.758004	1.67242	0.556865
HELQ	0.630849	0.388393	1.024659	0.062669
MRPS18C	0.836522	0.40658	1.721113	0.627732
CGGBP1	0.839333	0.522966	1.347085	0.468073
GPR155	0.897863	0.682897	1.180498	0.440363
DAPL1	1.108927	0.876219	1.403439	0.389582
PMVK	1.924275	1.195663	3.096887	0.007018
PBXIP1	0.939927	0.673787	1.311189	0.715286
PYGO2	1.316854	0.806541	2.150052	0.271155
HIPK1	0.546695	0.352603	0.847627	0.006959
LENEP	0.982385	0.641998	1.503244	0.934739
DCST2	1.382563	0.916258	2.086181	0.122758
DCST1	1.350543	0.697462	2.61515	0.37277
COL6A3	0.933108	0.708953	1.228135	0.621358
YY1AP1	1.180119	0.656478	2.121441	0.579942
KBTBD8	1.845328	1.388663	2.452168	2.41E-05
LMOD3	2.120728	1.041555	4.318053	0.038248
SLC22A15	1.006696	0.903239	1.122003	0.903996
ATP1A1	1.261043	0.834769	1.904992	0.270488
SLC15A2	1.122636	0.809791	1.556343	0.487631
EIF4E3	1.070494	0.891152	1.285928	0.466526
PROK2	1.048422	0.948809	1.158494	0.353231
LRRC58	1.146739	0.802682	1.638272	0.451865
FSTL1	0.899898	0.759212	1.066653	0.223971
ELF3	1.120758	0.613266	2.048211	0.71095
TMEM183/	1.071078	0.562286	2.040258	0.834568
IGFBP7	0.87083	0.709516	1.068819	0.185761
TRIM46	1.033117	0.864638	1.234426	0.719822
KRTCAP2	1.002635	0.75248	1.335951	0.985664
CXCR1	1.126746	0.9421	1.347583	0.191277
ARPC2	2.248102	1.352447	3.736904	0.001782
CCT3	1.520807	0.961863	2.404557	0.072875
TMEM79	1.102229	0.714672	1.699953	0.659712
SSR2	0.958311	0.51368	1.787807	0.893528
RNF25	1.821245	1.068921	3.103065	0.027446
STK36	1.100615	0.776463	1.560093	0.590171
NEK10	0.800518	0.587767	1.090278	0.158067
CCDC141	1.185789	0.745203	1.886862	0.472124
FEV	0.430231	0.155707	1.188764	0.103841
EOMES	1.106814	0.870537	1.407221	0.407481
CWC22	0.955985	0.59007	1.54881	0.854918
AZI2	0.610575	0.432628	0.861714	0.005006
TGFBR2	0.82537	0.590716	1.153236	0.260772
ANKZF1	1.037409	0.666772	1.614071	0.870642
HDAC11	1.72103	1.192548	2.48371	0.003722
TRAT1	0.808237	0.685036	0.953595	0.011633
FBLN2	1.056112	0.88921	1.254343	0.533907
GLB1L	1.078436	0.819745	1.418764	0.589467
STT3B	0.75855	0.554258	1.038142	0.084321
CHCHD4	1.63812	1.007811	2.66264	0.046442
FCRL1	0.895161	0.73638	1.088178	0.266261
SERPINI1	1.380612	1.102928	1.728209	0.004877
CLASP2	0.659717	0.402398	1.081583	0.099139
SUCLG1	2.281476	1.16469	4.469111	0.0162
NUAK2	1.1755	0.950634	1.453557	0.135536
SPTA1	0.930664	0.803618	1.077796	0.337288
PRKCI	0.665292	0.42638	1.038073	0.072598
MNDA	0.970384	0.865677	1.087754	0.605809

PYHIN1	1.031442	0.8636	1.231905	0.73262
IFI16	1.196815	0.90022	1.59113	0.216268
AIM2	1.258052	1.053368	1.502508	0.011282
EFHB	1.43848	0.788294	2.624943	0.236101
EIF5A2	0.858981	0.649269	1.13643	0.287139
RPL22L1	1.099853	0.787632	1.535841	0.576381
PPM1L	0.990126	0.694235	1.41213	0.956313
ICA1L	0.90032	0.595583	1.36098	0.618437
CTLA4	1.266149	0.97604	1.642487	0.075518
ICOS	1.0287	0.789186	1.340906	0.834258
RYBP	0.696844	0.472767	1.027126	0.068039
PPP4R2	0.584666	0.353718	0.966404	0.036328
CD200R1	1.194319	0.99001	1.440792	0.063586
GTPBP8	1.613115	0.94132	2.764353	0.081873
WDFY3	0.81883	0.693508	0.966799	0.018356
COX18	0.812813	0.403429	1.637624	0.561992
PTPN13	0.693427	0.52581	0.914477	0.009508
THOC7	1.384592	0.756779	2.533229	0.291071
ATXN7	0.458138	0.282148	0.743902	0.001599
PSMD6	0.86629	0.498124	1.506569	0.611183
ADAMTS9	0.828059	0.53506	1.281505	0.397125
PPM1K	0.892291	0.62731	1.269201	0.526131
GMPS	1.071621	0.655968	1.75065	0.782376
TIPARP	0.94608	0.723347	1.237398	0.685698
CCNL1	0.699099	0.496486	0.984396	0.040362
PTX3	1.106572	0.922362	1.327572	0.275695
HESX1	1.157613	0.902661	1.484574	0.248861
SLMAP	0.731873	0.424181	1.262757	0.262011
RPL9	0.798383	0.544814	1.169969	0.248151
RPP14	1.204616	0.615035	2.359378	0.58729
ABHD6	1.331275	1.038994	1.705777	0.023672
DNASE1L3	0.995069	0.80176	1.234987	0.964226
RBM47	1.125269	1.005075	1.259837	0.040581
APBB2	1.139378	0.81314	1.596505	0.448379
IL17RE	0.926378	0.831842	1.031658	0.163786
IL17RC	0.983009	0.817701	1.181735	0.855249
CRELD1	0.678509	0.452561	1.017265	0.060502
PRRT3	1.045616	0.80534	1.35758	0.737743
PCOLCE2	1.066011	0.870958	1.304748	0.535273
MTMR14	1.717736	1.081253	2.728887	0.021978
TTC14	0.85463	0.615108	1.187423	0.349179
CXCL3	1.108595	0.93516	1.314195	0.234964
CXCL5	1.147034	0.828614	1.587816	0.408337
PPBP	1.140156	1.040503	1.249352	0.004942
PF4	1.149127	1.044805	1.263864	0.004202
MTHFD2L	0.90246	0.658433	1.236929	0.523443
CXCL1	1.135921	0.973457	1.3255	0.105586
RCHY1	0.689037	0.407943	1.163819	0.16371
CPA3	0.891758	0.816382	0.974094	0.011007
GYG1	1.407711	0.998755	1.98412	0.050837
HPS3	0.612743	0.416053	0.902419	0.013146
TOPBP1	0.968882	0.626464	1.498463	0.887009
RYK	0.854424	0.571842	1.276646	0.442553
SNRK	0.677335	0.429246	1.068812	0.094127
TCF23	1.483386	1.12185	1.961433	0.005663
UCN	1.082182	0.844185	1.387276	0.533109
ZNF513	1.299027	0.902428	1.869923	0.159255
SLC4A1AP	0.869347	0.45794	1.650354	0.66857

PLB1	1.221142	0.973482	1.531808	0.084067
SPDYA	0.550293	0.353668	0.856234	0.008095
KIAA1143	0.931488	0.600214	1.445602	0.751622
KIF15	0.849697	0.658019	1.097211	0.21177
TGM4	0.58243	0.321816	1.054096	0.074115
WDR43	0.930703	0.595203	1.455316	0.752868
ZDHHC3	0.791246	0.454484	1.37754	0.407837
CDCP1	1.19608	1.046344	1.367244	0.008695
CLEC3B	0.966911	0.659549	1.417509	0.863126
LZTFL1	0.661921	0.450055	0.973526	0.036057
FYCO1	0.754892	0.49391	1.153775	0.193909
CCR1	1.144702	1.014837	1.291185	0.02783
LRRC2	0.754046	0.511219	1.112215	0.154554
FBXO40	1.161737	0.697978	1.933634	0.564128
DTX3L	1.236895	0.894818	1.709745	0.198047
ZNF148	0.694854	0.441607	1.093331	0.115454
NMNAT3	0.907899	0.667474	1.234927	0.538167
ZMYM6	0.991869	0.588438	1.671892	0.975552
YEATS2	0.822276	0.526275	1.284761	0.390101
ZC3H12A	1.144737	0.897283	1.460435	0.276692
MEAF6	0.99401	0.487732	2.025817	0.986805
SNIP1	0.818097	0.456733	1.465371	0.499606
DNALI1	0.804723	0.434538	1.49027	0.489553
POLR2H	1.142963	0.715487	1.825839	0.576084
LIPH	1.188802	0.869765	1.624865	0.278021
TMEM41A	0.934107	0.535248	1.630191	0.810397
RPN1	1.113007	0.729662	1.697751	0.619204
SENP2	0.784082	0.427121	1.43937	0.432552
HEYL	1.181179	0.924893	1.508482	0.182102
IFT122	0.806044	0.581571	1.117159	0.195419
RFC4	1.540387	0.965003	2.458844	0.070193
RPL39L	1.380045	1.092681	1.742984	0.006851
BAP1	1.327696	0.830694	2.122053	0.236144
TKT	1.136898	0.844685	1.530201	0.397313
PRKCD	1.347605	1.076685	1.686696	0.009182
RFT1	1.05617	0.581403	1.918625	0.857604
SFMBT1	0.812846	0.646409	1.022137	0.076286
GNL3	1.004961	0.666504	1.515291	0.981155
PBRM1	0.717272	0.454345	1.132355	0.153751
ARHGEF3	1.297658	0.971446	1.733413	0.077756
SLBP	1.355189	0.813498	2.25758	0.243106
LRPAP1	1.227966	0.871798	1.729644	0.240003
ZDHHC19	0.874977	0.518558	1.476371	0.616811
UBXN7	0.786028	0.49393	1.250867	0.30978
RNF168	0.832704	0.506469	1.369077	0.470497
PIGX	0.604257	0.352354	1.03625	0.067165
S100P	0.928279	0.831136	1.036776	0.18697
CLDN19	0.789027	0.532214	1.169762	0.23821
ERMAP	1.017299	0.701109	1.476085	0.928046
ZNF691	1.214897	0.777534	1.898276	0.392614
SGMS2	1.199341	1.04719	1.3736	0.008636
METAP1	0.814731	0.481618	1.378244	0.444923
DNAJB14	0.585217	0.36715	0.932804	0.024297
EMCN	1.070591	0.752836	1.522463	0.704187
BDH2	0.950166	0.614136	1.470058	0.818424
PGRMC2	0.968259	0.574251	1.632606	0.903683
CDC25A	1.0651	0.786658	1.442098	0.683331
CAMP	0.968742	0.839462	1.117931	0.66389

ZNF589	0.968827	0.660184	1.421765	0.871446
PLXNB1	0.702537	0.585837	0.842483	0.000139
CCDC51	1.777143	1.064837	2.965933	0.027781
ATRIP	1.477962	0.769036	2.8404	0.241171
SHISA5	1.952046	1.270119	3.0001	0.002285
SPRY1	1.061162	0.931631	1.208703	0.371451
APEH	1.994896	1.206379	3.298806	0.007122
INTU	1.035868	0.833381	1.287554	0.750826
RNF123	0.958533	0.659317	1.39354	0.824448
HSPA4L	0.765431	0.593939	0.986439	0.03888
MFSD8	0.50446	0.310648	0.81919	0.005671
CAMKV	1.320988	0.993098	1.757137	0.055827
MON1A	2.138867	1.240905	3.686625	0.0062
MST1R	1.230402	0.986313	1.534897	0.066091
RAD54L2	0.91263	0.537809	1.54868	0.73473
TEX264	1.695494	0.979628	2.934478	0.059238
GRM2	1.305748	0.777222	2.193682	0.313532
DUSP7	2.030179	1.468525	2.806644	1.82E-05
PPM1M	1.368886	1.079332	1.736119	0.00961
WDR82	1.682642	0.90445	3.130393	0.100405
NDST3	0.654065	0.523671	0.816928	0.000182
HMGB2	0.894754	0.635178	1.260411	0.524702
SAP30	0.8763	0.681719	1.126419	0.302665
SCRG1	0.681703	0.479324	0.969531	0.032995
MAD2L1	0.99431	0.720065	1.373006	0.972355
ANXA5	1.137479	1.011062	1.279703	0.032115
MAP9	1.293173	0.955651	1.749903	0.09571
FBXO8	0.987605	0.608867	1.601935	0.959693
HPGD	0.982953	0.851482	1.134724	0.814441
TMEM144	1.317126	1.06318	1.631727	0.011716
IL15	1.144283	0.955672	1.370119	0.142488
FAM160A1	1.034017	0.625717	1.708743	0.896153
ARFIP1	1.103774	0.747599	1.629639	0.619417
HHIP	0.570058	0.36268	0.896013	0.014857
ANAPC10	0.682415	0.365428	1.27437	0.23048
ABCE1	0.975376	0.67048	1.418922	0.896276
OTUD4	0.913758	0.562757	1.483685	0.71535
LSM6	1.002147	0.581086	1.728315	0.993845
TMEM184	0.961973	0.635704	1.455696	0.854461
ITGA2	1.084953	0.880235	1.337282	0.444713
MOCS2	1.026675	0.73273	1.43854	0.878424
EDIL3	0.975874	0.810503	1.174987	0.796572
TMEM161	0.635939	0.40104	1.008422	0.054316
ELOVL7	1.131627	0.960933	1.332642	0.138268
NDUFAF2	1.341826	0.74357	2.421422	0.328951
LMBRD2	0.794995	0.520922	1.213265	0.287474
NIPBL	0.727339	0.479798	1.102595	0.133654
SLC25A46	0.70398	0.4621	1.072469	0.102211
STARD4	1.256383	1.014646	1.555712	0.03632
PGGT1B	0.819967	0.536402	1.253437	0.359293
F2RL2	0.853468	0.64082	1.13668	0.278486
CCDC112	0.858523	0.685683	1.07493	0.183524
CMBL	0.951249	0.770962	1.173695	0.641099
PRRC1	0.562196	0.375173	0.842451	0.005259
F2RL1	1.013742	0.891819	1.152332	0.834641
AGGF1	0.961539	0.612845	1.50863	0.864488
WDR41	0.916418	0.596944	1.406868	0.689823
NDUFS4	0.885853	0.532597	1.473413	0.640566

SPINK1	0.839438	0.257519	2.736321	0.771581
GRPEL2	1.037657	0.725917	1.483272	0.839307
ARSK	0.721418	0.472809	1.100748	0.129849
RHOBTB3	0.743894	0.635734	0.870456	0.000224
TIGD6	1.409218	0.770521	2.57734	0.26543
SERINC5	0.605265	0.484578	0.756009	9.64E-06
CAGE1	0.860437	0.28778	2.572637	0.787937
CASP3	0.717087	0.522531	0.984085	0.039464
ERAP1	0.765625	0.551547	1.062796	0.110489
ERAP2	0.871029	0.707701	1.072051	0.192479
CMYA5	1.297225	0.359113	4.685965	0.691281
RICTOR	0.636979	0.426591	0.951126	0.027458
EBF1	1.089027	0.893883	1.326772	0.397273
ANKRA2	0.564546	0.333189	0.956553	0.033581
UBLCP1	1.567652	1.011398	2.429836	0.044362
UTP15	0.692558	0.427021	1.123216	0.136488
TLR3	0.929011	0.687246	1.255825	0.632084
NSA2	0.857712	0.55429	1.32723	0.490789
GFM2	0.641351	0.408567	1.006765	0.053525
TERT	1.129376	0.877031	1.454329	0.34569
CCDC127	1.342995	0.826859	2.181309	0.233383
ACSL6	0.573084	0.359571	0.913381	0.019236
IL3	1.249473	0.593999	2.62826	0.557173
CSF2	2.605527	0.336528	20.17297	0.359117
SHROOM1	0.90639	0.693954	1.183857	0.470717
GDF9	0.584105	0.324758	1.05056	0.07261
UQCRQ	1.226437	0.743286	2.023644	0.424377
LEAP2	0.652221	0.40138	1.059826	0.084458
GJB7	0.675775	0.391368	1.166863	0.159657
SLC35A1	1.210388	0.732008	2.001397	0.456785
TXLNB	1.196571	0.923701	1.550051	0.17416
CITED2	0.695439	0.535141	0.903754	0.006588
DCBLD1	1.08511	0.866721	1.358525	0.476211
SFXN1	1.399791	1.046137	1.872999	0.023603
SAMD3	1.072169	0.818928	1.40372	0.612232
PDSS2	0.849349	0.462662	1.559221	0.598312
STXBP5	0.642938	0.491779	0.840558	0.001237
IL31RA	1.231067	0.991177	1.529017	0.060132
ANKRD55	1.547305	1.16469	2.055614	0.002596
PI16	1.583274	1.142036	2.194989	0.005837
DAGLB	0.676802	0.459995	0.995796	0.047552
KIAA0895	0.686032	0.516976	0.910372	0.009042
STK17A	0.740817	0.479013	1.145711	0.17749
TRA2A	0.593909	0.34264	1.029441	0.063372
GALNT10	1.201438	0.795705	1.814056	0.382696
SAP30L	0.613511	0.403239	0.93343	0.022507
RPS14	0.972342	0.663537	1.424864	0.885613
MYOZ3	0.793083	0.61767	1.018311	0.069111
COG5	0.705647	0.454115	1.096501	0.121067
GPR85	0.826647	0.616575	1.108291	0.203146
SLU7	1.110512	0.651373	1.893288	0.700167
RP9	1.053136	0.615619	1.801594	0.850096
PTTG1	0.901838	0.636963	1.276858	0.560315
CAMLG	0.719212	0.469258	1.102306	0.130312
RELL2	1.427601	1.118602	1.821955	0.004229
KCNK5	1.064454	0.906705	1.249648	0.445326
ZNF12	0.629484	0.412969	0.959516	0.031388
SLC29A4	0.442102	0.292186	0.668938	0.000112

CDCA7L	0.802694	0.63257	1.018573	0.070524
SP8	1104.438	0.070986	17183457	0.154786
MIOS	1.016199	0.635115	1.625943	0.946574
USP49	0.82228	0.538334	1.255993	0.36527
SYTL3	1.142231	0.930032	1.402845	0.204721
ZNF704	0.962588	0.792303	1.169471	0.701072
FABP5	1.308926	0.997017	1.718413	0.052568
TAGAP	1.384467	0.973006	1.969924	0.070624
COL1A2	0.895754	0.734961	1.091726	0.275457
SLC13A4	0.715866	0.511187	1.002498	0.051722
BRI3	0.977516	0.721688	1.324031	0.883214
LMTK2	0.62738	0.442772	0.888959	0.008743
CTSB	1.184473	0.951707	1.474167	0.129377
DLC1	0.920361	0.78317	1.081584	0.313606
ADCY1	1.015835	0.658029	1.5682	0.943466
RAD21	0.872756	0.570366	1.335462	0.530599
MED30	0.783529	0.491555	1.248933	0.305126
PHKG1	0.663218	0.416538	1.055985	0.083556
DEFA4	1.006459	0.908059	1.115523	0.90238
OSGIN2	0.907654	0.566462	1.454355	0.687091
DEFB1	0.942088	0.857221	1.035357	0.215507
OXR1	1.203459	0.815374	1.776258	0.351137
GPR146	1.092735	0.759431	1.572322	0.63287
UNCX	0.758625	0.626151	0.919126	0.004784
TMEM184,	0.971082	0.710765	1.326739	0.853777
NOS3	1.655647	1.091951	2.510338	0.017588
MICALL2	0.85691	0.730795	1.004788	0.057282
CA3	1.279227	1.01841	1.606839	0.034279
INTS1	1.209177	0.880663	1.660236	0.240276
CDK5	1.698103	1.13537	2.539746	0.009934
SLC4A2	1.13953	0.793194	1.637089	0.479816
FASTK	1.044262	0.719897	1.514779	0.819477
TMUB1	1.452042	1.031977	2.043093	0.032305
PHAX	0.792986	0.536475	1.172146	0.244696
ALDH7A1	0.690922	0.505097	0.945111	0.020714
FOXK1	0.746973	0.441718	1.26318	0.276441
COX6C	1.387629	0.81448	2.364103	0.228166
OSR2	1.208046	0.675771	2.159571	0.523671
YWHAZ	1.429381	0.810566	2.520621	0.217086
BAALC	0.999162	0.907874	1.099629	0.986316
FZD6	1.109821	0.969196	1.27085	0.131721
CTHRC1	0.913411	0.713022	1.170118	0.473548
SLC25A32	0.791671	0.445231	1.407682	0.42631
TP53INP1	0.854138	0.649579	1.123114	0.259007
INTS8	0.801892	0.502567	1.279495	0.354387
FREM1	0.910528	0.725332	1.143009	0.419156
GEM	0.951565	0.512015	1.768456	0.875237
TMEM67	0.848452	0.649497	1.108352	0.228046
SNAPC3	0.783865	0.51721	1.187998	0.251006
NUDT2	1.069867	0.641914	1.78313	0.795547
TMEM65	1.023082	0.815328	1.283774	0.843794
PSIP1	0.974549	0.60291	1.575269	0.916199
UBAP1	1.348417	0.71237	2.552363	0.358513
SYK	1.51535	1.086482	2.113505	0.014341
NIPSNAP3	1.039461	0.803761	1.344278	0.768011
ABCA1	0.813615	0.675384	0.980138	0.029921
NFIL3	1.005234	0.827139	1.221675	0.958157
LETM2	0.586907	0.378618	0.909781	0.017184

METTL2B	0.589607	0.353809	0.982554	0.04261
FXN	1.006434	0.669405	1.513149	0.975408
TMEM71	1.193605	0.890612	1.599678	0.236189
MAMDC2	0.962379	0.856673	1.081128	0.518302
CPA6	0.877805	0.725491	1.062097	0.180124
ALDH1A1	1.091407	0.982222	1.212728	0.103861
KDM1B	1.332814	0.932831	1.904305	0.114556
HGSNAT	0.821831	0.566872	1.19146	0.300434
GKAP1	0.728702	0.52994	1.002013	0.051464
KIF27	0.769311	0.492167	1.202517	0.249829
HNRNPK	0.884299	0.553507	1.41278	0.606985
TRPV6	0.866566	0.549109	1.367556	0.538395
ANKS6	1.128748	0.919157	1.386131	0.247842
FBP1	1.083898	0.954305	1.231089	0.214957
ZHX1	1.113255	0.724008	1.711773	0.625016
CYBB	1.019982	0.921785	1.128639	0.701669
DYNLT3	1.068636	0.775084	1.473366	0.685395
MID1IP1	0.959622	0.696427	1.322284	0.801049
NCF1C	1.142582	1.01356	1.288028	0.029237
KIAA1958	0.674877	0.551067	0.826504	0.000143
PCDH19	0.001447	5.89E-07	3.55241	0.100654
PIGA	0.633325	0.404645	0.99124	0.045667
OR1K1	0.601798	0.318203	1.138143	0.118296
STRBP	0.946733	0.705504	1.270444	0.715274
CLDN3	1.507259	0.932382	2.436587	0.094078
GAPVD1	0.797164	0.49037	1.295898	0.360498
ATP7A	0.834141	0.59177	1.175778	0.300468
ZNF367	1.051172	0.756136	1.461328	0.766535
HDX	0.954051	0.669003	1.360552	0.795055
NDUFB6	1.321134	0.77943	2.239321	0.300953
NOL6	1.240066	0.81028	1.897818	0.32168
AQP3	0.981978	0.823161	1.171438	0.839882
VCP	1.702793	1.005216	2.884458	0.047781
PIGO	0.855746	0.508357	1.440524	0.557689
STOML2	1.946918	1.185479	3.19743	0.008484
BRWD3	0.689118	0.453887	1.04626	0.080516
SLITRK5	1.092413	0.931447	1.281195	0.277131
MELK	1.161603	0.861845	1.565619	0.325281
OTUD1	1.133498	0.892619	1.439379	0.303937
ARHGAP1:	0.929175	0.718898	1.200957	0.574701
HECTD2	0.918015	0.685462	1.229465	0.566011
SLC7A3	0.762101	0.452832	1.282591	0.306358
FBXO33	1.299923	0.676926	2.496284	0.43075
LRRC18	0.88271	0.685607	1.136476	0.333207
WRN	0.622579	0.431347	0.898591	0.011371
TSHR	0.716945	0.501829	1.024274	0.067521
CFL2	0.784301	0.494451	1.244064	0.301982
SUGT1	0.637111	0.378154	1.073398	0.090297
GTF2A1	1.116904	0.722358	1.726945	0.61902
ZCCHC24	1.107241	0.875103	1.400959	0.396101
PGM2L1	1.040948	0.737252	1.469745	0.819631
SLC16A9	0.941114	0.810224	1.09315	0.427014
FOLR2	1.198925	1.005054	1.430193	0.043802
INPPL1	1.31494	0.890455	1.941779	0.168634
PHOX2A	1.091654	0.366815	3.248799	0.874773
GJB2	0.824488	0.535348	1.26979	0.381077
CRYL1	1.302812	0.893668	1.899272	0.169
REEP3	1.198288	0.915196	1.568947	0.188336

PCF11	0.728732	0.391235	1.357371	0.318689
RPL10L	1.060623	0.560541	2.006848	0.856452
RPL36AL	1.169094	0.651559	2.09771	0.60044
ZNF22	0.979718	0.672361	1.427576	0.915044
KLHDC2	0.694048	0.367599	1.3104	0.260044
RPUSD4	0.84869	0.538237	1.33821	0.480126
ARF6	2.169602	1.336244	3.52269	0.001736
TTC8	0.915103	0.70582	1.186441	0.5031
TMEM63C	1.389552	1.100952	1.753804	0.005612
AKR1E2	1.052681	0.865795	1.279907	0.606665
KBTBD6	1.185578	0.793877	1.770545	0.405457
FAAH2	0.808784	0.552537	1.183867	0.274963
NUDT5	1.30725	0.899457	1.899927	0.160172
DACT1	1.060342	0.764443	1.470778	0.72561
PRPF18	0.862716	0.456435	1.630635	0.649384
TAF3	1.662861	0.816729	3.385587	0.160951
VDAC2	1.494982	0.808449	2.764519	0.199833
COMTD1	1.773309	1.351832	2.326194	3.52E-05
SLC18A2	0.929136	0.778315	1.109184	0.416039
PDZD8	0.826212	0.6664	1.024351	0.081754
ZNF503	1.194943	1.01359	1.408743	0.033947
QSOX2	0.710512	0.511223	0.987489	0.041859
NSD1	0.97439	0.623994	1.521548	0.909164
PRDX3	1.2253	0.810315	1.852811	0.335524
ENOX2	1.347522	0.898113	2.021812	0.149626
GHITM	2.042167	1.172178	3.55786	0.011708
CLEC1B	1.074368	0.875509	1.318395	0.492157
SNAPC4	1.082557	0.729278	1.606972	0.693888
PMPCA	1.213255	0.786431	1.871734	0.382187
TSC1	0.851493	0.560949	1.292526	0.450273
GFI1B	1.048195	0.909751	1.207708	0.514872
HPRT1	1.260183	0.837588	1.895993	0.267172
ZMYND19	1.721824	1.040904	2.848174	0.034338
STOX1	1.060984	0.78504	1.433924	0.700102
RET	1.029659	0.819195	1.294195	0.802179
DDX21	1.059953	0.752743	1.492542	0.73881
BMS1	0.864115	0.527314	1.416035	0.56221
STK32C	1.64082	1.219861	2.207046	0.001061
FUNDC2	1.670026	0.819352	3.403891	0.158079
NDRG2	1.258168	1.007721	1.570858	0.042573
ZNF219	0.779531	0.620989	0.97855	0.031803
CASP7	1.172446	0.760052	1.808599	0.471919
METTL3	0.970483	0.6305	1.493795	0.891696
SALL2	0.978454	0.766663	1.248753	0.861066
PRAP1	0.955101	0.636731	1.432658	0.824269
TRUB1	0.838994	0.5783	1.217207	0.355145
ZFYVE1	0.879013	0.522899	1.477652	0.626536
FRAT1	1.254564	0.956211	1.646009	0.101673
UBTD1	1.124573	0.905365	1.396856	0.288563
E2F7	1.103649	0.853273	1.427492	0.452502
ISCA2	1.33025	0.732806	2.414778	0.348214
PACSIN3	0.783731	0.569952	1.077694	0.133734
TTC7B	0.944411	0.686537	1.299147	0.725206
SLC39A13	1.047716	0.761181	1.442113	0.774918
PSMC3	2.032503	1.198433	3.44706	0.008499
RAPSN	1.070719	0.792071	1.447395	0.65683
AGBL2	0.93682	0.626255	1.401396	0.750774
TC2N	1.00699	0.842015	1.204289	0.939175

CPSF2	1.023981	0.664233	1.57857	0.91454
MOAP1	1.167198	0.776691	1.754043	0.45691
IFI27L1	1.584123	0.965944	2.597919	0.068352
IFI27	1.143862	0.936063	1.397791	0.188839
CLMN	0.828403	0.665049	1.031882	0.092976
PDZRN4	1.15882	0.947485	1.417293	0.151329
CCDC38	0.799184	0.336402	1.898605	0.611624
PTER	1.180603	0.809699	1.721409	0.38821
C1QL3	1.086851	0.780962	1.512551	0.621389
CACNB2	1.061528	0.742991	1.51663	0.742902
ARL5B	0.633793	0.464056	0.865614	0.004139
TAF1D	1.036525	0.659681	1.628642	0.876348
ABTB2	0.962128	0.776384	1.19231	0.724261
AMOTL1	1.040466	0.881357	1.228299	0.639442
HTRA1	1.043801	0.776987	1.40224	0.77593
LIPC	1.430052	1.061761	1.926091	0.018552
CEP57	0.723692	0.474308	1.104198	0.133572
TCP11L2	1.01247	0.743155	1.379384	0.937393
SPRED1	1.081082	0.909104	1.285592	0.377809
GPR176	1.116533	0.728529	1.711183	0.612849
JAM3	0.945067	0.760999	1.173657	0.609216
CMTM5	1.192459	1.012168	1.404866	0.035329
ADAMTS1	0.607611	0.431821	0.854965	0.004247
GPT2	0.855172	0.7211	1.014172	0.072143
AMN	0.859864	0.712588	1.037579	0.115231
RAB8B	1.04422	0.712966	1.52938	0.82412
IKBIP	0.999133	0.680929	1.466038	0.996464
RPUSD2	1.803514	1.068012	3.045531	0.027377
HIF1AN	0.901417	0.559569	1.452107	0.669649
NDUFB8	1.299387	0.649308	2.600316	0.45936
ZFYVE19	2.172778	1.353698	3.487457	0.001307
SPINT1	1.235314	1.053231	1.448876	0.009393
FBN1	1.060728	0.880828	1.277371	0.53411
AVPR1A	1.008037	0.748208	1.358099	0.958021
DEPDC4	0.975806	0.590876	1.6115	0.923767
LRTM2	0.845923	0.575898	1.242557	0.393692
BRD7	1.324359	0.666836	2.630224	0.422279
CKB	0.969341	0.819933	1.145975	0.715418
TRMT61A	1.356879	0.941454	1.955614	0.101742
BTRC	0.721292	0.460808	1.129022	0.152966
POLL	1.217111	0.712531	2.079012	0.471989
BAG5	1.326077	0.767574	2.290961	0.311674
API5	1.132984	0.695711	1.845095	0.615814
ZNF319	1.261782	0.867984	1.834244	0.223144
HPS6	2.140682	1.317377	3.478517	0.002121
SENP8	0.842839	0.424091	1.67506	0.625611
NOLC1	1.119626	0.775029	1.61744	0.547138
ALKBH3	1.01864	0.615478	1.685889	0.942725
COPS2	1.100392	0.678384	1.784923	0.698284
SGPL1	1.409843	0.998521	1.990601	0.050991
FRS2	0.693681	0.392482	1.226026	0.208147
CCT2	0.897616	0.636298	1.266254	0.538374
PCBD1	1.559148	1.113602	2.182954	0.009693
ARIH1	0.573603	0.309838	1.061911	0.076927
COX11	0.987564	0.64131	1.520767	0.954696
ZNF202	0.878544	0.557329	1.384891	0.577081
STXBP4	0.766174	0.521169	1.126357	0.1755
CYYR1	1.10783	0.969504	1.265891	0.132364

CUL5	0.849902	0.525163	1.375444	0.507886
C2	1.195026	0.981489	1.455021	0.076074
PLEKHF1	1.132765	0.874899	1.466633	0.344202
SMPD1	1.361889	0.936701	1.98008	0.105766
APBB1	1.081894	0.851528	1.37458	0.519364
SYNPO2L	1.860149	1.077007	3.212748	0.026012
NUDT13	0.68305	0.440085	1.060155	0.089221
TRIM44	1.716152	1.101024	2.674944	0.017081
ILK	0.806583	0.473991	1.372549	0.428087
TAF10	1.123611	0.618974	2.039669	0.701633
TPP1	1.285293	0.899241	1.837079	0.168449
DCHS1	0.947828	0.827396	1.085791	0.439631
CYB5A	0.924106	0.58966	1.448243	0.730604
USP54	0.948912	0.660243	1.363793	0.776897
RAG1	1.138583	0.896998	1.445232	0.286145
WDR88	0.972768	0.664788	1.423427	0.886959
ATP9B	0.659392	0.420998	1.032777	0.068899
PPFIBP2	1.243887	0.993897	1.556754	0.056587
CYB5R2	1.644058	1.17968	2.291239	0.003328
SERPINB8	0.914322	0.777095	1.075782	0.280339
RIC3	0.939069	0.640339	1.377162	0.747601
IDH3A	1.69214	1.160949	2.466378	0.006213
CRABP1	1.079767	0.882238	1.321521	0.456575
PLD4	1.222297	1.07001	1.396258	0.00311
ZMAT1	0.635923	0.476357	0.84894	0.002134
XRRA1	0.833479	0.557493	1.246091	0.374696
TRIM66	0.900467	0.649942	1.247559	0.528521
RNF169	0.803374	0.557108	1.158501	0.24111
RPL27A	1.350251	0.845358	2.156691	0.208813
CDYL2	1.112677	0.878639	1.409055	0.375539
CENPN	1.469515	0.956038	2.258773	0.079261
ATMIN	1.262128	0.743567	2.142334	0.388482
TMEM41B	0.827128	0.500023	1.368218	0.459846
LEO1	0.886346	0.562482	1.396682	0.603068
ZNF143	0.917131	0.553545	1.519533	0.737024
TMX3	0.80185	0.529824	1.213542	0.296244
MFAP4	0.919247	0.808303	1.045418	0.199456
WEE1	1.257397	0.89215	1.772177	0.190816
MAPK7	1.391825	1.06981	1.810769	0.013794
PRKCB	1.215478	0.858671	1.720551	0.271076
NDST2	0.491196	0.295497	0.816502	0.00611
MCM7	0.921403	0.643551	1.319217	0.654851
CCDC68	1.108008	0.639221	1.920592	0.714777
CLEC4E	0.986359	0.875299	1.11151	0.821699
ZNF3	0.506115	0.312429	0.819875	0.005659
CLEC4D	0.932059	0.822035	1.056808	0.272277
ZSCAN21	0.547267	0.357082	0.838746	0.005655
RIMKLB	0.955747	0.797558	1.14531	0.623925
TK2	1.625353	1.121575	2.355413	0.010285
TMED3	1.635252	0.965802	2.768734	0.067182
SEC11C	1.498586	0.975071	2.303177	0.065062
TMEM135	0.985141	0.633684	1.531527	0.946982
IQCD	0.773296	0.52891	1.130603	0.184648
NDEL1	0.882148	0.538976	1.443823	0.617897
CENPV	0.698057	0.512567	0.950673	0.022551
RRAD	1.412681	0.988769	2.018336	0.057707
HSP90B1	0.79873	0.612651	1.041327	0.096772
BLCAP	1.667857	0.799377	3.479893	0.172812

CHRFAM7.	1.197163	0.872962	1.641767	0.26408
ATF7IP2	1.202206	0.909561	1.589008	0.19569
TMPRSS5	0.804955	0.449426	1.441734	0.465604
COG1	0.916471	0.585994	1.433324	0.702263
PLEKHA7	1.162203	0.892671	1.513118	0.264172
ZNF606	0.916817	0.652272	1.288656	0.617087
B2M	0.984941	0.665353	1.458037	0.939566
ZNF592	1.413462	0.853998	2.339436	0.178288
NNMT	0.792215	0.626589	1.00162	0.051602
ACSM1	0.820086	0.717044	0.937935	0.003788
AP1G1	0.803879	0.458897	1.408206	0.445339
SLFN5	0.996362	0.800527	1.240104	0.973959
CATSPER2	1.021844	0.759313	1.375146	0.886585
SAAL1	0.977745	0.580758	1.646098	0.932514
YPEL4	0.925526	0.751348	1.140081	0.46689
PPIB	0.870815	0.657437	1.153447	0.334774
LDHC	1.423565	1.022727	1.981503	0.036335
LDHAL6A	0.884152	0.581904	1.343391	0.564019
FAM111A	0.952006	0.655359	1.382929	0.796273
KIF7	0.995994	0.798897	1.241717	0.971538
LDHD	1.095953	0.850347	1.412498	0.479101
PEX11A	0.990977	0.687316	1.428797	0.961277
TMEM170,	0.856945	0.520648	1.41046	0.543696
MESP1	1.505803	0.947029	2.394271	0.08364
ANPEP	0.94082	0.806119	1.09803	0.439067
RBPMS2	1.04283	0.916232	1.186922	0.52536
NAV2	0.749633	0.441753	1.27209	0.28551
ANKDD1A	1.208439	0.782818	1.865473	0.392741
DCTN5	1.086987	0.646369	1.827966	0.753134
TERF2IP	1.220592	0.634819	2.346884	0.550097
PLK1	0.987812	0.761723	1.281008	0.926322
CLPX	1.05494	0.616933	1.80392	0.845083
GPR182	0.660537	0.36502	1.1953	0.170552
ZBTB39	0.816901	0.50375	1.324717	0.412262
TAC3	1.110564	0.829988	1.485987	0.480313
MYO1A	0.652408	0.350712	1.213636	0.177472
NAB2	0.767831	0.573807	1.02746	0.075457
VPS39	1.234685	0.741449	2.056036	0.417804
STAT6	1.691607	1.140836	2.50828	0.008908
PATL1	1.643938	0.956665	2.824951	0.071927
STX3	1.176235	0.897295	1.541888	0.239878
MRPL16	2.091533	1.387519	3.152757	0.000425
PIP4K2C	1.272893	0.898853	1.802581	0.174065
MTMR10	0.806567	0.465323	1.398063	0.443691
YWHAB	1.447743	0.950204	2.2058	0.085031
SCG5	1.668933	1.165608	2.389599	0.005162
TSC22D4	0.955139	0.670862	1.359878	0.79901
MS4A7	1.096065	0.959775	1.25171	0.175755
MS4A14	1.08351	0.949622	1.236276	0.233322
DIS3L	1.183098	0.715706	1.955719	0.512053
CCNDBP1	1.286779	0.73883	2.241112	0.373087
EPB42	0.909656	0.795486	1.040213	0.166422
SMAD3	1.596466	1.195385	2.132119	0.00153
MAP1A	0.983503	0.863042	1.120779	0.802956
RCCD1	0.779072	0.486122	1.248561	0.299524
AKTIP	0.806631	0.532243	1.222474	0.311056
MAPRE2	1.31276	0.912856	1.887852	0.142081
MBD6	1.599396	1.083169	2.361653	0.01819

CNPY4	1.257898	0.747255	2.117494	0.387868
PDIA3	0.760427	0.526015	1.099302	0.145261
NUDT21	1.104391	0.75331	1.619092	0.610967
NKX3-1	0.745785	0.573581	0.96969	0.028543
DUSP18	1.631814	1.073943	2.479477	0.021781
TEF	1.343299	0.888397	2.031134	0.16181
MEI1	1.911612	1.273903	2.868554	0.001754
PBX3	1.255102	1.092898	1.441379	0.00129
GNGT2	1.731005	1.348833	2.22146	1.63E-05
PHB	1.84707	1.160173	2.940654	0.009706
SNRPD1	0.896977	0.542892	1.482002	0.671276
TTC16	1.34135	0.938939	1.916225	0.106581
SAMD14	1.102253	0.776866	1.563927	0.585458
PIP5KL1	1.188493	0.855865	1.650395	0.302611
TMEM92	1.161533	0.897008	1.504065	0.256105
FAM102A	1.108871	0.809132	1.519647	0.520398
ACSF2	1.701903	1.148024	2.523007	0.008117
GOLGA2	0.861858	0.531164	1.398436	0.547176
TRUB2	1.298828	0.74192	2.273768	0.360119
COQ4	1.031701	0.651371	1.6341	0.894188
SLC27A4	1.724479	1.177004	2.526607	0.00517
URM1	1.484678	0.888766	2.480143	0.131161
CERCAM	0.791344	0.663759	0.943453	0.009084
DOLPP1	1.181832	0.759524	1.83895	0.458932
ENDOG	1.386779	0.99721	1.928536	0.051973
SP2	1.255966	0.754342	2.09116	0.380936
PRR15L	1.354223	0.874983	2.09595	0.173615
COQ7	1.209659	0.559565	2.615022	0.628455
GPRC5B	0.660169	0.305229	1.427856	0.291407
CRK	1.451241	0.882627	2.386172	0.14214
FBXO22	0.887469	0.567979	1.386671	0.600076
TBC1D2B	0.722061	0.527591	0.988213	0.041949
NOD2	1.059397	0.920423	1.219354	0.421276
SNX20	1.419262	0.982516	2.050149	0.062046
LOXHD1	0.866578	0.704372	1.066139	0.175642
KATNAL2	0.944818	0.639366	1.396197	0.77573
HDHD2	0.472273	0.270288	0.8252	0.00842
ZNF91	0.667249	0.495334	0.89883	0.007776
RNF214	0.977931	0.523275	1.827624	0.944239
DPEP2	1.137666	0.878791	1.472801	0.327525
POP5	1.398219	0.780681	2.504244	0.259614
CD3D	0.946032	0.818968	1.092811	0.450911
TBC1D16	0.935758	0.768115	1.13999	0.509765
ACAA2	0.989239	0.716276	1.366225	0.947635
STIM1	1.092536	0.716219	1.666578	0.681234
RRM1	1.156201	0.831917	1.606893	0.387467
TRIM68	1.368571	0.900025	2.081037	0.142277
FN3K	1.12536	0.919806	1.37685	0.251109
IRGQ	0.748111	0.493221	1.134727	0.172151
ZNF226	0.817935	0.581493	1.150517	0.248293
ZNF180	1.026124	0.646567	1.628494	0.912858
PPP2R3B	0.878759	0.602606	1.281461	0.501911
ZNF668	1.812724	1.100191	2.986727	0.019557
ZNF646	1.36808	0.882258	2.121423	0.161433
VKORC1	1.363869	0.842633	2.207532	0.206567
GNG8	1.357024	0.947553	1.943441	0.095717
LPO	0.811111	0.730656	0.900425	8.57E-05
CA4	0.987521	0.695409	1.402337	0.944052

TPM4	0.810828	0.64083	1.025923	0.080676
RAB8A	0.728004	0.437981	1.210076	0.220778
GPX4	1.968723	1.341439	2.889337	0.000539
MIDN	1.20842	0.914741	1.596385	0.182645
JSRP1	1.098786	0.779003	1.549841	0.591385
KLHL26	1.534068	0.928222	2.535347	0.09504
GATAD2A	1.278389	0.720646	2.267795	0.401032
MVD	1.717499	1.186538	2.486059	0.004152
CDT1	1.027205	0.789188	1.337007	0.84181
TRAPPC2L	0.936152	0.579374	1.512634	0.787545
ANKRD11	0.854959	0.539219	1.355581	0.505204
PROCA1	1.00853	0.658063	1.545648	0.968895
RPL13	1.218937	0.866728	1.714274	0.255165
ZNF641	1.29931	0.921864	1.831297	0.134829
CACNB3	1.188138	0.944991	1.493846	0.140041
DHRS13	0.954076	0.679494	1.339615	0.786014
TP53I13	1.120841	0.816692	1.538261	0.48001
CORO6	0.978763	0.791588	1.210197	0.842874
RHEBL1	1.829532	1.194112	2.803076	0.005522
TUBA1A	1.286404	0.943517	1.7539	0.111304
TUBA1C	1.513392	1.026764	2.230656	0.036313
ZNF610	0.847365	0.671399	1.06945	0.16314
ZNF528	0.917982	0.727661	1.158082	0.470353
ZNF701	0.874275	0.604347	1.264765	0.475731
SERTAD3	1.282575	0.896347	1.835227	0.173397
RAB4B	0.945255	0.598913	1.491883	0.808928
GPD1	1.867508	0.78527	4.441259	0.15763
CYP2S1	0.984599	0.877427	1.104862	0.791805
AXL	0.89109	0.694039	1.144087	0.36583
NFKBID	1.109587	0.833637	1.476882	0.47599
TMC4	0.872086	0.623144	1.220479	0.424804
LAIR1	1.042905	0.762175	1.427036	0.792885
TTYH1	0.906822	0.602933	1.363875	0.63857
LENG8	1.006549	0.761902	1.329752	0.963357
CDC42EP5	1.378326	0.886256	2.143605	0.154429
LAIR2	1.019664	0.76565	1.357949	0.894024
ZNF526	1.094598	0.675776	1.772993	0.713374
TRAPPC9	1.293089	0.76907	2.174159	0.332278
KIR3DL1	1.415177	1.062545	1.884838	0.017555
ZNF146	0.913877	0.670706	1.245211	0.568292
ZNF283	0.769851	0.512187	1.157136	0.20839
PPP1R14A	1.043499	0.851091	1.279404	0.682203
SPINT2	1.2652	1.03307	1.54949	0.022932
YIF1B	1.214454	0.836119	1.763983	0.307645
PSCA	1.474368	0.979872	2.218411	0.062544
ATCAY	0.148152	0.035141	0.624596	0.009293
DAPK3	1.346172	0.843667	2.147978	0.212435
EEF2	1.050347	0.744307	1.482223	0.779842
TMIGD2	0.896084	0.797531	1.006814	0.064932
CHAF1A	1.461042	0.996603	2.141919	0.052073
UBXN6	1.444586	0.984043	2.120668	0.060403
SEMA6B	1.124152	0.89289	1.415311	0.319307
ZNF444	1.083377	0.776378	1.511771	0.63759
NXN	0.853748	0.597169	1.220568	0.385925
GLOD4	1.790134	0.831031	3.856148	0.136954
MFSD3	1.225432	0.906975	1.655707	0.185491
GPT	0.984146	0.754097	1.284374	0.90635
KIFC2	1.253469	0.978764	1.605273	0.07347

SLC43A2	1.229562	1.044946	1.446796	0.012788
RILP	1.47632	1.040373	2.094941	0.029137
SERPINF2	1.230893	0.946237	1.601182	0.12159
WDR81	1.16663	0.881818	1.543432	0.28048
SRR	0.864708	0.536171	1.394556	0.551098
TSR1	0.95638	0.646511	1.414768	0.823346
HSD11B1L	0.958222	0.658379	1.39462	0.823634
CYB5D2	1.258308	0.793495	1.9954	0.328713
KLK1	1.231043	0.95056	1.594288	0.115113
ZNF83	0.794999	0.561794	1.125008	0.195306
KRT80	1.155432	0.869528	1.535342	0.319213
KRT1	0.78658	0.626707	0.987237	0.03838
OTUB1	1.691274	1.156746	2.472807	0.006703
RCOR2	0.773646	0.540896	1.10655	0.159877
ANGPTL4	0.804836	0.545172	1.188178	0.274647
CD320	0.917047	0.747801	1.124597	0.405463
SPRYD3	2.162562	1.326602	3.525303	0.001978
IGFBP6	1.165444	0.842169	1.612813	0.355665
ZNF558	0.768479	0.508144	1.162192	0.212114
NDUFV1	1.725629	1.114192	2.672604	0.014509
CDK2AP2	1.118174	0.86085	1.452416	0.40255
NUDT8	1.513177	1.153829	1.98444	0.00275
TBX10	1.118781	0.862155	1.451793	0.398512
PRDX2	1.023159	0.874005	1.197766	0.775804
ZNF232	0.800551	0.575532	1.113547	0.186434
MIS12	1.109133	0.665705	1.847927	0.690867
CD300C	1.232957	1.085466	1.400489	0.001275
CD300A	1.513885	1.219645	1.879112	0.000169
TMEM88	1.179004	0.896698	1.550188	0.238328
EVPL	0.938636	0.827141	1.065161	0.326325
SRP68	1.488537	0.760244	2.914514	0.245899
MGAT5B	1.011484	0.775862	1.318663	0.932746
TMC8	1.311062	0.993909	1.729417	0.055273
TK1	1.337458	1.008688	1.773387	0.043377
TMEM68	0.707778	0.43984	1.138934	0.154452
TMEM99	1.082723	0.758945	1.544628	0.661075
GHDC	1.415	0.916925	2.18363	0.116843
ZNF598	1.18751	0.872645	1.615983	0.274252
RAB26	1.230889	0.942338	1.607797	0.127458
MLST8	1.505711	0.99597	2.276338	0.052281
E4F1	1.141127	0.803746	1.620127	0.460364
DNASE1L2	0.924519	0.641615	1.332164	0.673685
CASKIN1	0.451952	0.258228	0.791008	0.005421
ABCA3	0.819886	0.660053	1.018424	0.07266
KCTD5	1.738736	1.120208	2.698785	0.013662
SRRM2	1.097781	0.734814	1.640039	0.648757
ZNF597	0.732621	0.511257	1.04983	0.090069
NLRC3	1.005217	0.786711	1.284413	0.96681
SDHAF2	1.860665	0.932829	3.711372	0.07797
DDB1	1.390436	0.820843	2.355278	0.22028
VPS37C	1.645001	1.137248	2.379453	0.008221
VWCE	0.889186	0.731053	1.081524	0.239777
RAB3IL1	0.912413	0.74207	1.121858	0.384642
BEST1	1.183964	0.915995	1.530327	0.197125
FTH1	1.382779	1.094894	1.746359	0.006506
BSCL2	0.759847	0.380027	1.519284	0.437231
POLR2G	1.800307	1.041743	3.111232	0.035163
SLC3A2	1.390634	0.856343	2.258282	0.182518

ATG16L2	0.968948	0.685233	1.370132	0.858364
C2CD3	0.966113	0.614467	1.518997	0.881305
TTC21A	0.826065	0.55346	1.232939	0.349707
RPSA	1.108944	0.731894	1.680237	0.625723
CTNNB1	0.85979	0.569953	1.297018	0.471422
ULK4	0.537928	0.350258	0.826151	0.00462
FADD	2.149923	1.336654	3.458015	0.001596
LTBP3	0.968206	0.811198	1.155603	0.720406
NAALADL1	0.959593	0.82521	1.115859	0.592077
SAC3D1	1.229836	0.854773	1.769471	0.265036
BATF2	1.448718	1.140065	1.840933	0.002427
SF1	1.167952	0.668083	2.041829	0.585934
MAP4K2	1.240632	0.892329	1.724887	0.199696
CCDC88B	1.383873	1.12567	1.701302	0.002046
SCARA3	0.961282	0.810234	1.140488	0.650733
PBK	0.948231	0.742064	1.211675	0.670856
PNOC	1.627082	1.20333	2.200058	0.001565
COPS6	1.425628	0.840496	2.418114	0.188374
PAFAH1B2	0.841165	0.47209	1.498779	0.557264
ANKS3	0.750069	0.477512	1.178196	0.211949
NUDT16L1	1.278489	0.864085	1.891635	0.219028
KIAA1586	0.692438	0.487779	0.982966	0.039775
RAB4A	1.02649	0.63761	1.65255	0.9143
OR2B2	1.020081	0.635635	1.637048	0.934341
SETD5	0.569399	0.337544	0.960513	0.034774
VASN	0.863608	0.615255	1.21221	0.396667
THAP9	0.695261	0.42911	1.12649	0.139886
RNF187	1.582879	1.04648	2.394223	0.029619
HOOK3	1.150317	0.808879	1.635879	0.435732
MAPK1IP1	1.875973	0.861296	4.086024	0.113191
DDIT4	1.262129	1.110999	1.433816	0.000347
RBPJ	0.828094	0.516263	1.328273	0.433961
LMBRD1	1.23073	0.692596	2.186984	0.479094
ZCCHC4	0.591923	0.330612	1.05977	0.077629
PTGDR	1.099366	0.894968	1.350446	0.366713
TTC39C	1.368418	0.950106	1.970904	0.091987
GLYCTK	0.868598	0.542019	1.391949	0.558213
GNG4	0.719937	0.411532	1.259462	0.249511
UBTD2	1.067776	0.746887	1.52653	0.719139
POLR2J3	0.79814	0.49295	1.292275	0.359104
NKIRAS2	1.073162	0.626086	1.839484	0.797322
DNAJC7	0.850284	0.527749	1.369938	0.505106
KCNV2	1.004741	0.679242	1.486221	0.981109
IRF2BP2	0.771794	0.491289	1.212456	0.261004
NT5DC2	1.0185	0.858411	1.208444	0.833592
FOXI1	1.178135	0.698779	1.986328	0.538485
BMI1	1.147316	0.923231	1.425792	0.215153
THAP11	2.234747	1.326781	3.764071	0.002504
MMADHC	1.285436	0.777004	2.126561	0.32826
PDHB	0.831691	0.448514	1.542224	0.55859
PXK	0.649059	0.466455	0.903148	0.010337
PCMTD1	0.781711	0.518459	1.178633	0.239804
KCTD6	0.848365	0.567915	1.267307	0.421931
ACOX2	1.207975	0.959357	1.521021	0.108041
IRF2	1.458465	0.936518	2.271307	0.094965
CX3CR1	1.140115	1.03093	1.260863	0.010679
XIRP1	2.096813	0.401757	10.94349	0.379796
DEGS2	1.078641	0.829437	1.402719	0.572225

ARF4	0.718141	0.412007	1.251743	0.242842
FILIP1L	1.179947	0.835822	1.665754	0.346929
DTYMK	1.116381	0.692835	1.79885	0.651047
TAP1	1.276616	0.947914	1.719301	0.107879
ING5	0.820421	0.556461	1.20959	0.317645
ATG4B	1.05372	0.69461	1.598486	0.805604
MLKL	1.05918	0.773199	1.450935	0.720296
RFWD3	1.00044	0.680536	1.470722	0.998215
RHOH	0.929656	0.747524	1.156163	0.512055
KLHL30	1.682242	1.125416	2.514571	0.011211
COG7	1.050195	0.630999	1.747881	0.850539
CDC40	1.032169	0.632323	1.684856	0.899223
STIP1	1.624892	0.987636	2.673327	0.056006
HR	0.627544	0.382763	1.028864	0.064724
RAB31	1.172596	1.036264	1.326863	0.011575
REEP4	1.386271	0.93781	2.049185	0.101429
TNXB	1.028318	0.827726	1.27752	0.800873
BMP1	1.019506	0.771991	1.346378	0.891704
ATXN2L	1.1981	0.760001	1.888739	0.436422
POLR3D	0.834514	0.565189	1.232179	0.362889
FEN1	1.539595	1.080786	2.193176	0.016835
HEXIM2	1.090735	0.739587	1.608605	0.661281
FNTA	1.220534	0.626431	2.378081	0.558145
SERINC2	1.224808	1.063171	1.41102	0.004981
COL3A1	0.887176	0.729105	1.079518	0.231808
ING2	0.98654	0.579528	1.679403	0.960183
CDKN2AIF	0.807458	0.538542	1.210654	0.300709
SNRNP48	0.542072	0.305497	0.961849	0.036357
SLC20A2	0.538775	0.310974	0.933451	0.027415
TMUB2	1.557753	0.900174	2.695695	0.113169
STAT3	0.820701	0.507249	1.327849	0.420878
ZSWIM1	1.727897	1.056033	2.827211	0.029482
ADAM9	0.95628	0.758005	1.206418	0.706112
AXIN2	0.99046	0.56042	1.750493	0.973683
NDUFS5	1.219662	0.741815	2.005318	0.433785
ZNF30	0.840294	0.62221	1.134818	0.256375
UGT3A2	0.807792	0.713046	0.915128	0.000799
SLC16A4	0.720084	0.482279	1.075146	0.108344
IL7R	0.964064	0.800517	1.161023	0.699603
TMEM208	1.277511	0.702306	2.323821	0.422374
AHCYL1	1.839766	1.078396	3.13868	0.025293
DNAJC21	0.981223	0.549595	1.751834	0.948893
PKIG	0.967915	0.730964	1.281676	0.819925
FAM178B	0.957475	0.801483	1.143826	0.631981
SEMA4C	0.97128	0.807732	1.167943	0.756746
CNNM3	0.8276	0.515788	1.327912	0.432824
GSTM4	1.171319	0.815783	1.681807	0.391566
TET2	0.904502	0.712225	1.148687	0.410427
TCTN2	0.675187	0.455882	0.99999	0.049994
SHOX2	0.765393	0.385019	1.52155	0.44566
TSPAN5	1.08804	0.892669	1.326172	0.403386
ABHD15	1.394372	0.963833	2.01723	0.077656
ZBTB5	0.811157	0.534036	1.232079	0.326411
CHTF8	0.759575	0.35627	1.61943	0.476506
ADAL	0.998935	0.670048	1.489255	0.995829
LCMT2	1.546082	1.030382	2.319888	0.035333
SNTB2	1.033991	0.800599	1.335423	0.797877
IL12A	1.035154	0.826099	1.297112	0.764042

ZNF507	0.801581	0.605034	1.061976	0.123317
STX18	0.941977	0.523026	1.696514	0.842164
GFM1	1.024308	0.615267	1.705288	0.926419
DDX19A	0.920774	0.442954	1.914023	0.825028
ANKRD49	0.943483	0.583828	1.524697	0.812219
SFTPB	0.855987	0.400694	1.828612	0.68804
USP39	2.410637	1.103133	5.267881	0.02738
TNIP2	1.421904	0.951374	2.125148	0.08601
TMEM150,	0.801496	0.577018	1.113303	0.186905
RNF181	1.459378	0.872145	2.442007	0.15011
VAMP5	0.996552	0.809861	1.22628	0.973967
LRRC28	0.790065	0.619279	1.007951	0.057929
MAT2A	0.755897	0.555075	1.029373	0.075697
ENHO	1.095039	0.810589	1.479309	0.554115
ZNF608	0.807055	0.694646	0.937654	0.005092
INPP5D	1.021952	0.661331	1.579219	0.922099
LETM1	1.192185	0.716429	1.983872	0.498698
TMEM129	1.243419	0.823112	1.878349	0.300623
PPIC	1.830465	1.182817	2.832732	0.006656
SPRY3	1.337756	0.833349	2.14747	0.228191
CEP120	0.690708	0.426925	1.117472	0.131686
STXBP6	1.035561	0.654223	1.639177	0.881451
MFF	1.03718	0.525709	2.046271	0.916141
LGALS9	1.496188	1.133915	1.974203	0.004394
SIGLEC7	1.117763	0.966235	1.293053	0.134175
E2F6	0.75446	0.446817	1.273923	0.29181
FEM1B	1.213782	0.737135	1.99864	0.446423
COMMD8	1.20232	0.873953	1.654064	0.257579
UQCRFS1	1.265322	0.709074	2.257932	0.425781
MAP2K1	1.369249	0.99766	1.879241	0.05172
HNRNPH1	0.593638	0.377478	0.933578	0.023977
IRS1	0.833217	0.579395	1.198235	0.324954
MECP2	1.046845	0.613797	1.785421	0.866526
UPF3A	1.39358	0.833818	2.329122	0.205355
ROR2	0.768075	0.466897	1.263534	0.298825
BRD7P3	1.001376	0.761055	1.317584	0.992164
AR	0.879477	0.757565	1.021009	0.091627
DHRSX	0.575352	0.352031	0.940342	0.027428
HSPBAP1	1.078833	0.72383	1.607948	0.7094
ASMTL	1.191873	0.767075	1.85192	0.435016
SLC25A6	1.275608	0.904647	1.798686	0.165016
CHST14	0.95474	0.605916	1.504382	0.841758
PARM1	1.06023	0.78469	1.432523	0.703284
CSNK1G1	0.640266	0.438767	0.934301	0.020756
FAM110B	0.919748	0.746065	1.133864	0.433374
AFAP1L2	1.034896	0.830179	1.290096	0.760357
ZNF354A	1.047011	0.710149	1.543665	0.816592
ATF5	1.457887	1.001004	2.123303	0.049392
UBE2V2	0.975311	0.622629	1.527766	0.913067
ZBTB43	1.380429	0.896698	2.125111	0.143024
XPO6	1.804843	1.092853	2.980693	0.021063
MN1	1.029594	0.927288	1.143186	0.584942
APEX2	2.049177	1.232639	3.406615	0.005666
NSMCE1	2.336991	1.293023	4.223841	0.00494
CCDC126	1.019634	0.713343	1.45744	0.915044
IL13	1.18581	0.753391	1.866419	0.461487
CD2BP2	1.663414	0.937473	2.951494	0.081986
RGS14	1.595568	1.211145	2.102007	0.000894

TBC1D10B	1.783157	1.081318	2.940531	0.023433
LMAN2	1.085162	0.717677	1.640816	0.698441
RAB24	1.273732	0.921672	1.76027	0.142699
PRELID1	1.620951	1.156057	2.272797	0.005097
THBS3	1.200137	0.819974	1.756555	0.347886
CA5B	1.211725	0.87903	1.670338	0.240933
EFNA1	0.96161	0.793324	1.165594	0.690019
CXCL10	1.267911	1.084118	1.482862	0.00297
SH3TC2	1.458963	1.140166	1.866897	0.002676
CXCL11	1.639934	1.106544	2.430436	0.013726
ZRSR2	1.415438	0.910583	2.200199	0.122646
NMD3	0.853983	0.574703	1.268982	0.434734
ADRB2	0.888736	0.75361	1.04809	0.16098
B3GALNT1	1.028403	0.809896	1.305862	0.818237
GPRIN1	1.562597	1.038769	2.35058	0.03215
KCNAB1	0.975344	0.582436	1.633304	0.924389
MRPL1	0.988283	0.661167	1.477242	0.954172
SHE	1.063456	0.629554	1.796411	0.818088
PGM2	0.986663	0.649692	1.498409	0.94978
P2RY12	1.244896	1.009603	1.535025	0.040425
SLC33A1	0.751386	0.430371	1.311847	0.31475
SNUPN	1.177367	0.664692	2.085466	0.575641
CRADD	1.211063	0.814111	1.801563	0.344637
SIN3A	0.811579	0.461909	1.425952	0.467832
ARL13B	0.868396	0.533277	1.414108	0.570585
RNASE2	0.976988	0.878137	1.086966	0.66882
RNASE3	0.964731	0.875404	1.063173	0.468893
PTK2	1.188534	1.02745	1.374873	0.020105
PTAFR	1.144958	1.009401	1.298719	0.035247
PTPN9	1.642271	1.007765	2.67627	0.04648
RNASE6	1.130109	1.00834	1.266584	0.035488
SCN9A	0.966684	0.830399	1.125335	0.662098
SDC2	1.196386	0.987863	1.448924	0.066506
CD52	1.308494	1.141105	1.500437	0.000118
MMGT1	0.988032	0.607054	1.608105	0.961359
TM2D2	1.324527	0.705194	2.487787	0.382167
HTRA4	0.853176	0.581786	1.251163	0.416286
PLEKHA2	1.172135	0.777711	1.766597	0.447951
CLIC4	1.05526	0.819771	1.358396	0.676325
GPR183	1.058035	0.872057	1.283675	0.56735
CCDC8	0.984365	0.819604	1.182247	0.866101
ZEB2	0.968306	0.715205	1.310976	0.834958
PCBP1	1.354872	0.963478	1.905262	0.0808
HINT1	0.621275	0.421635	0.915442	0.016099
DTWD2	0.787567	0.541784	1.144851	0.21086
VPREB1	0.979794	0.877127	1.094477	0.717759
CLIC3	1.237611	1.002328	1.528124	0.047526
INO80E	1.519501	1.014739	2.275349	0.042256
DFFB	0.925516	0.635185	1.348552	0.686938
NFU1	1.244351	0.64618	2.396252	0.513195
CKAP2L	0.932367	0.708206	1.22748	0.617695
APLF	0.561941	0.323442	0.976304	0.040849
BOLA2B	1.464292	0.718342	2.98486	0.293922
RGPD8	0.888797	0.533431	1.480903	0.65086
HIC2	1.228954	0.805679	1.874602	0.338573
LUZP1	0.912821	0.650461	1.281002	0.597781
BUB1	0.866776	0.664054	1.131386	0.29288
SPNS1	0.894034	0.499818	1.599176	0.705773

LRRC45	1.140208	0.788886	1.647988	0.485069
AGPAT2	1.539429	1.089038	2.176089	0.014567
ASPSCR1	1.465087	0.91174	2.354268	0.114532
GP9	1.111783	0.948753	1.302827	0.190285
FASN	1.116254	0.85247	1.461663	0.423974
CNBP	1.563591	0.861474	2.837948	0.141646
MT1E	1.251649	1.060474	1.477288	0.007948
DUS1L	1.464753	0.965041	2.223224	0.073002
GPS1	1.586884	1.019961	2.468917	0.040599
RFNG	1.060659	0.758231	1.483714	0.730942
DCXR	1.390006	0.996566	1.938775	0.052416
ZNF32	1.148334	0.766739	1.719843	0.502132
LDB2	0.91777	0.518805	1.623545	0.768118
RAC3	1.002454	0.790398	1.271402	0.983877
NRG4	1.274764	1.047567	1.551236	0.015352
LIMS1	1.381393	1.003299	1.901971	0.047687
TAPT1	0.758444	0.472603	1.217169	0.251944
UGP2	0.736524	0.49276	1.100876	0.135883
HNRNPF	1.638667	0.996189	2.695504	0.051784
BTD	0.823194	0.514489	1.317129	0.417179
CSGALNA(	0.926869	0.649216	1.323268	0.675912
PCDH7	0.102053	0.000427	24.40927	0.414109
ROBO1	0.997493	0.832913	1.194593	0.978231
AVEN	0.629036	0.417399	0.947979	0.026742
P2RY1	1.254868	0.985863	1.597273	0.065139
CTNND2	0.899357	0.523596	1.544786	0.70074
TRIM56	1.272467	0.78878	2.052754	0.323376
AHSP	0.90746	0.825192	0.997929	0.045208
WNT10B	1.190577	0.885226	1.601257	0.248632
REPS2	1.143913	0.836547	1.564213	0.399714
SYAP1	1.276975	0.802515	2.031945	0.302237
ITGAM	1.205334	1.05506	1.377011	0.00598
PYDC1	1.089655	0.811194	1.463703	0.568512
TPST1	0.903572	0.745075	1.095787	0.302813
TOR1AIP2	1.372451	0.699511	2.692771	0.357211
S100G	0.773512	0.568836	1.051834	0.101484
TM4SF1	1.09966	0.96787	1.249396	0.144684
OTUD3	1.077521	0.681	1.70492	0.749791
GUSB	1.059495	0.758255	1.480413	0.73491
BRD3	0.755161	0.389664	1.463486	0.40548
KLF13	1.582246	1.102009	2.271762	0.012908
ZFPM2	1.319855	1.055685	1.65013	0.01487
ZNF764	0.873987	0.493381	1.548199	0.644301
ZNF747	1.106238	0.534454	2.289744	0.785607
ZNF768	1.698473	1.036576	2.783018	0.035506
TAS1R3	1.012875	0.689038	1.488911	0.948106
TMEM42	0.884128	0.53418	1.463333	0.631908
MAP3K2	0.794317	0.509045	1.239457	0.310421
PUSL1	1.861797	1.171922	2.95778	0.008496
SF3B5	1.481776	0.948859	2.313999	0.083785
ZNF35	0.782431	0.489011	1.251912	0.306264
IFFO2	1.326171	0.988632	1.778953	0.059609
NLGN2	1.001385	0.821136	1.2212	0.989094
MYO7B	0.929774	0.770953	1.121313	0.446127
CHD3	1.408795	1.029796	1.927277	0.032065
TMEM154	1.146957	0.89091	1.476592	0.28743
MYRIP	1.208552	0.876489	1.666417	0.247818
ALCAM	0.986802	0.757377	1.285725	0.921606

YWHAG	1.102815	0.685519	1.774132	0.686624
UBE2E3	1.069907	0.834688	1.371413	0.593724
CNTROB	0.893137	0.629357	1.267475	0.526867
TRAPPC1	1.730998	1.181826	2.535359	0.004833
KCNAB3	0.879429	0.601234	1.286347	0.507854
FAM153A	0.650989	0.397953	1.064916	0.08736
GPR37L1	0.884502	0.589246	1.327704	0.553707
TMEM192	0.769301	0.465267	1.272012	0.306678
ZNF778	0.771448	0.516493	1.152257	0.204931
NIPA1	1.233849	0.965308	1.577096	0.093343
FOXD4	0.777674	0.449036	1.346835	0.369533
GPR25	1.346464	1.057183	1.714902	0.015928
UBE2E1	0.536913	0.333809	0.863596	0.010325
HNRNPA3	0.638469	0.39498	1.032059	0.067074
SIK2	1.124025	0.685055	1.844276	0.643529
RNF150	0.851244	0.630449	1.149366	0.29313
CCDC144/	0.904624	0.744849	1.09867	0.312053
CHRNB1	0.645855	0.453516	0.919766	0.015366
GYPA	0.862947	0.728873	1.021685	0.08709
USP38	1.185751	0.735482	1.911681	0.484441
SLC16A5	1.09721	0.79155	1.520902	0.577635
NANP	1.386333	0.955488	2.011453	0.085399
PWWP2A	0.508023	0.317509	0.812852	0.004743
USP50	0.545179	0.316788	0.938232	0.028513
USP47	0.906049	0.562619	1.459113	0.68487
PDCD6IP	1.084357	0.644129	1.825458	0.76055
ZNF212	0.709357	0.41478	1.213143	0.209751
MRAP	0.700134	0.44828	1.093485	0.117093
FAM161A	0.801785	0.553734	1.160952	0.242107
ZNF282	0.953349	0.593141	1.532307	0.84358
GLB1	0.976336	0.646101	1.47536	0.909482
CRTAP	0.961161	0.625121	1.477842	0.856776
CNGB3	1.344205	0.327381	5.519206	0.681461
CMTM8	0.880824	0.708889	1.09446	0.252079
GABARAP	0.869704	0.546473	1.384122	0.555968
STX8	1.127646	0.595379	2.135759	0.712385
UBB	1.174151	0.720958	1.91222	0.518815
NFRKB	1.040484	0.708379	1.528287	0.83967
FABP4	0.919467	0.762866	1.108214	0.378118
PRDM10	0.82958	0.50903	1.351989	0.453401
B3GNT2	0.952649	0.678118	1.338322	0.779713
FOS	0.964032	0.810698	1.146366	0.678538
TMED10	1.134132	0.67078	1.917552	0.638546
SETMAR	0.754906	0.497152	1.146296	0.18707
SMAD1	1.05164	0.864434	1.279387	0.61467
EMX2	1.462765	0.850337	2.516274	0.169383
SP7	0.84508	0.522754	1.366149	0.492176
LRRN2	1.029523	0.837392	1.265736	0.782488
SLC30A1	1.33132	0.988731	1.792616	0.059389
DCLK2	0.809008	0.654017	1.00073	0.050792
ZNF804A	0.889885	0.762599	1.038416	0.138523
GPRC5C	1.16	0.878755	1.531257	0.294805
TMEM182	0.990564	0.654985	1.498074	0.964169
KRT8	1.035654	0.877107	1.222859	0.679432
ADORA2B	0.851536	0.631821	1.147655	0.291203
MGMT	1.194905	0.880587	1.621416	0.252872
METTL7B	1.337214	1.181315	1.513688	4.34E-06
NFXL1	0.672992	0.429486	1.054559	0.083962

DENND5B	0.803338	0.605501	1.065814	0.128992
CD14	1.081197	0.999776	1.169249	0.05066
DNAJC18	0.938525	0.685976	1.284052	0.69159
RALGAPB	0.747905	0.474678	1.178403	0.210474
SLC23A1	0.92856	0.716191	1.203901	0.575877
KRT72	1.088977	0.903583	1.31241	0.370688
KISS1	0.396951	0.064195	2.454545	0.320239
NUDT9	0.875011	0.530285	1.443833	0.601302
HSD17B13	0.624927	0.432325	0.903334	0.012393
PA2G4	1.486301	0.858351	2.573645	0.157157
ELOVL6	1.09247	0.908031	1.314373	0.348552
PFKFB3	1.032577	0.797112	1.337598	0.808188
ARL6IP1	0.883844	0.572217	1.365182	0.577774
SERPINB9	1.092825	0.937932	1.273297	0.255006
SMAGP	0.942835	0.704179	1.262375	0.692625
IRX1	0.718506	0.5936	0.869695	0.000692
CDH2	0.931478	0.758007	1.144647	0.499612
EMB	1.242303	0.935186	1.650277	0.13427
STAT2	1.232175	0.823707	1.843199	0.309585
NUDCD2	0.844476	0.510705	1.396383	0.510045
IRF2BP1	1.088954	0.759996	1.560298	0.642366
HSPA4	1.098252	0.719559	1.676246	0.663986
COMMD5	1.171974	0.729088	1.883893	0.512291
GTSF1	0.992603	0.907198	1.086048	0.871507
DPY19L2P	0.916021	0.705496	1.189368	0.510313
ZNF16	0.897482	0.549943	1.464648	0.665132
ARMC10	0.626738	0.371301	1.057905	0.080253
RNF34	1.020472	0.565631	1.841064	0.946334
ACYP2	0.833158	0.413591	1.678352	0.60947
TRABD	1.47734	1.138847	1.916442	0.00329
ATF7	1.71693	0.849041	3.471975	0.132457
SOCS6	0.753308	0.560828	1.011848	0.059874
ZNF296	1.695176	1.236313	2.324349	0.001049
HOXB9	1.187616	1.045102	1.349564	0.008381
TTLL6	0.857004	0.628912	1.167819	0.328386
POLH	1.087165	0.668547	1.767904	0.736204
KIF5B	1.026906	0.672304	1.568541	0.902227
AKAP13	1.229378	0.870689	1.735831	0.240693
CDCA4	1.508692	1.00893	2.256005	0.045148
CHCHD7	1.123769	0.822794	1.534841	0.463164
HTRA3	1.107597	0.981849	1.249449	0.096503
FOXN2	0.971849	0.683661	1.381519	0.873579
LMOD2	0.88579	0.651819	1.203743	0.43835
USP32	0.744829	0.491127	1.129587	0.165598
CEL	1.126138	0.893434	1.41945	0.314486
PPM1D	0.796672	0.468253	1.355434	0.401838
GPR27	1.061805	0.945272	1.192705	0.311983
KBTBD2	0.665435	0.403521	1.097351	0.110497
TRIAP1	1.226684	0.764716	1.967728	0.396775
LSM3	1.780148	1.007742	3.144581	0.046975
KIAA0232	0.939404	0.614314	1.436529	0.773003
MTSS1	1.150803	0.972325	1.362043	0.102351
TMEM43	1.572957	0.932281	2.653916	0.089655
RNF139	0.942591	0.605964	1.466221	0.793104
RPS9	1.792417	1.140822	2.816178	0.011358
PLA2G1B	0.875839	0.623093	1.231105	0.445378
CYTL1	0.884879	0.807978	0.969099	0.008374
TSEN34	1.624113	1.099929	2.398103	0.014729

TRH	0.869485	0.802299	0.942297	0.000653
GSTA4	1.094735	0.878548	1.36412	0.420025
NDUFA3	1.691706	1.004837	2.848093	0.047915
OSCAR	1.073802	0.946402	1.218351	0.269139
PAQR8	1.502125	1.153664	1.955838	0.002516
NUDT6	1.070529	0.725523	1.579595	0.731317
TANC2	1.0719	0.85744	1.339999	0.542126
DNAJC24	0.714636	0.392643	1.300687	0.271514
ZNF160	0.722405	0.484171	1.077862	0.111226
ZNF415	0.934953	0.758245	1.152842	0.529174
CEACAM3	1.066774	0.905432	1.256867	0.439766
PDGFD	1.170661	1.002407	1.367155	0.046554
S1PR1	0.961869	0.770899	1.200148	0.730636
PKIA	1.017202	0.841677	1.229332	0.859913
XKR6	1.127498	0.898095	1.415499	0.30117
TSNARE1	1.08951	0.794112	1.494793	0.59522
FPR2	1.102523	0.972723	1.249644	0.126708
FPR1	1.096742	0.99416	1.209909	0.06532
FEZ2	0.915637	0.512917	1.634553	0.765637
SOX7	1.29425	0.585049	2.863151	0.524318
ALK	2.02401	1.171008	3.498369	0.011557
MTM1	0.724066	0.478973	1.094575	0.125679
INSR	0.640133	0.486108	0.842962	0.001491
MFN1	0.818941	0.485222	1.382179	0.454479
GIMAP8	1.119145	0.974106	1.28578	0.111948
NRTN	0.882467	0.597614	1.303095	0.529528
KCNMB3	0.781443	0.472669	1.291927	0.33634
FUT3	0.887224	0.174189	4.519029	0.885453
ATP6V0E2	1.921841	1.435065	2.573732	1.17E-05
PRKCE	1.171113	0.929715	1.47519	0.179864
JAGN1	1.079087	0.687753	1.69309	0.740499
TADA3	1.662595	1.130507	2.445118	0.009787
SOCS5	0.992653	0.568862	1.732158	0.979288
C1GALT1C	1.024522	0.650775	1.612916	0.916671
MORN4	1.133575	0.877394	1.464556	0.337439
ZNF672	1.763866	1.155227	2.693169	0.008583
ZNF692	1.207478	0.793706	1.836957	0.378481
NAIF1	1.495294	0.853544	2.619552	0.159607
RBKS	1.121071	0.83584	1.503639	0.445514
GRIK1	0.803492	0.537202	1.201782	0.286815
TMEM126A	0.883151	0.490717	1.58942	0.678543
TMEM126B	0.999785	0.533069	1.875125	0.999467
TRIM8	1.536364	1.107268	2.131748	0.010178
NETO2	0.988415	0.824659	1.184689	0.899663
CLDN20	0.667929	0.485837	0.918268	0.012956
CDC42BPG	0.960704	0.731515	1.261699	0.773124
SCAND1	1.409185	1.016461	1.953642	0.039598
JUNB	1.165162	0.935212	1.451652	0.172946
TMEM37	0.950637	0.699332	1.292248	0.746555
LRG1	1.210149	1.024209	1.429846	0.025026
SHCBP1	1.145908	0.843493	1.556746	0.383635
SOSTDC1	1.015415	0.83561	1.233908	0.877737
NPTX1	1.017521	0.748217	1.383754	0.911828
FAM98B	1.183713	0.789013	1.775858	0.415111
ZNF439	0.676883	0.540206	0.848141	0.000696
ZNF440	0.576134	0.391615	0.847593	0.005119
GAA	1.110596	0.909437	1.356248	0.303544
CANT1	1.480401	0.833661	2.62887	0.180565

ZDHHC16	1.30274	0.691049	2.455874	0.413603
CHST11	0.739822	0.511915	1.069195	0.108743
EXOSC1	1.243128	0.573687	2.693744	0.581227
PGAM1	1.438728	1.057024	1.95827	0.020749
CHD7	0.828521	0.680922	1.008115	0.060214
ESCO2	1.080485	0.797459	1.463958	0.617416
KRT19	0.81588	0.049754	13.37914	0.88662
CLCN5	1.649196	1.312126	2.072855	1.80E-05
TPPP	1.307555	0.991644	1.724107	0.057368
KCND3	0.972734	0.4473	2.115382	0.944398
APLN	0.854673	0.640661	1.140174	0.285567
PDE7B	1.495065	1.205381	1.854366	0.000252
MRPL36	1.928983	1.112701	3.344094	0.019264
ZNF581	1.126712	0.756903	1.677203	0.556683
NAT1	1.511906	0.887388	2.575944	0.128384
GLOD5	0.840194	0.695399	1.015138	0.071188
ZNF524	1.295892	0.904262	1.857136	0.158005
MCC	0.768996	0.471774	1.253472	0.292022
ZBTB26	0.942824	0.640285	1.388316	0.76555
DSEL	1.06947	0.64201	1.78154	0.796442
POLR1C	0.932285	0.565215	1.537743	0.783613
ASXL1	0.831851	0.508299	1.361357	0.463845
DLK2	1.180298	0.745363	1.869026	0.47967
ZNF562	0.69785	0.453145	1.074701	0.102478
ZNF318	1.170987	0.874199	1.568534	0.289857
ZNF561	1.022487	0.663366	1.576022	0.91976
WIPF2	0.926624	0.468541	1.832566	0.826624
HOPX	1.228237	1.090487	1.383387	0.000706
LRRC8C	1.091864	0.792169	1.504941	0.591378
RSL1D1	0.933829	0.648893	1.343882	0.712417
LRRC8D	1.399362	0.858196	2.28178	0.177993
PPID	1.277223	0.745793	2.187332	0.372704
COL24A1	1.147616	0.964875	1.364966	0.119732
ETFDH	1.570879	0.894844	2.757645	0.11572
RXFP1	1.06858	0.959832	1.189649	0.225785
LPAR3	1.816274	1.173846	2.810294	0.007369
PTGER4	1.013766	0.821707	1.250716	0.898482
TBCA	0.850017	0.509953	1.416854	0.533056
ECEL1	1.004324	0.65165	1.547866	0.984401
BCL2L1	1.139377	0.859754	1.509943	0.363774
PLRG1	1.085687	0.652924	1.805288	0.751336
ZNF584	1.077757	0.699322	1.66098	0.734366
NMUR1	1.128801	0.950307	1.34082	0.167716
CLSTN1	1.085057	0.763036	1.542979	0.649519
CXXC5	1.158636	0.896979	1.496621	0.259548
ZNF274	1.058846	0.701494	1.598239	0.785469
PIK3CD	1.122785	0.775658	1.625261	0.5394
PTCRA	1.051386	0.842352	1.312293	0.657721
SLC25A33	0.791109	0.515253	1.214653	0.284132
ENC1	1.095238	0.891627	1.345345	0.386002
SPSB1	1.053688	0.796422	1.394057	0.714239
P2RY6	1.103338	0.869182	1.400574	0.41909
BPTF	0.673418	0.459452	0.987029	0.042673
S100Z	1.121465	0.927671	1.355742	0.236286
ZIK1	1.034338	0.809934	1.320917	0.786722
GPR82	0.912638	0.665452	1.251643	0.570558
GPR34	0.902941	0.740209	1.101448	0.31395
PLEKHG5	1.077773	0.785425	1.478937	0.642709

ATF7IP	0.859295	0.582866	1.266823	0.443848
RGS19	1.632911	1.196832	2.227881	0.001978
TCEA2	1.602162	1.123423	2.284913	0.009253
HDAC3	1.742402	1.015538	2.989512	0.043806
GPHN	0.80094	0.658523	0.974156	0.026278
VAT1L	1.121229	0.954462	1.317135	0.163713
TMEM51	1.031175	0.883125	1.204044	0.697853
CAMTA1	1.247011	0.679944	2.287005	0.475611
LGALS4	0.850552	0.56792	1.273841	0.432176
LRRC34	0.818833	0.601713	1.114299	0.203553
SPATA5L1	1.38166	0.715781	2.666991	0.335322
GATM	0.959167	0.817372	1.12556	0.609501
RASGRP4	1.295882	1.06667	1.574348	0.009058
SLFNL1	0.947538	0.687531	1.305873	0.741945
BCL2	0.739439	0.571938	0.955995	0.021259
UTF1	1.892716	1.479646	2.421101	3.80E-07
COL8A2	1.109173	0.923409	1.332308	0.267891
PWWP2B	1.723647	1.280434	2.320276	0.000331
ZNF540	0.886519	0.594289	1.322448	0.55499
ANGPTL7	0.825419	0.571472	1.192213	0.306417
FBXL14	1.077986	0.764101	1.520814	0.668891
EXOSC10	1.287624	0.751876	2.205119	0.357055
ZNF570	0.863165	0.527602	1.412151	0.557955
NINJ2	1.640697	1.210091	2.224533	0.001434
MLLT3	1.059149	0.905512	1.238854	0.472343
FAM90A1	1.006825	0.708421	1.430924	0.969747
RRM2	1.023358	0.783083	1.337357	0.865713
IFNB1	1.00446	0.496998	2.030071	0.990109
RPS21	1.187532	0.818077	1.72384	0.366033
C3AR1	1.090024	0.940203	1.26372	0.25319
PTEN	0.866768	0.586182	1.281662	0.473699
RPS7	1.042524	0.667837	1.627428	0.854584
RNASEH1	1.267837	0.678991	2.36735	0.456367
PRNP	1.187343	0.891495	1.581371	0.240222
FRMD5	0.920549	0.66431	1.275626	0.618924
CYP4F11	0.68485	0.512019	0.91602	0.010739
TLN2	1.157625	0.798441	1.678391	0.439938
ZNF217	0.972285	0.699844	1.350783	0.86694
SCG2	1.155999	0.777078	1.719691	0.474386
ATPAF2	0.889461	0.517043	1.530125	0.672137
CYP4F22	1.105969	0.84464	1.448153	0.463968
PPIH	1.311872	0.614878	2.798941	0.482616
ZNF57	0.383182	0.215684	0.680756	0.00107
JMJD1C	1.121085	0.820182	1.532382	0.473499
SYNPO	0.978482	0.68629	1.395076	0.904323
MAL	1.051749	0.877048	1.261249	0.586165
ZNF554	0.653631	0.413816	1.032423	0.06828
RAB33B	1.172232	0.746584	1.840556	0.489973
THOP1	1.382629	0.941524	2.030392	0.098411
LAMB2	0.954045	0.862058	1.055848	0.363125
USP19	1.165459	0.729926	1.860866	0.521302
ORMDL3	0.796543	0.570652	1.111852	0.181272
KLF11	1.166087	0.994618	1.367118	0.058297
SMN1	0.484627	0.297487	0.78949	0.003623
EIF2AK3	0.657133	0.416133	1.037705	0.071673
KRCC1	0.854898	0.601465	1.215116	0.382179
NME6	0.85609	0.5523	1.326976	0.487152
CYCS	1.135821	0.750468	1.719047	0.546956

CD8B	1.027591	0.760292	1.388867	0.859455
SLFN12	1.030984	0.835006	1.272957	0.776667
FRMD3	0.998402	0.834011	1.195196	0.986101
SNTB1	0.994142	0.700889	1.410094	0.973721
MTBP	0.685401	0.440661	1.066069	0.09372
MRPL13	1.507726	0.903416	2.51627	0.116119
MALT1	1.168591	0.81673	1.672041	0.394005
PRL	1.28635	1.012979	1.633495	0.038851
ISG20	1.344834	1.055983	1.712697	0.016328
MBOAT1	0.846892	0.57173	1.254484	0.407117
GPR22	0.838121	0.61702	1.138451	0.258422
CXCR6	0.781581	0.553807	1.103036	0.160904
CEBPB	1.287777	1.076561	1.540433	0.005656
AZU1	0.945107	0.879276	1.015868	0.125377
TPSAB1	0.906267	0.825437	0.995011	0.038933
PAIP1	0.847971	0.49367	1.456548	0.550197
CLEC7A	1.126302	1.022807	1.24027	0.015584
C1QTNF4	0.98411	0.890871	1.087107	0.752459
SERHL	1.385532	0.856991	2.240045	0.183405
NEGR1	0.979296	0.869171	1.103374	0.731046
ZNF131	0.967726	0.515777	1.815696	0.918614
MACROD2	1.071178	0.797024	1.439633	0.648491
DPAGT1	1.49778	0.858533	2.612999	0.154802
BSG	0.902751	0.656821	1.240764	0.528369
HINFP	0.927541	0.540179	1.592681	0.785094
SPTLC3	0.835001	0.679406	1.02623	0.086552
TP53RK	1.15321	0.76227	1.744649	0.49977
CLEC12A	1.032177	0.864	1.23309	0.727082
BPGM	0.848887	0.645866	1.115725	0.240094
POP7	1.556702	0.971437	2.494575	0.06584
ALG14	1.340055	0.650076	2.762368	0.427726
SUCLG2	1.240184	1.041746	1.476423	0.015534
STARD5	1.206514	0.668086	2.178877	0.5336
CSDC2	1.4904	0.79243	2.803138	0.215673
IL16	1.20139	0.849331	1.699381	0.299742
GNB2	1.287683	0.912442	1.81724	0.15026
PDZD3	1.009075	0.607738	1.675445	0.972143
C2CD2L	1.318784	0.899154	1.934253	0.156777
GNG12	1.017963	0.68744	1.5074	0.929176
PRSS27	1.027738	0.645782	1.635607	0.908122
DNAJB7	0.56641	0.299221	1.072187	0.080831
CLP1	1.537701	0.836756	2.825824	0.16577
TTC36	0.804955	0.610652	1.061085	0.123729
RSPH9	1.6849	0.965106	2.941532	0.066501
GTPBP2	1.267054	0.81698	1.965075	0.290444
FGGY	1.183564	0.665892	2.103681	0.565765
IL17D	0.692116	0.395722	1.210509	0.196985
TCEAL1	0.854583	0.545573	1.338614	0.492528
ZNF24	0.903288	0.608171	1.341613	0.614298
MANEA	0.969082	0.680714	1.379612	0.861652
AGXT	0.961758	0.752922	1.228518	0.754898
AFF1	1.097966	0.739543	1.630098	0.642989
ACOT12	0.91757	0.469803	1.792102	0.801138
FIBP	2.913425	1.852129	4.582861	3.72E-06
BANP	0.973802	0.557474	1.70105	0.92568
PPP1CA	1.706156	1.139492	2.554618	0.009486
HCFC1	1.144164	0.789258	1.658661	0.477189
CTSW	0.971408	0.864738	1.091238	0.624997

NIPAL4	0.765513	0.380782	1.538966	0.453272
PDE3A	1.015384	0.734478	1.403723	0.926387
RASGRP1	0.957962	0.743723	1.233915	0.739495
KLHL6	1.154149	0.813859	1.63672	0.421192
CHCHD1	1.930789	1.022712	3.645157	0.042435
MRPL52	1.37502	0.741559	2.549602	0.312074
SMPDL3A	1.123651	0.972766	1.29794	0.113045
RND1	1.968757	0.958279	4.044755	0.065189
RAD9A	1.127388	0.746831	1.701863	0.568233
EFEMP2	0.764367	0.585946	0.997117	0.047563
OR10AD1	1.543213	0.977444	2.436463	0.062596
TMEM134	0.879808	0.559835	1.382661	0.57877
ZMAT3	0.749098	0.589302	0.952225	0.018282
ZNF738	0.684357	0.476334	0.983227	0.040223
SLFN11	0.908528	0.735137	1.122815	0.374622
CORO1B	1.037855	0.760916	1.415589	0.8145
FUT10	0.909801	0.702609	1.17809	0.473411
LRRC20	1.407729	1.085904	1.824932	0.009815
MUS81	1.401716	0.877761	2.238431	0.157355
TMEM217	1.290219	1.043699	1.594966	0.018507
ZNF596	0.666751	0.394718	1.126266	0.129668
CFL1	2.190713	1.474164	3.255555	0.000104
TMCC1	0.782437	0.414806	1.475889	0.448608
RAB43	0.844952	0.578623	1.233866	0.383148
CBWD1	0.651734	0.366244	1.159763	0.145414
RAB37	1.200933	0.956877	1.507237	0.114184
DCP2	0.612046	0.431843	0.867446	0.005795
SNX32	1.30904	0.914954	1.872865	0.140589
RPL38	0.935734	0.575355	1.521838	0.788938
CYP7B1	1.127899	0.999708	1.272528	0.050557
RARG	1.631184	1.250068	2.128493	0.000313
CES3	1.100829	0.888012	1.364648	0.380811
SSH3	1.006745	0.724757	1.398449	0.96802
CES2	1.343289	0.784463	2.300204	0.282201
PDP2	1.00207	0.694424	1.446009	0.991184
SP3	0.57668	0.336769	0.987504	0.044879
DMXL1	0.726724	0.505636	1.044482	0.084563
ZNF621	0.960987	0.632524	1.460018	0.852067
EGFL7	1.056689	0.925026	1.207092	0.416719
NADSYN1	0.857279	0.578484	1.270437	0.442905
DHCR7	1.089179	0.868188	1.366423	0.460323
NBEA	0.887516	0.700697	1.124145	0.322397
RNASEH2(	1.331232	0.937485	1.890353	0.109788
MYEOV	2.248536	1.026405	4.925846	0.042857
ANKRD13I	0.95189	0.708189	1.279454	0.743847
MYD88	1.218145	0.921894	1.609595	0.165152
MRGPRD	0.745909	0.328827	1.692012	0.482999
OXSR1	0.862275	0.517131	1.437776	0.570003
SLC22A13	0.987802	0.532217	1.833376	0.968974
PHF8	0.809912	0.477796	1.372883	0.433632
LCLAT1	1.166256	0.726053	1.873351	0.524749
KAT5	1.288994	0.759644	2.187217	0.346714
SH3RF3	1.031876	0.832222	1.279428	0.774877
GXYLT2	0.737726	0.585929	0.928851	0.009656
HPSE2	1.126565	0.81954	1.54861	0.462898
DCAKD	1.069495	0.669691	1.70798	0.778482
TADA2B	1.139293	0.754103	1.721235	0.535637
CCDC96	0.972318	0.603177	1.56737	0.908259

RELA	1.544493	0.924026	2.581592	0.097217
EVC2	0.972725	0.77493	1.221005	0.811551
ZNF680	0.608533	0.437369	0.846683	0.003202
RXFP4	0.88313	0.68625	1.136492	0.33417
HPSE	1.126318	0.95731	1.325164	0.151566
COQ2	0.833328	0.490556	1.415611	0.500059
HSPA6	1.190135	0.99444	1.424339	0.057546
TRMT112	1.77603	1.163913	2.710065	0.007723
LRRN3	0.701206	0.483466	1.017011	0.061339
ADCK5	1.201595	0.843923	1.710856	0.308351
NOC3L	1.086849	0.771485	1.531125	0.633875
ESRRA	1.576025	1.080754	2.298259	0.018106
COMMD1	1.276333	0.651126	2.501859	0.477379
RAPH1	1.005658	0.591378	1.710157	0.983383
MTX1	2.305575	1.317557	4.034492	0.003434
PARP14	1.313925	1.012529	1.705036	0.040012
CYSLTR1	1.19446	0.9005	1.584381	0.217639
PARP15	1.261351	0.962278	1.653376	0.092668
CKS1B	1.330944	0.897416	1.973904	0.15511
ABCD2	1.126752	0.775103	1.637938	0.531816
ABLIM3	1.260366	1.025709	1.548708	0.027704
VANGL1	0.975732	0.801051	1.188503	0.807153
GLRX	0.983091	0.672851	1.436377	0.929756
IQCB1	0.976859	0.614272	1.553469	0.921202
GOLGB1	0.82358	0.573314	1.183093	0.293616
ZNF483	0.513672	0.284429	0.927681	0.027184
PLAC8L1	0.944449	0.53063	1.680989	0.845944
SLC2A14	1.064597	0.835652	1.356265	0.612381
GPR137	1.488989	0.984283	2.25249	0.059436
SNCG	1.943668	1.145656	3.29754	0.013734
MMRN2	0.904806	0.609157	1.343947	0.620208
TNKS	0.795839	0.518922	1.220529	0.29528
ZNF449	1.240618	0.798567	1.927368	0.33744
PPP1R3B	0.943702	0.682841	1.304218	0.725578
STOX2	0.642938	0.487601	0.847763	0.001745
MAP3K11	1.731741	1.190357	2.519351	0.004092
TRIB1	1.190588	0.994649	1.425126	0.057239
KCNK7	1.031307	0.724171	1.468707	0.864307
C1QB	1.051665	0.948667	1.165846	0.338113
C1QA	1.087112	0.983159	1.202055	0.103363
OLR1	1.035213	0.877469	1.221314	0.681599
GLIPR1L1	1.015987	0.753117	1.370608	0.917306
DAG1	1.249469	0.920222	1.696518	0.153523
ARV1	1.060775	0.658886	1.707797	0.808136
SAA1	0.849024	0.433432	1.663101	0.633286
EHBP1L1	1.618046	1.222739	2.141155	0.00076
THAP2	1.069637	0.722935	1.58261	0.736266
RNF26	1.522263	0.956521	2.422617	0.076319
PPP1R14B	1.29425	0.923659	1.81353	0.133983
SMARCC1	0.674283	0.454307	1.000771	0.050449
ZNF417	0.873253	0.590713	1.290932	0.496786
PTPRM	1.02877	0.889844	1.189386	0.701568
FKBP2	1.328711	0.684844	2.577918	0.400646
VEGFB	1.135156	0.794062	1.62277	0.486885
TNFRSF10	0.919787	0.684353	1.236216	0.579397
MST1	0.791312	0.630519	0.993109	0.043422
TNFRSF10	1.029423	0.859708	1.232643	0.752401
GMPPB	1.057209	0.708542	1.577452	0.78526

ZNF622	1.415641	0.830684	2.412518	0.201274
CSPG4	1.342943	1.094641	1.647569	0.0047
SNX33	0.963774	0.757407	1.226369	0.764075
CHD2	0.930023	0.503672	1.717275	0.816658
CCDC106	1.219002	0.947102	1.56896	0.124074
CCR9	1.026163	0.73341	1.435773	0.880205
SULT1B1	0.985169	0.820862	1.182365	0.872481
NUDT4	0.787027	0.487399	1.27085	0.32729
PC	0.977724	0.767032	1.246291	0.855641
NMNAT1	1.460339	0.911839	2.338779	0.115053
LRFN4	0.96941	0.78879	1.191388	0.767745
SLC19A1	1.184429	0.779447	1.79983	0.427876
HSPB7	1.78693	1.14204	2.795978	0.01104
RCE1	1.105304	0.743945	1.642187	0.620141
UQCRH	1.568509	0.897654	2.740724	0.113927
TAS1R1	0.735142	0.441751	1.223392	0.236389
EIF1AX	1.03043	0.680079	1.561268	0.88756
PSMD1	1.728019	0.926134	3.224208	0.085646
SUSD5	0.736732	0.352691	1.53895	0.416258
HEG1	1.094513	0.895042	1.338438	0.378986
TOMM20	1.024268	0.677753	1.547945	0.909392
AGFG1	0.967207	0.656264	1.425478	0.866195
STAT5B	0.673565	0.466143	0.973283	0.035365
CD7	1.024983	0.883303	1.189389	0.745095
CNP	1.01375	0.557372	1.843811	0.96431
JUP	0.919447	0.800645	1.055877	0.234157
HAP1	1.119925	0.265142	4.730404	0.877548
EIF1	1.107699	0.676026	1.815017	0.68476
RNF213	1.165437	0.856799	1.585254	0.32939
TIGD3	1.15561	0.74835	1.784505	0.514159
PLK3	1.21032	0.912512	1.60532	0.185294
NET1	1.196188	0.936741	1.527493	0.150977
DPY19L1	0.749388	0.555329	1.011261	0.059197
PHOSPHO	1.08063	0.914171	1.277399	0.363587
ZNF791	0.837919	0.432026	1.625153	0.600833
PHC3	0.646226	0.409793	1.019072	0.060289
GPR160	1.076064	0.839127	1.379902	0.563443
CBX2	0.853783	0.736254	0.990074	0.03644
SPTBN2	0.782226	0.581618	1.052026	0.104268
GOLIM4	1.174827	0.846393	1.630706	0.335501
RBM4B	0.695985	0.409286	1.183513	0.180908
HOXB2	1.174845	1.043985	1.322108	0.007487
SLCO4C1	1.043938	0.892111	1.221604	0.591791
RBM4	0.574249	0.354501	0.930214	0.024202
UBXN2A	0.688282	0.438929	1.079291	0.103627
RAX2	0.278111	0.07436	1.040153	0.05724
TCAP	0.889747	0.618054	1.280875	0.529752
CCS	1.33741	0.977932	1.829027	0.06872
KLHL15	0.834097	0.553021	1.258032	0.386942
FBXO45	1.178513	0.79445	1.748244	0.414306
GNG5	1.037385	0.705089	1.526288	0.852209
SLC25A30	0.995242	0.710699	1.393709	0.977855
CD34	1.01811	0.945256	1.09658	0.635652
CTSF	0.94084	0.804231	1.100653	0.446153
MSRB3	0.934999	0.780333	1.12032	0.466313
LEMD3	1.09969	0.665366	1.817525	0.710868
TLR10	1.049178	0.843179	1.305506	0.66686
TLR1	1.063916	0.873208	1.296275	0.538732

TLR6	1.199534	0.988038	1.456303	0.066009
FAM174A	0.839832	0.468847	1.504367	0.557272
RGMB	1.201474	0.344815	4.186426	0.773199
FAM53A	1.081513	0.621371	1.882405	0.781676
CYB561D1	0.857118	0.549055	1.338028	0.497456
ZDHHC24	0.799994	0.515388	1.241762	0.319864
SELP	1.096066	0.938769	1.27972	0.245829
CTU2	1.329783	0.835588	2.11626	0.229256
MGA	0.688072	0.490793	0.964651	0.030103
ARL13A	0.681342	0.453827	1.022914	0.064215
PIGG	0.911591	0.609301	1.363856	0.652486
PRPF8	1.111672	0.755452	1.63586	0.591184
ADCY6	1.080335	0.922317	1.265425	0.338213
PITPNA	1.502355	0.923925	2.442915	0.100803
DDX23	1.684153	0.937749	3.024657	0.081016
ZNHIT2	1.51879	0.99545	2.317267	0.052525
ZBTB4	1.194842	0.7739	1.844744	0.421792
TNK1	1.112877	0.86955	1.424293	0.395559
ZHX3	1.339339	0.955271	1.877823	0.090158
PHLDA3	0.951103	0.735962	1.229135	0.7016
SLC16A11	1.334094	0.837587	2.124921	0.224856
SLC16A13	1.479944	1.053488	2.079031	0.023795
STAG3L3	0.628037	0.453182	0.870358	0.005206
SLC6A19	1.094093	0.868786	1.37783	0.444649
SNHG11	1.266108	0.823565	1.946451	0.282235
EXO1	1.181707	0.878395	1.589753	0.269931
RALGAPA1	0.521332	0.308582	0.880764	0.014912
LIG4	1.057593	0.728673	1.534985	0.768294
GTF2IRD2I	0.813002	0.453214	1.458411	0.487465
ABRA	1.127516	0.74551	1.705267	0.569632
ATP2A2	0.881131	0.58123	1.335771	0.551076
ZWILCH	1.228088	0.775562	1.944654	0.38096
RPL4	1.221576	0.83378	1.789739	0.304381
SNAPC5	1.416045	0.753603	2.660794	0.279725
ZCCHC12	0.361798	0.080964	1.616745	0.183185
GALNTL6	0.767463	0.423729	1.390037	0.382502
LINGO2	1.351921	0.555962	3.287438	0.505997
BBS1	0.534941	0.294423	0.971939	0.040035
DENND4A	0.838517	0.540192	1.301595	0.432431
PELI3	1.446866	1.099042	1.904768	0.00846
TTC9B	1.035905	0.514347	2.086334	0.921336
MYO1H	0.878318	0.344376	2.240119	0.785924
TMEM81	1.151616	0.650883	2.037568	0.627747
MRPL11	2.189973	1.26617	3.787784	0.005044
IL20RB	0.926763	0.577886	1.486262	0.752299
AKIRIN1	0.981194	0.532194	1.809005	0.951498
MSL2	0.588137	0.347802	0.994545	0.047662
ZNF497	0.762576	0.556571	1.044832	0.091602
CMKLR1	1.179463	0.990732	1.404147	0.063552
ANGEL2	0.713226	0.418861	1.214463	0.213324
UGT8	0.828831	0.572656	1.199605	0.319633
KY	0.92326	0.639257	1.333437	0.670322
IQCK	0.743557	0.456028	1.212376	0.234872
ZNF266	0.98309	0.70587	1.369183	0.919627
SLC29A2	1.330376	1.104926	1.601826	0.002585
BRSK2	0.792931	0.465885	1.34956	0.392486
TMEM167A	1.080311	0.770994	1.513723	0.653546
SH3PXD2B	1.056894	0.878316	1.271781	0.557893

LARP7	1.120698	0.69165	1.815896	0.643536
FGFBP3	1.281223	0.793628	2.06839	0.310535
NR1D2	0.834658	0.621528	1.120873	0.229584
BRMS1	1.605826	1.002256	2.572871	0.048915
RPL15	0.895444	0.555036	1.444628	0.650872
HRAS	1.188112	0.863049	1.635606	0.290567
WDR49	1.283634	1.105561	1.490389	0.001049
SRP72	0.924449	0.576517	1.482359	0.744368
PCP2	1.234558	0.895923	1.701188	0.197704
RIN1	1.117991	0.915355	1.365485	0.274335
THAP6	0.802149	0.50546	1.272985	0.349467
CEP135	1.016778	0.697705	1.481769	0.930994
FZD4	1.016152	0.627932	1.64439	0.94798
CD248	1.063139	0.858481	1.316587	0.57464
PDZK1	0.686167	0.429366	1.096558	0.115348
PDE12	1.080138	0.626412	1.862507	0.781539
GLMN	0.719734	0.478116	1.083456	0.115054
YIF1A	1.284494	0.865925	1.90539	0.213341
CNIH2	1.170694	0.822515	1.66626	0.38154
NLRP6	1.042511	0.79105	1.373907	0.767523
NDUFA11	1.049605	0.659488	1.670492	0.838204
RSRC1	0.557615	0.329175	0.944587	0.02986
RAB1B	1.72369	1.196683	2.482786	0.003452
PTDSS2	0.960959	0.654011	1.411966	0.839262
SEZ6L2	0.867353	0.632599	1.189223	0.37683
ASPHD1	0.630826	0.311162	1.278889	0.201339
KCTD13	0.915824	0.471698	1.778115	0.795054
P2RY14	1.112013	0.955465	1.294211	0.170227
AMZ1	1.008875	0.756194	1.345989	0.9521
GPR171	0.972546	0.78344	1.207297	0.800778
FUT1	0.747065	0.567047	0.984232	0.038177
DHX36	0.588564	0.366038	0.946371	0.028711
FBXW8	0.942361	0.617747	1.437554	0.782914
KLC2	1.363131	0.991368	1.874305	0.056572
SLC22A1	1.862174	1.136552	3.051065	0.013585
CTBP2	1.320239	0.853034	2.04333	0.212521
CHST2	1.019311	0.832838	1.247534	0.852804
ZDHHC14	0.814418	0.571666	1.160253	0.255612
ATR	0.667684	0.449877	0.990943	0.044949
UBE2C	0.905427	0.693971	1.181314	0.464109
GK5	0.770619	0.492794	1.205073	0.25336
VCPIP1	1.031864	0.658261	1.617511	0.891216
DES	1.126651	0.901101	1.408656	0.295434
PDIK1L	0.895246	0.611327	1.311028	0.569663
RAG2	1.050233	0.811624	1.358991	0.709355
TRAF6	1.170987	0.663613	2.066279	0.585915
ZNF654	1.023752	0.671308	1.561232	0.913184
MRPS22	0.725959	0.416054	1.266702	0.259496
PACS1	1.180527	0.768064	1.81449	0.449209
WFDC5	0.638412	0.229255	1.777803	0.390432
MARCKSL1	0.806258	0.663594	0.979593	0.030198
SH3BP5L	1.379043	0.85191	2.232347	0.190946
YPEL2	0.863992	0.61211	1.219523	0.405769
ABO	1.090867	0.98735	1.205237	0.087321
PSMD2	1.612741	0.97292	2.673328	0.063811
FAM131A	0.996376	0.651635	1.5235	0.98663
CSRP2	1.004516	0.812766	1.241505	0.966743
INHBC	1.007574	0.632109	1.606058	0.974697

PARL	0.73606	0.453513	1.19464	0.214899
DDIT3	1.119021	0.872698	1.434871	0.375338
PCCA	1.029842	0.831851	1.274957	0.78721
DCTN2	2.749812	1.400299	5.399897	0.003305
NPPA	1.277528	0.781369	2.088742	0.32885
ZNF408	1.051538	0.746058	1.482101	0.774122
CTDSP2	1.107515	0.739899	1.65778	0.61975
CKAP5	0.908484	0.62169	1.327579	0.619962
ARHGAP1	1.302409	0.864364	1.962448	0.206544
MED16	1.090828	0.780198	1.525135	0.61116
GOLGA8A	1.045187	0.902548	1.210368	0.554953
TP53I11	1.162619	0.967973	1.396405	0.107013
DOLK	1.777718	1.138941	2.774752	0.01132
PHYHD1	0.837164	0.647996	1.081555	0.173817
CATSPER1	1.02336	0.804311	1.302066	0.850951
CCNE2	0.924716	0.625881	1.366235	0.69431
CST6	0.833743	0.560982	1.239126	0.368433
ZNF519	0.746288	0.475338	1.171685	0.203539
LSM1	1.794141	0.899934	3.576863	0.096822
BANF1	1.402467	0.902661	2.179018	0.132464
APOF	0.782054	0.193172	3.166143	0.730422
TMEM9B	1.536848	0.91235	2.58881	0.106271
NRIP3	0.998078	0.809929	1.229934	0.985599
PTPN2	1.011728	0.574307	1.782313	0.967807
EIF1AD	1.209186	0.61175	2.390078	0.584807
SMAD2	0.72515	0.436512	1.204647	0.214606
EIF3F	0.981162	0.525793	1.830908	0.952354
ZNF25	0.745356	0.488809	1.136551	0.17215
ARL10	0.968199	0.793269	1.181705	0.750595
CLTB	1.738019	1.200602	2.515995	0.003405
LPL	1.117198	0.965535	1.292683	0.136541
RFESD	0.707465	0.49172	1.017871	0.062247
CCDC14	0.86009	0.614299	1.204225	0.380093
TBC1D10C	1.201522	0.864729	1.66949	0.273981
SART1	1.775557	1.068783	2.949713	0.026635
PPP2R2D	0.618841	0.377854	1.013523	0.056573
MCTP1	1.145009	0.882104	1.48627	0.30895
POLD4	1.513013	0.904098	2.532037	0.114976
OR52W1	0.529891	0.253866	1.106037	0.090736
LRRC25	1.178092	1.052384	1.318816	0.004416
DPP10	0.916093	0.738329	1.136657	0.425909
CLCF1	0.939558	0.725668	1.216492	0.636182
TSGA10IP	0.906956	0.507556	1.620647	0.741592
GPR152	0.798212	0.520121	1.224987	0.302377
UBQLNL	1.069523	0.760056	1.504994	0.699744
KCNE3	1.030652	0.824136	1.288919	0.791291
CABP4	1.140791	0.772209	1.6853	0.508229
ALG10B	1.02928	0.709015	1.494212	0.879381
DRAP1	1.737851	1.140834	2.647297	0.010067
LONRF3	0.933249	0.785876	1.108258	0.430817
UCP3	1.04103	0.680183	1.593311	0.853092
UCP2	1.921646	1.412139	2.614987	3.25E-05
PAAF1	1.363925	0.745704	2.494679	0.313706
MRPL48	0.964838	0.464907	2.002363	0.92345
RAB6A	1.079866	0.626633	1.860914	0.781999
P2RY2	0.860814	0.732643	1.011409	0.068447
FOSL1	1.130989	0.945481	1.352894	0.178096
ERCC4	1.083335	0.661679	1.773692	0.750327

CCDC85B	1.305204	0.964014	1.76715	0.084907
TMEM70	2.209639	1.165333	4.18979	0.015154
RPS6KB2	1.287264	0.795457	2.08314	0.303859
TOM1L2	1.029911	0.689984	1.537306	0.885336
ZNF77	0.601669	0.413713	0.875015	0.007845
B3GNTL1	0.823521	0.608569	1.114394	0.208341
MLXIP	0.974847	0.634533	1.497678	0.90743
AURKAIP1	1.685879	1.141083	2.49078	0.008724
TTLL11	1.285484	0.753717	2.192425	0.356543
TOMM5	0.735614	0.489161	1.106235	0.14022
SLC35E3	1.195314	0.731189	1.954044	0.476801
ZNF169	0.851056	0.54076	1.339403	0.485793
RUVBL1	1.238716	0.803731	1.909118	0.332056
SFN	1.781402	1.049581	3.023484	0.032415
MSRA	0.979471	0.555779	1.726161	0.942804
ETV4	1.012146	0.658908	1.554755	0.956038
GAPT	1.115408	0.883258	1.408574	0.358968
BAIAP2	0.959472	0.739676	1.24458	0.75529
ZDHHC21	0.750127	0.508695	1.106144	0.146811
PLEKHF2	1.022115	0.673321	1.551592	0.918191
A2M	1.053968	0.861417	1.28956	0.60959
ARL4D	1.339712	0.921088	1.948595	0.12603
DOK7	0.882249	0.569838	1.365939	0.574299
LRRN1	0.77884	0.580286	1.045331	0.095973
UBE2O	1.028538	0.700711	1.509738	0.885741
ORAI3	0.743151	0.517343	1.06752	0.108187
UNC119B	0.93214	0.64765	1.341596	0.705247
DENND2C	1.424213	0.981801	2.065982	0.062435
ASCL3	0.7796	0.506396	1.200199	0.258058
TUBB6	1.039627	0.887156	1.218303	0.631036
LYSMD3	0.766164	0.514574	1.140764	0.189687
B3GALT6	1.203351	0.763145	1.897482	0.425652
ZNF613	0.7878	0.560336	1.107603	0.170046
JAKMIP2	1.21309	0.979484	1.502411	0.076724
MBLAC2	0.777276	0.553226	1.092065	0.146414
ZNF683	1.064752	0.859795	1.318566	0.56518
SLC35A4	1.806823	1.155152	2.826131	0.009545
IP6K1	1.036661	0.587071	1.830555	0.901232
SSNA1	1.558739	1.048152	2.318047	0.028363
CSTF3	0.877618	0.509707	1.511088	0.637729
YES1	0.915251	0.746404	1.122294	0.394709
CHMP6	0.969918	0.656673	1.432585	0.87801
DLEU1	0.784152	0.537821	1.143308	0.206284
UFSP1	0.838034	0.488056	1.438977	0.521793
TMEM39A	0.918669	0.545938	1.545877	0.749365
TCP11L1	1.008784	0.695591	1.462993	0.963221
GPX2	0.853614	0.632742	1.151586	0.300171
CCDC57	0.67229	0.519345	0.870276	0.00257
HSF5	1.146934	0.917922	1.433082	0.227684
SPHK1	1.036908	0.799673	1.344522	0.784524
BNIP3	0.585216	0.399671	0.856899	0.005892
MYPOP	1.054526	0.73636	1.510166	0.772007
ATAD5	0.805241	0.561874	1.154019	0.238094
ZNF404	0.987587	0.792711	1.23037	0.911316
RTTN	0.662674	0.401515	1.093699	0.107485
ACBD7	0.938909	0.671195	1.313404	0.712814
ANAPC2	1.139461	0.792817	1.637668	0.480524
ZBTB8OS	0.900001	0.439826	1.841641	0.773038

ZNF135	1.091818	0.927759	1.284888	0.290337
COX8A	1.418637	1.031216	1.95161	0.031646
TAC4	0.830452	0.591839	1.165266	0.282384
ZSCAN2	0.631686	0.41291	0.966377	0.034207
B3GNT4	0.827412	0.435681	1.571357	0.562638
CDC26	1.656716	0.915903	2.996723	0.095025
HSD11B2	0.978221	0.637893	1.500119	0.919597
CRLF3	0.641848	0.412025	0.999864	0.04993
RNPEP	1.56897	1.101374	2.235087	0.012603
EID2	1.219381	0.624075	2.382547	0.561668
EID2B	1.154741	0.69058	1.930879	0.583338
KCMF1	1.413129	0.605224	3.299497	0.424123
DNAJC30	1.18907	0.664021	2.129281	0.560186
SPRYD4	0.886599	0.550712	1.427348	0.620308
VPS37D	1.025637	0.562244	1.870952	0.934221
CLEC14A	0.887883	0.754426	1.04495	0.152461
CLK2	1.497472	0.85564	2.620754	0.157363
LPCAT4	1.010165	0.675811	1.509939	0.960668
SLCO3A1	1.185056	0.873299	1.608106	0.275655
ZNF575	0.838141	0.513273	1.368627	0.480361
WDR25	1.076242	0.510535	2.268792	0.84688
DIRAS1	0.865007	0.693903	1.078302	0.197199
PHLDB3	1.714599	1.170437	2.511753	0.005643
PRR15	0.732939	0.565098	0.950632	0.019205
GNG7	0.923968	0.754112	1.132083	0.445476
CNTD1	0.986532	0.540294	1.801326	0.964792
KBTBD11	0.823012	0.688672	0.983558	0.03217
B3GNT5	0.998954	0.749714	1.331054	0.994299
LMNB2	1.480868	0.998362	2.196569	0.05096
MEX3C	0.718296	0.485158	1.063466	0.098408
RNF152	0.894541	0.523417	1.528807	0.683594
MYO1D	0.907183	0.749532	1.097992	0.317238
FOXL1	0.76121	0.484838	1.195122	0.235823
LRRC37A	0.846336	0.521698	1.372987	0.499131
FOXC2	0.544618	0.211856	1.400051	0.207155
CCDC121	1.037207	0.708017	1.519451	0.85125
ACSF3	1.104517	0.677969	1.79943	0.689743
BOK	1.110403	0.703808	1.751891	0.652608
ZNF843	0.936549	0.538143	1.629909	0.816628
TTTY14	0.952014	0.818732	1.106994	0.5228
PFN4	0.768927	0.508552	1.162613	0.212884
CDK5R1	1.090069	0.720392	1.649448	0.683211
ZNF285B	0.624196	0.384809	1.012504	0.056183
RUFY1	1.206835	0.796059	1.829577	0.375842
BASP1	1.122815	0.998513	1.26259	0.052976
LRRC37A3	0.713034	0.465034	1.093291	0.12091
IRX5	0.823084	0.697447	0.971353	0.021228
METRNL	1.133084	0.950898	1.350174	0.16241
FAM91A1	1.175487	0.773007	1.787526	0.449628
WSB2	1.343098	0.902613	1.998544	0.145758
SOX11	0.189506	0.00759	4.731786	0.31097
TYMS	1.008579	0.732211	1.38926	0.958304
PXMP2	0.86642	0.531789	1.411621	0.564794
TCEANC	0.965022	0.603315	1.543586	0.881897
PNMA1	1.382754	1.014636	1.884427	0.040173
ANKLE2	0.800448	0.515382	1.243188	0.321735
C8G	1.205107	0.807583	1.798306	0.360962
FUT2	0.906243	0.650751	1.262046	0.560148

EFCAB5	0.91507	0.559723	1.496014	0.723424
GCNT4	1.036077	0.739218	1.452152	0.83698
MUC20	0.878265	0.639221	1.206704	0.423242
THAP4	1.749056	1.089742	2.807268	0.02056
NFATC2IP	1.089735	0.61211	1.940049	0.770274
FAM89B	1.528196	1.008685	2.315276	0.045418
SHMT1	1.527639	1.01356	2.302459	0.042938
DPP7	1.354472	1.007178	1.821518	0.044719
SEC24C	1.12253	0.741661	1.698989	0.584644
SMCR8	1.415214	0.892609	2.243794	0.139718
MTHFR	1.35578	0.943327	1.94857	0.100029
DEAF1	1.172195	0.842854	1.630223	0.345127
MTX3	1.124755	0.909016	1.391696	0.279247
TMEM80	1.316413	0.824887	2.100826	0.249016
FBXO46	0.954603	0.587804	1.550292	0.851042
ZDHHC13	1.194346	0.794098	1.79633	0.393744
SLC38A9	0.676693	0.424532	1.07863	0.100639
ACER2	0.673586	0.478512	0.948185	0.023516
WDR73	0.621993	0.363029	1.065686	0.083919
POLE	0.928667	0.720886	1.196336	0.566846
SCN4B	0.964016	0.641909	1.447754	0.8598
RHOG	1.449136	1.072373	1.958271	0.015744
EPS8L2	1.246654	0.897436	1.731763	0.188623
ANO6	1.205247	0.881649	1.647617	0.241873
ZBTB34	1.311253	0.840355	2.046023	0.232575
TALDO1	2.175452	1.271533	3.721957	0.004558
ULK1	0.959786	0.711649	1.294442	0.787975
RPS6KA3	1.056166	0.732088	1.523705	0.77011
B3GNT8	1.527204	1.150802	2.026719	0.003359
PUS1	1.326607	0.85898	2.048809	0.202492
CHD9	0.658823	0.432332	1.00397	0.052194
TRIM72	0.92396	0.578094	1.476753	0.740982
MAN1B1	1.044446	0.704218	1.549048	0.828799
KCNA3	1.073897	0.911392	1.265376	0.394415
FZD8	1.220898	0.961611	1.5501	0.1013
FBXO39	0.993631	0.737829	1.338119	0.966444
TOP3A	1.268668	0.737626	2.182026	0.389746
CASKIN2	0.798394	0.641299	0.993971	0.044003
ZBTB38	1.456808	1.148977	1.847112	0.001893
FLJ13224	0.790083	0.555286	1.124162	0.190366
RPL13AP3	1.057358	0.560437	1.994882	0.863282
CCDC71	1.120699	0.726467	1.72887	0.606419
LRRN4CL	0.630148	0.258919	1.533636	0.308859
FLJ40194	1.708294	1.060724	2.751203	0.027635
TIMM22	1.966731	0.989074	3.910757	0.053776
HIC1	0.944187	0.636462	1.400695	0.775336
PPFIA3	1.011623	0.772227	1.325233	0.933156
MAGEF1	1.747464	1.188294	2.569762	0.004558
UMODL1	0.903455	0.783358	1.041965	0.162987
SAMD9L	1.168032	0.915719	1.489865	0.210978
PAWR	1.090996	0.943464	1.2616	0.240045
TGIF1	0.544317	0.415988	0.712234	9.27E-06
NAP1L5	1.22305	0.80941	1.848075	0.339073
CD19	1.15206	0.941196	1.410164	0.169941
OR2T8	0.820534	0.632165	1.065032	0.137153
NR2C2	0.77731	0.534609	1.130194	0.187136
ACOT4	1.407555	1.088136	1.820738	0.009237
ARIH2	1.181301	0.636836	2.191258	0.597127

RBM44	0.747229	0.477747	1.168716	0.201671
ZBTB33	1.124757	0.77048	1.641935	0.54246
ZBED2	0.847301	0.729514	0.984104	0.030023
IRX3	0.886241	0.793169	0.990234	0.032897
OR2B11	1.093942	0.756109	1.582721	0.633754
SLC25A22	1.460802	1.032351	2.067073	0.032377
RABEP2	1.569891	1.070608	2.302016	0.020926
ATOX1	0.574822	0.357416	0.92447	0.022377
TBL1XR1	0.637734	0.407233	0.998703	0.049341
CD163	1.114314	1.02183	1.215169	0.014347
ZNF491	0.544953	0.386759	0.767852	0.000521
RPLP2	1.340196	0.86384	2.079236	0.191294
JUN	0.94046	0.818445	1.080664	0.386593
CSTF2T	0.889868	0.555543	1.425391	0.627383
GBA	1.464866	1.010931	2.12263	0.043652
ACAD9	0.990098	0.622252	1.575399	0.966505
IL17RA	1.003737	0.853365	1.180606	0.964075
PNPLA2	0.938555	0.711073	1.238811	0.65431
MBOAT4	1.094626	0.700514	1.710467	0.691363
AGTRAP	1.185382	0.962742	1.45951	0.109104
CD163L1	1.083874	0.786314	1.494037	0.622818
THAP5	0.829627	0.558764	1.231791	0.35434
SUMO4	0.844806	0.619585	1.151894	0.286386
DNAJC28	1.012814	0.624372	1.642919	0.958855
NAALADL:	0.775487	0.50935	1.18068	0.235803
CD151	0.992334	0.804451	1.2241	0.942718
POLR2L	1.682242	1.19285	2.372417	0.003023
FAM20C	1.117121	0.953129	1.309329	0.171525
FLII	1.466544	1.030685	2.086723	0.033344
SOX12	0.961837	0.74344	1.244391	0.76715
HNRNPA0	0.981709	0.60531	1.592164	0.940355
FAM87B	0.69492	0.426164	1.133164	0.144602
CHID1	0.966034	0.670506	1.391819	0.852859
ZNF620	0.927182	0.634991	1.353825	0.695457
ZNF518A	0.673233	0.487164	0.930371	0.016519
TMEM187	1.284745	0.882846	1.8696	0.190535
ZNF619	0.832415	0.532953	1.300143	0.4201
AP3S1	0.623537	0.37465	1.037766	0.069165
GRB2	1.351338	0.820193	2.226443	0.237245
ZBTB41	0.729334	0.472533	1.125695	0.154073
UBE2N	1.682949	0.960241	2.949592	0.069026
ARL6IP6	1.016021	0.592916	1.741054	0.953877
ZNF354C	1.091862	0.892996	1.335014	0.391598
MAMDC4	0.739901	0.547087	1.000671	0.050511
CENPBD1	1.313676	0.843893	2.044978	0.226939
ODF3	0.836207	0.50602	1.381848	0.485189
BET1L	1.104219	0.686446	1.77625	0.68272
RPS27	1.048959	0.70171	1.568048	0.815742
RIC8A	1.48134	0.900744	2.436173	0.121592
IMP3	3.400043	1.695085	6.81989	0.000569
ASB8	1.499617	0.769304	2.92323	0.234107
ODF3B	1.072369	0.913212	1.259263	0.393996
DPY19L2	0.813306	0.713077	0.927624	0.002073
GPR150	0.87571	0.581582	1.318591	0.525059
TSPYL6	0.815258	0.467973	1.420266	0.470798
DMAP1	1.056954	0.67145	1.663789	0.810885
IMPDH2	1.102867	0.759823	1.600789	0.606503
ALS2CL	0.991231	0.832529	1.180186	0.921188

MLF1	1.279605	0.983848	1.66427	0.065984
NDUFAF3	1.584634	0.915356	2.743267	0.100155
GRAMD1C	1.427804	1.029441	1.980321	0.032858
STAP2	0.940315	0.695488	1.271328	0.689218
TSSK6	0.961837	0.680399	1.359687	0.825631
BOLA1	1.633956	1.026696	2.600393	0.03835
PDE4DIP	1.853663	1.29615	2.650978	0.000722
DDX10	0.895171	0.552558	1.450221	0.652797
NDUFV2	0.731756	0.509667	1.05062	0.090574
DALRD3	1.029276	0.673662	1.572613	0.893858
ZNF114	0.690365	0.525819	0.906402	0.007644
ZNF518B	1.2217	0.922709	1.617573	0.162035
LCORL	0.573191	0.361409	0.909076	0.018027
PARD6G	0.644113	0.465986	0.89033	0.007738
SH2B1	1.201175	0.79109	1.823841	0.389672
ZC3H12D	1.383639	0.963367	1.987257	0.078761
VN1R1	0.991931	0.736007	1.336845	0.957562
SH2D4B	0.824641	0.45171	1.505464	0.530117
RNF212	0.893396	0.687272	1.16134	0.399615
PRSS36	1.669392	1.293209	2.155003	8.36E-05
ZNF543	0.895935	0.631891	1.270311	0.537328
GALNT11	0.755065	0.511721	1.11413	0.15693
WDR6	1.007531	0.732054	1.386672	0.963277
GEN1	0.777114	0.534425	1.13001	0.186799
TMPRSS9	0.747372	0.558708	0.999743	0.049798
AQP11	0.548941	0.378517	0.796096	0.001565
TMEM11	1.718931	1.071547	2.757436	0.024669
ZNF354B	0.83643	0.58868	1.188448	0.318949
KCNG2	0.863131	0.666654	1.117514	0.264045
ZFAND2A	1.057902	0.712326	1.571128	0.780293
PLEKHM3	1.019483	0.680351	1.52766	0.925499
ZNF223	0.649628	0.366955	1.15005	0.138816
BEND3	0.512158	0.3312	0.791987	0.002625
NT5DC1	1.006406	0.681745	1.485678	0.974365
GLDC	1.021891	0.617532	1.691025	0.932845
TUBAL3	1.112156	0.871026	1.420037	0.393919
P4HTM	0.904804	0.641954	1.275277	0.567798
DTX3	1.087617	0.921267	1.284003	0.321346
KLHL11	0.623005	0.40509	0.958147	0.03119
CTXN1	0.774425	0.542	1.10652	0.16031
SLC25A20	1.456252	0.853616	2.484336	0.167834
CA8	0.868084	0.669015	1.126387	0.287126
CD28	0.948149	0.750465	1.197907	0.655376
EPM2AIP1	0.731585	0.477072	1.121877	0.151926
MAF	1.06777	0.834292	1.366587	0.602465
CTNNBIP1	0.779417	0.52938	1.147553	0.206719
GTPBP6	1.020284	0.693444	1.501173	0.91882
ERN1	1.03429	0.768488	1.392028	0.823956
GPR35	1.370734	1.087087	1.728393	0.007679
ZNF713	0.724306	0.478566	1.096234	0.127153
PARP10	1.609375	1.18998	2.17658	0.002007
SUZ12	0.769406	0.499573	1.184983	0.234175
NSUN3	0.79446	0.456155	1.383668	0.416326
KCTD12	1.112068	1.009393	1.225187	0.031624
RPP25	1.51089	1.135498	2.010384	0.004627
GRINA	1.650641	1.240825	2.195809	0.000578
THBD	1.155628	0.94377	1.415043	0.161552
GP5	1.178189	0.911967	1.522126	0.209552

COX5A	2.341001	1.405684	3.898661	0.001081
STX19	0.588485	0.382399	0.905638	0.015926
ZHX2	1.042956	0.790695	1.375698	0.765931
CPNE7	1.091904	0.965072	1.235405	0.162828
CD300LB	1.204748	1.072176	1.353712	0.001739
MPI	2.102747	1.241521	3.561393	0.005698
TRIM73	0.654587	0.368846	1.161689	0.147653
OPLAH	1.228948	1.000208	1.51	0.049769
TMEM52	0.909976	0.591843	1.399116	0.66733
MSC	1.189235	0.937271	1.508934	0.153674
APOLD1	0.859984	0.643129	1.14996	0.308931
EXOSC4	1.91475	1.295279	2.830485	0.001125
DPY19L3	0.536831	0.365885	0.787645	0.001471
TAF7	1.084632	0.747936	1.572898	0.668355
PFAS	1.192099	0.83272	1.706576	0.337093
ZNF552	0.90553	0.616535	1.329989	0.612879
GAK	1.134427	0.740736	1.737361	0.561935
ZBTB7A	1.673595	1.034428	2.707699	0.035918
TUFM	1.682467	1.087663	2.602548	0.019412
RMI1	1.148089	0.827439	1.592997	0.408563
FBXO34	1.465418	0.861449	2.492832	0.158608
EIF3K	1.613054	0.955854	2.722111	0.073317
MRFAP1L1	1.183277	0.818308	1.711025	0.371138
SNX18	0.999669	0.728095	1.372537	0.998364
AURKB	1.006157	0.764042	1.324996	0.965137
MRFAP1	1.507326	0.783442	2.900065	0.219075
TMEM107	1.169544	0.875359	1.562596	0.289397
RRS1	1.593583	1.058337	2.399528	0.02565
RCC2	1.281844	0.722863	2.273078	0.395569
OR13D1	0.866627	0.625363	1.200971	0.38985
IGSF22	0.781076	0.419024	1.455954	0.436779
DPM3	1.343503	0.950071	1.899858	0.09487
CYC1	1.991072	1.293334	3.06523	0.001757
PER1	1.022138	0.861699	1.212449	0.801544
HTR1F	1.200841	1.078979	1.336466	0.000801
TMTC2	0.973684	0.826571	1.146981	0.749653
FARSA	0.892007	0.63774	1.24765	0.504429
SPTY2D1	0.554548	0.345685	0.889608	0.014483
SAMD4B	0.833544	0.479353	1.449443	0.518923
GIMAP7	1.090717	0.941954	1.262974	0.245776
EDC3	1.047931	0.627844	1.749096	0.857841
FUCA1	1.403167	1.076451	1.829045	0.012256
PXT1	0.364872	0.070058	1.900313	0.231134
GGN	1.012752	0.618761	1.657614	0.959798
ZNF664	0.855471	0.580096	1.261567	0.43092
CALR	0.702758	0.548812	0.899886	0.005172
MAGED1	1.185706	0.96911	1.450712	0.097906
LDLRAD3	1.122769	1.004669	1.25475	0.04114
CDH4	0.878441	0.743753	1.03752	0.126955
RAD23A	1.76653	1.156743	2.697772	0.008439
GADD45G	1.416117	0.96221	2.084149	0.077634
MEIS3P1	0.865475	0.624808	1.198845	0.384817
DAND5	0.87433	0.447818	1.707063	0.694018
TMEM151A	1.819256	0.385279	8.590374	0.449872
PTPN11	1.042501	0.697504	1.558139	0.839136
NSUN7	0.877193	0.643309	1.196111	0.407589
WSCD1	1.945309	1.065001	3.553263	0.030399
CLK3	0.986541	0.642285	1.515313	0.950655

HLA-DQB:	1.143573	1.018051	1.28457	0.023726
GATA2	0.933282	0.808902	1.076787	0.344059
ARID3B	1.797194	1.249514	2.58493	0.001572
PACS2	1.585047	1.096443	2.291388	0.014301
ELMOD2	0.726255	0.485868	1.085575	0.118855
EGR3	0.964268	0.822472	1.130509	0.653883
GPC5	0.719293	0.478665	1.080885	0.112819
VWA1	1.118557	0.910984	1.373428	0.284718
GEMIN4	1.045986	0.71692	1.526093	0.815549
FJX1	1.423775	0.75882	2.67143	0.271162
KLHL28	0.586074	0.355408	0.966448	0.036287
MKRN3	1.036392	0.883812	1.215314	0.659994
SHARPIN	1.654989	1.119148	2.447387	0.011606
LBX2	1.1141	0.780831	1.589612	0.55132
DNHD1	1.0234	0.716997	1.460743	0.898614
SLITRK4	1.068446	0.933012	1.223539	0.3384
GCC1	1.204587	0.708583	2.04779	0.491747
RNF151	0.977542	0.404878	2.360187	0.959719
CIITA	1.178396	1.013238	1.370476	0.033118
ZFPM1	1.127153	0.907748	1.399589	0.278509
ALOX15B	1.01067	0.859908	1.187862	0.897543
PLD6	0.799875	0.570415	1.121641	0.195495
CDC42EP4	0.844638	0.667919	1.068114	0.158604
ZBTB42	1.030939	0.740365	1.435555	0.856857
MAF1	1.693706	1.078852	2.658976	0.022034
FCER1A	1.070924	0.961246	1.193117	0.213874
FLJ37453	1.282852	0.740386	2.222773	0.37445
APOBEC3E	1.125915	0.890135	1.424149	0.322556
PIPOX	0.973621	0.68139	1.391184	0.88328
CDH5	1.091685	0.790131	1.508326	0.594837
MYADM	1.200212	0.927909	1.552425	0.16451
SERTAD2	0.664794	0.418523	1.055978	0.083761
AKAP5	0.668666	0.419121	1.066791	0.09128
NKPD1	0.711256	0.464547	1.088986	0.116945
GIPC3	1.024168	0.872065	1.202799	0.770957
CITED4	1.076785	0.906704	1.278769	0.399002
ABCA13	0.906493	0.708523	1.159778	0.434863
TIGD5	1.010398	0.710962	1.435946	0.954002
PDXDC1	0.866022	0.525794	1.426404	0.57208
ZNF154	0.986339	0.81174	1.198492	0.889934
R3HDM2	0.885939	0.418755	1.874337	0.751428
B3GNT3	1.365582	0.873783	2.134183	0.171403
ITLN1	1.337559	1.030348	1.736369	0.028924
SEPHS2	2.414671	1.331563	4.378793	0.003697
GPBAR1	1.199808	1.028255	1.399983	0.020674
ZNF784	0.933717	0.611274	1.426247	0.751015
BBS10	1.074759	0.753303	1.53339	0.690913
FIZ1	1.165845	0.677099	2.007379	0.579939
PUF60	1.517592	1.015331	2.26831	0.041935
DCTPP1	1.73151	1.171983	2.558166	0.005835
ZNF771	1.036594	0.590416	1.819951	0.900406
TSHZ1	0.733269	0.453539	1.18553	0.205635
PSTK	0.640327	0.367651	1.115241	0.115331
SOCS4	1.045577	0.656422	1.665442	0.851152
ZADH2	0.474512	0.278337	0.808952	0.006164
ZNF48	1.357756	0.886269	2.080071	0.159956
TMEM150I	1.078669	0.925561	1.257104	0.332263
ANKRD18/	0.861811	0.738193	1.006132	0.059757

TMEM86B	1.083985	0.73287	1.603318	0.686361
TRNAU1AI	3.16053	1.4082	7.093417	0.005273
EXOC3	1.229224	0.78475	1.925444	0.3674
CSNK1A1L	0.71859	0.354193	1.457884	0.359909
LYNX1	1.384985	1.070205	1.79235	0.013296
MED14	1.165351	0.673286	2.017036	0.584591
FAHD1	1.737857	0.962894	3.136531	0.066587
RCC1	1.500099	0.974028	2.310301	0.065691
MYLPF	1.171332	0.813912	1.685709	0.394541
F2	0.679656	0.433314	1.066046	0.09267
PRKRA	0.469315	0.302379	0.728413	0.000744
ZNRF2	0.685473	0.454734	1.033293	0.071301
FGD6	1.004688	0.815397	1.237922	0.964975
OAZ2	0.780666	0.447099	1.363097	0.383915
PNPLA1	1.078625	0.869502	1.338045	0.491261
CCDC43	0.807783	0.453289	1.439511	0.468987
KCTD4	0.66188	0.464235	0.943671	0.022588
FZD2	0.901226	0.771232	1.053132	0.190677
TIGD2	1.076028	0.766482	1.510584	0.672019
HCLS1	1.810668	1.274123	2.573157	0.00093
ZNF609	0.602878	0.416182	0.873325	0.007444
PAK2	0.89308	0.562618	1.417643	0.631481
CCDC66	0.527934	0.324559	0.858748	0.010069
MCFD2	1.238822	0.723282	2.121827	0.435373
HARBI1	1.284146	0.705676	2.336809	0.412934
GAS1	0.997266	0.727863	1.366383	0.986406
ZNF571	0.796986	0.588857	1.078677	0.141696
KCNE1	1.319773	1.12939	1.542249	0.000481
NRIP1	1.151546	0.998634	1.327873	0.052239
BHLHA15	1.101238	0.766199	1.582781	0.602333
RNF182	1.02366	0.769349	1.362033	0.872504
TSPYL5	1.122089	0.947182	1.329295	0.18275
FUT7	1.295504	1.055802	1.589628	0.013133
SSTR2	0.460955	0.275178	0.772153	0.003257
ZNF594	0.976421	0.734822	1.297455	0.869322
PCGF5	1.402182	1.01308	1.940729	0.041517
PRF1	1.065382	0.899946	1.261231	0.461988
YOD1	0.994155	0.748162	1.32103	0.967761
TMEM64	0.784692	0.600676	1.025081	0.075357
CHRM4	0.959411	0.688459	1.337	0.806676
SHISA2	0.248053	0.087201	0.70562	0.008958
S1PR5	1.262799	1.01246	1.575038	0.038471
GPR157	1.042526	0.758342	1.433206	0.79759
PIPSL	1.077196	0.638473	1.817386	0.780511
CHST13	0.987226	0.843016	1.156105	0.873223
SLC36A4	1.008917	0.767486	1.326297	0.949274
ZDHHC20	0.912459	0.628123	1.325506	0.630614
ZFP3	1.061676	0.840028	1.341808	0.616423
MAP3K15	0.822963	0.590621	1.146704	0.249658
PPA1	1.440981	1.130382	1.836924	0.003184
PSMG4	0.723575	0.464708	1.126645	0.152102
MAP6D1	0.557718	0.325625	0.955238	0.03344
ZNF443	0.632997	0.454176	0.882224	0.006939
SSR4	1.026012	0.685212	1.536313	0.900783
CAPS2	1.066044	0.78298	1.451441	0.684609
ZNF792	0.974121	0.743425	1.276405	0.849196
CUEDC1	1.431601	1.162492	1.763007	0.000732
SCRIB	1.050771	0.758609	1.455452	0.765753

KCTD2	1.143832	0.638937	2.047701	0.651055
D2HGDH	1.238781	0.929905	1.650254	0.143378
OXTR	1.018405	0.624952	1.659563	0.941648
FAM83H	1.144521	0.920265	1.423426	0.225064
ZNF572	0.919464	0.640254	1.320435	0.649325
ST20	0.821204	0.556562	1.211681	0.320946
PITPNB	0.974675	0.555241	1.710955	0.928807
TCEAL8	1.073393	0.69926	1.647701	0.746007
LRRC57	1.247623	0.664084	2.343926	0.491673
MRPL14	1.97149	1.220708	3.184031	0.005514
GPR137C	1.015177	0.647081	1.592665	0.947732
BBS12	1.291506	0.904511	1.844076	0.159222
ZFP82	0.705933	0.506201	0.984475	0.040153
NQO1	1.432834	0.850598	2.413612	0.176447
OR56B1	1.034495	0.687106	1.557518	0.870955
AEN	1.326527	0.881358	1.996549	0.17556
FKRP	1.159555	0.67256	1.999179	0.59426
TRAPPC5	1.198034	0.687282	2.08835	0.523944
RPH3AL	1.168539	0.922472	1.480242	0.196685
SLC25A42	0.898943	0.678765	1.190542	0.457342
FCRL6	1.162184	0.957131	1.411167	0.129122
SLC26A11	1.136098	0.904356	1.427223	0.272968
HIGD1A	1.476976	1.021054	2.136478	0.038398
OR52N4	1.079292	0.823134	1.415164	0.58096
MAPK15	0.986368	0.674331	1.442796	0.943608
EHMT1	0.811692	0.534511	1.232611	0.327676
F2R	1.130188	0.96326	1.326043	0.13338
ZNF707	1.258276	0.798311	1.983262	0.322349
NPM1	0.85908	0.61565	1.198764	0.371582
PJA1	1.033257	0.659923	1.617797	0.886274
DHTKD1	1.068263	0.758336	1.504854	0.705654
PENK	1.936193	0.936113	4.00469	0.074763
ZNF746	0.959381	0.621088	1.481934	0.851725
POLR2A	1.221638	0.802493	1.859705	0.35045
FRAT2	1.330659	0.989797	1.788905	0.058486
TMEM102	1.230941	0.824517	1.8377	0.309521
CCL13	1.215557	0.731639	2.019548	0.451078
DDX60L	1.163322	0.90268	1.499221	0.242458
AATK	1.044134	0.812363	1.342028	0.735936
DDN	0.896834	0.594938	1.351925	0.603072
SAGE1	1.223799	1.083777	1.381911	0.001123
ZNF467	1.179088	0.976536	1.423653	0.0867
ZNF678	0.792119	0.538304	1.165609	0.237035
TMEM45A	1.323387	0.84239	2.079027	0.224073
RAP2B	1.079066	0.700659	1.661839	0.729809
ZBTB2	1.150986	0.636608	2.08098	0.64166
RNF135	1.192838	0.796265	1.786922	0.392476
ACBD4	1.569828	1.036252	2.378146	0.033337
SGSH	1.112329	0.949853	1.302597	0.186376
MAB21L2	1.037913	0.701377	1.535928	0.85237
FANCB	1.100343	0.728842	1.661204	0.649123
SETD2	0.824294	0.520168	1.306234	0.410718
TMIE	0.913428	0.657542	1.268894	0.589238
MEX3D	1.246565	0.910005	1.707599	0.16987
MRPS23	1.565264	0.902891	2.713562	0.110473
OR52H1	0.886149	0.639279	1.228352	0.468158
GPR135	0.852687	0.594595	1.222808	0.386276
P2RY13	1.154618	1.020922	1.305823	0.022037

ZFP41	1.018192	0.663326	1.562905	0.934281
PHLDA2	1.200804	1.000478	1.441242	0.049403
ATG9B	1.172123	0.742392	1.850602	0.495504
GPR88	0.949926	0.734611	1.228351	0.695277
PLAG1	1.033806	0.876546	1.21928	0.69292
YIPF6	0.53262	0.292243	0.970712	0.039683
ZBTB20	0.807983	0.58922	1.107967	0.185659
AMIGO1	0.728728	0.57191	0.928545	0.010479
GPR3	1.185693	0.890102	1.579446	0.244335
SIAH2	1.544408	0.987374	2.415694	0.056872
SLC9A9	1.244761	1.044682	1.483159	0.01433
LSM10	1.945125	1.334337	2.835499	0.00054
RELL1	0.73735	0.551735	0.985411	0.039467
RFX7	0.844487	0.579107	1.231478	0.379848
SLC35C1	1.660227	1.046285	2.634421	0.031395
TIGIT	1.03793	0.736816	1.462101	0.831366
RNF41	2.715115	1.210714	6.088842	0.015351
SLC2A4	0.743467	0.512869	1.077746	0.117643
CLDN7	0.801015	0.561709	1.142274	0.220449
ZNF329	0.864936	0.574944	1.301196	0.486191
ZNF101	1.551964	0.923636	2.607731	0.096925
ADO	1.749433	0.967323	3.163904	0.064304
PRKAG1	2.257457	1.121933	4.542262	0.022461
GINS3	1.302282	0.852003	1.990532	0.222433
OR52K2	0.979396	0.833523	1.150797	0.800254
CCDC149	0.945726	0.775523	1.153283	0.581481
MRPS11	1.755793	0.888248	3.470661	0.105423
SNRPE	1.023593	0.626321	1.672853	0.925867
RTKN2	0.929635	0.676942	1.276653	0.652111
CHST15	1.123258	1.001257	1.260125	0.047548
TRPC2	1.170356	0.952308	1.438329	0.134804
IDH2	1.293772	0.839478	1.993912	0.243171
TNRC18	0.701242	0.503763	0.976136	0.035457
TMEM30B	0.960462	0.474441	1.944366	0.910738
DEXI	1.348889	1.029061	1.768118	0.030202
NOP10	2.021432	1.314118	3.109453	0.001359
FAM89A	0.978163	0.816062	1.172464	0.811232
TDRKH	1.17414	0.900321	1.531236	0.236048
ZNF708	0.616691	0.437517	0.869242	0.005778
MRPL41	1.364327	1.024721	1.816482	0.033405
CREB3L2	0.638526	0.385658	1.057193	0.0812
P2RY8	0.702151	0.516367	0.954777	0.024127
TP53TG1	1.17578	0.621872	2.223061	0.618287
UNC5C	1.174155	0.90075	1.530548	0.235192
TSEN54	0.87643	0.631889	1.215607	0.429394
RGMA	0.668036	0.475949	0.937647	0.019692
UBA7	1.365118	1.010529	1.844132	0.042538
MRPS16	2.94033	1.46203	5.91338	0.002483
LDOC1	1.140097	0.919912	1.412985	0.231103
ARL6IP4	1.096529	0.623584	1.92817	0.748975
EXT1	1.109564	0.872403	1.411199	0.396779
SHMT2	1.439509	0.892674	2.321324	0.135103
ATP6AP2	1.33755	0.860147	2.079924	0.196649
ZAR1	0.994427	0.557634	1.773356	0.984891
BACE2	0.958249	0.770713	1.191418	0.701129
UBE2E2	1.051345	0.869852	1.270706	0.604557
SYNM	0.929541	0.586149	1.474105	0.756135
IZUMO1	0.590423	0.268364	1.298979	0.190278

B4GALNT4	0.98894	0.807018	1.211871	0.914607
AP1S2	1.218274	1.010756	1.468398	0.038242
DCAF4L1	1.586012	0.870942	2.888175	0.13152
ZSCAN22	0.765348	0.530449	1.104267	0.152799
KCNJ14	0.92105	0.635867	1.334136	0.663542
FBXL6	1.235346	0.910771	1.675592	0.17415
C1S	1.020029	0.796661	1.306024	0.875039
GLTPD2	1.395774	0.879275	2.21567	0.15728
KBTBD3	0.828016	0.465067	1.474219	0.521379
CLN8	1.411921	1.003628	1.986314	0.047618
PLCXD1	1.428898	0.972281	2.099959	0.069238
NXPH4	1.0701	0.576526	1.986233	0.829998
CACNB4	0.803988	0.644485	1.002968	0.05315
TRAPPC6B	0.691968	0.435061	1.100582	0.119903
PGBD4	1.072978	0.699202	1.646565	0.747174
NPLOC4	1.429764	0.861239	2.373588	0.166863
CAPN12	1.301857	0.870242	1.947541	0.199265
EXOC7	1.027095	0.651232	1.619889	0.908443
KPNA2	1.215052	0.820233	1.799917	0.331273
NCF1B	1.134678	0.997973	1.29011	0.053733
BGN	0.911417	0.769851	1.079016	0.281497
CEP97	0.668499	0.443872	1.006803	0.053915
FES	1.215128	0.892335	1.65469	0.216138
GLRX5	1.145783	0.747304	1.756738	0.532554
FAM104B	1.043596	0.556621	1.956615	0.894142
MXRA7	0.826428	0.672083	1.016219	0.070695
LIMK2	1.559666	1.018567	2.388215	0.040895
MFSD5	2.266976	1.301983	3.947197	0.00382
ADI1	1.544817	0.894169	2.668914	0.119
SPNS3	1.141305	0.97004	1.342807	0.111097
SATB1	0.759785	0.530352	1.088471	0.134192
CSF1R	1.080411	0.934113	1.249622	0.297488
EPHB3	0.966831	0.836901	1.116931	0.646871
EPGN	1.080747	0.962971	1.212929	0.187159
HS3ST4	1.35313	1.043681	1.754329	0.02245
TRAK1	0.997313	0.586411	1.696137	0.992077
PLCB1	0.892467	0.665841	1.196227	0.446552
SKA2	1.305281	0.887453	1.919829	0.175925
NDN	1.137253	1.008067	1.282995	0.036568
TTC3	0.778534	0.572757	1.058241	0.10993
GALR2	0.898357	0.610937	1.320996	0.585849
TSKU	0.947508	0.693657	1.294256	0.7347
ANXA2	1.088575	0.951276	1.24569	0.217277
RGS6	1.049725	0.764412	1.441529	0.764274
HOXB4	1.142158	1.035506	1.259794	0.007871
PAQR7	0.902821	0.613009	1.329647	0.604767
PAPPA	1.058739	0.744908	1.504786	0.750331
MAFA	1.151899	0.433963	3.057567	0.776474
NGRN	0.902211	0.51145	1.591524	0.722331
RPS17	1.077232	0.709959	1.634501	0.726552
CRIP2	1.161211	0.982024	1.373094	0.080491
DDX28	2.093492	1.225898	3.575099	0.006812
ACBD3	0.701039	0.388647	1.26453	0.23794
VMO1	1.199545	0.916164	1.570581	0.185775
ALG12	0.835873	0.584052	1.196269	0.326998
LCK	1.103877	0.904126	1.347758	0.331862
COL18A1	1.077059	0.840161	1.380753	0.558049
RBM10	1.290761	0.808416	2.060899	0.285023

GLUD2	1.910631	1.163744	3.136869	0.010484
RPL35A	0.793875	0.499208	1.262475	0.329442
SLC25A18	1.163118	0.447218	3.025019	0.756675
ZNF721	0.808347	0.664775	0.982928	0.032961
CEP63	0.553464	0.331818	0.923163	0.023436
WFDC10B	2.521976	0.708453	8.977818	0.153315
EWSR1	1.167767	0.607177	2.245936	0.64209
ODF3L1	1.793562	1.336729	2.406521	9.82E-05
HMGN4	1.201628	0.656859	2.198205	0.551132
SPATA13	1.164775	0.813594	1.667541	0.404765
CNOT10	0.834552	0.473017	1.472413	0.532401
MTA1	1.130954	0.765947	1.669904	0.535968
ZNF662	1.039559	0.886278	1.219351	0.633593
CADM1	1.34653	1.057537	1.714497	0.015787
ZNF320	0.797274	0.587696	1.081589	0.14541
PYCR1	1.016571	0.831353	1.243054	0.872763
SPNS2	0.943947	0.812703	1.096385	0.450109
AP2A2	1.20472	0.840547	1.726673	0.310517
SLC8A1	1.257296	1.071273	1.47562	0.005067
SLC25A21	0.488487	0.246286	0.968873	0.040319
ABAT	1.158317	0.791436	1.69527	0.449476
SLC25A10	1.000438	0.645008	1.551729	0.998438
CAMK1D	1.297261	1.080172	1.55798	0.005347
LYSMD4	0.741882	0.542993	1.013622	0.060794
AFMID	1.13013	0.735576	1.73632	0.576616
GAS6	1.262108	1.018393	1.564148	0.033466
NEB	0.727318	0.411485	1.285567	0.27326
BEGAIN	1.015567	0.86413	1.193544	0.851276
GPC6	0.884946	0.719243	1.088824	0.247891
CSMD1	1.255743	0.903259	1.745778	0.175513
GPR19	1.657179	0.984258	2.790164	0.057398
RABIF	1.047924	0.598122	1.835987	0.870038
TMEM119	0.795347	0.56857	1.112573	0.181203
FANCF	1.101231	0.768232	1.578571	0.599683
CALN1	1.033883	0.912287	1.171686	0.601697
RUVBL2	1.131655	0.780726	1.640323	0.51374
PTTG1IP	1.422503	0.927167	2.182469	0.106593
DDX41	1.223673	0.788085	1.900019	0.368572
DAZAP2	1.478463	0.898881	2.43175	0.123543
ZNF623	0.954424	0.639064	1.425405	0.819693
CCDC125	0.600864	0.403336	0.895131	0.012255
BCOR	1.394479	0.876502	2.21856	0.160448
JRKL	1.020727	0.697931	1.492818	0.915762
KIAA2026	0.680077	0.413689	1.118	0.128468
FHL3	1.190746	0.903489	1.569333	0.215188
TMEM89	1.256811	0.765174	2.064333	0.366624
SF3A3	1.276049	0.771888	2.109503	0.341882
ASB7	1.283565	0.719271	2.29057	0.398208
TREX2	1.507151	0.955905	2.376287	0.077423
GPR132	1.429799	1.145172	1.78517	0.001595
MX2	1.309045	1.049742	1.632401	0.016804
EP400	0.922806	0.585824	1.453629	0.728955
MEX3B	0.896308	0.764846	1.050366	0.176137
PI4KAP2	0.947505	0.677943	1.324248	0.752227
PSMG1	1.881853	1.136337	3.116476	0.014028
KLRC4	1.001456	0.772682	1.297965	0.991227
SERHL2	0.968134	0.69596	1.34675	0.84751
PCBP3	0.823809	0.710966	0.954563	0.009919

SETD3	1.301241	0.732306	2.312187	0.369319
TNFAIP8L3	0.644159	0.447532	0.927176	0.017939
ZNRF3	1.081009	0.790437	1.478397	0.625787
SFXN4	0.9893	0.642363	1.523616	0.961059
FAM167B	1.018165	0.734506	1.41137	0.91396
MRPL54	1.333293	0.893349	1.989894	0.159145
ZNF438	0.91427	0.718664	1.163116	0.465551
CCR3	1.111305	0.816139	1.513223	0.502833
ZNF530	1.035863	0.646535	1.659636	0.883519
NDUFB1	1.912977	1.123964	3.255871	0.016818
KLHL25	0.71915	0.50176	1.030727	0.072626
TRMT12	0.981785	0.649269	1.484596	0.930569
BMP8A	0.902478	0.696992	1.168545	0.436338
EFHC2	0.941949	0.789585	1.123714	0.506489
NOG	0.859337	0.758044	0.974166	0.017837
UPP1	1.144736	0.979153	1.338321	0.089946
TRIM52	0.858337	0.540657	1.36268	0.517141
CMTM4	1.181704	0.967249	1.443707	0.102248
TMEM50A	1.49182	0.788794	2.821431	0.2186
ASCL2	1.117174	0.931797	1.33943	0.231347
TBK1	1.190907	0.749977	1.89107	0.458987
CBX6	1.162538	0.872464	1.549056	0.303777
MACC1	1.107257	0.92778	1.321454	0.258821
TBL3	1.423417	0.969501	2.089856	0.071563
KREMEN1	0.861471	0.645137	1.150348	0.312185
TRAIP	1.117854	0.747049	1.672712	0.587962
CHEK2	0.973414	0.611487	1.549556	0.909556
AIFM3	1.21392	0.999609	1.474177	0.050463
ZNF703	1.077216	0.90187	1.286655	0.411904
EMILIN3	2.472558	0.038097	160.4716	0.670698
OLFML1	1.109111	0.745116	1.650921	0.609862
RBM12B	0.883913	0.623634	1.252822	0.488061
CCR4	1.239783	0.970731	1.583407	0.085074
LIN9	0.99142	0.680272	1.444883	0.964236
BTBD9	1.091957	0.610019	1.954646	0.767127
NUDT14	1.470131	1.156808	1.868319	0.001627
PNMA3	1.176494	0.946339	1.462625	0.143361
IQGAP3	1.119978	0.884132	1.418737	0.347627
TOB2	1.207149	0.782303	1.862718	0.39497
SCN5A	0.749805	0.339444	1.656259	0.476392
ARSI	1.817929	0.713538	4.63166	0.210346
UTY	0.984792	0.897081	1.08108	0.747472
TTC32	0.793662	0.498762	1.262925	0.32953
DNAH2	0.679365	0.281115	1.641806	0.390503
SH2D1A	1.000071	0.87663	1.140895	0.999152
PRKX	0.845325	0.589725	1.211707	0.360364
KCNH8	1.049323	0.813776	1.353048	0.710494
SMTN	1.092725	0.784185	1.522659	0.600397
NPW	0.995898	0.865447	1.146012	0.954244
ST6GALNA	1.334569	0.895149	1.989696	0.156666
PTP4A2	1.469866	0.852552	2.534161	0.165759
ACTG1	1.138609	0.813595	1.593459	0.449066
DENND5A	0.89421	0.614796	1.300612	0.558579
DIABLO	0.584321	0.348215	0.980518	0.041905
VPS33B	1.945885	0.931632	4.06434	0.076475
TBX1	0.677598	0.544663	0.842979	0.000478
ADAP2	1.150743	1.002321	1.321142	0.046275
FAM120C	0.656567	0.406929	1.059348	0.084752

BRD7P2	0.972964	0.535455	1.767951	0.928328
EIF3C	0.96357	0.594738	1.561134	0.880181
CLDN5	0.96342	0.739246	1.255574	0.782727
NIPSNAP1	1.173907	0.816262	1.688256	0.387112
LRTOMT	0.813284	0.576776	1.146773	0.238469
ADRA2C	1.011468	0.854339	1.197495	0.894691
NR2C2AP	1.610765	0.944078	2.748252	0.080317
CRELD2	1.042558	0.781289	1.391197	0.777061
SCFD2	1.206753	0.74875	1.944911	0.440264
UBE2F	1.02661	0.669308	1.574652	0.904224
KCNJ12	1.007742	0.788949	1.287211	0.950756
GPR173	0.996794	0.871887	1.139595	0.962503
PPP1R2	1.655941	0.985442	2.782649	0.056834
TSPYL2	1.078518	0.848679	1.370602	0.53647
PGP	1.37687	0.964047	1.966471	0.078642
SNRNP35	1.01498	0.478173	2.15442	0.969113
IRAK1	1.90553	1.332802	2.724368	0.000408
OLIG1	0.961162	0.844885	1.093441	0.547092
PCDH9	0.897501	0.705635	1.141537	0.378188
ACOT1	1.656883	1.03372	2.655711	0.035928
OAF	1.027963	0.784215	1.347472	0.841701
KCNK12	0.90804	0.665845	1.238329	0.542221
POU6F1	0.815424	0.629381	1.056461	0.12252
TM2D3	0.996833	0.550059	1.80649	0.991657
TSSC4	1.312405	0.897417	1.919294	0.160959
TACSTD2	0.90494	0.78657	1.041122	0.162553
CLECL1	0.980001	0.854397	1.12407	0.772828
ZDHHC23	0.900554	0.723915	1.120293	0.347073
SRPK3	1.188005	0.841056	1.678078	0.328247
EFNA5	1.344951	0.576411	3.1382	0.493005
PKP3	0.762743	0.456452	1.274561	0.301197
MAP7D2	1.583366	1.210012	2.071921	0.00081
CSF1	1.169015	0.981714	1.39205	0.079633
PLA2G6	1.147983	0.841688	1.565742	0.383453
MAML2	0.945579	0.758592	1.178657	0.618645
A3GALT2	1.05974	0.768965	1.460467	0.722908
SS18L1	0.587304	0.338602	1.018676	0.05821
TOP1MT	1.460034	1.117613	1.907367	0.005513
COPB2	0.892126	0.577803	1.37744	0.606516
LRRC19	0.601155	0.40316	0.896386	0.01254
THAP7	2.020075	1.277944	3.193177	0.002614
KNTC1	0.873658	0.626686	1.217958	0.425576
CCR10	1.729405	1.027145	2.911802	0.03933
WDR27	0.671796	0.472894	0.954358	0.026367
TXNRD2	1.389942	0.841258	2.296487	0.19871
FOXO4	1.172219	0.742046	1.851767	0.495798
PTP4A3	1.506772	1.276771	1.778205	1.23E-06
FOXD4L1	0.972315	0.626315	1.509458	0.900435
PROS1	1.047862	0.855401	1.283627	0.651605
GAST	1.373909	0.353971	5.332709	0.646178
HDDC3	1.758731	0.947262	3.265341	0.073719
BEX5	1.137354	0.890857	1.452055	0.301758
ZFP1	0.857454	0.57396	1.280973	0.452712
CEND1	1.0083	0.648203	1.56844	0.970751
DUSP8	1.352983	1.023656	1.788261	0.033649
SOCS3	0.910636	0.772815	1.073036	0.263546
LPAR5	0.973449	0.787903	1.20269	0.803046
XPOT	0.699882	0.479117	1.022371	0.064959

PDE4B	0.936696	0.766094	1.14529	0.523789
SNN	1.366557	1.036818	1.801164	0.026651
KCNH7	0.723402	0.282141	1.854784	0.500307
NELL2	0.830874	0.633517	1.089714	0.18056
KRBA2	0.777897	0.542155	1.116145	0.172737
MED12	0.862762	0.605436	1.229457	0.414007
ZNF93	0.831425	0.58081	1.190177	0.313118
CDCA2	0.970354	0.741225	1.270313	0.826669
ZBTB40	0.89862	0.610532	1.322647	0.5878
EIF4ENIF1	0.950811	0.547653	1.650758	0.857781
LRRC26	0.837285	0.589871	1.188474	0.320345
ATL3	0.968526	0.616776	1.520879	0.889531
NDUFA12	1.879882	1.135979	3.110935	0.014047
TCTE3	1.066947	0.624074	1.824103	0.81279
UBE2G2	1.753366	1.005656	3.057004	0.047721
OSBP2	0.97759	0.817524	1.168995	0.803797
APOO	1.431776	0.835727	2.452935	0.19133
PRR16	1.0368	0.909586	1.181806	0.588444
TMED9	1.154716	0.684531	1.947858	0.58973
TMEM186	1.454735	0.818694	2.584914	0.201275
SDR42E1	1.328888	1.001698	1.762948	0.04864
RBM33	0.729194	0.454546	1.169793	0.190321
ARMCX2	1.075883	0.843087	1.372959	0.556573
BTBD6	1.116958	0.738724	1.688853	0.600032
RBM43	0.946173	0.643611	1.390969	0.778378
SUMO3	2.235476	1.429143	3.496749	0.000425
IMMP2L	0.789162	0.544294	1.14419	0.211558
JAG2	1.460527	0.970395	2.198218	0.069386
FMNL1	1.257804	0.915027	1.728986	0.157675
LCN12	1.373061	0.955328	1.973456	0.086706
WT1	0.822924	0.697438	0.970989	0.020959
ZFP90	1.223493	0.870416	1.719791	0.245605
MUC6	0.767266	0.556272	1.058289	0.106382
NOC4L	1.546936	1.009381	2.37077	0.045196
USP18	1.234447	1.028615	1.481467	0.023629
NDUFA6	2.369376	1.328867	4.22461	0.00346
CHRM5	0.376013	0.195724	0.722375	0.003323
SORCS2	1.159793	0.969109	1.387997	0.105754
TMEM121	1.194851	1.029855	1.386282	0.018876
TMEM106/	0.646432	0.433243	0.964527	0.032609
SIVA1	1.058317	0.746142	1.501101	0.750607
BRI3BP	0.848679	0.58668	1.22768	0.383744
DGAT1	1.420889	1.013949	1.991151	0.041308
ROBO2	1.363582	0.674742	2.755655	0.387622
AP3M1	1.568261	0.887984	2.769692	0.121003
F8	1.638463	0.984167	2.727748	0.057618
CA13	1.308654	1.029843	1.662948	0.02777
UBOX5	0.913202	0.529648	1.574515	0.743905
MAFF	1.065788	0.839857	1.352497	0.600159
BRF1	1.332378	0.876194	2.026072	0.179623
SEMA4B	0.985778	0.737393	1.317831	0.922963
CIB1	1.205339	0.838658	1.732342	0.312885
SLC24A3	0.809437	0.716553	0.91436	0.000675
INTS5	1.7287	1.070213	2.792343	0.025265
RPS27L	1.159608	0.74221	1.811737	0.5154
MANEAL	0.999886	0.815732	1.225612	0.999123
ANO9	1.292495	1.045514	1.597819	0.017724
FAF1	1.121848	0.65598	1.918568	0.674515

FAM43A	0.96049	0.794529	1.161118	0.677041
HSF1	1.522655	1.009376	2.296942	0.045021
PURA	1.109064	0.844412	1.456662	0.456764
INPP5J	1.040236	0.780536	1.386344	0.787787
MIXL1	1.177666	0.808136	1.716167	0.394666
MFSD6L	0.89524	0.679375	1.179695	0.431817
LRRC37B	0.507995	0.34027	0.758393	0.000924
DDX51	1.169318	0.762285	1.793694	0.473653
NOMO2	0.84111	0.581816	1.215961	0.357493
SIGIRR	1.339658	0.922906	1.9446	0.124051
NRBP2	1.388156	1.139922	1.690447	0.001103
IFITM2	1.118007	0.868782	1.438725	0.386023
TNFAIP2	1.233798	1.071852	1.420212	0.003428
ZNF445	0.632129	0.447122	0.893687	0.009426
PGBD2	1.007397	0.689327	1.472231	0.96963
RAB11B	1.326792	0.90625	1.942487	0.145993
PRMT3	0.886503	0.54839	1.433083	0.622998
GP1BA	1.178175	0.912865	1.520593	0.20781
PRPF39	0.700819	0.429892	1.142491	0.153946
PPIL6	1.141705	0.838855	1.553891	0.399422
ZNF74	0.883926	0.63918	1.222388	0.455705
CDNF	1.004225	0.655884	1.53757	0.984523
KLHL33	1.056122	0.897877	1.242258	0.509704
RBM11	1.123602	0.754592	1.673064	0.566149
GALNT17	0.61246	0.084317	4.448792	0.627961
ZBTB37	1.011652	0.73204	1.398066	0.944046
IL3RA	1.522154	1.117094	2.074091	0.007781
CCDC137	2.192881	1.283739	3.745874	0.00405
RGPD2	1.06445	0.654461	1.731277	0.801292
ARL15	0.848535	0.596375	1.207313	0.361316
CDK10	1.303408	0.872334	1.947505	0.195898
TMEM105	1.179531	1.019302	1.364947	0.026649
SOCS1	1.602706	1.281091	2.005062	3.67E-05
TCN2	1.104702	0.962261	1.268229	0.157429
GAS2L1	1.028874	0.871354	1.21487	0.737065
ATP6V0A2	0.694052	0.471072	1.022579	0.064741
HGS	1.307914	0.911844	1.876022	0.144694
TNFAIP8L1	1.090709	0.662354	1.796091	0.73296
MAPK11	0.890924	0.71381	1.111983	0.307098
SP140L	1.312282	0.825364	2.086454	0.250675
MRPL30	0.907454	0.510112	1.614299	0.74107
SMYD3	1.020804	0.729089	1.429235	0.904553
METTL7A	1.374713	1.00594	1.878677	0.045811
FAM174B	1.012198	0.733111	1.397531	0.941276
TMEM179I	1.542058	0.930041	2.556815	0.093184
GPRIN3	1.164435	0.967348	1.401677	0.107599
STAC3	1.407754	1.077556	1.839136	0.012153
MUC1	1.081583	0.815452	1.434567	0.586284
IRF7	1.306233	1.060677	1.608639	0.011924
BRCC3	1.237701	0.774468	1.978009	0.372653
PDE6G	1.104111	0.878318	1.38795	0.396182
NR2F2	0.899424	0.548379	1.47519	0.674563
DLK1	0.869466	0.760598	0.993918	0.040428
AHNAK2	1.191987	0.955409	1.487147	0.119744
OLFML2A	1.248828	1.111225	1.403471	0.000191
SP1	1.39608	0.824269	2.364564	0.214562
WASH3P	0.965741	0.673839	1.384092	0.849435
MRPL40	3.020208	1.417905	6.433194	0.004169

PDIA2	0.882032	0.614699	1.265627	0.495655
PCGF3	0.612311	0.378395	0.990829	0.045773
LMLN	0.886078	0.671849	1.168618	0.391717
P4HB	0.839341	0.662651	1.063144	0.146435
PSMD13	2.681311	1.330351	5.404161	0.005812
PBX1	0.850525	0.658802	1.098044	0.214129
NDUFA4L2	1.023457	0.62076	1.687392	0.927581
ZFP36L1	0.953141	0.800227	1.135276	0.59064
UBE2L3	3.506161	1.632551	7.530036	0.001296
BRWD1	0.656183	0.433204	0.993935	0.046737
SNAI3	1.142247	0.835209	1.562158	0.405057
ZBTB3	1.225719	0.783837	1.916706	0.372261
LYG2	0.75857	0.559823	1.027875	0.074652
PRAME	0.973935	0.888023	1.068158	0.575109
MYBL1	1.232642	0.937341	1.620976	0.134427
DRG1	1.538795	0.833564	2.840682	0.16822
ANKFY1	0.735544	0.55422	0.976192	0.033435
YTHDF3	0.521088	0.312901	0.867793	0.012249
ZNF696	1.123255	0.764441	1.65049	0.553882
ADARB2	1.009399	0.781913	1.303069	0.942761
IFIT1	1.281965	1.070729	1.534875	0.006854
KCNQ5	0.839447	0.71603	0.984136	0.031001
MORF4L1	0.716611	0.413441	1.242089	0.23506
NLRP9	0.893122	0.380109	2.098521	0.795379
WDR53	1.617869	0.799507	3.273893	0.18097
DMWD	0.991442	0.762955	1.288356	0.948729
PIGP	0.844241	0.541256	1.31683	0.455361
IKZF1	1.622205	0.980316	2.684389	0.059755
PCYT2	1.034699	0.736791	1.45306	0.843915
NAT8L	0.815544	0.61035	1.089722	0.167925
BCAP31	1.940653	1.122405	3.355414	0.01763
GNB1L	0.671265	0.51962	0.867165	0.002282
DNAH14	0.948711	0.706191	1.274517	0.726675
EVI2B	1.043777	0.810113	1.344838	0.740368
ZNF829	0.880762	0.611731	1.268111	0.494779
THNSL1	0.915377	0.692221	1.210474	0.535139
TRIM69	1.487759	1.010964	2.189424	0.043876
ATP6V0C	1.051185	0.80922	1.365501	0.708409
IFITM1	1.015119	0.858012	1.200992	0.861147
LAMP1	0.99346	0.610501	1.616643	0.978928
TAS2R60	1.029859	0.868905	1.220627	0.734367
KLHDC8B	1.022528	0.846128	1.235703	0.817639
SETD4	1.251303	0.696136	2.249212	0.453667
PTCH1	0.764857	0.598469	0.977504	0.032213
RNPC3	0.831212	0.582891	1.185323	0.307254
ZNF267	1.059411	0.669117	1.677362	0.805553
IRS2	0.927739	0.77592	1.109262	0.410709
BICD2	1.282809	0.844104	1.949522	0.243493
CCIN	1.104671	0.836479	1.45885	0.482942
TMLHE	1.158147	0.698137	1.921264	0.569679
SDHAP3	1.381346	1.136211	1.679369	0.001191
RASA3	1.550029	1.153356	2.083128	0.003661
LRCH3	0.625798	0.367351	1.066075	0.084608
NDUFA13	0.914003	0.56807	1.470596	0.710953
ZNF566	0.734722	0.528363	1.021675	0.066878
ZNF529	0.677933	0.474106	0.969391	0.033143
ZNF284	0.724463	0.443036	1.184658	0.198928
DLEU7	0.838886	0.57214	1.229994	0.36825

KRT73	1.224772	0.936573	1.601654	0.138541
AIDA	1.299493	0.798654	2.114409	0.291534
CD300LF	1.410896	1.140409	1.745539	0.001525
CYP2R1	0.78723	0.495119	1.25168	0.311945
LRRC70	0.937514	0.711693	1.234988	0.646312
ANKRD46	0.701923	0.477385	1.032073	0.071946
PIP5K1C	1.351898	0.91114	2.00587	0.13421
CYP4F2	0.739968	0.614733	0.890717	0.001456
ZBTB6	1.248331	0.884268	1.762281	0.207373
TAS2R42	0.85445	0.544495	1.340847	0.493852
POLR3C	1.078228	0.633839	1.834182	0.781117
WWOX	0.903469	0.565294	1.443951	0.671332
BCL9L	1.118201	0.838332	1.491501	0.447168
POLR1D	1.549303	0.749313	3.203387	0.237493
KIF18B	0.988645	0.772479	1.265301	0.927718
ZNRF1	0.731763	0.55504	0.964755	0.026803
CYP4F12	0.883547	0.465371	1.67749	0.705053
ZNF749	0.800289	0.57834	1.107414	0.178848
BTLA	0.965688	0.806527	1.156257	0.703976
ZNF17	0.778792	0.535569	1.132472	0.19062
GPAT2	1.315773	1.024238	1.690289	0.031764
TOR3A	2.00629	1.183991	3.399688	0.009664
PPP1CC	1.488277	0.915015	2.420691	0.109132
ZNF555	0.729967	0.560634	0.950445	0.019419
NAP1L3	0.979345	0.736342	1.302542	0.885943
PRELID2	0.755368	0.549806	1.037785	0.083432
BACE1	0.956498	0.693357	1.319504	0.78643
RGS9BP	0.588596	0.430202	0.805309	0.000921
SLC36A3	1.014895	0.231068	4.45762	0.984376
RXRA	1.158113	0.956726	1.401891	0.132046
ANKRD37	1.117304	0.72188	1.729328	0.618706
NUDT17	1.215177	0.781304	1.889989	0.387134
ZNF75D	0.990744	0.694388	1.413581	0.959103
KRT10	1.360744	0.901089	2.054874	0.142999
CD300E	1.084702	1.002691	1.173421	0.042666
NKRF	1.49389	0.842084	2.650217	0.169962
GLDN	1.223074	0.965881	1.548753	0.094571
FCAR	1.063182	0.949317	1.190704	0.289128
KPNA4	1.234631	0.705899	2.159395	0.459946
ZNF501	1.006132	0.731085	1.384657	0.970069
ZNF197	0.725456	0.520517	1.011083	0.058107
SPATA12	0.790126	0.448711	1.391316	0.414509
AQP7P1	1.53885	1.126261	2.102586	0.006798
RPS23	0.990409	0.699395	1.402512	0.956701
GNG2	0.773931	0.643307	0.931078	0.006586
BTN3A2	1.203303	0.918909	1.575715	0.178545
AKAP14	0.82112	0.333615	2.021004	0.668012
INSIG1	1.036346	0.690341	1.555772	0.863255
ZNF396	0.952755	0.4778	1.899838	0.890682
TMEM222	1.391771	0.899804	2.152722	0.137405
ARHGAP3(	1.797773	1.262168	2.560665	0.001154
CYP4F3	0.92081	0.790186	1.073026	0.290524
SMYD4	0.949815	0.618787	1.45793	0.813817
GPATCH8	0.829178	0.499287	1.377037	0.4692
CEACAM1	1.013744	0.833861	1.232433	0.891057
NF2	1.194906	0.627722	2.274574	0.587704
SPATC1	1.268913	1.019449	1.579422	0.032971
UBE2H	0.996167	0.622503	1.594126	0.987226

HPDL	1.313002	0.941785	1.830541	0.108231
KATNA1	1.084035	0.576197	2.039461	0.802403
ARAP1	1.478824	1.136905	1.923573	0.003541
KIF24	0.959769	0.681449	1.351763	0.814209
PDE2A	1.169675	0.836352	1.635843	0.359789
PRG2	1.020897	0.738618	1.411055	0.900332
PRR5	1.119655	0.864185	1.450648	0.392378
ZFP91	0.907862	0.513009	1.606627	0.739955
BCDIN3D	1.377513	0.799082	2.374655	0.249027
LYRM7	0.752283	0.543563	1.041149	0.086025
BCR	0.879414	0.6847	1.129501	0.314267
FSCN2	0.945806	0.653355	1.369162	0.767829
SPIN4	0.771358	0.611289	0.973342	0.028694
SPIN2B	1.111882	0.68018	1.81758	0.672329
HYAL3	0.928709	0.767634	1.123583	0.446652
CXCR3	1.231468	0.990918	1.530413	0.060424
ZNF397	0.813922	0.563495	1.175644	0.272446
TPCN1	0.890043	0.699105	1.133129	0.344405
LILRB4	1.188789	1.069602	1.321257	0.001335
TNFRSF4	0.96813	0.851	1.101381	0.622519
HEXIM1	2.467115	1.52211	3.998826	0.000247
PDZD7	1.409813	1.119609	1.775237	0.003492
POFUT2	1.018438	0.676017	1.534304	0.93037
ERCC6L	1.044317	0.741944	1.469919	0.803652
TMEM17	0.825065	0.606921	1.121614	0.219672
TNFRSF18	1.008914	0.881908	1.154211	0.897133
RTN4RL2	0.818575	0.565422	1.18507	0.28893
ZDHHC17	0.725007	0.461597	1.138732	0.162718
P2RY4	0.984797	0.65928	1.471035	0.940353
ZNF395	0.849806	0.599737	1.204145	0.360063
ZACN	0.915673	0.510101	1.643709	0.767897
PPARA	1.031749	0.708526	1.502423	0.870518
KANK3	0.704926	0.46395	1.071064	0.101359
EMID1	0.953465	0.83565	1.087891	0.478867
ACTL7A	0.847669	0.343432	2.092241	0.719961
RHD	0.969877	0.830274	1.132955	0.699703
ESPN	1.112687	0.885497	1.398167	0.359483
GPR141	0.949194	0.795638	1.132386	0.562509
TMPRSS6	2.638531	1.306948	5.326793	0.006794
TMEM216	0.876149	0.626831	1.224632	0.438995
RPS19BP1	1.550298	1.010395	2.378697	0.044717
TEAD1	1.005317	0.62449	1.618382	0.982582
OR2AK2	0.965393	0.748538	1.245073	0.78614
PLCD1	1.042743	0.729983	1.489504	0.818051
ENTPD5	0.841967	0.541597	1.30892	0.444796
MITF	1.027992	0.776187	1.361485	0.847287
HEATR4	0.826569	0.537791	1.270411	0.385087
NAP1L1	0.940423	0.657895	1.344282	0.736146
LILRA5	1.134083	1.031147	1.247294	0.009549
CMC1	0.947715	0.452727	1.983895	0.886707
SLIT1	1.282939	0.914588	1.799644	0.149046
AKR1C1	1.122025	0.894302	1.407734	0.319848
SPATA21	0.746484	0.455047	1.224572	0.246964
RNF220	1.285076	0.915398	1.804045	0.147278
ZNF546	0.815086	0.537621	1.235748	0.335551
TSPYL4	0.832838	0.566952	1.223418	0.351209
MT1X	1.428613	1.021839	1.997316	0.036949
GCNT1	0.8436	0.649015	1.096526	0.203651

SESTD1	0.979284	0.807277	1.187941	0.831783
FNBP1	1.152896	0.729119	1.82298	0.54279
DYNC2H1	0.74929	0.538313	1.042954	0.087138
BCAM	0.990463	0.808479	1.213411	0.926291
RSBN1L	0.604918	0.369616	0.990017	0.045513
WDR86	1.118358	0.923691	1.354049	0.251612
EPOR	1.07058	0.889165	1.289009	0.47157
TAF9B	1.010936	0.610313	1.674537	0.966307
PCDHB13	0.790495	0.545469	1.145588	0.214256
FPR3	1.184347	0.956849	1.465934	0.120029
KCNJ11	0.633392	0.433662	0.925109	0.018142
COL4A1	0.865229	0.596361	1.255316	0.445821
GJA4	1.089143	0.881043	1.346396	0.429937
PTMA	0.974834	0.545462	1.742196	0.931437
HSPA14	1.288147	0.718623	2.30903	0.395142
SIRT7	1.062184	0.700747	1.610046	0.7762
IFT140	1.032066	0.722587	1.474092	0.862233
TLR5	1.139946	0.989999	1.312604	0.068716
USP7	1.021793	0.609702	1.712413	0.934778
NANOS3	0.826917	0.624674	1.094639	0.184153
NHLRC1	1.35588	1.04447	1.760138	0.022209
COX8C	1.222191	0.783456	1.906617	0.376508
PLEKHN1	1.206826	0.883928	1.647678	0.236674
ZNF385C	0.710266	0.577068	0.87421	0.001244
MAGEH1	0.96294	0.698698	1.327117	0.817516
TET3	0.687794	0.454472	1.040901	0.07667
ZNF286A	1.094395	0.754416	1.587587	0.634621
ISG15	1.413055	1.138068	1.754487	0.001741
EXD3	1.006896	0.755825	1.341366	0.962546
ZKSCAN4	0.904182	0.626944	1.304015	0.589792
DHRS4L2	1.724659	1.062985	2.798204	0.02729
SAMD11	0.693384	0.567763	0.846799	0.00033
VMAC	0.801461	0.493173	1.302464	0.371684
SPRY4	1.457817	1.01679	2.090136	0.040311
TRPV2	1.342314	1.003544	1.795443	0.047278
TMEM203	2.352665	1.227044	4.51087	0.009994
KBTBD12	1.168692	0.901963	1.514298	0.238262
DNAJB13	1.631248	1.135905	2.3426	0.008048
TCEA1	1.066295	0.686834	1.6554	0.774858
NHEJ1	1.069892	0.57331	1.996596	0.831925
FANCA	1.093916	0.768783	1.556553	0.617918
SECISBP2	0.723791	0.478313	1.095251	0.126147
OR52B6	0.852976	0.612745	1.187391	0.346062
SEMA4D	1.624736	1.057089	2.497202	0.026889
LIN28B	0.04063	2.70E-09	611531.9	0.704035
DNAH17	0.882632	0.532179	1.463867	0.628629
MCRS1	1.869723	1.176156	2.972281	0.008145
TMEM72	1.065062	0.7411	1.530639	0.733357
FANCM	0.894826	0.578306	1.384583	0.617816
ZNF70	0.790918	0.581447	1.075853	0.135121
CARD9	1.008951	0.841565	1.20963	0.923298
PEAR1	1.176205	1.034353	1.337512	0.013321
TMEM220	0.782791	0.555097	1.103882	0.162593
EIF4EBP1	1.562912	1.092017	2.236865	0.014639
P2RX2	0.780226	0.538121	1.131258	0.190433
TTC24	1.122561	0.94392	1.33501	0.191097
FAM122A	1.305228	0.810288	2.102487	0.273464
CLEC17A	1.112346	0.811541	1.524648	0.508058

LDLRAD2	1.260152	1.047049	1.516627	0.01443
PMS2CL	0.406084	0.214081	0.770291	0.005799
CYHR1	1.137959	0.760548	1.702654	0.529606
COL14A1	0.847571	0.674542	1.064983	0.155738
CPSF4L	0.354593	0.15299	0.821857	0.015632
KLHL17	1.086553	0.867109	1.361533	0.470805
PLA2G2C	1.367819	1.043883	1.792279	0.023122
ZSCAN23	0.635414	0.492858	0.819202	0.000468
RINL	1.896258	1.372532	2.619826	0.000104
MORN2	0.948338	0.654218	1.374688	0.779459
S100A3	1.674877	1.13342	2.474998	0.009638
UBQLN2	1.373794	0.764435	2.468896	0.288314
RILPL1	0.440904	0.2746	0.707927	0.0007
ZNF490	0.944419	0.542126	1.645239	0.839978
CLCN1	1.604764	1.184416	2.174292	0.002272
ARL4C	1.129102	0.908973	1.402541	0.272467
TREML4	1.054376	0.859489	1.293453	0.611591
RAB42	0.906077	0.594337	1.381332	0.64664
SCGB1C1	0.707142	0.370363	1.350161	0.293655
GPR89B	0.965998	0.601911	1.550317	0.88603
EYS	1.270003	0.484734	3.327406	0.626697
MAPK12	0.906472	0.711974	1.154103	0.425528
COL4A5	0.998146	0.895615	1.112414	0.973226
AGRN	0.763821	0.638068	0.914357	0.00333
FAM166A	0.96507	0.653299	1.425627	0.858249
ZNF626	1.005701	0.839625	1.204626	0.950774
HEPACAM	0.846288	0.600039	1.193595	0.341467
ZC3H6	0.786654	0.56033	1.104394	0.165651
PRKAR1B	1.036326	0.762434	1.408609	0.819757
DCUN1D3	0.9559	0.689089	1.326019	0.787088
POTEE	4.468614	0.602129	33.1632	0.143217
ZNF793	1.046423	0.863175	1.268572	0.644096
COMMD6	0.981834	0.601698	1.602128	0.941502
IL17REL	1.112628	0.563618	2.19642	0.758412
RUFY4	1.341994	1.076063	1.673646	0.009039
ZNF383	0.729811	0.430649	1.236794	0.241876
HES4	1.136034	0.933324	1.382771	0.203422
ZNF669	0.699322	0.442106	1.106186	0.126357
CENPP	0.965891	0.611264	1.526256	0.881814
PLSCR1	1.221235	0.889739	1.676239	0.216113
ZNF559	0.645205	0.457919	0.909089	0.012252
SBK1	1.013652	0.799994	1.284372	0.910607
SLC38A3	0.437084	0.195195	0.978727	0.044194
OR6N2	0.299087	0.068962	1.297132	0.106869
GTF2F2	0.907774	0.520512	1.583162	0.733122
PRR19	1.104259	0.772555	1.578383	0.586352
ZP3	1.055944	0.745224	1.496218	0.759499
PDCD1	1.254238	1.036069	1.518348	0.020158
CLEC2A	1.062462	0.869241	1.298633	0.554109
GPR21	1.035135	0.80776	1.326512	0.784944
TCTEX1D4	1.080066	0.847065	1.377157	0.534444
SELL	0.99754	0.855985	1.162505	0.974836
CHM	0.849498	0.532436	1.355367	0.49379
CERKL	0.637864	0.435943	0.933312	0.020593
IER5L	1.551419	1.162159	2.071059	0.002887
FAM83G	1.889699	1.4139	2.525611	1.71E-05
HBA2	0.943864	0.871978	1.021676	0.15289
DUSP28	1.742181	0.953146	3.184393	0.071228

NBR1	1.182298	0.830921	1.682265	0.352044
RALGAPA2	0.716507	0.477148	1.07594	0.108032
NDOR1	1.296508	0.903489	1.860492	0.158778
NKAIN2	1.125815	0.947313	1.337952	0.178479
PAQR9	0.917491	0.64336	1.308426	0.634422
CLN3	0.921758	0.576913	1.472729	0.733273
FAM72B	1.155583	0.821827	1.624882	0.405658
SUMO2	0.711391	0.397875	1.271948	0.25072
NANOS1	0.54376	0.348112	0.849367	0.007418
HMX3	1.280394	1.010403	1.622529	0.040794
DPYD	1.18842	0.999559	1.412966	0.050587
S100A16	1.093577	0.974144	1.227652	0.129516
PTAR1	0.997831	0.693896	1.434894	0.990653
CC2D2B	0.607424	0.412181	0.895148	0.011741
RHCE	0.955606	0.811239	1.125663	0.586844
PARVB	2.040732	1.415216	2.942723	0.000134
SLC4A5	0.771994	0.481976	1.236525	0.281633
UROS	1.222391	0.772792	1.933559	0.390726
ZDHHC9	0.743137	0.551603	1.001178	0.050912
QRFP	0.669204	0.418989	1.068844	0.092708
TMEM120	0.941617	0.606232	1.462546	0.788882
RBM34	0.648484	0.376521	1.116886	0.11842
NOXA1	1.191454	1.000827	1.418391	0.048924
BCL2L15	0.989462	0.643247	1.522022	0.961544
FZD9	1.625291	0.335321	7.877749	0.546432
SPRED3	1.123305	0.71366	1.76809	0.615394
ZNF548	0.644522	0.446464	0.930442	0.019036
MTF1	1.193225	0.803139	1.772776	0.381788
TMCO2	1.264754	0.667323	2.397044	0.471513
TMEM201	1.237772	0.872646	1.755671	0.231655
NHLRC3	0.534724	0.359419	0.795532	0.002012
HMX2	1.72406	1.198708	2.479655	0.00331
ZDHHC11	1.732993	1.220865	2.459948	0.002094
CNR2	1.163503	0.897882	1.507703	0.252084
ENTPD8	1.233847	0.857055	1.77629	0.258361
RPL14	1.069825	0.695911	1.644642	0.758368
BEND4	1.106924	0.97871	1.251935	0.105805
ZNF563	0.983376	0.697366	1.386686	0.923836
TMC3	0.80332	0.362106	1.78214	0.590102
FBF1	1.035798	0.642896	1.66882	0.885077
KLRG2	0.885871	0.685478	1.144847	0.354367
ASTL	1.328676	0.968947	1.821957	0.077709
MSL1	0.712698	0.404167	1.256752	0.241875
LRRK2	1.035079	0.917327	1.167947	0.57579
TRMT2B	0.791039	0.402725	1.553774	0.496161
FAM120A(	2.763517	1.459279	5.233424	0.001809
NOC2L	1.282179	0.837363	1.963286	0.252858
ZNF292	0.640494	0.445126	0.921611	0.01641
HUS1B	1.314969	0.737445	2.344774	0.35347
KCTD21	1.279279	0.83962	1.949162	0.251649
ADAT2	1.008843	0.704306	1.445059	0.961701
KIR2DL4	1.715314	1.341284	2.193647	1.71E-05
ZNF567	0.752561	0.505275	1.120869	0.161937
NDUFA4	1.323399	0.788642	2.220761	0.28872
ALKBH2	1.195297	0.765843	1.865572	0.432207
RNFT1	0.812805	0.538658	1.226476	0.323441
RELN	1.263667	0.84176	1.897043	0.258921
FAM111B	1.008371	0.844386	1.204204	0.92665

LITAF	1.118083	0.820995	1.522677	0.478755
VSTM1	0.91844	0.839824	1.004415	0.062394
TMEM120A	1.191479	0.886288	1.601761	0.245879
ARID2	0.773249	0.498986	1.198257	0.249874
SF3B3	1.300885	0.79662	2.124354	0.293142
BLOC1S3	2.114881	1.226239	3.647514	0.007073
SP6	1.854512	0.632663	5.436089	0.260334
PLAC9	0.92546	0.64673	1.324317	0.671804
ZNF573	0.66767	0.481082	0.926627	0.015707
ZNF527	0.737607	0.449526	1.210305	0.228385
S100A13	1.528977	0.970491	2.408853	0.067129
ZNF33A	0.73507	0.529528	1.020395	0.065869
ZNF600	1.104866	0.772516	1.580199	0.584909
BTBD8	0.791165	0.52747	1.186688	0.257438
MAOA	1.219943	0.980044	1.518566	0.075154
TSPYL1	1.151118	0.659734	2.008493	0.620233
PNRC2	1.725332	1.058133	2.81323	0.02878
FHIT	1.048577	0.763639	1.439835	0.769376
ZKSCAN3	0.64985	0.475897	0.887387	0.006696
RRP7A	1.745033	1.064114	2.861668	0.02737
LIN54	1.061826	0.648732	1.737968	0.811393
FAM53B	1.811535	1.233166	2.661167	0.002461
ALG1L	0.551618	0.308487	0.98637	0.04483
GSPT2	1.307046	1.028863	1.660444	0.028308
CXCL17	0.992169	0.73381	1.34149	0.959257
HMGB1	0.904301	0.583848	1.400641	0.652261
SH2D5	0.709724	0.310505	1.622223	0.416258
ZFP92	1.029519	0.802721	1.320396	0.81876
NCR1	1.082052	0.88228	1.327059	0.448892
BLOC1S2	2.27927	1.411777	3.679812	0.000749
SYCP2	0.968359	0.809775	1.157999	0.724568
IL1RAP	1.234339	0.979931	1.554797	0.073807
PAX5	0.973559	0.831398	1.140027	0.739337
ZNF699	0.987363	0.609991	1.598199	0.958723
TDRD7	1.174548	0.904597	1.525056	0.227257
KIAA0895L	1.151295	0.840978	1.576118	0.379297
HLA-DRB1	1.279579	1.108729	1.476756	0.000748
MYT1	1.000155	0.740969	1.350002	0.999191
AKR1C3	1.156825	0.961027	1.392514	0.123611
SPATS2L	1.64777	1.296309	2.094521	4.50E-05
ZNF250	0.690238	0.44388	1.073328	0.099803
WDSUB1	0.490917	0.281337	0.856623	0.012252
ZNF79	1.031572	0.632673	1.681974	0.900828
S100A4	1.456434	1.213383	1.748171	5.43E-05
PLEKHG4	1.079847	0.897881	1.29869	0.414558
FAT4	0.898071	0.52472	1.53707	0.694984
ZNF681	0.817366	0.633847	1.05402	0.120079
ACADSB	0.795011	0.561283	1.126067	0.196526
STK40	1.377556	0.911676	2.081508	0.128286
TMEM63A	1.096986	0.791927	1.519558	0.57768
CTSE	0.771944	0.610968	0.975334	0.030062
SEMA4A	1.137649	0.935727	1.383143	0.195802
MPHOSPH	1.159494	0.695394	1.933332	0.570504
GREB1	0.597842	0.348192	1.026488	0.062157
SIRPB2	0.949387	0.825589	1.091748	0.466251
ZNF766	0.753401	0.496067	1.144227	0.184159
RYR1	1.653122	1.284888	2.126887	9.24E-05
SRGAP3	0.791423	0.64228	0.975198	0.028113

TUBB	1.245269	0.840999	1.843874	0.273387
LCOR	1.287306	0.81823	2.025293	0.2747
SUPT5H	1.589283	0.969973	2.604011	0.065923
XPNPEP3	0.866587	0.487663	1.539942	0.625447
ZNF107	0.679429	0.52467	0.879837	0.003382
PPIA	1.271728	0.715602	2.260045	0.412589
ZNF471	0.913941	0.708567	1.178841	0.488327
ZNF836	0.929075	0.652573	1.322734	0.683162
ZNF493	0.62972	0.467942	0.847428	0.002268
GTF2IRD2	1.197837	0.677301	2.118425	0.5349
SUPT3H	0.843994	0.577578	1.233297	0.380798
NIF3L1	1.353103	0.708972	2.582453	0.359142
ATP2A1	1.255921	0.863663	1.826334	0.232967
POM121	0.683866	0.418181	1.118348	0.129964
ZBTB44	0.721093	0.47131	1.103255	0.131794
GIMAP5	1.033287	0.773566	1.380208	0.824549
NLGN3	0.919376	0.622983	1.356781	0.672044
CD55	0.910413	0.660878	1.254169	0.565785
ZNF565	0.648275	0.37788	1.112153	0.115496
NTNG2	0.825806	0.737857	0.924238	0.000865
WDR5	1.634504	0.847517	3.152272	0.142581
LONP1	1.235755	0.817433	1.868155	0.315416
TRRAP	0.749884	0.531249	1.058497	0.101693
NUDT11	0.970313	0.752958	1.250412	0.815837
FUT4	0.917241	0.728323	1.155162	0.462863
ASB13	1.63017	1.134778	2.341828	0.008192
ZNF34	1.41596	0.792754	2.529085	0.239904
ZNF140	0.803591	0.510937	1.263872	0.343942
INCA1	0.892656	0.609516	1.307324	0.559671
PTPN1	2.23303	1.420169	3.511147	0.000503
EVL	1.132593	0.89927	1.426454	0.290108
THEM5	0.991341	0.737958	1.331726	0.953951
EPHB4	0.835036	0.673385	1.035494	0.10054
PRTN3	0.979682	0.909508	1.05527	0.588284
ZNF765	0.735758	0.49467	1.094346	0.129803
ZNF124	0.514223	0.378765	0.698125	2.01E-05
XRCC6	1.853896	1.085987	3.1648	0.02368
S100A5	1.966191	1.262229	3.062761	0.002792
TSC22D2	1.399166	0.773152	2.532057	0.267069
ASMT	1.268018	0.37748	4.259483	0.700909
ZNF569	0.827741	0.544785	1.257663	0.375723
ARMCX4	0.836318	0.562591	1.243227	0.376876
YRDC	0.952129	0.64331	1.409195	0.806283
ZNF777	1.458377	0.920761	2.309898	0.107808
PIK3R4	1.057875	0.629569	1.777564	0.831734
ZNF775	0.98921	0.67568	1.448225	0.955518
ZNF605	0.823999	0.539961	1.257452	0.369362
TRAPPC2	1.06617	0.570205	1.993529	0.840963
MYL6B	1.395865	0.857346	2.27264	0.179897
ZNF799	0.645302	0.39182	1.06277	0.085286
SIAH1	0.442167	0.265931	0.735196	0.001657
IPO4	0.713872	0.375391	1.357553	0.30404
NCOR2	1.656543	1.167529	2.350378	0.004688
SULT1A1	1.005818	0.743824	1.360093	0.969943
ARL9	1.161316	0.880004	1.532554	0.290632
PRPF40A	0.850376	0.504146	1.434387	0.543451
GDAP2	0.985994	0.64251	1.513104	0.948531
TCEAL3	0.905692	0.639897	1.281891	0.576251

ANAPC7	0.707014	0.375746	1.330334	0.282386
TPK1	1.068294	0.79153	1.441829	0.665874
SLC6A9	0.98804	0.812435	1.201602	0.904078
AFAP1	0.669086	0.365801	1.223823	0.192114
NACA	0.811943	0.539747	1.221408	0.317333
MYO18A	1.014247	0.762432	1.349231	0.922604
MAN2A2	0.992283	0.734877	1.339851	0.959676
MME	1.078156	0.8944	1.299666	0.429913
FAM72A	1.143807	0.752856	1.737775	0.528926
CACNA1H	0.89795	0.720721	1.11876	0.337274
SULF2	1.087048	0.95466	1.237795	0.207785
HBG2	0.855401	0.718405	1.018521	0.079449
PLXNB2	1.388725	1.108839	1.739258	0.004241
XRCC2	0.99863	0.737198	1.352775	0.992939
MYO6	1.274058	0.769971	2.108164	0.34587
HDAC2	0.821741	0.514667	1.312029	0.410855
ZNF782	0.718636	0.484746	1.065377	0.100029
ZNF846	0.776528	0.548572	1.099209	0.153731
MMP1	1.23367	0.436878	3.483673	0.691754
TCF4	1.190582	1.011982	1.400702	0.035414
ZNF136	0.639735	0.404792	1.011038	0.055754
ZKSCAN5	0.898912	0.499314	1.618305	0.722396
ZNF502	1.094885	0.880641	1.361249	0.414549
TRAPPC4	2.055571	1.094966	3.858905	0.024943
TTC30B	1.025903	0.666704	1.578628	0.907415
TECPR2	0.879378	0.599215	1.290531	0.511325
TLR7	1.149369	1.018857	1.296599	0.02359
ZFP62	0.850594	0.595071	1.215838	0.37466
ERI2	0.958363	0.680218	1.350241	0.807888
TOMM7	1.01826	0.625551	1.657505	0.94197
HSH2D	0.975624	0.804481	1.183175	0.80199
TRPV1	0.871731	0.542903	1.399726	0.569926
ZNF33B	0.954555	0.657478	1.385864	0.806844
ZNF512B	1.072185	0.810233	1.418826	0.625798
AMZ2	0.985891	0.594526	1.634885	0.956088
ZNF431	0.66122	0.464677	0.940894	0.021534
NF1	0.677211	0.474546	0.966429	0.031701
VKORC1L1	0.74846	0.509154	1.100243	0.140489
ZNF418	1.176595	0.982503	1.409029	0.077052
DAPK1	1.156502	0.970612	1.377995	0.103881
HLA-DQA	1.124834	0.990065	1.277948	0.07082
COL27A1	0.757058	0.553223	1.035996	0.082034
GM2A	1.342442	0.986413	1.826972	0.061074
S100A2	2.481914	1.083898	5.683095	0.031511
ZNF700	0.630962	0.451146	0.882448	0.007132
CD47	0.747917	0.520724	1.074235	0.115874
TLE1	0.842226	0.703375	1.008487	0.061759
MAML3	1.029664	0.694662	1.526223	0.884252
STRN3	0.549346	0.314496	0.959568	0.035292
ZNF239	1.201854	0.942274	1.532943	0.138599
CHRNG	1.659221	0.829331	3.31956	0.15241
ZSCAN16	0.955328	0.631024	1.446304	0.829001
ADA	0.97912	0.790205	1.213199	0.84701
ARID5A	0.997057	0.747564	1.329817	0.983998
PPTC7	0.861441	0.554708	1.337786	0.506607
TOMM20L	0.692062	0.434345	1.102696	0.121464
RGPD4	0.622236	0.19816	1.953866	0.416416
NHLRC2	0.930889	0.644006	1.34557	0.703224

ZFP28	0.900079	0.609792	1.328556	0.59617
CBWD3	0.314051	0.120862	0.816037	0.017443
SCN8A	1.014946	0.766478	1.343961	0.917521
LAMB3	1.054091	0.747522	1.486388	0.763851
KPNA5	0.717868	0.47832	1.077386	0.109568
ANKRD36I	0.850501	0.622205	1.16256	0.309901
ARHGEF12	1.037484	0.851258	1.264451	0.715437
PDLIM7	1.503557	1.176934	1.920823	0.0011
FLNA	1.451876	1.117916	1.885602	0.005178
RPS26P11	0.975644	0.683376	1.392908	0.892031
SRGAP1	1.306825	1.051745	1.62377	0.015721
FAM3C	0.72056	0.42759	1.214263	0.218382
SLC39A10	0.871463	0.614743	1.23539	0.439688
CASP4	1.097674	0.748669	1.609373	0.633119
AP2A1	1.483634	1.020478	2.156998	0.038816
ZNF585A	0.828295	0.533775	1.285321	0.400731
FUT11	1.252742	0.6634	2.365636	0.487224
ANXA4	1.598952	1.122674	2.277285	0.009288
LAGE3	1.617484	1.126883	2.321674	0.009113
WDR5B	0.898447	0.605485	1.333157	0.594829
WDR45	1.415525	0.845014	2.371215	0.186772
METTL9	1.266983	0.916988	1.750564	0.151404
ZNF138	0.650984	0.477792	0.886957	0.006527
ZNF429	0.748654	0.575109	0.974567	0.031443
ZNF470	1.108924	0.724923	1.696334	0.633567
SERTAD1	1.675557	1.176241	2.386833	0.004248
ZNF100	0.650782	0.442606	0.956872	0.028954
ZNF398	0.670665	0.40235	1.117912	0.125424
ANXA6	1.178519	0.927511	1.497456	0.178893
ZNF441	0.788006	0.563936	1.101108	0.162799
GMFB	1.157519	0.728884	1.838221	0.535345
SIGLEC15	0.823707	0.639764	1.060537	0.13255
ZNF420	0.869866	0.577157	1.311024	0.505347
ZNF763	0.566473	0.363482	0.882828	0.012057
ZMYM1	0.76373	0.54672	1.066877	0.114016
MAFG	1.664494	1.063088	2.606124	0.02592
ARRDC1	1.102935	0.782469	1.554649	0.575892
KIAA1671	1.36245	0.835217	2.222499	0.215433
IGF2R	1.219338	1.045802	1.42167	0.011351
GAL3ST4	0.689847	0.517182	0.920157	0.011533
DYNC1H1	0.946316	0.665168	1.346296	0.759015
PCBP2	0.756825	0.3819	1.499828	0.424633
ZGPAT	1.174681	0.804266	1.715694	0.404861
SLC25A29	0.724449	0.581812	0.902054	0.003959
PGAP1	0.935172	0.711949	1.228383	0.630032
SRC	1.352021	1.128821	1.619354	0.001052
ZNF682	0.853512	0.631281	1.153974	0.30333
ZNF772	0.953803	0.732195	1.242484	0.725889
ZNF257	0.915197	0.748281	1.119347	0.388387
ADAM32	1.125347	0.697612	1.815344	0.628365
ACSL5	1.760377	1.136266	2.727291	0.011345
LRRC8B	1.114192	0.816197	1.520986	0.495905
ABCB8	1.220583	0.823001	1.810234	0.321565
SND1	1.041901	0.656314	1.654021	0.861808
ZNF785	0.736904	0.458957	1.183179	0.206332
SULT1A2	0.936297	0.616143	1.422806	0.757853
NEK5	0.617847	0.410657	0.929572	0.020867
PSMD12	0.991568	0.590726	1.664406	0.974437

PIWIL2	0.816658	0.484257	1.377222	0.447503
SLC22A4	0.82638	0.665273	1.026502	0.084788
ENTPD4	0.878888	0.62166	1.242551	0.464942
C1D	0.963674	0.571048	1.626252	0.889772
TBC1D9B	1.404612	0.919429	2.145825	0.11608
SERPINA1	1.043687	0.949657	1.147026	0.374726
TPSB2	0.915534	0.824168	1.017027	0.099929
KANK2	1.187379	0.961456	1.466388	0.110725
GTF2E2	1.358742	0.712808	2.59001	0.351649
IL27	1.191803	0.857572	1.656298	0.296049
GUCA2A	1.406965	0.490749	4.033735	0.525193
ZNF165	0.829936	0.563859	1.221572	0.344573
SYNGAP1	1.280187	0.974341	1.682036	0.076171
FITM2	0.765294	0.451966	1.295841	0.319497
BLM	1.009945	0.685513	1.487922	0.960077
ZNF720	0.70604	0.482801	1.0325	0.072649
DDI2	1.125214	0.77896	1.625379	0.529533
SVIL	0.793313	0.574864	1.094772	0.158845
TRIM33	0.667709	0.376135	1.185304	0.167775
LRP10	1.471897	1.104756	1.961048	0.008279
PELI1	1.194487	0.99132	1.439291	0.061718
ZNF655	0.618117	0.417398	0.915358	0.016331
MRPL21	2.0448	1.088965	3.839618	0.026076
LYPD2	1.235166	0.955746	1.596278	0.106515
UAP1L1	1.168939	0.890555	1.534344	0.260689
FBXL22	1.054001	0.596925	1.861068	0.85613
ZNF786	0.883078	0.623689	1.250346	0.483439
ZNF517	0.83302	0.57173	1.213725	0.341427
ZNF675	0.720778	0.473895	1.096281	0.125935
SLC22A5	1.242979	0.962682	1.604886	0.095263
DACT3	1.16782	0.603798	2.258706	0.644836
ADARB1	1.167438	0.887283	1.536051	0.268829
HTT	1.257604	0.818333	1.93267	0.295798
OR6N1	0.156751	0.025999	0.945059	0.043217
C5AR1	1.108376	1.008176	1.218534	0.033304
VEPH1	1.306945	0.770993	2.215464	0.320162
SHPK	1.066959	0.506368	2.24817	0.864663
IPP	1.023857	0.651863	1.608133	0.918482
MAP3K5	1.310766	0.938924	1.829869	0.111893
CYP2F1	0.716689	0.279739	1.836151	0.487693
GSTK1	1.263244	0.856322	1.863536	0.238783
HNRNPAB	2.270223	1.277402	4.034683	0.005199
STMN3	1.029525	0.823853	1.286543	0.798026
PDGFA	0.936563	0.70397	1.246003	0.652741
GYPE	1.010672	0.859845	1.187955	0.89757
SPN	0.774763	0.542959	1.105529	0.159461
ZNF695	1.112862	0.821146	1.508211	0.490535
ZNF628	1.130055	0.851468	1.499791	0.397219
SLC2A10	1.157904	0.917227	1.461734	0.217496
ZNF665	0.815609	0.540966	1.229687	0.33057
RPF2	1.061029	0.688755	1.634518	0.788164
SLC28A3	0.80011	0.684527	0.935211	0.005088
FAM177B	1.154786	0.891627	1.495616	0.275425
MIB2	1.05619	0.789173	1.413551	0.713134
MYO5A	1.045251	0.793266	1.377281	0.753178
GZMM	1.111141	0.882251	1.399413	0.370533
ATG7	0.953082	0.690294	1.315909	0.770307
SIPA1L1	0.835425	0.642849	1.085689	0.178625

TTC30A	0.77509	0.480395	1.250563	0.296549
ELANE	0.920175	0.86509	0.978767	0.008256
RAB40C	0.932085	0.677719	1.281923	0.665351
PIGN	0.53771	0.348431	0.829812	0.005068
ZNF624	0.762869	0.502776	1.157512	0.203244
HHLA3	1.376418	0.956731	1.980209	0.085141
HOXA4	1.205691	1.036491	1.402512	0.015329
TOPORS	0.932658	0.606365	1.434533	0.750969
BCO2	0.963121	0.642034	1.444786	0.855892
ENTPD6	1.258812	0.813627	1.947585	0.301284
KLKP1	1.239054	0.515586	2.977686	0.631835
FAR1	1.103759	0.782445	1.557022	0.573854
ZNF841	0.917893	0.750608	1.122461	0.403946
VN1R5	1.189643	0.693299	2.041328	0.528467
ZNF615	0.88838	0.610145	1.293494	0.536946
CDC42SE1	1.418207	1.015029	1.98153	0.040621
MPEG1	1.103976	1.016695	1.198751	0.018574
SERPINB2	1.099172	0.980153	1.232642	0.10585
DPP4	1.390801	1.012322	1.910783	0.041797
PDCD1LG2	1.411749	1.038507	1.919135	0.027725
ZNF433	0.698367	0.495863	0.983571	0.039901
DNAH10	0.989857	0.646018	1.516702	0.962653
SPTAN1	1.041193	0.723618	1.498143	0.827865
NMB	1.013604	0.714866	1.437182	0.939542
KLHL14	1.266614	0.819678	1.957246	0.287133
FAM114A1	0.847211	0.668224	1.07414	0.170901
RPE	0.932828	0.590913	1.472581	0.765313
ZNF460	0.734642	0.568122	0.949971	0.018708
CR1L	0.862089	0.666424	1.115202	0.258551
PHF2	1.013958	0.635988	1.616555	0.953553
RPS26	1.018128	0.785023	1.32045	0.892277
PSAP	1.150786	0.946658	1.39893	0.158618
S100A10	1.046388	0.931039	1.176028	0.446709
LHFPL5	0.685817	0.444166	1.058941	0.088834
RPL37A	1.031926	0.682224	1.56088	0.881677
CFD	0.92907	0.848952	1.01675	0.109831
EME2	1.317475	0.907024	1.913666	0.147728
KLHDC1	0.77267	0.535548	1.114781	0.1679
ZNF81	0.828289	0.53777	1.275757	0.392627
TAF13	0.797743	0.511056	1.245253	0.319944
ZNF780A	0.900187	0.633563	1.279015	0.557365
ATAD3A	1.419865	1.00712	2.001765	0.045452
FAM118B	1.141634	0.731957	1.780608	0.559169
ZNF461	0.688107	0.461083	1.02691	0.067255
SLC9A8	1.388812	0.874067	2.206696	0.164456
OCLN	1.172354	1.002319	1.371234	0.046708
[truncated: 82,327 more chars]
